# Supplementary material for: Modeling of mRNA deadenylation rates reveal a complex relationship between mRNA deadenylation and decay
Source: EMBO J. 2024 Oct 11;43(24):6525–54. doi: 10.1038/s44318-024-00258-3 (PMC11649921; doi:10.1038/s44318-024-00258-3)
Supplement: Supplementary file 1 — Appendix [file 44318_2024_258_MOESM1_ESM.pdf]

APPENDIX

***

Modeling of mRNA deadenylation rates reveal a complex relationship between mRNA deadenylation and decay

Agnieszka Czarnocka-Cieciura[1,#], Jarosław Poznański[2,#], Matti Turtola[3], Rafał Tomecki[2,4], Paweł S. Krawczyk[1], Seweryn Mroczek[4,1], Wiktoria Orzeł[1], Upasana Saha[5], Torben Heick Jensen[5], Andrzej Dziembowski[1,4]*, Agnieszka Tudek[2,]*

[1] International Institute of Molecular and Cell Biology, Księcia Trojdena 4, 02-109 Warsaw, Poland
[2] Institute of Biochemistry and Biophysics, Polish Academy of Sciences, Adolfa Pawińskiego 5A, 02-106 Warsaw, Poland
[3] Department of Life Technologies, University of Turku, Biocity, Tykistökatu 6, 205240 Turku, Finland
[4] University of Warsaw, Faculty of Biology, Miecznikowa 1, 02-089 Warsaw, Poland
[5] Aarhus University, Department of Molecular Biology and Genetics – Universitetsbyen 81, 8000 Aarhus, Denmark

[#] those authors contributed equally

* correspondence should be addressed to: atudek@ibb.waw.pl or adziembowski@iimcb.gov.pl

***

TABLE OF CONTENT

1. Appendix Methods - *page 2*

1.1. Western blot analysis – *page 2*
1.2. Recombinant Xrn1 expression, purification and testing – *page 2*
1.3. Xrn1 in vitro digestion of the pA+ fraction – *page 3*
1.4. Reverse transcription and quantitative PCR analysis – *page 3*
1.5. RNaseH digestion of *RPS5* 3'end – *page 4*
1.6. Northern blotting – *page 4*
1.7. Bulk pA-tail length analysis – *page 4*
1.8. Calculation of the modified gamma parameters – *page 5*
1.9. Bioinformatic analyses – *page 5*
1.10. Description of dataset quality control and considerations regarding the estimation of decay and quantile deadenylation coefficients – *page 6*

2. Appendix Tables – *page 10*

3. Appendix References – *page 17*

4. Appendix Figures – *page 18*

5. R code for calculation of the modified gamma distribution parameters – *page 27*

1. APPENDIX METHODS

1.1. Western blot analysis

4-6 OD units of cells were resuspended in 8M urea and incubated at 80 °C. Cells were subsequently mixed with glass beads and disrupted on a vortex for 5 min. The urea cell extract was centrifuged and the supernatant was collected. The protein concentration was measured, and after adjusting to equal concentrations, extracts were resuspended in Laemmli buffer. 30 µg of protein was loaded on a 6 or 10 % denaturing SDS-PAGE gel. After gel migration, proteins were transferred to Amersham nitrocellulose Protran Western blotting membranes (GE10600001) using semi-dry transfer (semi-dry blot apparatus, Bio-Rad, cat. no.1703940). Blots were blocked in TBS-T and 5 % milk and probed with relevant antibodies overnight. Antibodies used were: anti-FLAG (F1804, Sigma), anti-PGK1 (discontinued Novex Life Technology), anti-Pab1 (Santa Cruz Cat. no.: sc57953) or anti-Rpb3 (Abcam 1Y26 cat. no.: ab81859). After washing in TBS-T blots were probed with appropriate secondary antibodies conjugated with HRP in TBS-T 5 % milk for 1 hour, washed, and developed with ECL substrate (Clarity Western ECL, BioRad).

1.2. Recombinant Xrn1 expression, purification and testing

Nucleotide sequence encoding *Thermothelomyces* (*Myceliophthora*) *thermophilus* Xrn1, with codons optimized for heterologous expression in *Escherichia coli*, was synthesized by GenScript, provided as a derivative of pUC57 plasmid, and re-cloned into NcoI/XhoI sites of the pET-28b(+) vector (Novagen) using sequence and ligation-independent cloning (SLIC) with primer pair TtXrn1For (5'-ttttgtttaactttaagaaggagatataccATGGGCGTCCCGAAGTTTTTCC-3') - TtXrn1Rev (5'- atctcagtggtggtggtggtggtgctcgagGCTCTGCAGTGCTGCGGTCTG-3') for insert amplification in PCR.

Resulting pET-28-TtXrn1-6xHis recombinant vector was introduced into *E. coli* BL21-CodonPlus(DE3)-RIL chemo-competent cells (Agilent; *E. coli* B F– ompT hsdS[rB– mB–] dcm+ Tetr gal λ[DE3] endA Hte [argU ileY leuW Camr]) by heat shock-based transformation. Transformants were selected in a standard solid Luria-Broth (LB) medium supplemented with 50 µg/mL kanamycin, and then used to inoculate 50-100 mL of liquid LB containing 50 µg/mL kanamycin and 34 µg/mL chloramphenicol. Following overnight incubation at 37 °C with shaking (120 rpm), 30 mL of the starter culture was utilized to inoculate 1 L of Auto Induction Medium (AIM) Super Broth Base including Trace elements (AIMSB02, Formedium) containing 2% glycerol and both antibiotics. Bacteria were grown for 72 h at 18 °C in an orbital shaker (150 rpm) and eventually collected by centrifugation at 5000 rpm in a Sorvall H6000A/HBB6 swinging-bucket rotor for 15 min at 4 °C.

Bacterial pellet was re-suspended in 100 mL of the lysis buffer (20 mM Tris-HCl pH 7.5, 200 mM NaCl, 10 mM imidazole, 10 mM 2-mercaptoethanol, 1 mM phenylmethylsulfonyl fluoride (PMSF), 0.02 µM pepstatinA, 0.02 µg/ml chymostatin, 0.006 µM leupeptin, 20 µM benzamidine hydrochloride), incubated with lysozyme (50 µg/mL; Roth) for 30 min in a cold cabinet with head-over-tail rotation, and then broken in the EmulsiFlex-C3 High Pressure homogenizer at 1500 Bar. Homogenate was centrifuged in a Sorvall WX ULTRA SERIES ultracentrifuge (F37L rotor) at 33000 rpm for 45 min at 4°C.

The extract (supernatant after high-speed ultracentrifugation) was used for protein purification using the ÄKTA Xpress system (GE Healthcare), employing nickel affinity chromatography on the 5 mL column compatible with ÄKTA, which was manually filled with Ni-NTA Superflow resin (Qiagen). The column was equilibrated with 25 mL of low-salt (LS) buffer (20 mM Tris-HCl pH 7.4, 200 mM NaCl, 10 mM imidazole, 10 mM 2-mercaptoethanol) prior to extract loading. After protein binding, the resin was sequentially washed with 40 mL of LS buffer,

25 ml of high-salt (HS) buffer (20 mM Tris-HCl pH 7.4, 1 M NaCl, 10 mM imidazole, 10 mM 2-mercaptoethanol), and again with 20 mL of LS buffer. Bound proteins were recovered by elution with 30 mL of buffer E (50 mM Tris-HCl pH 7.4, 200 mM NaCl, 300 mM imidazole, 10 mM 2-mercaptoethanol). Further protein purification was achieved by performing size-exclusion separation of 5 mL of the eluate from the affinity chromatography step on a Hiload 16/60 Superdex S200 column (GE Healthcare), with the use of 1.2 column volumes of gel-filtration (GF) buffer (Tris-HCl pH 7.4; 150 mM NaCl), followed by ion-exchange chromatography on a Resource Q 1 mL column (GE Healthcare), using linear gradient of NaCl (150 mM-1M) in Tris-HCl pH 7.4, 1 mM DTT.

Two fractions corresponding to the maximum of A280nm absorbance were collected after ion-exchange separation, pooled together, mixed with glycerol (30% v/v), aliquoted, snap-frozen in liquid nitrogen and stored at -80 °C. Purified *Tt*Xrn1 was inspected in 10% SDS-PAGE stained with Coomassie Brilliant Blue R-250, along with commercial yeast Xrn1 (NEB; M0338) as a positive control (data not shown). PageRuler Prestained Protein Ladder, 10 to 180 kDa (ThermoScientific), was used as a molecular weight marker during electrophoresis.

Enzyme specificity towards 5'-monophosphorylated termini was tested by analyzing efficiency of degradation of synthetic 30-mer oligoribonucleotide substrates (5'-ACUCACUCACUCACCAAAAAAAAAAAAACC-3') bearing fluorescein amidite (FAM) at the 3'-end, and either 5'-monophosphate (5'P-RNA30-FAM-3'), 5'-hydroxyl (5'HO-RNA30-FAM-3') or 5'-Gppp (5'Gppp-RNA30-FAM-3') in 1x NEBuffer 3 (NEB; B7003), in 20% denaturing polyacrylamide gels, followed by fluorescence scanning in Typhoon™ FLA 9500 biomolecular imager. Furthermore, the ability to eliminate 18S and 25/28S ribosomal RNAs from total yeast/human RNA samples was examined by treating 1 µg of respective RNA with TtXrn1 for 30 and 60 minutes and running degradation products in 1% agarose gel in 1x TBE containing ethidium bromide. In all biochemical analyses, parallel reactions were carried out using equivalent amounts of commercial Xrn1 as positive control.

1.3. Xrn1 *in vitro* digestion of the pA$^+$ fraction

To remove uncapped mRNAs the 35 ng of pA$^+$ fraction was digested with home-made *Thermothelomyces (Myceliophthora) thermophilus* Xrn1 *in vitro* at 37 °C in Neb3 buffer (B7003S) for 1 hour in a total volume of 20 µl. The sample was then inactivated 10 min at 80 °C, and the RNA was extracted and precipitated as described above.

1.4. Reverse transcription and quantitative PCR analysis

cDNA synthesis was done using SuperScript IV reverse transcriptase from LifeTechnologies (18090050). The reaction mix contained random hexamers (final concentration 2.5 ng/µl), oligo-dT$_{(18)}$ (final concentration 5 µM) as primers and 0.5 mM dNTPs. 1 µg of total RNA or up to 100 ng of pA$^+$ fraction was used as a template and denatured together with oligo-dT and random hexamers for 5 min at 65 °C. After cooling the sample on ice for 2 min, the reaction mix was complemented with buffer, enzyme and RiboLock RNase inhibitor (LifeTechnology; EO 0382) and incubated for 20 min at room temperature and next for 40 min at 50 °C. The enzyme was inactivated for 10 min at 80 °C. cDNA was diluted to 400 µl. 2 µl of cDNA was used in a total of 5 µl reaction mix for qPCR analysis with commercial SYBR reaction mix [Platinum SYBR™ Green qPCR SuperMix-UDG from LifeTechnology (11733046) or RT qPCR Mix SYBR from A&A BIOTECHNOLOGY (2008-1000)] on a LightCycler LC480 Roche apparatus. Data were extracted using the 2nd derivative max method. Oligonucleotide sequences are indicated in Appendix Table S2.

1.5. RNaseH digestion of *RPS5* 3'end

20 µg of total RNA was mixed with 2 µM RNaseH-targeting oligonucleotide (5'-GGCCAAAGTTTCAGCAATGGTC-3') in annealing buffer (50 mM Tris-HCl, 50 mM KCl, pH 8.3) in a total volume of 12 µl and incubated for 2 min at 85°C to be then slowly cooled to 37°C. Where indicated, 2 µM of oligo-$dT_{(18)}$ oligonucleotide (5'-TTTTTTTTTTTTTTTTTT-3') was also included in order to target pA-tail trimming by RNaseH. Next, 8 µl of a mix preheated to 37°C and containing 2.5 U of RNase H (NEB #M0523; New England Biolabs), 2.5× RNase H reaction buffer (1× buffer: 50 mM Tris-HCl, 75 mM KCl, 3 mM MgCl2, 10 mM DTT at pH 8.3), 25 mM DTT, and 4 U RiboLock RNase inhibitor (Thermo Scientific) was added and incubated for 30 min at 37°C. Subsequently, RNA was precipitated by the addition of 100 µl absolute ethanol and 20 µl solution containing 600 mM sodium acetate (pH 5.3), 10 mM EDTA, 5 µg tRNA, and 5 µg glycogen and incubated at −20°C. Sample pellets were washed with 70 % ethanol and resuspended in RNA loading buffer (formamide with 10mM Tris-HCl pH 8.0, 5 mM EDTA, 0.02 % xylene cyanol).

1.6. Northern blotting

RNA samples were separated on 6% urea-polyacrylamide gels by electrophoresis, transferred to Hybond-N+ membrane (GE Healthcare), and hybridized over-night at 50 ˚C in ULTRA-Hyb Oligo Hybridization buffer (Invitrogen AM8663) with 1 pmol of 5' terminally 32P-labelled DNA oligonucleotide probe for RPS5 (5'-CTTAACGGTTAGACTTGGCAACACGTTCCAATTCATCCTTCTTCTTGATAGCGTAAGAAG-3'). The membrane was washed four times with 2XSSC buffer (300 mM NaCl, 30 mM trisodium citrate, pH 7.0) containing 0.5% SDS, each time rotating for 30 min at 42 ˚C and exposed to phosphorimager screen. The upper part of the membrane was subsequently cut out, hybridized with a labeled DNA probe for SCR1 (5'-GGTCACCTTTGCTGACGCTGG-3'), and washed and exposed as above. Phosphorimager scans were processed and quantitated with ImageJ software. The indicated pA tail lengths were approximated from DNA size markers.

1.7. Bulk pA-tail length analysis

500 ng of oligo(dT)-selected poly(A)+ RNA was subjected to 3'-end labeling in 20 µl of reaction mixture containing: 2 µl of 10x Reaction Buffer for T4 RNA Ligase (Thermo Scientific), 2 µl of 10 mM ATP, 2 µl of 100 mM DTT (Invitrogen), 2 µl of 100% DMSO (Thermo Scientific), 0.5 µl of RiboLock RNase Inhibitor (40 U/µl; Thermo Scientific), 2 µl of T4 RNA Ligase 1 (EL0021; Thermo Scientific) and 1.5 µl of [5'-32P]pCp (3000Ci/mmol, 10mCi/ml; Hartmann Analytic), for 1 hour at 37 °C. Enzyme was heat-inactivated at 95 °C for 5 min. Labeled RNA was digested with 0.5 µg of RNase A and 10 U of RNase T1 (both from Thermo Scientific) for 20 min. at 37 °C. Reaction was stopped by addition of an equal amount of formamide loading dye (90% formamide in 1xTBE, 0.03% xylene cyanol, 0.03% bromophenol blue) and incubation at 95 °C for 5 min, followed by flash freezing in liquid nitrogen. 5 µl of the sample was run in denaturing 12% sequencing polyacrylamide/8 M urea/1xTBE gel. 3'-end labeled 20 nt-, 31 nt- and 51 nt-long synthetic RNA oligonucleotides ($CCCCACCACCAUCACUUA_3$, $CCCCACCACCAUCACUUA_{14}$, $CCCCACCACCAUCACUUA_{34}$; all from Future Synthesis) were run in parallel to the labeled pA-tails as molecular size markers. Following electrophoresis, the gel was transferred onto Whatman 3MM filter paper, dried at 80 °C for 2 hours under vacuum, and exposed overnight to a PhosphorImager screen (FujiFilm), which was scanned using a FLA 9000 scanner.

1.8. Consideration regarding estimation of the modified gamma parameters and specific guidelines for adaptation of the R code for other datasets.

The classical gamma distribution properties enable the estimation of the mean, variance, and mode from the gamma parameters. Likewise, those parameters can be devised from the known experimental mean and variance. The modified gamma distribution does not inherit those properties, and the estimation of its parameters is best performed using the Nonlinear Least Squares procedure, which we implemented in R (Appendix section 5 and Mendeley data: doi: 10.17632/2j3hh37zzs.1). The procedure is compatible with the Mex67-depletion dataset only (results listed in Appendix Table S3 and Mendeley data) and its further use requires adaptation to the particularities of novel datasets. The R code assumes that the left arm of the distribution (representing the shortest pA-tails) is fixed and dictated by the $tanh(\beta * N_A)$ function (where N_A represents the pA-tail lengths from 1 to X and β is a fixed value). In the case of the Mex67-AA dataset $\beta = 0.096$ and the course of the function is presented in Figure 4A. The β parameter can be adjusted manually to best represent the actual experimental datasets but should remain equal for the entire data collection. While the modified gamma parameters are not linked to the distribution mean and variance, one has to bear in mind that the *γ_rate* is nearly identical to the slope factor of the pA-tail distribution represented in the log-linear scale (Figure 4J). Therefore, having calculated *γ_rate* and after having manually adjusted β to the dataset collection, the *γ_shape* parameter can also be fitted incrementally, without the need to use the R code.

The attached R code also performs simulation of deadenylation with the devised α scaling factor, as described in the Methods section.

The general limitation of the modified and classical gamma distributions is that they cannot represent bi-modal distributions. However, such experimental distributions for budding yeast datasets are scarce, mainly due to the lack of cytoplasmic adenylases, but are quite common in other eukaryotes. Therefore, we recommend that the modified gamma modeling be used on budding yeast datasets exclusively.

1.9. Bioinformatic analyses

Data analysis in RStudio. Unless stated otherwise data analysis was performed in RStudio (version 1.2.5033 for Mac, 4.2.3 for Windows or 2024.042 build 764 for Windows; RStudio Team, 2019; Posit team, 2024 respectively). Graphs were prepared either using the standard RStudio build-in commands or using ggplot2 package.

Computation of correlation coefficients using Rstudio. Correlation coefficients presented in this study were computed using rstatix package (0.7.2) in Rstudio version 2024.042 build 764. The function used was cor_test performed using Spearman's correlation test with a 0.95 confidence interval and a two-sided correlation significance test.

Binning transcripts by pA-tail length. For each set of samples, transcripts were divided into non-overlapping bins based on their median pA-tail lengths (binwidth=5). Each bin contained transcripts represented by over 30 reads. Then, for each condition and poly(A) length bin, the number of transcripts in each bin was plotted (only transcripts detected in each dataset were included).

Binning reads by pA-tail length. For each set of samples, reads were divided into bins of multiple of 5 pA-tails length. Then for each condition and poly(A) length bin read abundances (normalized to sequencing depth) were plotted.

Gene ontology analysis. Gene ontology analysis was performed using the BioMart R package based

on goslim terms. Only transcripts represented by 10 reads in each biological repeat of the chase experiment on Mex67-AA were included in the GO-term analysis. As for all other analyses the 'mean_mean' decay and deadenylation coefficients were used.

1.10. Description of dataset quality control and considerations regarding the estimation of decay and quantile deadenylation coefficients

General quality control metrics. As specified in the Methods section, the Nanopolish 0.13.2 polya function was used to estimate the quality of pA-tail length estimation, and only the reads flagged as PASS were taken for further analyses. The Expanded View Dataset 2 lists the DRS sequencing runs along with the total library read count performed for this study. The Appendix Table S4 lists the PASS counts number for libraries other than the chase experiments, whereas the bar plots in Appendix Figure S3A show the read number and quality distribution in each chase data point. Prior to further analyses, DRS replicates were compared for reproducibility of individual transcript count and mean pA-tail length by producing a Spearman rho correlation coefficient for all reads and mRNAs transcriptionally repressed during heat stress, including RPG mRNAs (Appendix Table S4). Such in-depth analysis for the chase DRS datasets is specified in the subsequent section. Overall, the mRNA abundance was reproduced with a Spearman rho factor within the range of 0.8 to 0.99. The reproducibility of mean pA-tail length for all mRNA ranged from 0.25 to 0.6, which, as expected, strongly depended on the sequencing depth. Importantly, for highly abundant RPG mRNAs, which were the main functional group of mRNAs analyzed in our study, the mean pA-tail length was replicated with a Spearman rho coefficient oscillating between 0.73 and 0.99. Those parameters indicated that the obtained data was of good quality, especially for RPG mRNAs, and could be used to model mRNA decay and deadenylation dynamics.

Chase datasets quality control and modeling strategy. Expanded View Dataset 2 lists the number of reads in each chase library, regardless of quality, while Appendix Figure S3A divides those counts into quality classes for each chase dataset sample.

The high reproducibility between the dataset biological replicates was crucial for the estimation of deadenylation and decay rates. Before commencing modeling the data was visually inspected in order to assess the reproducibility of the time-dependent phenotypes associated with each mutant (Mex67-AA) or growth condition (wild-type under heat stress at 37°C or thiolutin at 25°C). For each chase sample Appendix Figure S3B shows a scatterplot comparing the log2 mRNA abundance to mean pA-tail length. As previously observed (Tudek et al, 2021), in all control samples the mean pA tail length was inversely correlated with mRNA abundance. Specifically, mRNAs expressed at high levels, primarily the ribosomal protein-encoding mRNAs (RPG mRNAs) indicated by red dots, exhibited the shortest mean pA-tails.

During the Mex67-AA depletion at 25°C (see Appendix Figure S3B), we observed a gradual reduction in the mRNA mean pA-tail lengths and a decrease in abundance of most transcripts. As stated in the main text (in reference to Figures 1B, 2A, and EV1K), the terminal time-points of each chase experiment were excluded from subsequent deadenylation modeling. It is because for those samples, we observed an increase in mean pA-tail length, which was likely an effect of hyperadenylation occurring on a few nuclear mRNAs in the Mex67-depleted cells (Jensen et al., 2001). These hyperadenylated species are scarce compared to the cytoplasmic mRNA fraction. However, with significant loss of cytoplasmic mRNAs, they become dominant over time and thus strongly affect the estimation of the mean pA-tail length.

Heat stress at 37°C induced transcriptomic changes distinct from those observed during the Mex67-depletion chase at 25°C (refer to Appendix Figure S3B). Most mRNAs did not exhibit significant changes in abundance or mean pA-tail length. However, the group of transcripts

identified by Vinayachandran et al (2018) as being transcriptionally silenced during the acute stress response underwent substantial changes in both abundance and adenylation status (blue dots for non-RPG and red dots for RPG mRNAs). These changes were indicative of deadenylation and decay. Despite this, a notable recovery in mean pA-tail length and mRNA abundance was observed at the final time points of each chase experiment (18-20 minutes), likely due to the resumption of transcription in this group of mRNAs. Consequently, these time points were excluded from deadenylation modeling, as marked in Figure 6A.

Cell treatment with thiolutin at 25°C qualitatively resembled the events occurring during heat stress response (Appendix Figure S3B). Specifically, there was a gradual decrease in both the abundance and mean pA-tail length of mRNAs transcriptionally silenced during heat stress, including RPG mRNAs (Vinayachandran et al, 2018). In contrast, the abundance and pA-tail length of other transcripts remained unchanged. Given this, we conclude that thiolutin at the low concentration used in our experiments, triggers a transcriptional response akin to reaction to heat stress, as previously suggested by Adams and Gross (1991). Notably, no recovery in pA-tail length or mRNA abundance was observed in the terminal chase data points (20 minutes and beyond). Therefore, all samples were included in the deadenylation and decay modeling.

The next quality control step involved assessing the reproducibility of mRNA abundance and pA-tail length estimations across each replicate control sample (Appendix Figure S4A). This focus on control samples was justified due to the dynamic changes in mRNA levels and mean pA-tail length observed in the later chase data points. Read counts among individual samples correlated strongly, with Spearman's rho coefficients ranging from 0.85 to 0.96, a level of reproducibility comparable to other sequencing techniques such as Illumina. The reproducibility of mean pA-tail lengths for the entire transcriptome had rho values between 0.26 and 0.49 (black dots). However, when restricting the analysis to datasets containing at least 10 reads per transcript, the rho coefficient increased to a range of 0.52 to 0.70 (grey dots). Notably, for highly expressed RPG mRNAs, this coefficient ranged from 0.69 to 0.94. These observations prompted us to conduct deadenylation modeling exclusively on datasets with at least 10 reads per transcript. This also indicated that any estimations of deadenylation rates for RPG mRNAs are highly reliable, regardless of library size, as these transcripts are typically represented by many dozens to hundreds of reads individually.

Next, to assess the reproducibility of decay and deadenylation rate estimates across replicates, we performed a trial calculation for the Mex67-AA chase dataset (Appendix Figure S4B). To this end, we estimated the decay and deadenylation coefficient for each replicate separately using the given replicate control sample as a reference. Therefore, the decay factor was estimated from five samples in each replicate, while the deadenylation factor was estimated from four (for replicate A and B) or three (for replicate H) samples (Appendix Figure S4C, calculation setting presented in the top panel). As described in the Methods section and the main text, the exponential deadenylation factor was calculated separately from the 75th, 80th, 85th, 90th , and 95th quantiles and then averaged. Next, we compared the decay and deadenylation coefficients across replicates. The decay coefficient demonstrated high reproducibility, with Spearman's rho values ranging between 0.65 and 0.79 across all mRNAs and between 0.72 and 0.84 for transcripts represented by at least 10 reads. The Spearman's rho correlation coefficients for the quantile deadenylation rate were lower than those for decay, but this outcome was anticipated due to the greater complexity of estimation of pA-tail length statistics, as previously discussed. For mRNAs represented by at least 10 reads, the rho values were 0.52, 0.47, and 0.38. The lowest correlations were observed with replicate H, which had the poorest overall read count and three instead of four time points used for modeling deadenylation. Notably, when considering only the abundant ribosomal protein-encoding mRNAs (RPG mRNAs), the correlation was substantially stronger across all replicates, with rho values ranging from 0.60 to 0.74. We also performed the same

calculations on the thiolutin and heat stress chase experiments. For this analysis, we narrowed the datasets to 517-519 transcriptionally down-regulated mRNAs described by Vinayachandran et al (2018). We observed a high correlation of decay (0.66-0.81 for heat stress and 0.75 for thiolutin) and deadenylation rates (0.42-0.7 for heat stress and 0.66 for thiolutin) across all replicates. In summary, we demonstrated that the three chase datasets exhibited strong reproducibility of key phenotypic features, including mRNA abundance and mean pA-tail length. Furthermore, these datasets proved effective for calculating exponential decay and deadenylation coefficients, yielding highly satisfactory results.

The quality of deadenylation and decay rate estimation depends on the sequencing depth of the obtained datasets and the number of time-points. Therefore, to enhance the modeling power, we increased the number of samples used to estimate decay and deadenylation coefficients. This was achieved by treating each time point in the biological replicate chase dataset as a control for the consecutive time-points (Appendix Figure S4C, compare modeling strategies shown in the the top and bottom schemes). Analyzing the Mex67-AA chase dataset, we encountered a challenge. To observe cytoplasmic decay events, it was essential to fully inhibit new mRNA synthesis after efficient Mex67 depletion. This time delay could potentially set the control samples considerably apart from the other time points in the dataset, introducing bias in estimates derived from the control sample alone (Appendix Figure S4C). To address this issue, the precise onset of the Mex67-depletion phenotype was calculated during the modified gamma modeling and estimated to be 2.67 min. For the heat stress response, experimental evidence from RNA polymerase II ChIP assays demonstrated that transcriptional silencing of both RPG and non-RPG mRNAs occurs within 3 minutes (Vinayachandran et al, 2018). Therefore, we assumed the time delay between the onset of the phenotype and transcriptional shutdown to be negligible, implying that transcriptional shut-down happens almost instantaneously following a rapid temperature shift. The correction applied increased the modeling power, especially for the deadenylation rate calculations.

After establishing the uniformity of the chase replicates, we conducted a bulk estimation of decay and deadenylation coefficients from all replicates. In case of the deadenylation rate, for each quantile separately (95th, 90th, 85th, 80th 75th, 50th, 15th, 10th and 5th) the mean, median and standard deviation of the deadenylation rate was calculated. Next, using the 95th-75th quantiles the mean and medians were calculated from respective intermediate values, and listed along with a standard deviation in Expanded View Dataset 1. Throughout the main manuscript the deadenylation rate '*(...)mean_mean*' values were used, including for calculation of the terminal adenosine half-lives. Next, we compared the standard deviation of the decay and deadenylation coefficient estimate calculated for each chase experiment (Appendix Figure S4D). This showed that the value of the standard deviation was substantially lower (up to 2-fold) than the decay rate for the heat stress and thiolutin chase experiments. In the case of the Mex67-AA chase the relatively high standard deviations for the decay rate estimate encouraged further investigations. We cross-compared our mRNA half-life estimates to four published datasets (see text related to Figure 1D.-E. and Appendix Figure S1C.-D.; Miller et al, 2011; Neymotin et al, 2014; Chan et al, 2018; Presnyak et al, 2015) and obtained good correlations with independent studies based on metabolic 4tU-labelling in otherwise wild-type cells (rho = 0.74 for Miller et al, 2011; rho = 0.62 for Chan et al, 2018 and rho = 0.44 for Neymotin et al, 2014). Lower reproducibility was observed in a chase dataset obtained through heat-inactivation of a strain encoding a mutated RNA polymerase II (rho = 0.31, Presnyak et al., 2015). This suggests that the relatively high standard deviation of the decay rate estimate in the Mex67-AA chase is not concerning in terms of data quality, as our estimates are comparable to those previously published. In contrast, the standard deviations of our deadenylation rate estimates were much lower than the coefficient itself (Appendix Figure S4D). We validated our deadenylation rate estimates by comparing the decay functions drawn using the obtained estimates with the

experimental quantile pA-tail length values used for the calculations. For this purpose, we selected twelve mRNAs characterized by varying quantile deadenylation rates (slow or fast deadenylation; see Figure EV4A). This analysis revealed that the quantile deadenylation rates accurately predicted the changes in experimental quantile pA-tail lengths. Coupled with the low standard deviation of our quantile deadenylation rate estimates, this indicates the reliability of the obtained coefficients.

2. APPENDIX TABLES

Appendix Table S1. Yeast strains used in this study.

Name	Identifier	Source
Wild-type W303:<br>MAT A; *leu2-3,112; trp1-1, can1-100; ura3-1, ade2-1; leu2-3,112; his3-11,15*	T. H. Jensen collection Y159; A. Tudek collection Y13	Euroscarf Cat#BMA64-1A
Dcp2-AID-FLAG:<br>As W303, *OsTIR1:URA3, DCP2::mAID-6flag::HygR*	T. H. Jensen collection Y3798; A. Tudek collection Y45	This study
Dcp2-AID-FLAG Mex67-AA:<br>As W303; *tor1-1 fpr1::LEU RPL13-2xFKBP12::loxP-TRP1-loxP OsTIR::URA MEX67:FRB::kanMX DCP2-mAID-6FLAG::HygR*	T. H. Jensen collection Y3927; A. Tudek collection Y62	This study
Pab1-AID-6HA; Mex67-AA:<br>As W303, *tor1-1 fpr1::LEU RPL13-2xFKBP12::loxP-TRP1-loxP OsTIR::URA MEX67-FRB::KANmx PAB1-mAID-6HA::HygR*	T. H. Jensen collection Y3879	This study
dhh1Δ:<br>as W303, *DHH1::URA3ca*	A. Tudek collection Y68	This study
lsm1Δ:<br>as W303, *LSM1::URA3ca*	A. Tudek collection Y69	This study
Mex67-AA:<br>as W303, *tor1-1 fpr1::NAT RPL13-2xFKBP12::TRP1 MEX67-FRB::kanMX6*	T. H. Jensen collection 2618; A. Tudek collection Y39	Euroscarf from Haruki et al, 2018: Cat#HHY182
Wild-type to match Tucker et al. (2001) strains:<br>*MATa leu2-3,112 trp1-1 ura3-52 his4-539 cup1::LEU2/PGK1pG/MFA2pG*	R. Parker collection yRP840; A. Tudek collection Y79	Tucker et al, 2001
pan2Δ:<br>*MATa leu2-3,112 trp1-1 ura3-52 his4-539 cup1::LEU2/PGK1pG/MFA2pG pan2Δ::URA3*	R. Parker collection yRP1619; A. Tudek collection Y80	Tucker et al, 2001
ccr4Δ:<br>*MATa leu2-3,112 trp -1 ura3-52 his4-539 cup1::LEU2/PGK1pG/MFA2pG ccr4Δ::NEO*	R. Parker collection yRP1616; A. Tudek collection Y81	Tucker et al, 2001
pan2Δ ccr4Δ:<br>*MATa leu2-3,112 trp -1 ura3-52 his4-539 cup1::LEU2/PGK1pG/MFA2pG ccr4Δ::NEO pan2Δ::URA3*	R. Parker collection yRP1620; A. Tudek collection Y82	Tucker et al, 2001
Xrn1-AID:<br>as W303, His+ tor1-1 fpr1::LEU RPL13-2xFKBP12::loxP-TRP1-loxP HIS+ OsTIR::URA3 XRN1::AID::KANmx	T. H. Jensen collection Y3688; A. Tudek collection Y43	Tudek et al. 2018

Appendix Table S2. Oligonucleotides used in this study

**Name**	**Sequence**	**Source**
25S_rRNA_FWD	ATTCCCACTGTCCCTATCTACT	Custom (LifeTechnology)
25S_rRNA_REV	CTTGGCTGTGGTTTCGCT	Custom (LifeTechnology)
HHF1_FWD	ACTGCCCGGTTTTTCTTCT	Custom (LifeTechnology)
HHF1_REV	CCTAAACCCGCTATAATACACTCAT	Custom (LifeTechnology)
Hsp104_FWD	AGCTGAAGAATGTCTGGAAGT	Custom (LifeTechnology)
Hsp104_REV	CGTCATCACCTAACGTGTCA	Custom (LifeTechnology)
Markers for bulk RNA pA-tail length	CCCCACCACCAUCACUUA(3)<br>CCCCACCACCAUCACUUA(14)<br>CCCCACCACCAUCACUUA(34)	Future Synthesis
oligo-dT(18)	TTTTTTTTTTTTTTTTTT	Custom (LifeTechnology)
Random hexamers		Invitrogen (48190-011)
Rpl21B_FWD	ACAGATCTCGTACACGTTACA	Custom (LifeTechnology)
Rpl21B_REV	CGACAATGTCACCAACCT	Custom (LifeTechnology)
Rpl36A_FWD	AAGGTAAGAAGGTCACTAGCA	Custom (LifeTechnology)
Rpl36A_REV	GTTGGAAGCAGCACCTTT	Custom (LifeTechnology)
RPL28_FWD	CGGTAAAGGTCGTATCGGT	Custom (LifeTechnology)
RPL28_REV	TCCATGTTAATTCTGTGGTGATGT	Custom (LifeTechnology)
RPS5 Northern blot probe	CTTAACGGTTAGACTTGGCAACACG<br>TTCCAATTCATCCTTCTTCTTGATAG<br>CGTAAGAAG	Custom (LifeTechnology)
RPS5 RNaseH-targeting oligonucleotide	GGCCAAAGTTTCAGCAATGGTC	Custom (LifeTechnology)
RPS13_FWD	ATTTCTTCTTCTGCTATTCCATACTC<br>T	Custom (LifeTechnology)
RPS13_REV	CCCTTTCTCGCGTACTTGAC	Custom (LifeTechnology)
SSA4_FWD	AAATTGTACTTGTTGGTGGTTCA	Custom (LifeTechnology)
SSA4_REV	GGGTTAATCGAACGGTTTGG	Custom (LifeTechnology)
SCR1 Northern blot probe	GGTCACCTTTGCTGACGCTGG	Custom (LifeTechnology)
TDH3_FWD	CTCTCACTCTTCCATCTTCGAT	Custom (LifeTechnology)
TDH3_REV	CGTACCAGGAGACCAACTT	Custom (LifeTechnology)

Appendix Table S3. List of the *γ_shape* and *γ_rate* parameters of the modified gamma distribution*.

distribution	replicate	Name	time	*γ_shape*	*γ_shape* standard error	*γ_rate*	*γ_rate* standard error
all	A	ORFs_repA	0	4,7382963016	0,1701733983	0,0443559959	0,0010031239
all	A	ORFs_repA	12	4,5458072251	0,1709085326	0,0762571365	0,0020174607
all	A	ORFs_repA	14	4,6083475182	0,168448713	0,0815975926	0,002133893
all	A	ORFs_repA	16	4,8639966029	0,1753657642	0,087245794	0,0022913976
all	A	ORFs_repA	30	5,2342823366	0,1819466638	0,1141303491	0,0030768485
all	A	ORFs_repA	8	4,3443018564	0,1549567484	0,0655559453	0,0015840391
all	B	ORFs_repB	0	3,9477063331	0,0911411231	0,0511873469	0,0007594507
all	B	ORFs_repB	10	4,2942553682	0,1421460508	0,071834078	0,001648011
all	B	ORFs_repB	12	4,5649936518	0,1616177987	0,075070907	0,0018626591
all	B	ORFs_repB	14	4,6732992589	0,1589867501	0,0807358774	0,0019612077
all	B	ORFs_repB	20	4,9889139759	0,161404659	0,09254039	0,0022133365
all	B	ORFs_repB	4	4,198432916	0,1294216769	0,0522990157	0,0010384718
all	H	ORFs_repH	0	4,0679072475	0,0776561027	0,0548363985	0,0006835345
all	H	ORFs_repH	16	4,473338404	0,146948969	0,0982197119	0,0024128111
all	H	ORFs_repH	30	4,446216747	0,127695574	0,1256616164	0,0028422138
all	H	ORFs_repH	4	4,0473310821	0,0939395946	0,0600702287	0,000927981
all	H	ORFs_repH	60	3,6058199436	0,1301168254	0,145361458	0,0041971596
all	H	ORFs_repH	8	6,2086731339	0,3371068626	0,0634482699	0,0023046447
GAS1	A	GAS1_Mex67_repA	0	12,8878769438	2,2655815304	0,0265802005	0,0032441697
GAS1	A	GAS1_Mex67_repA	12	6,3919655705	1,3689190922	0,0547458302	0,0092779551
GAS1	A	GAS1_Mex67_repA	14	6,2650623134	0,6454890465	0,0697053807	0,0050987687
GAS1	A	GAS1_Mex67_repA	16	8,9633308236	0,7226478602	0,0852400776	0,0051341791
GAS1	A	GAS1_Mex67_repA	30	5,9157295834	0,9502770809	0,1229971594	0,0158369891
GAS1	A	GAS1_Mex67_repA	8	9,8906015085	2,0648593467	0,038581405	0,0060285048
GAS1	B	GAS1_Mex67_repB	0	12,6040841543	1,4189279791	0,0348463035	0,0025265516
GAS1	B	GAS1_Mex67_repB	10	10,5083794243	0,9226133331	0,0649782919	0,0039115551
GAS1	B	GAS1_Mex67_repB	12	9,6327202168	0,9413267313	0,0635621115	0,0041820153
GAS1	B	GAS1_Mex67_repB	14	8,0122699547	0,6846031221	0,0723561782	0,0042777458
GAS1	B	GAS1_Mex67_repB	20	7,5828969199	0,5517643955	0,0974706489	0,0053612539
GAS1	B	GAS1_Mex67_repB	4	14,067586699	2,2942563187	0,0328918473	0,003448606
HHF1	B	HHF1_Mex67_repB	0	6,5107924093	0,5834895098	0,0467976725	0,0027494249
HHF1	B	HHF1_Mex67_repB	10	5,1562236603	0,4261955779	0,0869064592	0,0054112257
HHF1	B	HHF1_Mex67_repB	12	4,2297377129	0,3584940978	0,0831228148	0,00519282
HHF1	B	HHF1_Mex67_repB	14	3,9341234102	0,4833755925	0,0819018723	0,0073712193
HHF1	B	HHF1_Mex67_repB	20	2,7006263757	0,5798381797	0,0668342626	0,0092484496

HHF1	B	HHF1_Mex67_repB	4	5,9981626059	0,579284607	0,0472671672	0,002942895
HHF1	A	HHF1_Mex67_repA	0	6,6145650838	1,0320558602	0,0291373227	0,0030225197
HHF1	A	HHF1_Mex67_repA	12	3,7479265145	1,3662826645	0,0709915031	0,0208753089
HHF1	A	HHF1_Mex67_repA	14	3,572616036	0,6737905223	0,0821874673	0,0121809386
HHF1	A	HHF1_Mex67_repA	16	5,3275541288	0,757245699	0,1021711043	0,0113029587
HHF1	A	HHF1_Mex67_repA	30	0,1297825643	0,5114497365	0,0067028791	0,0058751255
HHF1	A	HHF1_Mex67_repA	8	6,3802674481	1,1872879096	0,0573295013	0,0077858905
high	A	Mex67_high_abundance_ORFS_	0	4,9654002402	0,1430404473	0,0544502193	0,0010223591
high	A	Mex67_high_abundance_ORFS_	8	4,4507002021	0,1369398016	0,0700066827	0,00148884
high	A	Mex67_high_abundance_ORFS_	12	4,7092277065	0,1653948932	0,078703171	0,001967263
high	A	Mex67_high_abundance_ORFS_	14	4,7505973006	0,1662417261	0,0831457526	0,0020981125
high	A	Mex67_high_abundance_ORFS_	16	5,057551509	0,1753887568	0,088430846	0,002246691
high	A	Mex67_high_abundance_ORFS_	30	5,6661134788	0,1847040146	0,1179515794	0,0030100054
high	B	Mex67_high_abundance_ORFS_repB	0	4,1583258244	0,0848307042	0,0600664537	0,0008161981
high	B	Mex67_high_abundance_ORFS_repB	10	4,3737259612	0,1377950671	0,074463203	0,001645561
high	B	Mex67_high_abundance_ORFS_repB	12	4,65940655	0,1590682664	0,0769404313	0,0018574119
high	B	Mex67_high_abundance_ORFS_repB	14	4,7985933133	0,1616056564	0,0823170797	0,0019938044
high	B	Mex67_high_abundance_ORFS_repB	20	5,2350598403	0,1717914696	0,0943295448	0,0023023997
high	B	Mex67_high_abundance_ORFS_repB	4	4,3113343862	0,1094349881	0,0597594955	0,0010081333
low	A	Mex67_low_abundance_ORFS_	0	4,7934942737	0,2585077417	0,0361313324	0,0012129324
low	A	Mex67_low_abundance_ORFS_	8	4,2598497515	0,1874837742	0,0606844373	0,0017680253
low	A	Mex67_low_abundance_ORFS_	12	4,3086098793	0,1826785709	0,0728297541	0,002136068
low	A	Mex67_low_abundance_ORFS_	14	4,3769701007	0,1751196888	0,0791355528	0,0022357259
low	A	Mex67_low_abundance_ORFS_	16	4,5450343598	0,1770653555	0,0852472269	0,0023951438
low	A	Mex67_low_abundance_ORFS_	30	4,3424692286	0,1930820392	0,1056367641	0,00355708
low	B	Mex67_low_abundance_ORFS_repB	0	3,8488234017	0,1266423076	0,0427803314	0,0008794938
low	B	Mex67_low_abundance_ORFS_repB	10	4,1916359993	0,1522345815	0,0682804239	0,00168943
low	B	Mex67_low_abundance_ORFS_repB	12	4,4227910912	0,1683514236	0,0723216818	0,0019028602
low	B	Mex67_low_abundance_ORFS_repB	14	4,469556271	0,1584036705	0,0782098125	0,001955639
low	B	Mex67_low_abundance_ORFS_repB	20	4,5480351401	0,1499378985	0,0892115375	0,0021480054
low	B	Mex67_low_abundance_ORFS_repB	4	4,218966619	0,1793497749	0,0450593145	0,0012005542
non_RPG	A	Mex67_NON_RPG_ORFS_	0	4,9672842436	0,2041426011	0,0420164582	0,0010823057
non_RPG	A	Mex67_NON_RPG_ORFS_	8	4,5478286662	0,1737482453	0,0640898907	0,001648397
non_RPG	A	Mex67_NON_RPG_ORFS_	12	4,7113500016	0,1880873248	0,0745866179	0,0020824396
non_RPG	A	Mex67_NON_RPG_ORFS_	14	4,7899489509	0,1817291368	0,0800705223	0,0021630123
non_RPG	A	Mex67_NON_RPG_ORFS_	16	5,0501213504	0,1880011016	0,0859854215	0,0023234117
non_RPG	A	Mex67_NON_RPG_ORFS_	30	5,3781243432	0,1882079531	0,1125937656	0,0030494312

non_RPG	B	Mex67_NON_RPG_ORFS_repB	0	4,0641918842	0,1090397351	0,0484523351	0,0008273292
non_RPG	B	Mex67_NON_RPG_ORFS_repB	10	4,4557985875	0,1593752927	0,069907468	0,001720062
non_RPG	B	Mex67_NON_RPG_ORFS_repB	12	4,7420000963	0,1795998684	0,0732750569	0,001931857
non_RPG	B	Mex67_NON_RPG_ORFS_repB	14	4,8351224907	0,1729630996	0,0790030243	0,0020061326
non_RPG	B	Mex67_NON_RPG_ORFS_repB	20	5,1578102871	0,169904363	0,0910024898	0,0022076077
non_RPG	B	Mex67_NON_RPG_ORFS_repB	4	4,3683962591	0,1548699682	0,0498433958	0,0011278062
RPG	A	Mex67_RPG_ORFS_	0	4,3858961152	0,1058700093	0,0575355128	0,0009149546
RPG	A	Mex67_RPG_ORFS_	12	4,1553868064	0,1301502105	0,0845869745	0,0019155143
RPG	A	Mex67_RPG_ORFS_	14	4,1607554066	0,1346566299	0,0893391874	0,0021160282
RPG	A	Mex67_RPG_ORFS_	16	4,3703662408	0,1408138464	0,0938420789	0,0022411947
RPG	A	Mex67_RPG_ORFS_	30	4,8103893624	0,1690452851	0,1210655792	0,0033317169
RPG	A	Mex67_RPG_ORFS_	8	3,8644026949	0,1139602911	0,0739623327	0,0015281639
RPG	B	Mex67_RPG_ORFS_repB	0	3,8555028598	0,0582620036	0,0664051418	0,0006846856
RPG	B	Mex67_RPG_ORFS_repB	10	3,9489536256	0,0993100557	0,0818493924	0,0014775611
RPG	B	Mex67_RPG_ORFS_repB	12	4,1741445384	0,1156586365	0,0843256963	0,0016897826
RPG	B	Mex67_RPG_ORFS_repB	14	4,2886229838	0,1223996468	0,0897249208	0,0018799829
RPG	B	Mex67_RPG_ORFS_repB	20	4,531451744	0,1373959954	0,1005650055	0,0022971299
RPG	B	Mex67_RPG_ORFS_repB	4	3,9586374237	0,0731793719	0,0658968609	0,0008289751
RPL36A	A	RPL36A_Mex67_repA	0	2,8192824408	0,3875104317	0,0647922677	0,0062227307
RPL36A	A	RPL36A_Mex67_repA	12	1,3721045181	0,4193238798	0,0647006171	0,0138986434
RPL36A	A	RPL36A_Mex67_repA	14	1,6425974593	0,2100203444	0,0972620201	0,0087508354
RPL36A	A	RPL36A_Mex67_repA	16	1,5166212554	0,2149594425	0,0870663529	0,0083362171
RPL36A	A	RPL36A_Mex67_repA	30	1,7650534662	0,1940463142	0,145168502	0,0120251798
RPL36A	A	RPL36A_Mex67_repA	8	0,7462916293	0,3792584594	0,0445654237	0,0168873429
RPL36A	B	RPL36A_Mex67_repB	0	2,1049159955	0,2743369736	0,0703452894	0,0062238436
RPL36A	B	RPL36A_Mex67_repB	10	1,9638093588	0,2320665218	0,0882218348	0,0074217638
RPL36A	B	RPL36A_Mex67_repB	12	2,3951711021	0,303675421	0,0859376154	0,0078766261
RPL36A	B	RPL36A_Mex67_repB	14	2,1362480698	0,1726494221	0,104737701	0,0062705979
RPL36A	B	RPL36A_Mex67_repB	20	2,0780686213	0,2028633333	0,1074758501	0,0075829429
RPL36A	B	RPL36A_Mex67_repB	4	2,3159699391	0,2573549803	0,0817314839	0,0064354323
RPL4A	B	RPL4A_Mex67_repB	0	9,172571753	0,6554593614	0,0471791874	0,0021513514
RPL4A	B	RPL4A_Mex67_repB	10	9,2060871326	0,6338473303	0,0696616726	0,0033154902
RPL4A	B	RPL4A_Mex67_repB	12	9,6473512415	0,5408277986	0,0773711923	0,0030662237
RPL4A	B	RPL4A_Mex67_repB	14	8,9636686903	0,4260715937	0,0858034461	0,0029696257
RPL4A	B	RPL4A_Mex67_repB	20	7,5764018507	0,310144215	0,1031378864	0,0032820025
RPL4A	B	RPL4A_Mex67_repB	4	11,5571626981	0,9525045631	0,0490049967	0,0025948041
RPL4A	A	RPL4A_Mex67_repA	0	12,4593701276	0,9660117938	0,0480937052	0,0024011968
RPL4A	A	RPL4A_Mex67_repA	12	9,1933802778	0,8813587195	0,0786350812	0,0055350599

RPL4A	A	RPL4A_Mex67_repA	14	8,6831377247	0,5264877475	0,0911187785	0,0041281053
RPL4A	A	RPL4A_Mex67_repA	16	8,8569352177	0,3520108131	0,1137183769	0,00355103
RPL4A	A	RPL4A_Mex67_repA	30	8,9253033819	1,5156237934	0,1760605628	0,0258553617
RPL4A	A	RPL4A_Mex67_repA	8	8,0298469685	0,8059447231	0,055392504	0,0037789706

*a .csv file is deposited at Mendeley.

Appendix Table S4. Basic DRS data statistics for datasets other than the chase experiments.

Sample:	All PASS reads		mRNAs reads		Heat stress repressed mRNA reads		RPG mRNA reads	
	count number	rho: counts/ mean pA-tail	count number	rho: counts/ mean pA-tail	count number	rho: counts/ mean pA-tail	count number	rho: counts/ mean pA-tail
Pab1-AID control 1<br>Pab1-AID control 2	1461085<br>326593	0.93/0.42	1433042<br>320052	0.93/0.43	755069<br>167353	0.98/0.73	585883<br>129810	0.98/0.73
Pab1-AID 1h auxin 1<br>Pab1-AID 1h auxin 2	1118293<br>1145958	0.97/0.48	1096564<br>1123276	0.96/0.55	567206<br>540441	0.99/0.85	442538<br>421278	0.99/0.95
Pab1-AID 2h auxin 1<br>Pab1-AID 2h auxin 2	569602<br>1171693	0.93/0.42	554508<br>1148828	0.93/0.46	259303<br>543553	0.98/0.81	199356<br>415730	0.93/0.87
Control for Xrn1-AID and Dcp2AID<br>replicate 1<br>replicate 2	1136743<br>1195900	0.97/0.44	1104241<br>1172287	0.97/0.54	380041<br>416018	0.99/0.68	243639<br>266540	0.98/0.93
Dcp2-AID replicate 1<br>Dcp2-AID replicate 2	315388<br>687910	0.95/0.34	302813<br>662182	0.95/0.39	95793<br>169021	0.97/0.51	58419<br>92399	0.96/0.75
Xrn1-AID replicate 1<br>Xrn1-AID replicate 2	306564<br>737127	0.95/0.34	296792<br>713751	0.95/0.41	141424<br>340845	0.98/0.64	101982<br>243608	0.99/0.89
pan2Δ ccr4Δ 25°C rep1<br>*pan2Δ ccr4Δ* 25°C rep2<br>*pan2Δ ccr4Δ* 25°C rep3	428496<br>541867<br>424573	(0.94-0.93-0.94)/(0.26-0.25-0.26)	418937<br>528708<br>413829	(0.94-0.93-0.94)/(0.28-0.28-0.27)	187184<br>254563<br>196043	(0.99-0.99-0.99)/(0.64-0.66-0.68)	127163<br>174407<br>131024	(0.99-0.98-0.99)/(0.87-0.86-0.89)
pan2Δ ccr4Δ 37°C rep1<br>*pan2Δ ccr4Δ* 37°C rep2<br>*pan2Δ ccr4Δ* 37°C rep3	592954<br>156325<br>176421	(0.89-0.89-0.87)/(0.28-0.30-0.24)	581615<br>152947<br>172571	(0.89-0.88-0.88)/(0.30-0.31-0.24)	219324<br>61010<br>66955	(0.97-0.98-0.97)/(0.53-0.66-0.52)	144836<br>39698<br>42464	(0.97-0.97-0.98)/(0.8-0.76-0.76)
Mex67-AA 25°C rep1<br>Mex67-AA 25°C rep2	2184459<br>1118897	0.96/0.47	2141570<br>1098396	0.96/0.49	1081952<br>539068	0.99/0.83	756476<br>374611	0.96/0.91
Mex67-AA 37°C rep1<br>Mex67-AA 37°C rep2	841310<br>1415229	0.96/0.53	827924<br>1392269	0.96/0.56	295373<br>392041	0.97/0.66	202933<br>243062	0.97/0.77
Mex67-AA 37°C +rapamycin rep1<br>Mex67-AA 37°C +rapamycin rep2	1406000<br>1049059	0.88/0.41	1381734<br>1028633	0.89/0.43	750886<br>549364	0.97/0.45	576594<br>413477	0.97/0.83
WT 30°C replicate1<br>WT 30°C replicate2<br>WT 30°C replicate3<br>WT 30°C replicate4<br>WT 30°C replicate5<br>WT 30°C replicate6<br>WT 30°C replicate7<br>WT 30°C replicate8	457235<br>960094<br>644548<br>3844655<br>874196<br>525983<br>774954<br>596447	*(0.94-0.96-0.95-0.94-0.56-0.96-0.97-0.96)/(0.54-0.65-0.61-0.55-0.76-0.71-0.75-0.70)	447134<br>940138<br>633208<br>3745464<br>867826<br>515572<br>755067<br>581467	*(0.93-0.96-0.95-0.92-0.54-0.95-0.97-0.96)/(0.55-0.65-0.61-0.59-0.78-0.73-0.77-0.72)	244466<br>528309<br>348321<br>1605245<br>137717<br>293606<br>415717<br>311665	*(0.99-0.99-0.99-0.96-0.95-0.98-0.99-0.99)/(0.80-0.91-0.87-0.87-0.64-0.84-0.80-0.80)	178995<br>394153<br>255176<br>1175348<br>92547<br>223015<br>313297<br>230320	*(0.95-0.97-0.94-0.92-0.83-0.91-00.96-0.97)/(0.93-0.96-0.94-0.89-0.84-0.89-0.94-0.92)
ccr4Δ 30°C replicate 1<br>*ccr4Δ* 30°C replicate 2	459838<br>285056	0.79/0.49	449734<br>279388	0.77/0.52	205757<br>154516	0.89/0.80	137171<br>118949	0.51/0.86
pan2Δ 30°C replicate 1<br>*pan2Δ* 30°C replicate 2	311266<br>475953	0.93/0.37	304145<br>464686	0.92/0.37	167681<br>258399	0.99/0.59	121460<br>191672	0.98/0.88
pop2Δ 30°C replicate 1<br>*pop2Δ* 30°C replicate 2	287286<br>525820	0.95/0.50	279923<br>512622	0.94/0.55	122635<br>217714	0.98/0.84	87657<br>154100	0.98/0.90
WT 30°C<br>WT 30°C +Xrn1 *in vitro*	1291153<br>1090351	0.96/0.55	1251152<br>1042261	0.95/0.61	620428<br>550406	0.99/0.80	449596<br>402027	0.98/0.86

*due to the large number of replicates in this datasets the Spearman rho coefficients were calculated between a given replicate and the sum of all reads.

3. APPENDIX REFERENCES

Bilska, A., Kusio-Kobiałka, M., Krawczyk, P. S., Gewartowska, O., Tarkowski, B., Kobyłecki, K., Nowis, D., Golab, J., Gruchota, J., Borsuk, E., Dziembowski, A., Mroczek, S. (2020) Immunoglobulin expression and the humoral immune response is regulated by the non-canonical poly(A) polymerase TENT5C. Nat Commun. 11(1):2032. doi: 10.1038/s41467-020-15835-3.

Kadanoff, L. P. 2009. More is the Same; Phase Transitions and Mean Field Theories. Journal of Statistical Physics. 137 (5-6) , pp.777-797. DOI 10.1007/s10955-009-9814-1

Posit team (2024). RStudio: Integrated Development Environment for R. Posit Software, PBC, Boston, MA. URL http://www.posit.co/

RStudio Team (2019). RStudio: Integrated Development for R. RStudio, Inc., Boston, MA URL http://www.rstudio.com/

Tucker, M., Valencia-Sanchez, M. A., Staples, R. R., Chen, J., Denis, C. L., Parker, R. (2001) The transcription factor associated Ccr4 and Caf1 proteins are components of the major cytoplasmic mRNA deadenylase in Saccharomyces cerevisiae. Cell. 104(3):377-86. doi: 10.1016/s0092-8674(01)00225-2.

4. APPENDIX FIGURES

Appendix Figure S1

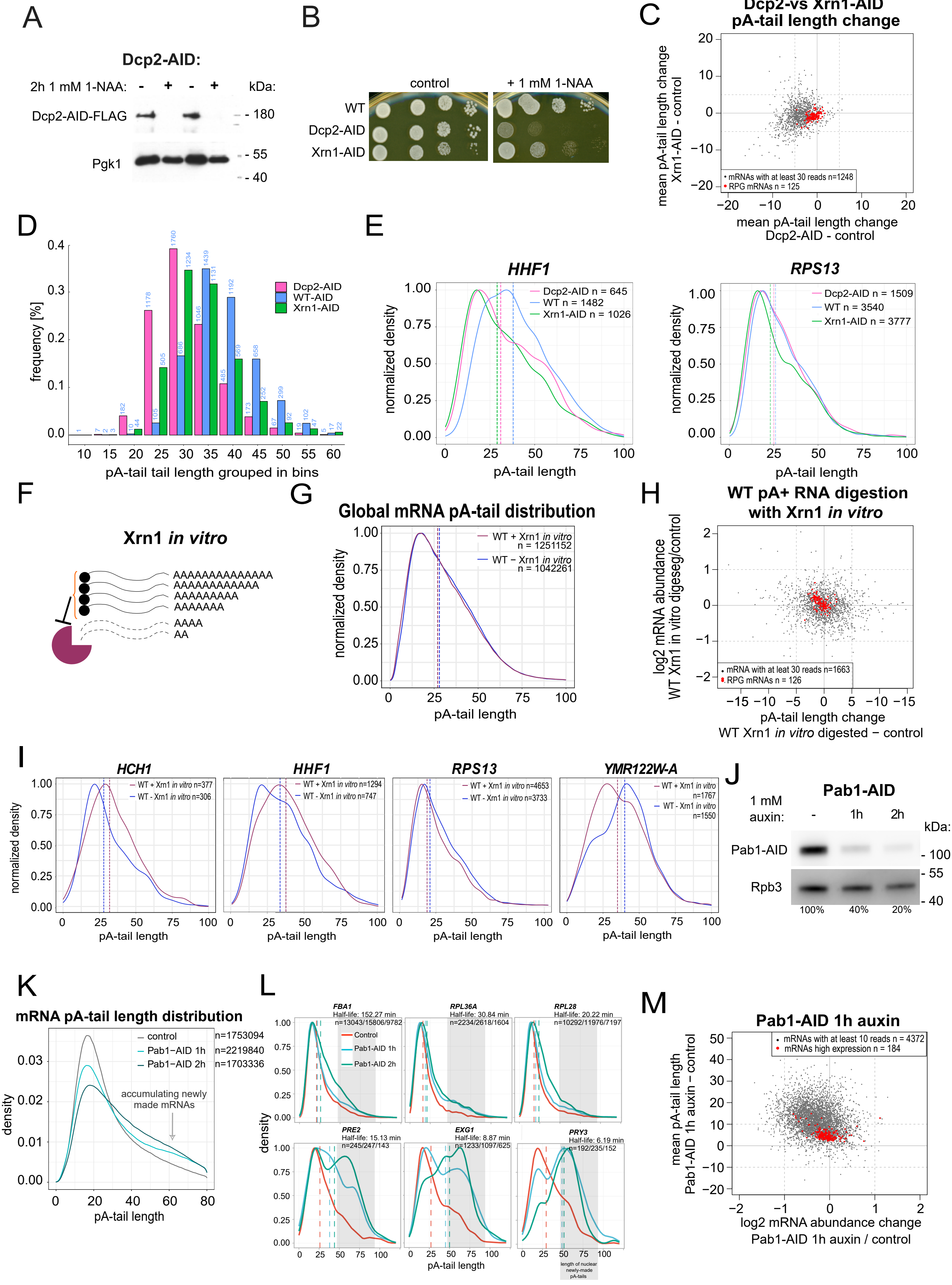

Appendix Figure S1. Assessment of the impact of Dcp2, Xrn1 and Pab1 on mRNA pA-tail lengths *in vivo*. **A.** Western blot shows the efficiency of depletion of Dcp2 using the AID system for 2 hours. PGK1 is used as a loading control. **B.** Growth test produced using 10-fold serial dilutions of Dcp2-AID and Xrn1-AID cells grown on a control or auxin-supplemented medium. **C.** Scatterplot compares the absolute change in mean pA-tail length in Dcp2- and Xrn1-depleted cells compared to control. **D.** Proportion of transcripts with specific median pA-tail lengths in designated value ranges presented for control, Dcp2- and Xrn1-depleted cells. **E.** pA-tail length distribution of *HHF1* and *RPS13* mRNAs in control and Dcp2- or Xrn1-depleted cells. **F.** Scheme shows that Xrn1 can only digest mRNAs devoid of a cap, potentially altering pA-tail distribution if transcripts are massively decapped only at a defined pA-tail length. **G.** Global pA-tail length distribution of coding transcripts in wild-type sample digested or not with Xrn1 *in vitro*. **H.** Absolute change in mean pA-tail length in relation to the log2 change in mRNA abundance in between a wild-type sample digested or not with Xrn1 *in vitro*. **I.** pA-tail length distributions of *HCH1*, *HHF1*, *RPS13* and *YMR122W-A* mRNAs in a control wild-type sample compared to the same sample digested with Xrn1 *in vitro*. **J.** Western blot shows depletion of Pab1 protein using the auxin-inducible degron for 1 and 2 hours. Rpb3 is shown as a loading control. The percentages shown below the Rpb3 loading control designate the estimated amount of Pab1-AID remaining in the sample relative to the control. **K.** Distribution of all coding transcript pA-tail lengths in control wild-type cells compared to 1 or 2 hours Pab1-depleted cells. **L.** pA-tail length distribution of *FBA1*, *RPL36A*, *RPL28*, *PRE3*, *EXG1* and *PRY3* mRNAs in control cells compared to strains depleted for Pab1 using the AID system for 1 or 2 hours. The mRNAs are ranked by half-life time. The number of reads that sum into each distribution is indicated on the panel with n='control'/'1h depletion'/'2h depletion'. **M.** Comparison of log2 change in mRNA abundance to absolute change in mean pA-tail length for cells depleted for Pab1 for 1 hour compared to wild-type control.

Appendix Figure S2

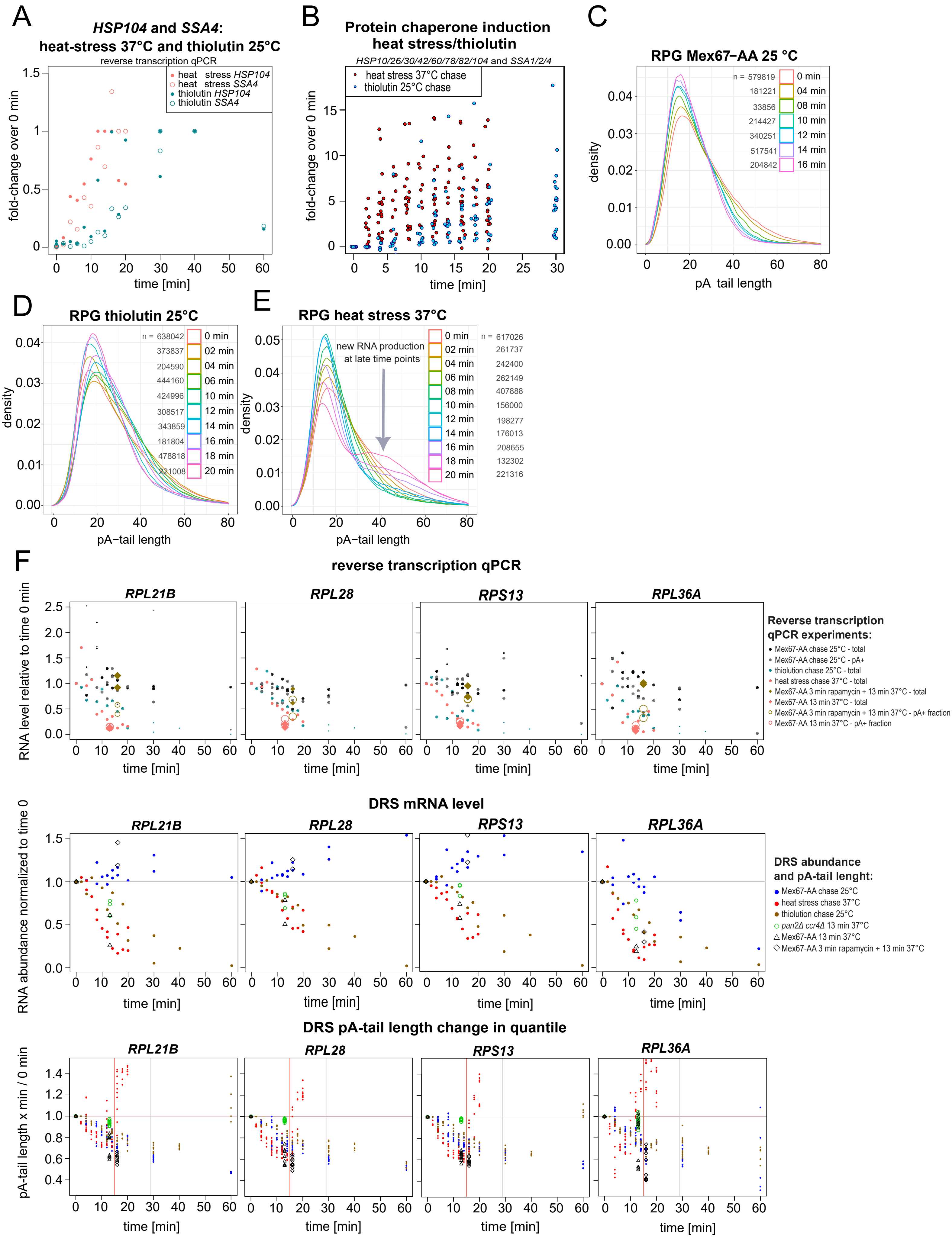

Appendix Figure S2. Transcriptomic changes upon heat stress and thiolutin treatment. **A.** Graph shows up-regulation of *HSP104* and *SSA4* mRNA levels during heat stress at 37 °C and thiolutin treatment at 25 °C by reverse transcription coupled to qPCR. **B.** Plot shows the up-regulation of a collection of chaperone mRNAs in the heat-stress at 37 °C and thiolutin treatment at 25 °C chase sequencing data. Those transcripts are known to be transcriptionally induced during heat stress (Vinayachandran et al, 2018). **C.-E.** Global distributions of RPG mRNA pA-tail lengths in chase experiments: Mex67-depletion (C.), thiolutin treatment at 25 °C (D.) and heat stress at 37 °C (E.). The arrow on the heat stress density plot points to a peak of long pA-tailed mRNAs accumulating in later heat stress chase time points. Since transcriptional down-regulation of RPGs during heat stress is only temporary (Vinayachandran et al, 2018), those can only be new transcripts. The contribution of new mRNAs in late heat stress time points was too important (they strongly distorted quantile values) and therefore those samples were not used for deadenylation modeling. **F.** Series of graphs show in rows from top to bottom time dependent: (top) mRNA abundance by reverse-transcription coupled to qPCR, mRNA, (middle) abundance by DRS and (bottom) change in the pA-tail length in upper quantiles (75-95th) normalized to control for single RPG mRNAs: *RPL21B*, *RPL36A*, *RPS13*, and *RPL28* in Mex67-depletion, heat-stress 37 °C and thiolutin 25 °C chase sequencing data. Legends for each type of experiment are given separately.

Appendix Figure S3

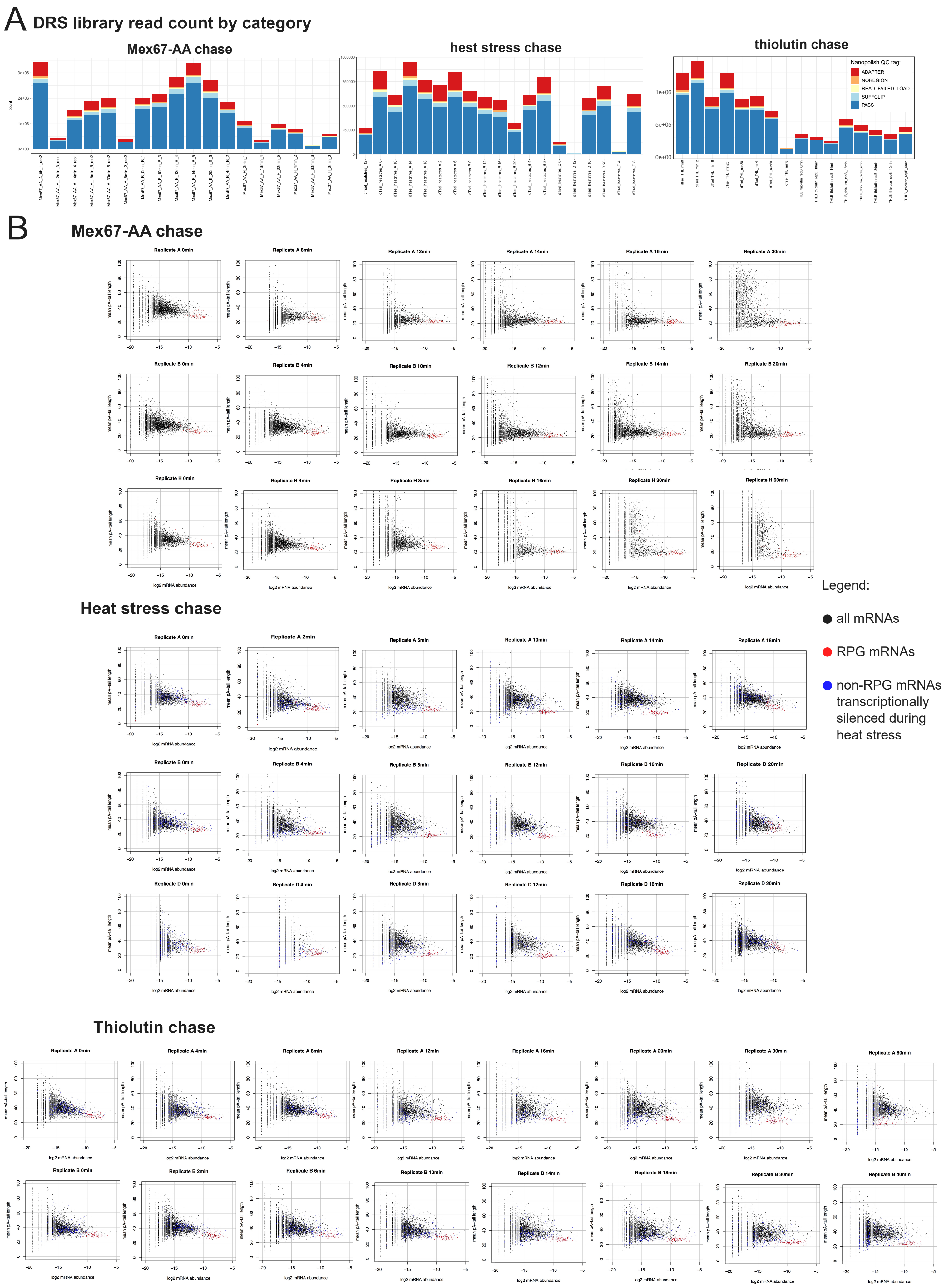

Appendix Figure S3. General DRS chase quality control. **A.** Bar plots show each DRS chase sample read count with division into read quality control tags. Only the PASS counts were used for further analyses. **B.** Matrixes of scatterplots comparing the log2 mRNA abundance on y-scale to the transcripts' mean pA-tail length on the x-axis for the three chase experiments (Mex67-depletion, heat stress and thiolutin treatment). Where appropriate selected groups of mRNAs are highlighted as indicated in the legend. The number of mRNAs shown on each figure is indicated in each panel legend.

Appendix Figure S4

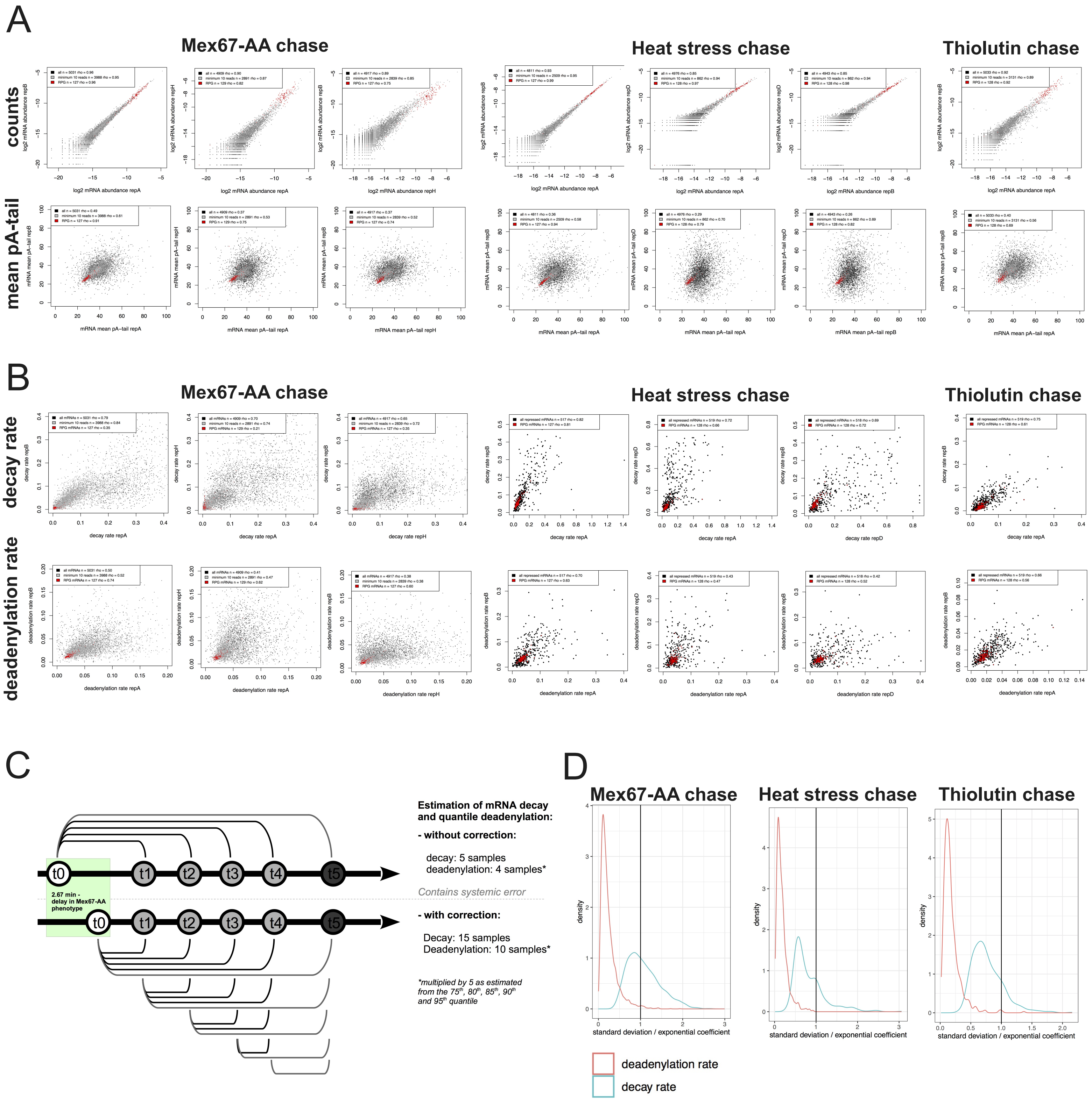

Appendix Figure S4. DRS quality control and decay/deadenylation modeling strategy. **A.** Scatter plots showing the correlation between read count in log2-scale (top row) and mean pA-tail length (bottom row) in the three chase experiments. Only the control samples for each replicate were analyzed as only these samples are expected to be similar. The plots show all detected mRNAs (black dots), mRNAs detected with at least 10 reads (grey dots) and RPG mRNAs (red dots). The number of mRNAs analyzed for each group and the Spearman rho is indicated on the Figure panel. **B.** Scatter plots showing the correlation between decay (top row) and deadenylation coefficients obtained from the preliminary data analysis (see Appendix main text). The classes of mRNAs analyzed are the same as in Appendix Fig. S4A. The good correlation between replicates justified merging the replicates (Appending Fig. S4C and main text). **C.** Scheme compares two strategies for calculation of decay and quantile deadenylation rates. The top version shows the classical approach where the unique control sample is used to estimate the decay coefficient in relation to all the data-points in the replicate. In case of the Mex67-depletion this introduces a bias that is due to the time required for the phenotype onset to be fully established. This exact time was calculated when the modified gamma model was built and defined to be equal to 2.67 min. This allowed to shift the control sample on the time-line and to treat every time-point in the chase experiment as a control for the consecutive sample. This approach greatly increases the number of coefficients that can be used to estimate the decay and quantile deadenylation rates. In the latter case this number is further increased by 5-fold as each quantile deadenylation coefficient is estimated from five quantiles: 75th, 80th, 85th, 90th and 95th. The accuracy of the calculated decay and deadenylation coefficients was further tested by comparing mRNA half-lives to published datasets (Figure 1) and by evaluating the compliance of the functions drawn using the quantile deadenylation coefficients compared to actual experimental data (EV Figure 5A). **D.** Density plots compare the ratio between the standard deviation and its corresponding decay or quantile deadenylation coefficient calculated using the bulk method shown in Appendix Figure S4C (bottom version; see also main text). The number of measurments is the same as in Appendix Figure S4B.

5. R code for calculation of the modified gamma distribution parameters

#this code should calculate the modified gamma distribution gamma_shape and gamm_rate parameters. Remember that estimation of the modified gamma distribution parameters is most accurate on distributions containing a large number of reads.

#download dataframes and prepare a separate directory; then set the directory path in setwd(). The end result table and pdf files containing figues will be downloaded in this directory.

#note that this code works with the Mex67-chase dataset dataframes as it extracts the time point from the column header using the colnames() command. If you want to use it on other dataframes, then either adjust the column name or change the colnames() commands accordingly.
0.096 parameter (beta = JP_DECAP) should be adjusted to the given datasets and remain equal for all distributions.

```
setwd("set chosen directory")

getwd()# will check the chosen directory location



JP_DECAP<-0.096

JP_dist<-function(x,decap,shape,rate) {

  return(((tanh(x*decap))^shape)*exp(-rate*x))

}



JP_RES<-NULL

JP_LogN<-NULL

JP_Log2<-NULL


now<-Sys.time()

sname<-paste0("JP4AT-",format(Sys.time(),format="%y%m%d-%H%M"))

pdf(file=paste0(sname,".pdf"))

nn<-60  # number of curves produced for the simulation step

ll<-80  # fixed parameter describing the maximum pA-tail length; do not modify

zz<-80

pp<-0.5 # fixed parameter not to be changed

tt<-60  # maximum time in minutes

scale<-1 # curve density for the simulation step; - scale * nn is the maximum in silico evolution time

fname<-"ORFs_repB" #file name of one of the data frames



for (fname in c("name of dataframe in chosen directory")) {



  all_raw<-read.csv(paste0(fname,".csv"))

  all<-all_raw[order(all_raw[,1]),]

  all<-NULL

  ii<-0

  for(ii in 0:(ll+1)) {
```

```
  ss<-all_raw[all_raw[,1]==ii,]

  if (length(ss[,1])==0) {

    ss<-all_raw[1,]

    ss[1,1]<-ii

    ss[1,-1]<-0

  }

  ss<-gsub("NA",0,ss)

  ss<-as.numeric(ss)

  all<-rbind(all,as.numeric(ss))

}


colnames(all)<-gsub("_","",gsub("25C","",gsub("min","",gsub(fname,"",colnames(all_raw)))))

colnames(all)<-gsub("min","",gsub("Mex67AA","",colnames(all)))

#rownames(all)<-all[,1]

all<-as.data.frame(all)


#all[,1]<-as.numeric(0:(ll+1))

for(ii in 2:7) {

  #  all[,ii]<-as.numeric(all[,ii])

  all[,ii]<-all[,ii]/max(all[,ii],na.rm = TRUE)

}


dist<-all[1:(ll+2),1:2]

names(dist)<-c("n","o")

#dist$n<-0:(ll+1)

#dist$o<-0


dist$o[is.na(dist$o)]<-0

dist$o<-dist$o/max(dist$o)

#dist$o<-dist$o*0

#dist$o[70]<-1


old<-dist$o

old2<-dist$o


plot(dist$o,type="n",col="blue",log="",xlim=c(0,100),xlab = "N-Tail",

     ylab = " experimental PolyA distribution",lwd=2,main=fname)
```

```
for ( ii in 2:7) {
  cc<-as.double(colnames(all)[ii])/tt
  lines(all[,ii],type="l",col=rgb(1-cc,0,cc),lwd=2)
}

plot(dist$o,type="n",col="blue",log="xy",xlab = "N-Tail",xlim=c(1,200),
     ylab = "normalized experimental PolyA distribution",lwd=2,main=fname)
for ( ii in 2:7) {
  cc<-as.double(colnames(all)[ii])/tt
  lines(all[,ii],type="l",col=rgb(1-cc,0,cc),lwd=2)
}

plot(dist$o,type="l",col="red",log="xy",xlab = "N-Tail",ylab = "modelled PolyA distribution",lwd=2,main=fname)


wcap<-(0:(ll+1))*JP_DECAP
wcap<-(tanh(wcap))^pp


beta<-0
for (beta in 0:(nn-1)) {
  new<-dist$o*0
  for (ii in 1:(ll+1)) {
    for (jj in (ll+2-ii):1) {
      new[ii]<-(new[ii]+dist$o[ii+jj])*beta*scale/jj
    }
    new[ii]<-new[ii]+dist$o[ii]
  }
  new<-new*exp(-beta*scale)
  old<-cbind(old,new)
  colnames(old)[beta+2]<-paste0("b",beta*scale)


  wcap<-tanh((0:(ll+1))*JP_DECAP)
  wcap<-wcap^((beta*scale)^pp)
  old2<-cbind(old2,new*wcap)
  colnames(old2)[beta+2]<-paste0("b",beta*scale)


  #lines(new,type="l",col="green")
  lines(new*wcap,type="l",col=rgb(0,beta/nn,1-beta/nn))
```

```
}

write.table(old,file=paste0(fname,"_models.txt"),sep="\t")

plot(old[,1],col="blue",type="n",xlim=c(1,100),lwd=4,log="",xlab = "N-Tail",
     ylab = "modelled PolyA distribution",main=fname)
for(ii in 2:length(colnames(old))) {
  lines(old[,ii],col=rgb(0,(ii-1)/nn,1-(ii-1)/nn),type="l")
}
lines(old[,1],col="red",type="l",lwd=2)

plot(old[,1],col="blue",type="n",xlim=c(1,100),lwd=4,log="",xlab = "N-Tail",
     ylab = "normalized modelled PolyA distribution",main=fname)
for(ii in 2:length(colnames(old))) {
  #  old[,ii]<-old[,ii]/max(old[,ii])
  lines(old[,ii]/max(old[,ii]),col=rgb(0,(ii-1)/nn,1-(ii-1)/nn),type="l")
}
lines(old[,1],col="red",type="l",lwd=3)

#old2<-old*wcap
#old2[,1]<-old[,1]

plot(old2[,1],col="blue",type="n",xlim=c(1,100),lwd=4,log="",xlab = "N-Tail",
     ylab = "modelled PolyA distribution",main=fname)
for(ii in 2:length(colnames(old2))) {
  lines(old2[,ii],col=rgb(0,(ii-1)/nn,1-(ii-1)/nn),type="l")
}
lines(old2[,1],col="red",type="l",lwd=3)

plot(old2[,1],col="blue",type="n",xlim=c(1,100),lwd=4,log="",xlab = "N-Tail",
     ylab = "normalized modelled PolyA distribution",main=fname)
for(ii in 2:length(colnames(old))) {
  #  old2[,ii]<-old2[,ii]/max(old2[,ii])
  lines(old2[,ii]/max(old2[,ii]),col=rgb(0,(ii-1)/nn,1-(ii-1)/nn),type="l")
}
lines(old2[,1],col="red",type="l",lwd=3)
```

```
plot(dist$o,type="n",col="blue",log="",xlim=c(0,100),xlab = "N-Tail",
     ylab = "normalized experimental PolyA distribution",lwd=2,main=fname)
for ( ii in 2:7) {
  cc<-as.double(colnames(all)[ii])/tt
  lines(all[,ii],type="l",col=rgb(0,1-cc,cc),lwd=2)
}

plot(dist$o,type="n",col="blue",log="",xlim=c(0,100),xlab = "N-Tail",
     ylab = "normalized experimental PolyA distribution",lwd=2,main=fname)
for ( ii in 2:7) {
  cc<-as.double(colnames(all)[ii])/tt
  lines(all[,ii],type="l",col=rgb(0,1-cc,cc),lwd=2)
}
for(ii in 2:length(colnames(old))) {
  lines(old2[,ii]/max(old2[,ii]),col=rgb(0,(ii-1)/nn,1-(ii-1)/nn),type="l")
}


for ( ii in 2:7) {

  ###############################################
  #Glowne dopasowanie
  ###############################################

  cc<-as.double(colnames(all)[ii])/tt
  max<-9e99
  kk<-1
  for(jj in 2:length(colnames(old))) {
    chi2<-old2[,jj]/max(old2[,jj])-all[1:(ll+2),ii]
    chi2<-chi2*chi2
    chi2<-sum(chi2*chi2)
    if (chi2<max) {
      max<-chi2;
      kk<-jj
      #      print(c(ii,jj,chi2,"\n"))
```

```
  }
}

plot(all[,ii],type="p",pch=19,col=rgb(0,1-cc,cc),log="",xlim=c(0,100),xlab = "N-Tail",
     ylab = "normalized experimental PolyA distribution",lwd=2,main=paste0(fname," min ",cc*tt,"; in silico ",kk-1))
lines(old2[,kk]/max(old2[,kk]),col=rgb(0,(kk-1)/nn,1-(kk-1)/nn),type="l",lw=2)

dd<-all[,c(1,ii)]
dd[,1]<-as.numeric(dd[,1])
dd[,2]<-as.numeric(dd[,2])
dd<-dd[dd[,2]>0,]
dd<-dd[dd[,1]<=zz,]
x<-as.numeric(dd[,1])
y<-as.numeric(dd[,2])

rate<-0.05
shape=3

fit0<-nls(y ~ a*JP_dist(x,JP_DECAP,shape,rate),start=list(a=8e3),control=nls.control(maxiter=500))
co<-coefficients(fit0)
lines(x,predict(fit0),lw=0.5)

fit1<-nls(y ~ a*JP_dist(x,JP_DECAP,shape,rate),start=list(a=co[1],rate=rate),control=nls.control(maxiter=500))
co<-coefficients(fit1)
lines(x,predict(fit1),lw=0.5)

fit2<-nls(y ~ a*JP_dist(x,JP_DECAP,shape,rate),start=list(a=co[1],shape=shape,rate=co[2]),control=nls.control(maxiter=500))
co<-coef(fit2)
lines(x,predict(fit2),lw=1)

fit3<-nls(y ~ a*JP_dist(x,JP_DECAP,shape,rate),start=list(a=co[1],shape=co[2],rate=co[3]),control=nls.control(maxiter=500),weights = 1/(1+y))
co<-coef(fit3)
lines(x,predict(fit3),lw=3,col="black")

ssss<-summary(fit3)
JP_RES<-rbind(JP_RES,c(fname,cc*tt,co,ssss$parameters[2,],ssss$parameters[3,]))
```

```
if (as.numeric(colnames(all)[ii])<15) {

  mean<-3.75
  width<-0.31

  plot(all[,ii],type="p",pch=19,col=rgb(0,1-cc,cc),log="",xlim=c(0,100),xlab = "N-Tail",
       ylab = "normalized experimental PolyA distribution with logN",lwd=2,main=paste0(fname," min ",cc*tt,"; in silico ",kk-1))
  #    lines(old2[,kk]/max(old2[,kk]),col=rgb(0,(kk-1)/nn,1-(kk-1)/nn),type="l",lw=2)


  ww<-x
  aa<-(ww>60)+(ww<20)

  fit4<-nls(y ~ a*JP_dist(x,JP_DECAP,shape,rate),
            start=list(a=co[1],shape=co[2],rate=co[3]),control=nls.control(maxiter=500),weights = aa/(1+y))
  coss<-coef(fit4)
  lines(x,predict(fit4),lw=0.1,col="black")


  shape=coss[2]
  rate<-coss[3]


  fit5<-nls(y ~ a*JP_dist(x,JP_DECAP,shape,rate)+b*dlnorm(x,mean,width),
            start=list(a=coss[1],b=1),control=nls.control(maxiter=500),weights = 1/(1+y))
  coss<-coef(fit5)
  lines(x,predict(fit5),lw=0.1,col="black")

  fit6<-nls(y ~ a*JP_dist(x,JP_DECAP,shape,rate)+b*dlnorm(x,mean,width),
            start=list(a=coss[1],shape=shape,rate=rate,b=coss[2]),control=nls.control(maxiter=500),weights = 1/(1+y))
  coss<-coef(fit6)
  lines(x,predict(fit6),lw=.5,col="black")

  shape<-coss[2]
  rate<- coss[3]

  ok<-tryCatch({
    fit7<-nls(y ~ abs(a)*JP_dist(x,JP_DECAP,shape,rate)+abs(b)*dlnorm(x,mean,width),
              start=list(a=coss[1],b=coss[4],mean=mean,width=width),control=nls.control(maxiter=500),weights = 1/(1+y))
    coss<-coef(fit7)
    lines(x,predict(fit7),lw=1,col="black")
```

```
},error=function(cond) {
  lines(x,predict(fit6),lw=.1,col="black")
  print(c("7",as.numeric(colnames(all)[ii]),fname))
}
)

ok<-tryCatch({
  fit8<-nls(y ~ abs(a)*JP_dist(x,JP_DECAP,shape,rate)+abs(b)*dlnorm(x,mean,width),
        start=list(a=coss[1],shape=shape,rate=rate,b=coss[2],mean=coss[3],width=coss[4]),control=nls.control(maxiter=500),weights = 1/(1+y))

  coss<-coef(fit8)
  lines(x,predict(fit8),lw=3,col="red")
  lines(x,coss[4]*dlnorm(x,coss[5],coss[6]),col="blue",lw=2,lty=1)
  lines(x,coss[1]*JP_dist(x,JP_DECAP,coss[2],coss[3]),col="green",lw=2,lty=1)
  ssss<-summary(fit8)
  JP_LogN<-rbind(JP_LogN,c(fname,cc*tt,coss,ssss$parameters[1,],ssss$parameters[2,],ssss$parameters[3,],ssss$parameters[4,],ssss$parameters[5,],ssss$parameters[6,]))
},error=function(cond) {
  #     lines(x,predict(fit7),lw=.1,col="black")
  print(c("8",as.numeric(colnames(all)[ii]),fname))
}
)

ok<-tryCatch({
  mean<-3.72
  width<-0.284

  fit9<-nls(y ~ abs(a)*JP_dist(x,JP_DECAP,shape,rate)+abs(b)*dlnorm(x,mean,width),
        start=list(a=coss[1],shape=coss[2],rate=coss[3],b=coss[4]),control=nls.control(maxiter=5000),weights = 1/(1+y))

  coss<-coef(fit9)
  ssss<-summary(fit9)
  JP_Log2<-rbind(JP_Log2,c(fname,cc*tt,coss,ssss$parameters[1,],ssss$parameters[2,],ssss$parameters[3,],ssss$parameters[4,]))

  lines(x,predict(fit9),lw=3,col="red",lty=2)
  lines(x,coss[4]*dlnorm(x,mean,width),col="blue",lw=2,lty=2)
  lines(x,coss[1]*JP_dist(x,JP_DECAP,coss[2],coss[3]),col="green",lw=2,lty=2)
```

```
    },error=function(cond) {
      print(c("9",as.numeric(colnames(all)[ii]),fname))
    }
    )

    lines(x,predict(fit3),lw=3,col="black")
  }

}

#lines(old2[,22]/max(old2[,22]),type="l",col=rgb(1,0,0),lwd=3,lty=4)
#lines(old2[,18]/max(old2[,18]),type="l",col=rgb(1,0,0),lwd=3,lty=4)
#lines(old2[,15]/max(old2[,15]),type="l",col=rgb(1,0,0),lwd=3,lty=4)
#lines(old2[,4]/max(old2[,4]),type="l",col=rgb(1,0,0),lwd=3,lty=4)
#lines(old2[,2]/max(old2[,2]),type="l",col=rgb(1,0,0),lwd=3,lty=4)

dall<-all[,1]
dall<-cbind(dall,all[,2]-all[,3])
dall<-cbind(dall,all[,2]-all[,4])
dall<-cbind(dall,all[,2]-all[,5])

dall[,2]<-dall[,2]/max(dall[,2],na.rm = TRUE)
dall[,3]<-dall[,3]/max(dall[,3],na.rm = TRUE)
dall[,4]<-dall[,4]/max(dall[,4],na.rm = TRUE)

plot(dall[,2],type="n",col="blue",xlim=c(0,80),xlab = "N-Tail",
     ylab = "normalized difference in experimental PolyA distributions",lwd=2,main=fname)
lines(dall[,2],type="p",pch=19)

x<-dall[,1]
y<-dall[,2]
w<-0+(x>35)
fitex<-nls(y~a*dlnorm(x,mean,width),start=c(a=10,mean=3.8,width=0.1),weights = w)
res<-summary(fitex)
lines(predict(fitex),col="blue",lw=2)
#lines(dall[,4],type="p")
#lines(all[,1],10*dlnorm(all[,1],meanlog=3.8,.1),col="green")
```

```
  write.table(dall,file = paste0(fname,"diff.txt"),sep="\t")

  syn<-coef(fitex)

}

dev.off()

write.table(JP_RES,paste0(sname,"JP_RES.txt"),sep="\t")

write.table(JP_LogN,paste0(sname,"JP_LogN.txt"),sep="\t")

write.table(JP_Log2,paste0(sname,"JP_Log2.txt"),sep="\t")
```
