# Supplementary figures and images for: Modeling of mRNA deadenylation rates reveal a complex relationship between mRNA deadenylation and decay
Source: EMBO J. 2024 Oct 11;43(24):6525–54. doi: 10.1038/s44318-024-00258-3 (PMC11649921; doi:10.1038/s44318-024-00258-3)

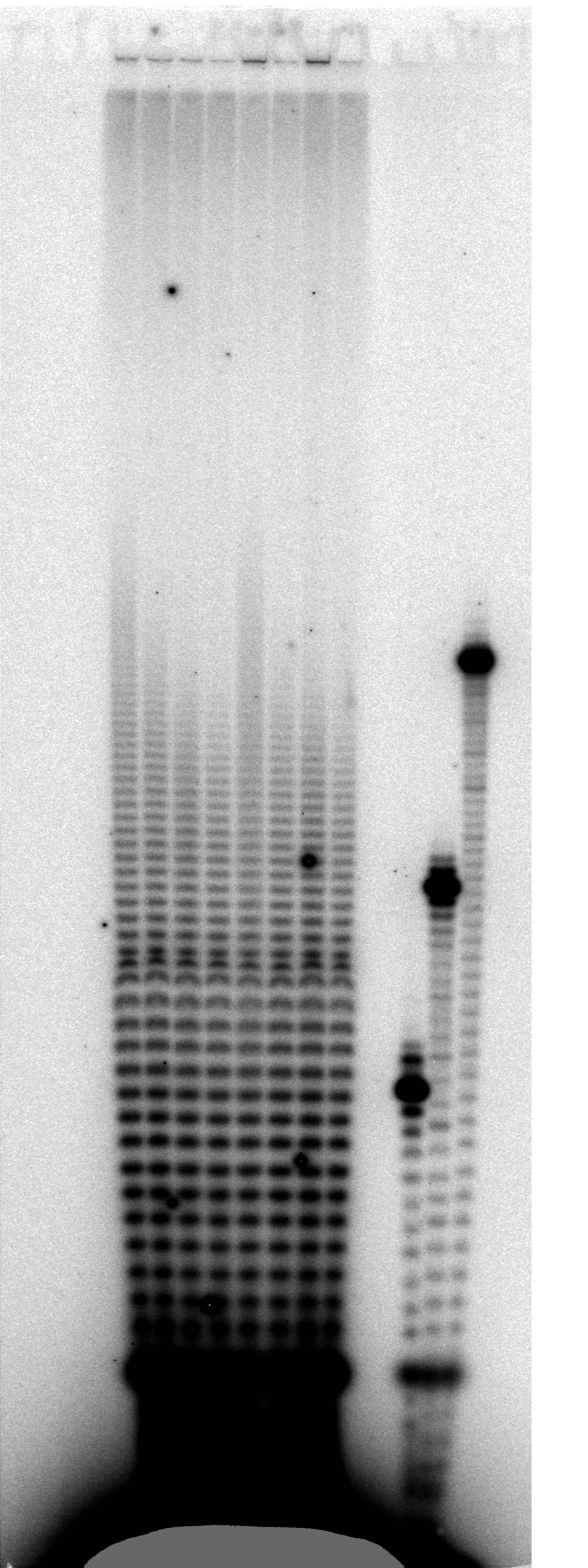

Supplement: Supplementary file 6 — Source data Fig. 2 [file 44318_2024_258_MOESM6_ESM.zip › Figure 2/Figure 2B/Mex67 time course greyscale for quantification (1).tif]

# Amount of pA+ RNA fraction Mex67-chase

slope:  $-0.01306$ , intercept:  $0.99015$

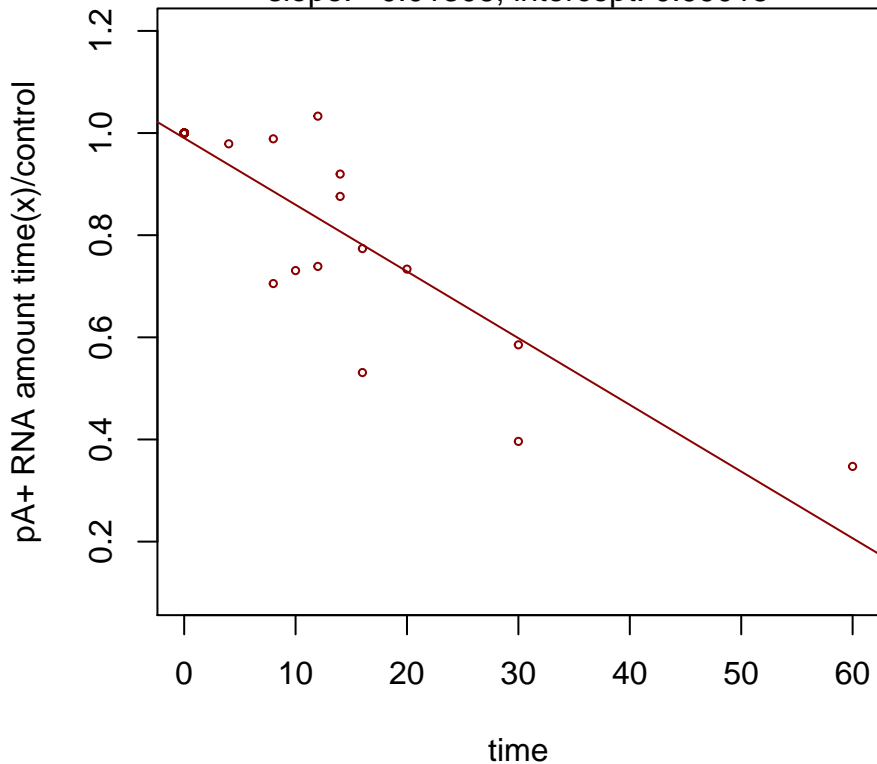

Supplement: Supplementary file 10 — Source data Fig. 4 [file 44318_2024_258_MOESM10_ESM.zip › Figure 4/FIGURE 4I/Figure_EV3B_pAplus_RNA_recovered_from_total_Mex67AA.pdf]

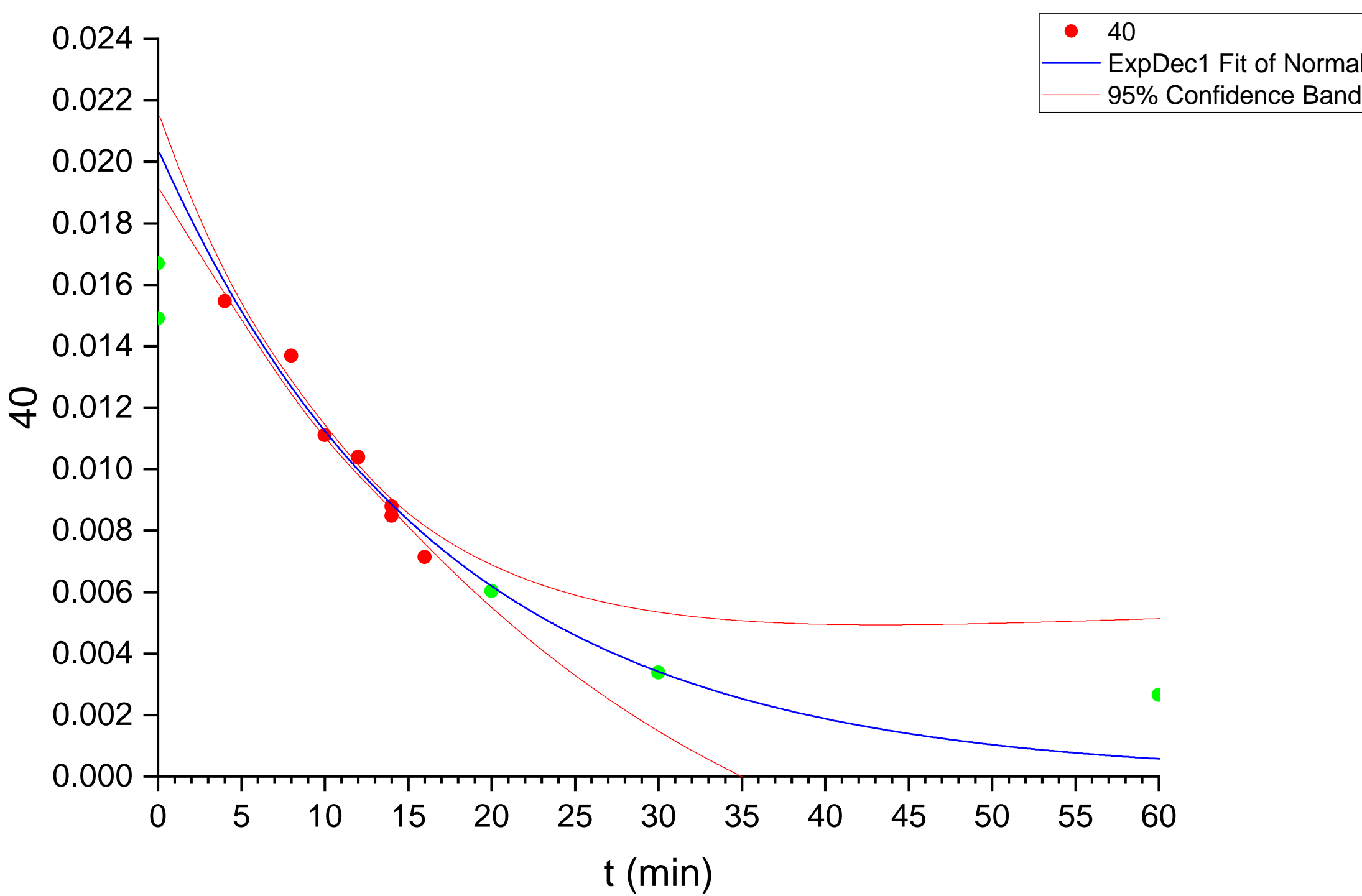

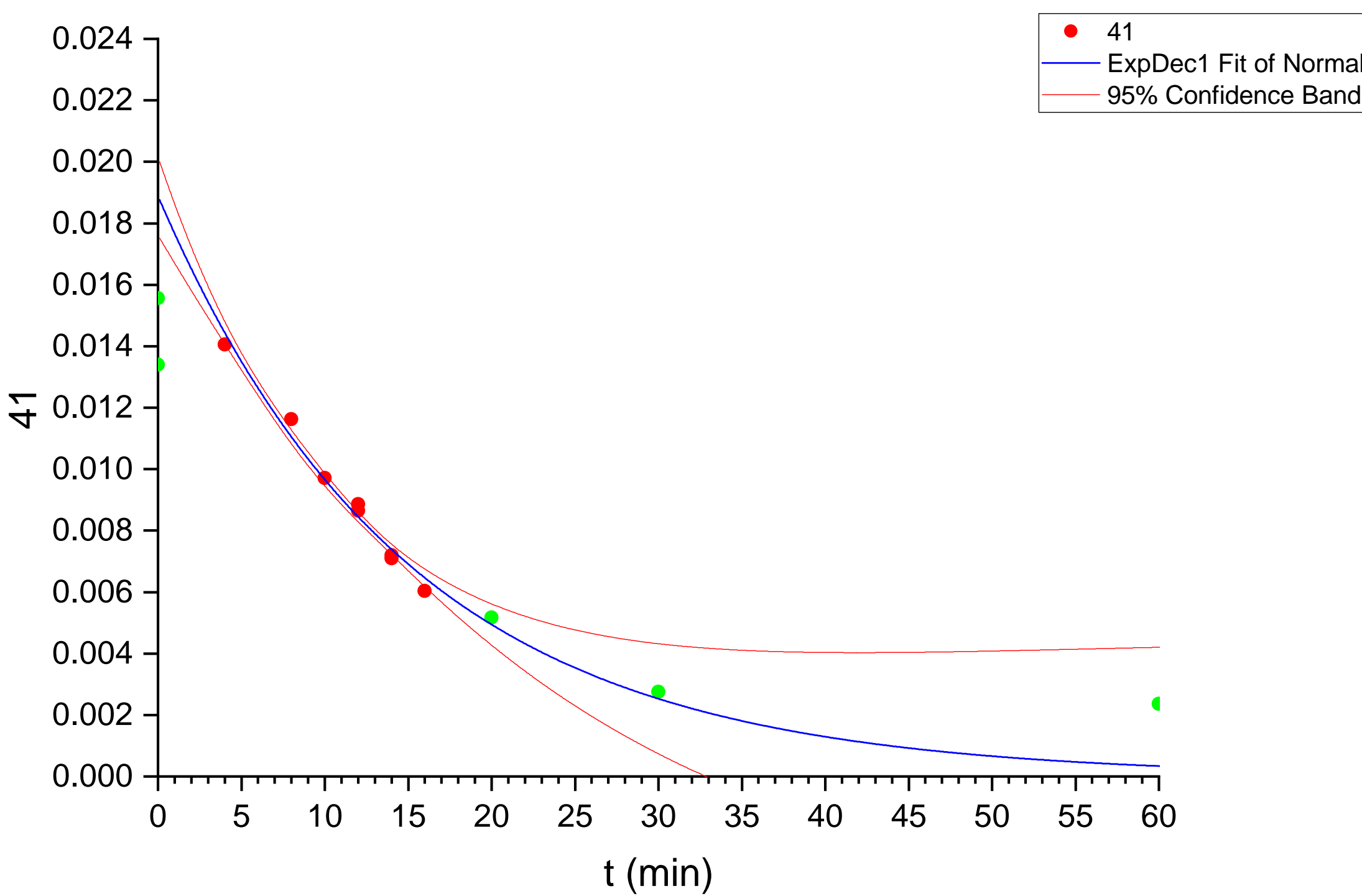

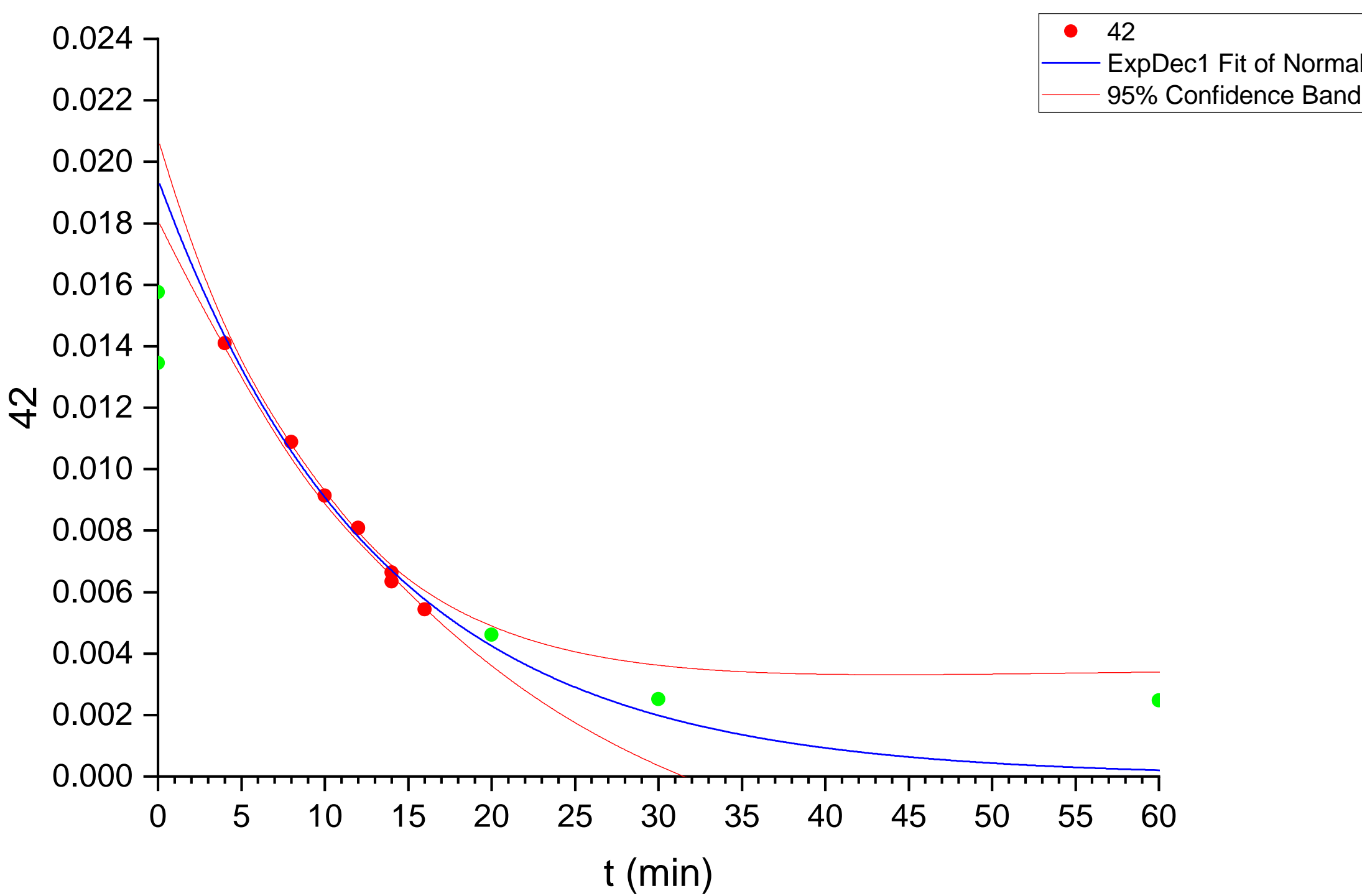

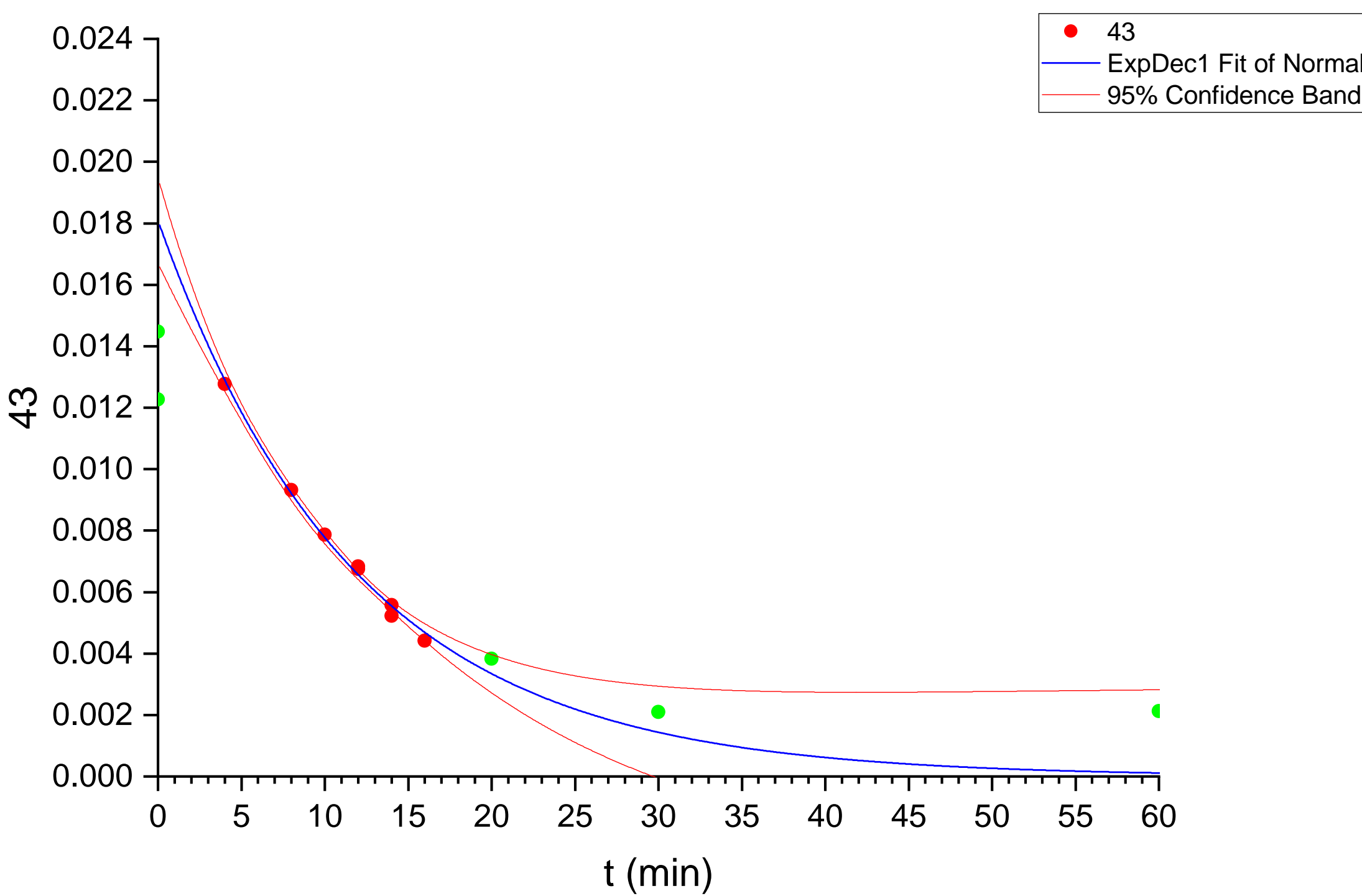

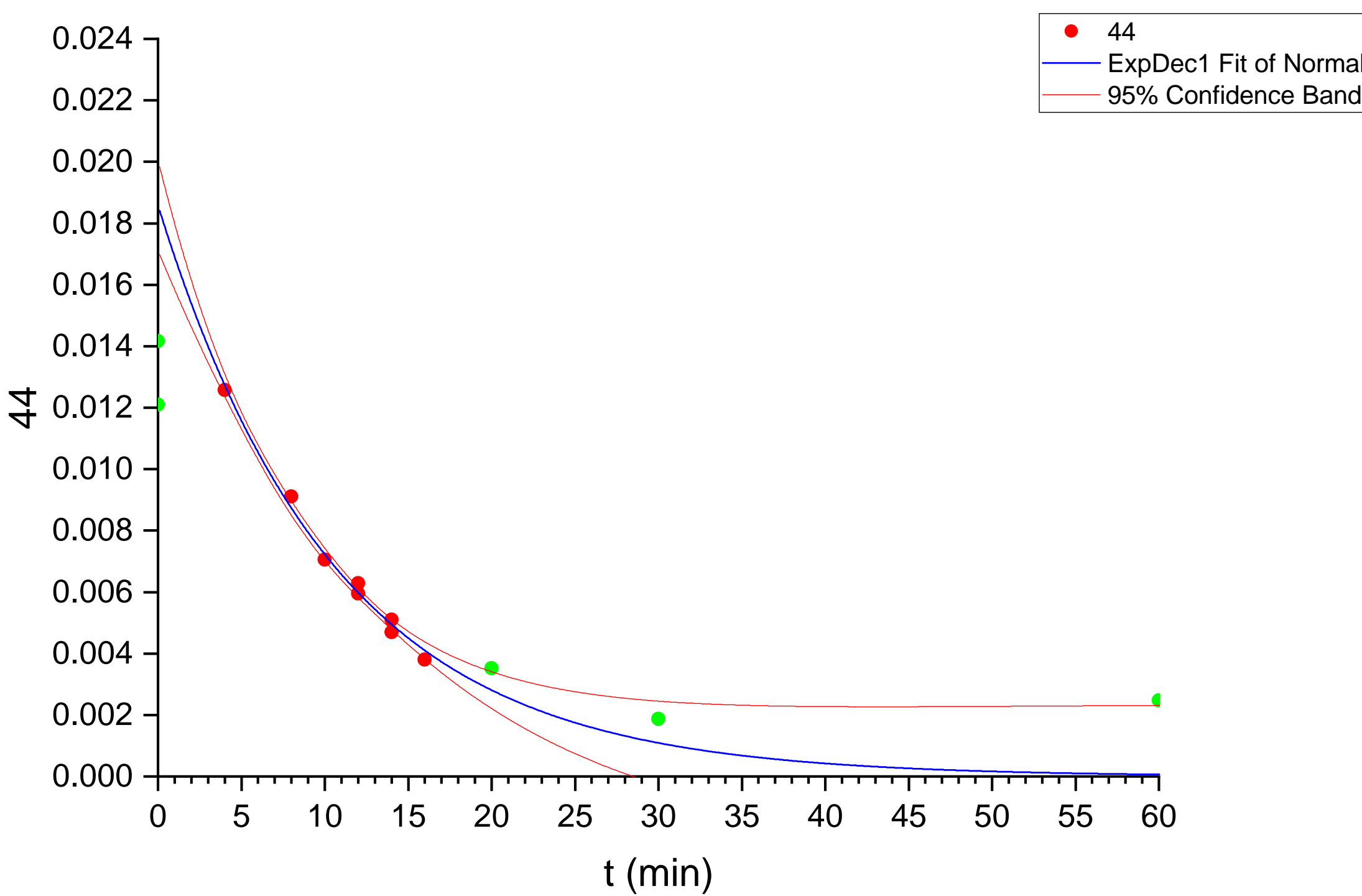

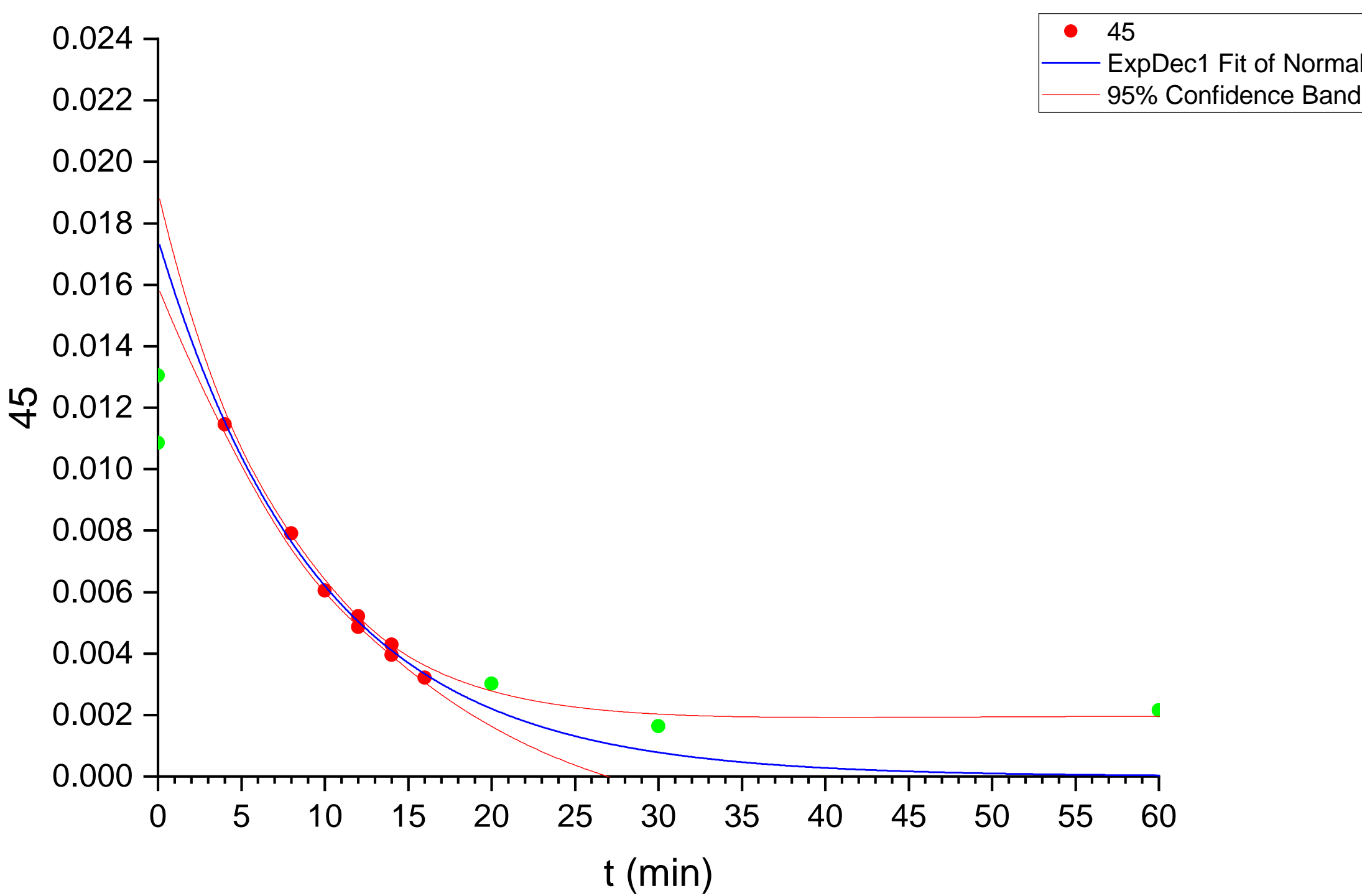

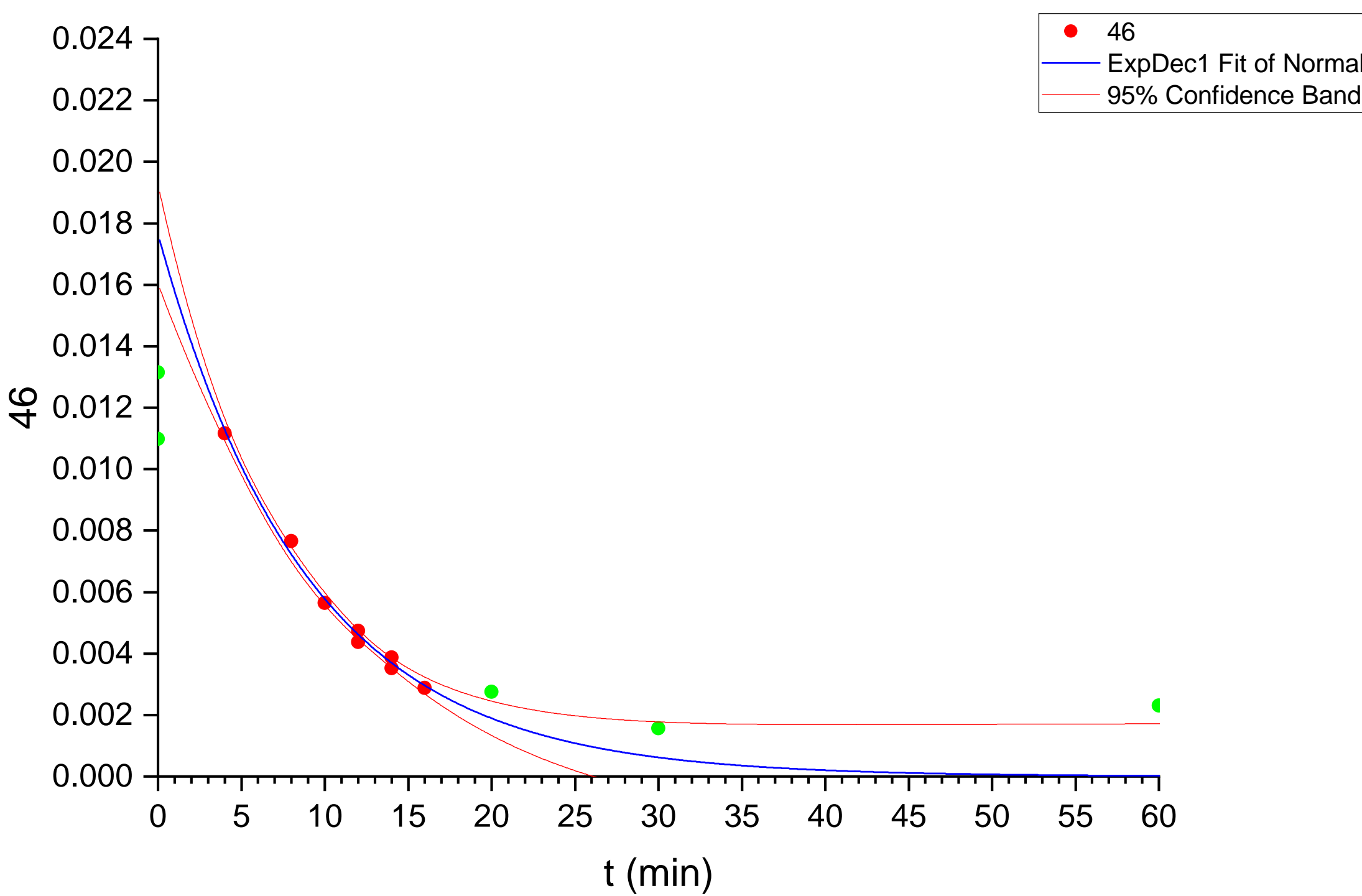

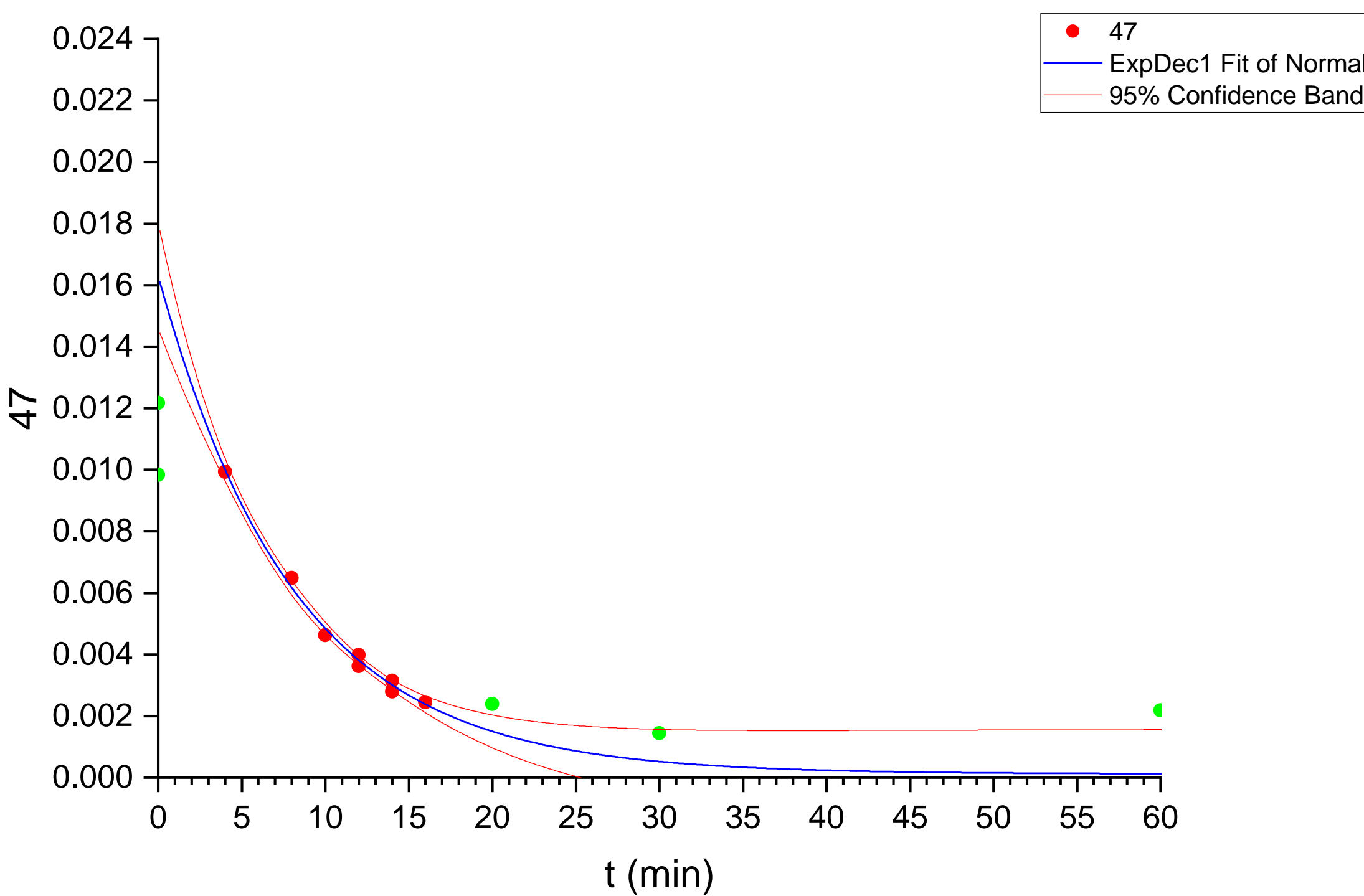

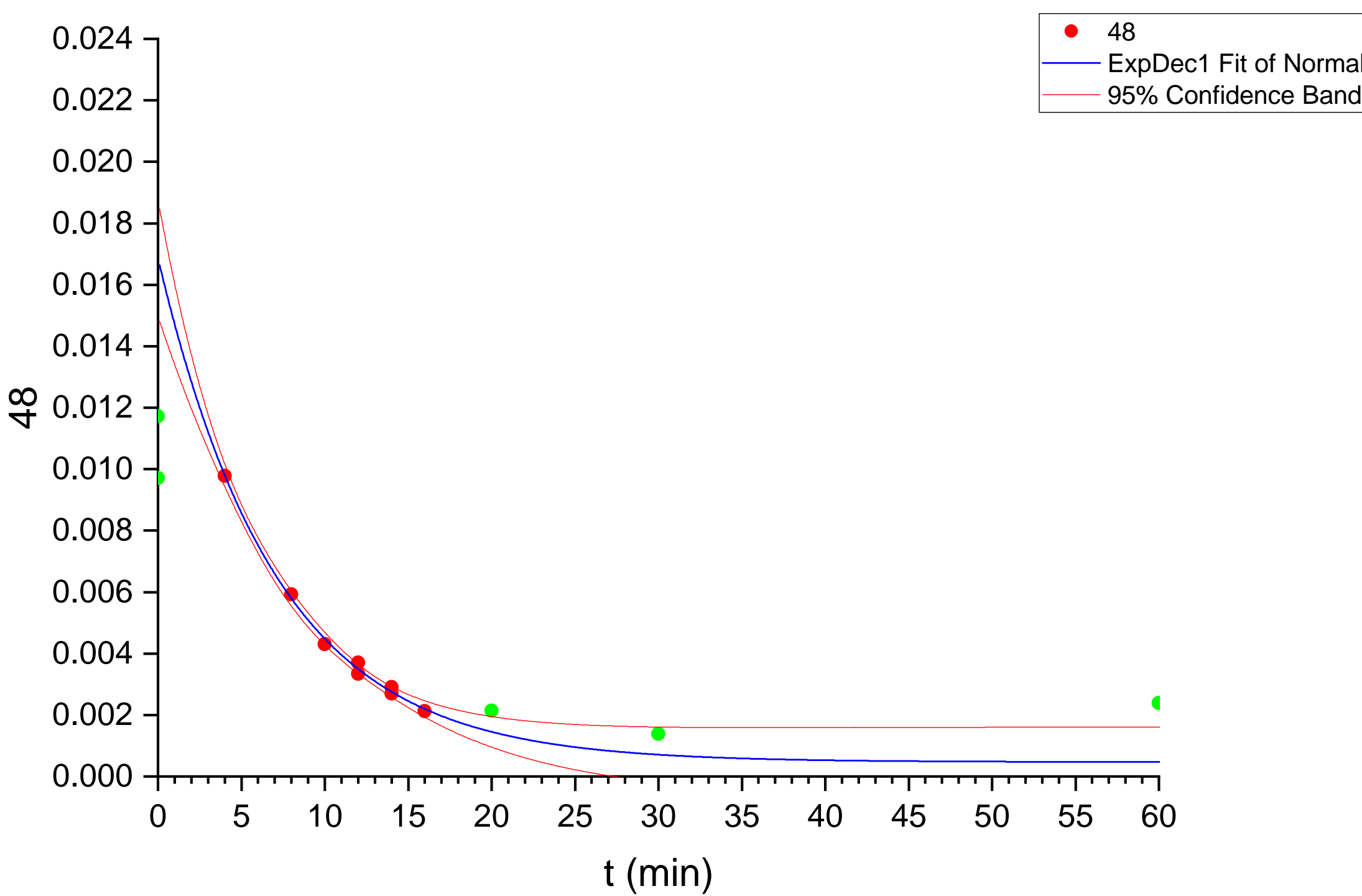

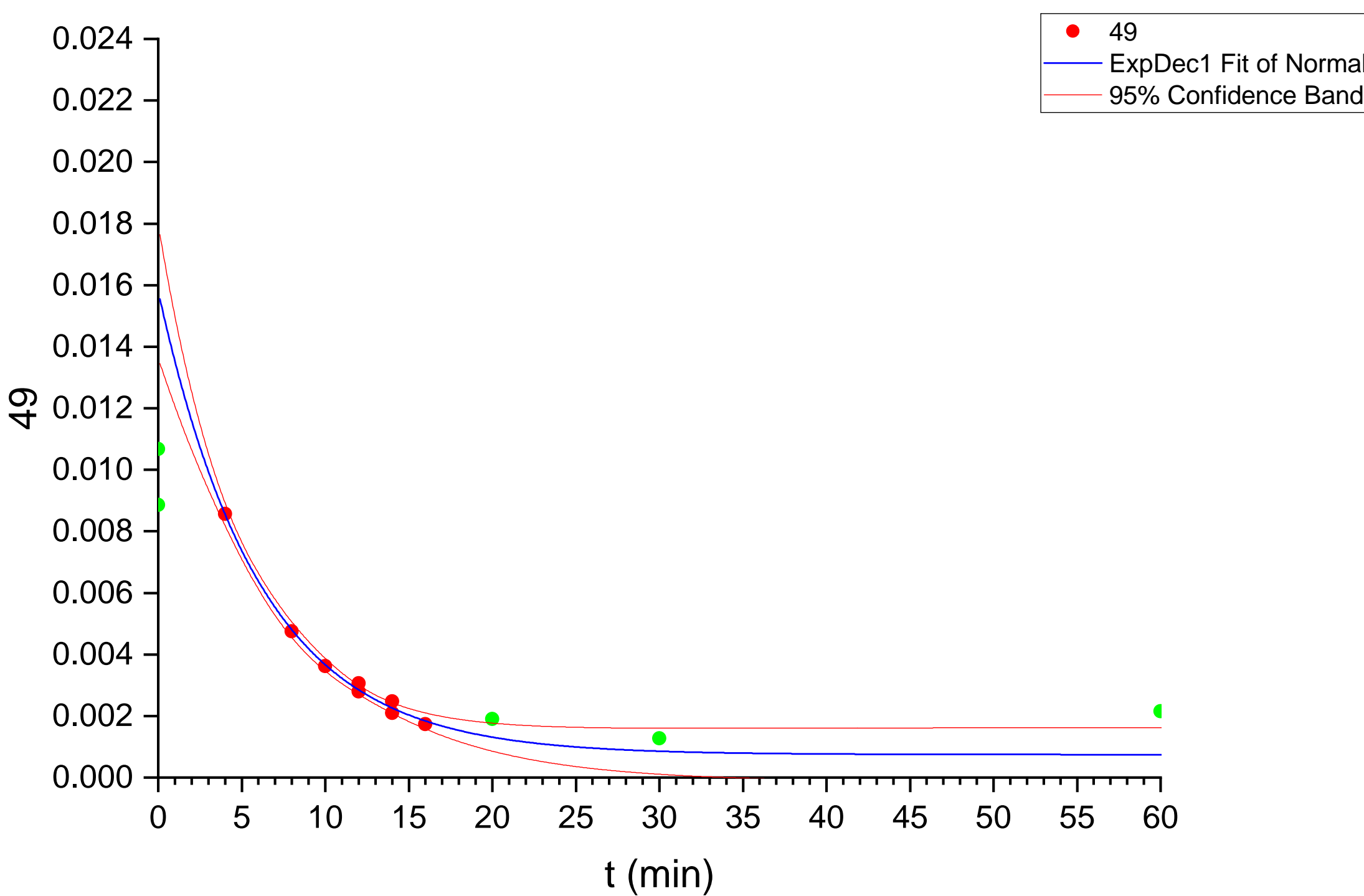

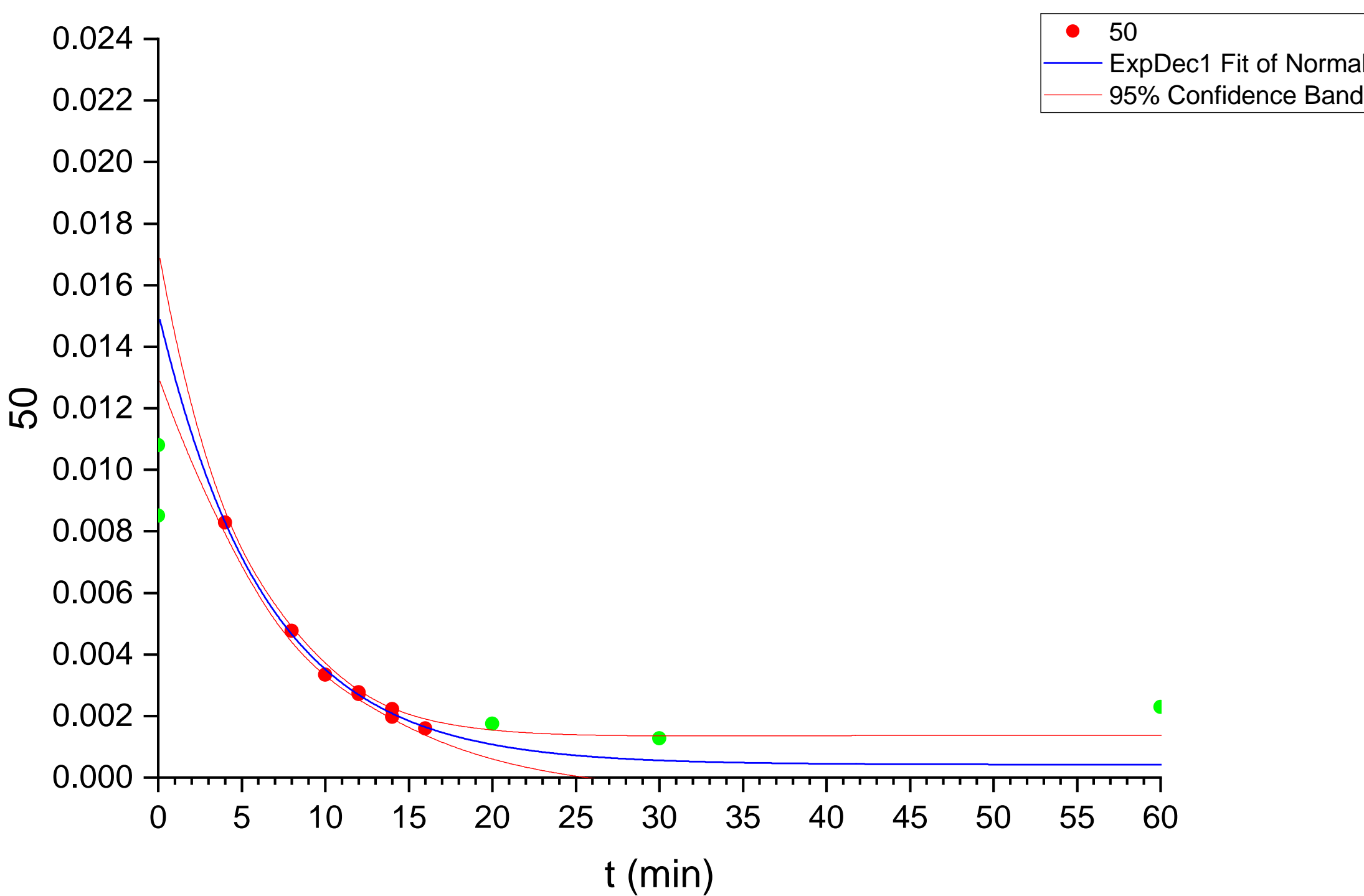

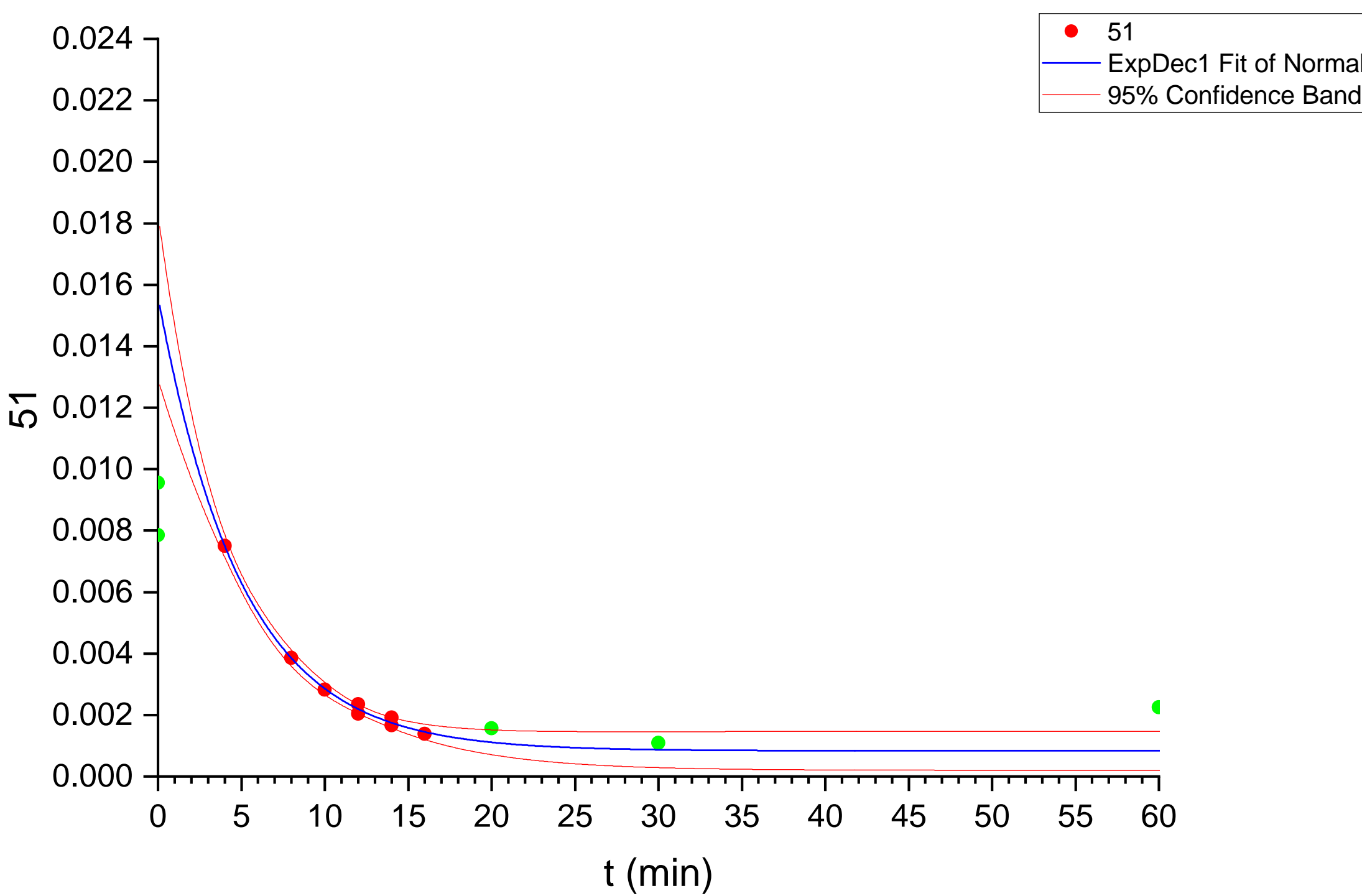

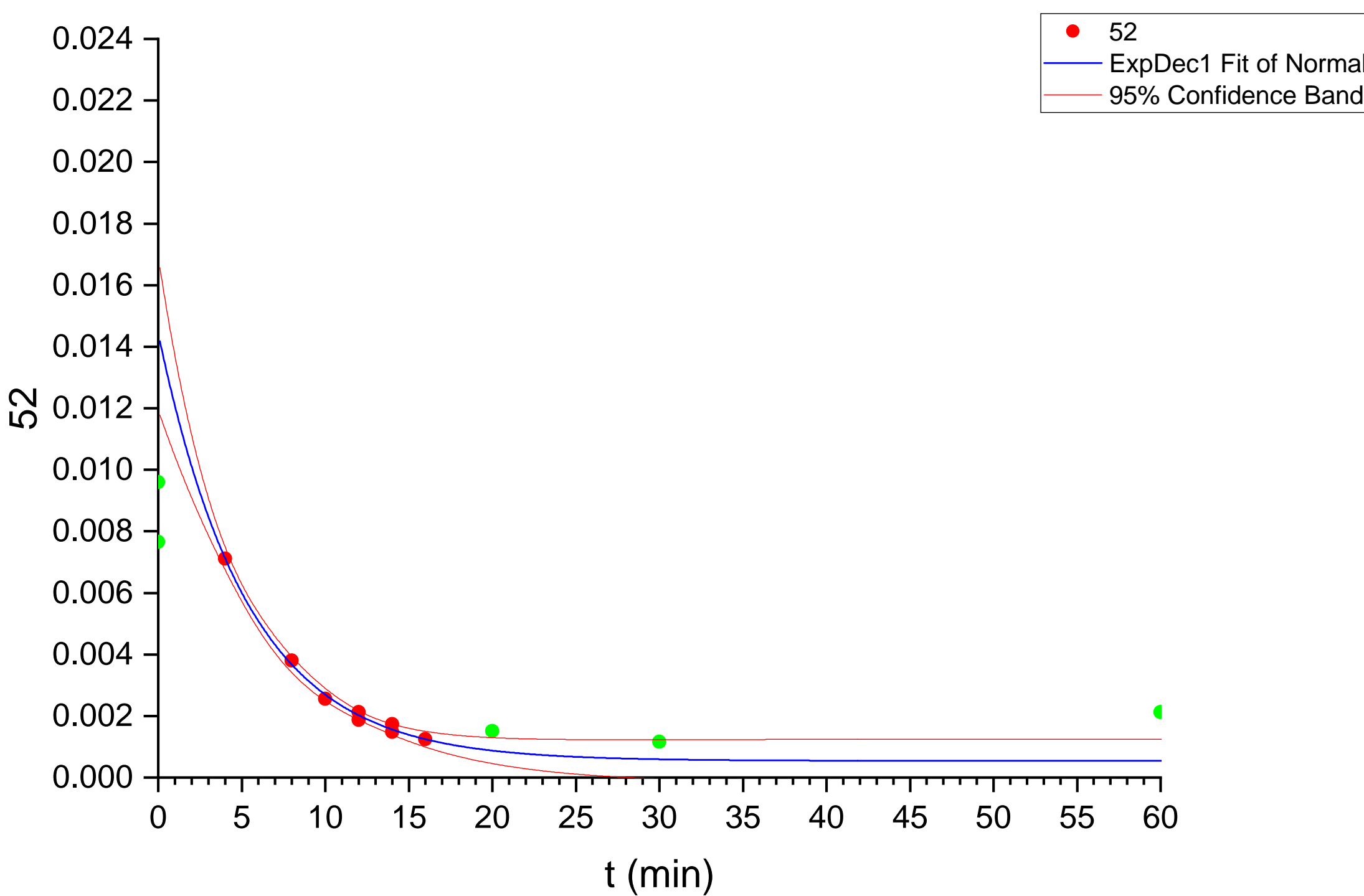

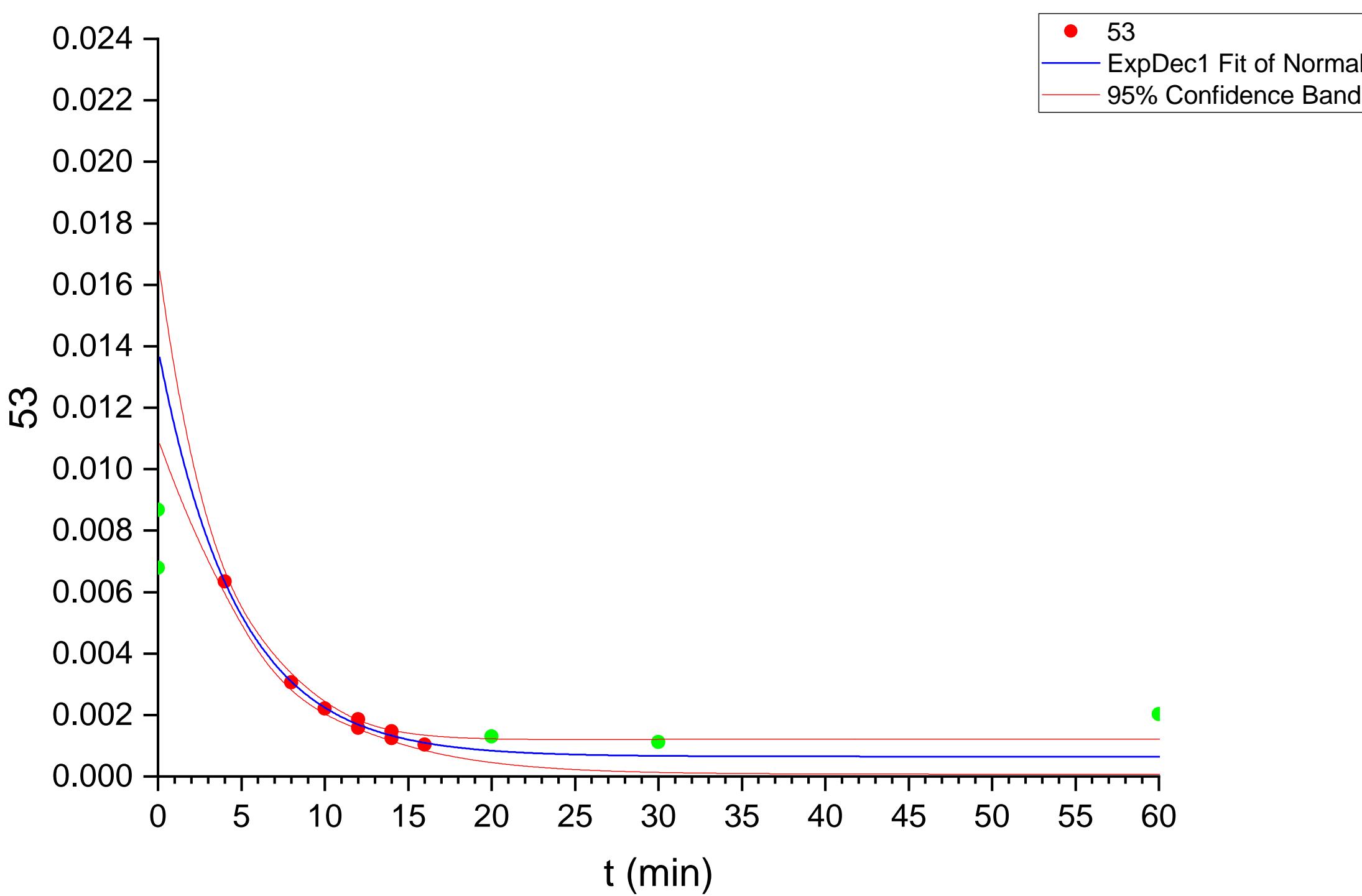

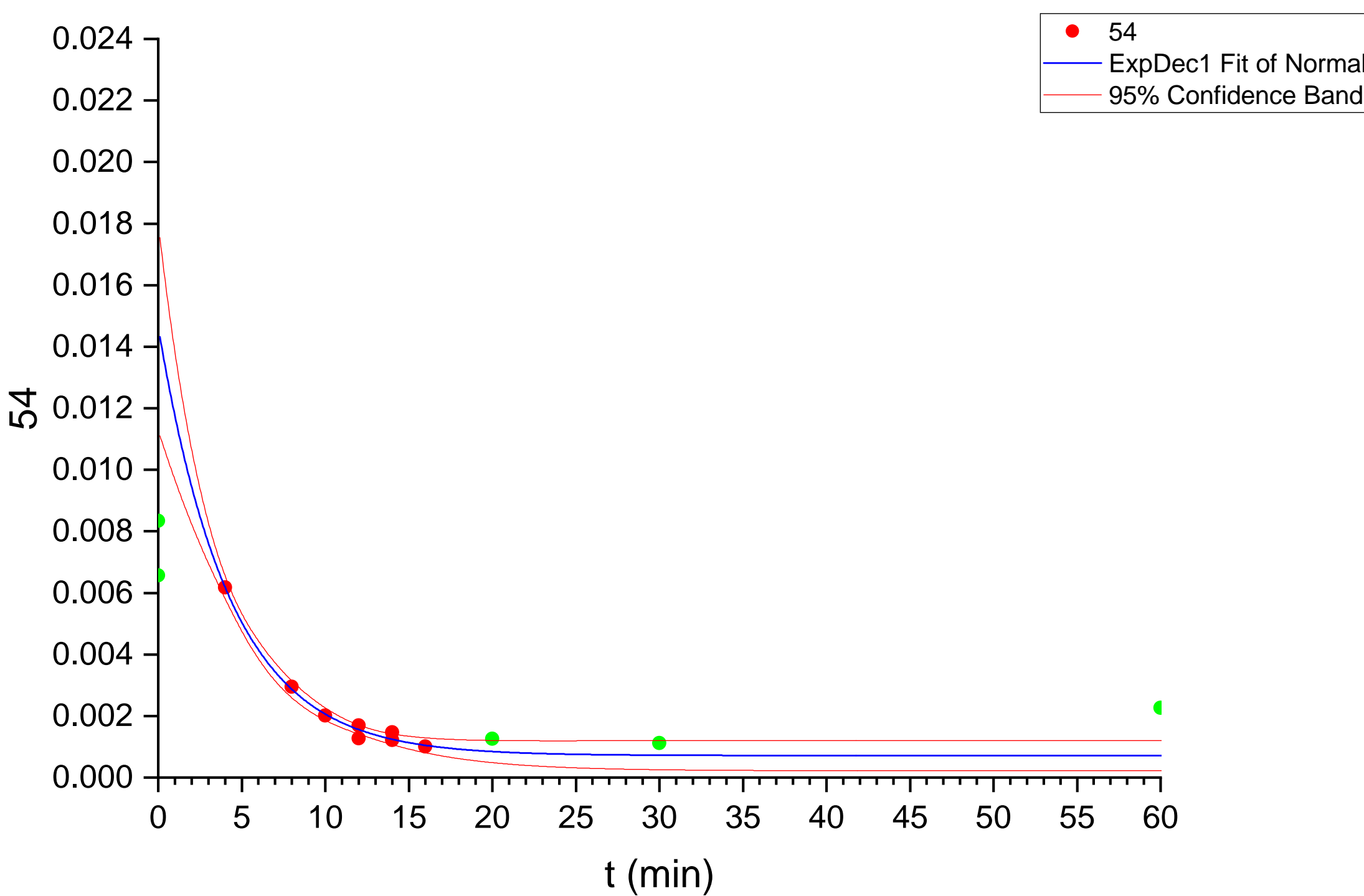

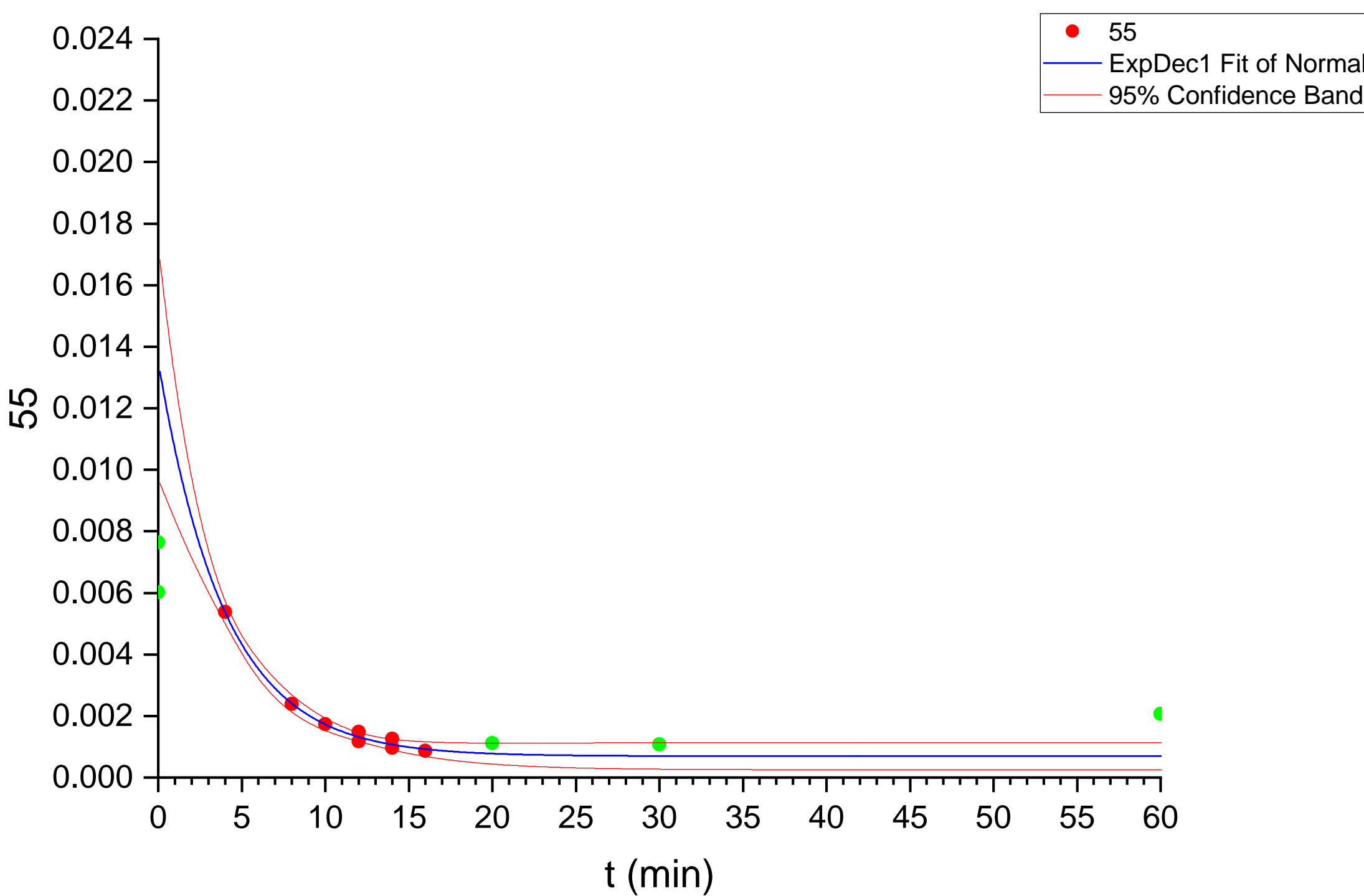

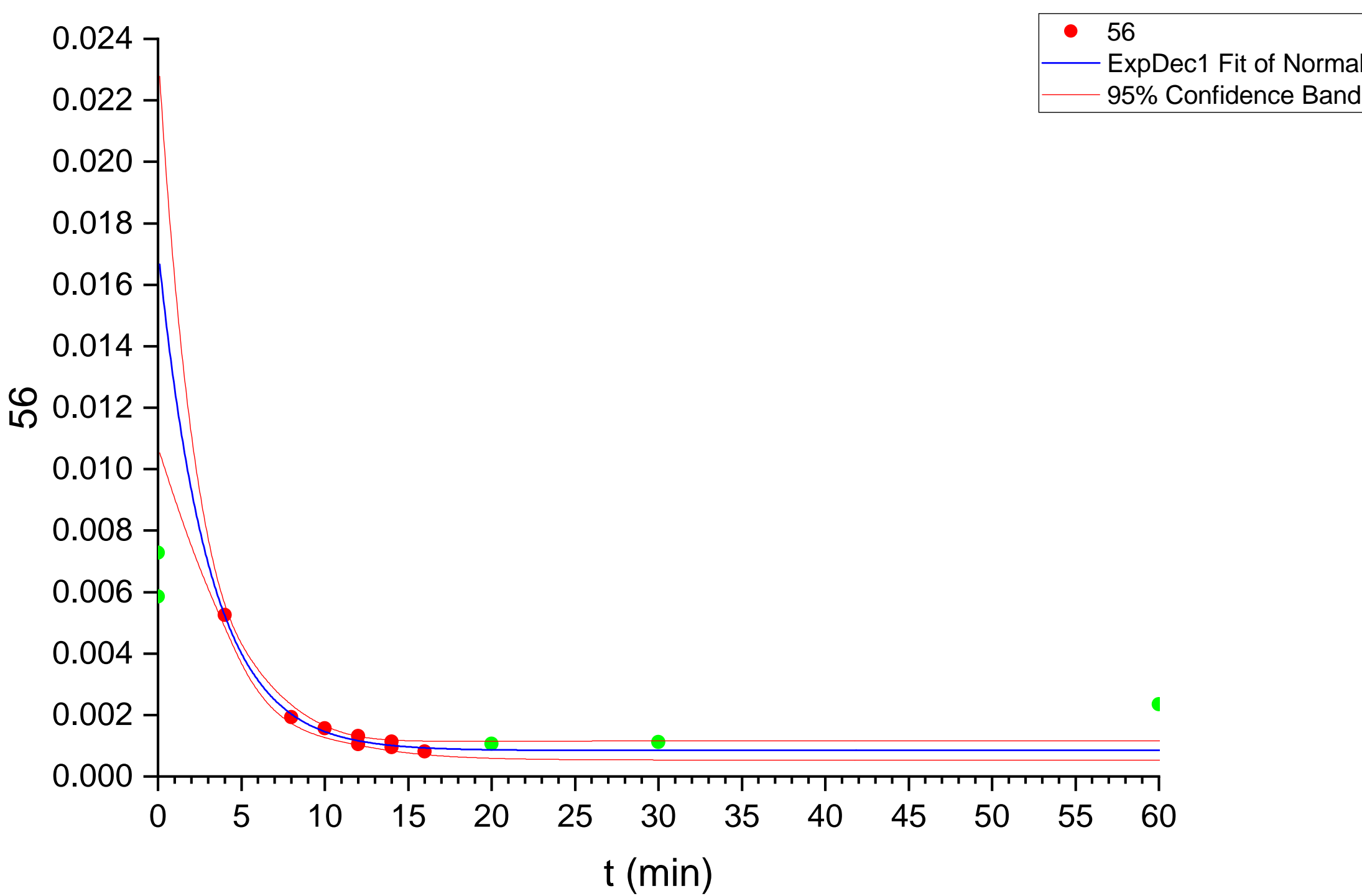

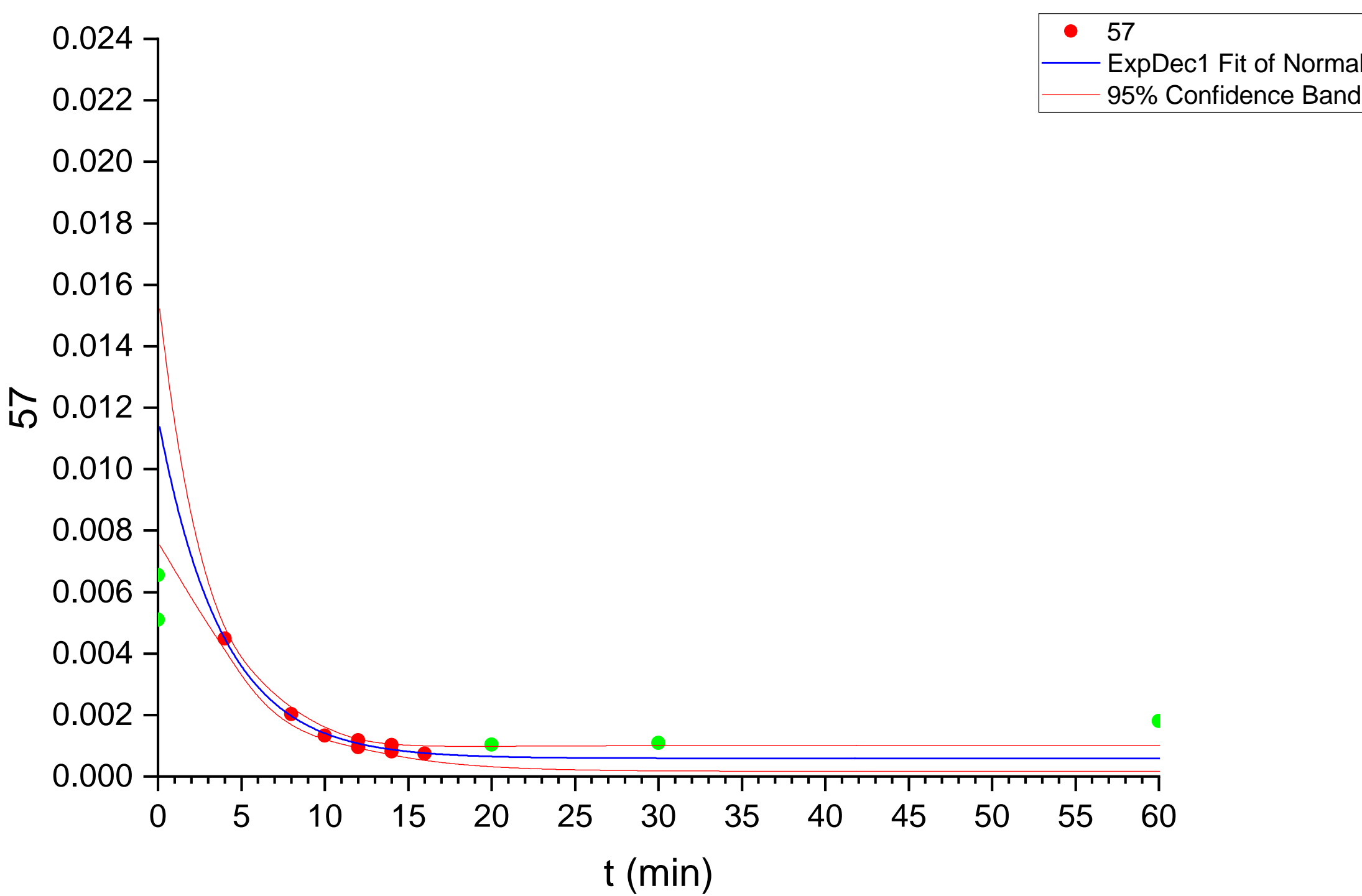

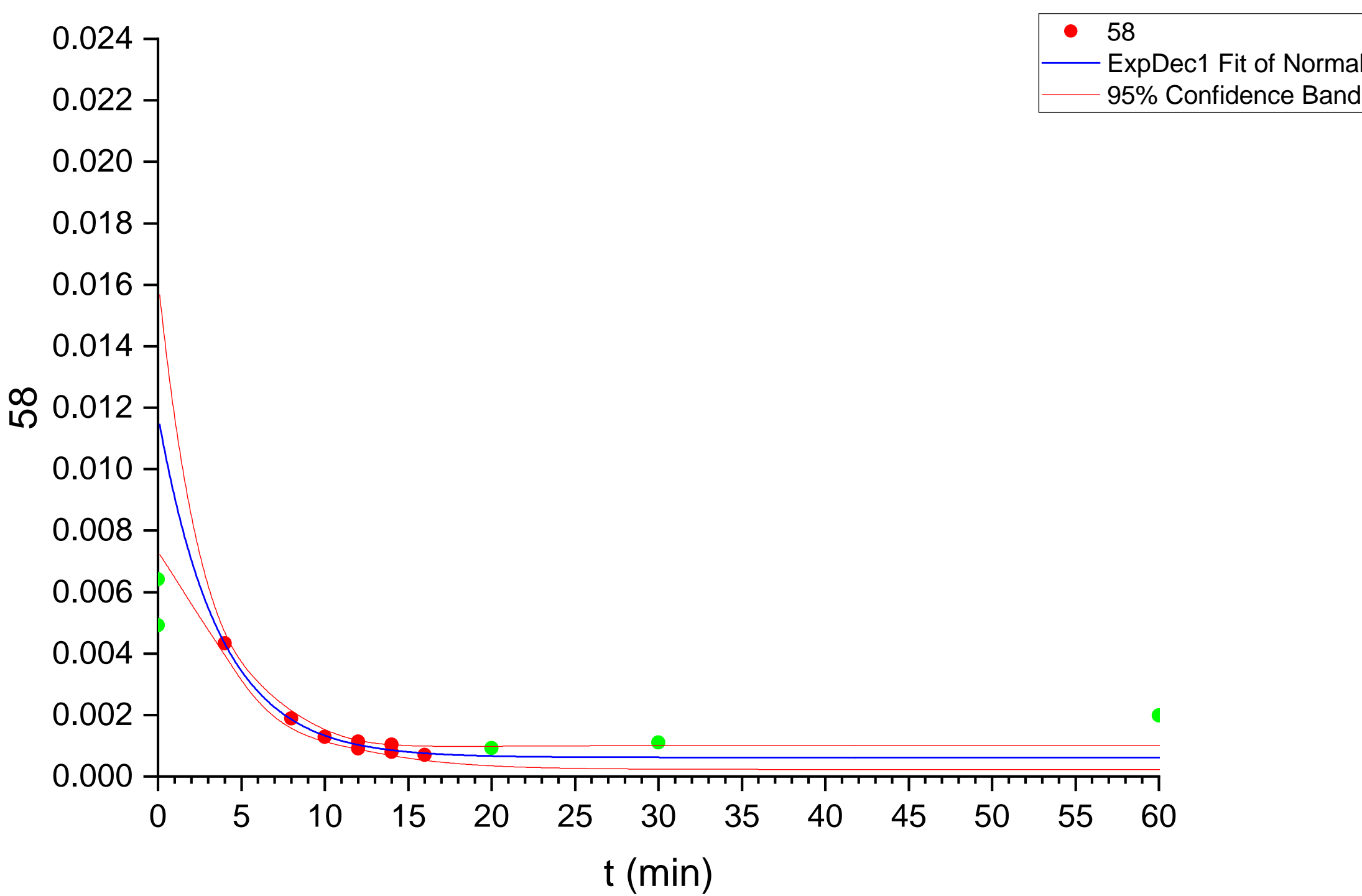

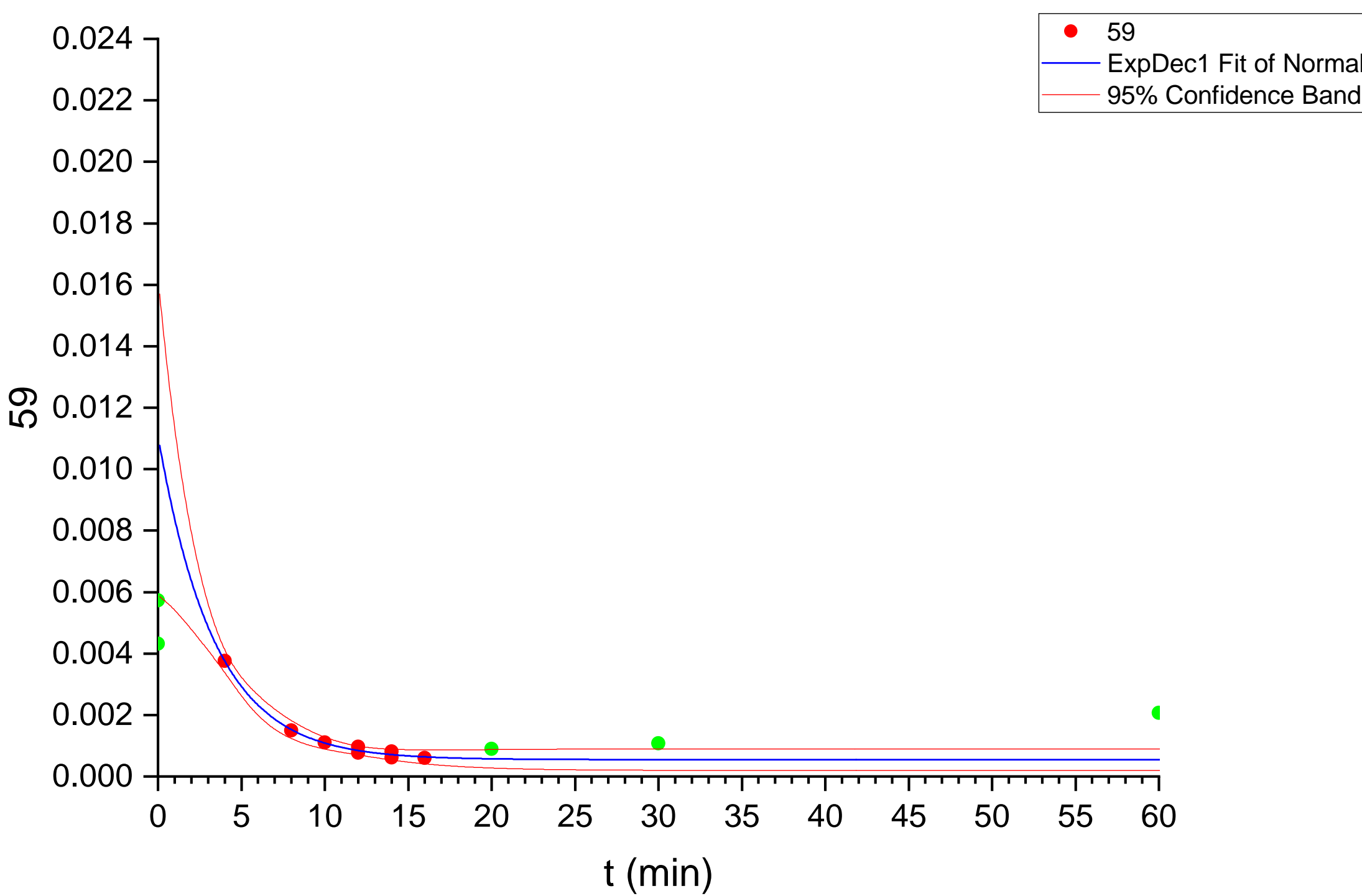

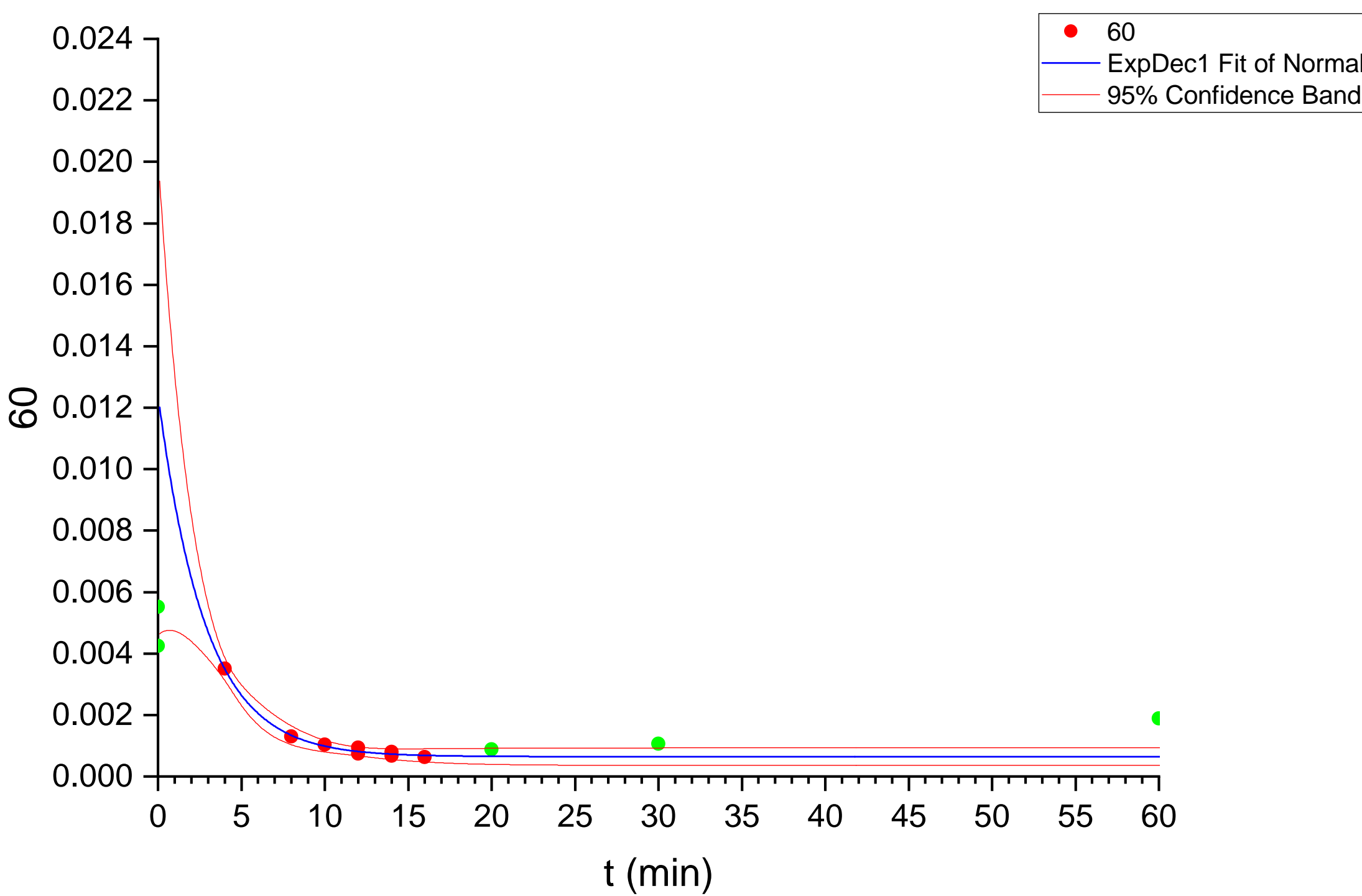

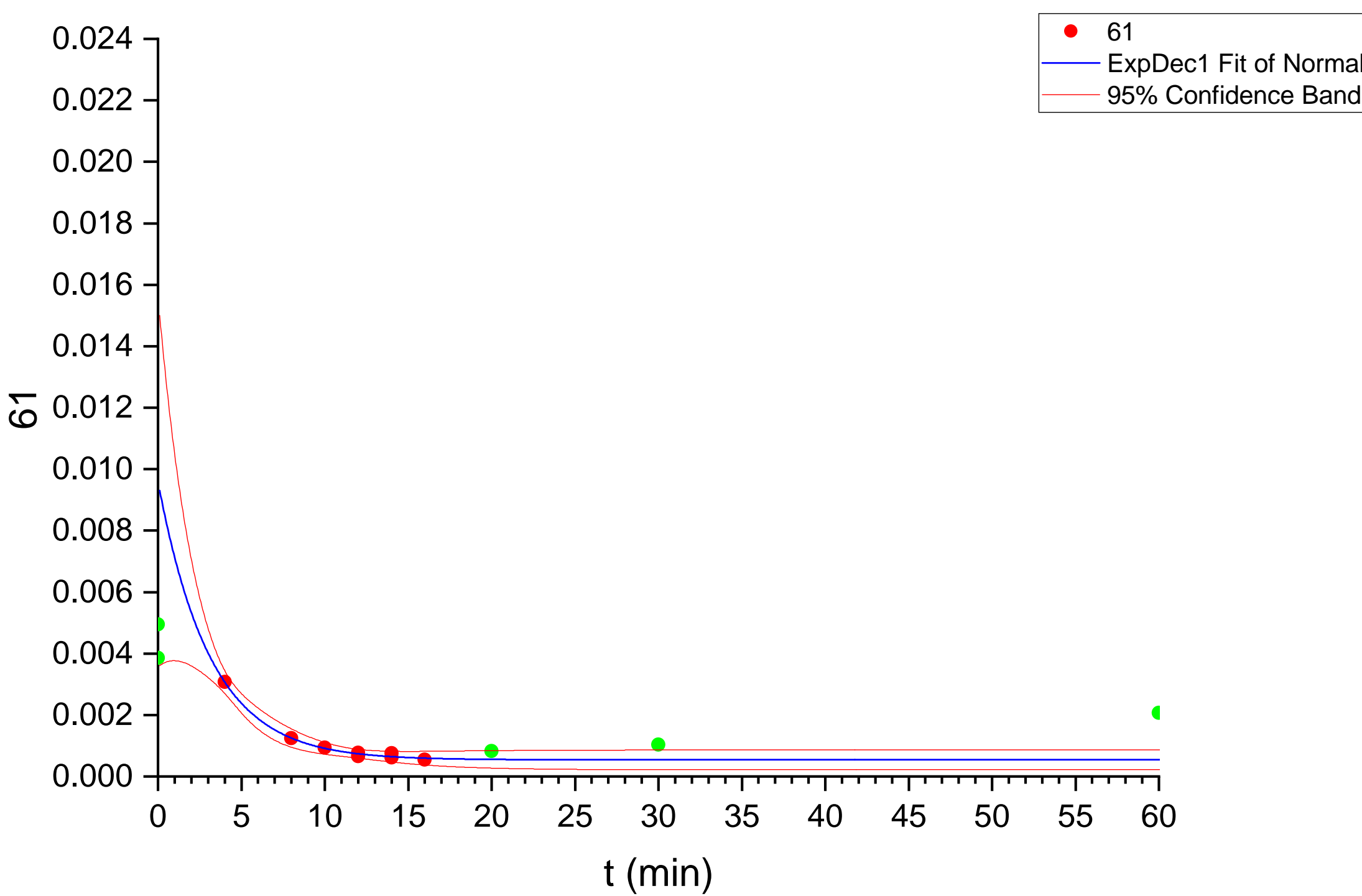

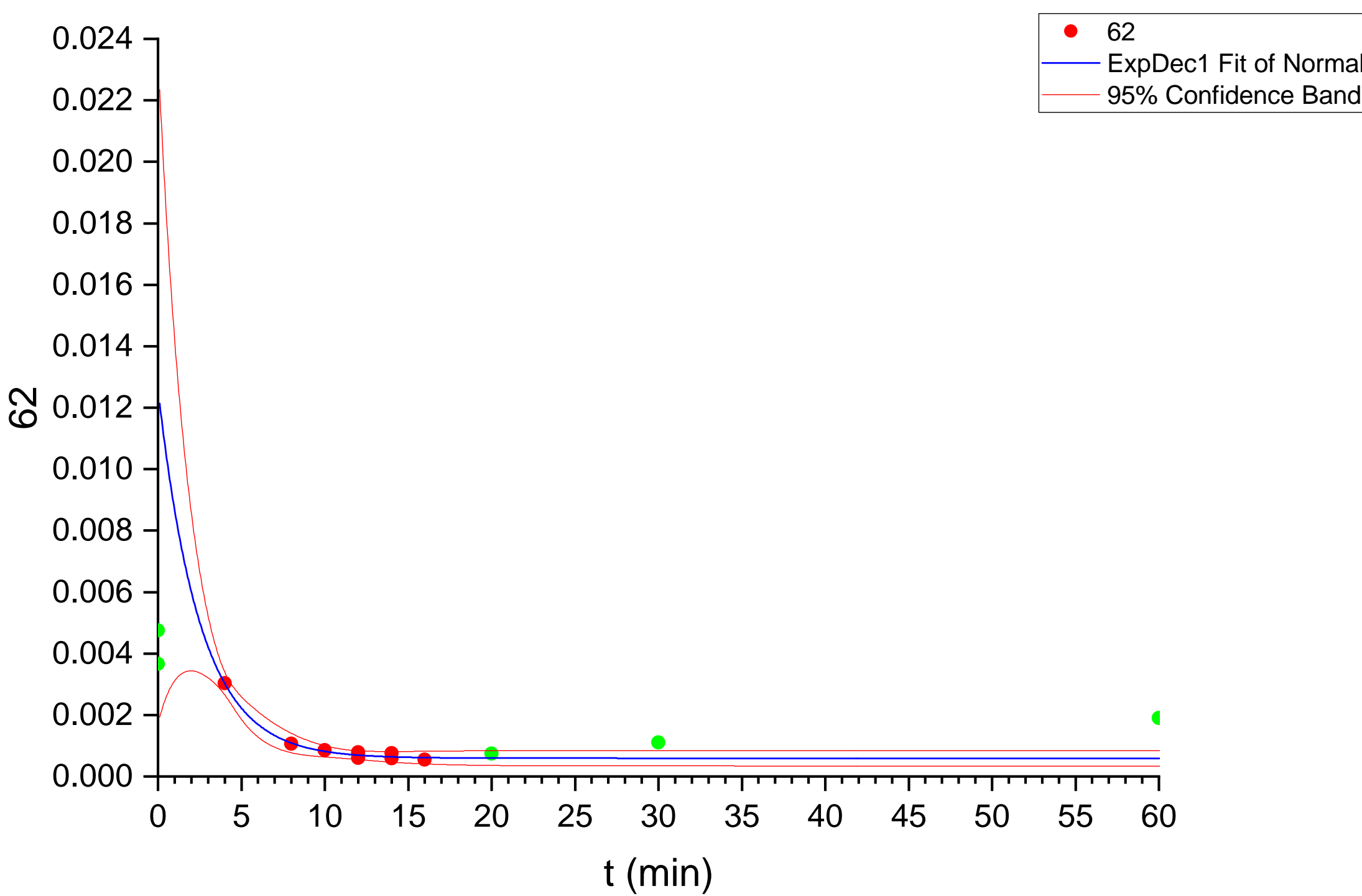

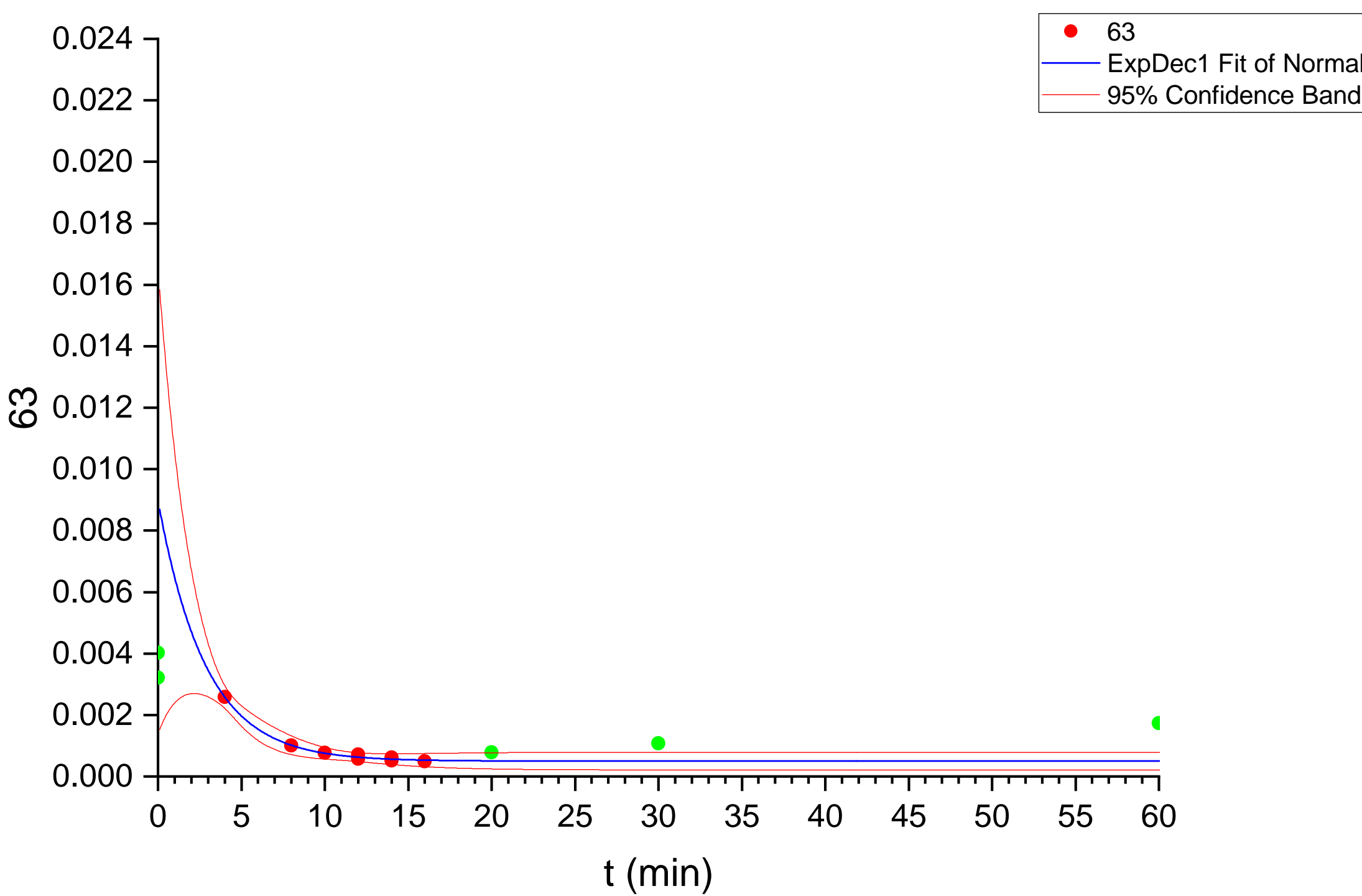

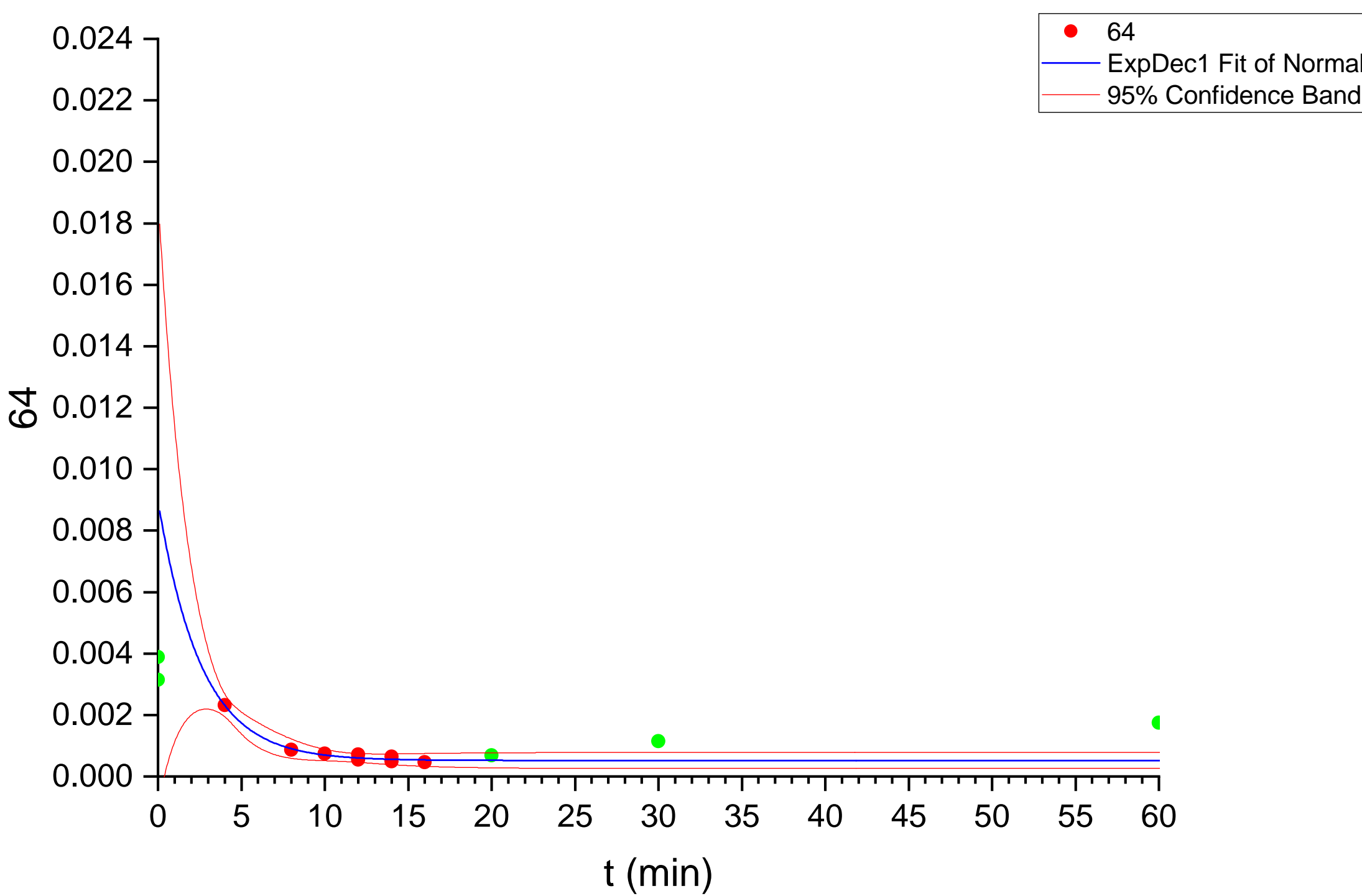

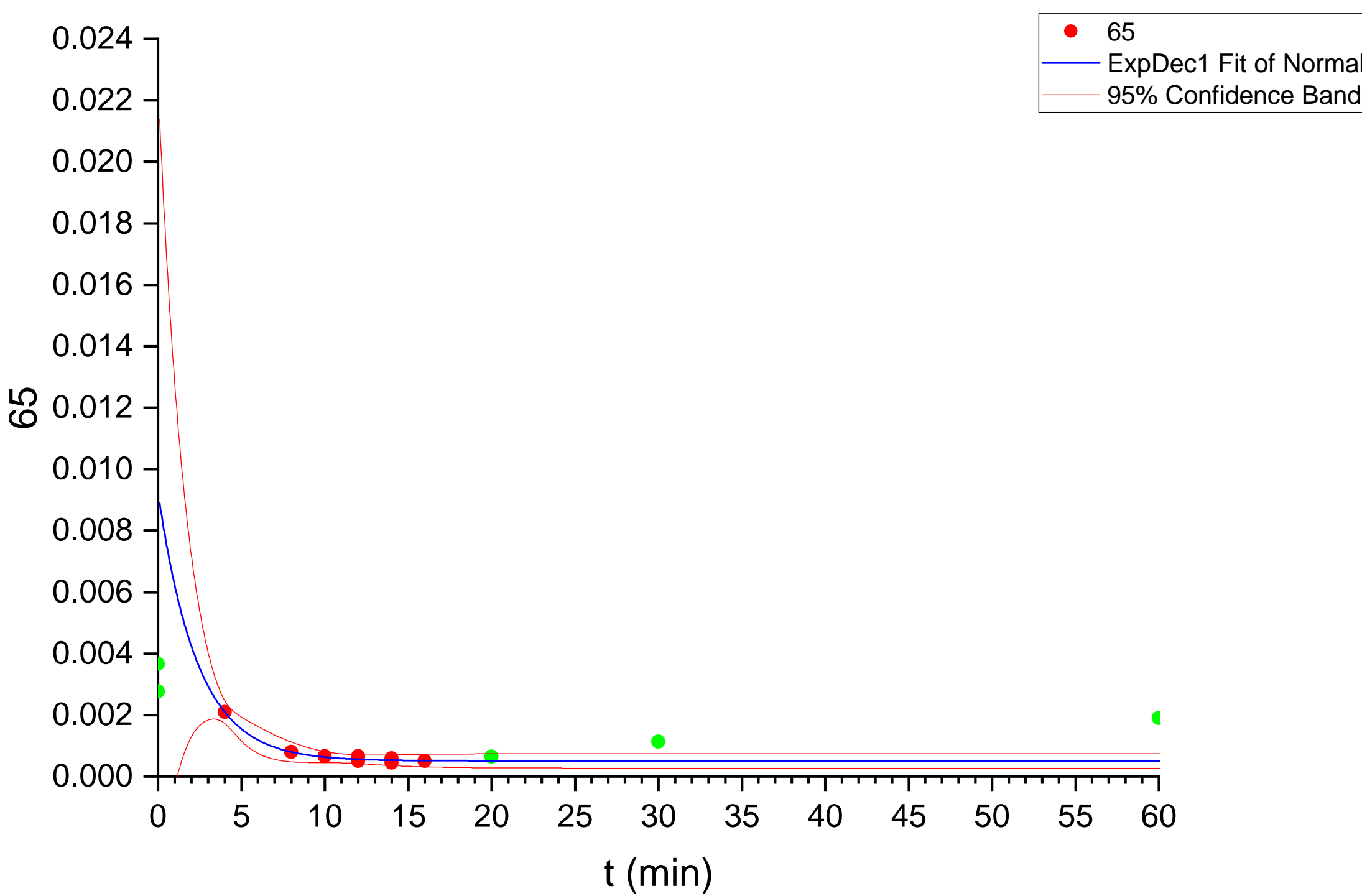

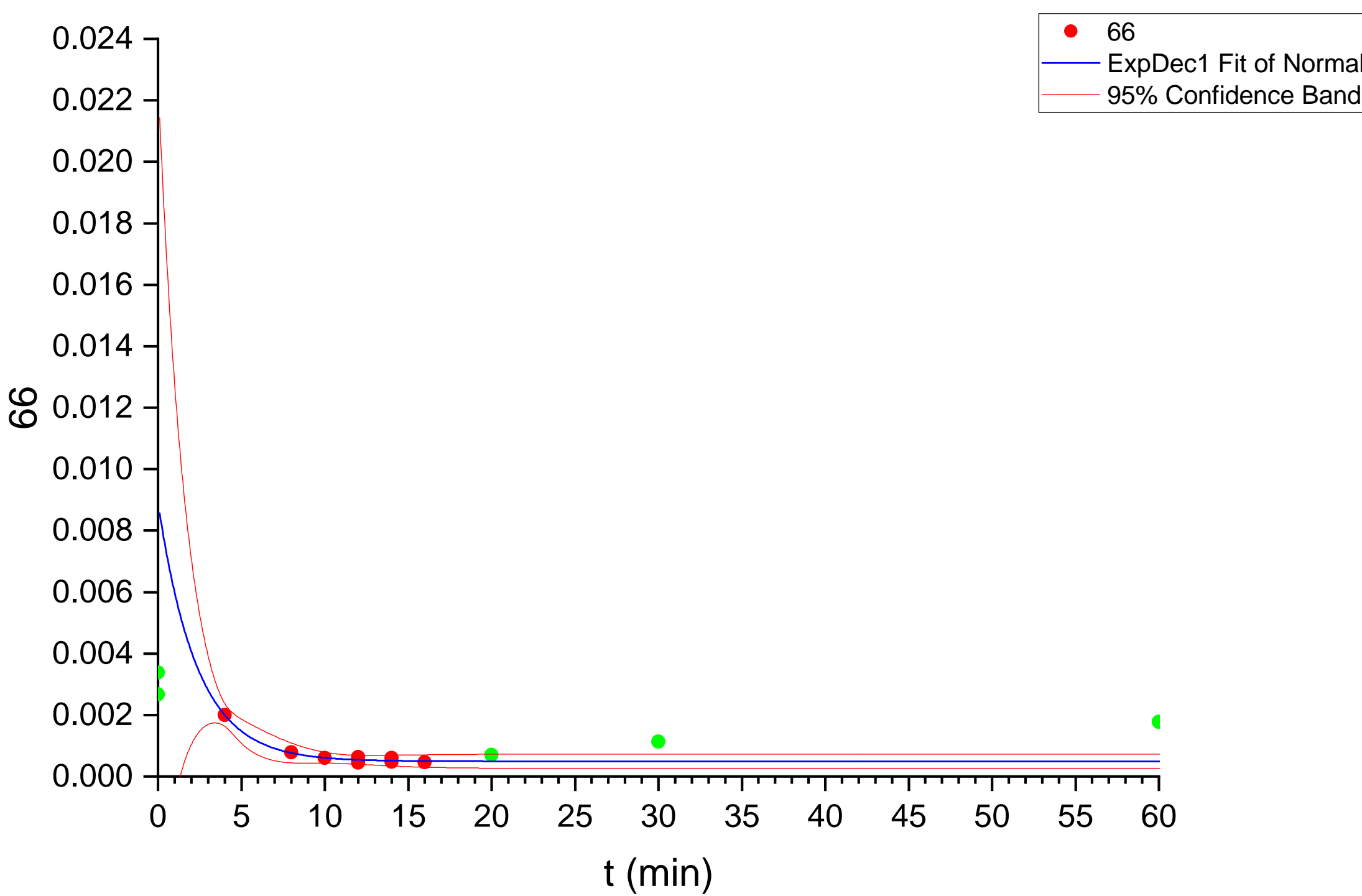

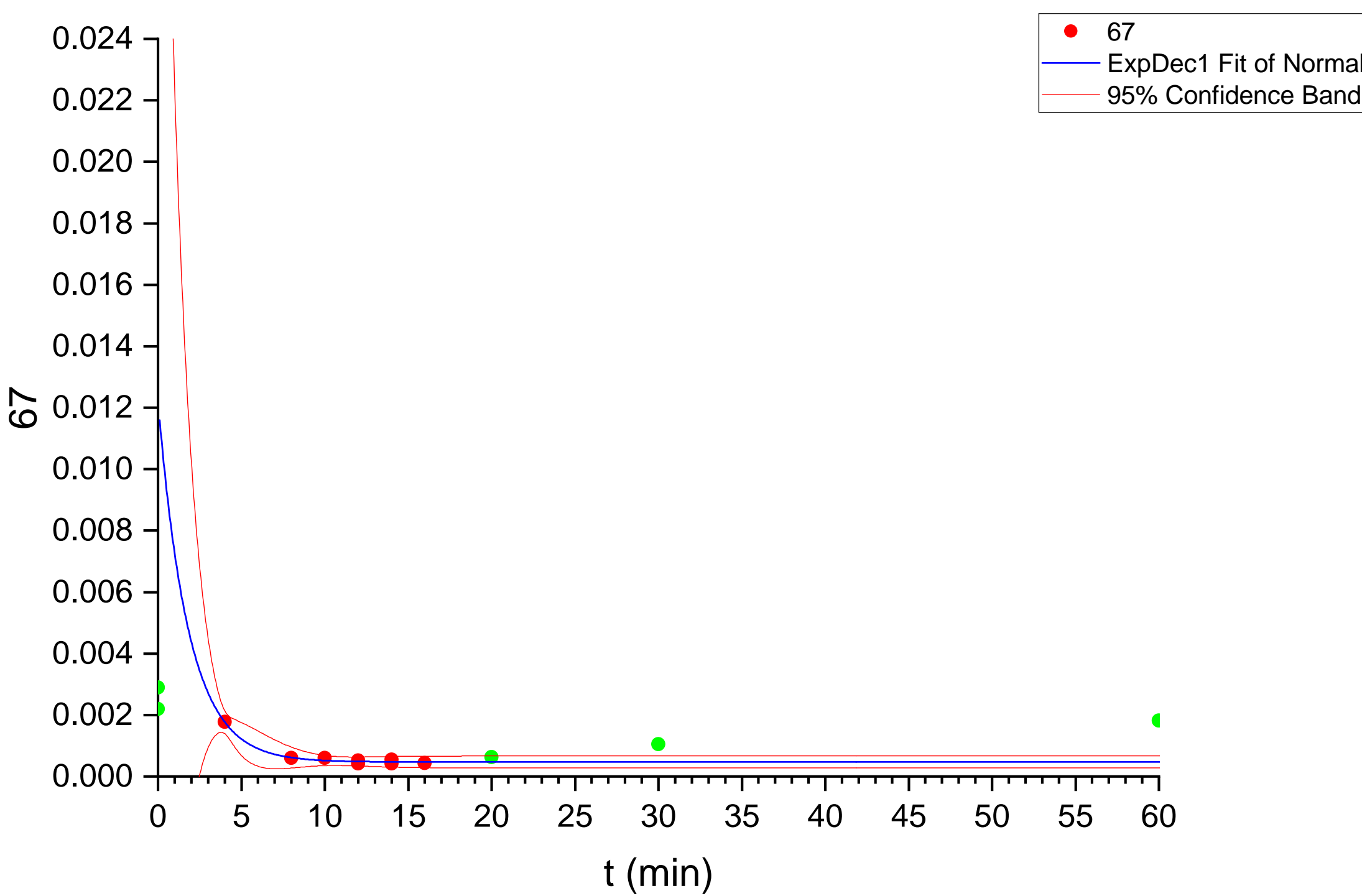

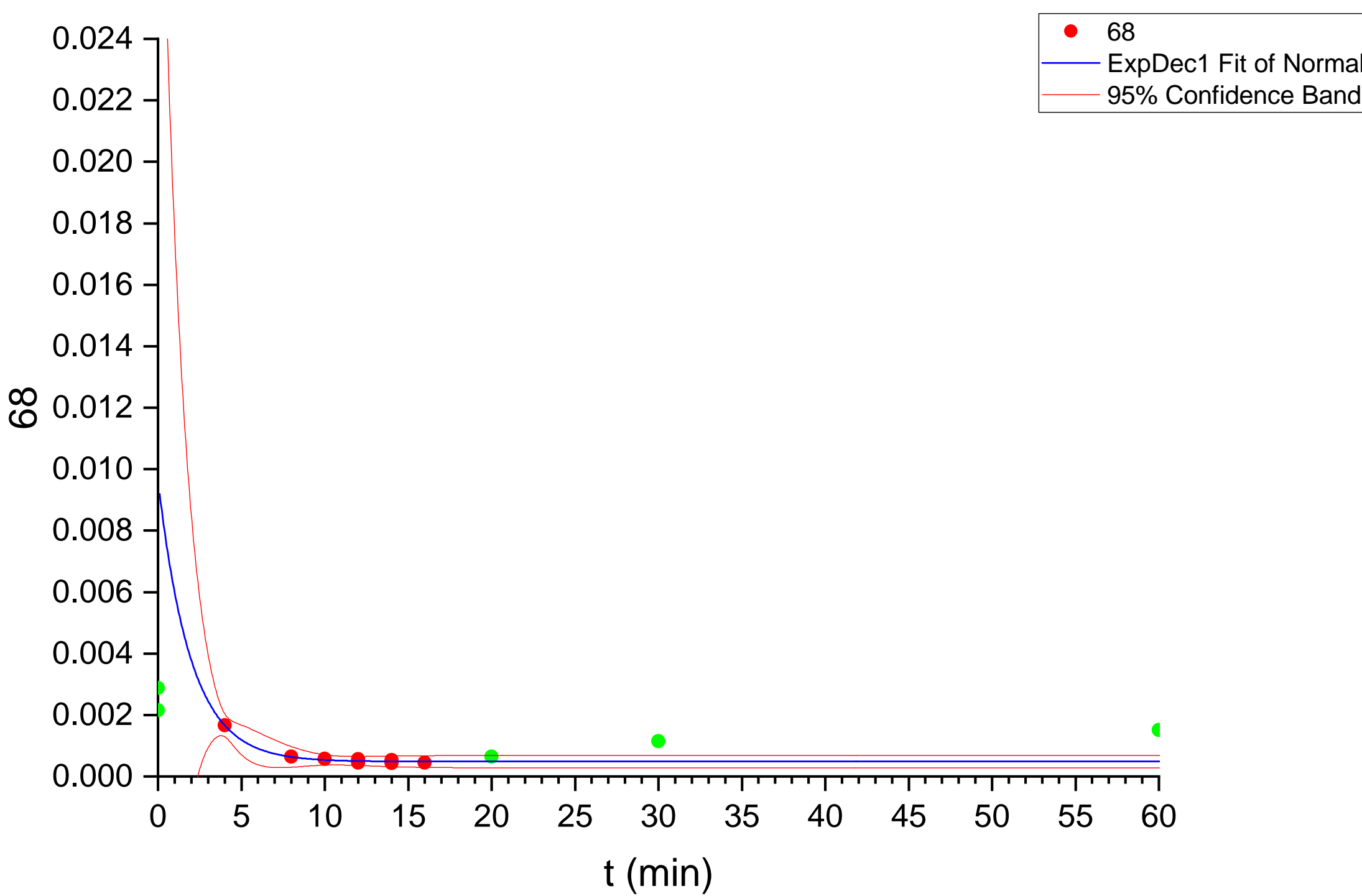

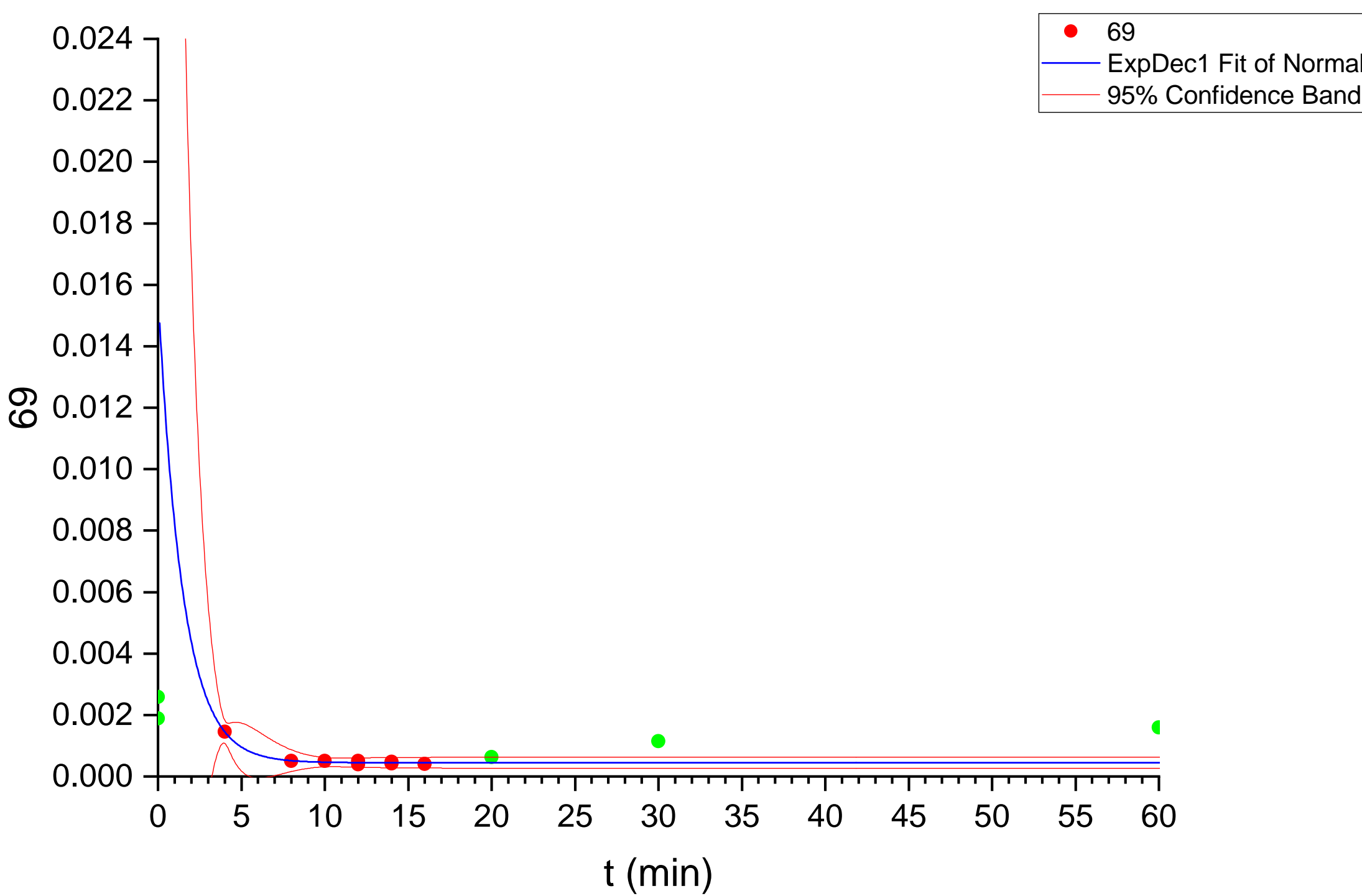

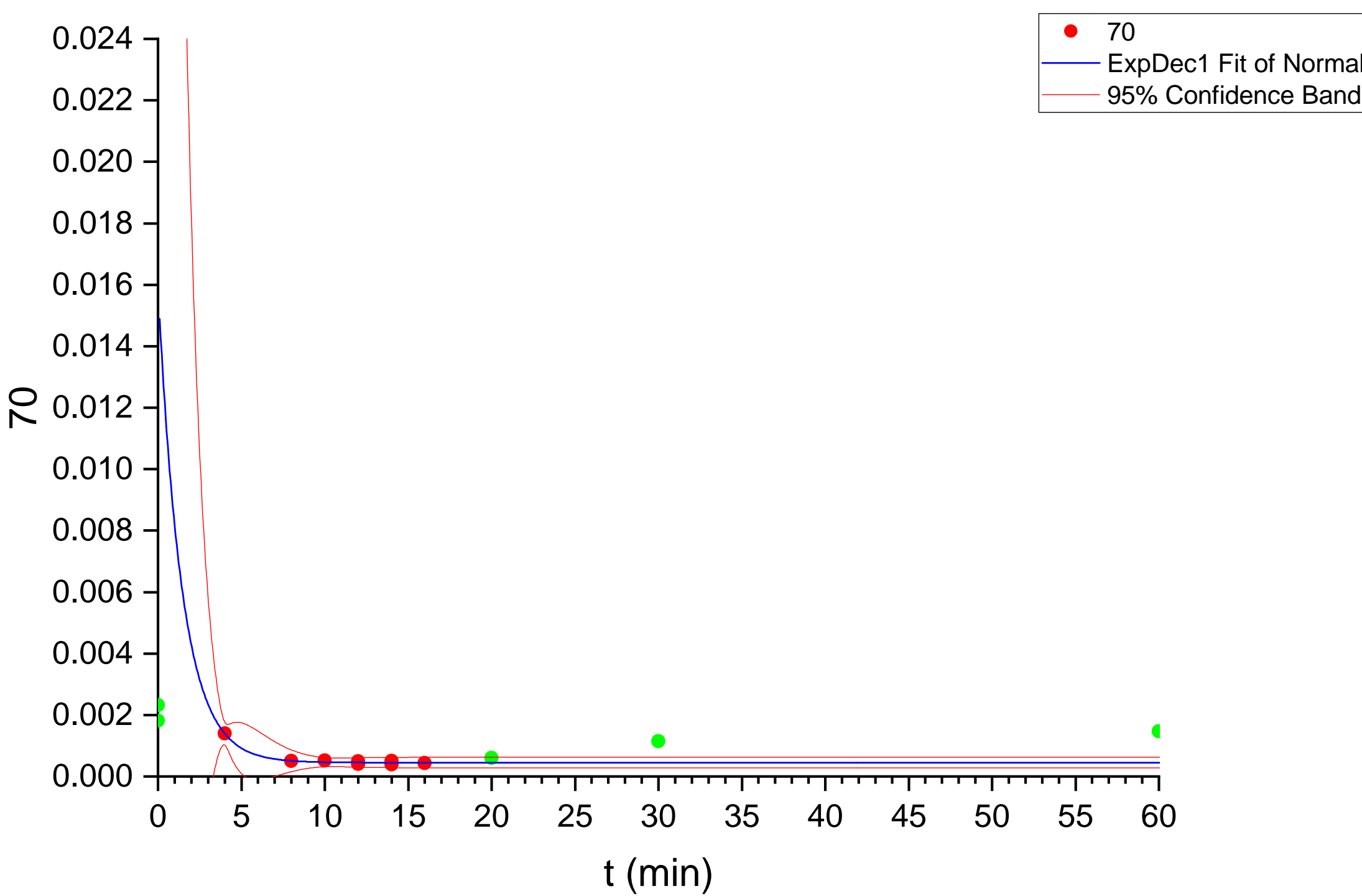

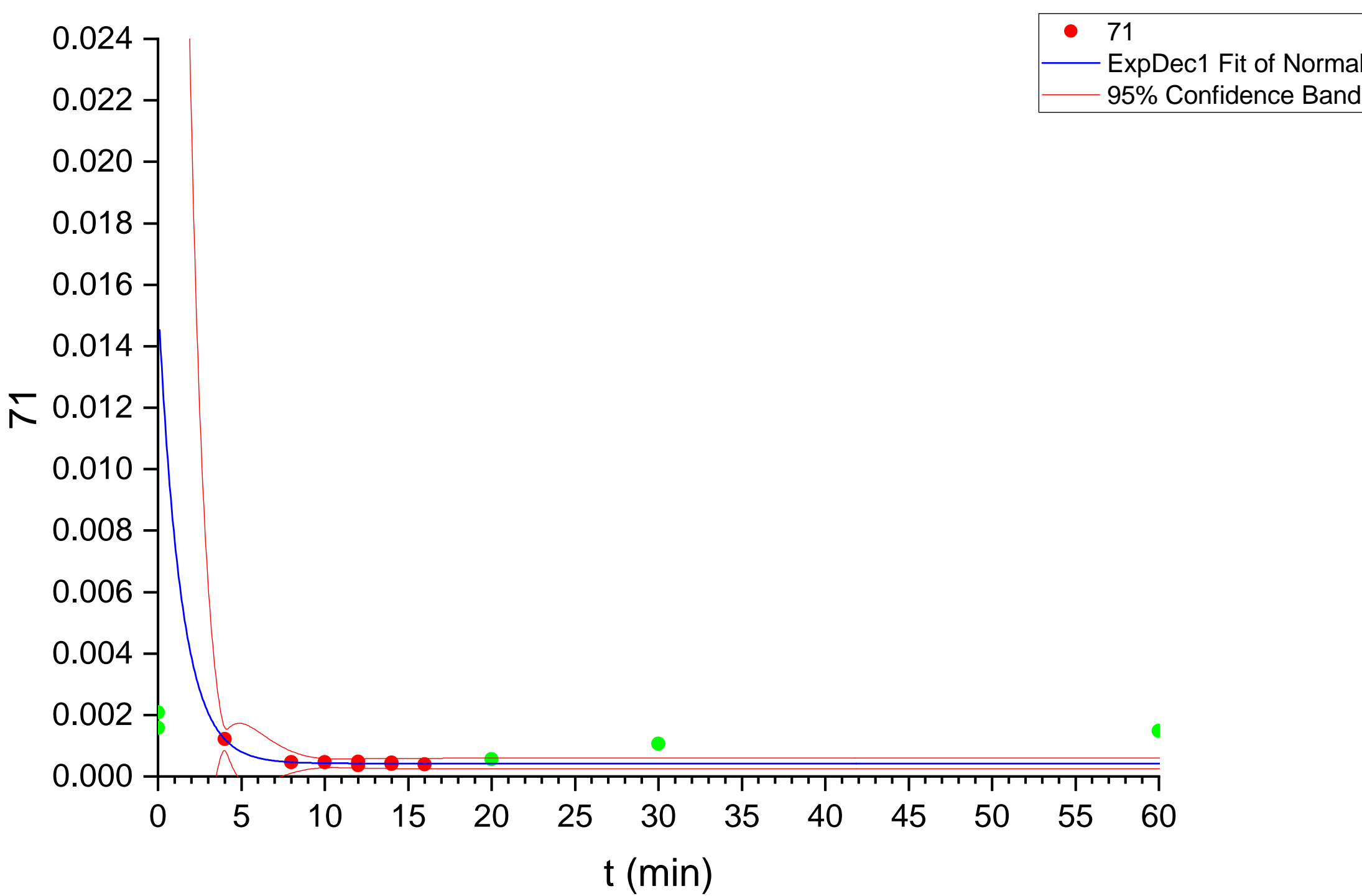

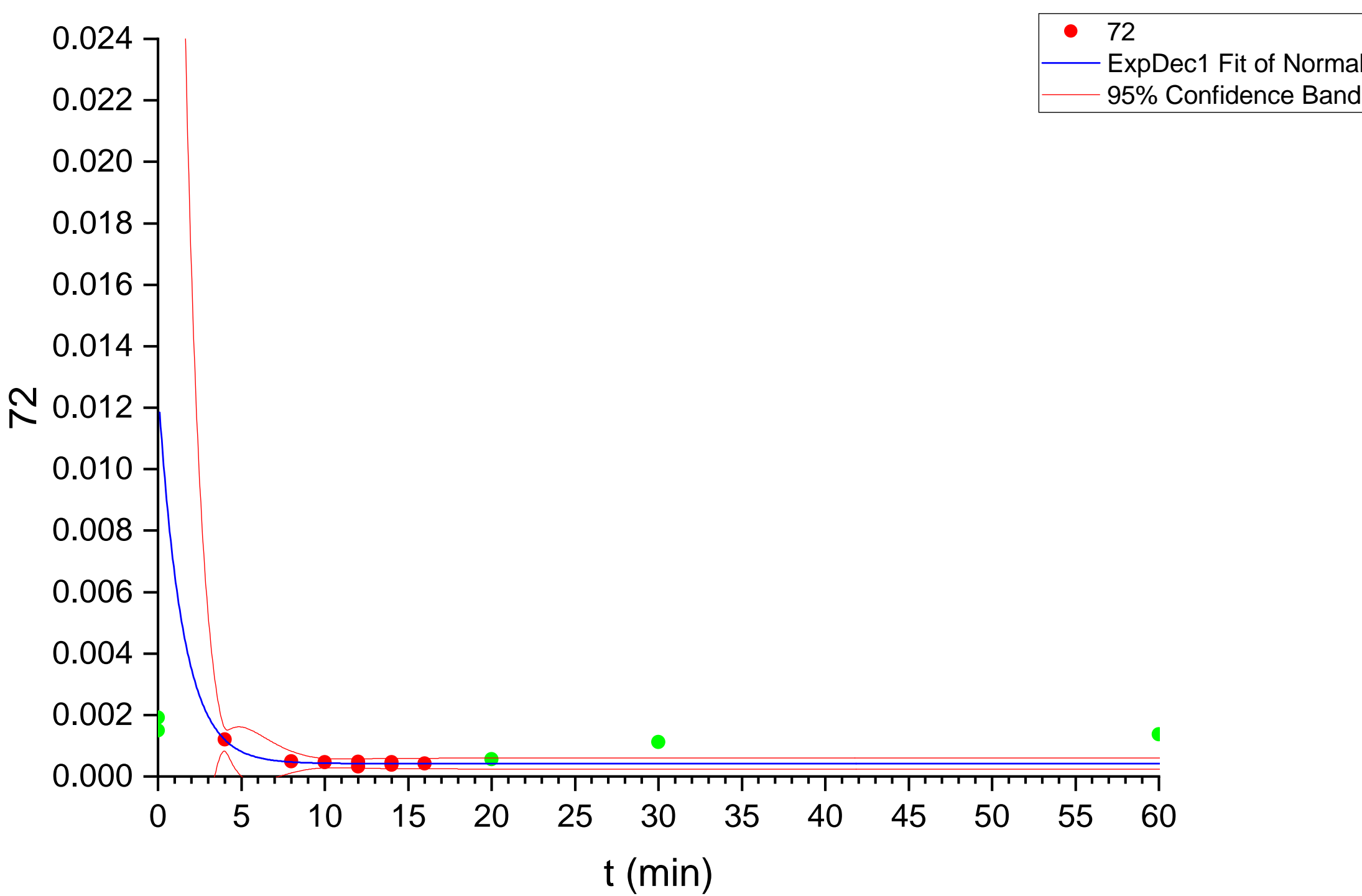

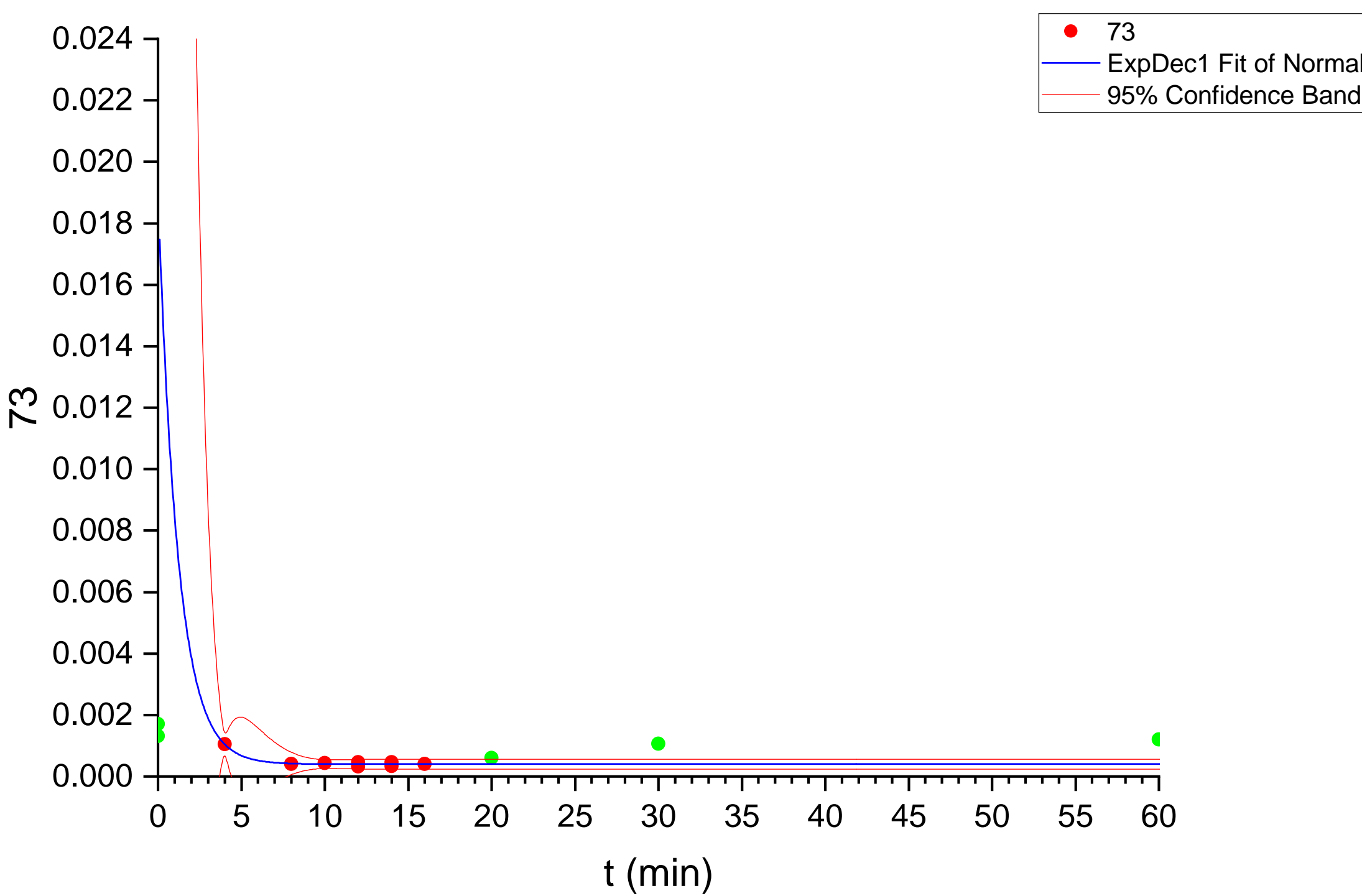

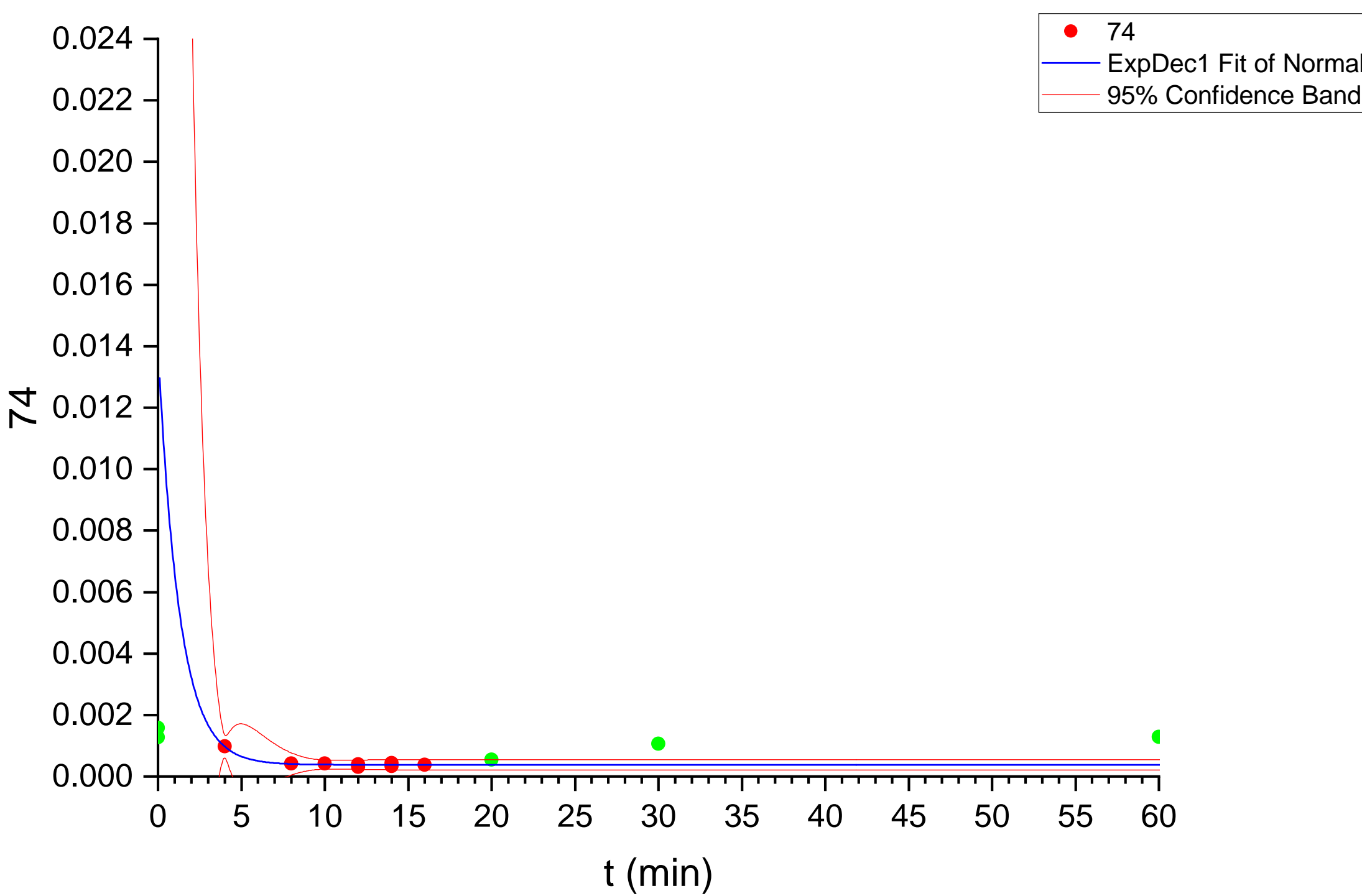

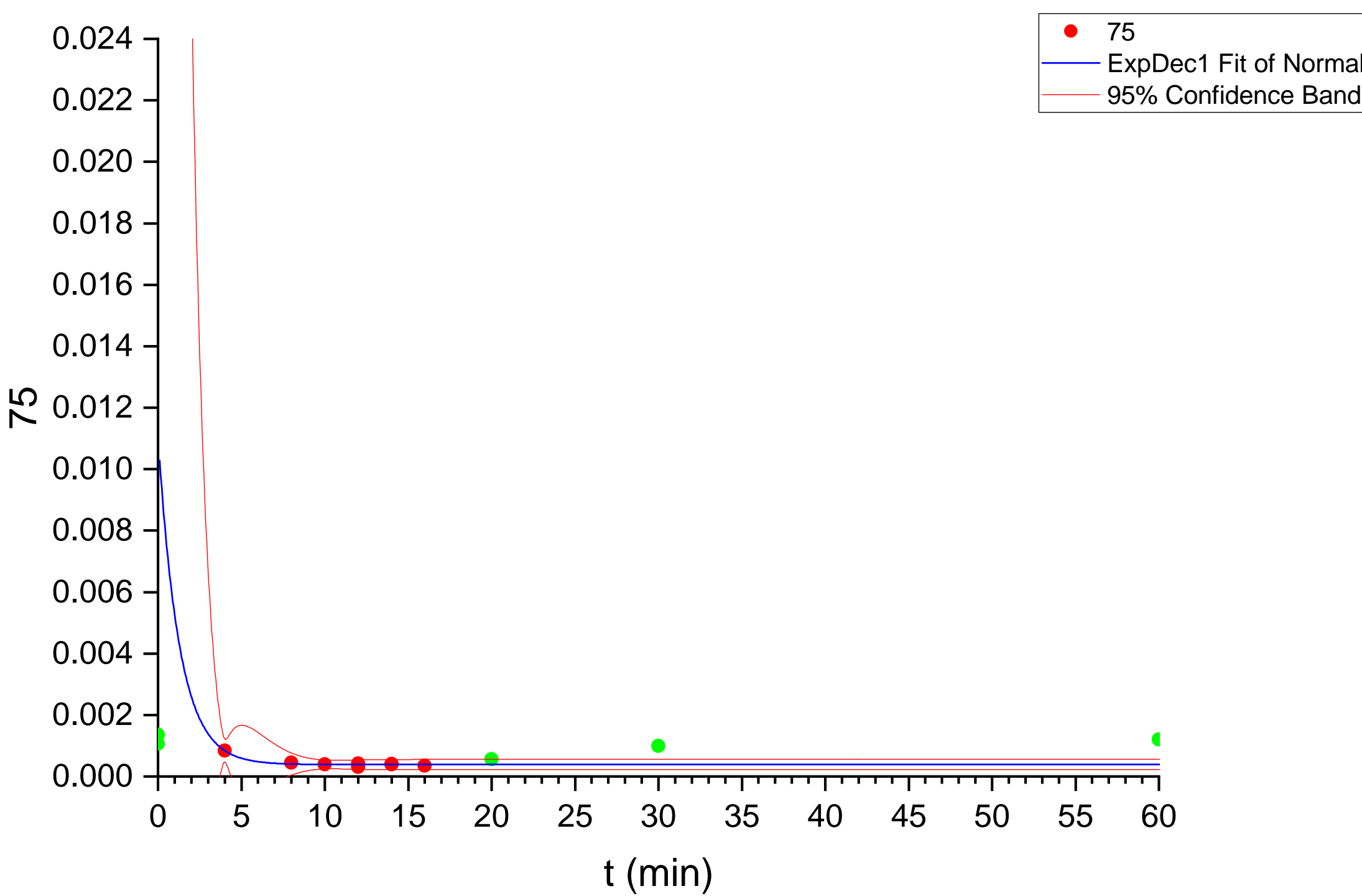

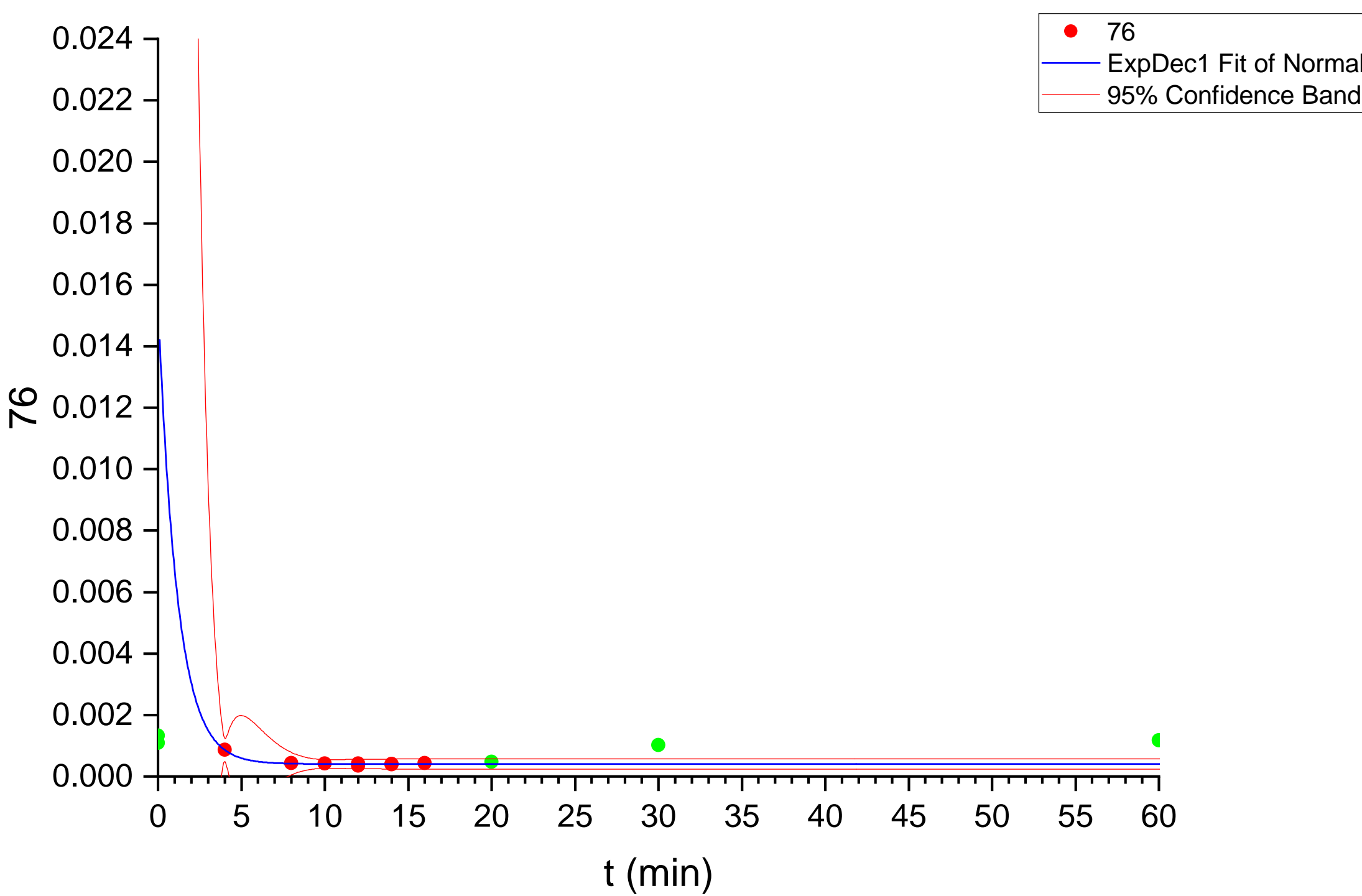

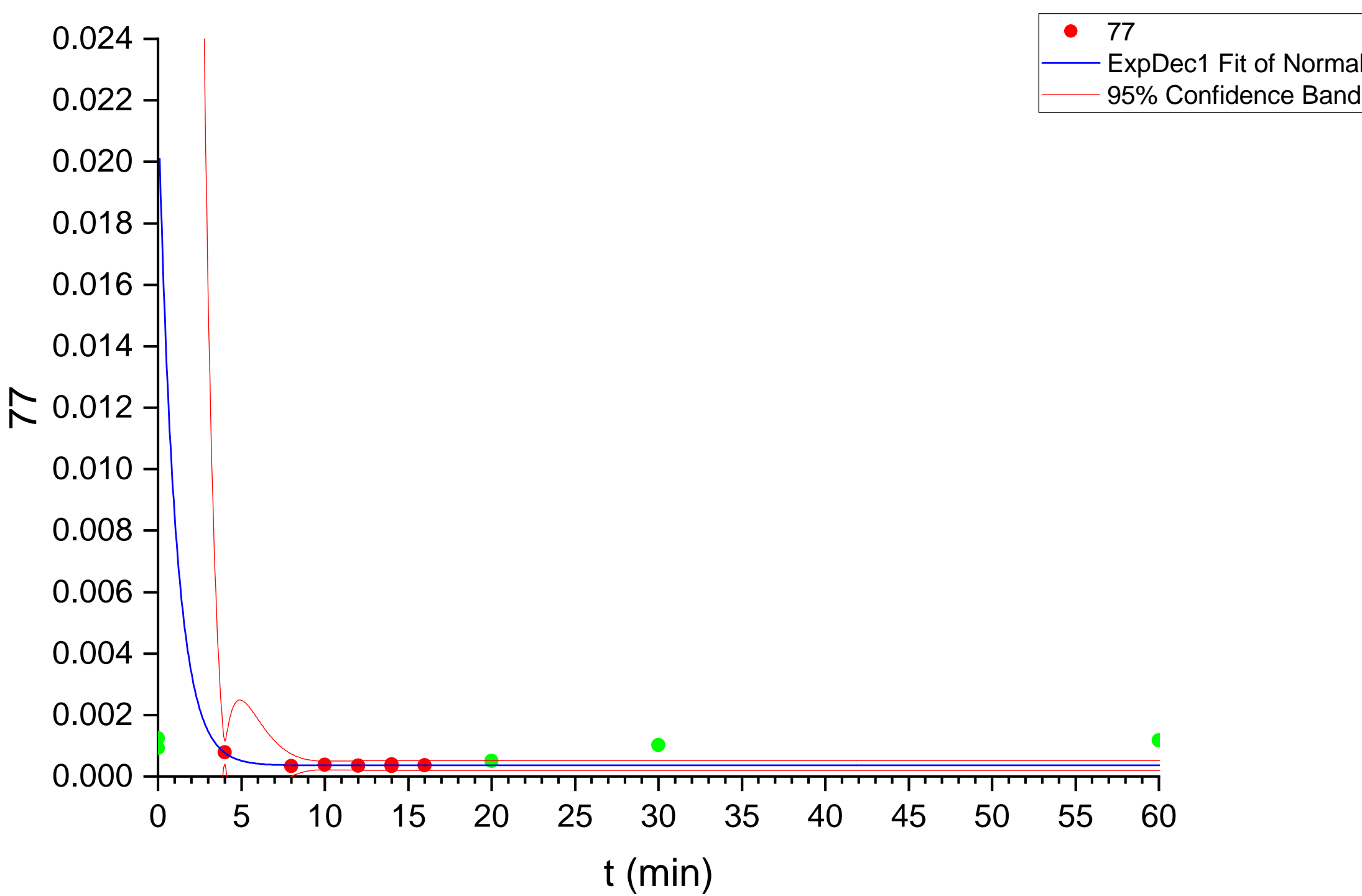

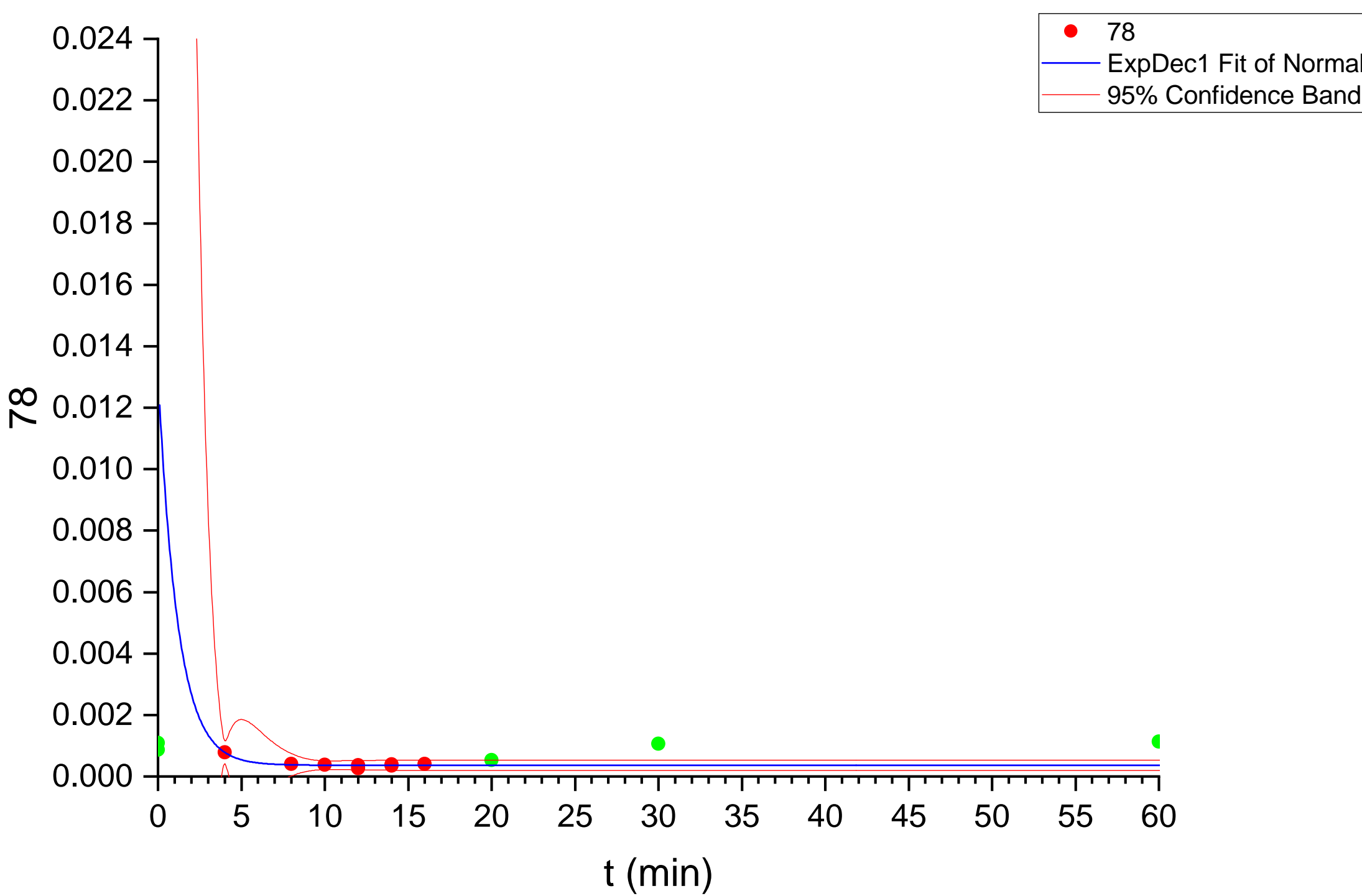

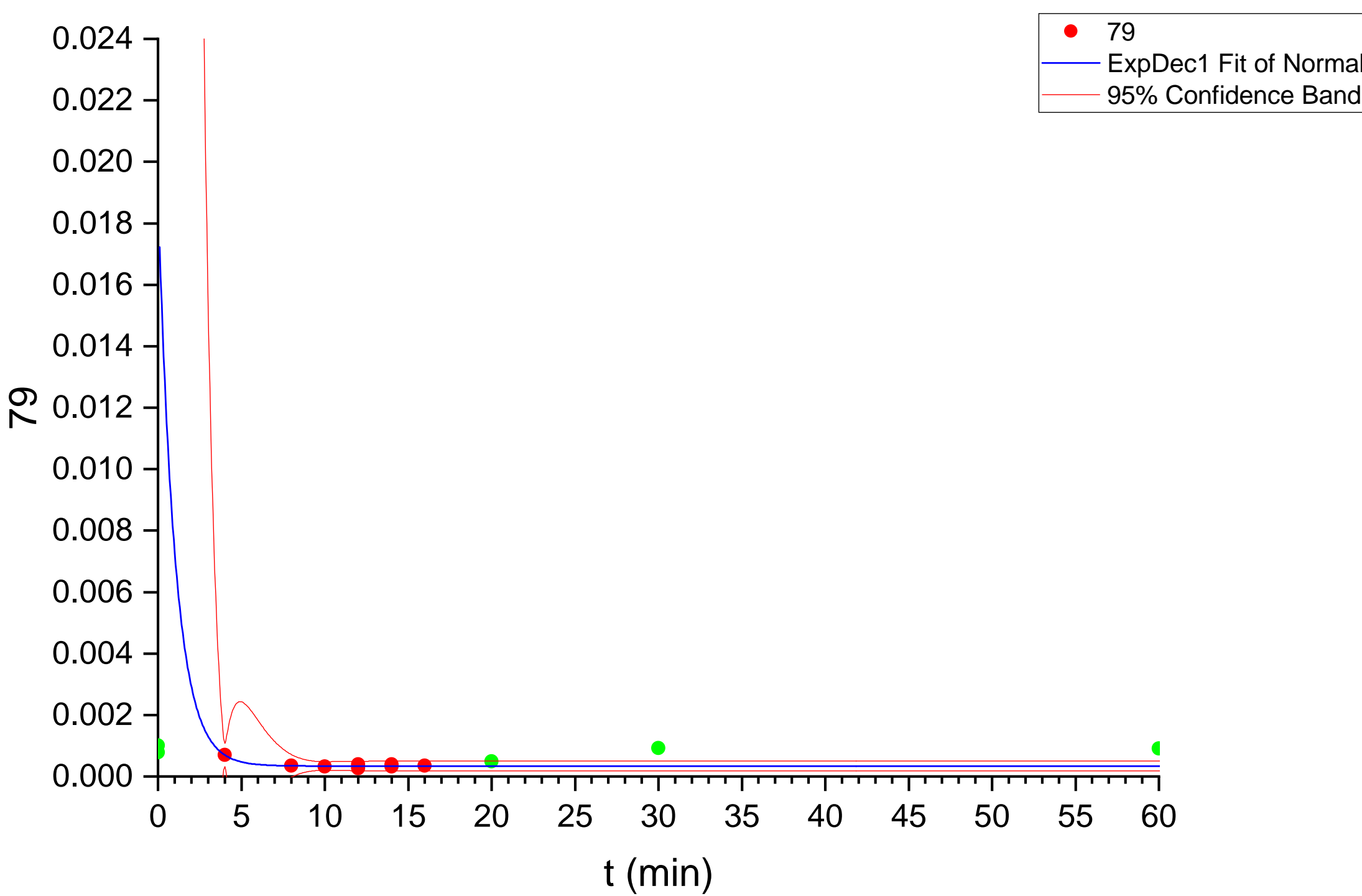

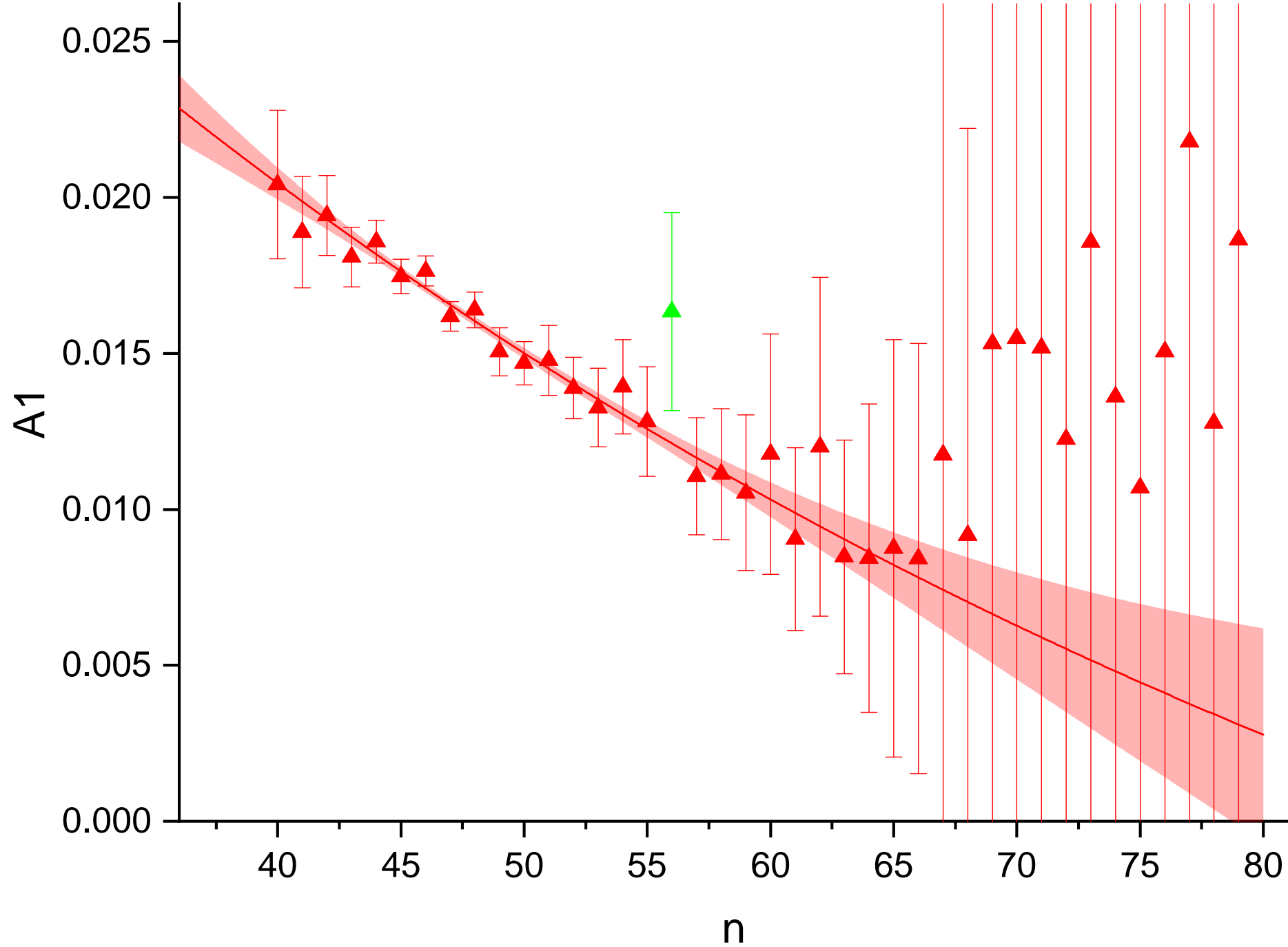

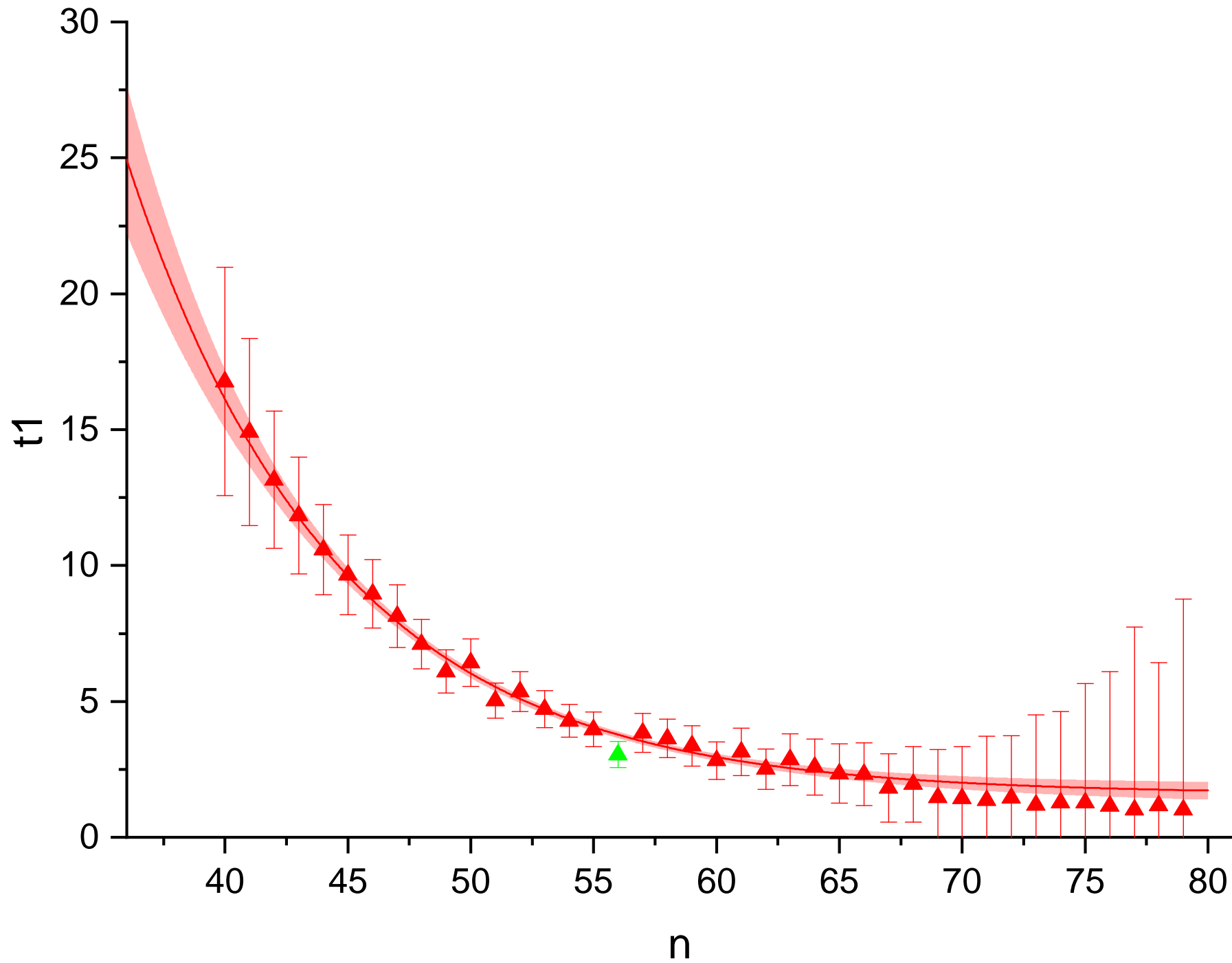

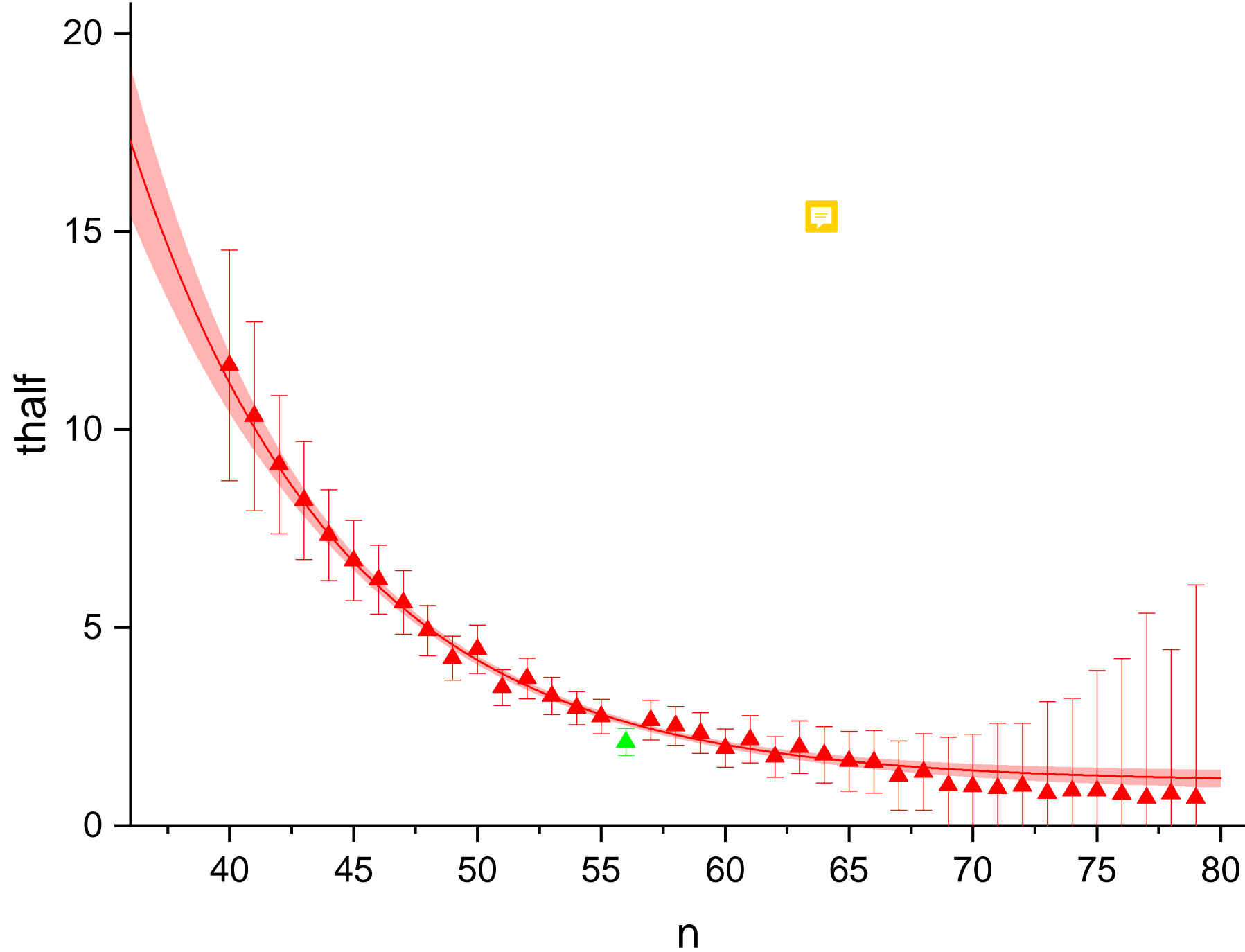

Supplement: Supplementary file 10 — Source data Fig. 4 [file 44318_2024_258_MOESM10_ESM.zip › Figure 4/FIGURE 4I/JP4AT-local_40-79.pdf]

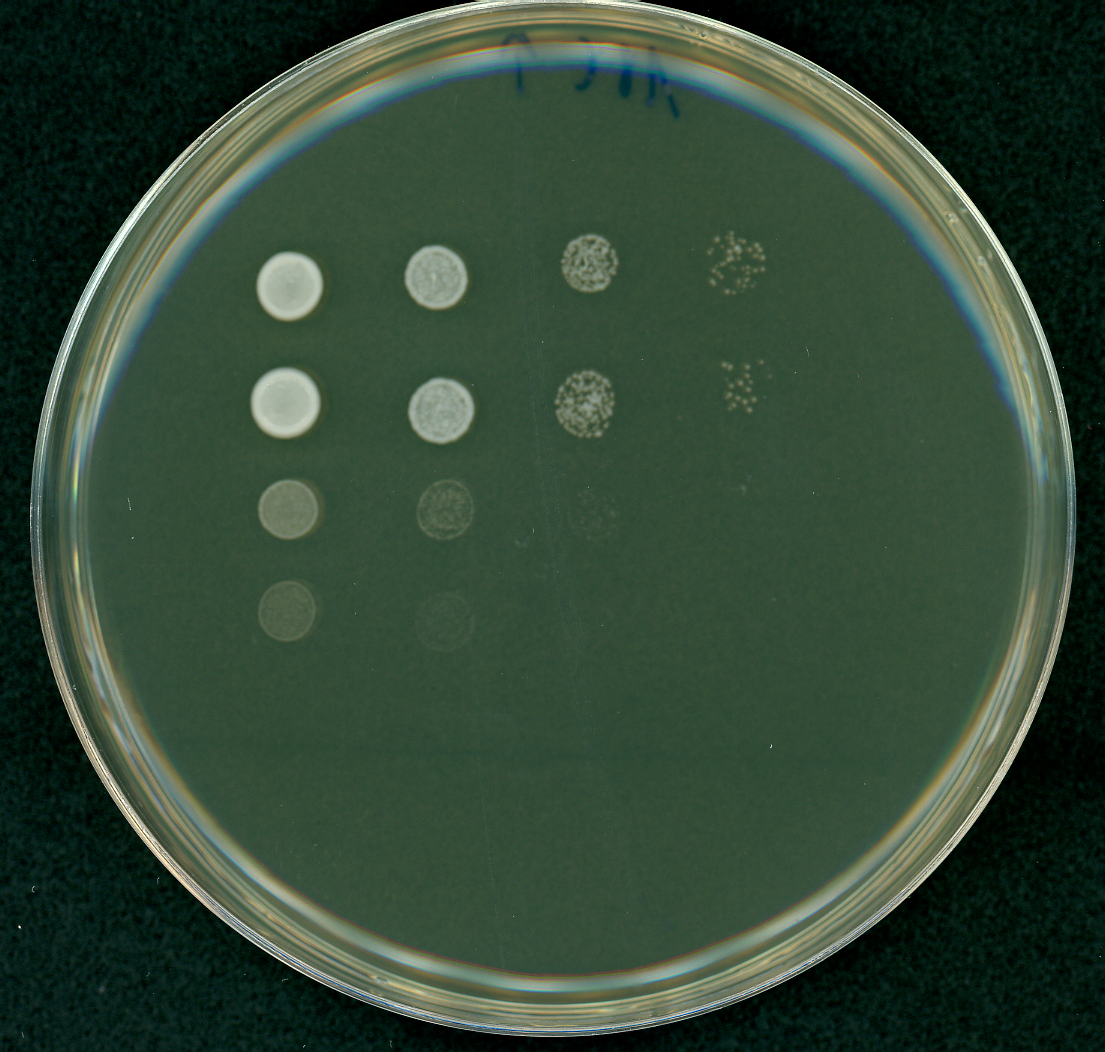

Supplement: Supplementary file 12 — Source data Fig. 6 [file 44318_2024_258_MOESM12_ESM.zip › Figure 6/FIGURE 6I/02072022_spottest_wtpan2ccr4double_18C_010.tif]

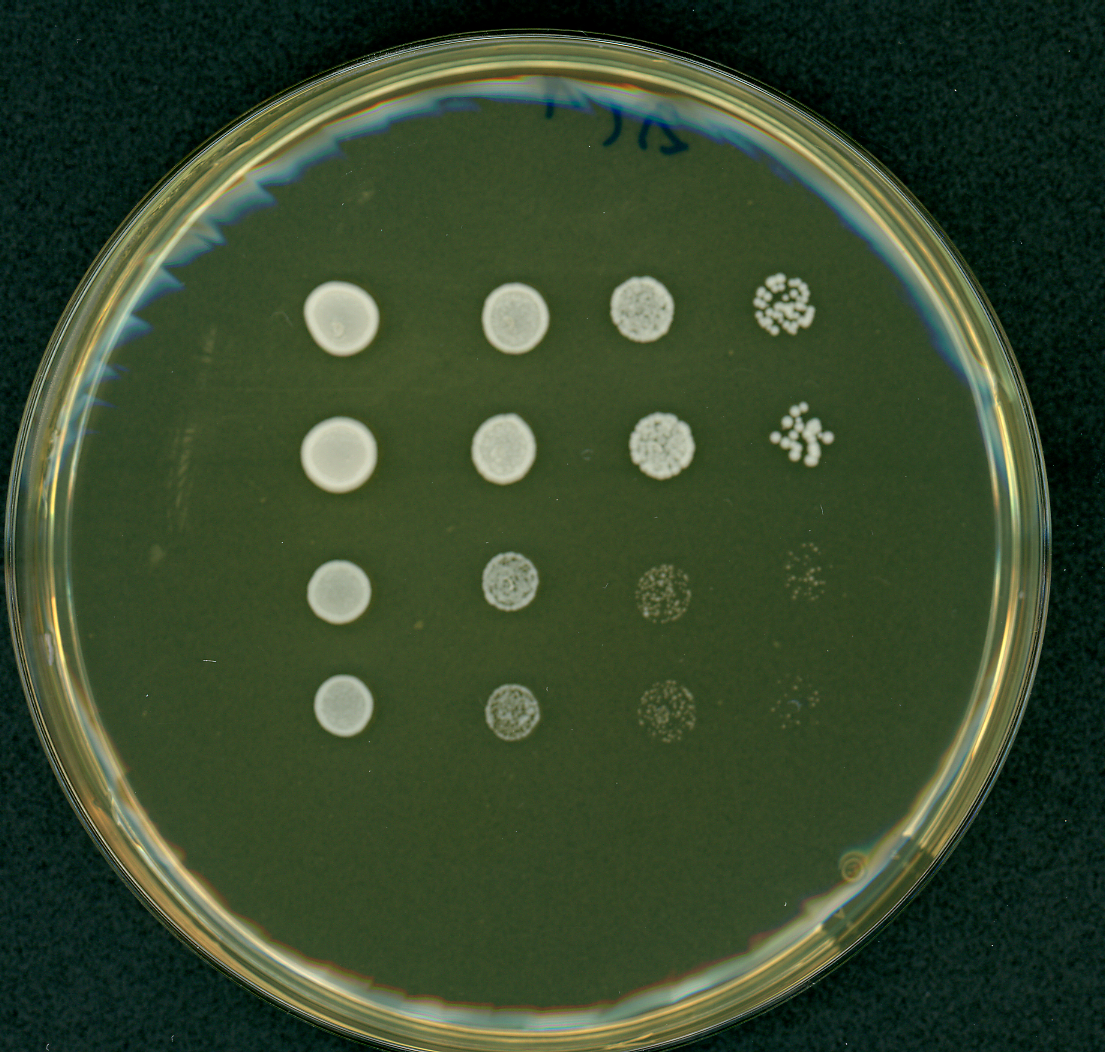

Supplement: Supplementary file 12 — Source data Fig. 6 [file 44318_2024_258_MOESM12_ESM.zip › Figure 6/FIGURE 6I/02072022_spottest_wtpan2ccr4double_25C_012.tif]

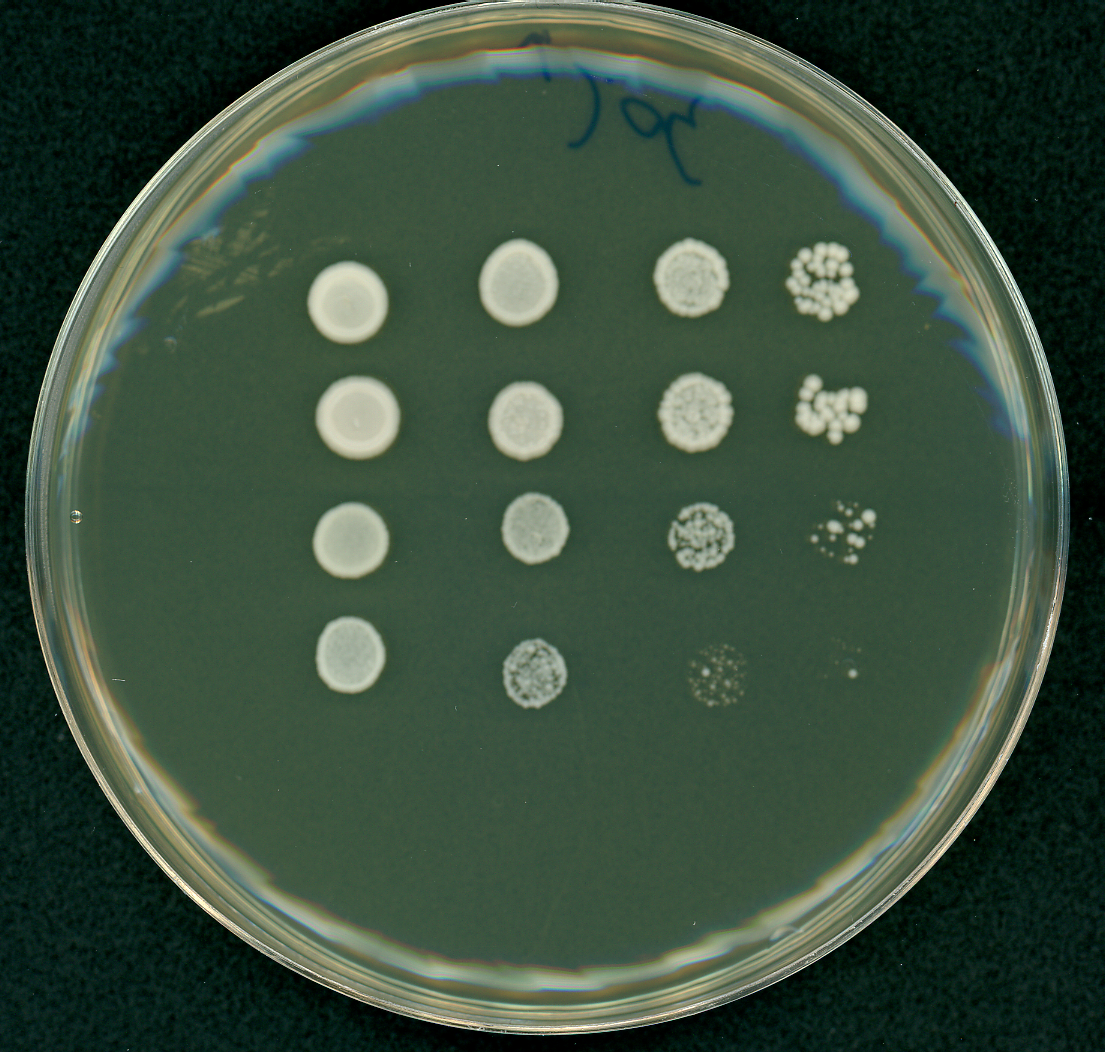

Supplement: Supplementary file 12 — Source data Fig. 6 [file 44318_2024_258_MOESM12_ESM.zip › Figure 6/FIGURE 6I/02072022_spottest_wtpan2ccr4double_30C_013.tif]

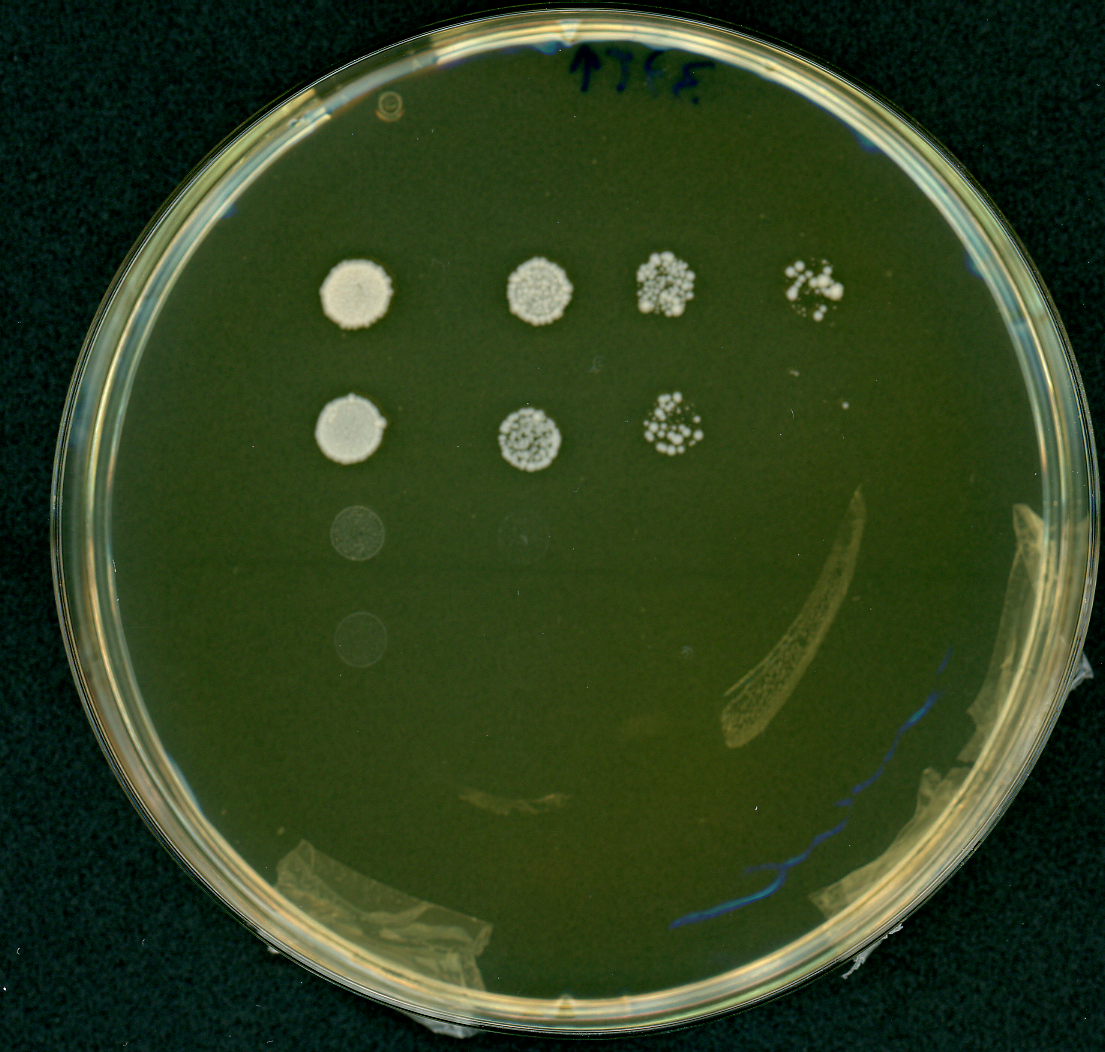

Supplement: Supplementary file 12 — Source data Fig. 6 [file 44318_2024_258_MOESM12_ESM.zip › Figure 6/FIGURE 6I/02072022_spottest_wtpan2ccr4double_37C_011.tif]
