# Supplementary material for: Modeling of mRNA deadenylation rates reveal a complex relationship between mRNA deadenylation and decay
Source: EMBO J. 2024 Oct 11;43(24):6525–54. doi: 10.1038/s44318-024-00258-3 (PMC11649921; doi:10.1038/s44318-024-00258-3)
Supplement: Supplementary file 10 — Source data Fig. 4 [file 44318_2024_258_MOESM10_ESM.zip › Figure 4/FIGURE 4C/Modified_R_code_output_file_Mex67_depletion_chase_ .pdf]

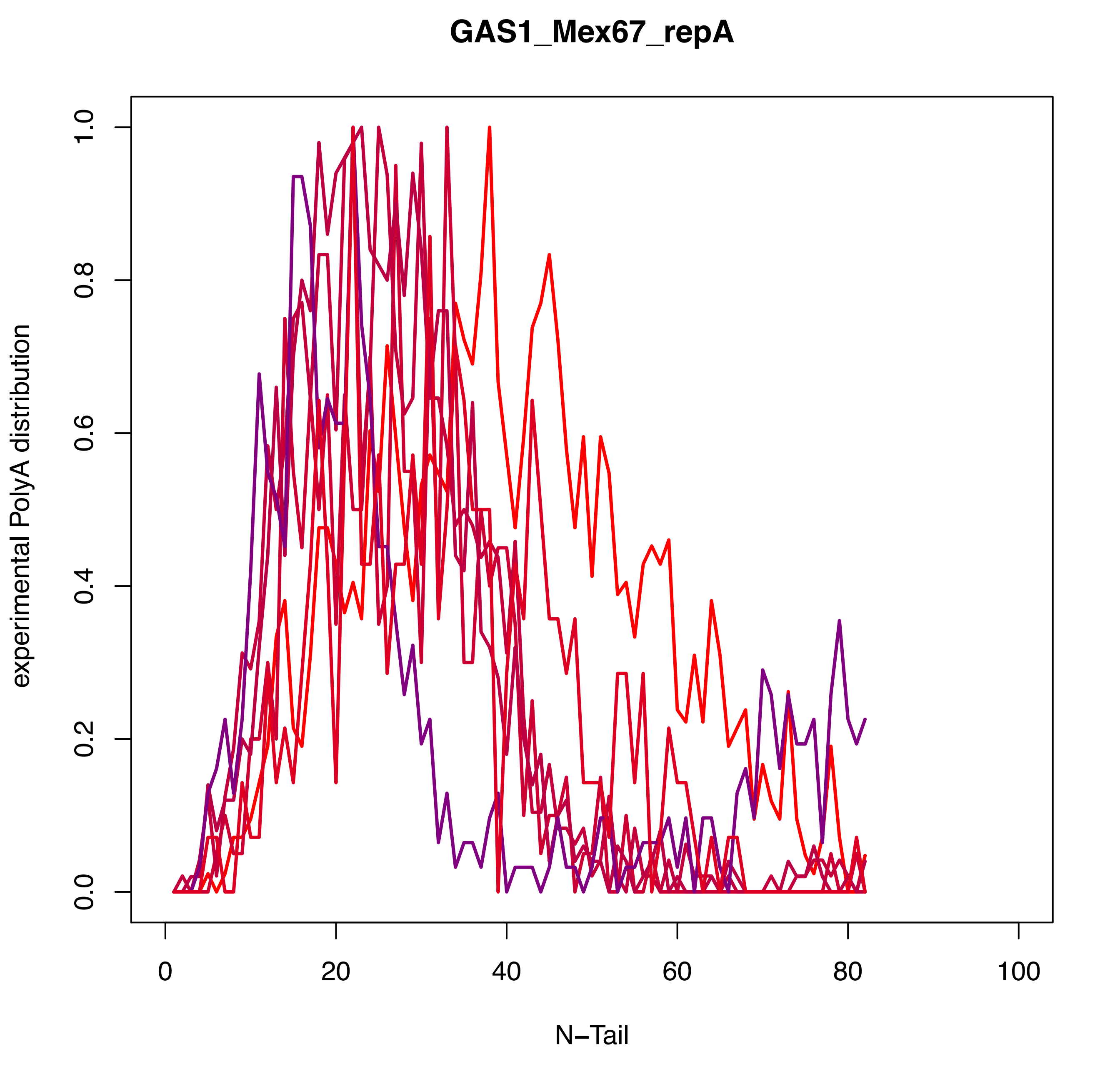
GAS1_Mex67_repA
experimental PolyA distribution
0.0
0.2
0.4
0.6
0.8
1.0
0
20
40
60
80
100
N−Tail

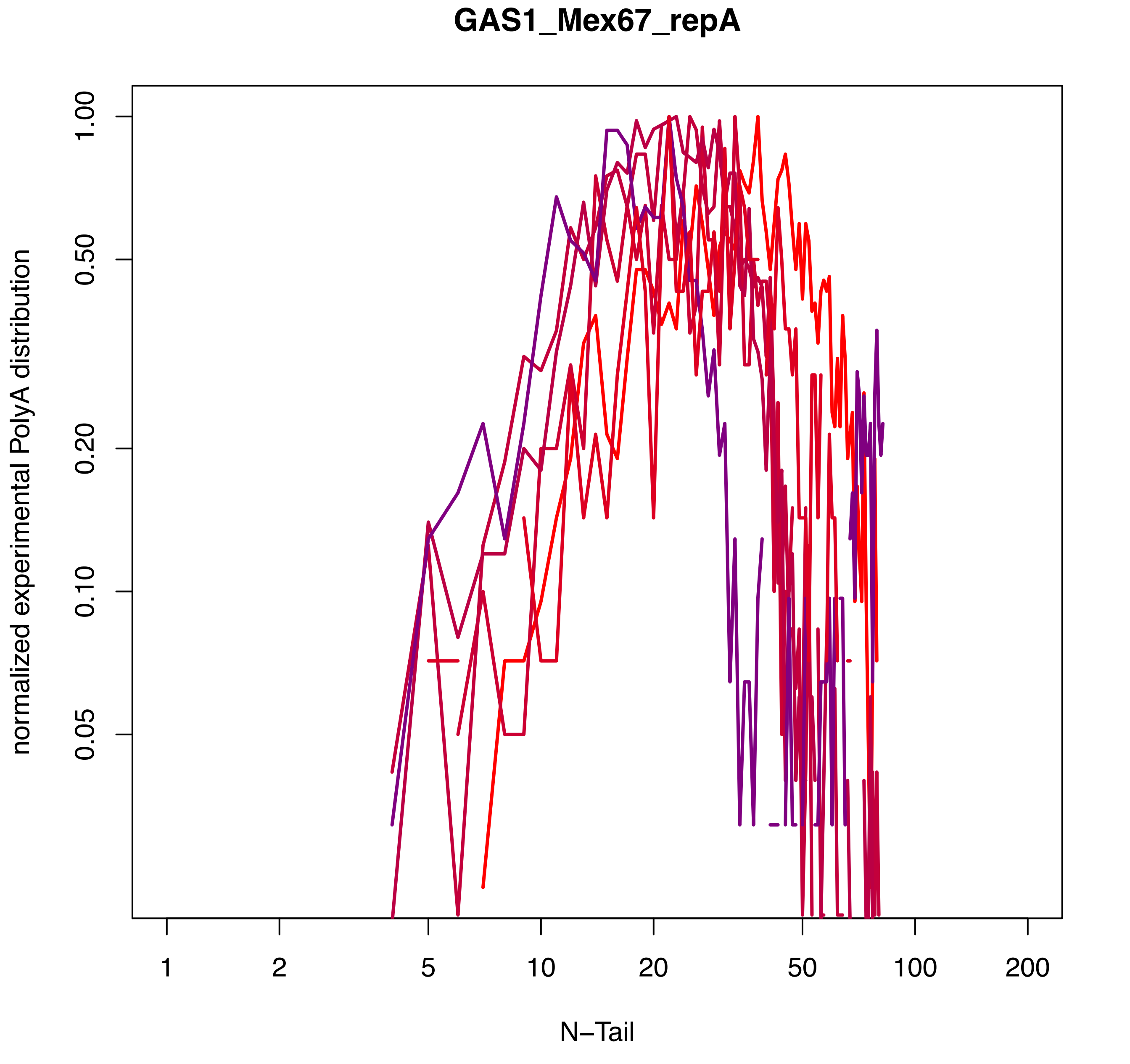

GAS1_Mex67_repA
normalized experimental PolyA distribution
N−Tail
1.00
0.50
0.20
0.10
0.05
1
2
5
10
20
50
100
200

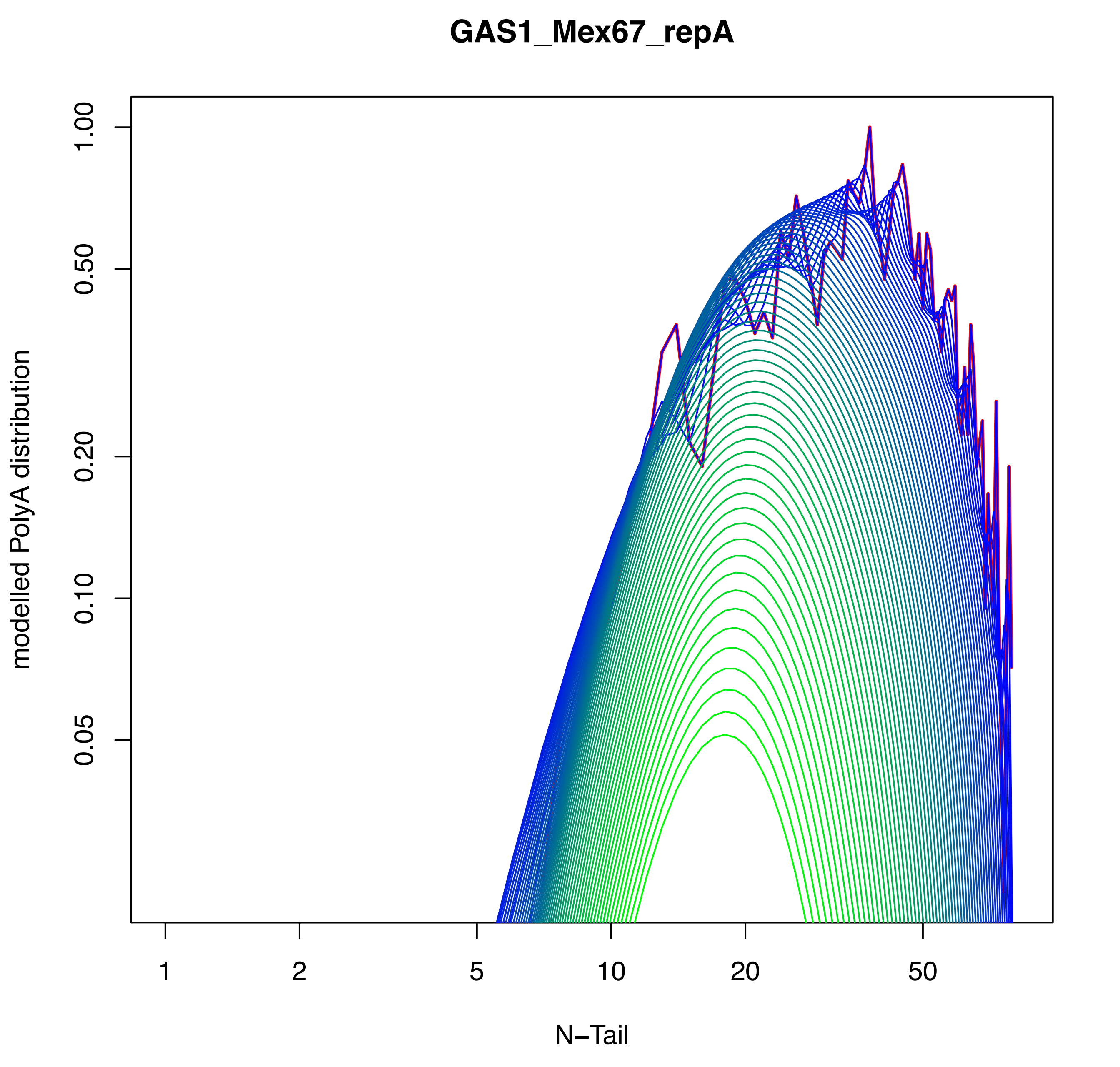
GAS1_Mex67_repA
modelled PolyA distribution
1.00
0.50
0.20
0.10
0.05
1
2
5
10
20
50
N−Tail

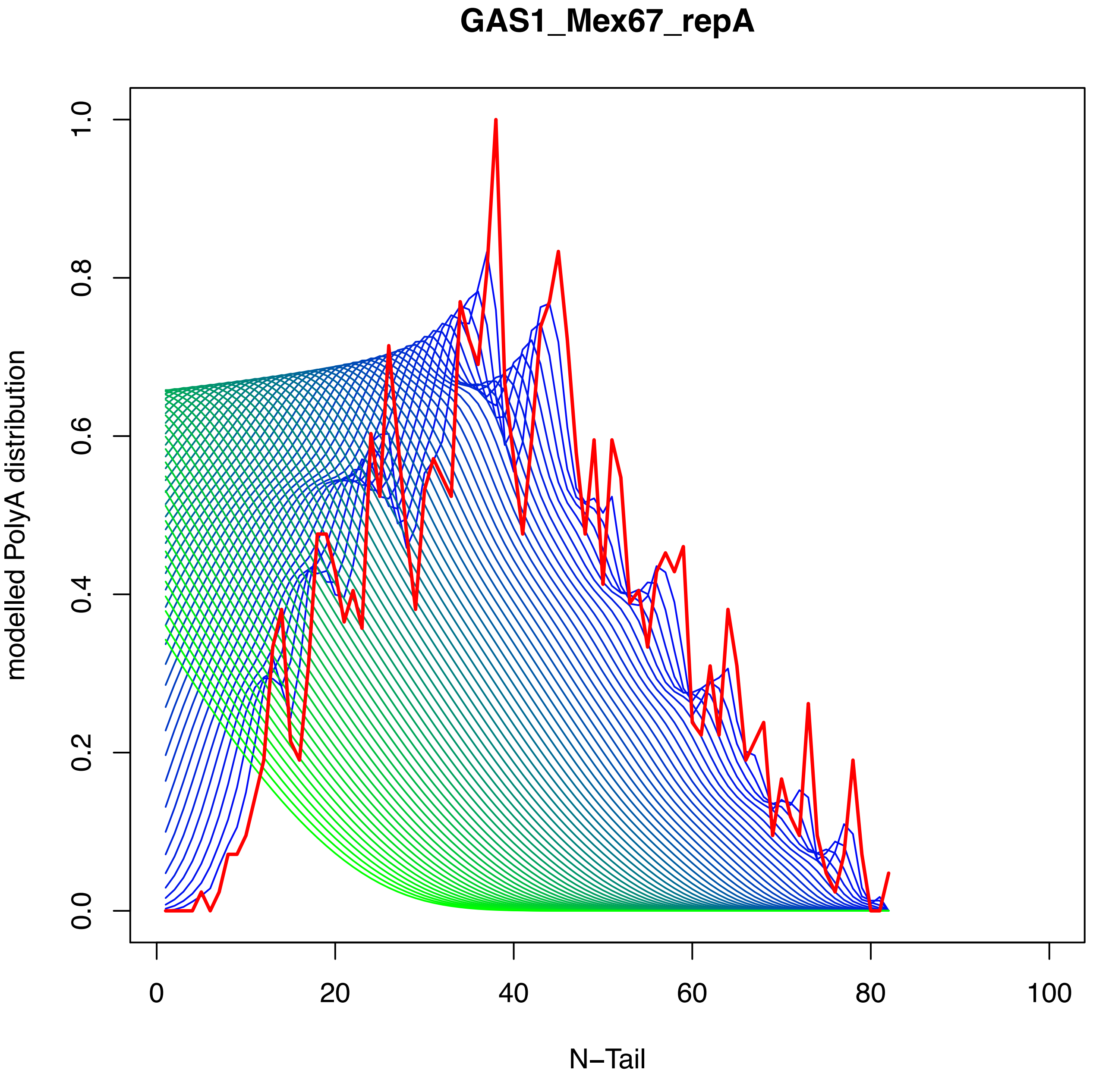

GAS1_Mex67_repA
modelled PolyA distribution
N−Tail
0.0
0.2
0.4
0.6
0.8
1.0
0
20
40
60
80
100

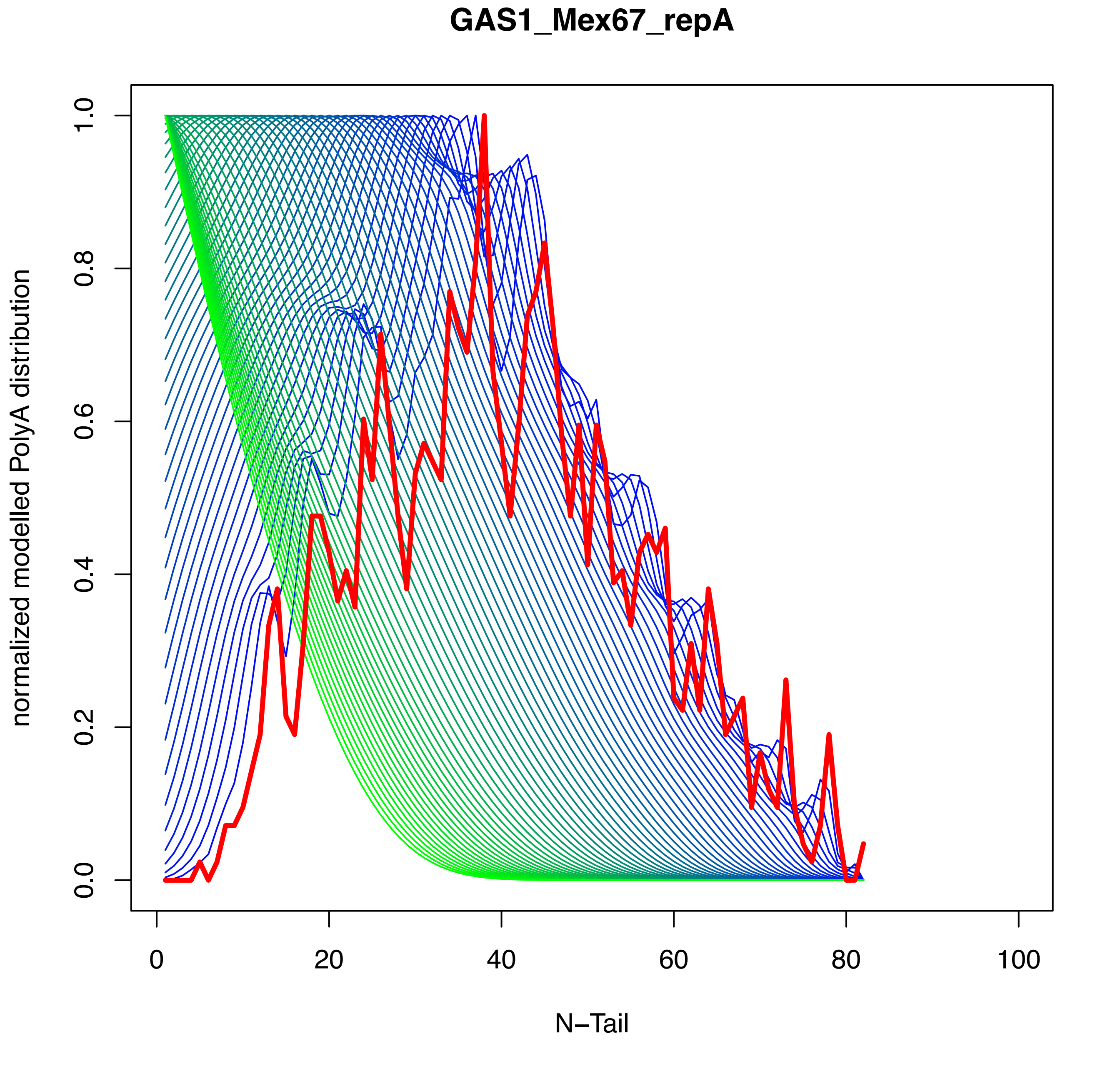
GAS1_Mex67_repA
normalized modelled PolyA distribution
0.0
0.2
0.4
0.6
0.8
1.0
0
20
40
60
80
100
N−Tail

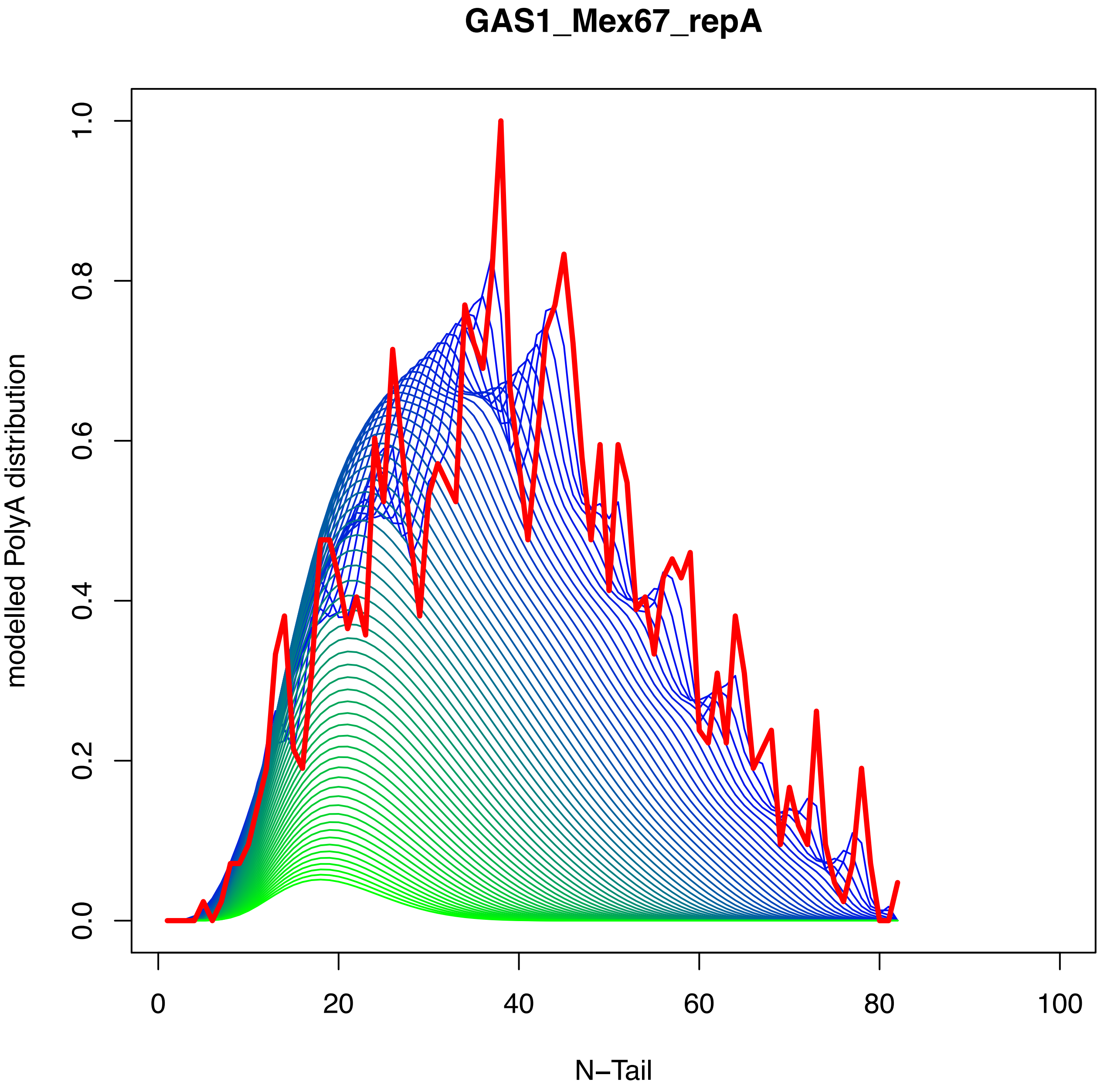

GAS1_Mex67_repA
modelled PolyA distribution
N−Tail
0.0
0.2
0.4
0.6
0.8
1.0
0
20
40
60
80
100

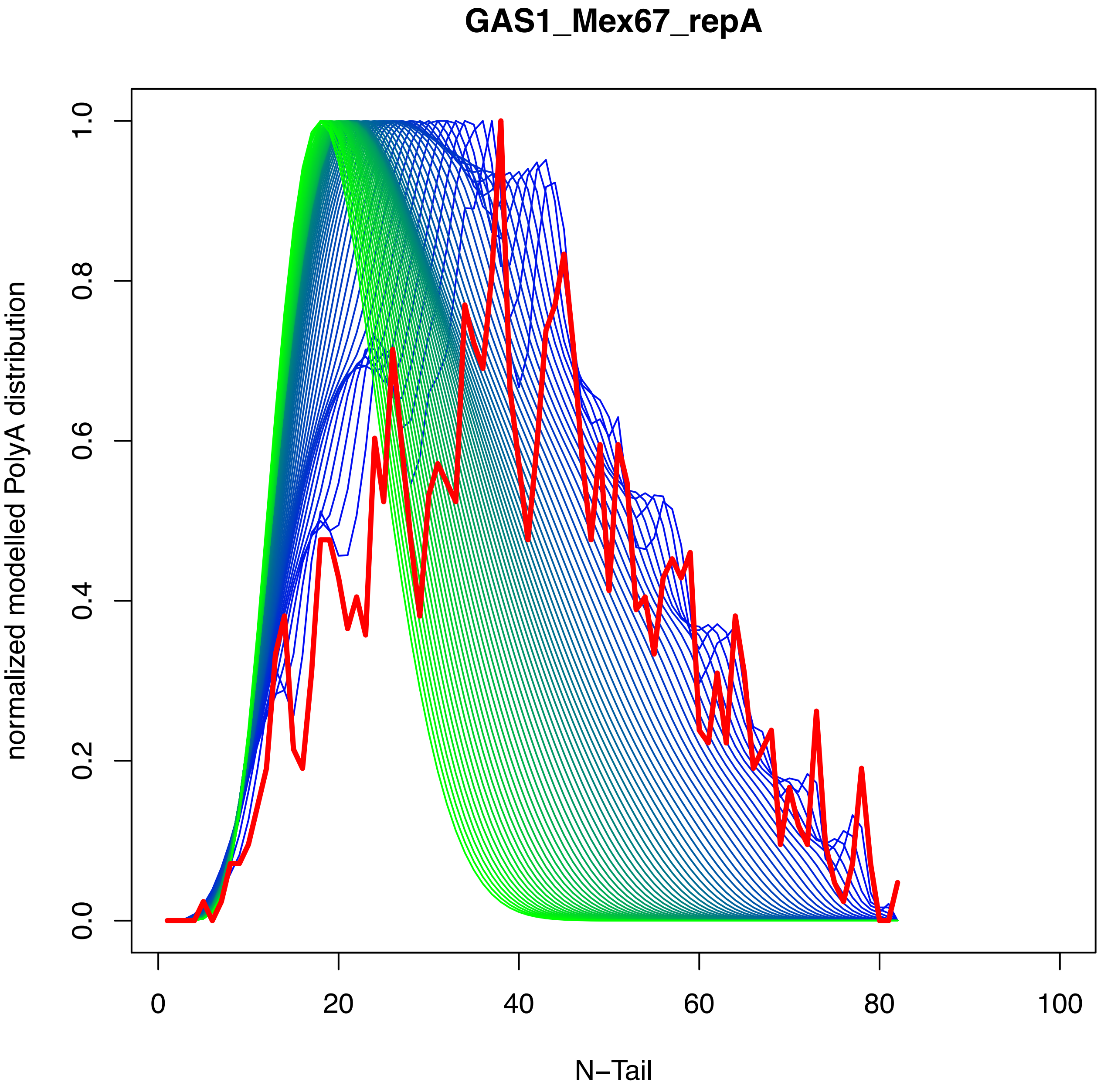
GAS1_Mex67_repA
normalized modelled PolyA distribution
N−Tail
0.0
0.2
0.4
0.6
0.8
1.0
0
20
40
60
80
100

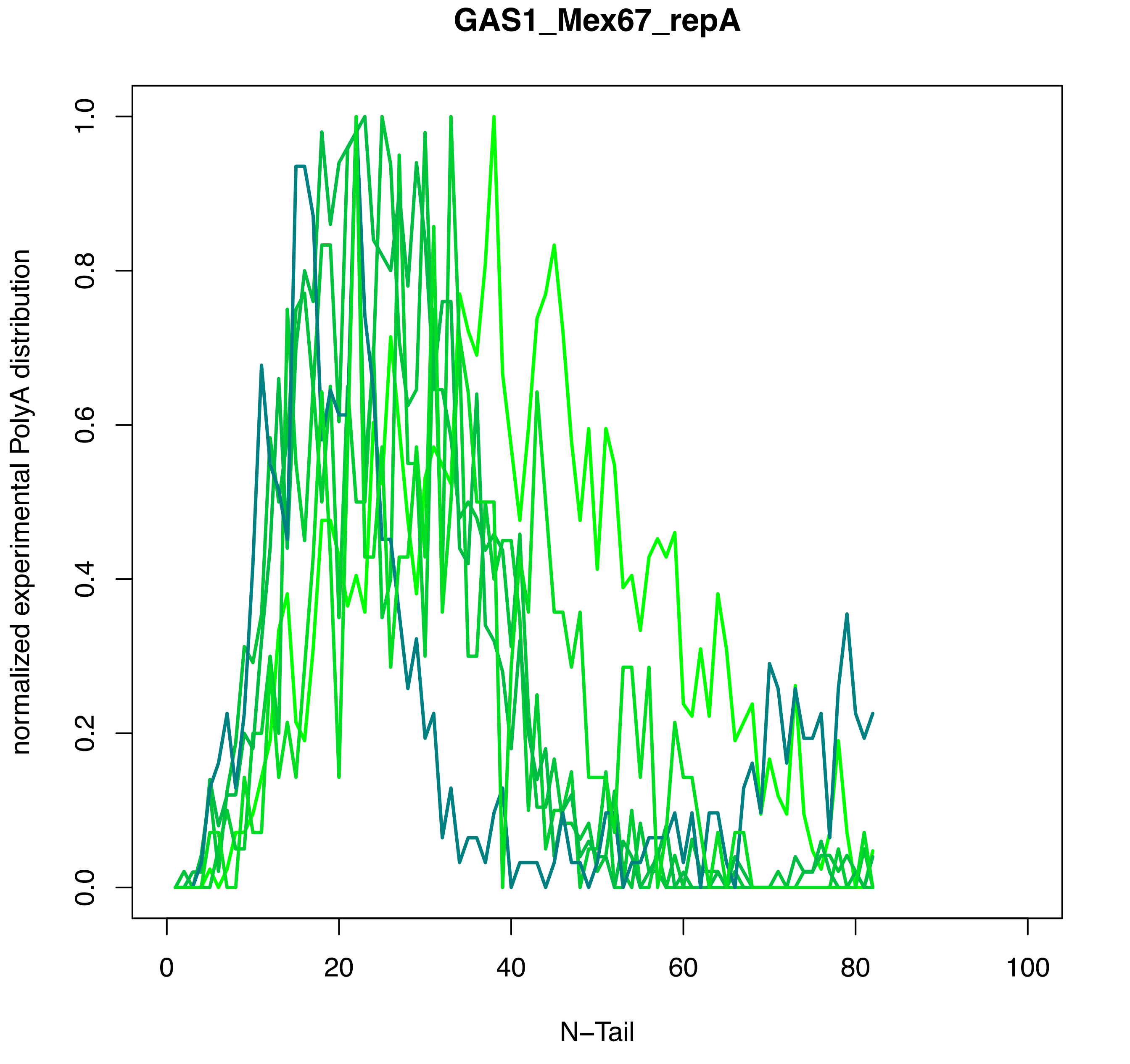
GAS1_Mex67_repA
normalized experimental PolyA distribution
N−Tail
0.0
0.2
0.4
0.6
0.8
1.0
0
20
40
60
80
100

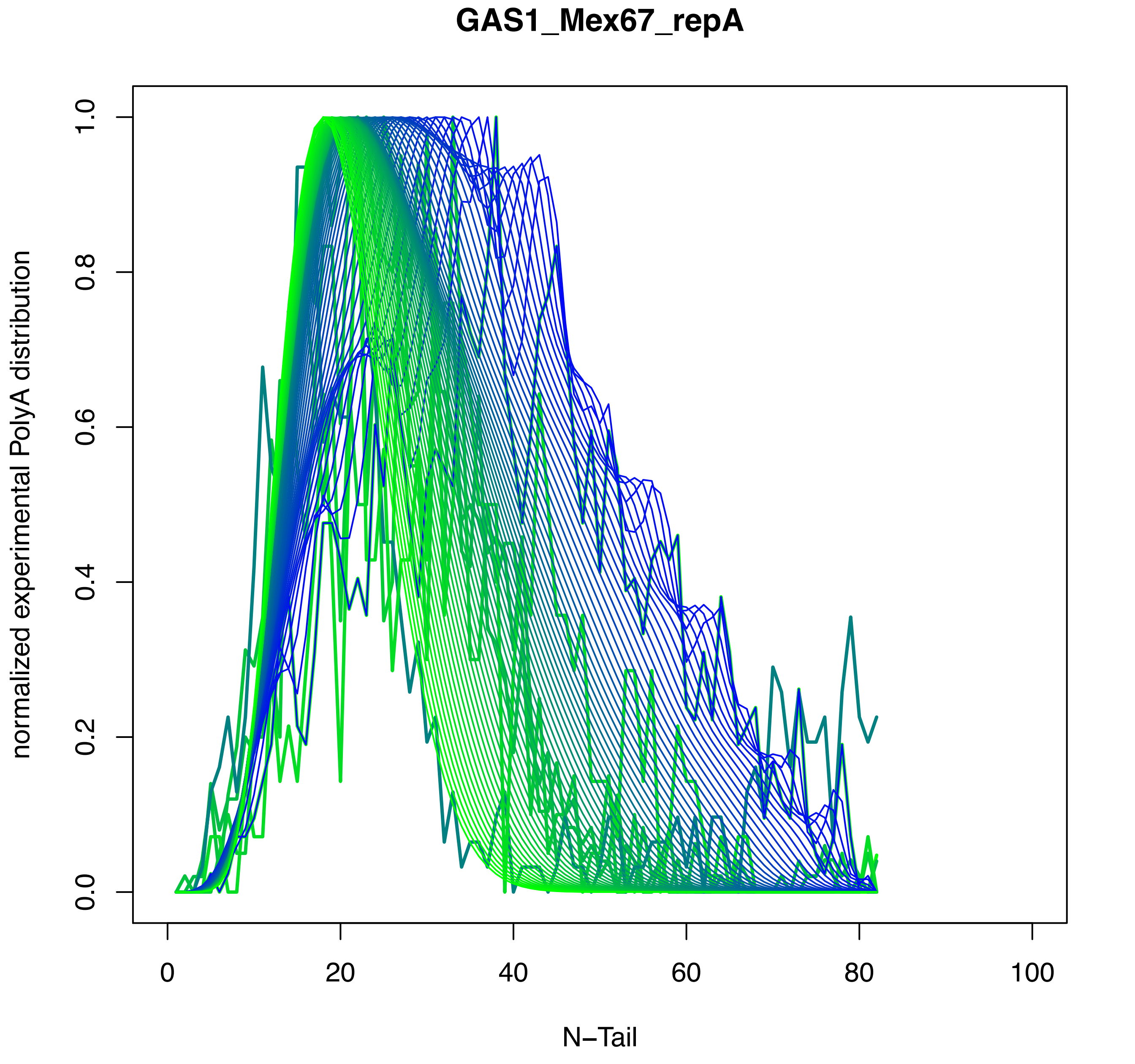

GAS1_Mex67_repA
normalized experimental PolyA distribution
N−Tail
0.0
0.2
0.4
0.6
0.8
1.0
0
20
40
60
80
100

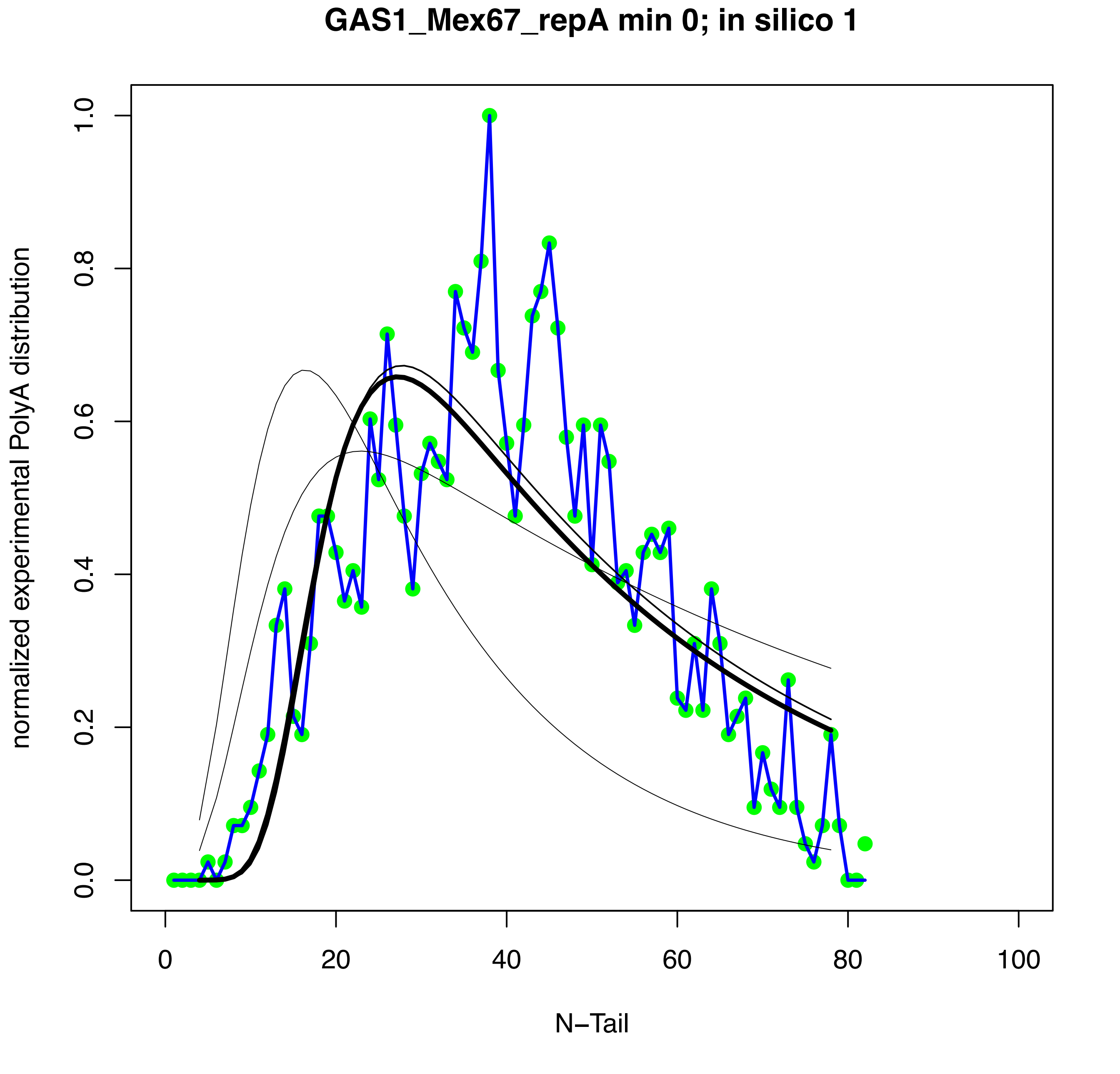
GAS1_Mex67_repA min 0; in silico 1
normalized experimental PolyA distribution
N−Tail
0.0
0.2
0.4
0.6
0.8
1.0
0
20
40
60
80
100

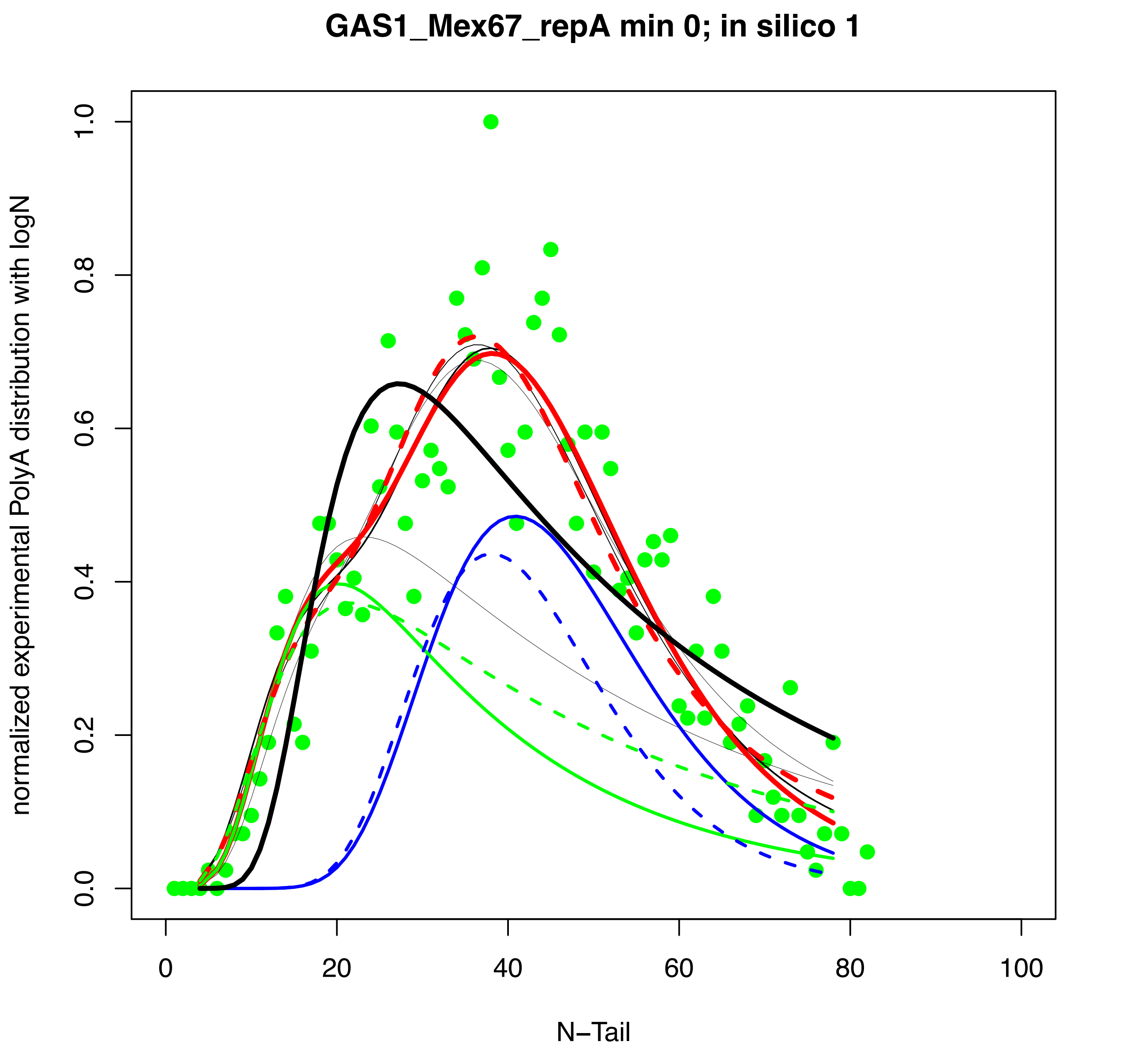
GAS1_Mex67_repA min 0; in silico 1
normalized experimental PolyA distribution with logN
N−Tail
0.0
0.2
0.4
0.6
0.8
1.0
0
20
40
60
80
100

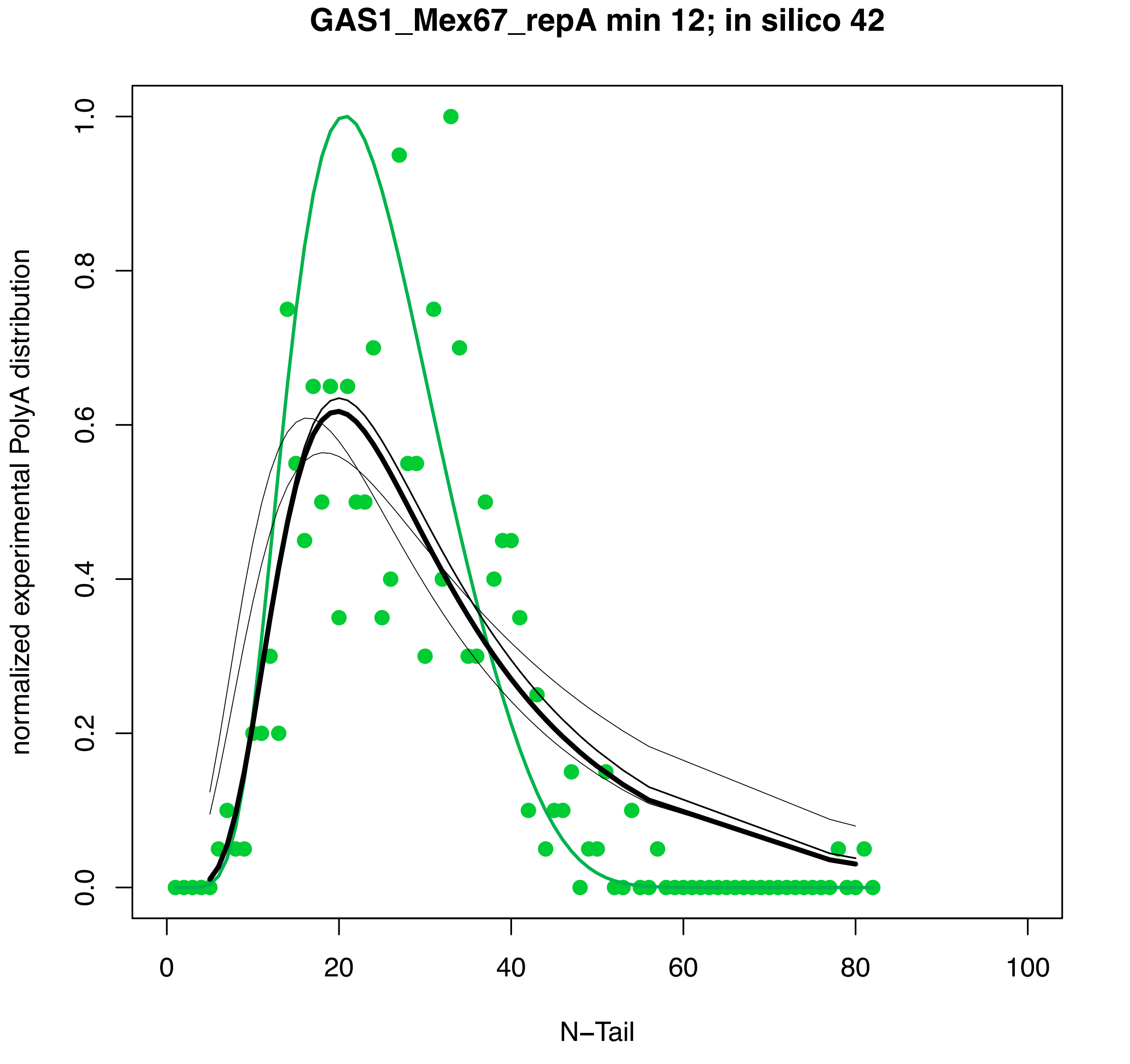

GAS1_Mex67_repA min 12; in silico 42
normalized experimental PolyA distribution
0.0
0.2
0.4
0.6
0.8
1.0
0
20
40
60
80
100
N−Tail

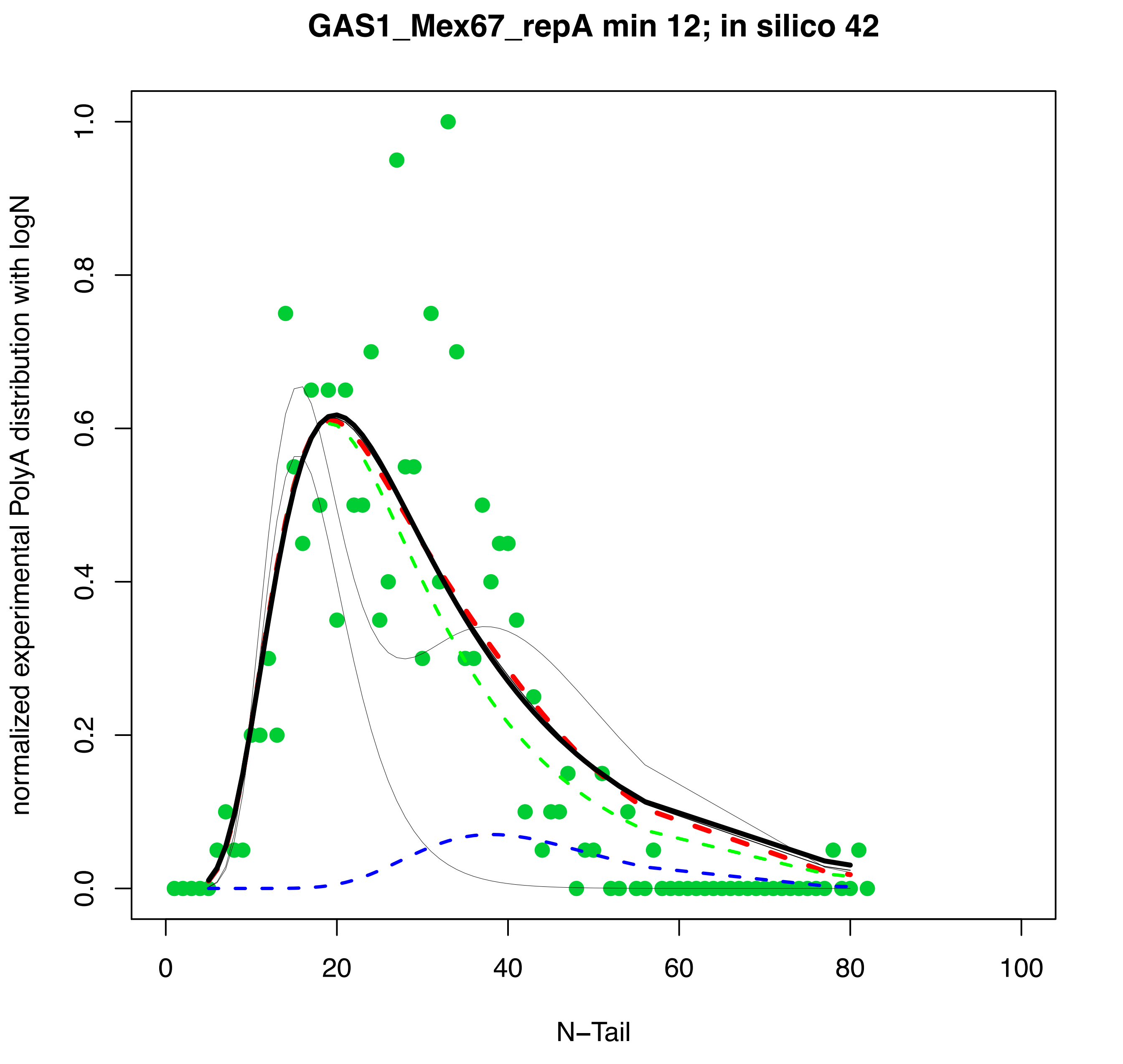

GAS1_Mex67_repA min 12; in silico 42
normalized experimental PolyA distribution with logN
N−Tail
0.0
0.2
0.4
0.6
0.8
1.0
0
20
40
60
80
100

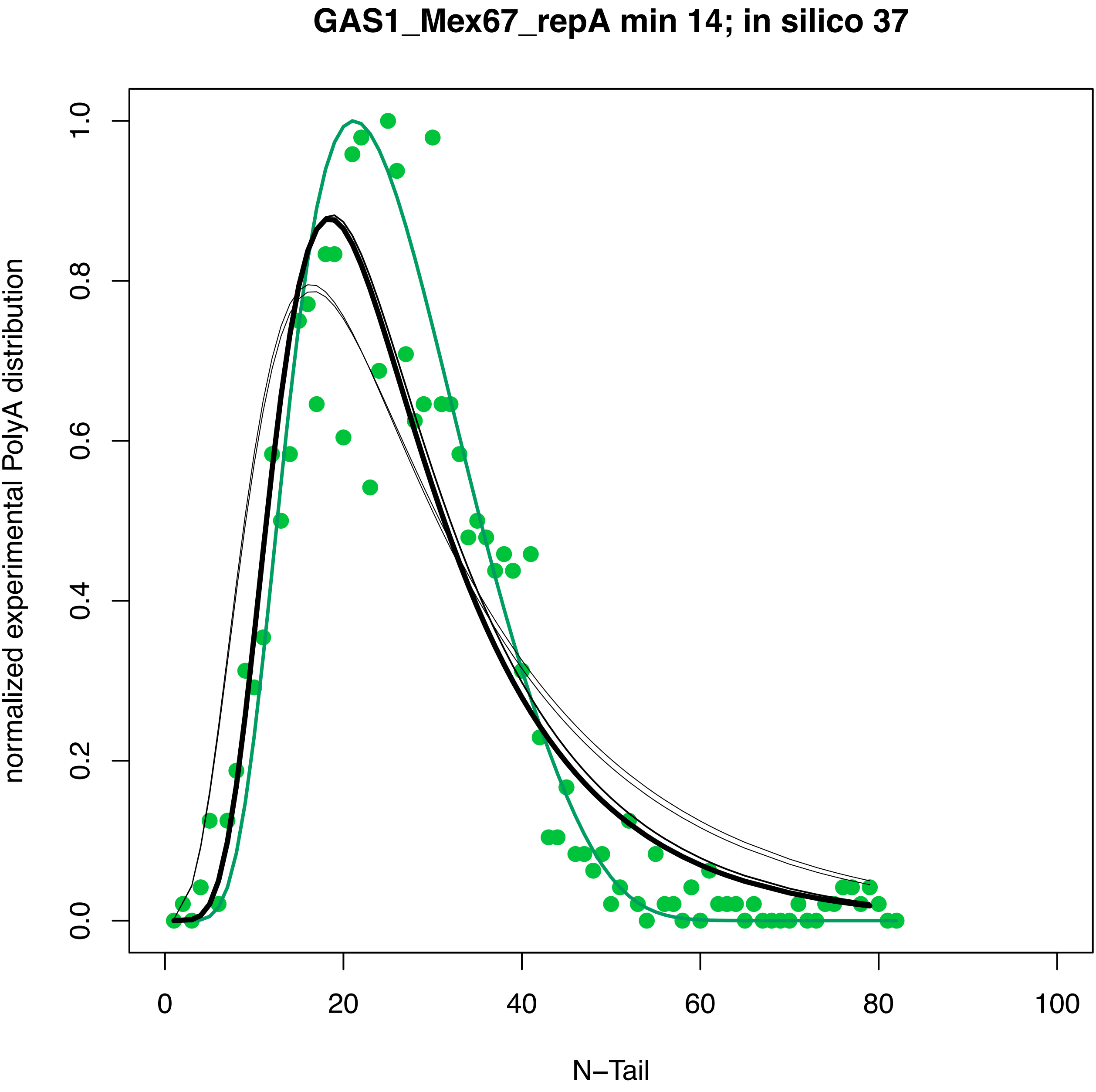

GAS1_Mex67_repA min 14; in silico 37
normalized experimental PolyA distribution
N−Tail
0.0
0.2
0.4
0.6
0.8
1.0
0
20
40
60
80
100

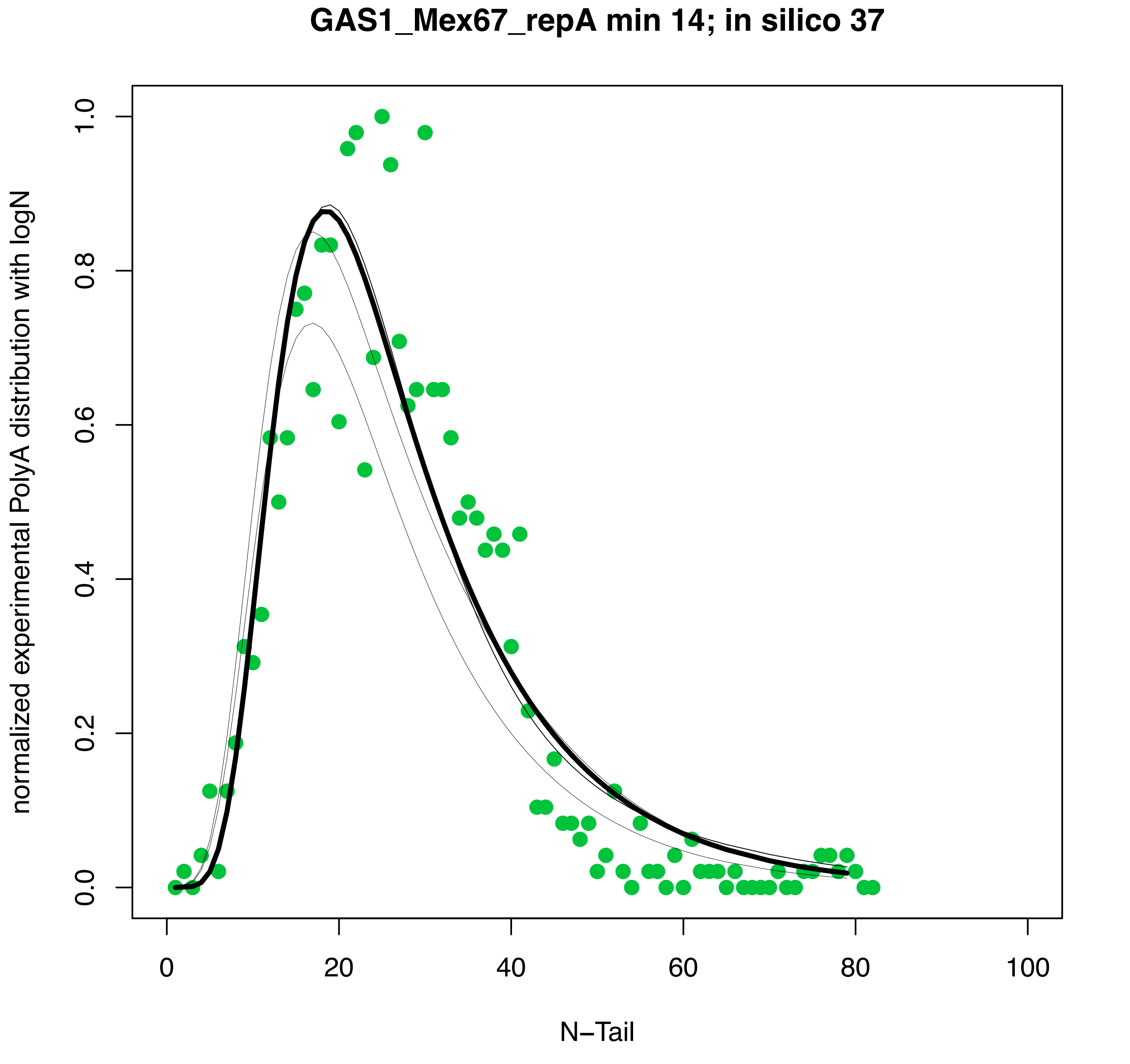

GAS1_Mex67_repA min 14; in silico 37
normalized experimental PolyA distribution with logN
N−Tail
0.0
0.2
0.4
0.6
0.8
1.0
0
20
40
60
80
100

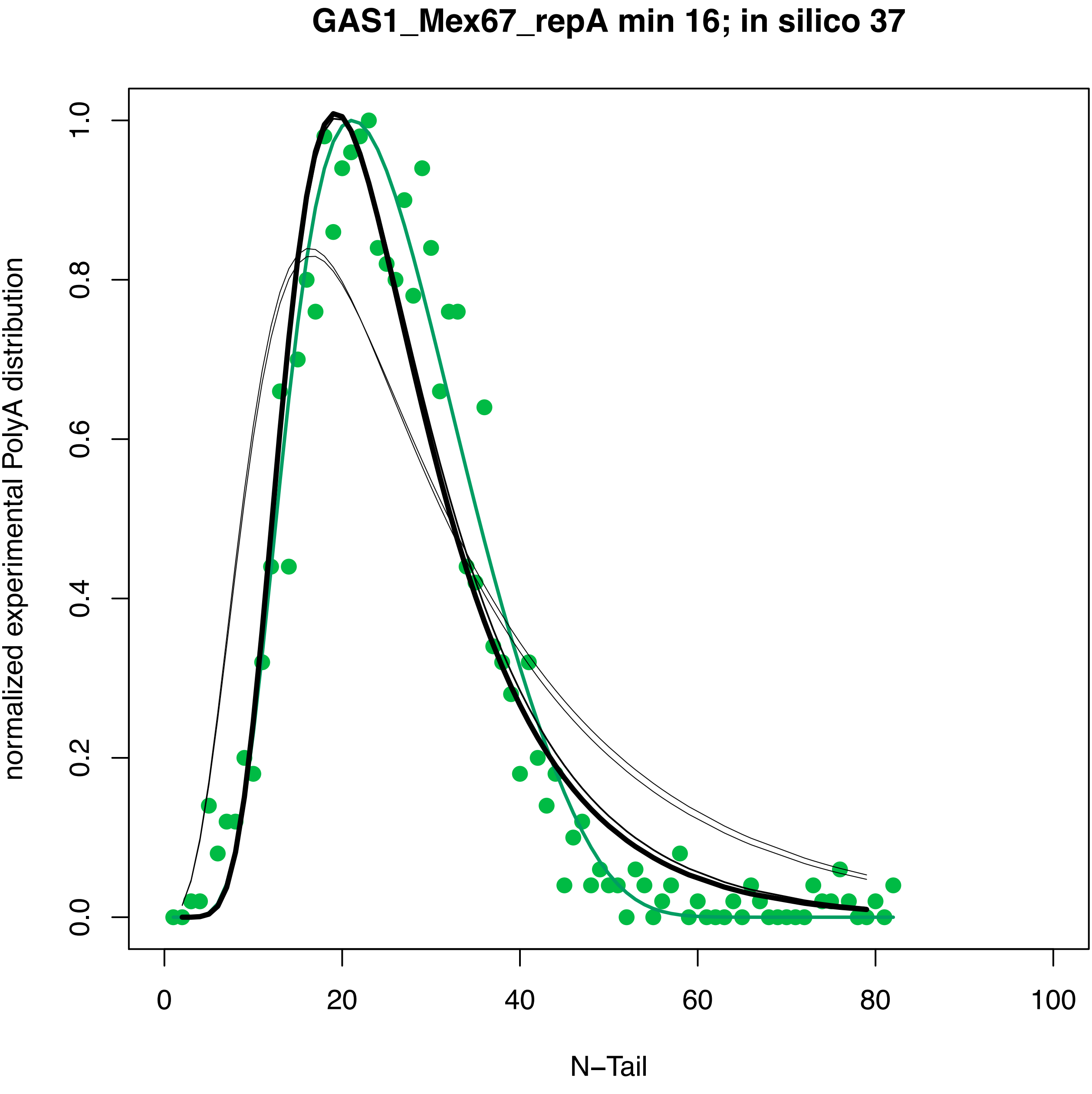

GAS1_Mex67_repA min 16; in silico 37
normalized experimental PolyA distribution
N−Tail
0.0
0.2
0.4
0.6
0.8
1.0
0
20
40
60
80
100

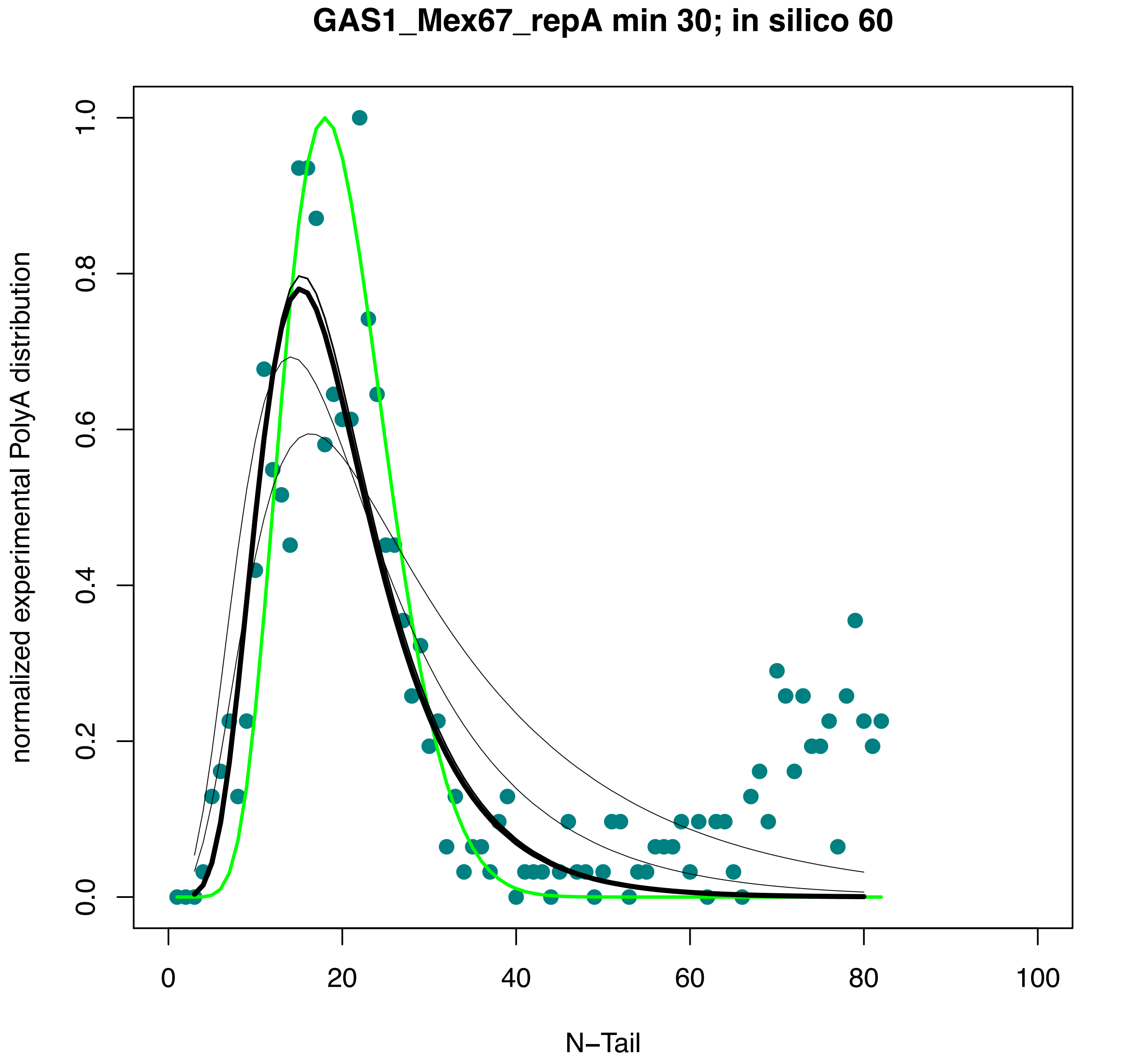

GAS1_Mex67_repA min 30; in silico 60
normalized experimental PolyA distribution
N−Tail
0.0
0.2
0.4
0.6
0.8
1.0
0
20
40
60
80
100

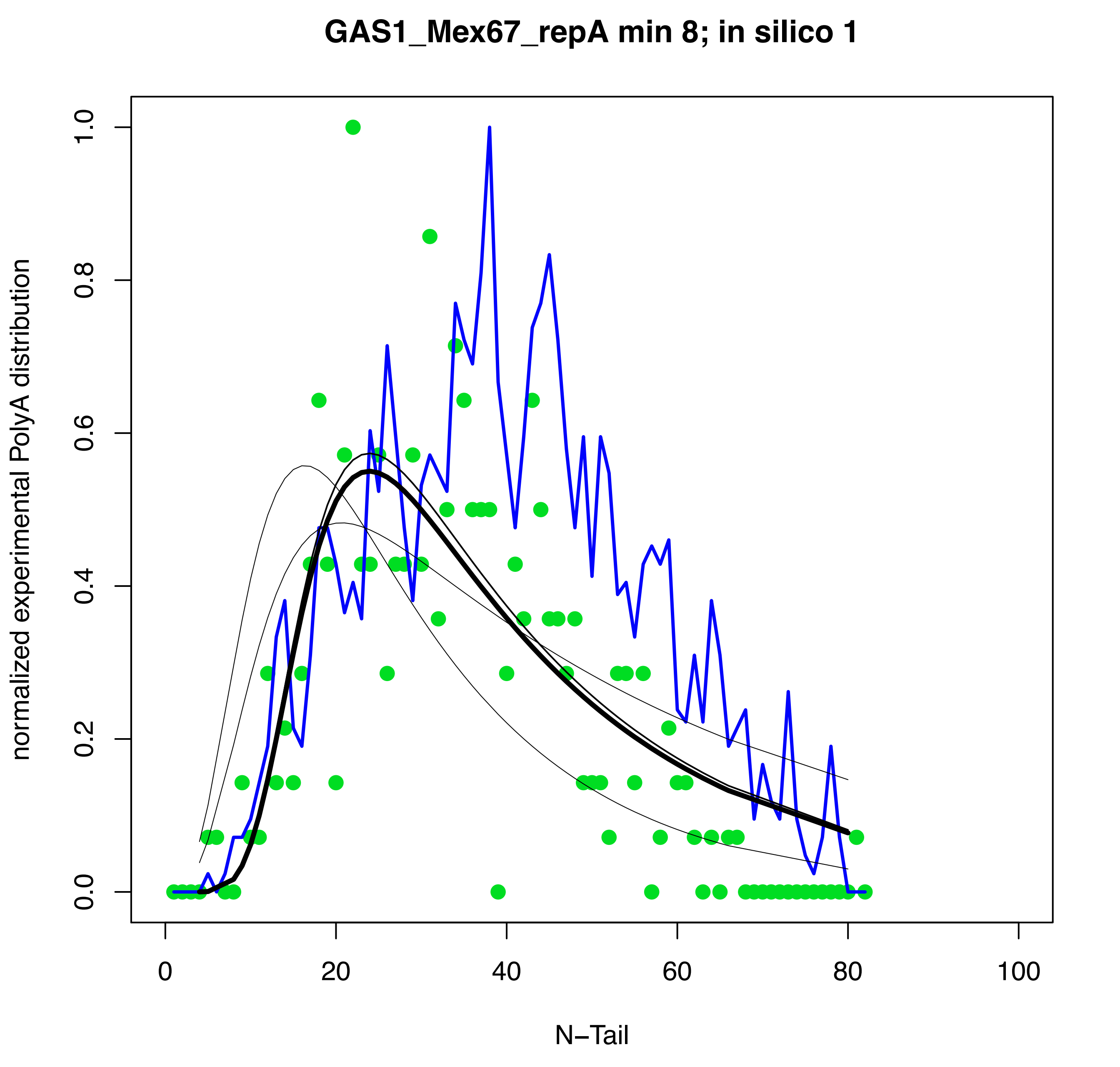

GAS1_Mex67_repA min 8; in silico 1
normalized experimental PolyA distribution
N−Tail
0.0
0.2
0.4
0.6
0.8
1.0
0
20
40
60
80
100

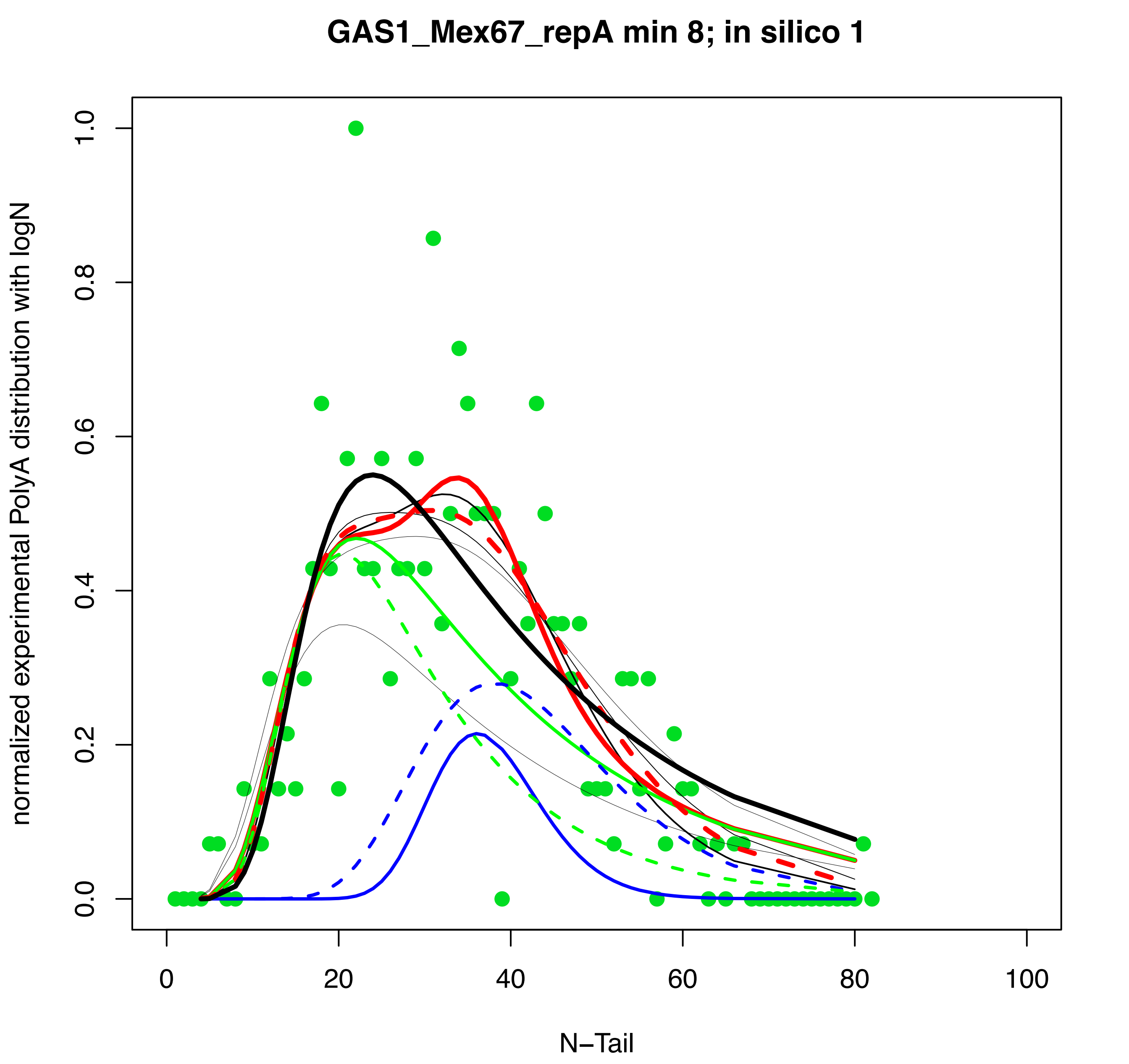

GAS1_Mex67_repA min 8; in silico 1
normalized experimental PolyA distribution with logN
N−Tail
0.0
0.2
0.4
0.6
0.8
1.0
0
20
40
60
80
100

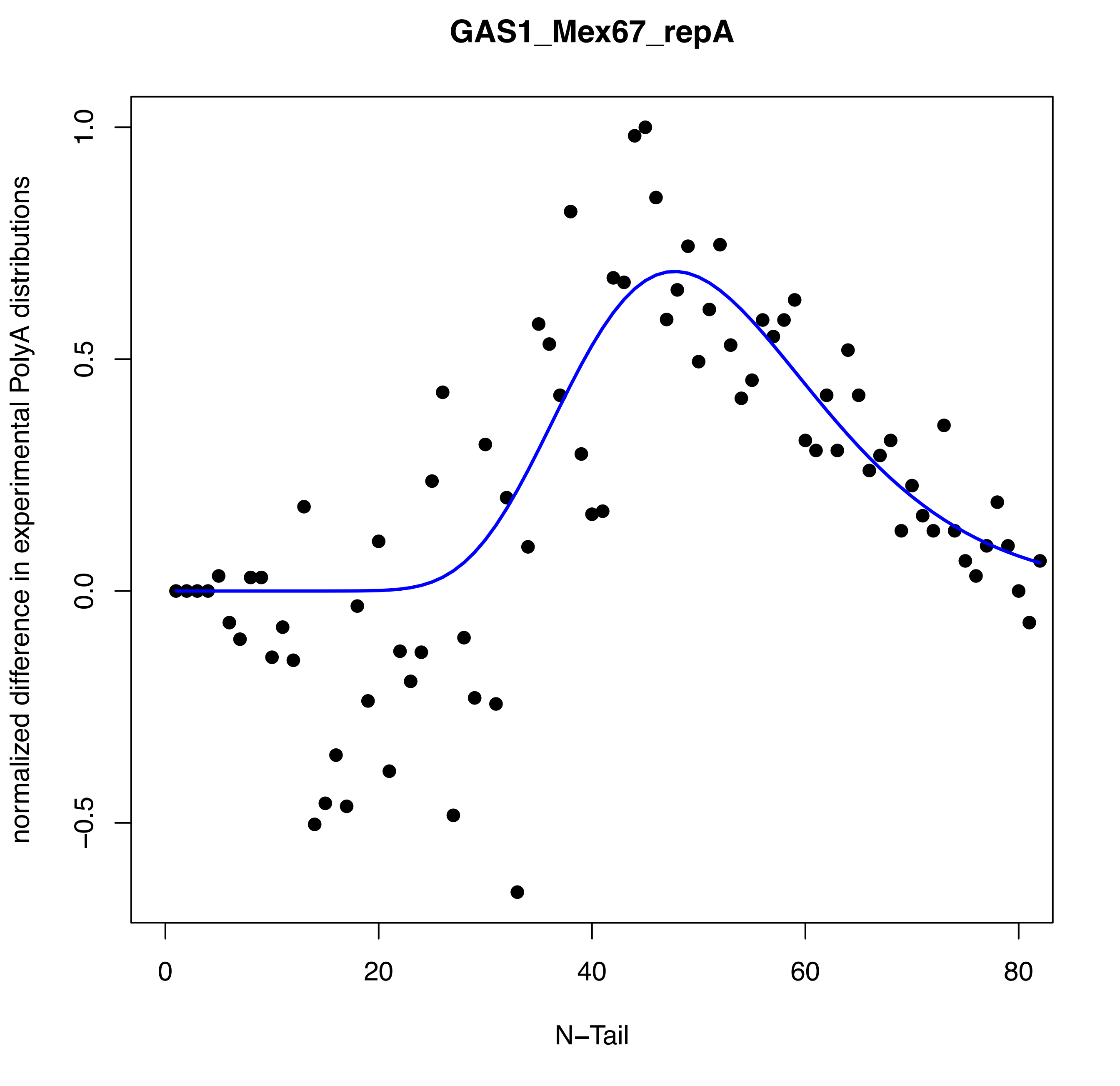

GAS1_Mex67_repA
normalized difference in experimental PolyA distributions
N−Tail
1.0
0.5
0.0
−0.5
0
20
40
60
80

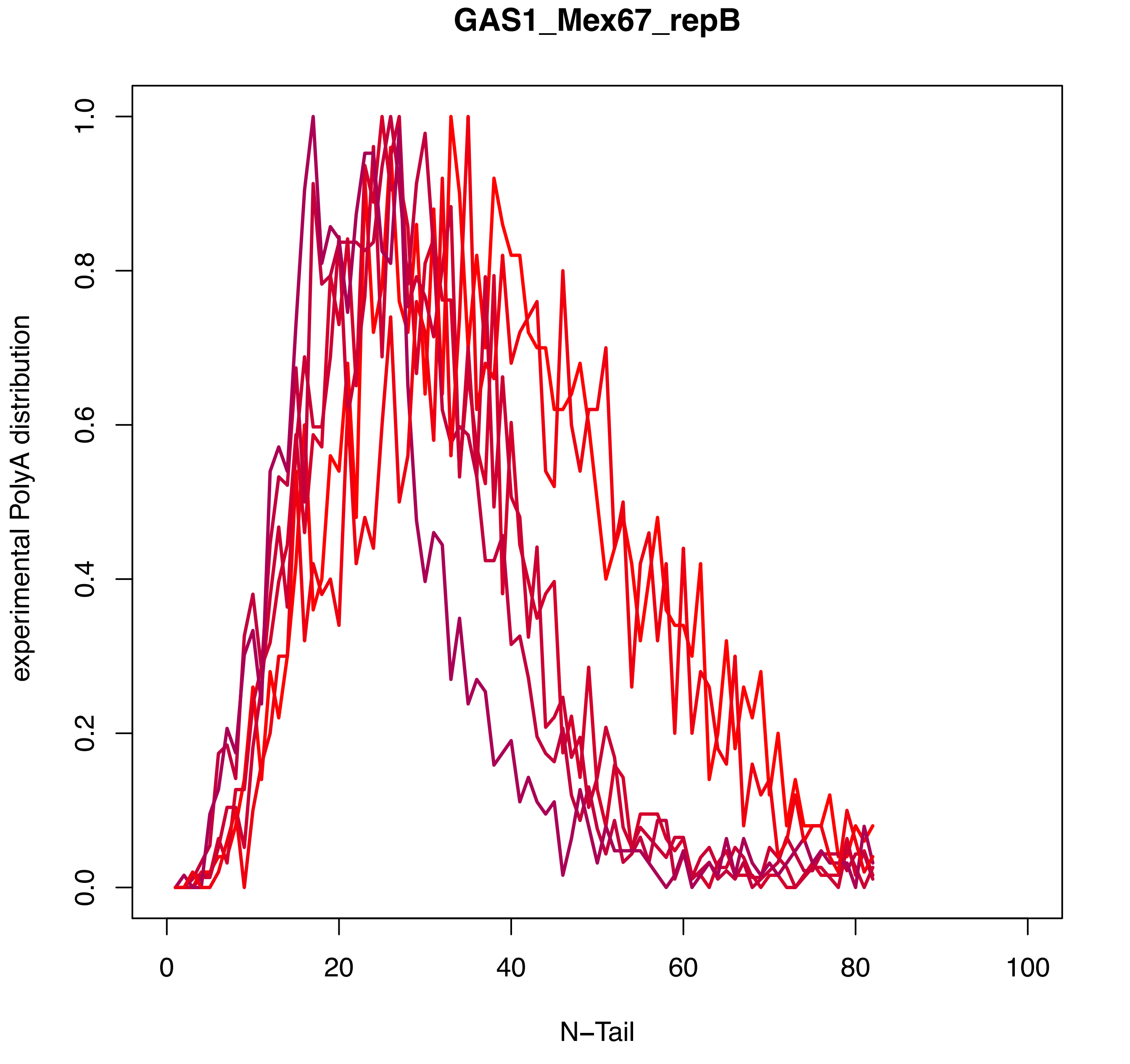

GAS1_Mex67_repB
experimental PolyA distribution
N−Tail
0.0
0.2
0.4
0.6
0.8
1.0
0
20
40
60
80
100

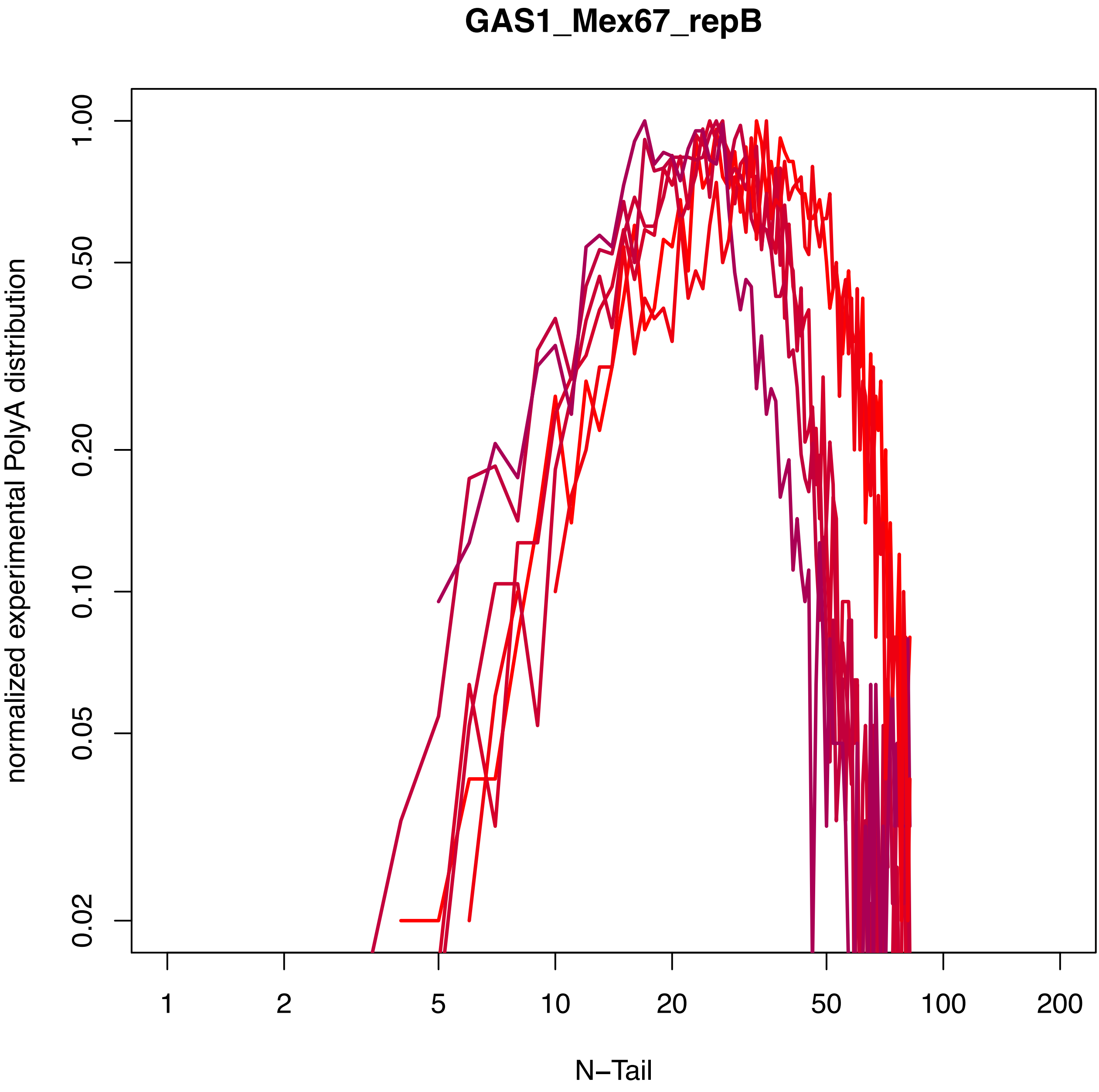
GAS1_Mex67_repB
normalized experimental PolyA distribution
N−Tail
1.00
0.50
0.20
0.10
0.05
0.02
1
2
5
10
20
50
100
200

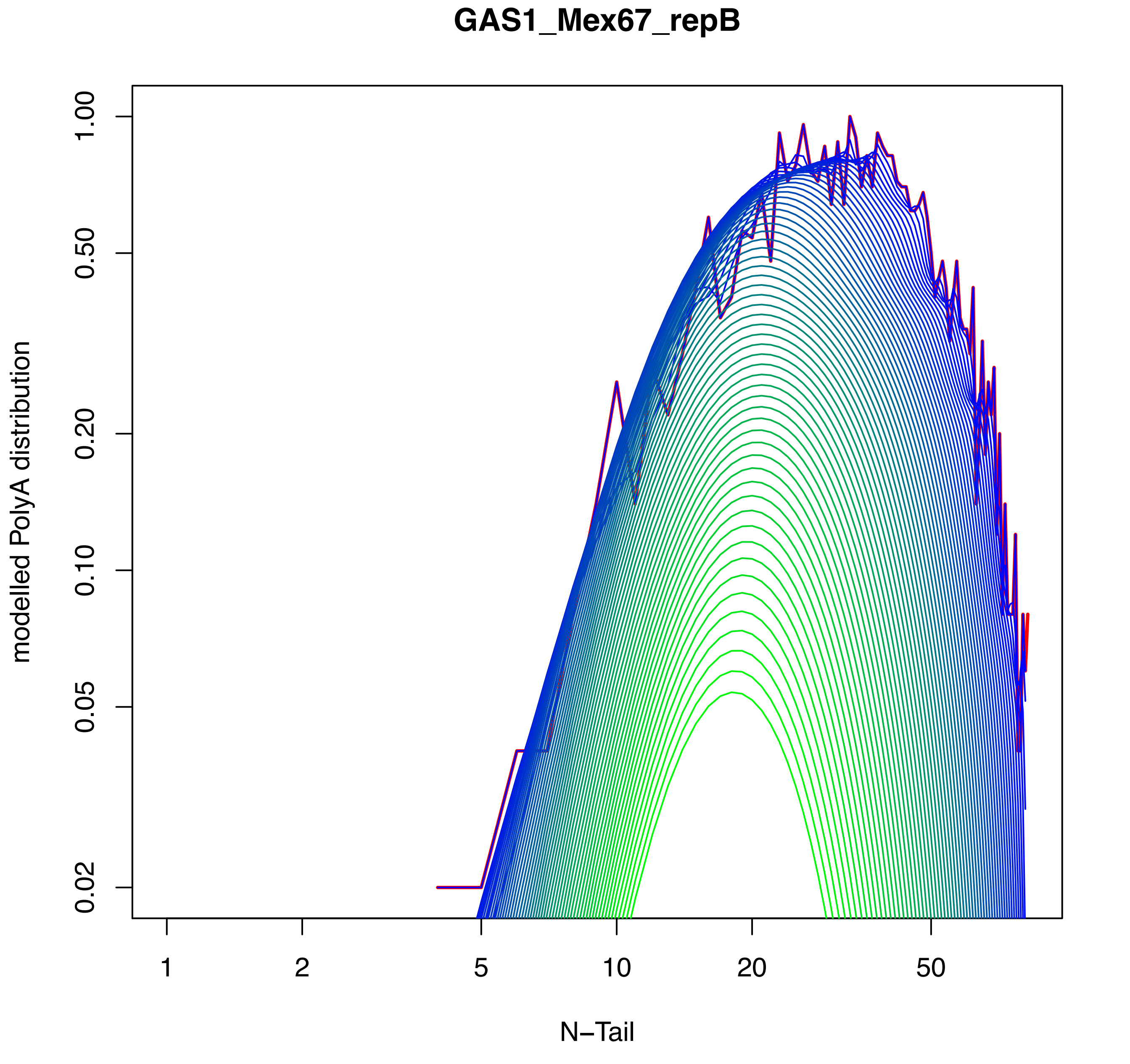

GAS1_Mex67_repB
modelled PolyA distribution
N−Tail
1.00
0.50
0.20
0.10
0.05
0.02
1
2
5
10
20
50

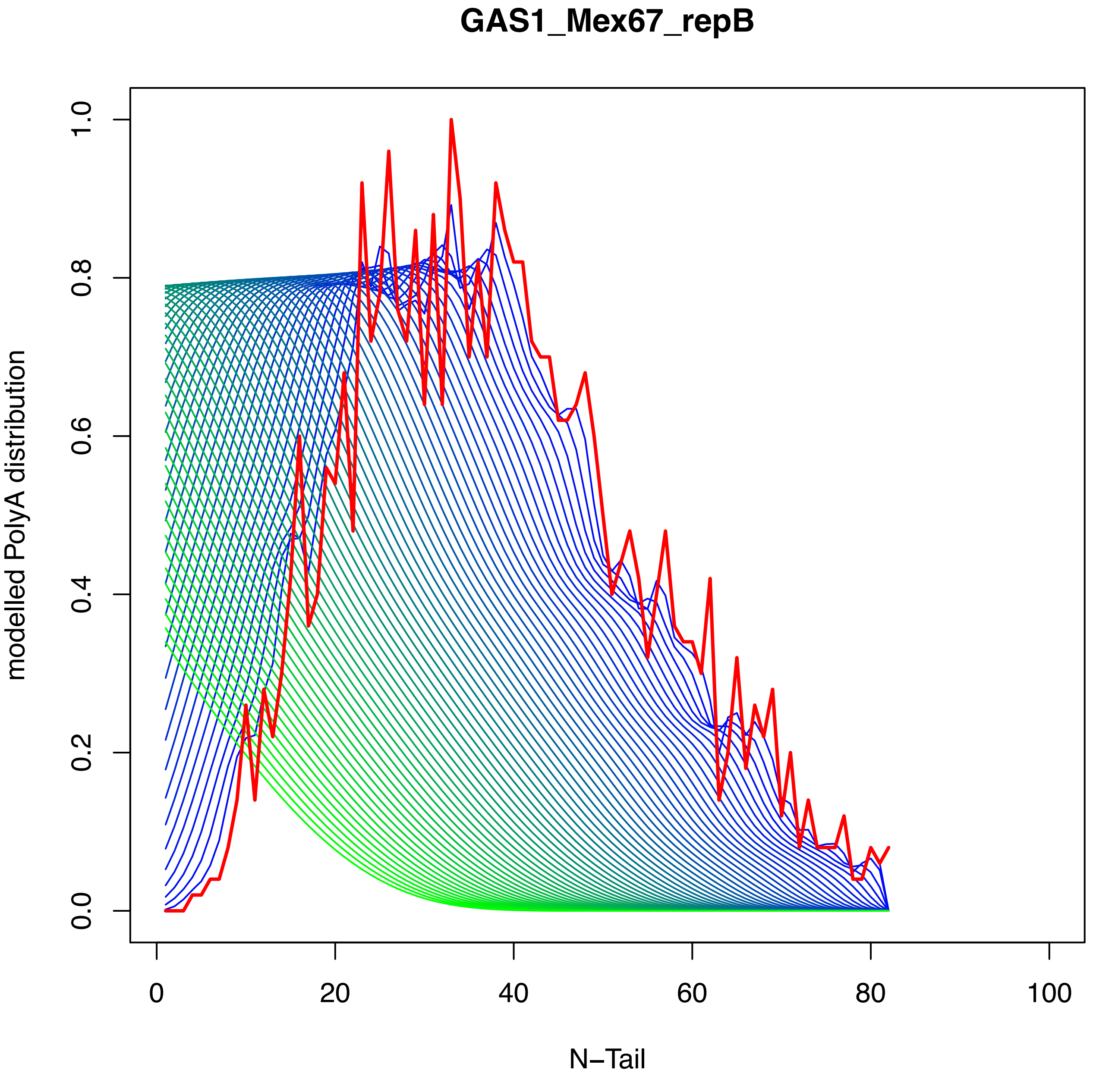
GAS1_Mex67_repB
modelled PolyA distribution
N−Tail
0.0
0.2
0.4
0.6
0.8
1.0
0
20
40
60
80
100

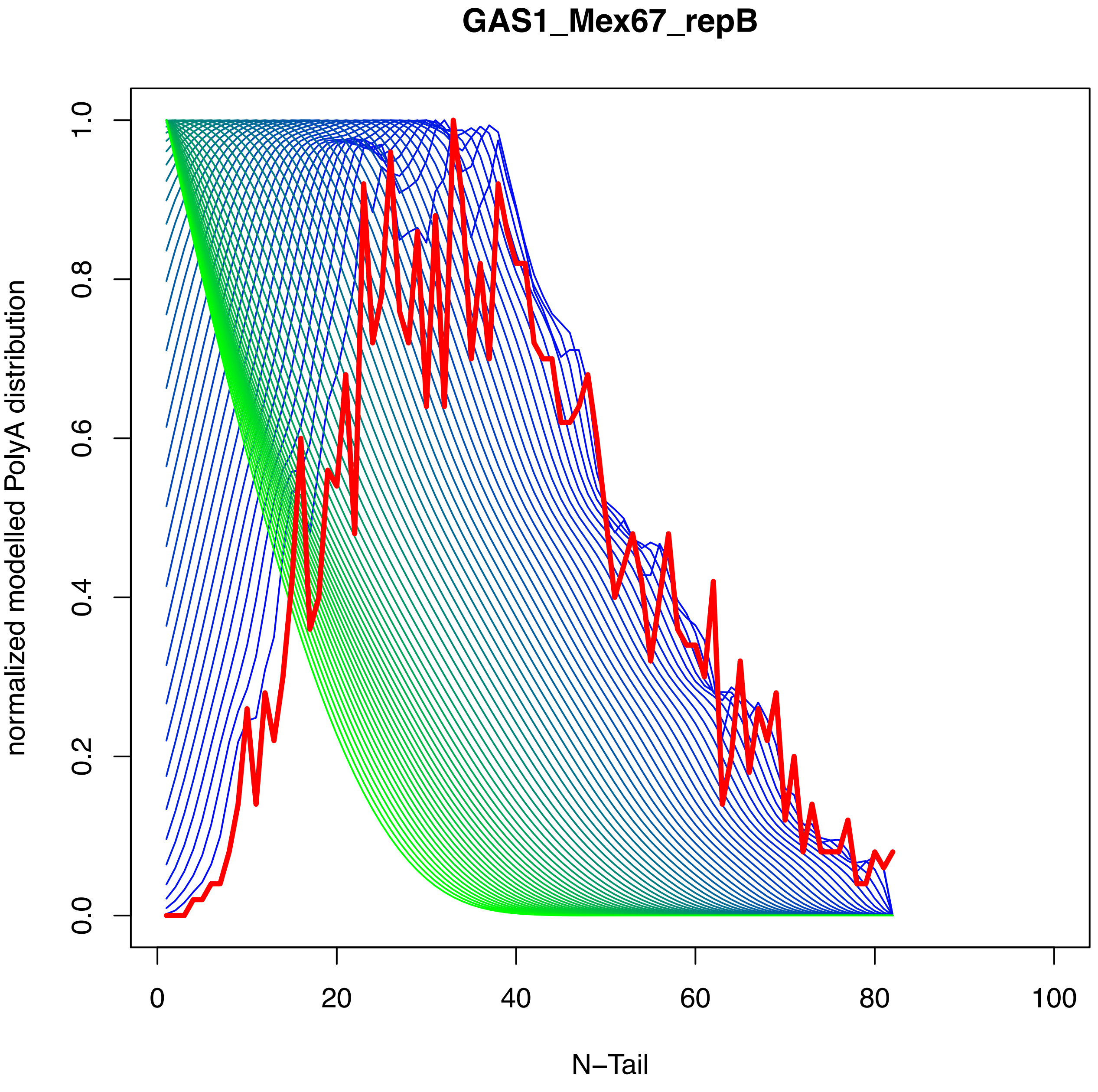

GAS1_Mex67_repB
normalized modelled PolyA distribution
N−Tail
0.0
0.2
0.4
0.6
0.8
1.0
0
20
40
60
80
100

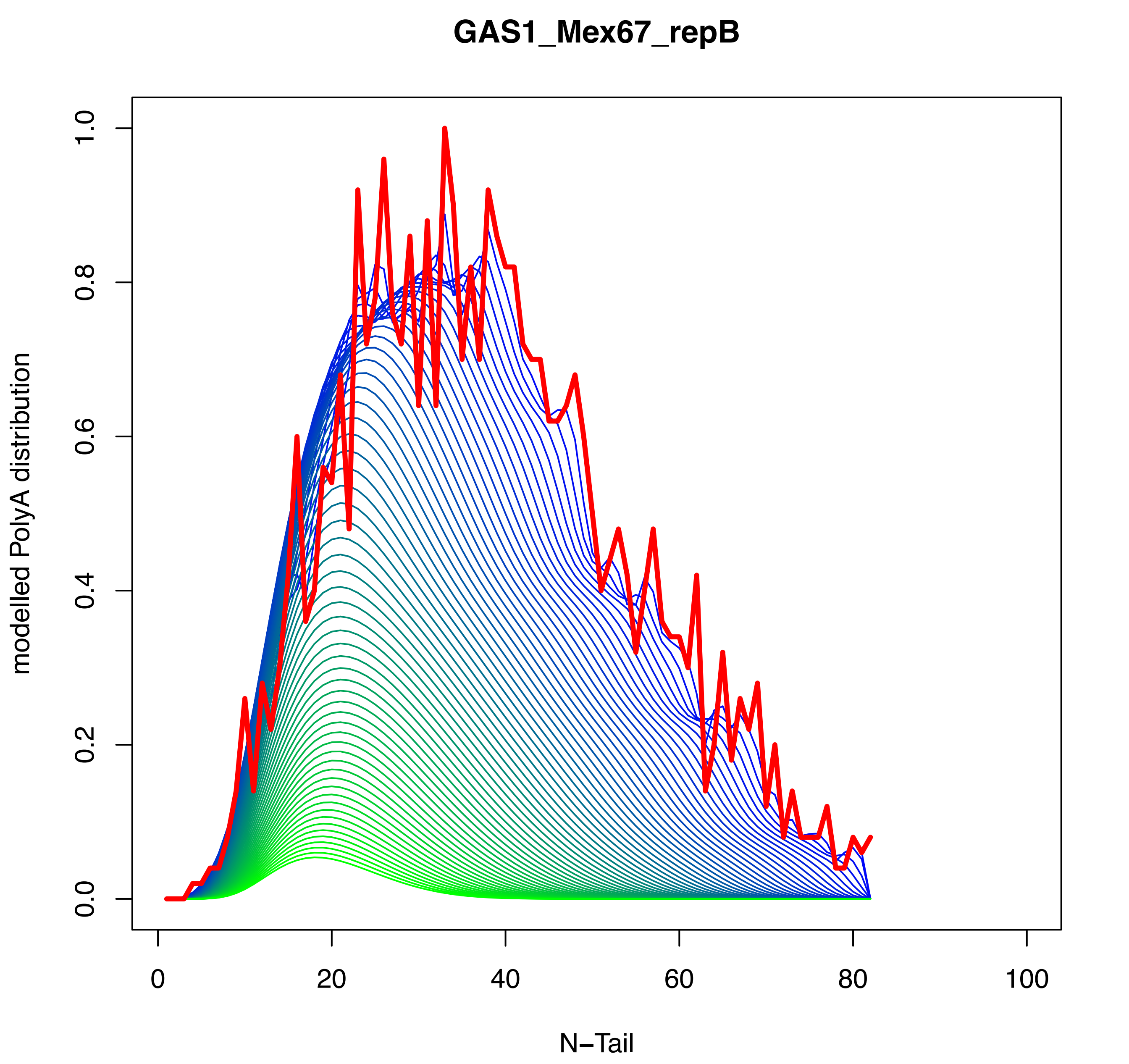
GAS1_Mex67_repB
modelled PolyA distribution
N−Tail
0.0
0.2
0.4
0.6
0.8
1.0
0
20
40
60
80
100

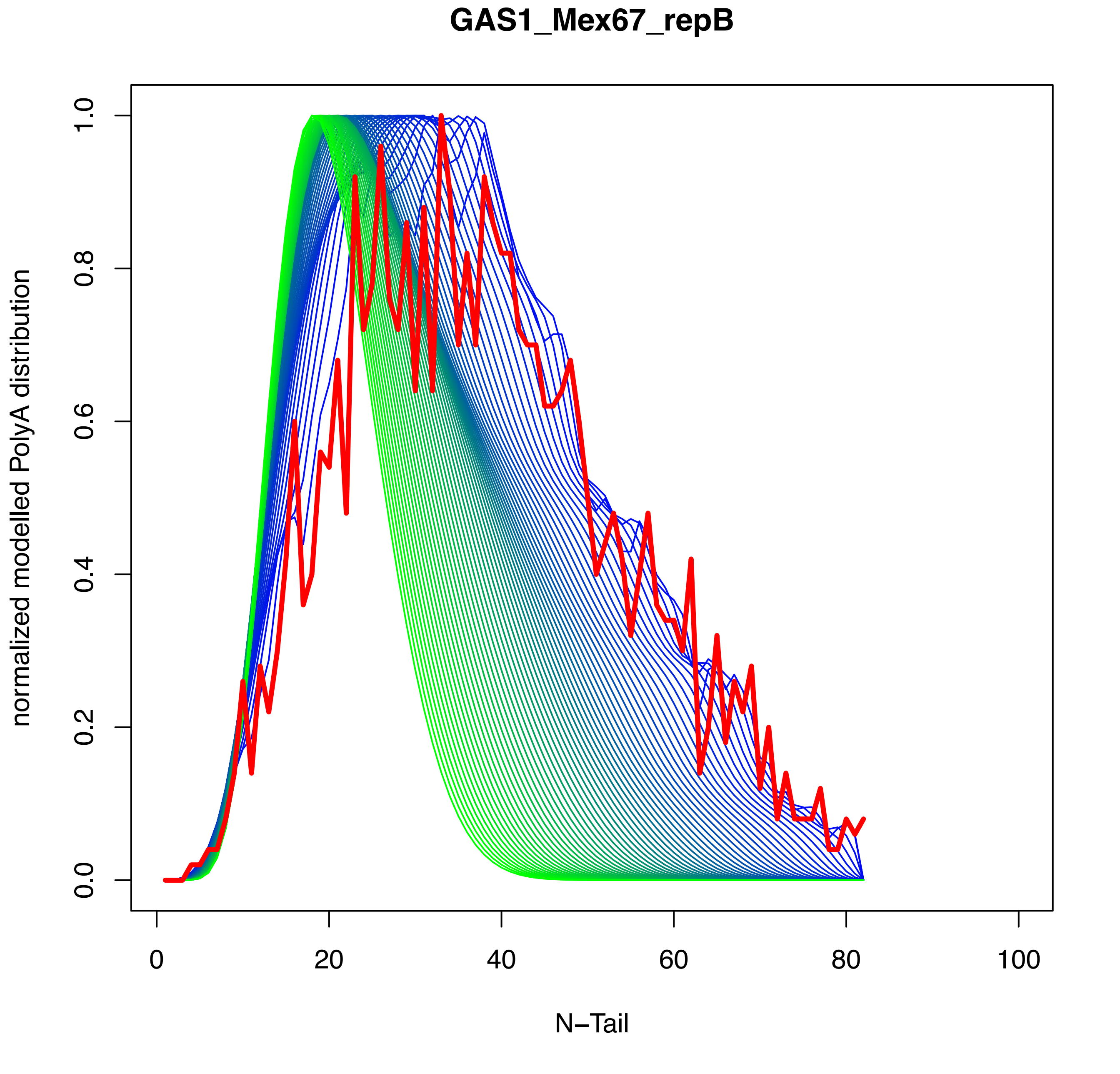
GAS1_Mex67_repB
normalized modelled PolyA distribution
N−Tail
0.0
0.2
0.4
0.6
0.8
1.0
0
20
40
60
80
100

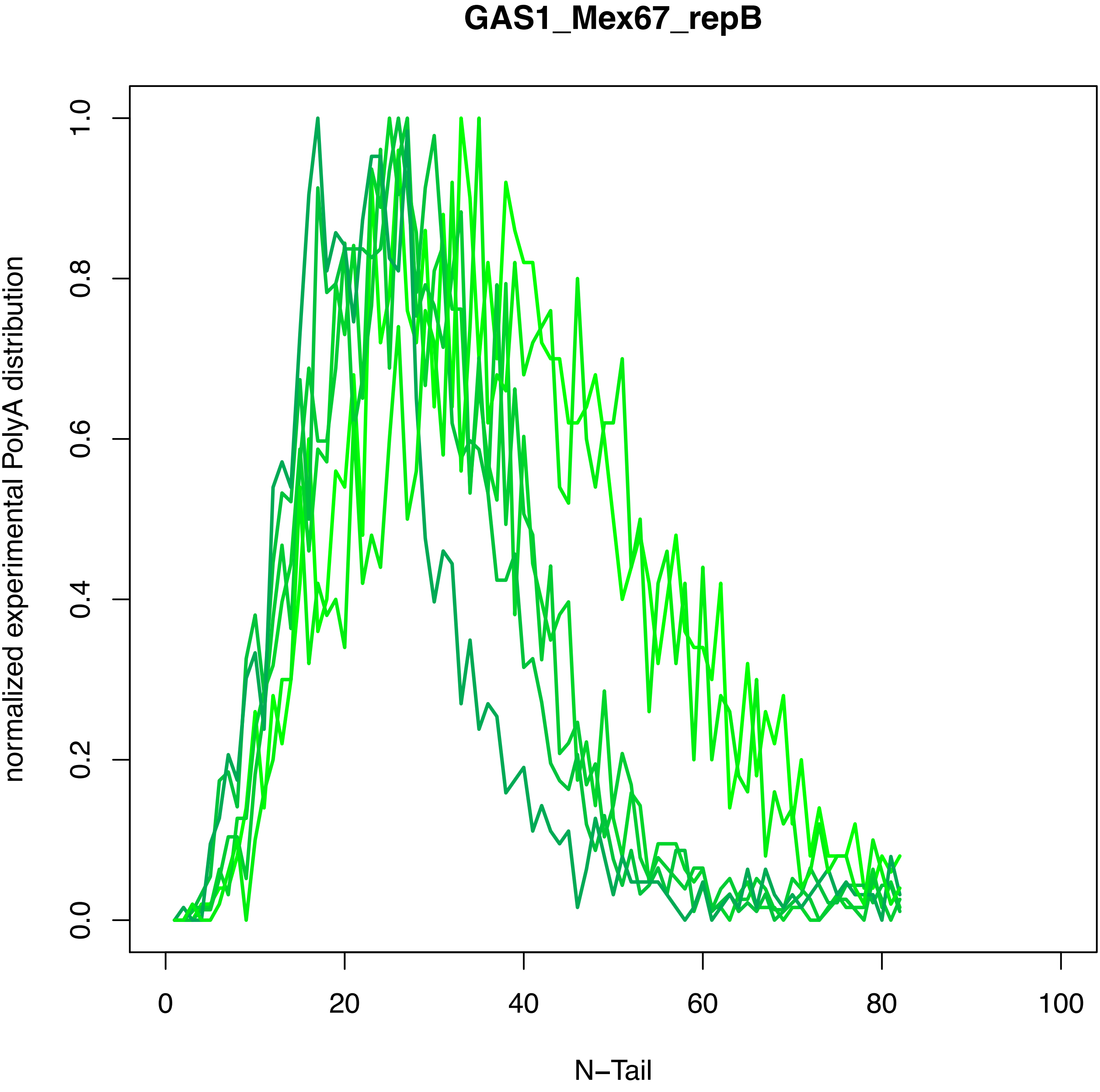
GAS1_Mex67_repB
normalized experimental PolyA distribution
N−Tail
0.0
0.2
0.4
0.6
0.8
1.0
0
20
40
60
80
100

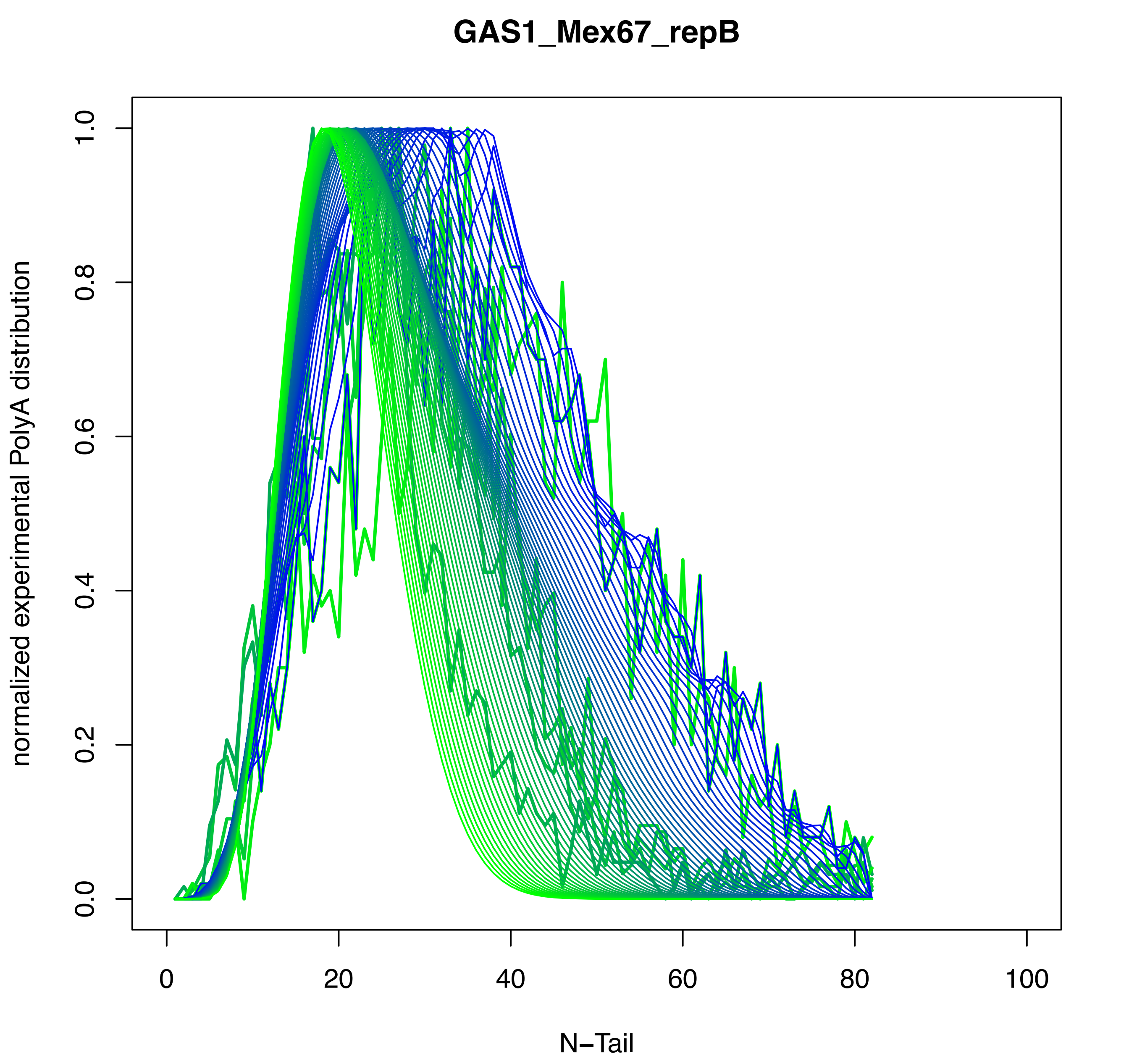
GAS1_Mex67_repB
normalized experimental PolyA distribution
N−Tail
0.0
0.2
0.4
0.6
0.8
1.0
0
20
40
60
80
100

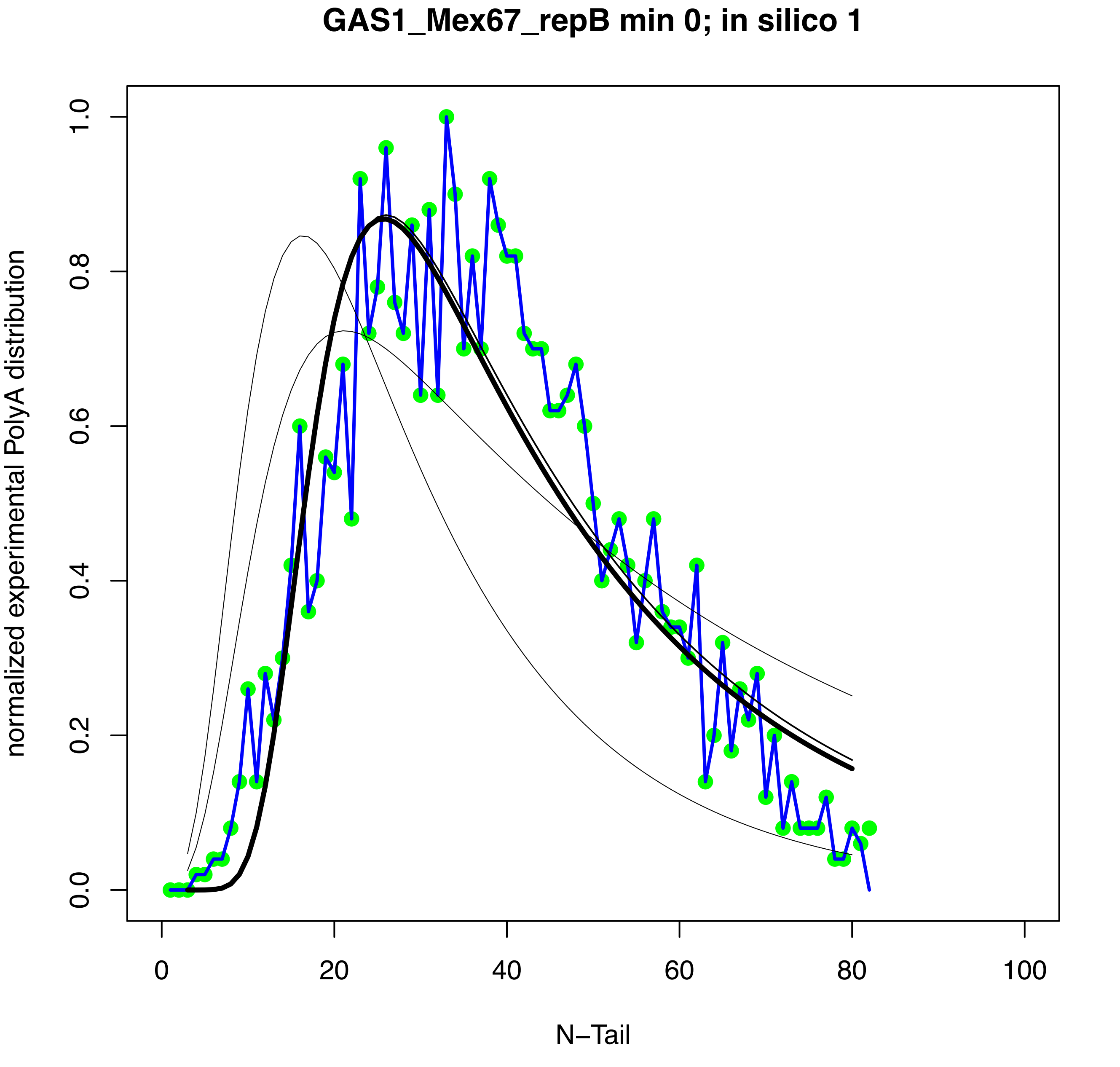
GAS1_Mex67_repB min 0; in silico 1
normalized experimental PolyA distribution
N−Tail
0.0
0.2
0.4
0.6
0.8
1.0
0
20
40
60
80
100

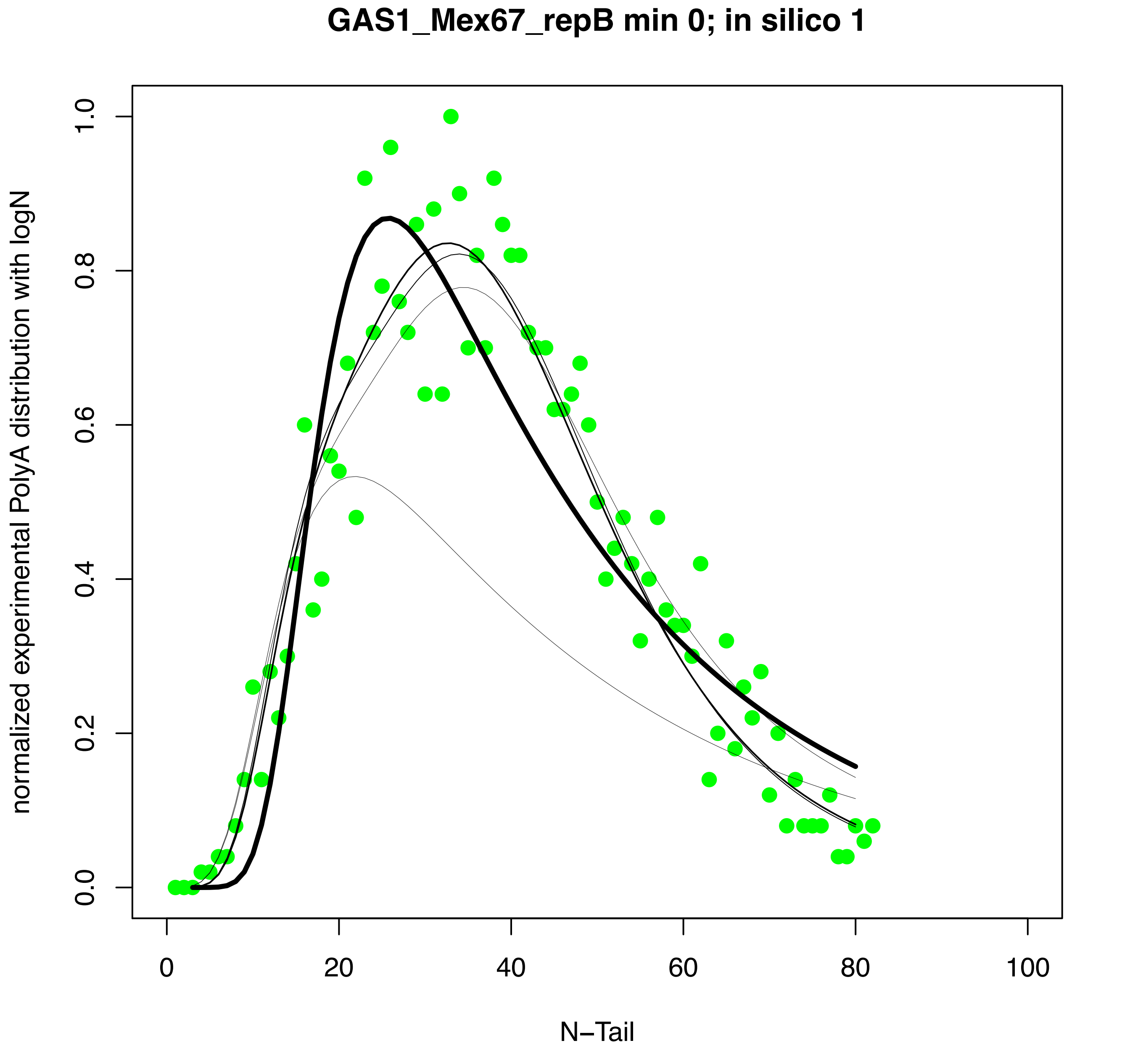

GAS1_Mex67_repB min 0; in silico 1
normalized experimental PolyA distribution with logN
N−Tail
0.0
0.2
0.4
0.6
0.8
1.0
0
20
40
60
80
100

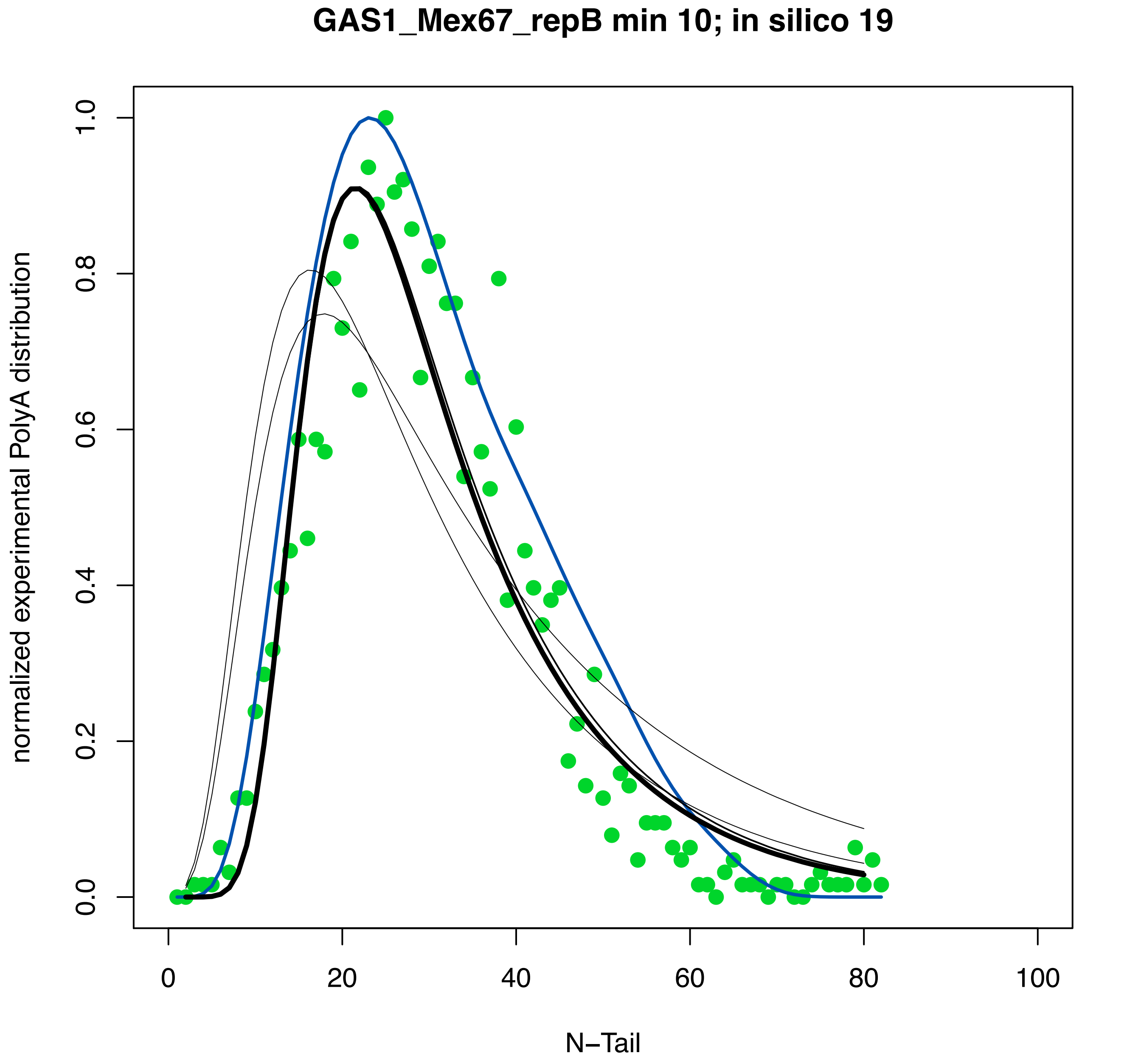
GAS1_Mex67_repB min 10; in silico 19
normalized experimental PolyA distribution
0.0
0.2
0.4
0.6
0.8
1.0
0
20
40
60
80
100
N−Tail

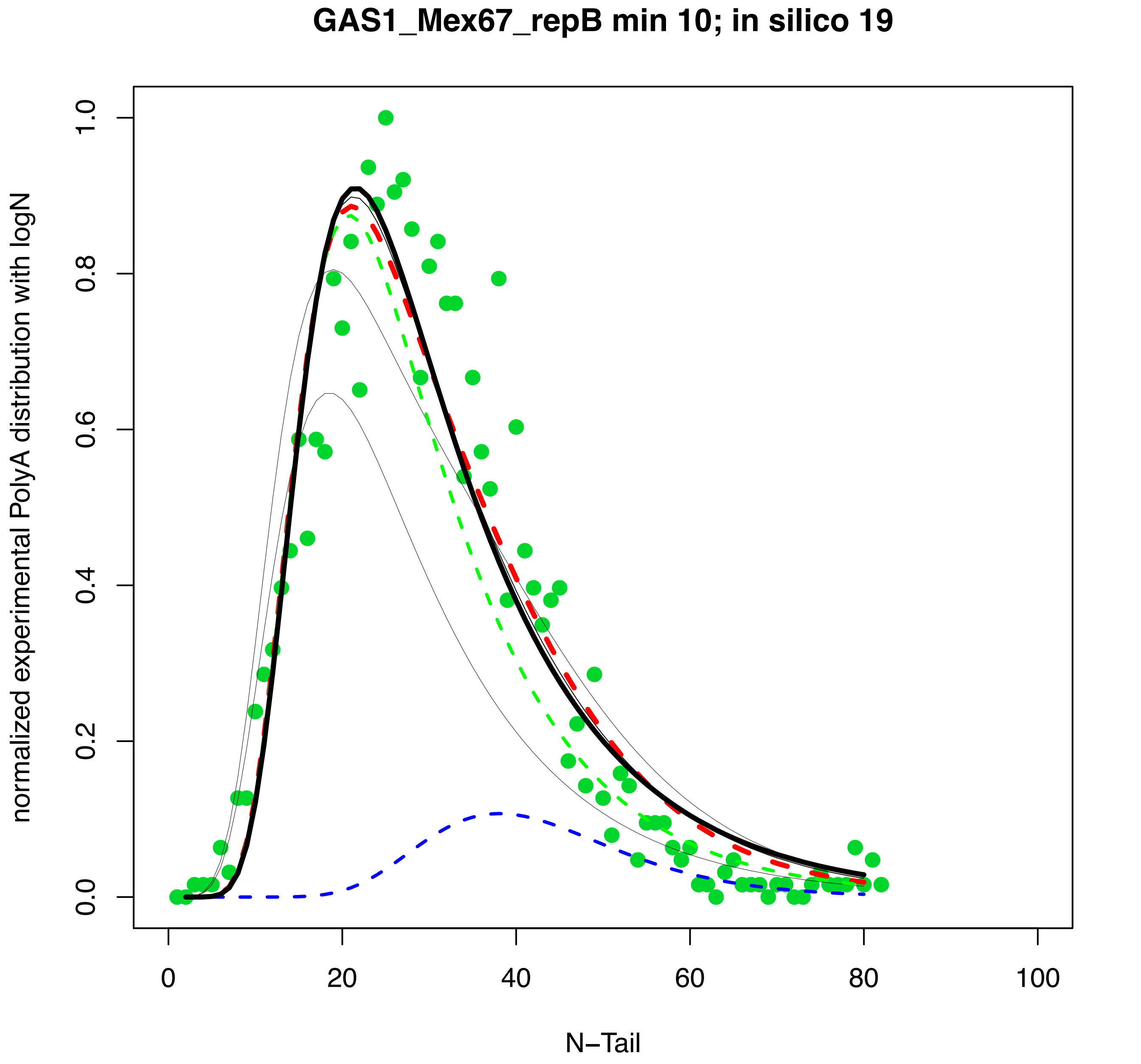

GAS1_Mex67_repB min 10; in silico 19
normalized experimental PolyA distribution with logN
N−Tail
0.0
0.2
0.4
0.6
0.8
1.0
0
20
40
60
80
100

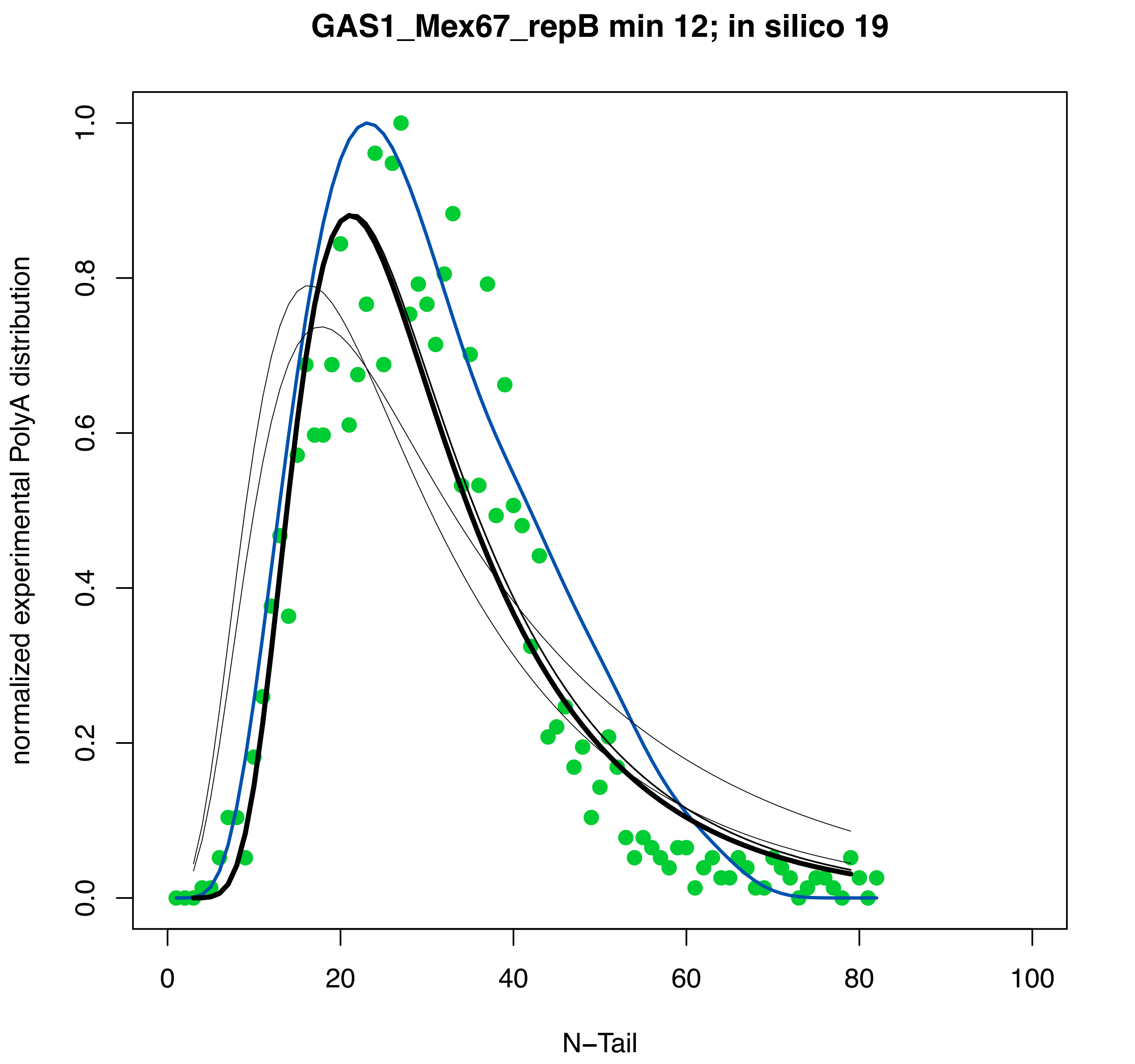
GAS1_Mex67_repB min 12; in silico 19
normalized experimental PolyA distribution
N−Tail
0.0
0.2
0.4
0.6
0.8
1.0
0
20
40
60
80
100

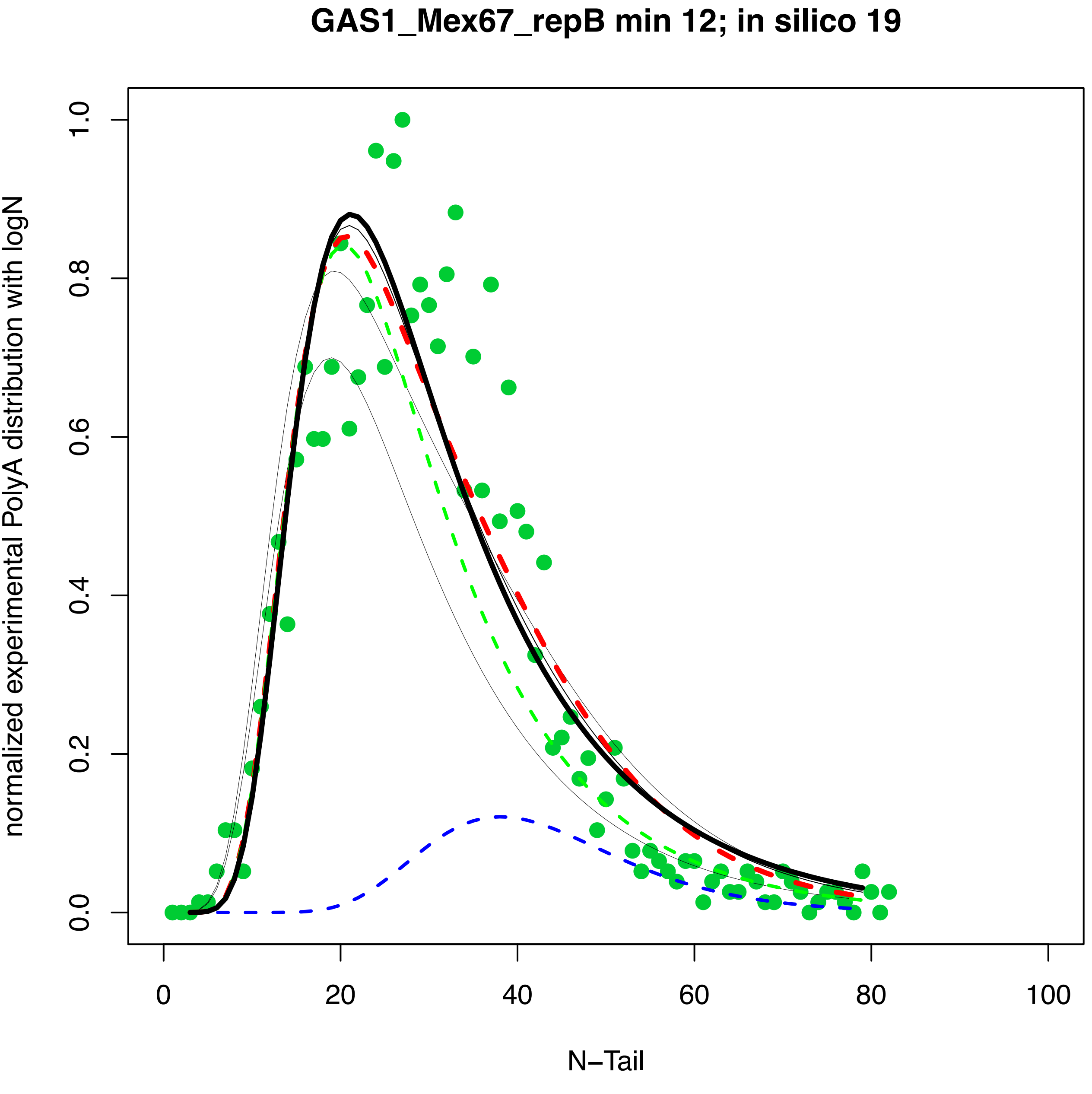

GAS1_Mex67_repB min 12; in silico 19
normalized experimental PolyA distribution with logN
N−Tail
0.0
0.2
0.4
0.6
0.8
1.0
0
20
40
60
80
100

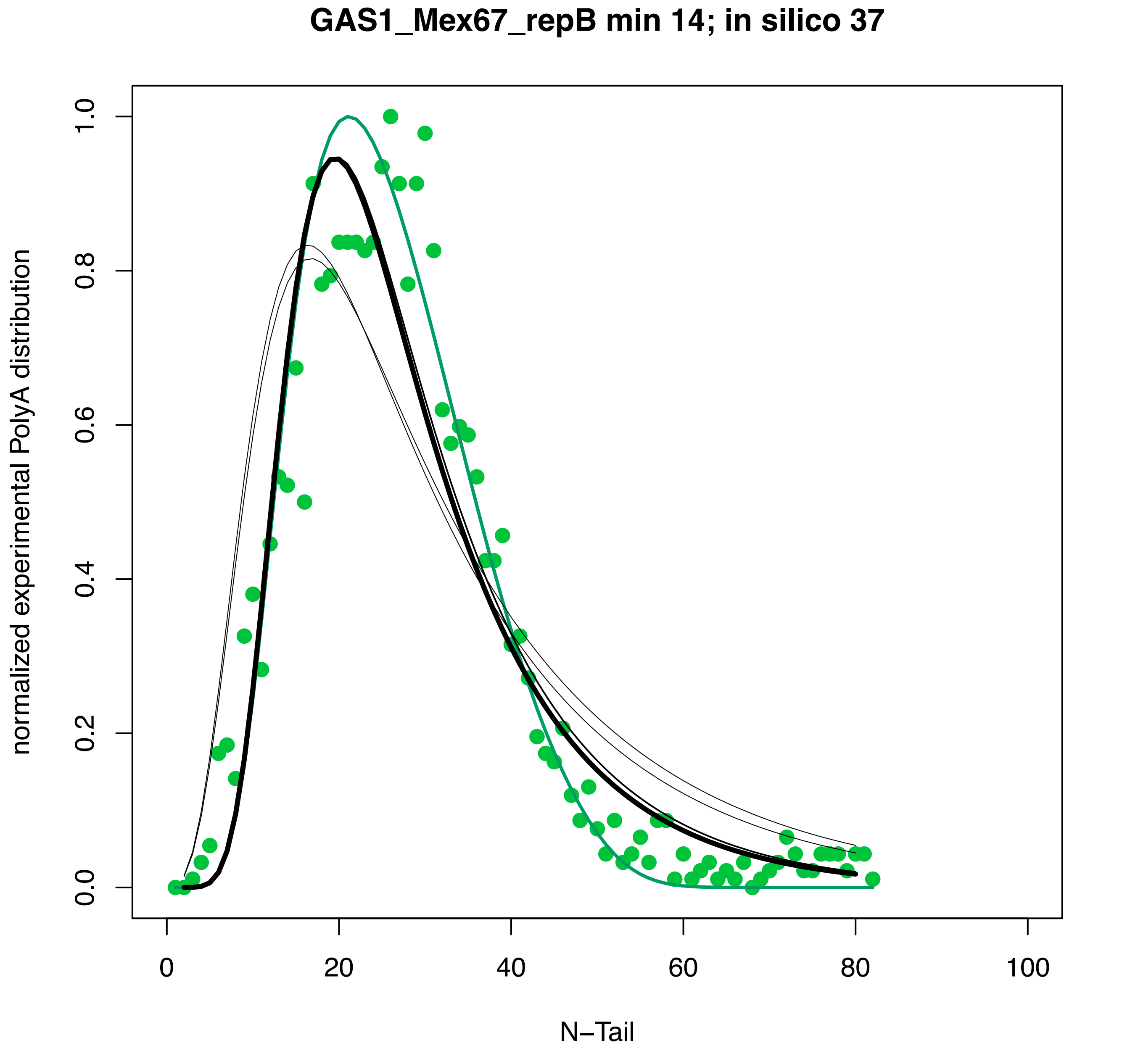

GAS1_Mex67_repB min 14; in silico 37
normalized experimental PolyA distribution
N−Tail
0.0
0.2
0.4
0.6
0.8
1.0
0
20
40
60
80
100

GAS1_Mex67_repB min 14; in silico 37

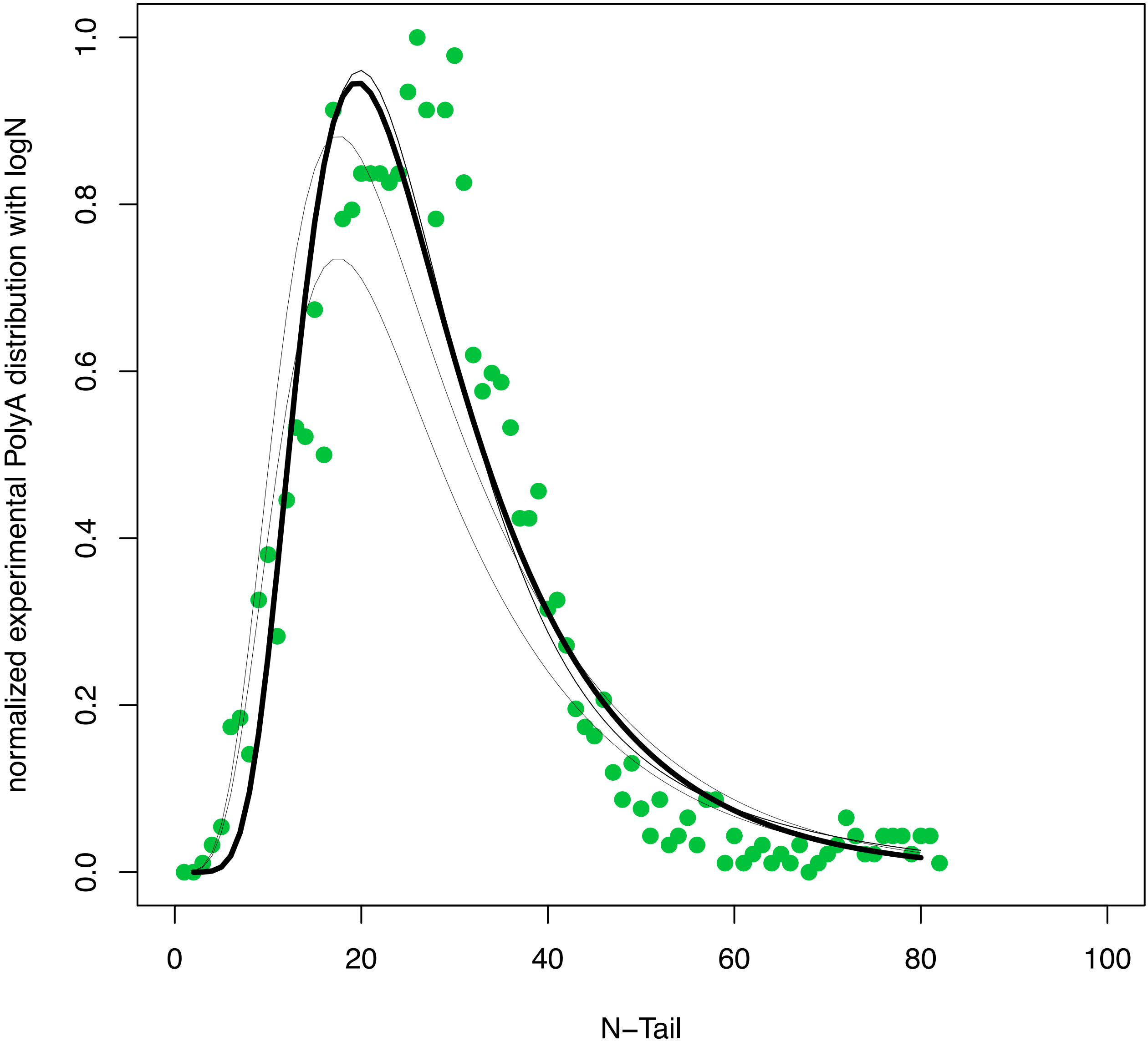

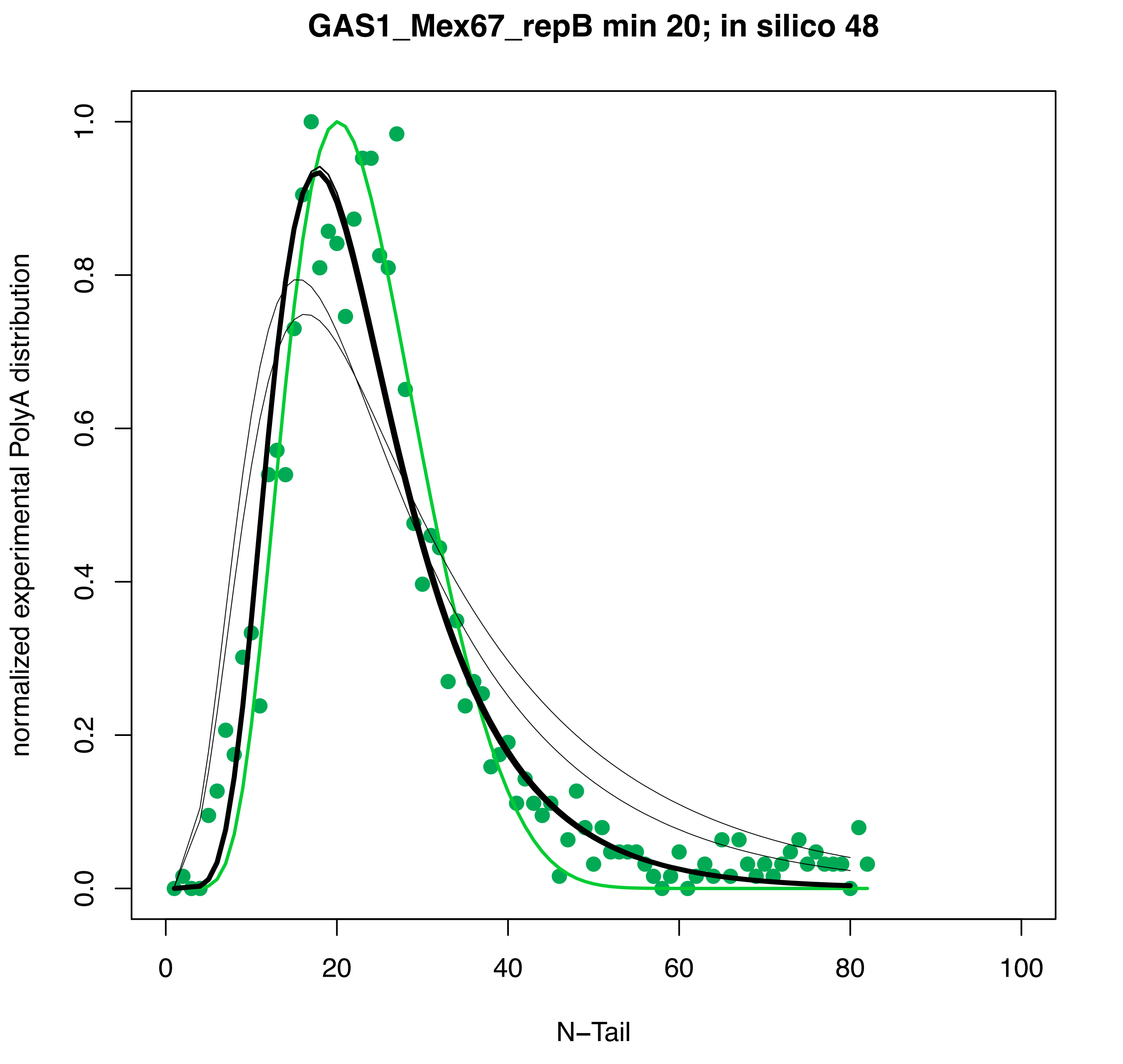

GAS1_Mex67_repB min 20; in silico 48
normalized experimental PolyA distribution
0.0
0.2
0.4
0.6
0.8
1.0
0
20
40
60
80
100
N−Tail

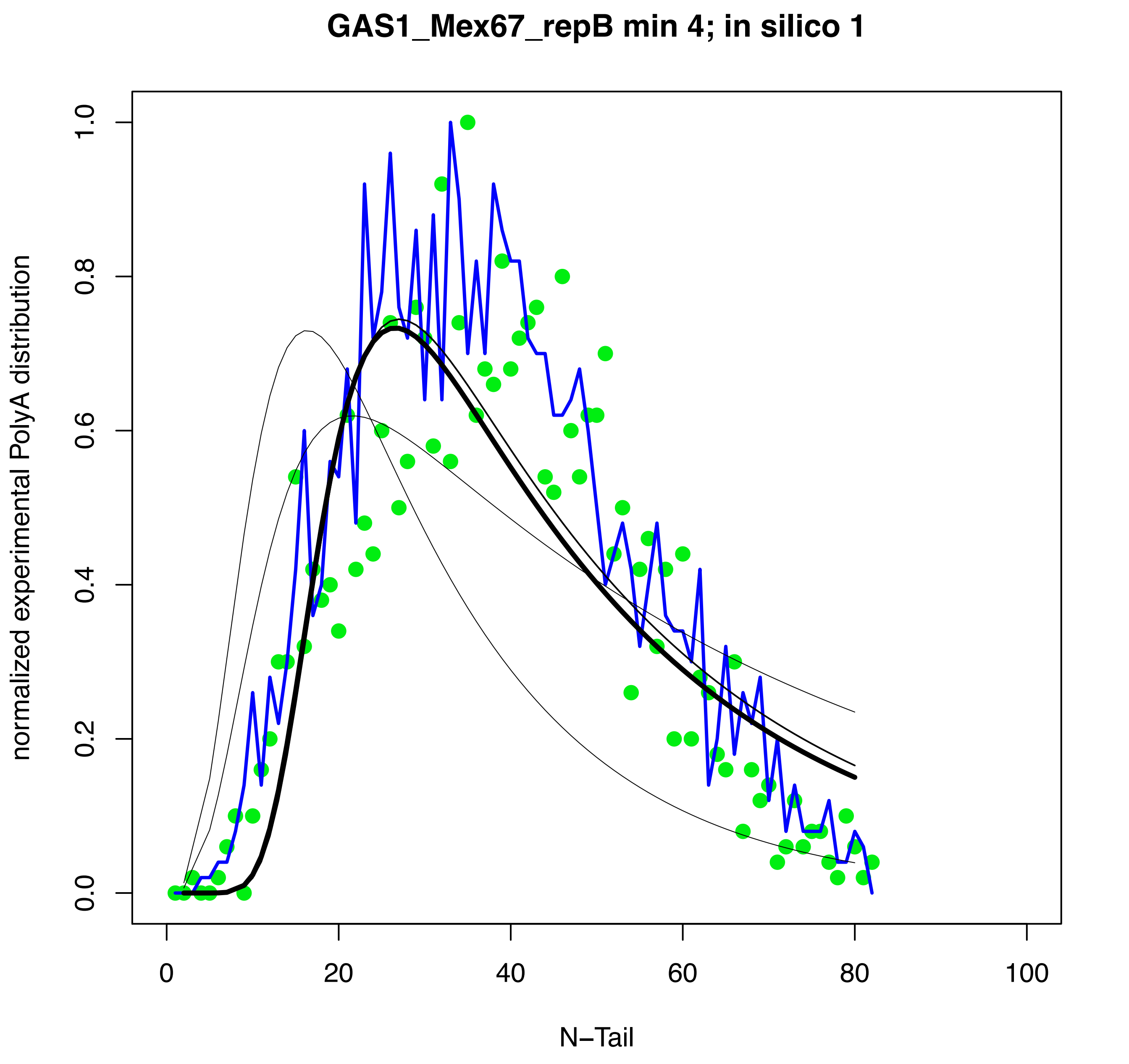
GAS1_Mex67_repB min 4; in silico 1
normalized experimental PolyA distribution
N−Tail
0.0
0.2
0.4
0.6
0.8
1.0
0
20
40
60
80
100

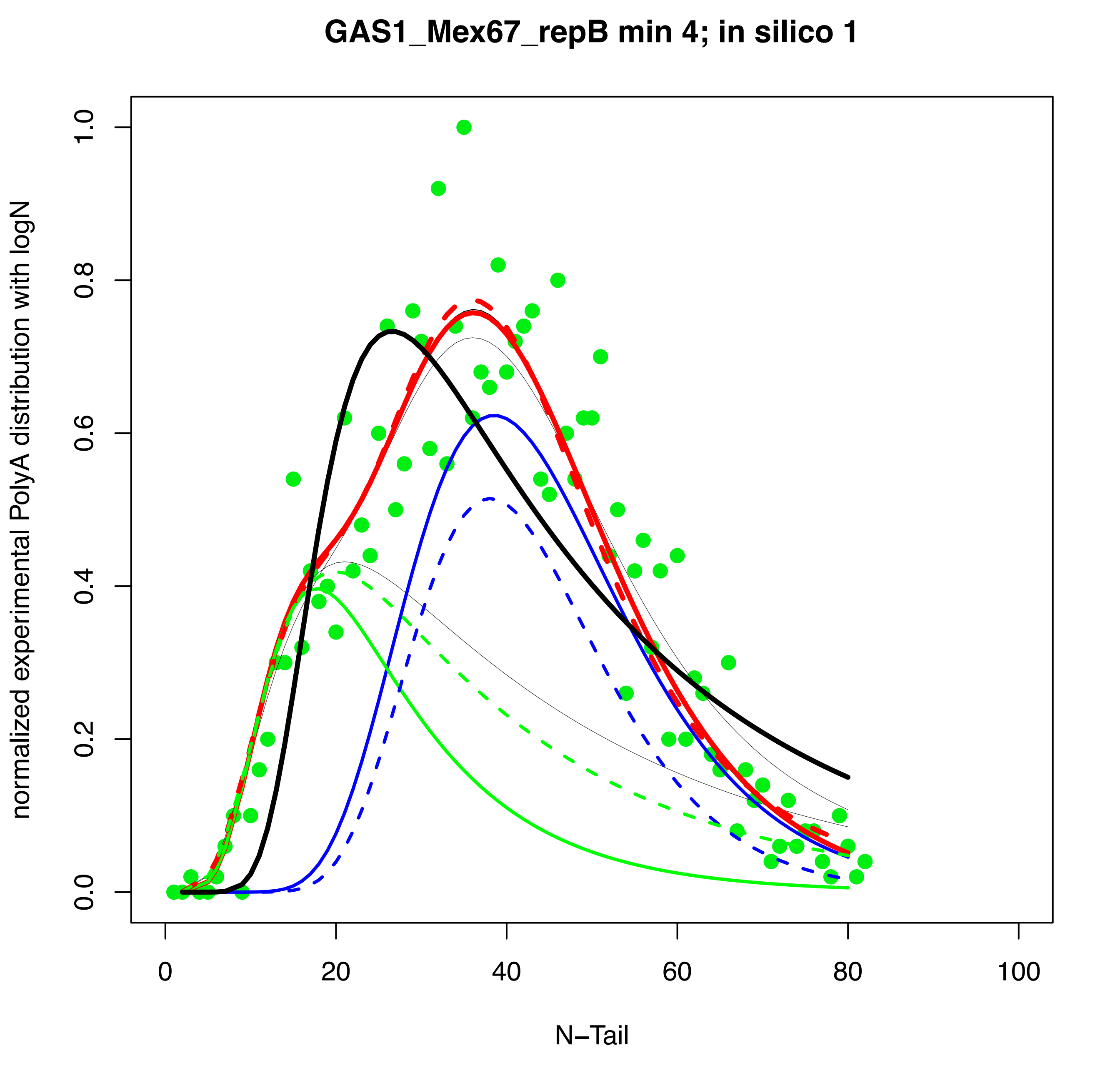

GAS1_Mex67_repB min 4; in silico 1
normalized experimental PolyA distribution with logN
N−Tail
0.0
0.2
0.4
0.6
0.8
1.0
0
20
40
60
80
100

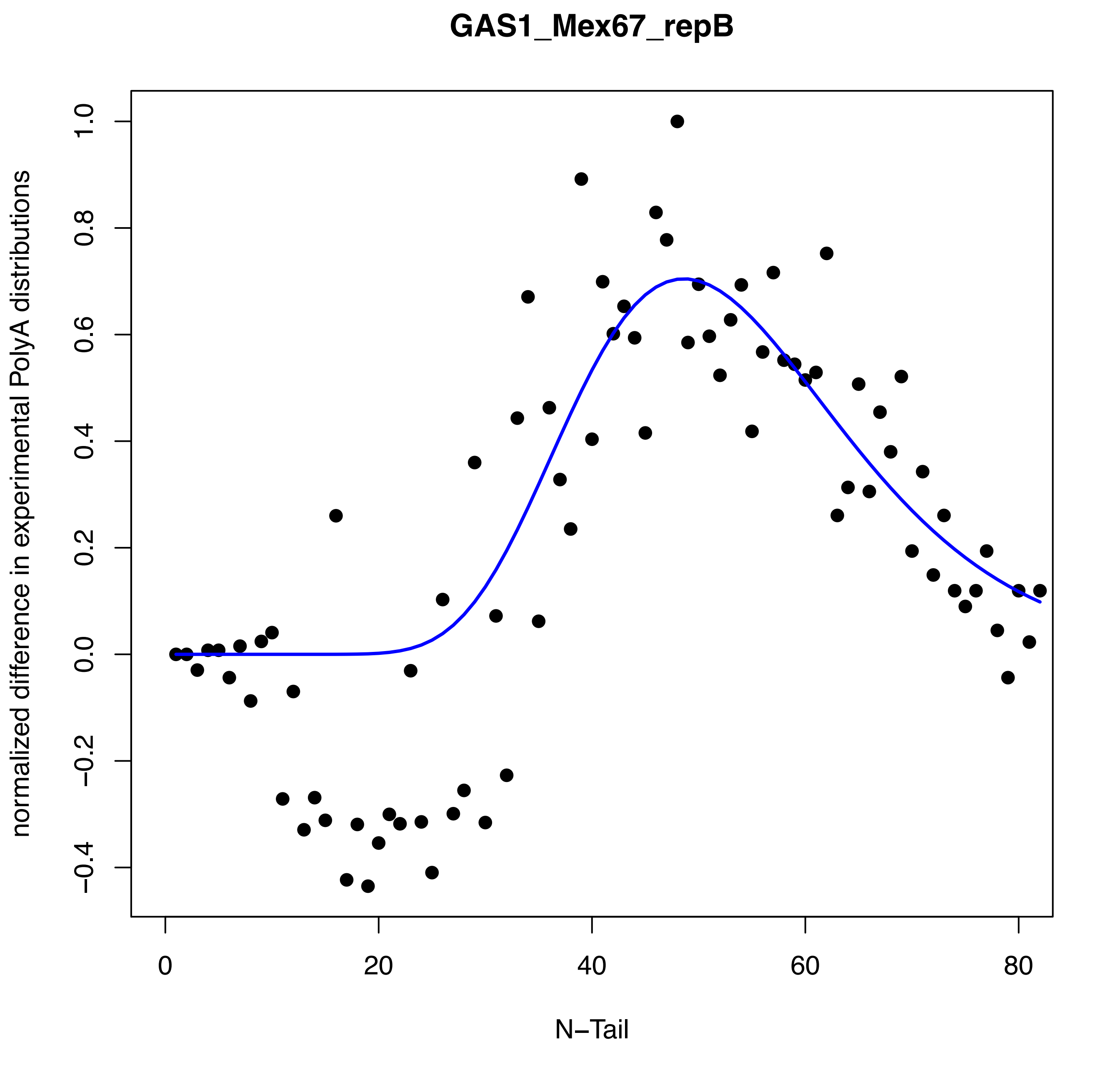

GAS1_Mex67_repB
normalized difference in experimental PolyA distributions
1.0
0.8
0.6
0.4
0.2
0.0
−0.2
−0.4
0
20
40
60
80
N−Tail

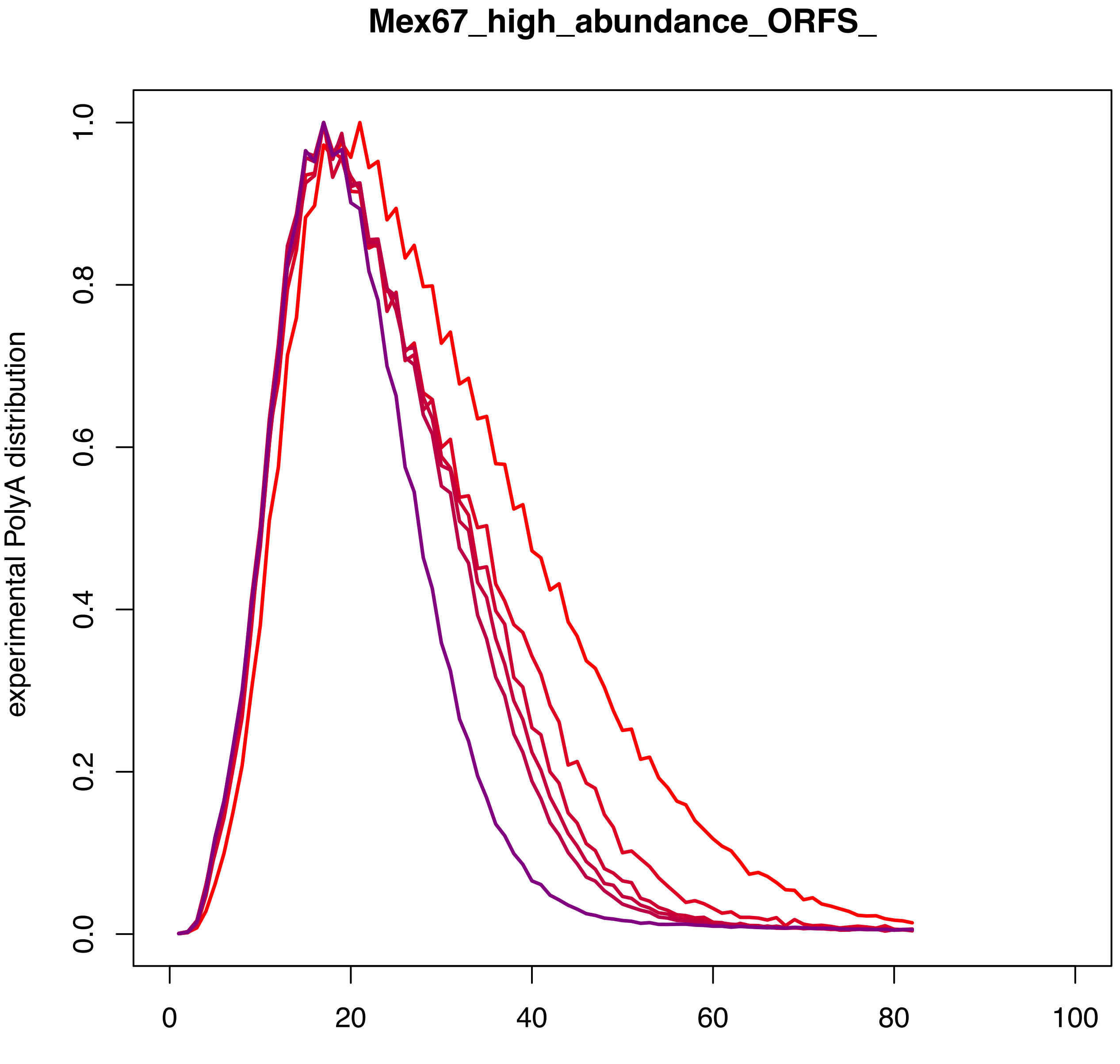

Mex67_high_abundance_ORFS_
experimental PolyA distribution
0.0
0.2
0.4
0.6
0.8
1.0
0
20
40
60
80
100
N−Tail

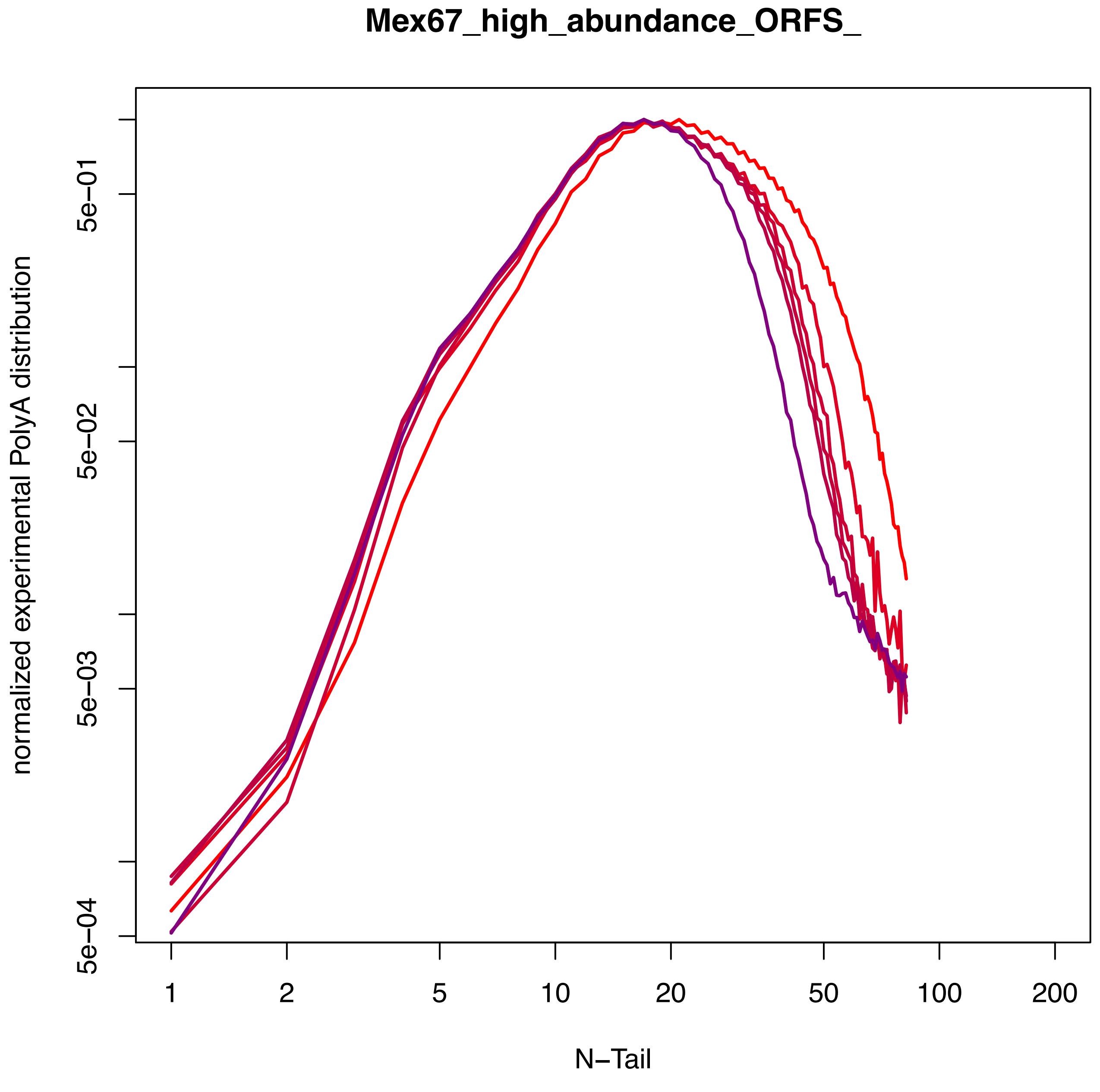
Mex67_high_abundance_ORFS_
normalized experimental PolyA distribution
5e−01
5e−02
5e−03
5e−04
1
2
5
10
20
50
100
200
N−Tail

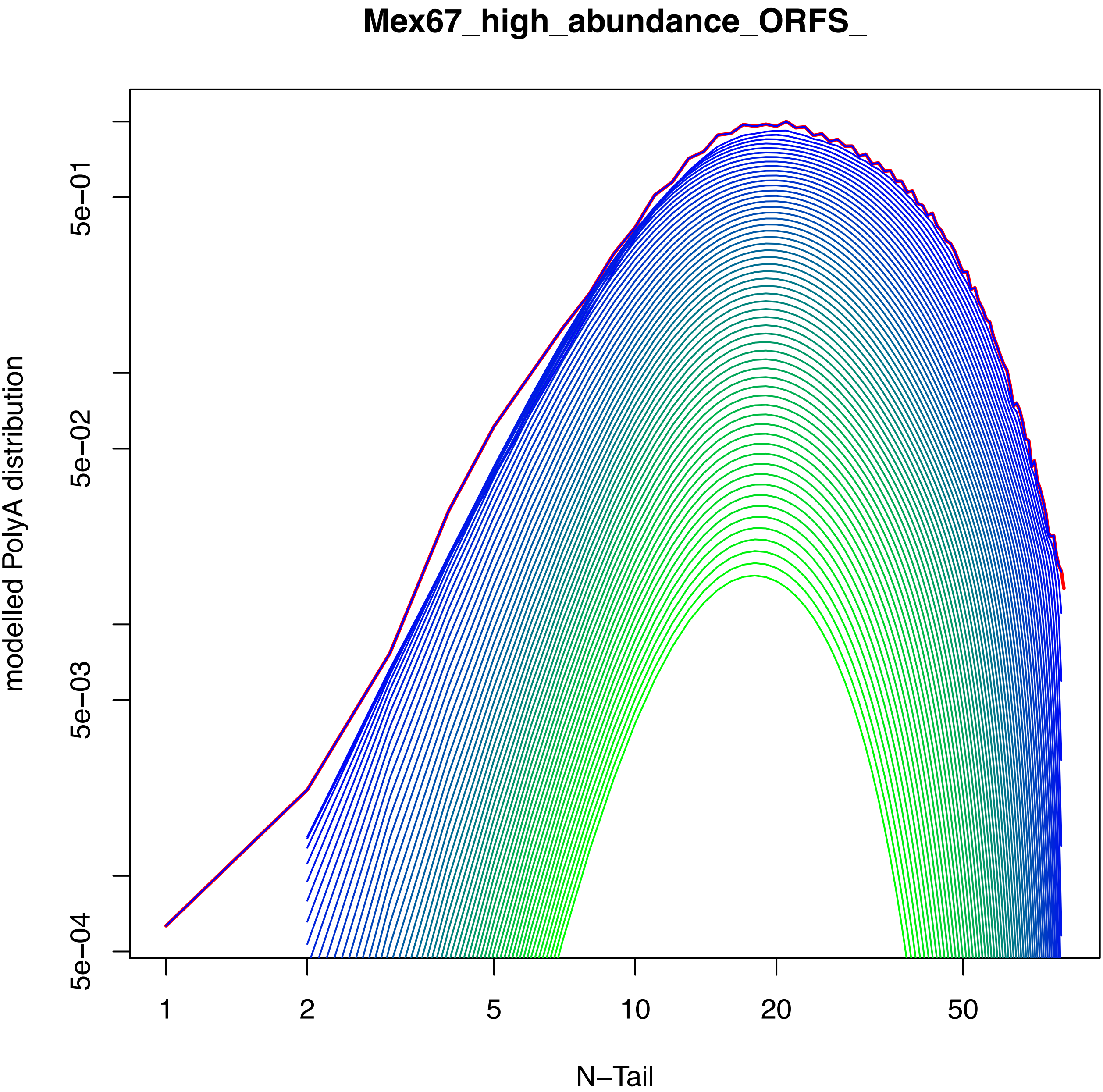

Mex67_high_abundance_ORFS_
modelled PolyA distribution
5e−01
5e−02
5e−03
5e−04
1
2
5
10
20
50
N−Tail

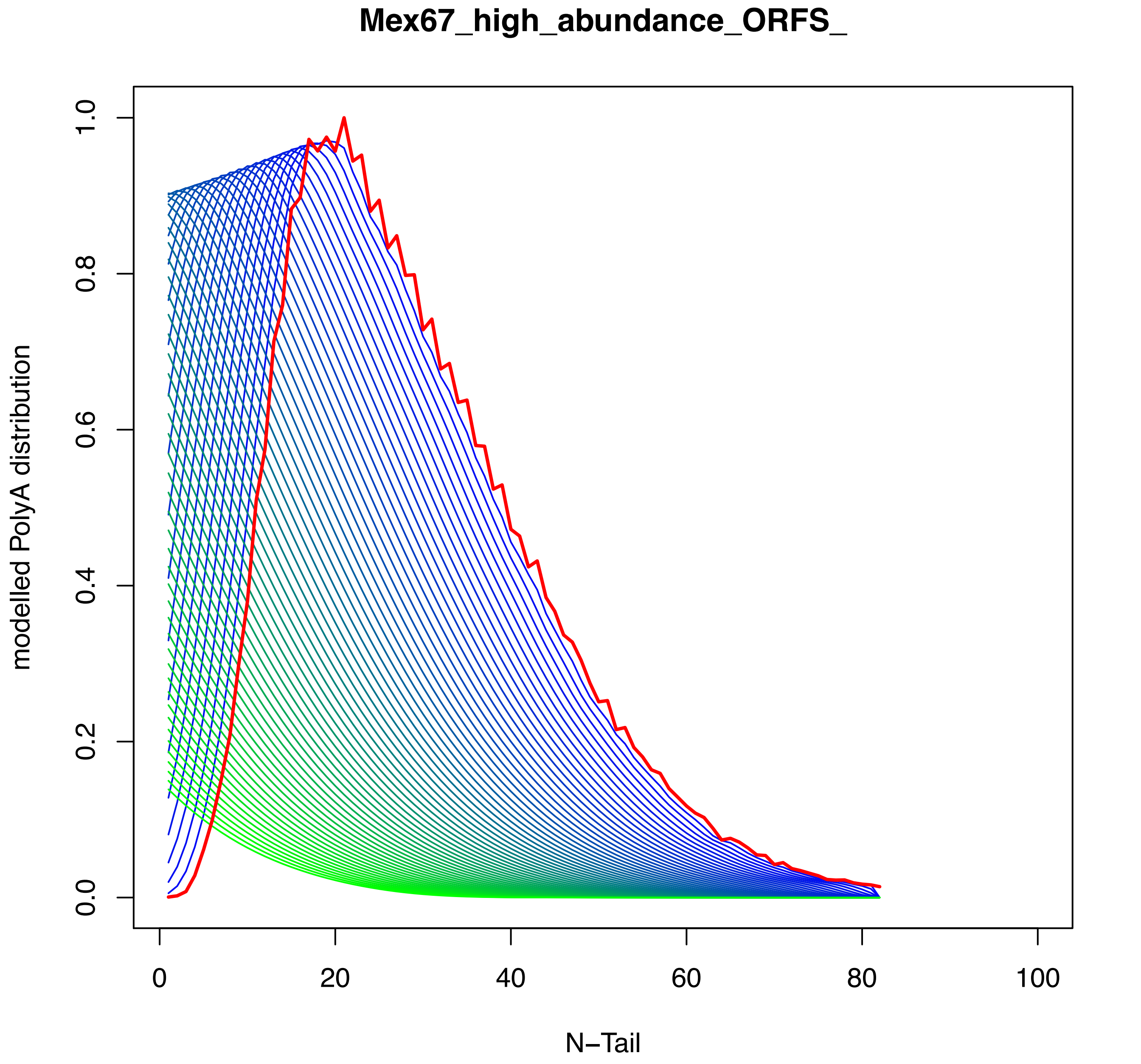

Mex67_high_abundance_ORFS_
modelled PolyA distribution
N−Tail
0.0
0.2
0.4
0.6
0.8
1.0
0
20
40
60
80
100

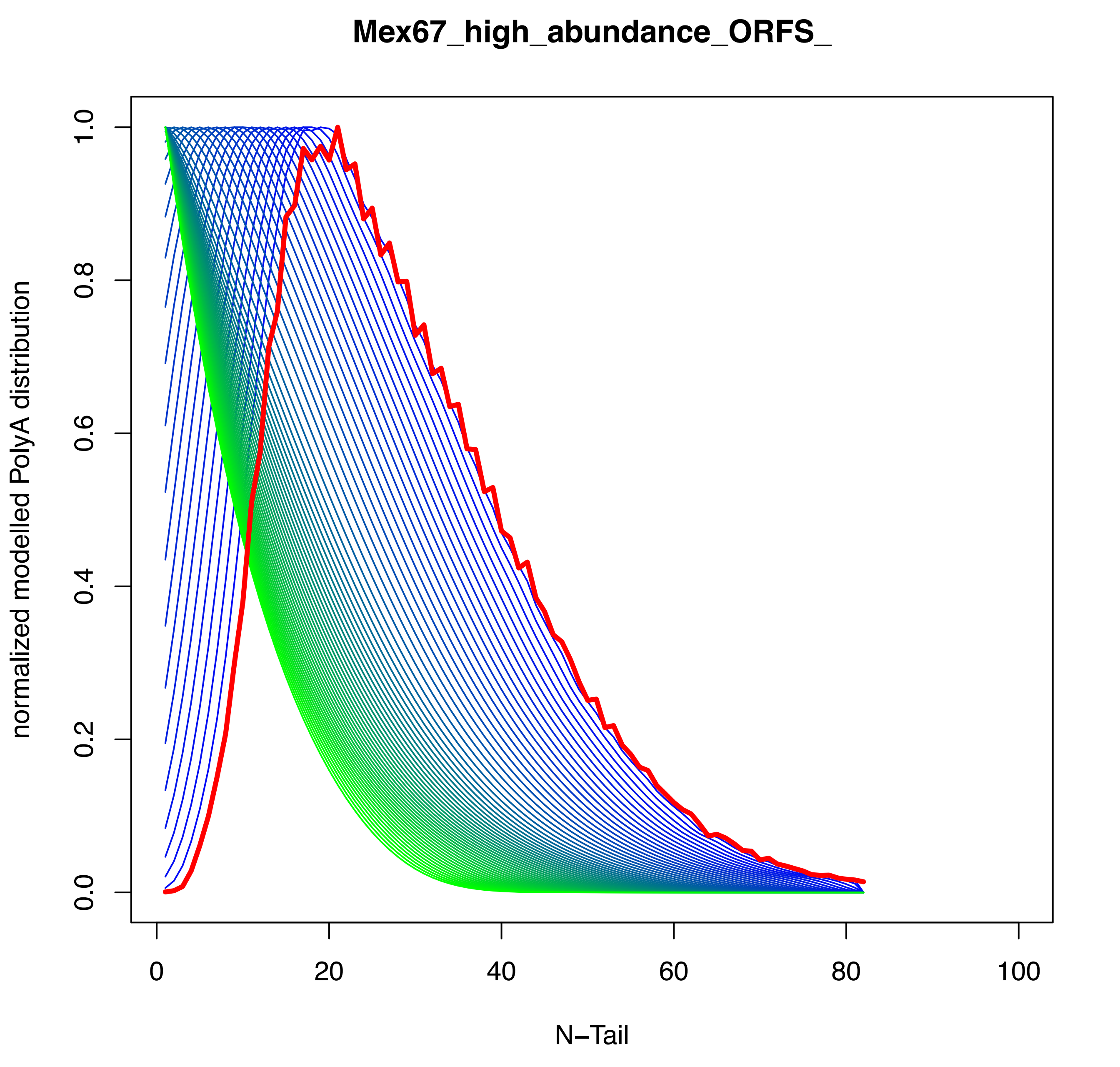

Mex67_high_abundance_ORFS_
normalized modelled PolyA distribution
N−Tail
0.0
0.2
0.4
0.6
0.8
1.0
0
20
40
60
80
100

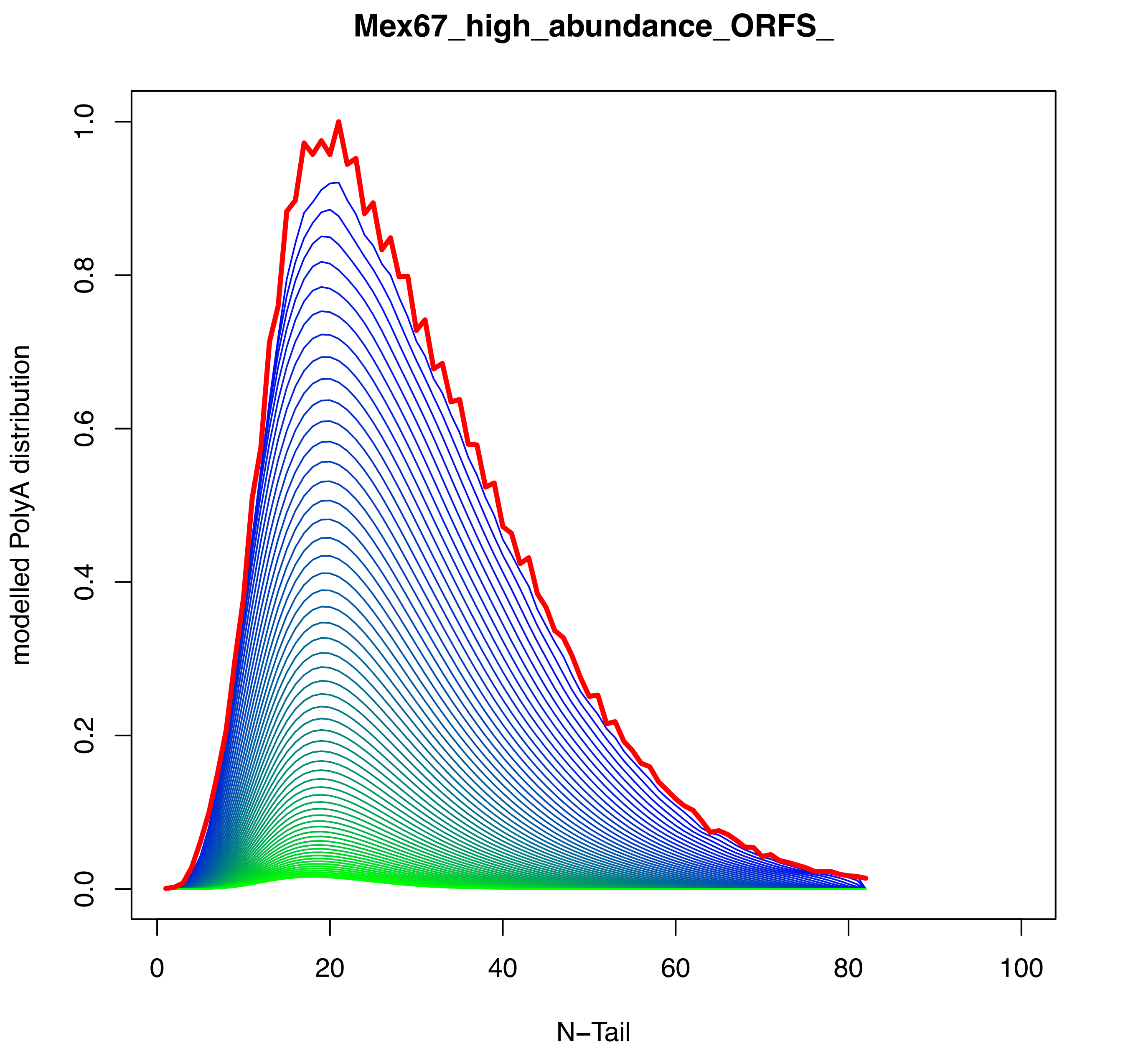

Mex67_high_abundance_ORFS_
modelled PolyA distribution
N−Tail
0.0
0.2
0.4
0.6
0.8
1.0
0
20
40
60
80
100

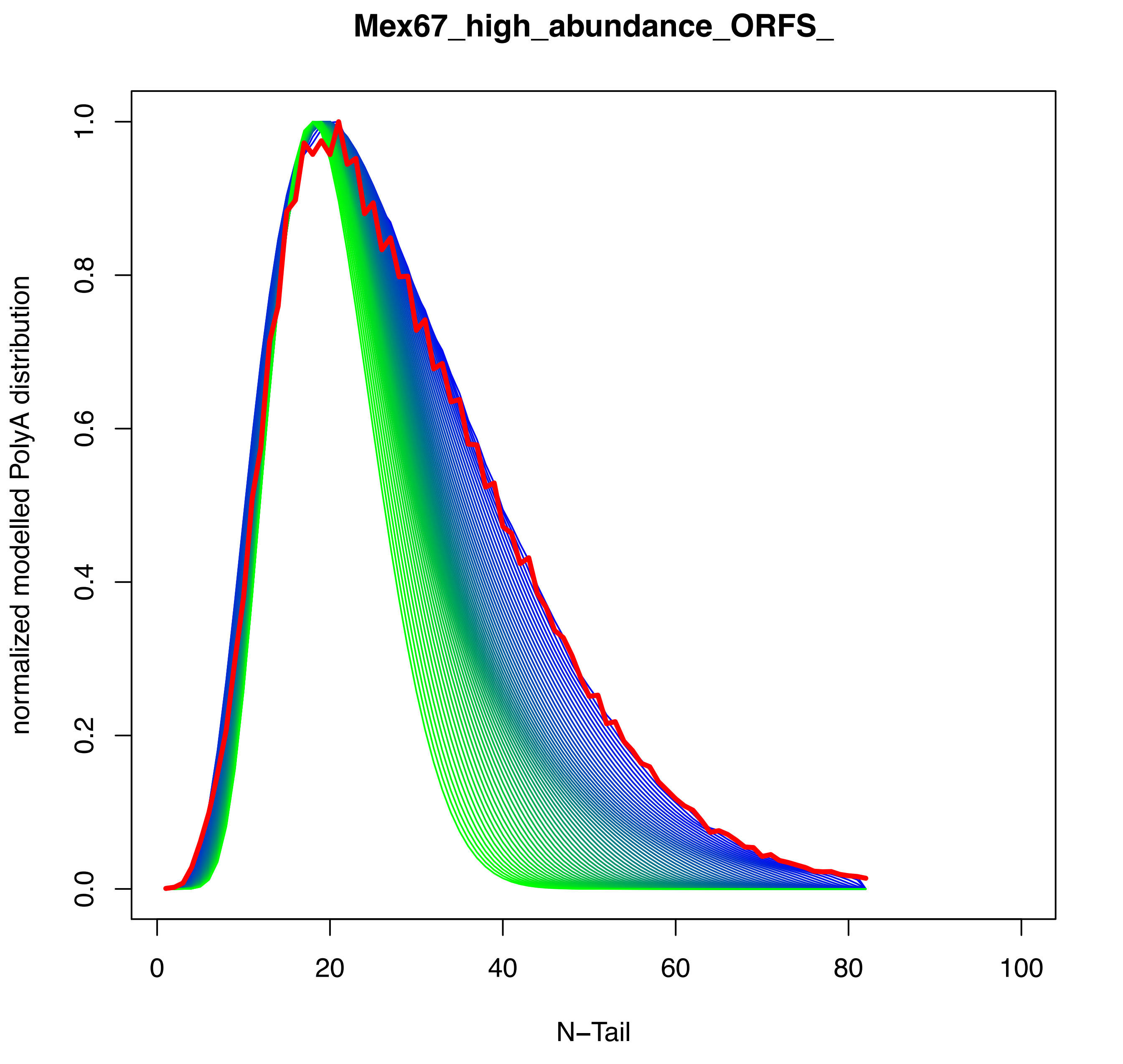
Mex67_high_abundance_ORFS_
normalized modelled PolyA distribution
N−Tail
0.0
0.2
0.4
0.6
0.8
1.0
0
20
40
60
80
100

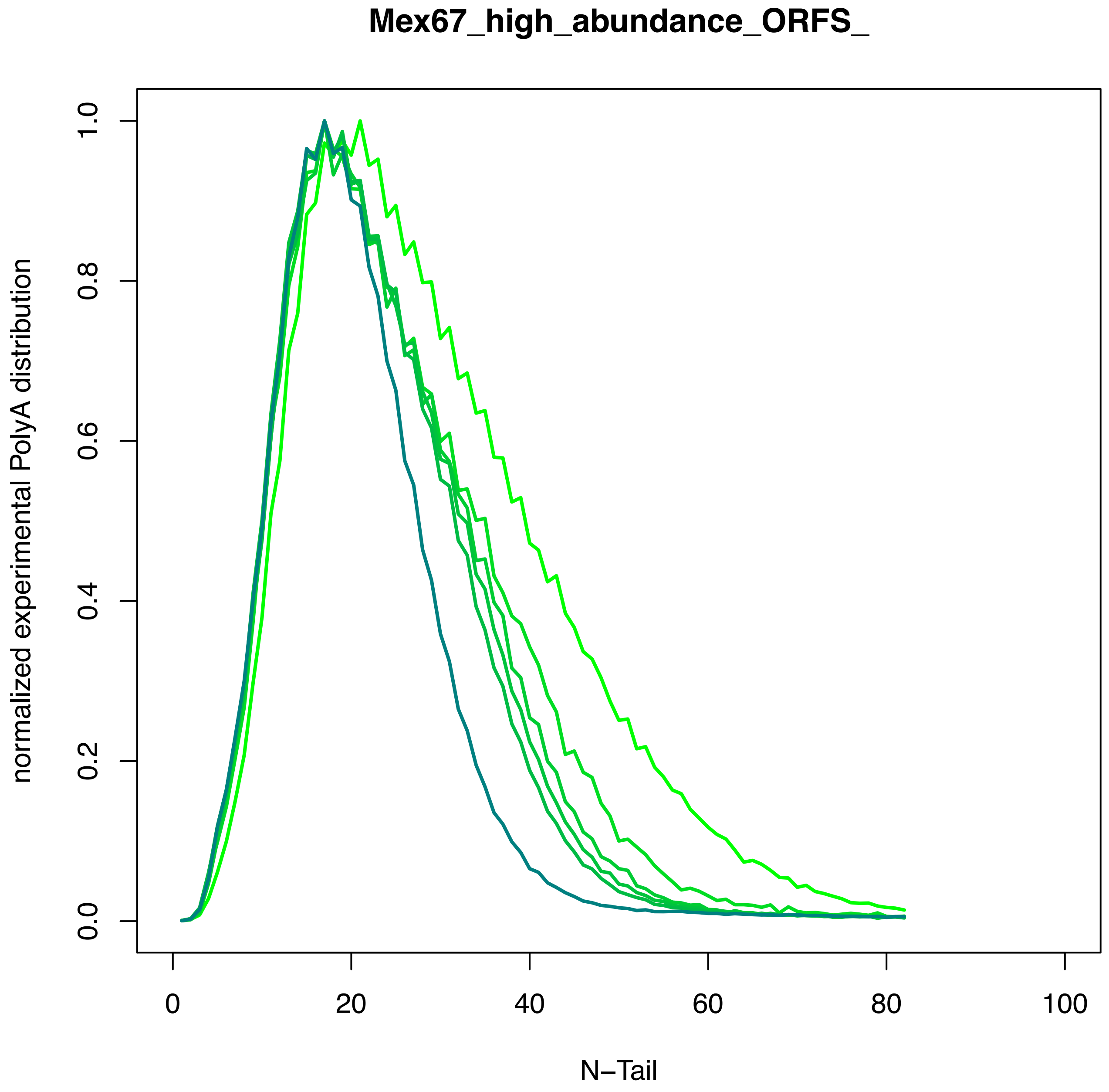

Mex67_high_abundance_ORFS_
normalized experimental PolyA distribution
N−Tail
0.0
0.2
0.4
0.6
0.8
1.0
0
20
40
60
80
100

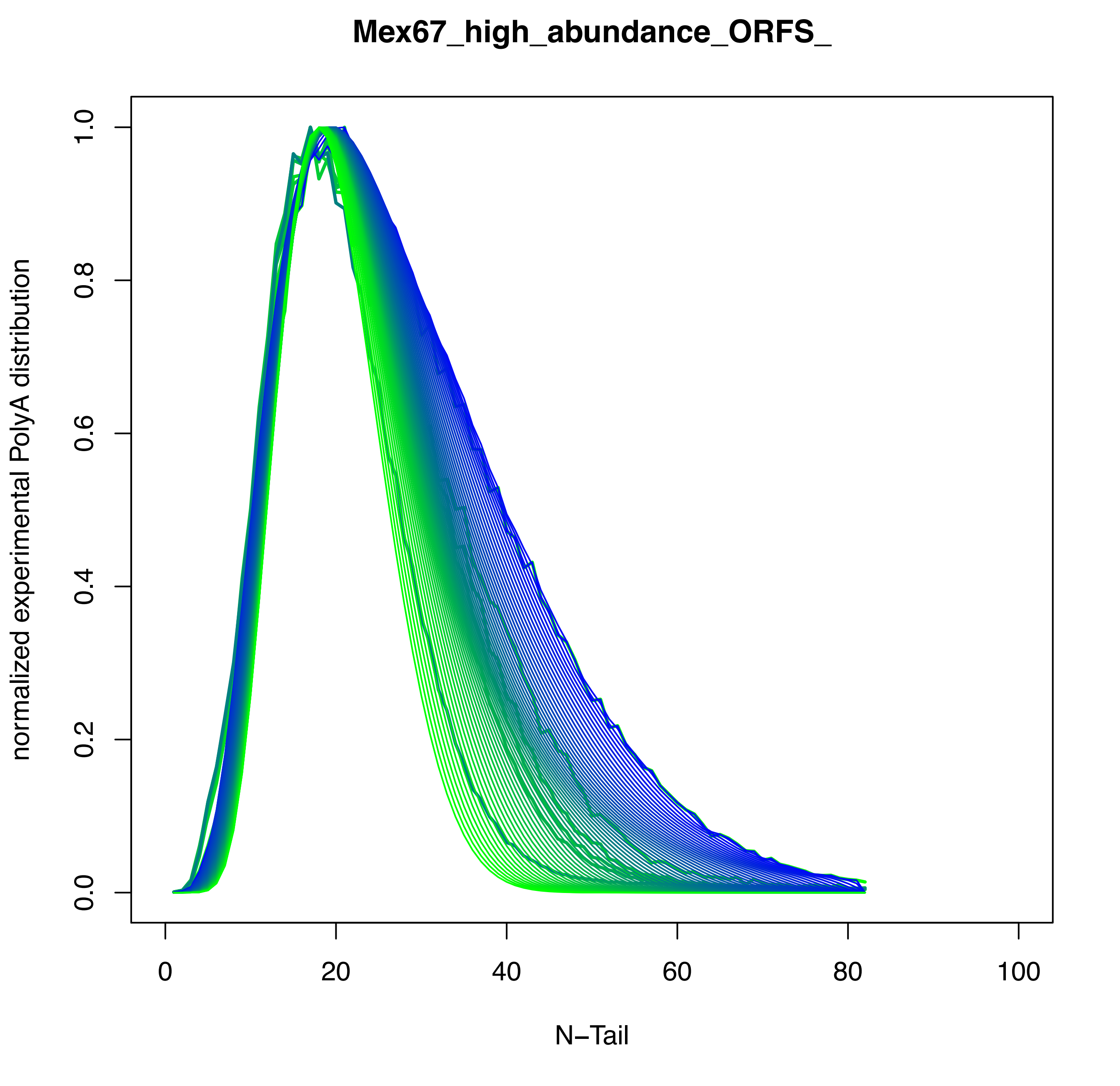
Mex67_high_abundance_ORFS_
normalized experimental PolyA distribution
0.0
0.2
0.4
0.6
0.8
1.0
0
20
40
60
80
100
N−Tail

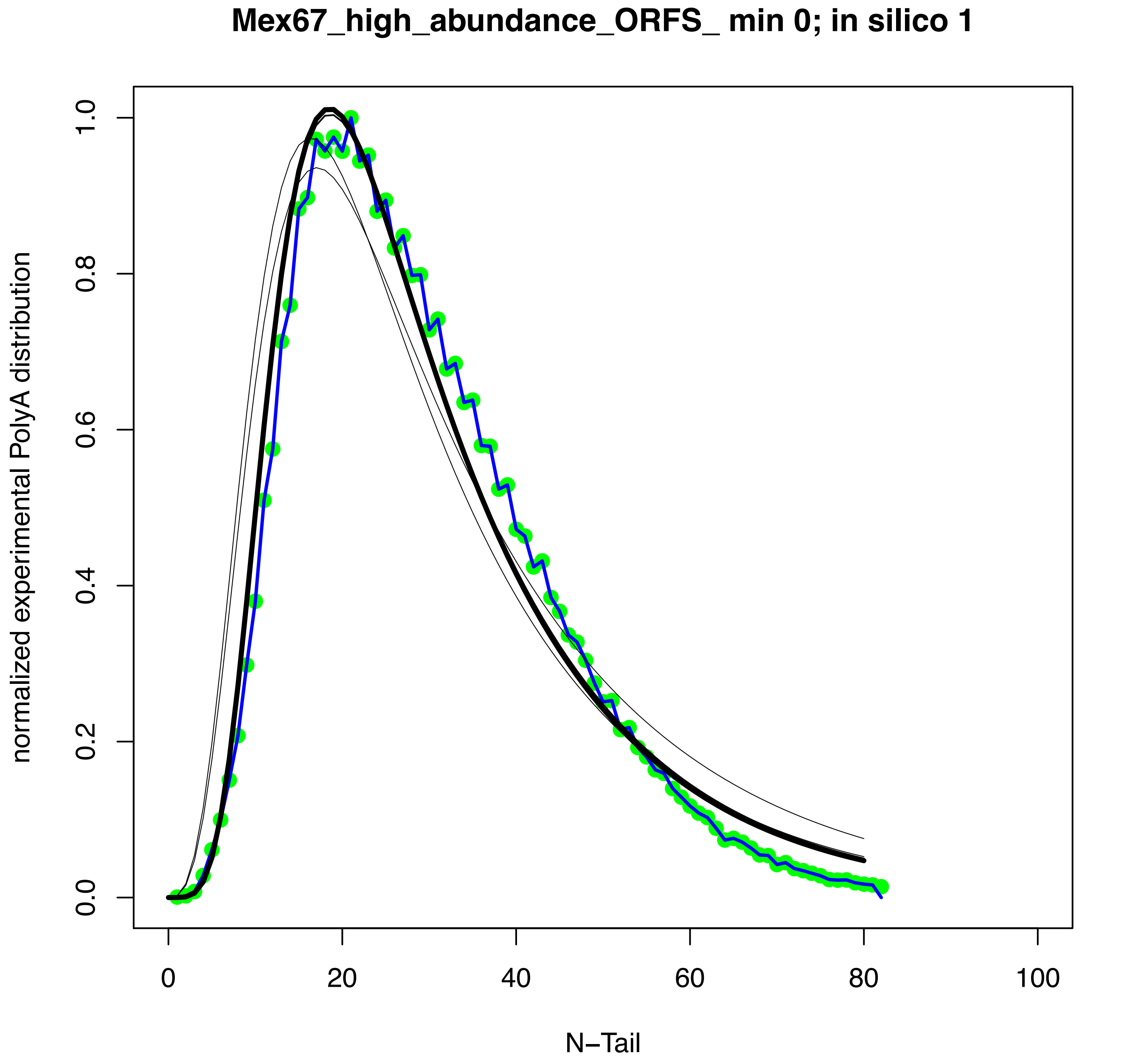
Mex67_high_abundance_ORFS_ min 0; in silico 1
normalized experimental PolyA distribution
N−Tail
0.0
0.2
0.4
0.6
0.8
1.0
0
20
40
60
80
100

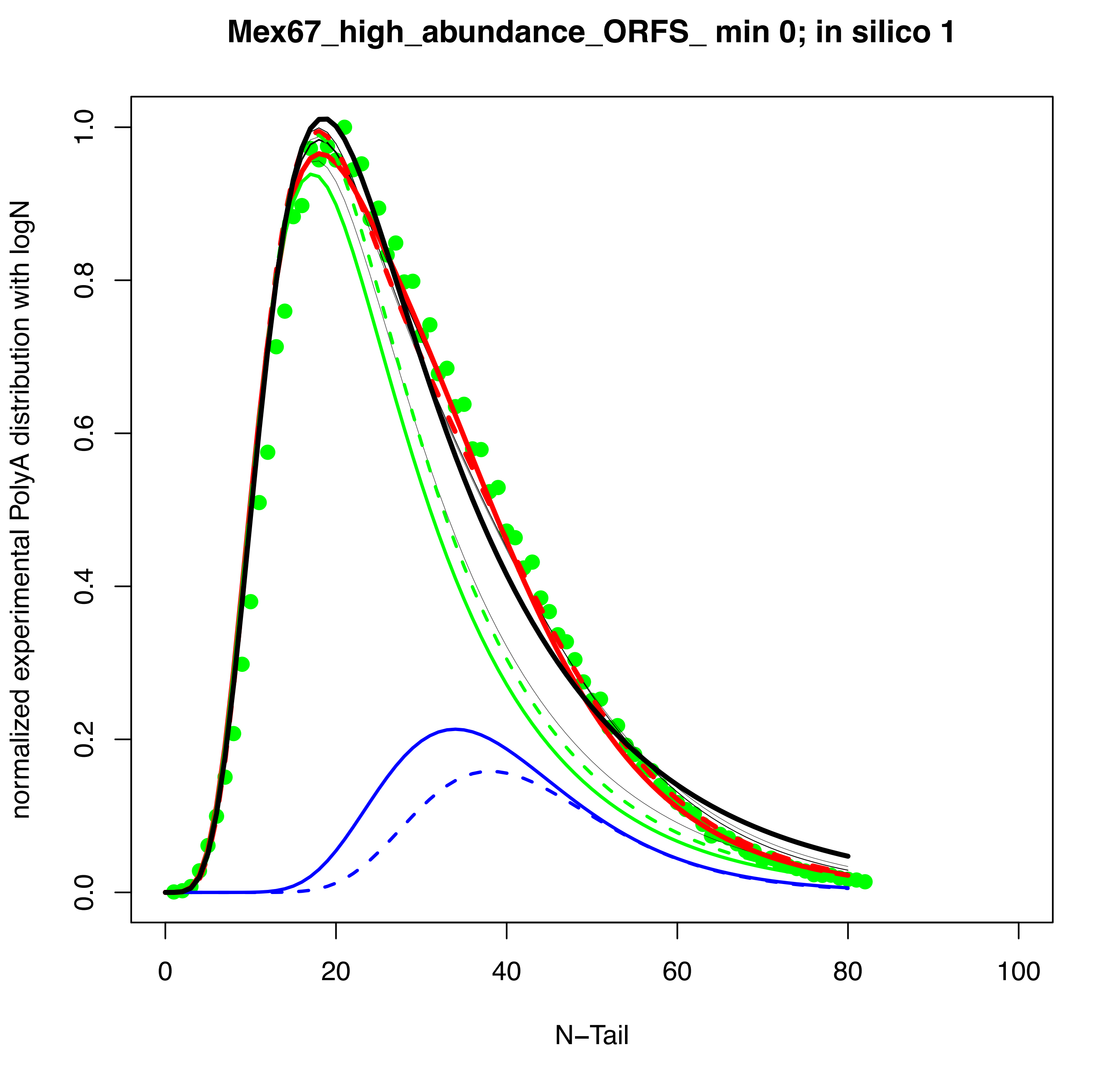

Mex67_high_abundance_ORFS_ min 0; in silico 1
normalized experimental PolyA distribution with logN
N−Tail
0.0
0.2
0.4
0.6
0.8
1.0
0
20
40
60
80
100

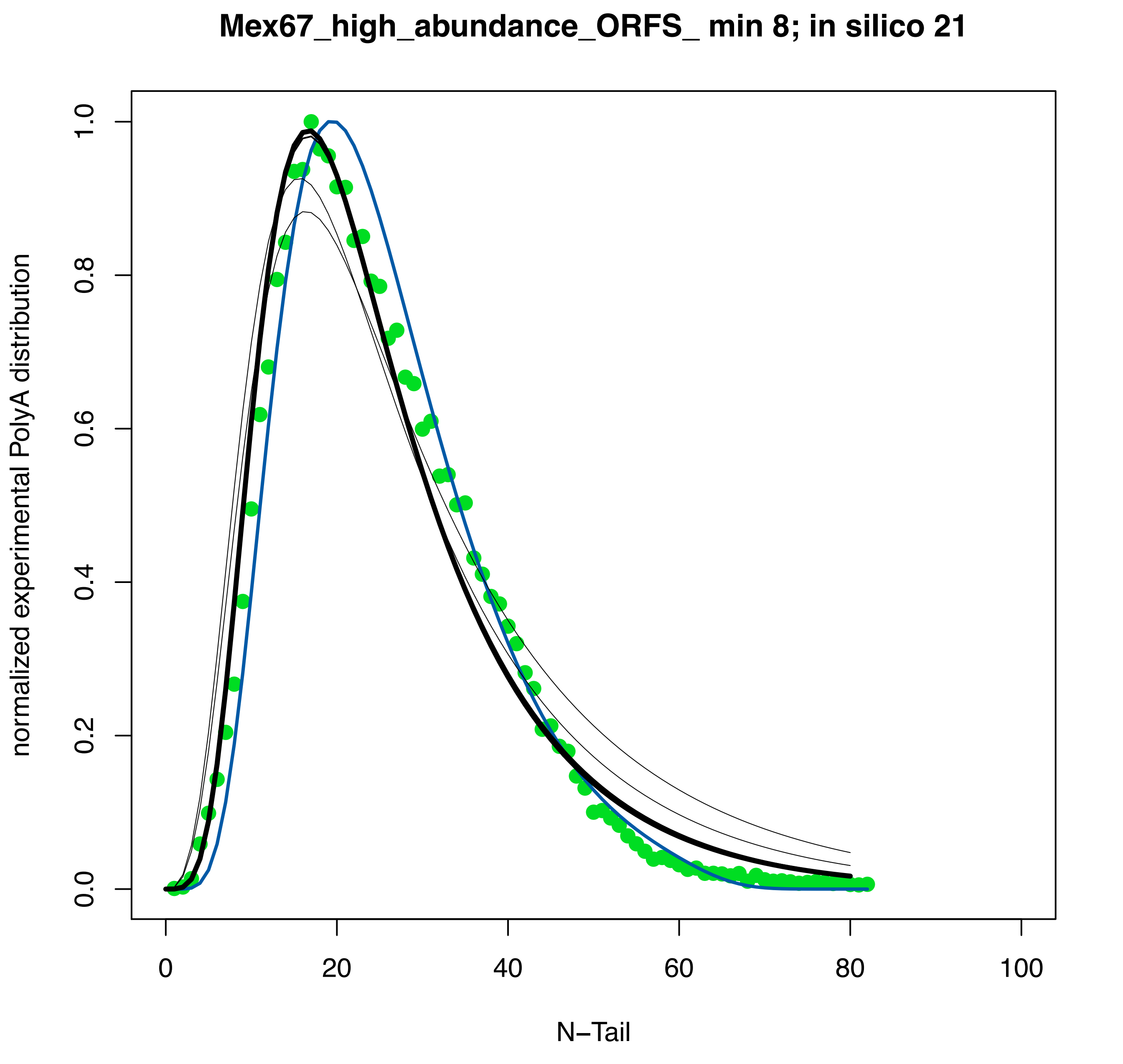

Mex67_high_abundance_ORFS_ min 8; in silico 21
normalized experimental PolyA distribution
0.0
0.2
0.4
0.6
0.8
1.0
0
20
40
60
80
100
N−Tail

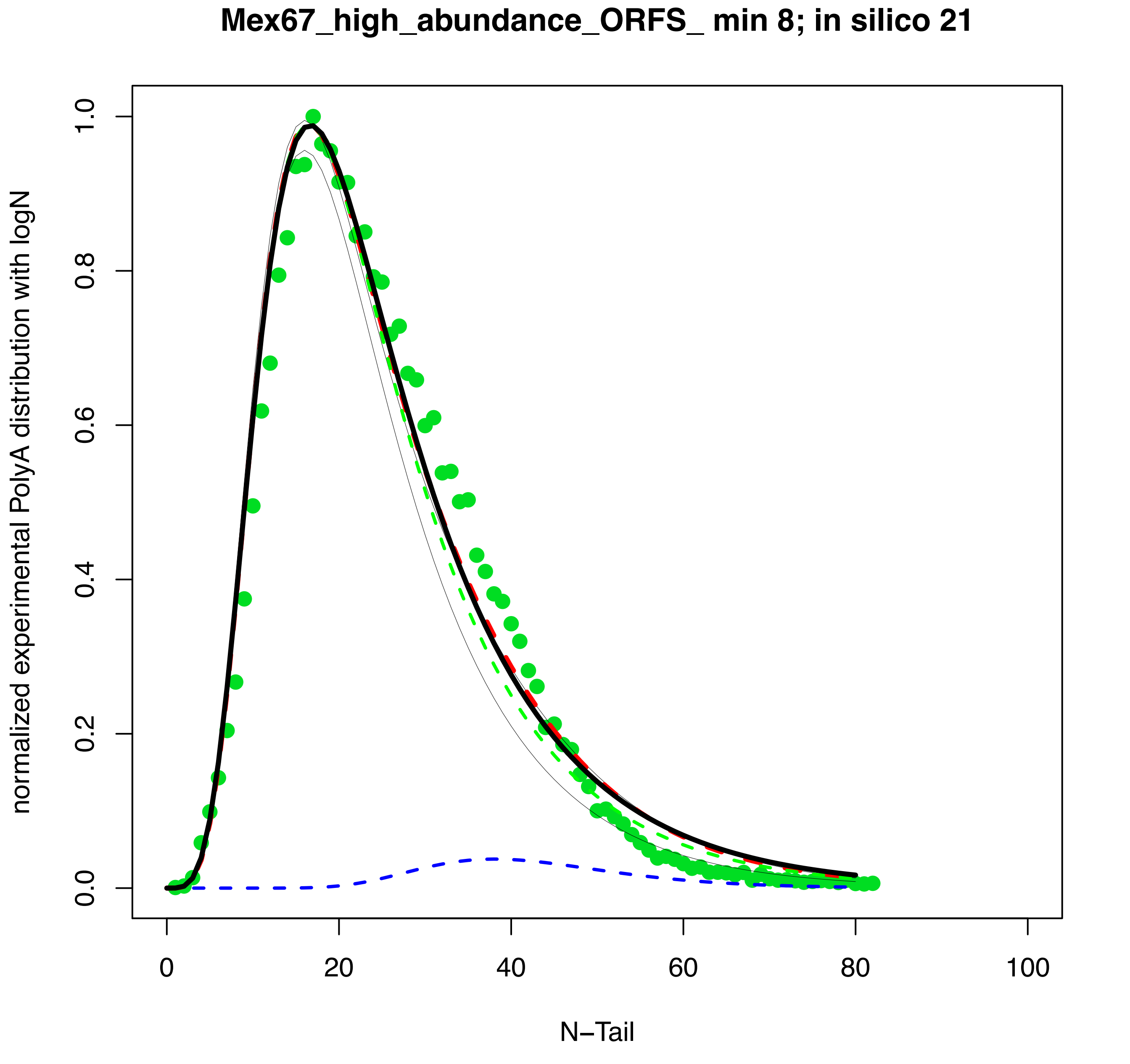

Mex67_high_abundance_ORFS_ min 8; in silico 21
normalized experimental PolyA distribution with logN
N−Tail
0.0
0.2
0.4
0.6
0.8
1.0
0
20
40
60
80
100

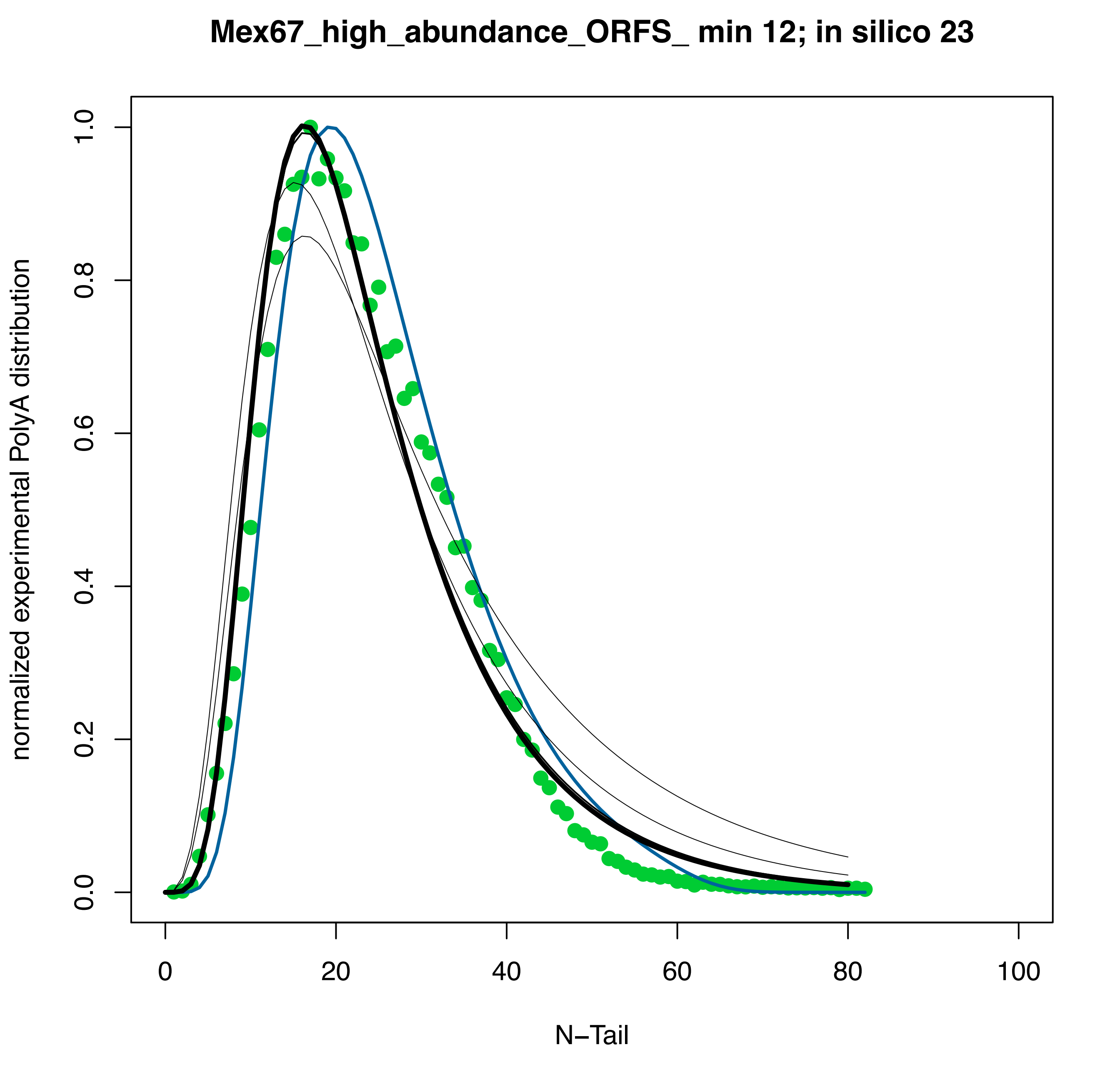

Mex67_high_abundance_ORFS_ min 12; in silico 23
normalized experimental PolyA distribution
N−Tail
0.0
0.2
0.4
0.6
0.8
1.0
0
20
40
60
80
100

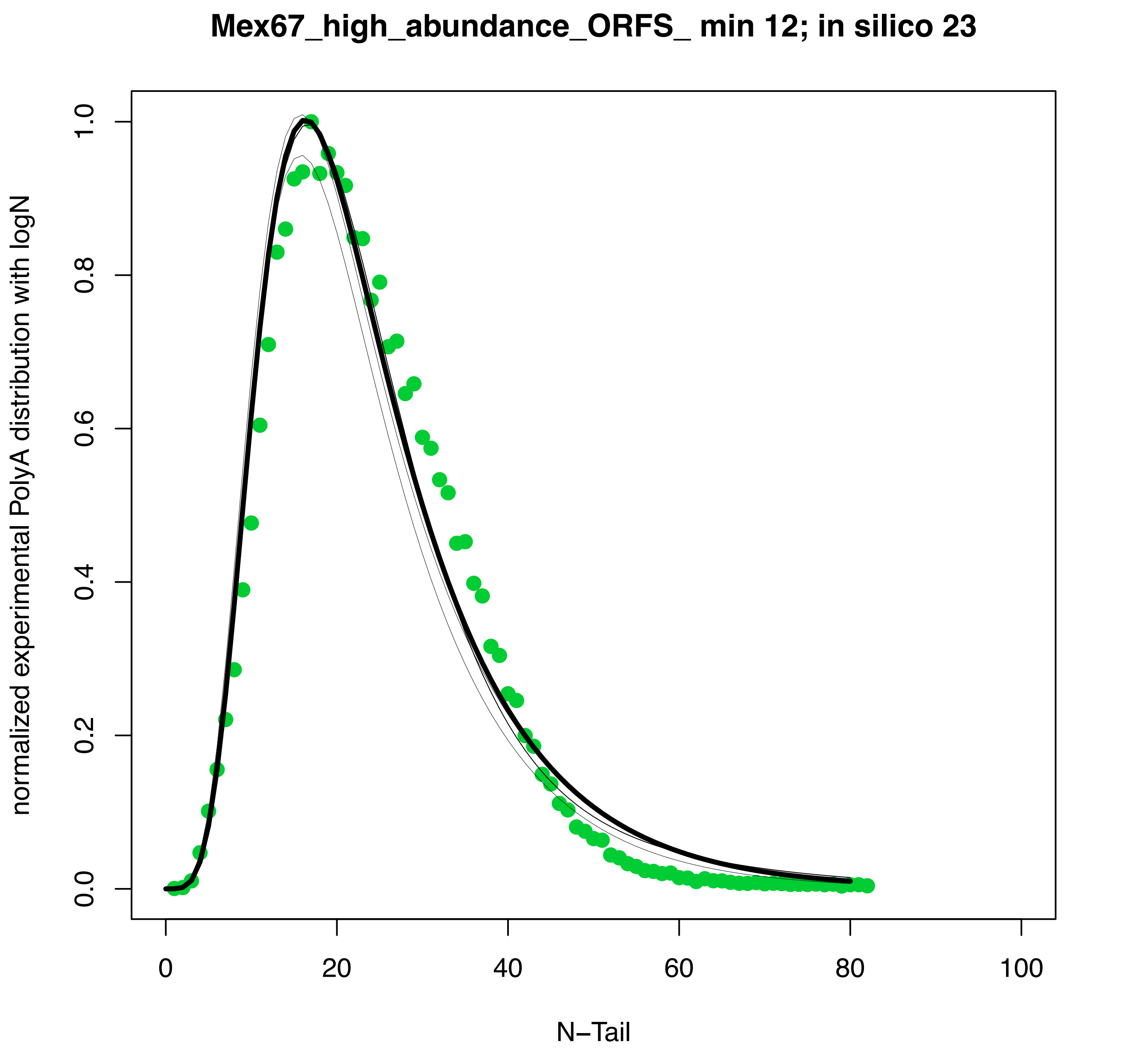

Mex67_high_abundance_ORFS_ min 12; in silico 23
normalized experimental PolyA distribution with logN
N−Tail
0.0
0.2
0.4
0.6
0.8
1.0
0
20
40
60
80
100

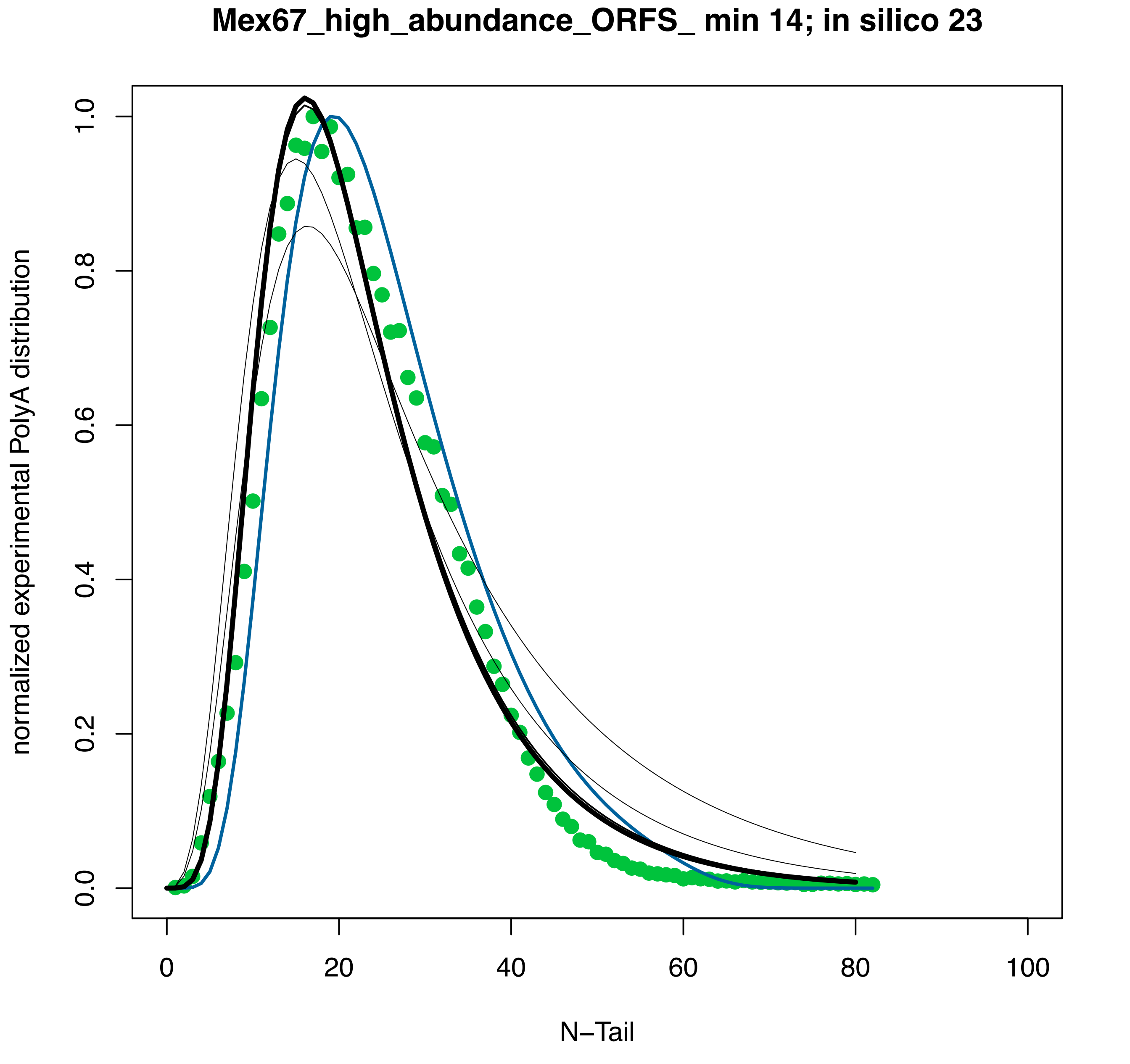

Mex67_high_abundance_ORFS_ min 14; in silico 23
normalized experimental PolyA distribution
N−Tail
0.0
0.2
0.4
0.6
0.8
1.0
0
20
40
60
80
100

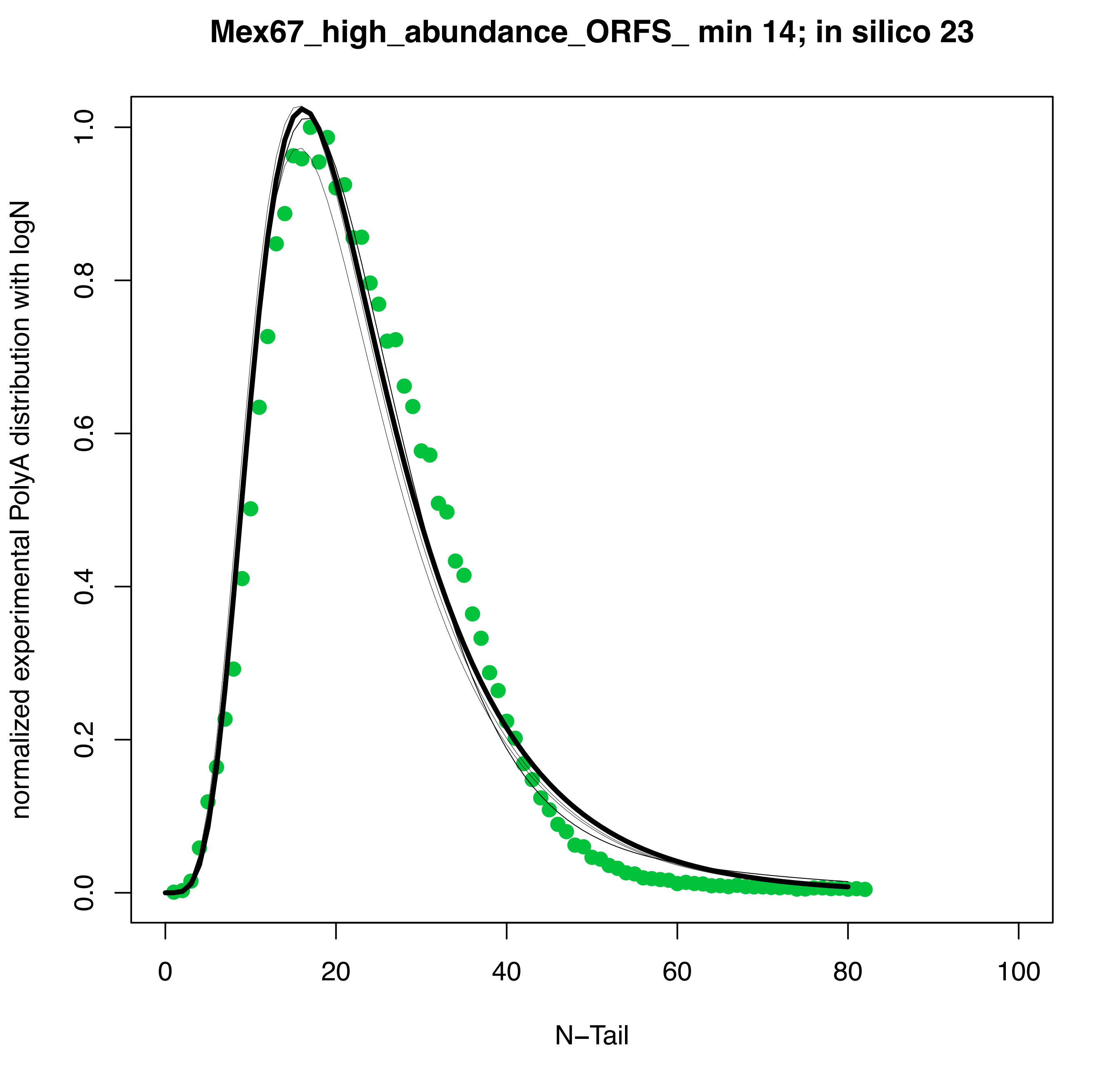
Mex67_high_abundance_ORFS_ min 14; in silico 23
normalized experimental PolyA distribution with logN
N−Tail
0.0
0.2
0.4
0.6
0.8
1.0
0
20
40
60
80
100

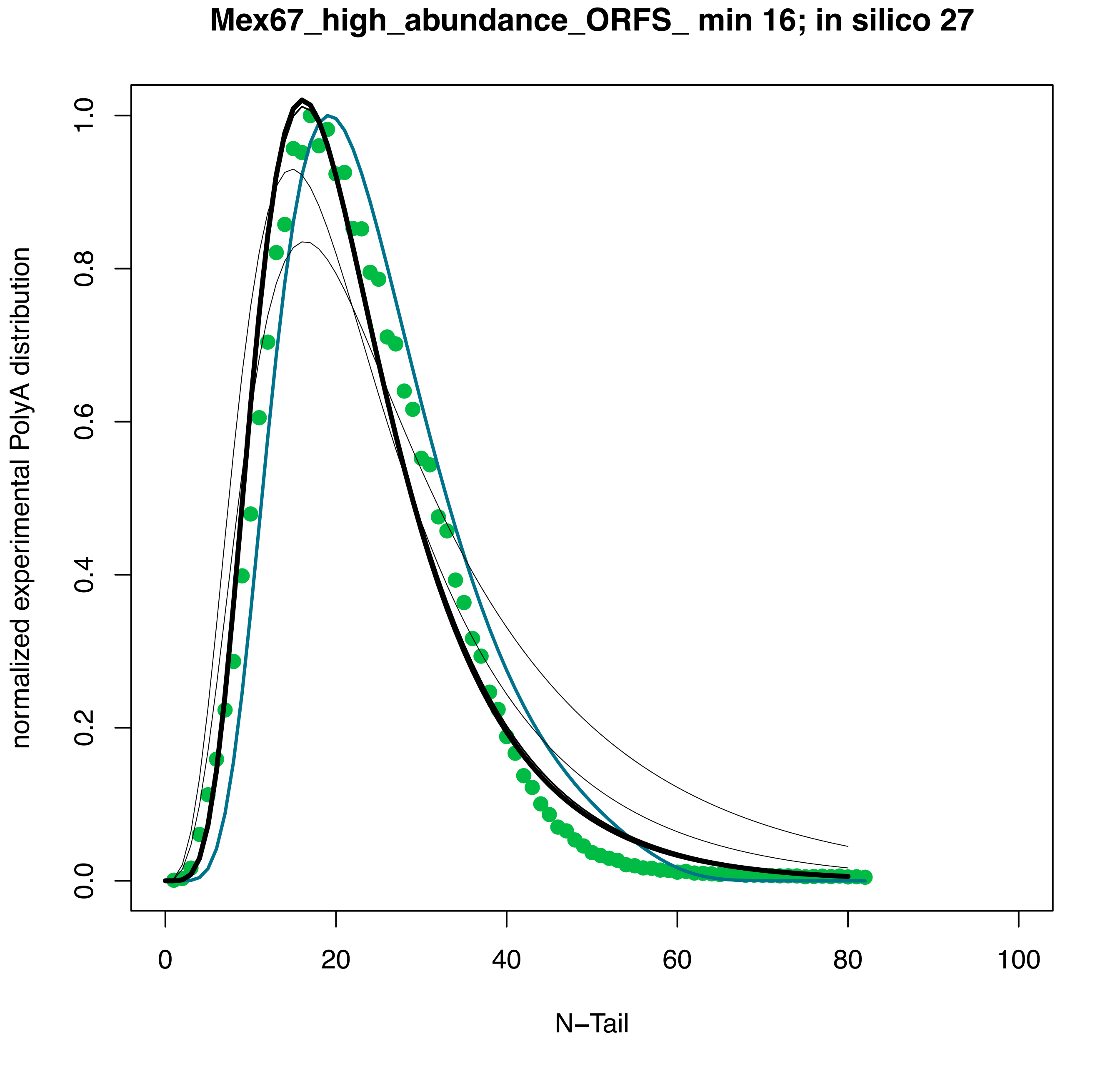
Mex67_high_abundance_ORFS_ min 16; in silico 27
normalized experimental PolyA distribution
N−Tail
0.0
0.2
0.4
0.6
0.8
1.0
0
20
40
60
80
100

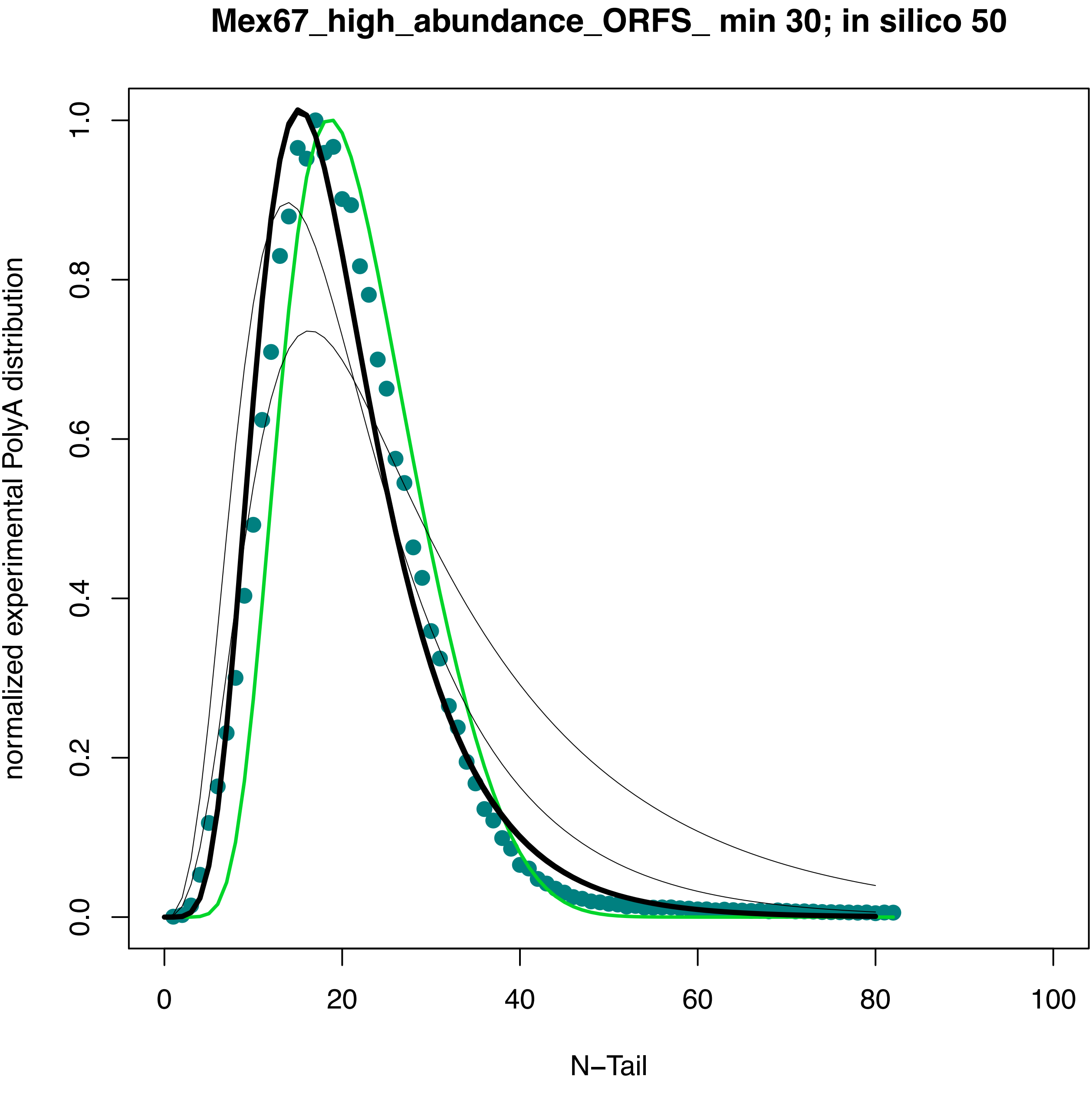

Mex67_high_abundance_ORFS_ min 30; in silico 50
normalized experimental PolyA distribution
N−Tail
0.0
0.2
0.4
0.6
0.8
1.0
0
20
40
60
80
100

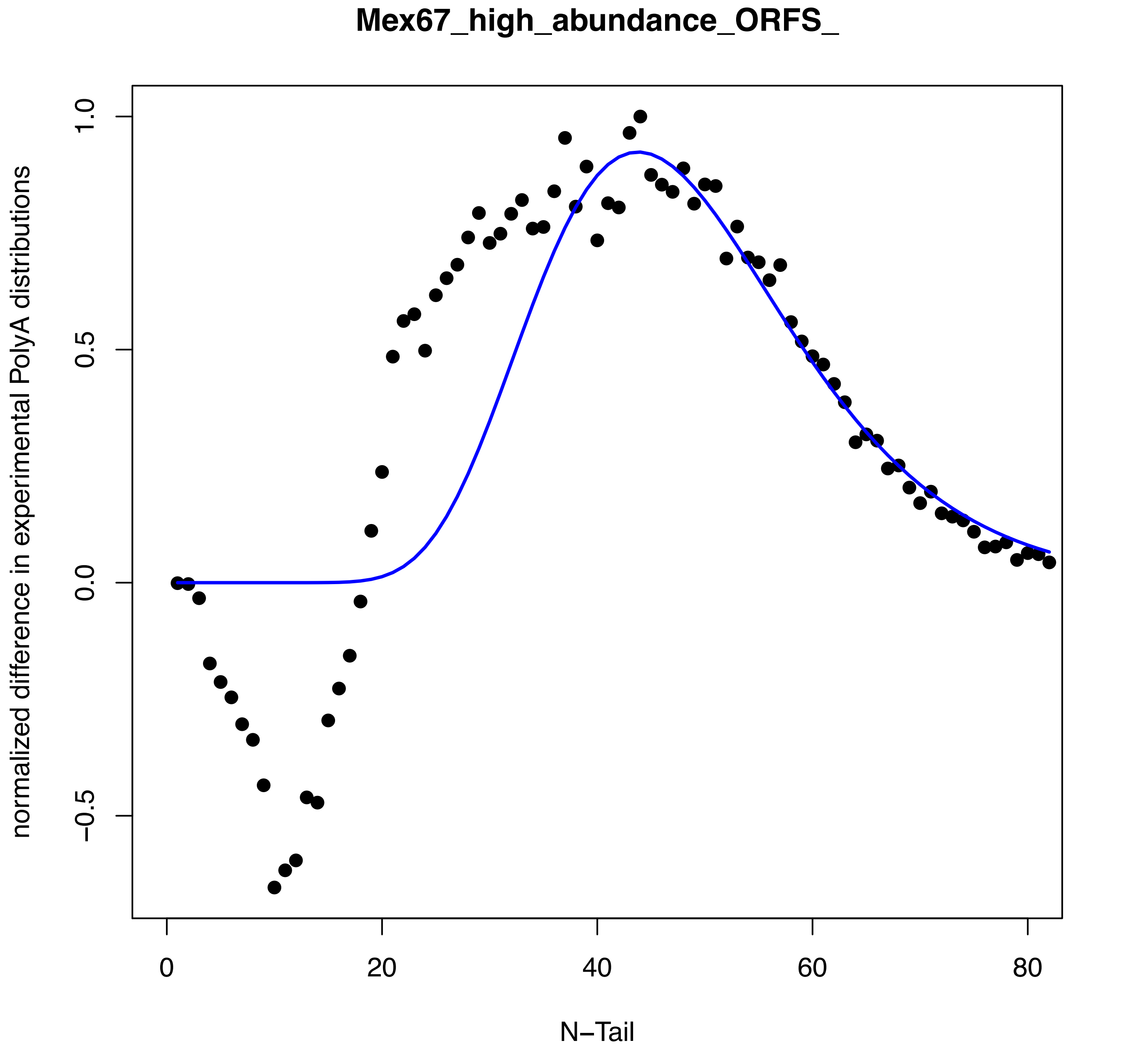
Mex67_high_abundance_ORFS_
normalized difference in experimental PolyA distributions
N−Tail
1.0
0.5
0.0
−0.5
0
20
40
60
80

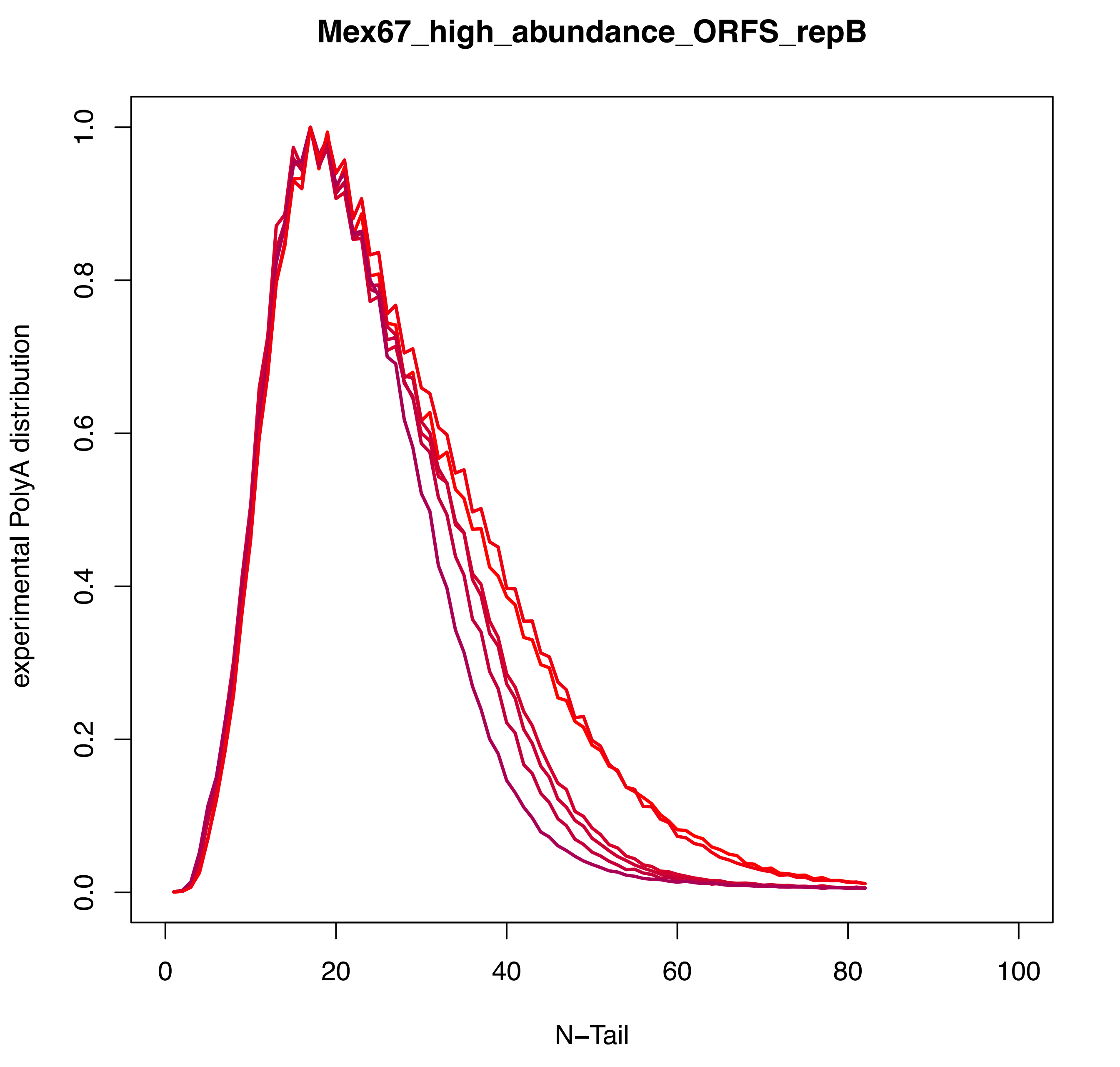
Mex67_high_abundance_ORFS_repB
experimental PolyA distribution
N−Tail
0.0
0.2
0.4
0.6
0.8
1.0
0
20
40
60
80
100

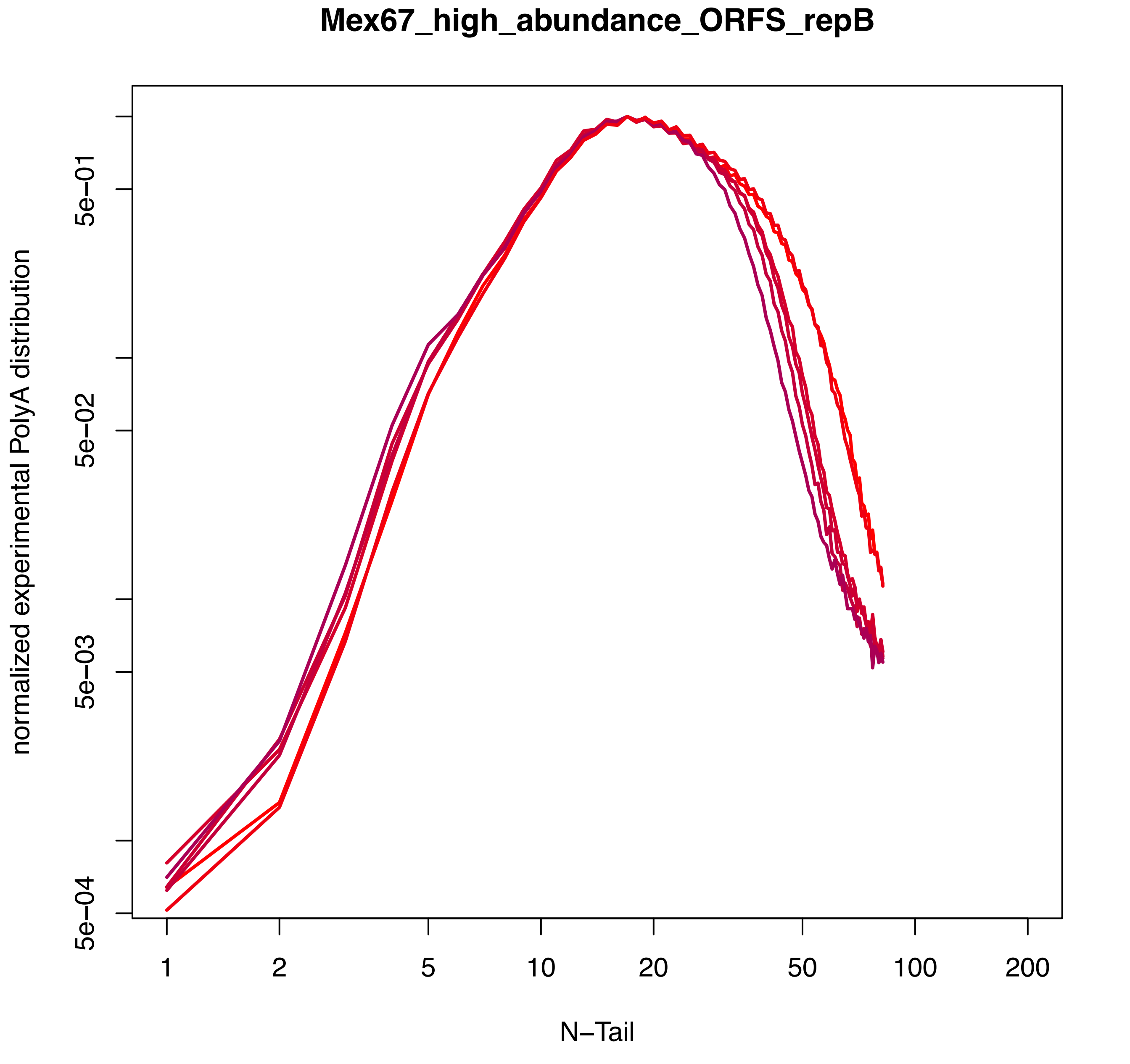
Mex67_high_abundance_ORFS_repB
normalized experimental PolyA distribution
5e−01
5e−02
5e−03
5e−04
1
2
5
10
20
50
100
200
N−Tail

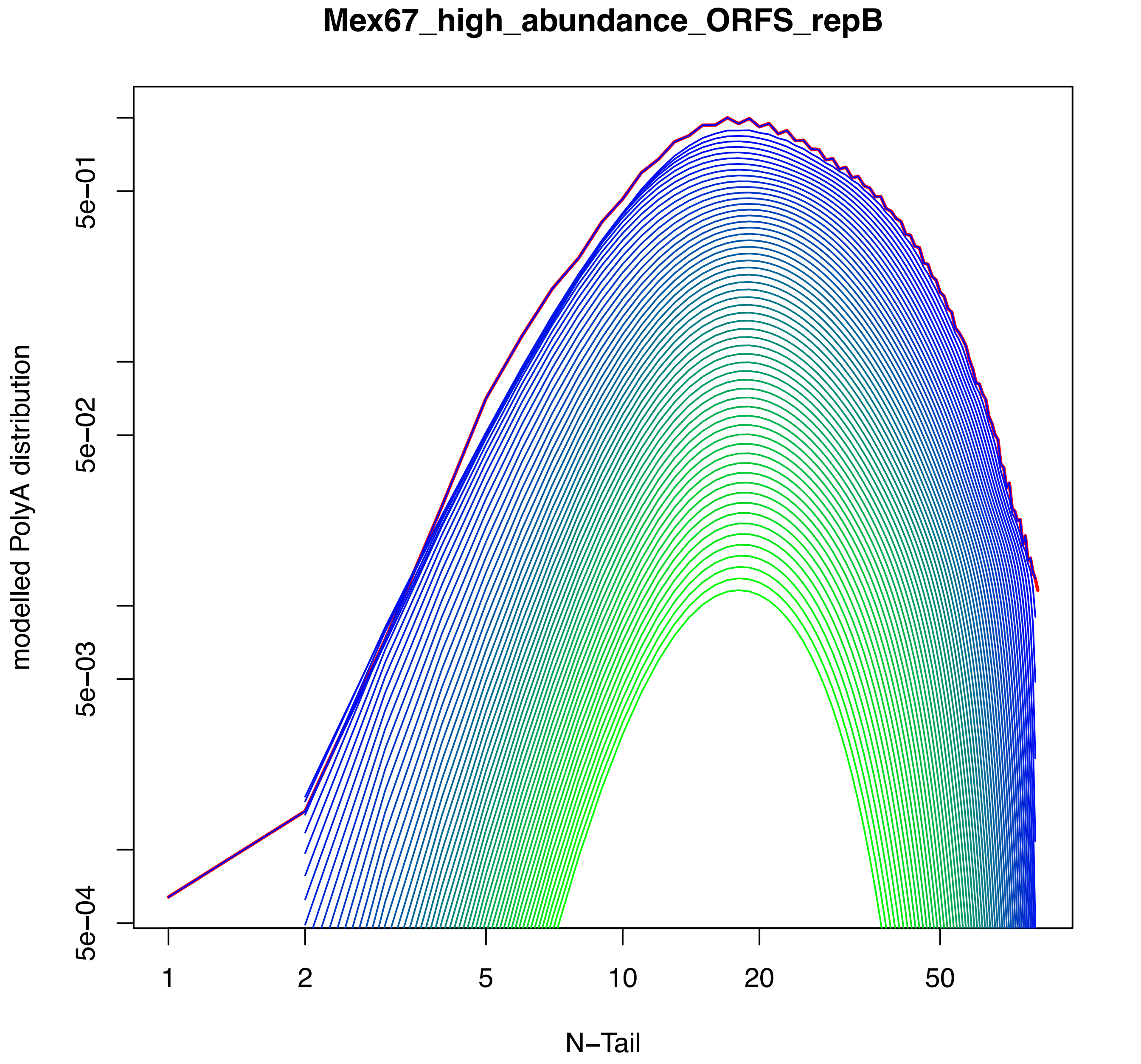
Mex67_high_abundance_ORFS_repB
modelled PolyA distribution
N−Tail
5e−01
5e−02
5e−03
5e−04
1
2
5
10
20
50

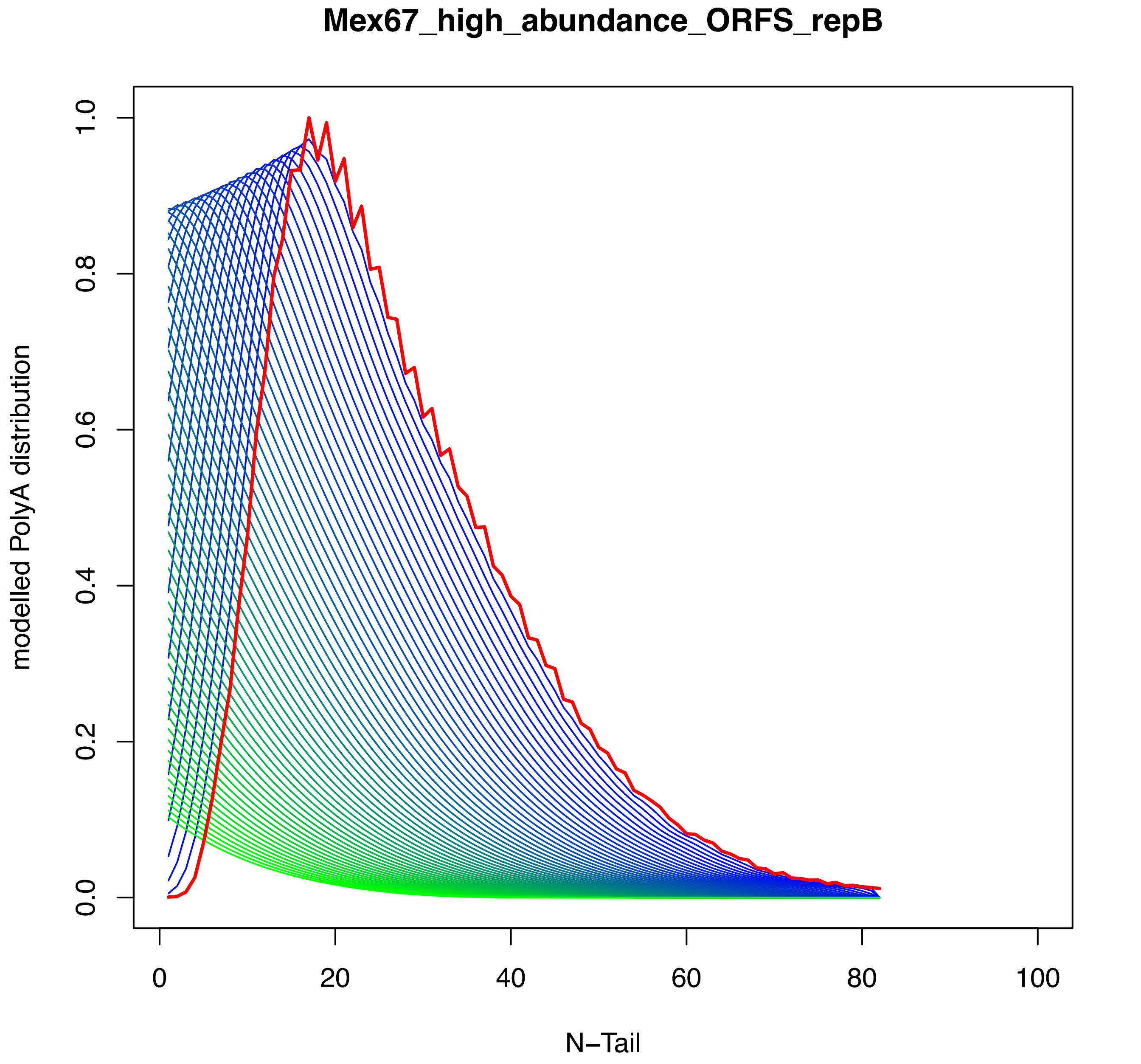
Mex67_high_abundance_ORFS_repB
modelled PolyA distribution
N−Tail
0.0
0.2
0.4
0.6
0.8
1.0
0
20
40
60
80
100

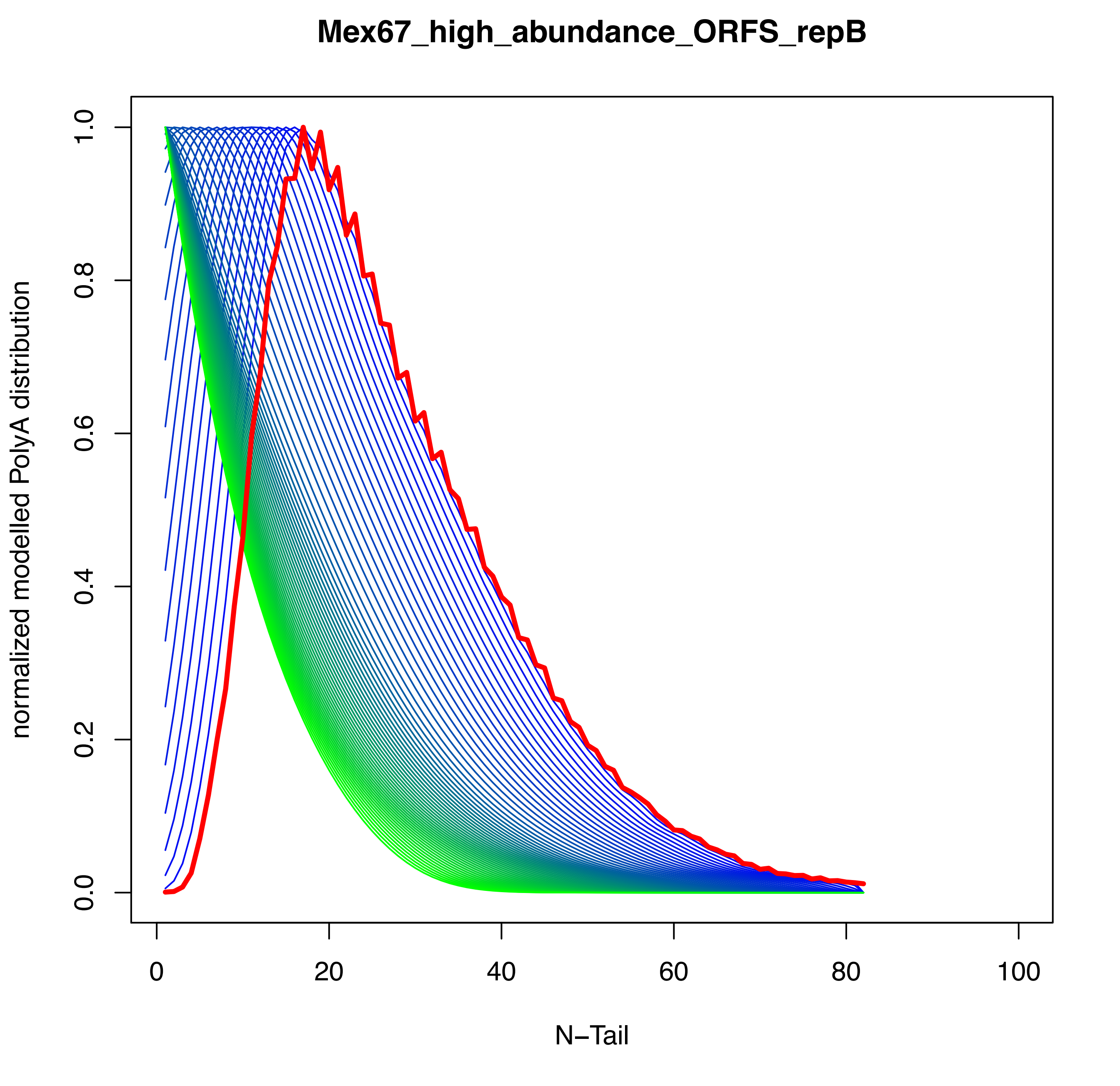
Mex67_high_abundance_ORFS_repB
normalized modelled PolyA distribution
0.0
0.2
0.4
0.6
0.8
1.0
0
20
40
60
80
100
N−Tail

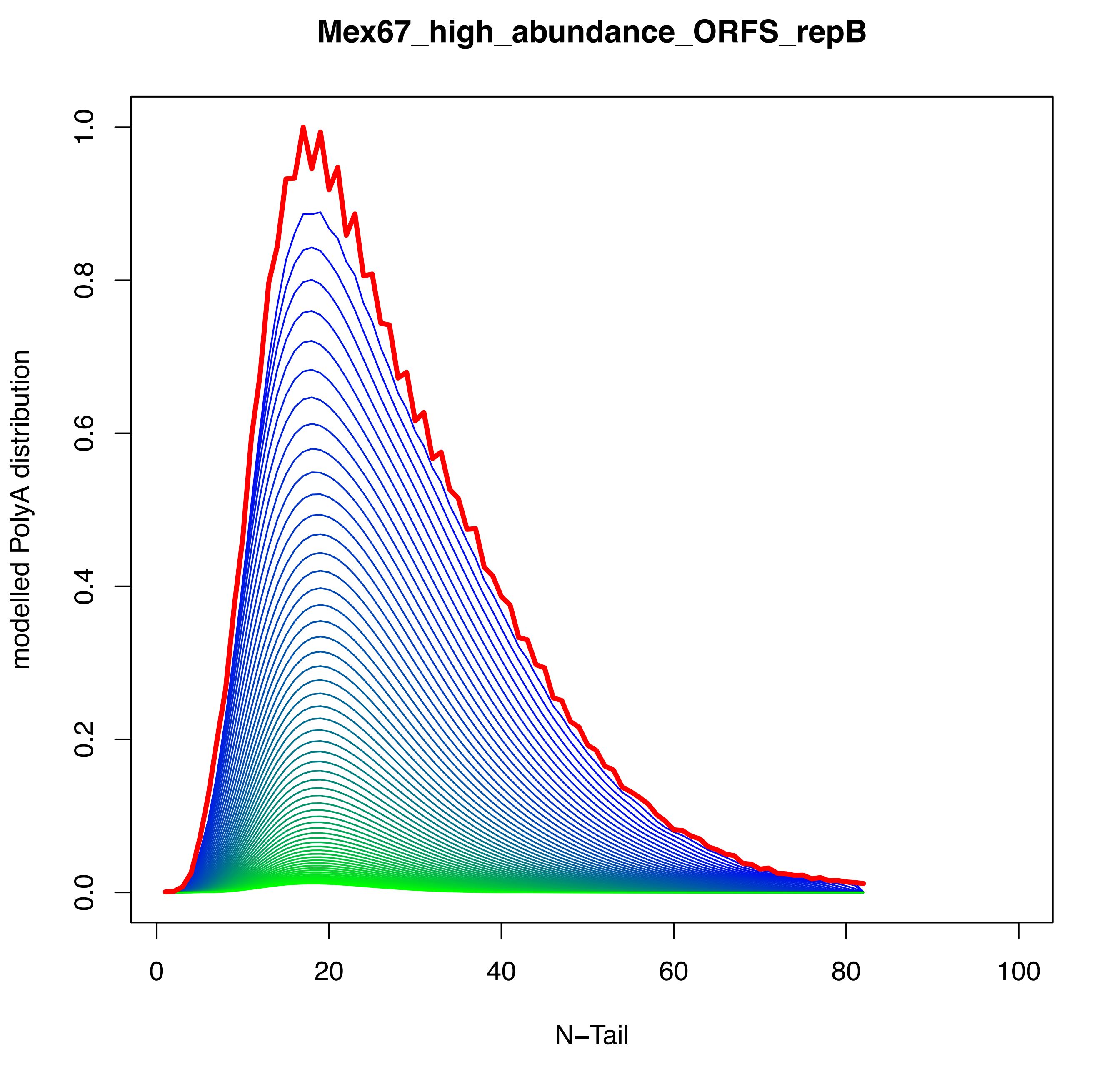
Mex67_high_abundance_ORFS_repB
modelled PolyA distribution
N−Tail
0.0
0.2
0.4
0.6
0.8
1.0
0
20
40
60
80
100

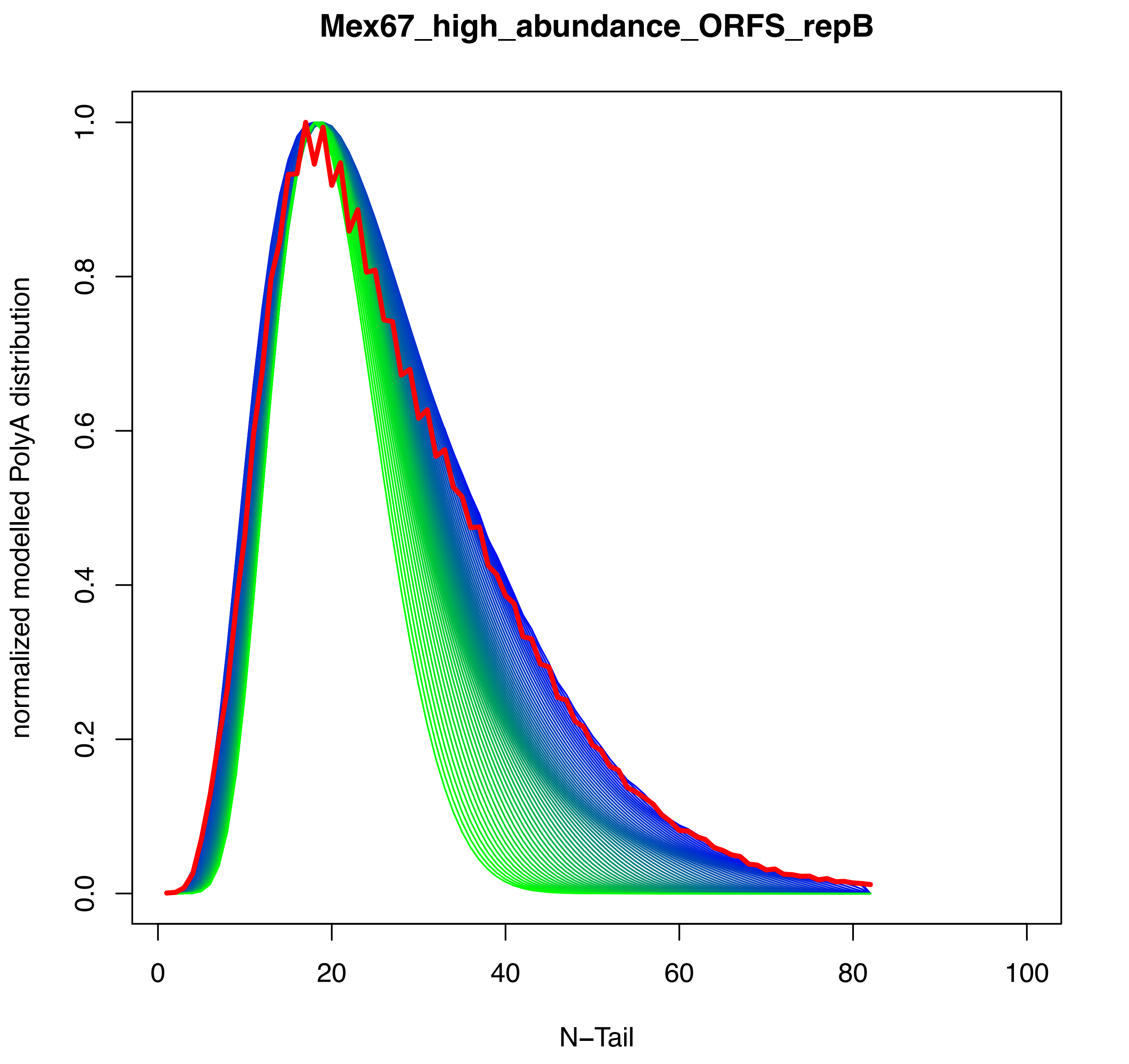
Mex67_high_abundance_ORFS_repB
normalized modelled PolyA distribution
N−Tail
0.0
0.2
0.4
0.6
0.8
1.0
0
20
40
60
80
100

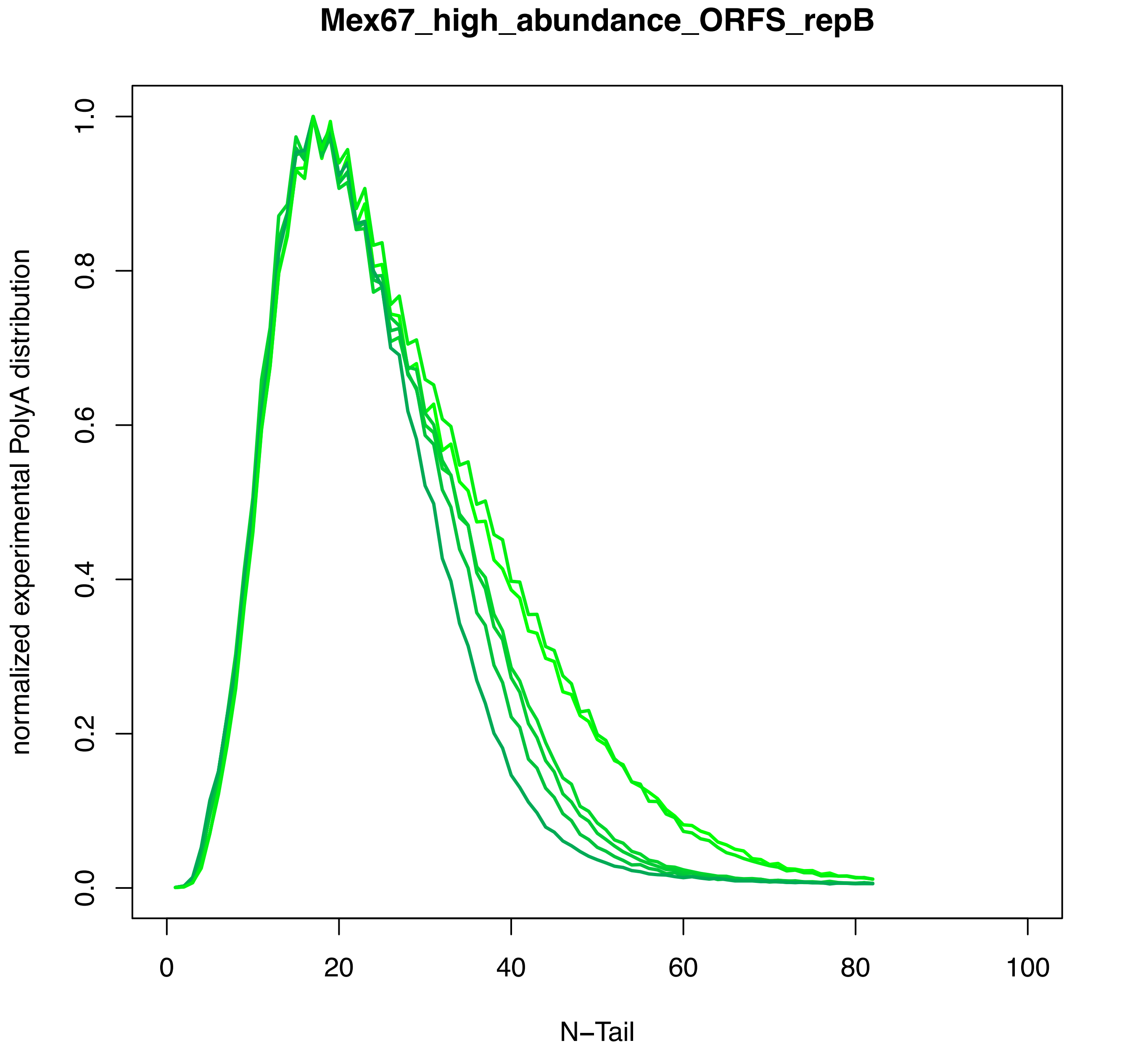
Mex67_high_abundance_ORFS_repB
normalized experimental PolyA distribution
0.0
0.2
0.4
0.6
0.8
1.0
0
20
40
60
80
100
N−Tail

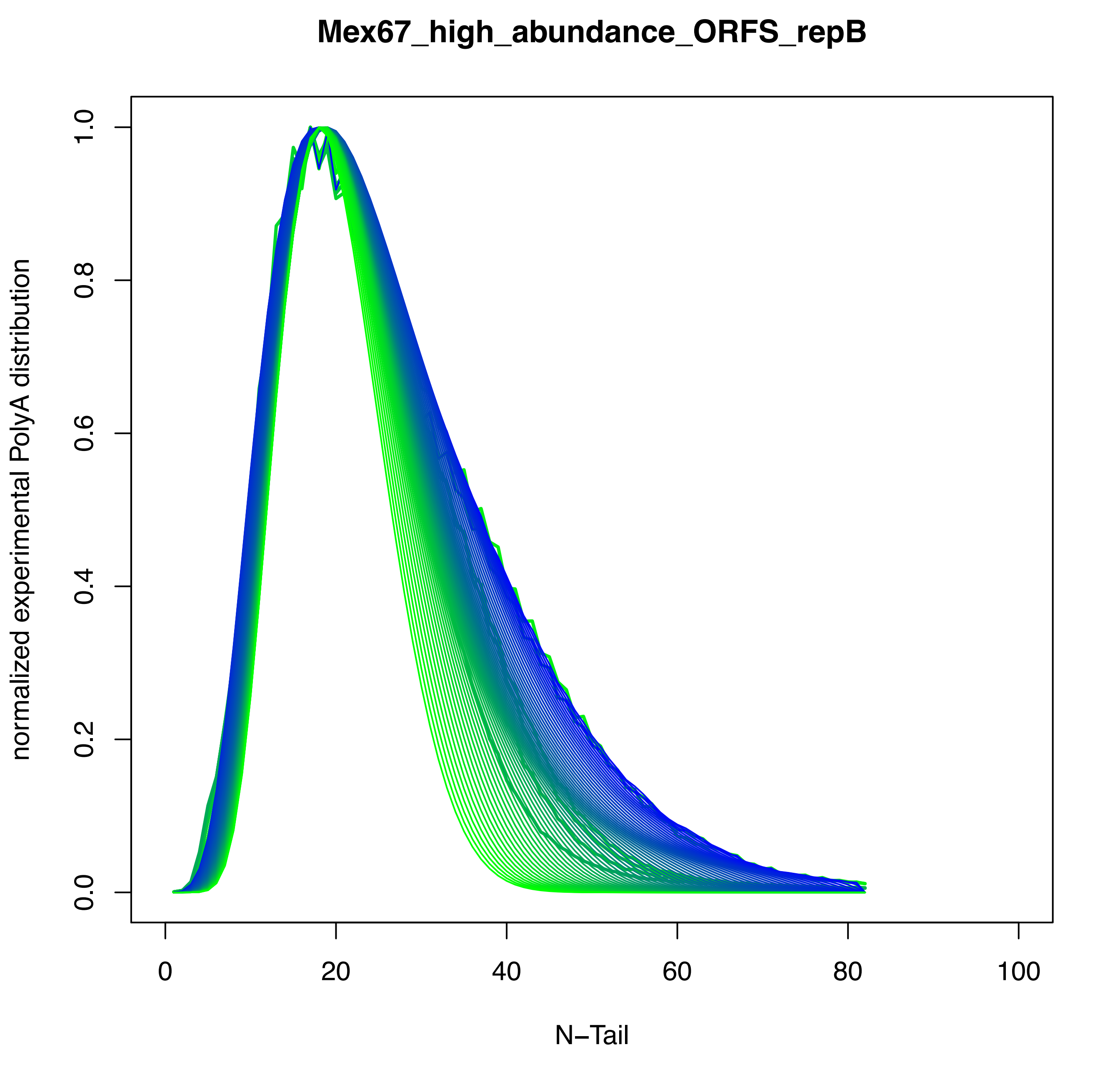
Mex67_high_abundance_ORFS_repB
normalized experimental PolyA distribution
N−Tail
0.0
0.2
0.4
0.6
0.8
1.0
0
20
40
60
80
100

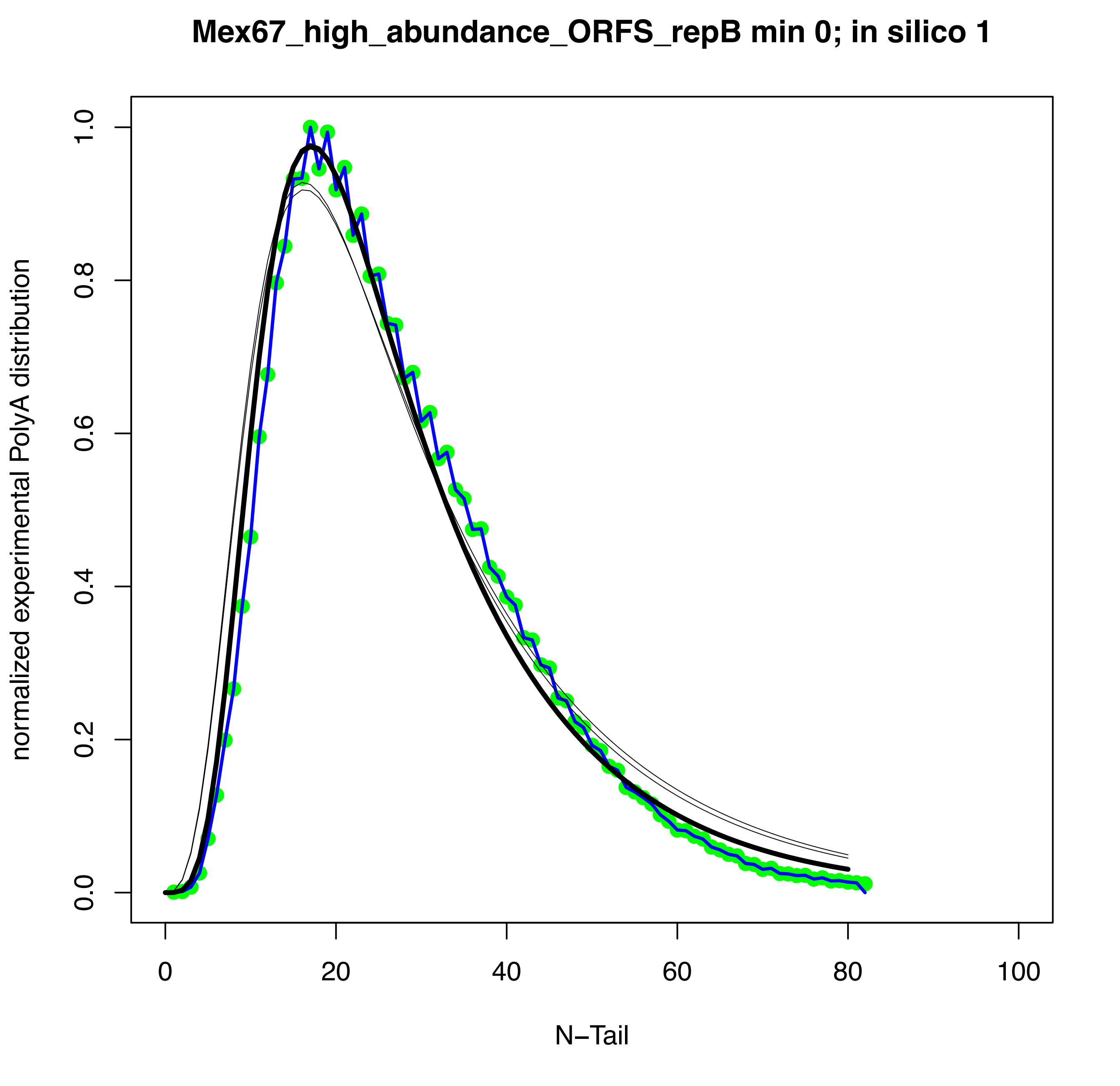
Mex67_high_abundance_ORFS_repB min 0; in silico 1
normalized experimental PolyA distribution
N−Tail
0.0
0.2
0.4
0.6
0.8
1.0
0
20
40
60
80
100

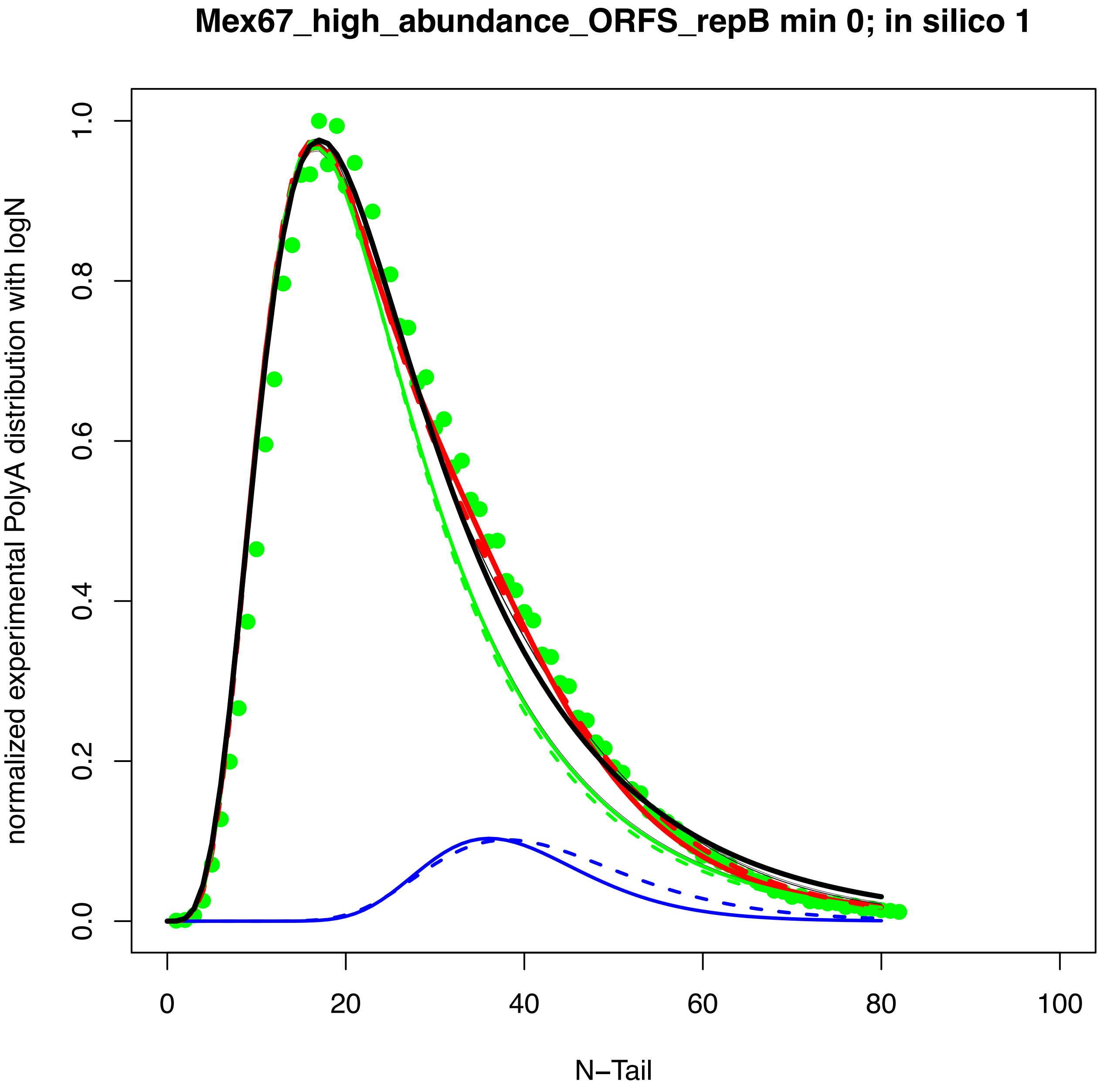
Mex67_high_abundance_ORFS_repB min 0; in silico 1
normalized experimental PolyA distribution with logN
N−Tail
0.0
0.2
0.4
0.6
0.8
1.0
0
20
40
60
80
100

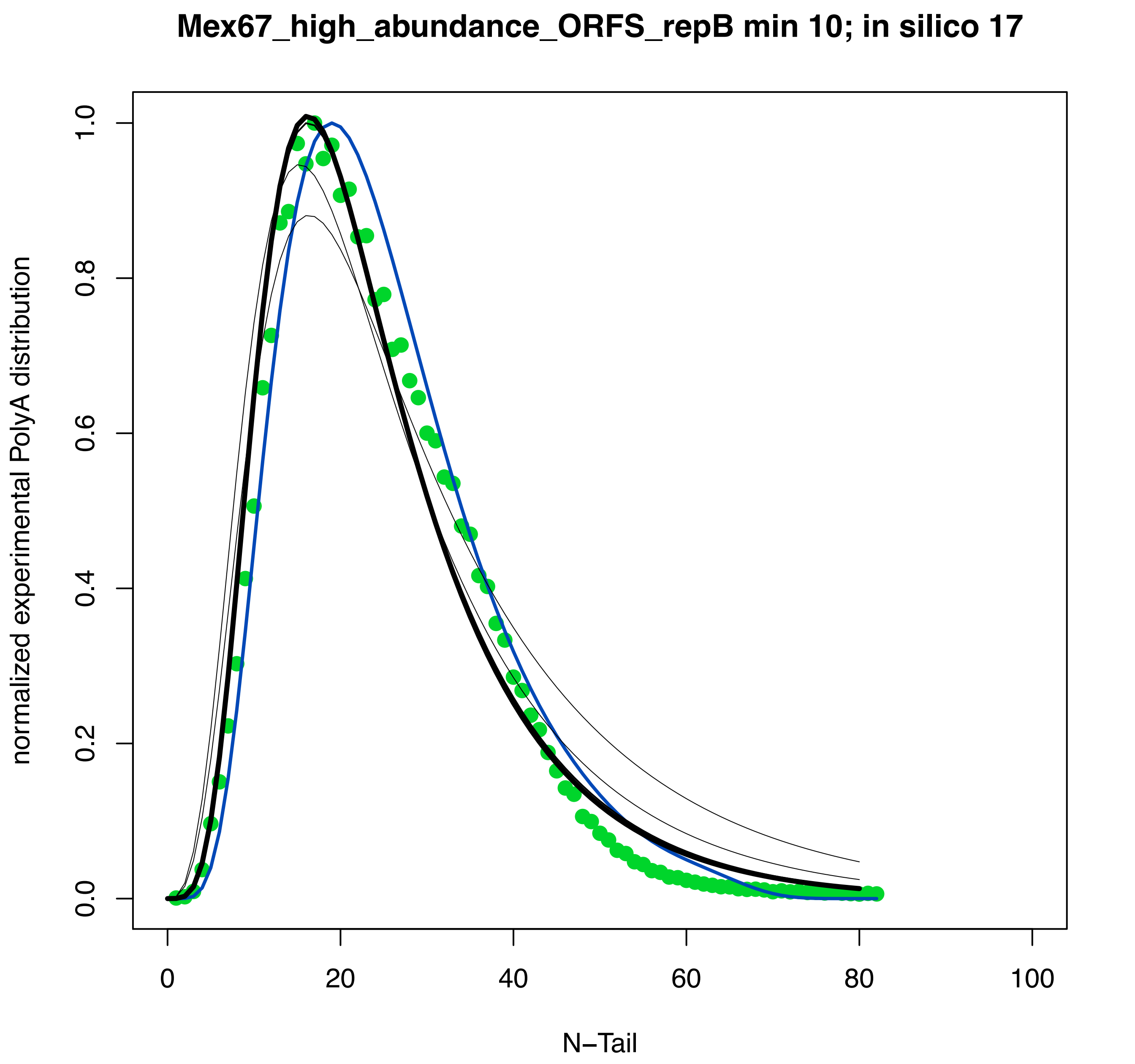
Mex67_high_abundance_ORFS_repB min 10; in silico 17
normalized experimental PolyA distribution
N−Tail
0.0
0.2
0.4
0.6
0.8
1.0
0
20
40
60
80
100

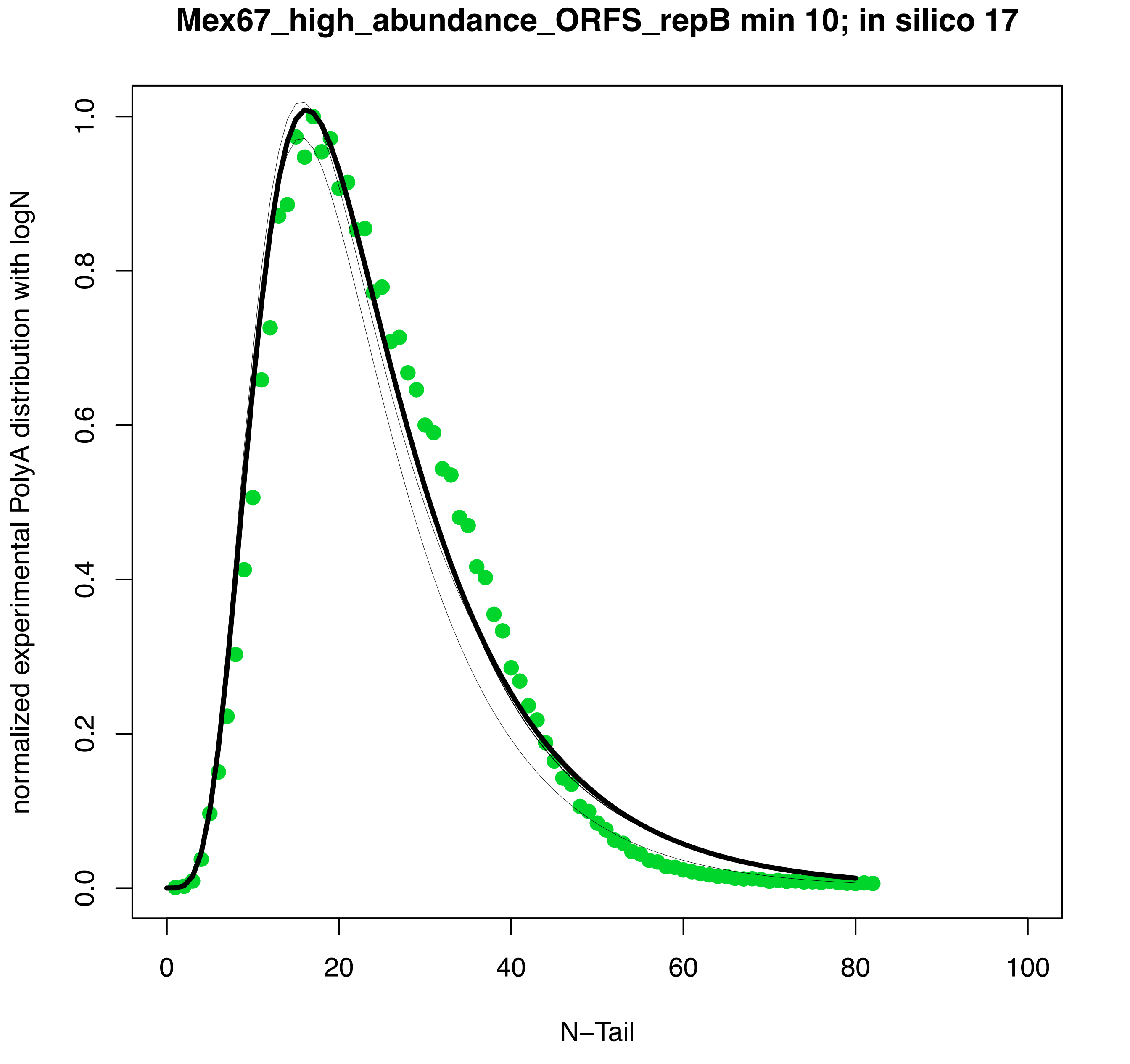

Mex67_high_abundance_ORFS_repB min 10; in silico 17
normalized experimental PolyA distribution with logN
N−Tail
0.0
0.2
0.4
0.6
0.8
1.0
0
20
40
60
80
100

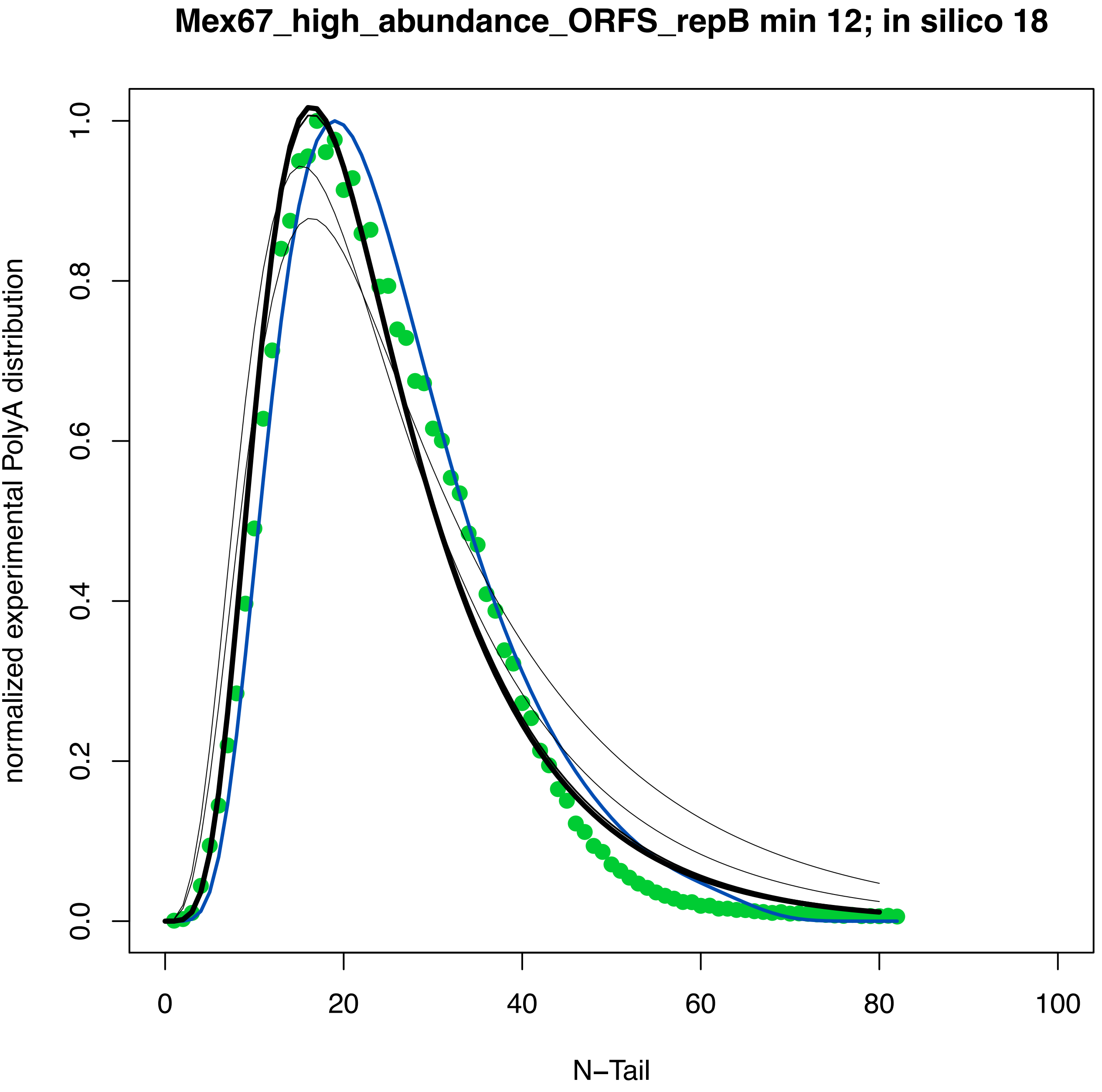

Mex67_high_abundance_ORFS_repB min 12; in silico 18
normalized experimental PolyA distribution
N−Tail
0.0
0.2
0.4
0.6
0.8
1.0
0
20
40
60
80
100

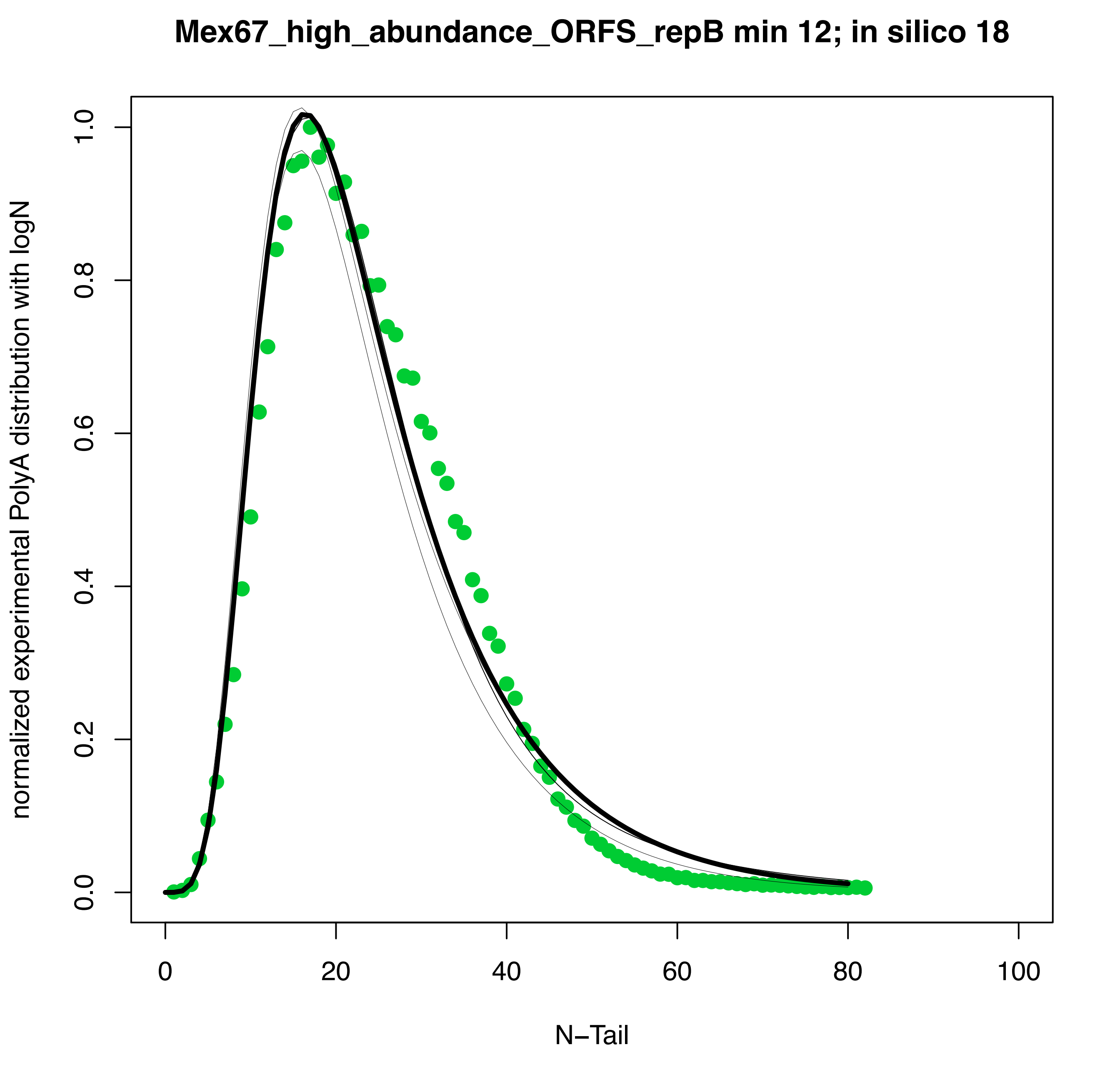
Mex67_high_abundance_ORFS_repB min 12; in silico 18
normalized experimental PolyA distribution with logN
N−Tail
0.0
0.2
0.4
0.6
0.8
1.0
0
20
40
60
80
100

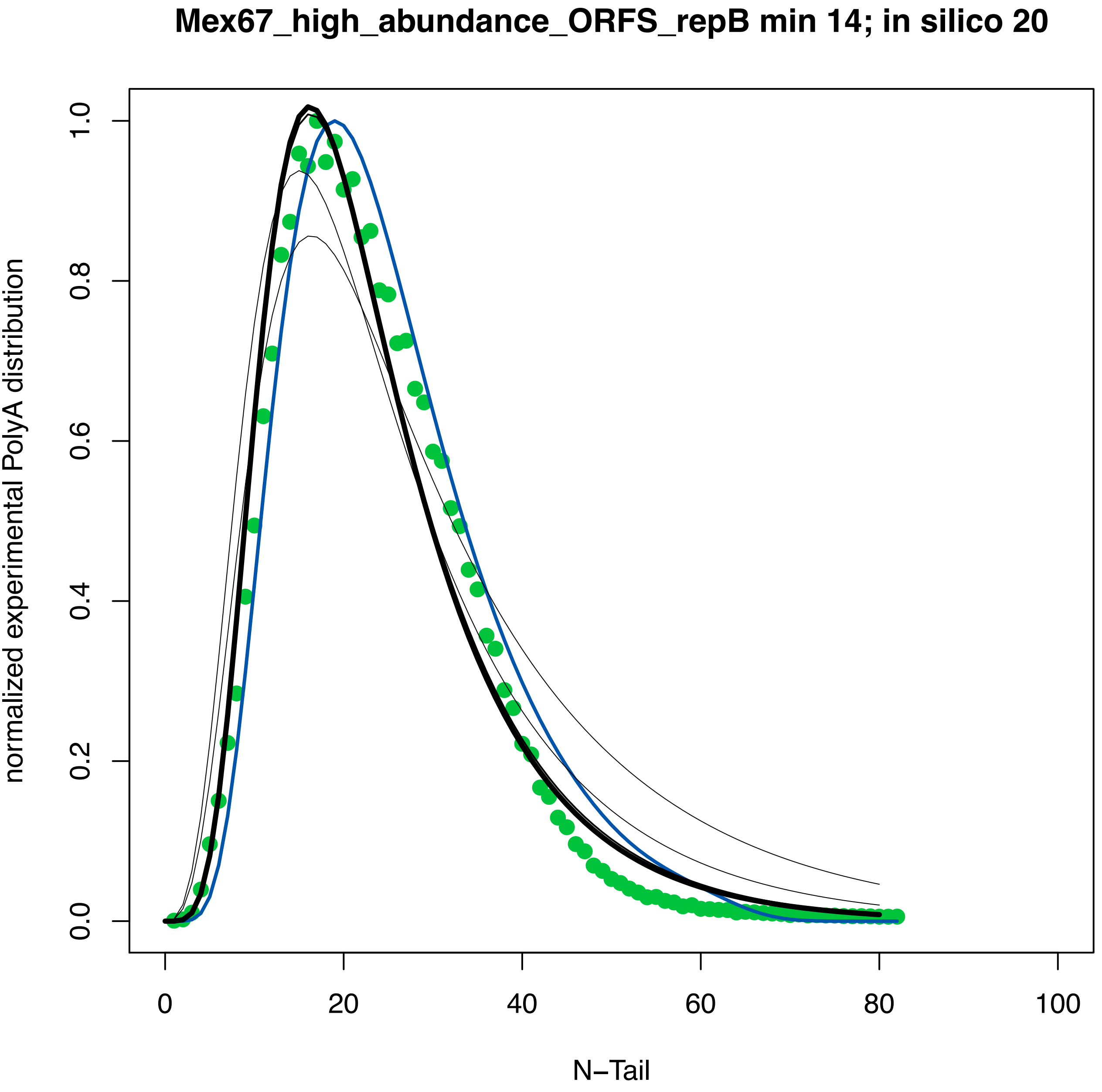
Mex67_high_abundance_ORFS_repB min 14; in silico 20
normalized experimental PolyA distribution
N−Tail
0.0
0.2
0.4
0.6
0.8
1.0
0
20
40
60
80
100

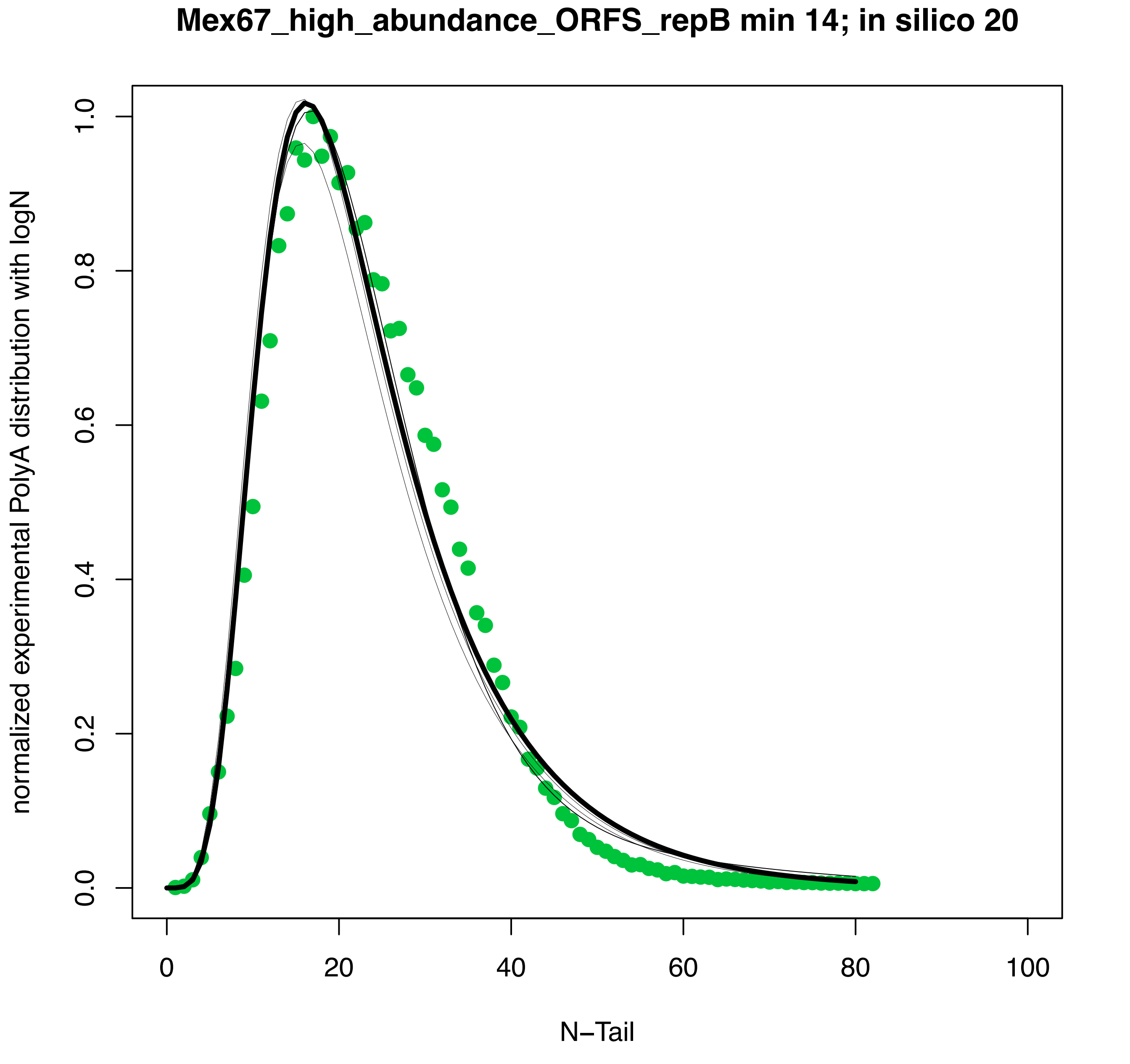

Mex67_high_abundance_ORFS_repB min 14; in silico 20
normalized experimental PolyA distribution with logN
N−Tail
0.0
0.2
0.4
0.6
0.8
1.0
0
20
40
60
80
100

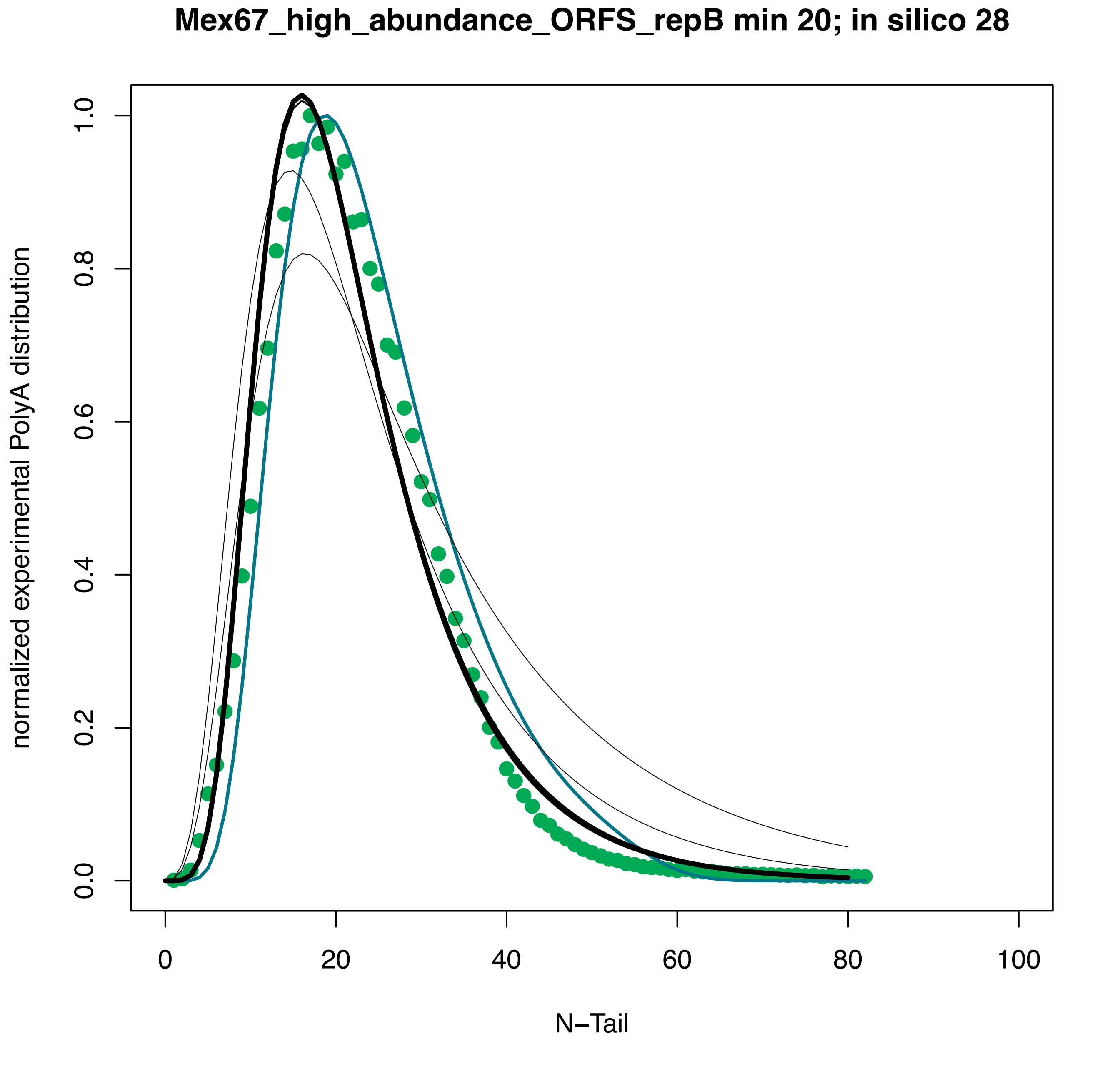

Mex67_high_abundance_ORFS_repB min 20; in silico 28
normalized experimental PolyA distribution
N−Tail
0.0
0.2
0.4
0.6
0.8
1.0
0
20
40
60
80
100

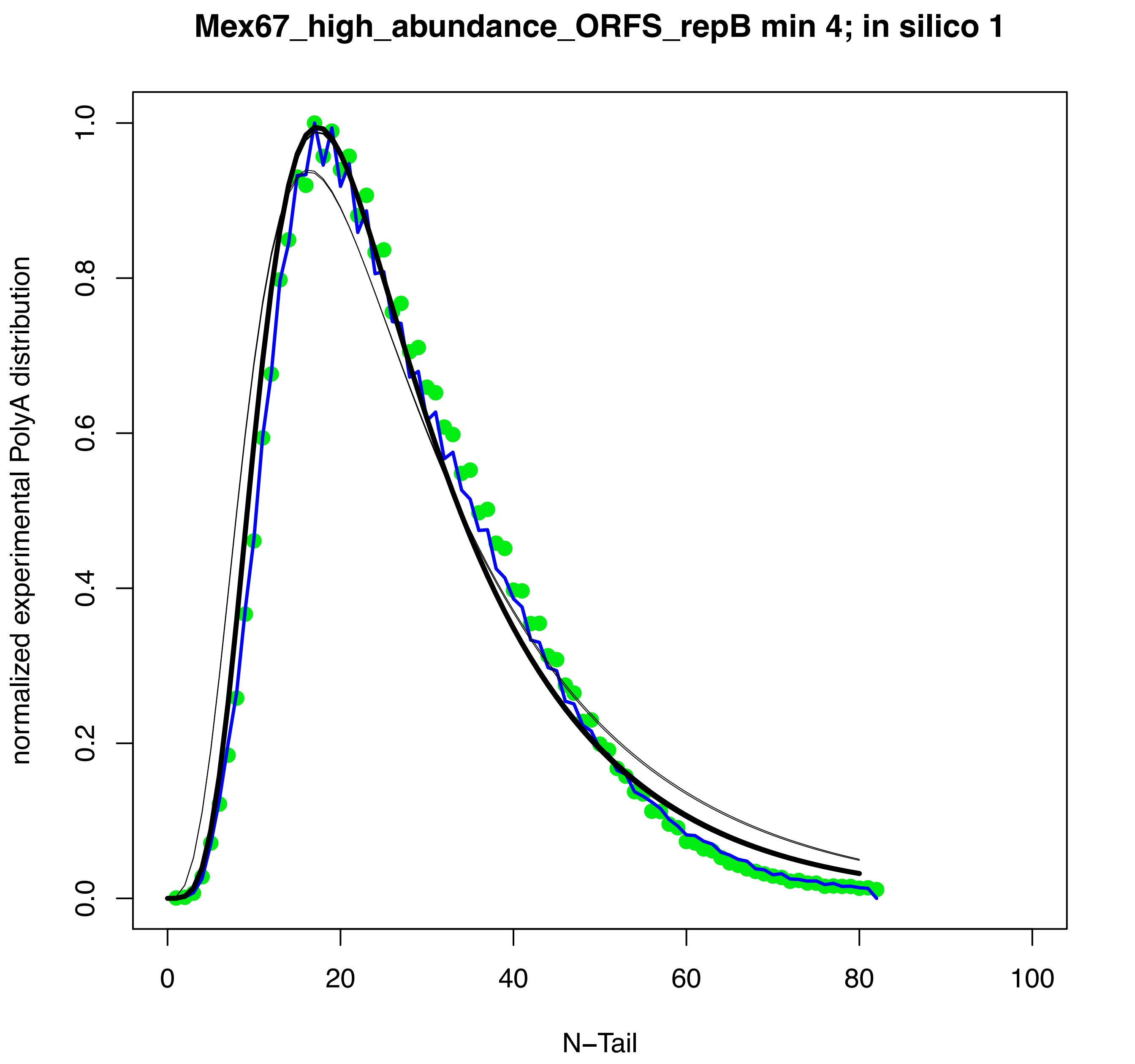

Mex67_high_abundance_ORFS_repB min 4; in silico 1
normalized experimental PolyA distribution
N−Tail
0.0
0.2
0.4
0.6
0.8
1.0
0
20
40
60
80
100

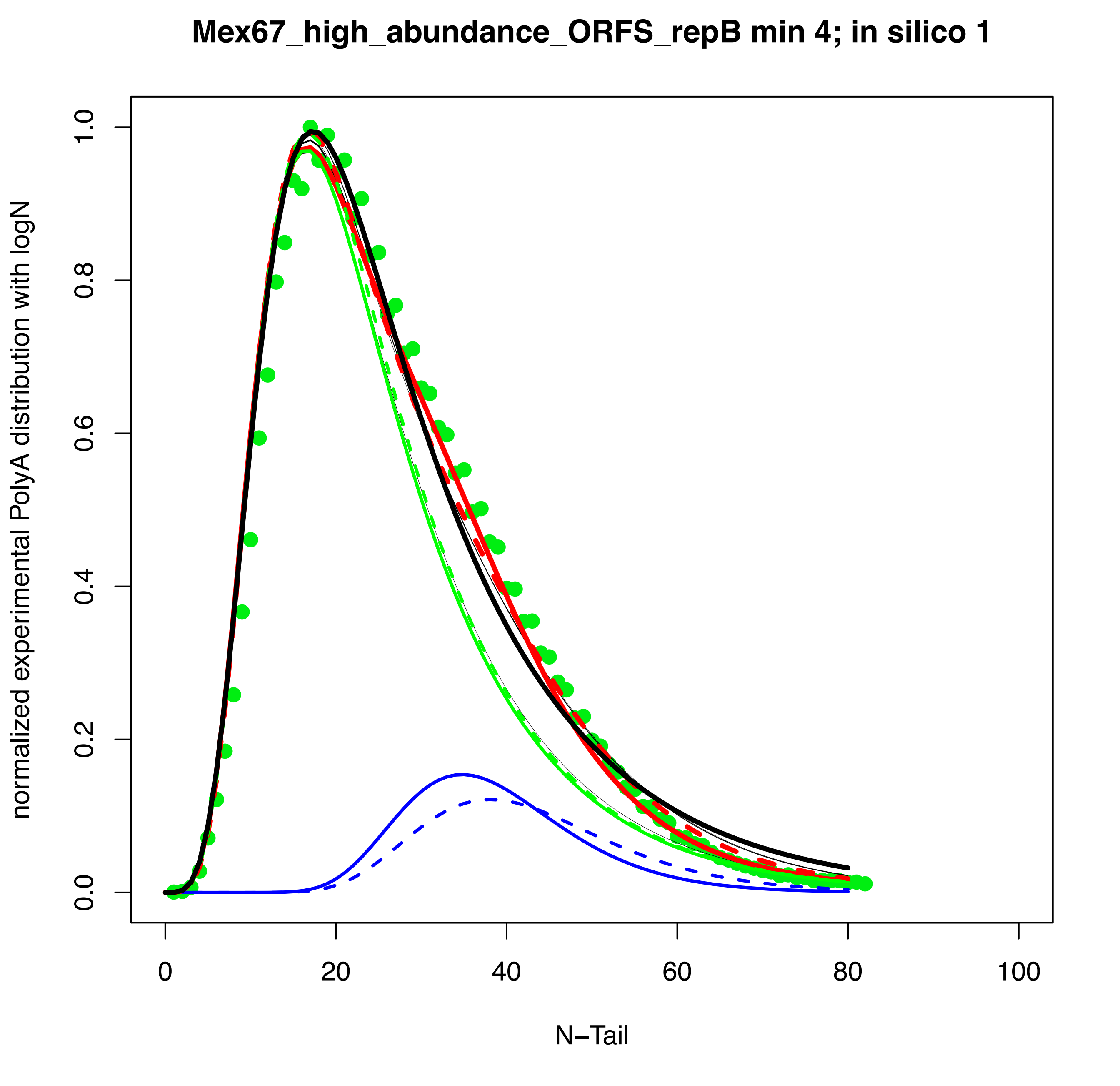

Mex67_high_abundance_ORFS_repB min 4; in silico 1
normalized experimental PolyA distribution with logN
N−Tail
0.0
0.2
0.4
0.6
0.8
1.0
0
20
40
60
80
100

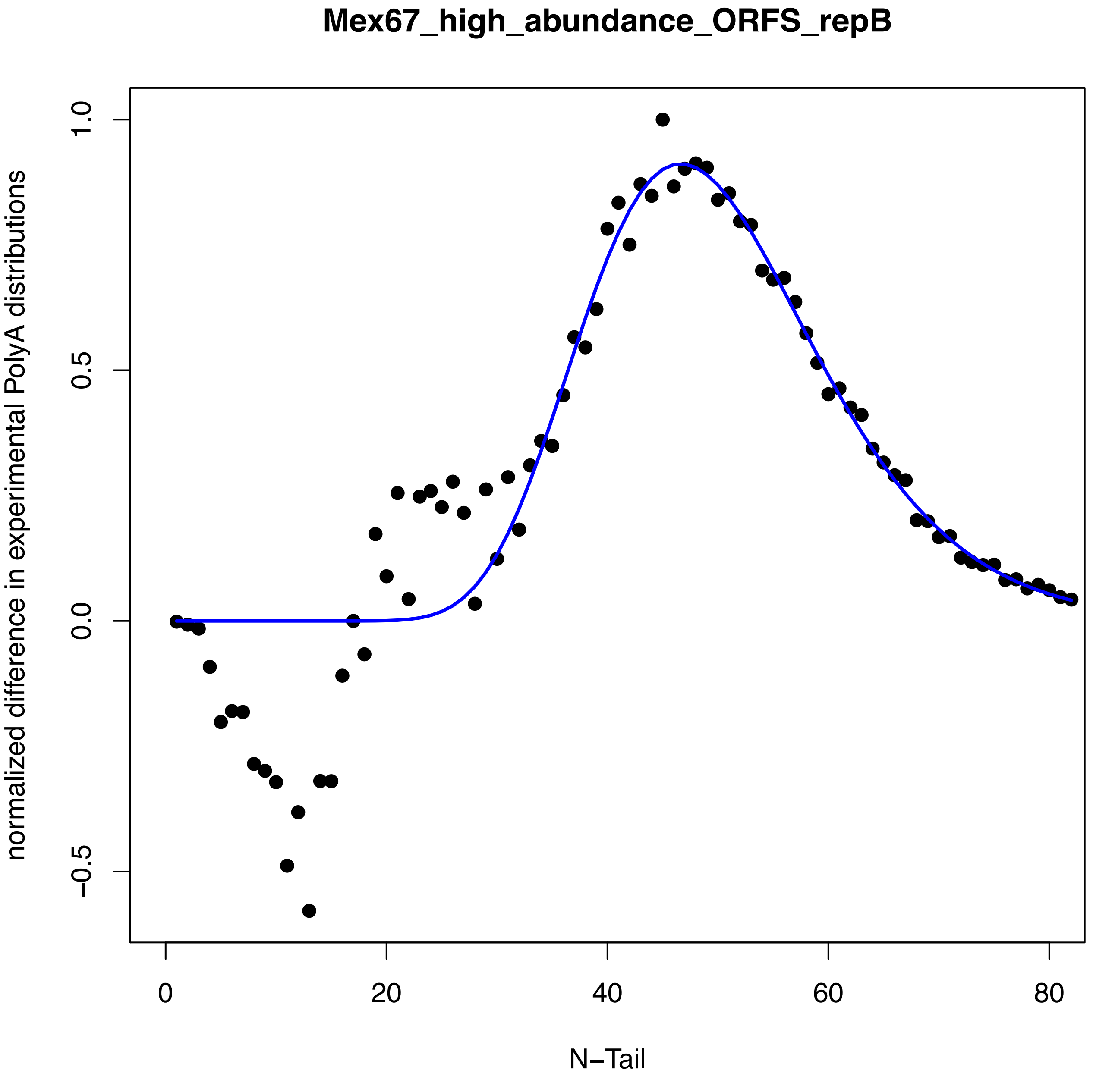

Mex67_high_abundance_ORFS_repB
normalized difference in experimental PolyA distributions
N−Tail
1.0
0.5
0.0
−0.5
0
20
40
60
80

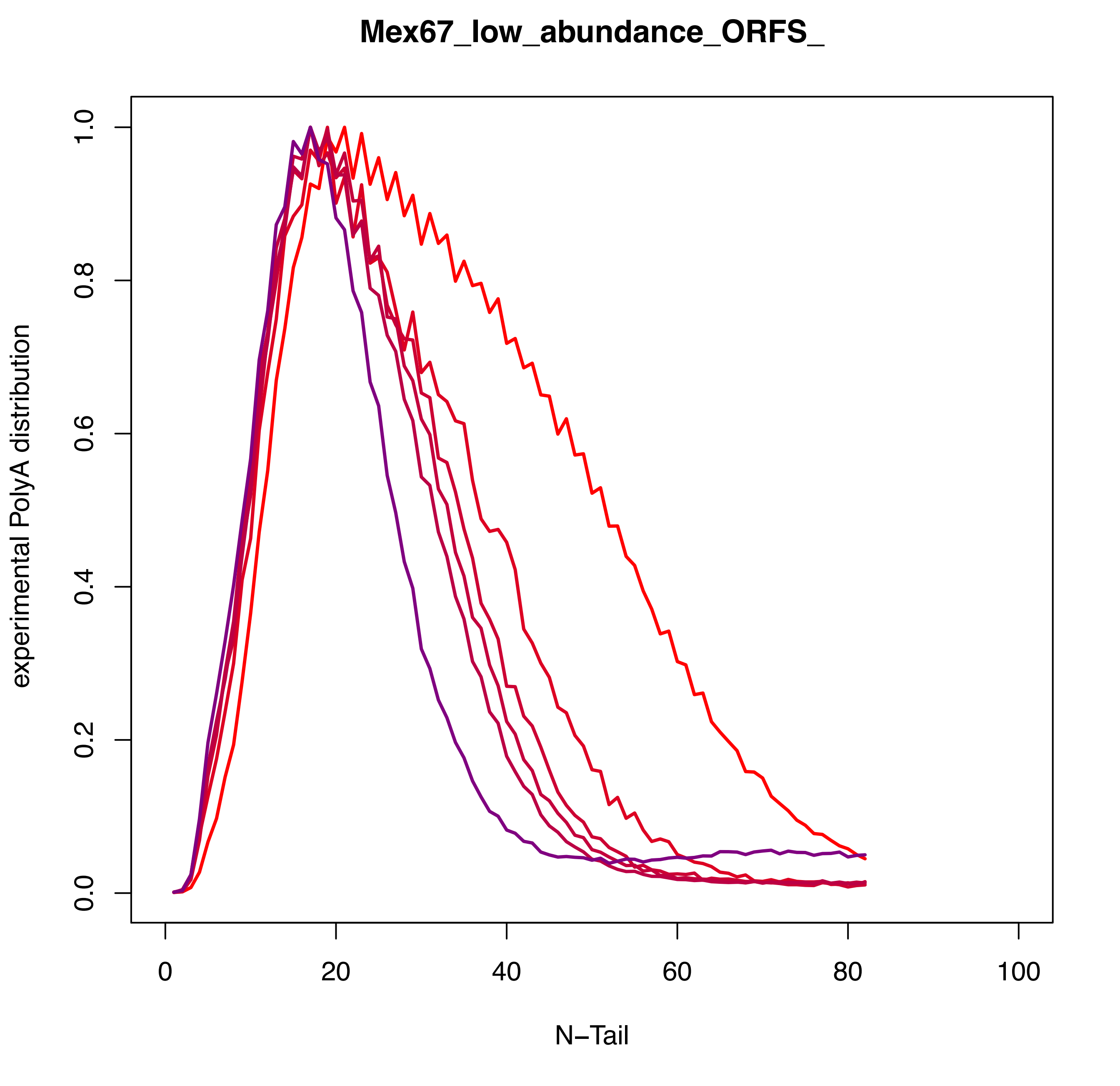

Mex67_low_abundance_ORFS_
experimental PolyA distribution
N−Tail
0.0
0.2
0.4
0.6
0.8
1.0
0
20
40
60
80
100

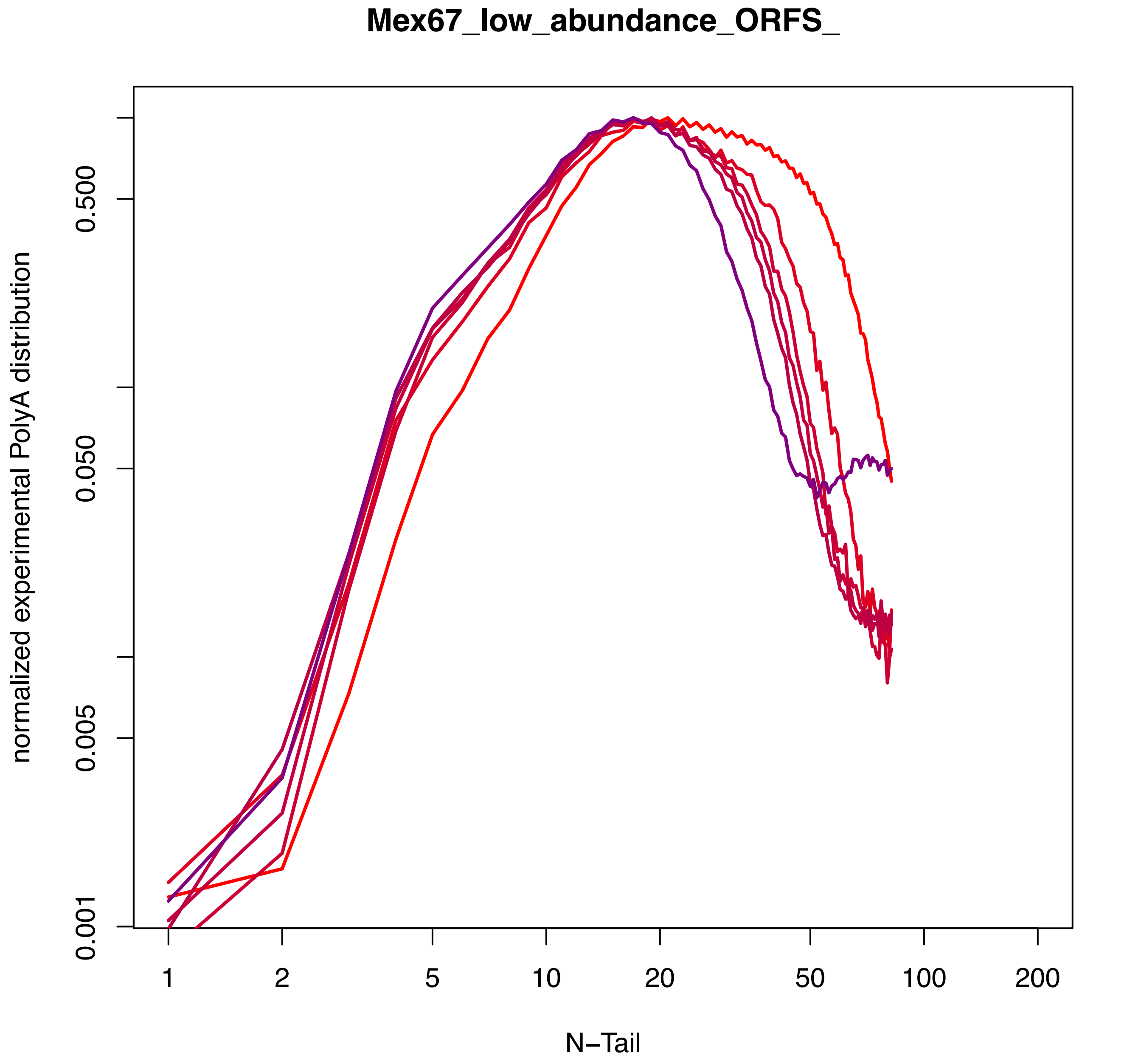

Mex67_low_abundance_ORFS_
normalized experimental PolyA distribution
0.500
0.050
0.005
0.001
1
2
5
10
20
50
100
200
N−Tail

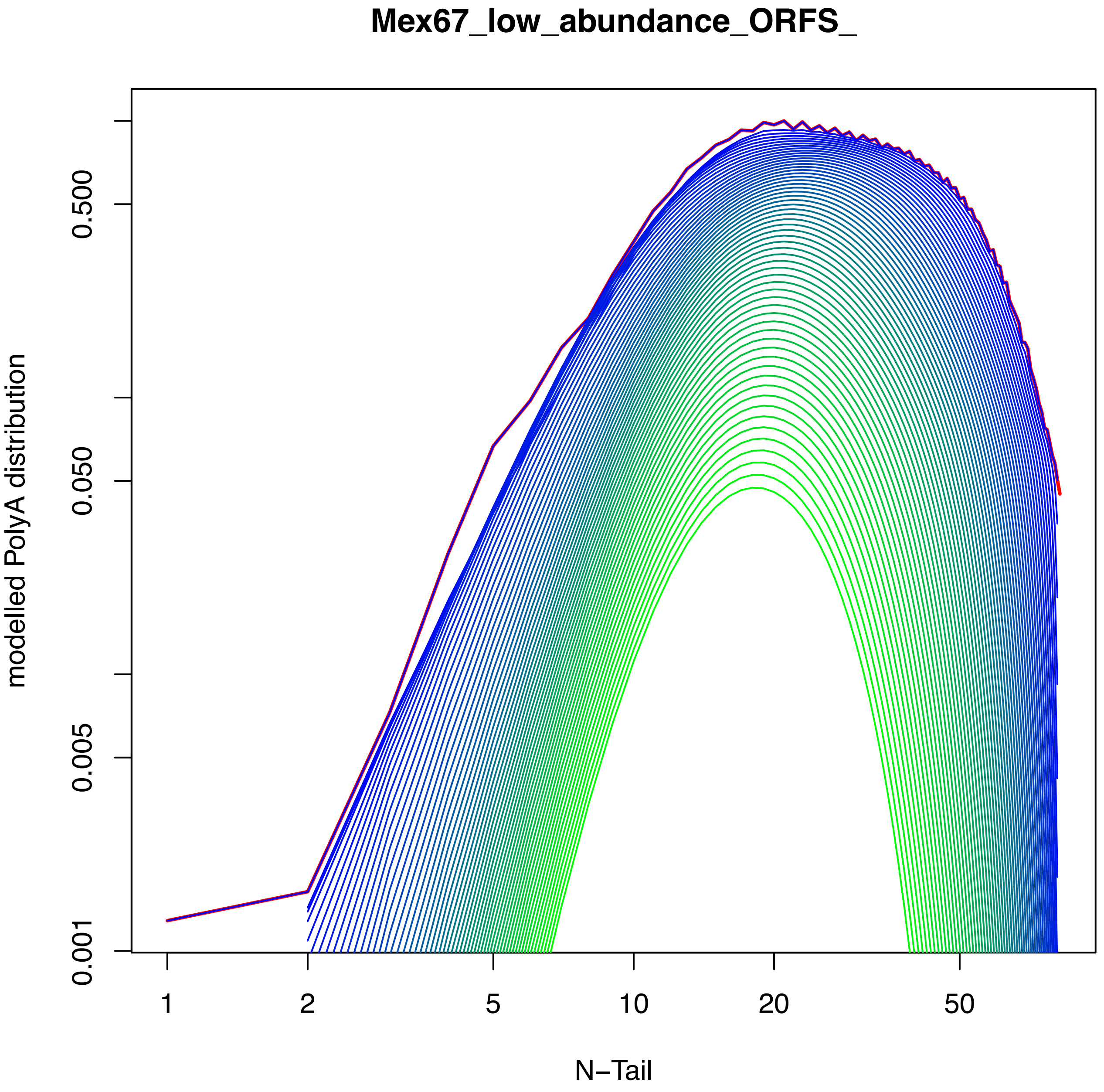
Mex67_low_abundance_ORFS_
modelled PolyA distribution
0.001
0.005
0.050
0.500
1
2
5
10
20
50
N−Tail

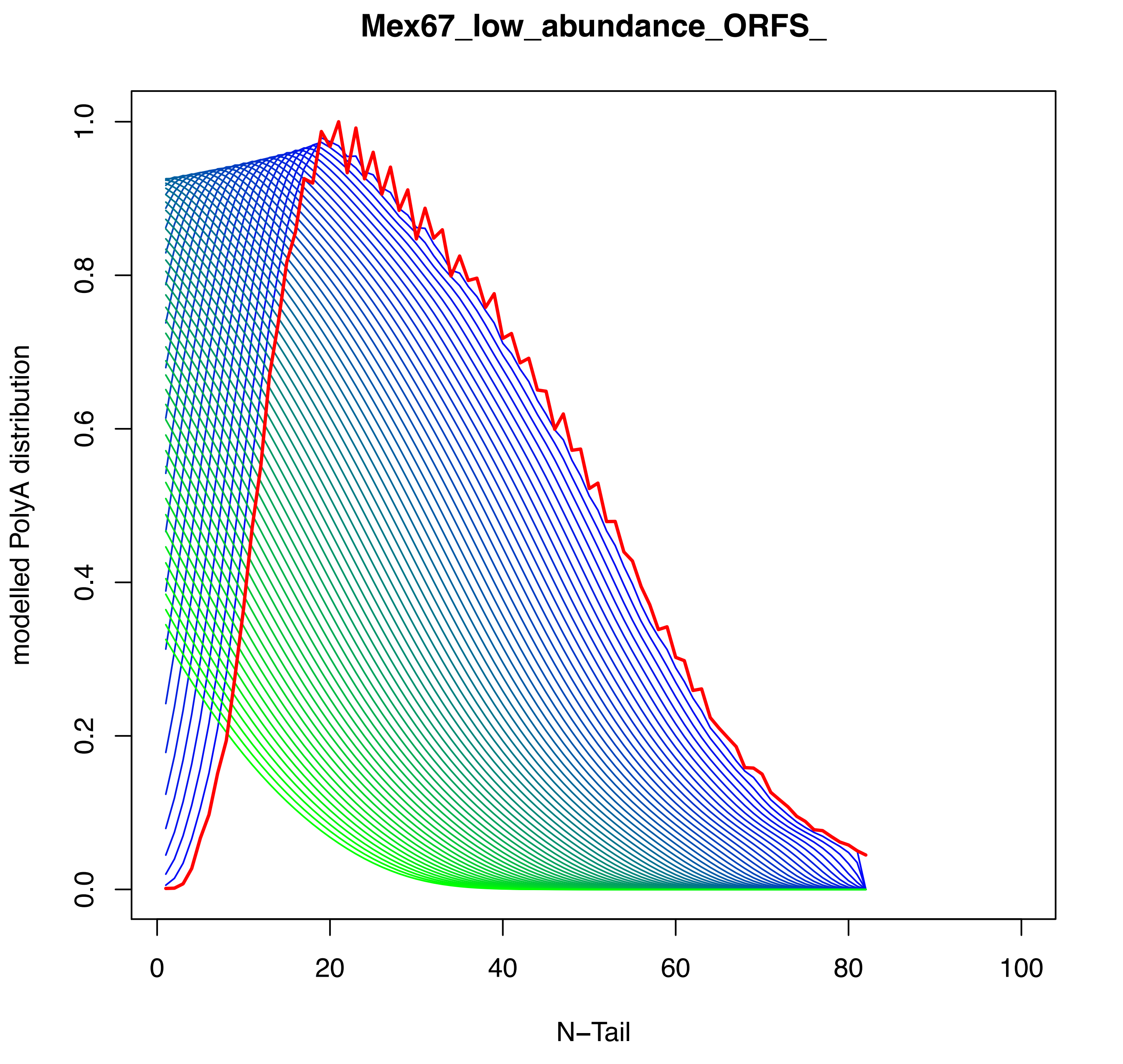
Mex67_low_abundance_ORFS_
modelled PolyA distribution
N−Tail
0.0
0.2
0.4
0.6
0.8
1.0
0
20
40
60
80
100

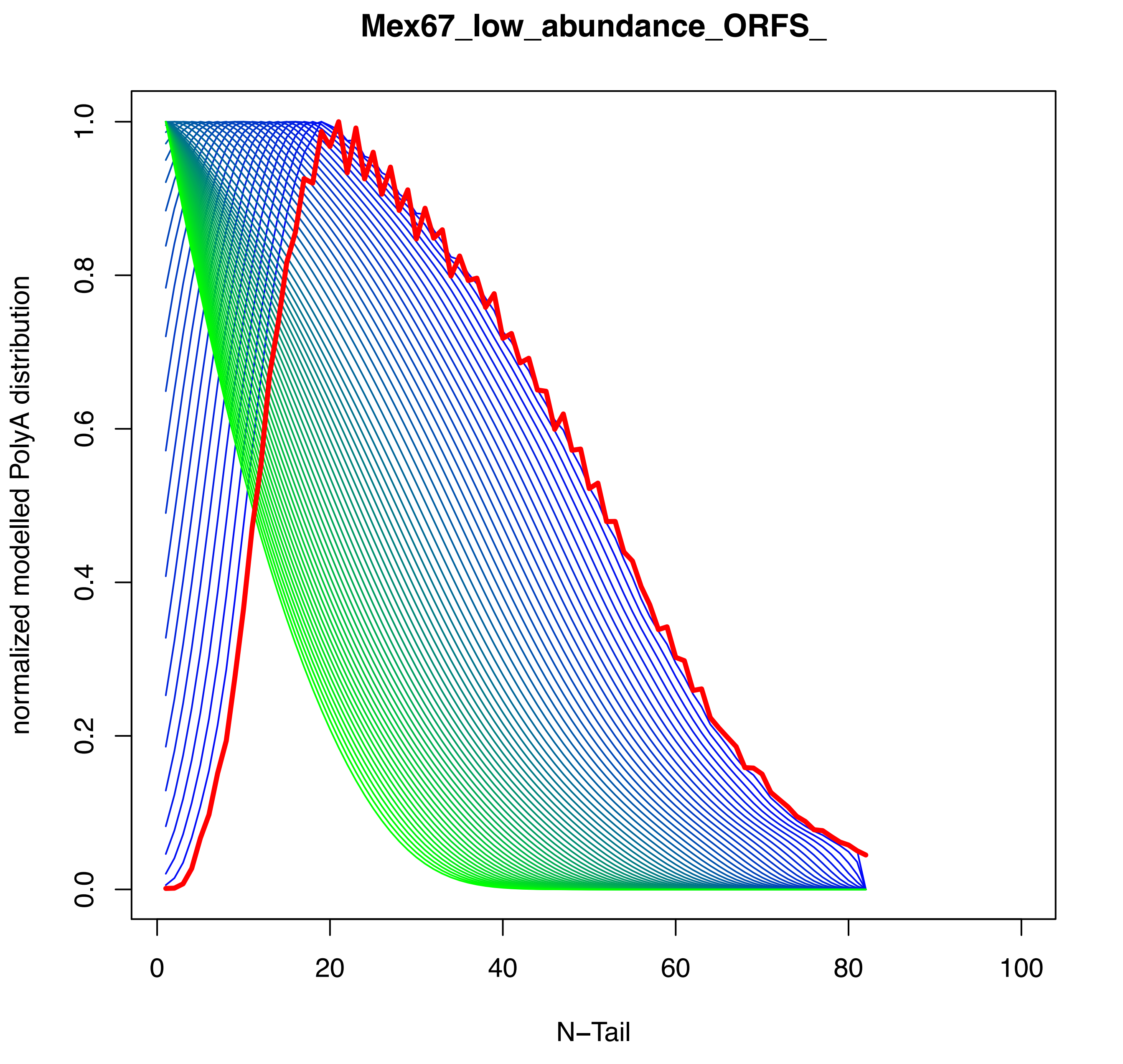

Mex67_low_abundance_ORFS_
normalized modelled PolyA distribution
N−Tail
0.0
0.2
0.4
0.6
0.8
1.0
0
20
40
60
80
100

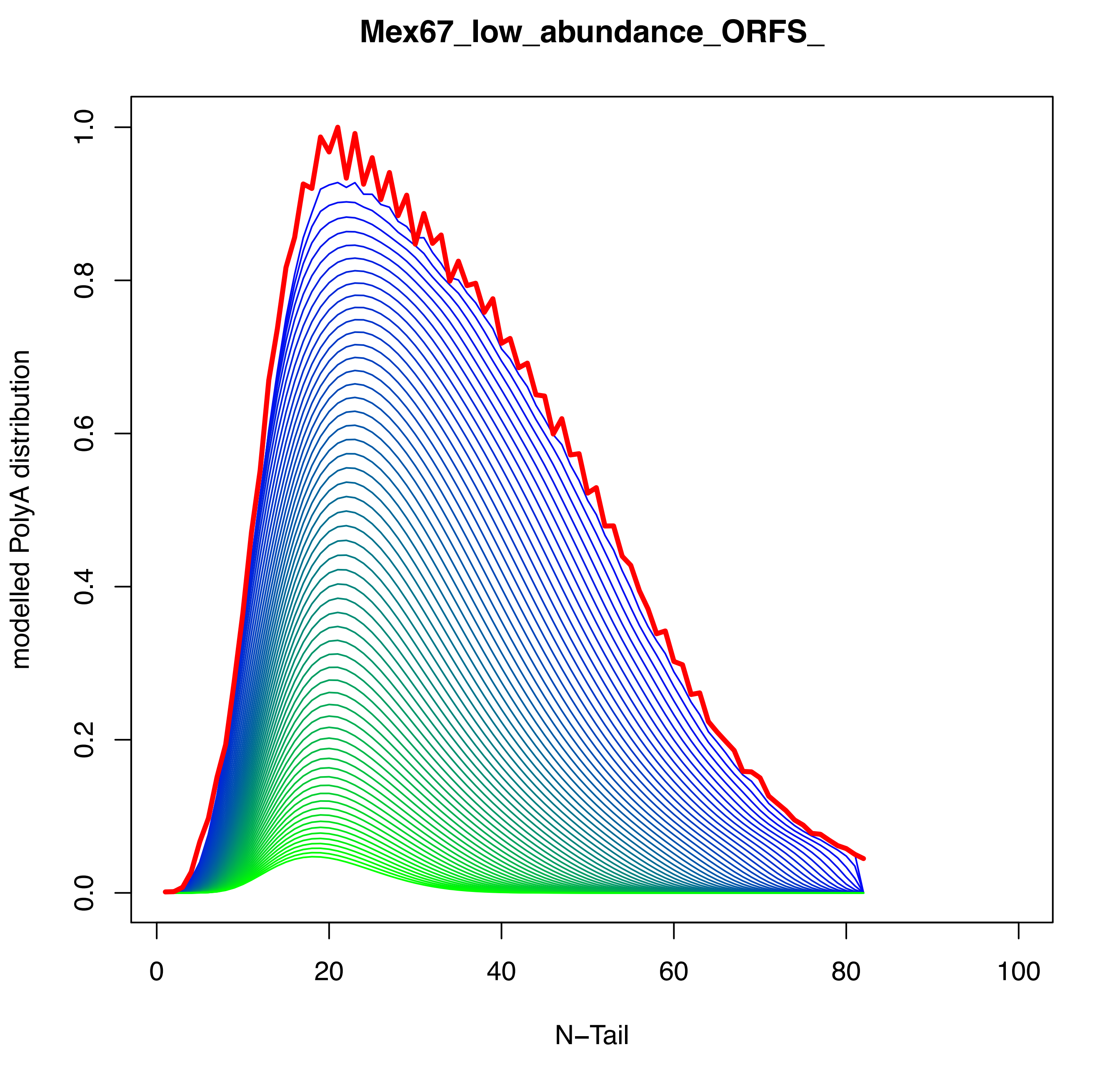

Mex67_low_abundance_ORFS_
modelled PolyA distribution
N−Tail
0.0
0.2
0.4
0.6
0.8
1.0
0
20
40
60
80
100

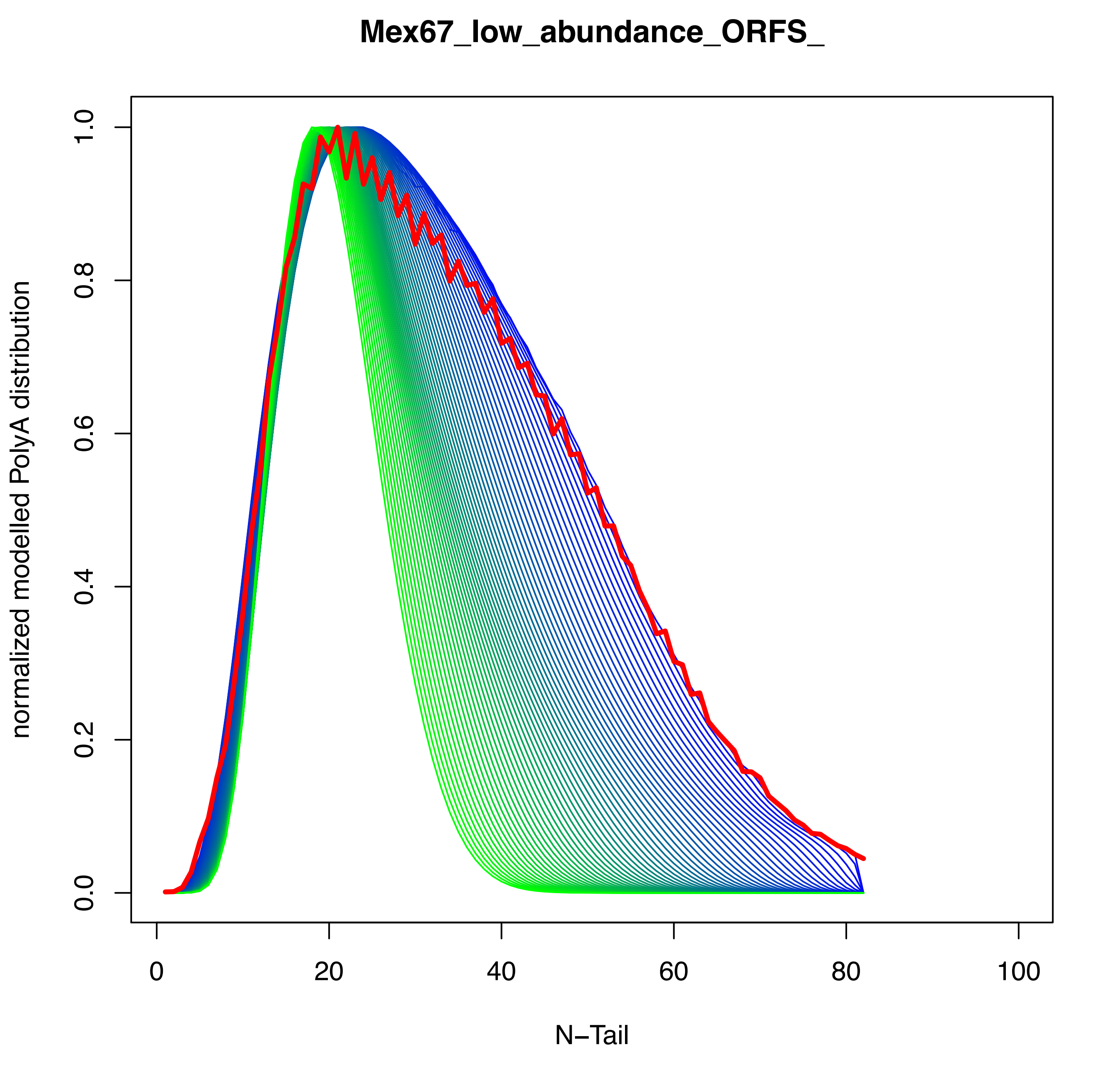

Mex67_low_abundance_ORFS_
normalized modelled PolyA distribution
N−Tail
0.0
0.2
0.4
0.6
0.8
1.0
0
20
40
60
80
100

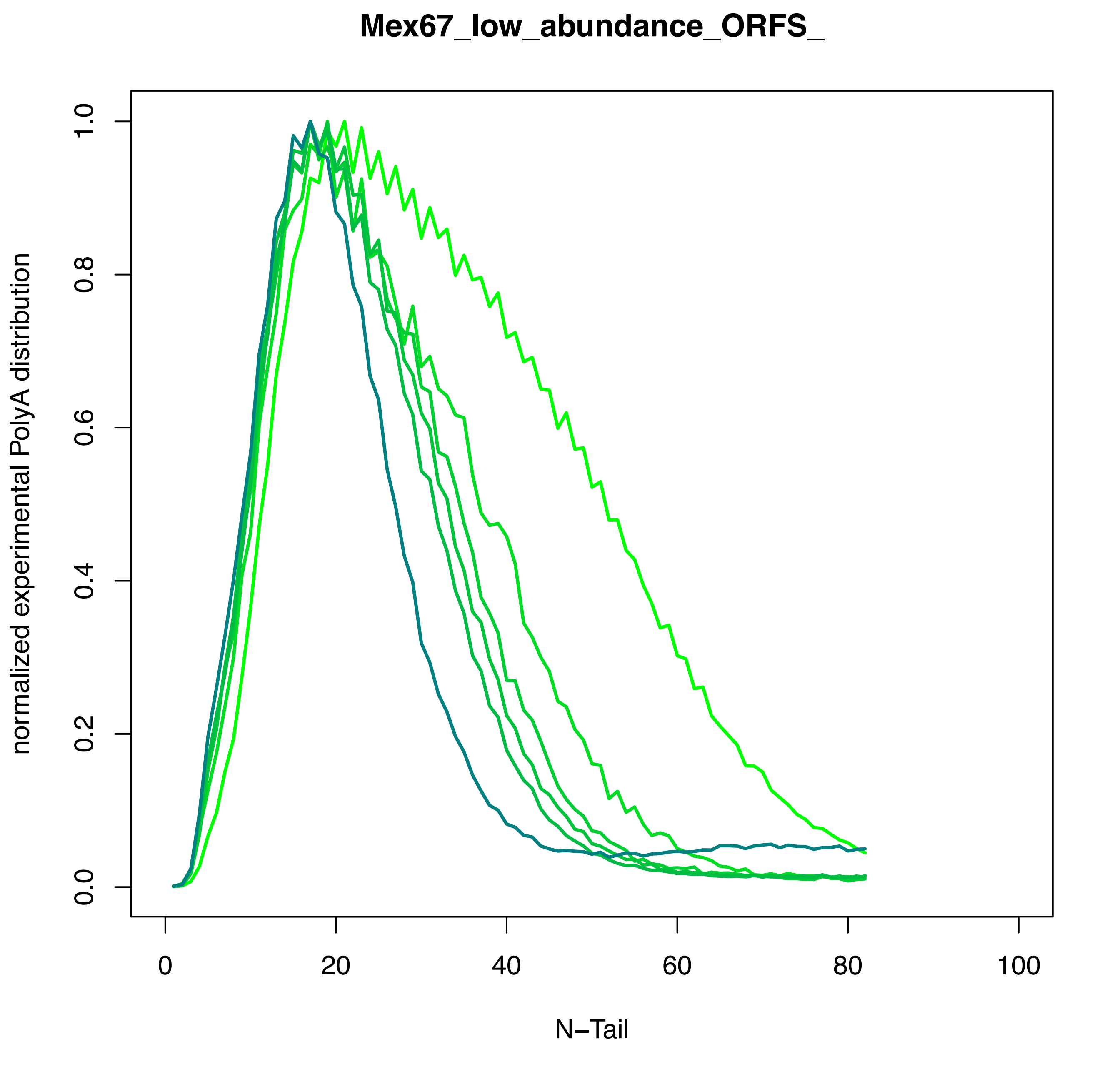

Mex67_low_abundance_ORFS_
normalized experimental PolyA distribution
N−Tail
0.0
0.2
0.4
0.6
0.8
1.0
0
20
40
60
80
100

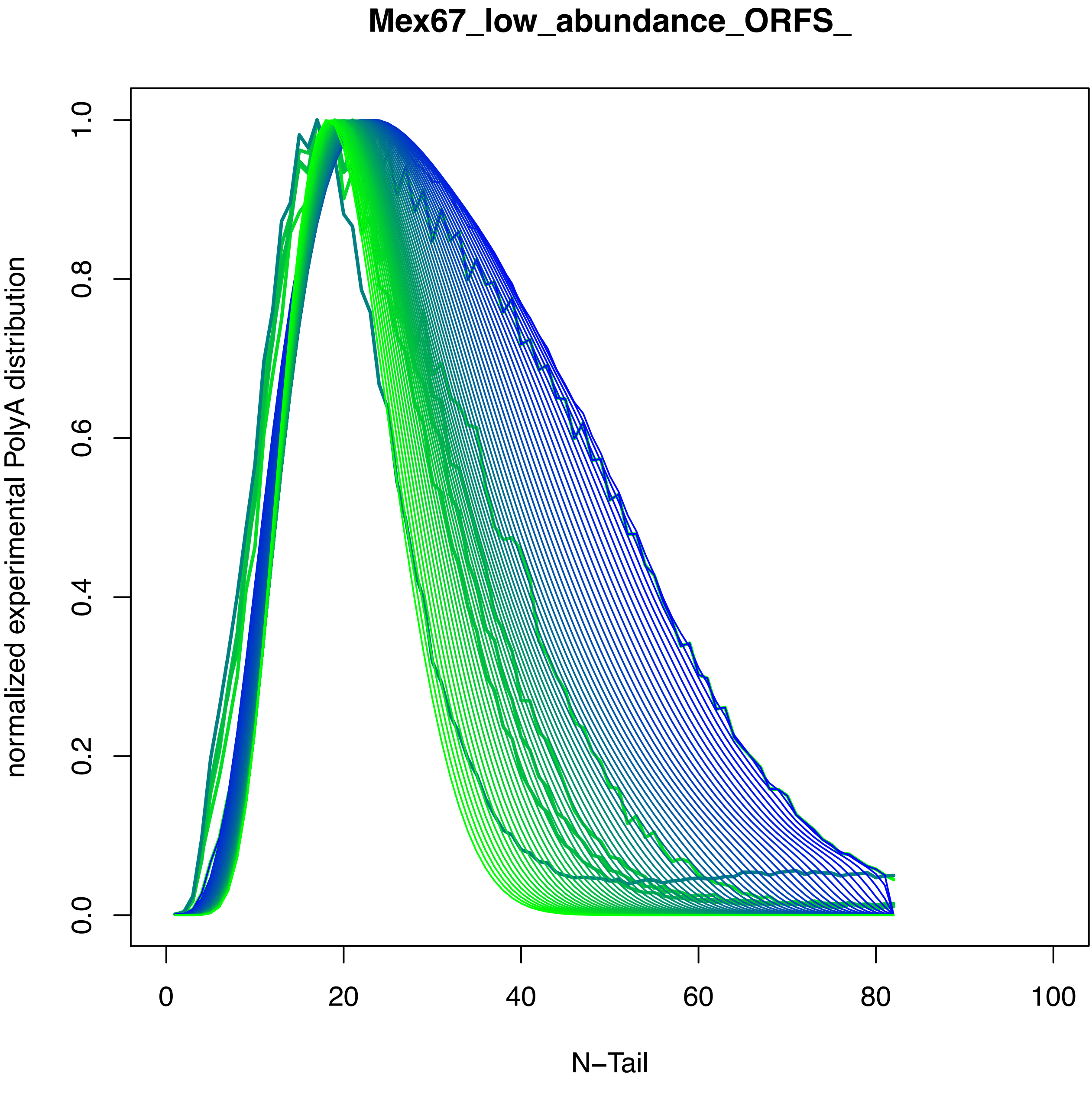

Mex67_low_abundance_ORFS_
normalized experimental PolyA distribution
N−Tail
0.0
0.2
0.4
0.6
0.8
1.0
0
20
40
60
80
100

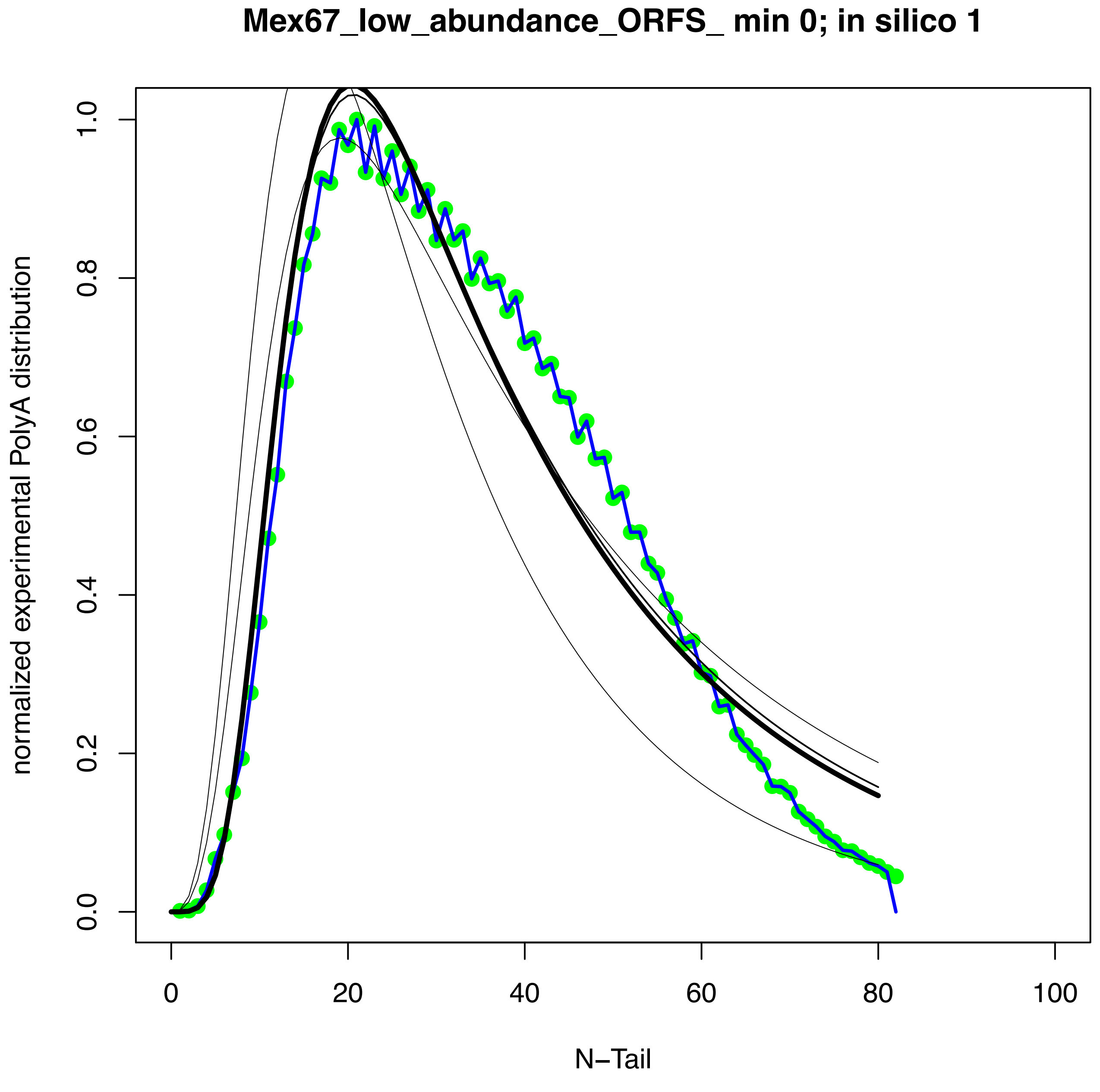
Mex67_low_abundance_ORFS_ min 0; in silico 1
normalized experimental PolyA distribution
N−Tail
0.0
0.2
0.4
0.6
0.8
1.0
0
20
40
60
80
100

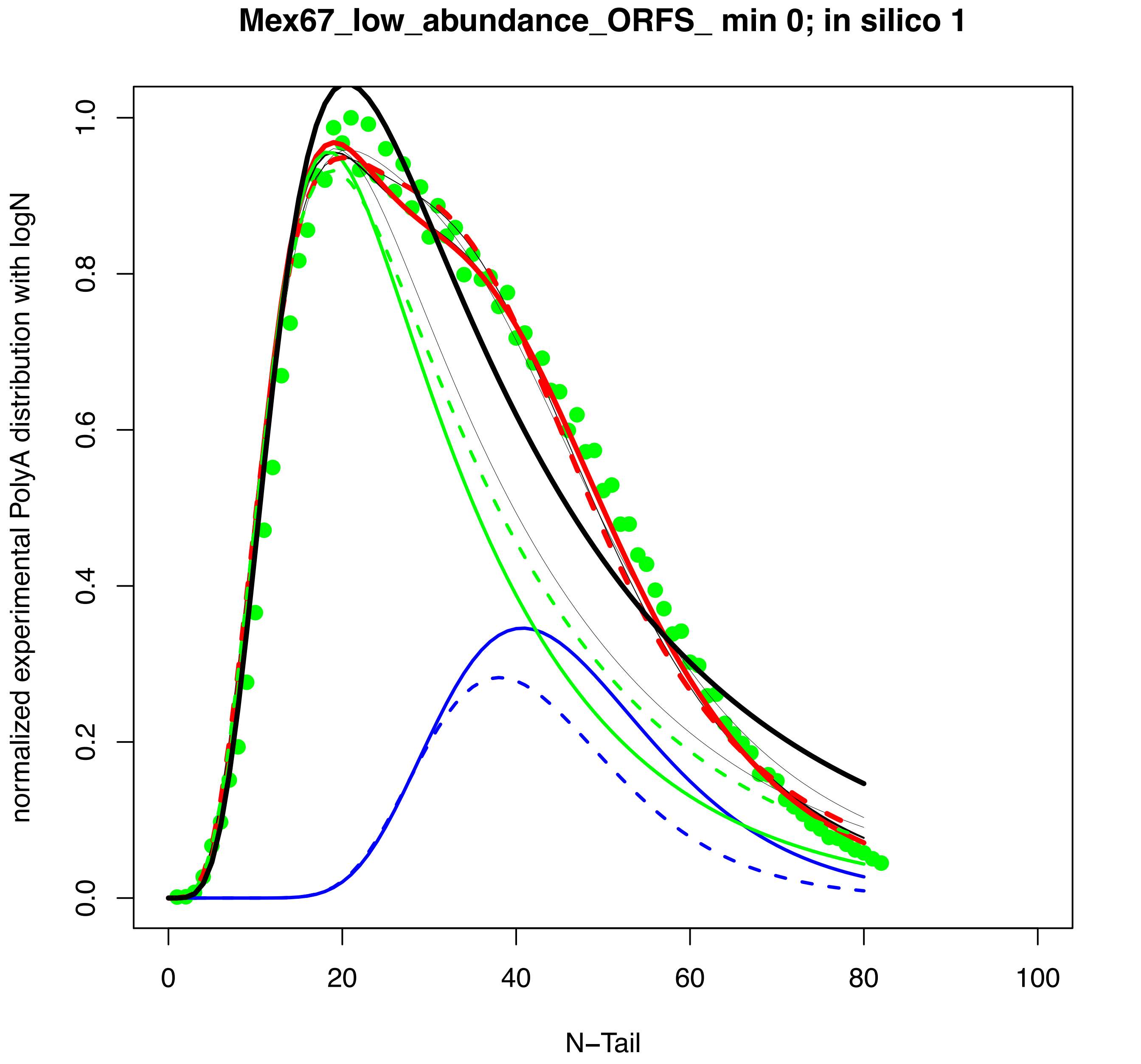

Mex67_low_abundance_ORFS_ min 0; in silico 1
normalized experimental PolyA distribution with logN
N−Tail
0.0
0.2
0.4
0.6
0.8
1.0
0
20
40
60
80
100

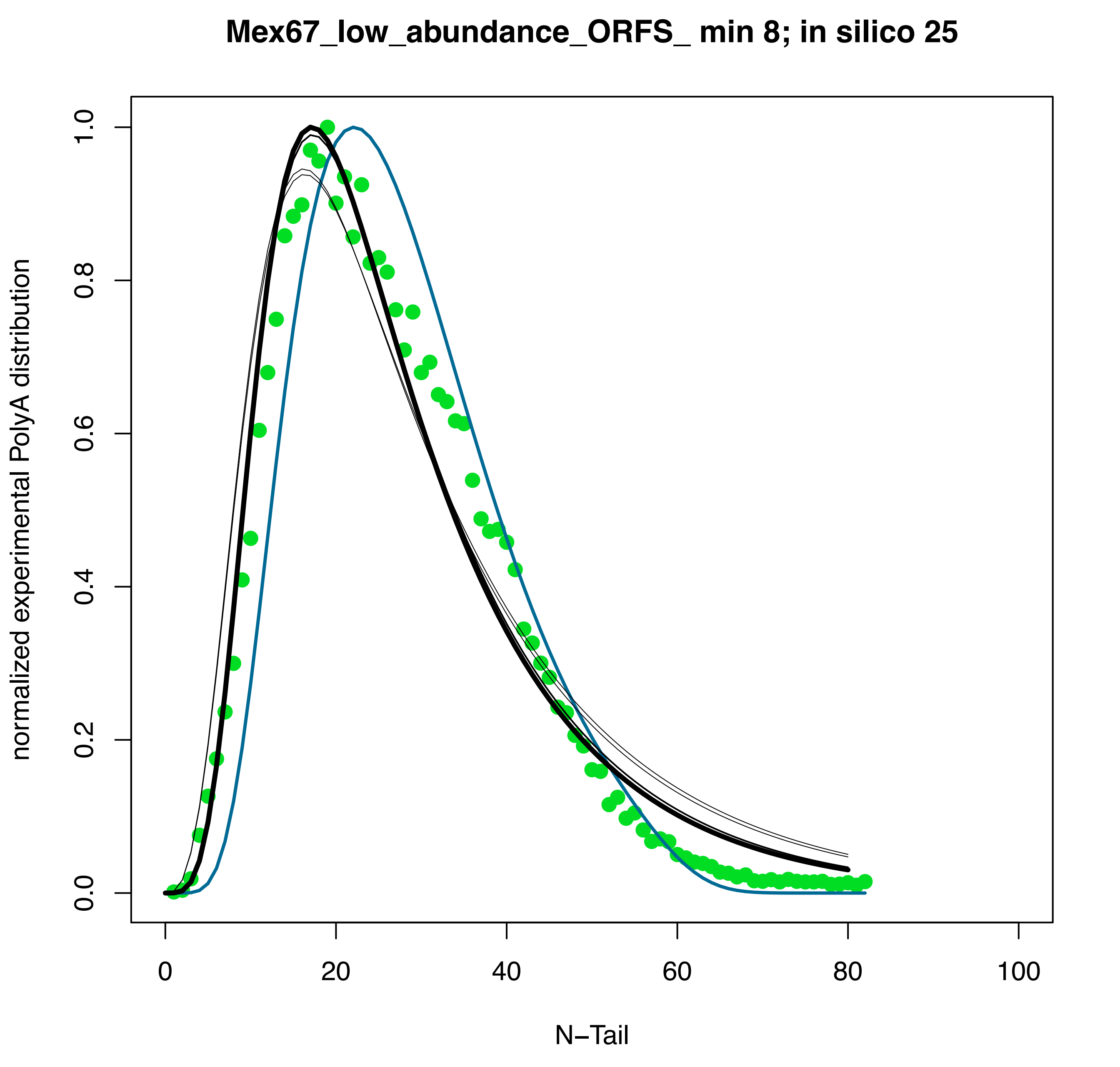
Mex67_low_abundance_ORFS_ min 8; in silico 25
normalized experimental PolyA distribution
N−Tail
0.0
0.2
0.4
0.6
0.8
1.0
0
20
40
60
80
100

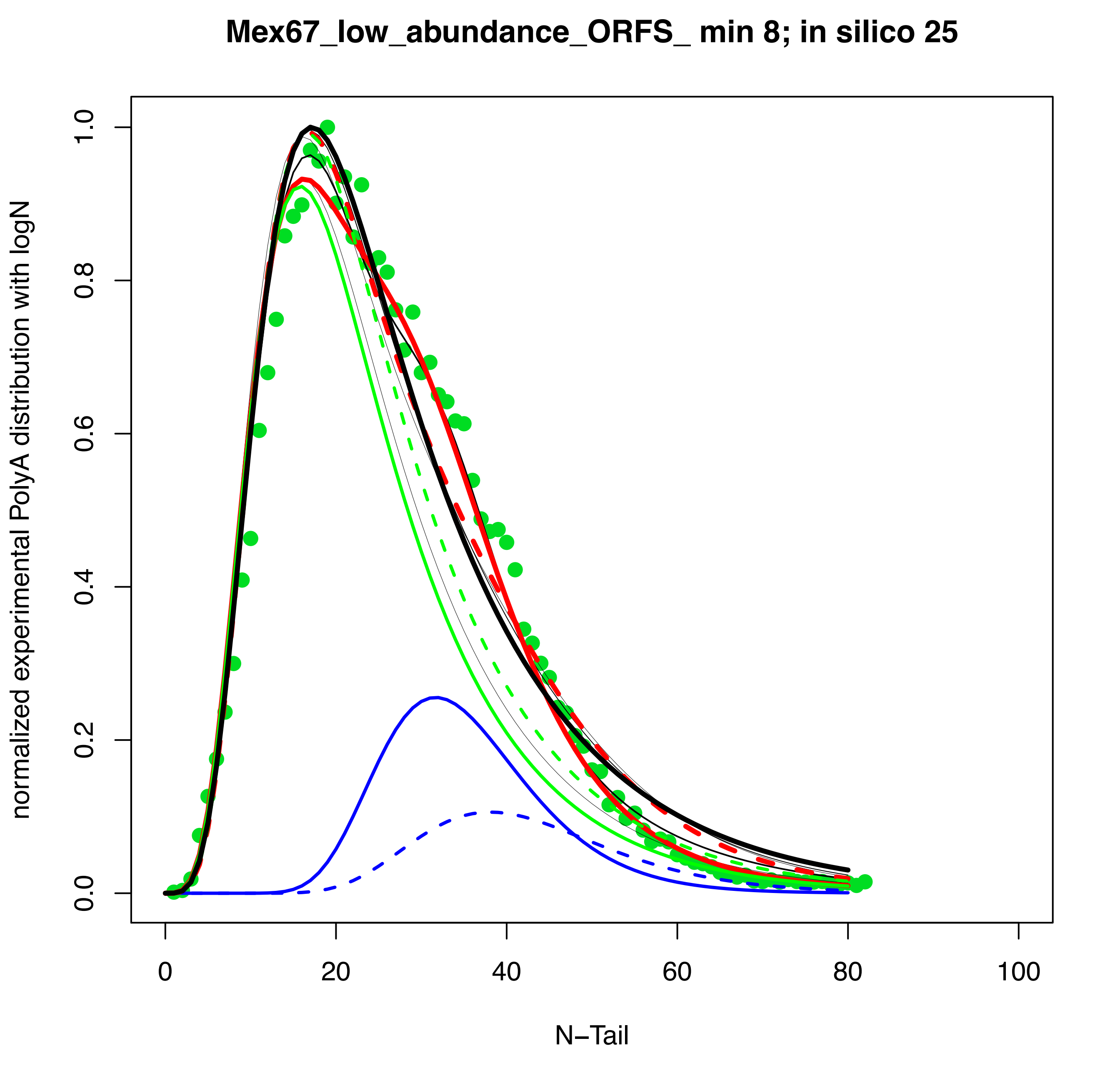

Mex67_low_abundance_ORFS_ min 8; in silico 25
normalized experimental PolyA distribution with logN
N−Tail
0.0
0.2
0.4
0.6
0.8
1.0
0
20
40
60
80
100

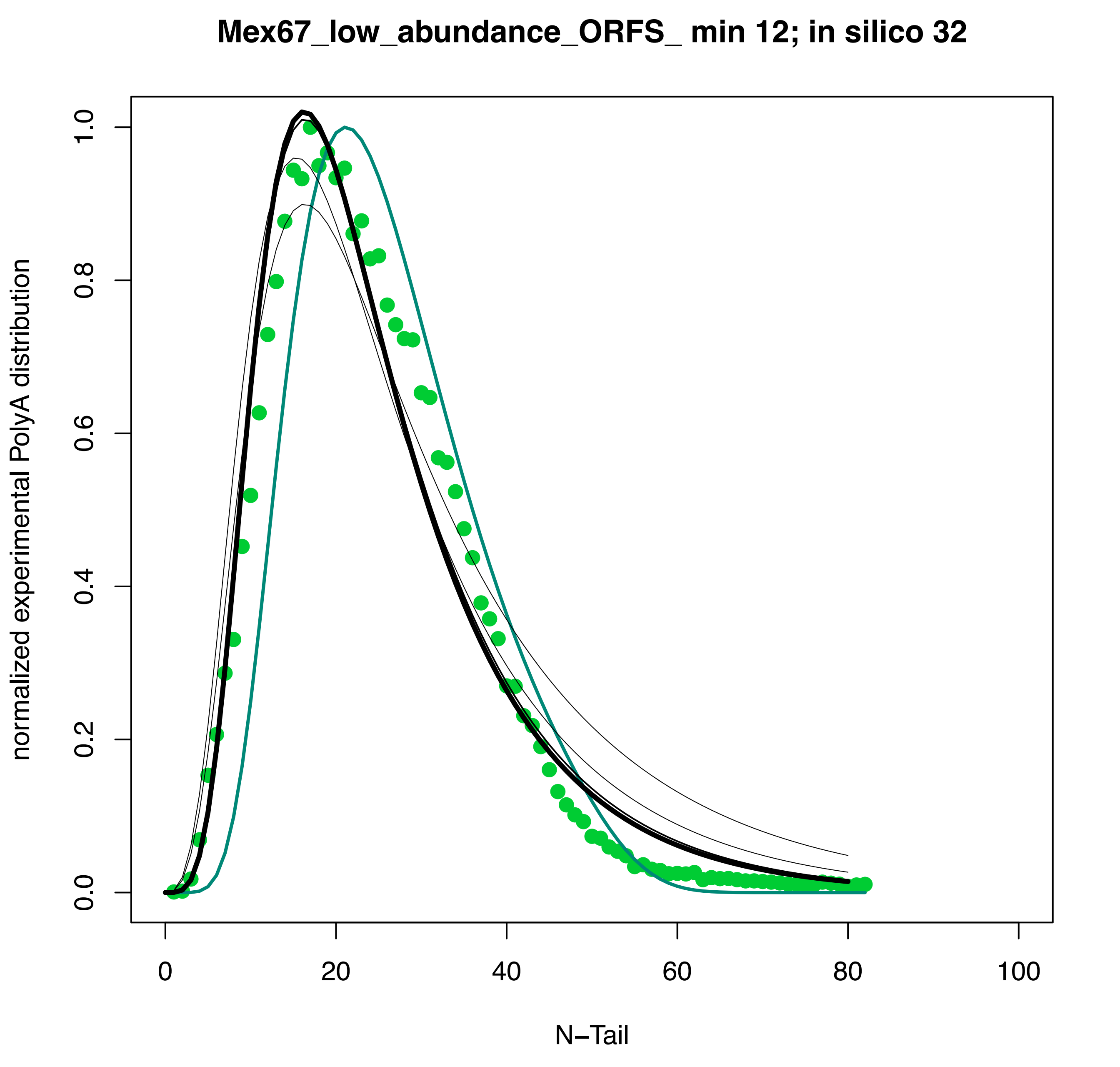
Mex67_low_abundance_ORFS_ min 12; in silico 32
normalized experimental PolyA distribution
N−Tail
0.0
0.2
0.4
0.6
0.8
1.0
0
20
40
60
80
100

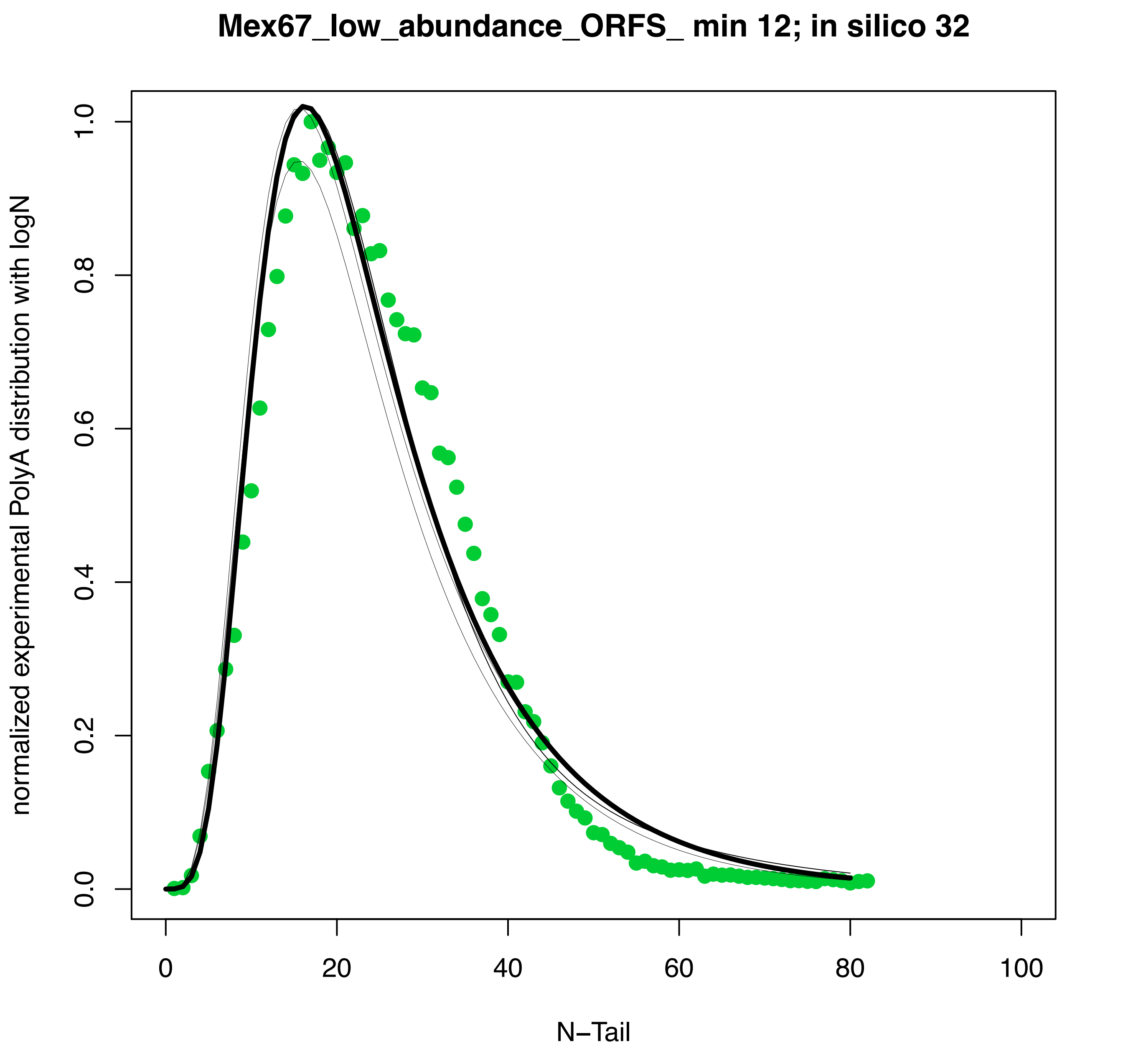

Mex67_low_abundance_ORFS_ min 12; in silico 32
normalized experimental PolyA distribution with logN
0.0
0.2
0.4
0.6
0.8
1.0
0
20
40
60
80
100
N−Tail

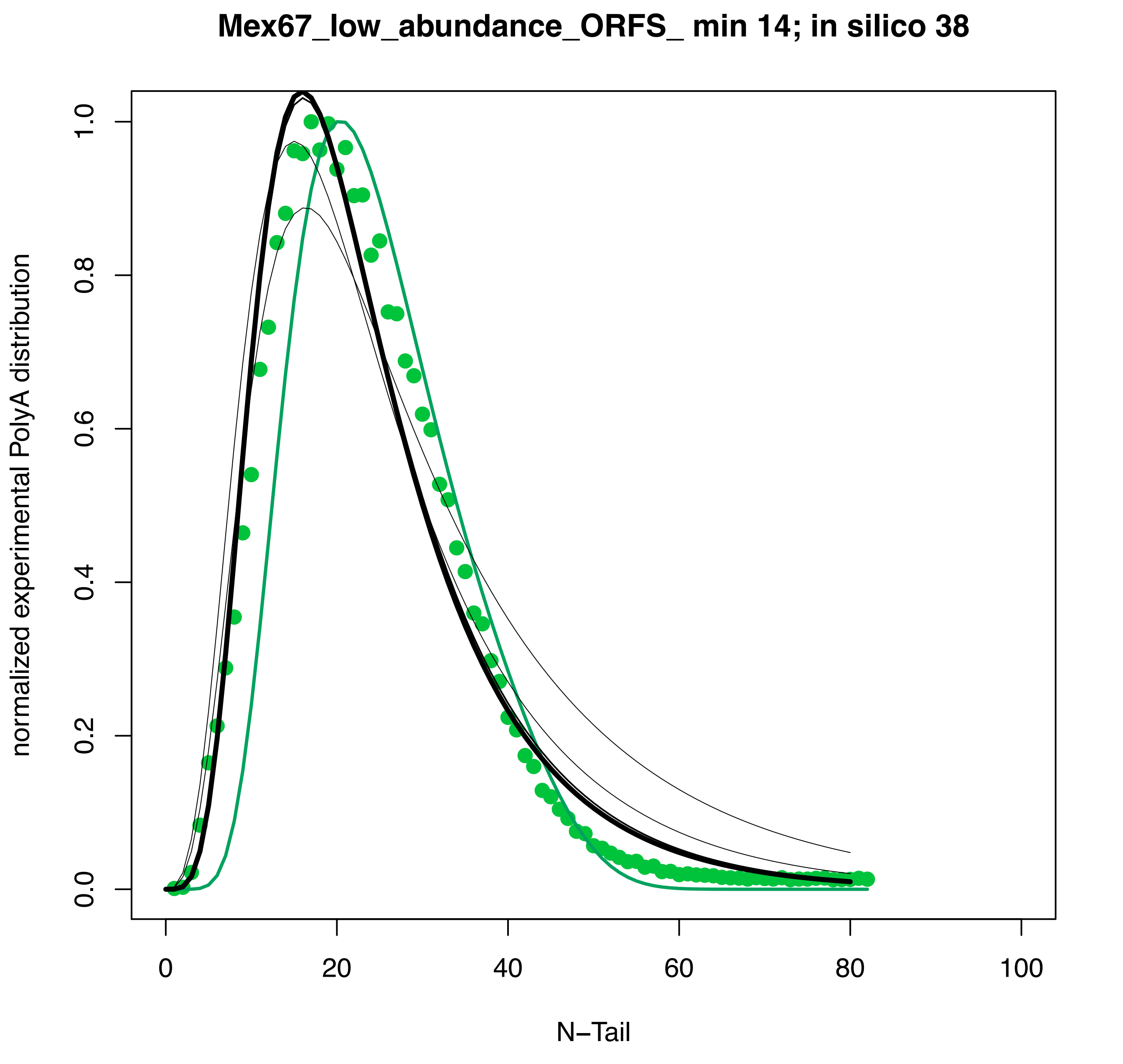

Mex67_low_abundance_ORFS_ min 14; in silico 38
normalized experimental PolyA distribution
N−Tail
0.0
0.2
0.4
0.6
0.8
1.0
0
20
40
60
80
100

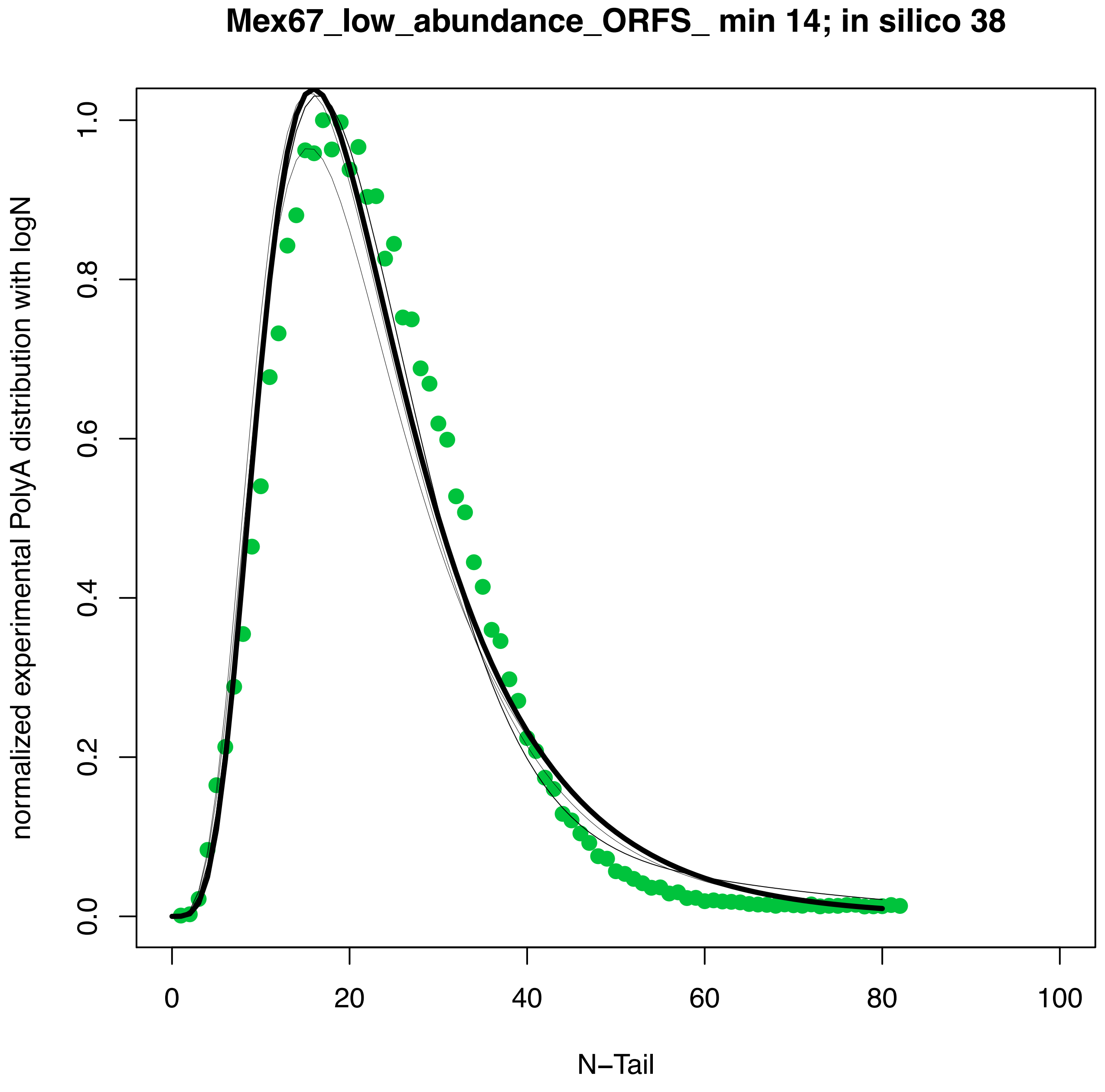

Mex67_low_abundance_ORFS_ min 14; in silico 38
normalized experimental PolyA distribution with logN
N−Tail
0.0
0.2
0.4
0.6
0.8
1.0
0
20
40
60
80
100

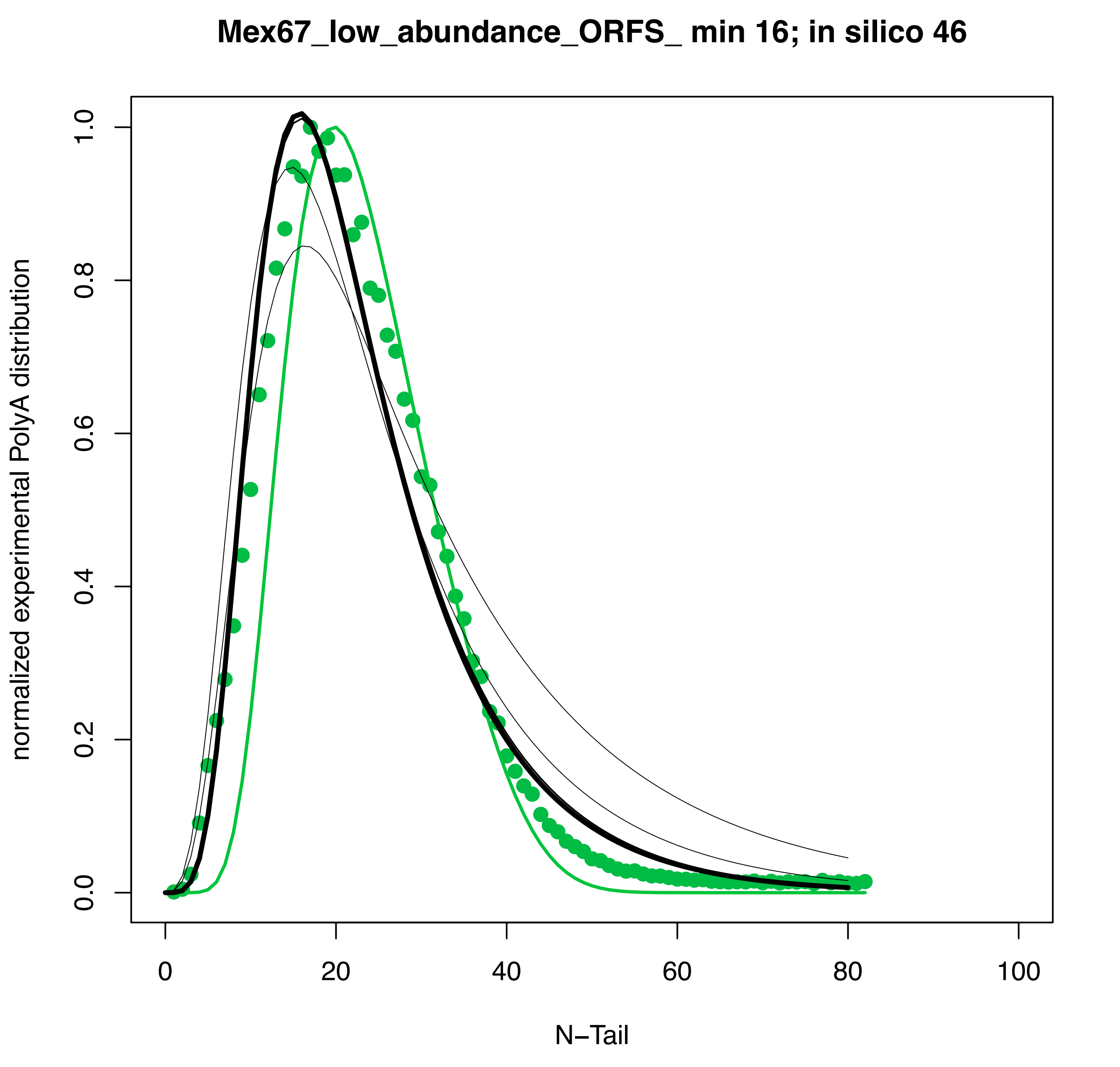

Mex67_low_abundance_ORFS_ min 16; in silico 46
normalized experimental PolyA distribution
N−Tail
0.0
0.2
0.4
0.6
0.8
1.0
0
20
40
60
80
100

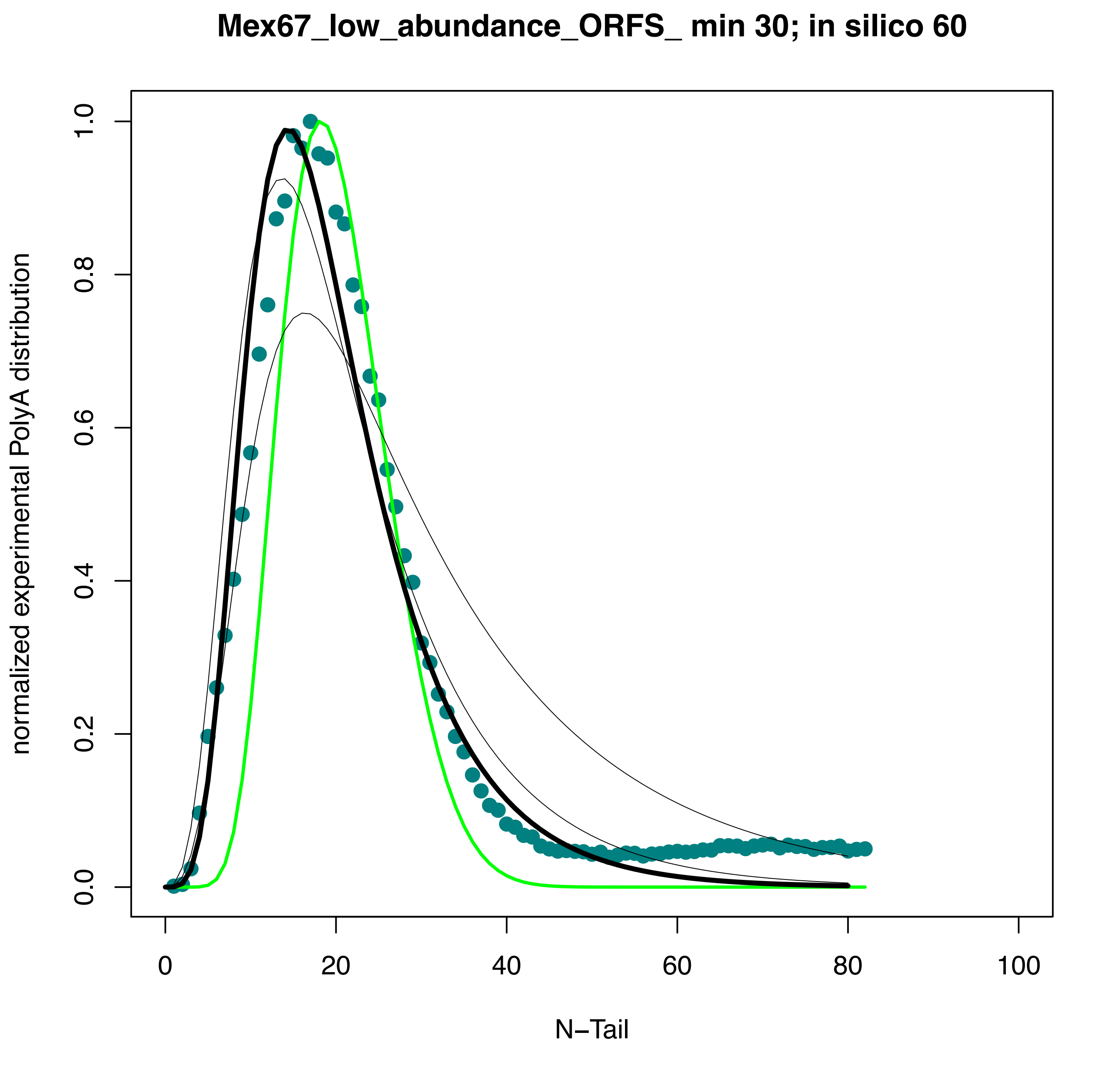

Mex67_low_abundance_ORFS_ min 30; in silico 60
normalized experimental PolyA distribution
0.0
0.2
0.4
0.6
0.8
1.0
0
20
40
60
80
100
N−Tail

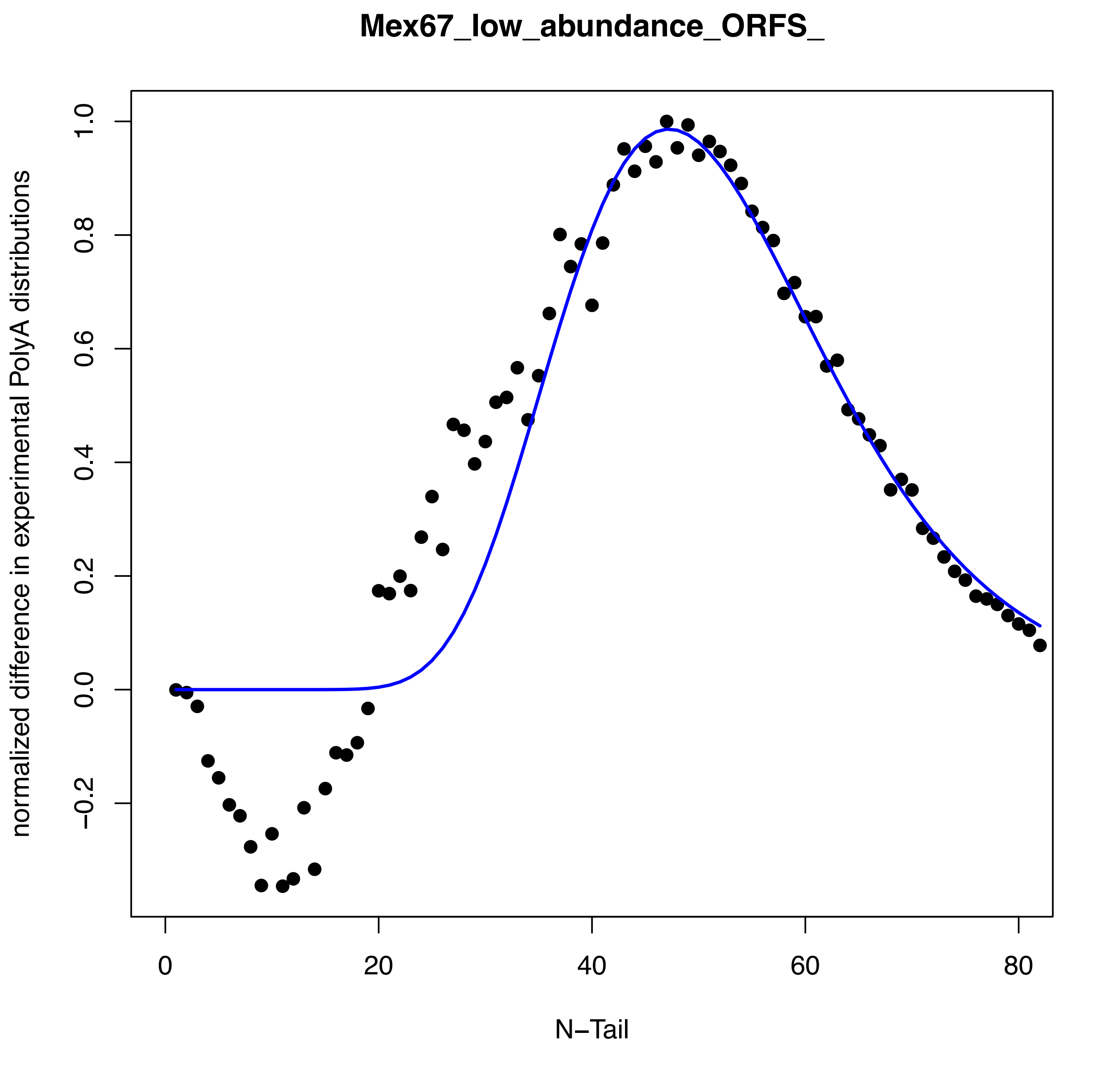
Mex67_low_abundance_ORFS_
normalized difference in experimental PolyA distributions
N−Tail
1.0
0.8
0.6
0.4
0.2
0.0
−0.2
0
20
40
60
80

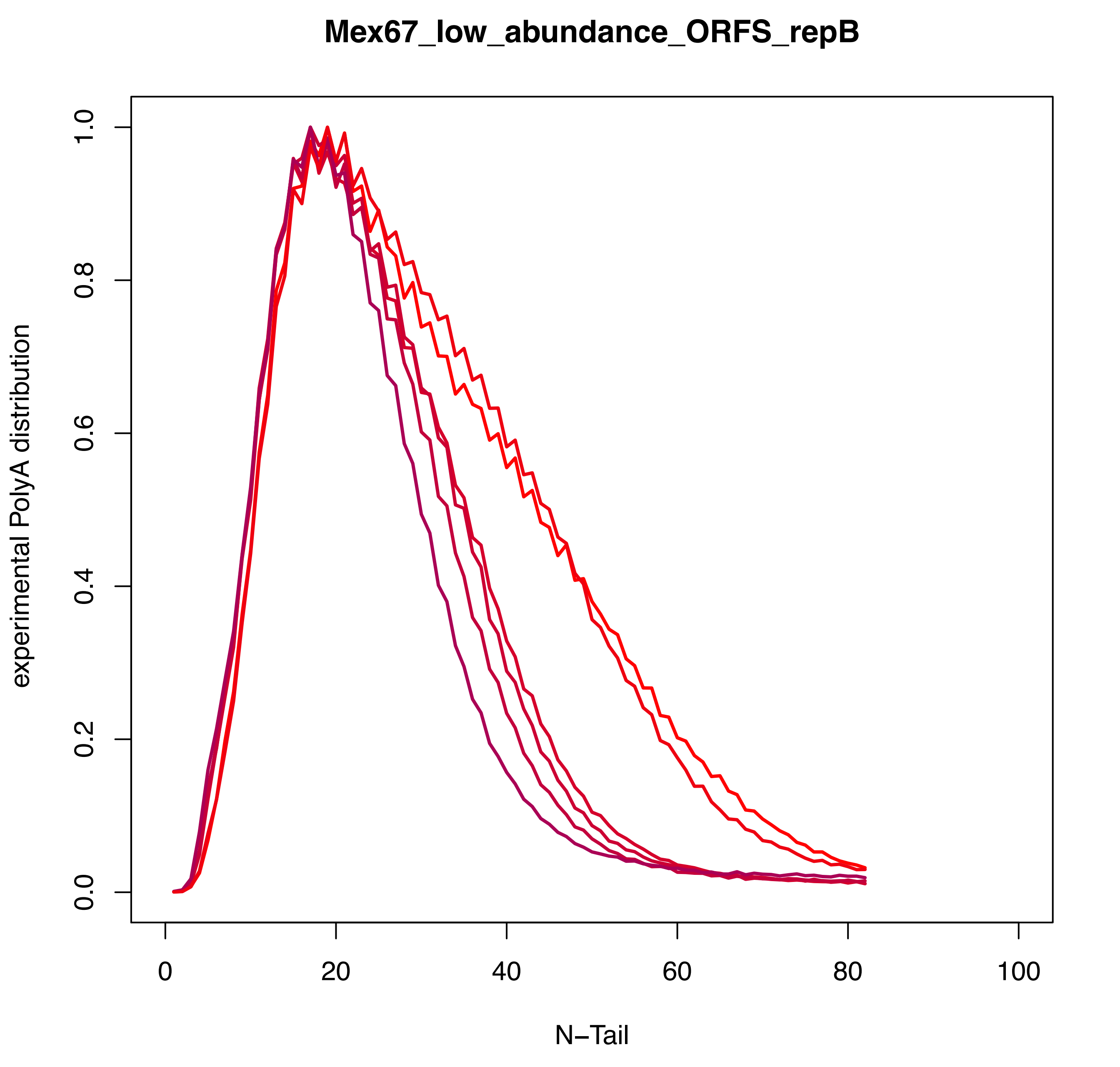

Mex67_low_abundance_ORFS_repB
experimental PolyA distribution
N−Tail
0.0
0.2
0.4
0.6
0.8
1.0
0
20
40
60
80
100

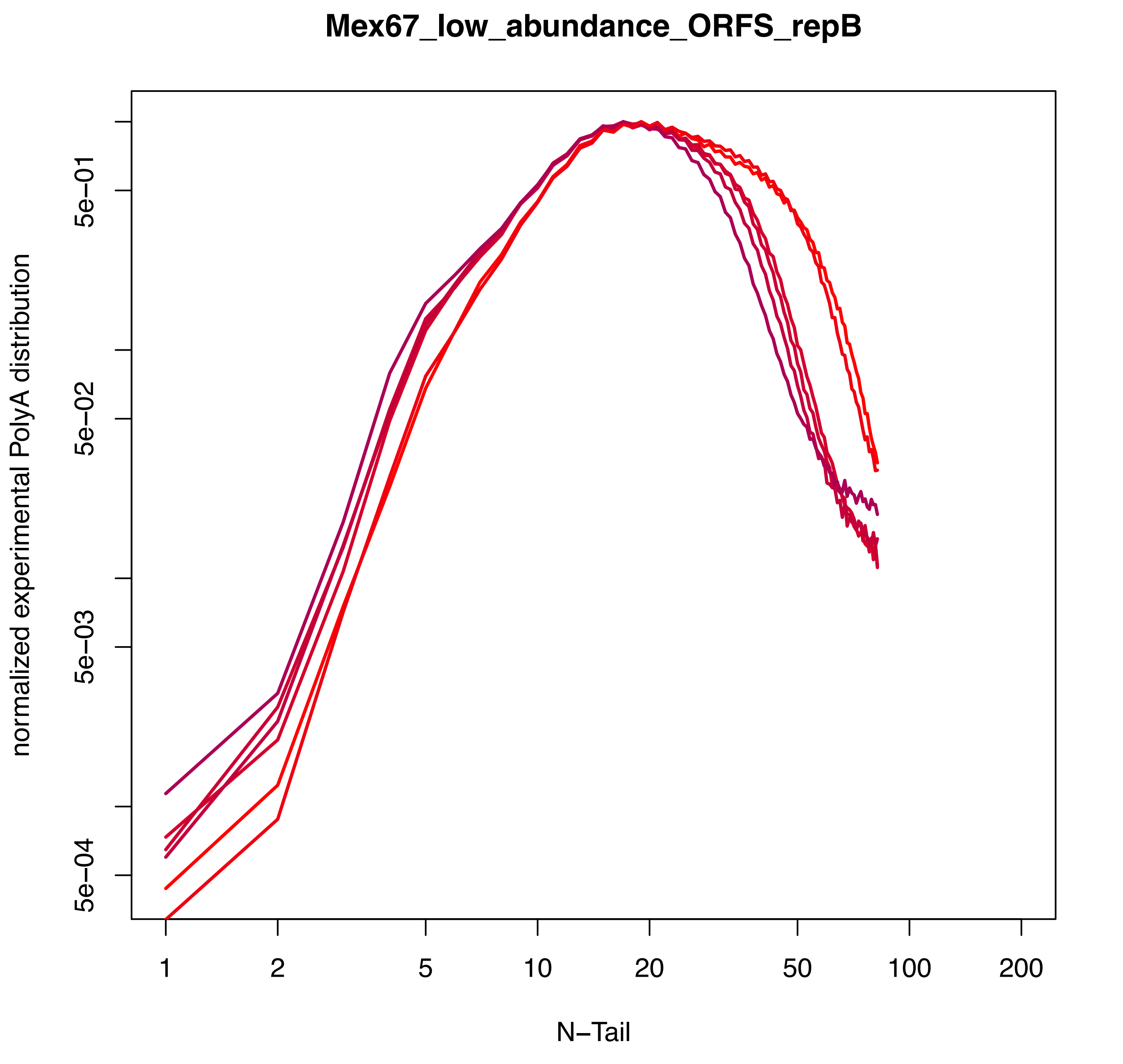

Mex67_low_abundance_ORFS_repB
normalized experimental PolyA distribution
5e−04
5e−03
5e−02
5e−01
N−Tail
1
2
5
10
20
50
100
200

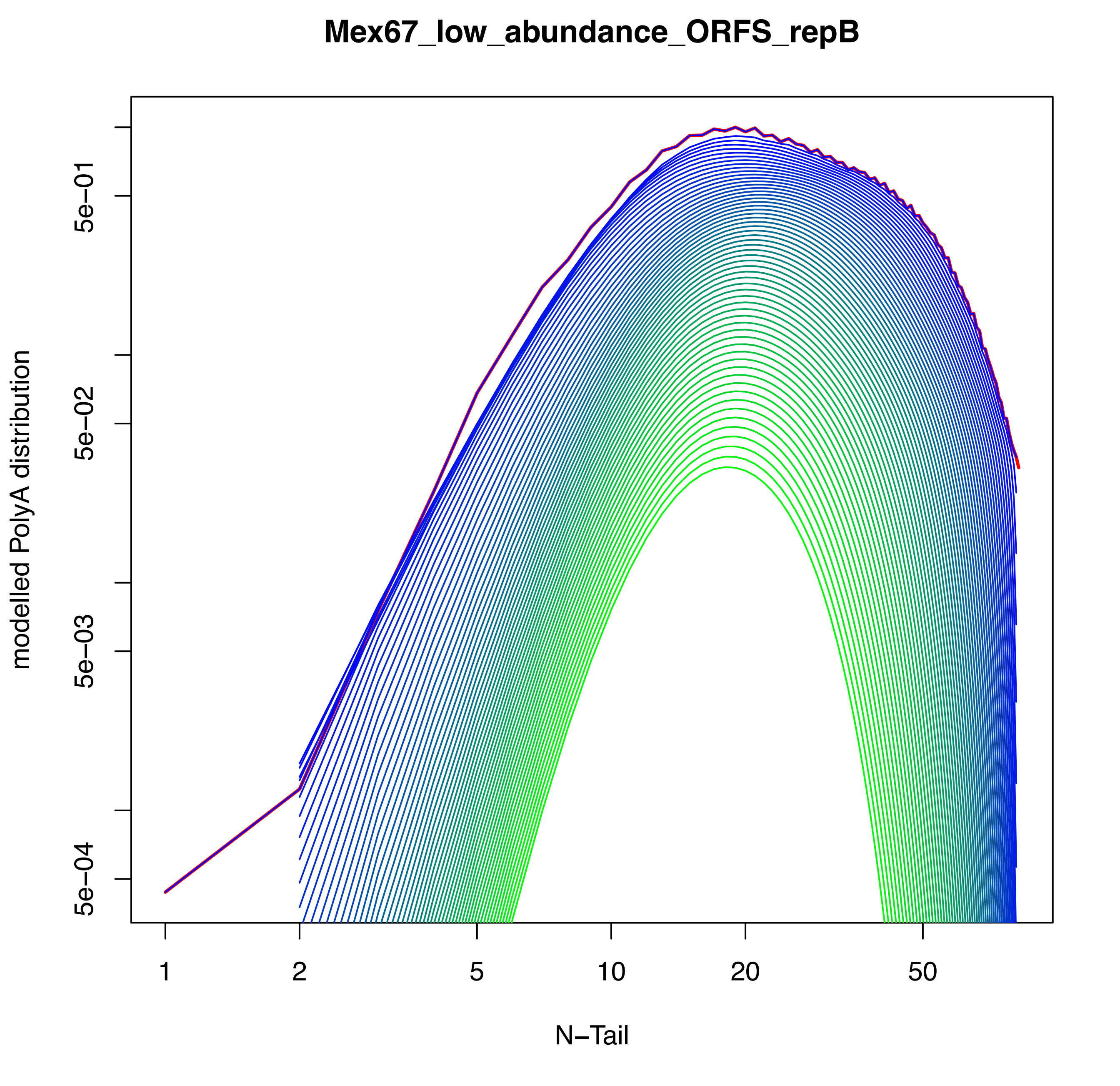

Mex67_low_abundance_ORFS_repB
modelled PolyA distribution
5e−01
5e−02
5e−03
5e−04
1
2
5
10
20
50
N−Tail

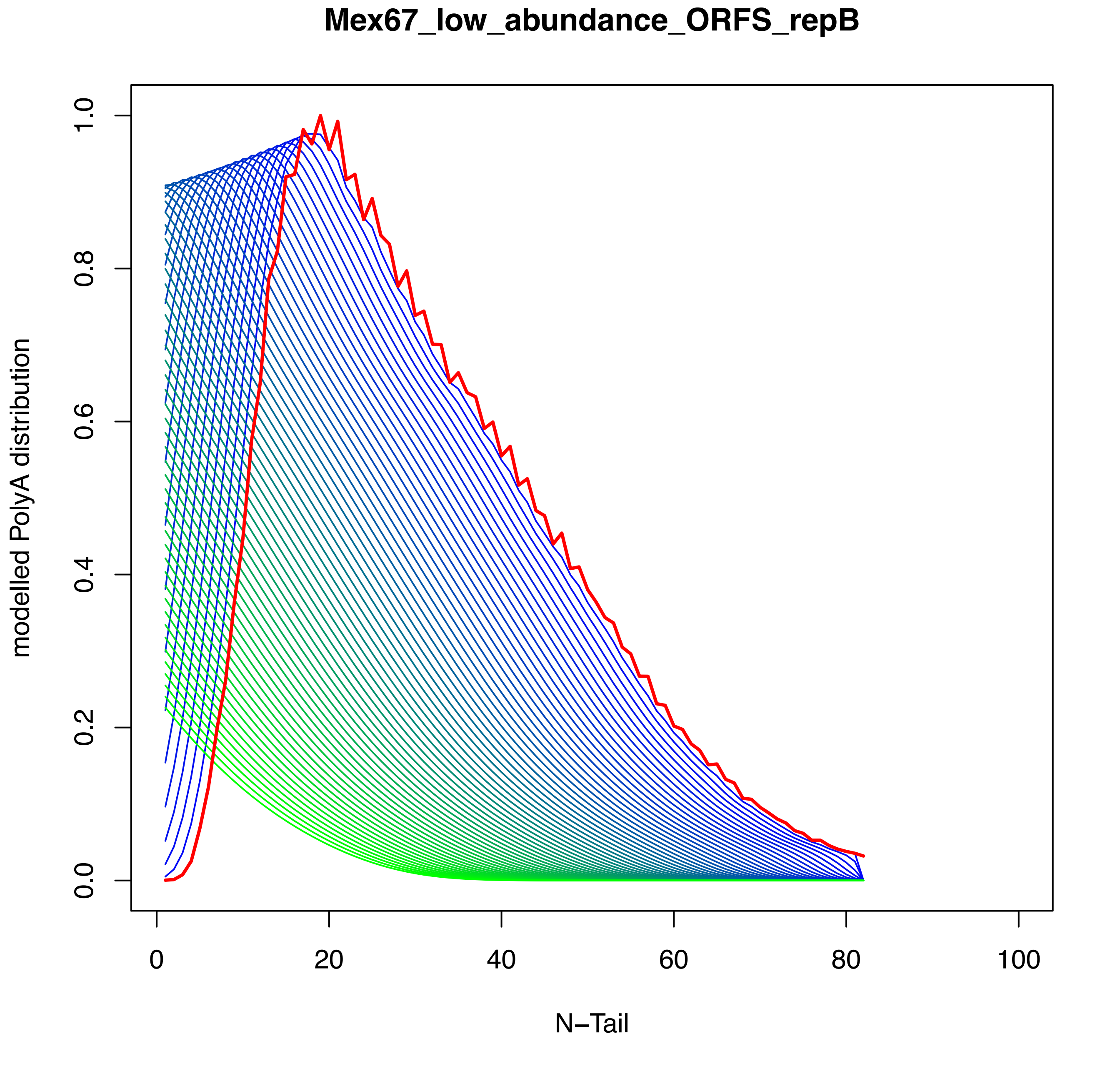

Mex67_low_abundance_ORFS_repB
modelled PolyA distribution
N−Tail
0.0
0.2
0.4
0.6
0.8
1.0
0
20
40
60
80
100

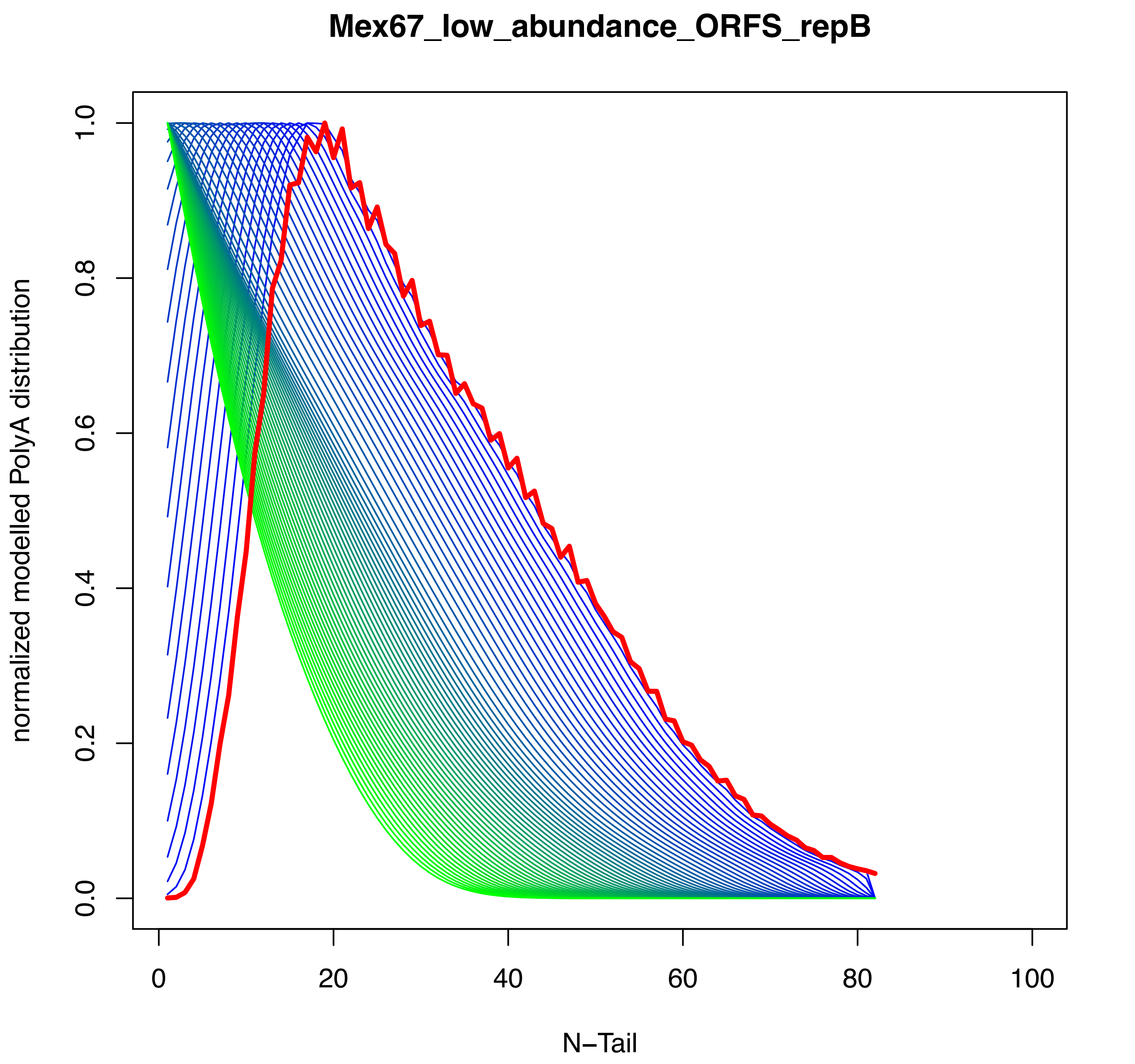
Mex67_low_abundance_ORFS_repB
normalized modelled PolyA distribution
N−Tail
0.0
0.2
0.4
0.6
0.8
1.0
0
20
40
60
80
100

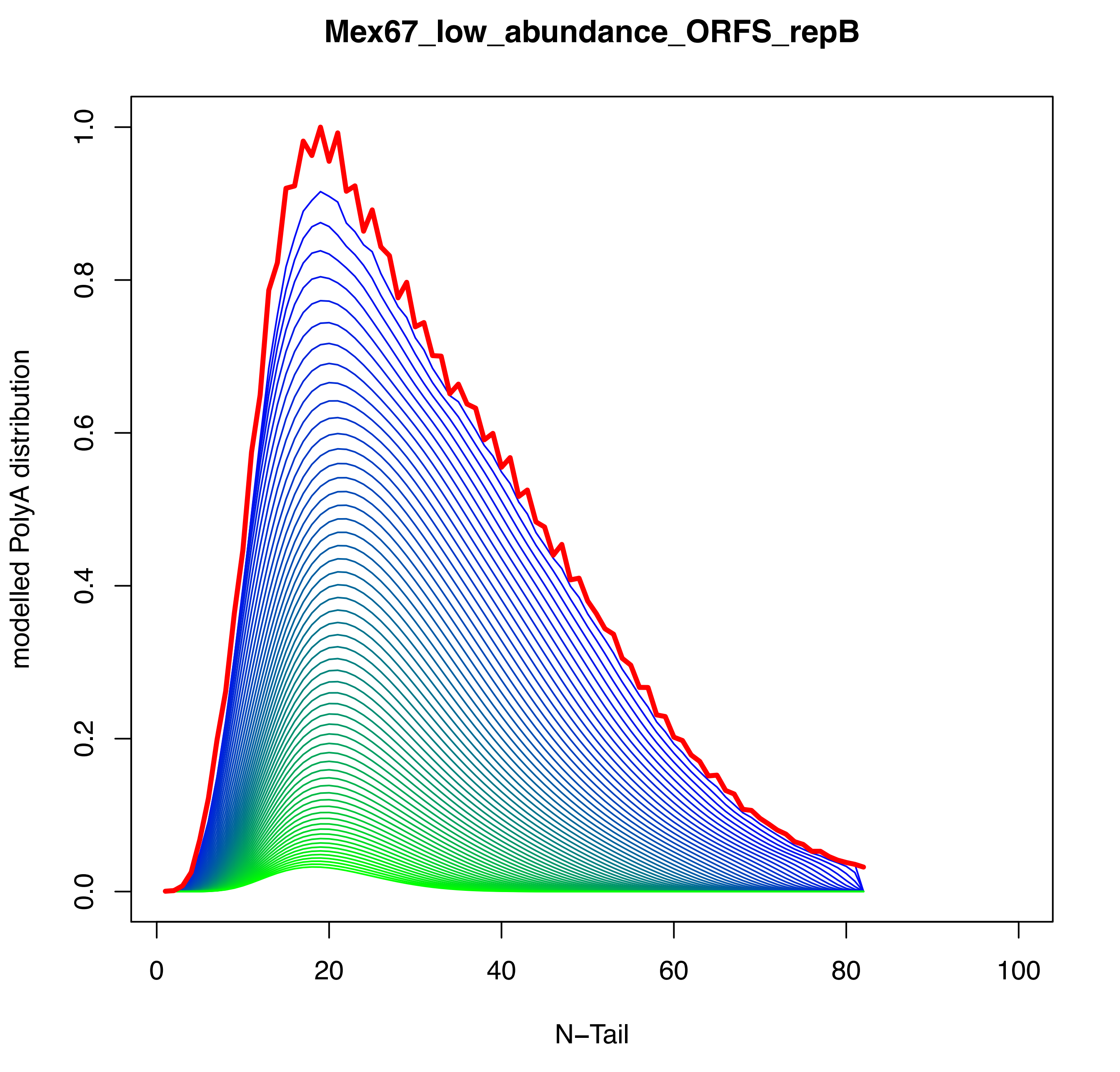
Mex67_low_abundance_ORFS_repB
modelled PolyA distribution
N−Tail
0.0
0.2
0.4
0.6
0.8
1.0
0
20
40
60
80
100

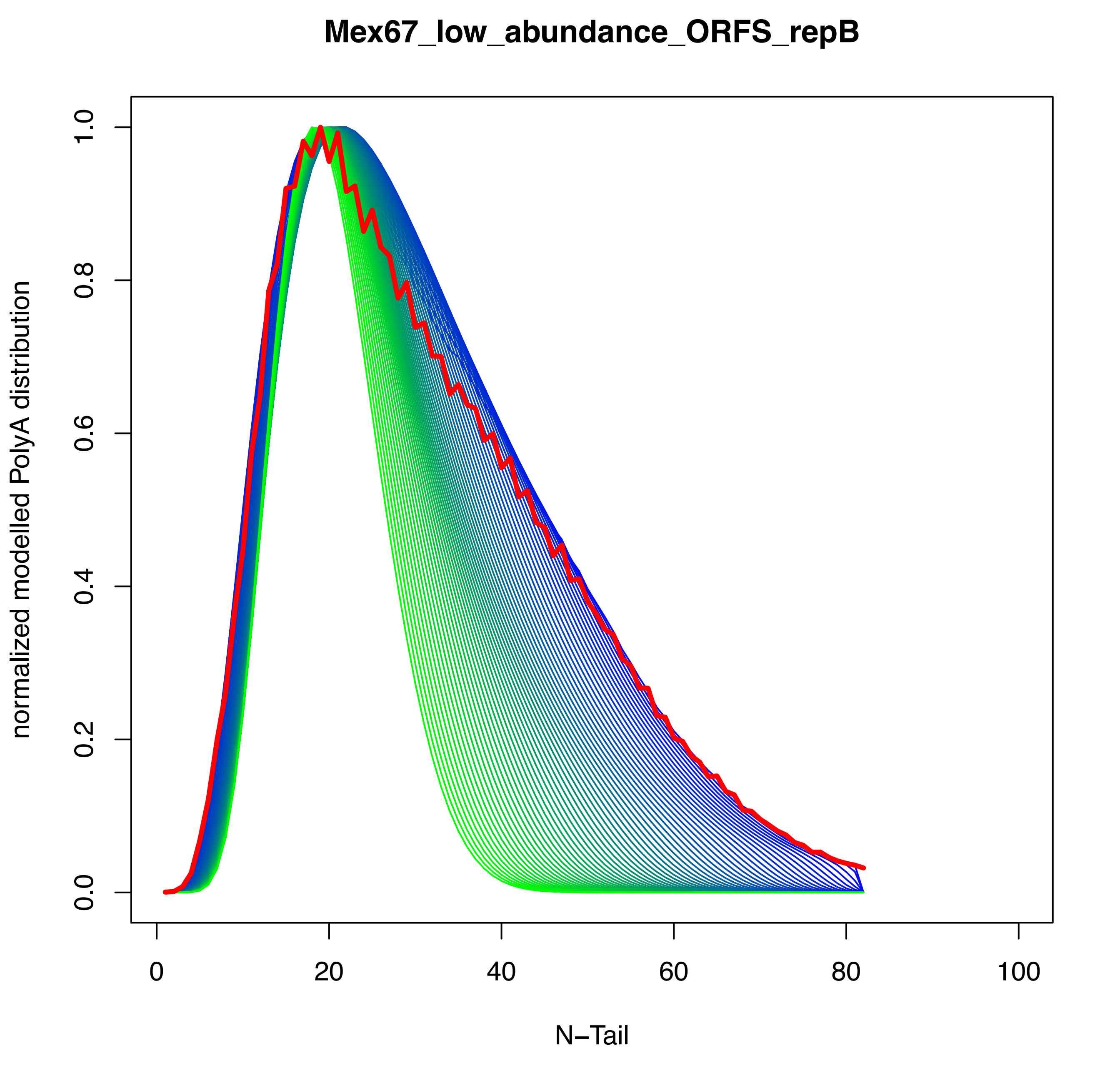

Mex67_low_abundance_ORFS_repB
normalized modelled PolyA distribution
N−Tail
0.0
0.2
0.4
0.6
0.8
1.0
0
20
40
60
80
100

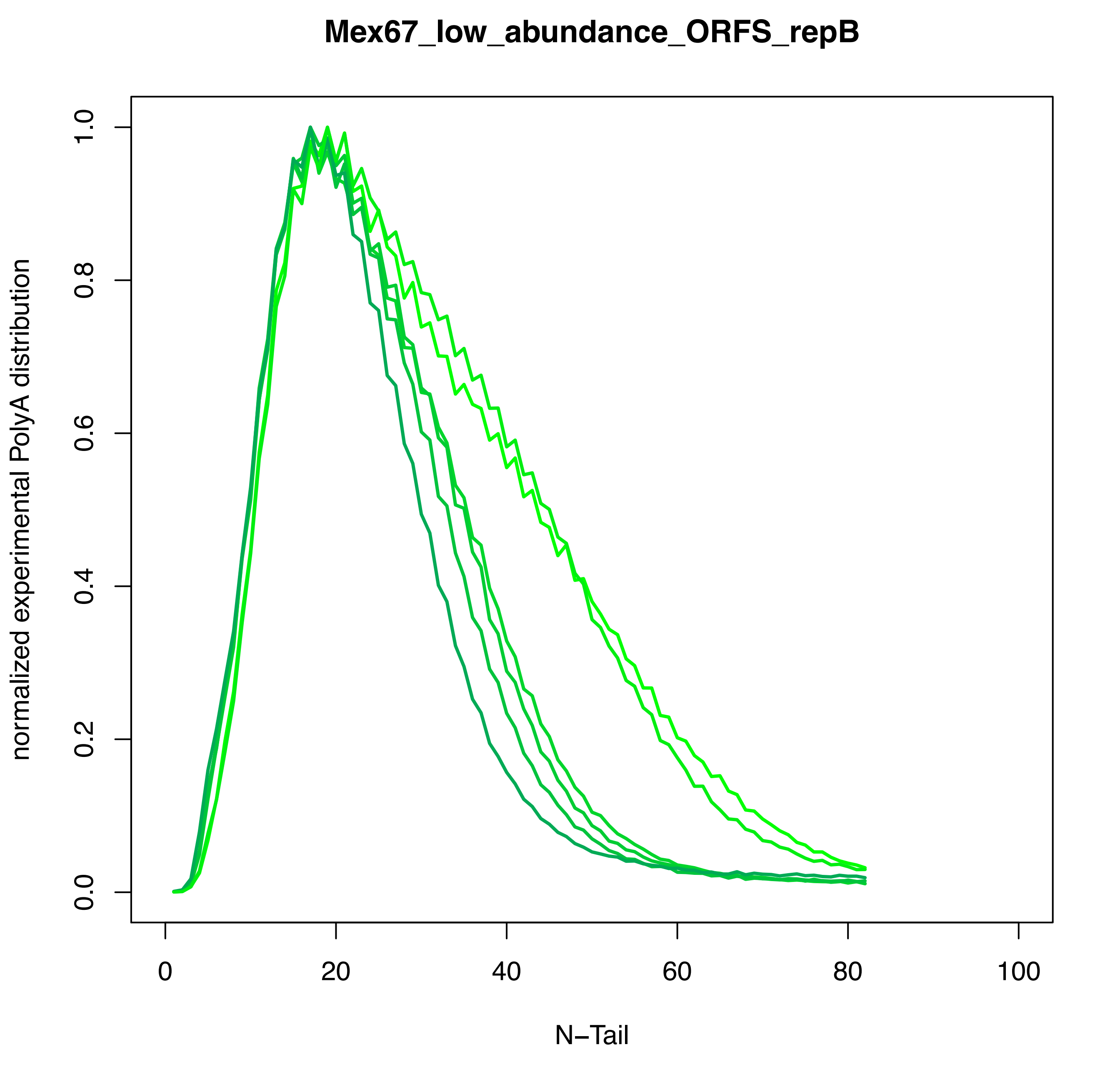

Mex67_low_abundance_ORFS_repB
normalized experimental PolyA distribution
N−Tail
0.0
0.2
0.4
0.6
0.8
1.0
0
20
40
60
80
100

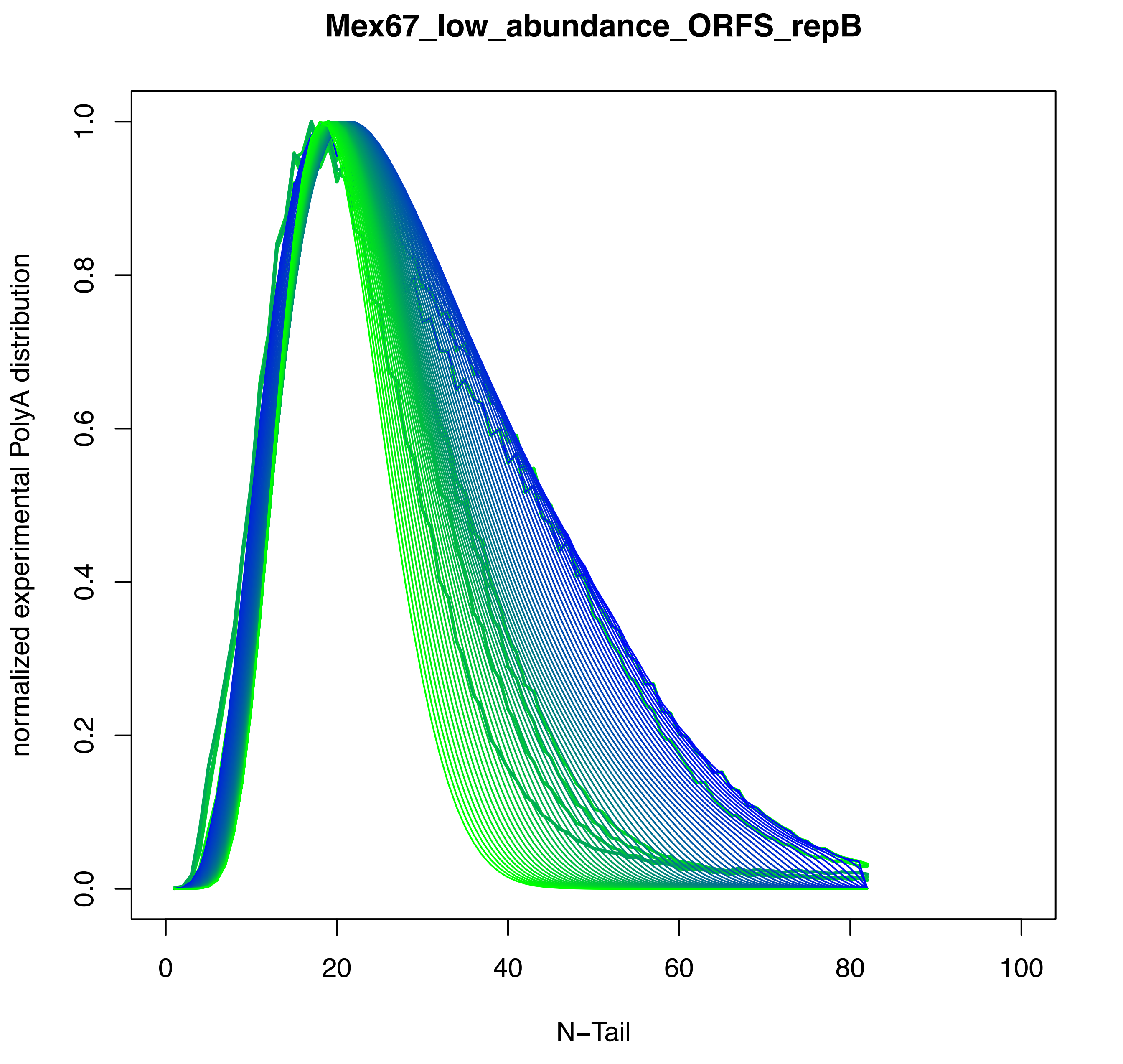
Mex67_low_abundance_ORFS_repB
normalized experimental PolyA distribution
N−Tail
0.0
0.2
0.4
0.6
0.8
1.0
0
20
40
60
80
100

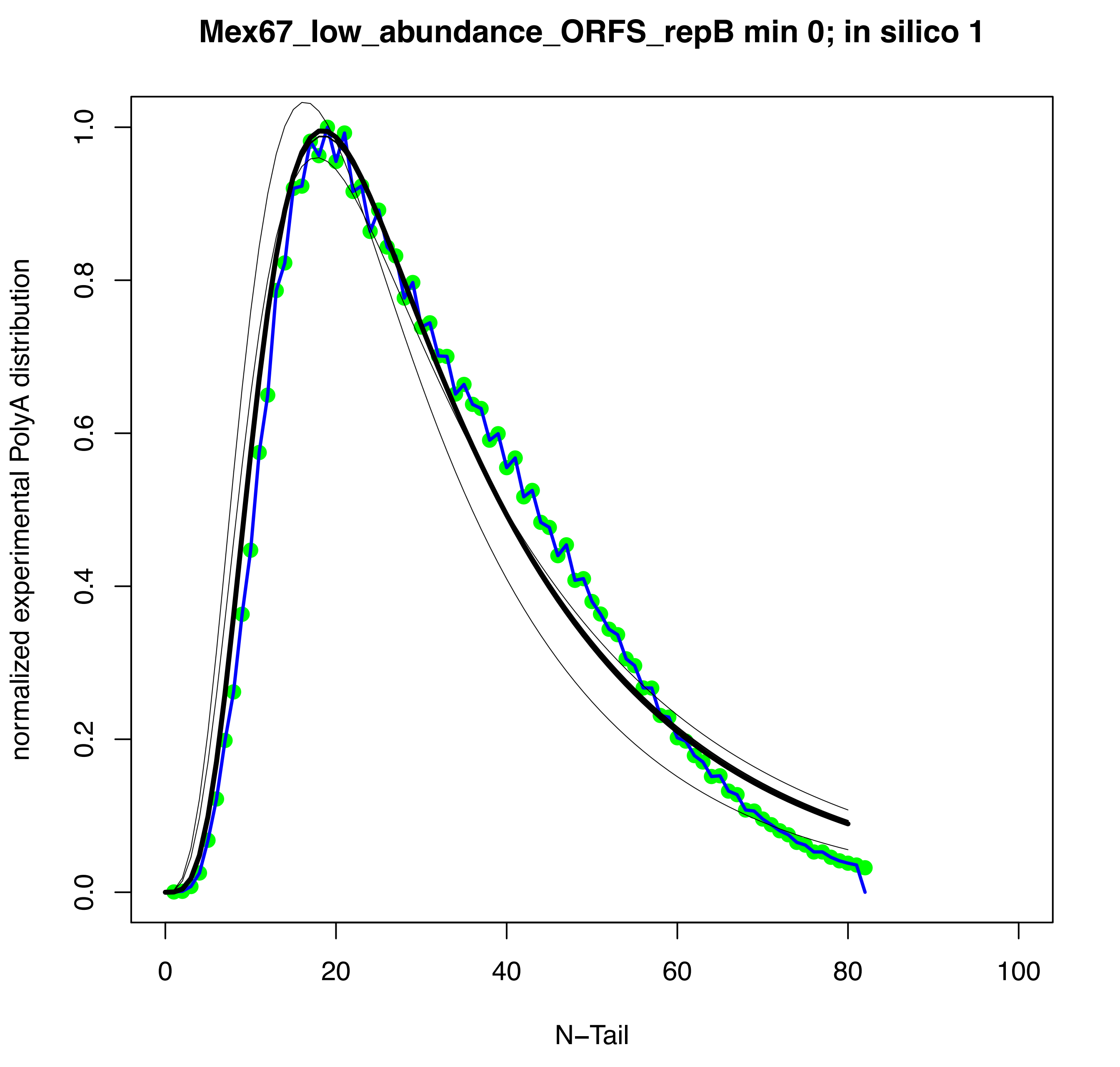

Mex67_low_abundance_ORFS_repB min 0; in silico 1
normalized experimental PolyA distribution
N−Tail
0.0
0.2
0.4
0.6
0.8
1.0
0
20
40
60
80
100

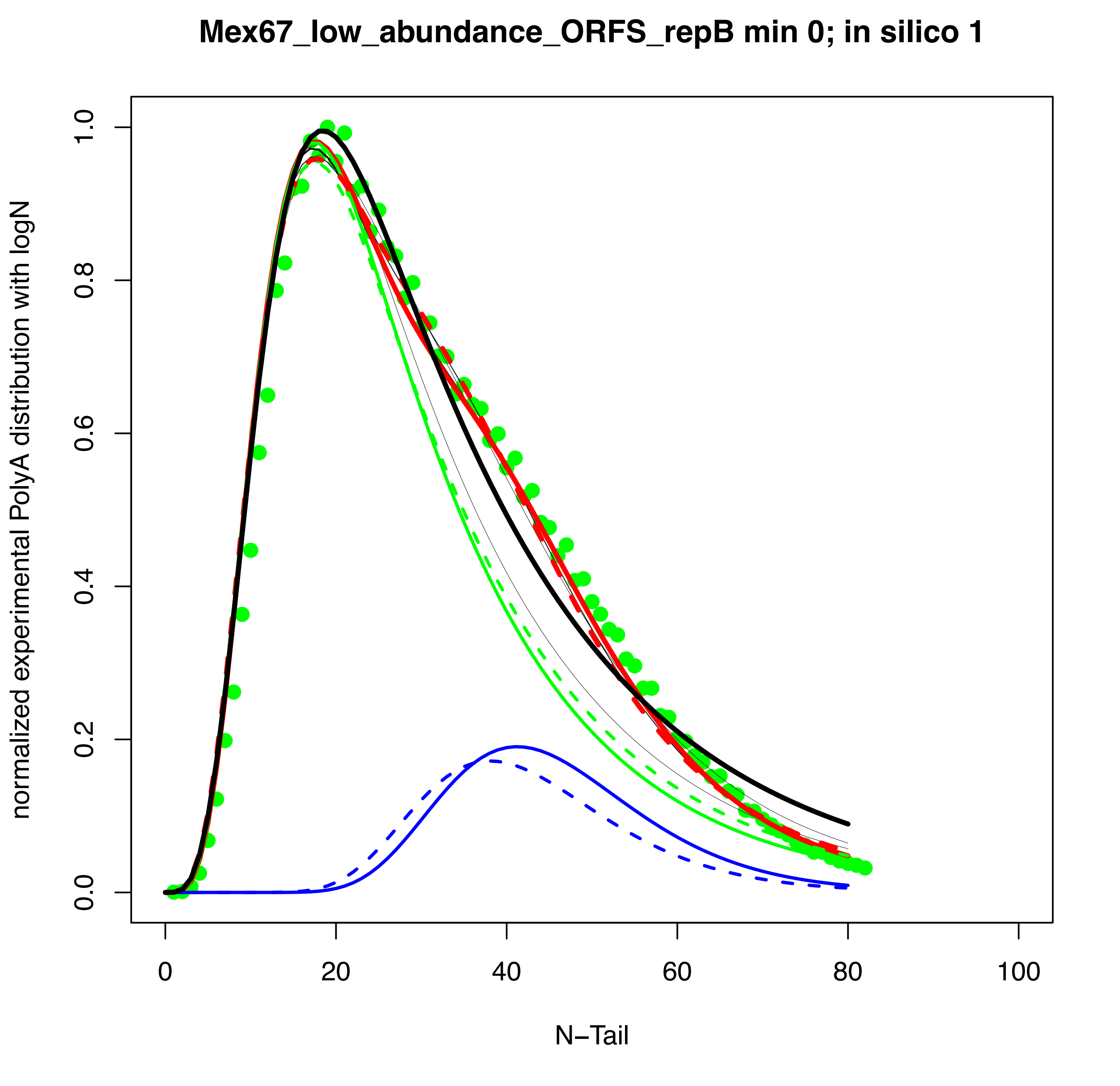

Mex67_low_abundance_ORFS_repB min 0; in silico 1
normalized experimental PolyA distribution with logN
N−Tail
0.0
0.2
0.4
0.6
0.8
1.0
0
20
40
60
80
100

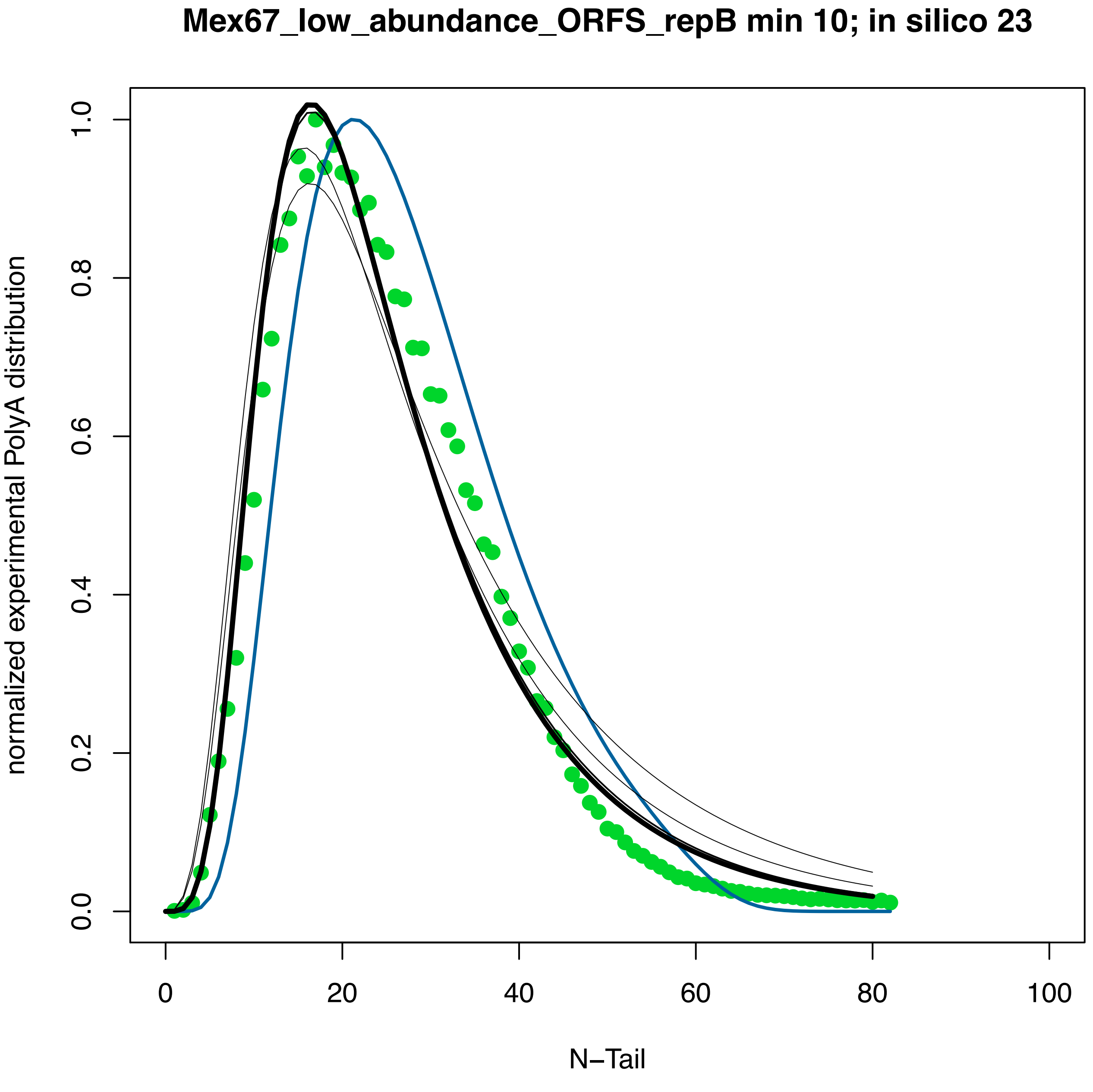

Mex67_low_abundance_ORFS_repB min 10; in silico 23
normalized experimental PolyA distribution
N−Tail
0.0
0.2
0.4
0.6
0.8
1.0
0
20
40
60
80
100

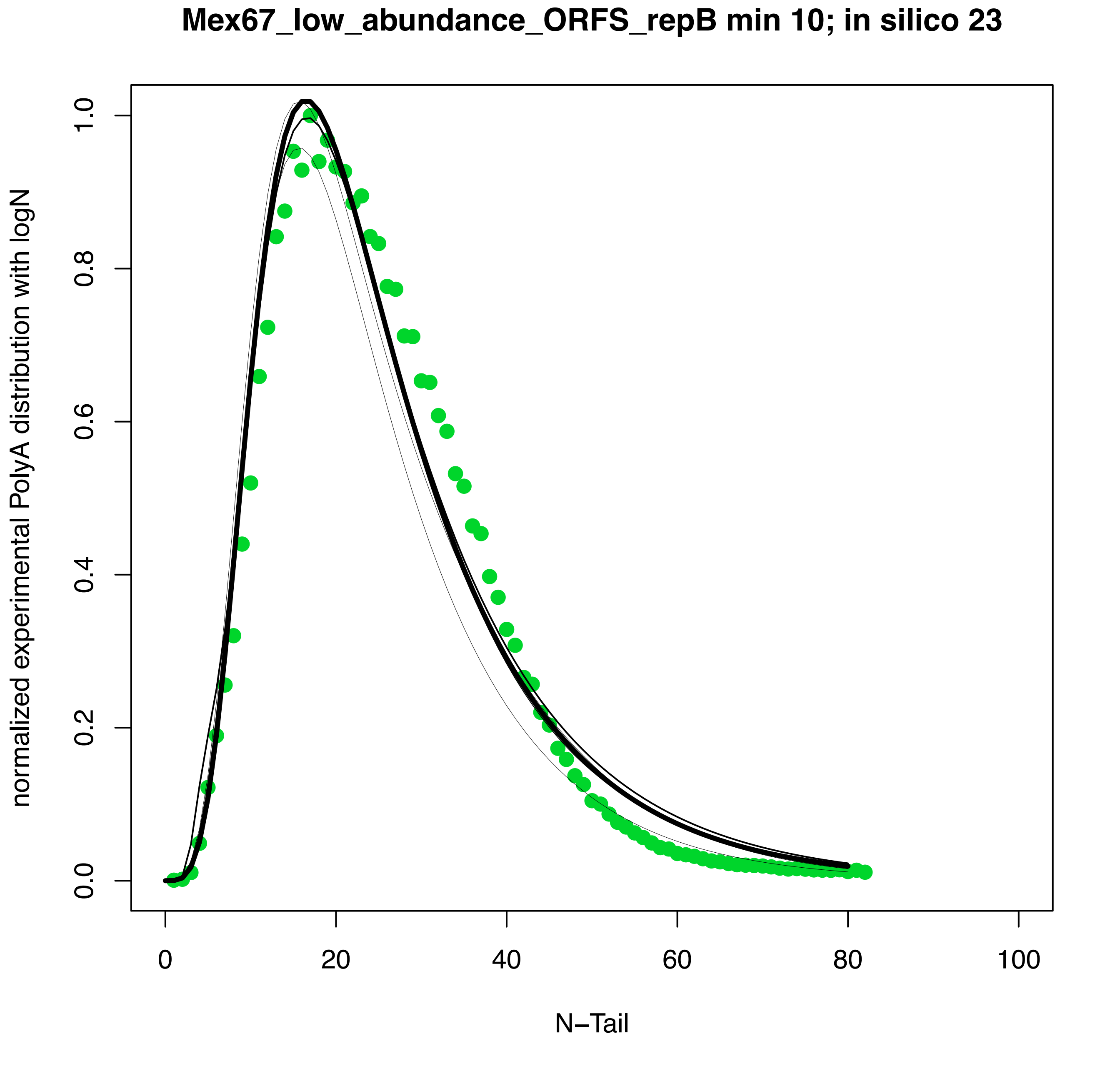

Mex67_low_abundance_ORFS_repB min 10; in silico 23
normalized experimental PolyA distribution with logN
0.0
0.2
0.4
0.6
0.8
1.0
0
20
40
60
80
100
N−Tail

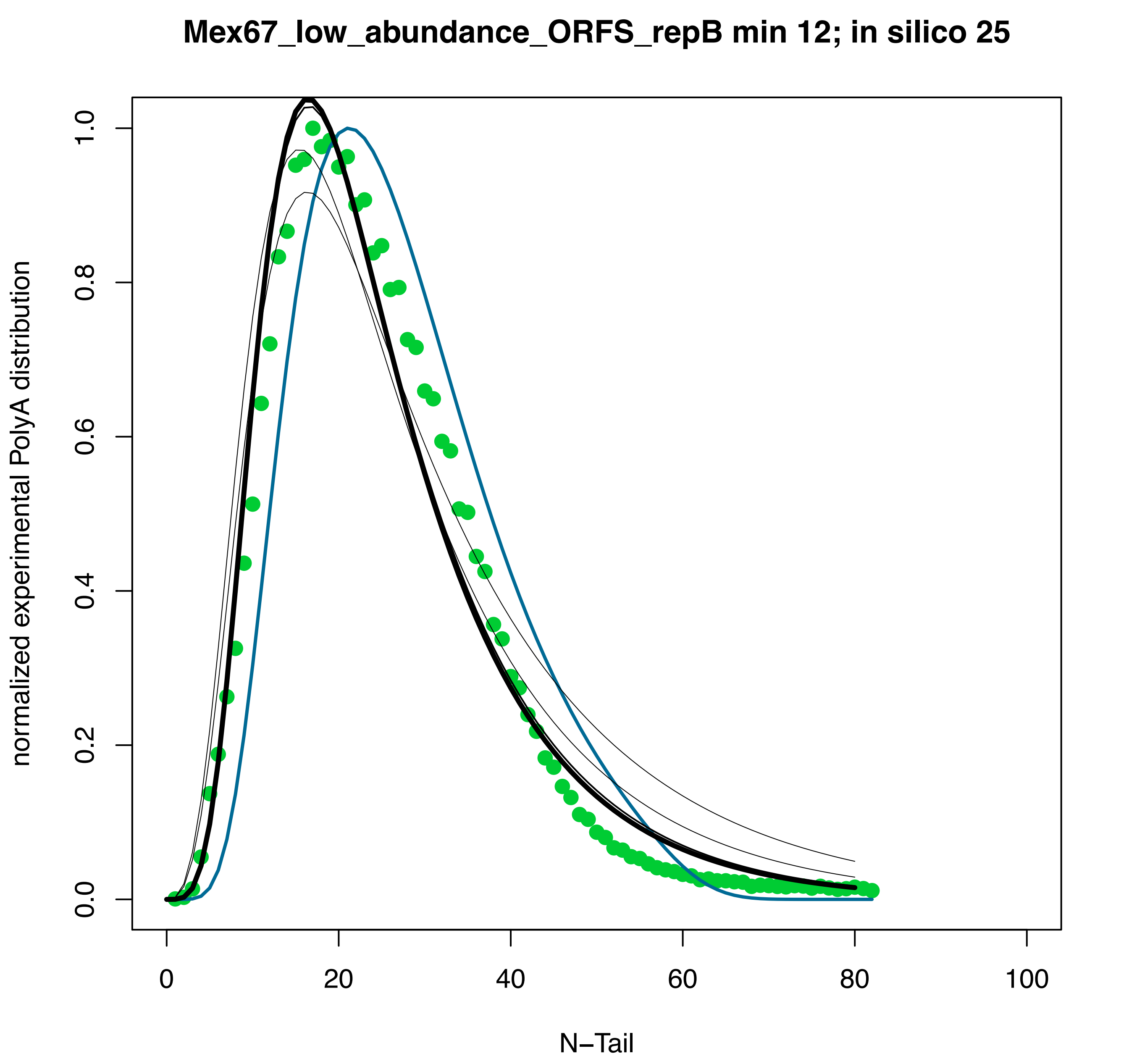

Mex67_low_abundance_ORFS_repB min 12; in silico 25
normalized experimental PolyA distribution
N−Tail
0.0
0.2
0.4
0.6
0.8
1.0
0
20
40
60
80
100

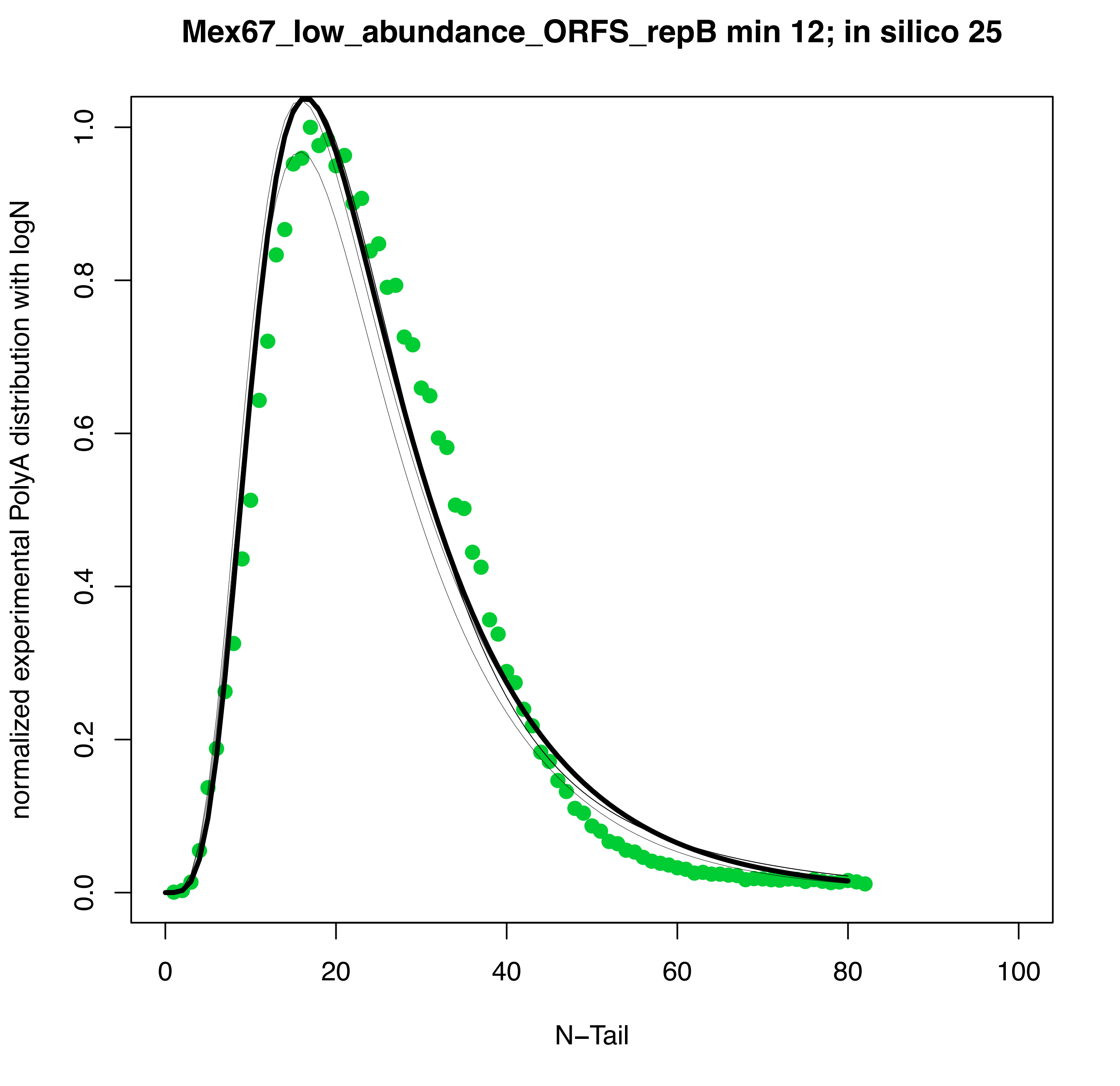
Mex67_low_abundance_ORFS_repB min 12; in silico 25
normalized experimental PolyA distribution with logN
N−Tail
0.0
0.2
0.4
0.6
0.8
1.0
0
20
40
60
80
100

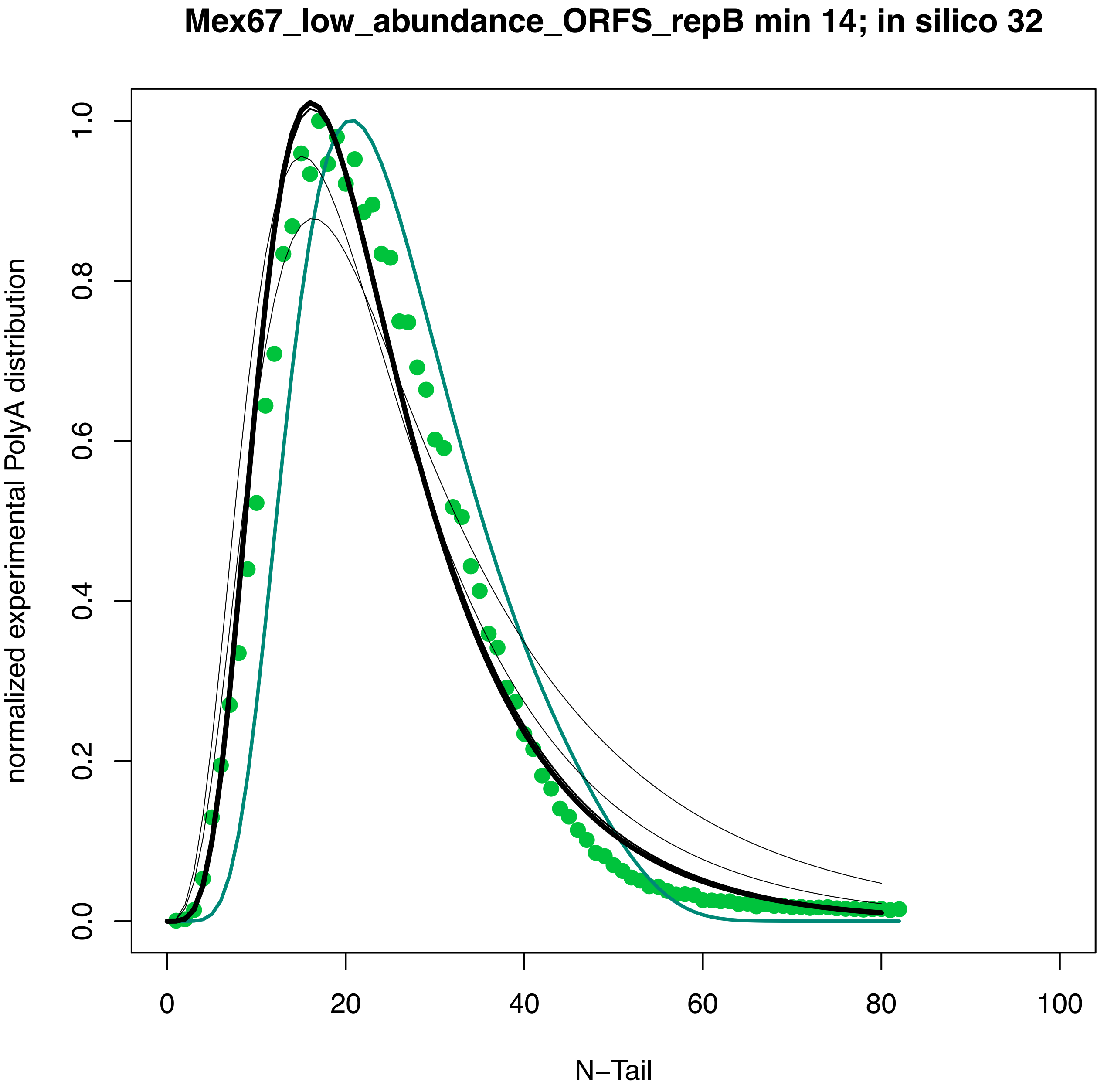

Mex67_low_abundance_ORFS_repB min 14; in silico 32
normalized experimental PolyA distribution
N−Tail
0.0
0.2
0.4
0.6
0.8
1.0
0
20
40
60
80
100

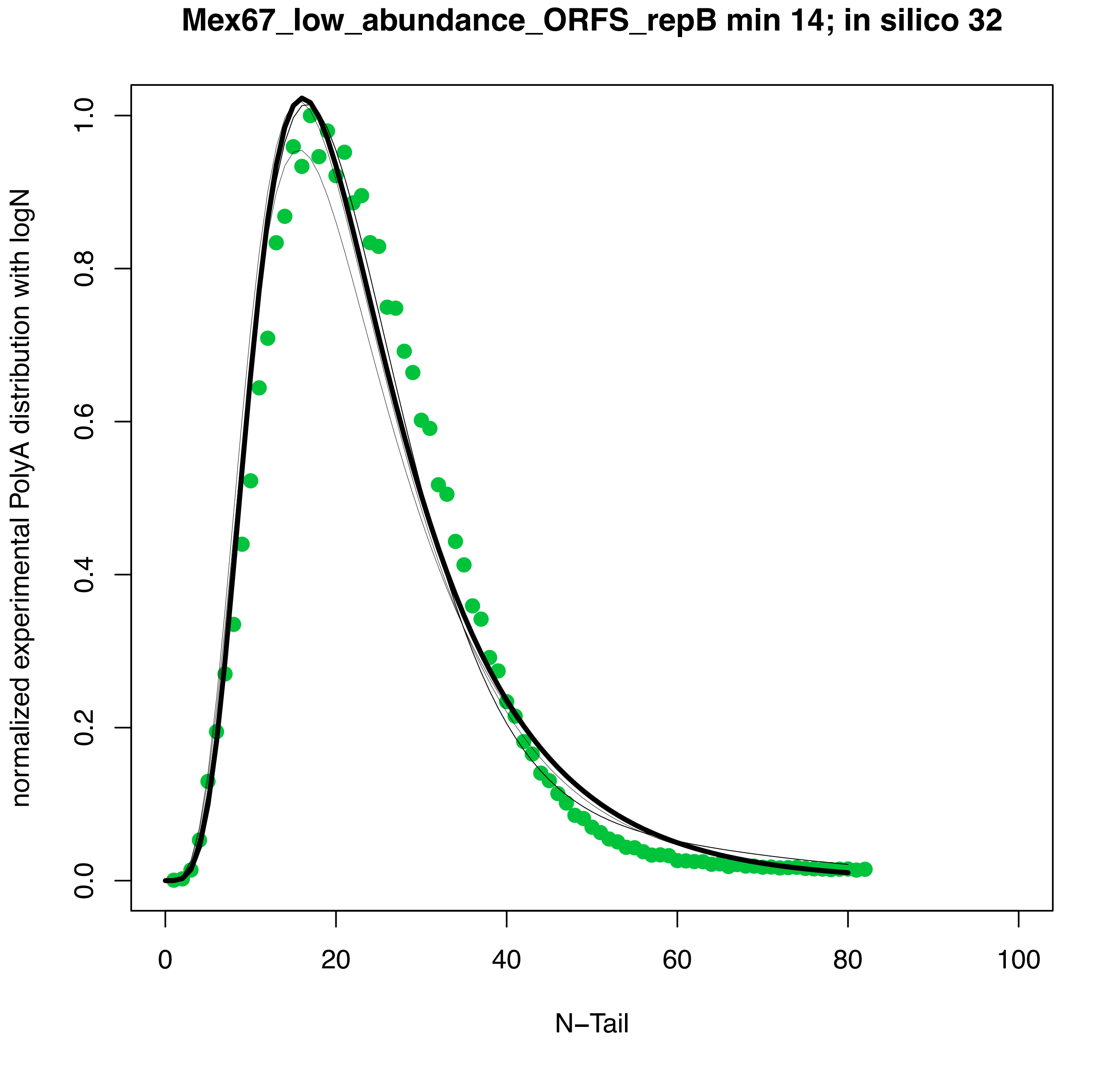
Mex67_low_abundance_ORFS_repB min 14; in silico 32
normalized experimental PolyA distribution with logN
N−Tail
0.0
0.2
0.4
0.6
0.8
1.0
0
20
40
60
80
100

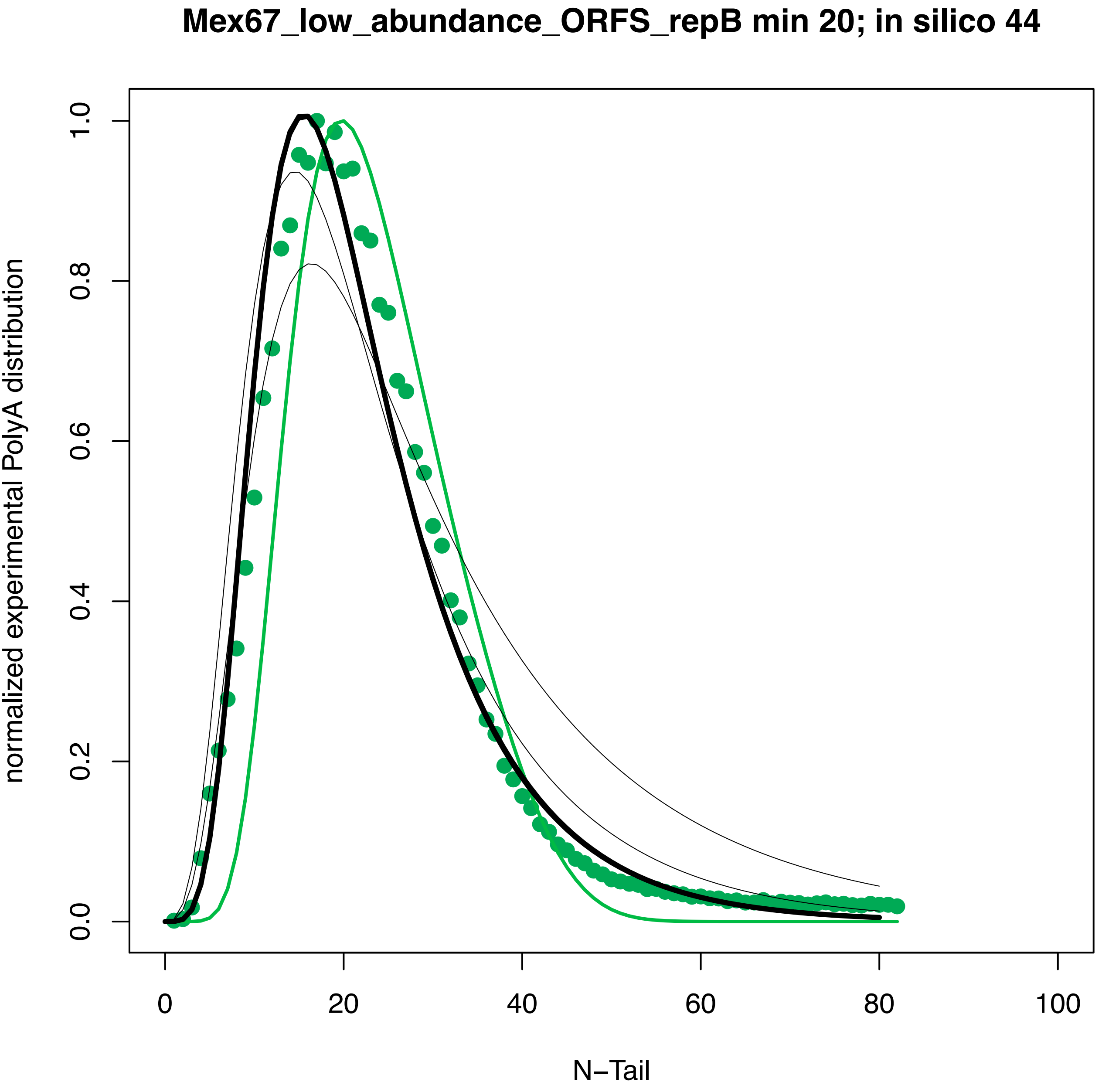
Mex67_low_abundance_ORFS_repB min 20; in silico 44
normalized experimental PolyA distribution
N−Tail
0.0
0.2
0.4
0.6
0.8
1.0
0
20
40
60
80
100

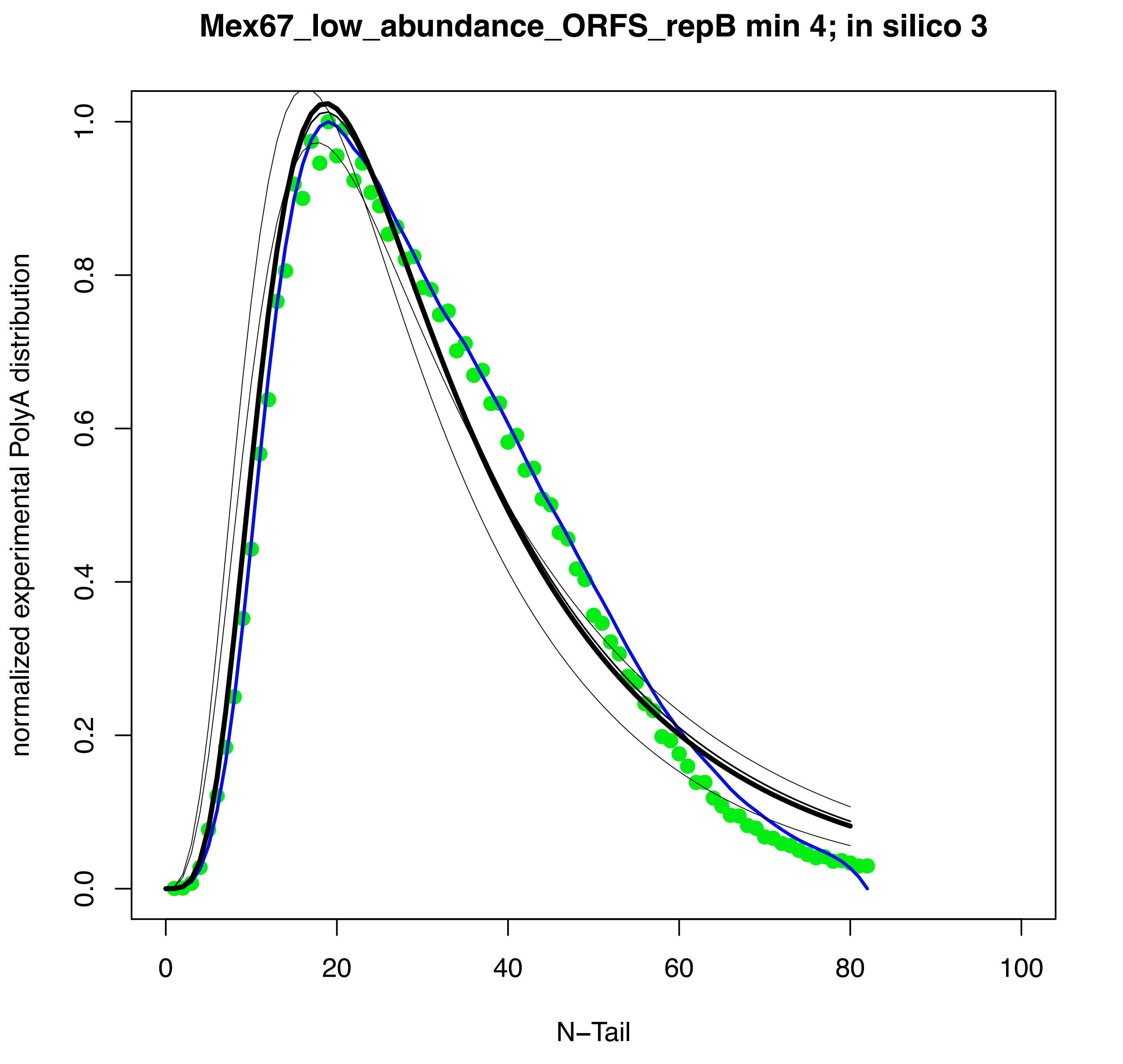

Mex67_low_abundance_ORFS_repB min 4; in silico 3
normalized experimental PolyA distribution
N−Tail
0.0
0.2
0.4
0.6
0.8
1.0
0
20
40
60
80
100

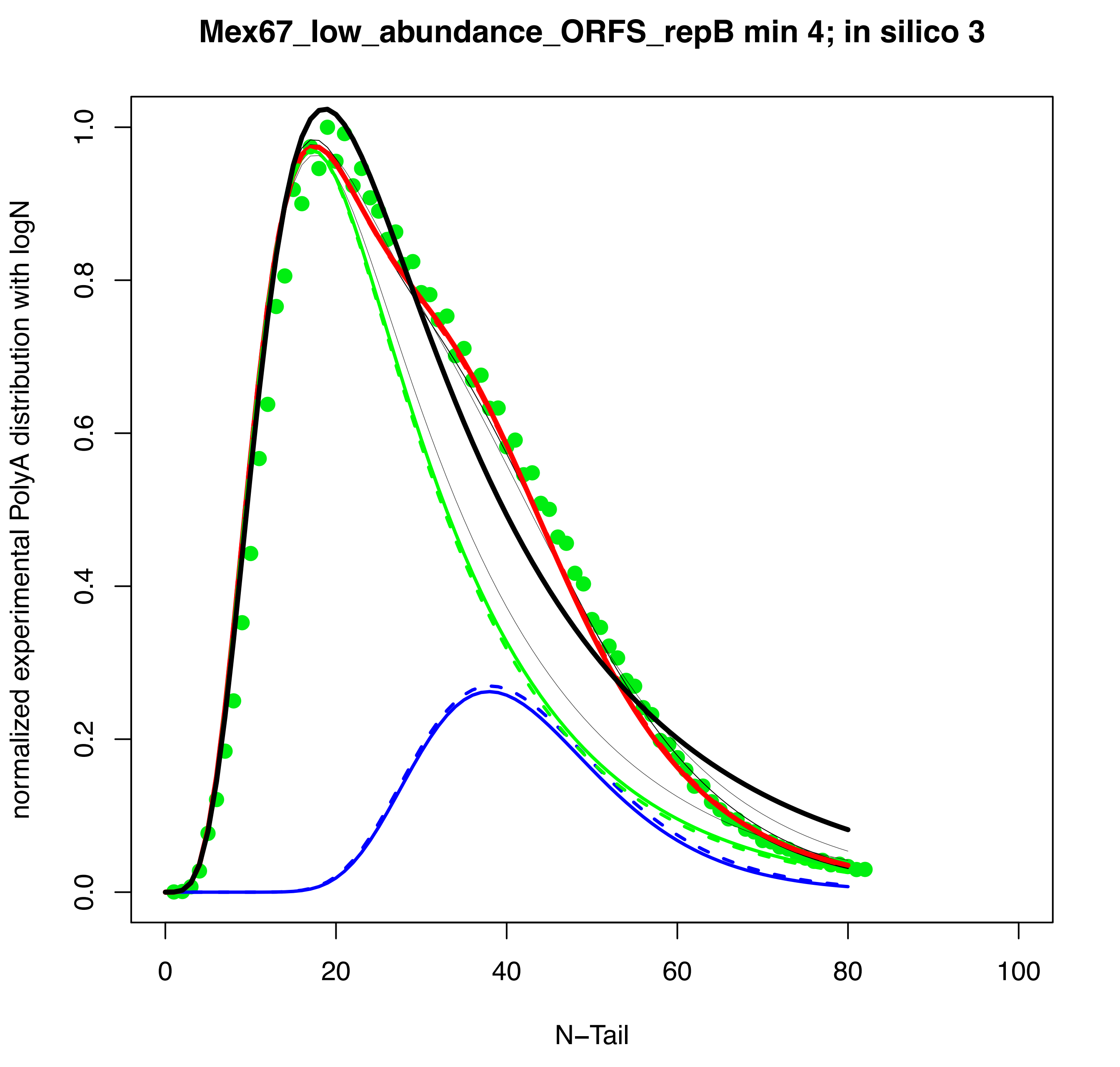

Mex67_low_abundance_ORFS_repB min 4; in silico 3
normalized experimental PolyA distribution with logN
N−Tail
0.0
0.2
0.4
0.6
0.8
1.0
0
20
40
60
80
100

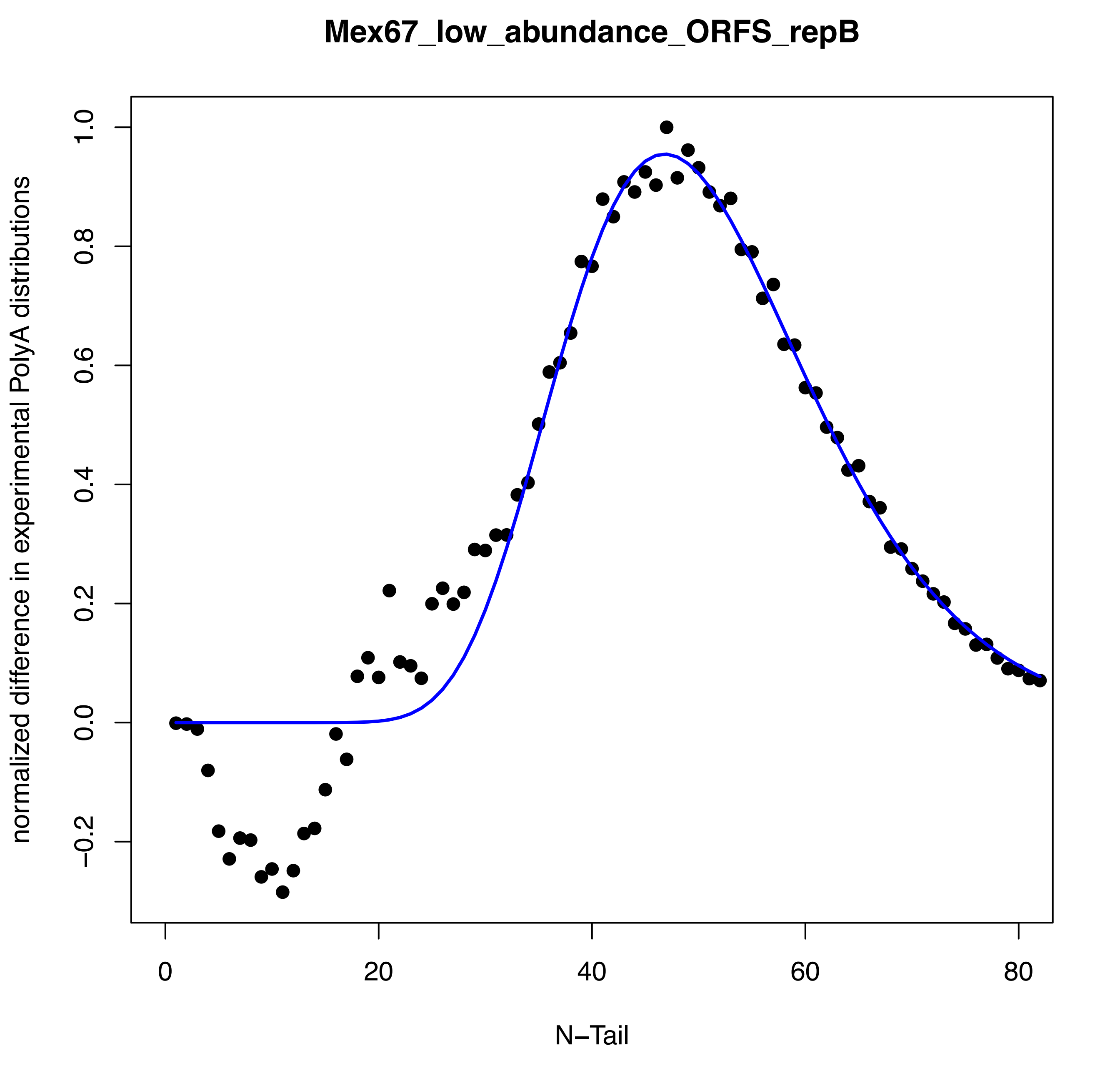

Mex67_low_abundance_ORFS_repB
normalized difference in experimental PolyA distributions
N−Tail
0
20
40
60
80
−0.2
0.0
0.2
0.4
0.6
0.8
1.0

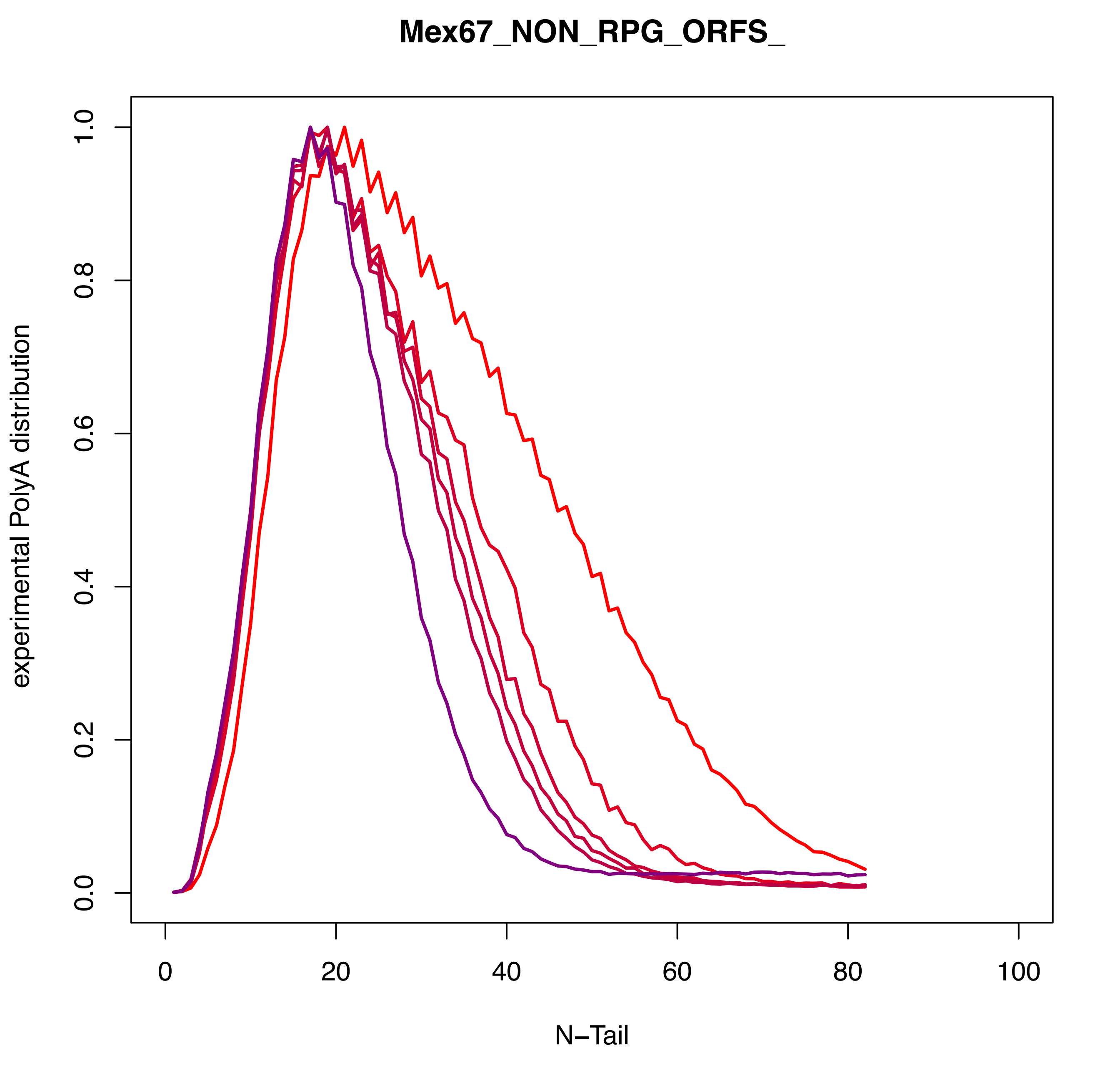
Mex67_NON_RPG_ORFS_
experimental PolyA distribution
N−Tail
0.0
0.2
0.4
0.6
0.8
1.0
0
20
40
60
80
100

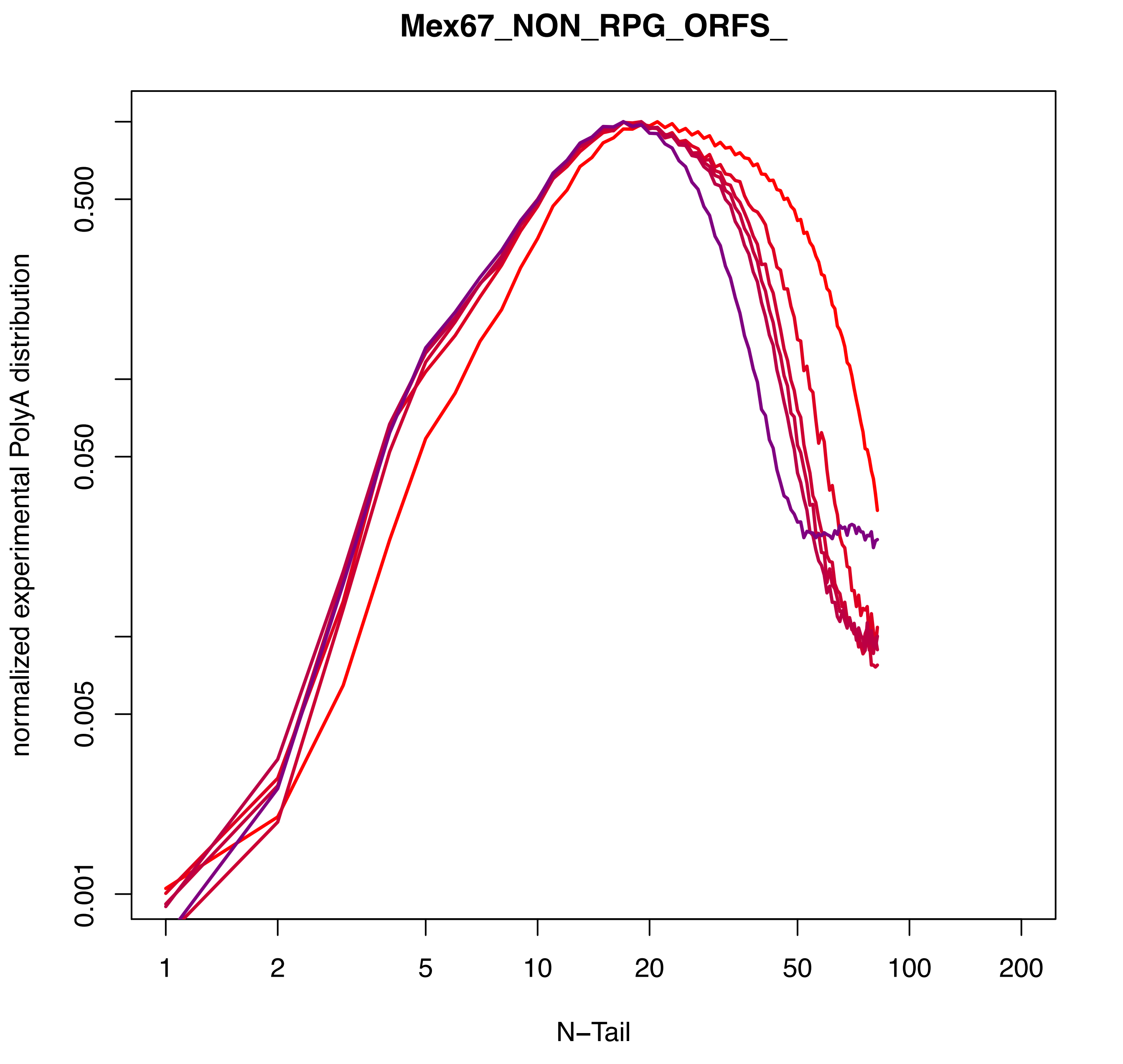

Mex67_NON_RPG_ORFS_
normalized experimental PolyA distribution
0.001
0.005
0.050
0.500
1
2
5
10
20
50
100
200
N−Tail

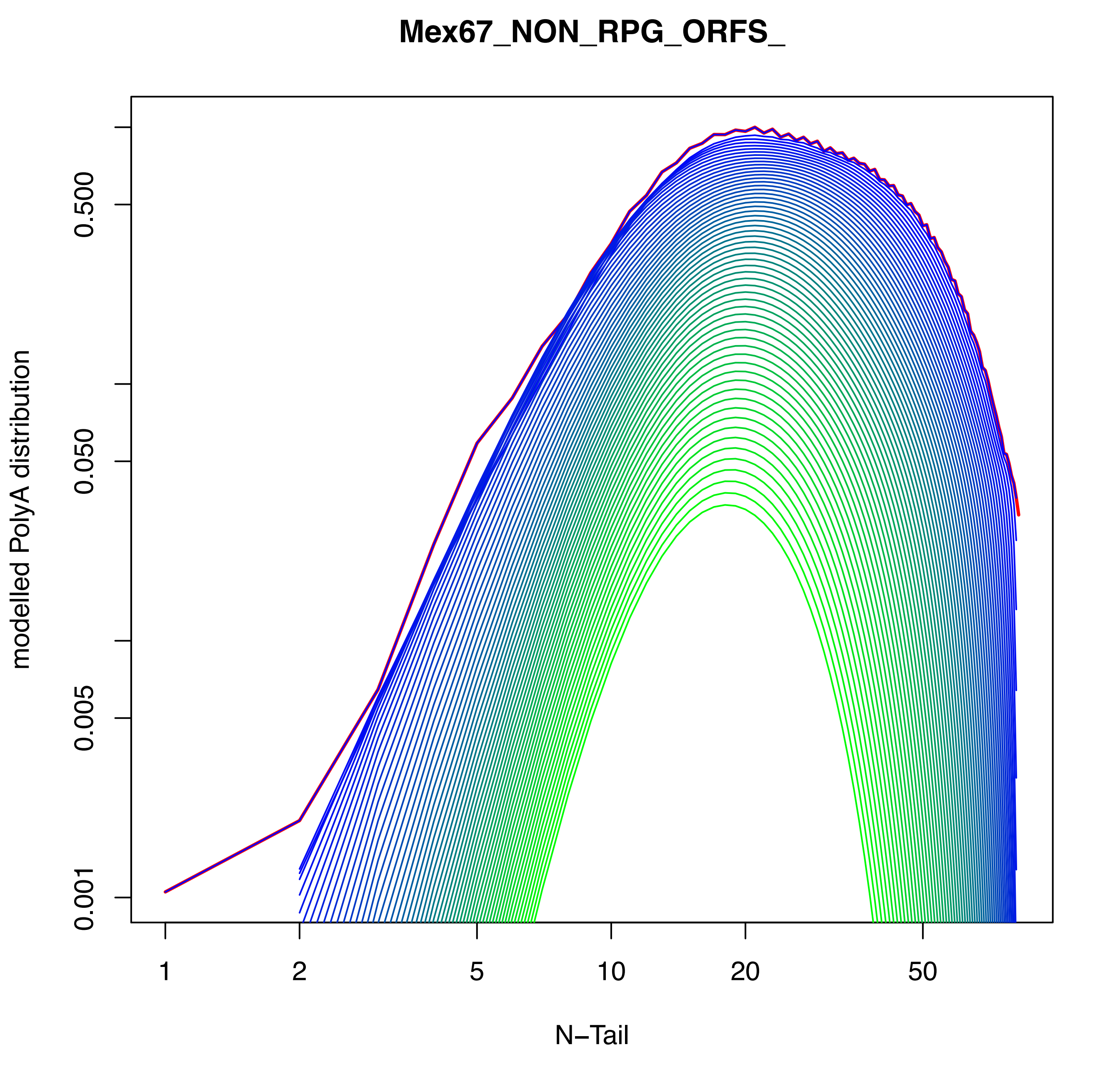

Mex67_NON_RPG_ORFS_
modelled PolyA distribution
0.001
0.005
0.050
0.500
1
2
5
10
20
50
N−Tail

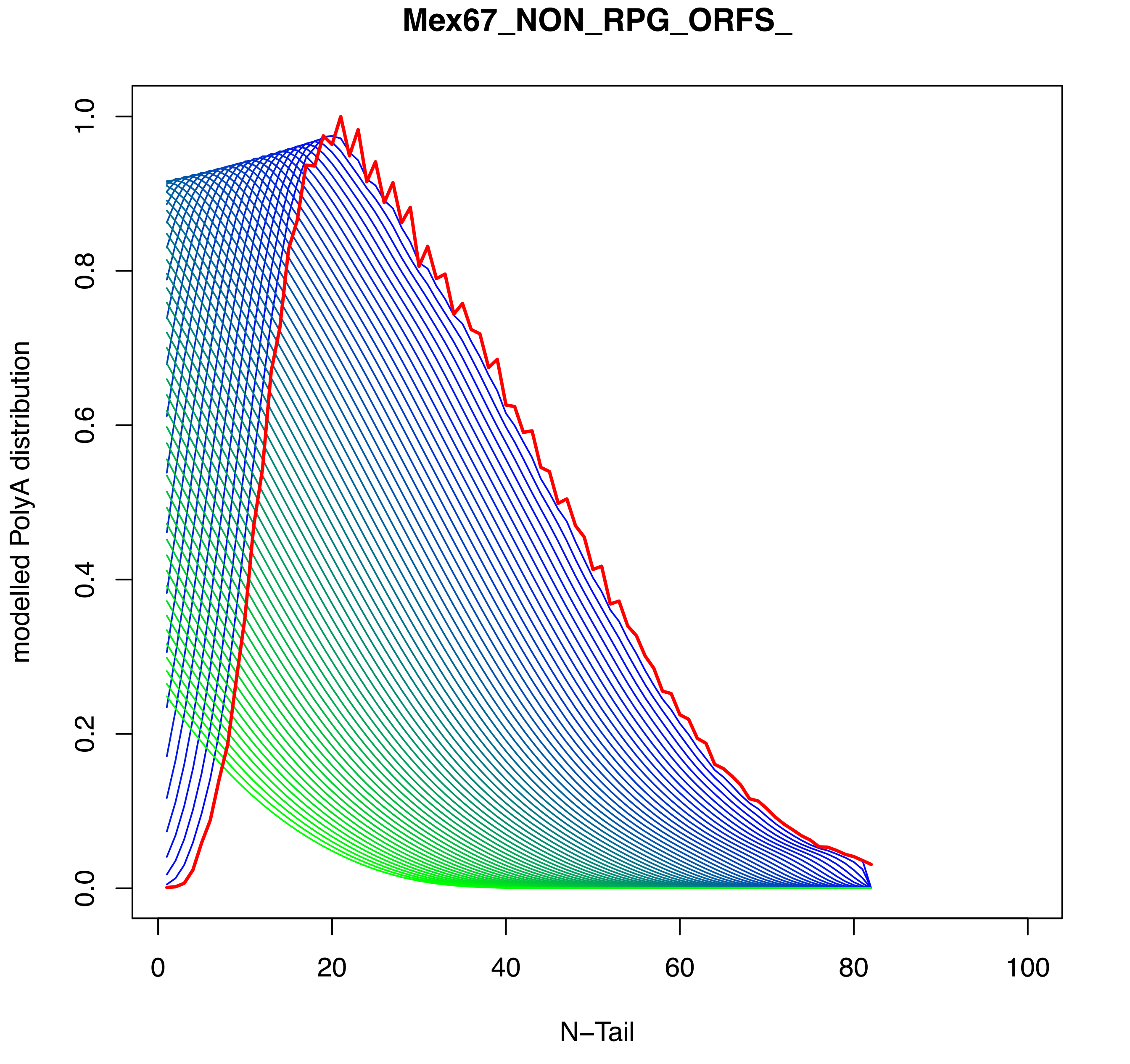

Mex67_NON_RPG_ORFS_
modelled PolyA distribution
N−Tail
0.0
0.2
0.4
0.6
0.8
1.0
0
20
40
60
80
100

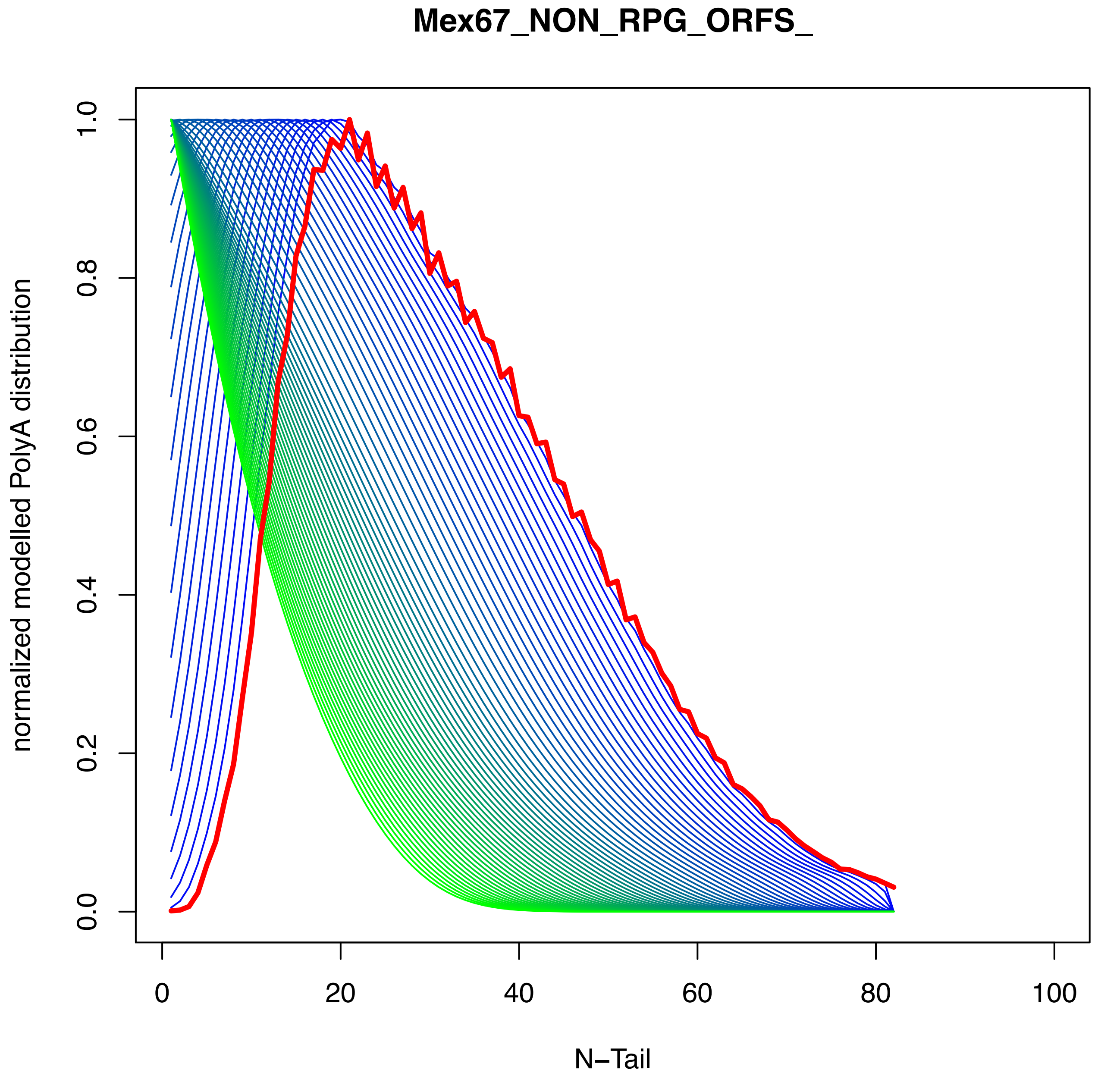

Mex67_NON_RPG_ORFS_
normalized modelled PolyA distribution
N−Tail
0.0
0.2
0.4
0.6
0.8
1.0
0
20
40
60
80
100

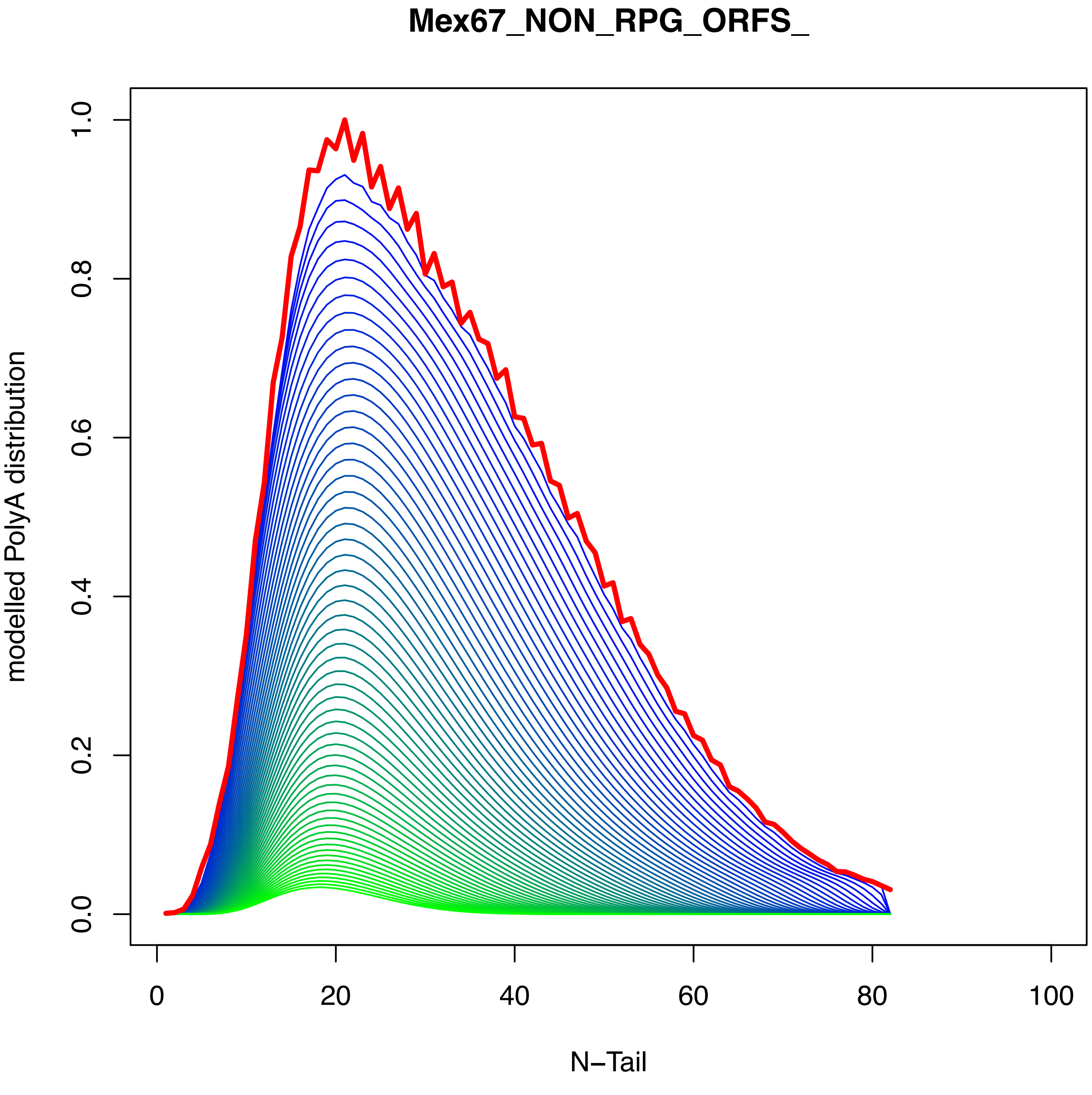

Mex67_NON_RPG_ORFS_
modelled PolyA distribution
N−Tail
0.0
0.2
0.4
0.6
0.8
1.0
0
20
40
60
80
100

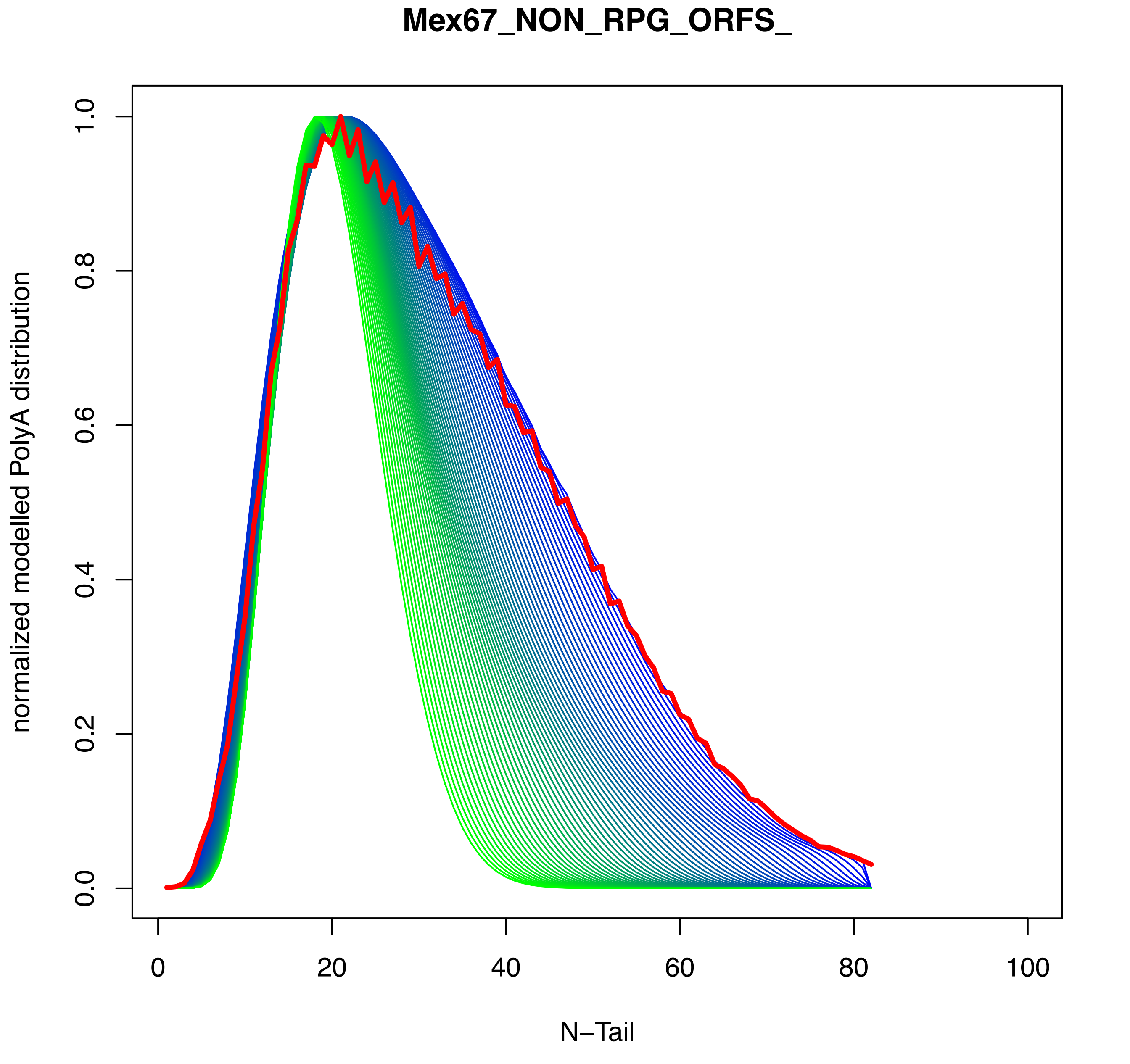

Mex67_NON_RPG_ORFS_
normalized modelled PolyA distribution
N−Tail
0.0
0.2
0.4
0.6
0.8
1.0
0
20
40
60
80
100

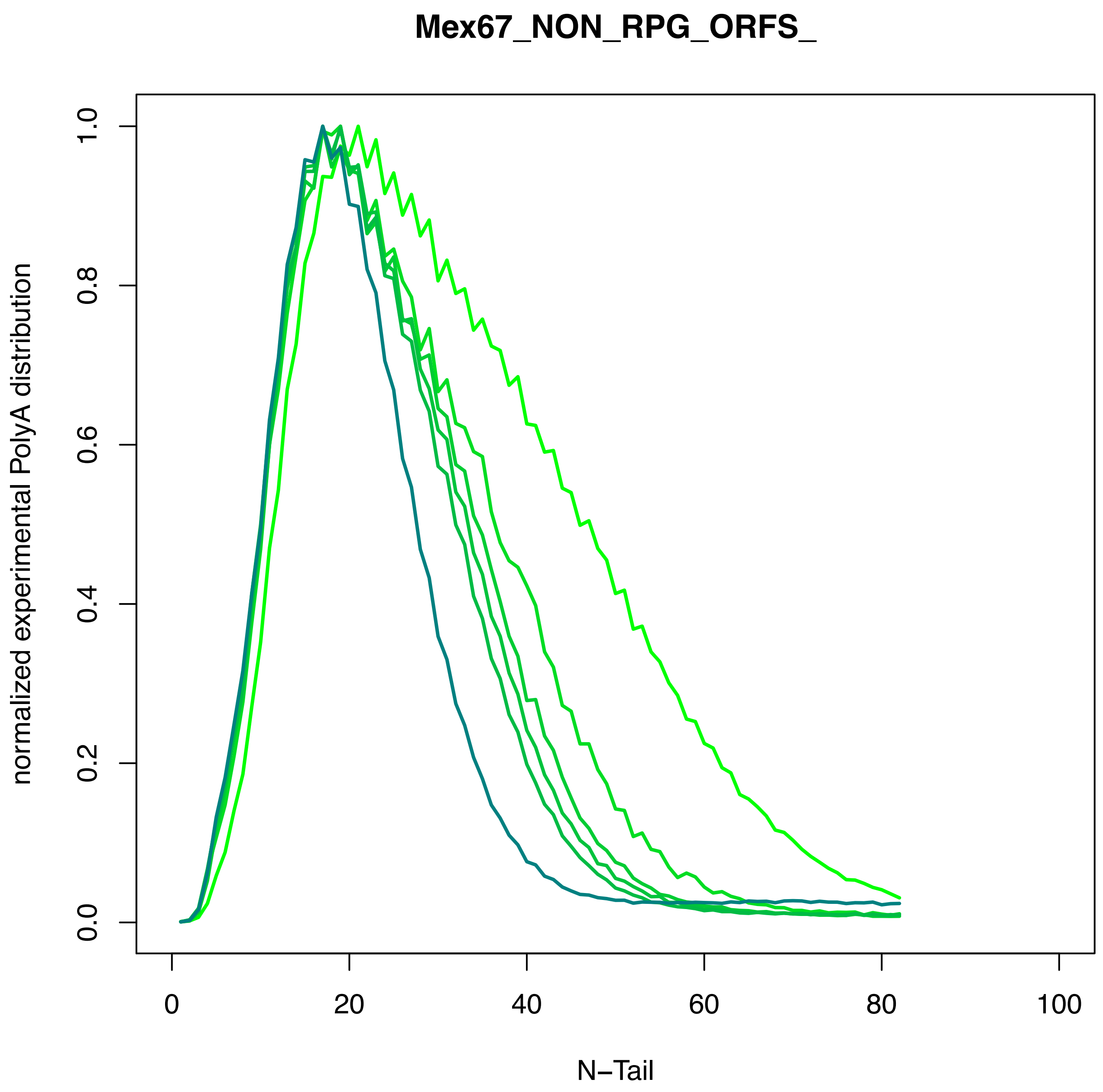

Mex67_NON_RPG_ORFS_
normalized experimental PolyA distribution
N−Tail
0.0
0.2
0.4
0.6
0.8
1.0
0
20
40
60
80
100

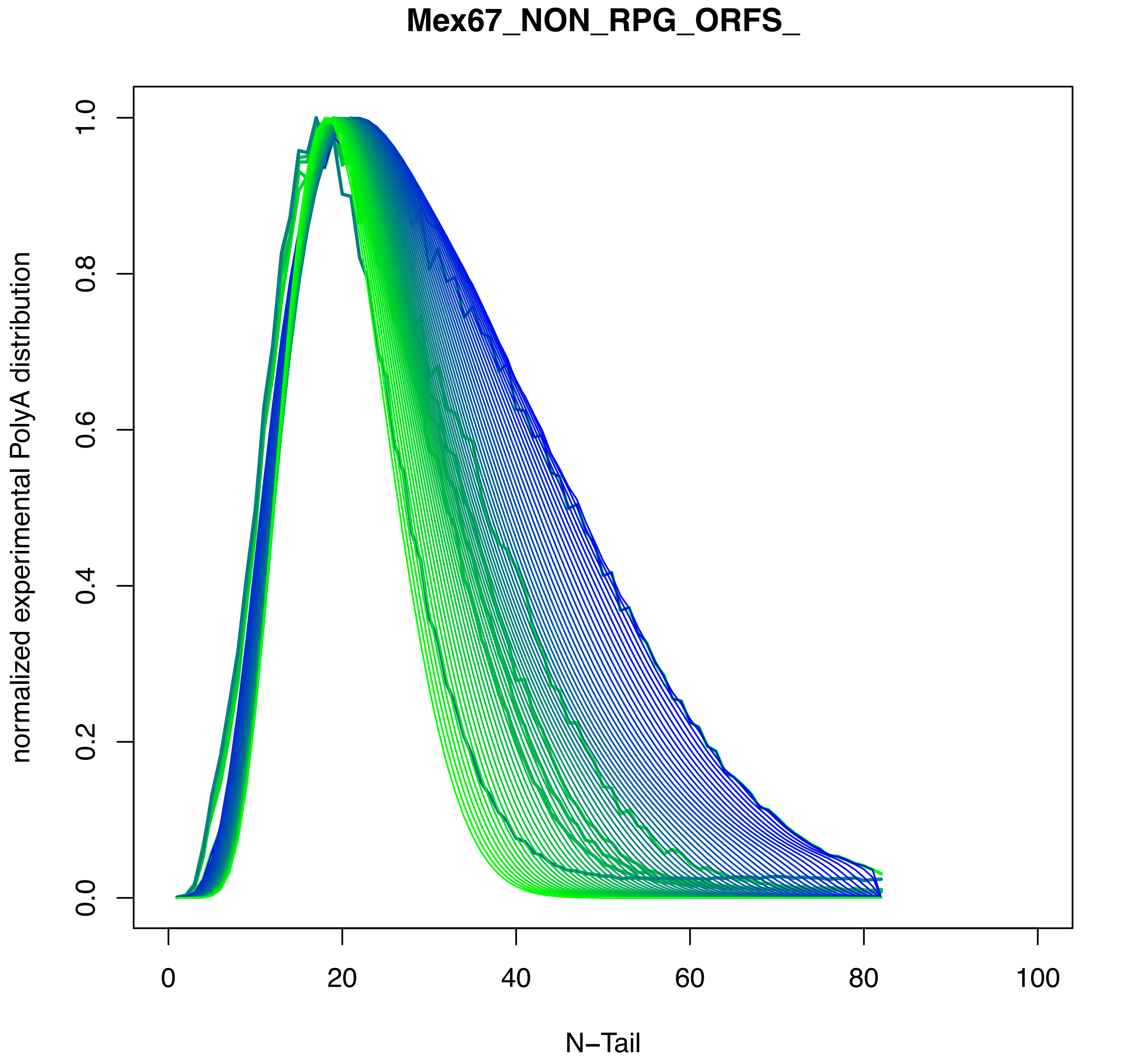

Mex67_NON_RPG_ORFS_
normalized experimental PolyA distribution
N−Tail
0.0
0.2
0.4
0.6
0.8
1.0
0
20
40
60
80
100

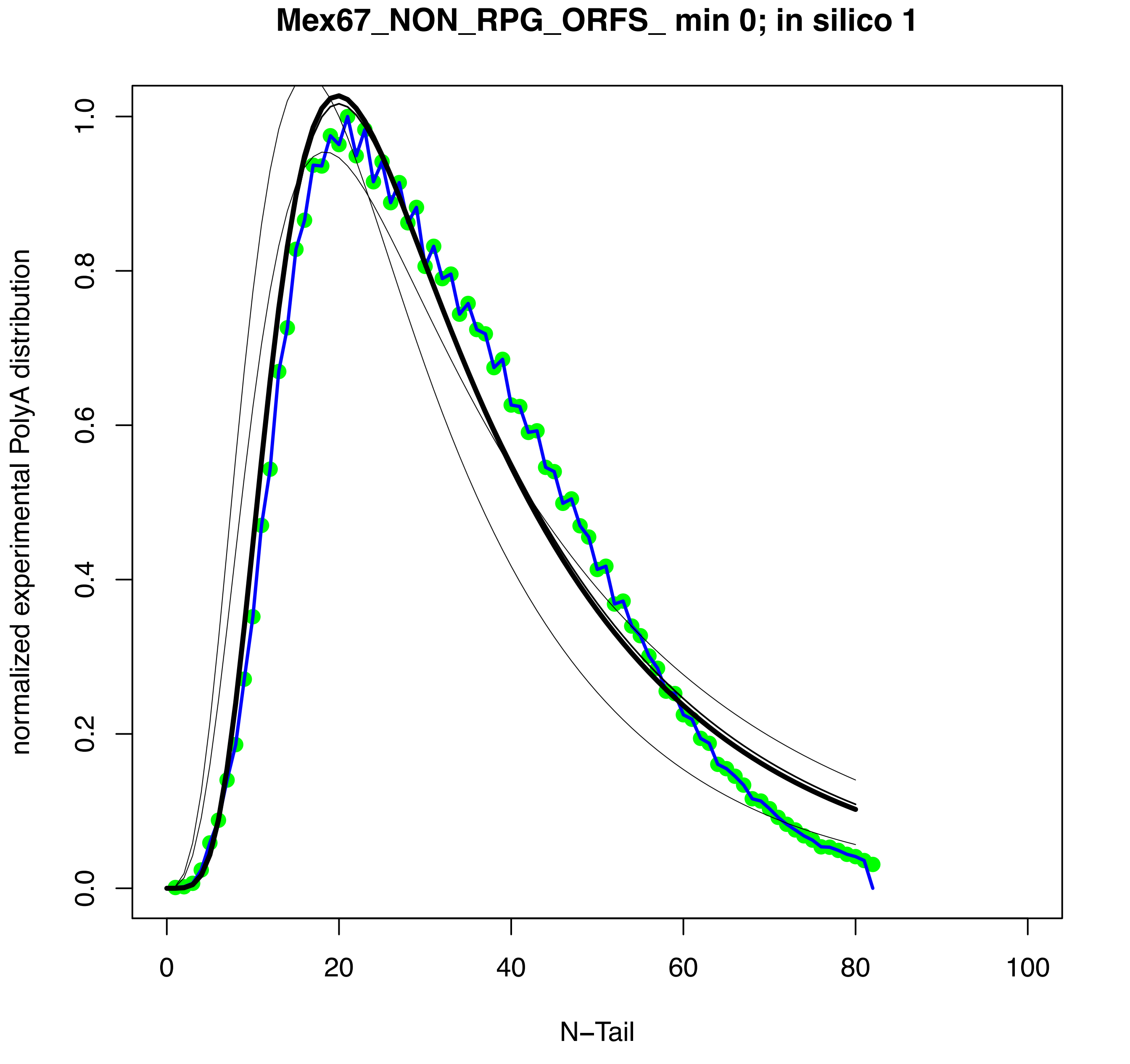
Mex67_NON_RPG_ORFS_ min 0; in silico 1
normalized experimental PolyA distribution
N−Tail
0.0
0.2
0.4
0.6
0.8
1.0
0
20
40
60
80
100

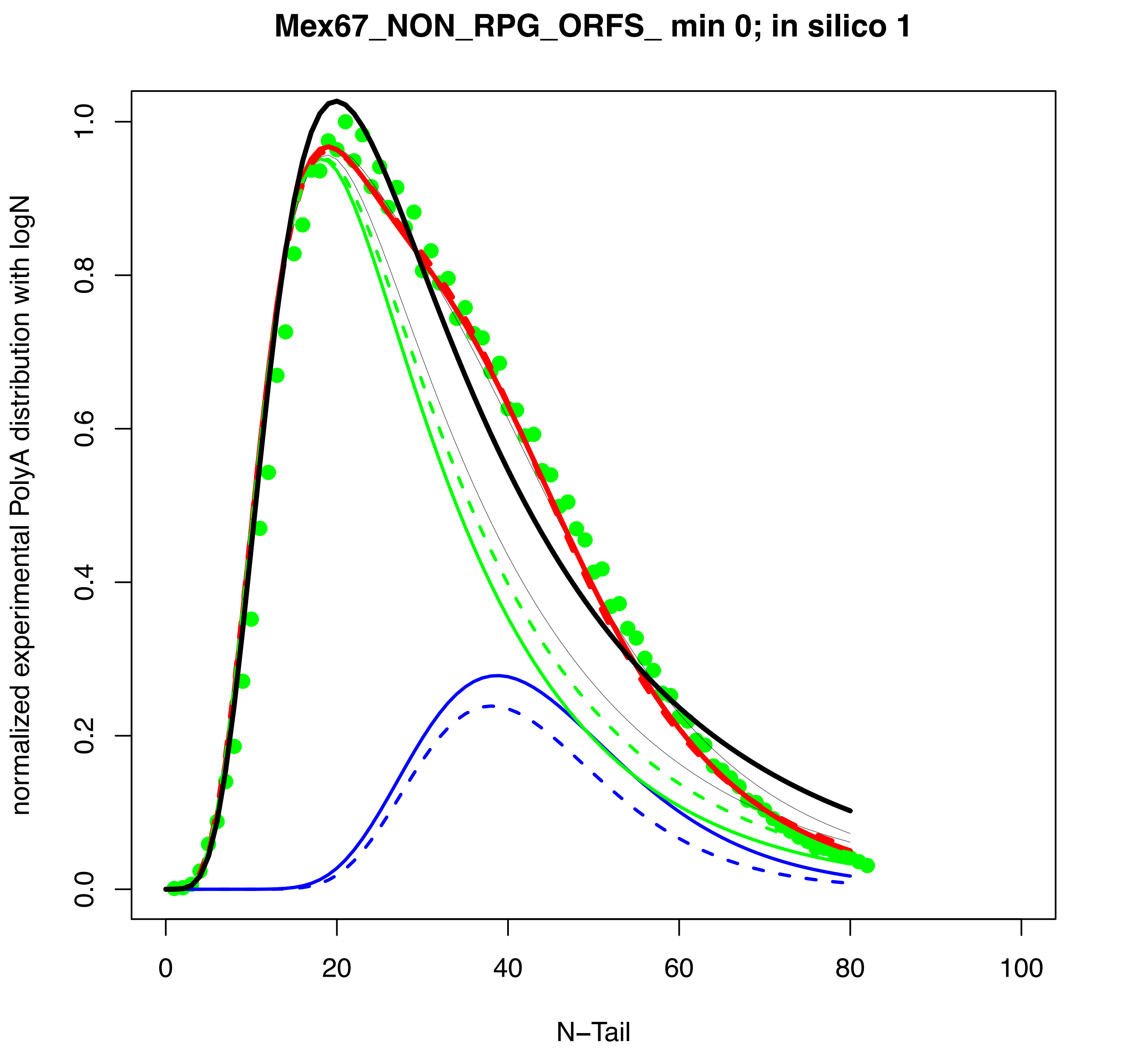

Mex67_NON_RPG_ORFS_ min 0; in silico 1
normalized experimental PolyA distribution with logN
N−Tail
0
20
40
60
80
100
0.0
0.2
0.4
0.6
0.8
1.0

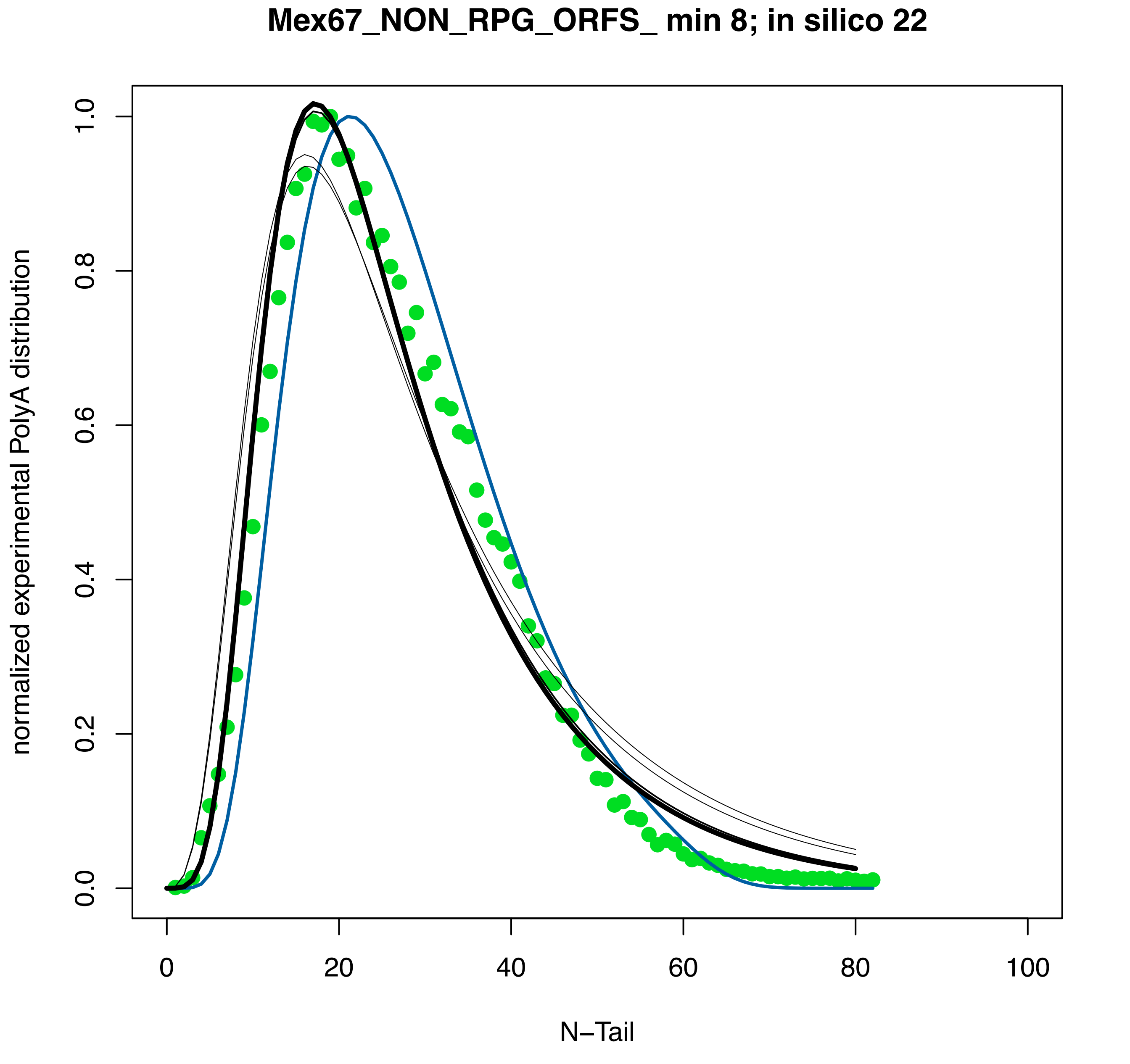

Mex67_NON_RPG_ORFS_ min 8; in silico 22
normalized experimental PolyA distribution
N−Tail
0.0
0.2
0.4
0.6
0.8
1.0
0
20
40
60
80
100

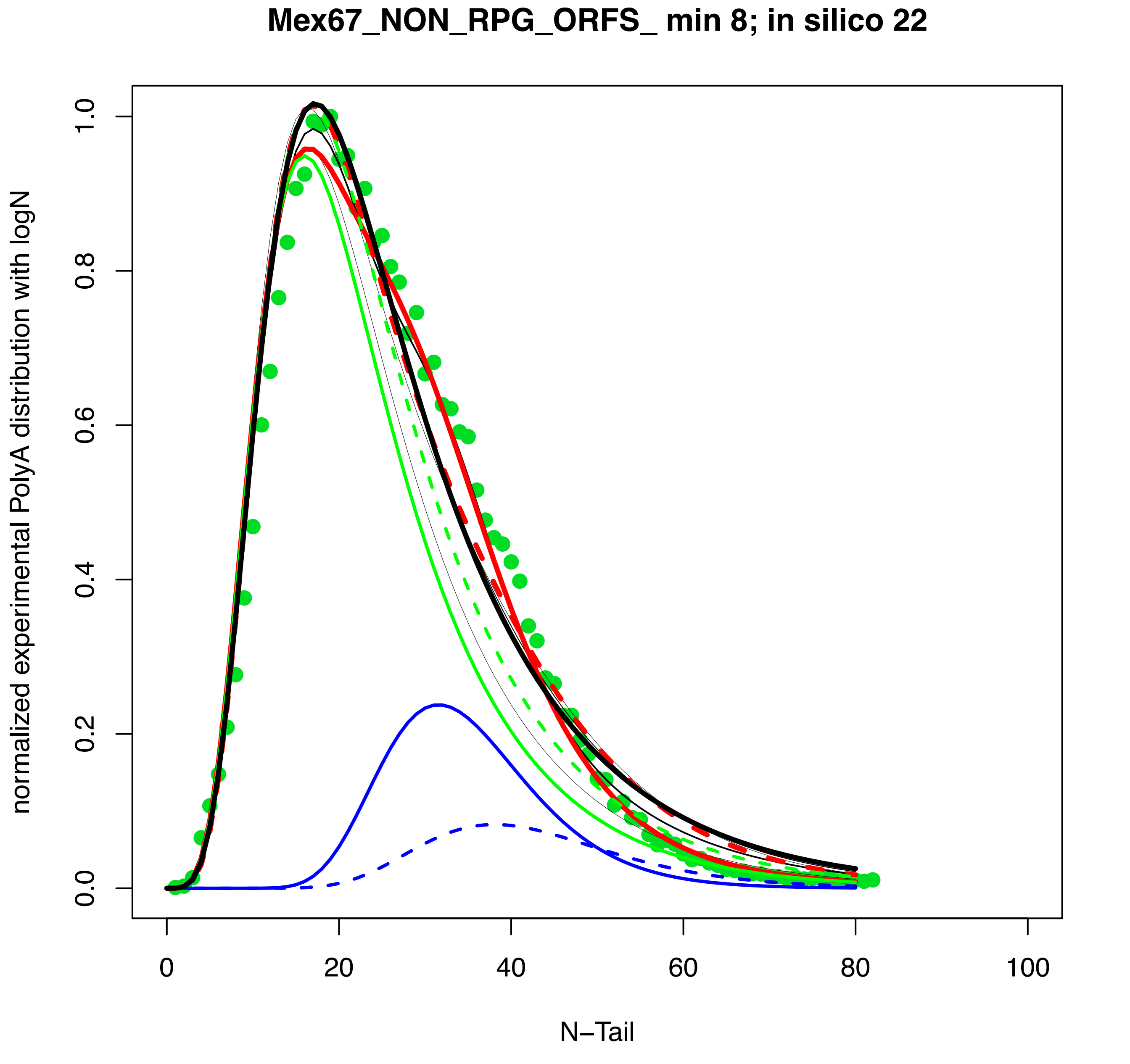

Mex67_NON_RPG_ORFS_ min 8; in silico 22
normalized experimental PolyA distribution with logN
N−Tail
0.0
0.2
0.4
0.6
0.8
1.0
0
20
40
60
80
100

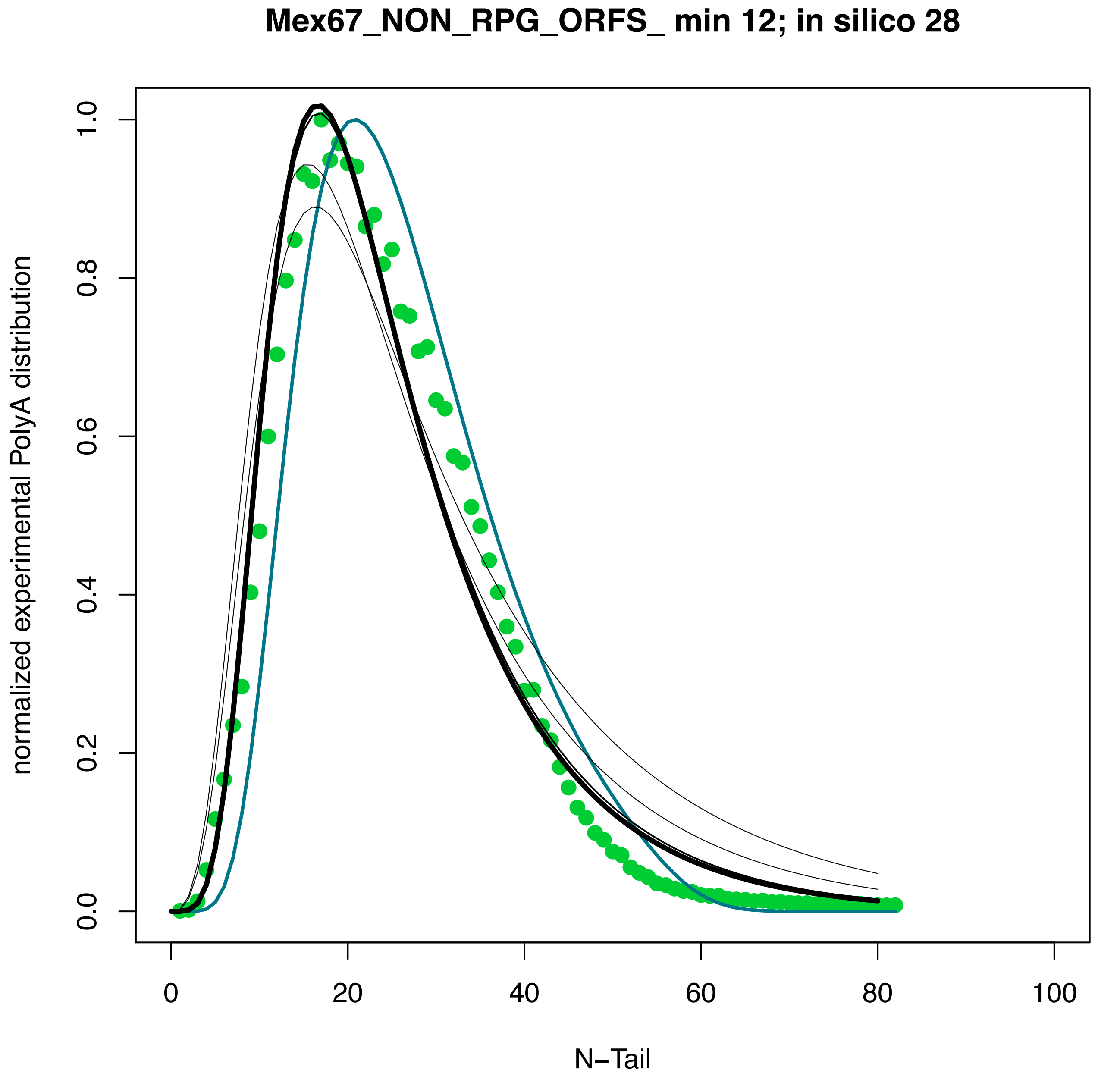

Mex67_NON_RPG_ORFS_ min 12; in silico 28
normalized experimental PolyA distribution
N−Tail
0.0
0.2
0.4
0.6
0.8
1.0
0
20
40
60
80
100

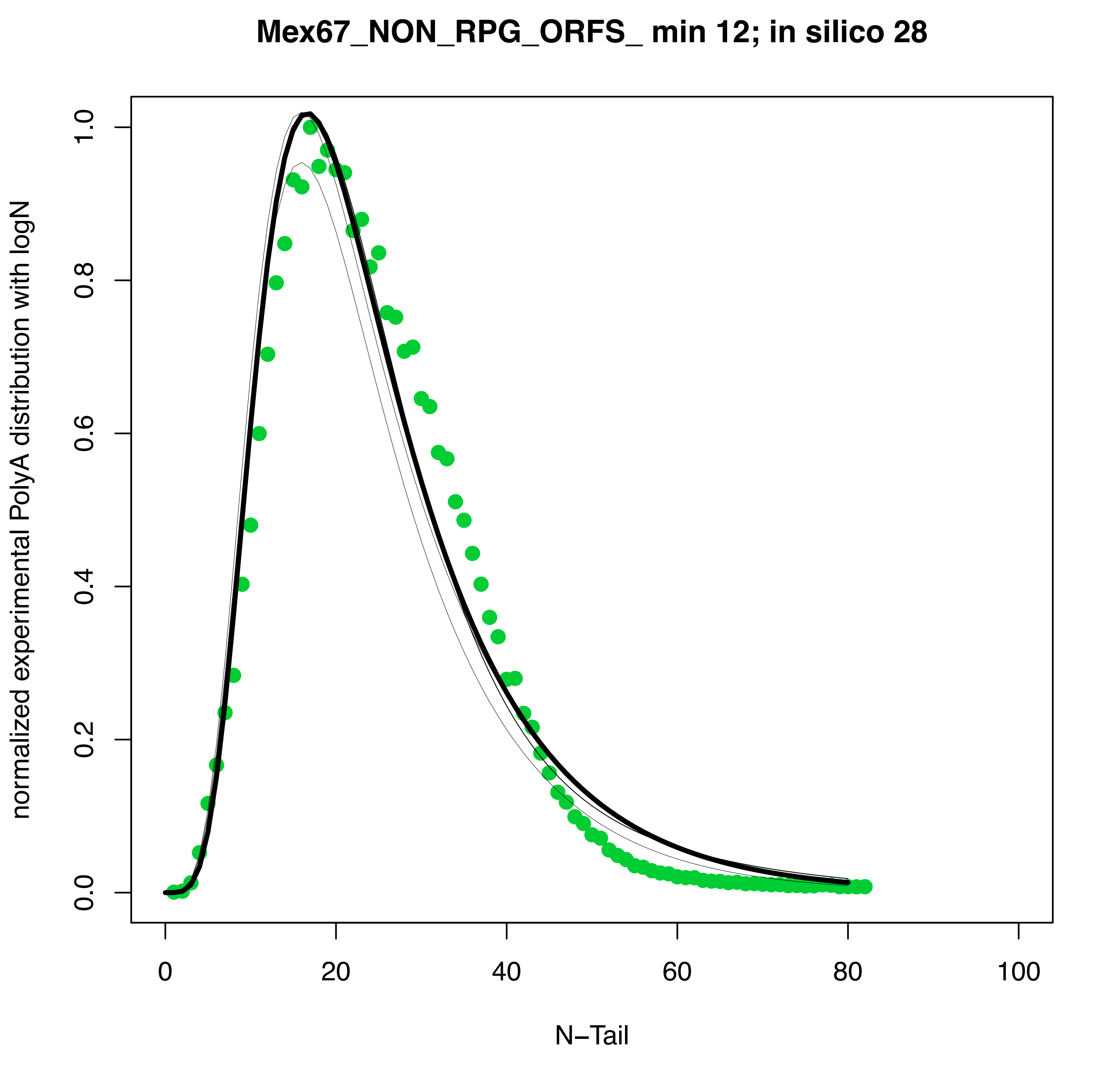

Mex67_NON_RPG_ORFS_ min 12; in silico 28
normalized experimental PolyA distribution with logN
N−Tail
0.0
0.2
0.4
0.6
0.8
1.0
0
20
40
60
80
100

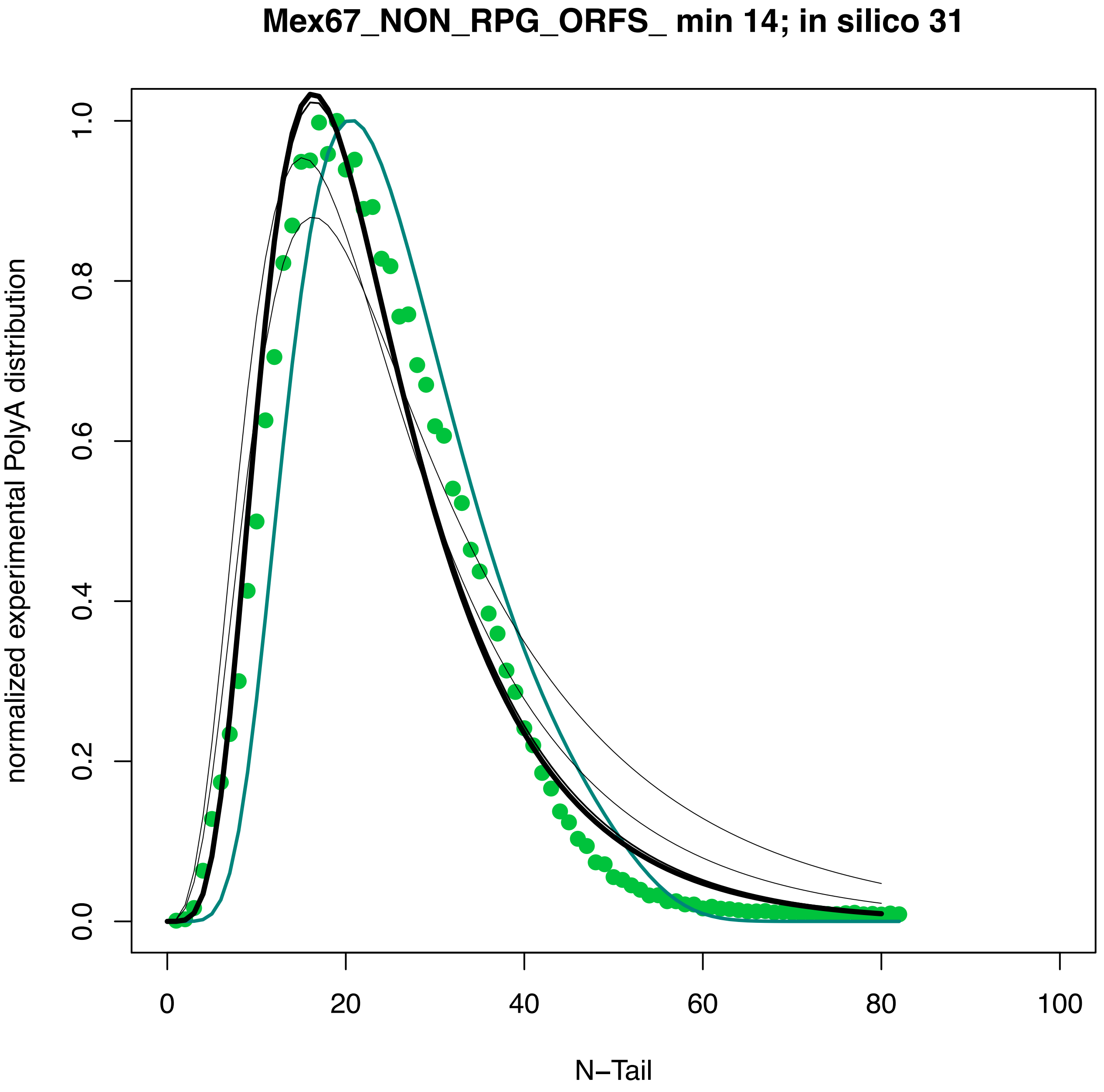
Mex67_NON_RPG_ORFS_ min 14; in silico 31
normalized experimental PolyA distribution
N−Tail
0.0
0.2
0.4
0.6
0.8
1.0
0
20
40
60
80
100

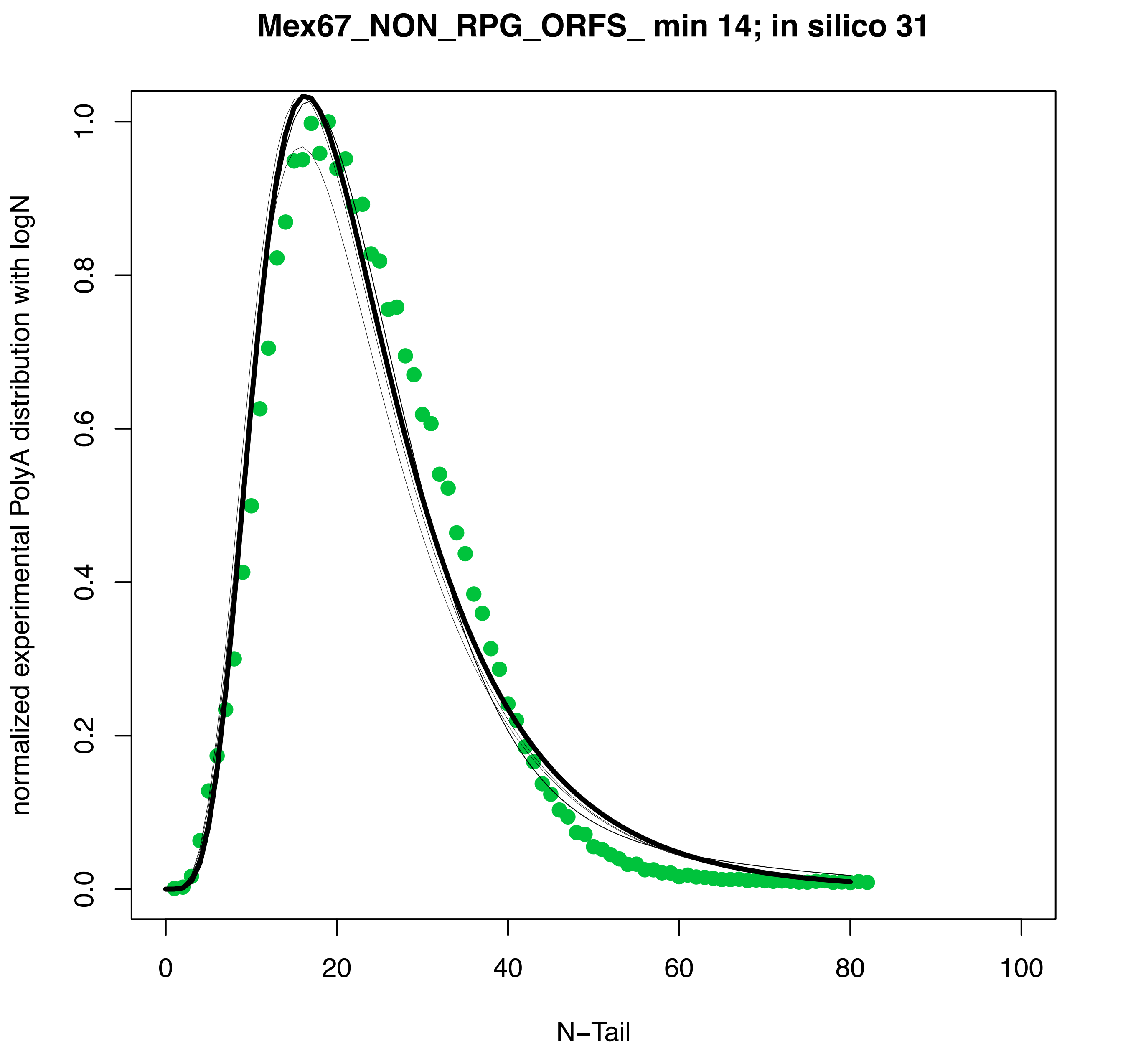
Mex67_NON_RPG_ORFS_ min 14; in silico 31
normalized experimental PolyA distribution with logN
N−Tail
0.0
0.2
0.4
0.6
0.8
1.0
0
20
40
60
80
100

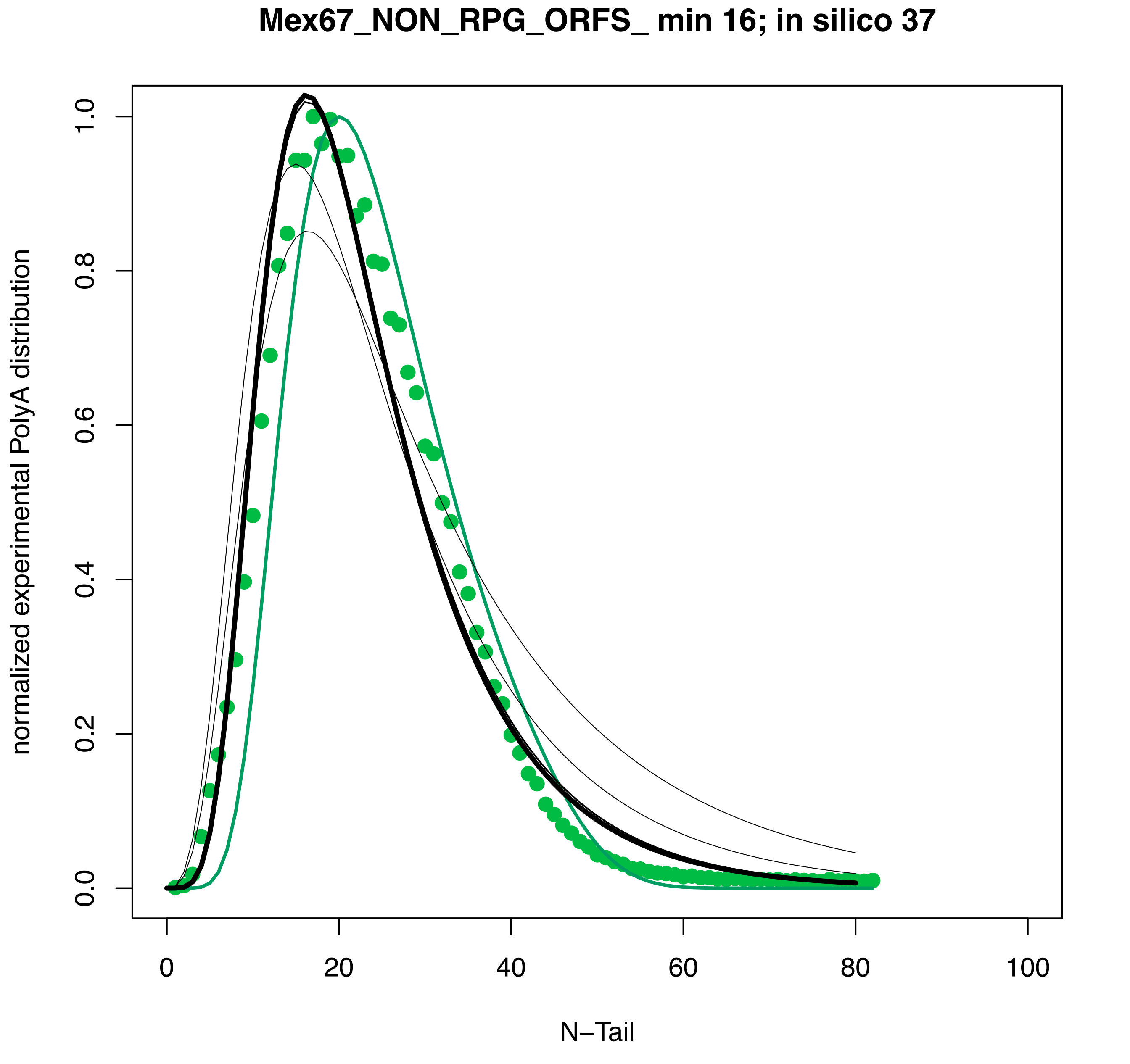

Mex67_NON_RPG_ORFS_ min 16; in silico 37
normalized experimental PolyA distribution
0.0
0.2
0.4
0.6
0.8
1.0
0
20
40
60
80
100
N−Tail

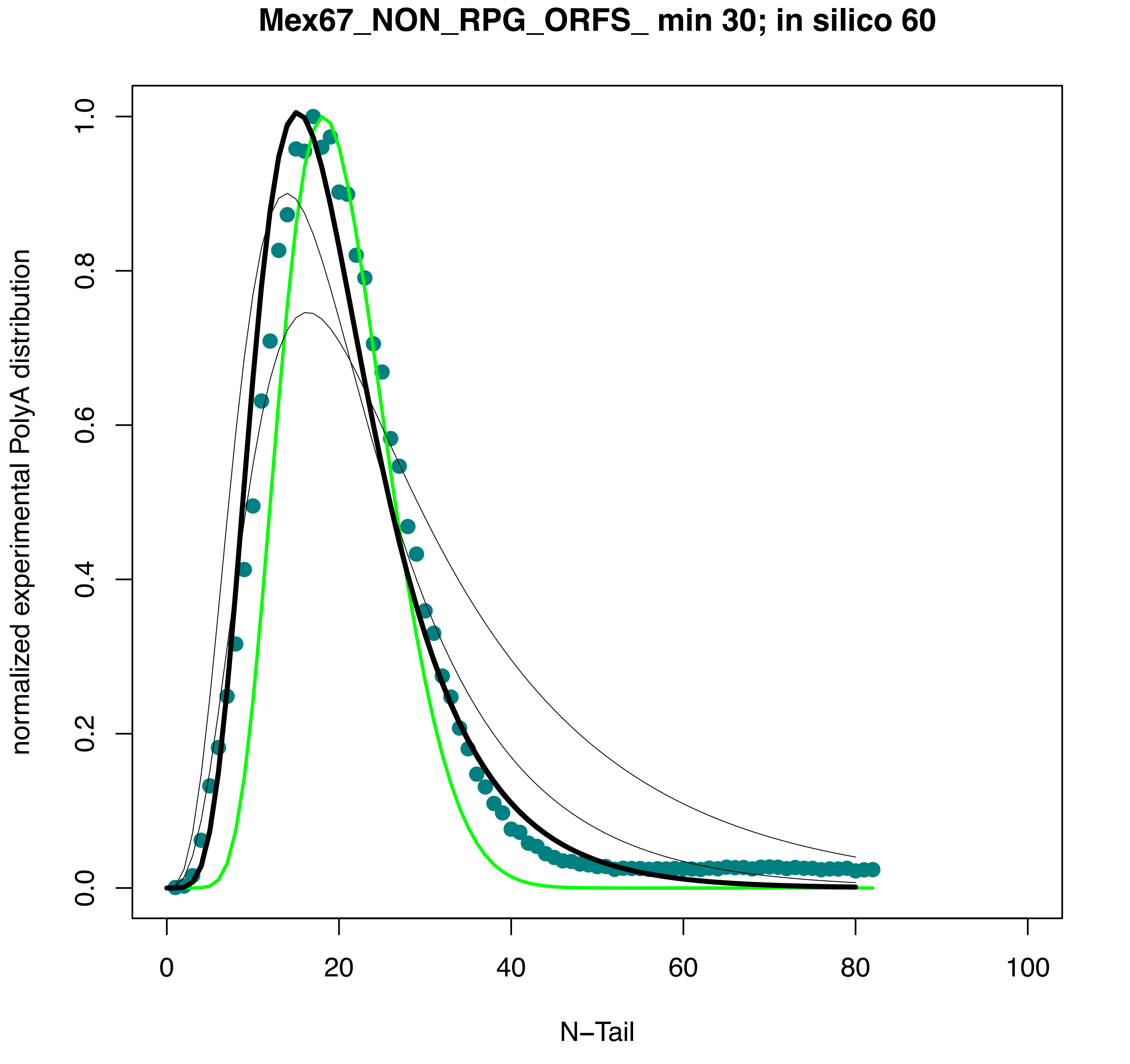
Mex67_NON_RPG_ORFS_ min 30; in silico 60
normalized experimental PolyA distribution
0.0
0.2
0.4
0.6
0.8
1.0
0
20
40
60
80
100
N−Tail

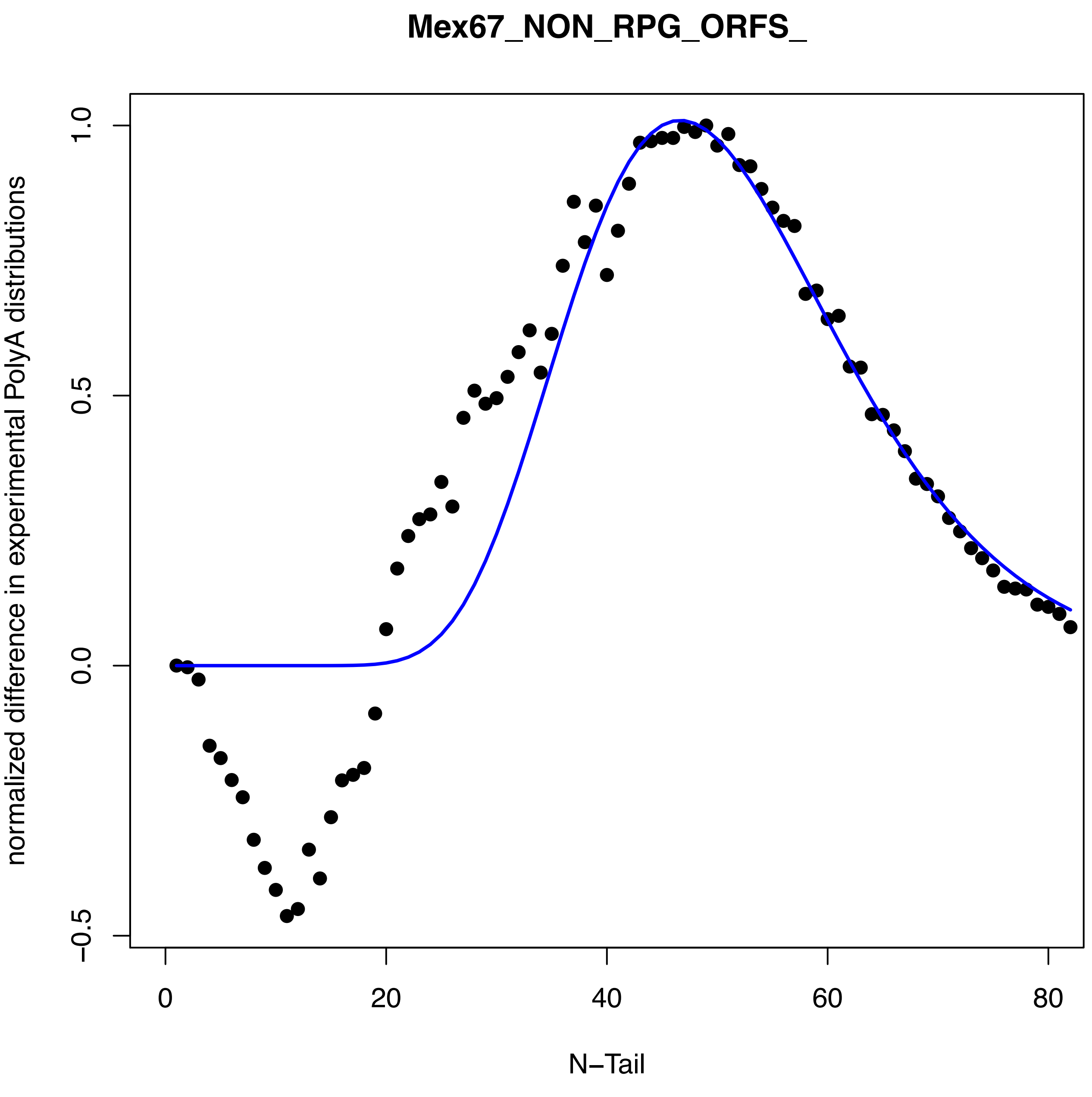

Mex67_NON_RPG_ORFS_
normalized difference in experimental PolyA distributions
N−Tail
1.0
0.5
0.0
−0.5
0
20
40
60
80

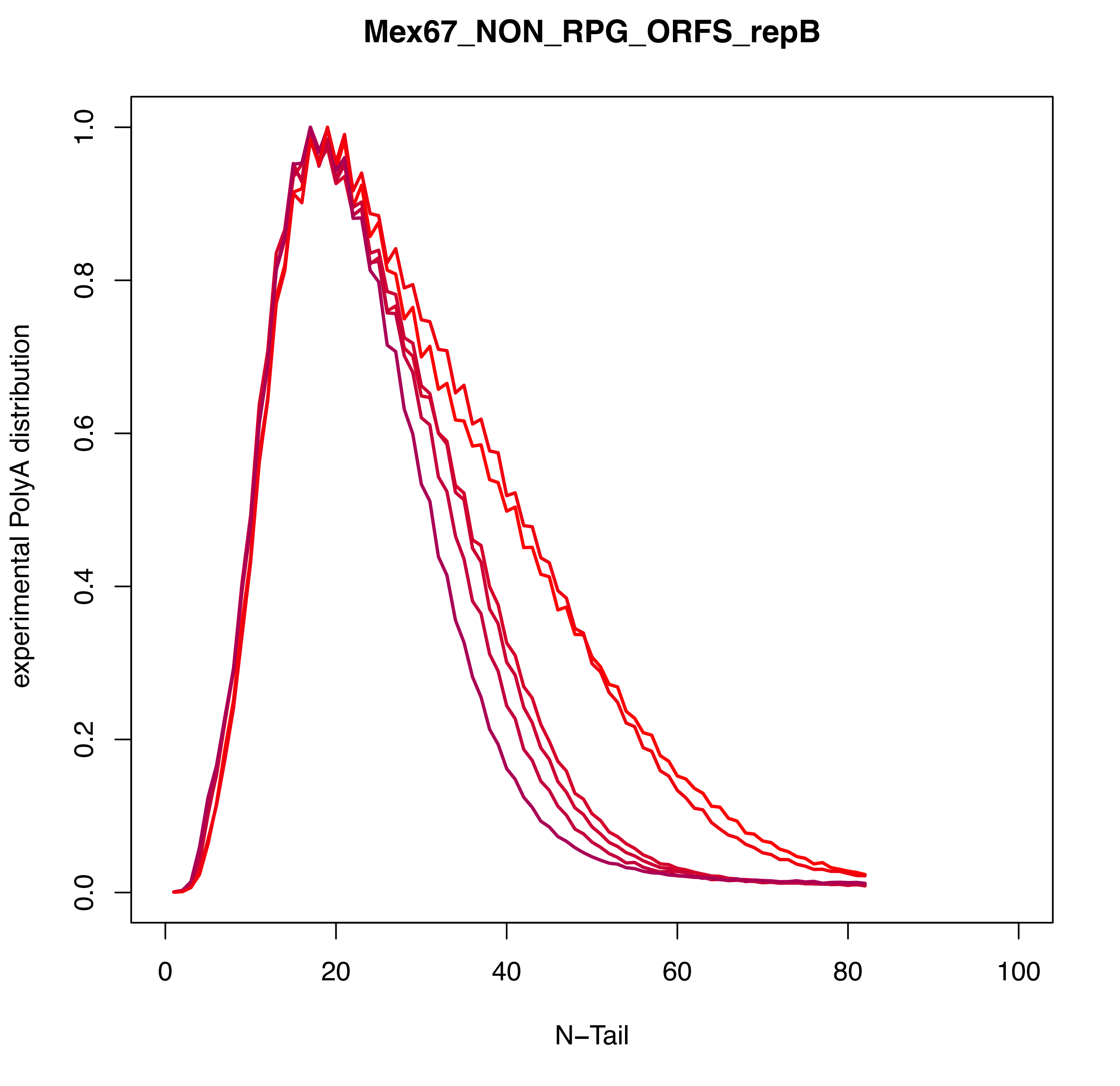

Mex67_NON_RPG_ORFS_repB
experimental PolyA distribution
N−Tail
0.0
0.2
0.4
0.6
0.8
1.0
0
20
40
60
80
100

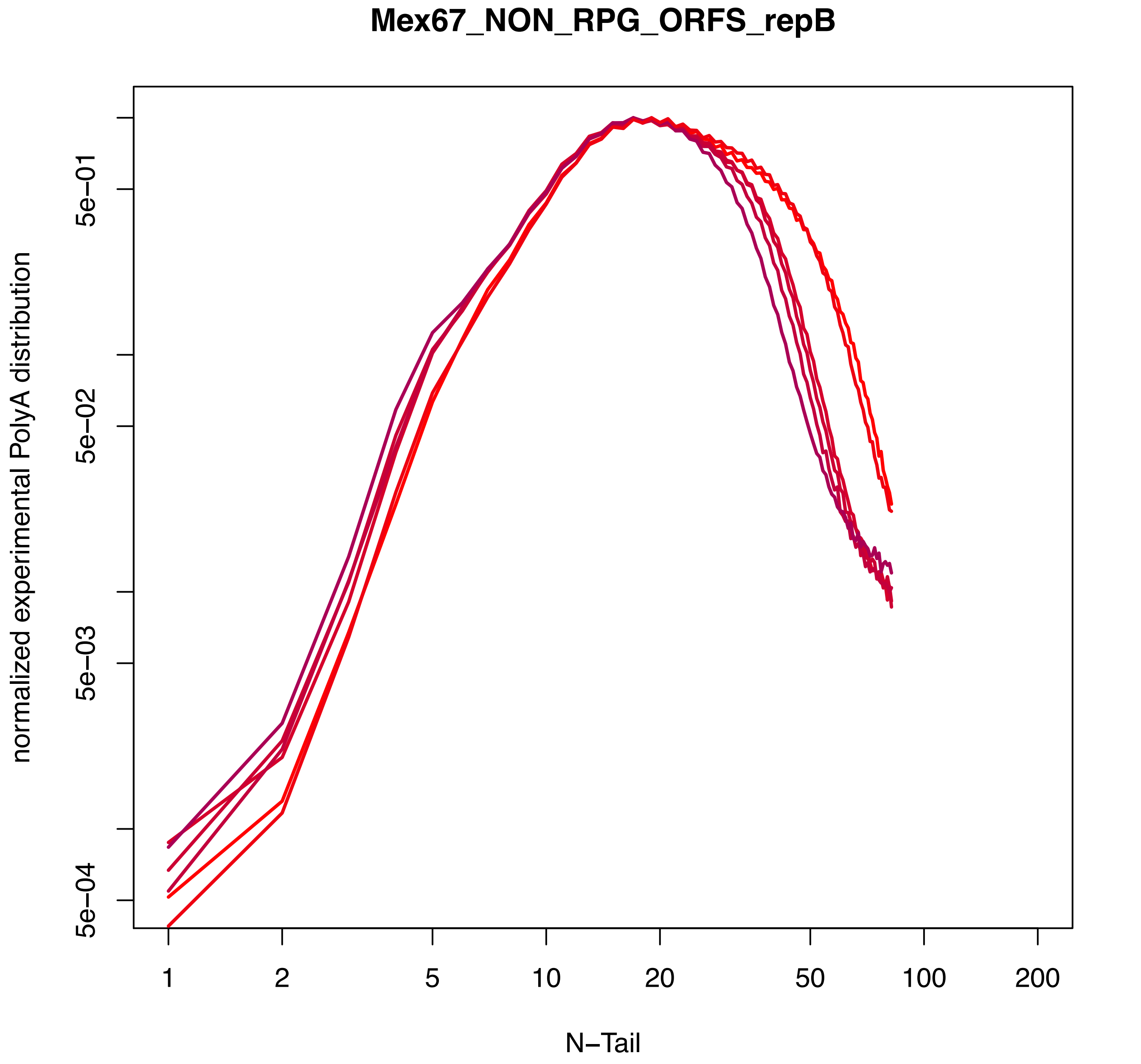
Mex67_NON_RPG_ORFS_repB
normalized experimental PolyA distribution
5e−01
5e−02
5e−03
5e−04
N−Tail
1
2
5
10
20
50
100
200

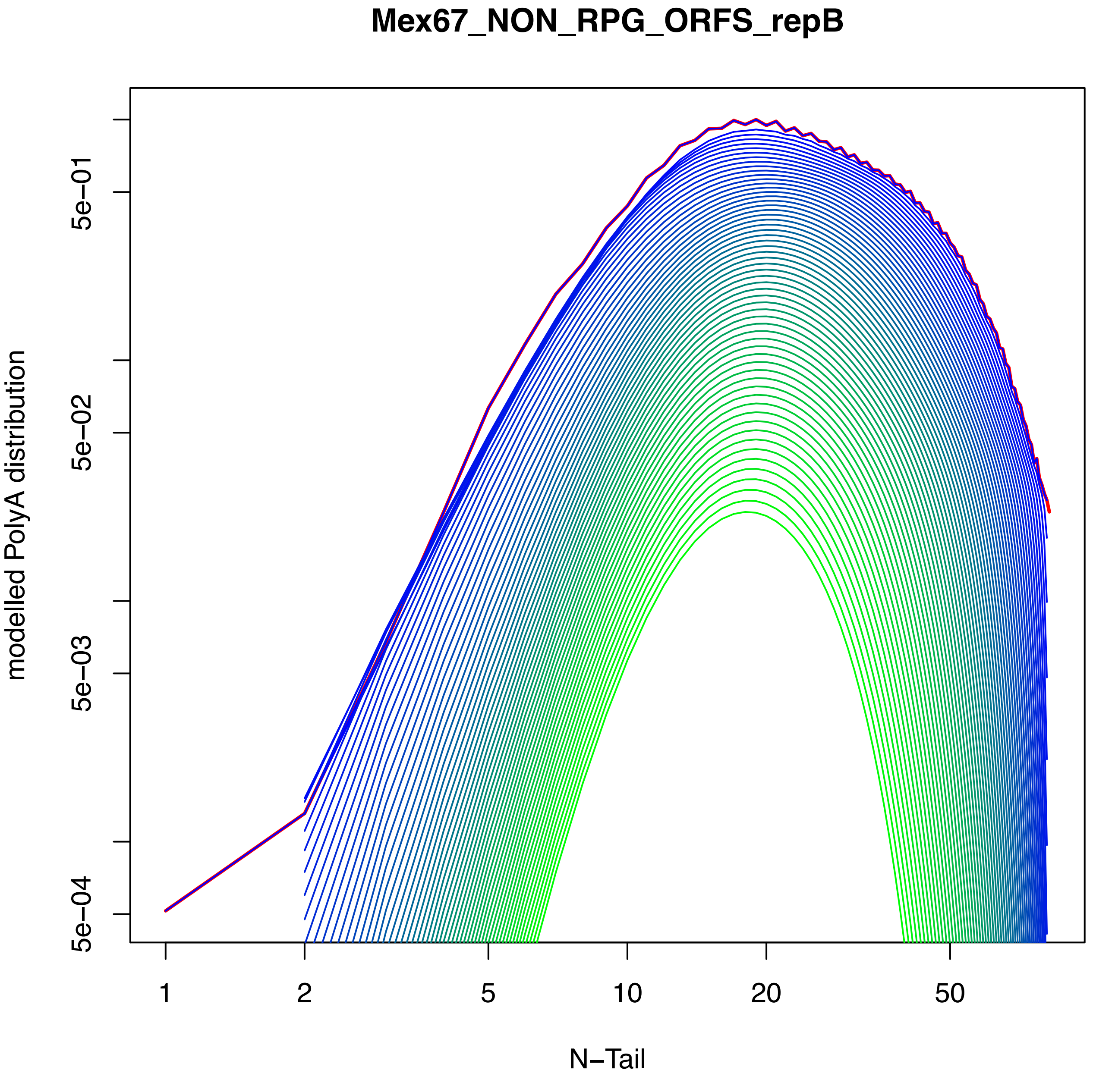
Mex67_NON_RPG_ORFS_repB
modelled PolyA distribution
5e−04
5e−03
5e−02
5e−01
1
2
5
10
20
50
N−Tail

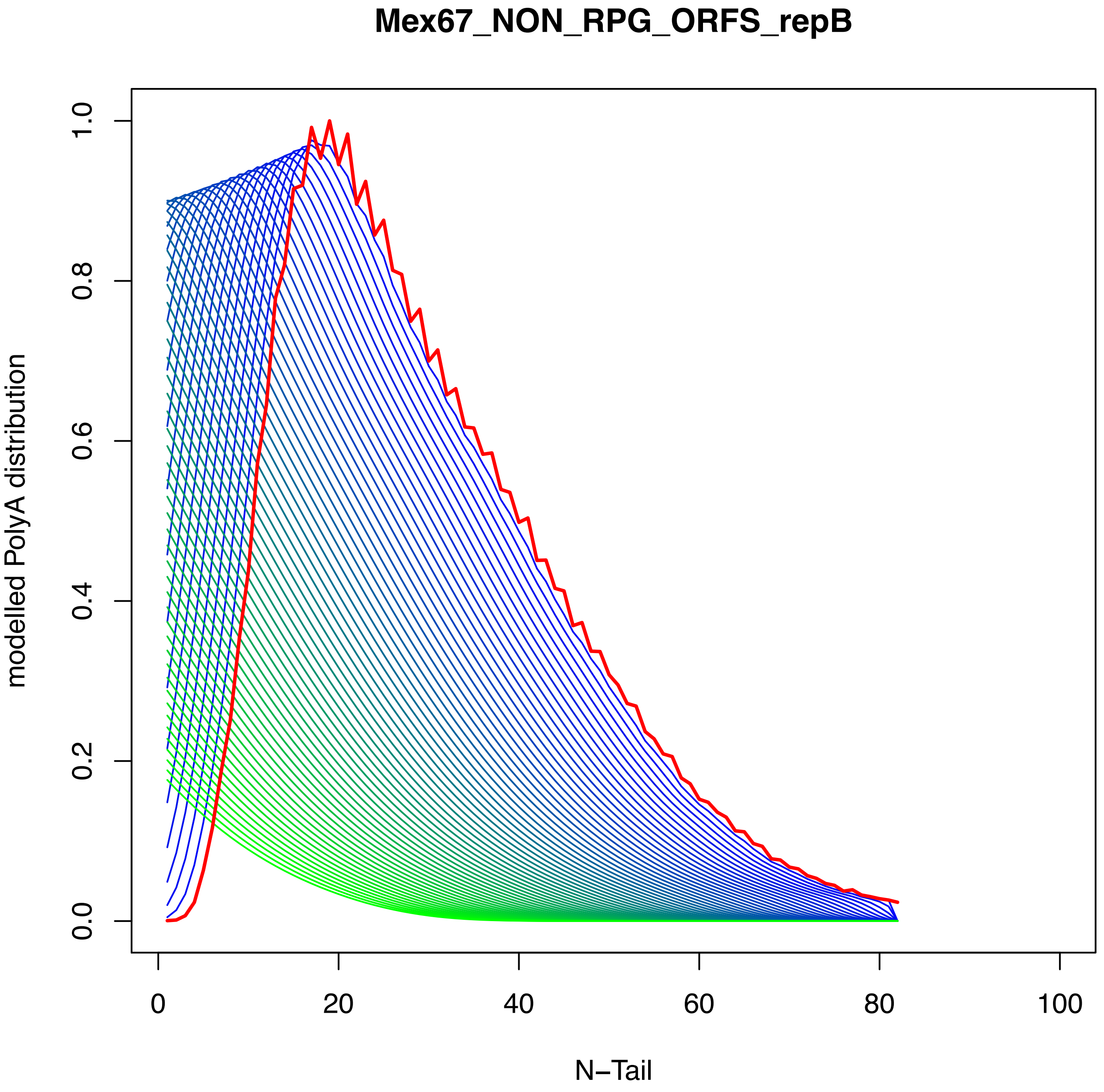
Mex67_NON_RPG_ORFS_repB
modelled PolyA distribution
N−Tail
0.0
0.2
0.4
0.6
0.8
1.0
0
20
40
60
80
100

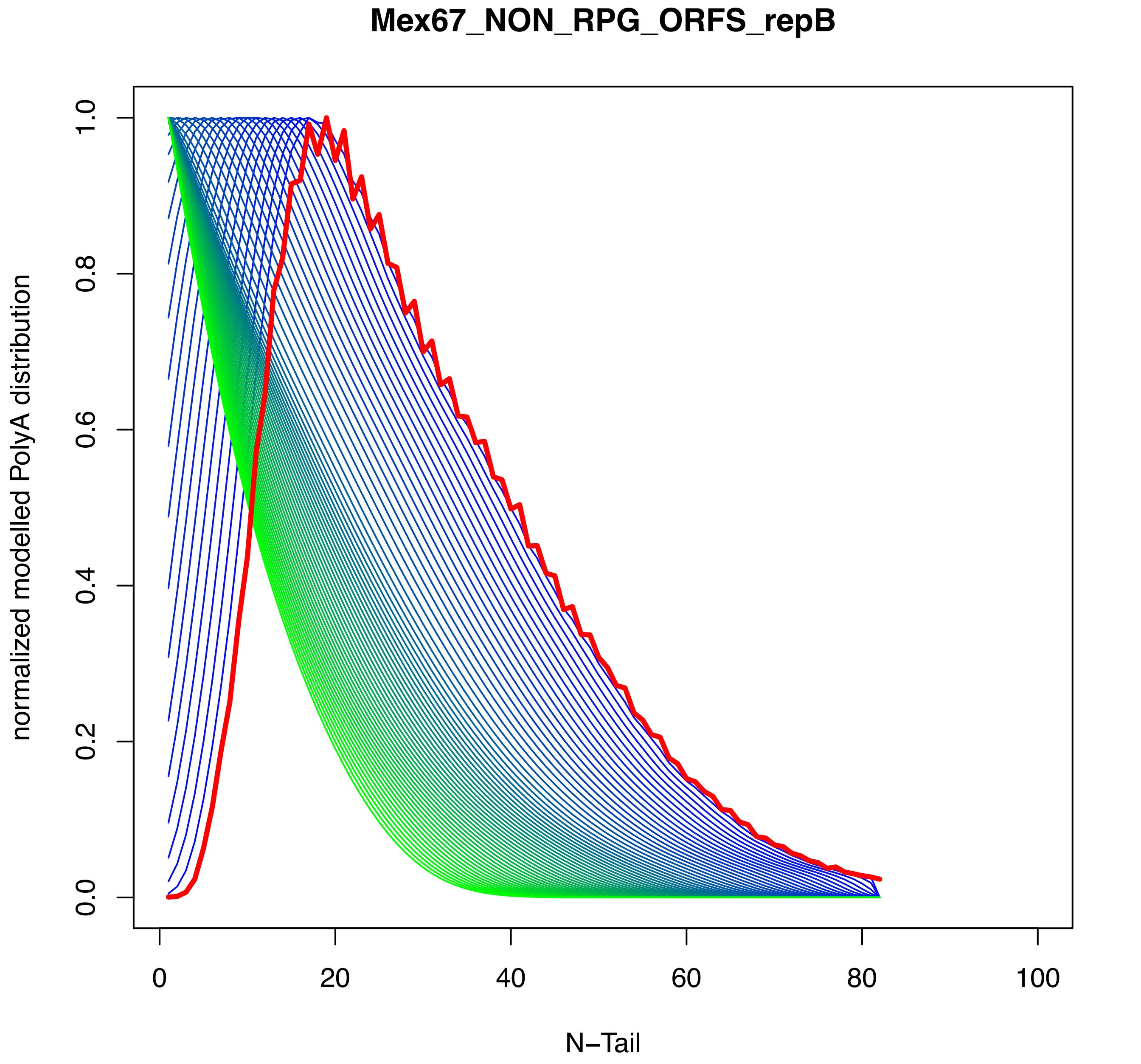

Mex67_NON_RPG_ORFS_repB
normalized modelled PolyA distribution
0.0
0.2
0.4
0.6
0.8
1.0
0
20
40
60
80
100
N−Tail

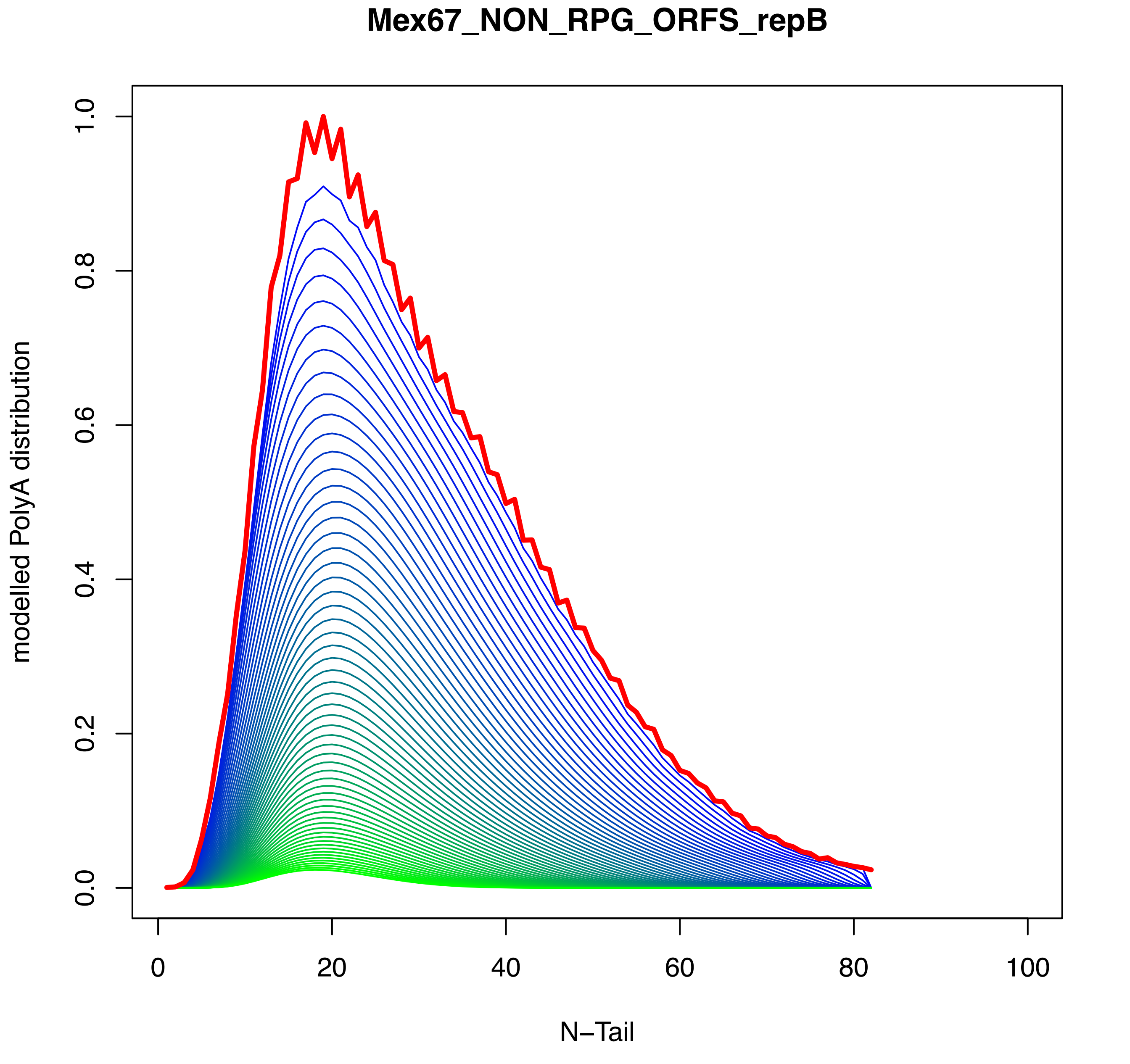

Mex67_NON_RPG_ORFS_repB
modelled PolyA distribution
0.0
0.2
0.4
0.6
0.8
1.0
0
20
40
60
80
100
N−Tail

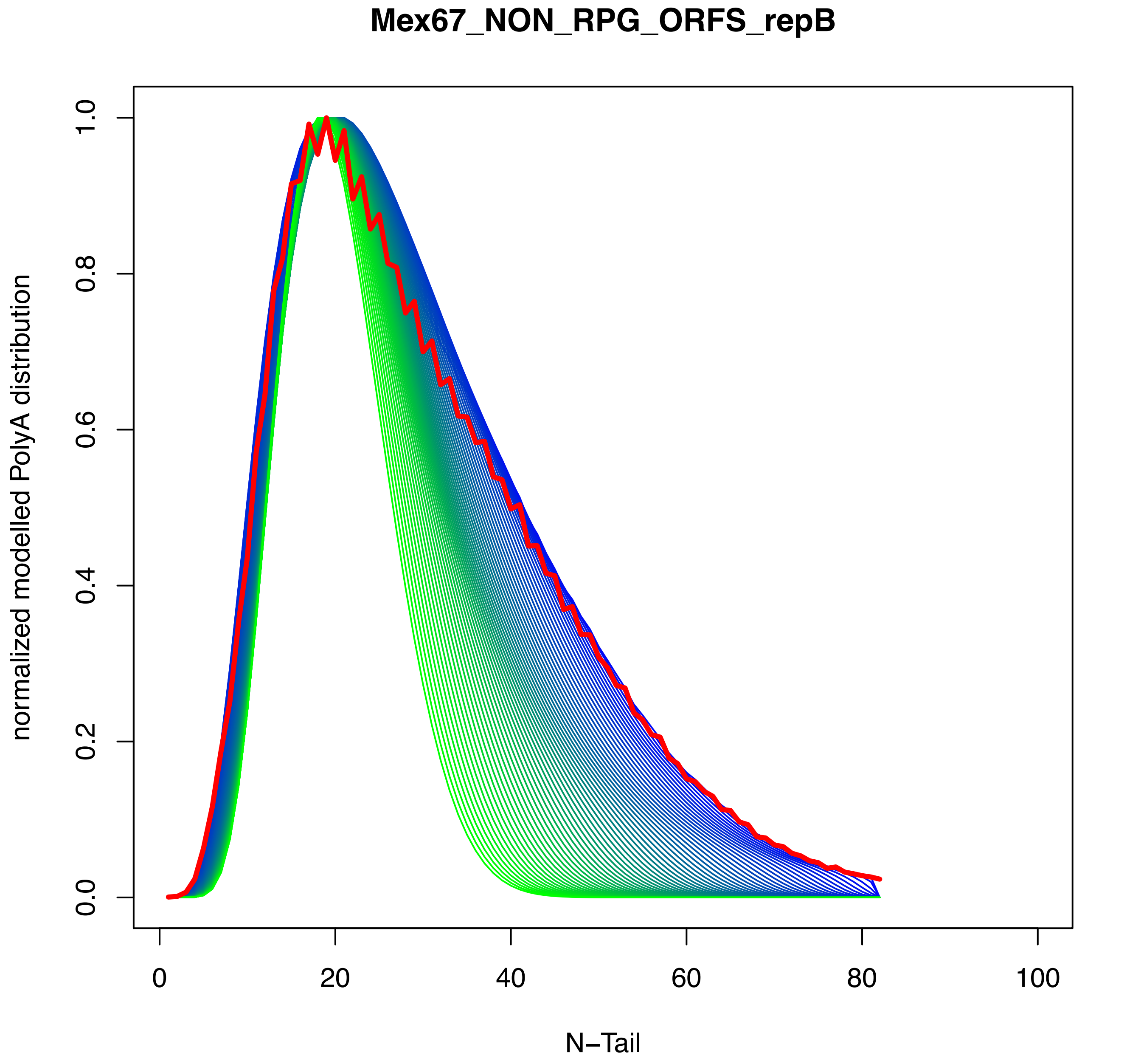

Mex67_NON_RPG_ORFS_repB
normalized modelled PolyA distribution
N−Tail
0.0
0.2
0.4
0.6
0.8
1.0
0
20
40
60
80
100

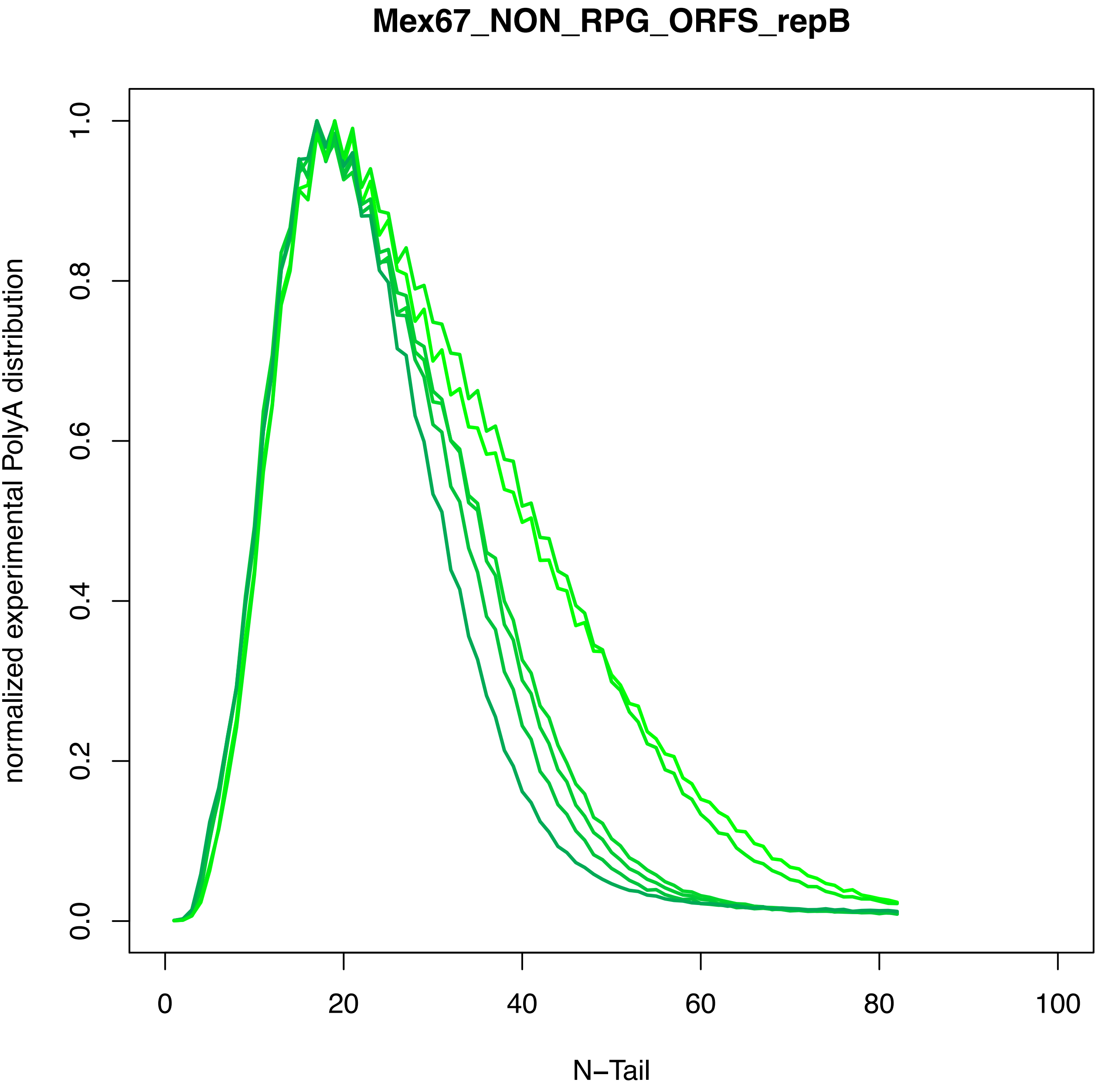
Mex67_NON_RPG_ORFS_repB
normalized experimental PolyA distribution
N−Tail
0.0
0.2
0.4
0.6
0.8
1.0
0
20
40
60
80
100

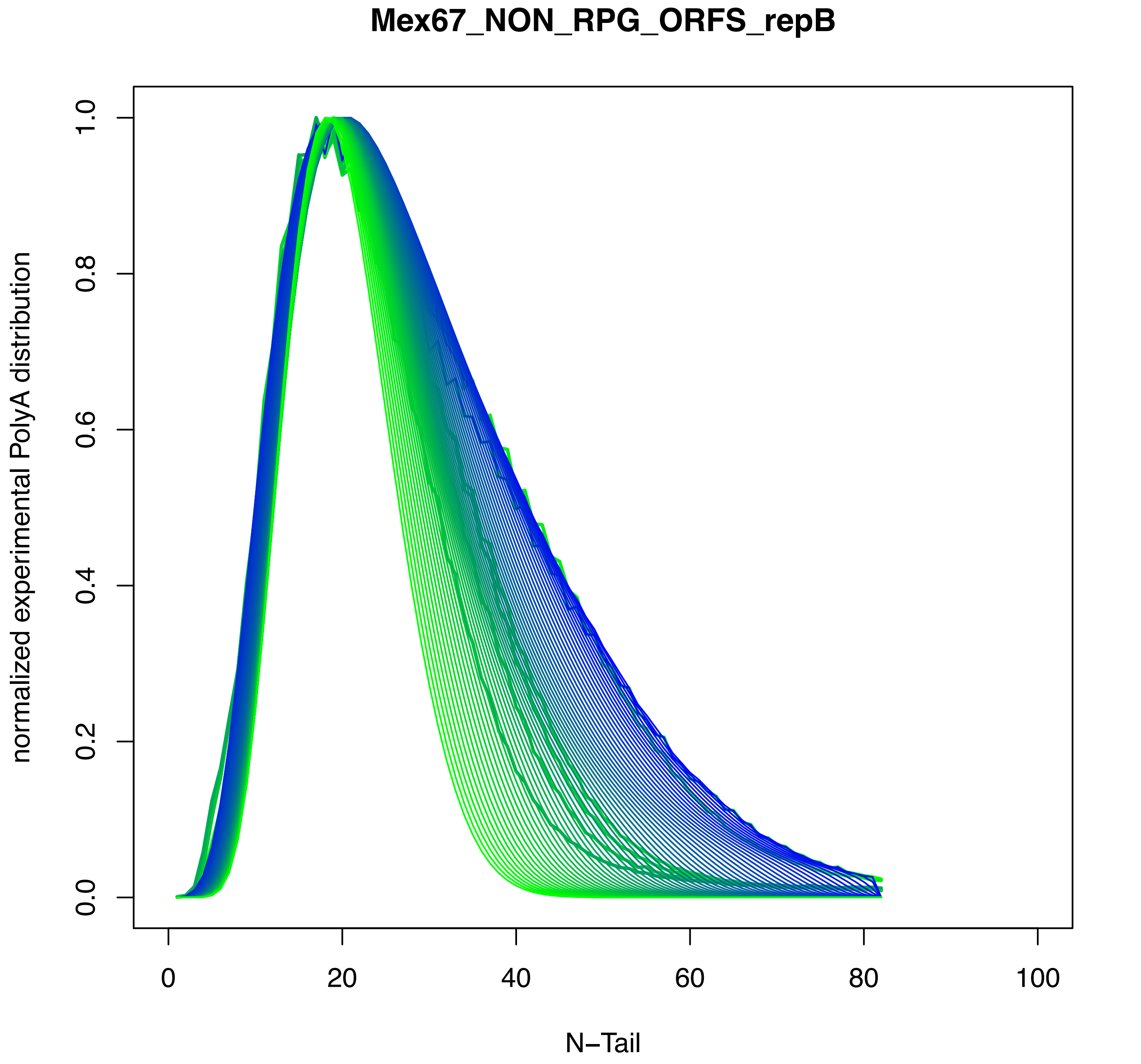
Mex67_NON_RPG_ORFS_repB
normalized experimental PolyA distribution
N−Tail
0.0
0.2
0.4
0.6
0.8
1.0
0
20
40
60
80
100

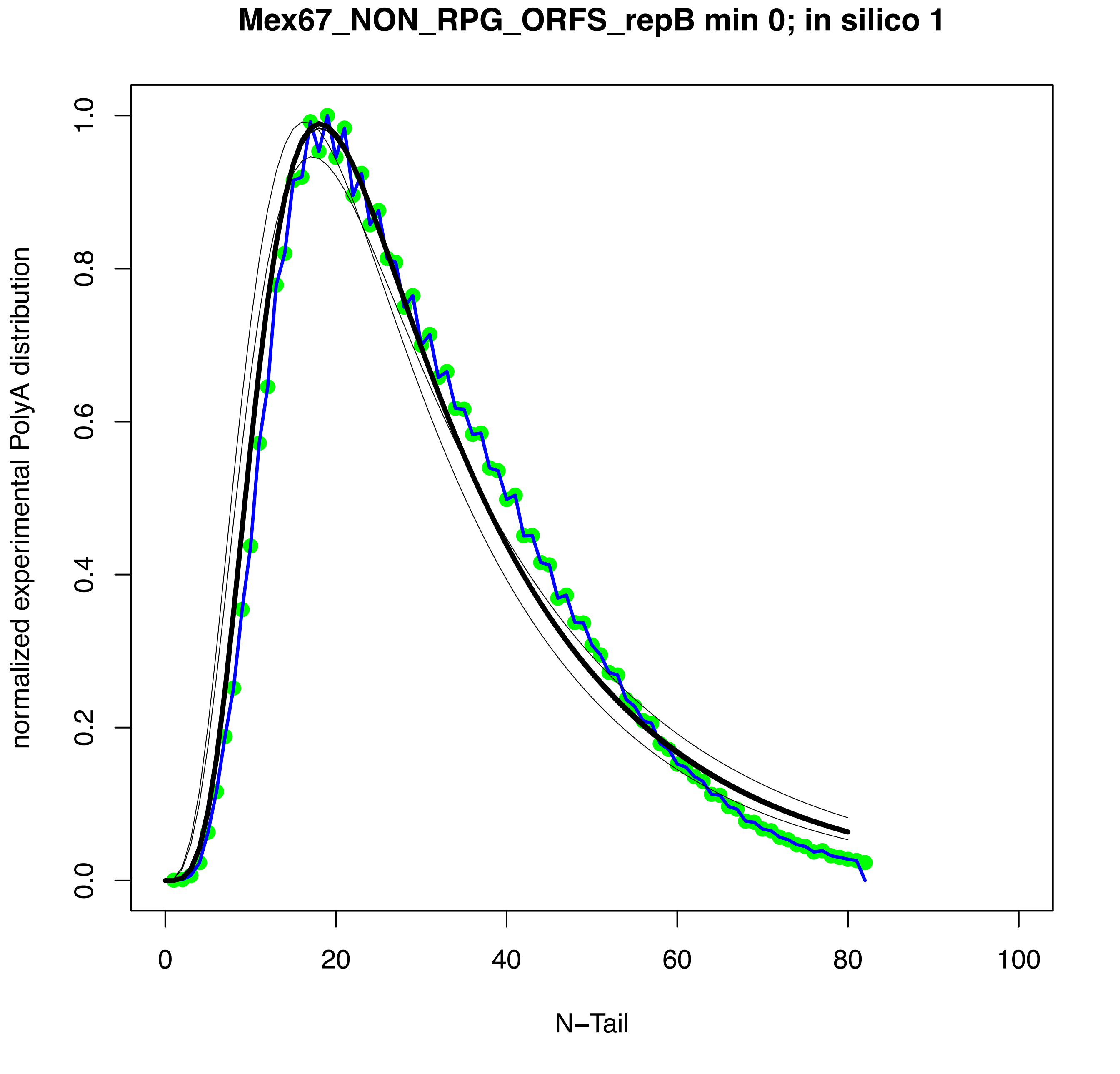

Mex67_NON_RPG_ORFS_repB min 0; in silico 1
normalized experimental PolyA distribution
N−Tail
0.0
0.2
0.4
0.6
0.8
1.0
0
20
40
60
80
100

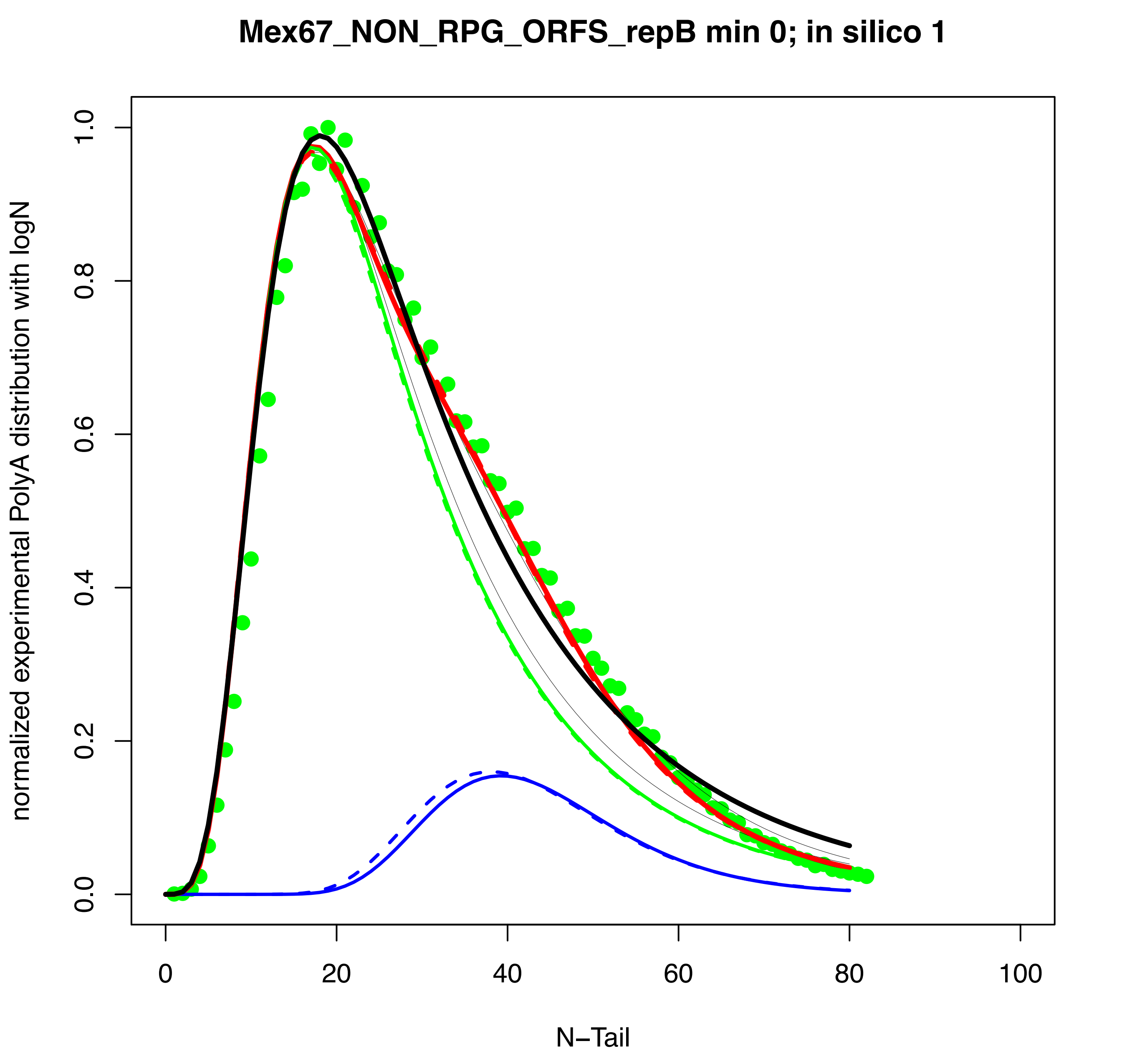

Mex67_NON_RPG_ORFS_repB min 0; in silico 1
normalized experimental PolyA distribution with logN
0.0
0.2
0.4
0.6
0.8
1.0
0
20
40
60
80
100
N−Tail

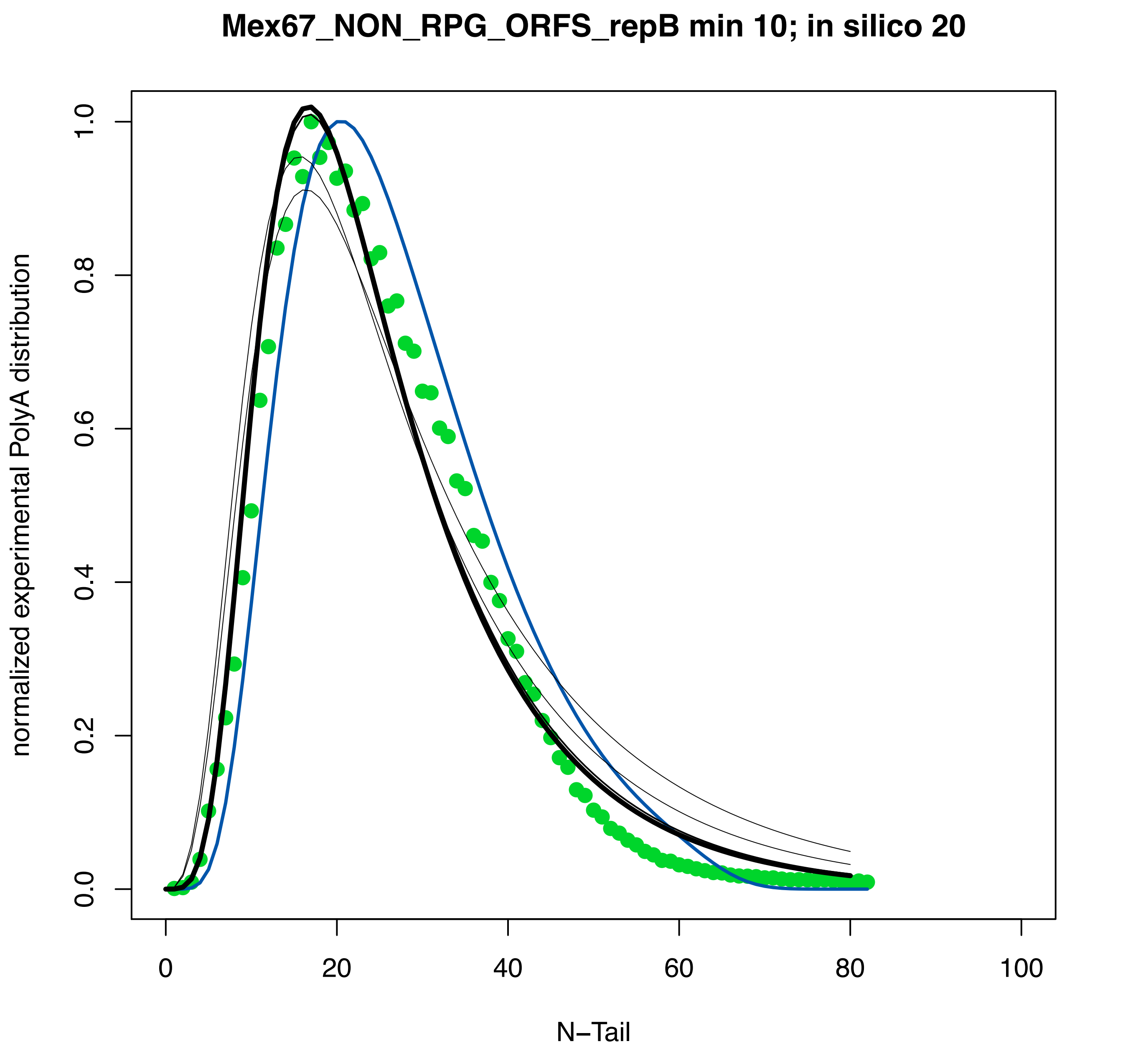
Mex67_NON_RPG_ORFS_repB min 10; in silico 20
normalized experimental PolyA distribution
0.0
0.2
0.4
0.6
0.8
1.0
0
20
40
60
80
100
N−Tail

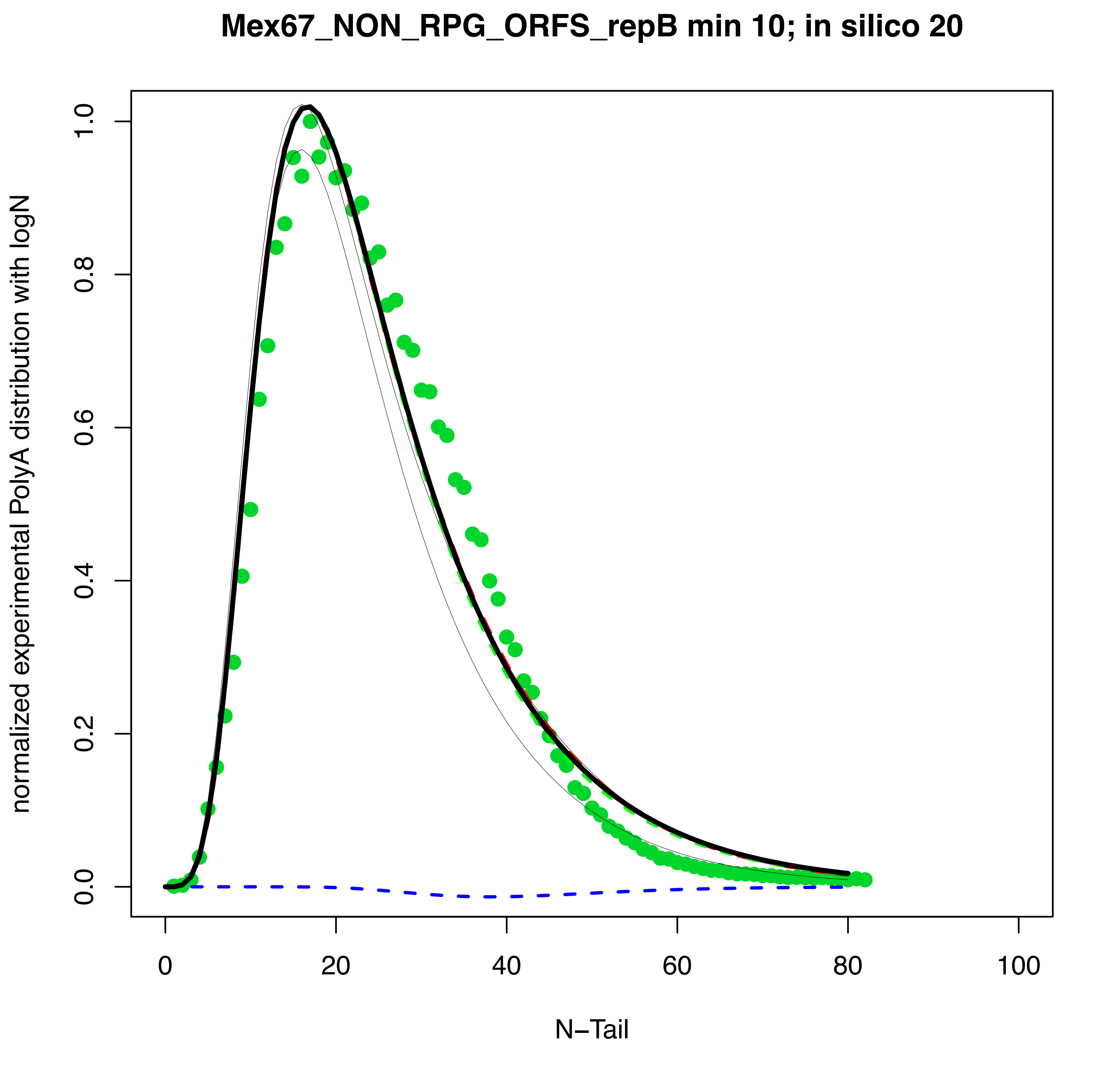
Mex67_NON_RPG_ORFS_repB min 10; in silico 20
normalized experimental PolyA distribution with logN
N−Tail
0.0
0.2
0.4
0.6
0.8
1.0
0
20
40
60
80
100

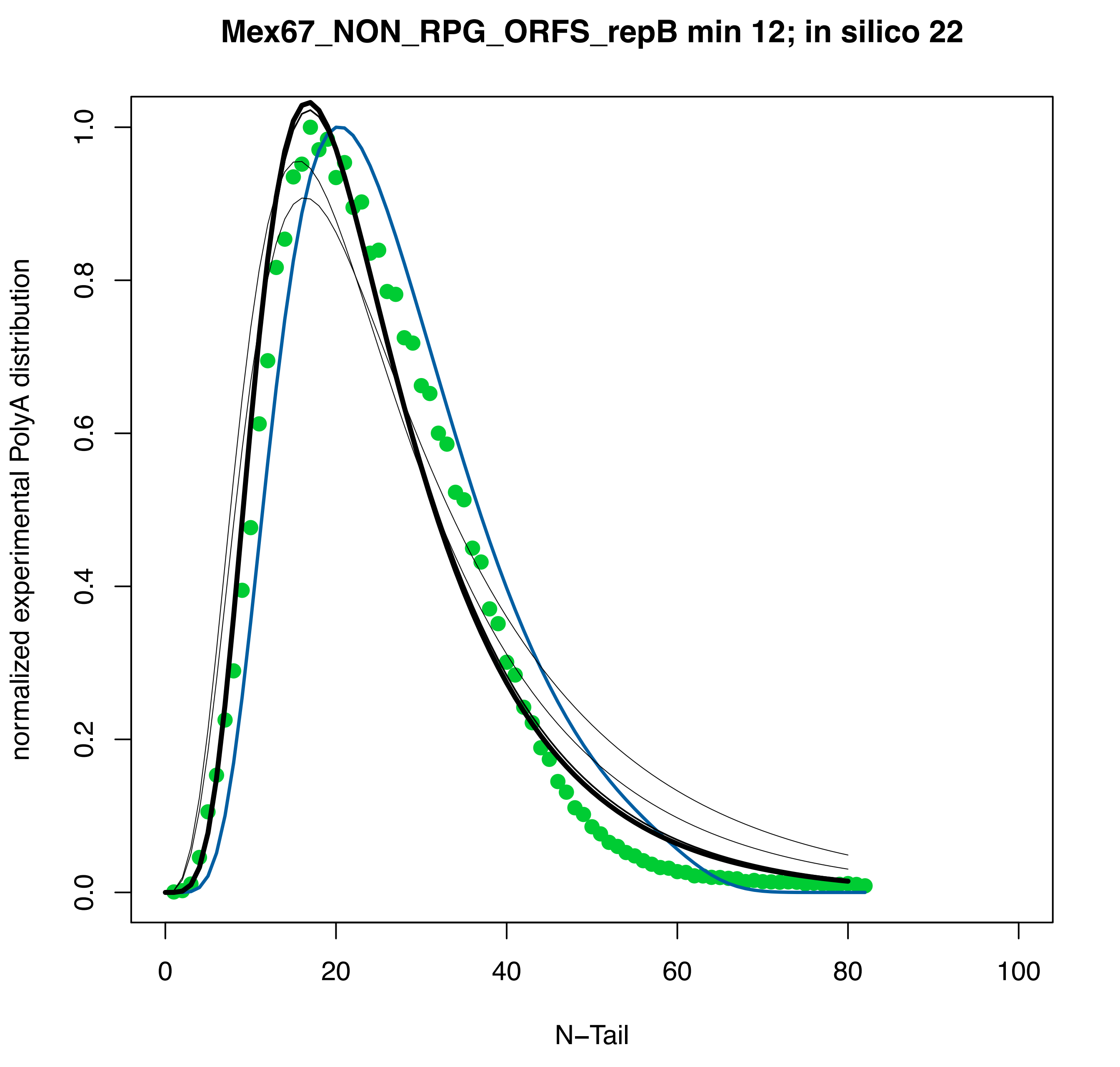

Mex67_NON_RPG_ORFS_repB min 12; in silico 22
normalized experimental PolyA distribution
N−Tail
0.0
0.2
0.4
0.6
0.8
1.0
0
20
40
60
80
100

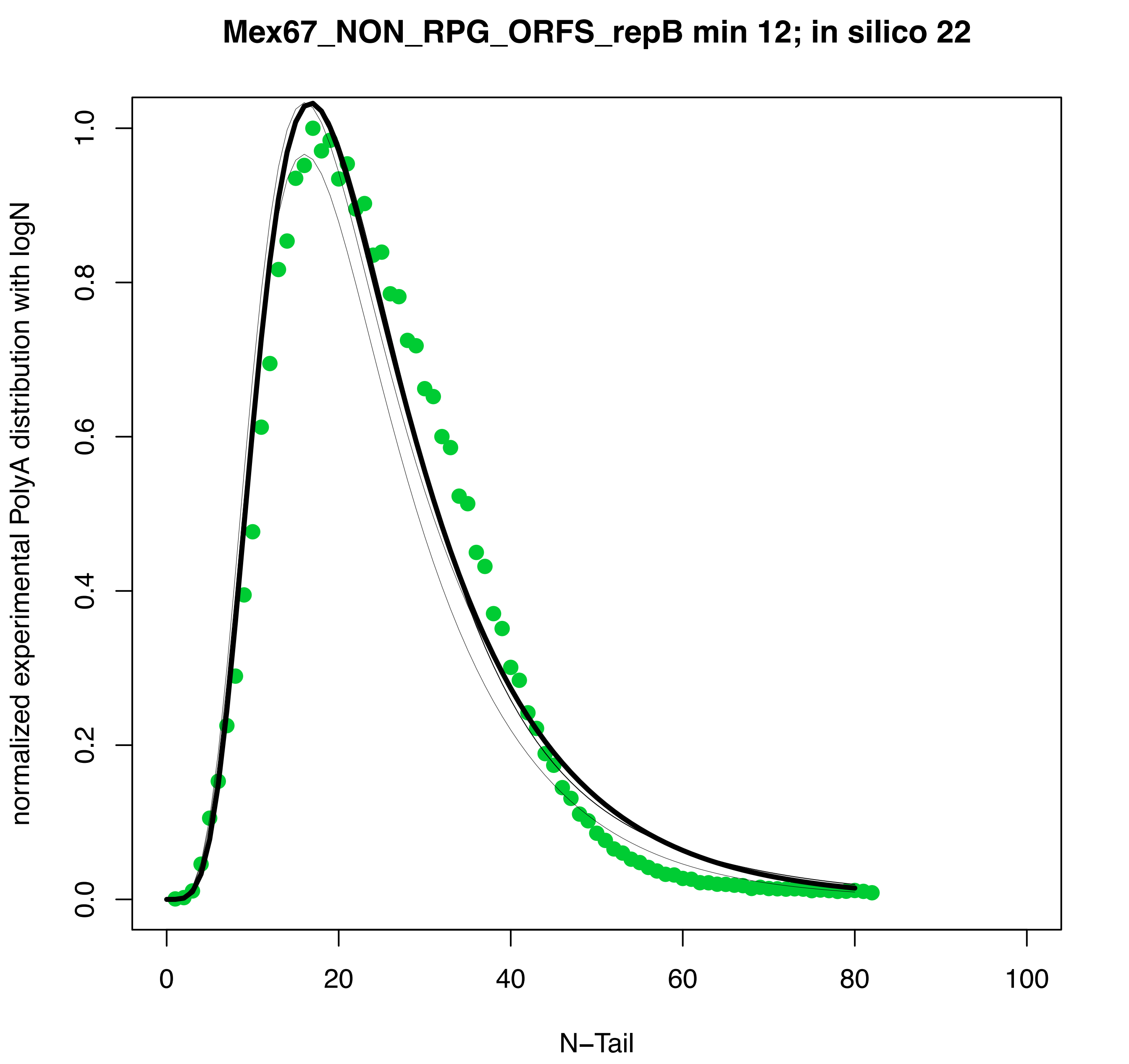

Mex67_NON_RPG_ORFS_repB min 12; in silico 22
normalized experimental PolyA distribution with logN
N−Tail
0.0
0.2
0.4
0.6
0.8
1.0
0
20
40
60
80
100

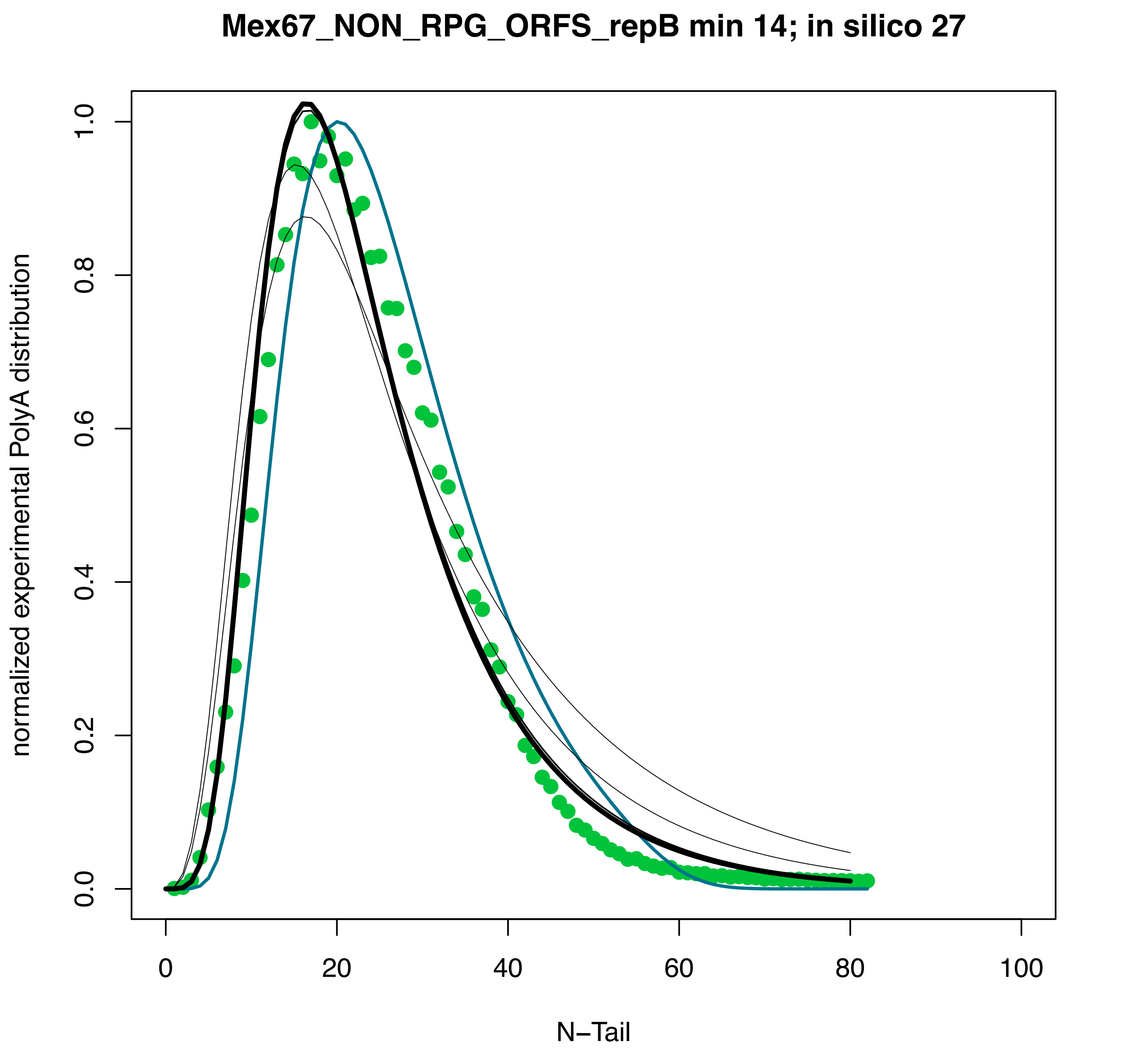

Mex67_NON_RPG_ORFS_repB min 14; in silico 27
normalized experimental PolyA distribution
N−Tail
0.0
0.2
0.4
0.6
0.8
1.0
0
20
40
60
80
100

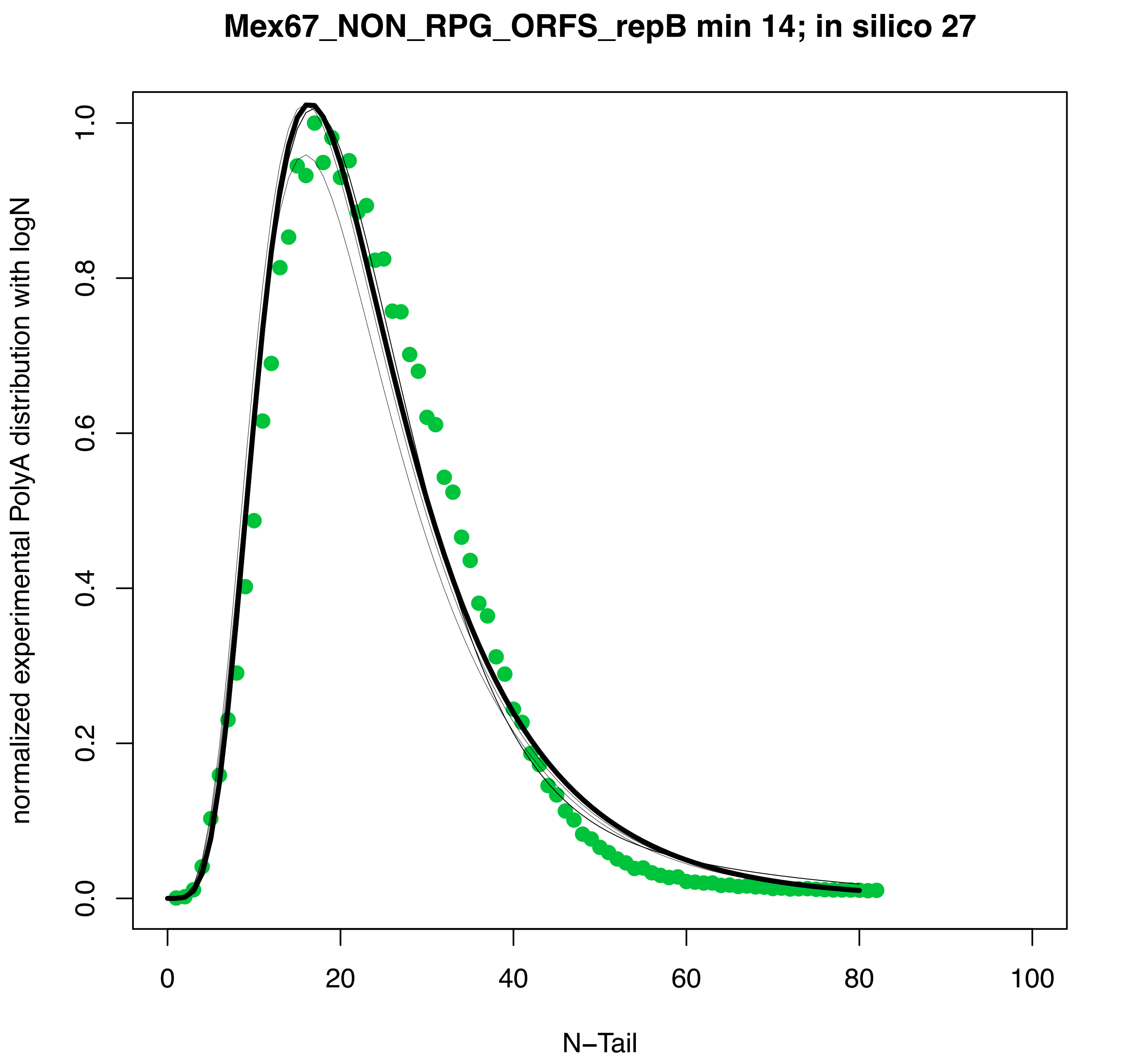

Mex67_NON_RPG_ORFS_repB min 14; in silico 27
normalized experimental PolyA distribution with logN
N−Tail
0.0
0.2
0.4
0.6
0.8
1.0
0
20
40
60
80
100

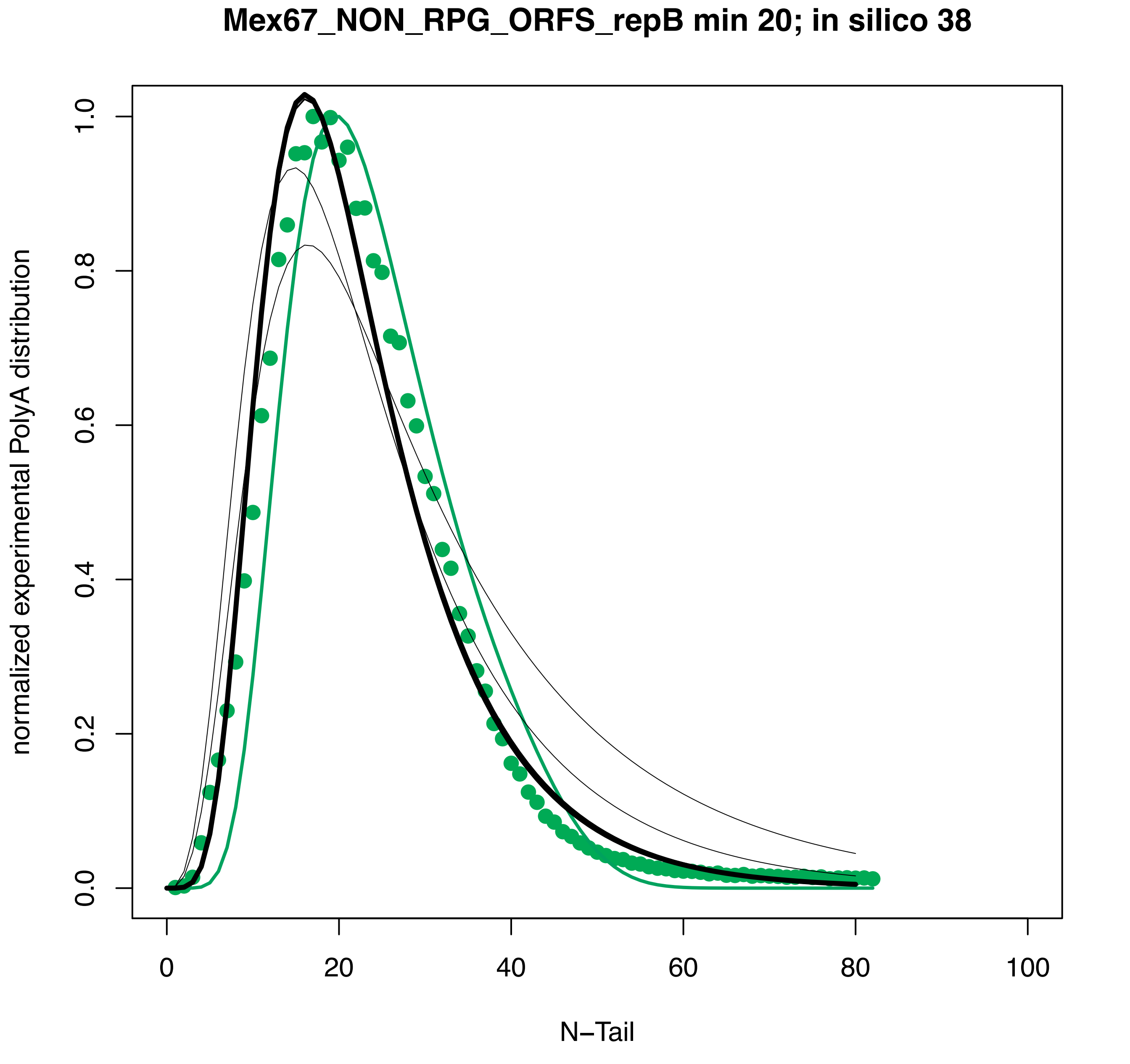

Mex67_NON_RPG_ORFS_repB min 20; in silico 38
normalized experimental PolyA distribution
N−Tail
0.0
0.2
0.4
0.6
0.8
1.0
0
20
40
60
80
100

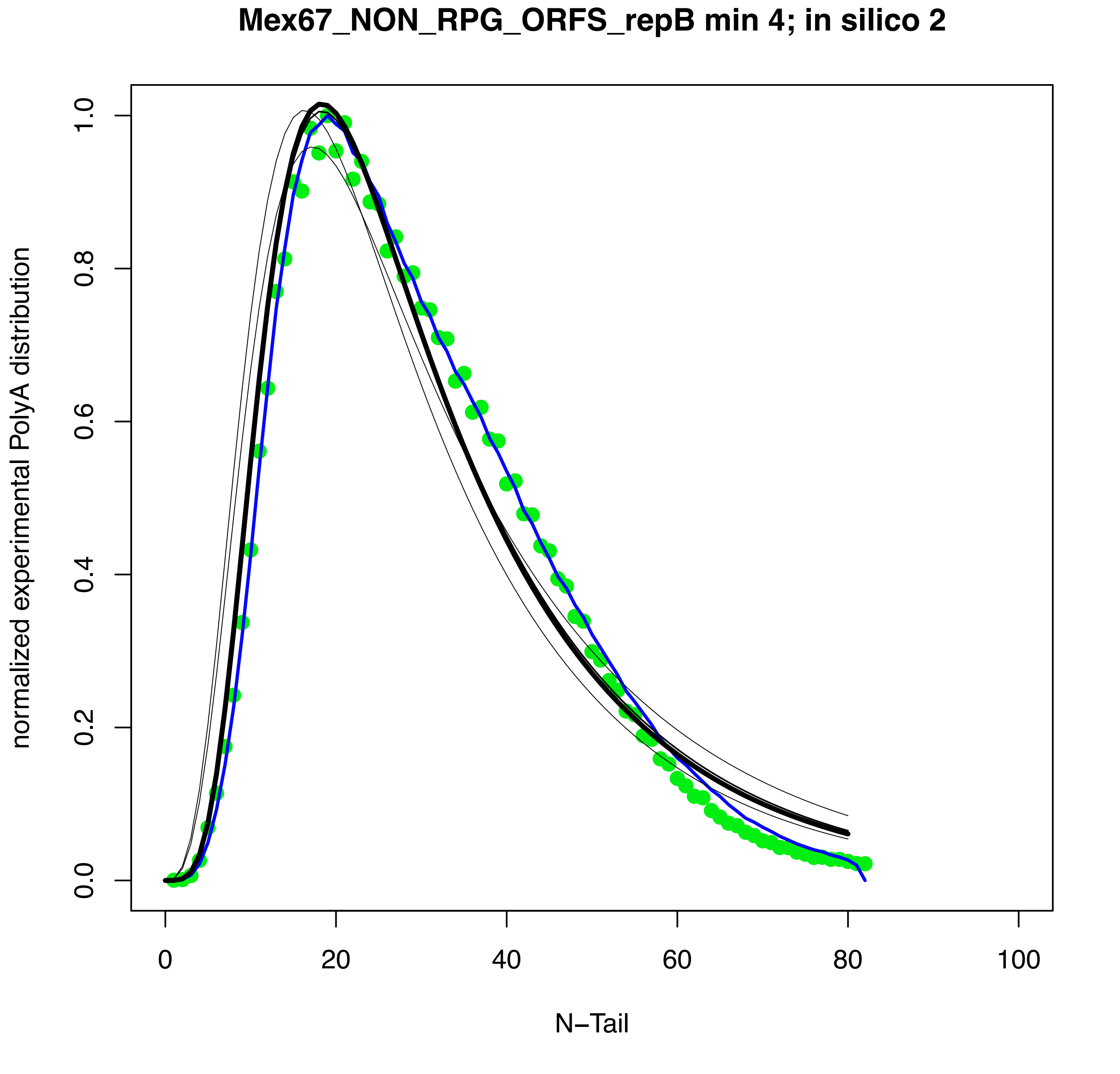
Mex67_NON_RPG_ORFS_repB min 4; in silico 2
normalized experimental PolyA distribution
N−Tail
0.0
0.2
0.4
0.6
0.8
1.0
0
20
40
60
80
100

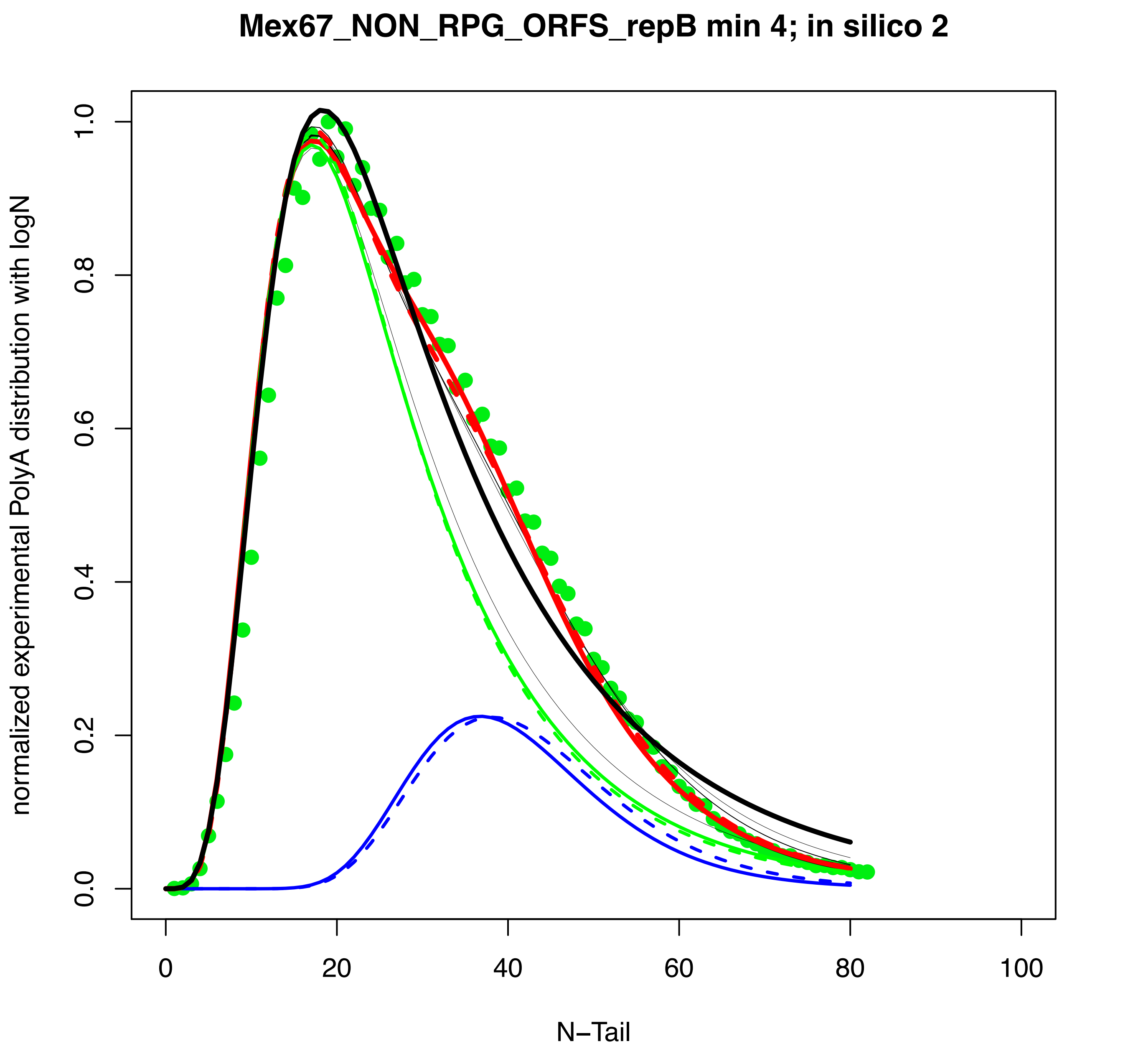

Mex67_NON_RPG_ORFS_repB min 4; in silico 2
normalized experimental PolyA distribution with logN
N−Tail
0.0
0.2
0.4
0.6
0.8
1.0
0
20
40
60
80
100

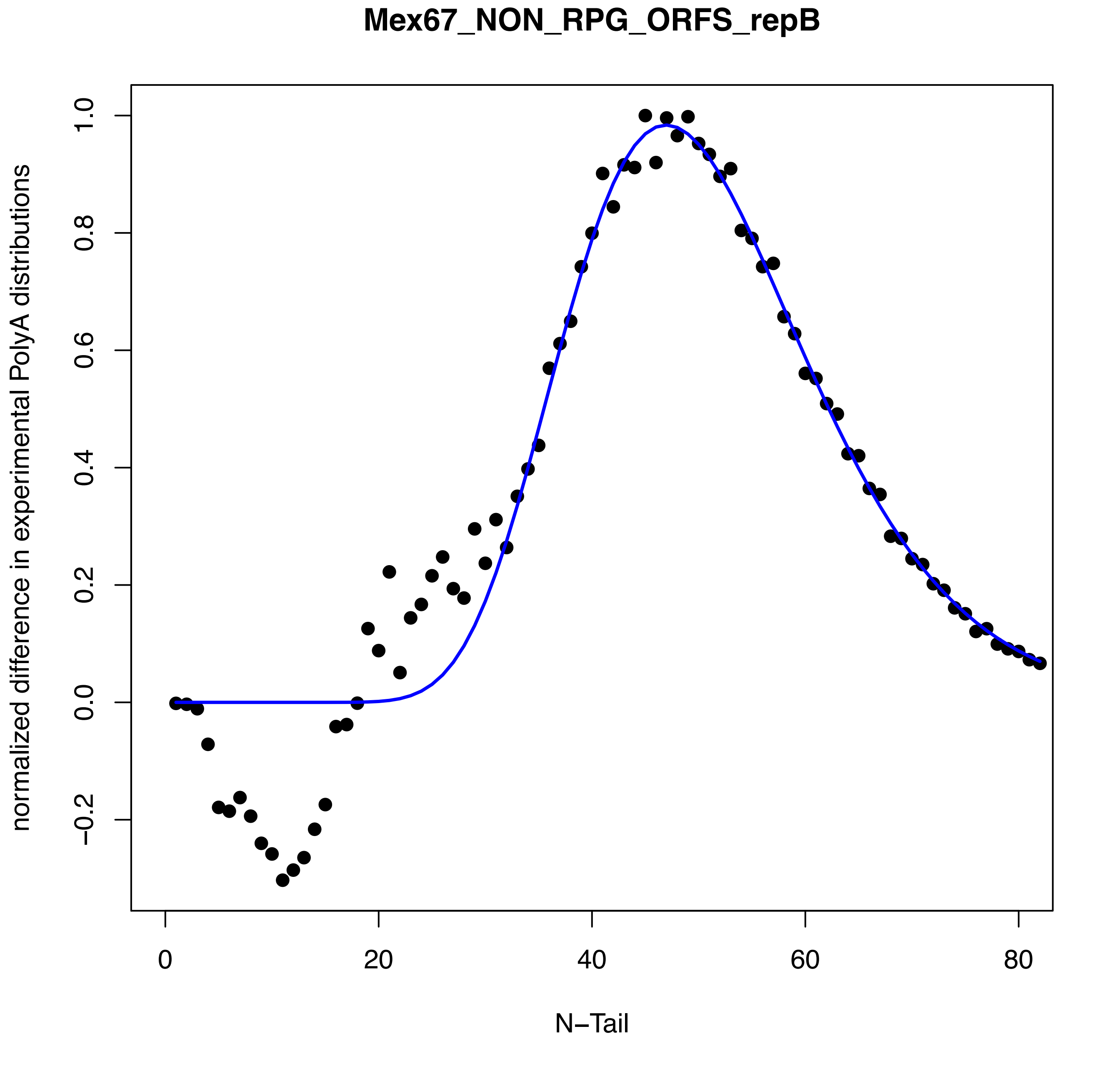

Mex67_NON_RPG_ORFS_repB
normalized difference in experimental PolyA distributions
N−Tail
−0.2
0.0
0.2
0.4
0.6
0.8
1.0
0
20
40
60
80

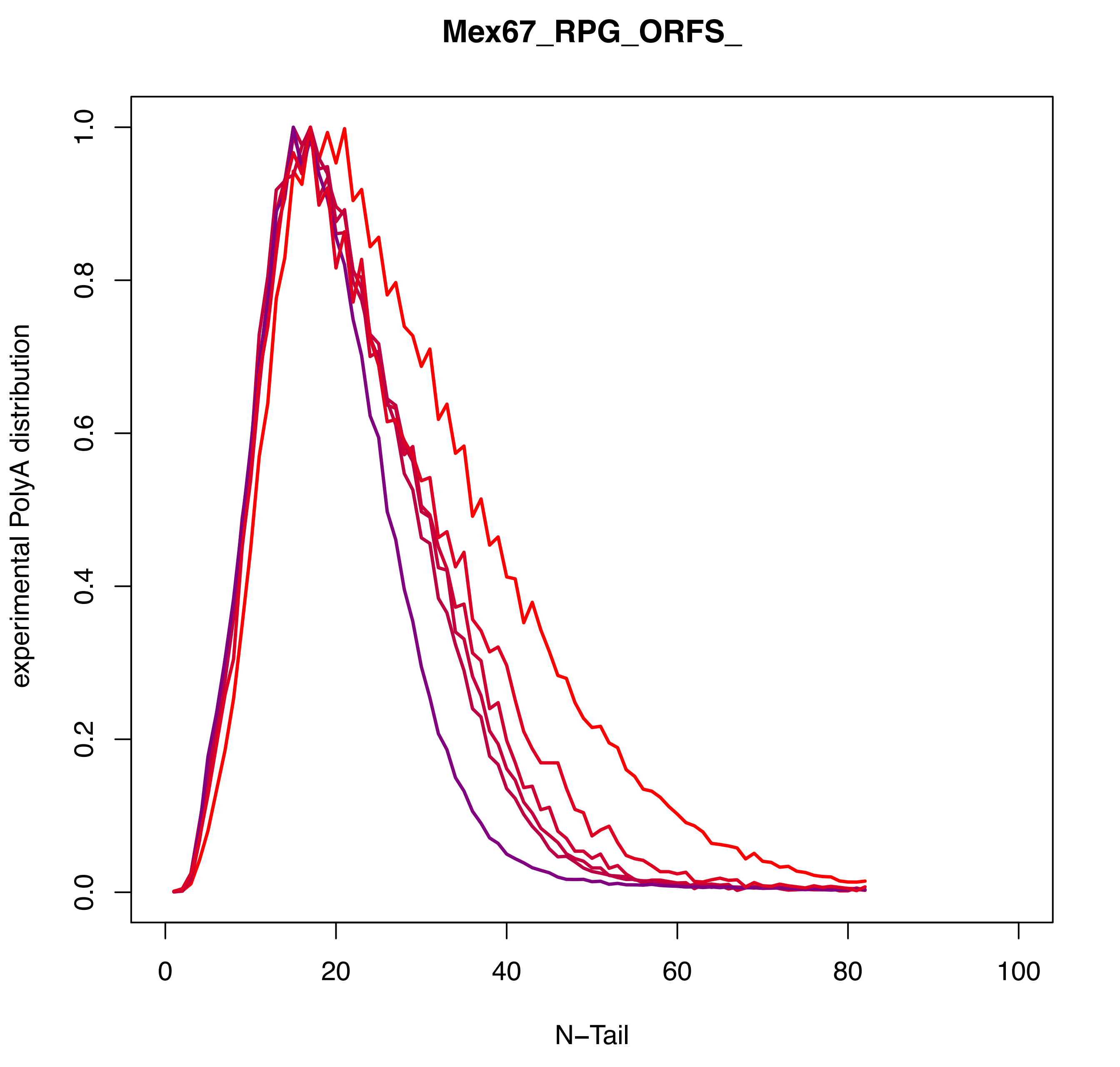

Mex67_RPG_ORFS_
experimental PolyA distribution
N−Tail
0.0
0.2
0.4
0.6
0.8
1.0
0
20
40
60
80
100

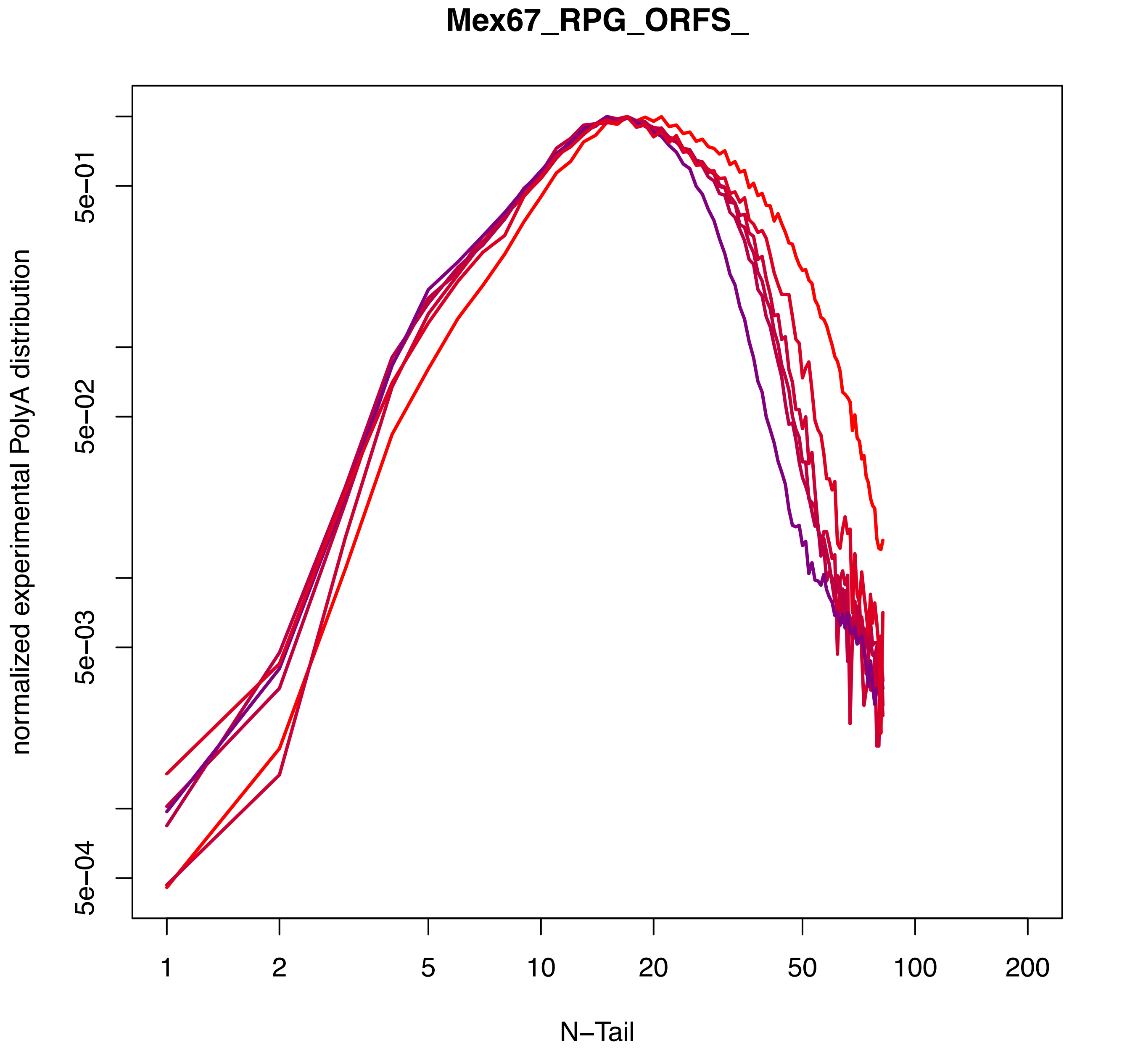

Mex67_RPG_ORFS_
normalized experimental PolyA distribution
5e−04
5e−03
5e−02
5e−01
N−Tail
1
2
5
10
20
50
100
200

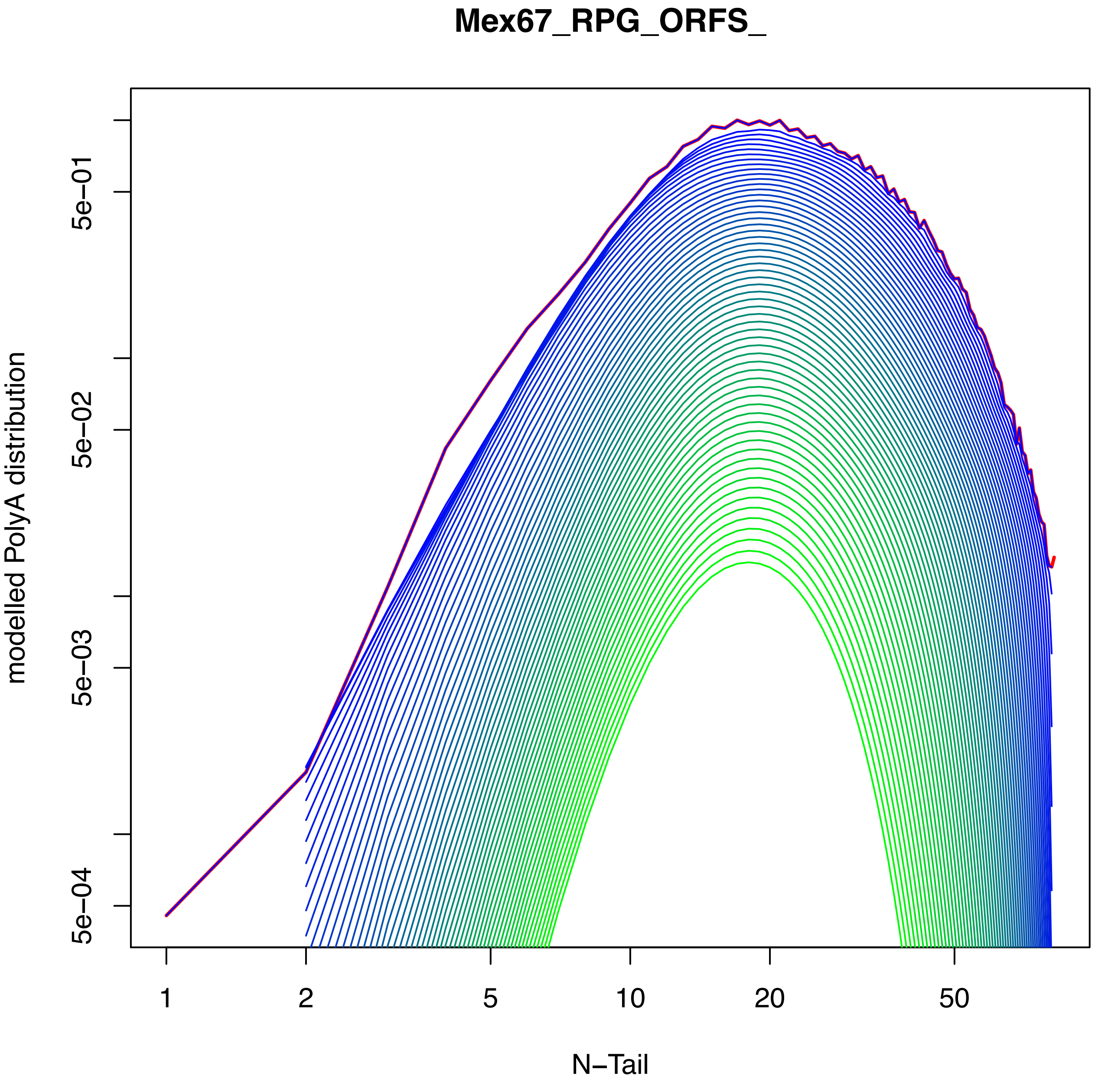
Mex67_RPG_ORFS_
modelled PolyA distribution
5e−01
5e−02
5e−03
5e−04
1
2
5
10
20
50
N−Tail

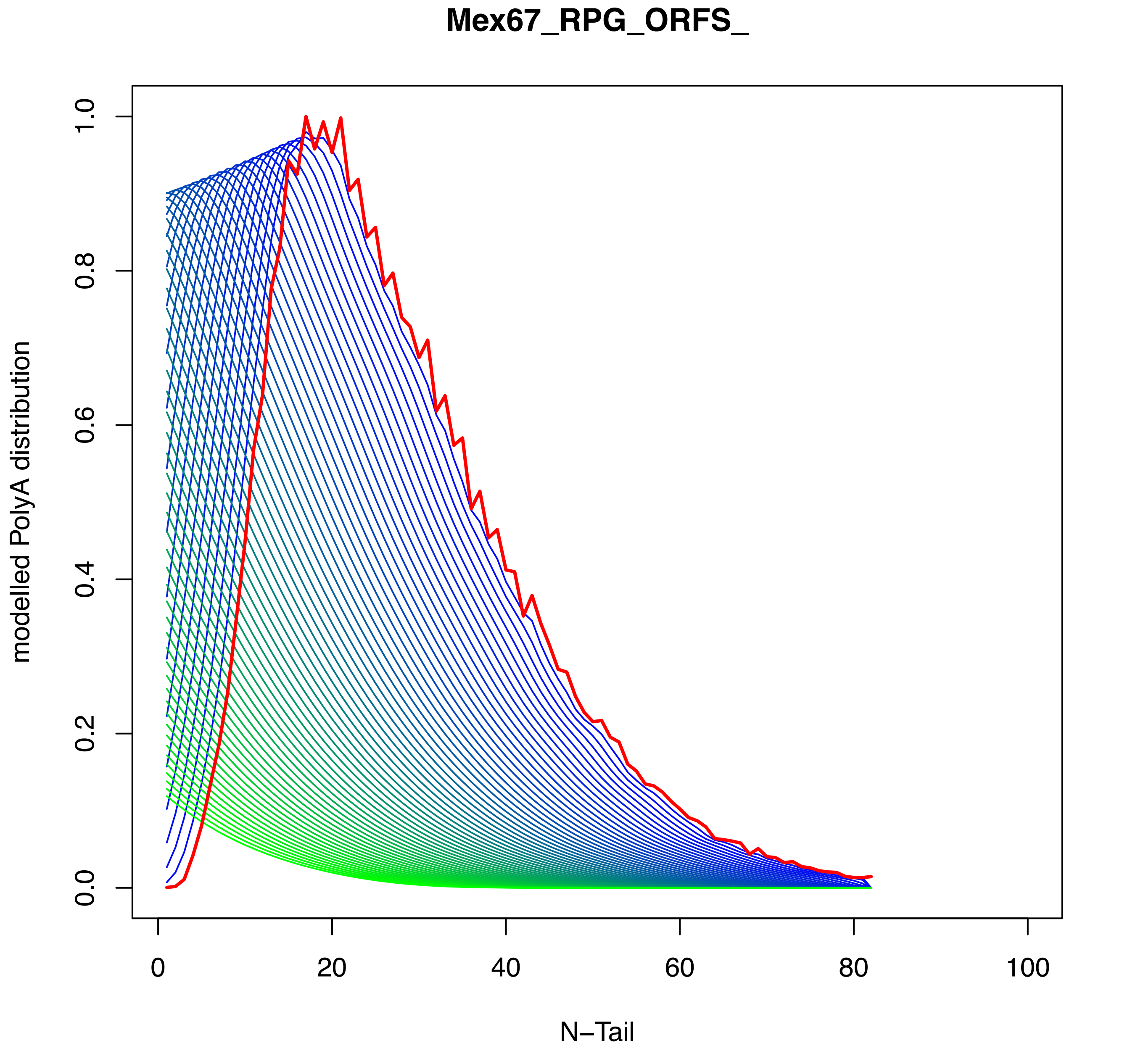

Mex67_RPG_ORFS_
modelled PolyA distribution
N−Tail
0.0
0.2
0.4
0.6
0.8
1.0
0
20
40
60
80
100

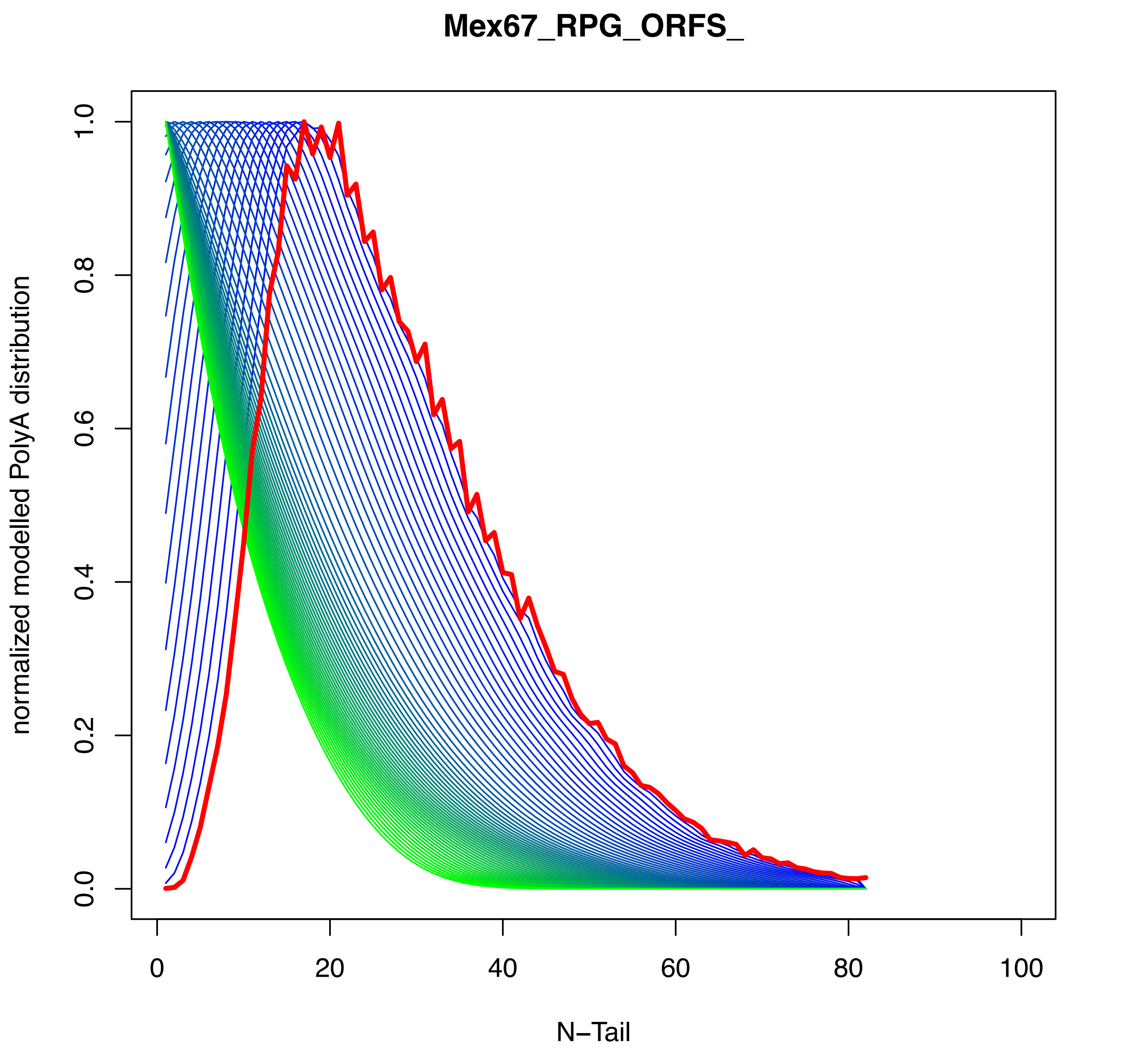
Mex67_RPG_ORFS_
normalized modelled PolyA distribution
N−Tail
0.0
0.2
0.4
0.6
0.8
1.0
0
20
40
60
80
100

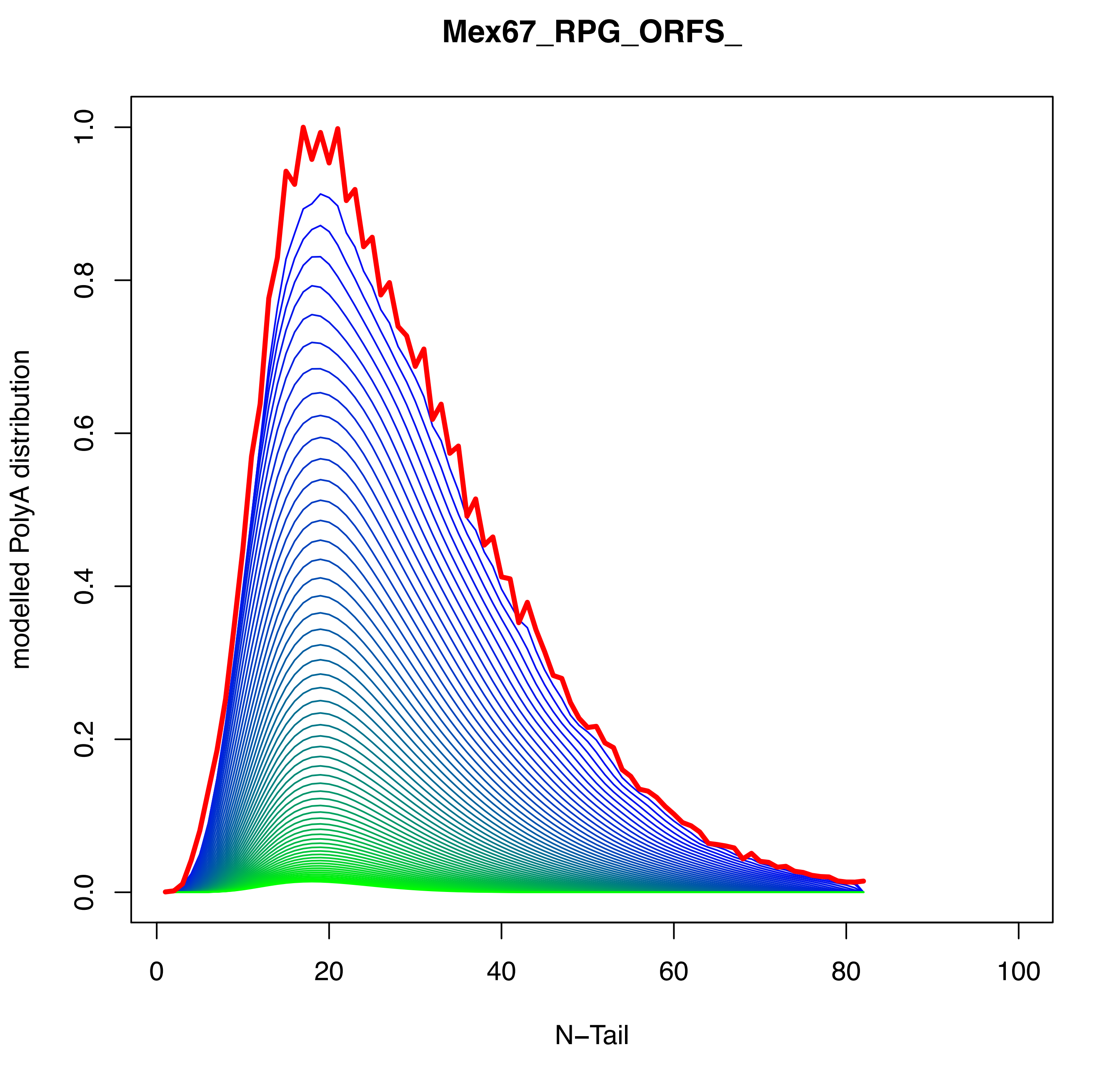

Mex67_RPG_ORFS_
modelled PolyA distribution
0.0
0.2
0.4
0.6
0.8
1.0
0
20
40
60
80
100
N−Tail

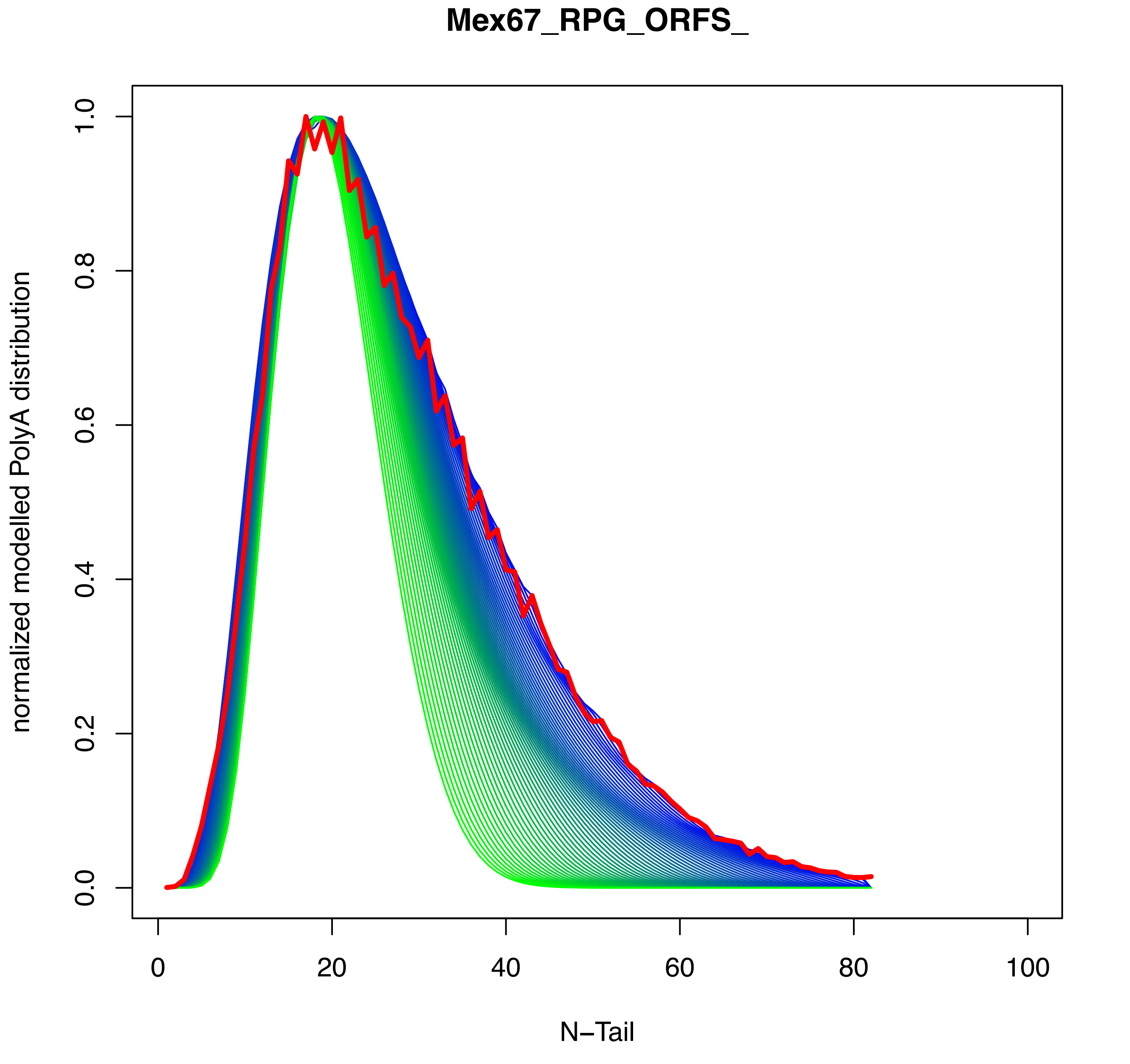
Mex67_RPG_ORFS_
normalized modelled PolyA distribution
N−Tail
0.0
0.2
0.4
0.6
0.8
1.0
0
20
40
60
80
100

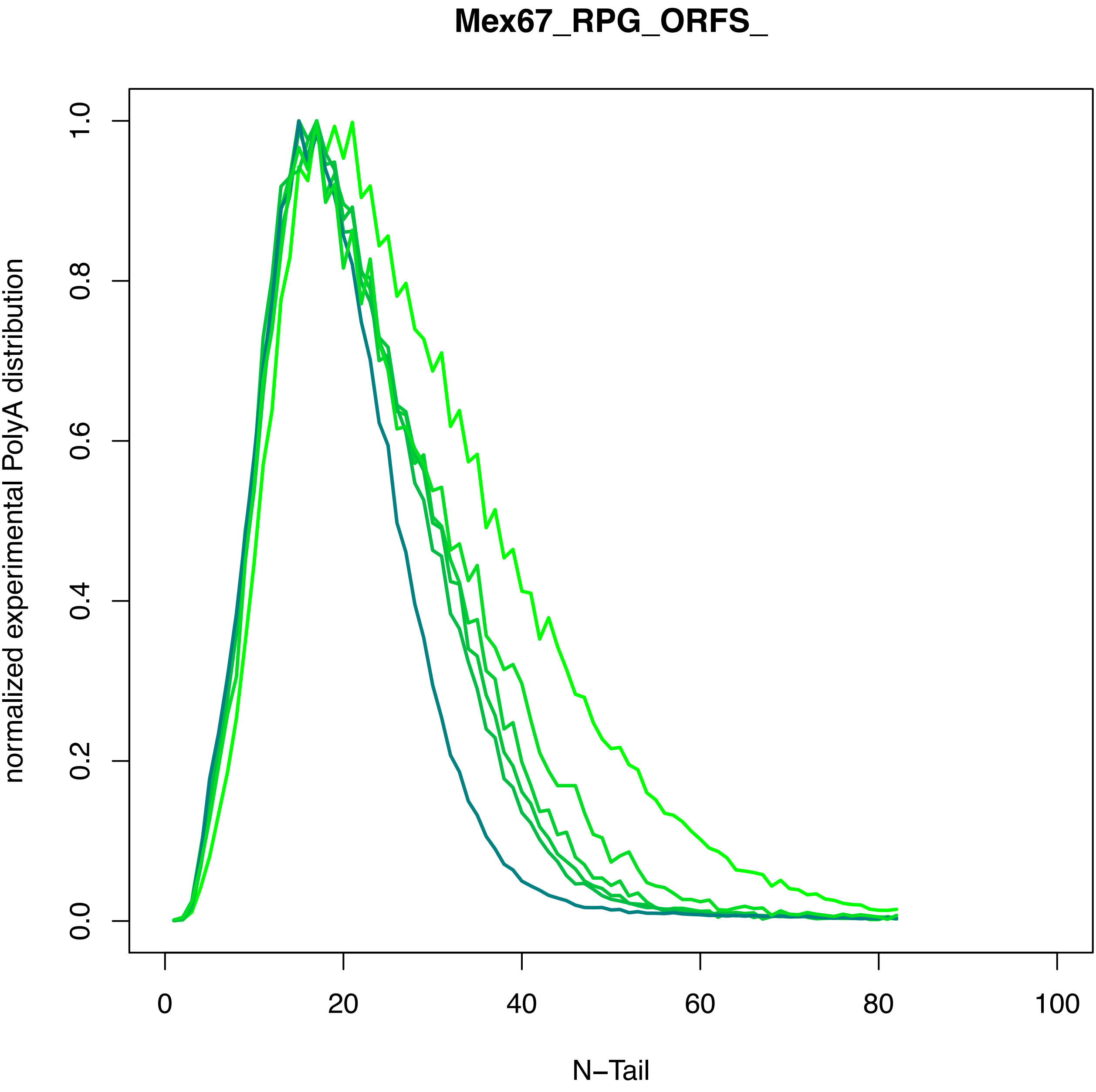

Mex67_RPG_ORFS_
normalized experimental PolyA distribution
N−Tail
0.0
0.2
0.4
0.6
0.8
1.0
0
20
40
60
80
100

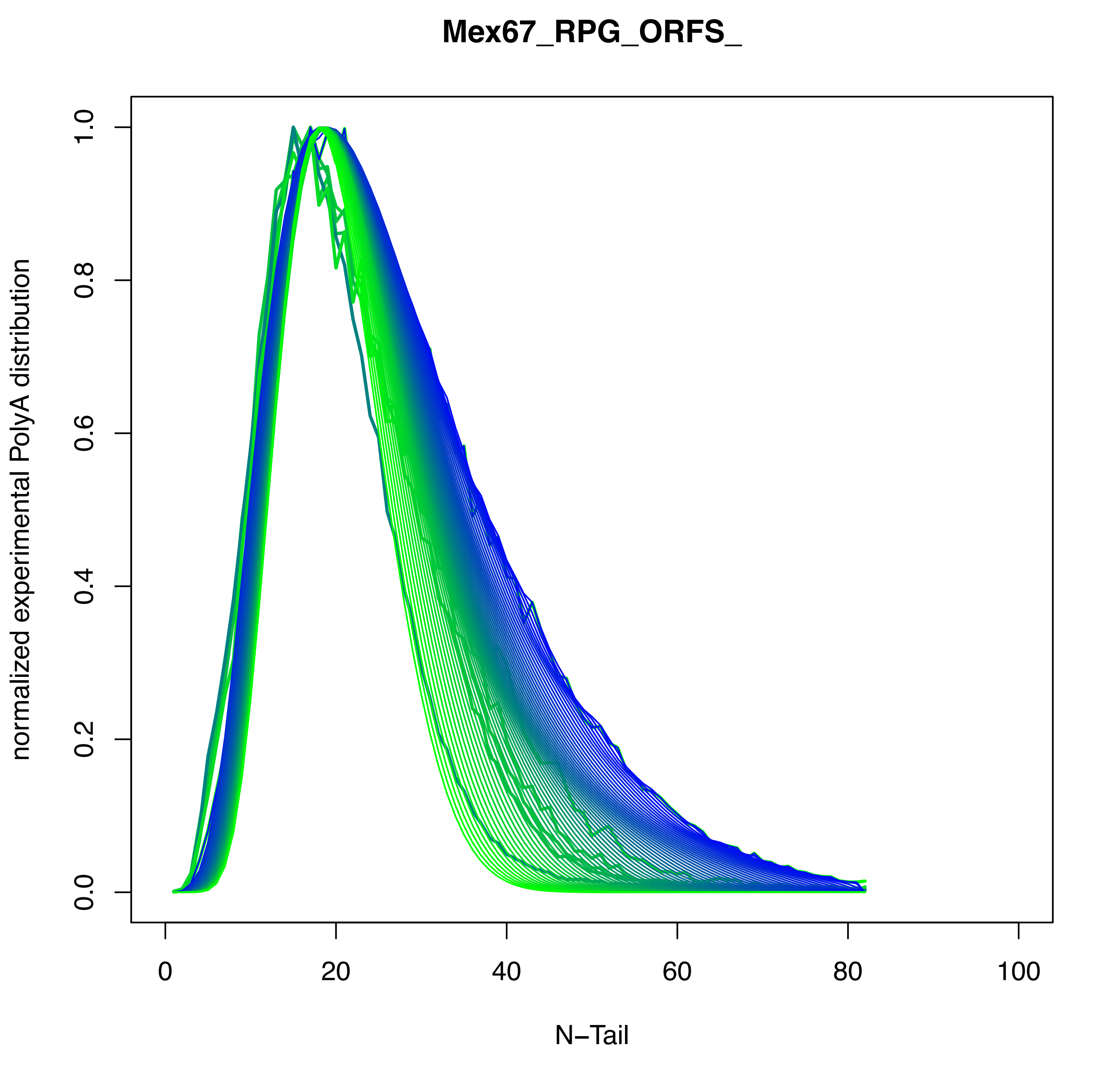
Mex67_RPG_ORFS_
normalized experimental PolyA distribution
N−Tail
0.0
0.2
0.4
0.6
0.8
1.0
0
20
40
60
80
100

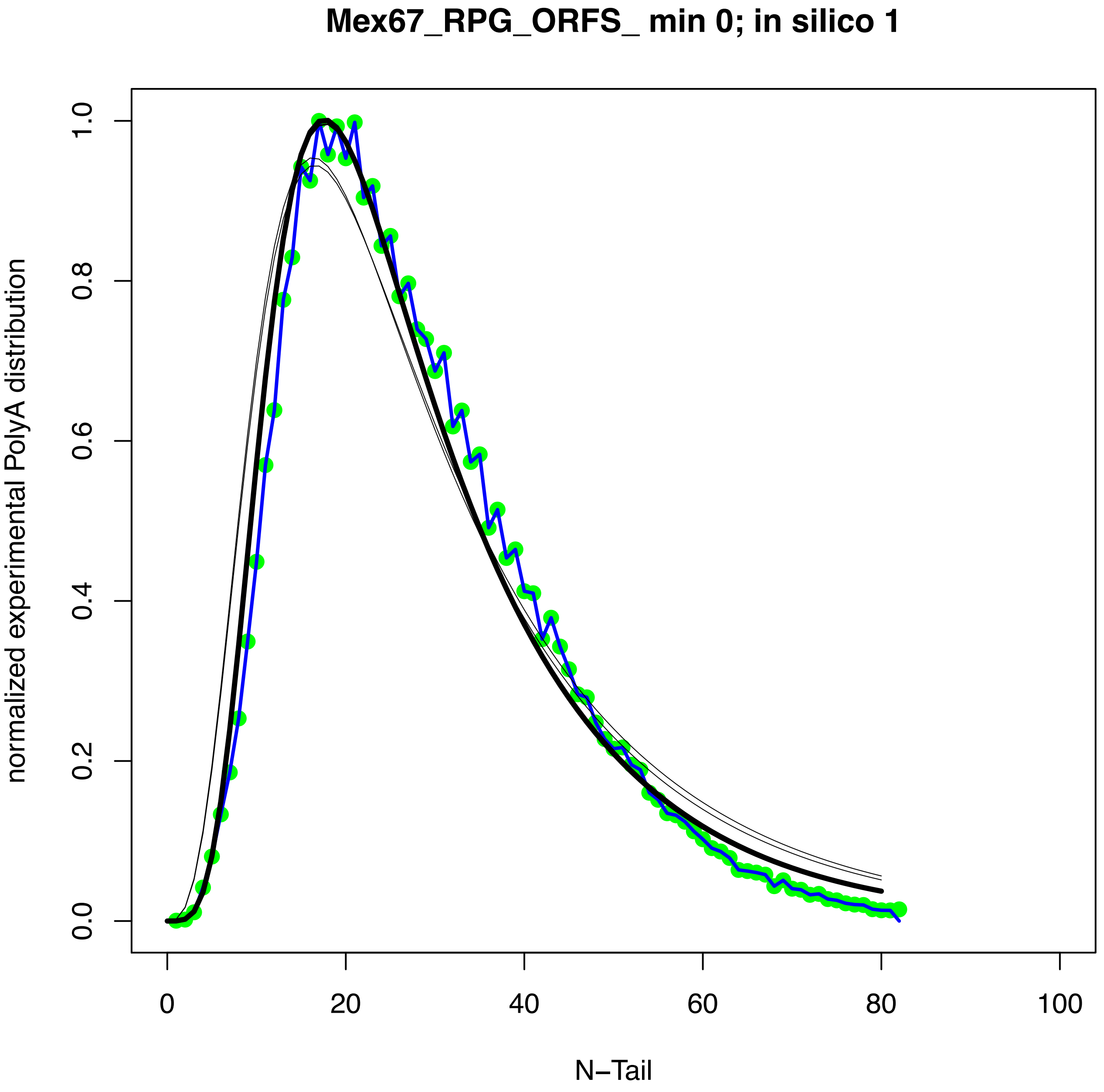
Mex67_RPG_ORFS_ min 0; in silico 1
normalized experimental PolyA distribution
N−Tail
0.0
0.2
0.4
0.6
0.8
1.0
0
20
40
60
80
100

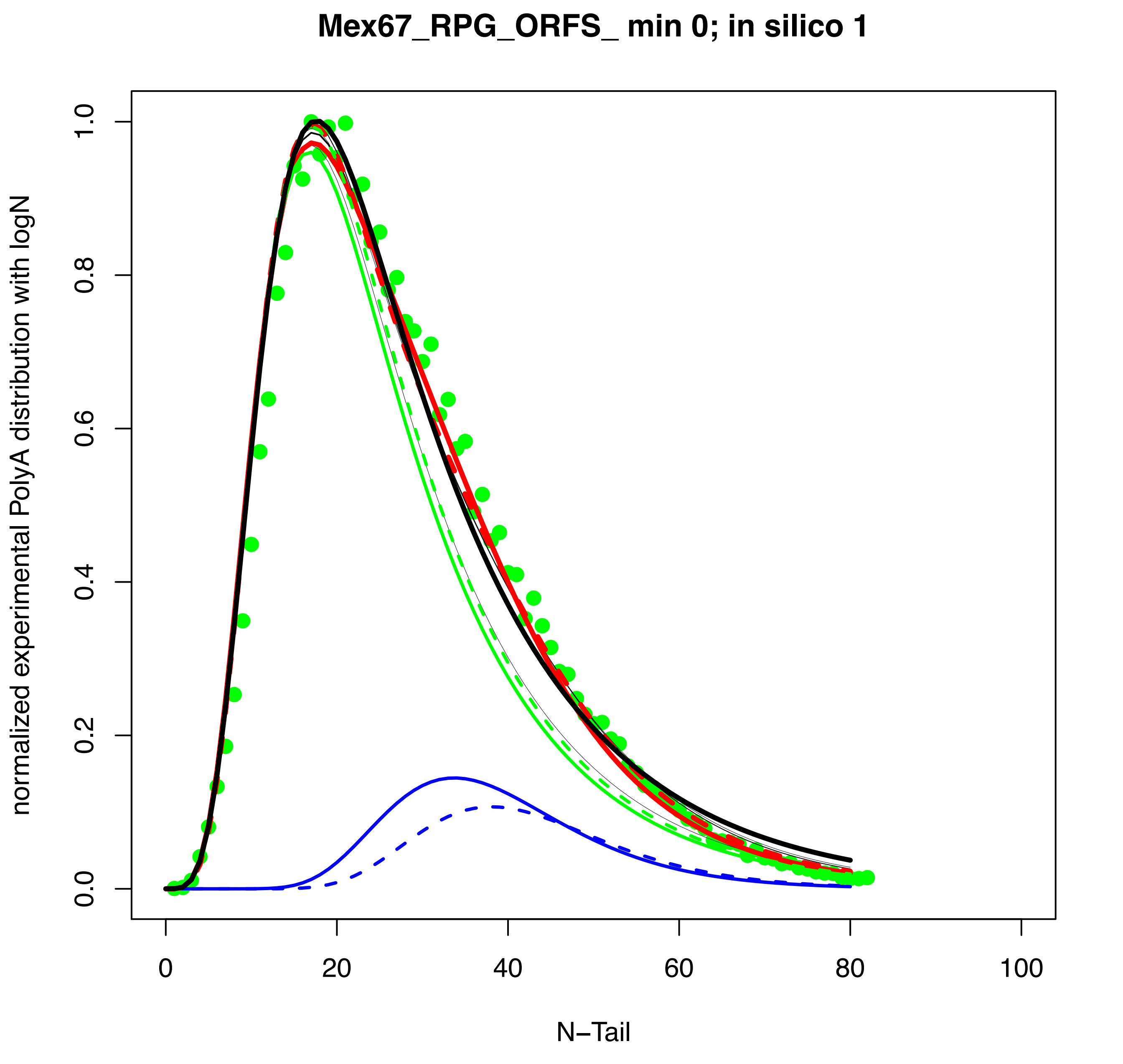

Mex67_RPG_ORFS_ min 0; in silico 1
normalized experimental PolyA distribution with logN
N−Tail
0.0
0.2
0.4
0.6
0.8
1.0
0
20
40
60
80
100

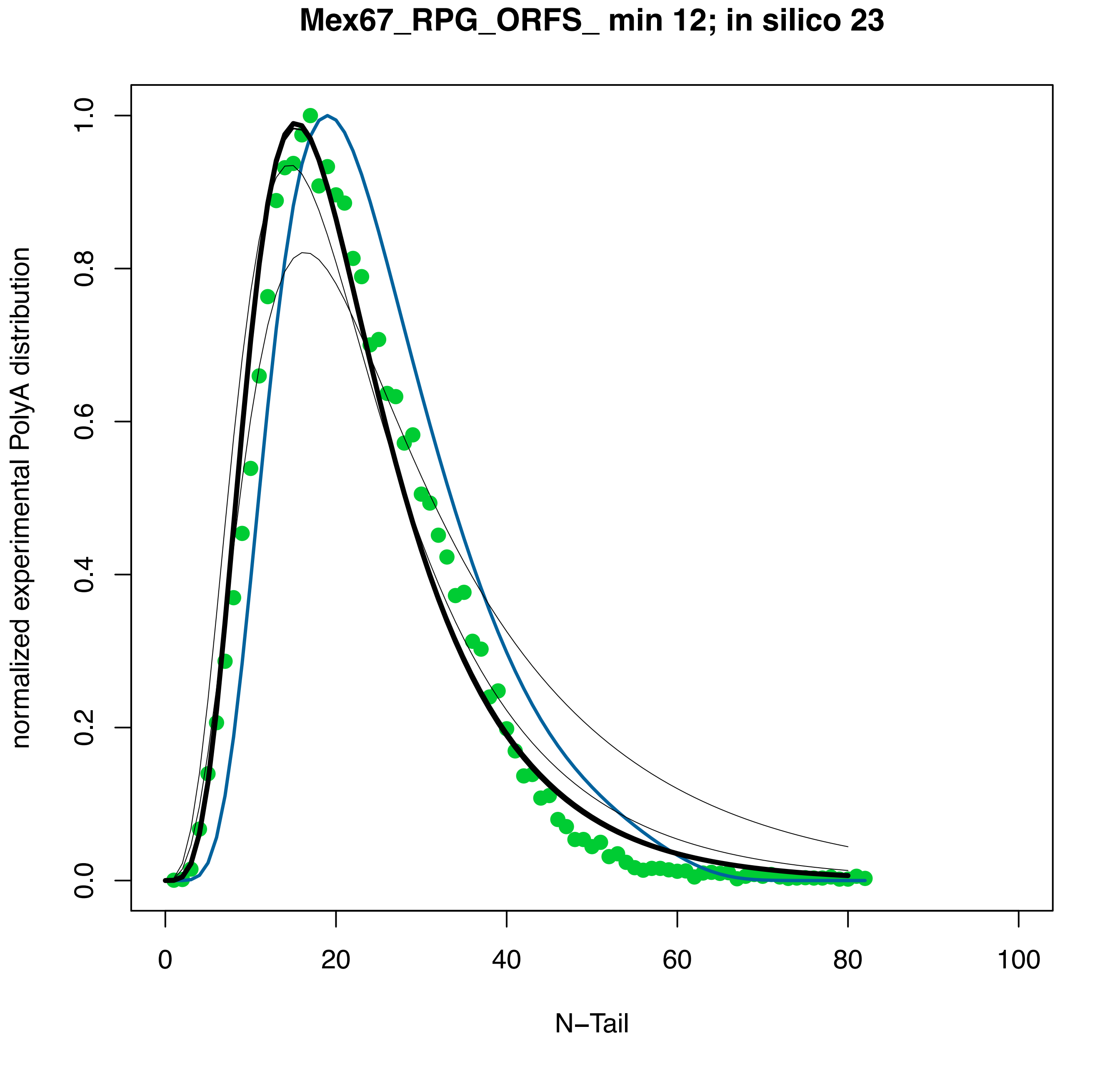

Mex67_RPG_ORFS_ min 12; in silico 23
normalized experimental PolyA distribution
N−Tail
0.0
0.2
0.4
0.6
0.8
1.0
0
20
40
60
80
100

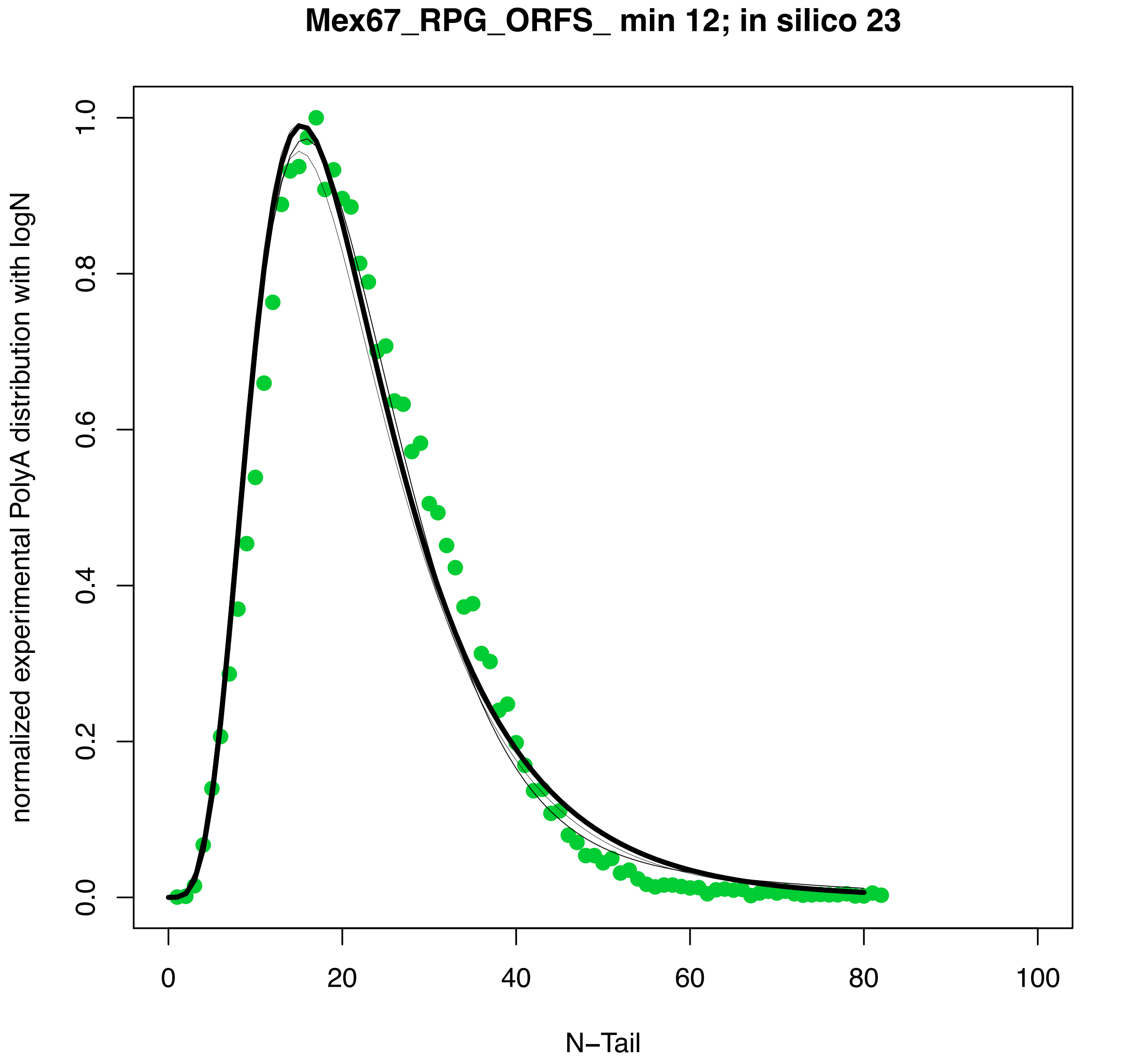
Mex67_RPG_ORFS_ min 12; in silico 23
normalized experimental PolyA distribution with logN
N−Tail
0.0
0.2
0.4
0.6
0.8
1.0
0
20
40
60
80
100

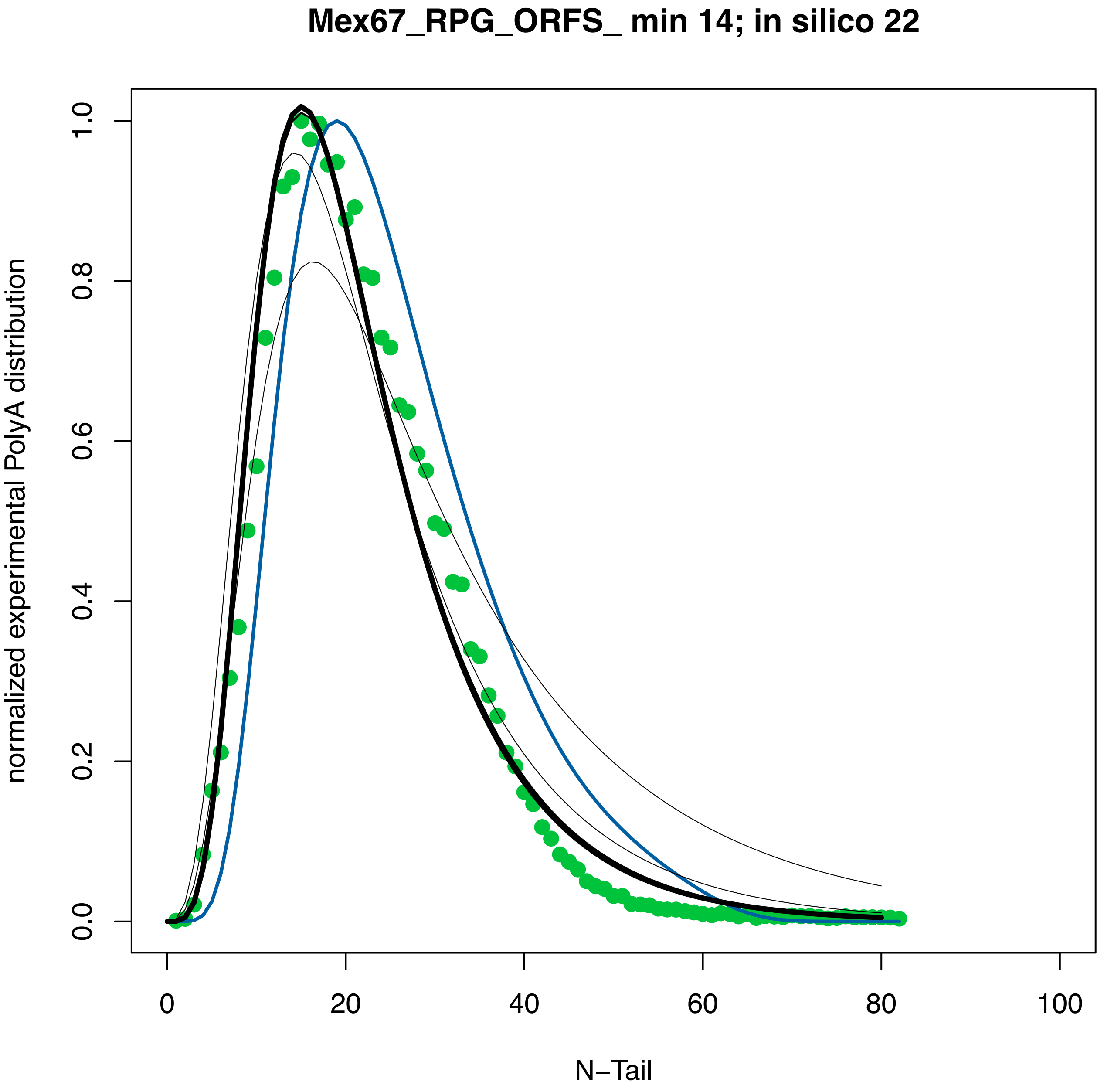

Mex67_RPG_ORFS_ min 14; in silico 22
normalized experimental PolyA distribution
N−Tail
0.0
0.2
0.4
0.6
0.8
1.0
0
20
40
60
80
100

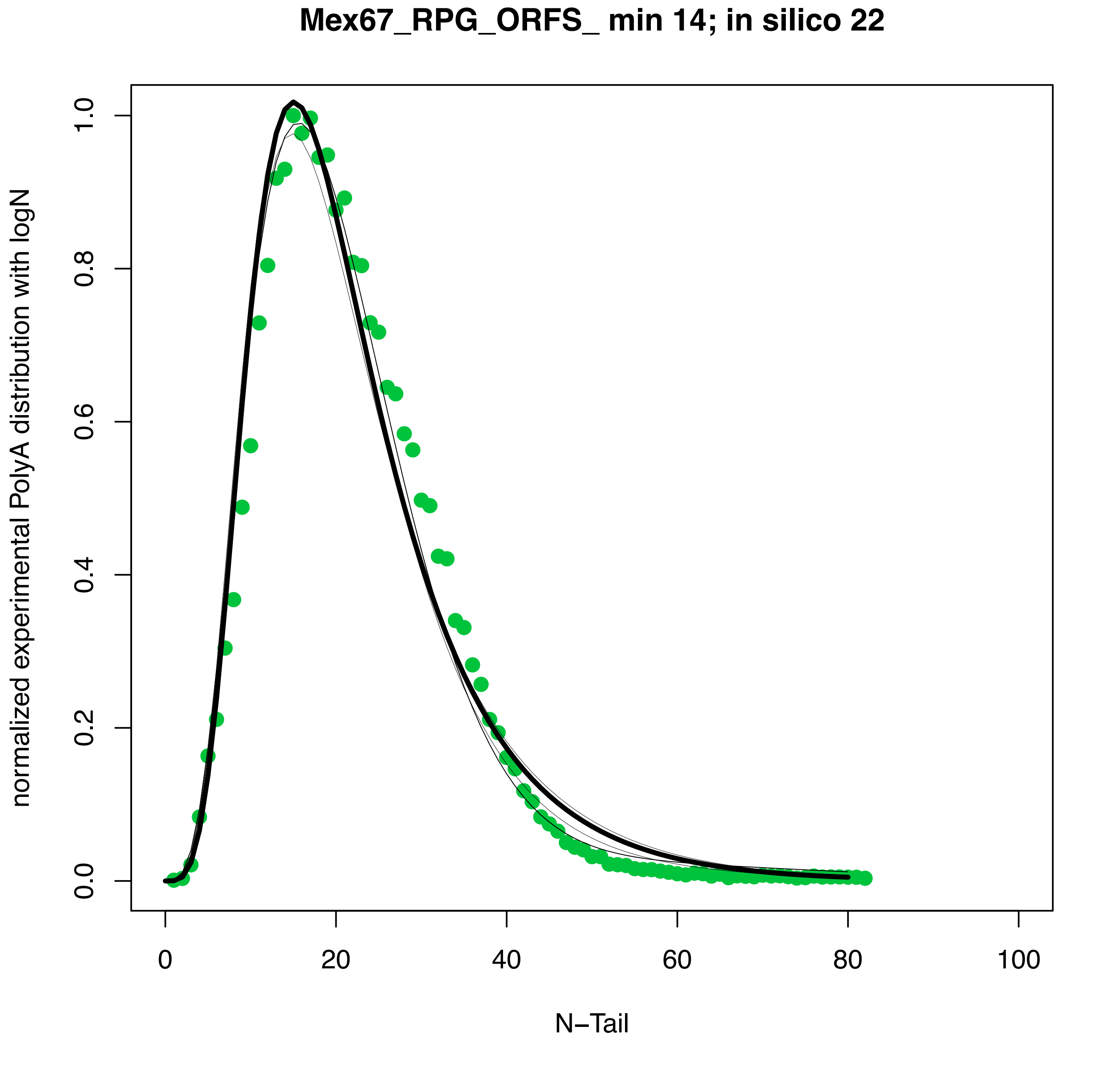

Mex67_RPG_ORFS_ min 14; in silico 22
normalized experimental PolyA distribution with logN
N−Tail
0.0
0.2
0.4
0.6
0.8
1.0
0
20
40
60
80
100

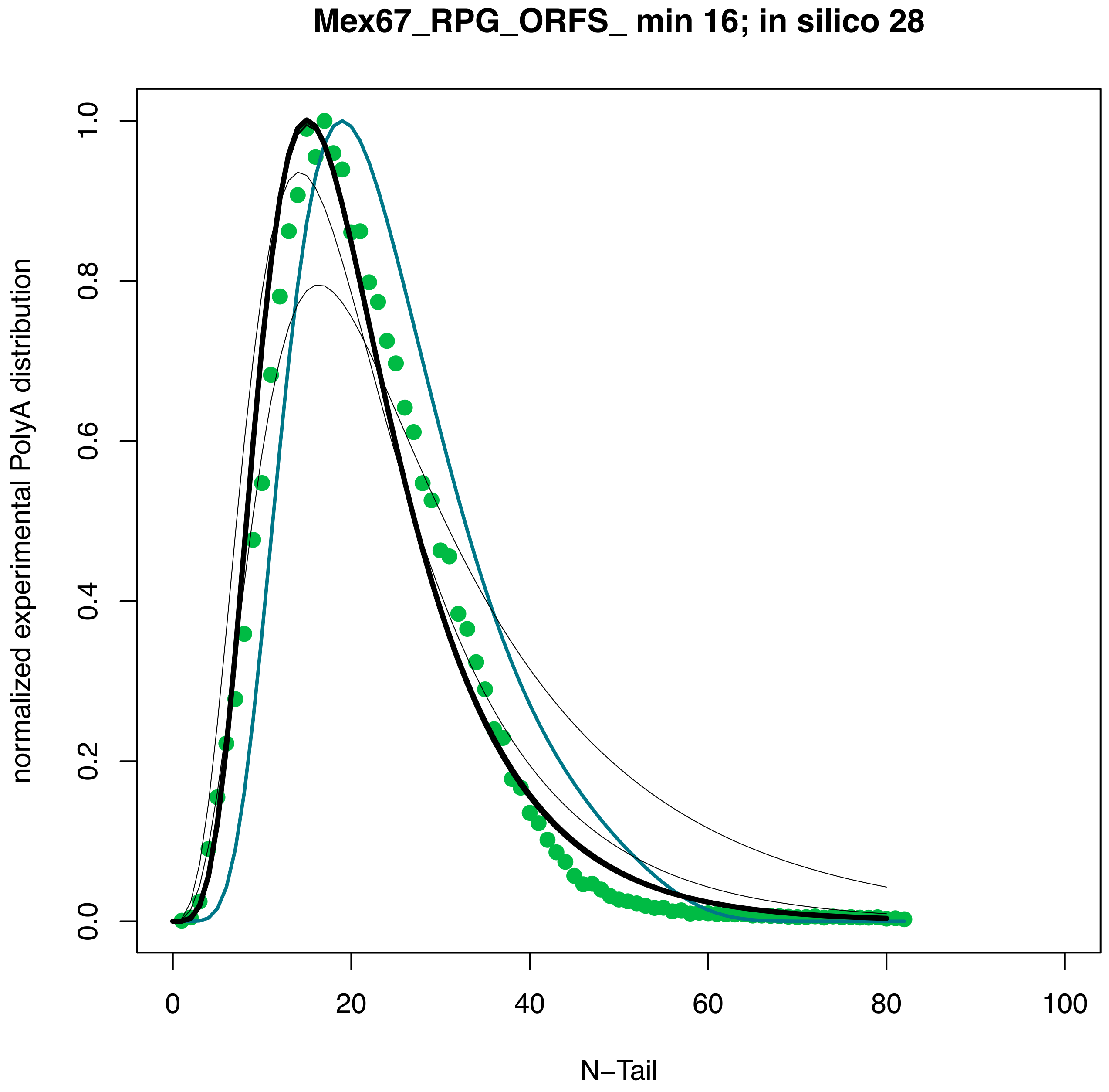

Mex67_RPG_ORFS_ min 16; in silico 28
normalized experimental PolyA distribution
N−Tail
0.0
0.2
0.4
0.6
0.8
1.0
0
20
40
60
80
100

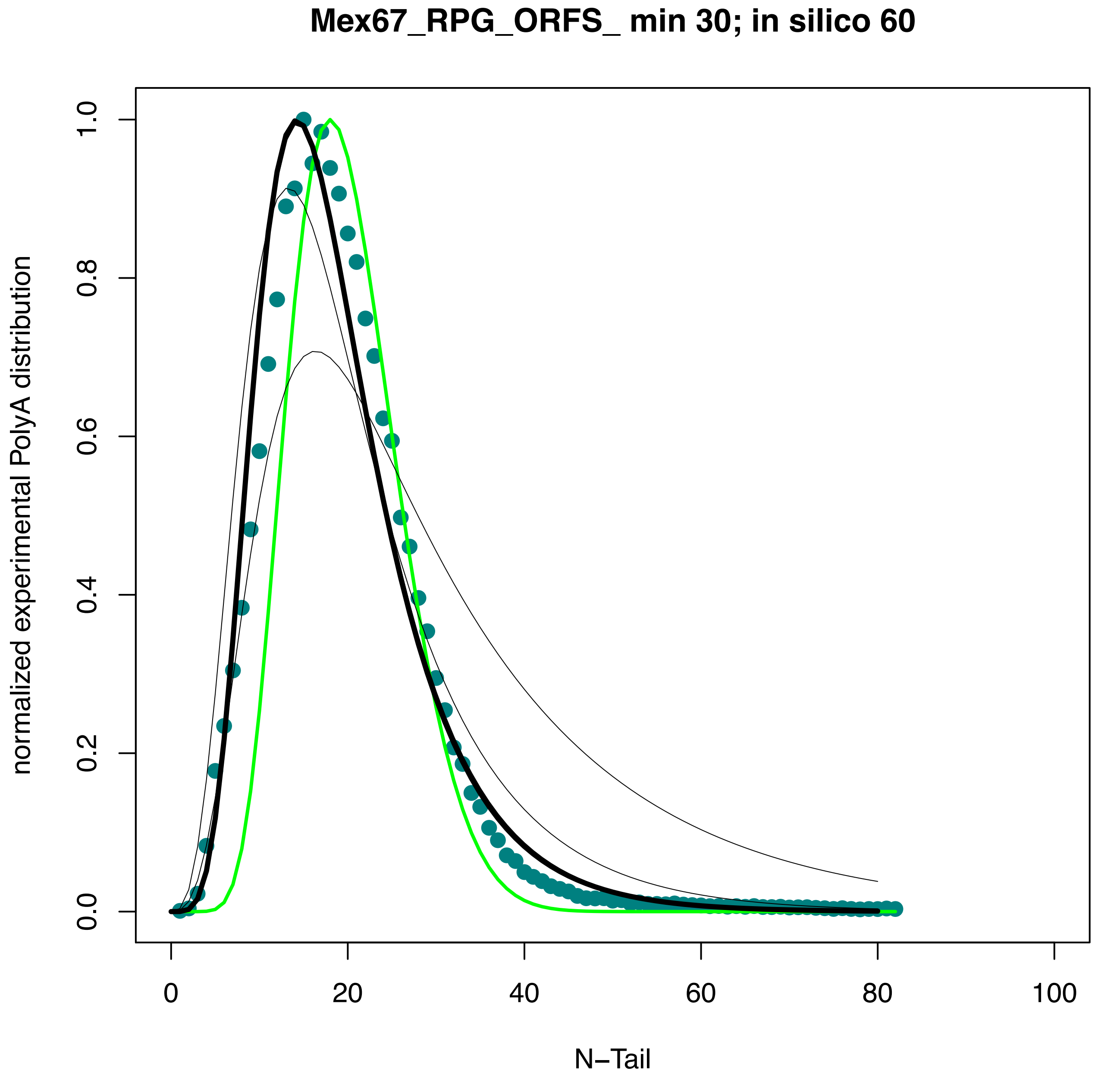

Mex67_RPG_ORFS_ min 30; in silico 60
normalized experimental PolyA distribution
0.0
0.2
0.4
0.6
0.8
1.0
0
20
40
60
80
100
N−Tail

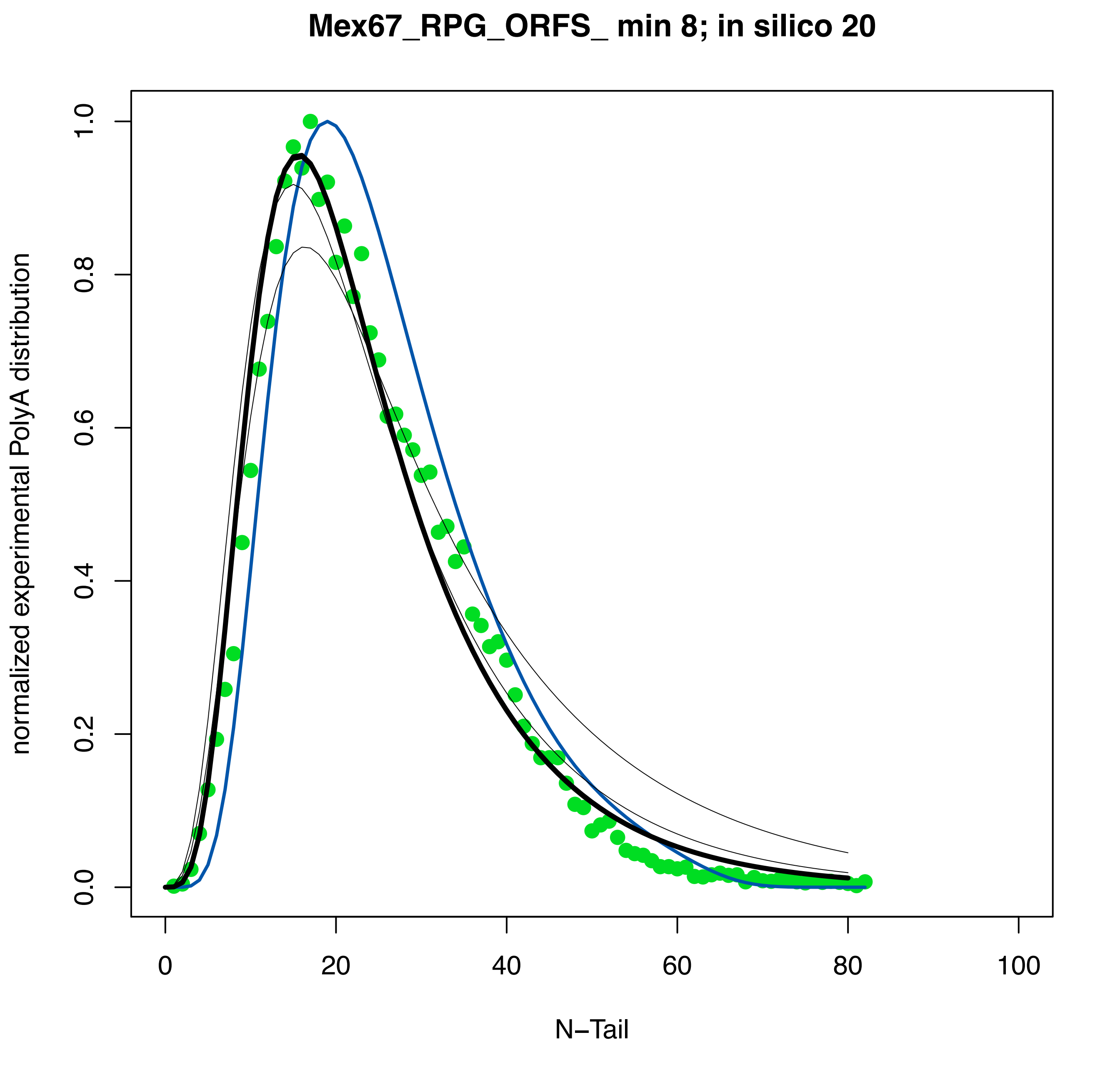

Mex67_RPG_ORFS_ min 8; in silico 20
normalized experimental PolyA distribution
N−Tail
0.0
0.2
0.4
0.6
0.8
1.0
0
20
40
60
80
100

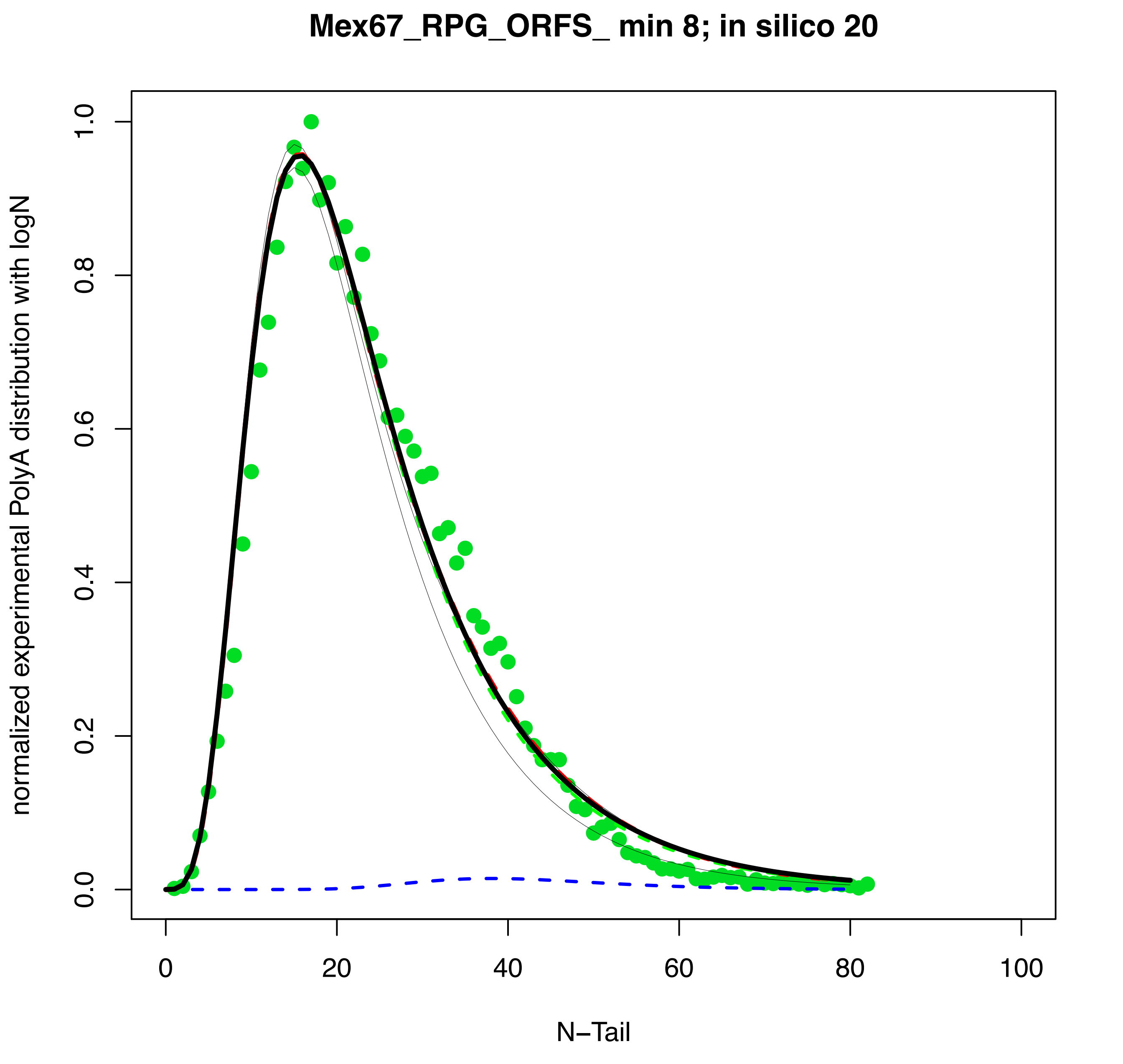

Mex67_RPG_ORFS_ min 8; in silico 20
normalized experimental PolyA distribution with logN
N−Tail
0.0
0.2
0.4
0.6
0.8
1.0
0
20
40
60
80
100

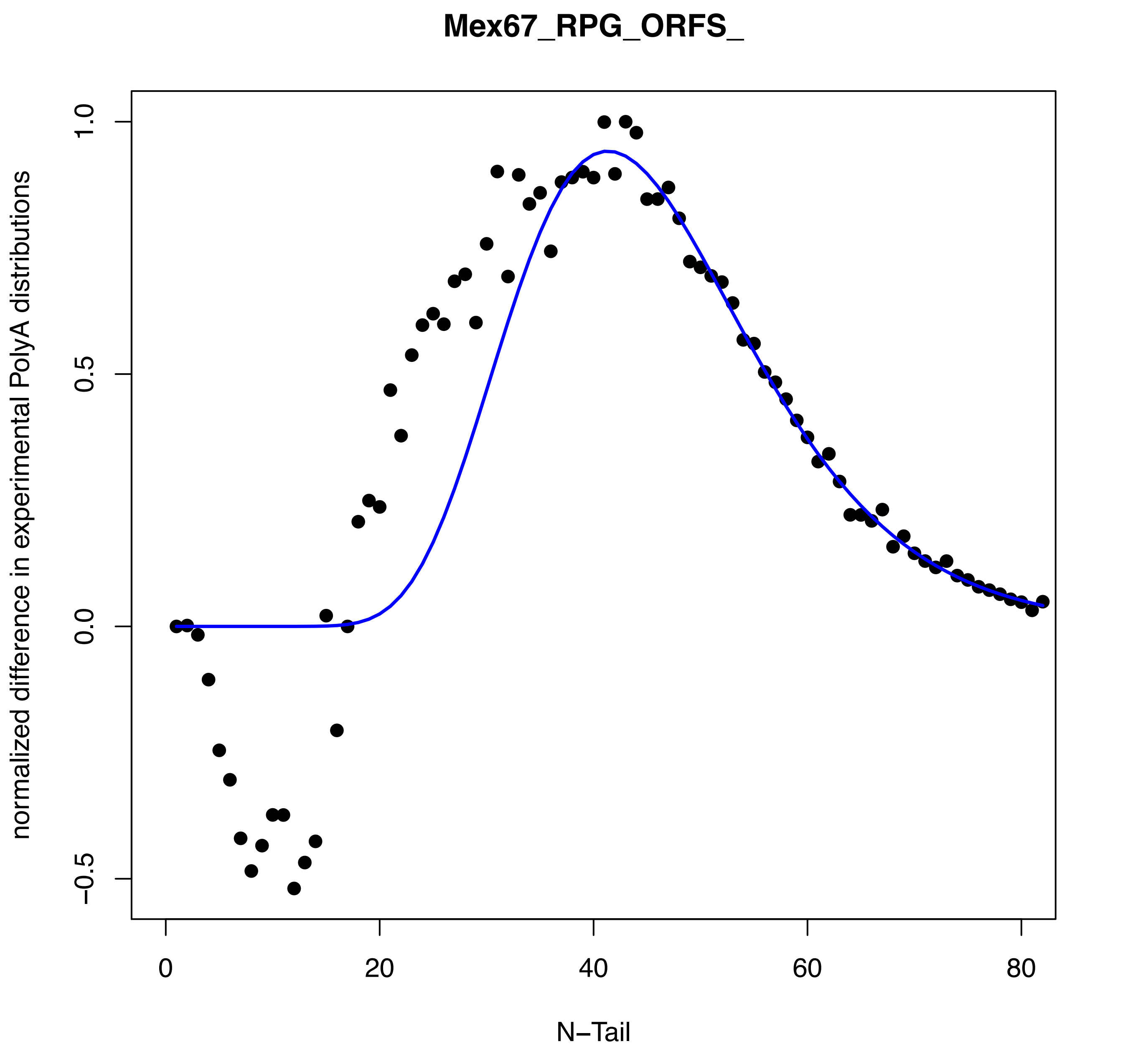

Mex67_RPG_ORFS_
normalized difference in experimental PolyA distributions
N−Tail
1.0
0.5
0.0
−0.5
0
20
40
60
80

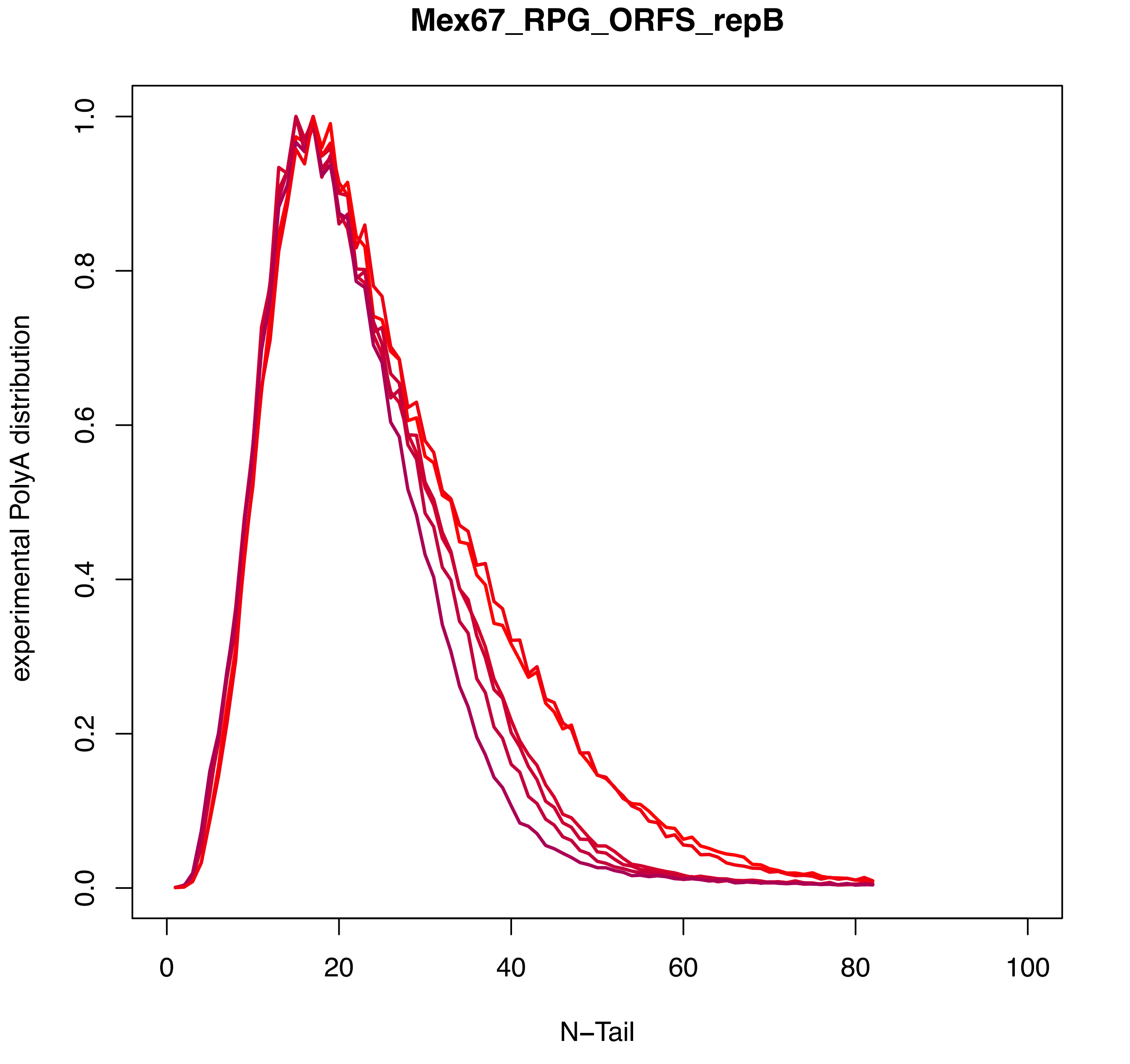

Mex67_RPG_ORFS_repB
experimental PolyA distribution
N−Tail
0.0
0.2
0.4
0.6
0.8
1.0
0
20
40
60
80
100

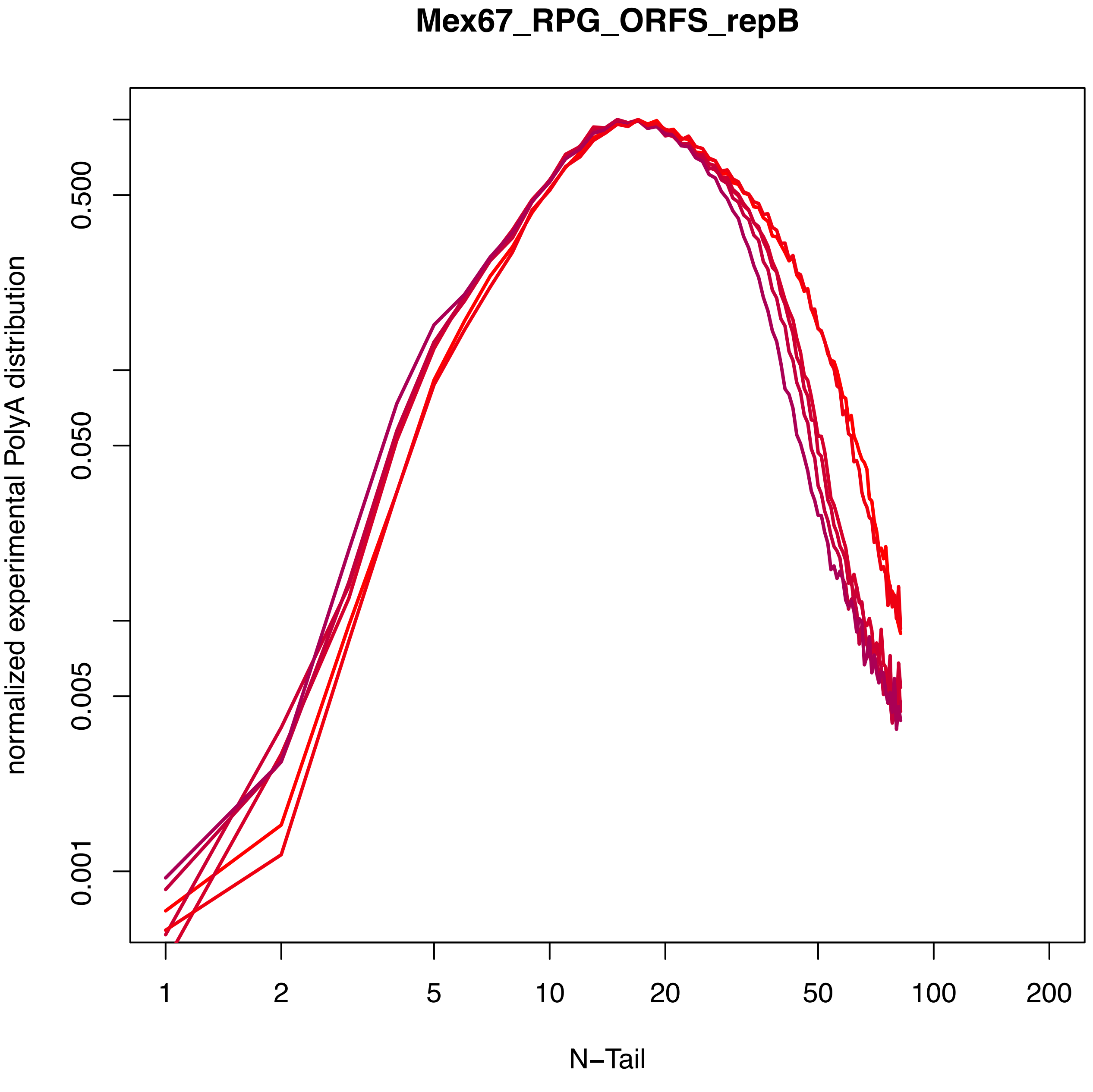
Mex67_RPG_ORFS_repB
normalized experimental PolyA distribution
0.001
0.005
0.050
0.500
N−Tail
1
2
5
10
20
50
100
200

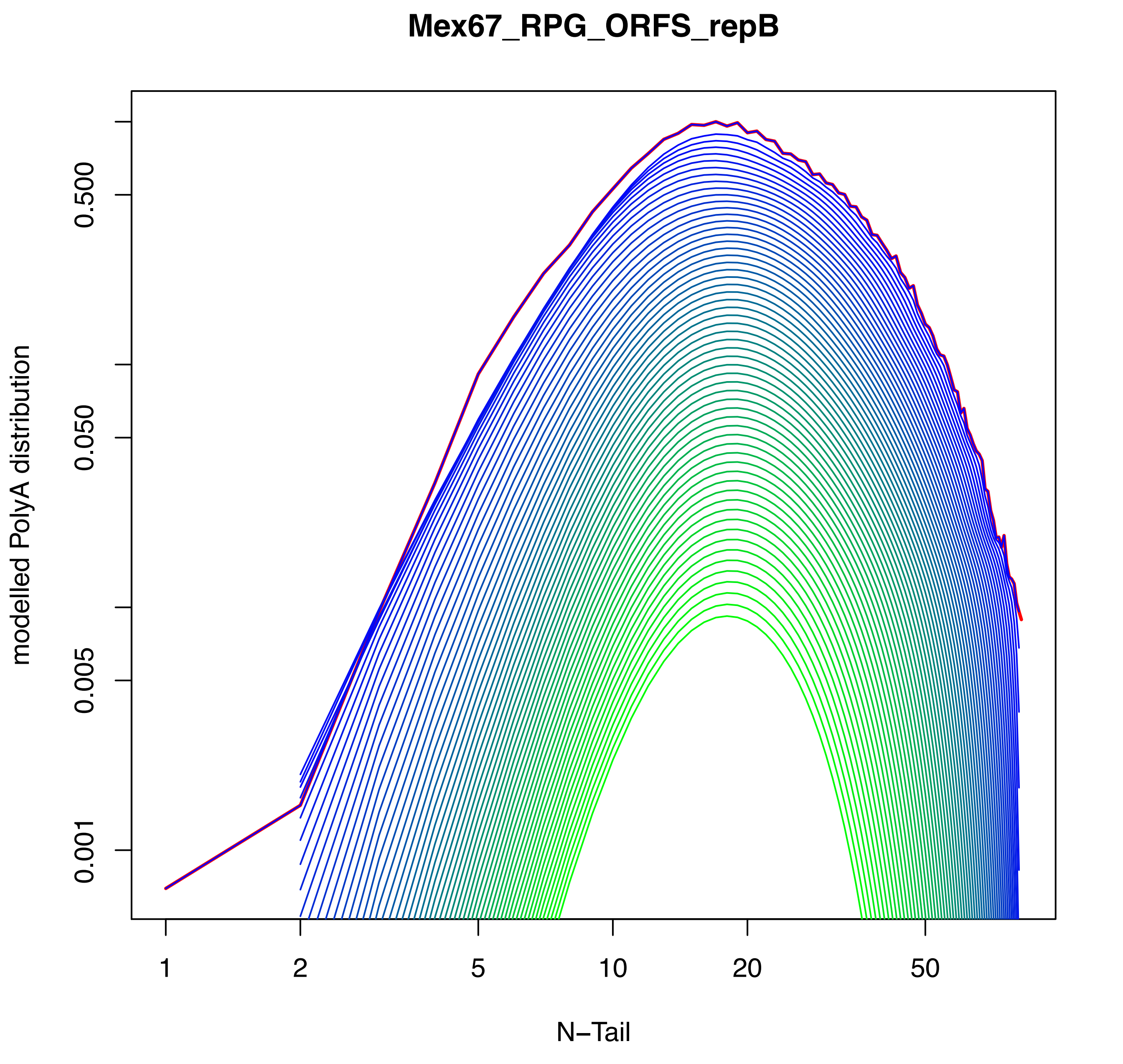

Mex67_RPG_ORFS_repB
modelled PolyA distribution
0.500
0.050
0.005
0.001
1
2
5
10
20
50
N−Tail

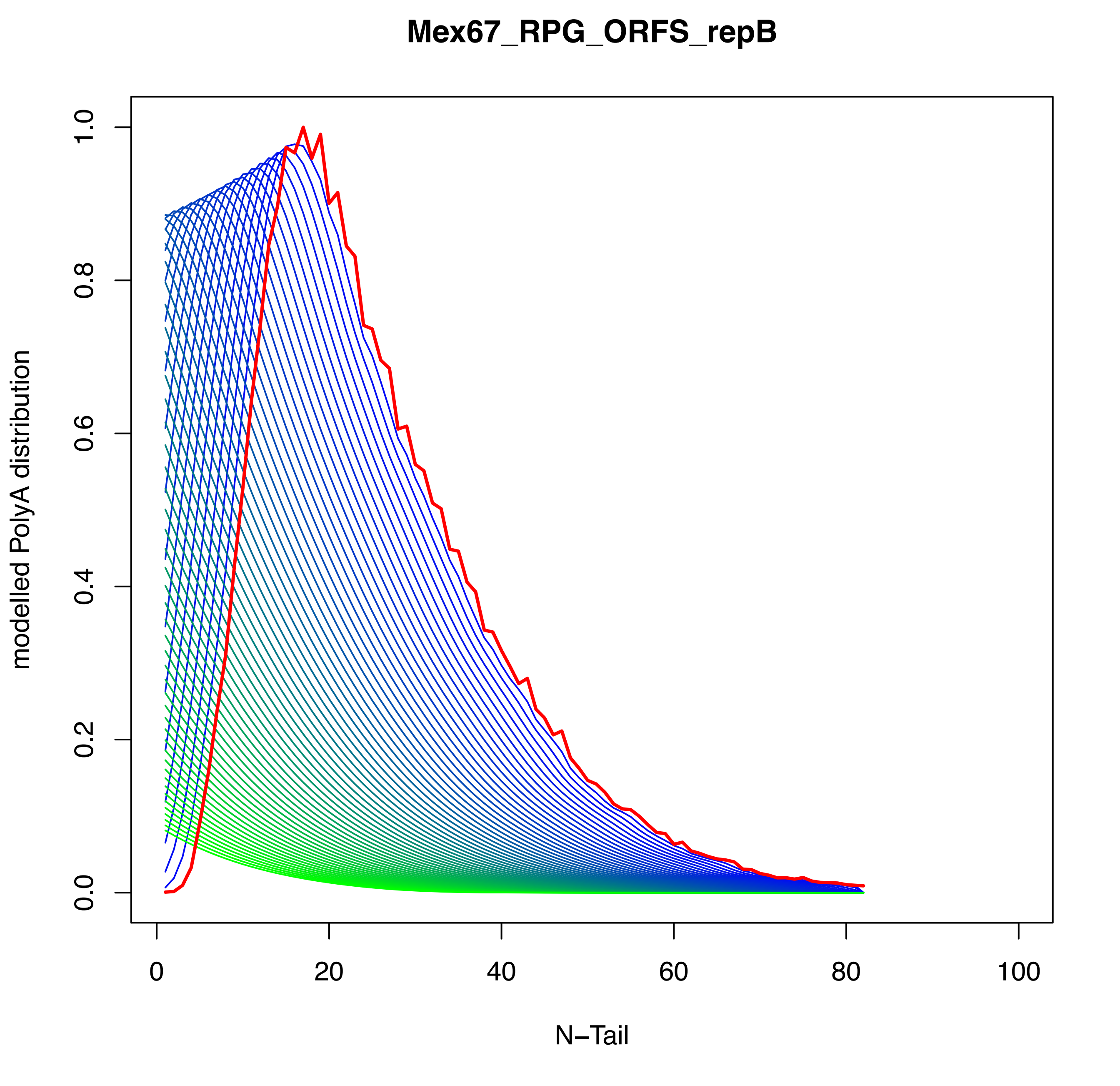
Mex67_RPG_ORFS_repB
modelled PolyA distribution
N−Tail
0.0
0.2
0.4
0.6
0.8
1.0
0
20
40
60
80
100

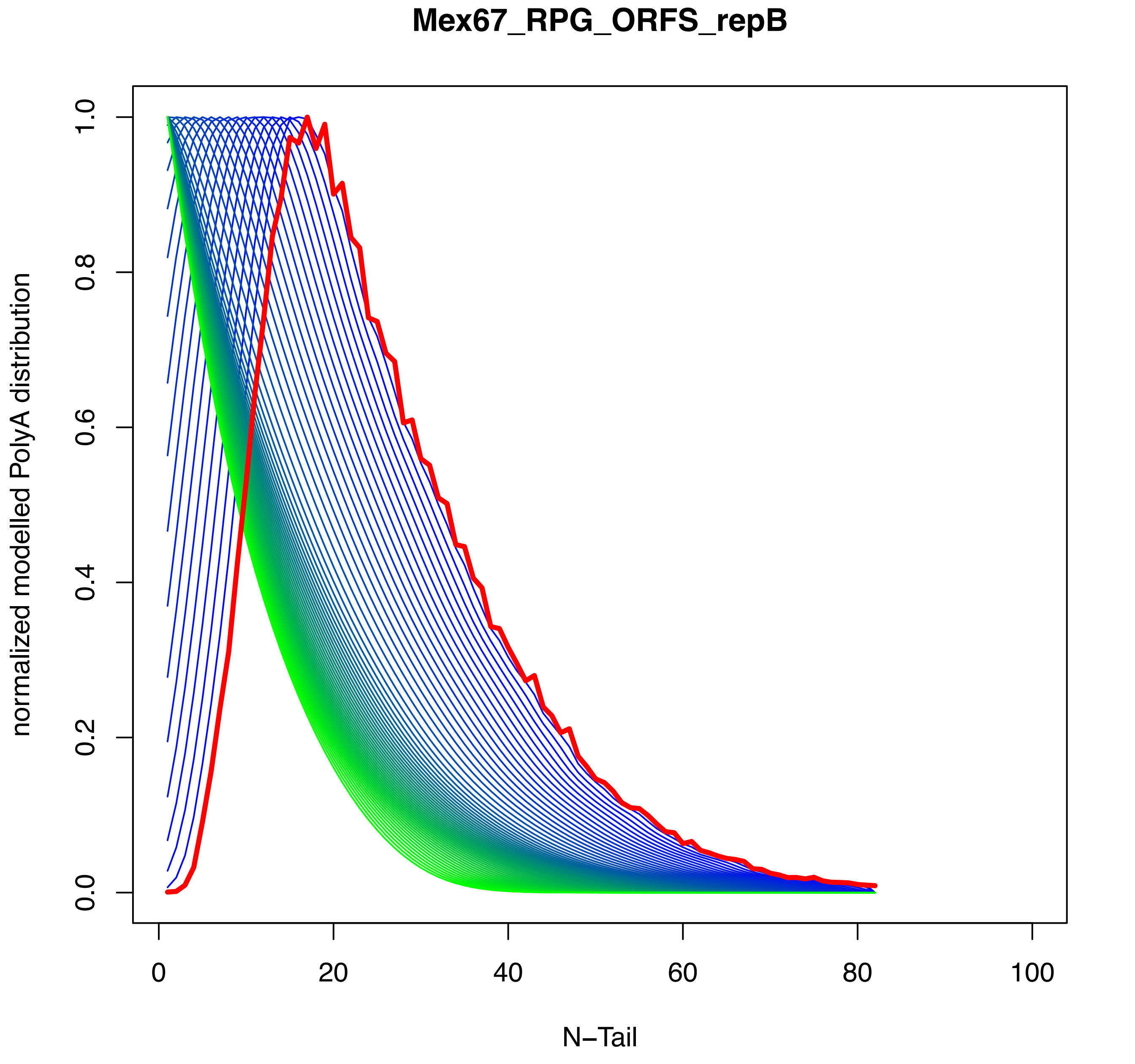
Mex67_RPG_ORFS_repB
normalized modelled PolyA distribution
N−Tail
0.0
0.2
0.4
0.6
0.8
1.0
0
20
40
60
80
100

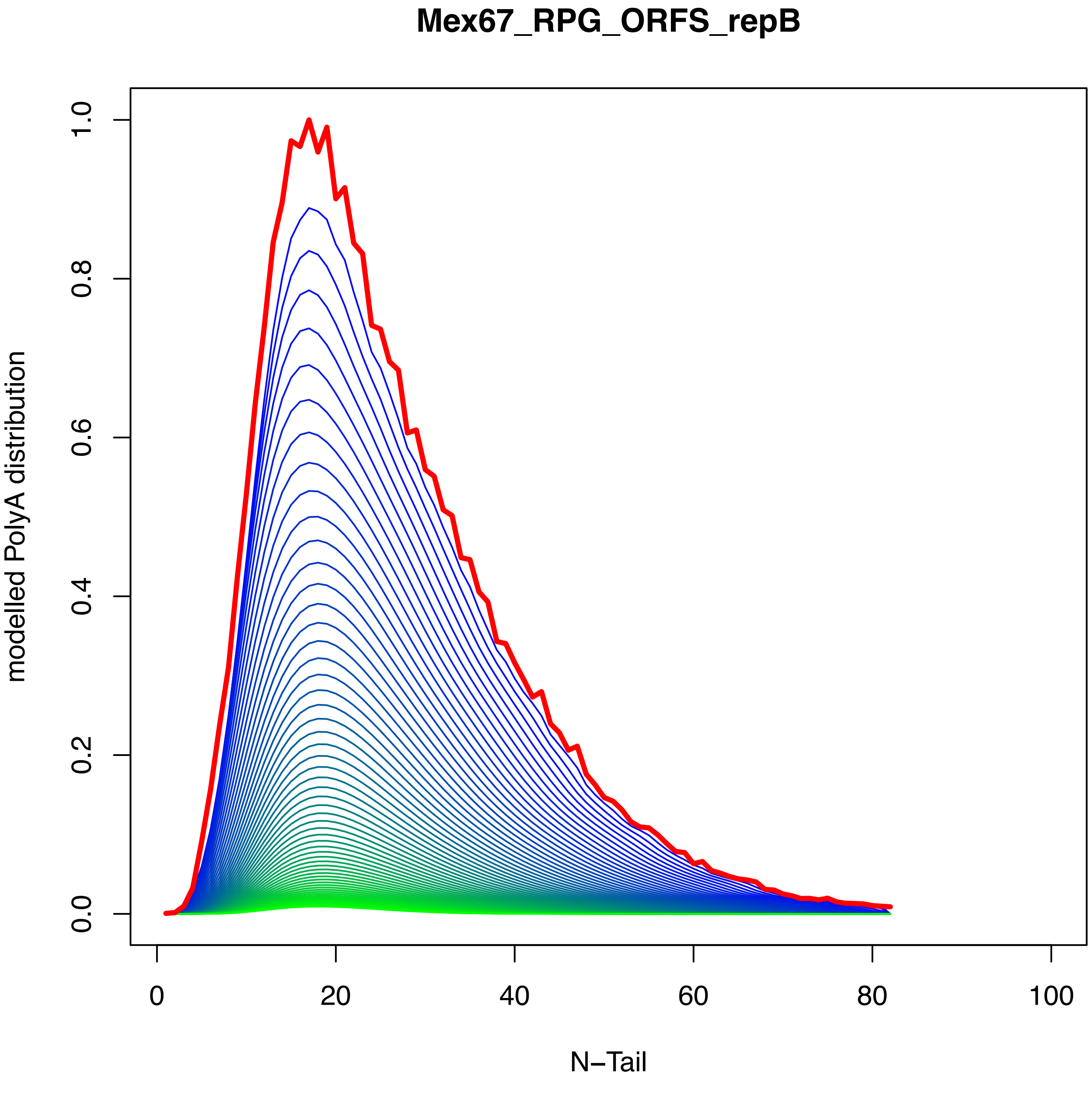

Mex67_RPG_ORFS_repB
modelled PolyA distribution
N−Tail
0.0
0.2
0.4
0.6
0.8
1.0
0
20
40
60
80
100

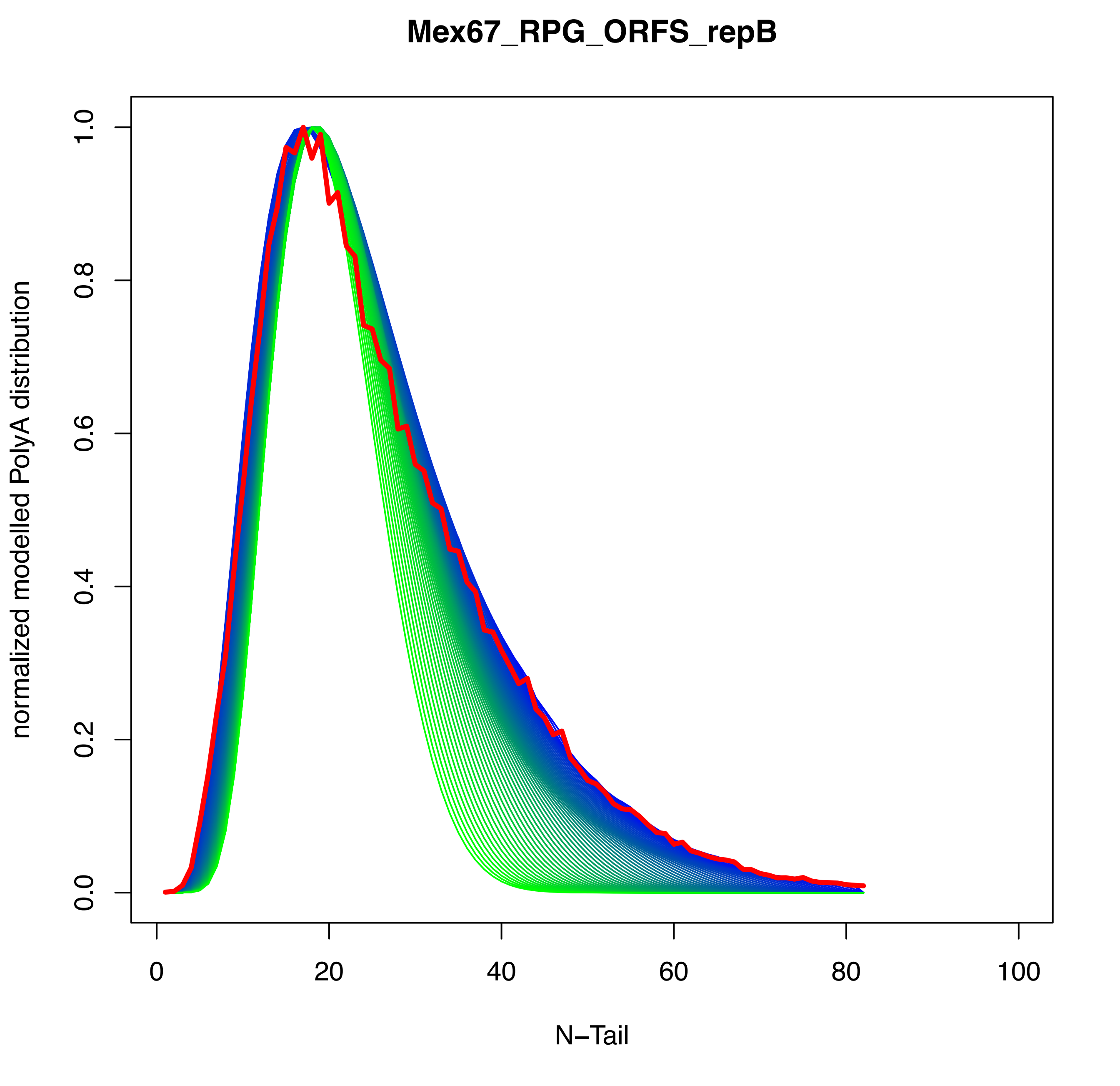
Mex67_RPG_ORFS_repB
normalized modelled PolyA distribution
N−Tail
0.0
0.2
0.4
0.6
0.8
1.0
0
20
40
60
80
100

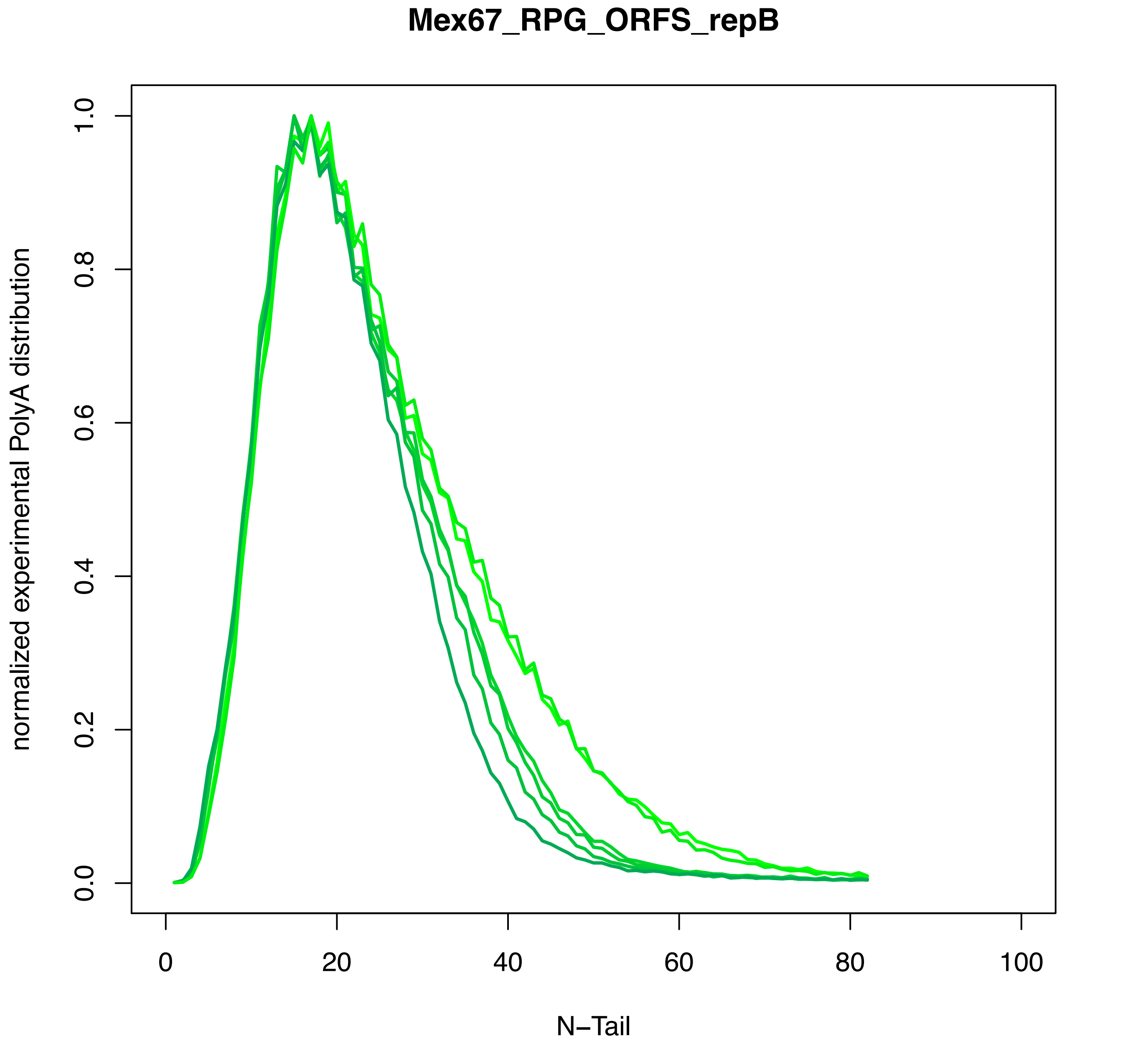

Mex67_RPG_ORFS_repB
normalized experimental PolyA distribution
N−Tail
0.0
0.2
0.4
0.6
0.8
1.0
0
20
40
60
80
100

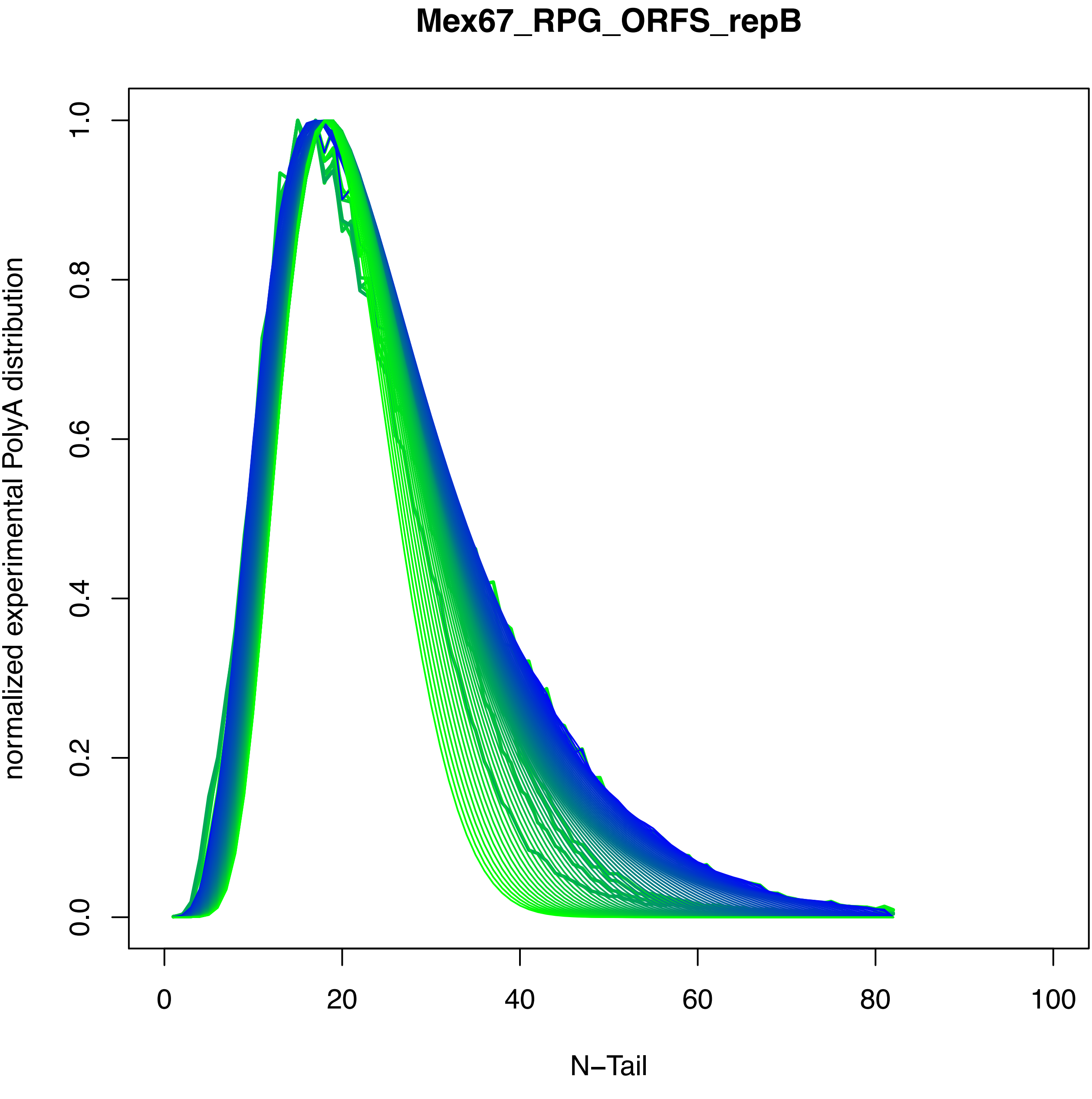

Mex67_RPG_ORFS_repB
normalized experimental PolyA distribution
N−Tail
0.0
0.2
0.4
0.6
0.8
1.0
0
20
40
60
80
100

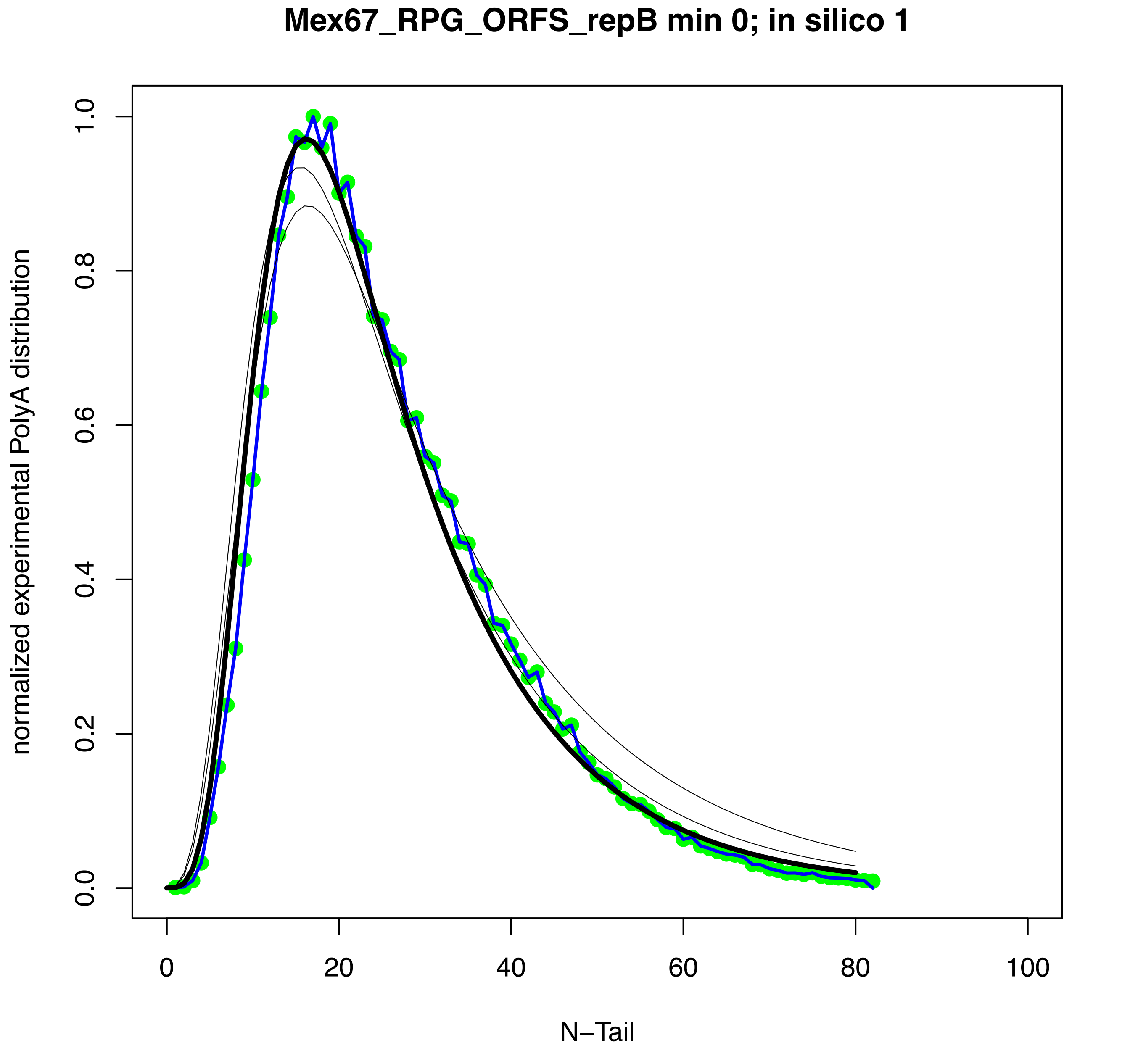

Mex67_RPG_ORFS_repB min 0; in silico 1
normalized experimental PolyA distribution
N−Tail
0.0
0.2
0.4
0.6
0.8
1.0
0
20
40
60
80
100

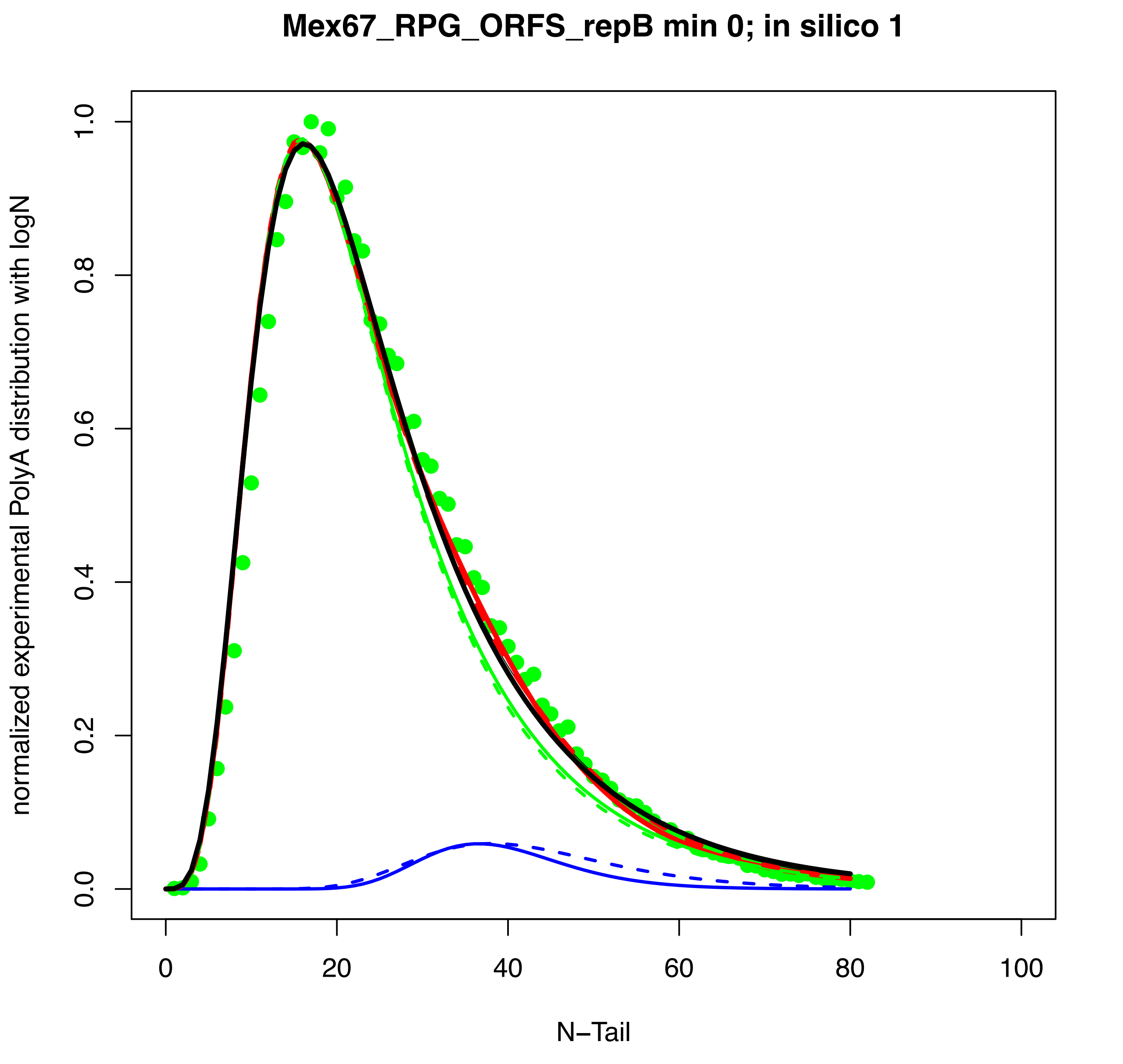

Mex67_RPG_ORFS_repB min 0; in silico 1
normalized experimental PolyA distribution with logN
N−Tail
0.0
0.2
0.4
0.6
0.8
1.0
0
20
40
60
80
100

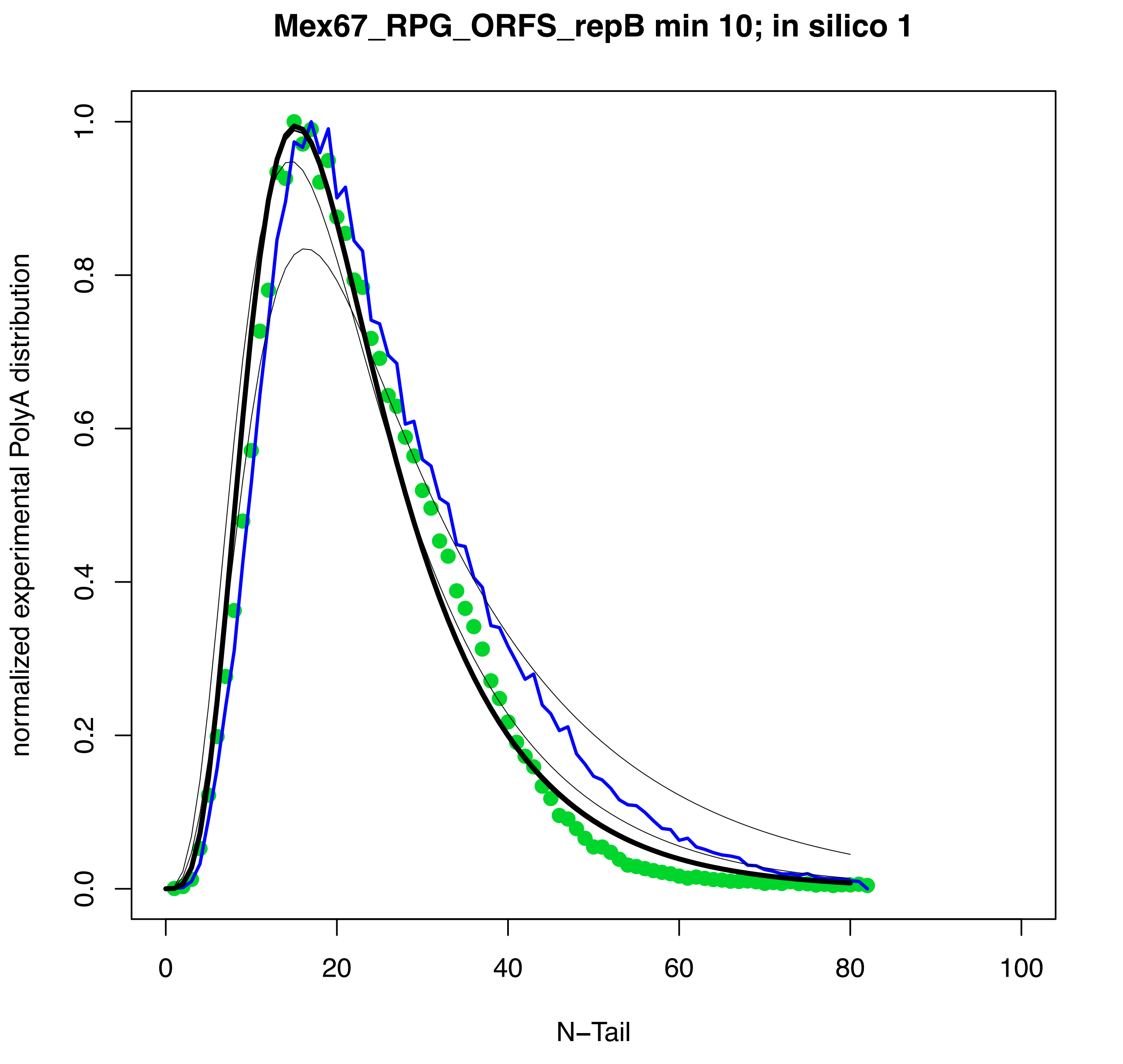

Mex67_RPG_ORFS_repB min 10; in silico 1
normalized experimental PolyA distribution
0.0
0.2
0.4
0.6
0.8
1.0
0
20
40
60
80
100
N−Tail

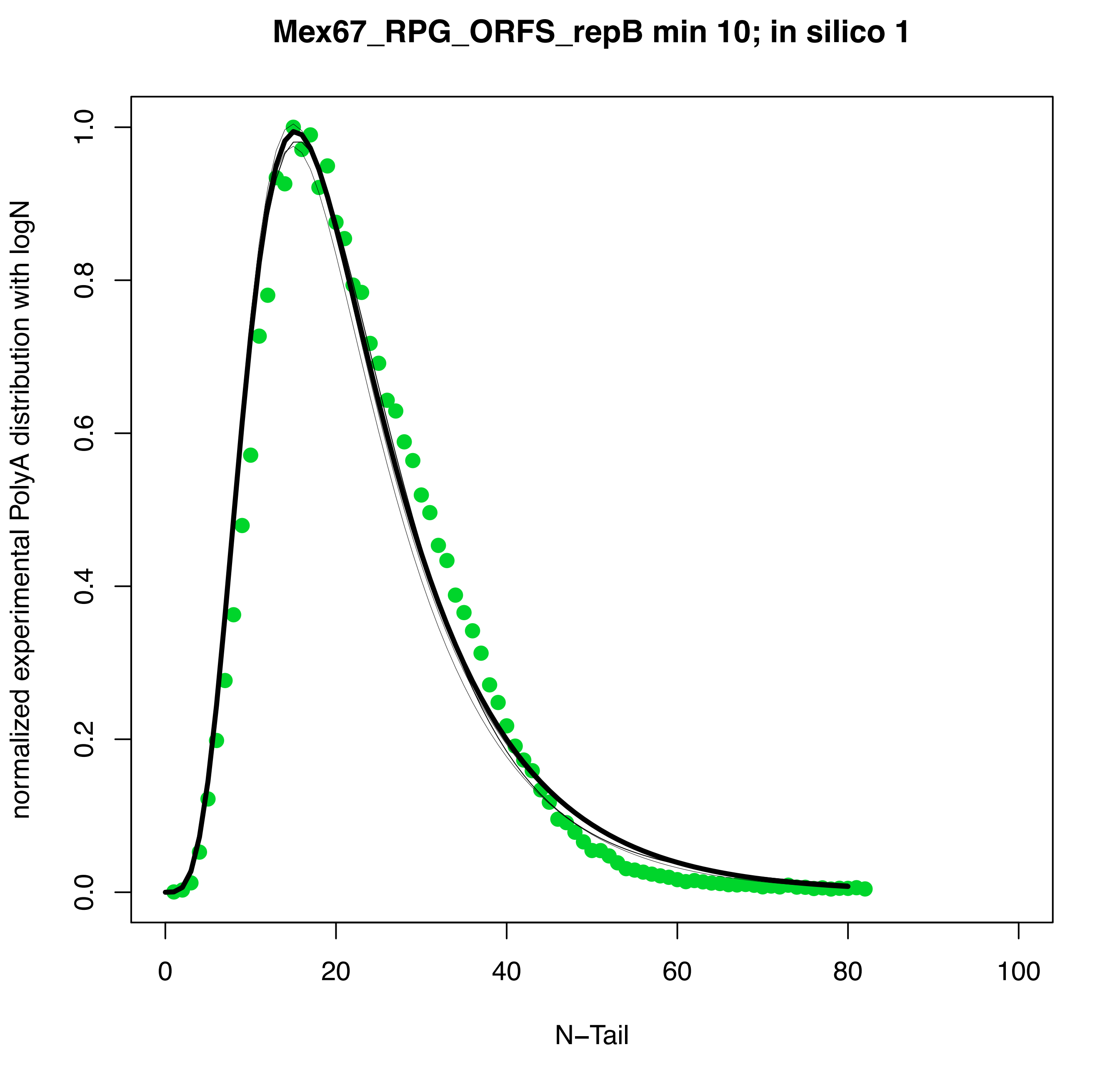

Mex67_RPG_ORFS_repB min 10; in silico 1
normalized experimental PolyA distribution with logN
N−Tail
0.0
0.2
0.4
0.6
0.8
1.0
0
20
40
60
80
100

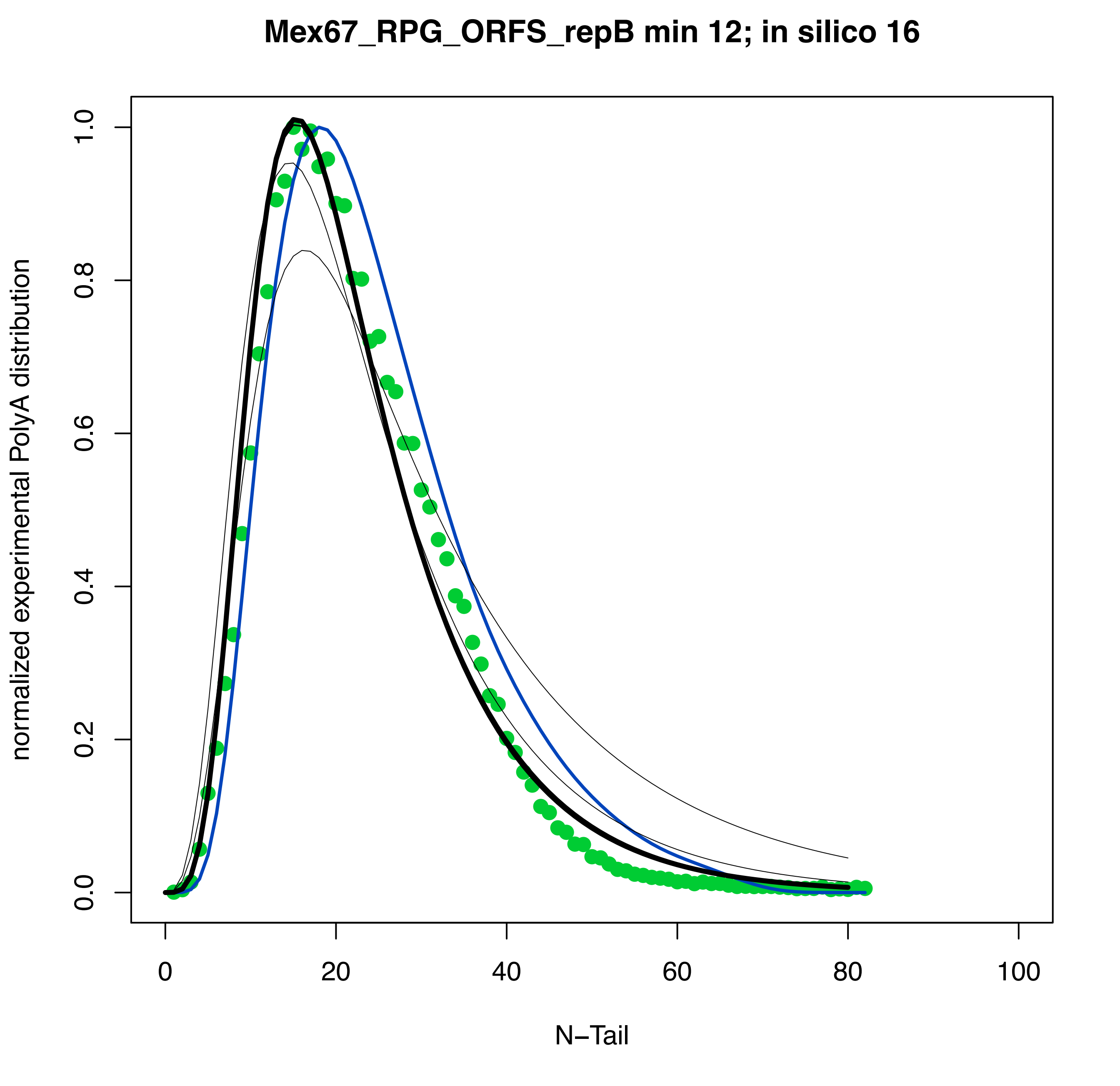

Mex67_RPG_ORFS_repB min 12; in silico 16
normalized experimental PolyA distribution
N−Tail
0.0
0.2
0.4
0.6
0.8
1.0
0
20
40
60
80
100

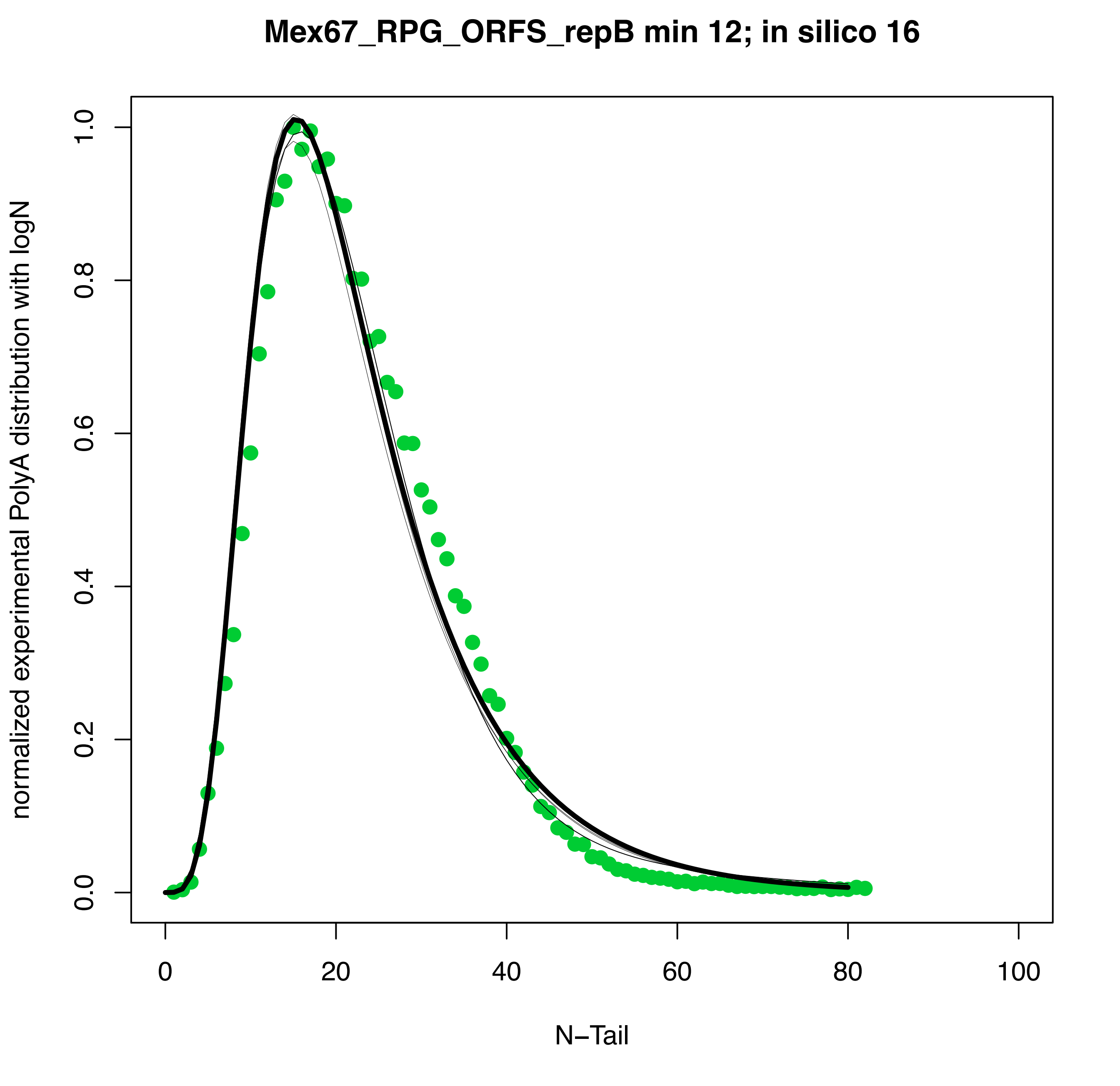

Mex67_RPG_ORFS_repB min 12; in silico 16
normalized experimental PolyA distribution with logN
N−Tail
0.0
0.2
0.4
0.6
0.8
1.0
0
20
40
60
80
100

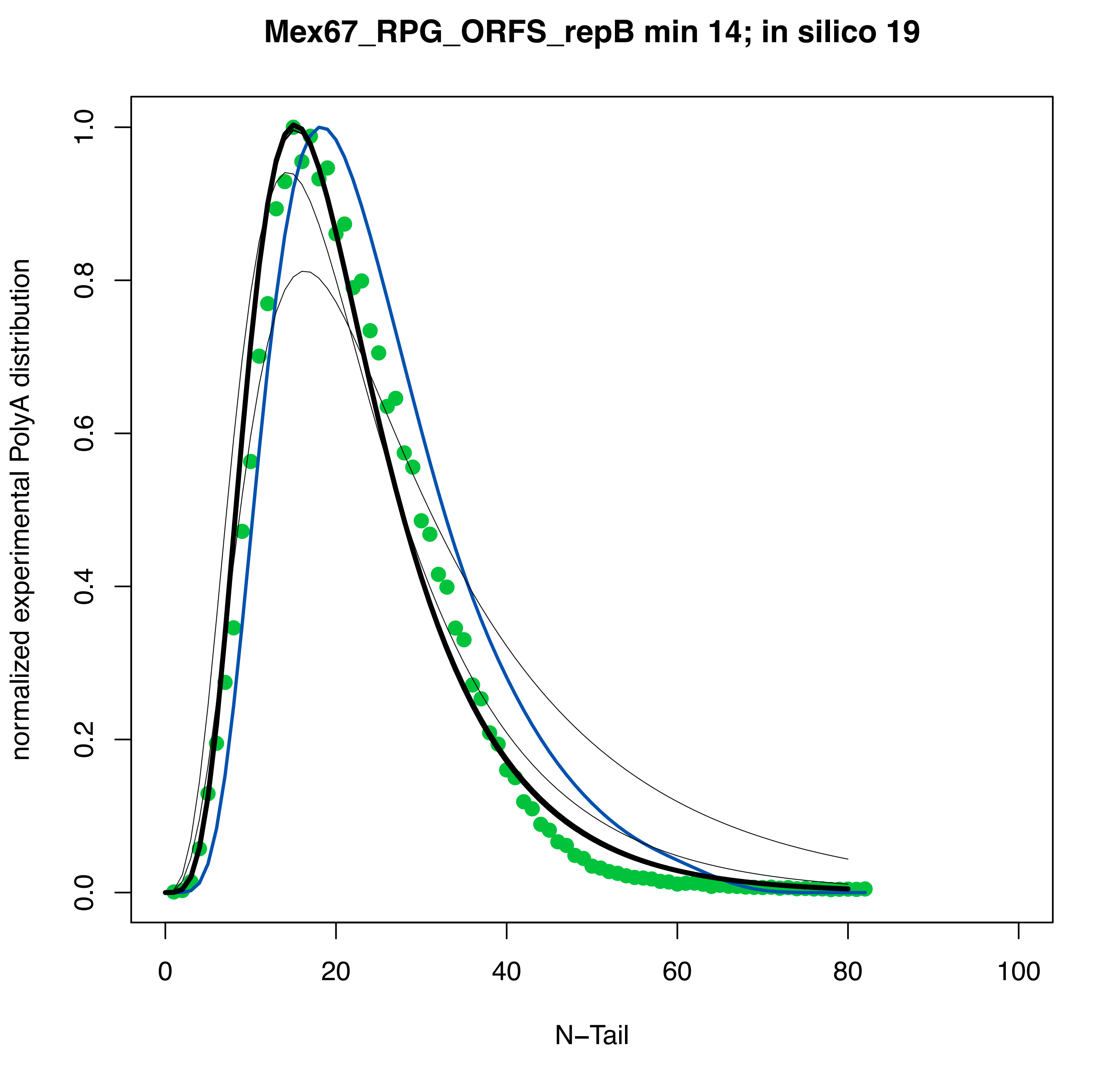

Mex67_RPG_ORFS_repB min 14; in silico 19
normalized experimental PolyA distribution
N−Tail
0.0
0.2
0.4
0.6
0.8
1.0
0
20
40
60
80
100

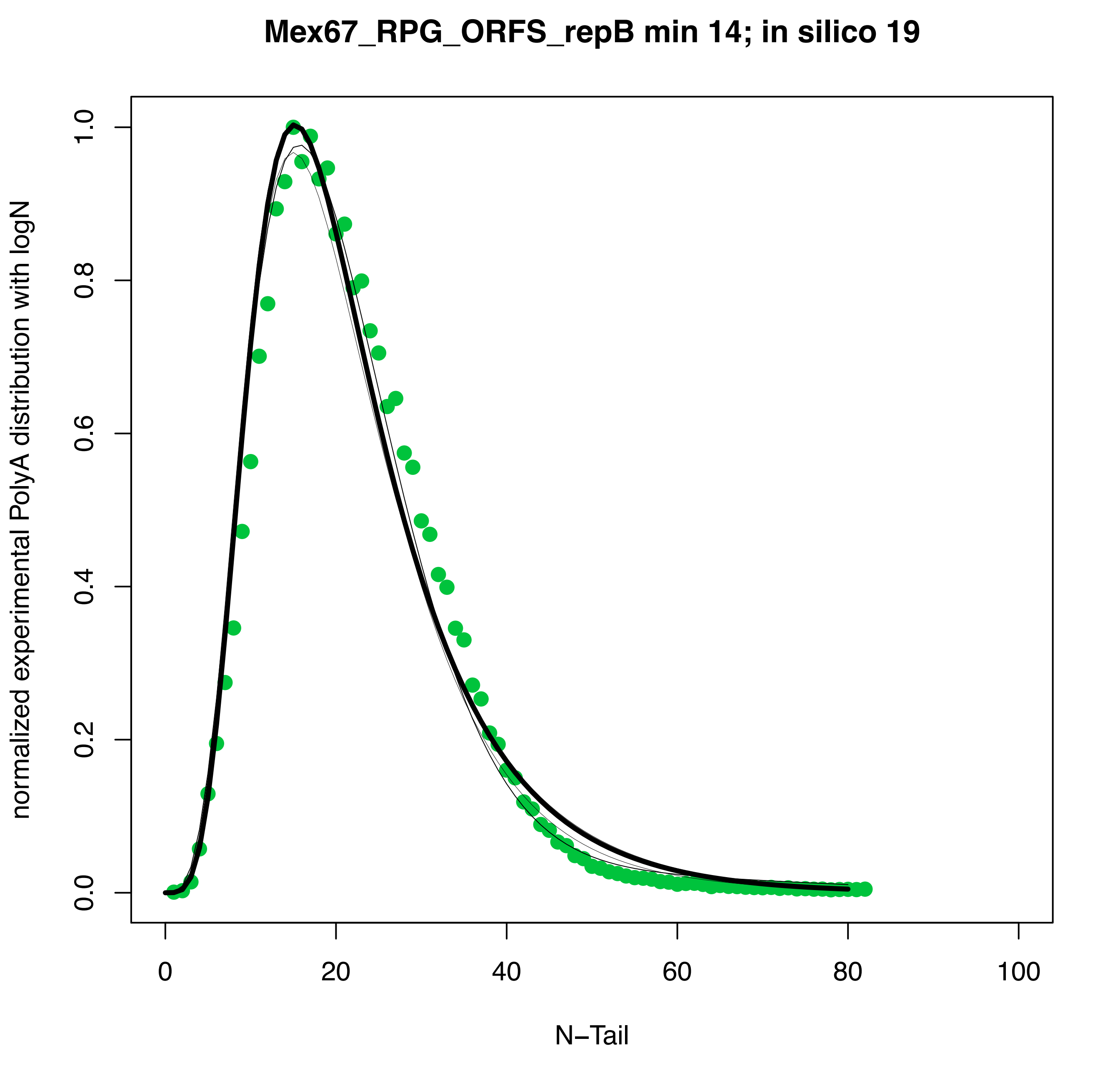

Mex67_RPG_ORFS_repB min 14; in silico 19
normalized experimental PolyA distribution with logN
N−Tail
0.0
0.2
0.4
0.6
0.8
1.0
0
20
40
60
80
100

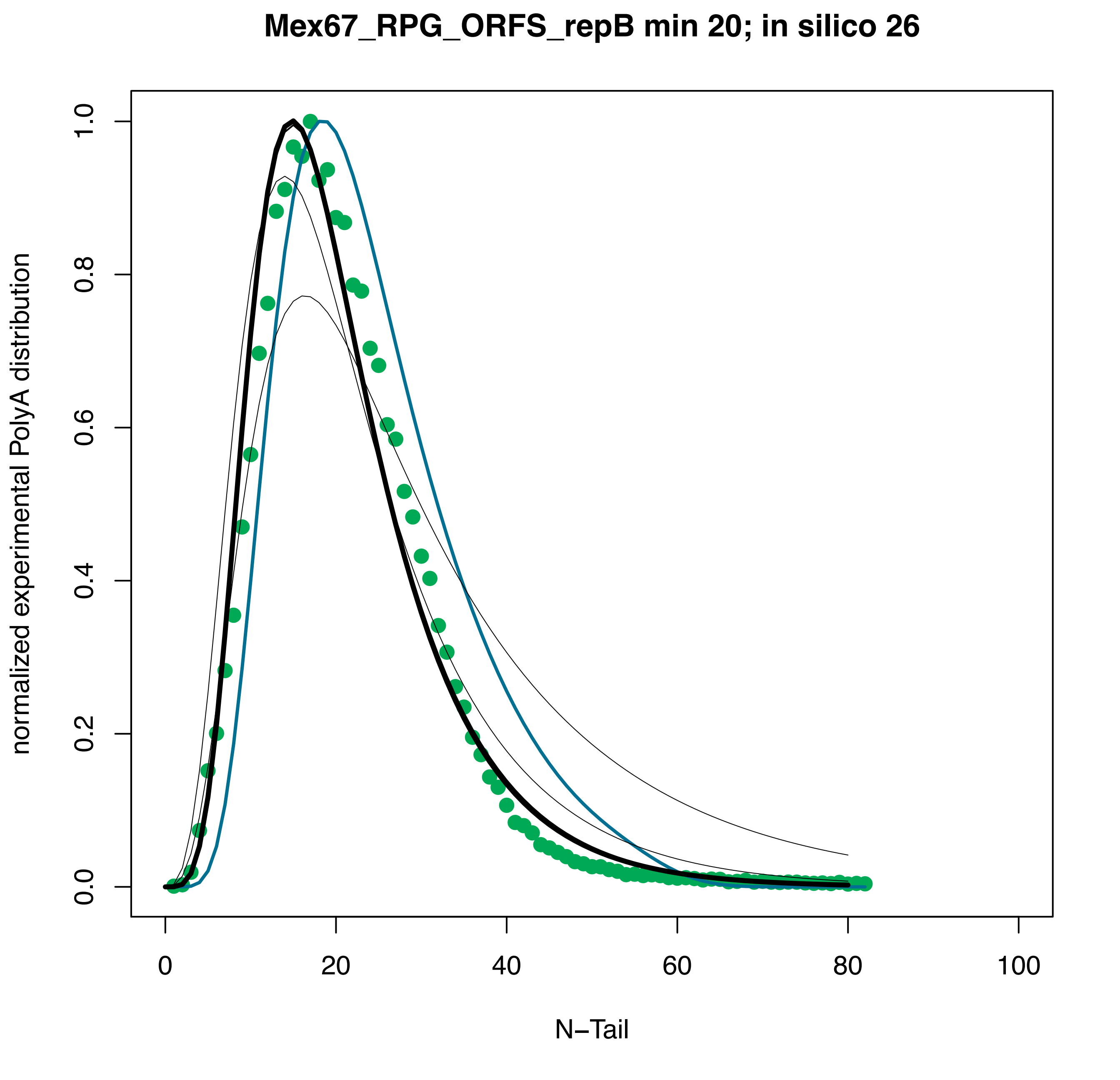

Mex67_RPG_ORFS_repB min 20; in silico 26
normalized experimental PolyA distribution
N−Tail
0.0
0.2
0.4
0.6
0.8
1.0
0
20
40
60
80
100

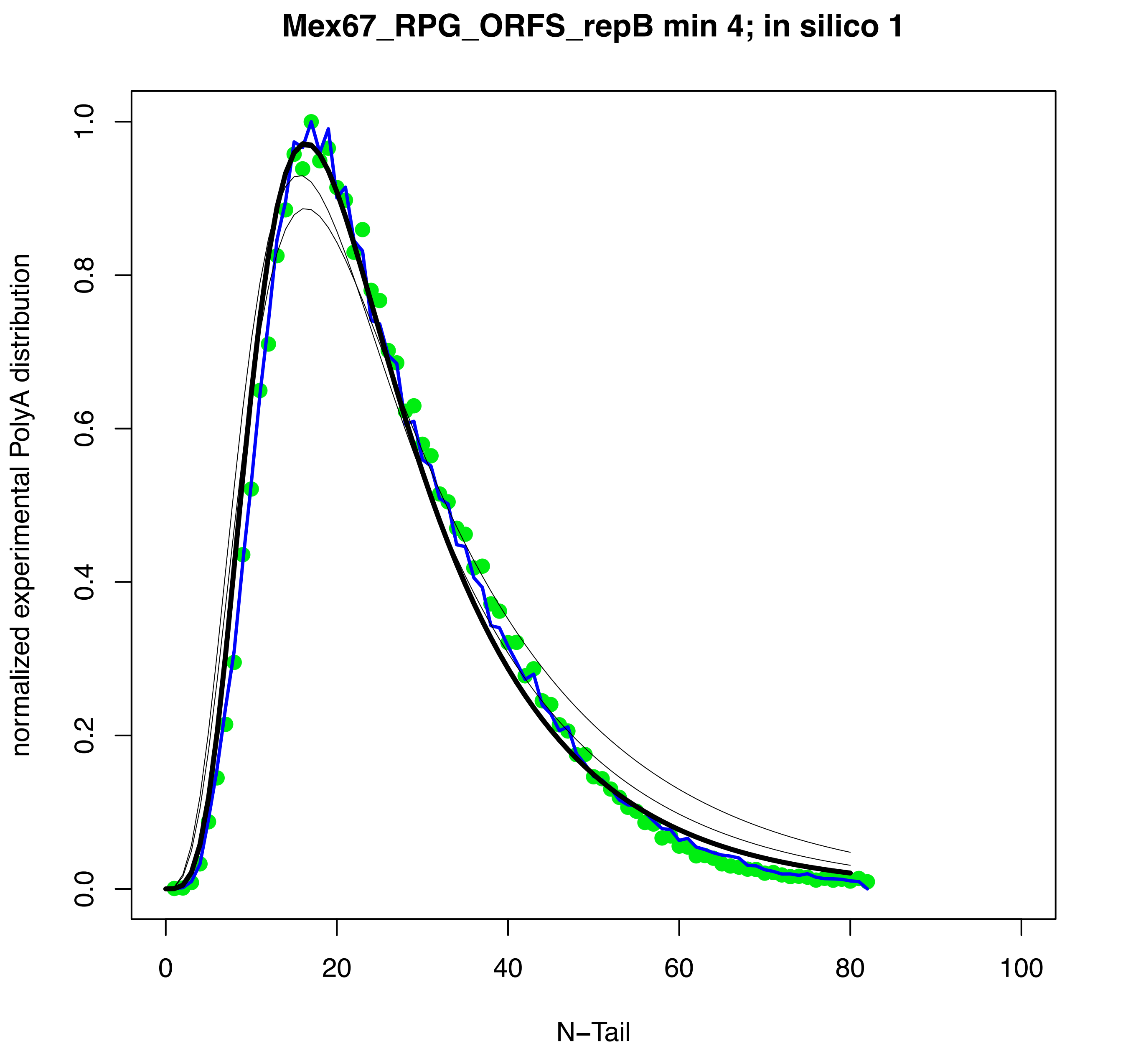

Mex67_RPG_ORFS_repB min 4; in silico 1
normalized experimental PolyA distribution
N−Tail
0.0
0.2
0.4
0.6
0.8
1.0
0
20
40
60
80
100

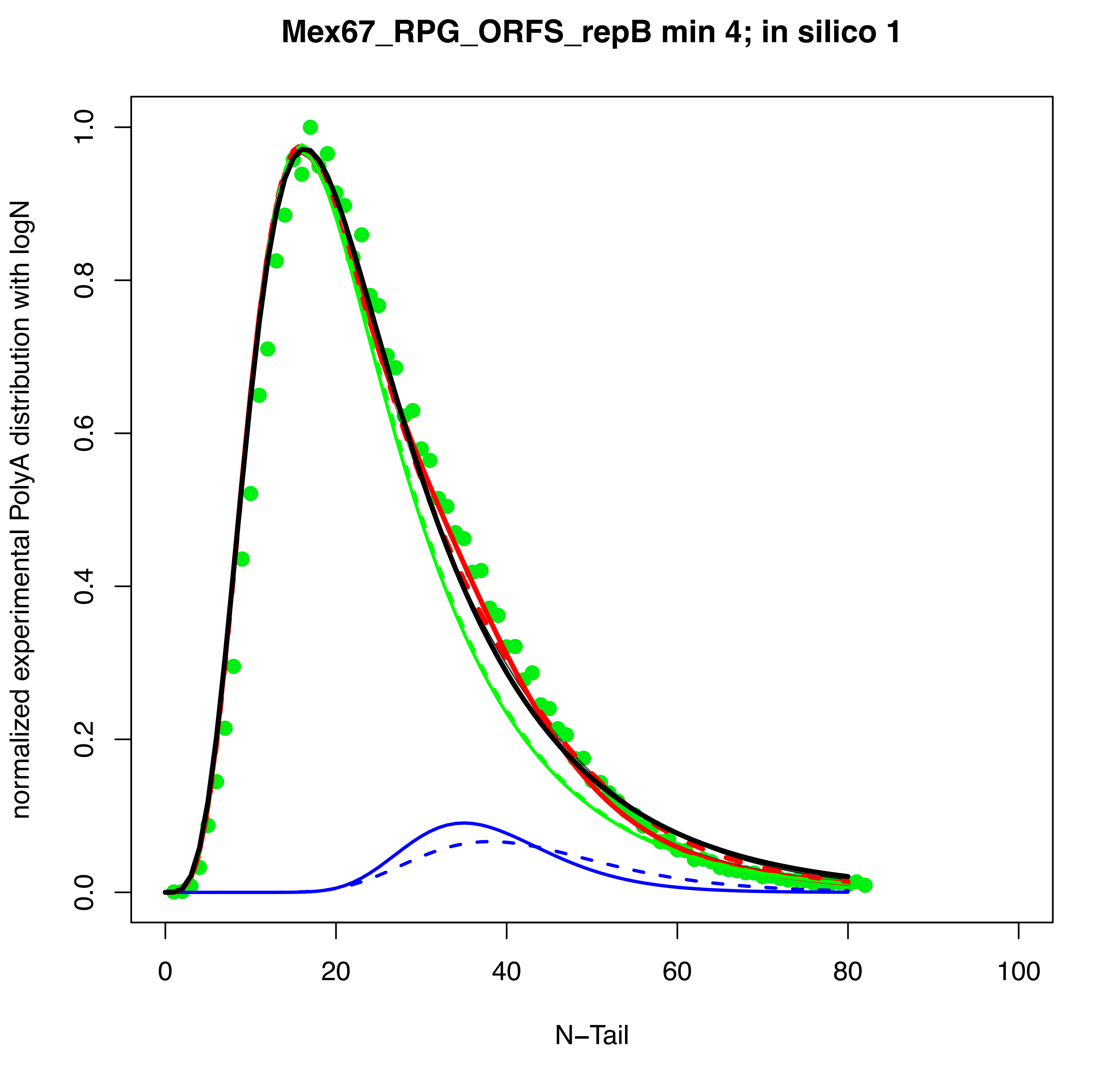

Mex67_RPG_ORFS_repB min 4; in silico 1
normalized experimental PolyA distribution with logN
N−Tail
0.0
0.2
0.4
0.6
0.8
1.0
0
20
40
60
80
100

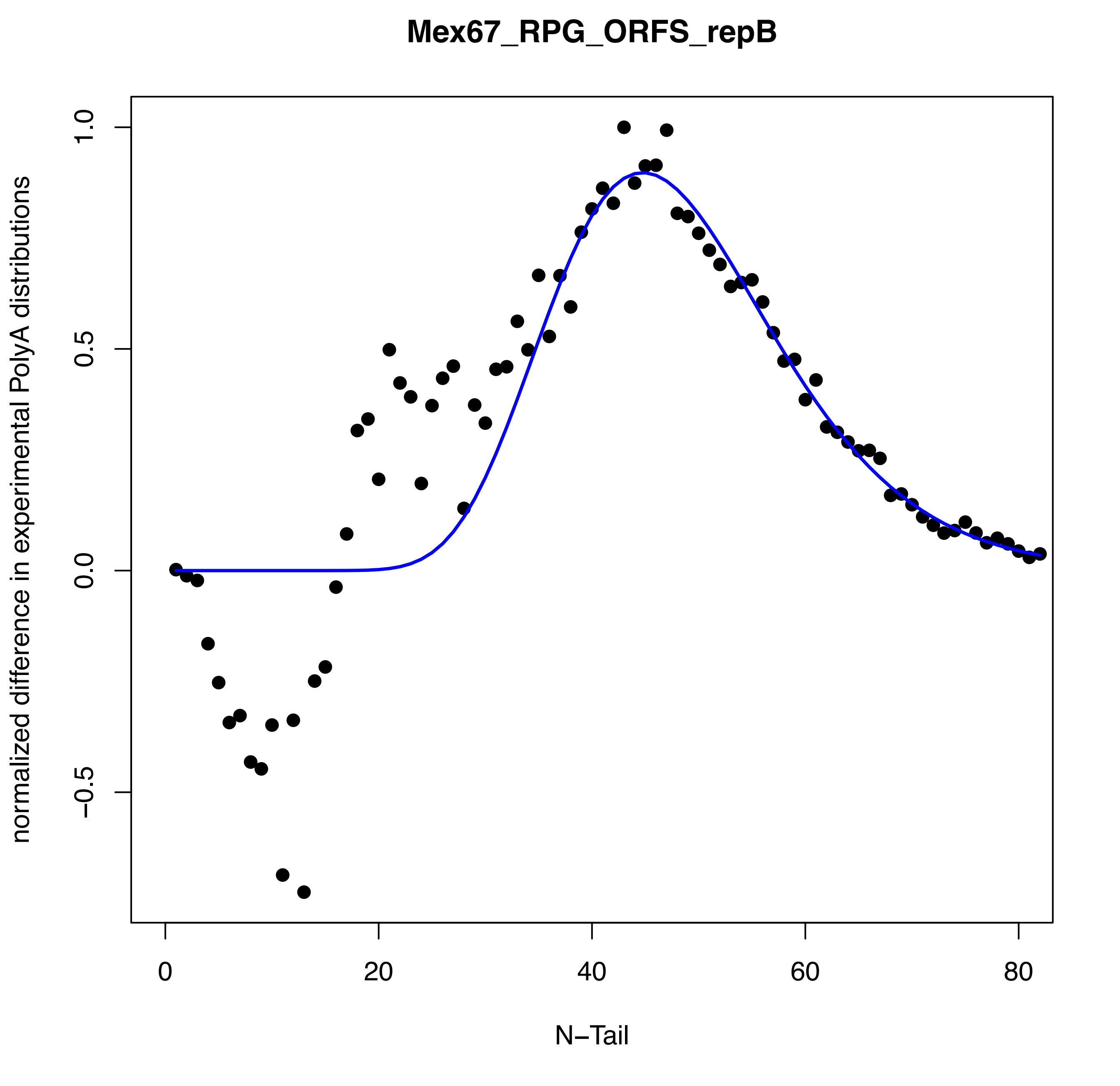

Mex67_RPG_ORFS_repB
normalized difference in experimental PolyA distributions
N−Tail
1.0
0.5
0.0
−0.5
0
20
40
60
80

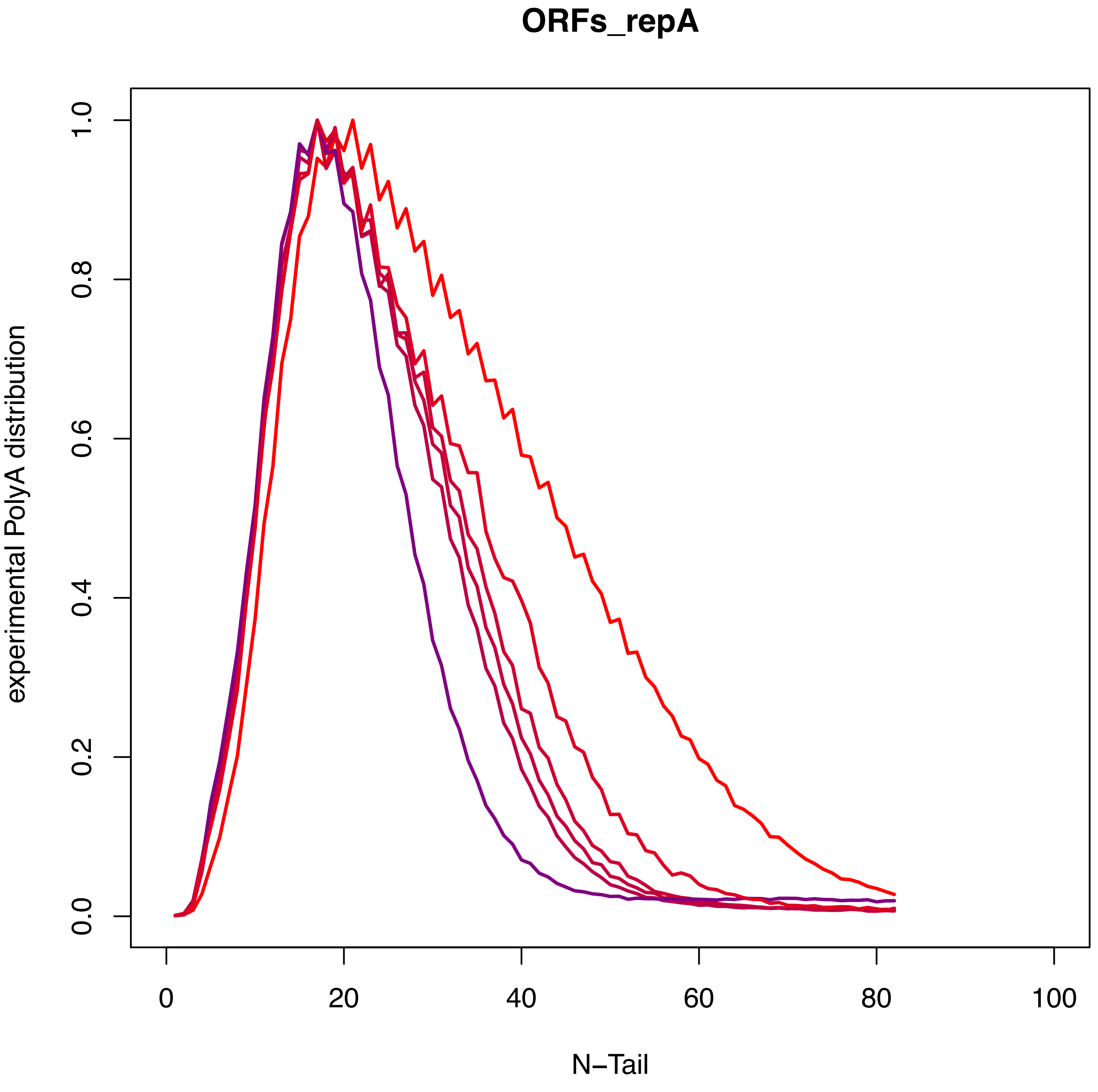
ORFs_repA
experimental PolyA distribution
N−Tail
0.0
0.2
0.4
0.6
0.8
1.0
0
20
40
60
80
100

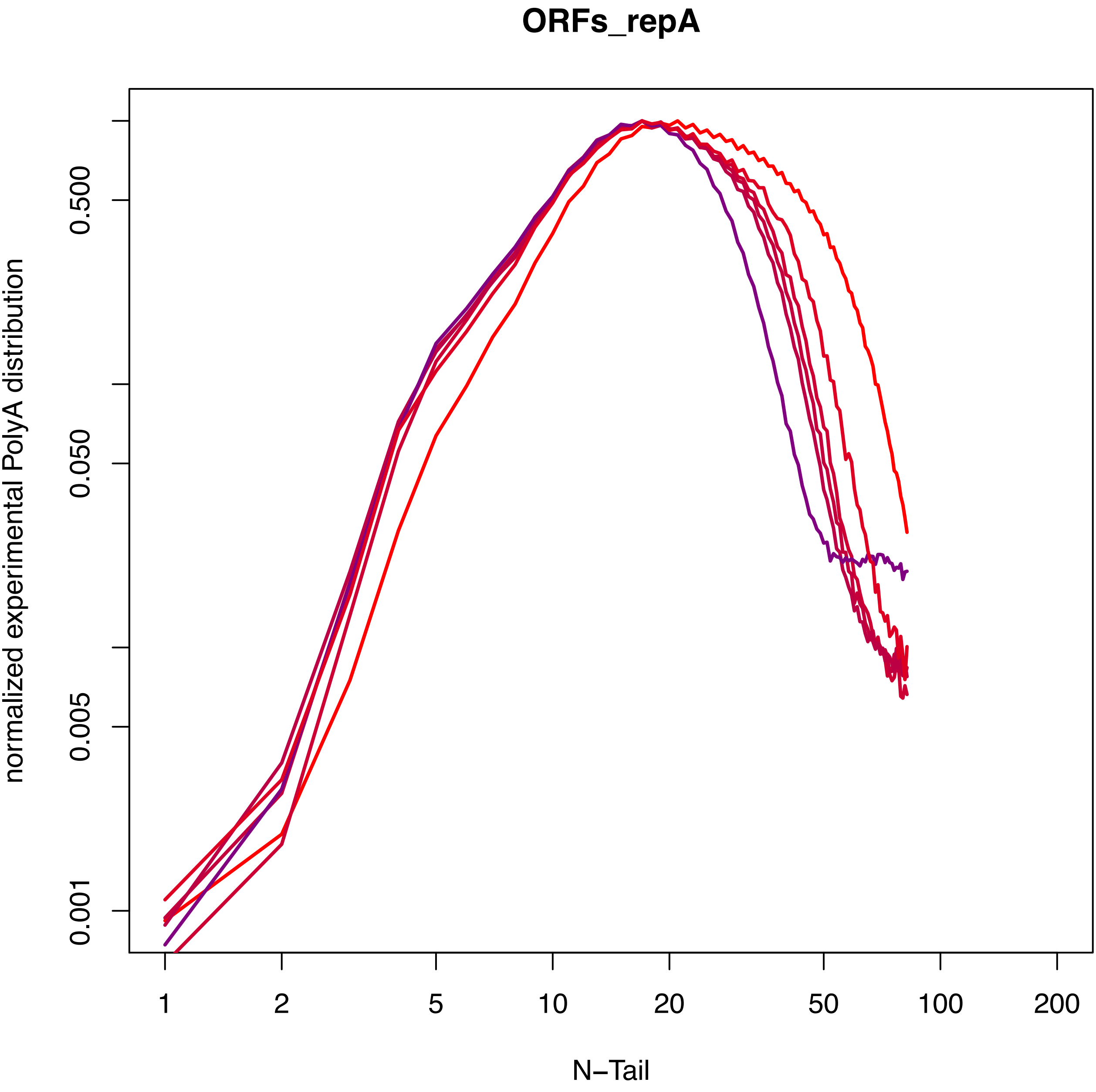
ORFs_repA
normalized experimental PolyA distribution
0.001
0.005
0.050
0.500
1
2
5
10
20
50
100
200
N−Tail

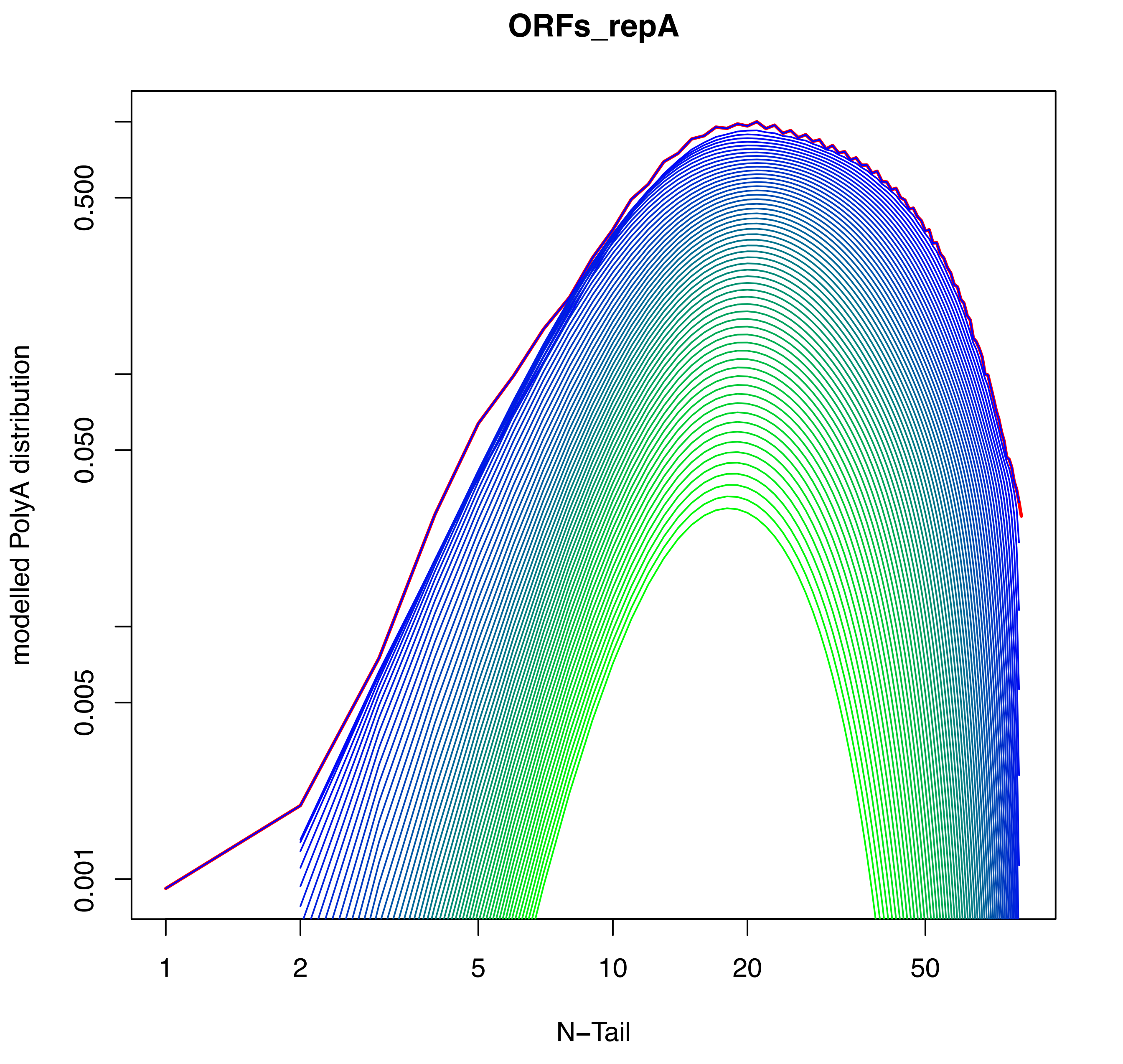
ORFs_repA
modelled PolyA distribution
0.001
0.005
0.050
0.500
1
2
5
10
20
50
N−Tail

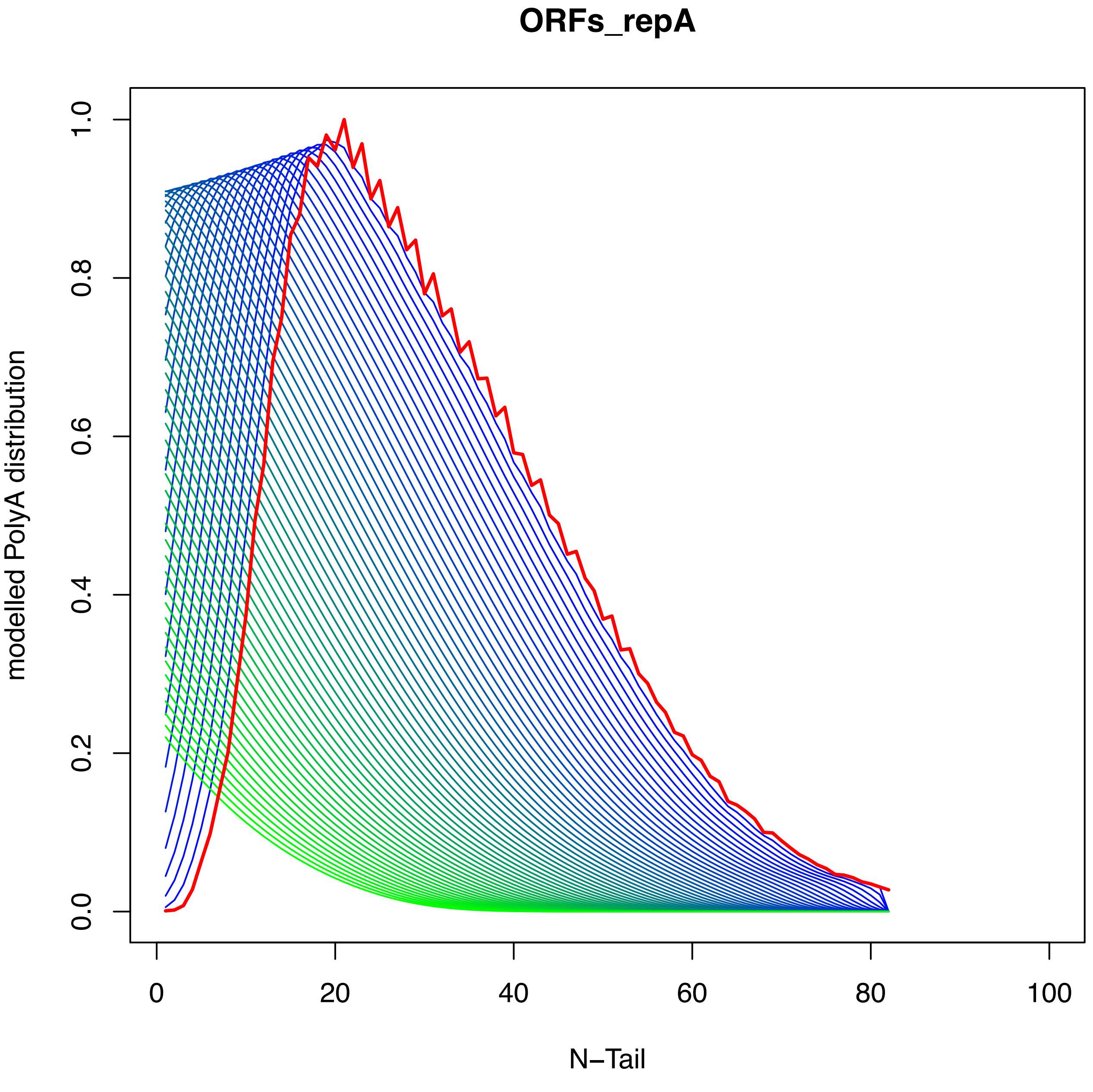
ORFs_repA
modelled PolyA distribution
N−Tail
0.0
0.2
0.4
0.6
0.8
1.0
0
20
40
60
80
100

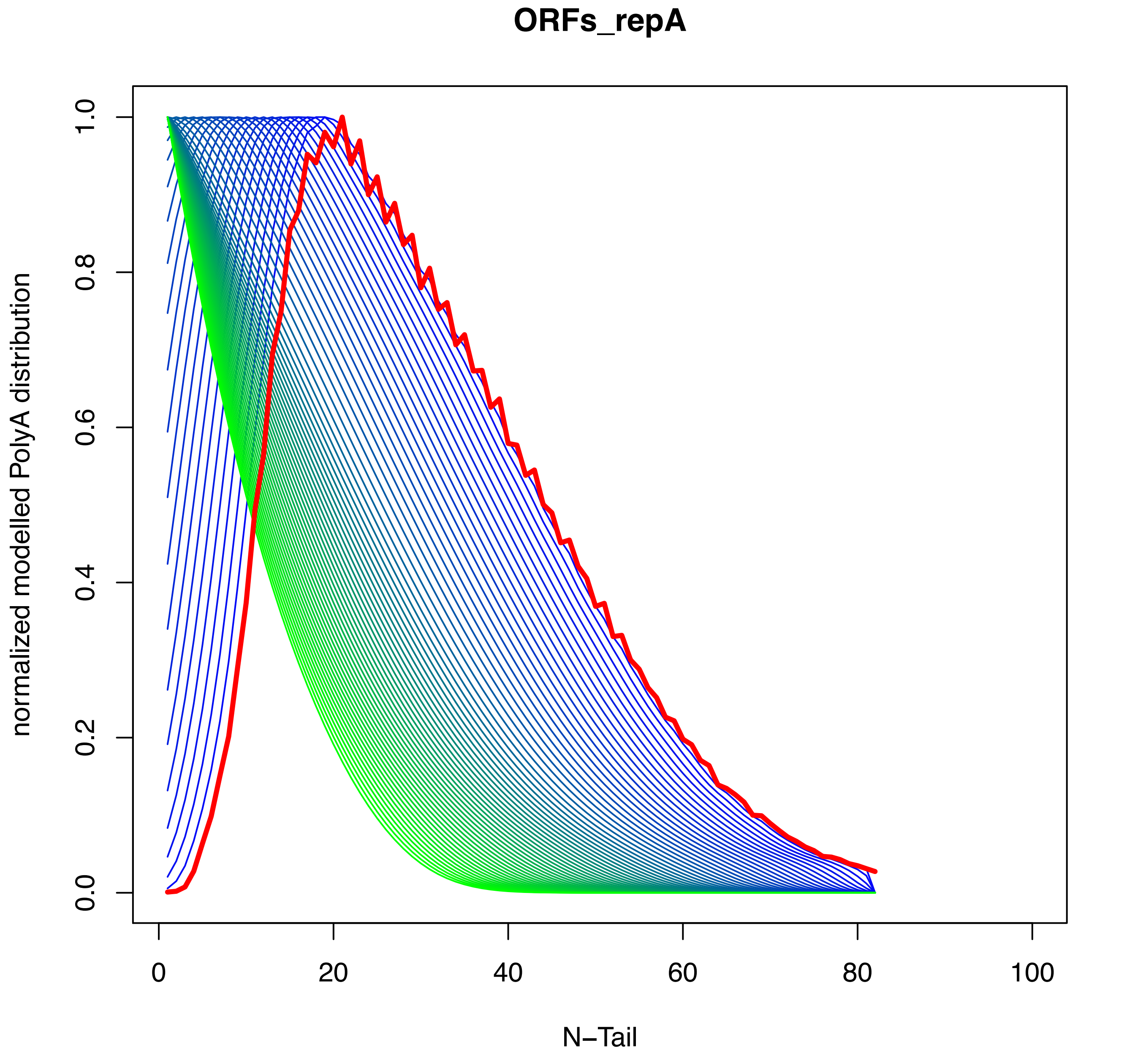

ORFs_repA
normalized modelled PolyA distribution
N−Tail
0.0
0.2
0.4
0.6
0.8
1.0
0
20
40
60
80
100

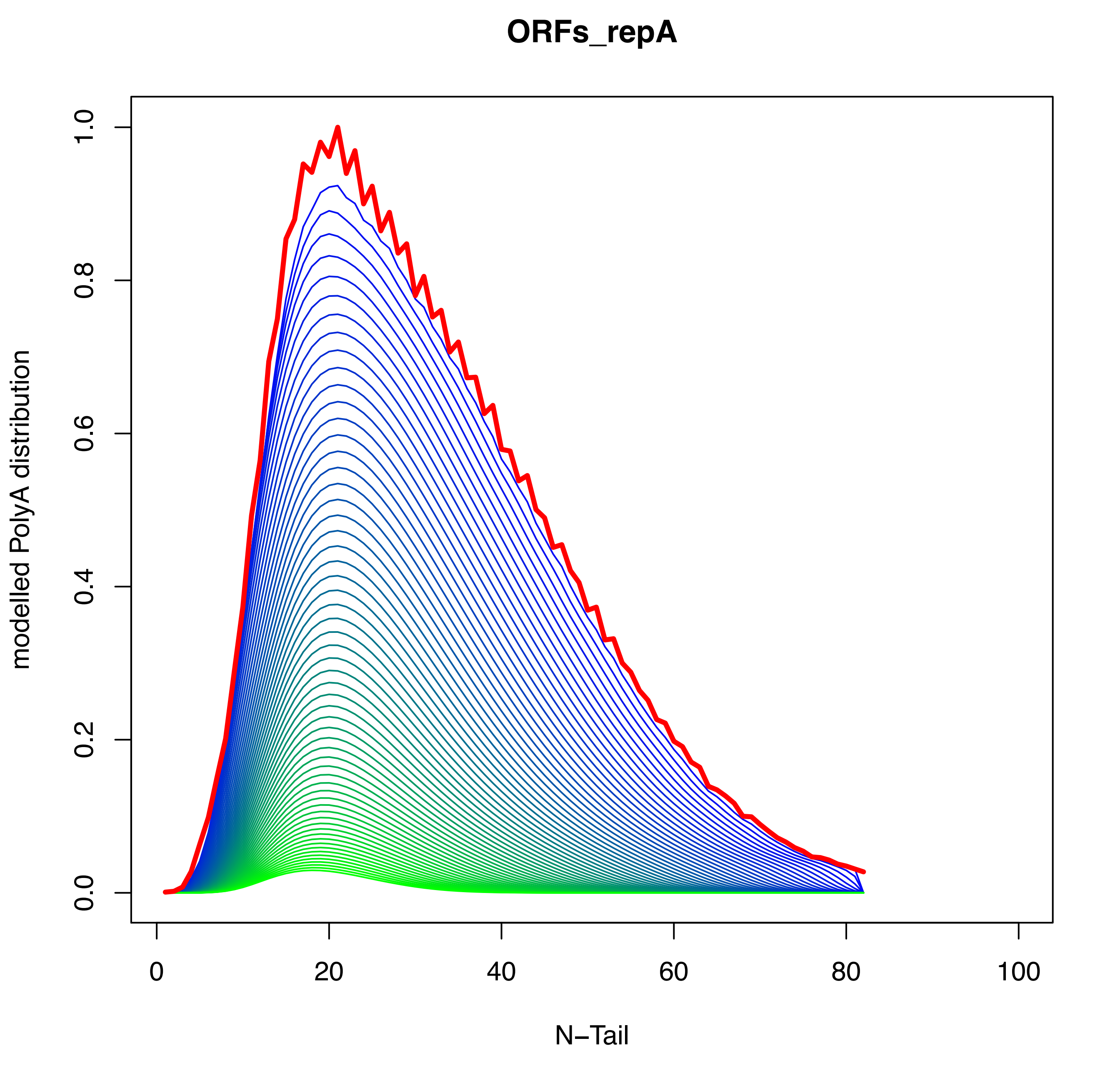

ORFs_repA
modelled PolyA distribution
N−Tail
0.0
0.2
0.4
0.6
0.8
1.0
0
20
40
60
80
100

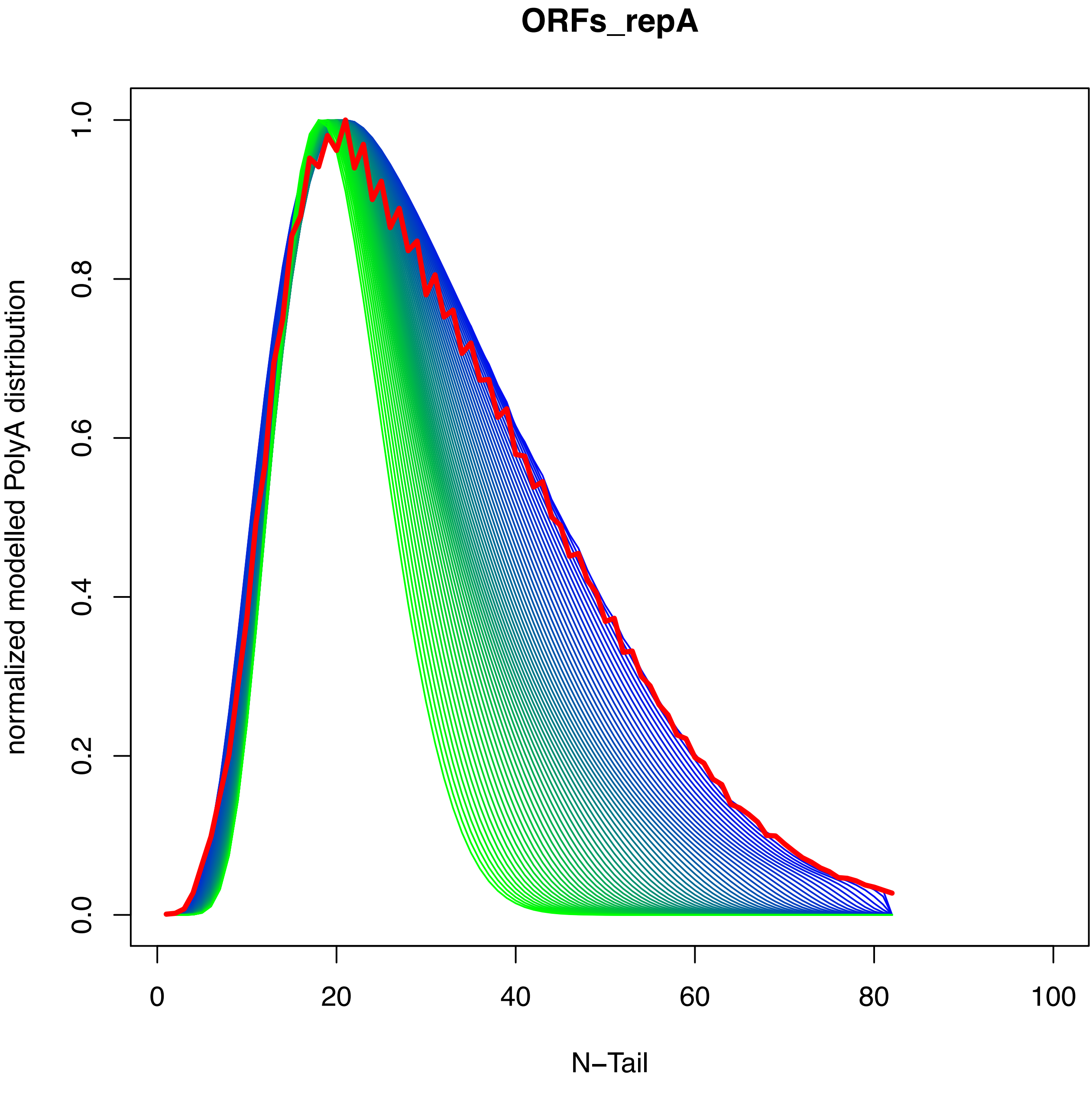
ORFs_repA
normalized modelled PolyA distribution
0.0
0.2
0.4
0.6
0.8
1.0
0
20
40
60
80
100
N−Tail

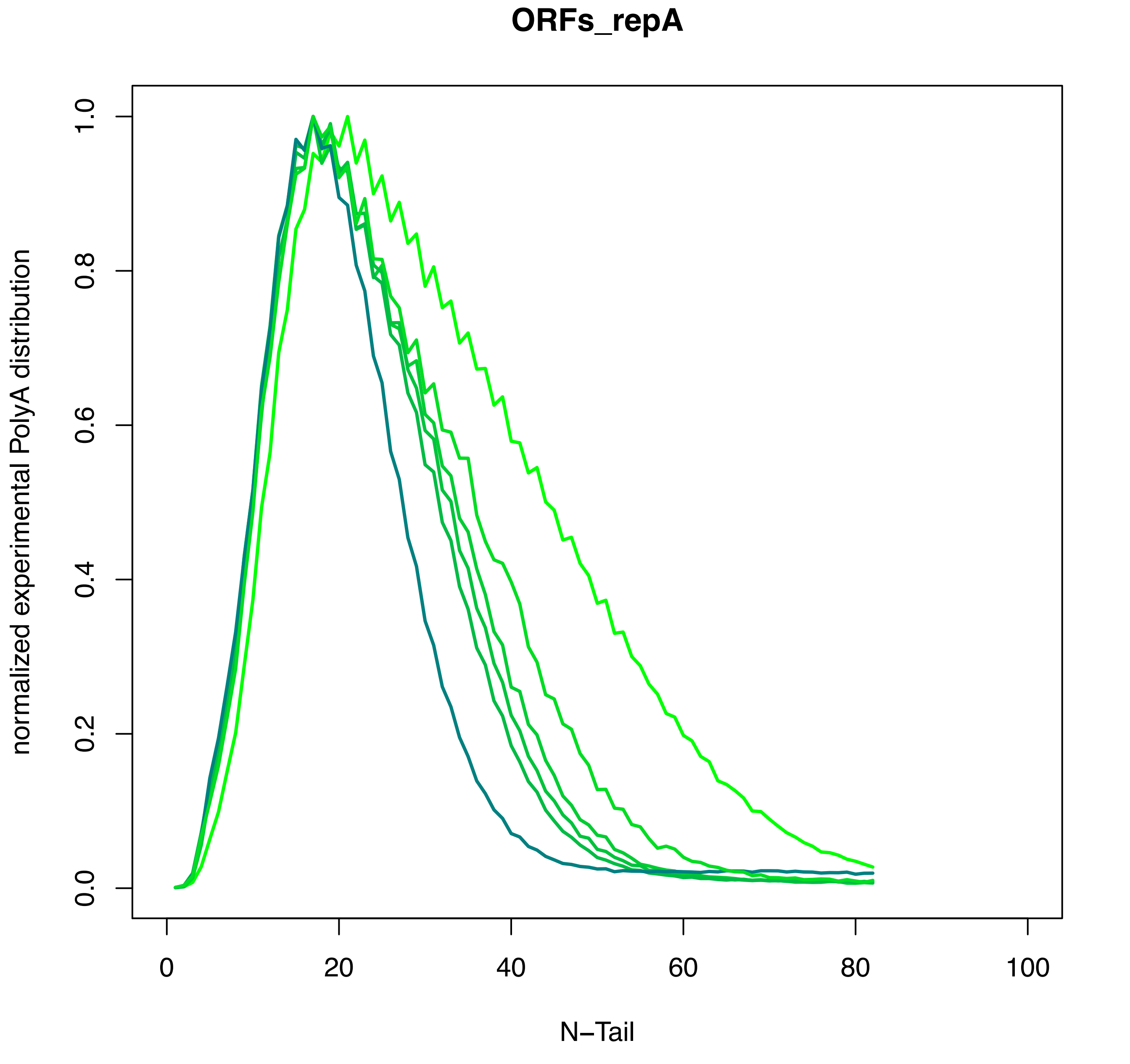
ORFs_repA
normalized experimental PolyA distribution
N−Tail
0.0
0.2
0.4
0.6
0.8
1.0
0
20
40
60
80
100

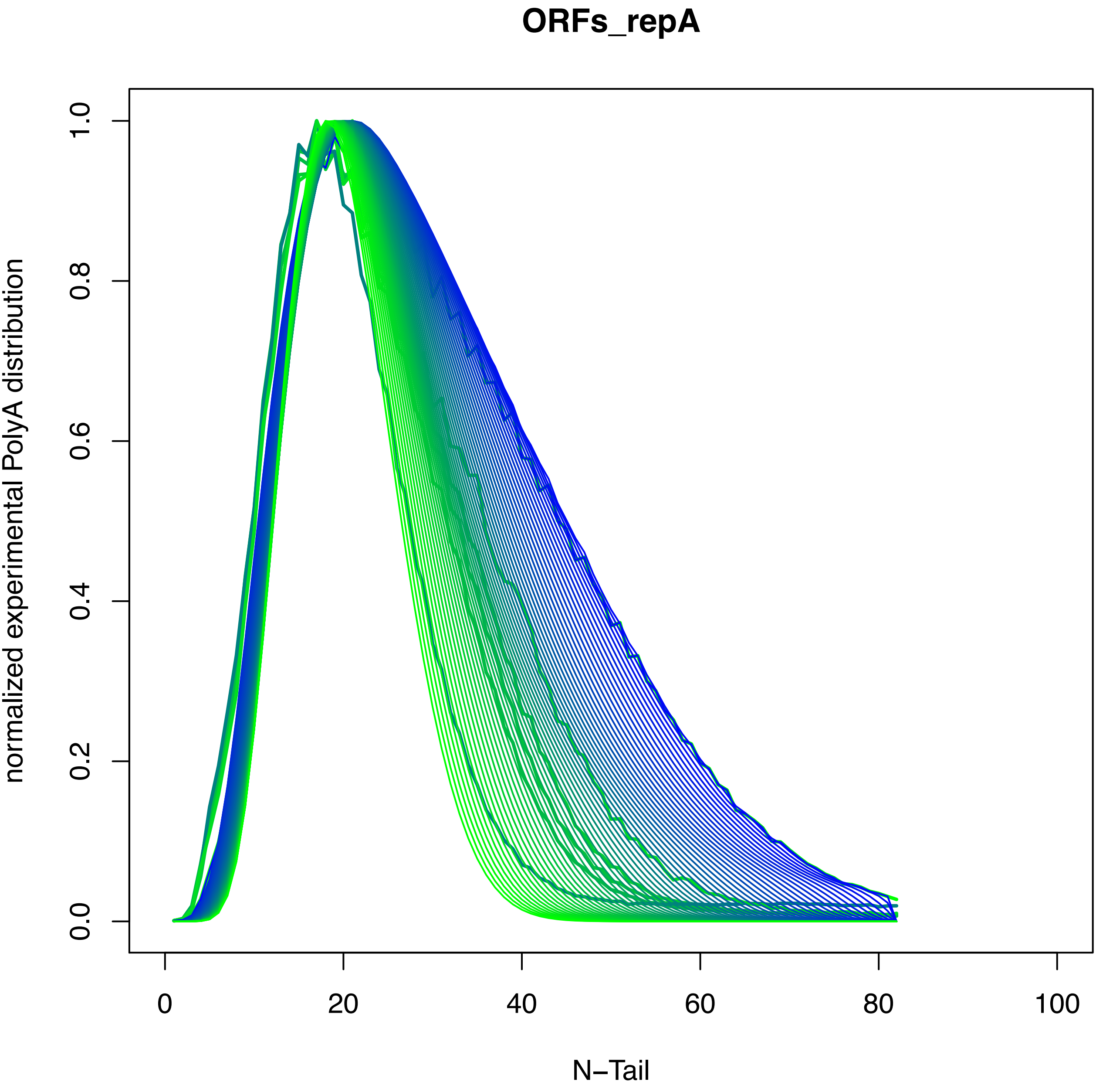

ORFs_repA
normalized experimental PolyA distribution
N−Tail
0.0
0.2
0.4
0.6
0.8
1.0
0
20
40
60
80
100

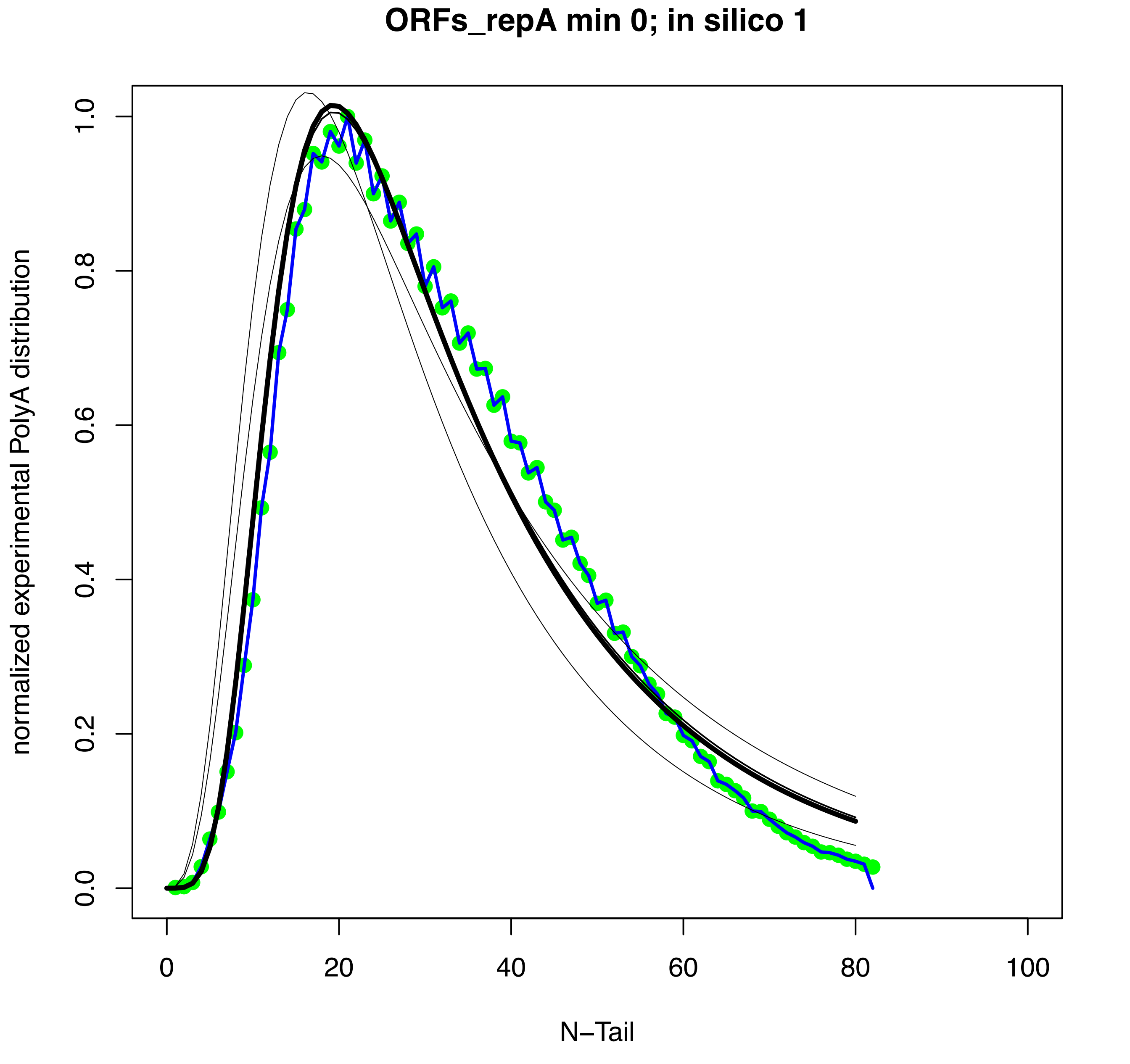

ORFs_repA min 0; in silico 1
normalized experimental PolyA distribution
N−Tail
0.0
0.2
0.4
0.6
0.8
1.0
0
20
40
60
80
100

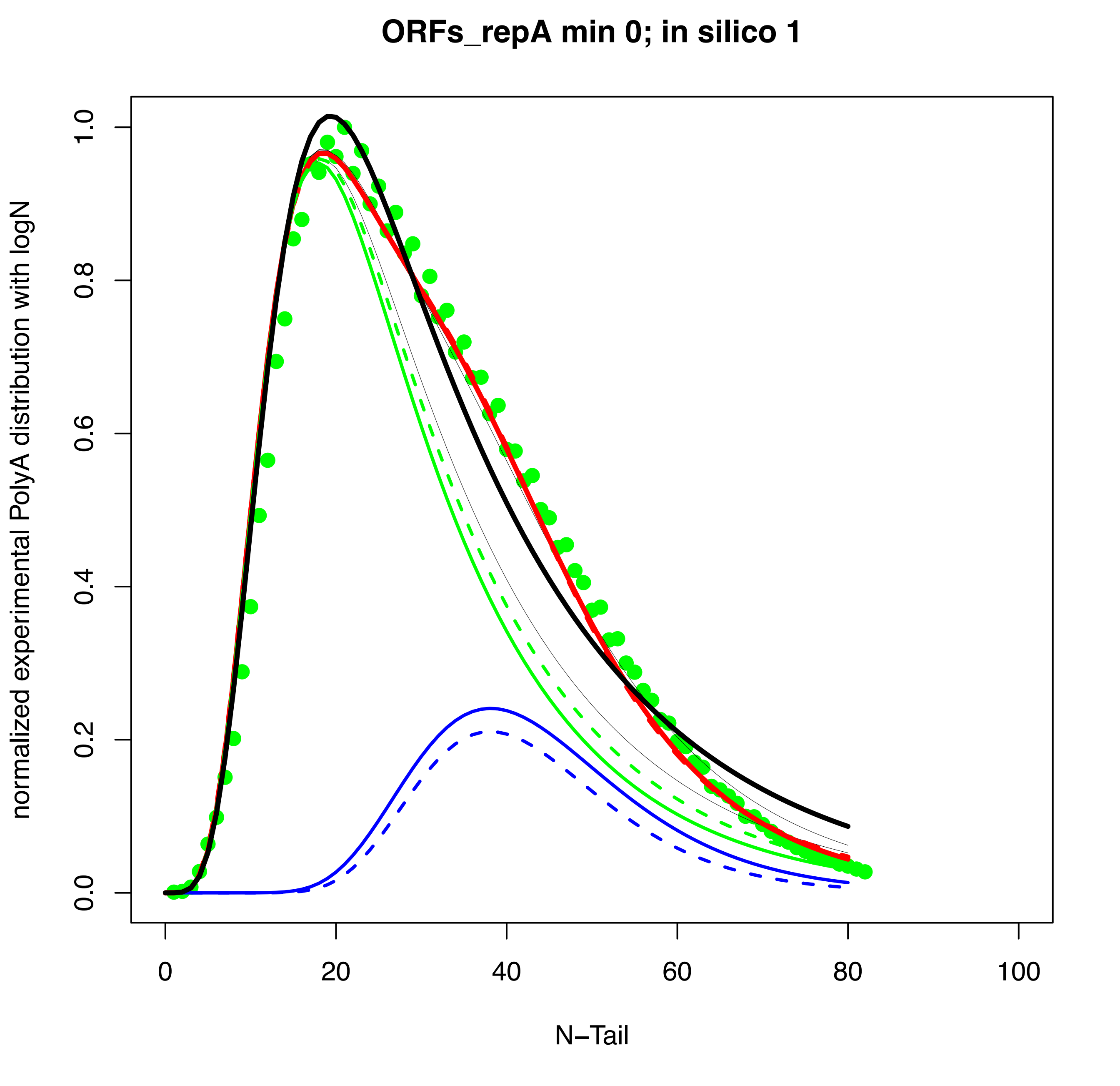

ORFs_repA min 0; in silico 1
normalized experimental PolyA distribution with logN
N−Tail
0.0
0.2
0.4
0.6
0.8
1.0
0
20
40
60
80
100

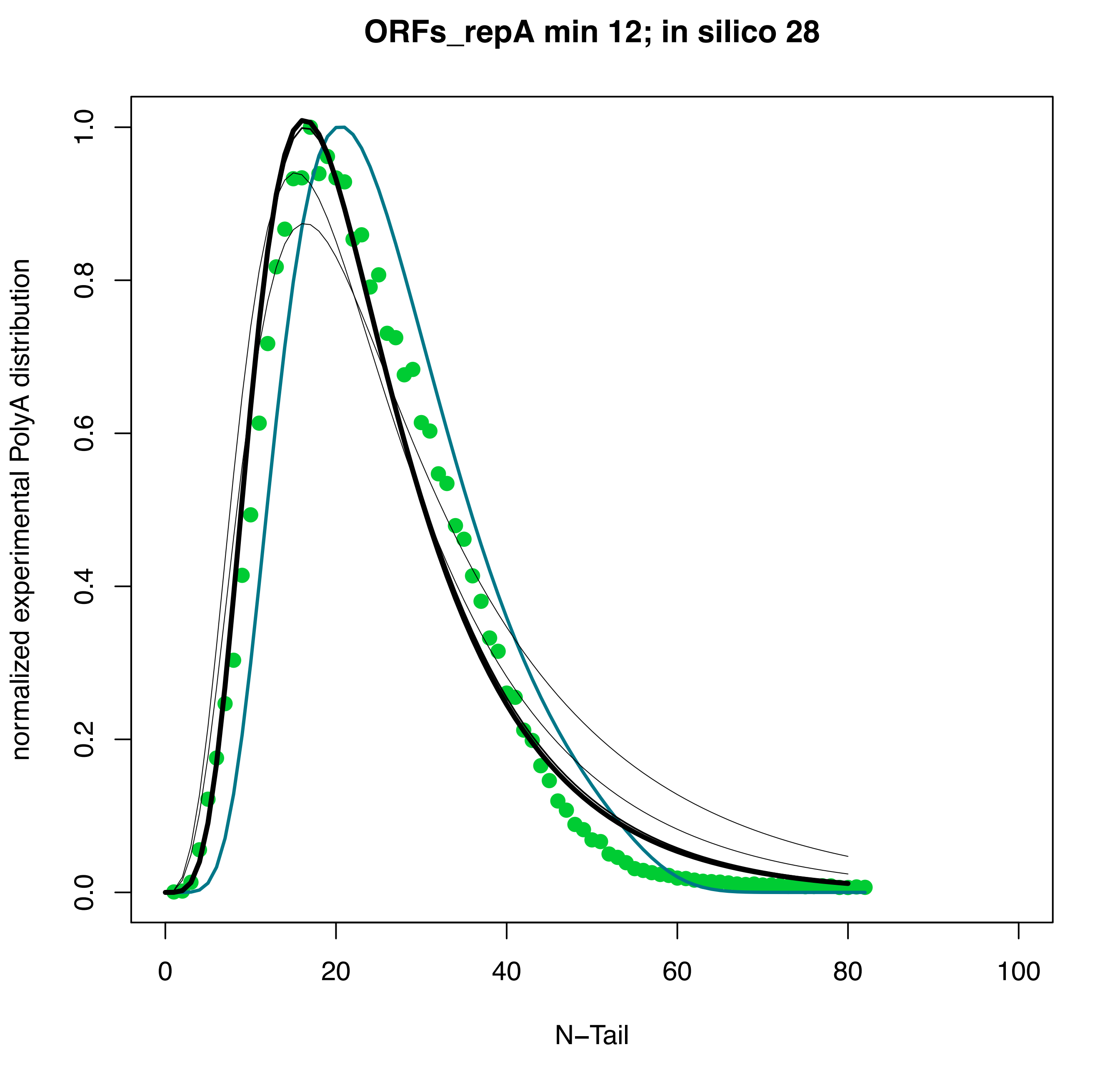

ORFs_repA min 12; in silico 28
normalized experimental PolyA distribution
N−Tail
0.0
0.2
0.4
0.6
0.8
1.0
0
20
40
60
80
100

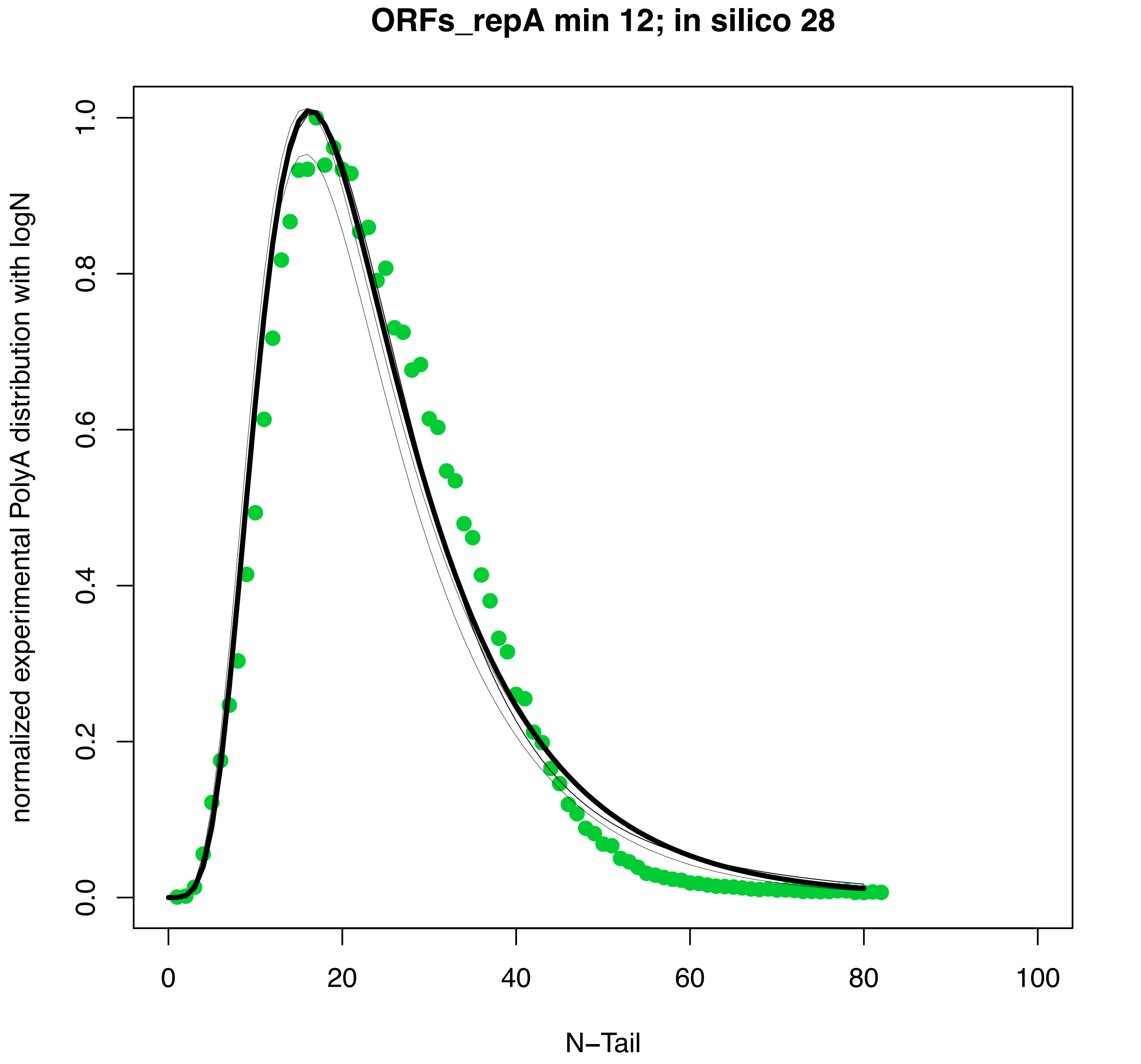

ORFs_repA min 12; in silico 28
normalized experimental PolyA distribution with logN
N−Tail
0.0
0.2
0.4
0.6
0.8
1.0
0
20
40
60
80
100

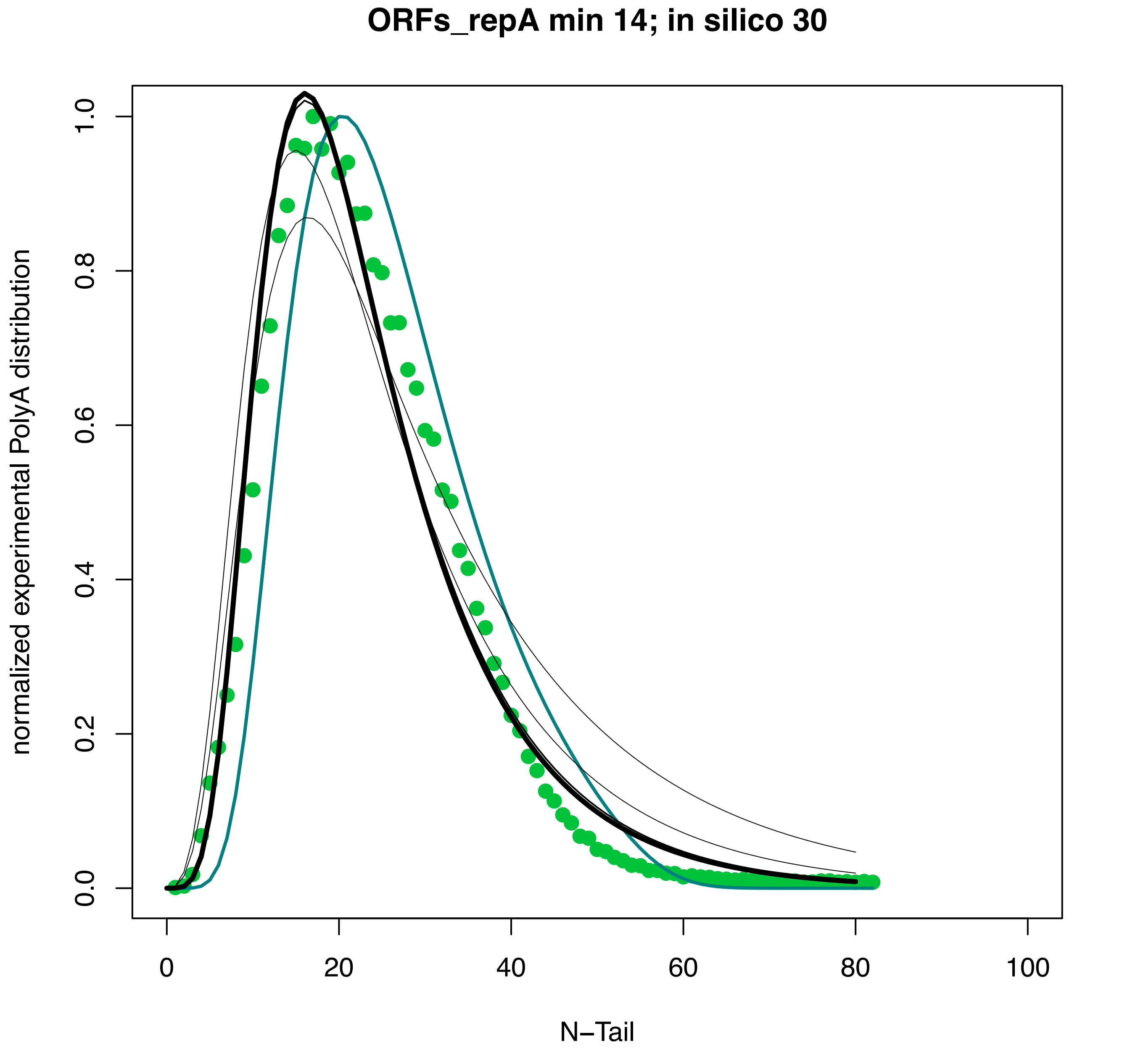

ORFs_repA min 14; in silico 30
normalized experimental PolyA distribution
N−Tail
0.0
0.2
0.4
0.6
0.8
1.0
0
20
40
60
80
100

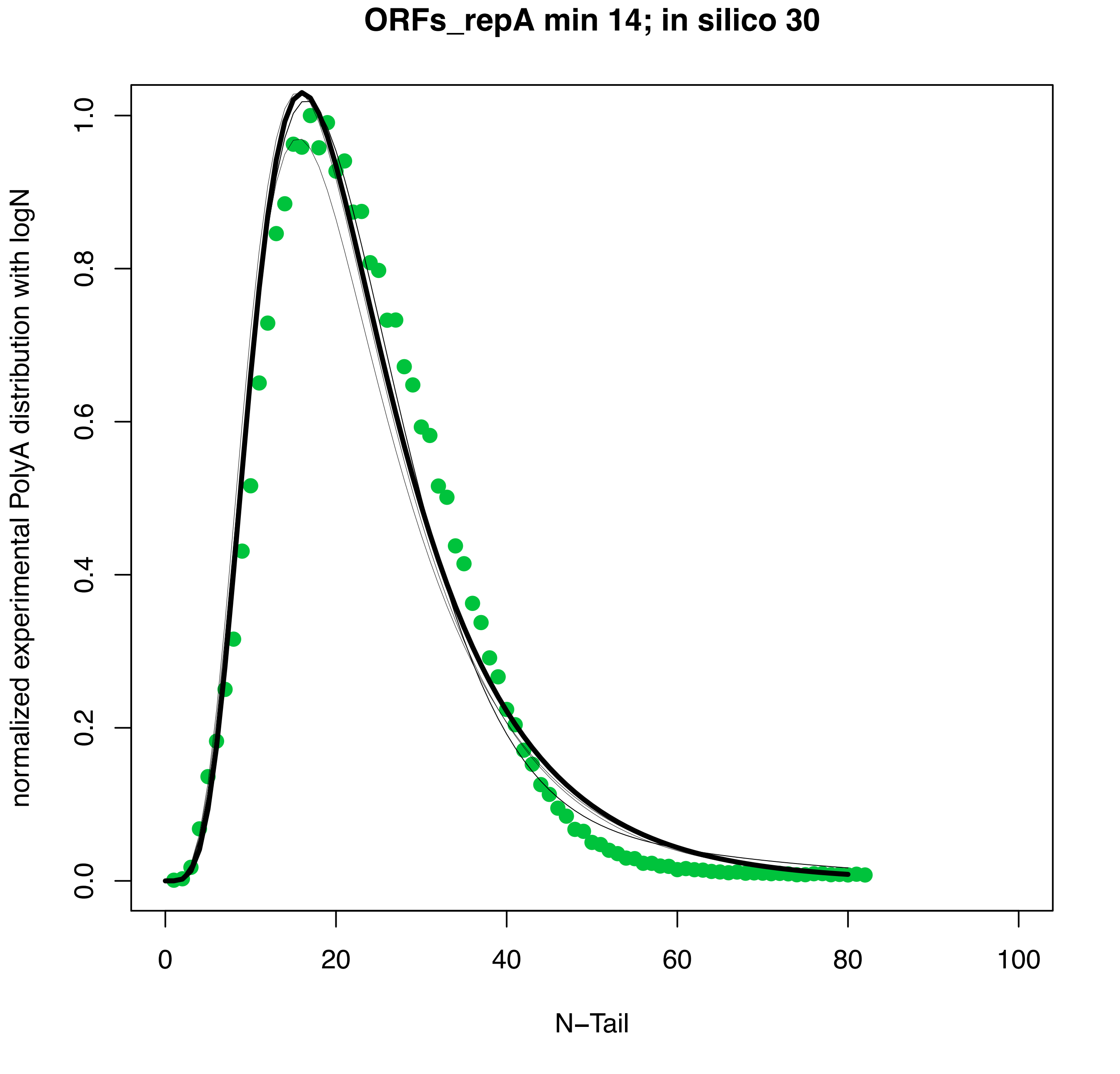

ORFs_repA min 14; in silico 30
normalized experimental PolyA distribution with logN
N−Tail
0.0
0.2
0.4
0.6
0.8
1.0
0
20
40
60
80
100

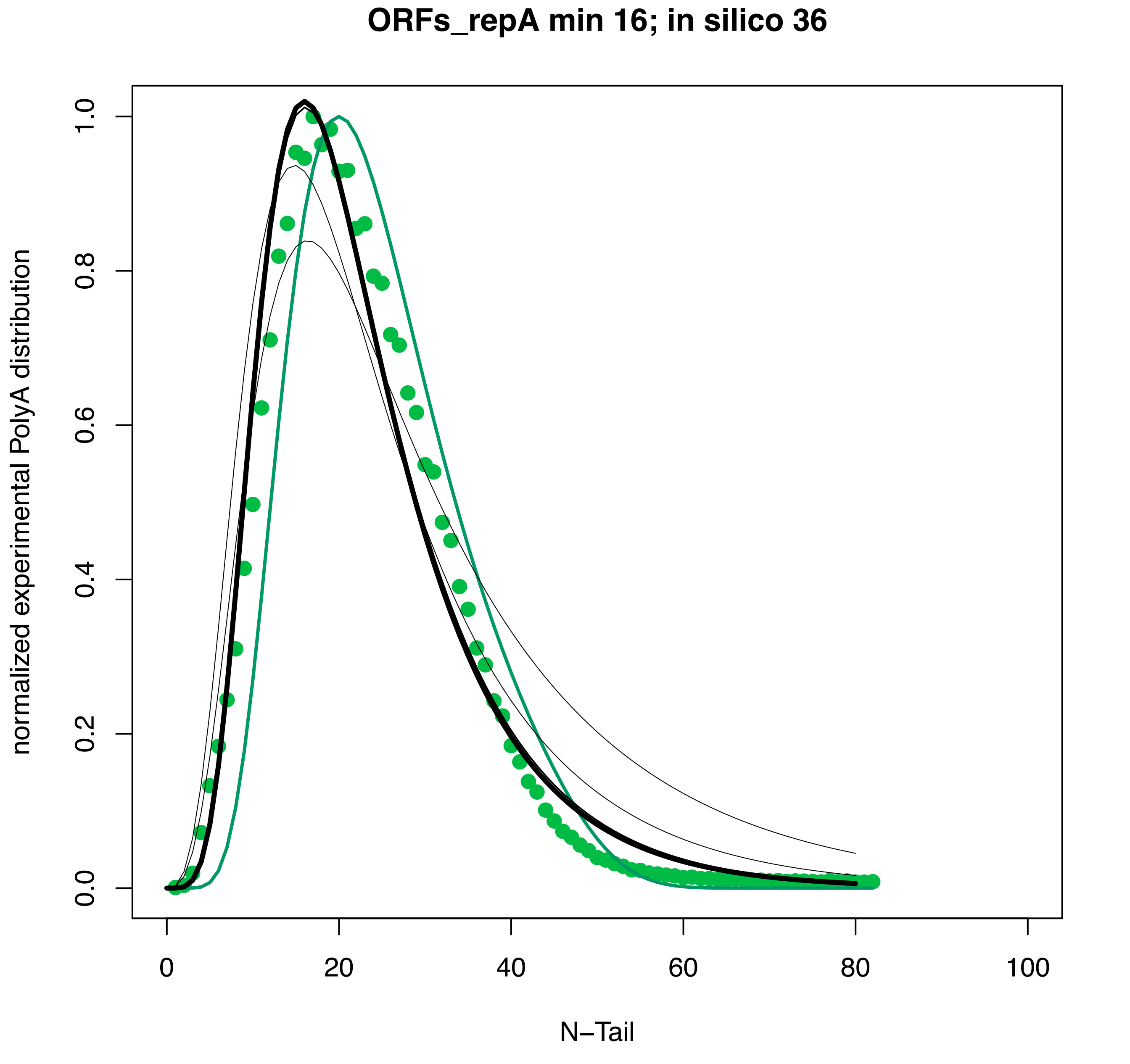

ORFs_repA min 16; in silico 36
normalized experimental PolyA distribution
N−Tail
0.0
0.2
0.4
0.6
0.8
1.0
0
20
40
60
80
100

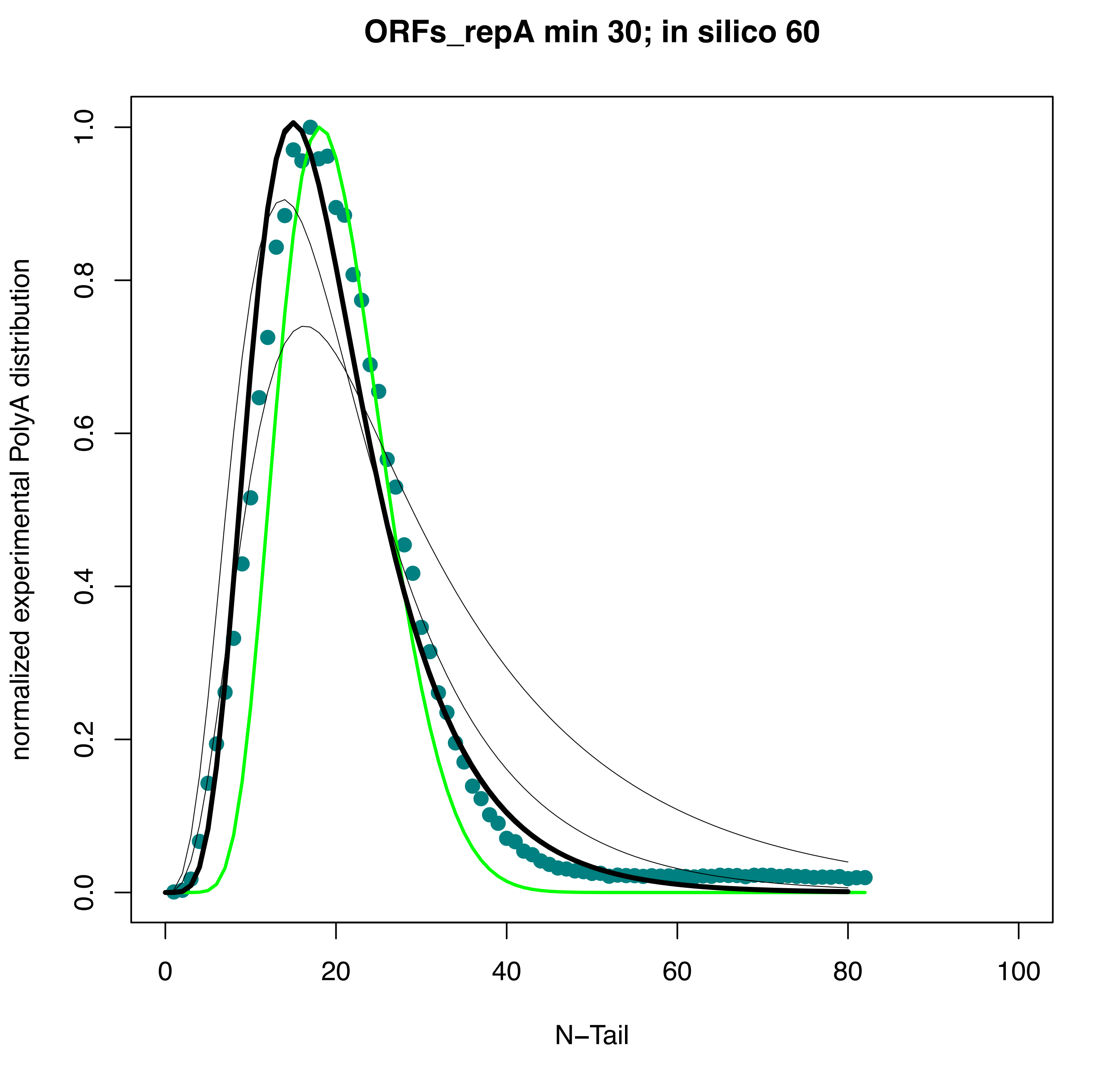

ORFs_repA min 30; in silico 60
normalized experimental PolyA distribution
N−Tail
0.0
0.2
0.4
0.6
0.8
1.0
0
20
40
60
80
100

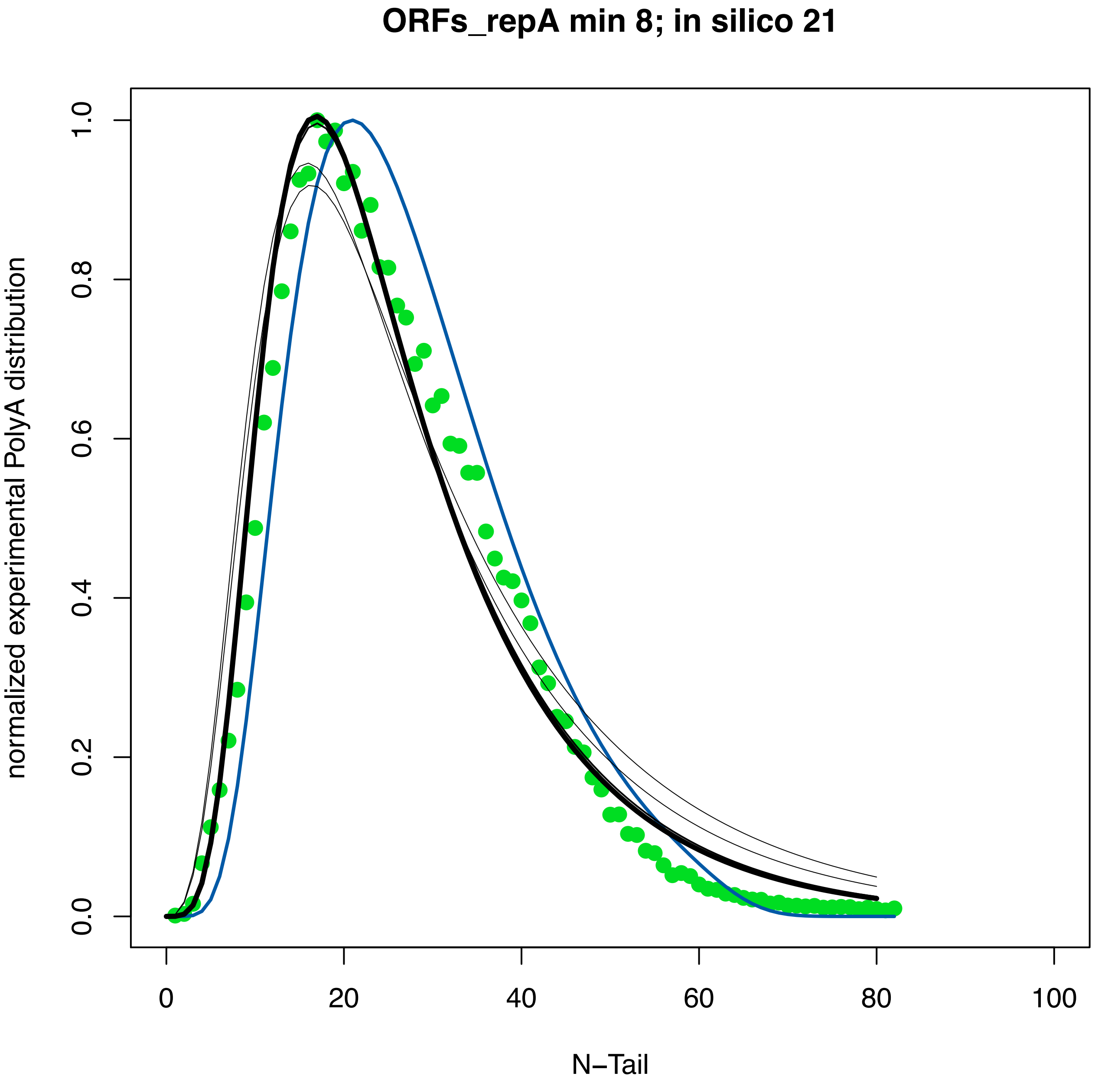

ORFs_repA min 8; in silico 21
normalized experimental PolyA distribution
N−Tail
0.0
0.2
0.4
0.6
0.8
1.0
0
20
40
60
80
100

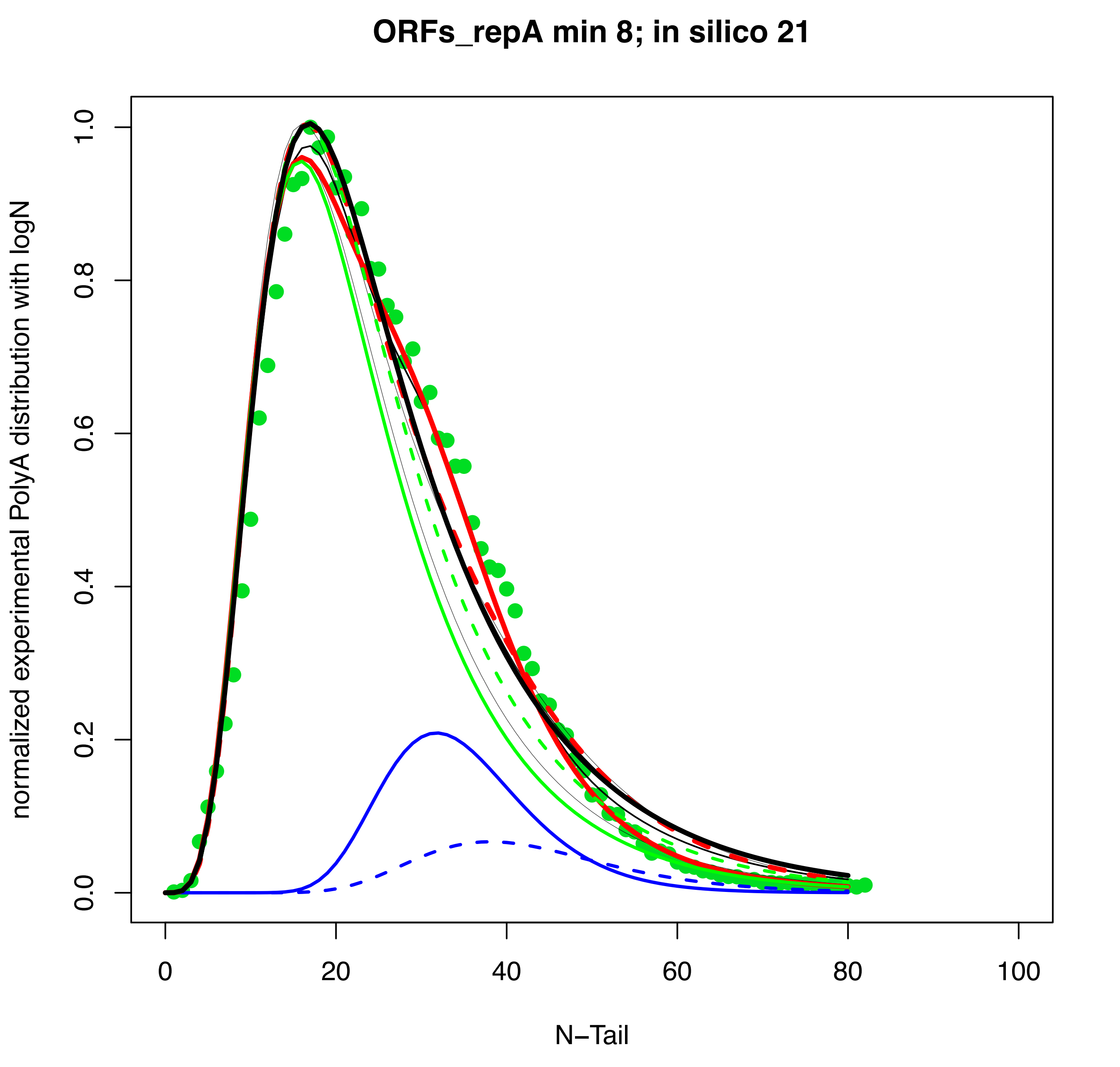

ORFs_repA min 8; in silico 21
normalized experimental PolyA distribution with logN
N−Tail
0.0
0.2
0.4
0.6
0.8
1.0
0
20
40
60
80
100

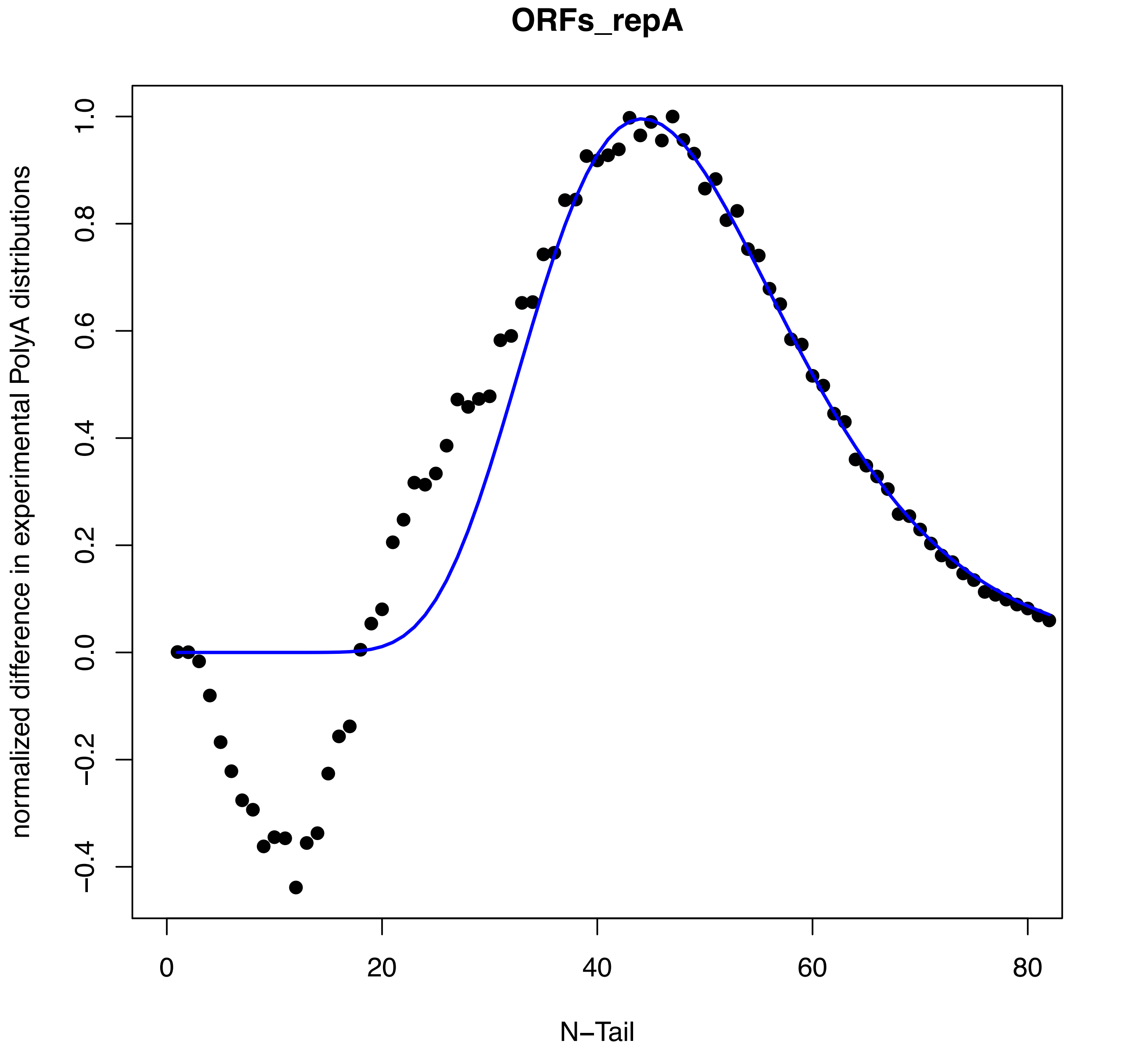

ORFs_repA
normalized difference in experimental PolyA distributions
N−Tail
1.0
0.8
0.6
0.4
0.2
0.0
−0.2
−0.4
0
20
40
60
80

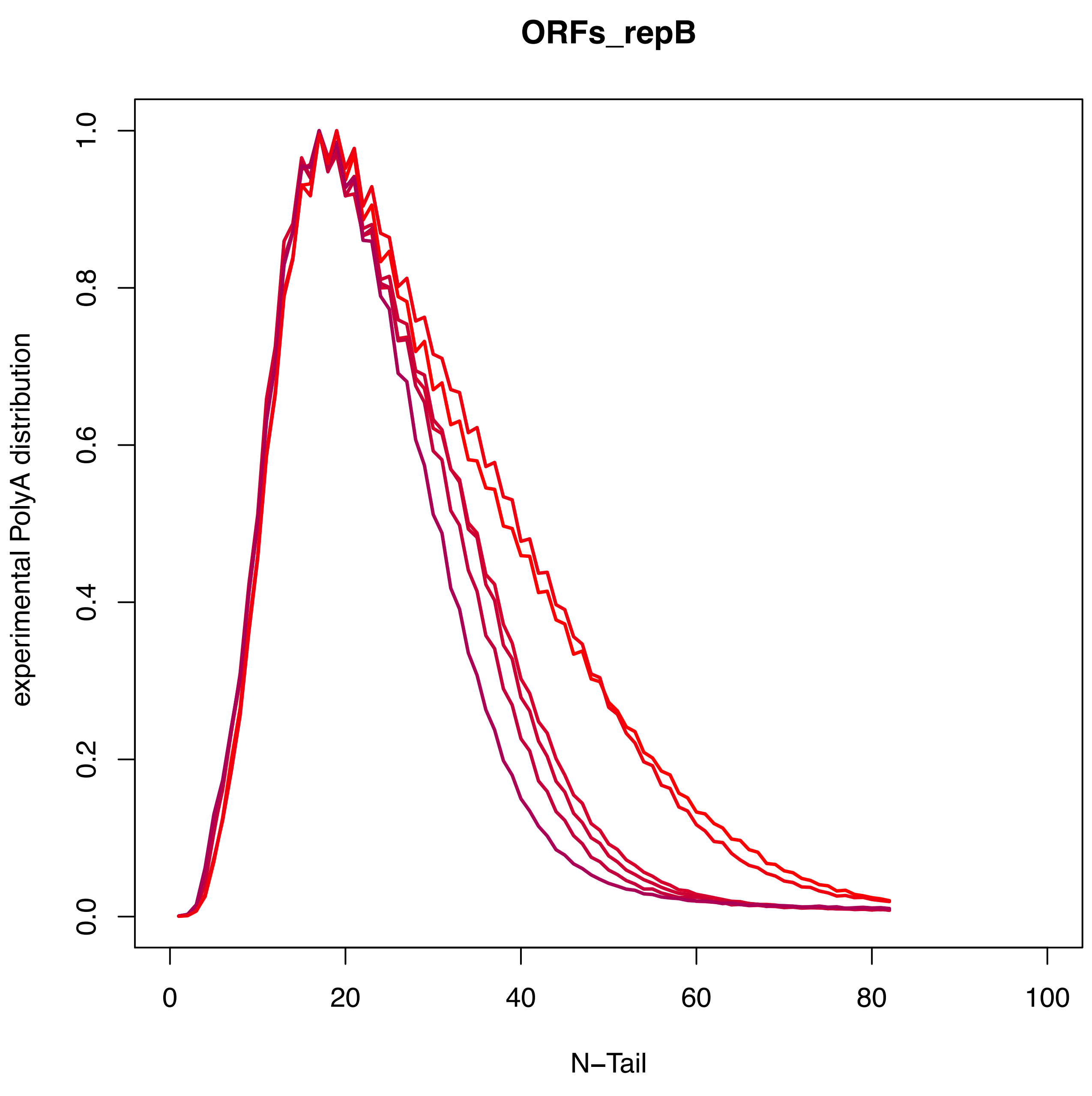

ORFs_repB
experimental PolyA distribution
N−Tail
0.0
0.2
0.4
0.6
0.8
1.0
0
20
40
60
80
100

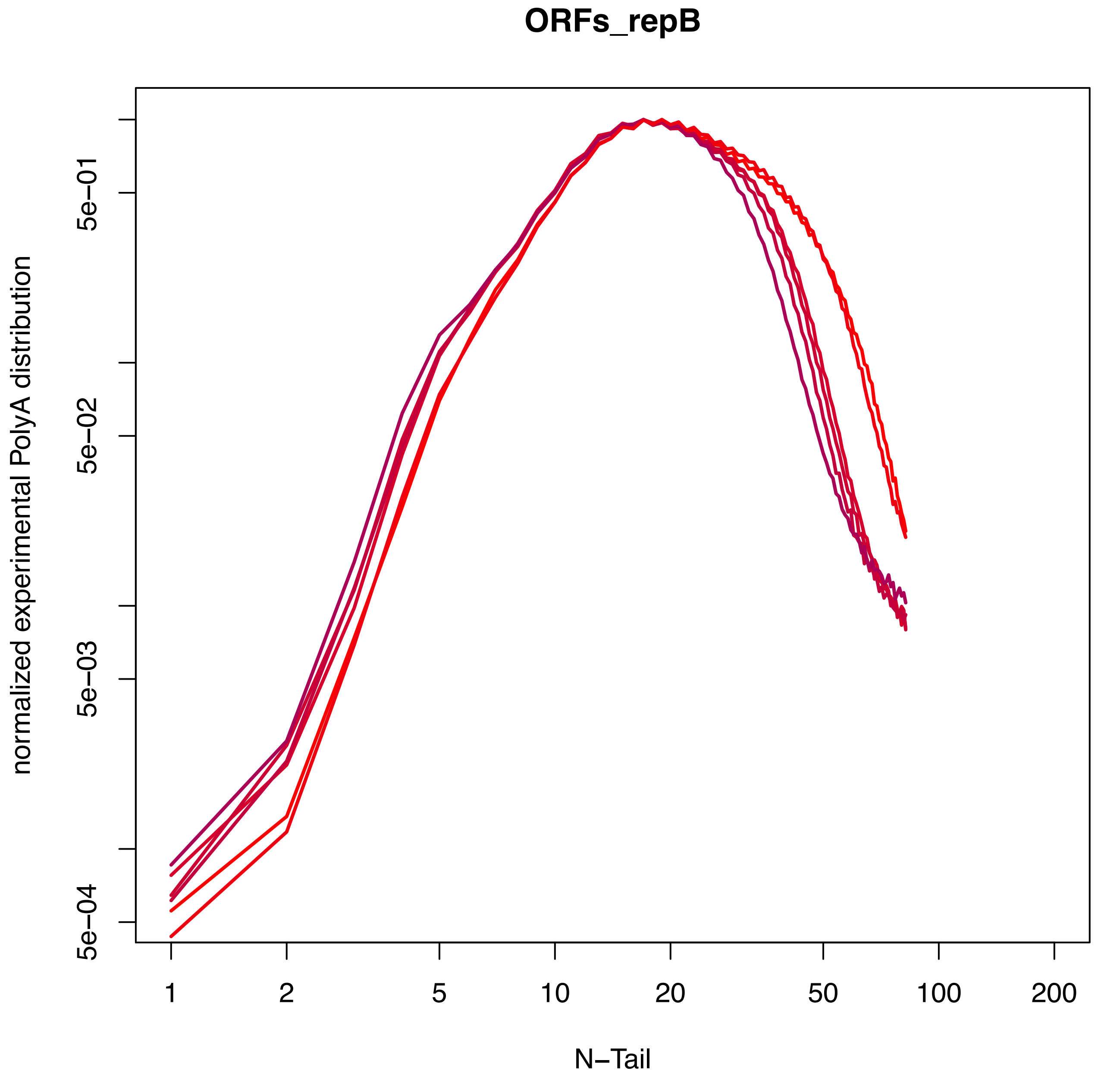

ORFs_repB
normalized experimental PolyA distribution
5e−04
5e−03
5e−02
5e−01
1
2
5
10
20
50
100
200
N−Tail

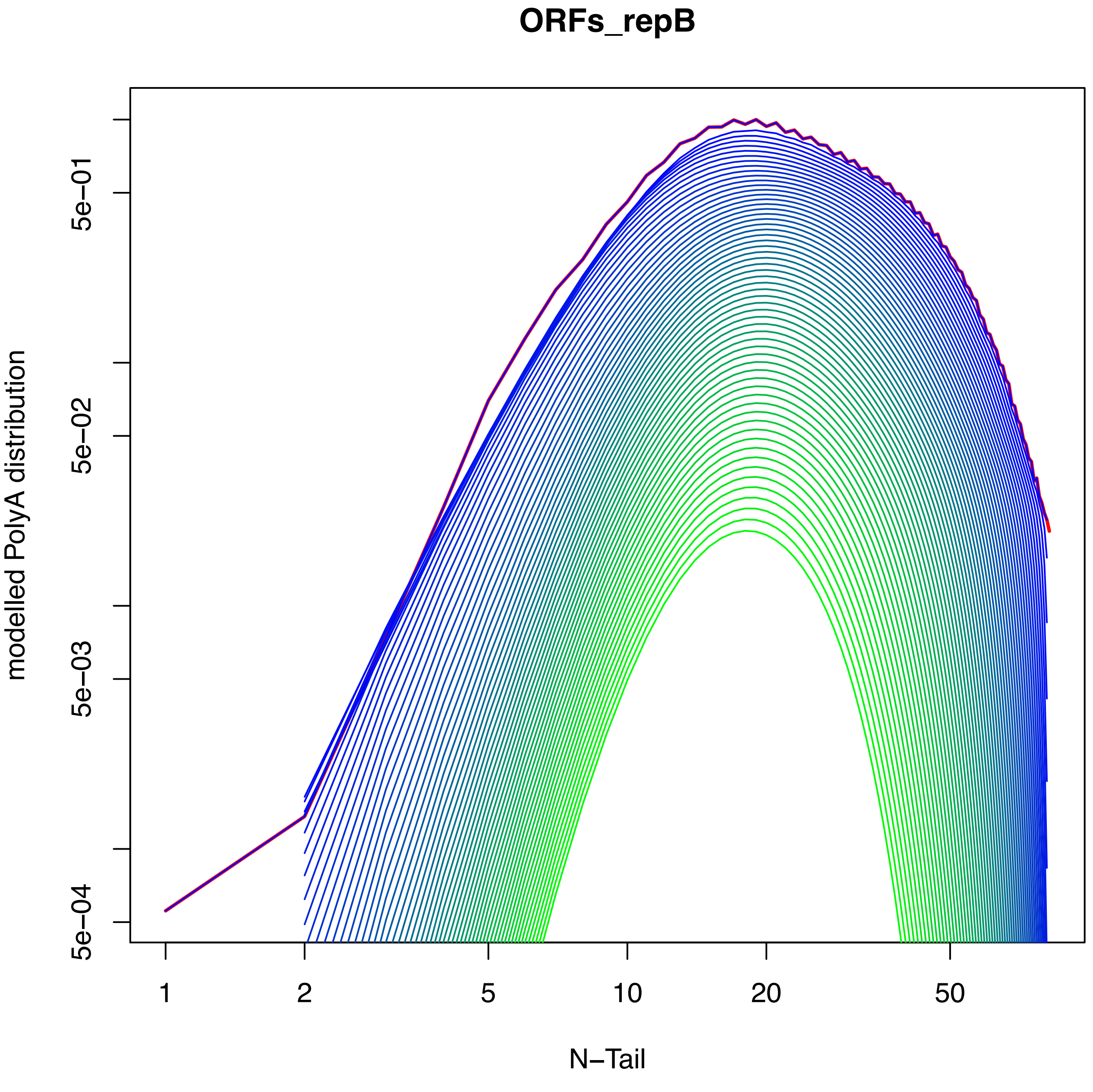

ORFs_repB
modelled PolyA distribution
5e−01
5e−02
5e−03
5e−04
1
2
5
10
20
50
N−Tail

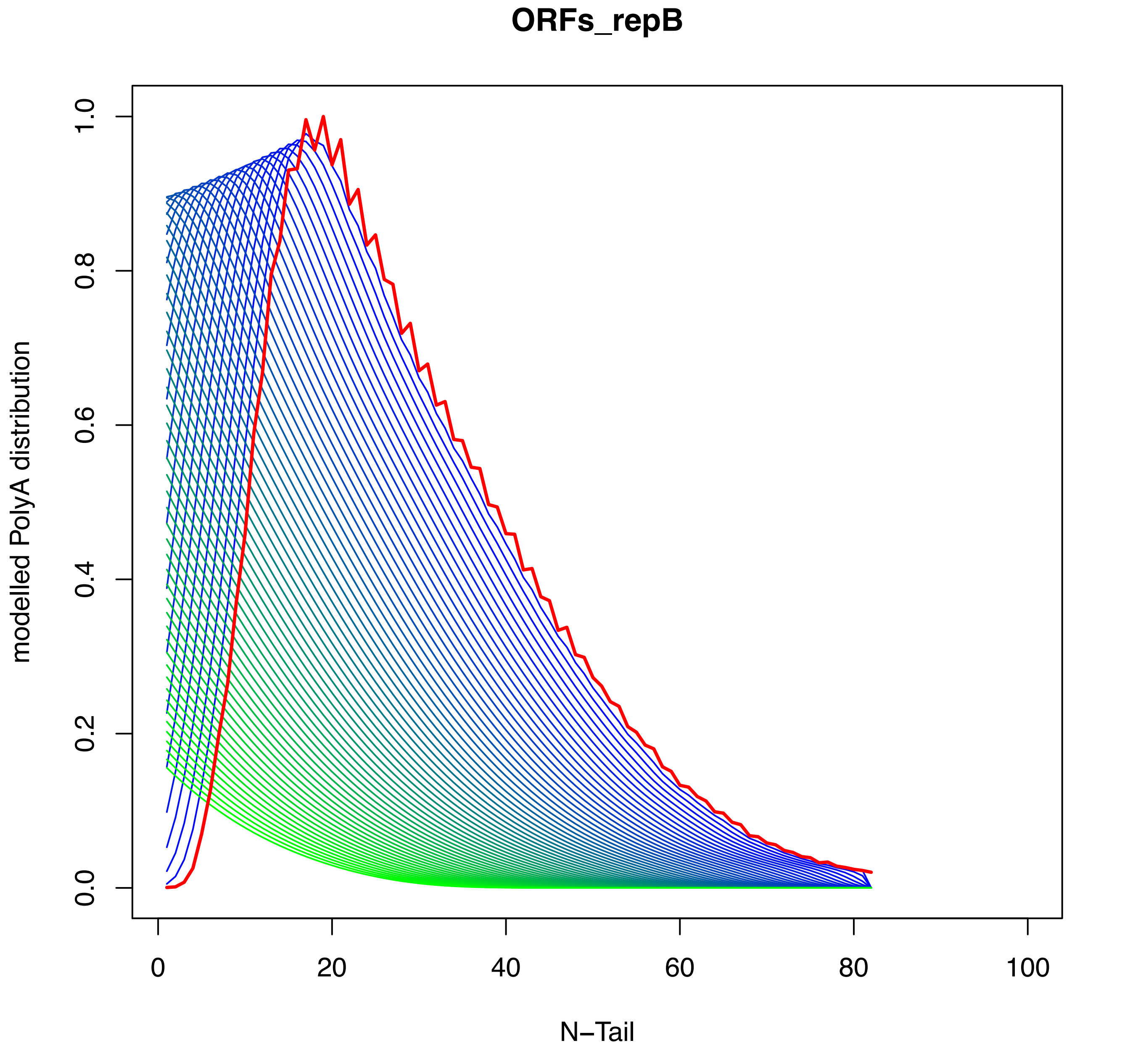

ORFs_repB
modelled PolyA distribution
N−Tail
0.0
0.2
0.4
0.6
0.8
1.0
0
20
40
60
80
100

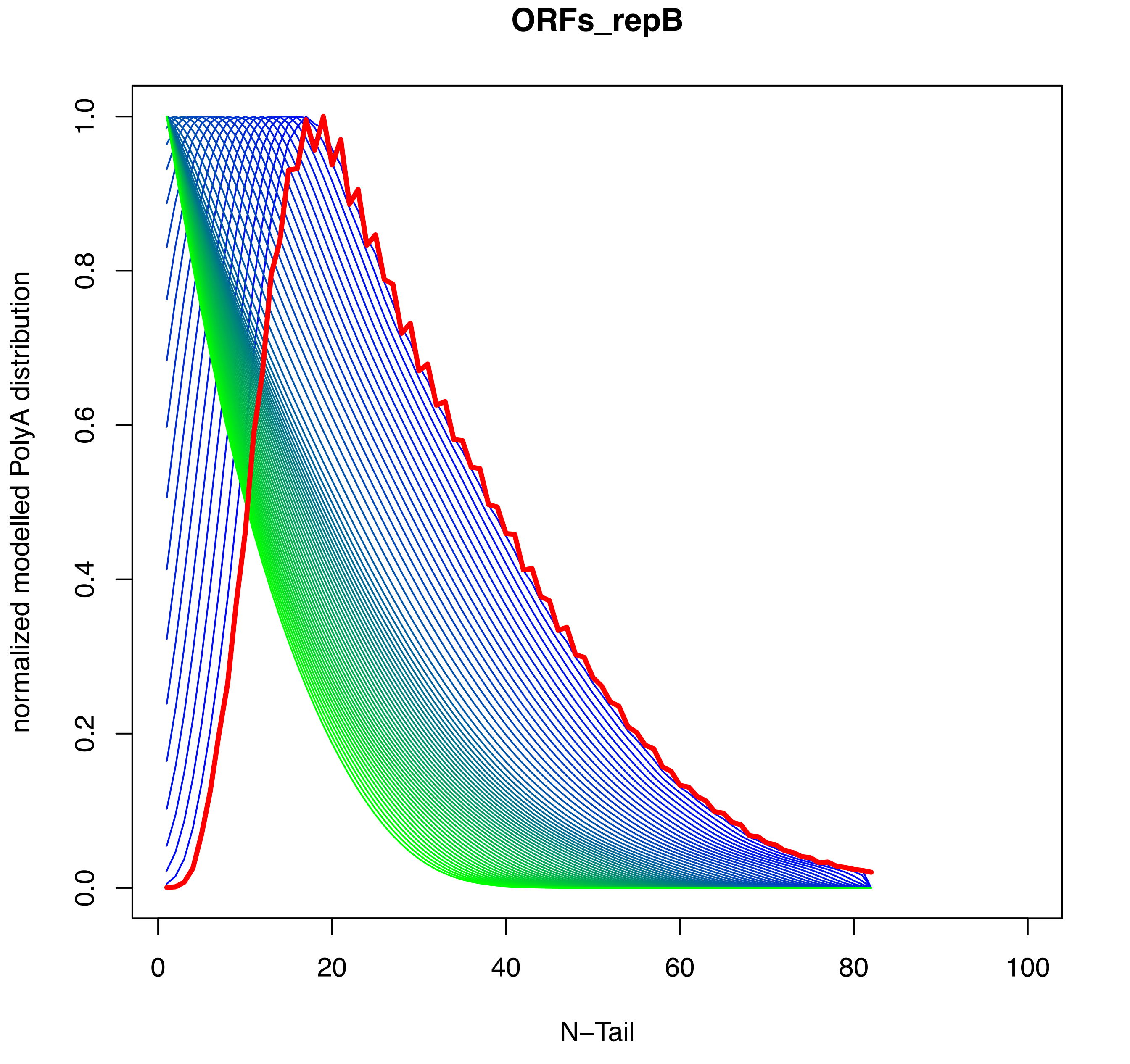

ORFs_repB
normalized modelled PolyA distribution
N−Tail
0.0
0.2
0.4
0.6
0.8
1.0
0
20
40
60
80
100

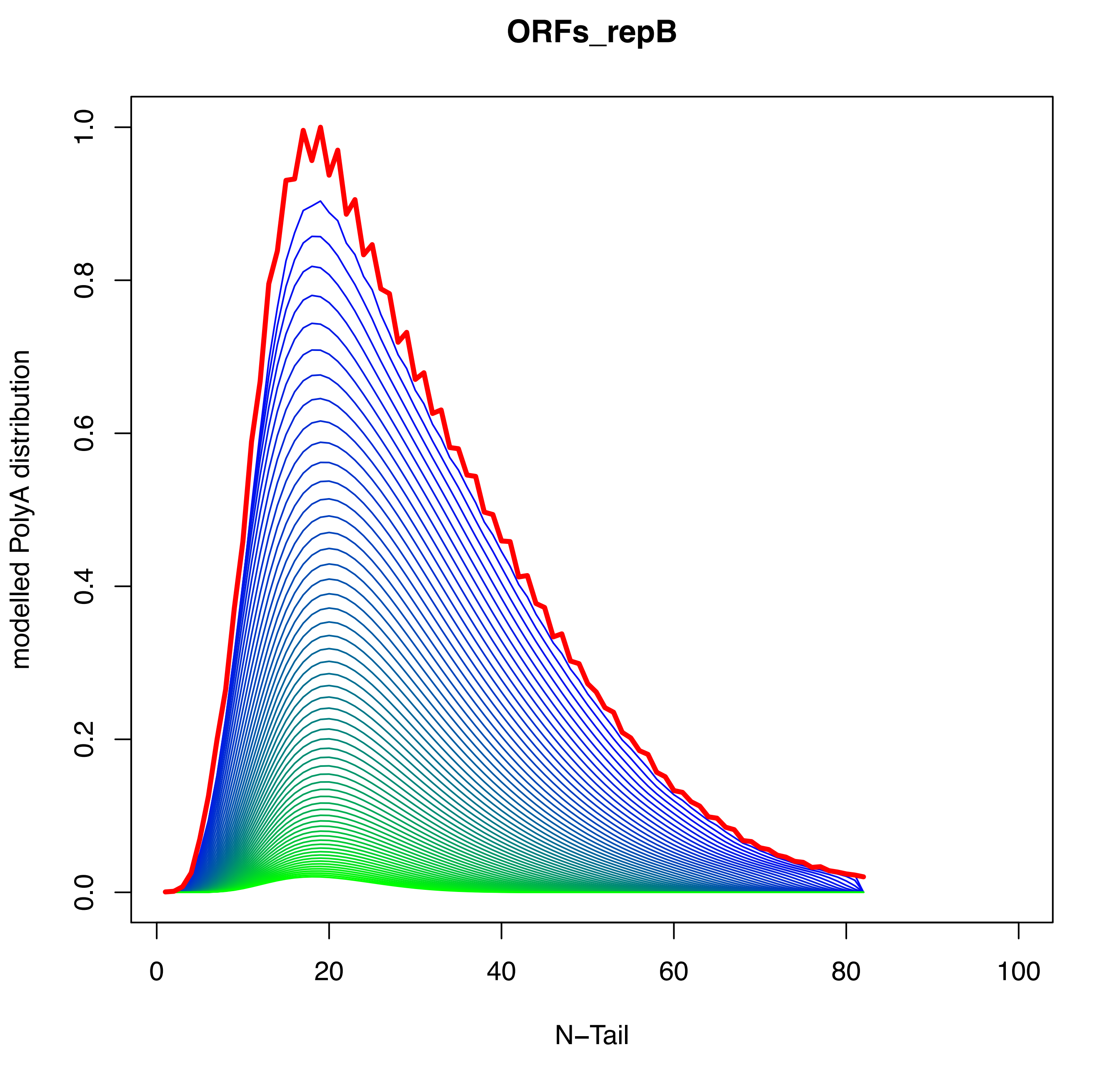
ORFs_repB
modelled PolyA distribution
N−Tail
0.0
0.2
0.4
0.6
0.8
1.0
0
20
40
60
80
100

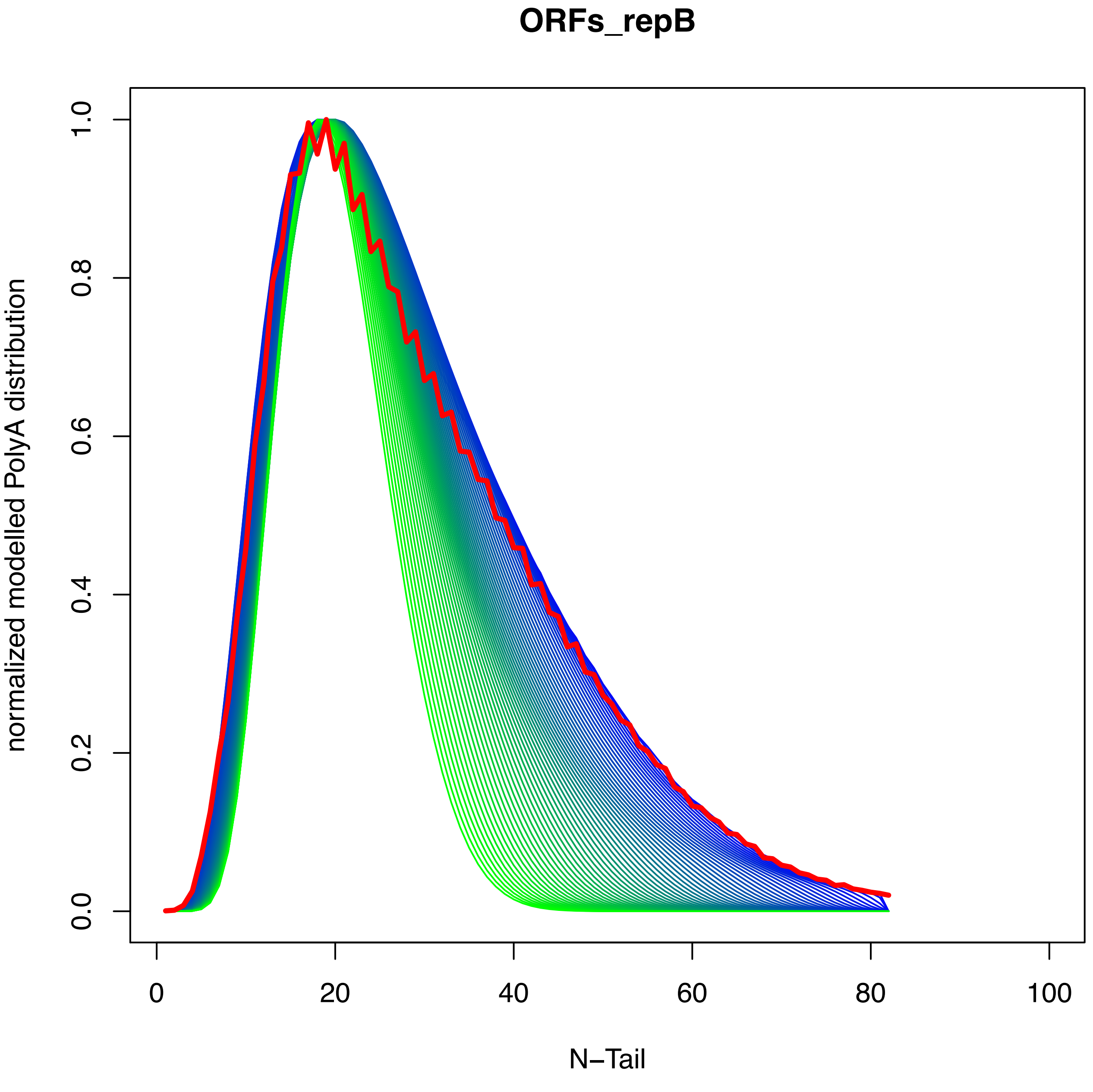

ORFs_repB
normalized modelled PolyA distribution
N−Tail
0.0
0.2
0.4
0.6
0.8
1.0
0
20
40
60
80
100

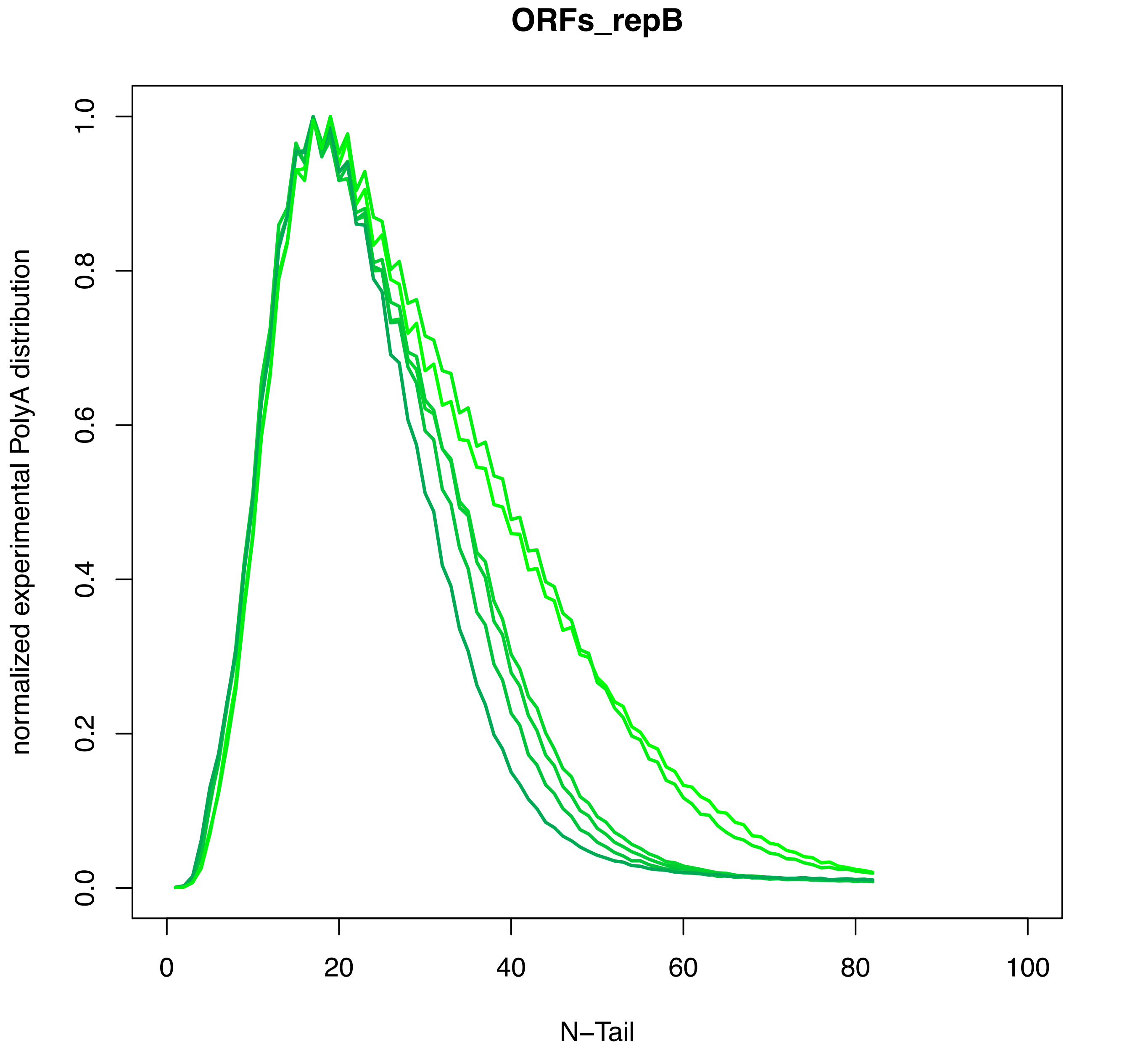

ORFs_repB
normalized experimental PolyA distribution
N−Tail
0.0
0.2
0.4
0.6
0.8
1.0
0
20
40
60
80
100

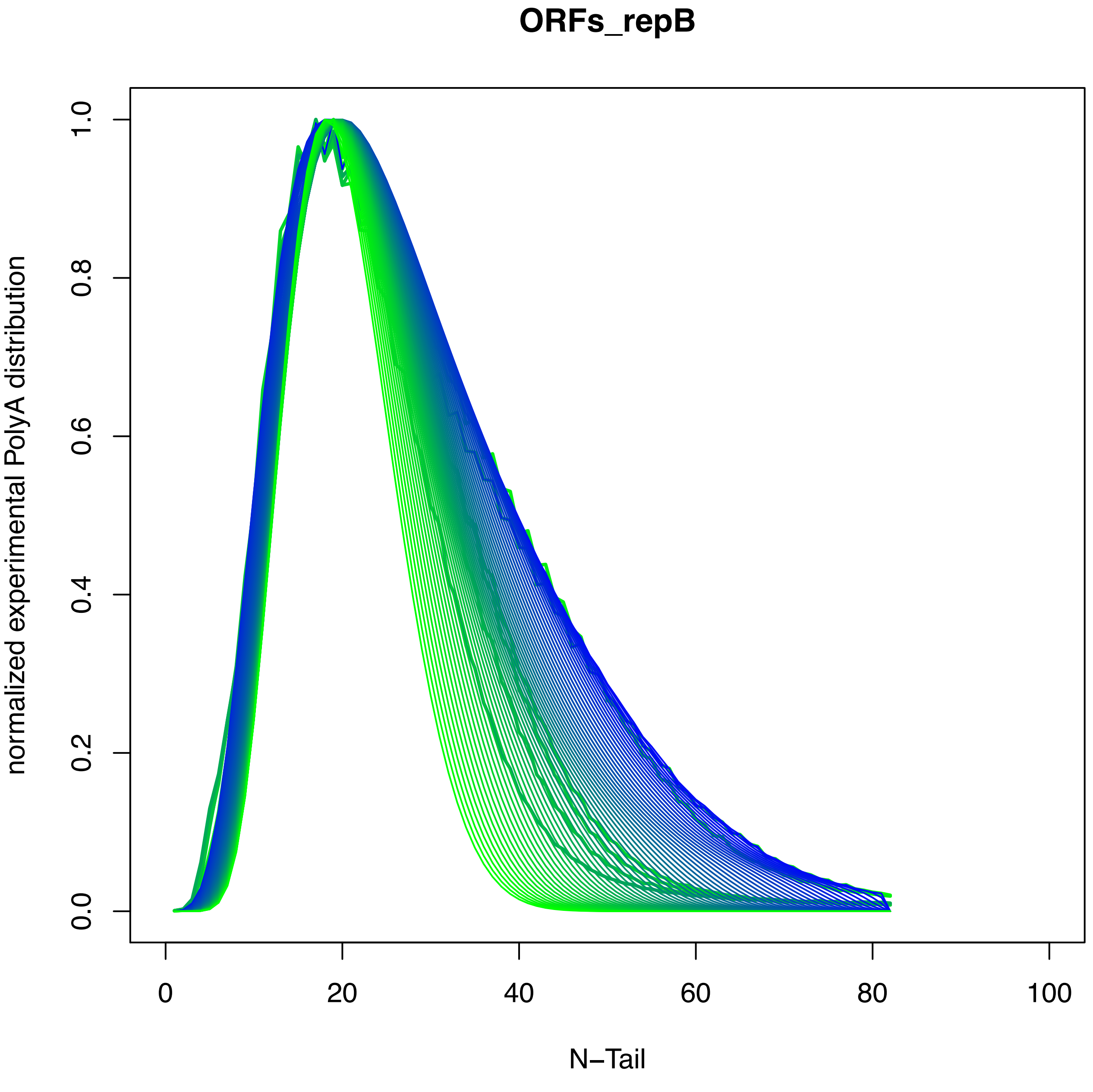

ORFs_repB
normalized experimental PolyA distribution
N−Tail
0.0
0.2
0.4
0.6
0.8
1.0
0
20
40
60
80
100

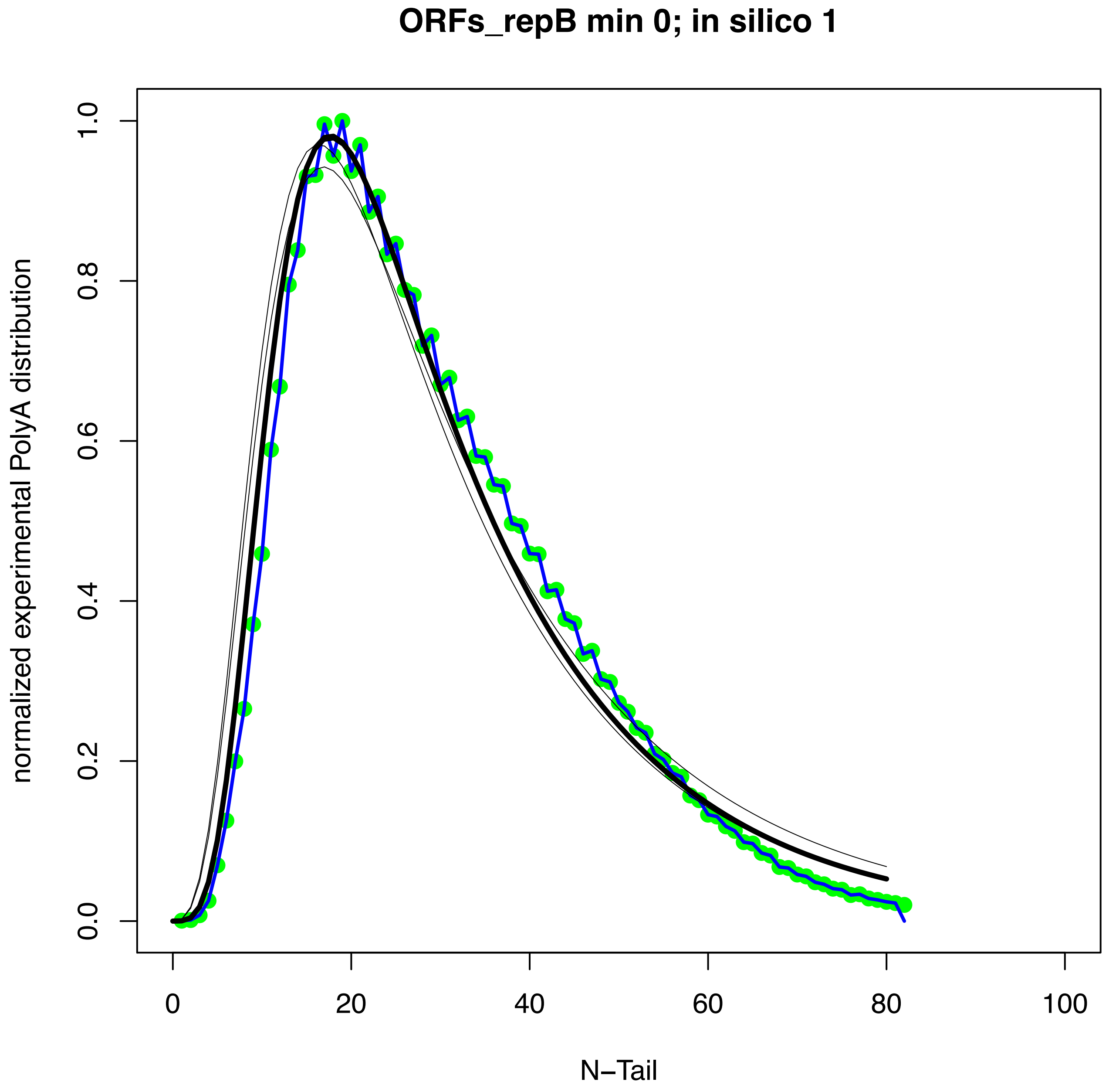
ORFs_repB min 0; in silico 1
normalized experimental PolyA distribution
0.0
0.2
0.4
0.6
0.8
1.0
0
20
40
60
80
100
N−Tail

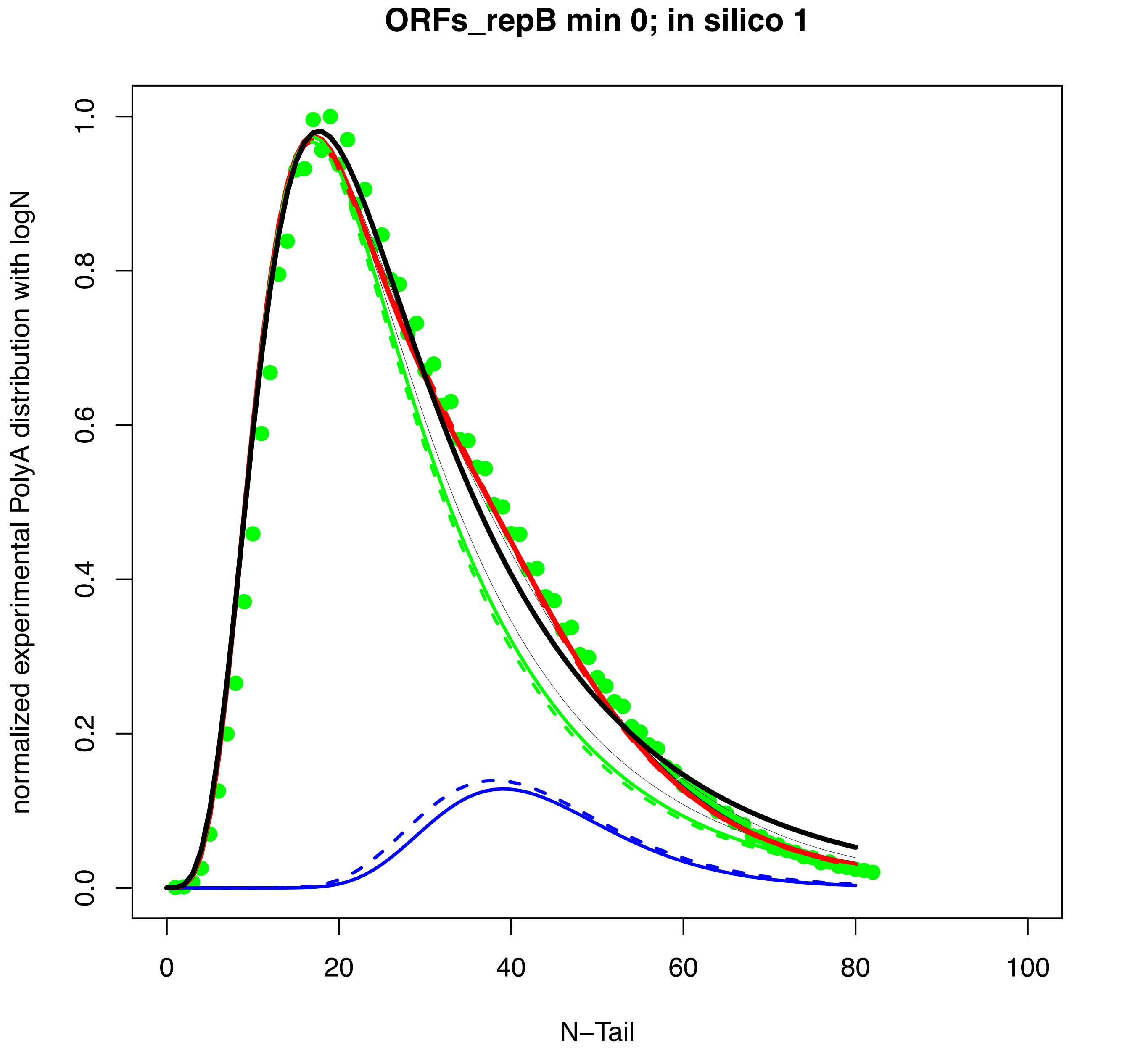

ORFs_repB min 0; in silico 1
normalized experimental PolyA distribution with logN
N−Tail
0.0
0.2
0.4
0.6
0.8
1.0
0
20
40
60
80
100

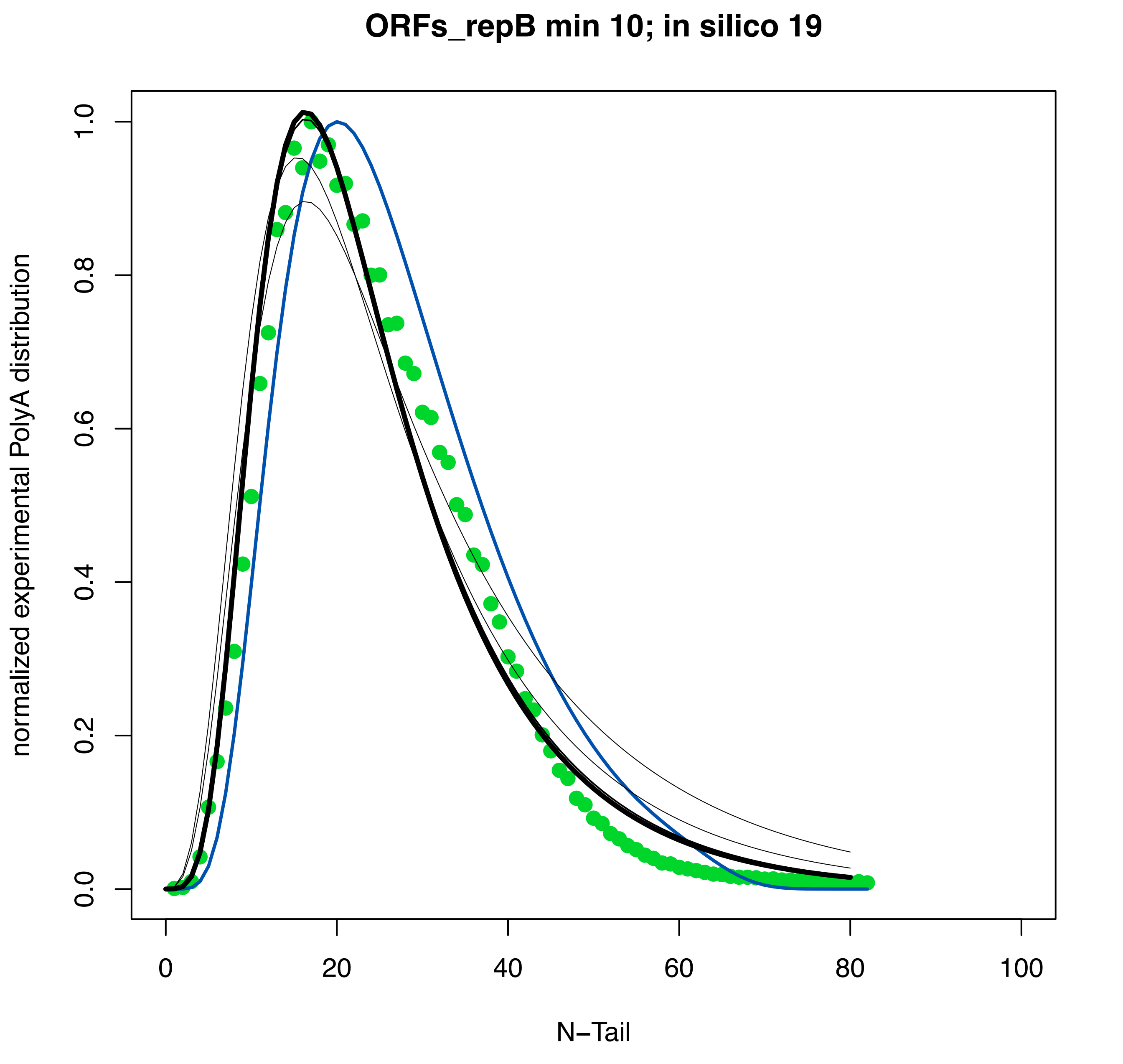

ORFs_repB min 10; in silico 19
normalized experimental PolyA distribution
0.0
0.2
0.4
0.6
0.8
1.0
0
20
40
60
80
100
N−Tail

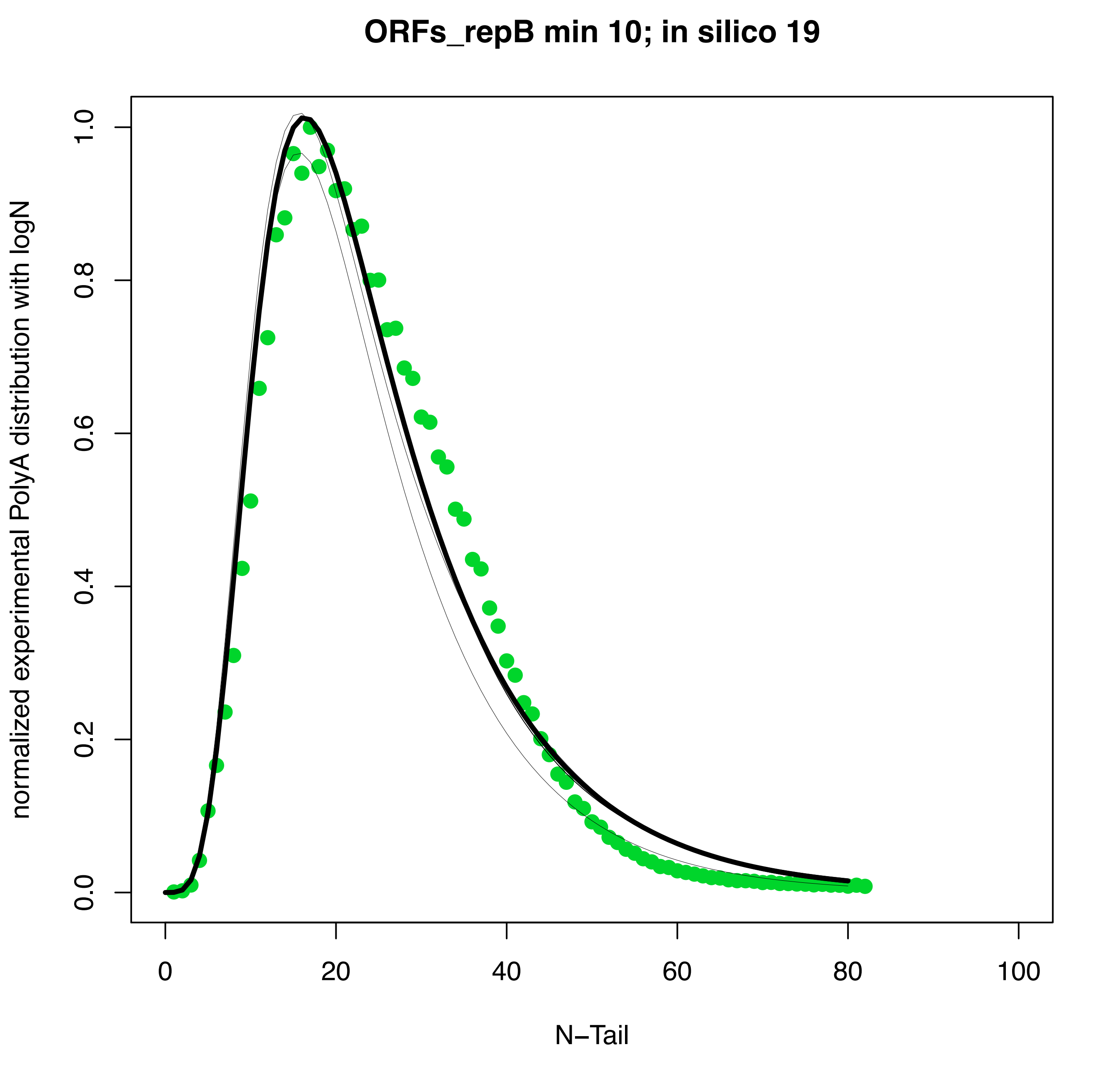
ORFs_repB min 10; in silico 19
normalized experimental PolyA distribution with logN
N−Tail
0.0
0.2
0.4
0.6
0.8
1.0
0
20
40
60
80
100

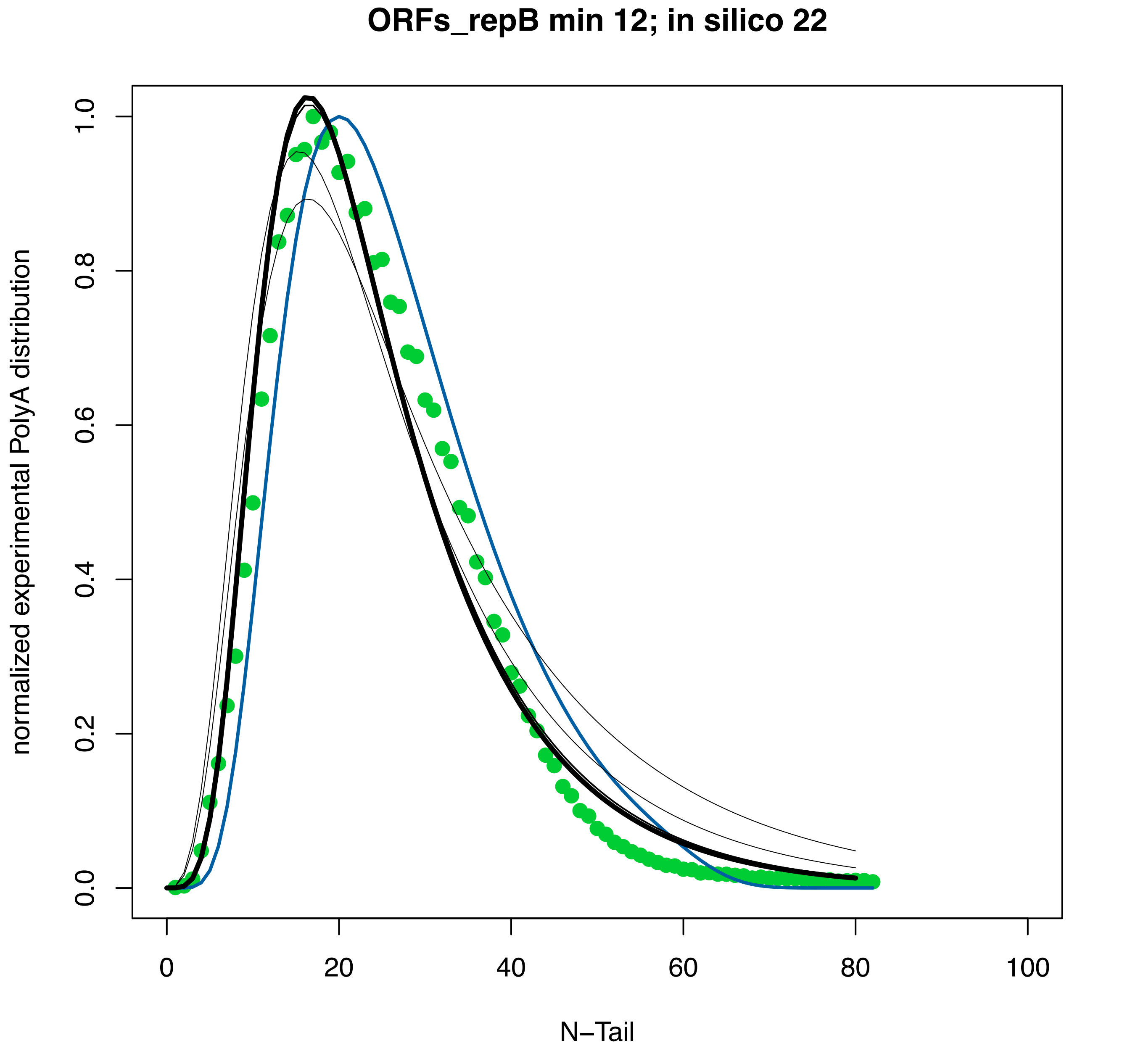

ORFs_repB min 12; in silico 22
normalized experimental PolyA distribution
N−Tail
0.0
0.2
0.4
0.6
0.8
1.0
0
20
40
60
80
100

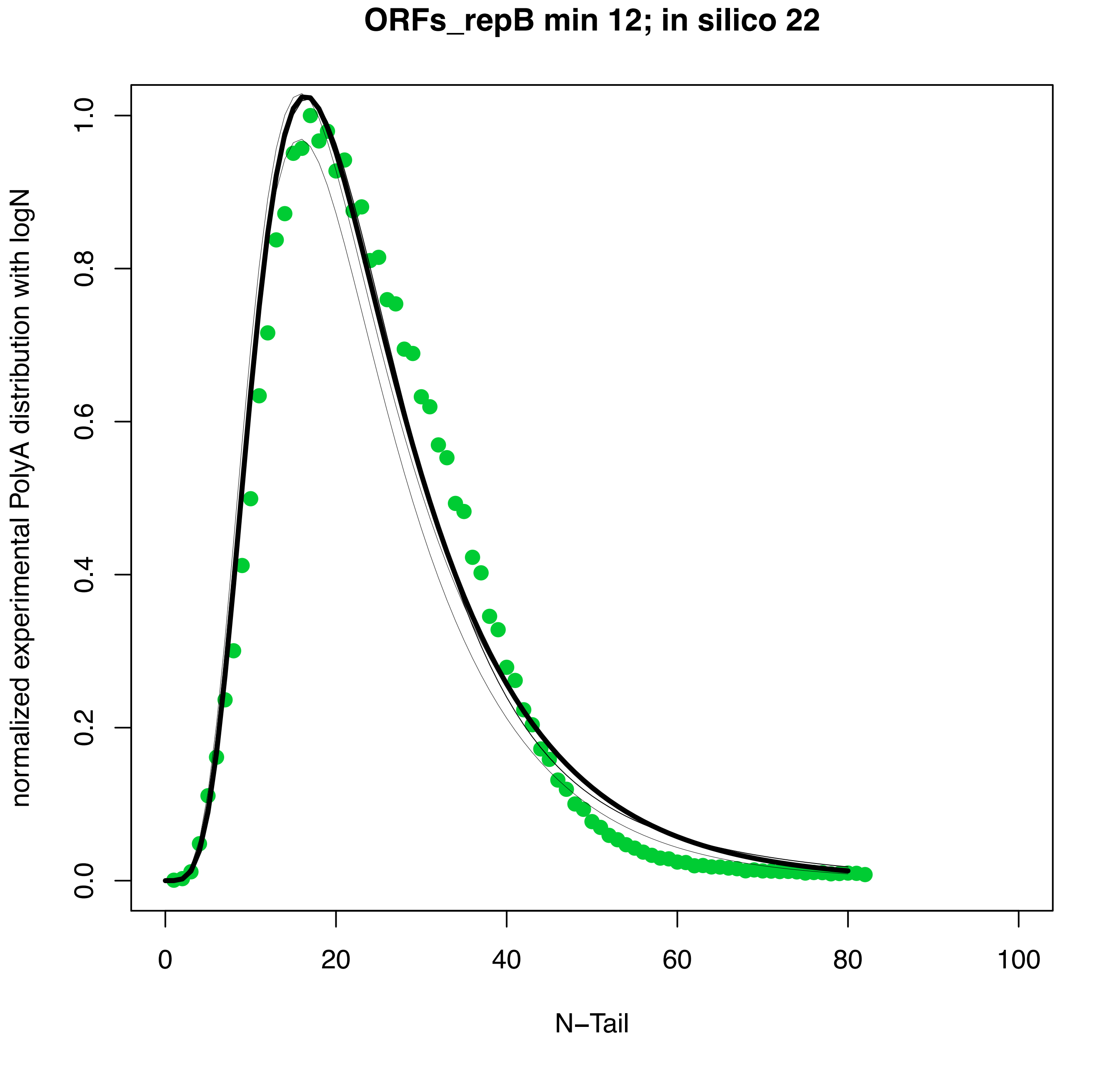

ORFs_repB min 12; in silico 22
normalized experimental PolyA distribution with logN
N−Tail
0.0
0.2
0.4
0.6
0.8
1.0
0
20
40
60
80
100

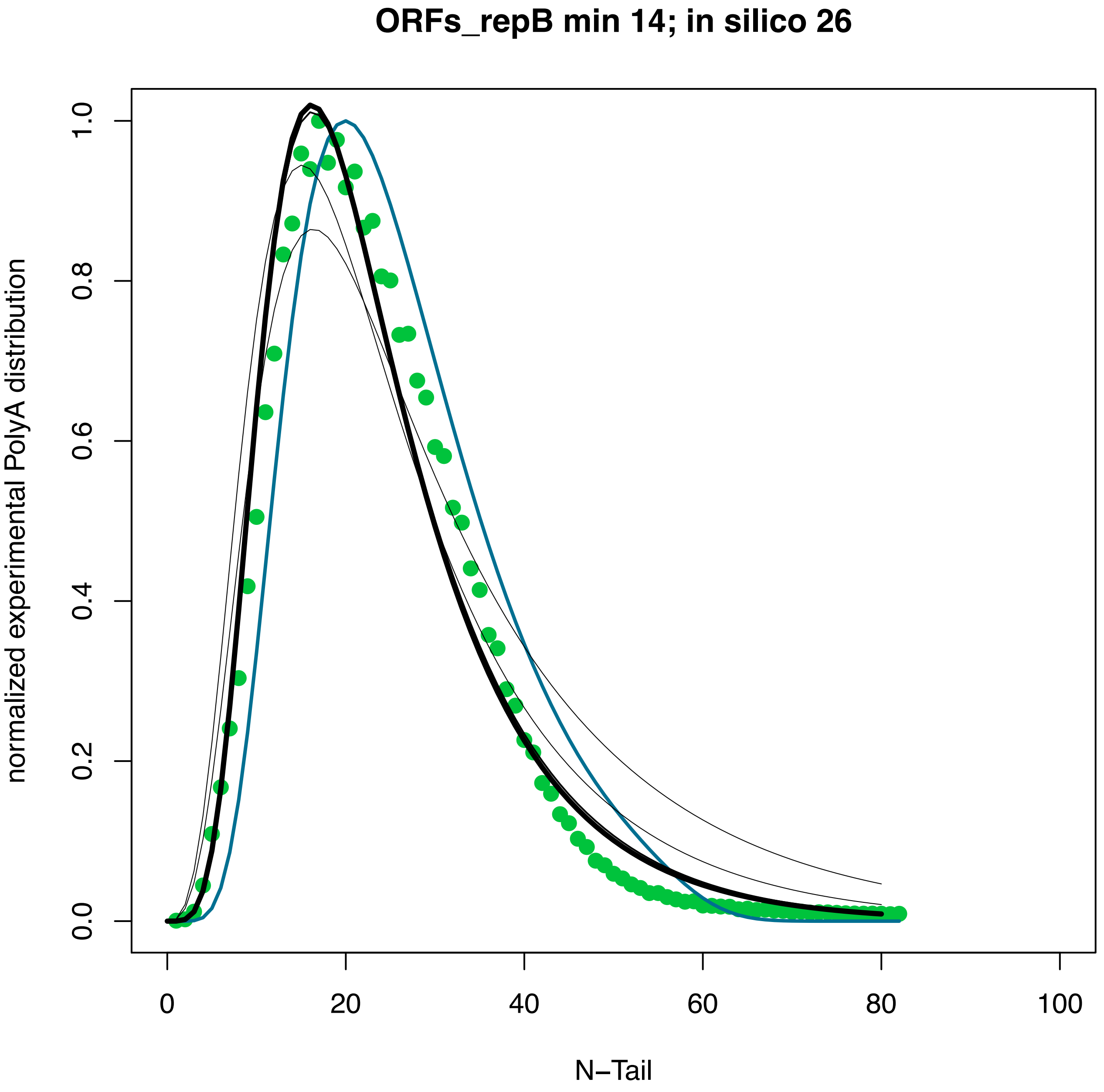

ORFs_repB min 14; in silico 26
normalized experimental PolyA distribution
N−Tail
0.0
0.2
0.4
0.6
0.8
1.0
0
20
40
60
80
100

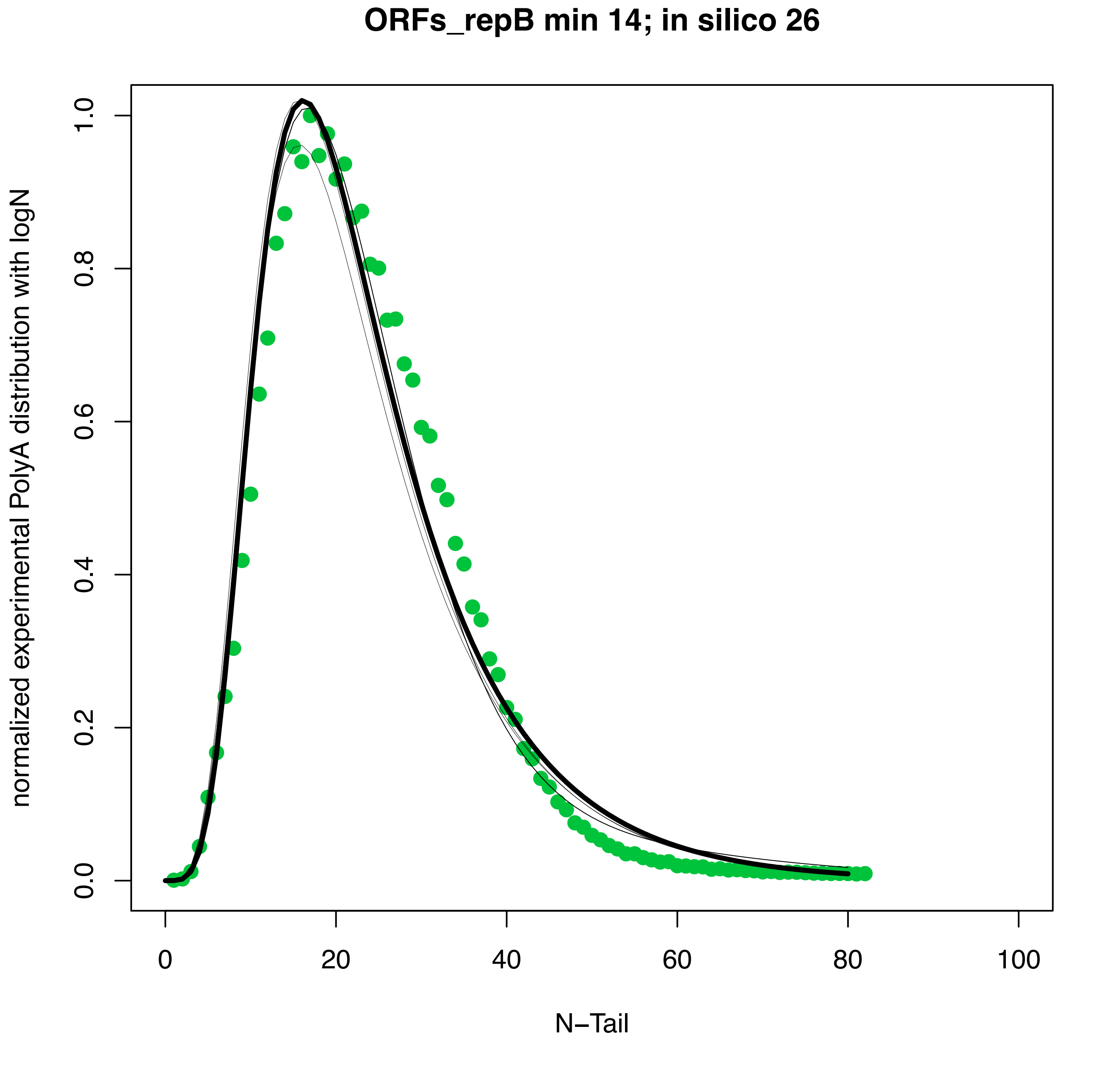

ORFs_repB min 14; in silico 26
normalized experimental PolyA distribution with logN
N−Tail
0.0
0.2
0.4
0.6
0.8
1.0
0
20
40
60
80
100

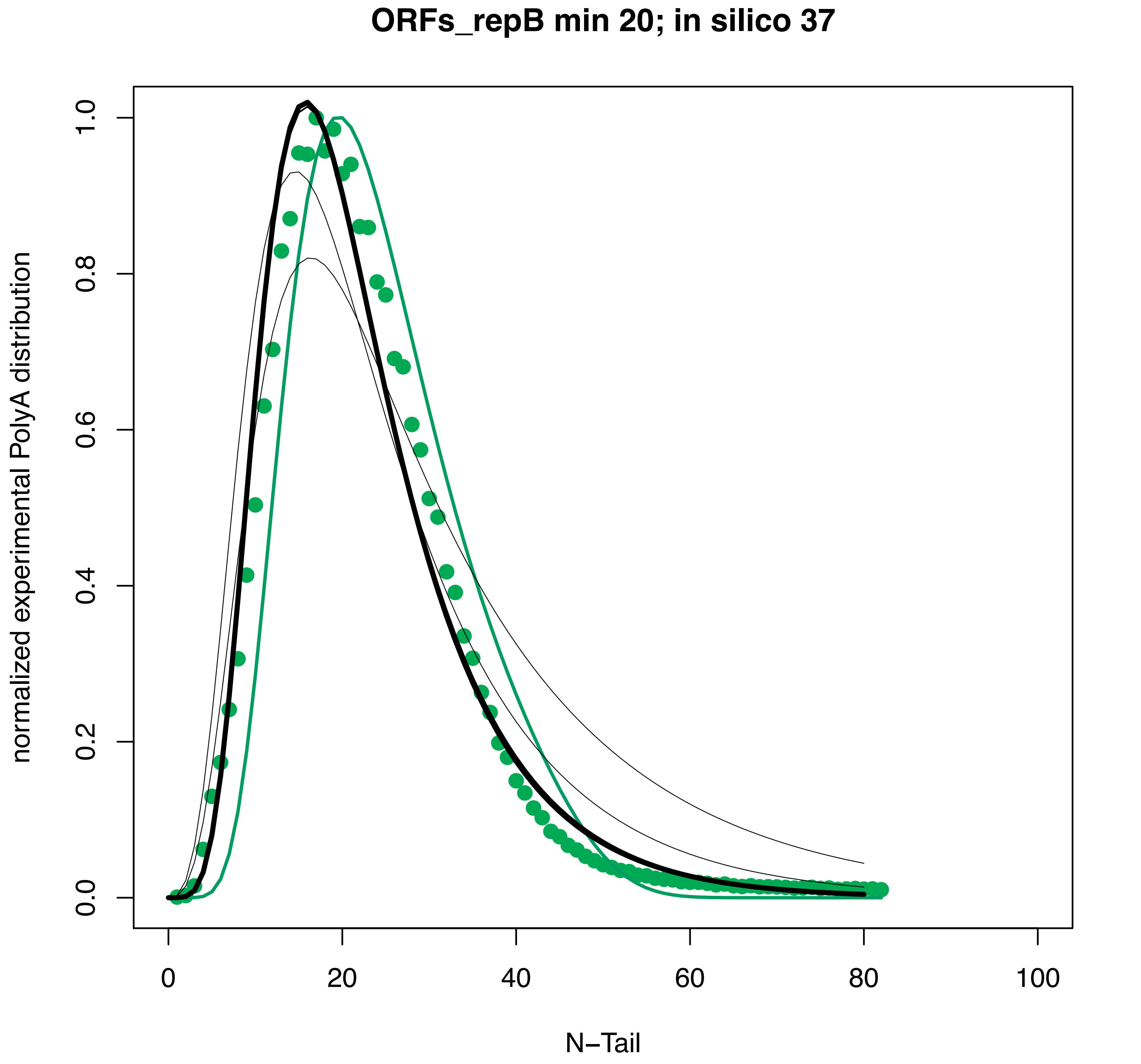
ORFs_repB min 20; in silico 37
normalized experimental PolyA distribution
N−Tail
0.0
0.2
0.4
0.6
0.8
1.0
0
20
40
60
80
100

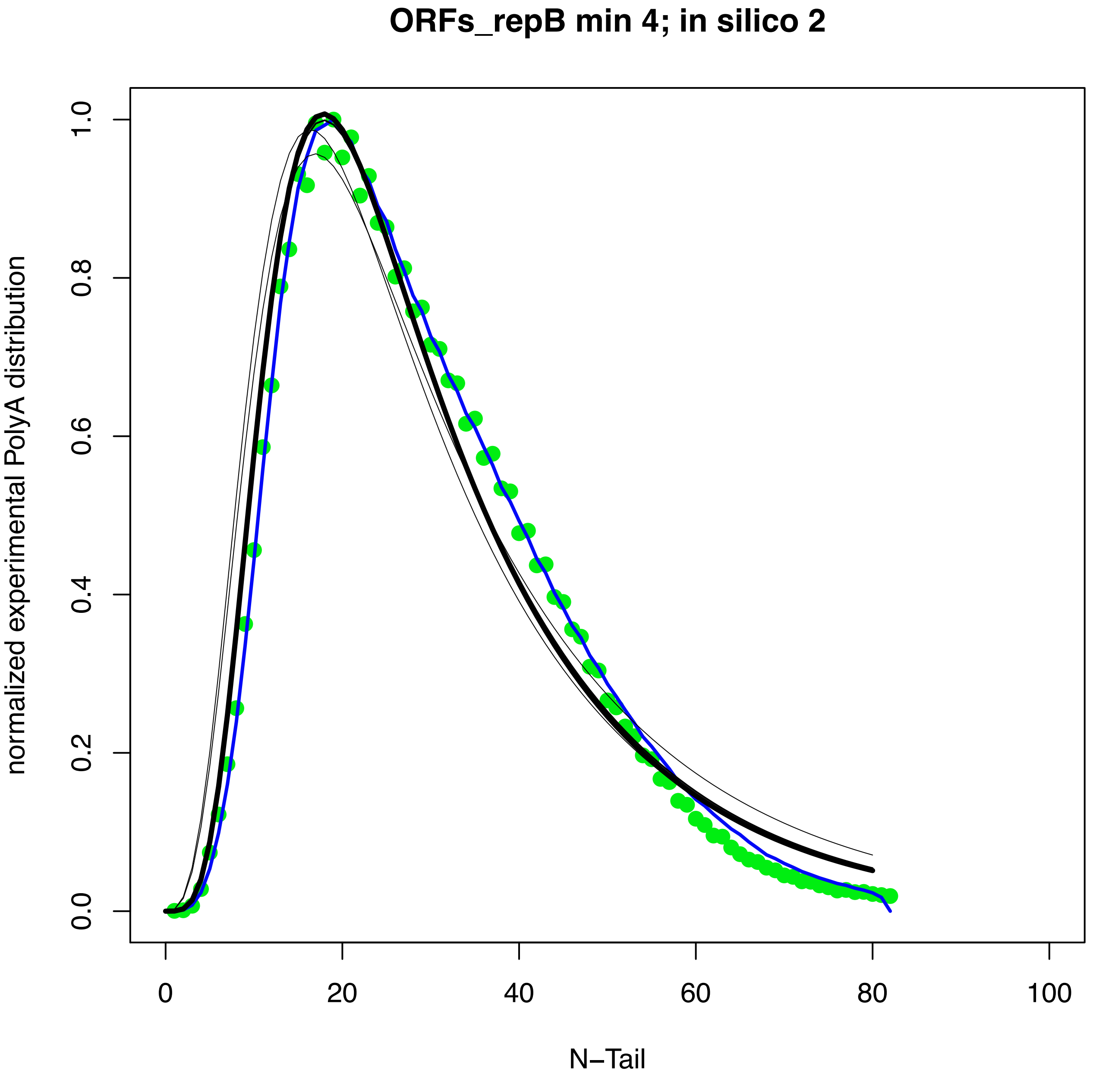
ORFs_repB min 4; in silico 2
normalized experimental PolyA distribution
N−Tail
0.0
0.2
0.4
0.6
0.8
1.0
0
20
40
60
80
100

ORFs_repB min 4; in silico 2

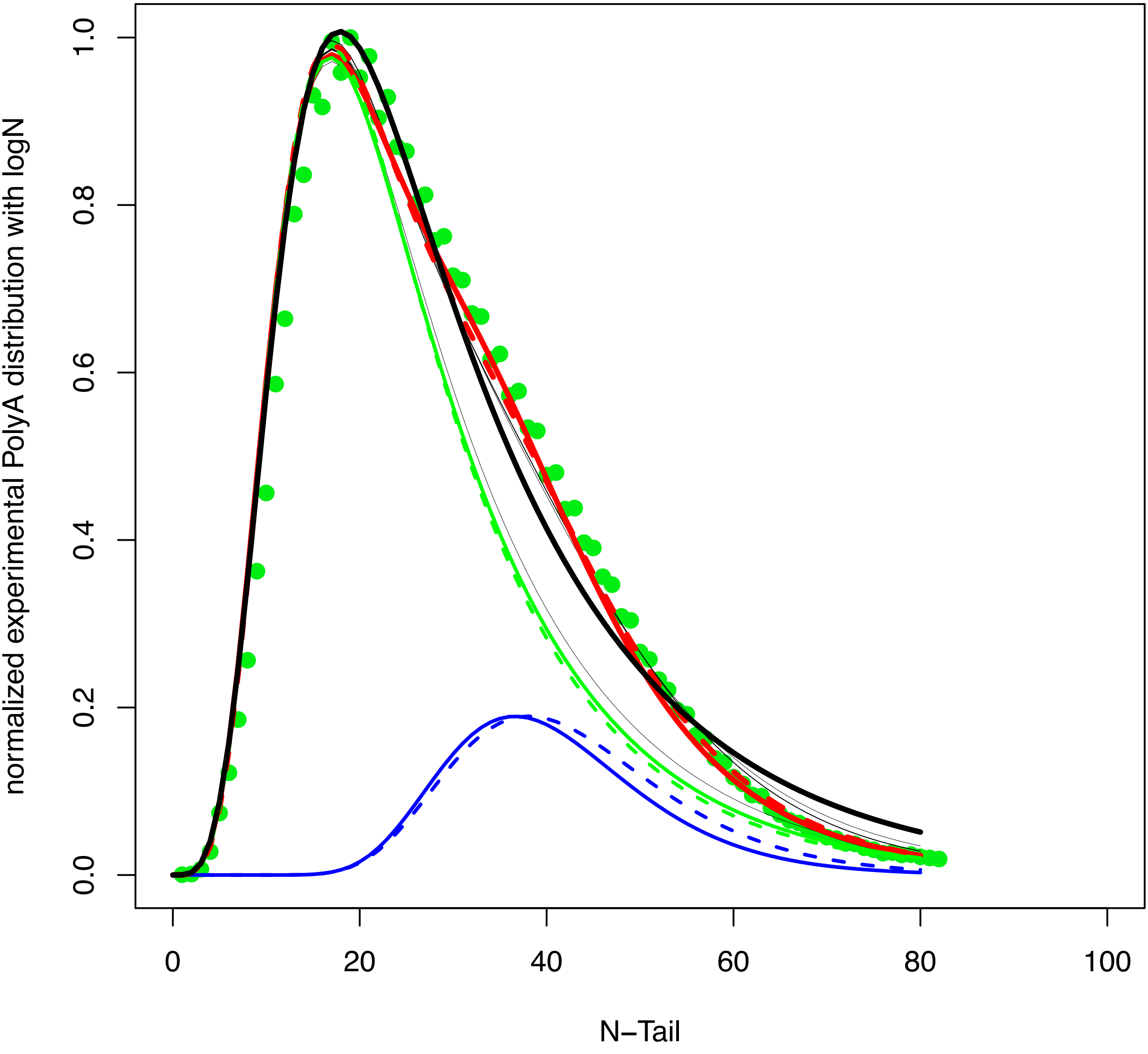

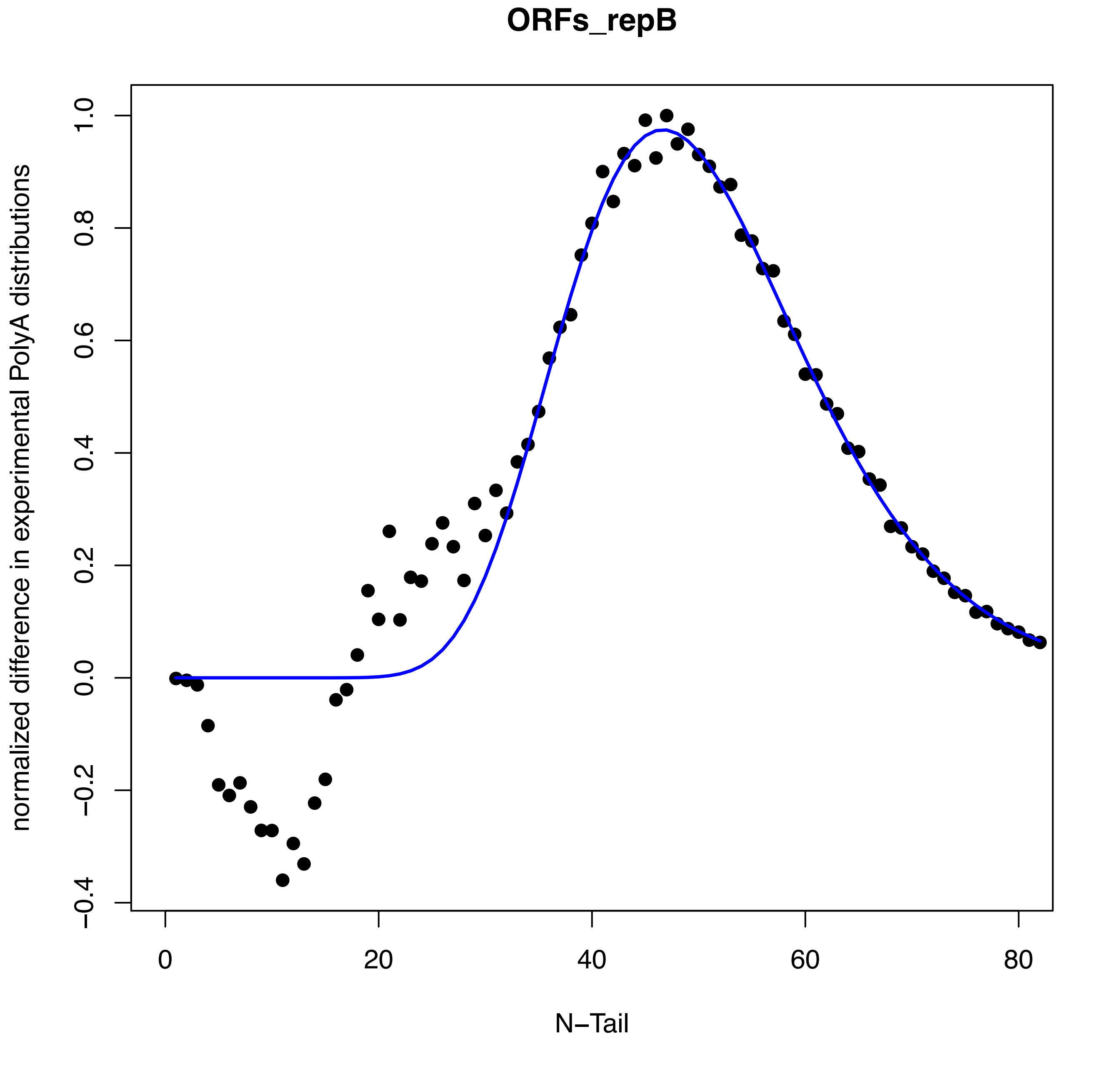

ORFs_repB
normalized difference in experimental PolyA distributions
−0.4
−0.2
0.0
0.2
0.4
0.6
0.8
1.0
0
20
40
60
80
N−Tail

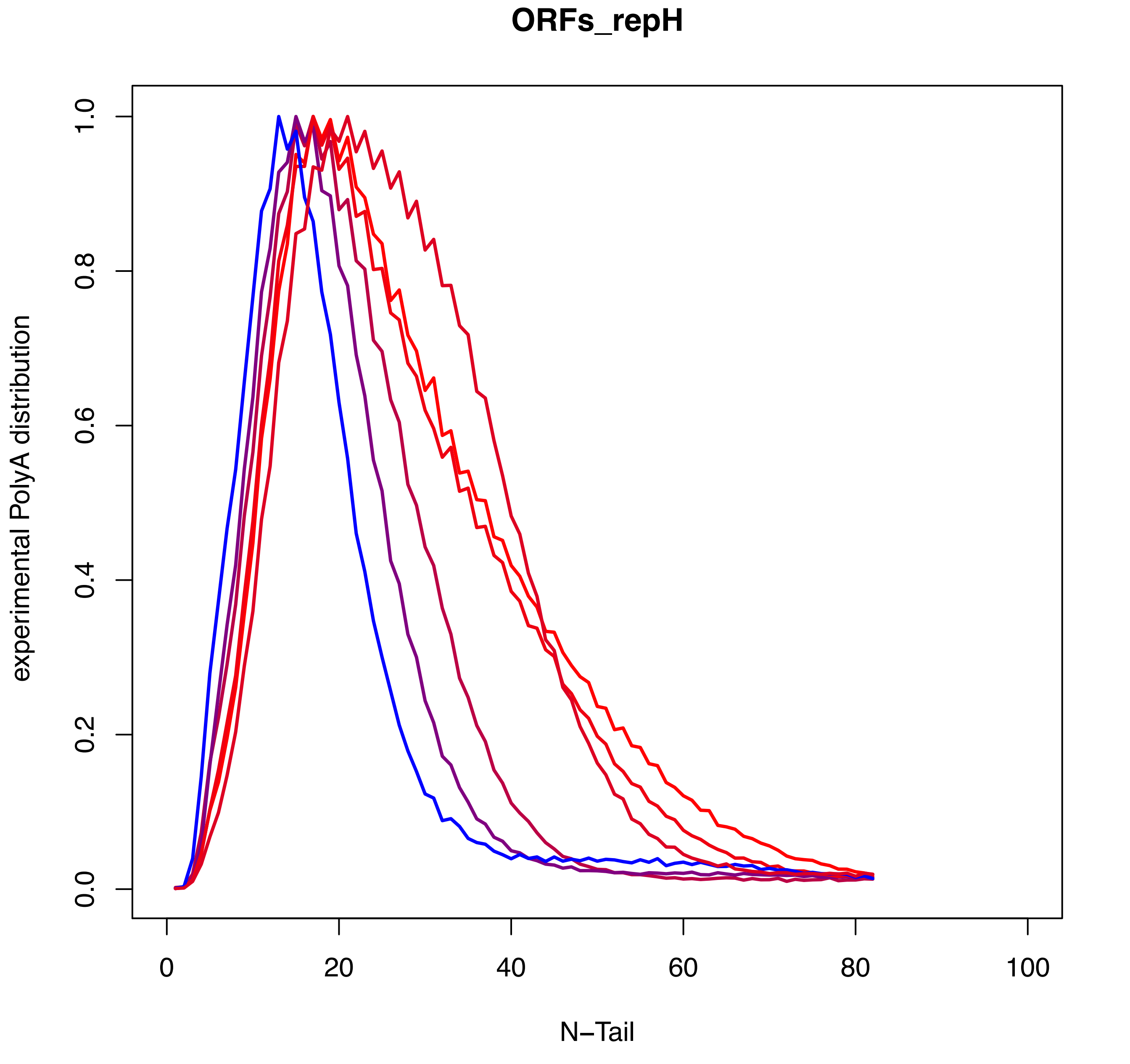
ORFs_repH
experimental PolyA distribution
N−Tail
0.0
0.2
0.4
0.6
0.8
1.0
0
20
40
60
80
100

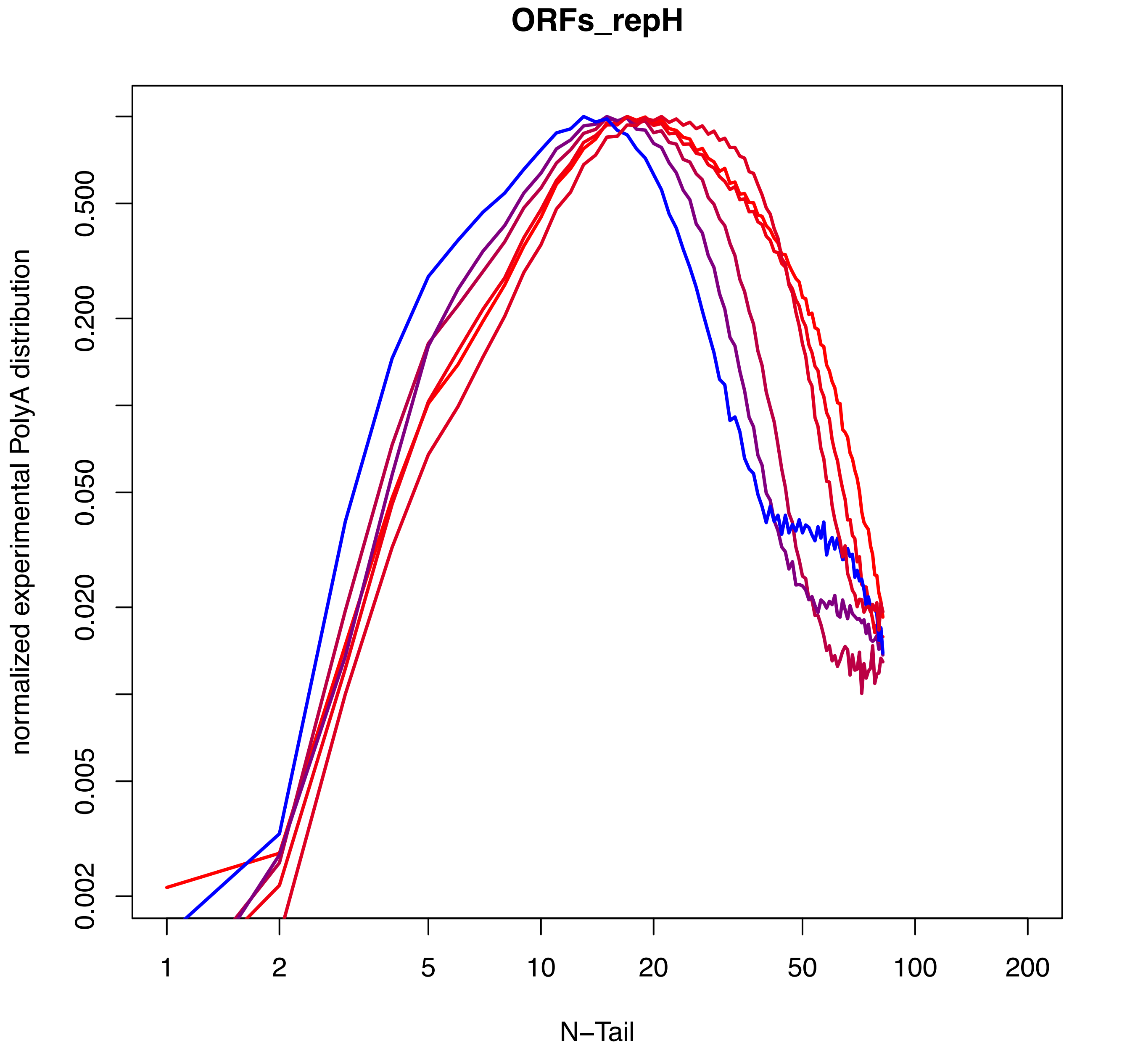

ORFs_repH
normalized experimental PolyA distribution
0.002
0.005
0.020
0.050
0.200
0.500
N−Tail
1
2
5
10
20
50
100
200

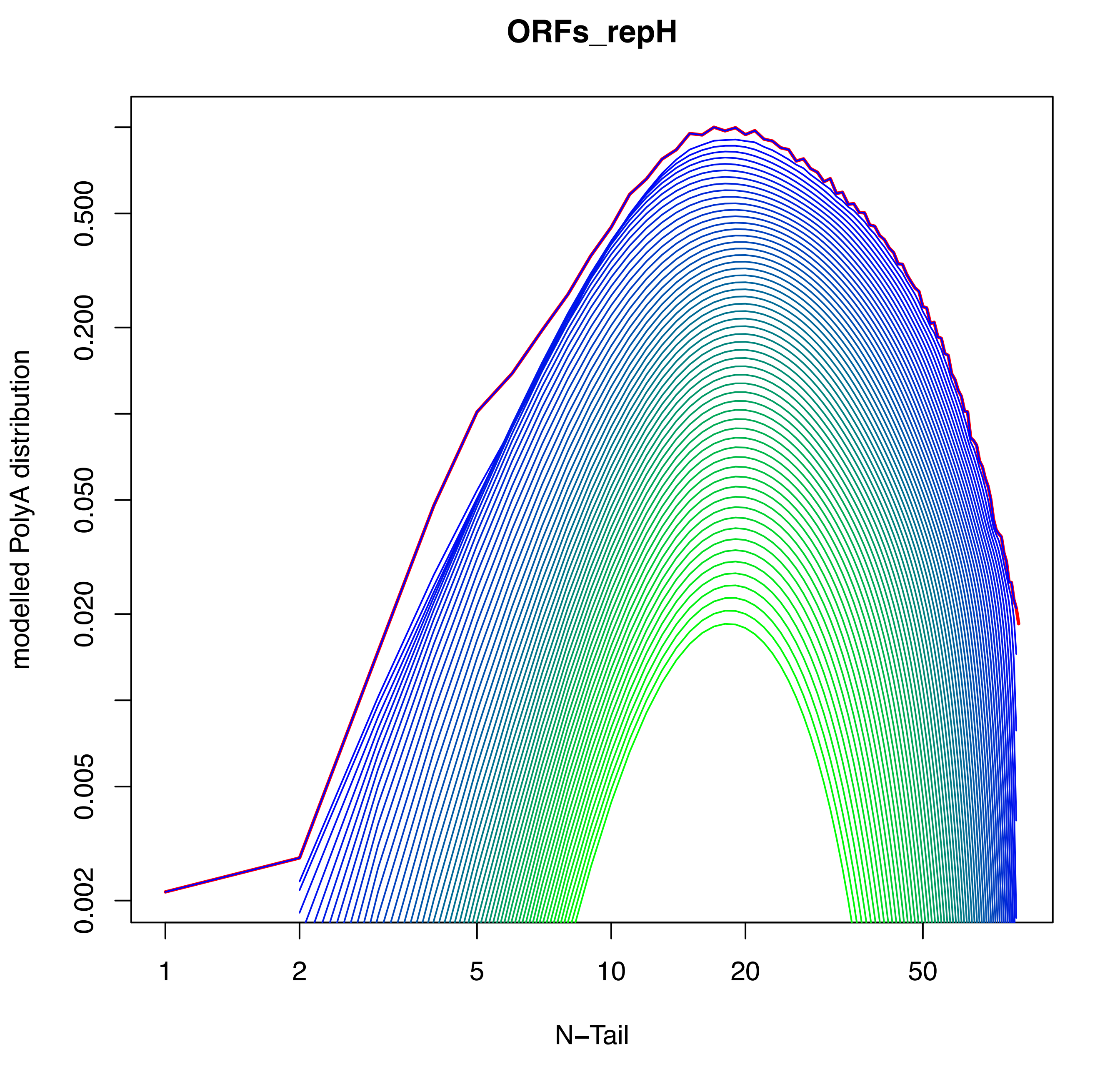

ORFs_repH
modelled PolyA distribution
0.002
0.005
0.020
0.050
0.200
0.500
1
2
5
10
20
50
N−Tail

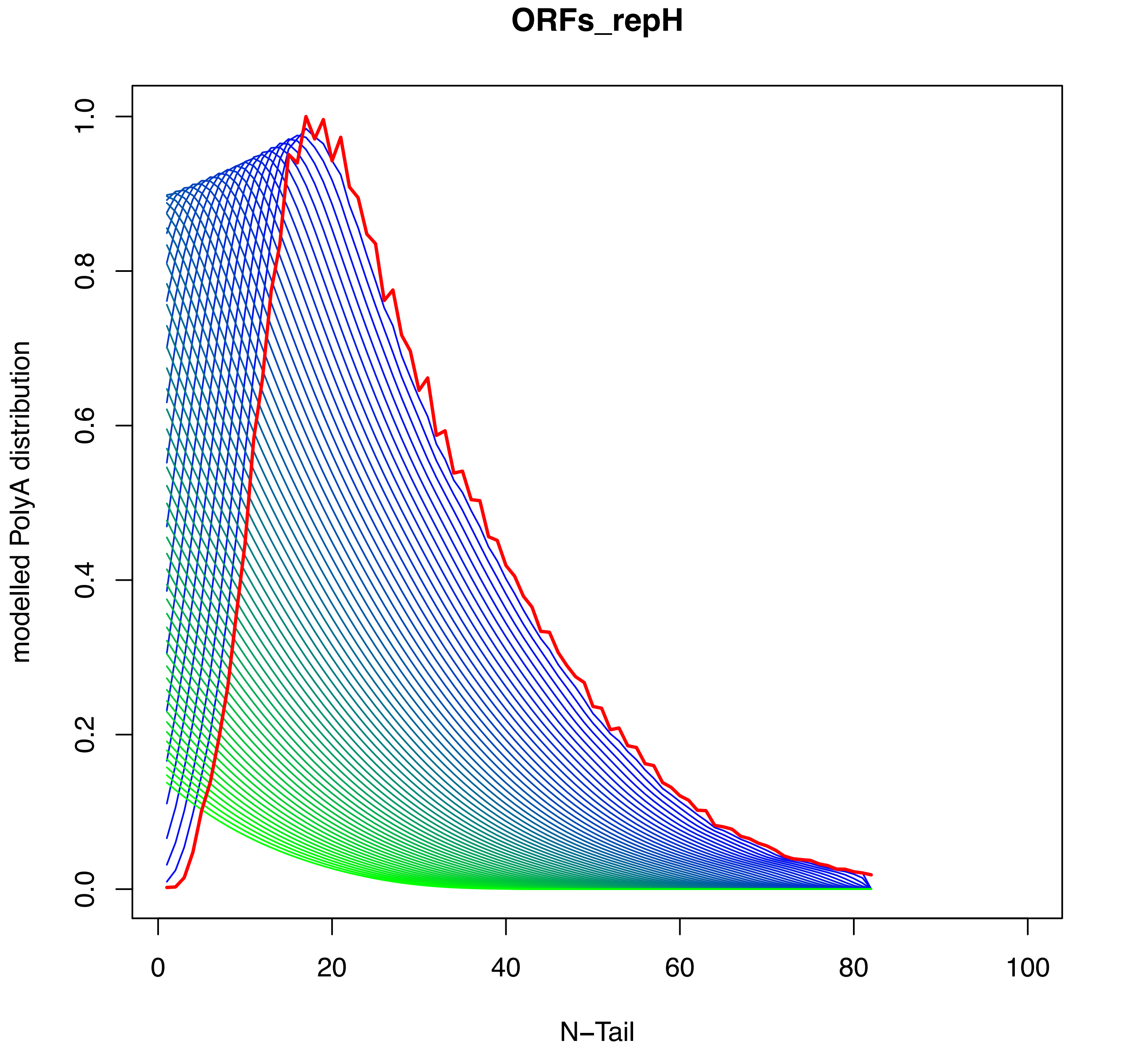

ORFs_repH
modelled PolyA distribution
N−Tail
0.0
0.2
0.4
0.6
0.8
1.0
0
20
40
60
80
100

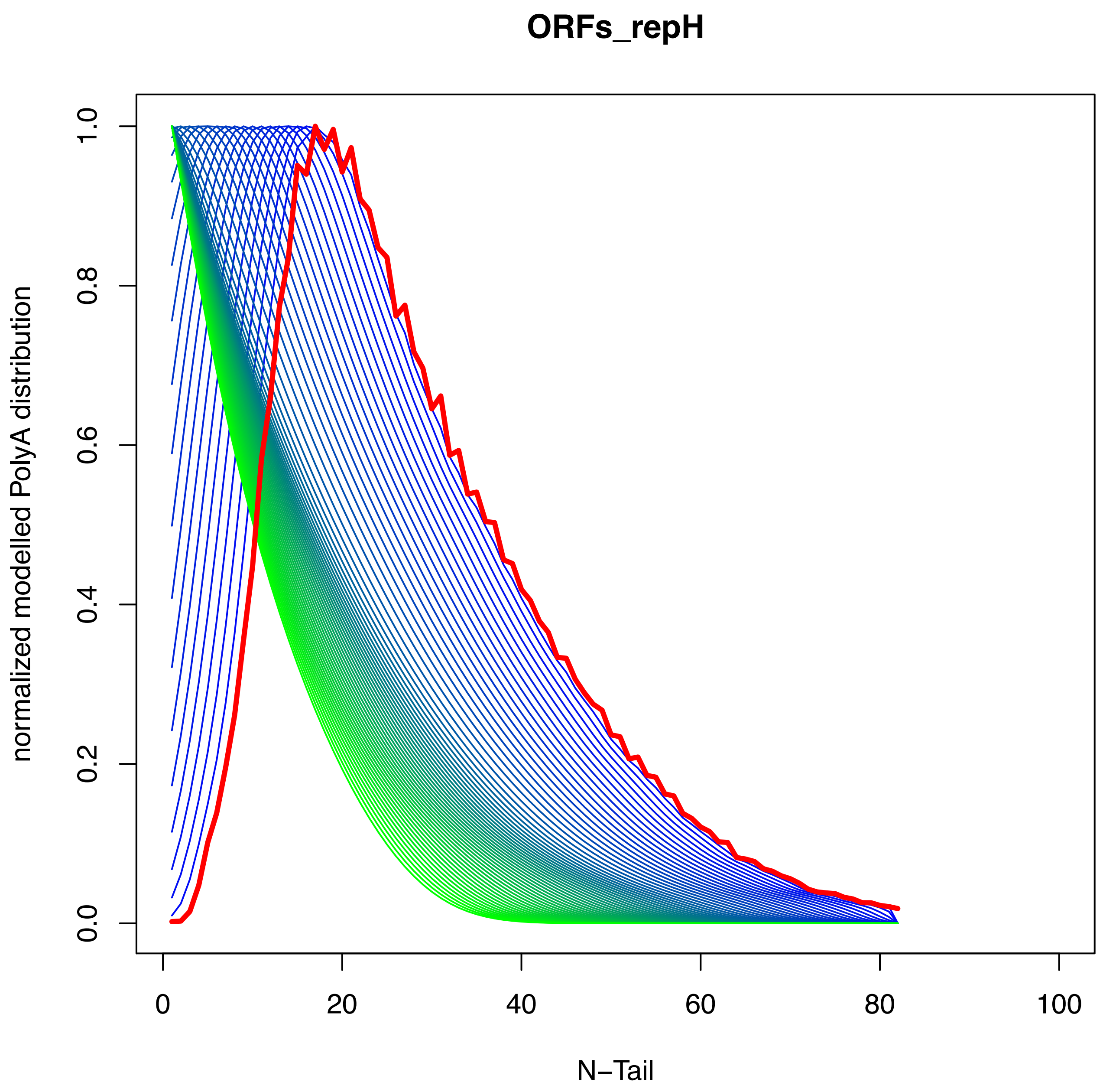

ORFs_repH
normalized modelled PolyA distribution
N−Tail
0.0
0.2
0.4
0.6
0.8
1.0
0
20
40
60
80
100

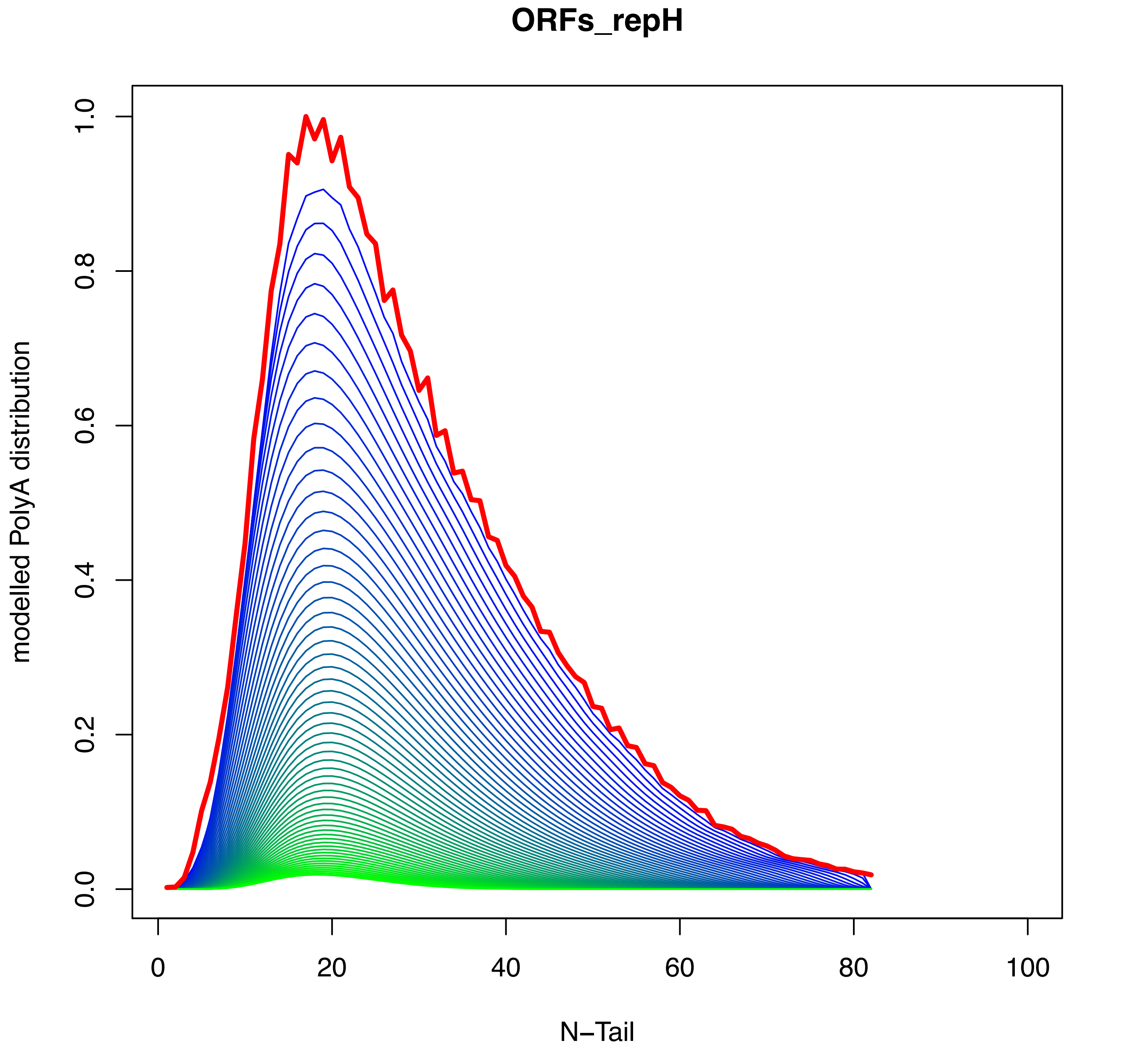

ORFs_repH
modelled PolyA distribution
N−Tail
0.0
0.2
0.4
0.6
0.8
1.0
0
20
40
60
80
100

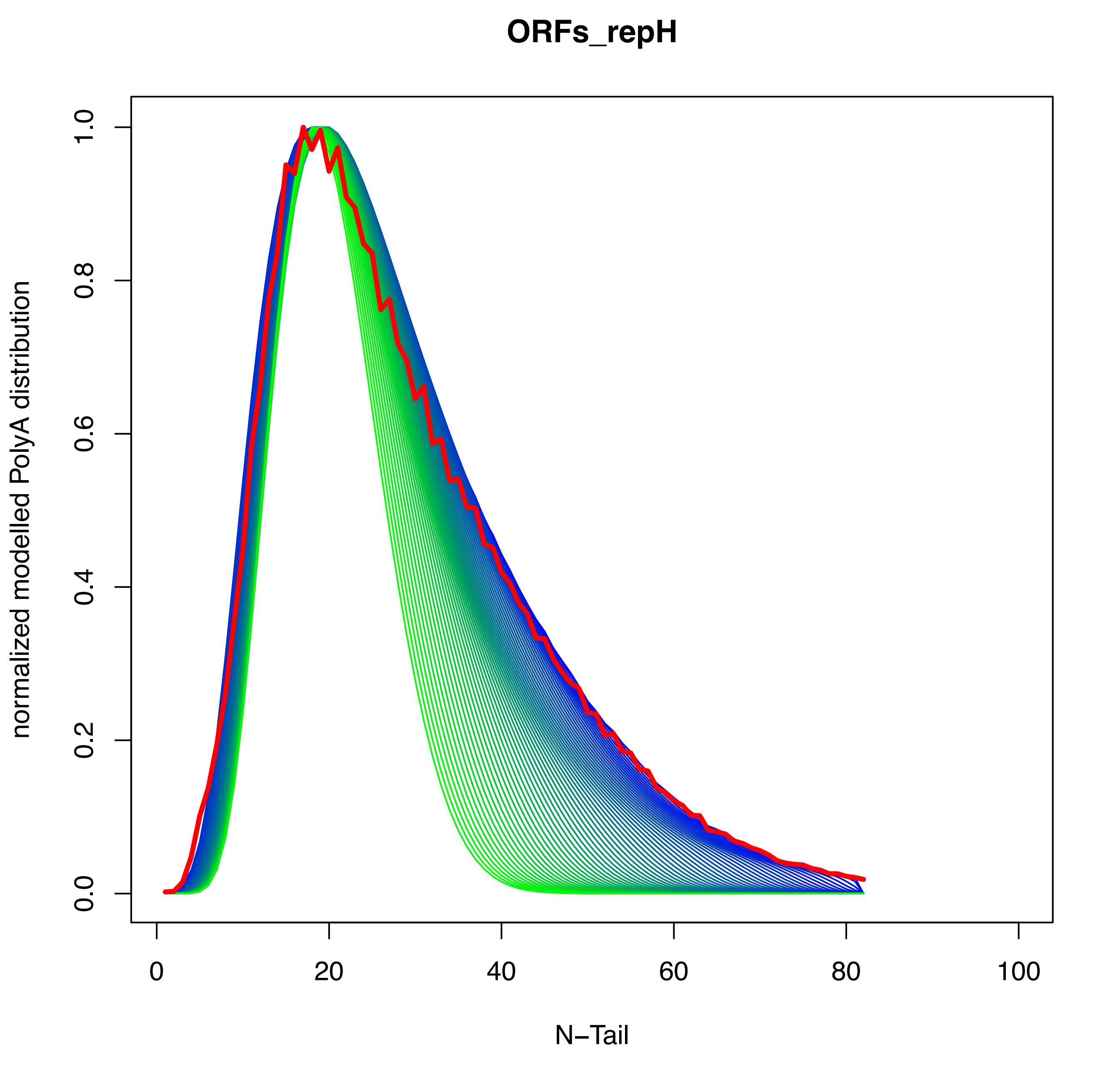

ORFs_repH
normalized modelled PolyA distribution
N−Tail
0.0
0.2
0.4
0.6
0.8
1.0
0
20
40
60
80
100

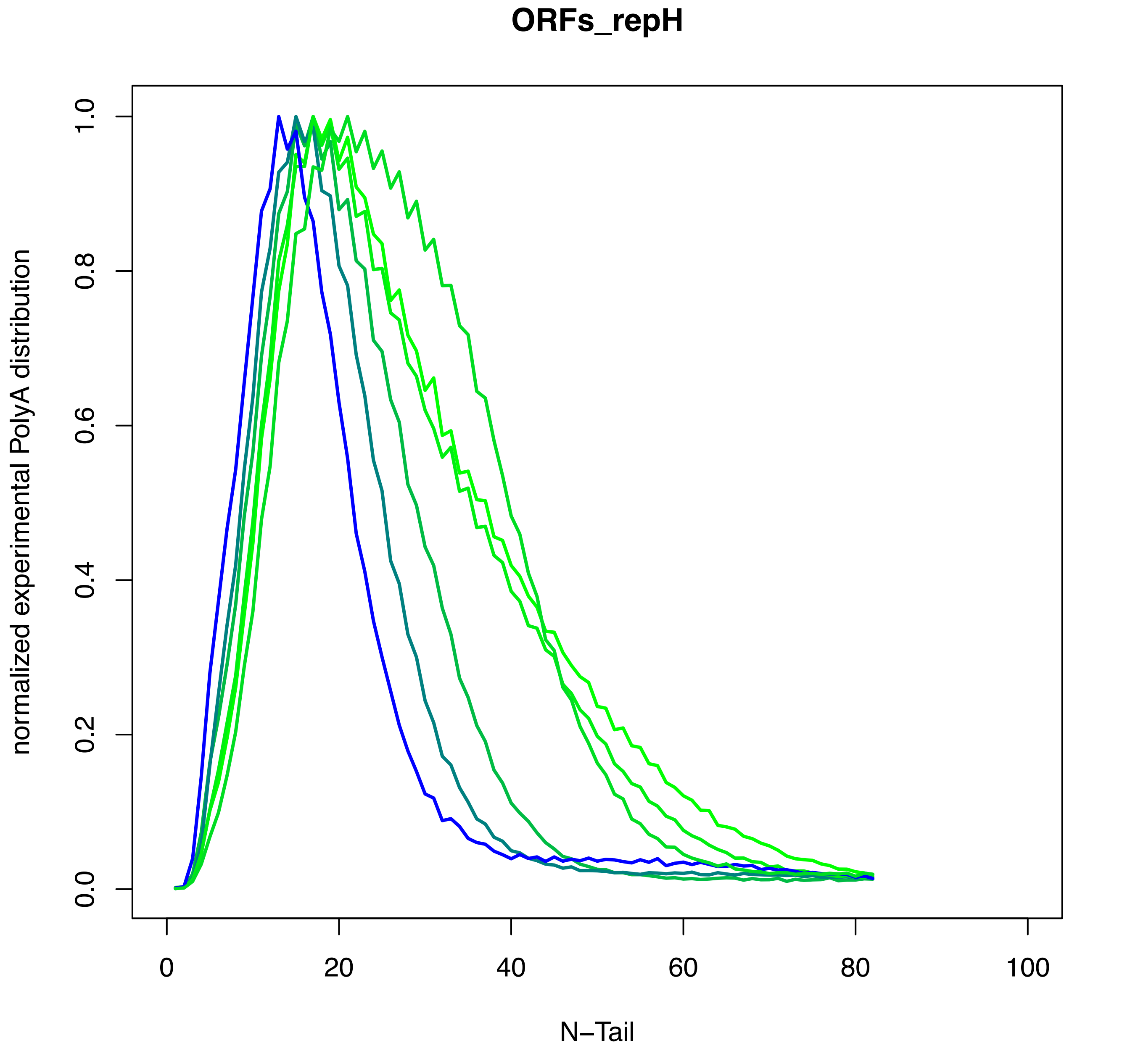
ORFs_repH
normalized experimental PolyA distribution
N−Tail
0.0
0.2
0.4
0.6
0.8
1.0
0
20
40
60
80
100

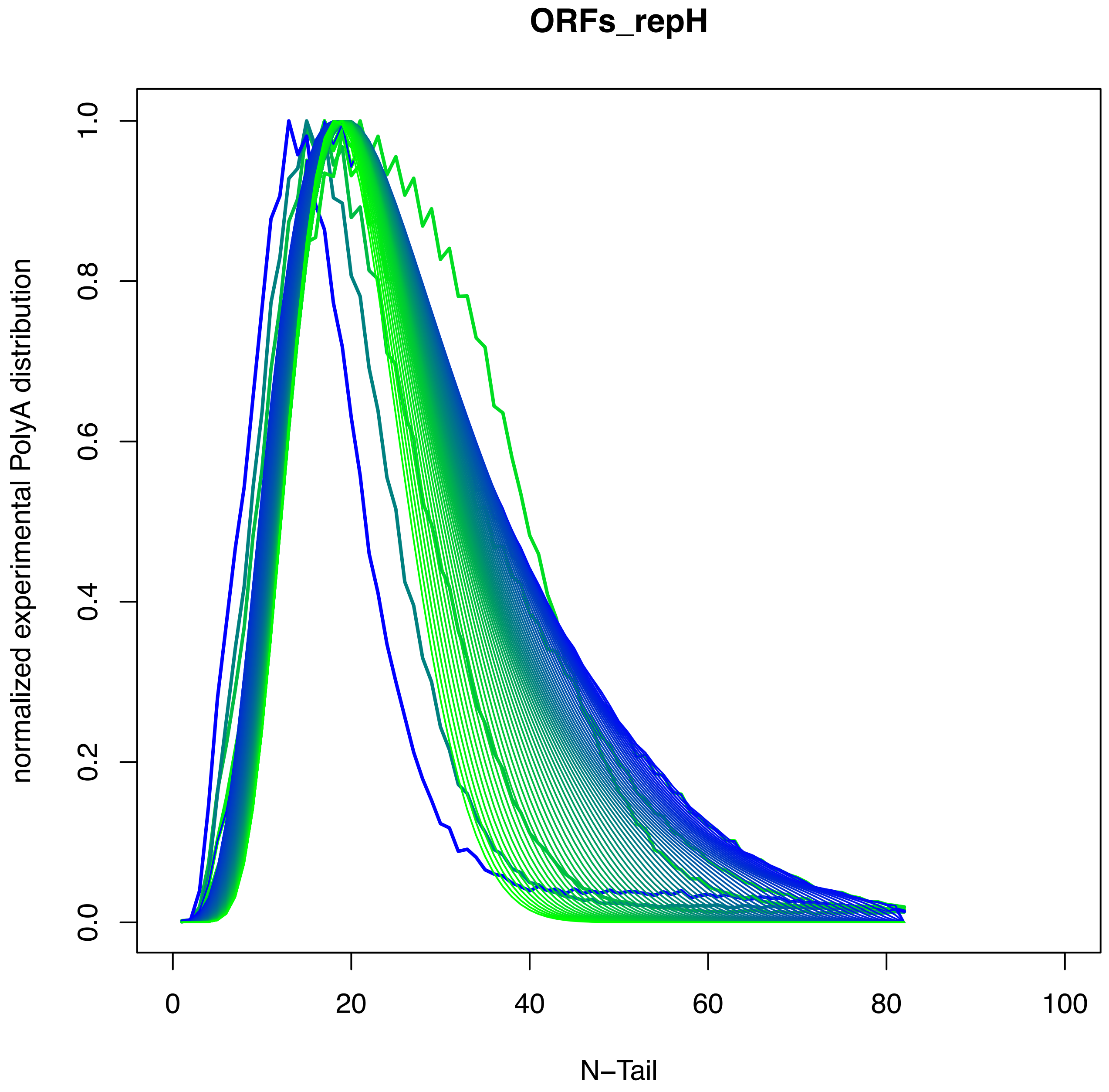
ORFs_repH
normalized experimental PolyA distribution
N−Tail
0.0
0.2
0.4
0.6
0.8
1.0
0
20
40
60
80
100

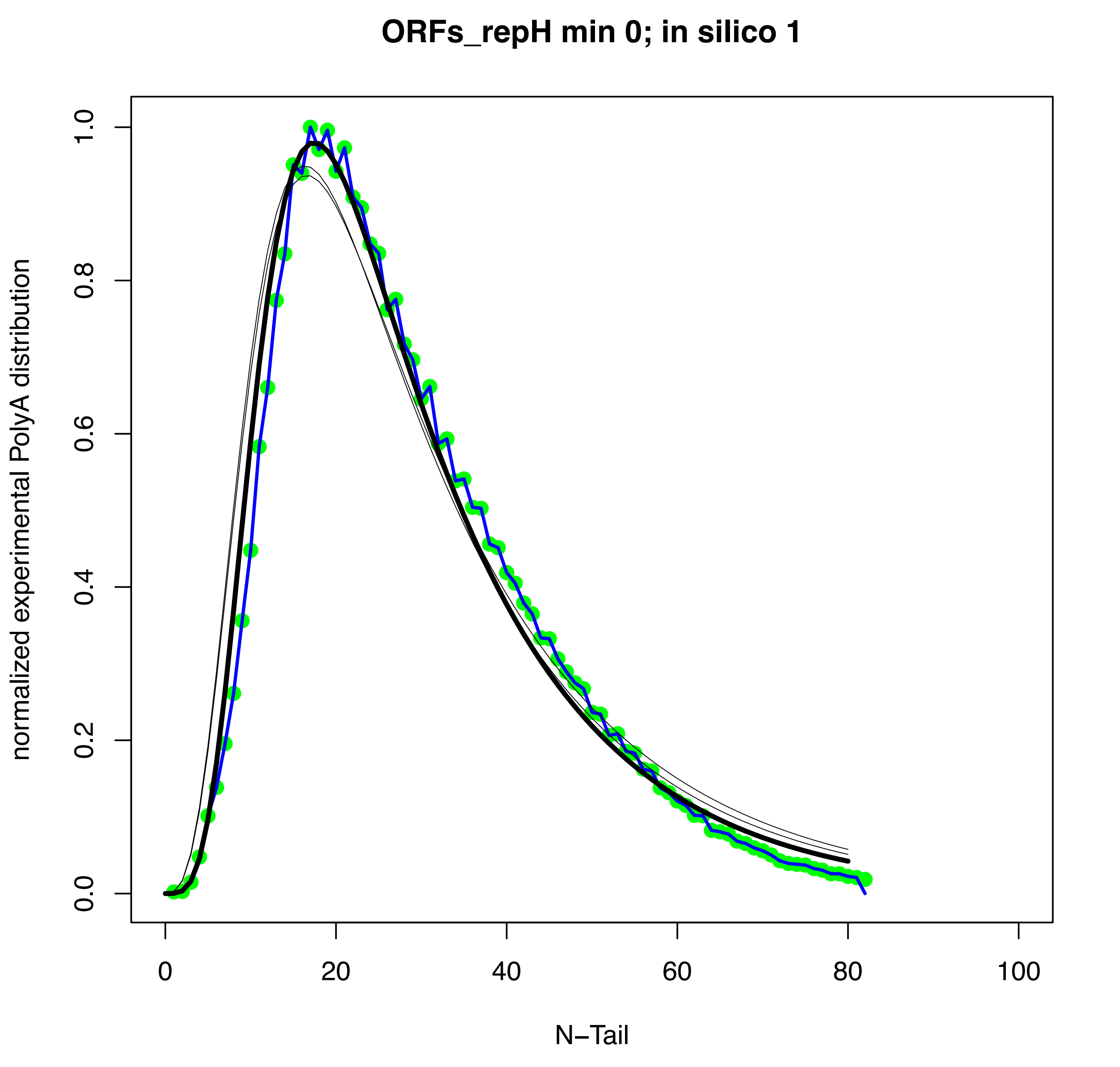

ORFs_repH min 0; in silico 1
normalized experimental PolyA distribution
0.0
0.2
0.4
0.6
0.8
1.0
0
20
40
60
80
100
N−Tail

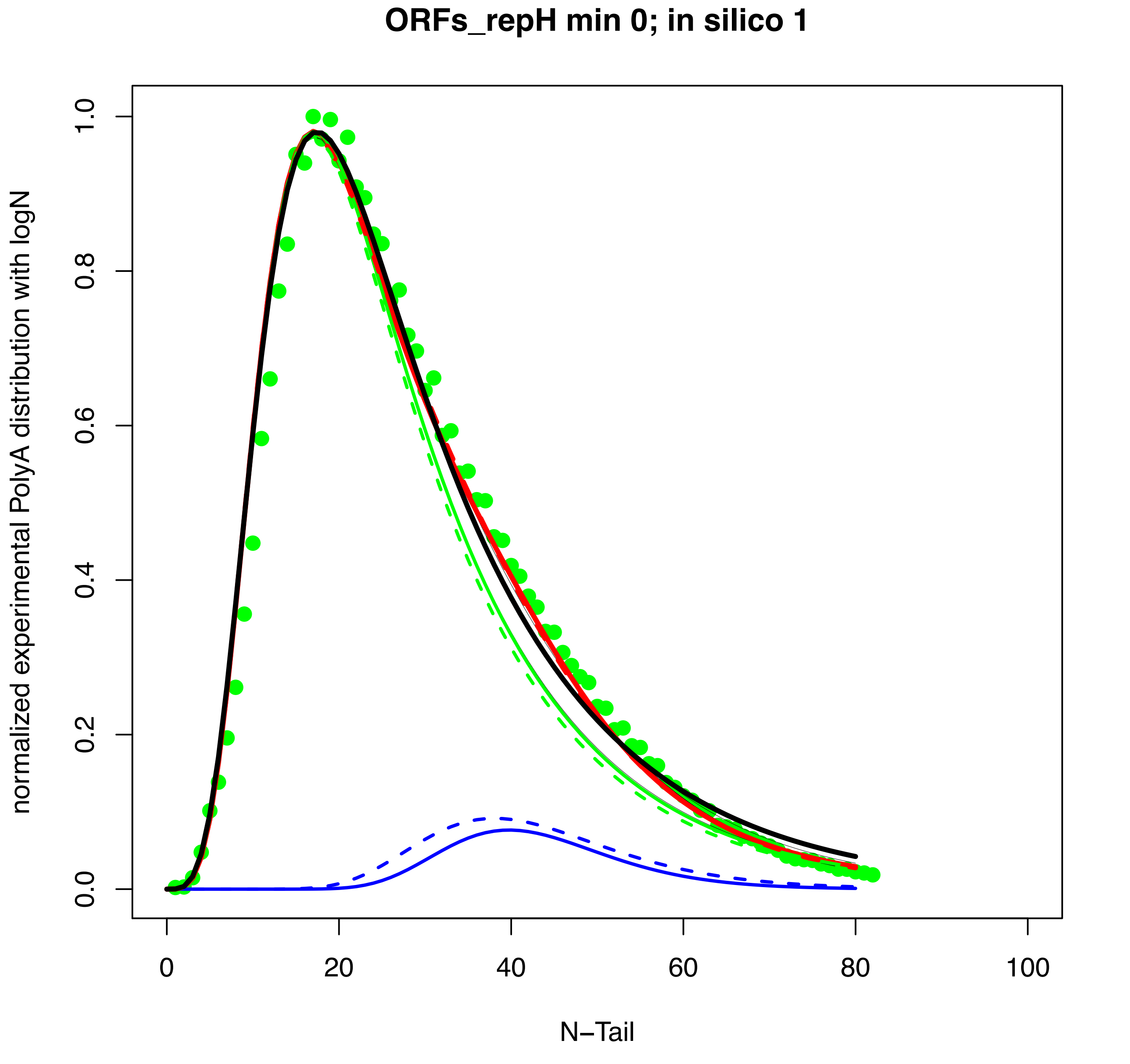
ORFs_repH min 0; in silico 1
normalized experimental PolyA distribution with logN
N−Tail
0.0
0.2
0.4
0.6
0.8
1.0
0
20
40
60
80
100

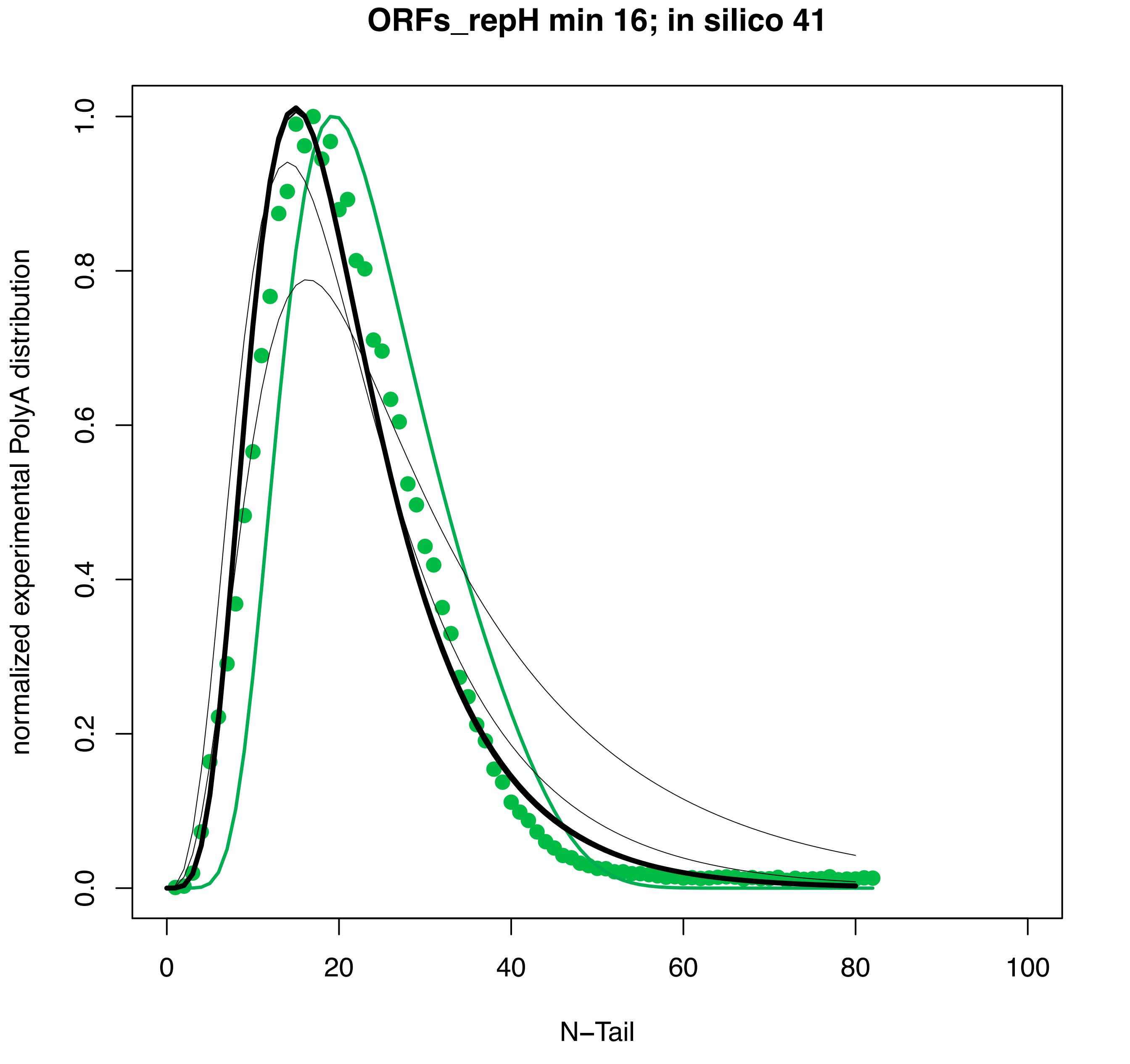
ORFs_repH min 16; in silico 41
normalized experimental PolyA distribution
0.0
0.2
0.4
0.6
0.8
1.0
0
20
40
60
80
100
N−Tail

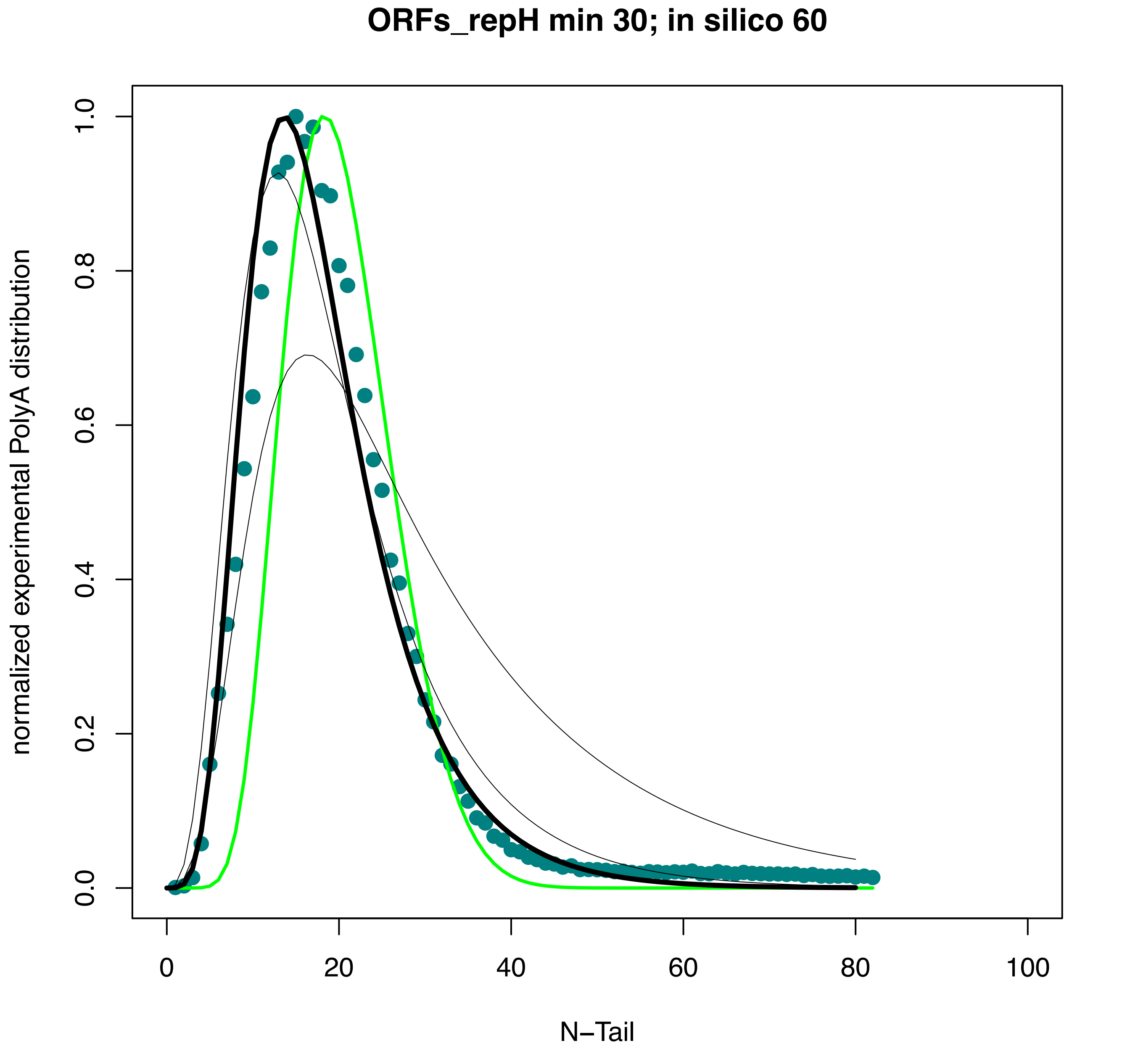
ORFs_repH min 30; in silico 60
normalized experimental PolyA distribution
N−Tail
0.0
0.2
0.4
0.6
0.8
1.0
0
20
40
60
80
100

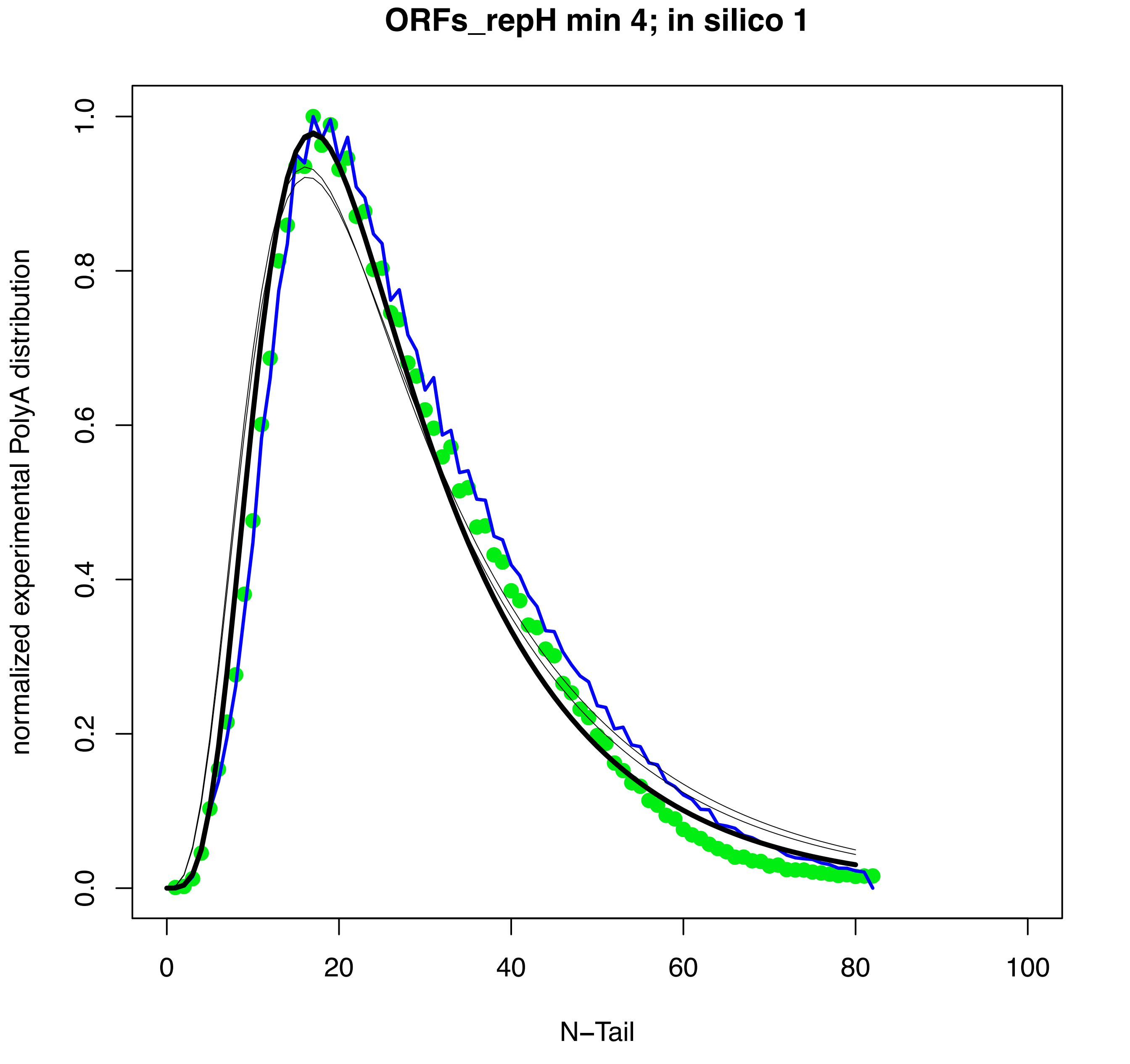
ORFs_repH min 4; in silico 1
normalized experimental PolyA distribution
N−Tail
0.0
0.2
0.4
0.6
0.8
1.0
0
20
40
60
80
100

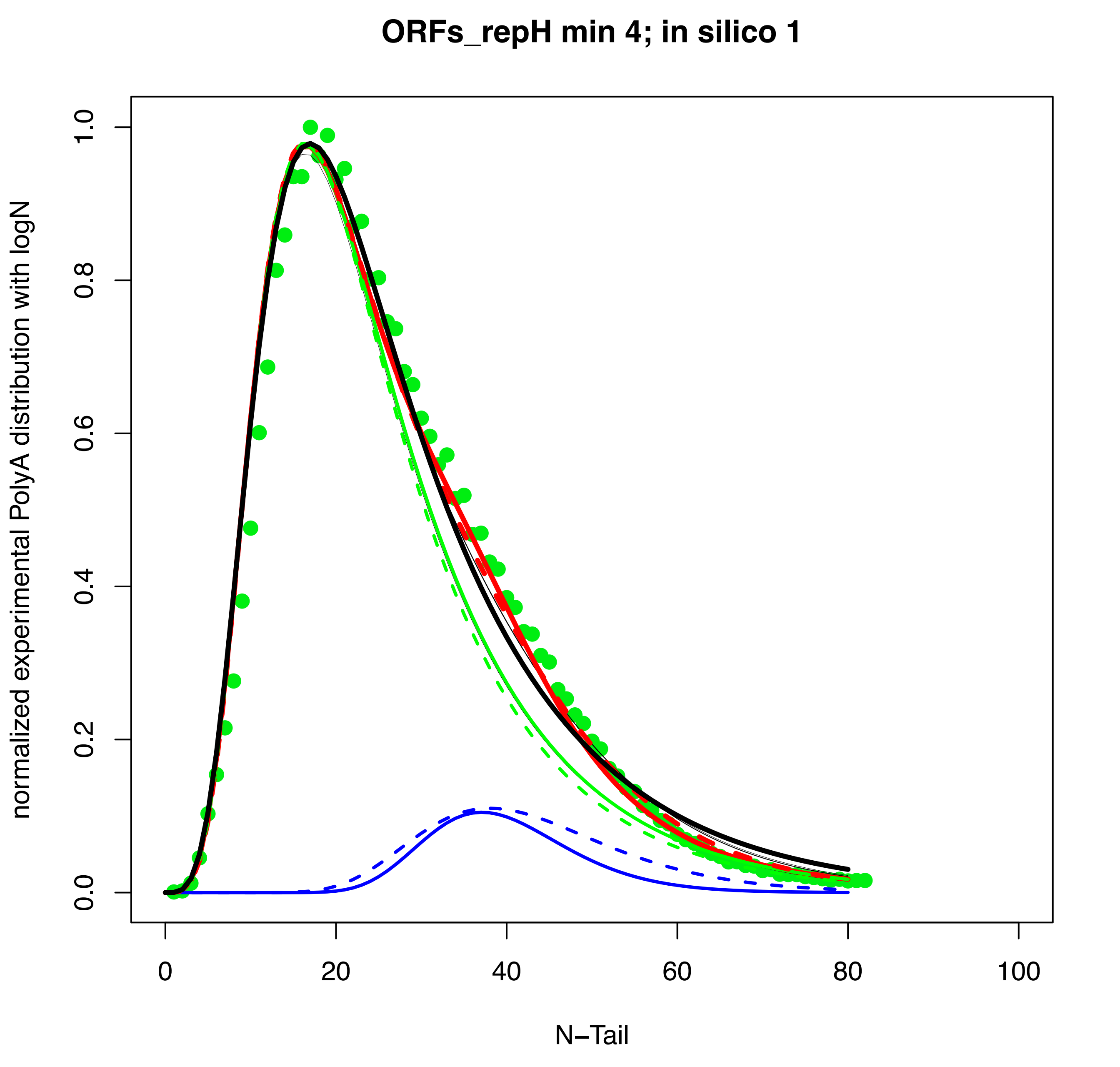

ORFs_repH min 4; in silico 1
normalized experimental PolyA distribution with logN
N−Tail
0.0
0.2
0.4
0.6
0.8
1.0
0
20
40
60
80
100

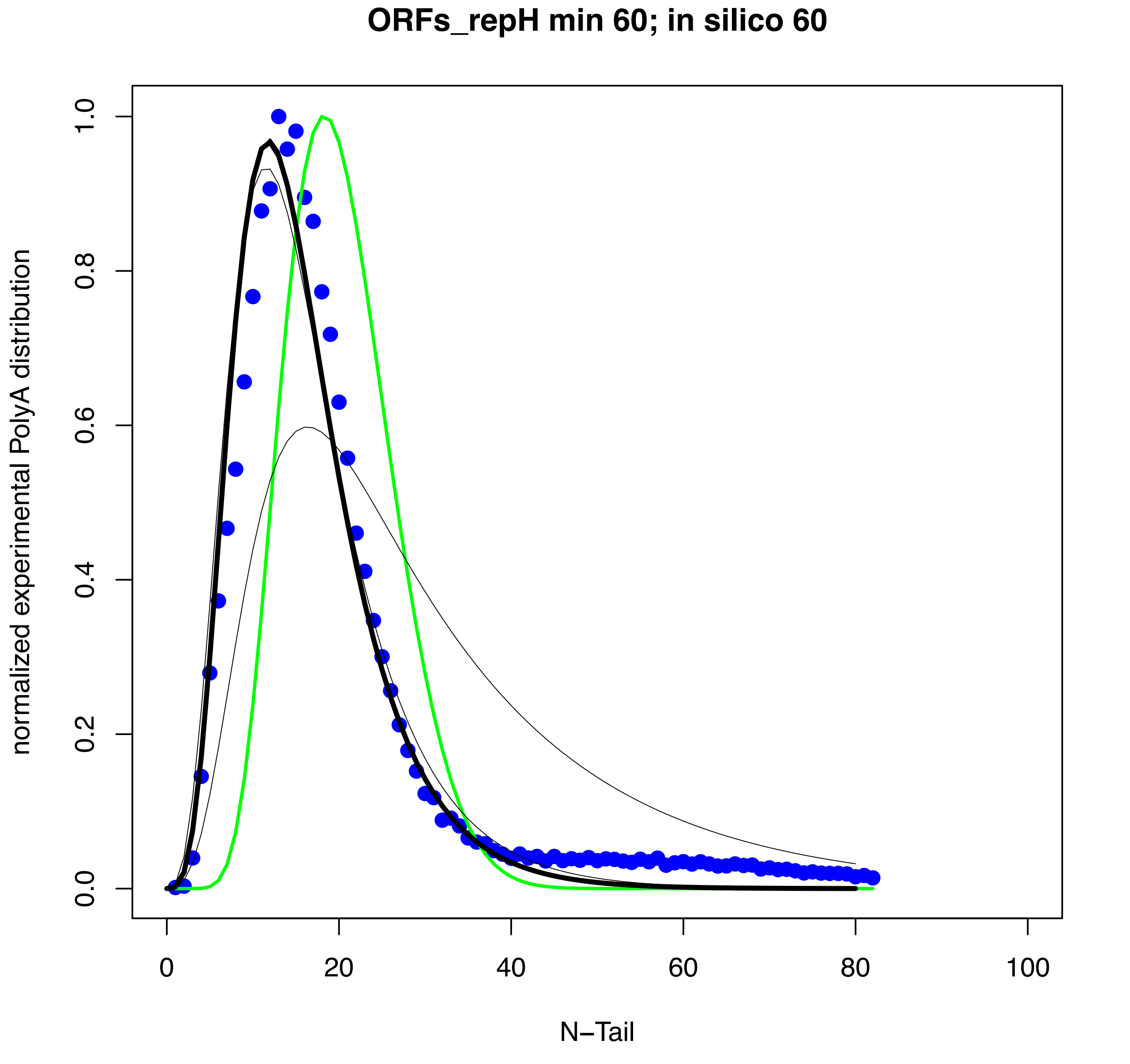

ORFs_repH min 60; in silico 60
normalized experimental PolyA distribution
N−Tail
0.0
0.2
0.4
0.6
0.8
1.0
0
20
40
60
80
100

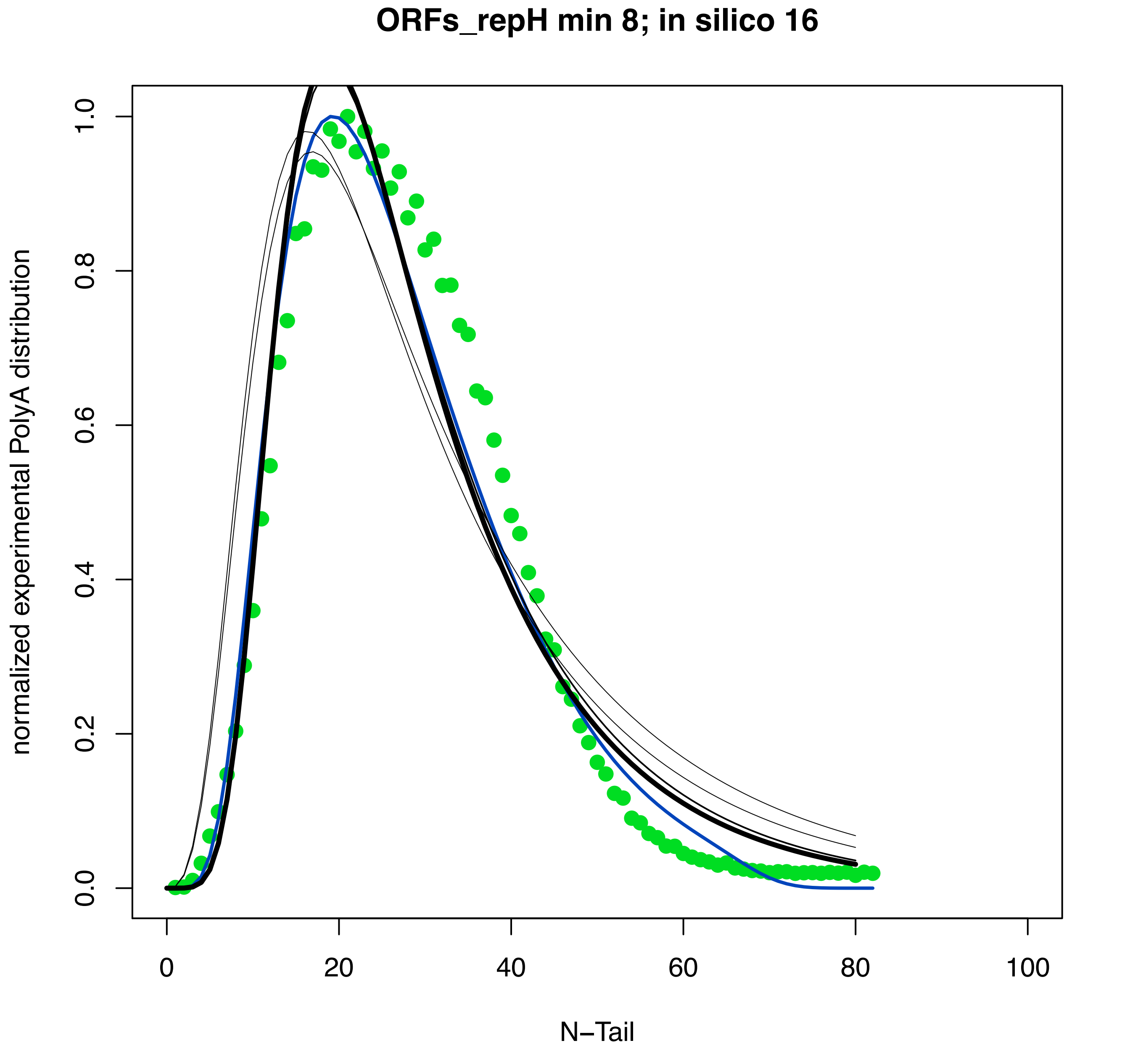

ORFs_repH min 8; in silico 16
normalized experimental PolyA distribution
N−Tail
0.0
0.2
0.4
0.6
0.8
1.0
0
20
40
60
80
100

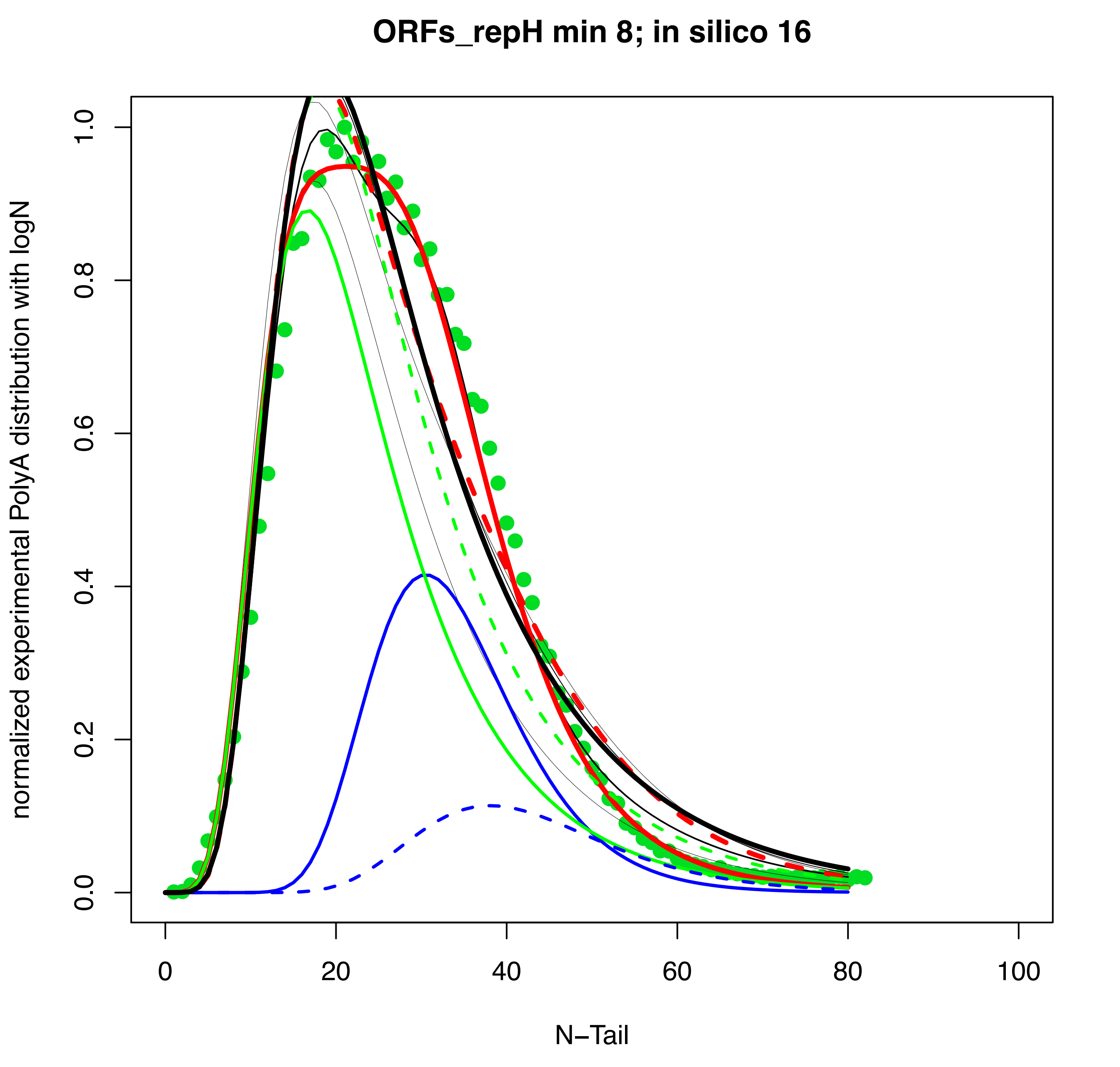
ORFs_repH min 8; in silico 16
normalized experimental PolyA distribution with logN
N−Tail
0.0
0.2
0.4
0.6
0.8
1.0
0
20
40
60
80
100

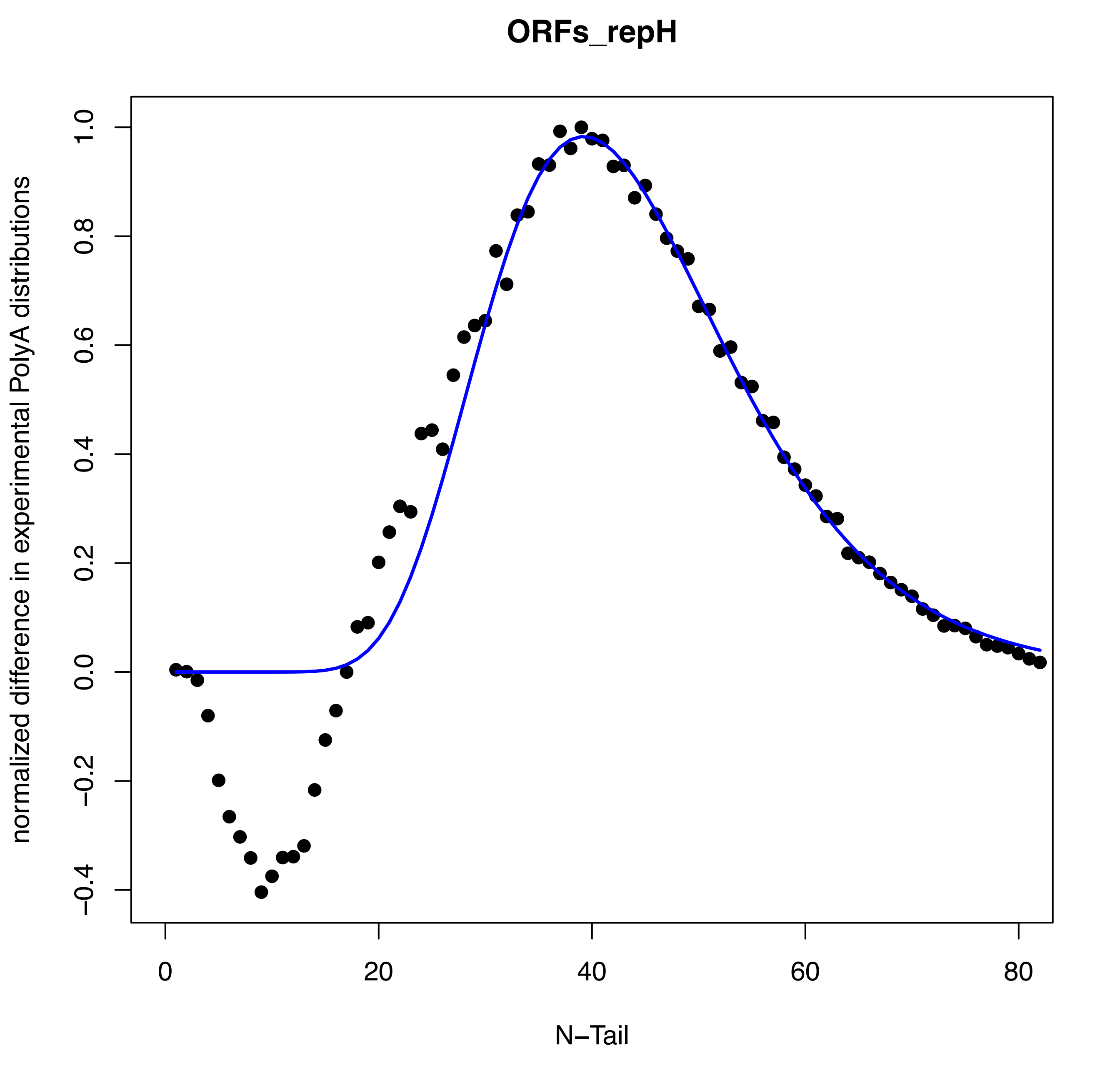

ORFs_repH
normalized difference in experimental PolyA distributions
N−Tail
1.0
0.8
0.6
0.4
0.2
0.0
−0.2
−0.4
0
20
40
60
80

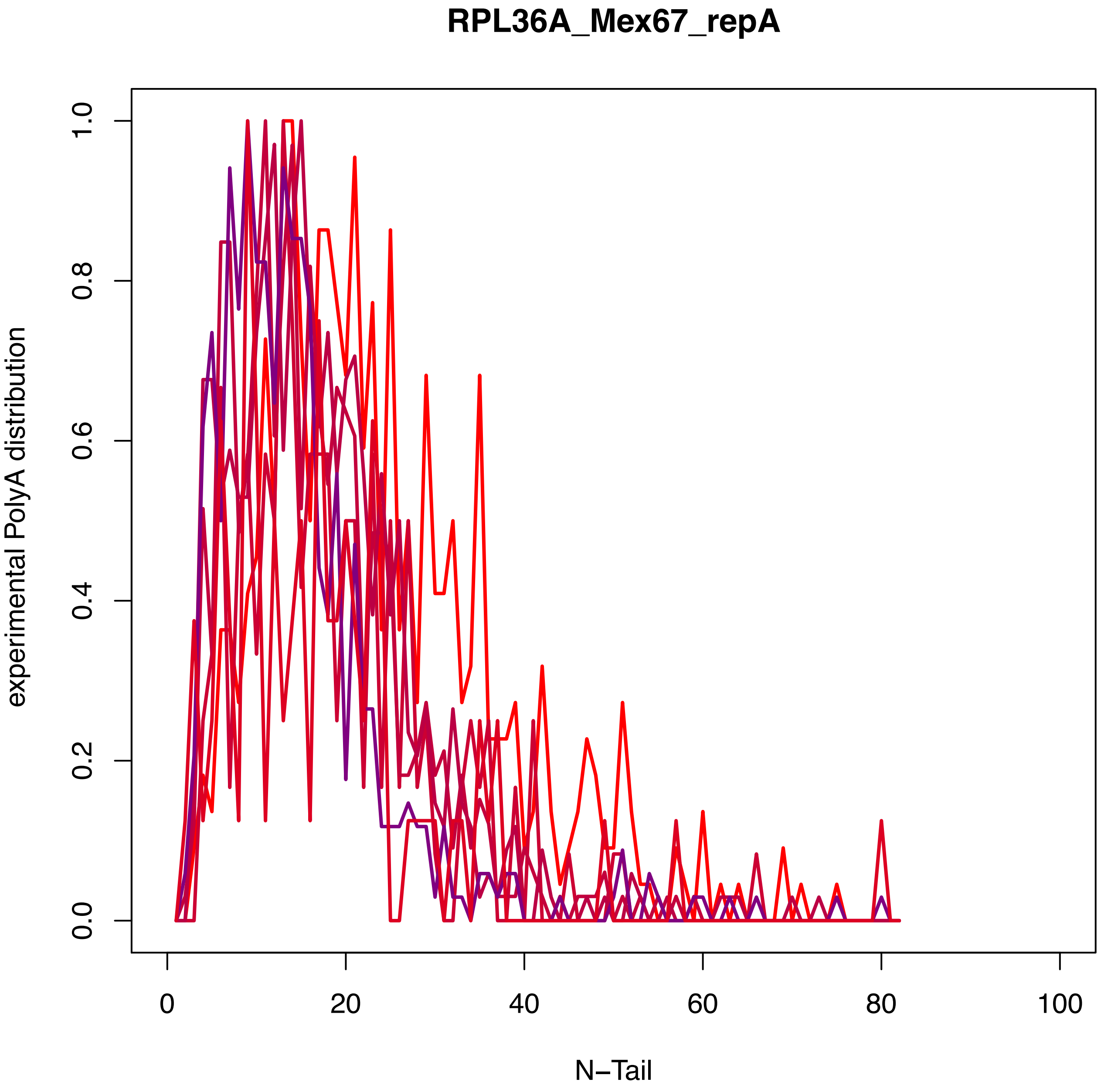

RPL36A_Mex67_repA
experimental PolyA distribution
N−Tail
0.0
0.2
0.4
0.6
0.8
1.0
0
20
40
60
80
100

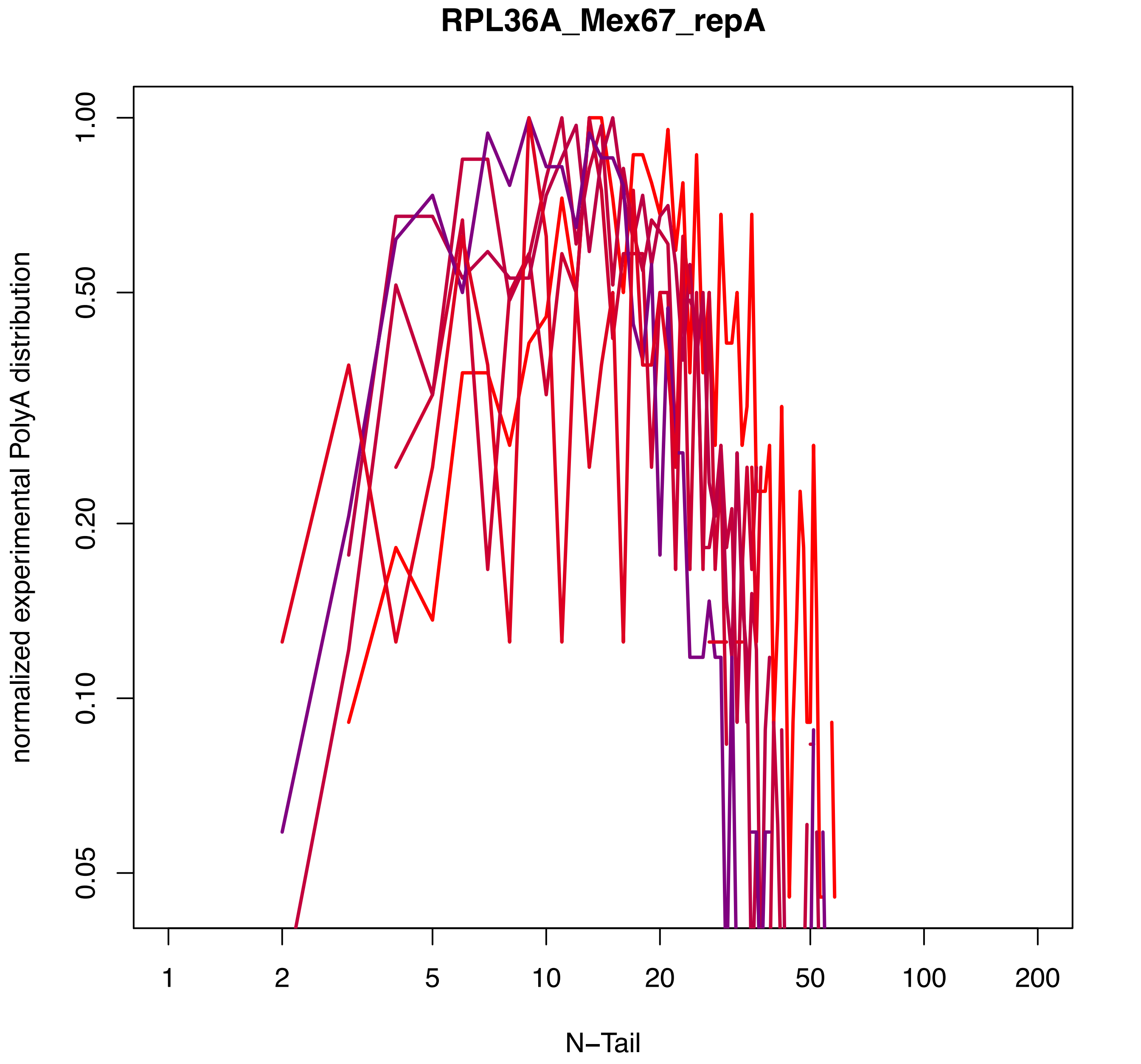

RPL36A_Mex67_repA
normalized experimental PolyA distribution
N−Tail
1.00
0.50
0.20
0.10
0.05
1
2
5
10
20
50
100
200

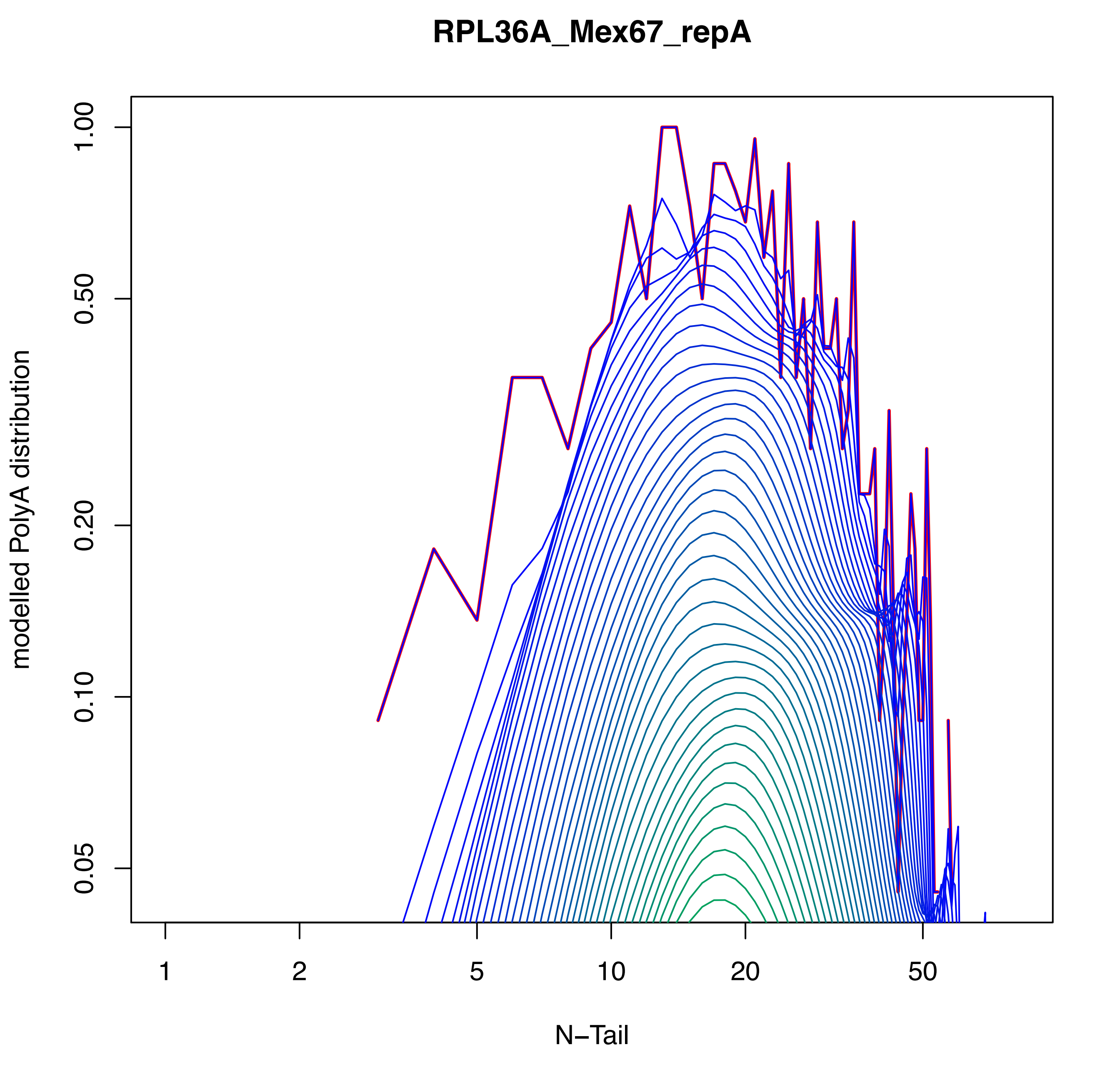
RPL36A_Mex67_repA
modelled PolyA distribution
N−Tail
1.00
0.50
0.20
0.10
0.05
1
2
5
10
20
50

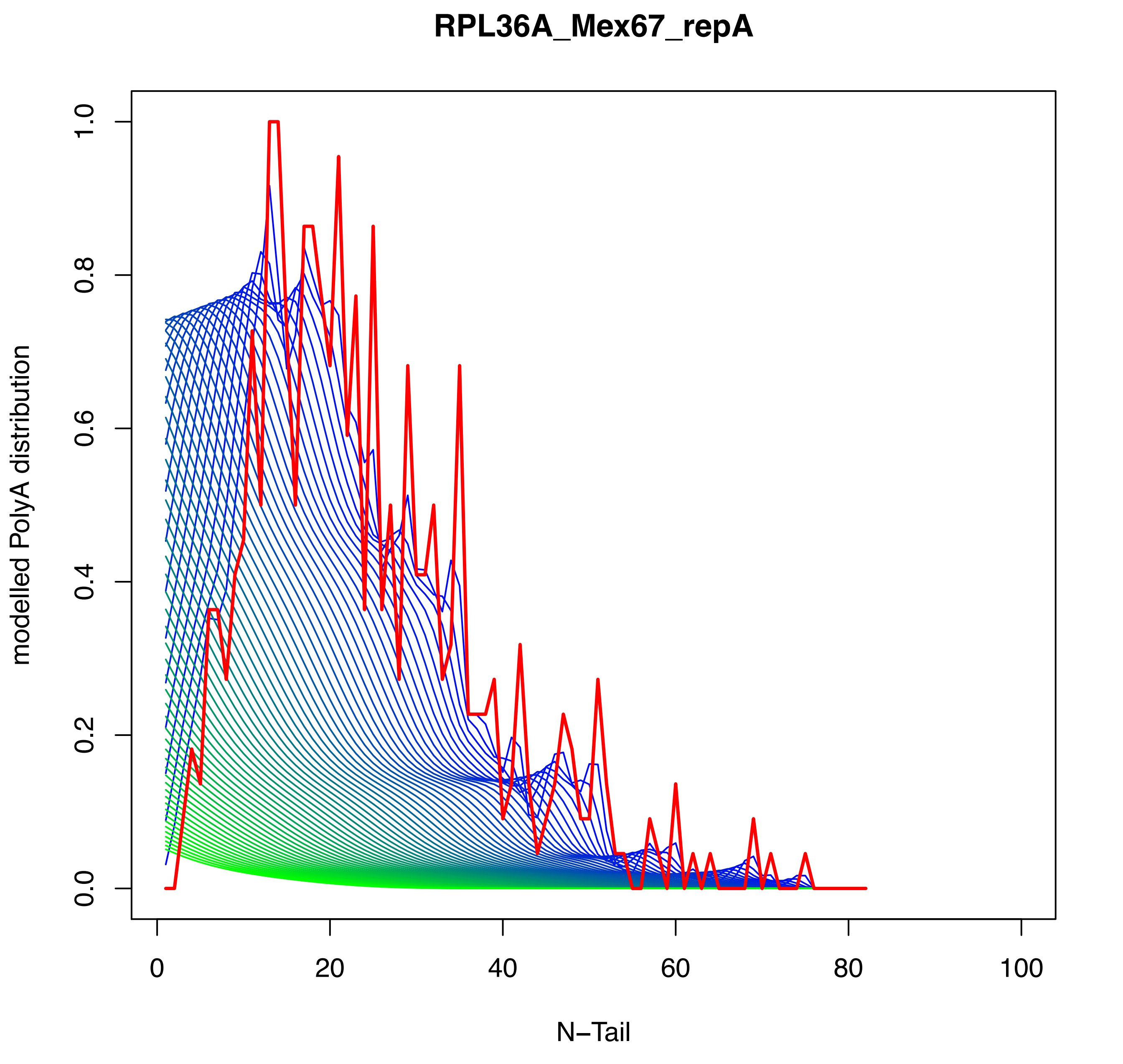
RPL36A_Mex67_repA
modelled PolyA distribution
0.0
0.2
0.4
0.6
0.8
1.0
0
20
40
60
80
100
N−Tail

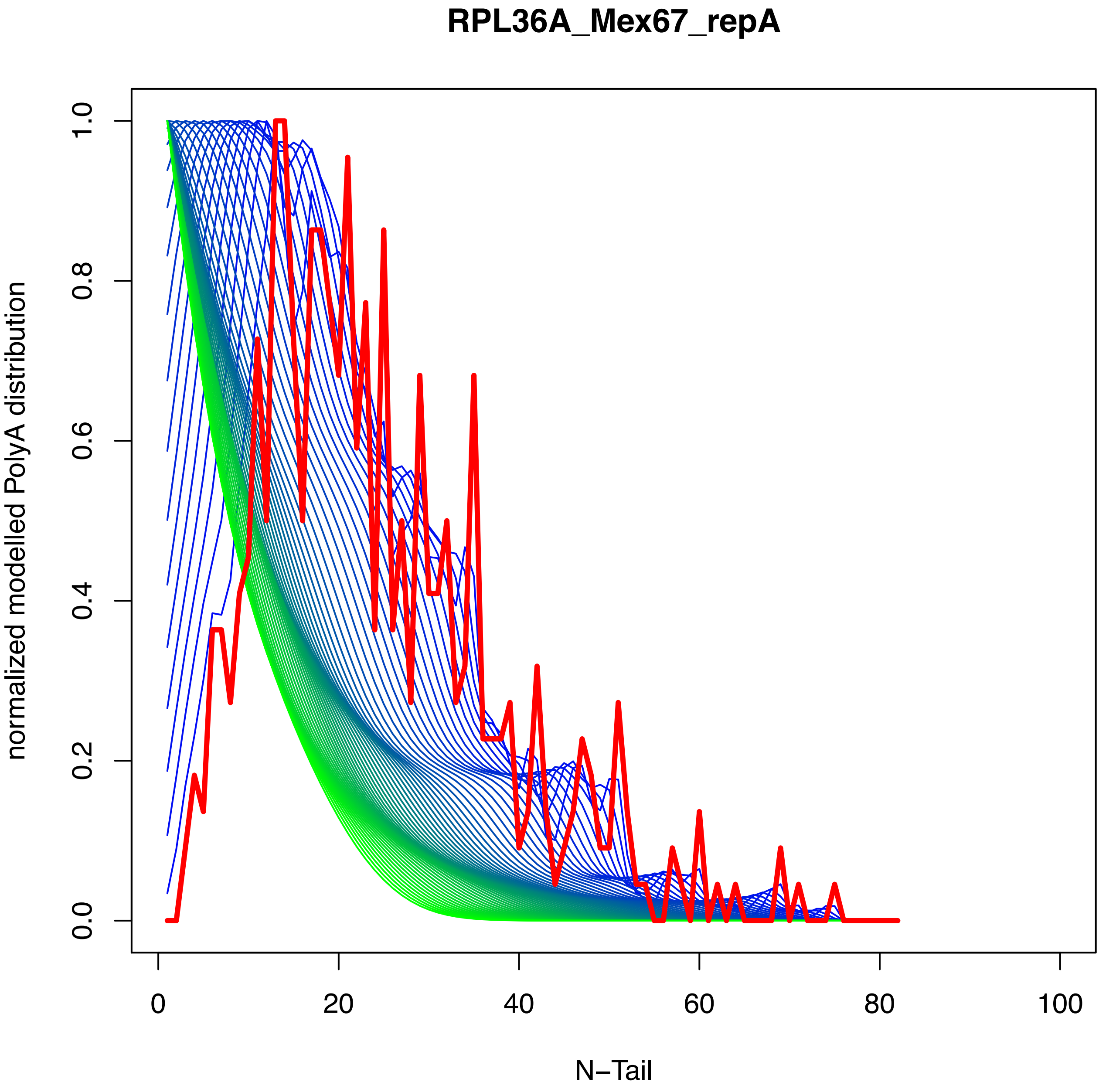

RPL36A_Mex67_repA
normalized modelled PolyA distribution
N−Tail
0.0
0.2
0.4
0.6
0.8
1.0
0
20
40
60
80
100

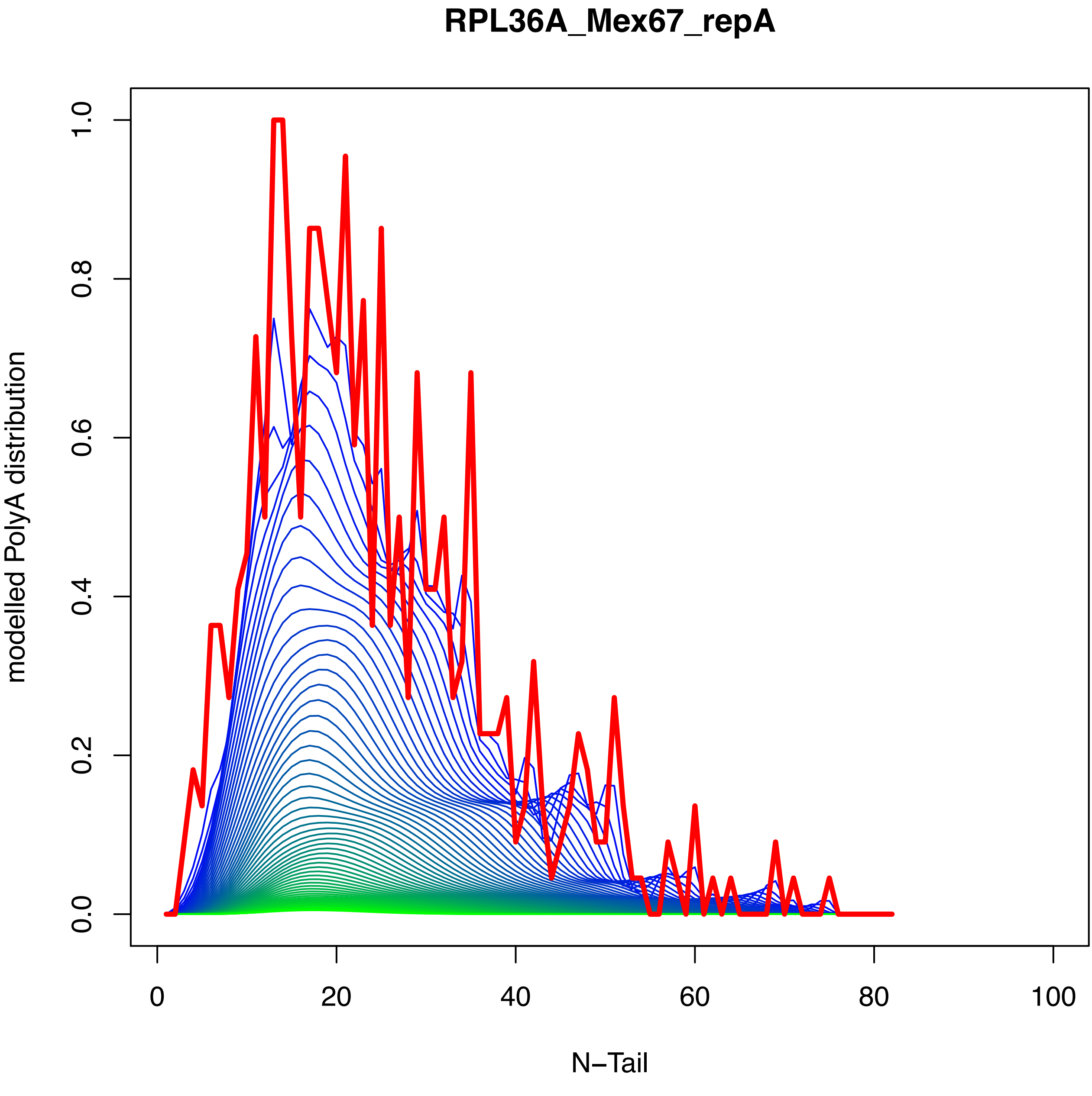

RPL36A_Mex67_repA
modelled PolyA distribution
N−Tail
0.0
0.2
0.4
0.6
0.8
1.0
0
20
40
60
80
100

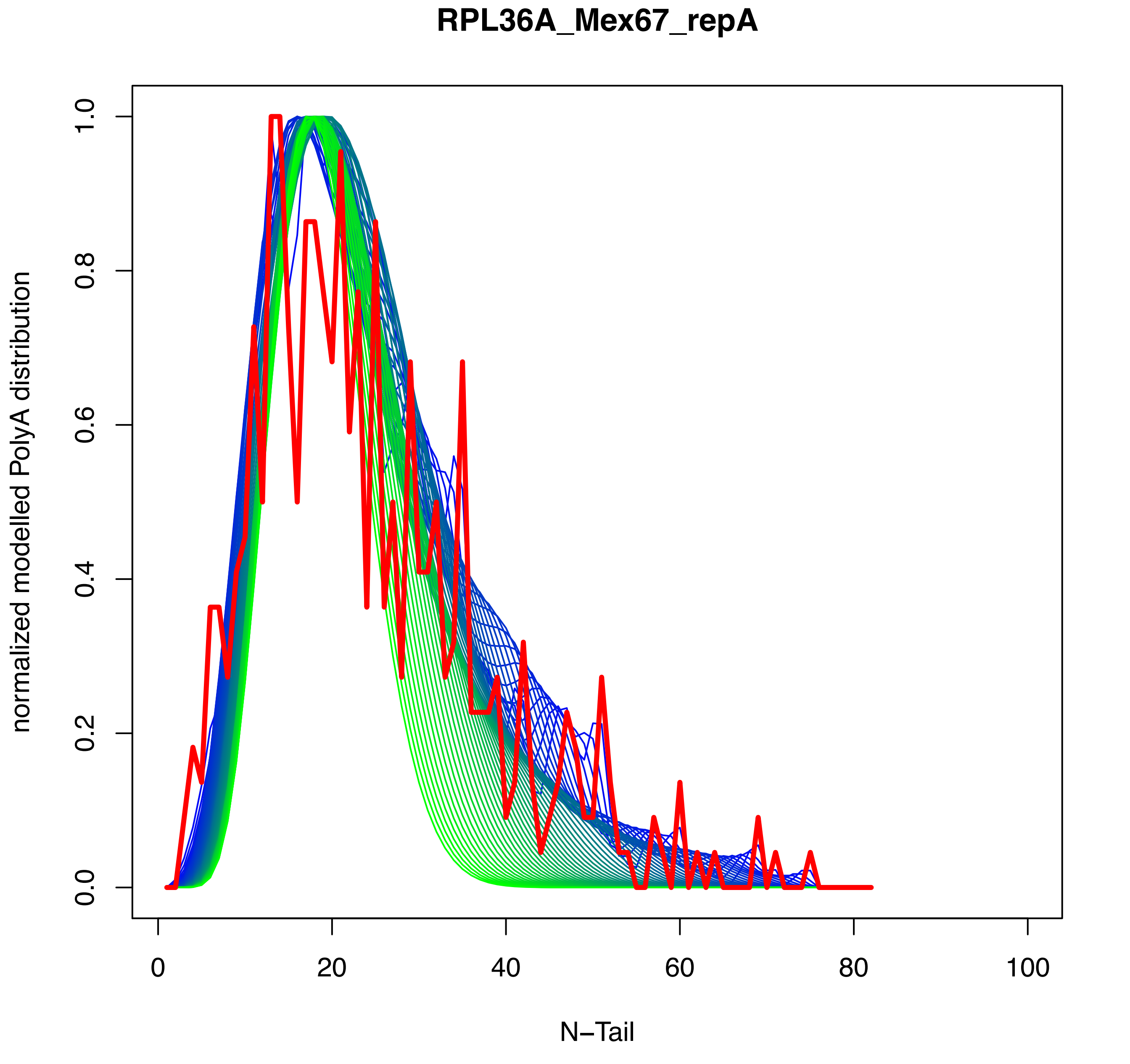

RPL36A_Mex67_repA
normalized modelled PolyA distribution
N−Tail
0.0
0.2
0.4
0.6
0.8
1.0
0
20
40
60
80
100

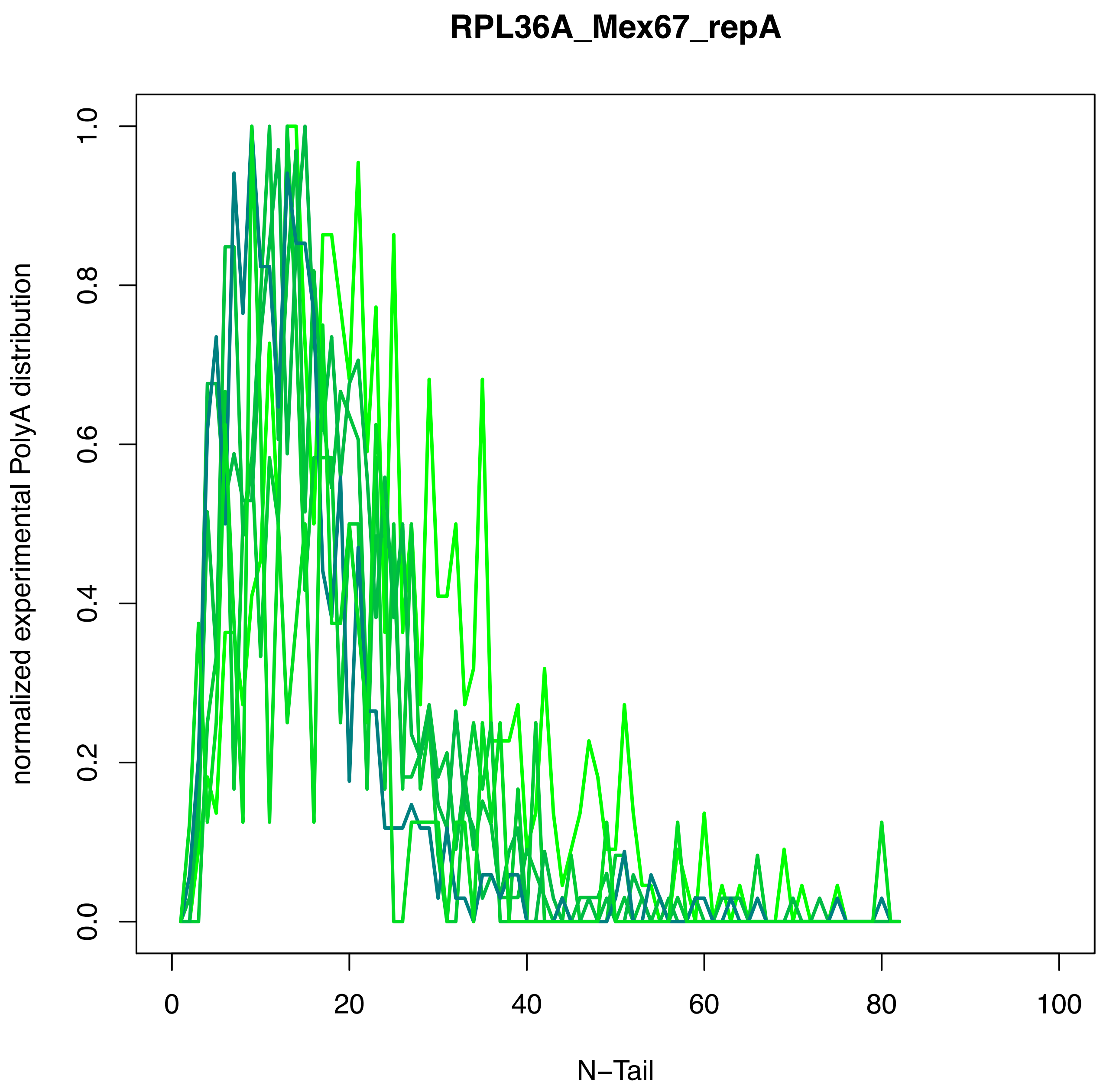

RPL36A_Mex67_repA
normalized experimental PolyA distribution
N−Tail
0.0
0.2
0.4
0.6
0.8
1.0
0
20
40
60
80
100

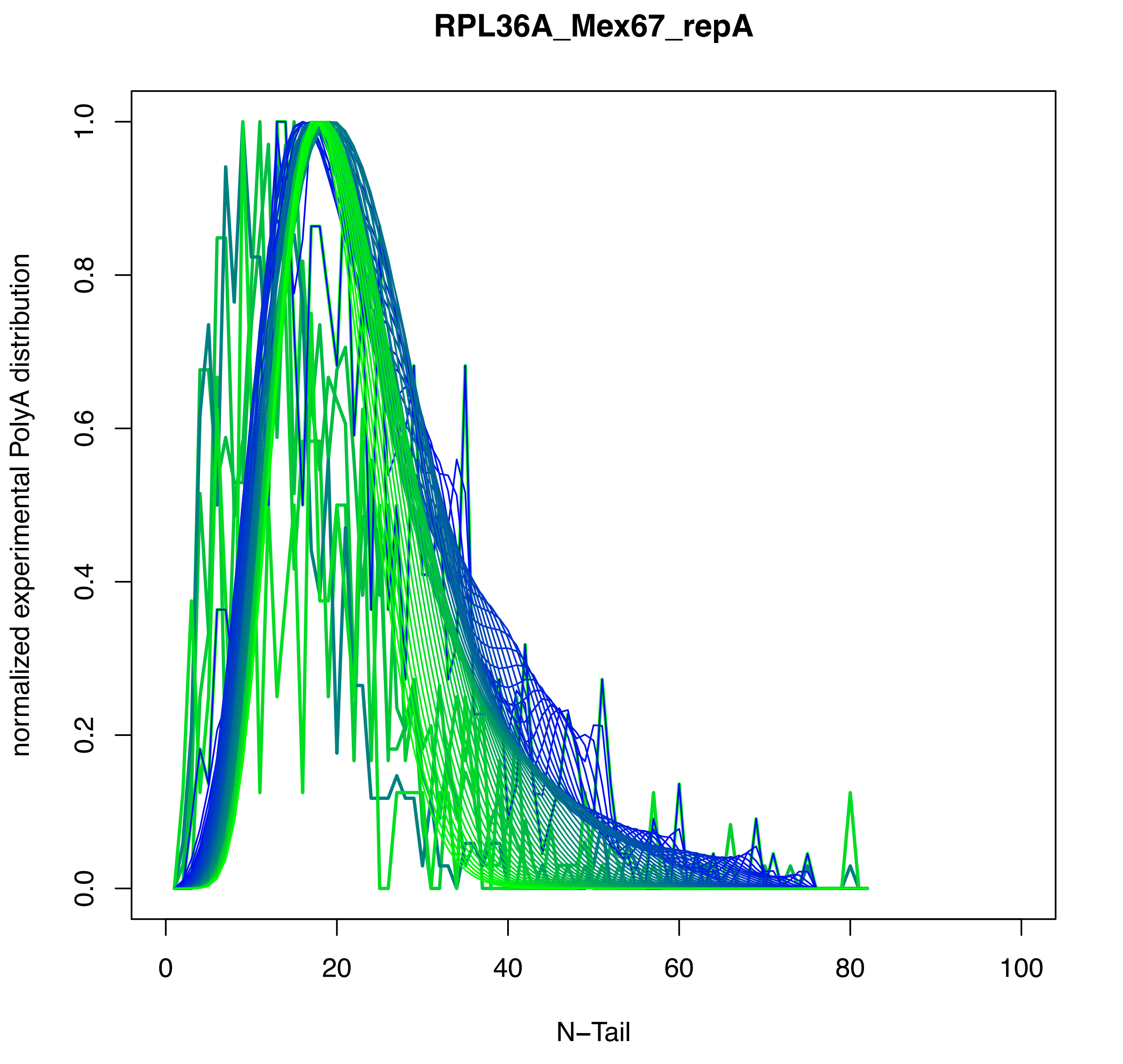

RPL36A_Mex67_repA
normalized experimental PolyA distribution
N−Tail
0.0
0.2
0.4
0.6
0.8
1.0
0
20
40
60
80
100

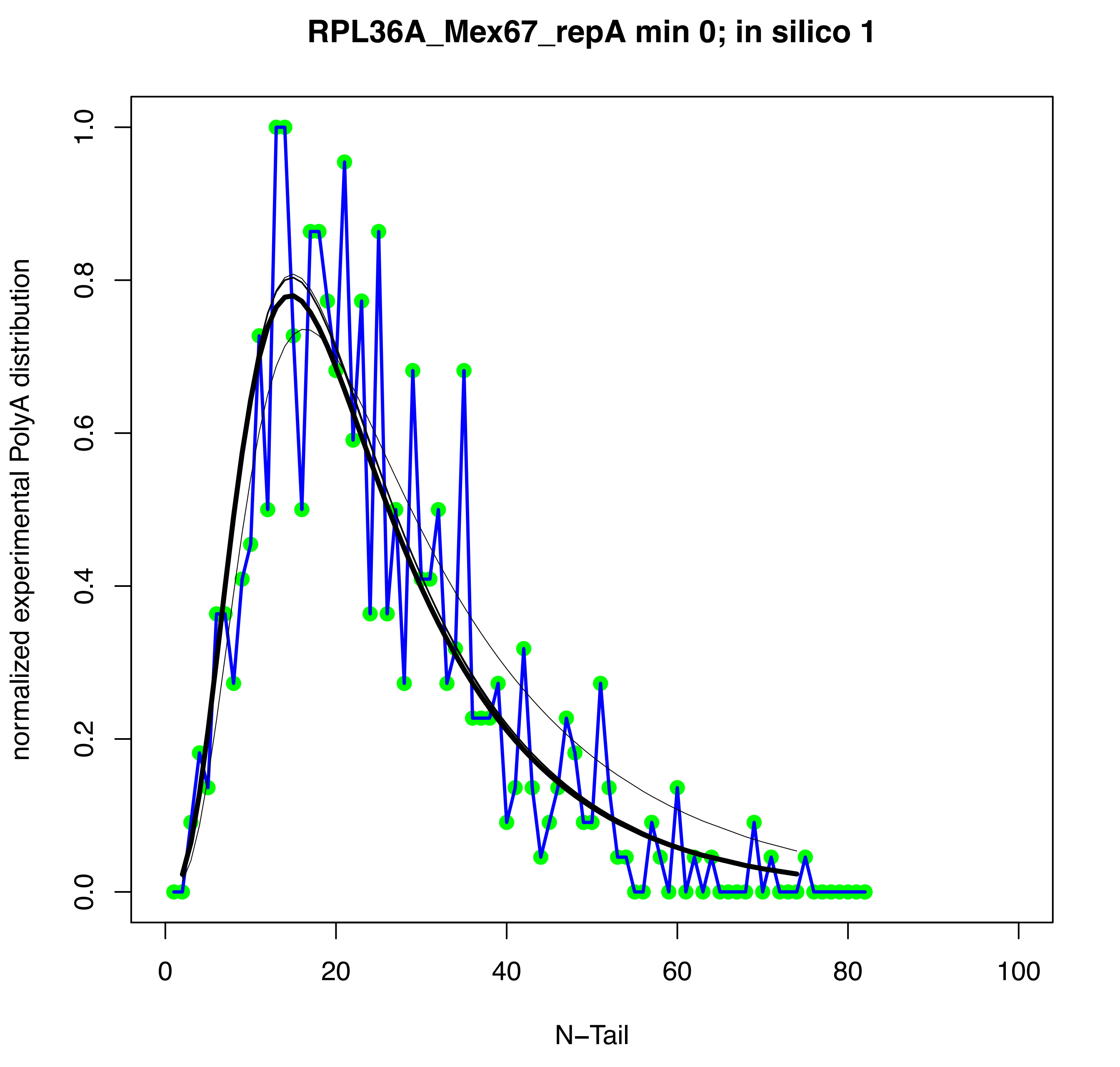
RPL36A_Mex67_repA min 0; in silico 1
normalized experimental PolyA distribution
N−Tail
0.0
0.2
0.4
0.6
0.8
1.0
0
20
40
60
80
100

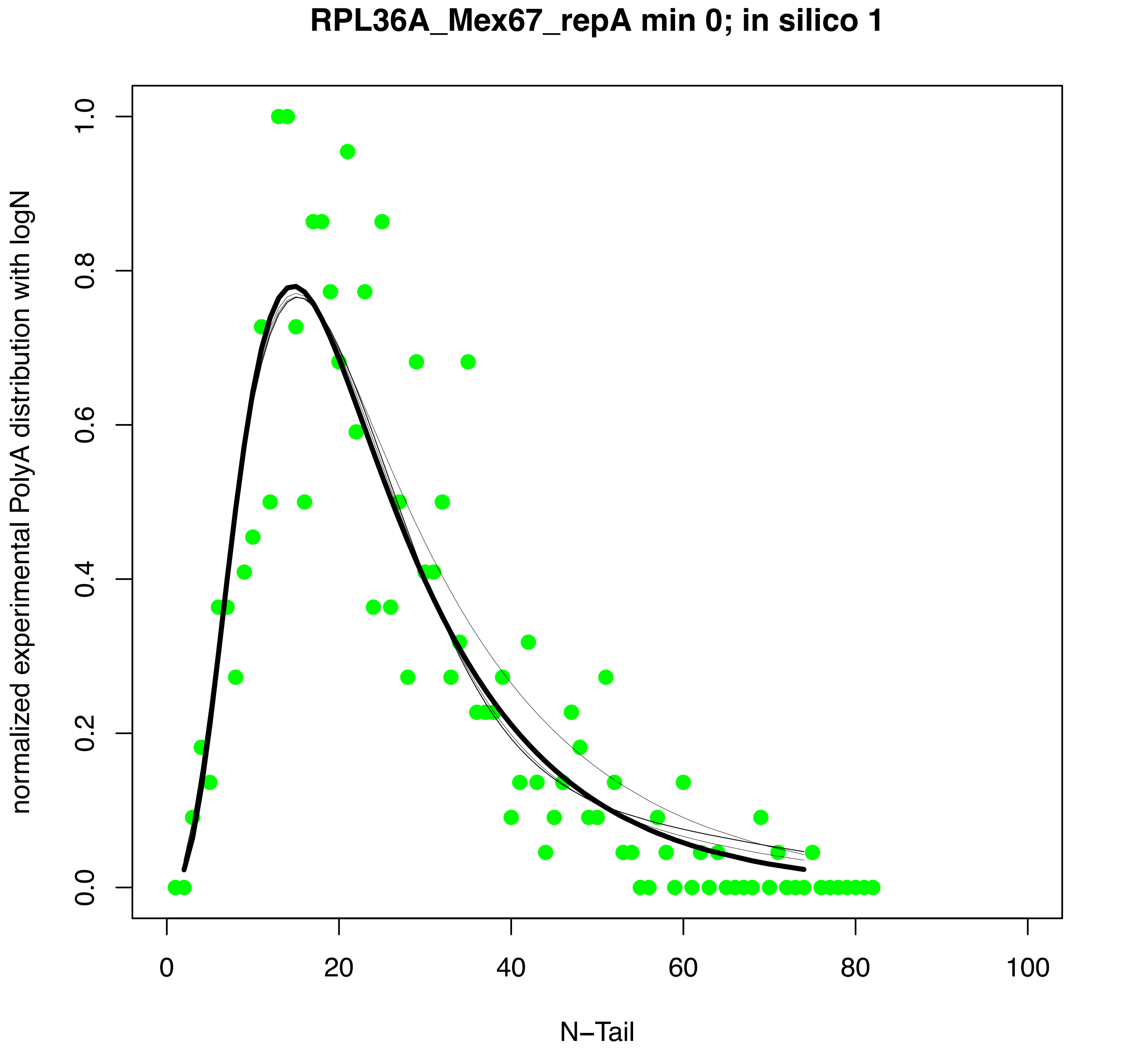

RPL36A_Mex67_repA min 0; in silico 1
normalized experimental PolyA distribution with logN
N−Tail
0.0
0.2
0.4
0.6
0.8
1.0
0
20
40
60
80
100

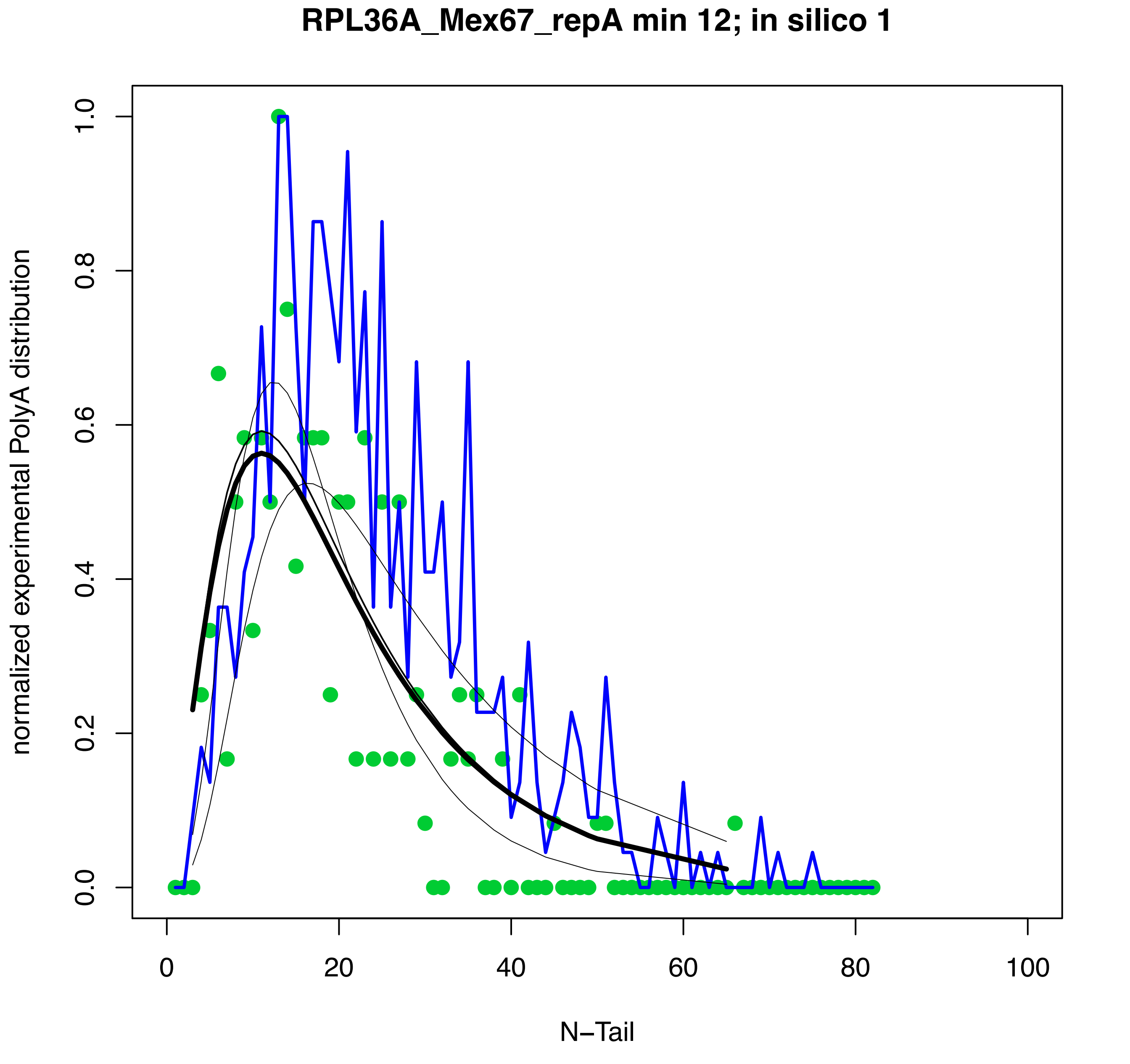
RPL36A_Mex67_repA min 12; in silico 1
normalized experimental PolyA distribution
N−Tail
0.0
0.2
0.4
0.6
0.8
1.0
0
20
40
60
80
100

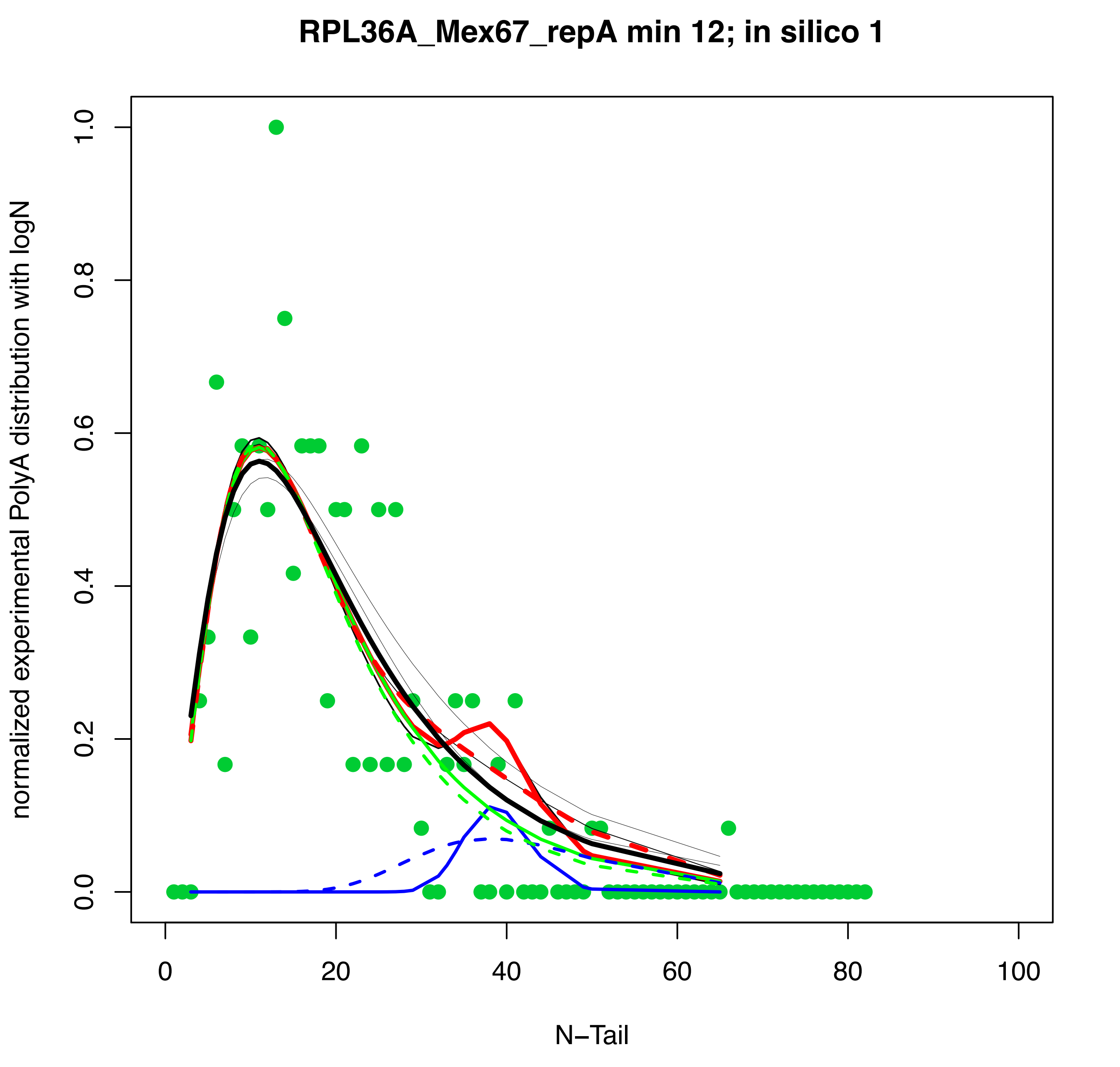
RPL36A_Mex67_repA min 12; in silico 1
normalized experimental PolyA distribution with logN
N−Tail
0.0
0.2
0.4
0.6
0.8
1.0
0
20
40
60
80
100

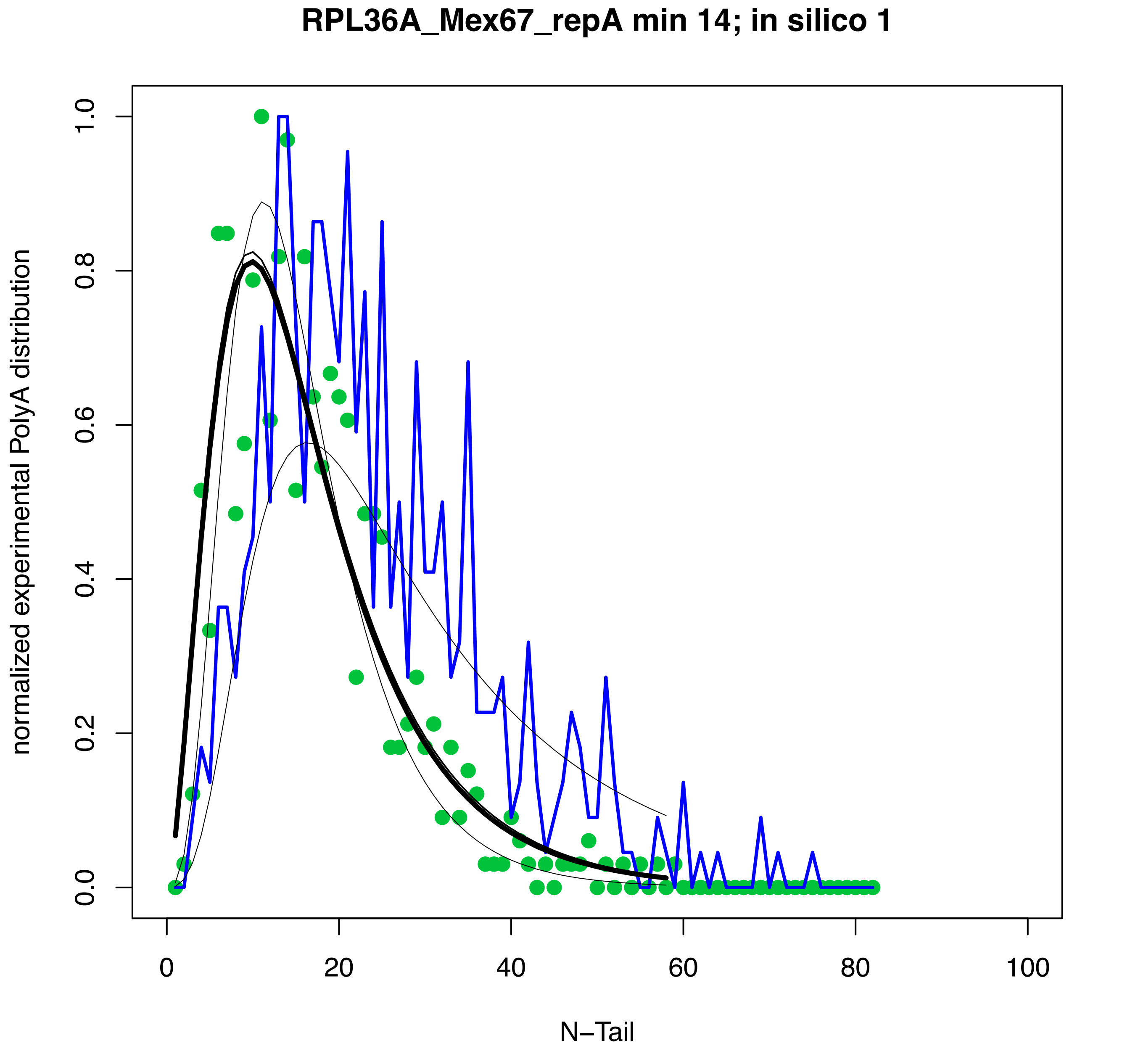

RPL36A_Mex67_repA min 14; in silico 1
normalized experimental PolyA distribution
N−Tail
0.0
0.2
0.4
0.6
0.8
1.0
0
20
40
60
80
100

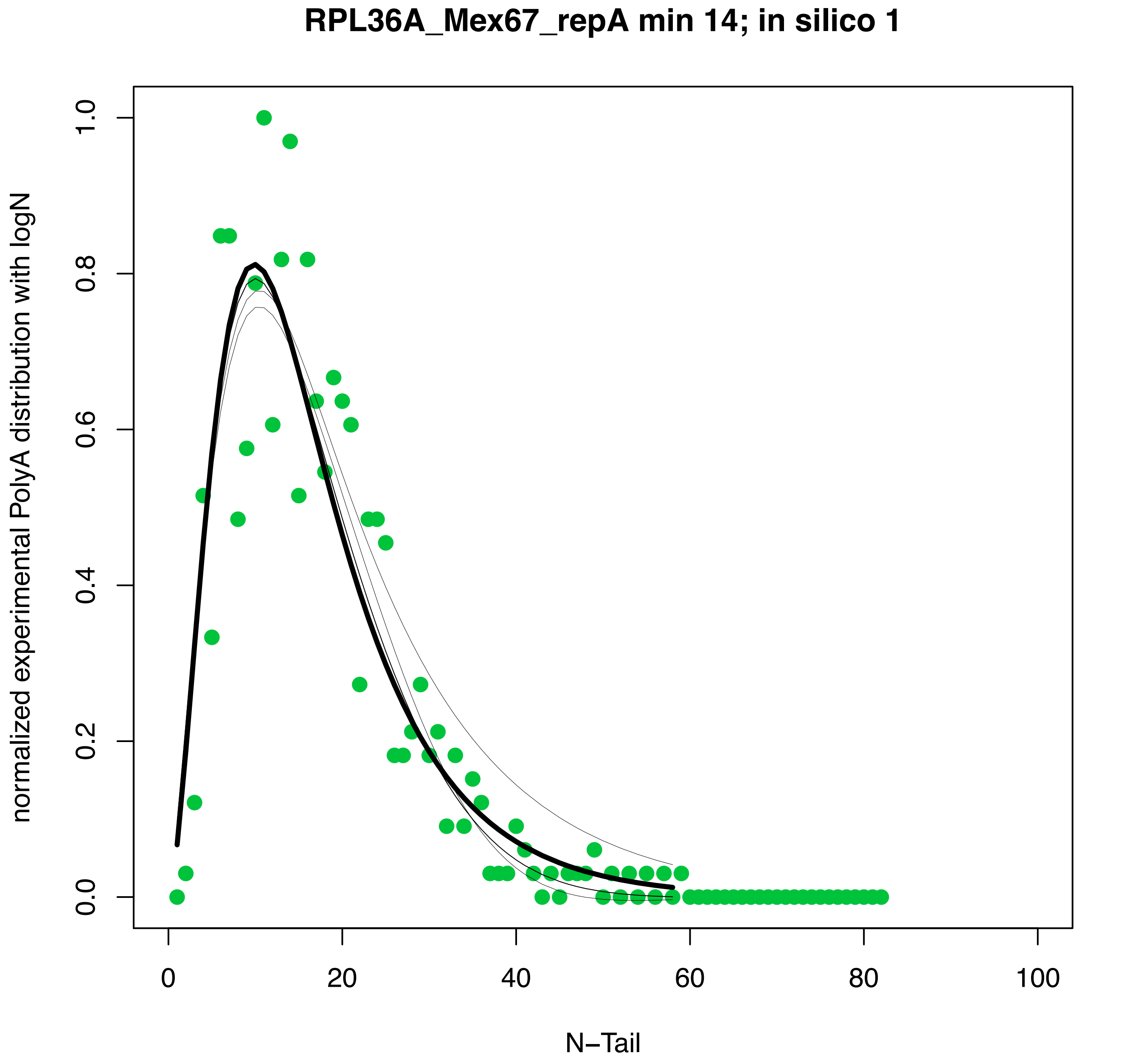

RPL36A_Mex67_repA min 14; in silico 1
normalized experimental PolyA distribution with logN
N−Tail
0.0
0.2
0.4
0.6
0.8
1.0
0
20
40
60
80
100

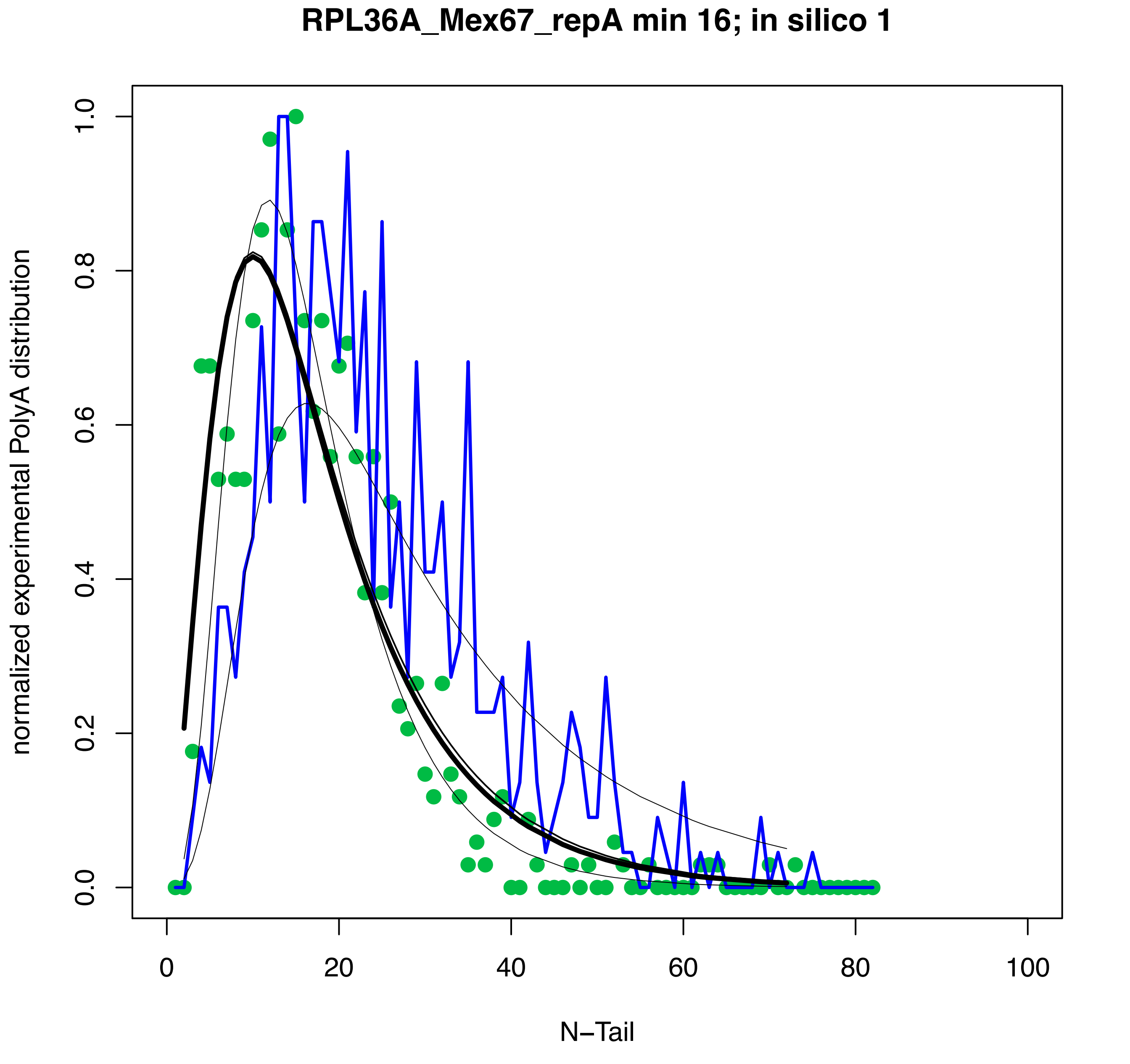
RPL36A_Mex67_repA min 16; in silico 1
normalized experimental PolyA distribution
N−Tail
0.0
0.2
0.4
0.6
0.8
1.0
0
20
40
60
80
100

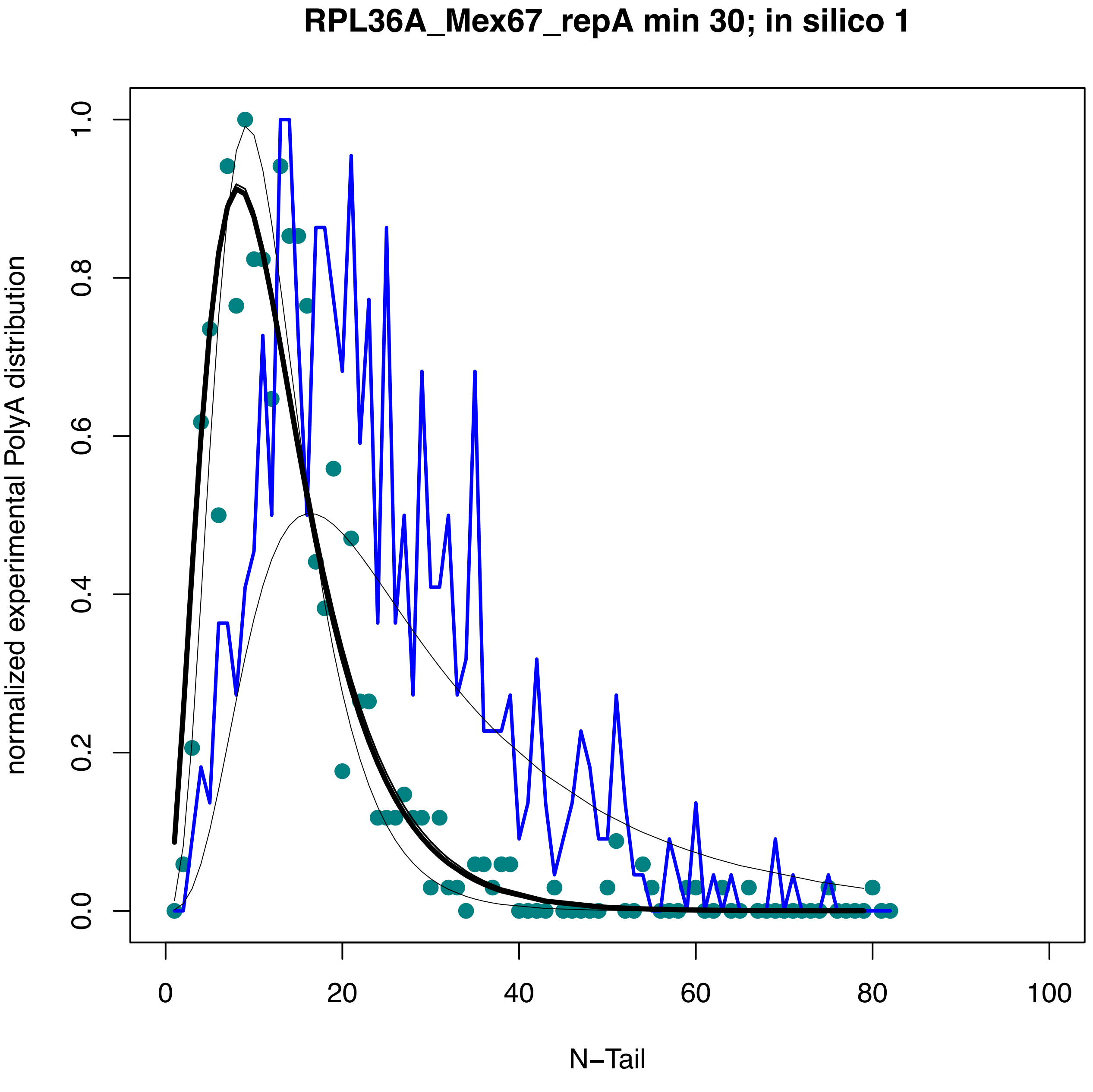
RPL36A_Mex67_repA min 30; in silico 1
normalized experimental PolyA distribution
N−Tail
0.0
0.2
0.4
0.6
0.8
1.0
0
20
40
60
80
100

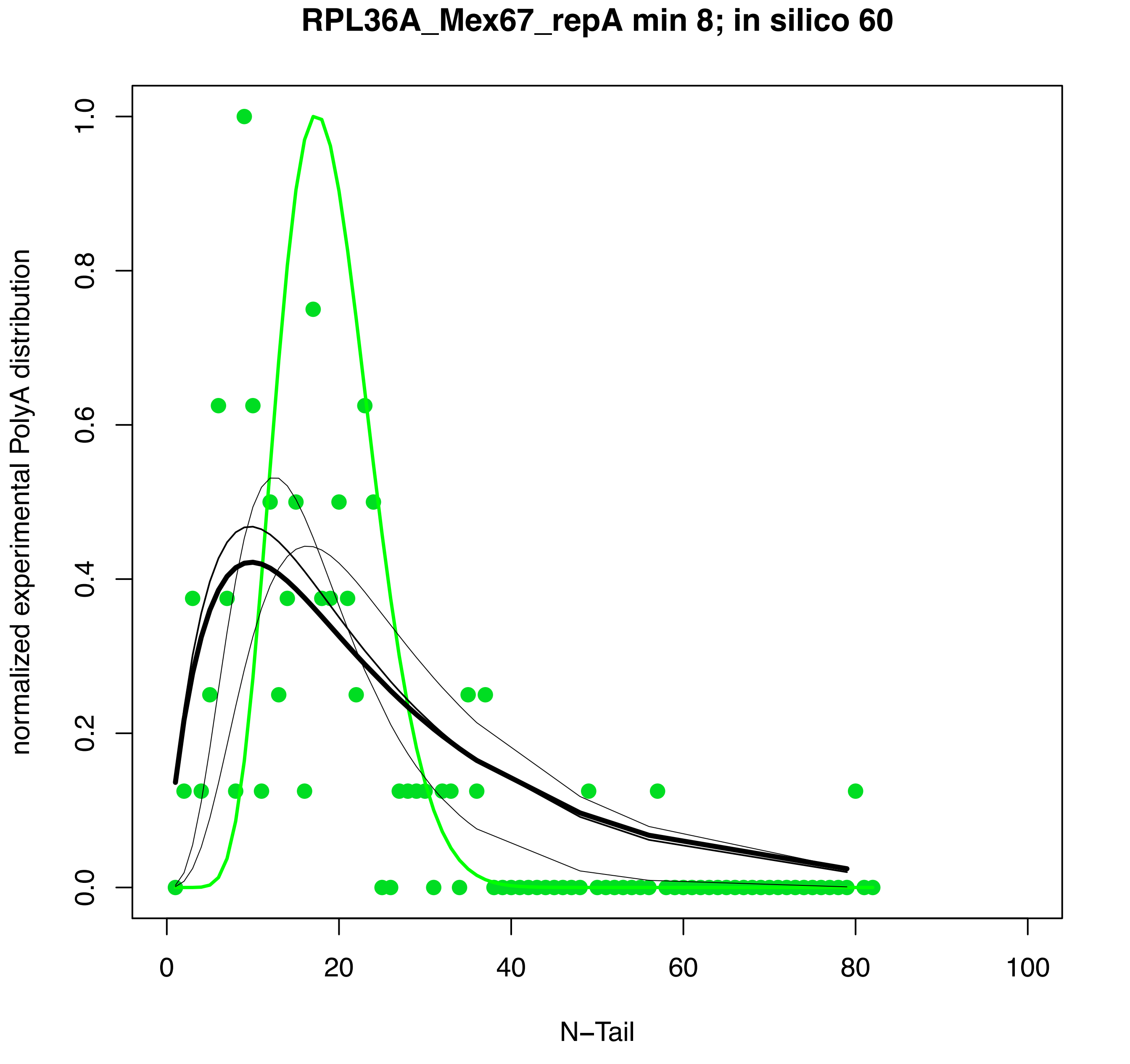

RPL36A_Mex67_repA min 8; in silico 60
normalized experimental PolyA distribution
N−Tail
0.0
0.2
0.4
0.6
0.8
1.0
0
20
40
60
80
100

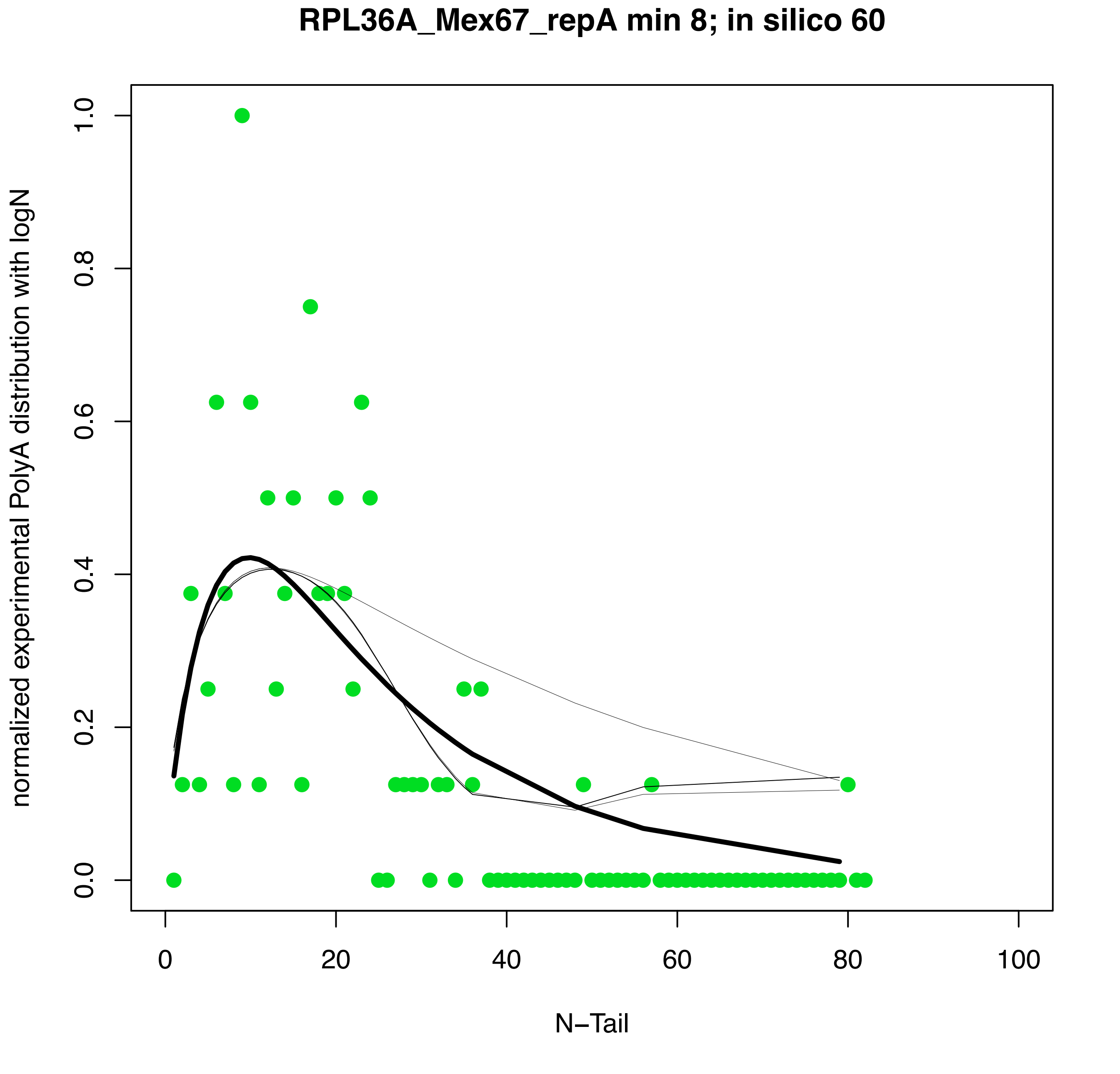

RPL36A_Mex67_repA min 8; in silico 60
normalized experimental PolyA distribution with logN
N−Tail
0.0
0.2
0.4
0.6
0.8
1.0
0
20
40
60
80
100

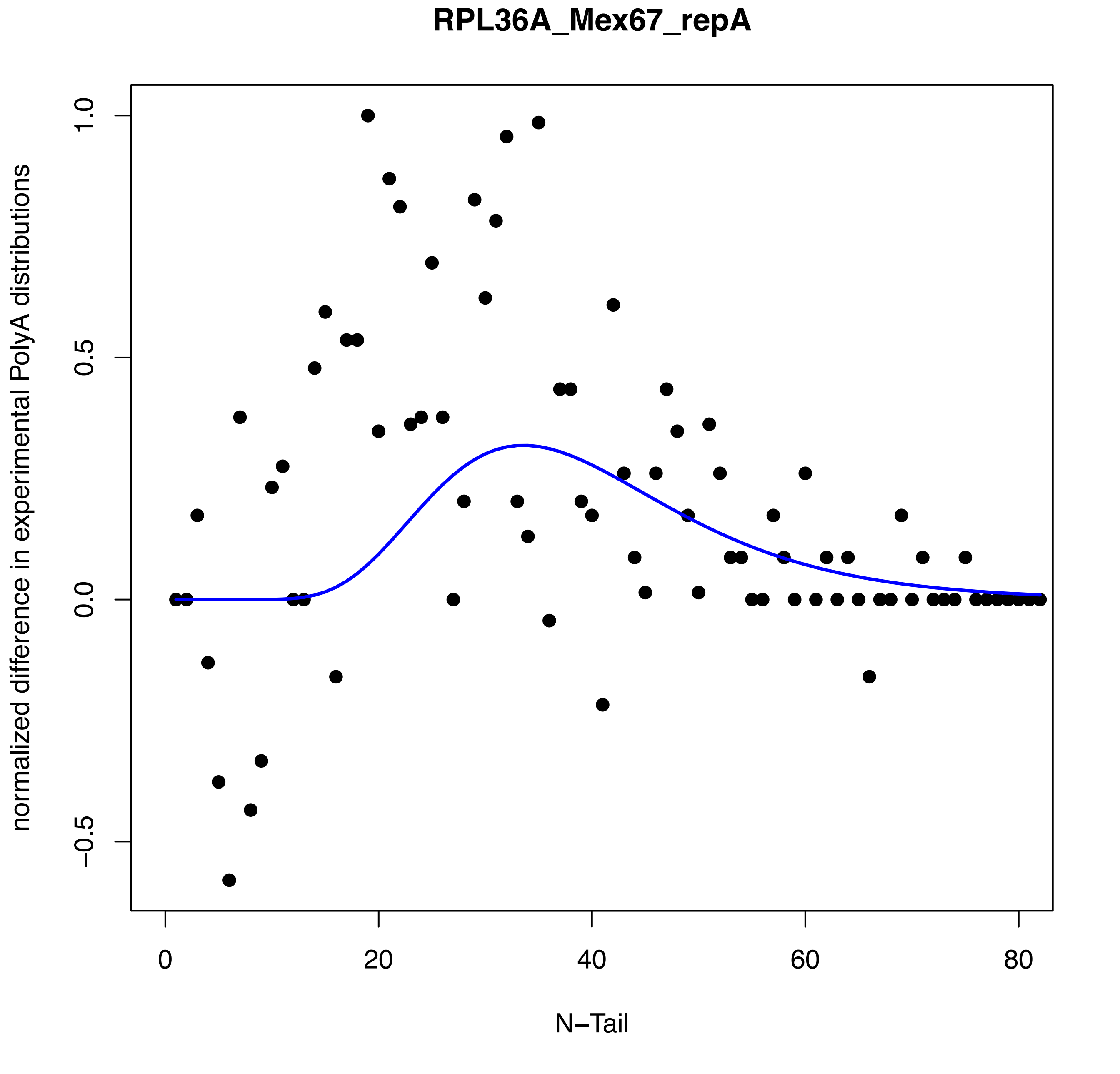

RPL36A_Mex67_repA
normalized difference in experimental PolyA distributions
N−Tail
1.0
0.5
0.0
−0.5
0
20
40
60
80

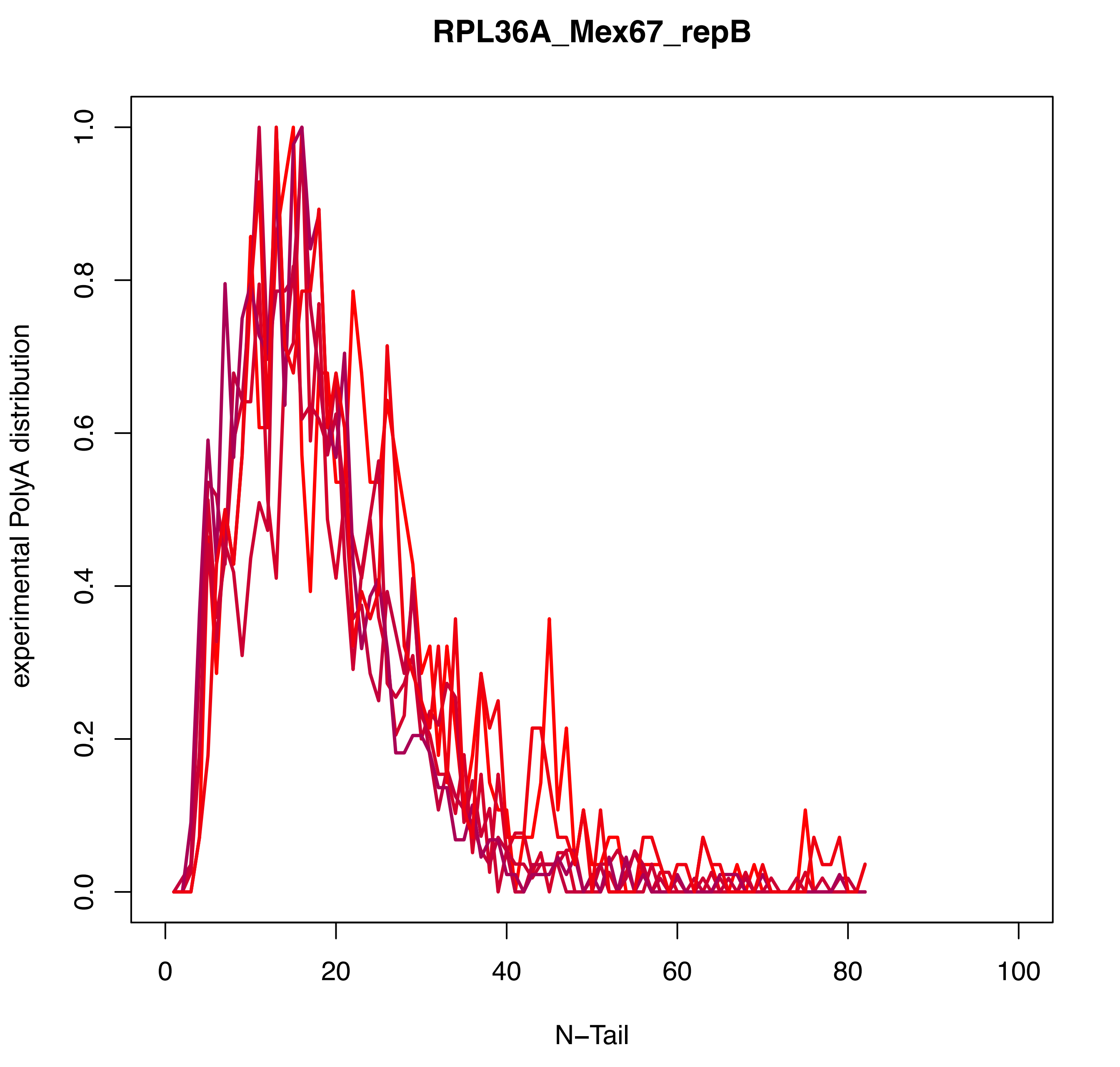

RPL36A_Mex67_repB
experimental PolyA distribution
N−Tail
0.0
0.2
0.4
0.6
0.8
1.0
0
20
40
60
80
100

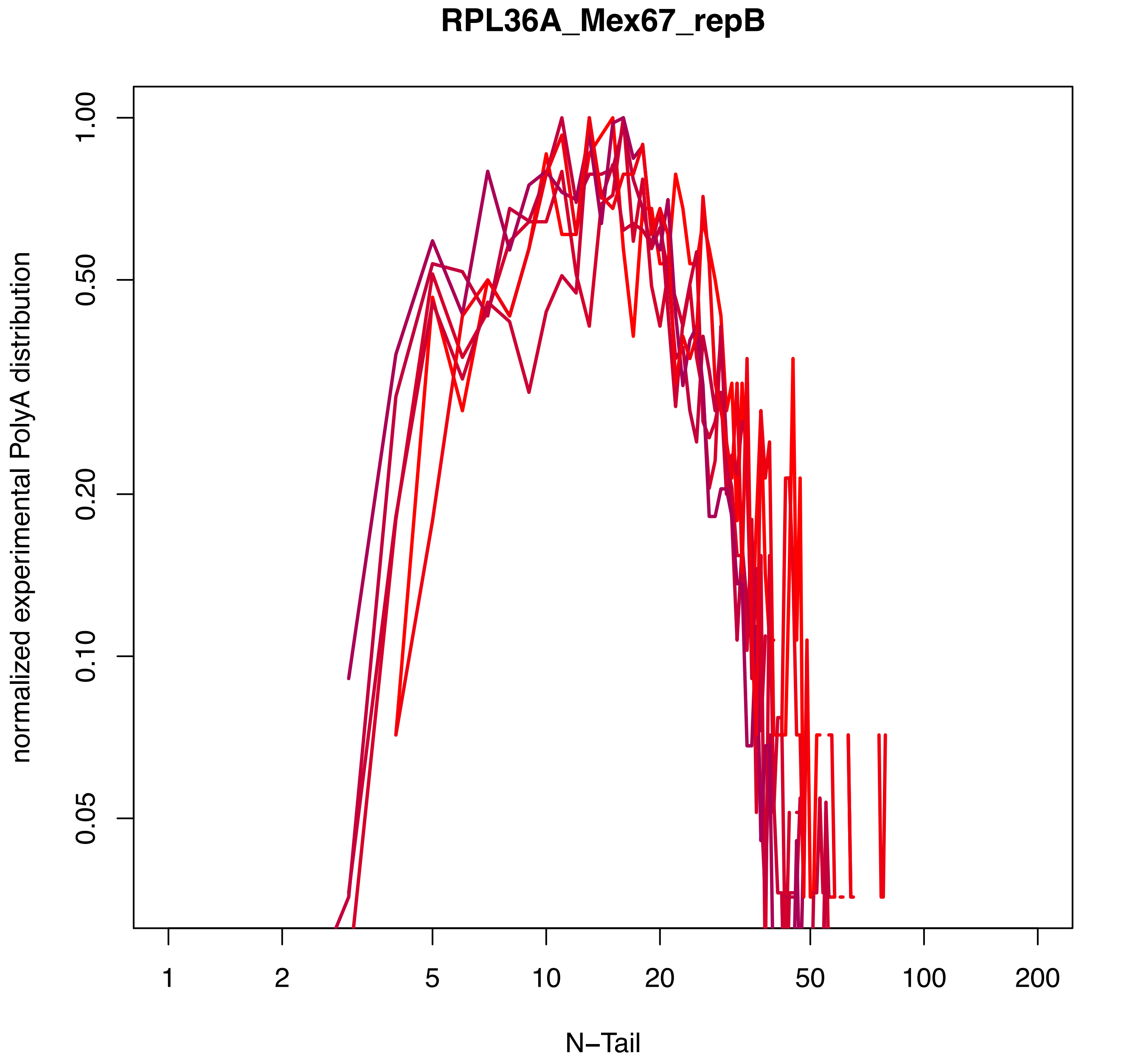

RPL36A_Mex67_repB
normalized experimental PolyA distribution
1.00
0.50
0.20
0.10
0.05
N−Tail
1
2
5
10
20
50
100
200

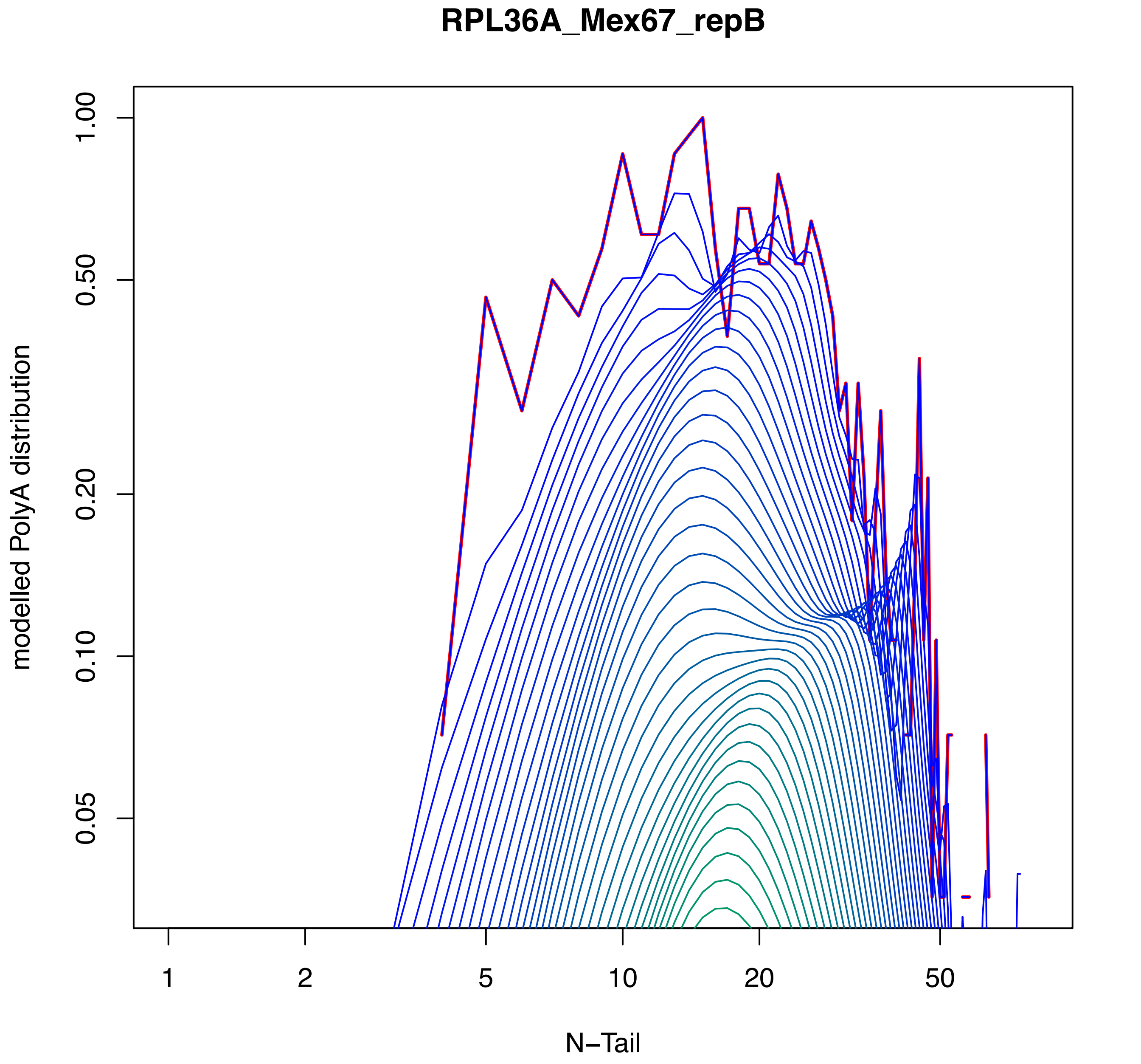

RPL36A_Mex67_repB
modelled PolyA distribution
N−Tail
1.00
0.50
0.20
0.10
0.05
1
2
5
10
20
50

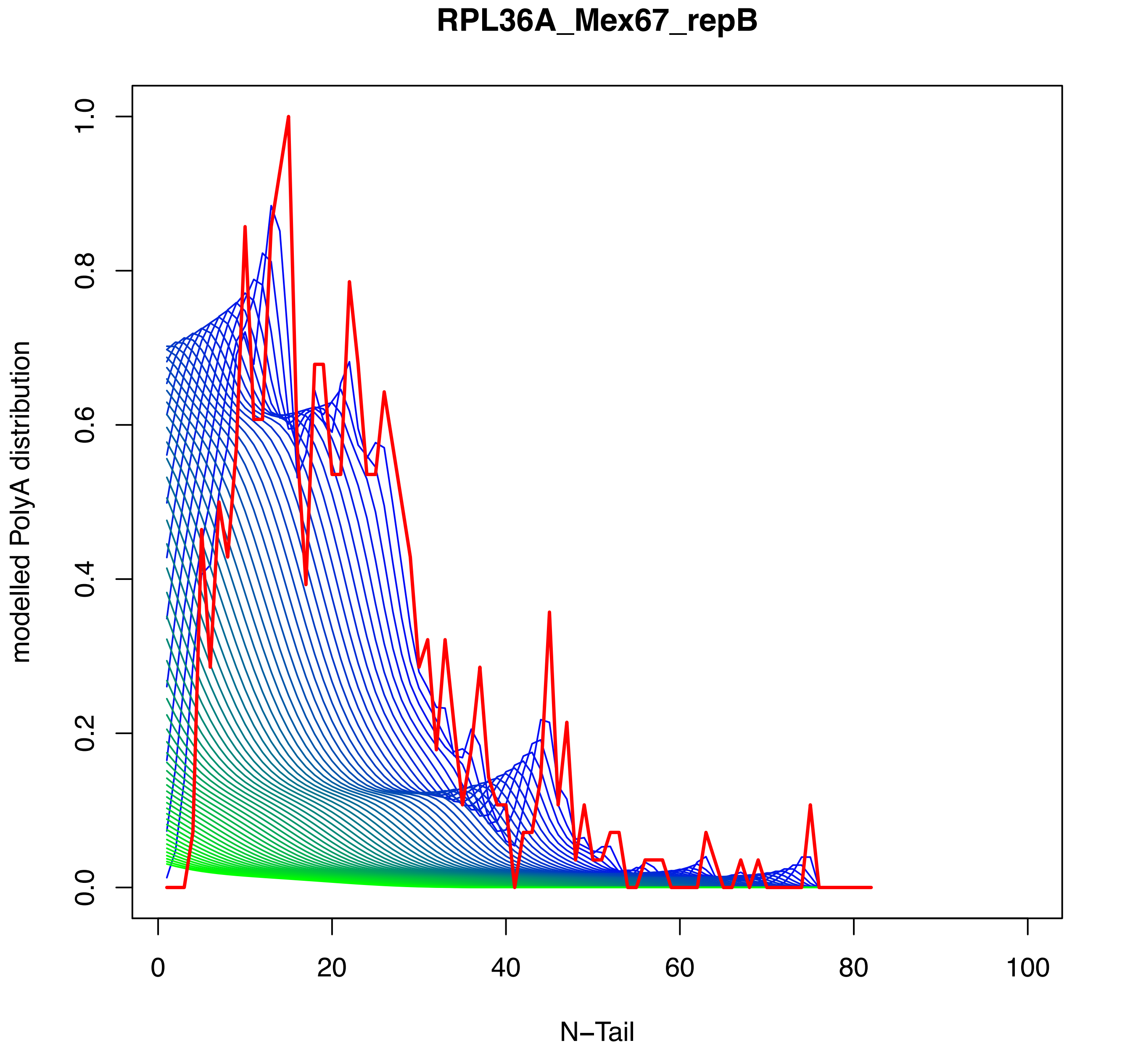

RPL36A_Mex67_repB
modelled PolyA distribution
N−Tail
0.0
0.2
0.4
0.6
0.8
1.0
0
20
40
60
80
100

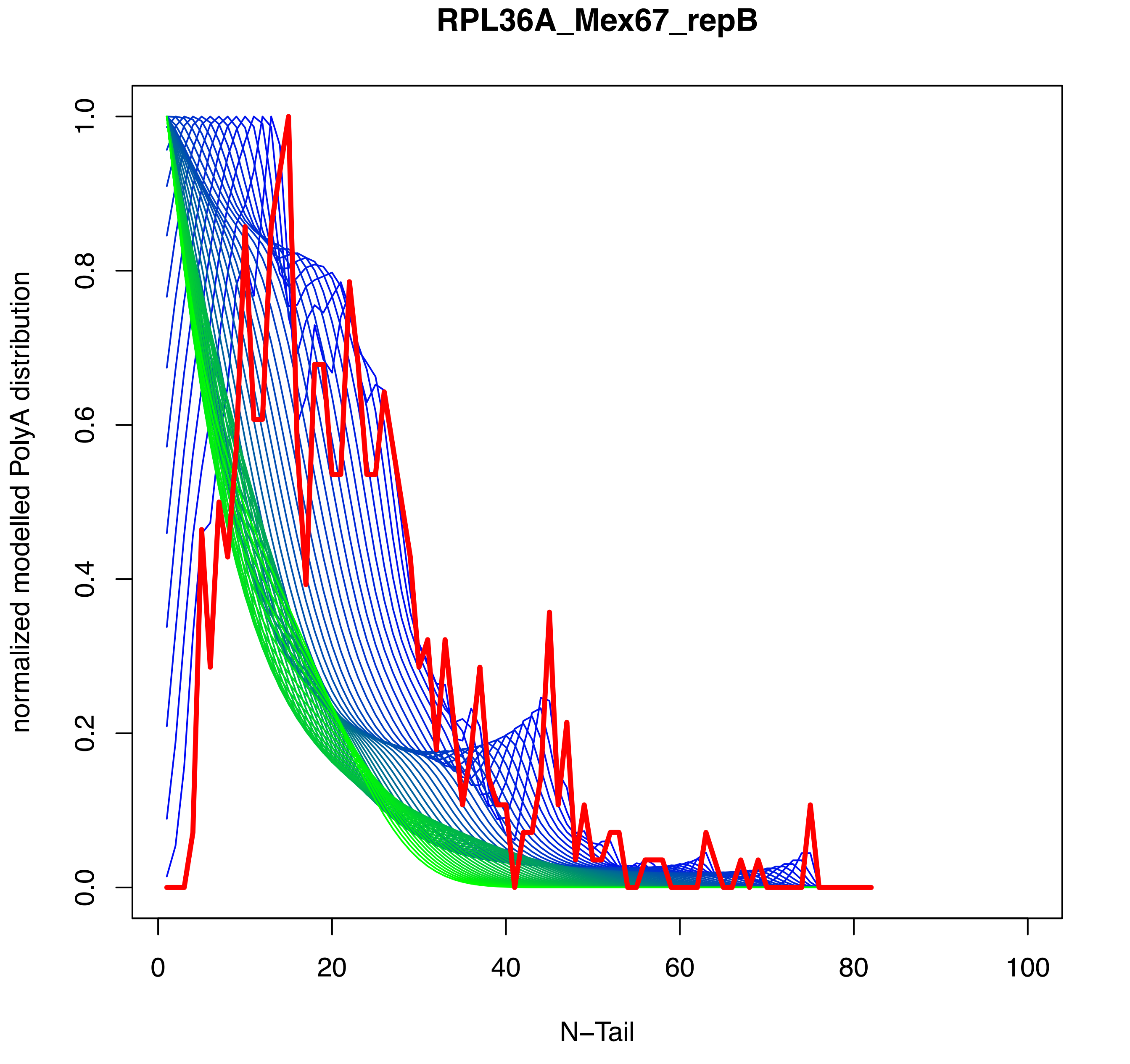

RPL36A_Mex67_repB
normalized modelled PolyA distribution
N−Tail
0.0
0.2
0.4
0.6
0.8
1.0
0
20
40
60
80
100

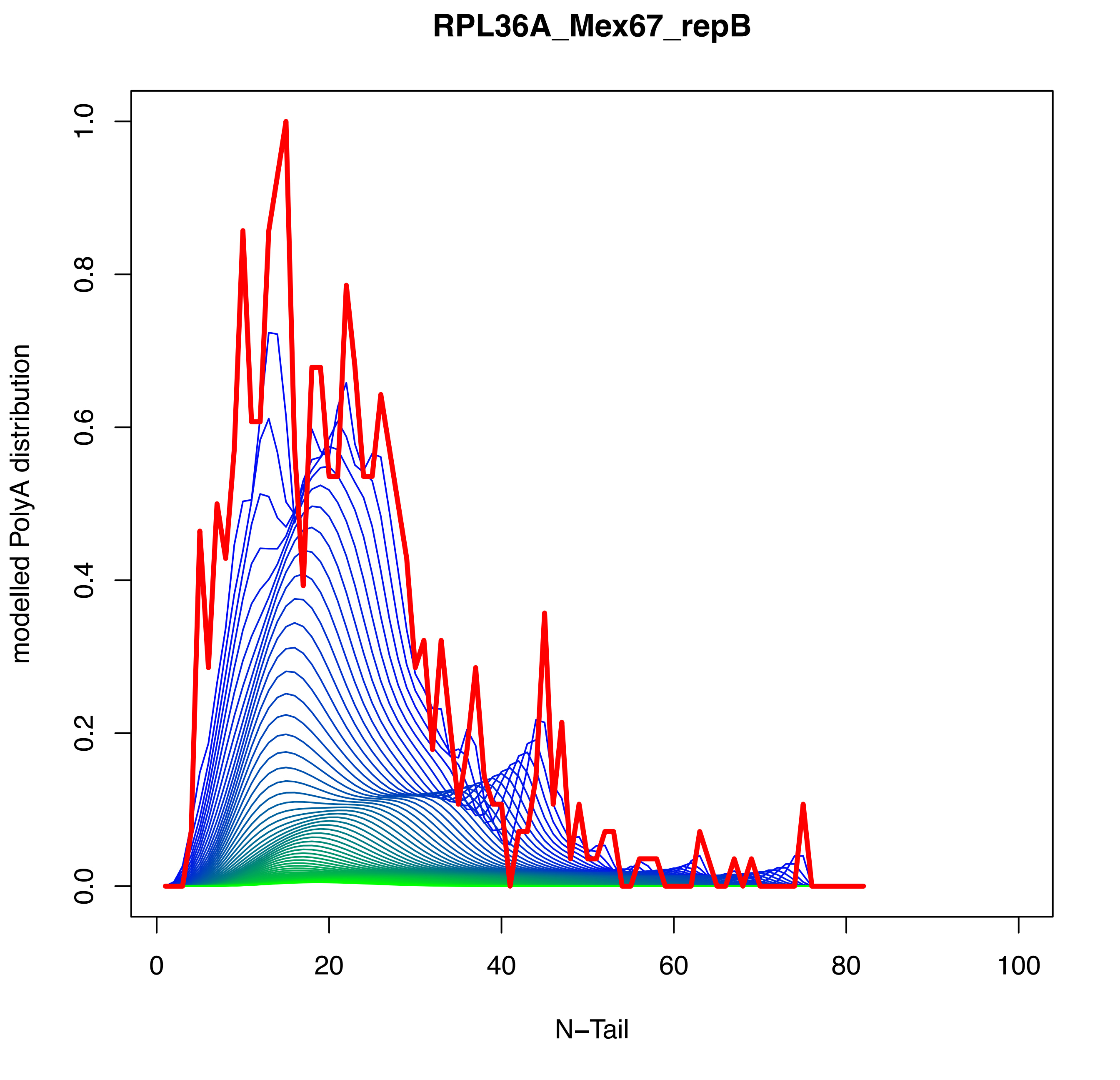

RPL36A_Mex67_repB
modelled PolyA distribution
0.0
0.2
0.4
0.6
0.8
1.0
0
20
40
60
80
100
N−Tail

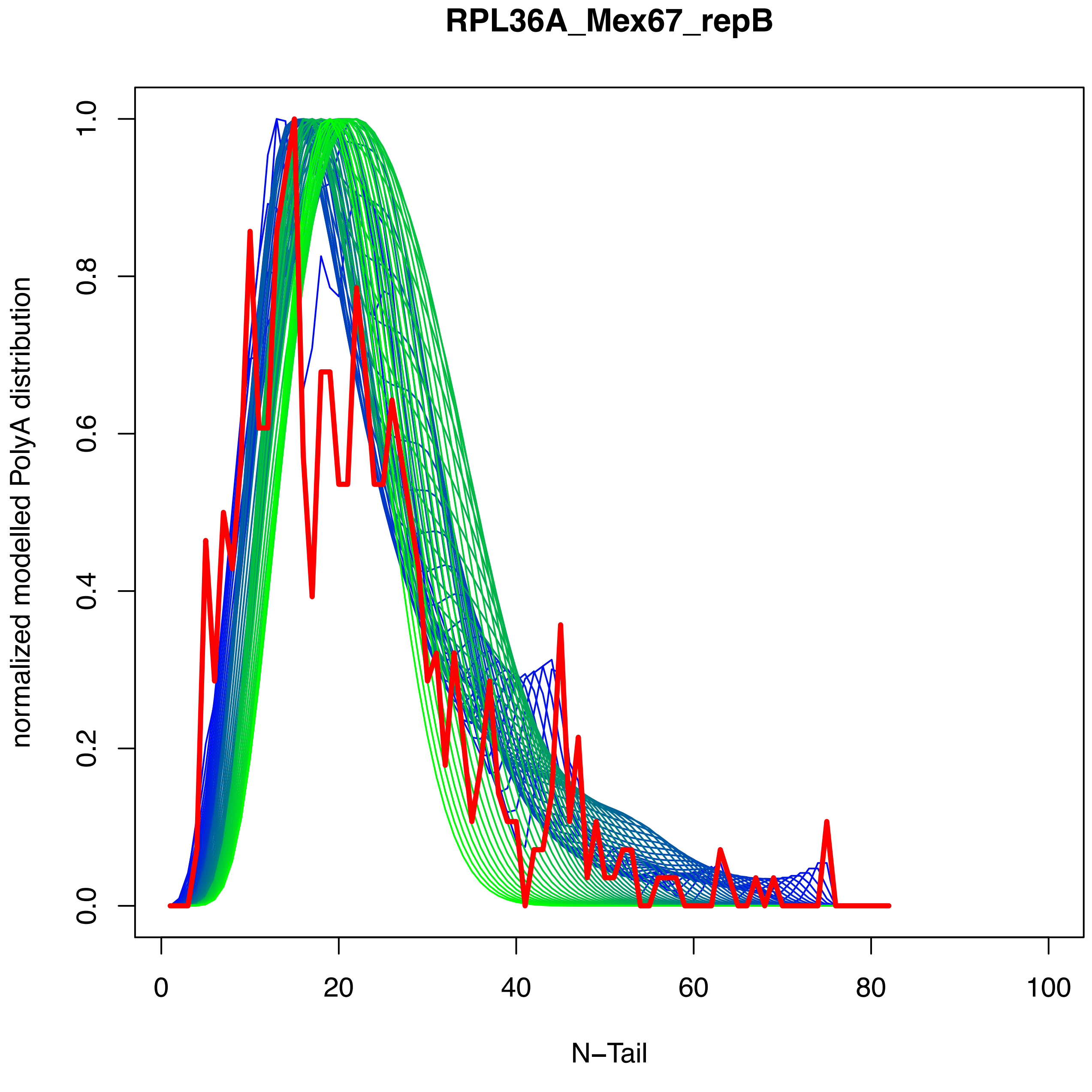

RPL36A_Mex67_repB
normalized modelled PolyA distribution
N−Tail
0.0
0.2
0.4
0.6
0.8
1.0
0
20
40
60
80
100

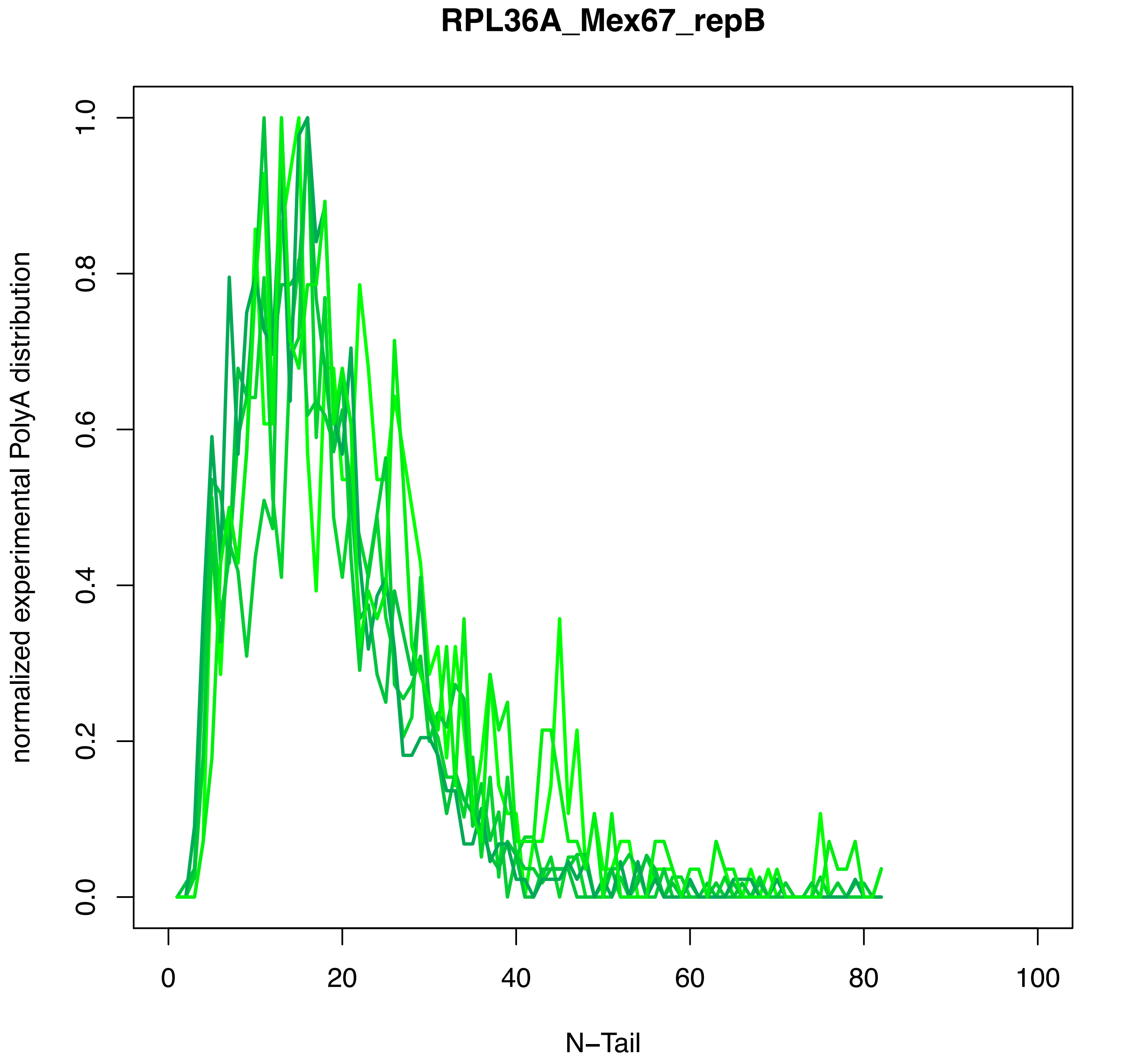
RPL36A_Mex67_repB
normalized experimental PolyA distribution
N−Tail
0.0
0.2
0.4
0.6
0.8
1.0
0
20
40
60
80
100

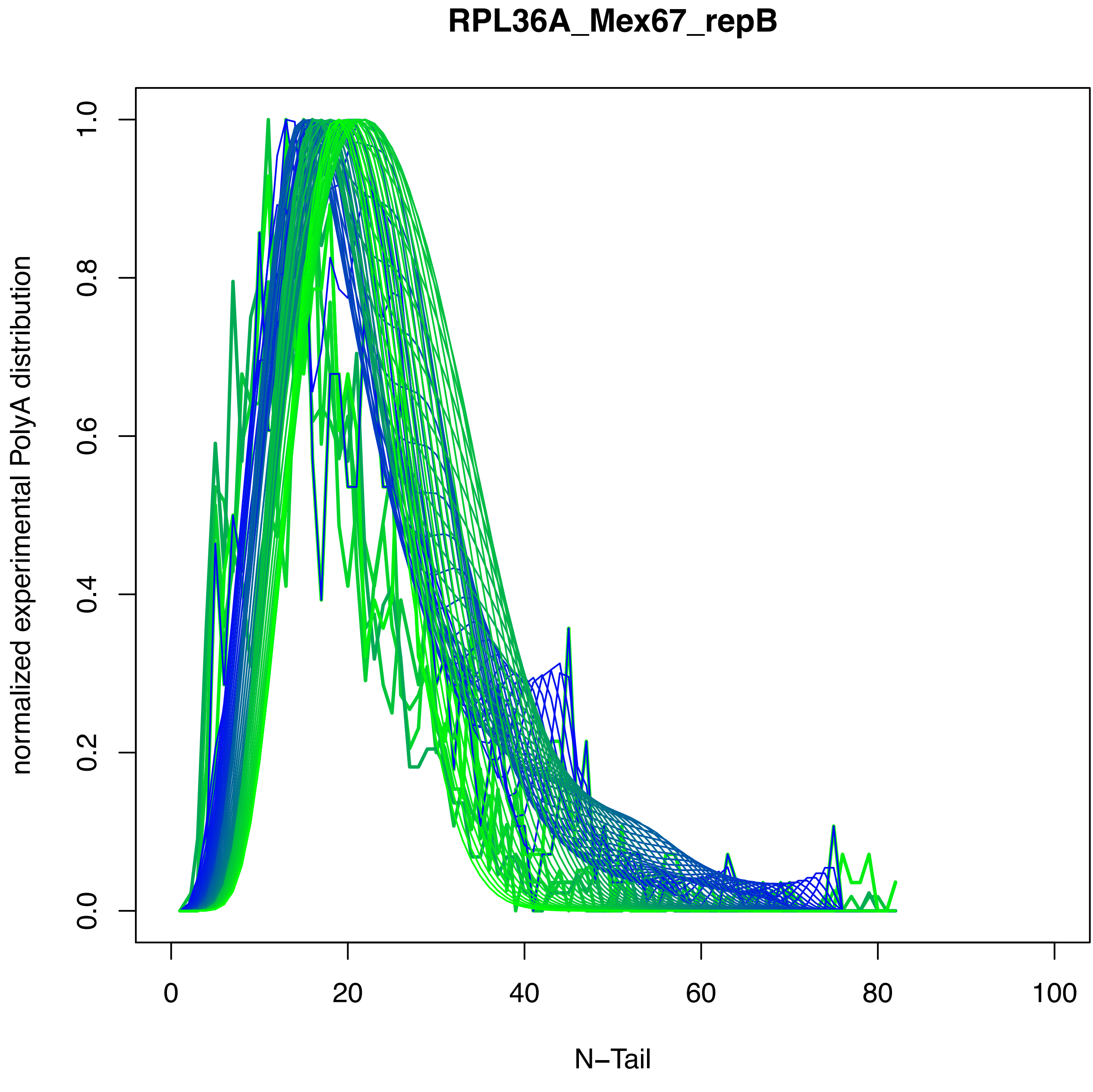
RPL36A_Mex67_repB
normalized experimental PolyA distribution
N−Tail
0.0
0.2
0.4
0.6
0.8
1.0
0
20
40
60
80
100

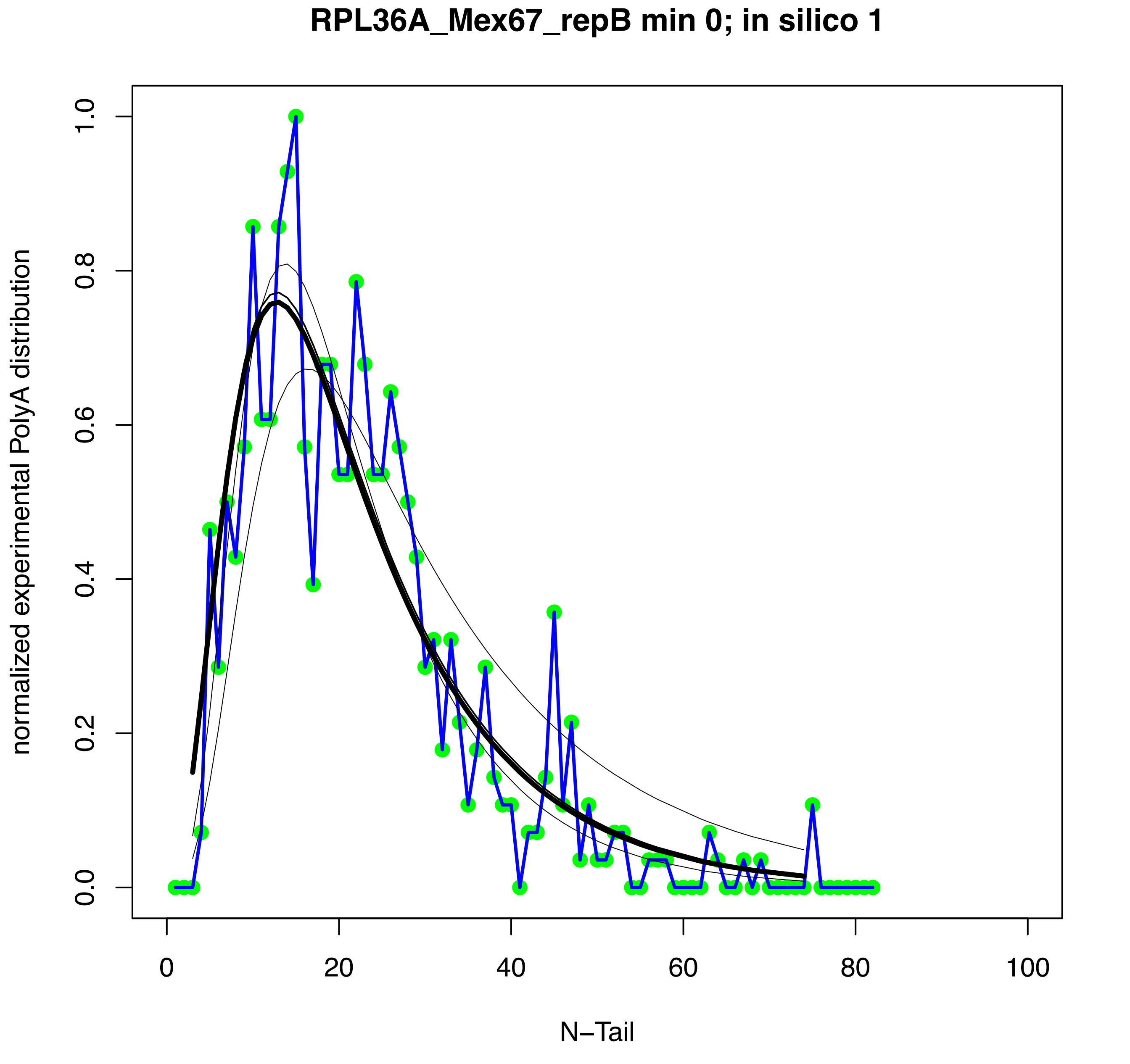

RPL36A_Mex67_repB min 0; in silico 1
normalized experimental PolyA distribution
N−Tail
0.0
0.2
0.4
0.6
0.8
1.0
0
20
40
60
80
100

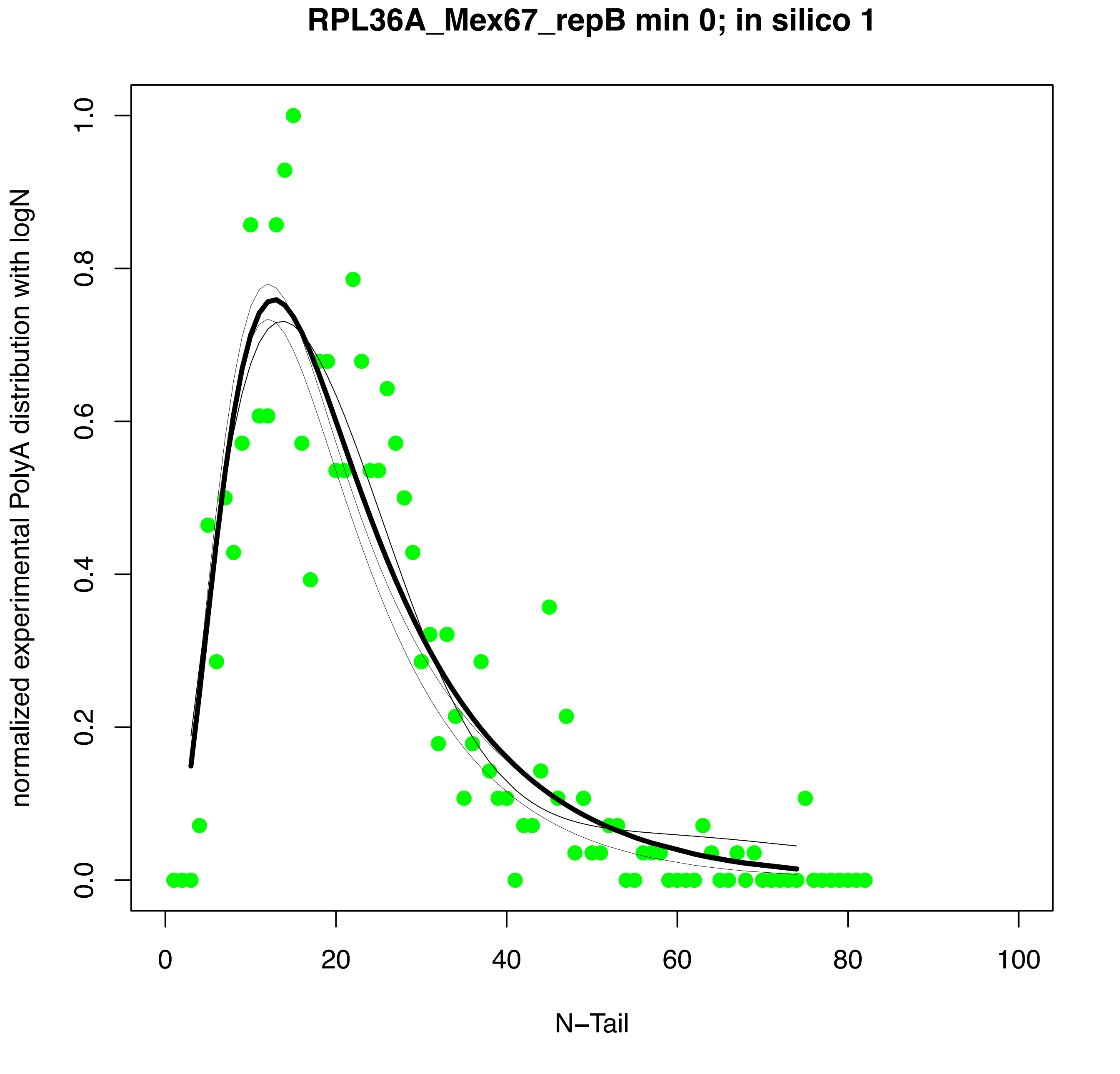

RPL36A_Mex67_repB min 0; in silico 1
normalized experimental PolyA distribution with logN
N−Tail
0.0
0.2
0.4
0.6
0.8
1.0
0
20
40
60
80
100

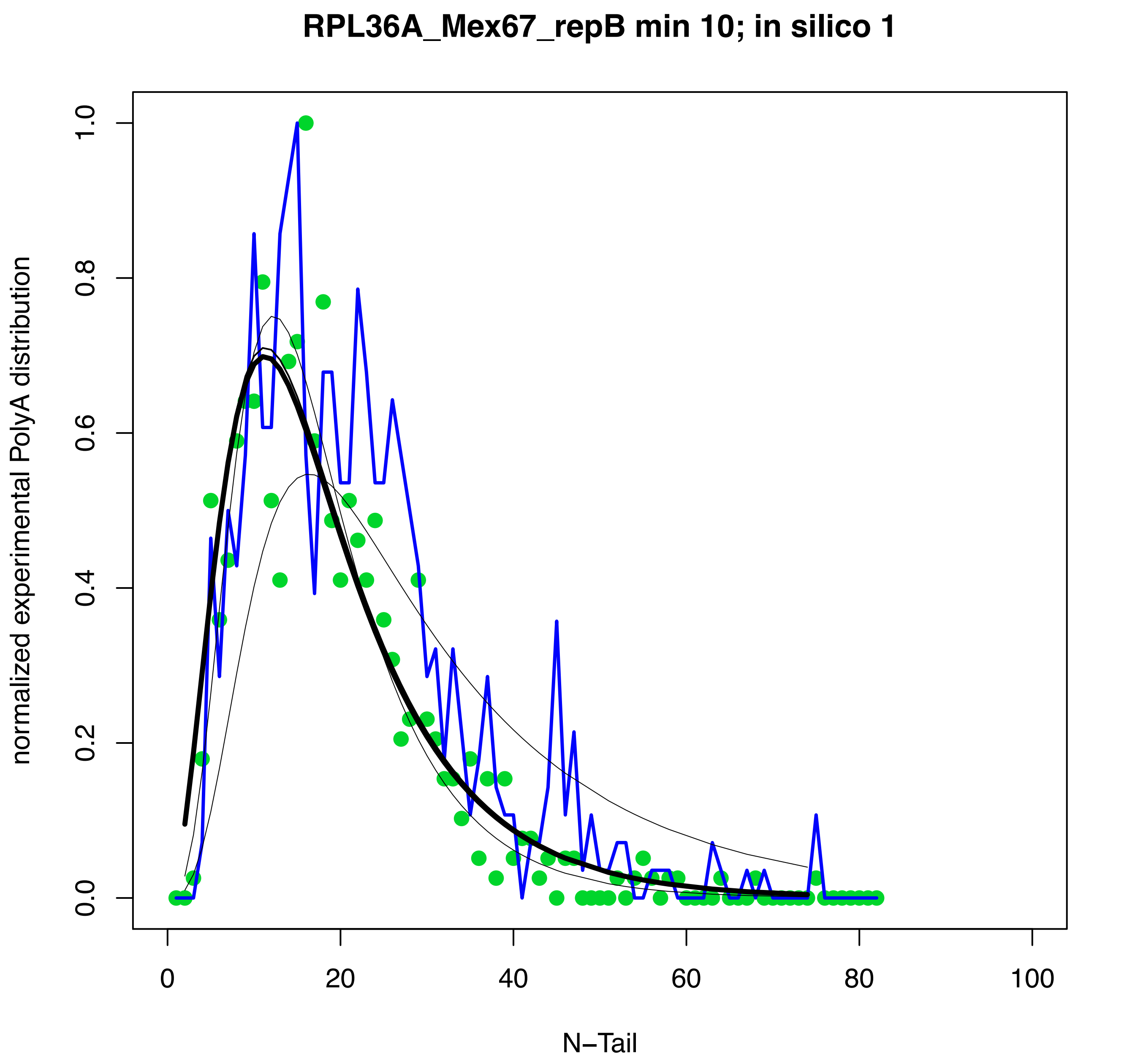
RPL36A_Mex67_repB min 10; in silico 1
normalized experimental PolyA distribution
N−Tail
0.0
0.2
0.4
0.6
0.8
1.0
0
20
40
60
80
100

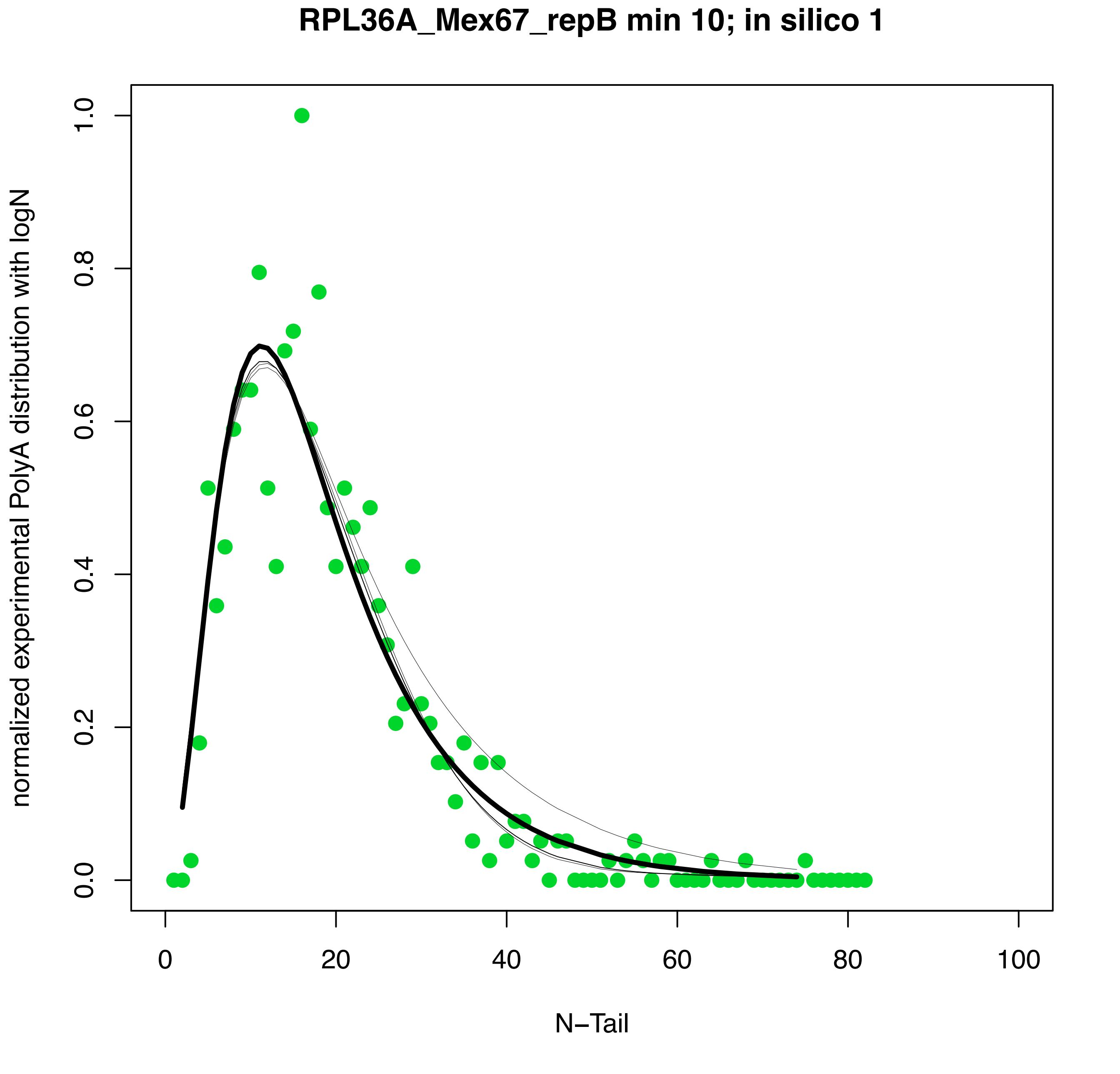

RPL36A_Mex67_repB min 10; in silico 1
normalized experimental PolyA distribution with logN
N−Tail
0.0
0.2
0.4
0.6
0.8
1.0
0
20
40
60
80
100

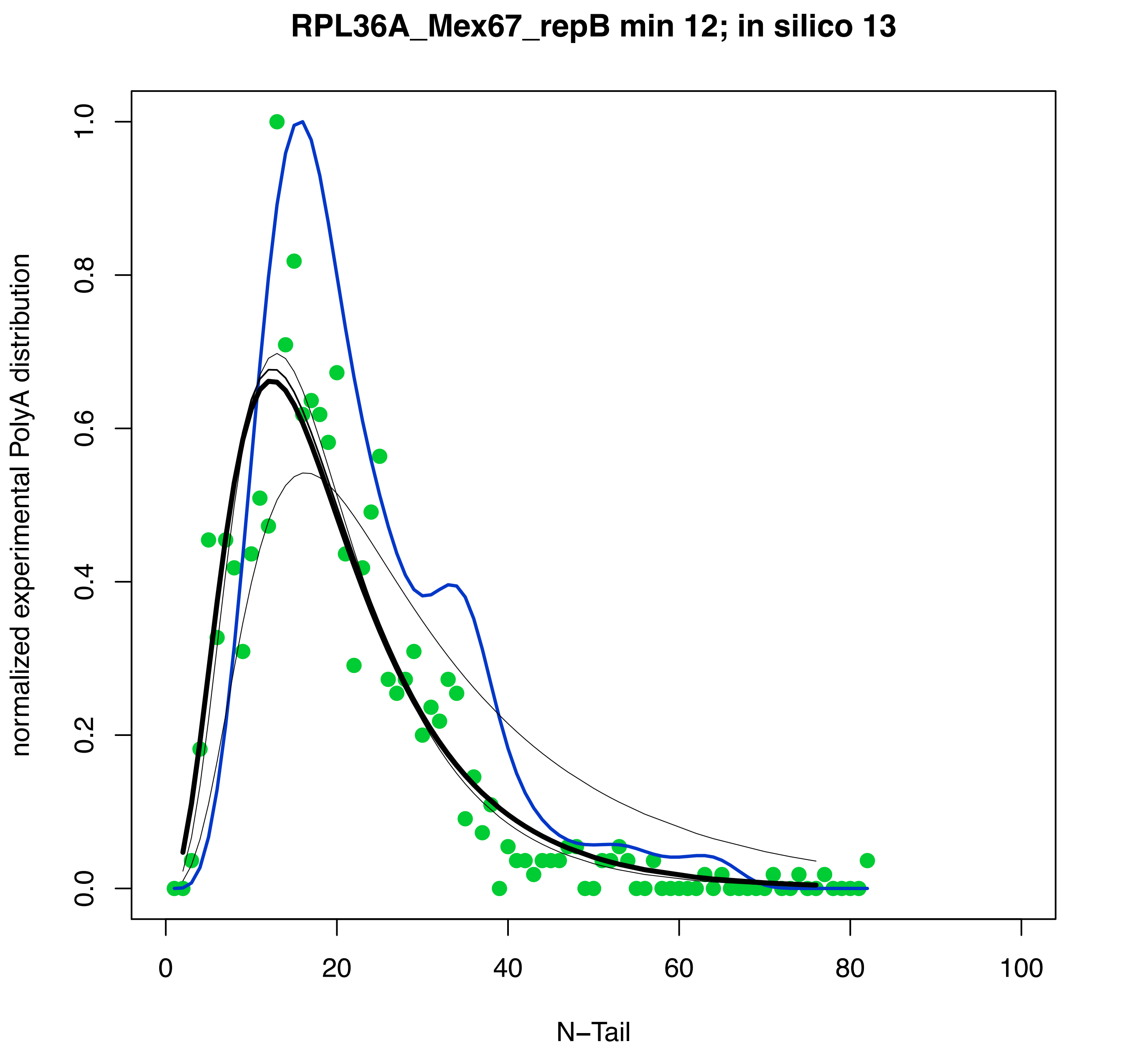
RPL36A_Mex67_repB min 12; in silico 13
normalized experimental PolyA distribution
N−Tail
0.0
0.2
0.4
0.6
0.8
1.0
0
20
40
60
80
100

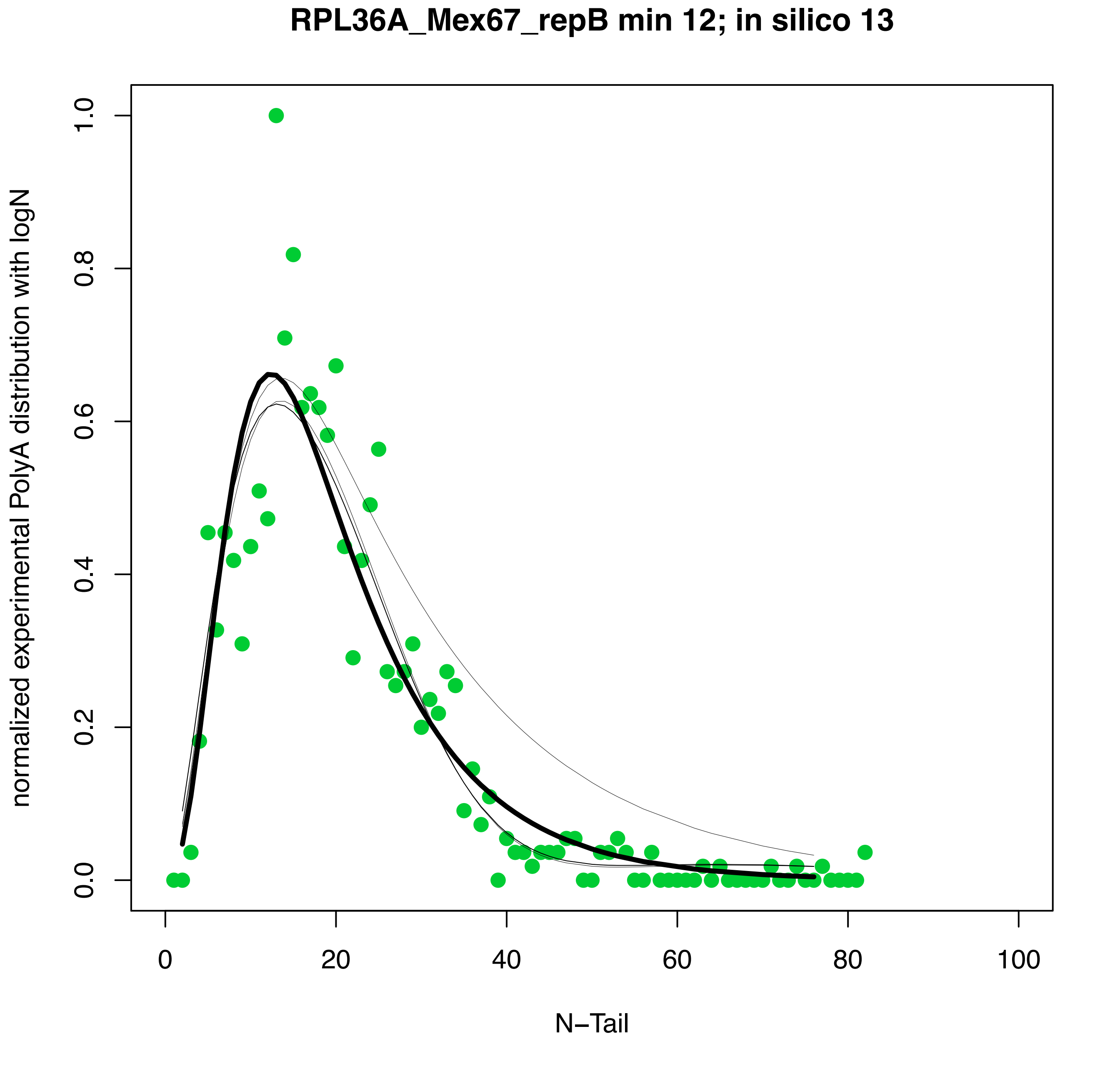
RPL36A_Mex67_repB min 12; in silico 13
normalized experimental PolyA distribution with logN
N−Tail
0.0
0.2
0.4
0.6
0.8
1.0
0
20
40
60
80
100

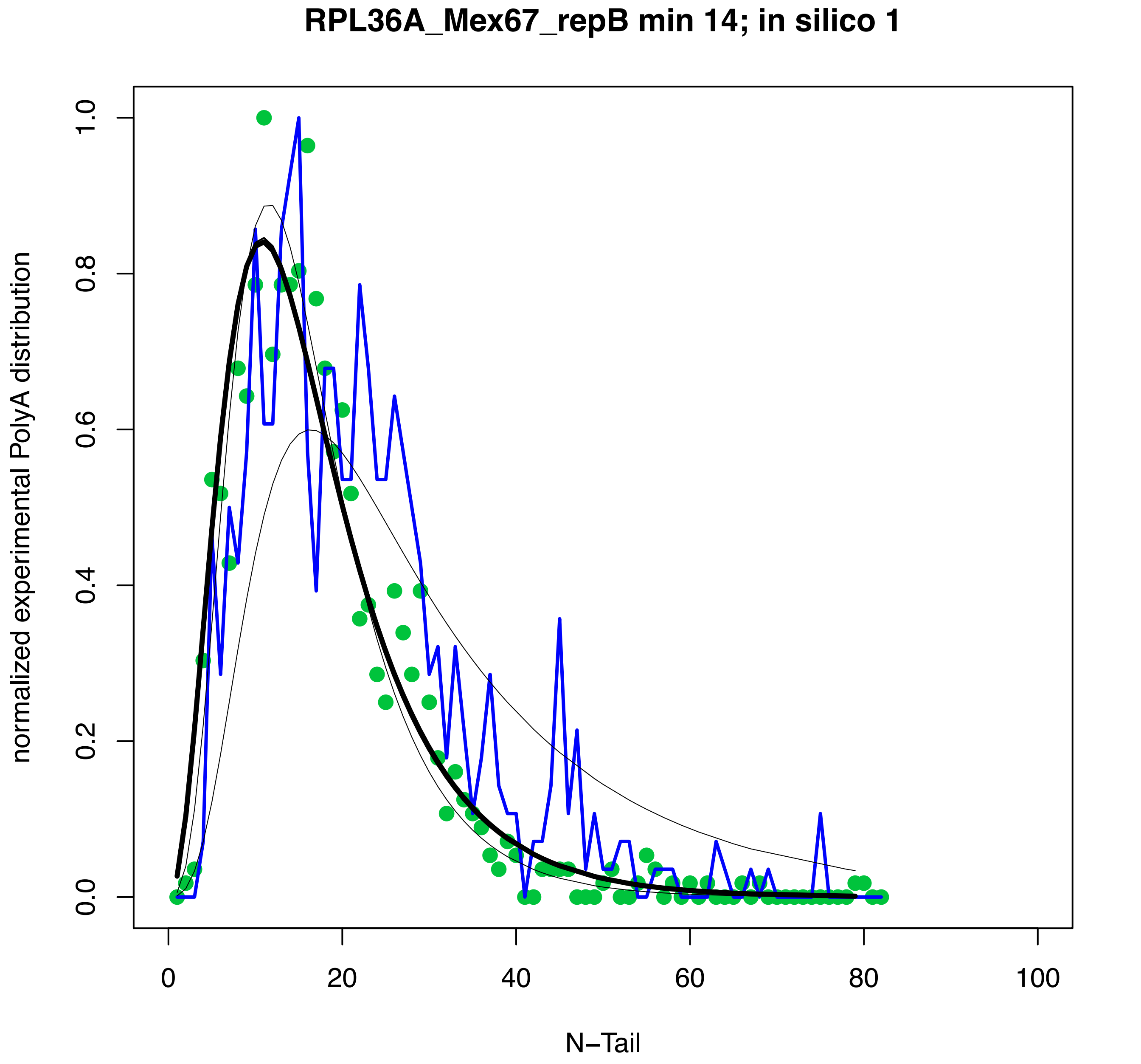

RPL36A_Mex67_repB min 14; in silico 1
normalized experimental PolyA distribution
0.0
0.2
0.4
0.6
0.8
1.0
0
20
40
60
80
100
N−Tail

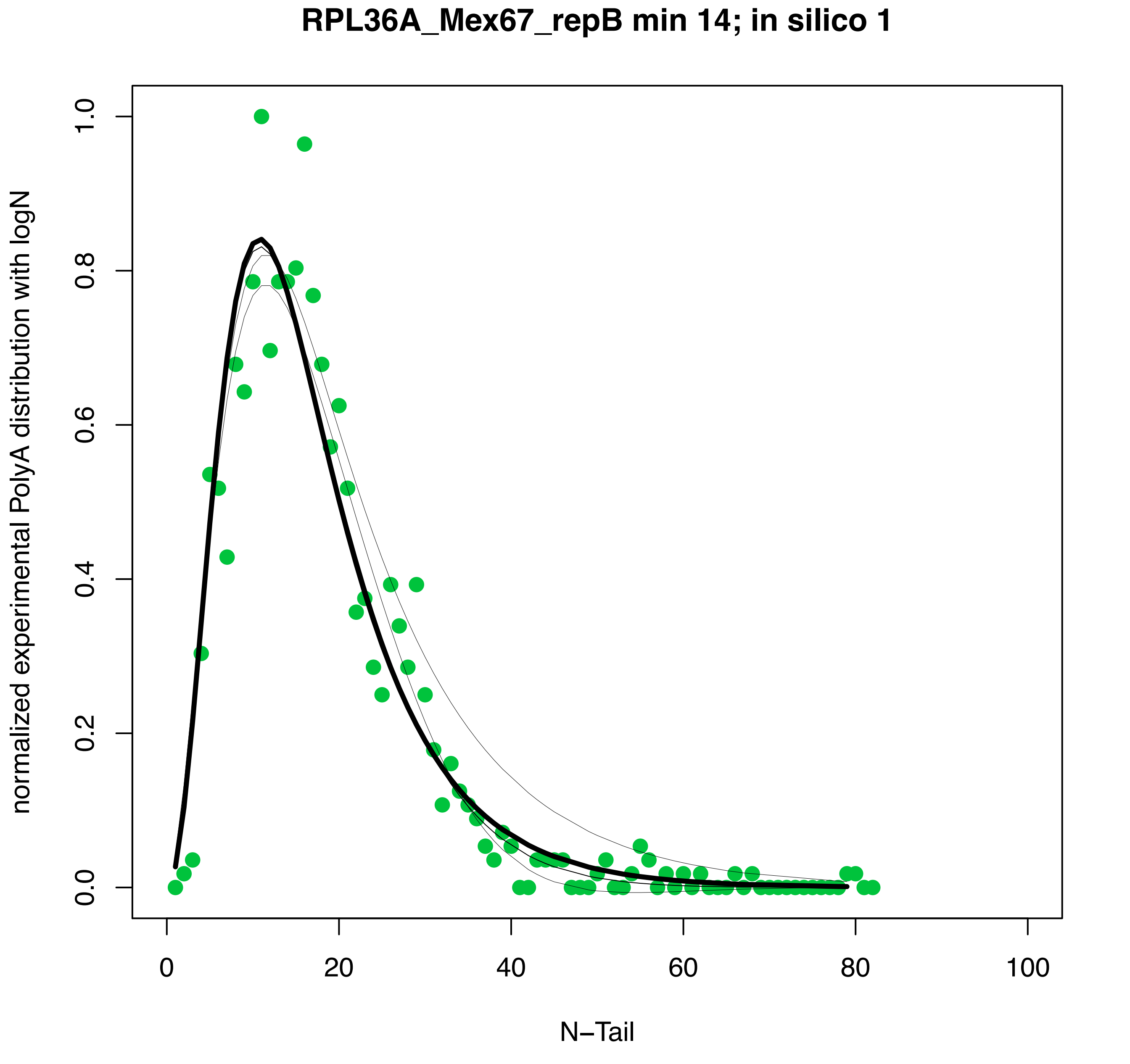
RPL36A_Mex67_repB min 14; in silico 1
normalized experimental PolyA distribution with logN
N−Tail
0.0
0.2
0.4
0.6
0.8
1.0
0
20
40
60
80
100

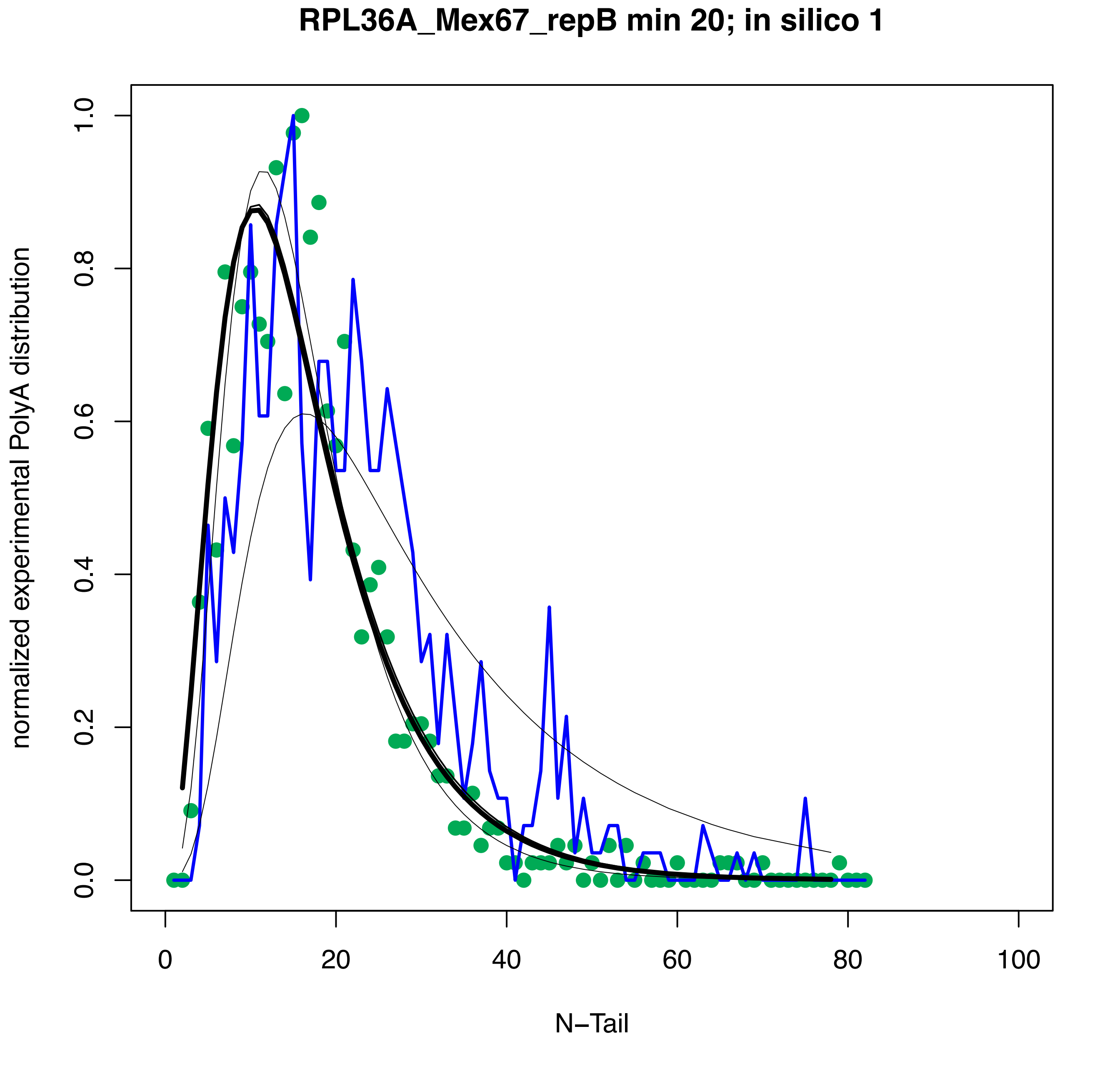

RPL36A_Mex67_repB min 20; in silico 1
normalized experimental PolyA distribution
N−Tail
0.0
0.2
0.4
0.6
0.8
1.0
0
20
40
60
80
100

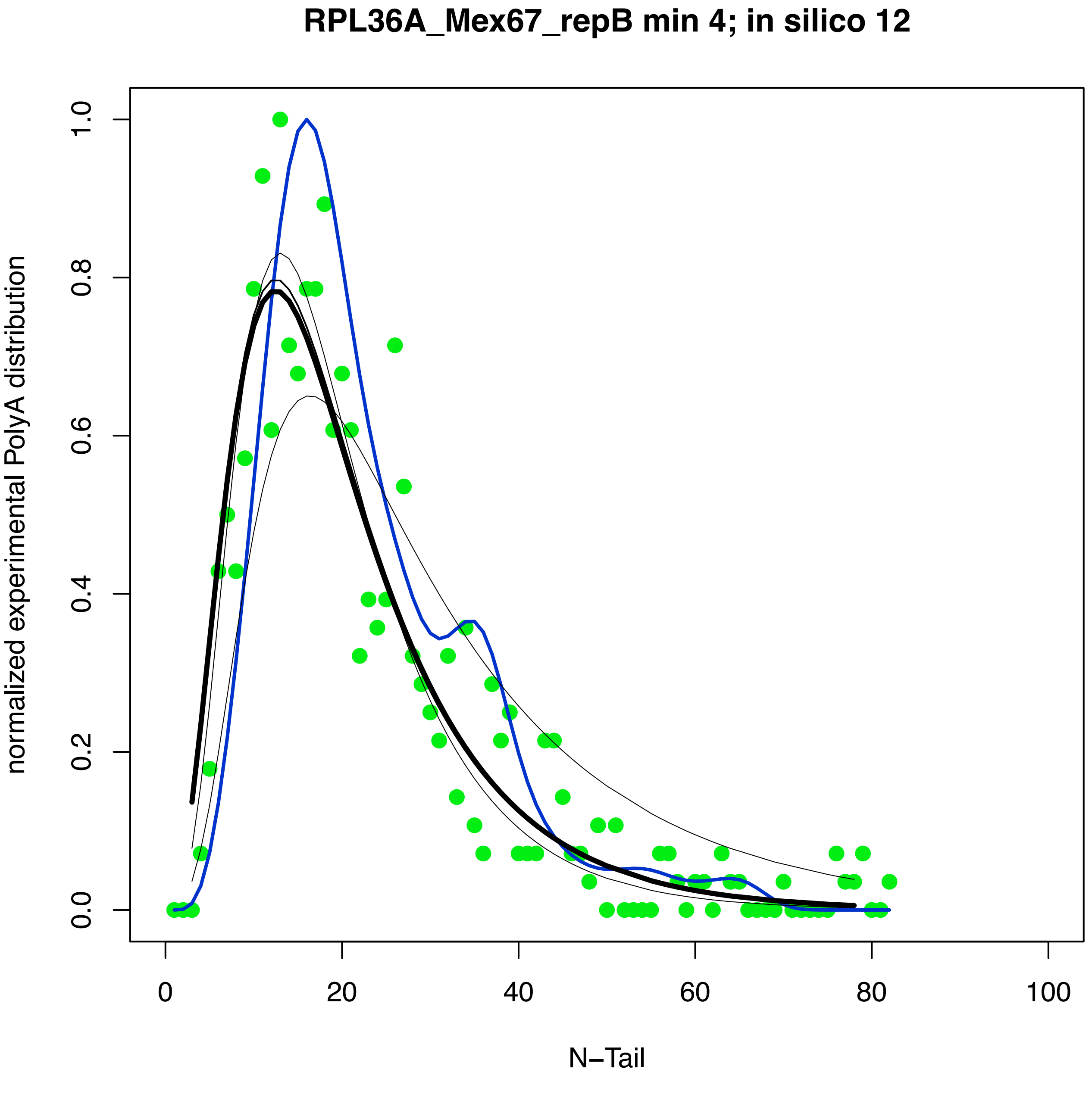

RPL36A_Mex67_repB min 4; in silico 12
normalized experimental PolyA distribution
N−Tail
0.0
0.2
0.4
0.6
0.8
1.0
0
20
40
60
80
100

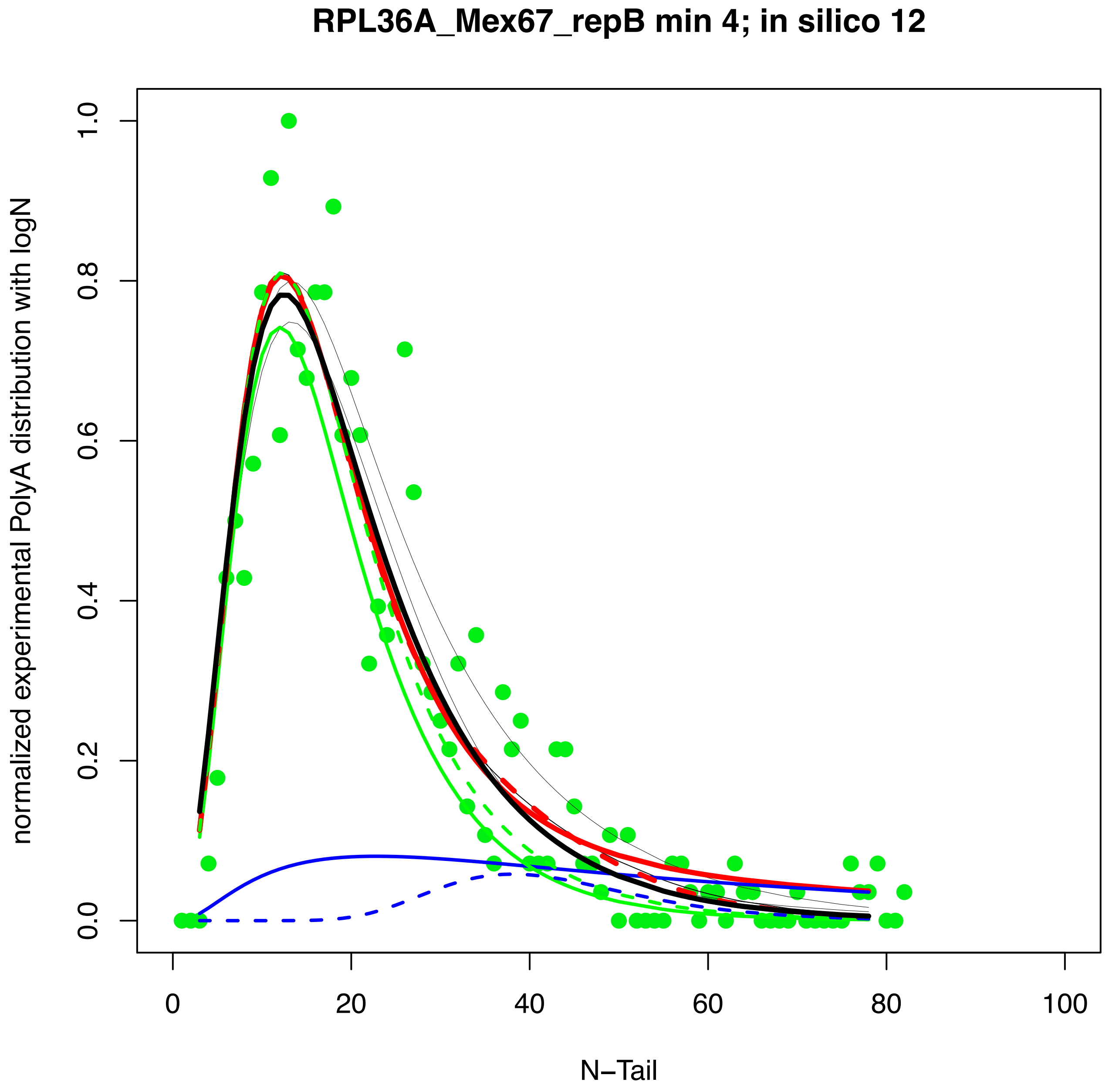

RPL36A_Mex67_repB min 4; in silico 12
normalized experimental PolyA distribution with logN
N−Tail
0.0
0.2
0.4
0.6
0.8
1.0
0
20
40
60
80
100

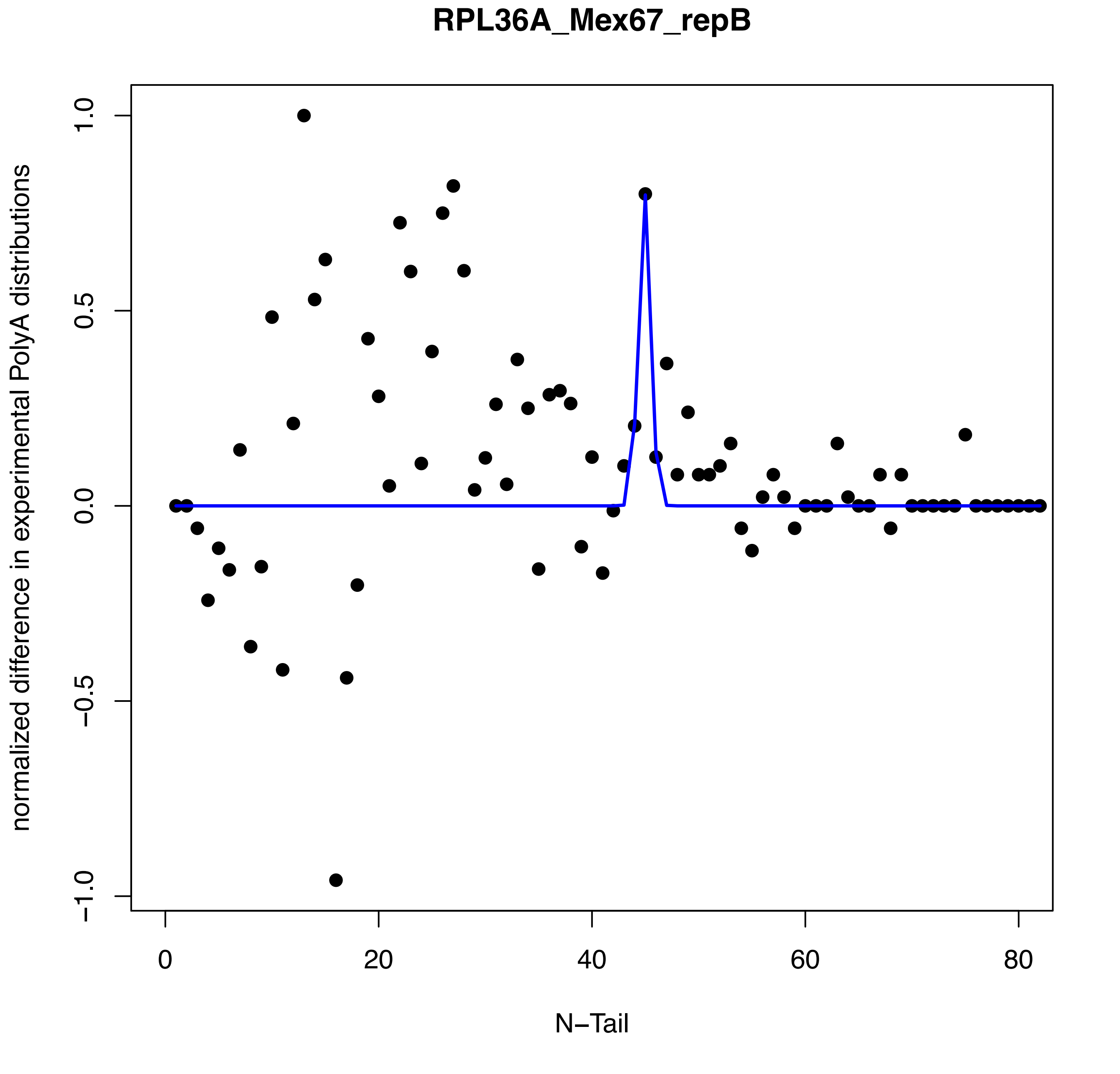

RPL36A_Mex67_repB
normalized difference in experimental PolyA distributions
N−Tail
1.0
0.5
0.0
−0.5
−1.0
0
20
40
60
80

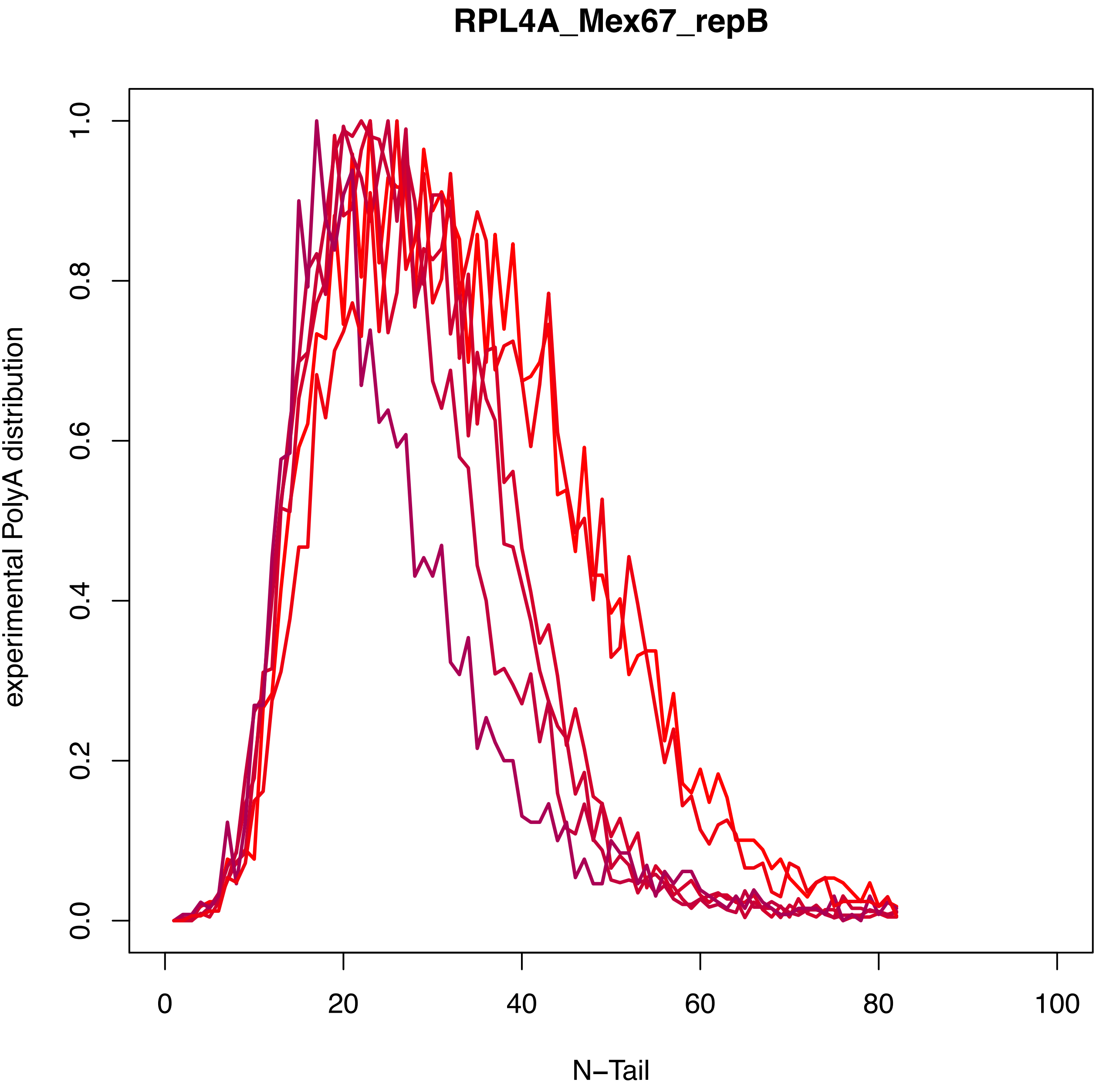

RPL4A_Mex67_repB
experimental PolyA distribution
N−Tail
0.0
0.2
0.4
0.6
0.8
1.0
0
20
40
60
80
100

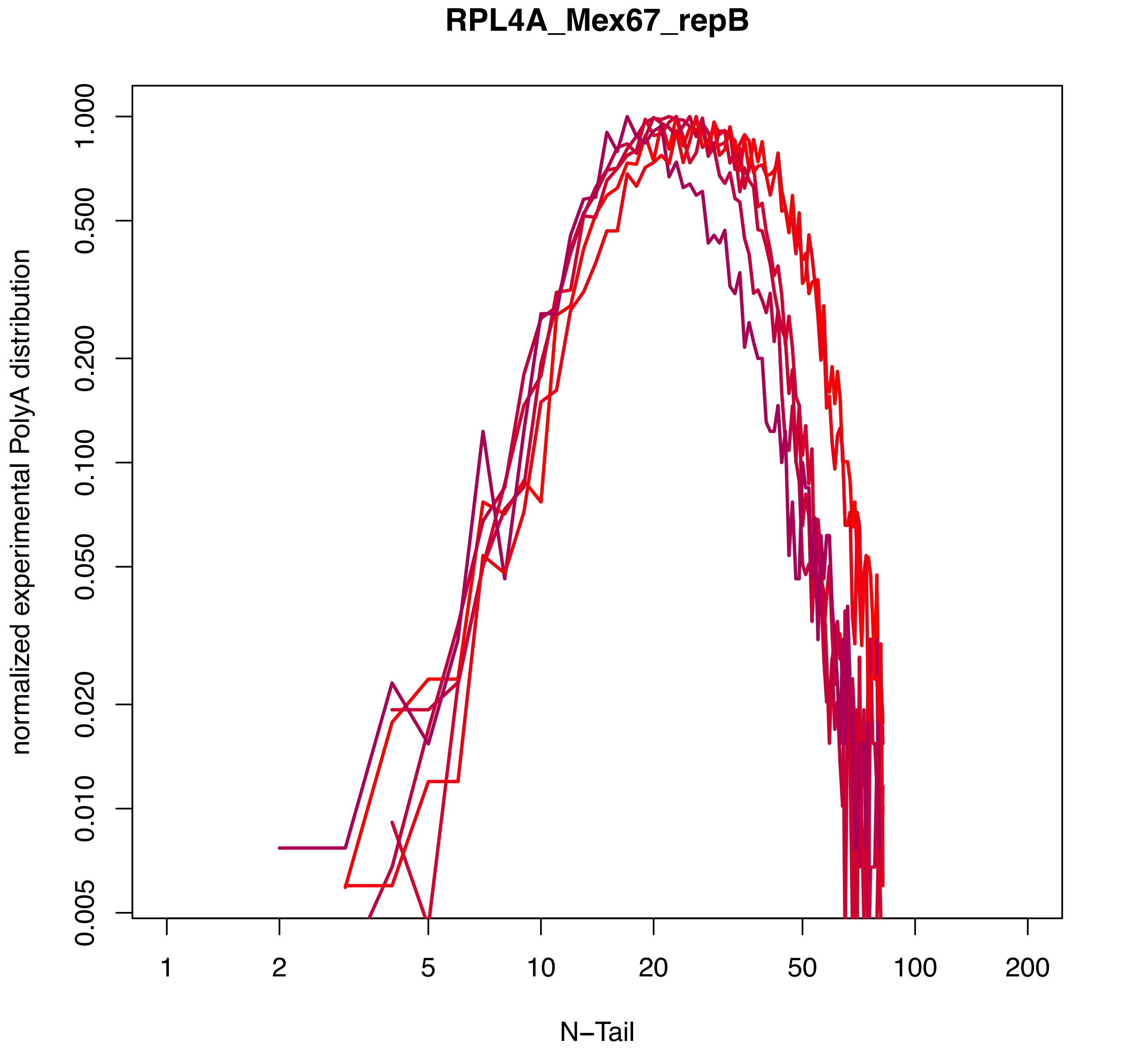

RPL4A_Mex67_repB
normalized experimental PolyA distribution
1.000
0.500
0.200
0.100
0.050
0.020
0.010
0.005
1
2
5
10
20
50
100
200
N−Tail

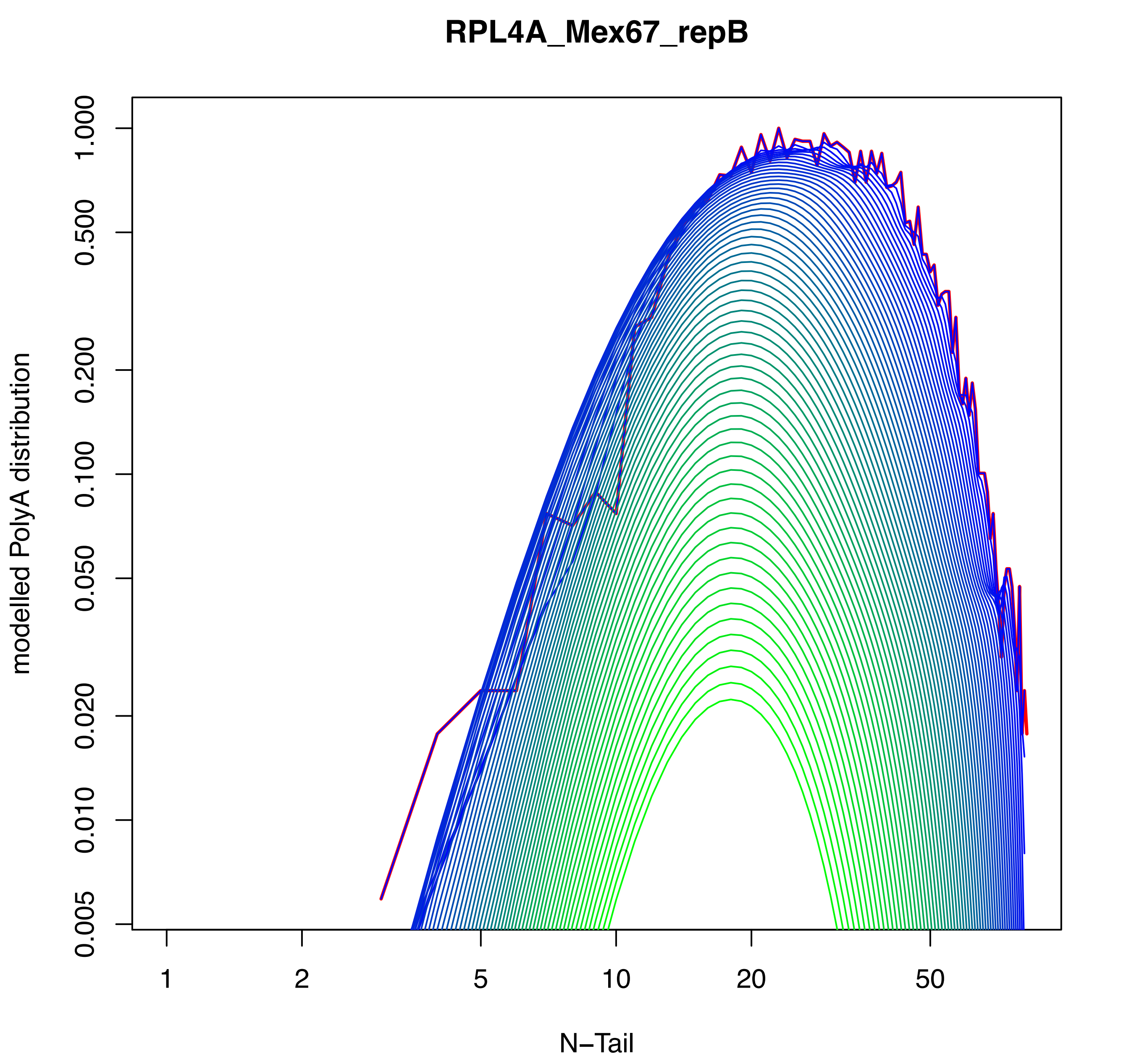

RPL4A_Mex67_repB
modelled PolyA distribution
N−Tail
1.000
0.500
0.200
0.100
0.050
0.020
0.010
0.005
1
2
5
10
20
50

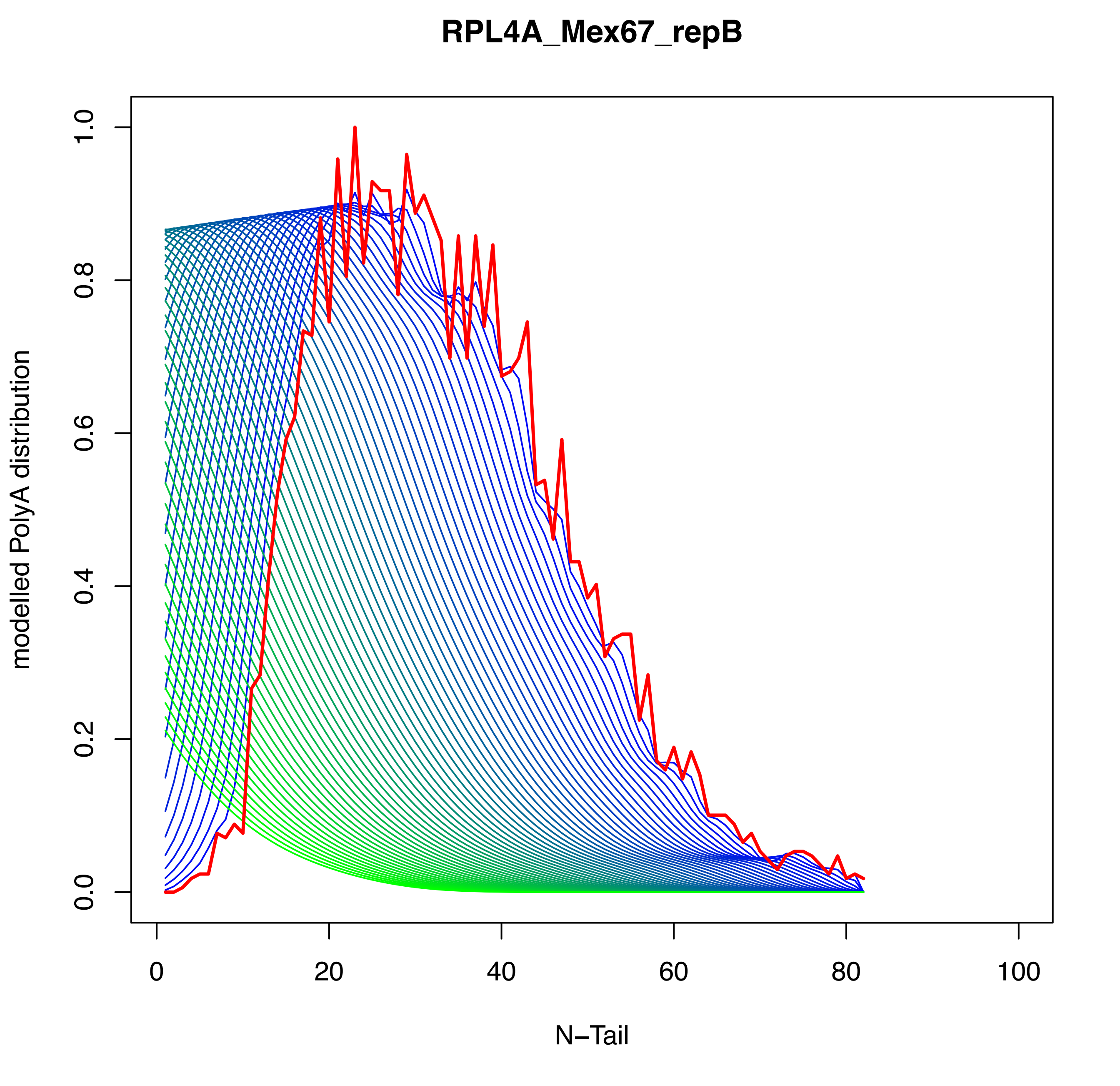

RPL4A_Mex67_repB
modelled PolyA distribution
N−Tail
0.0
0.2
0.4
0.6
0.8
1.0
0
20
40
60
80
100

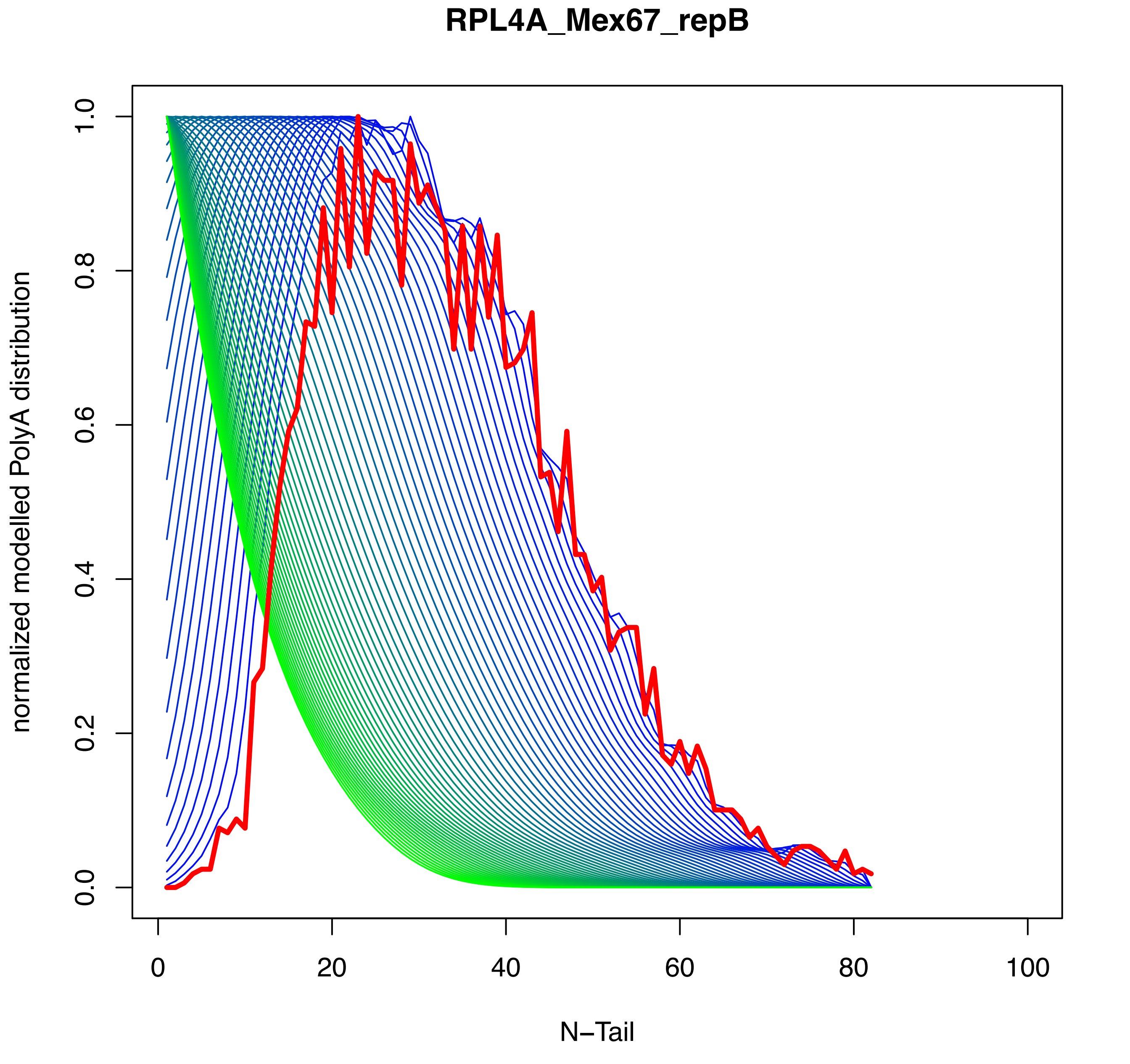

RPL4A_Mex67_repB
normalized modelled PolyA distribution
N−Tail
0.0
0.2
0.4
0.6
0.8
1.0
0
20
40
60
80
100

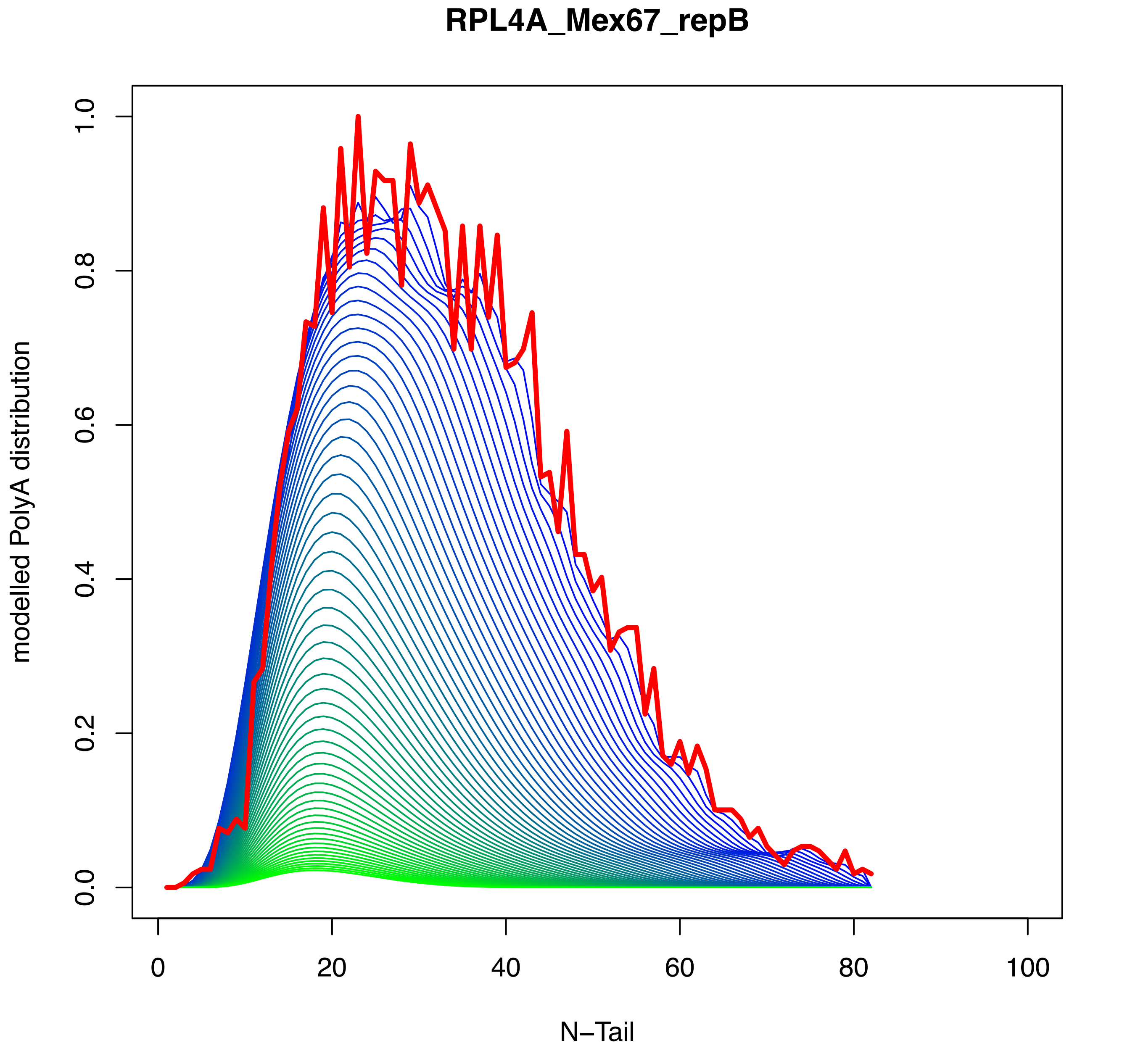

RPL4A_Mex67_repB
modelled PolyA distribution
N−Tail
0.0
0.2
0.4
0.6
0.8
1.0
0
20
40
60
80
100

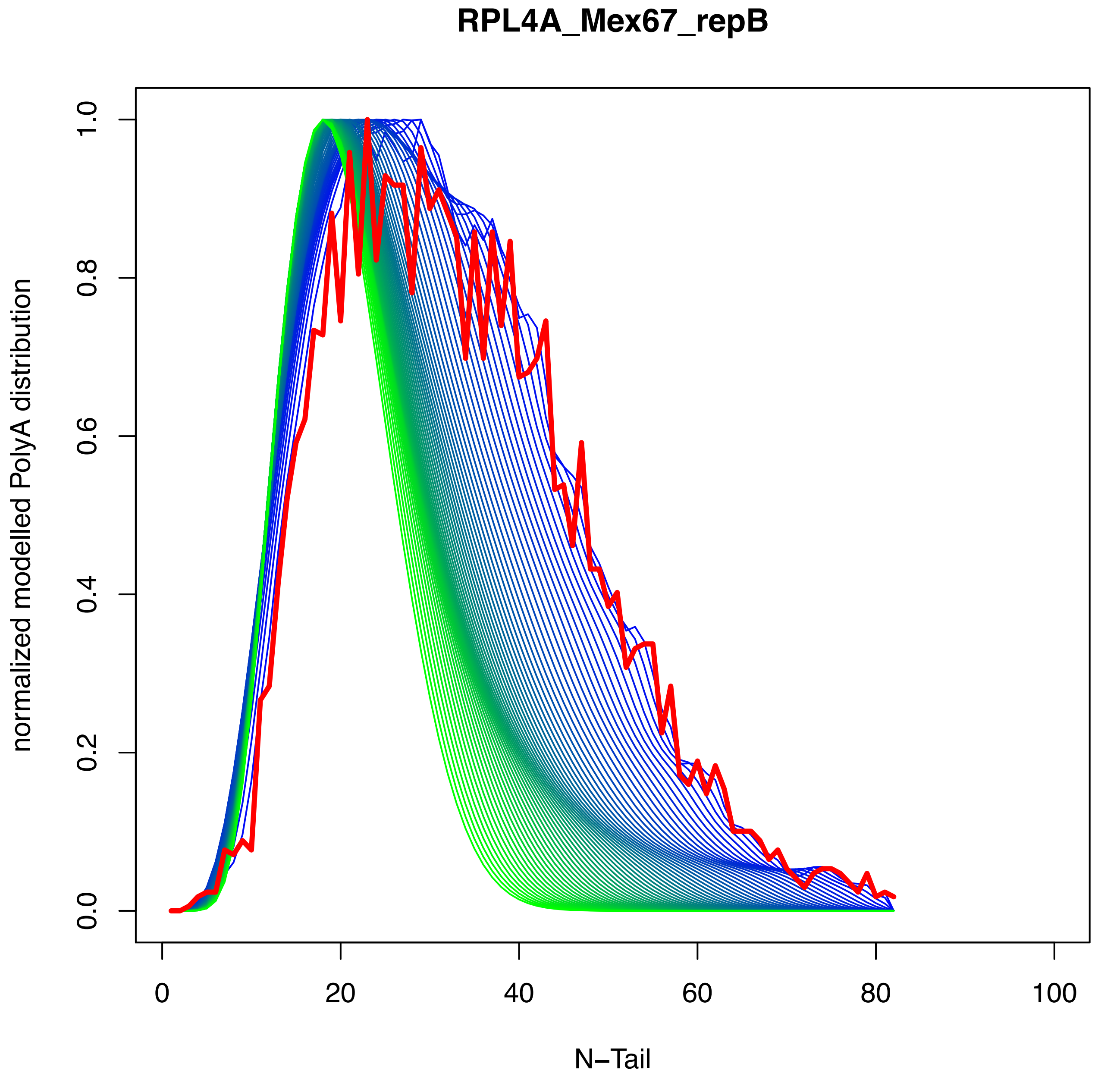

RPL4A_Mex67_repB
normalized modelled PolyA distribution
N−Tail
0.0
0.2
0.4
0.6
0.8
1.0
0
20
40
60
80
100

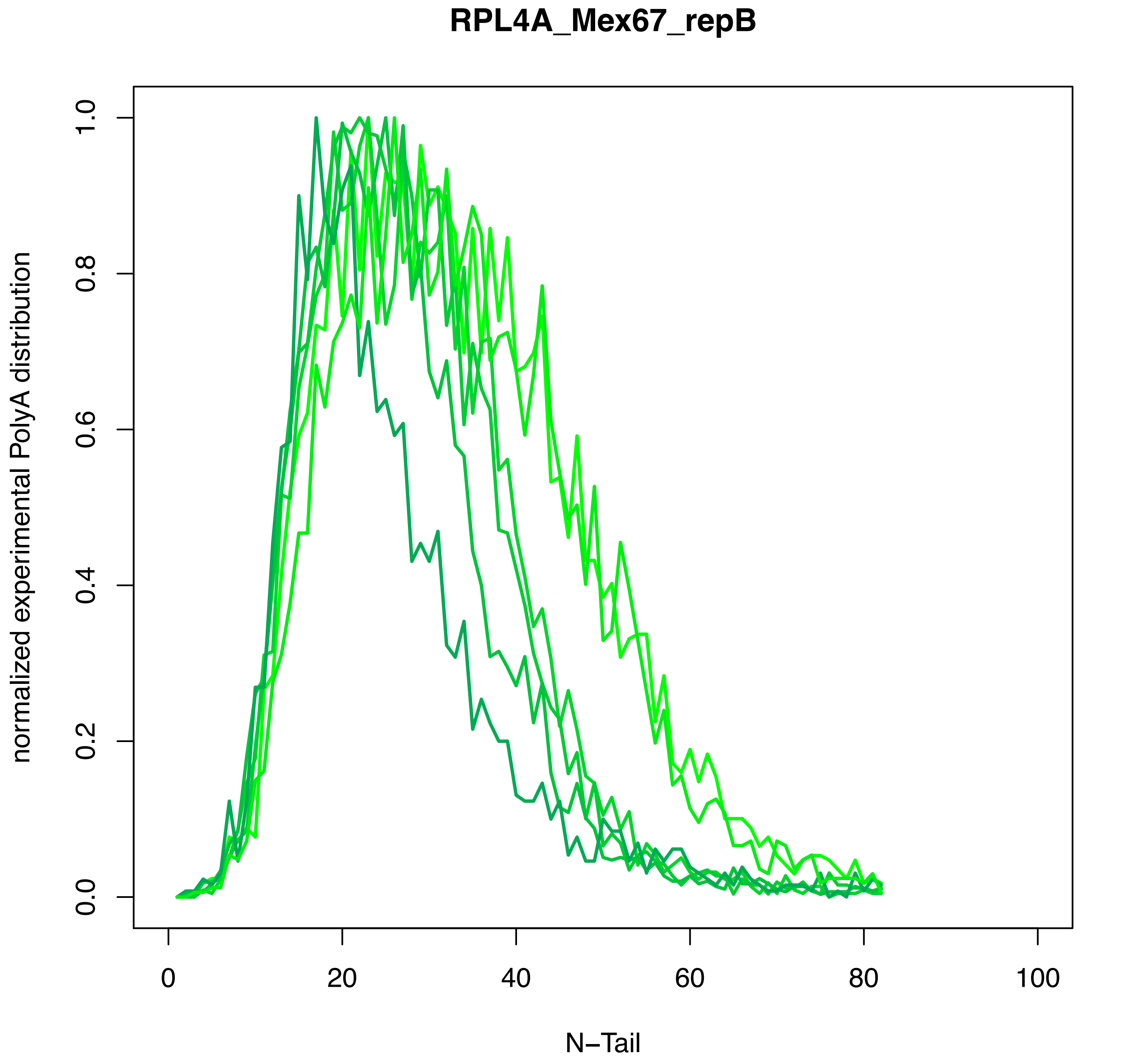
RPL4A_Mex67_repB
normalized experimental PolyA distribution
N−Tail
0.0
0.2
0.4
0.6
0.8
1.0
0
20
40
60
80
100

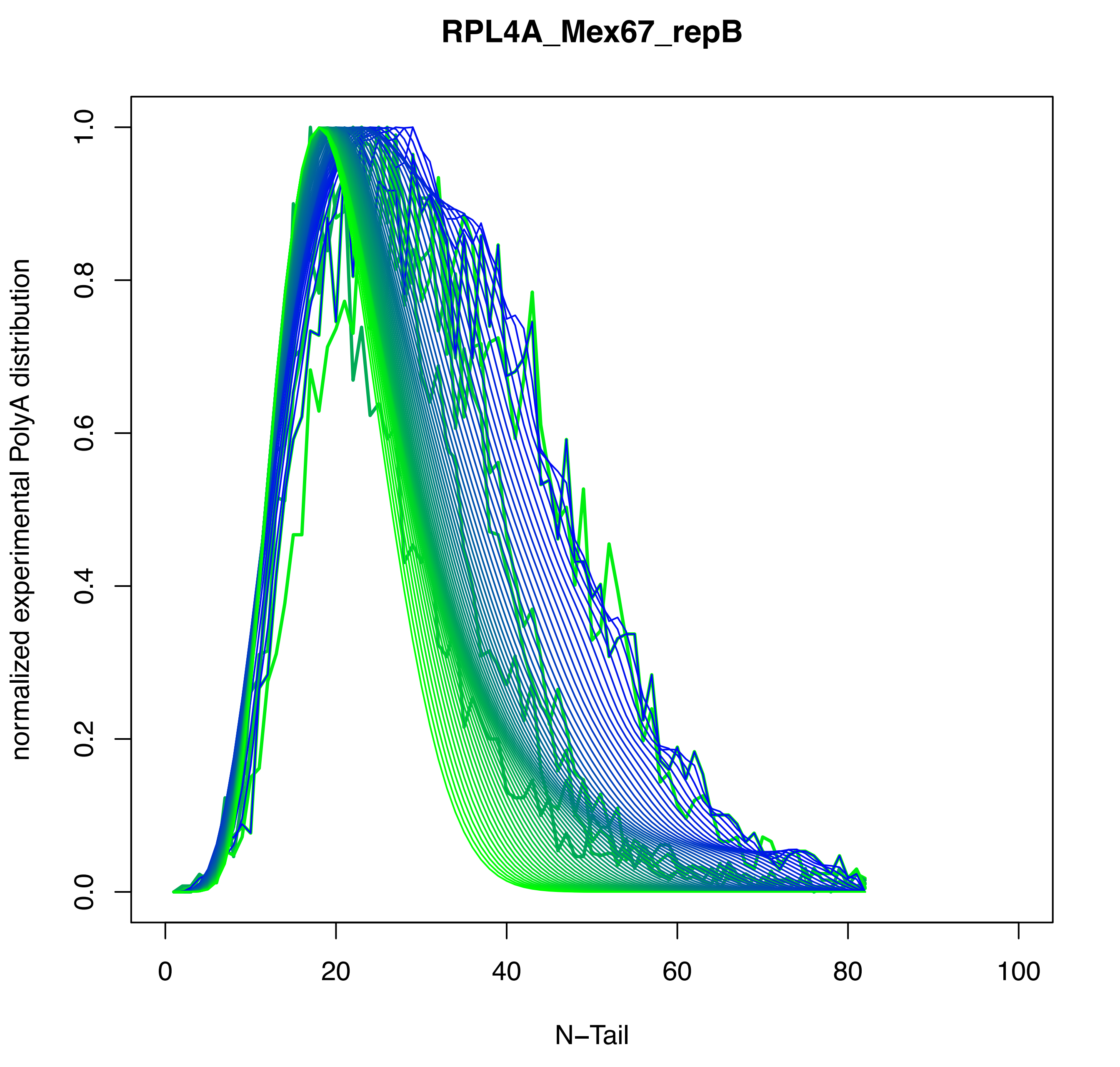
RPL4A_Mex67_repB
normalized experimental PolyA distribution
N−Tail
0.0
0.2
0.4
0.6
0.8
1.0
0
20
40
60
80
100

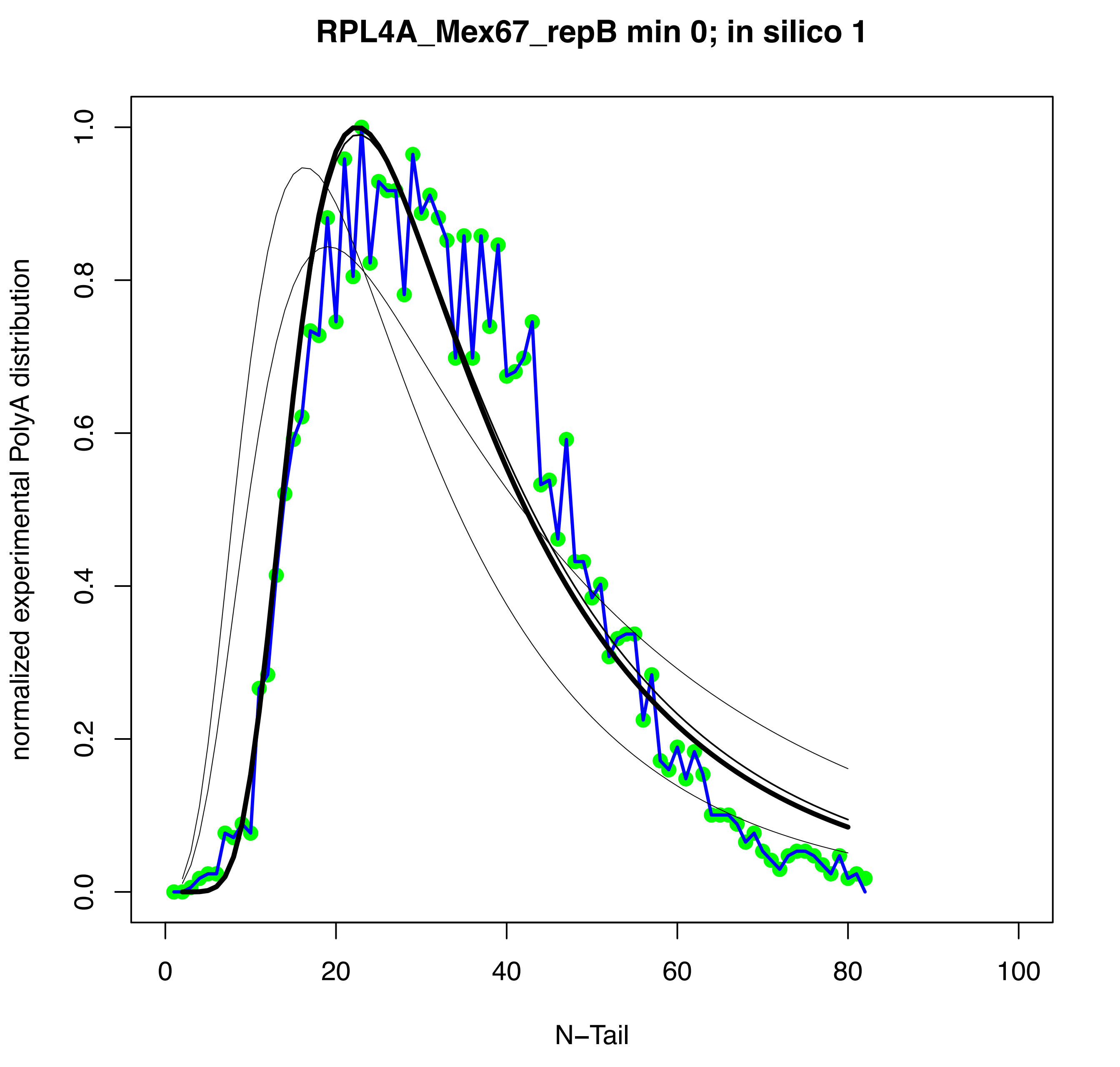

RPL4A_Mex67_repB min 0; in silico 1
normalized experimental PolyA distribution
0.0
0.2
0.4
0.6
0.8
1.0
0
20
40
60
80
100
N−Tail

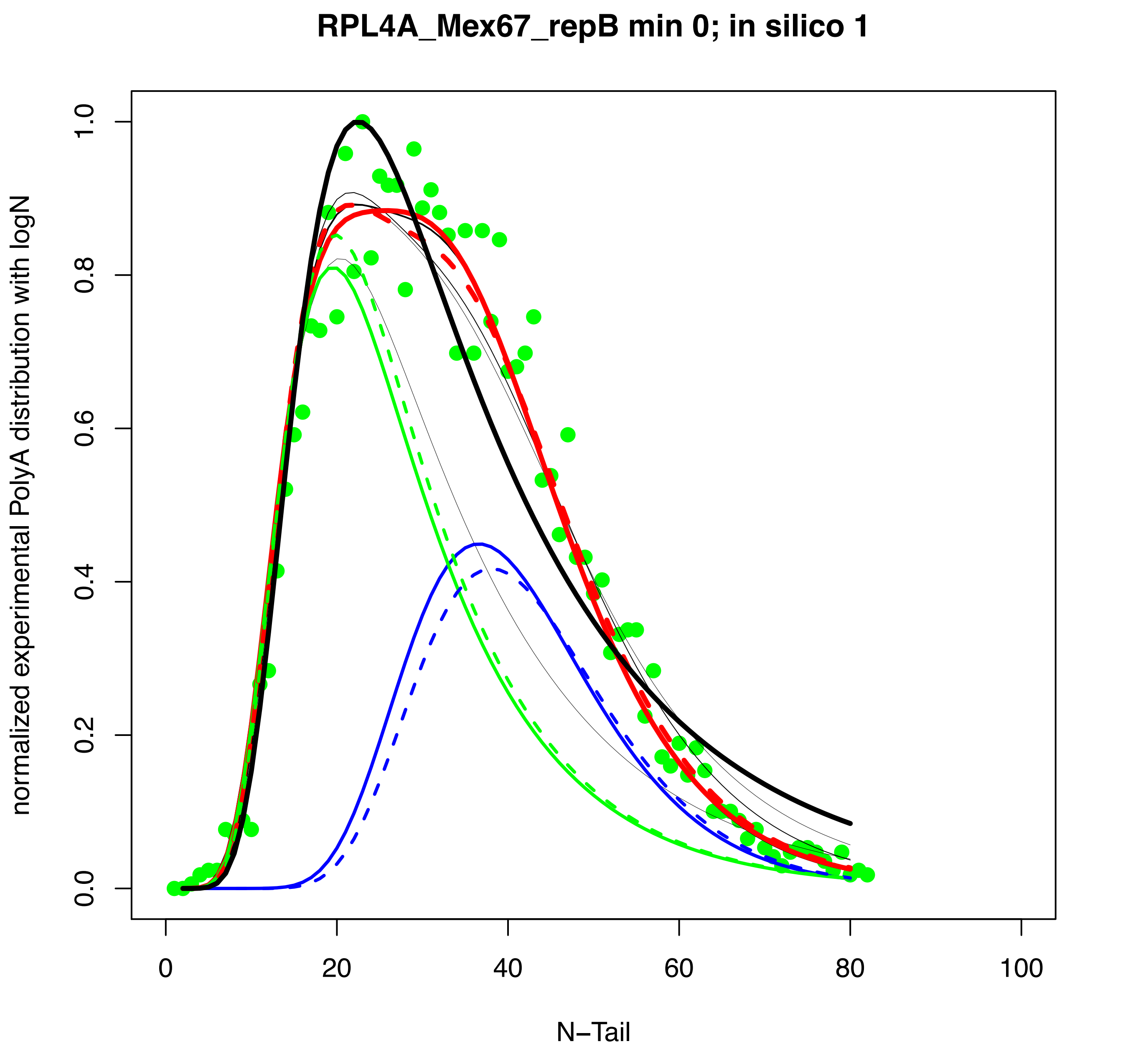
RPL4A_Mex67_repB min 0; in silico 1
normalized experimental PolyA distribution with logN
N−Tail
0.0
0.2
0.4
0.6
0.8
1.0
0
20
40
60
80
100

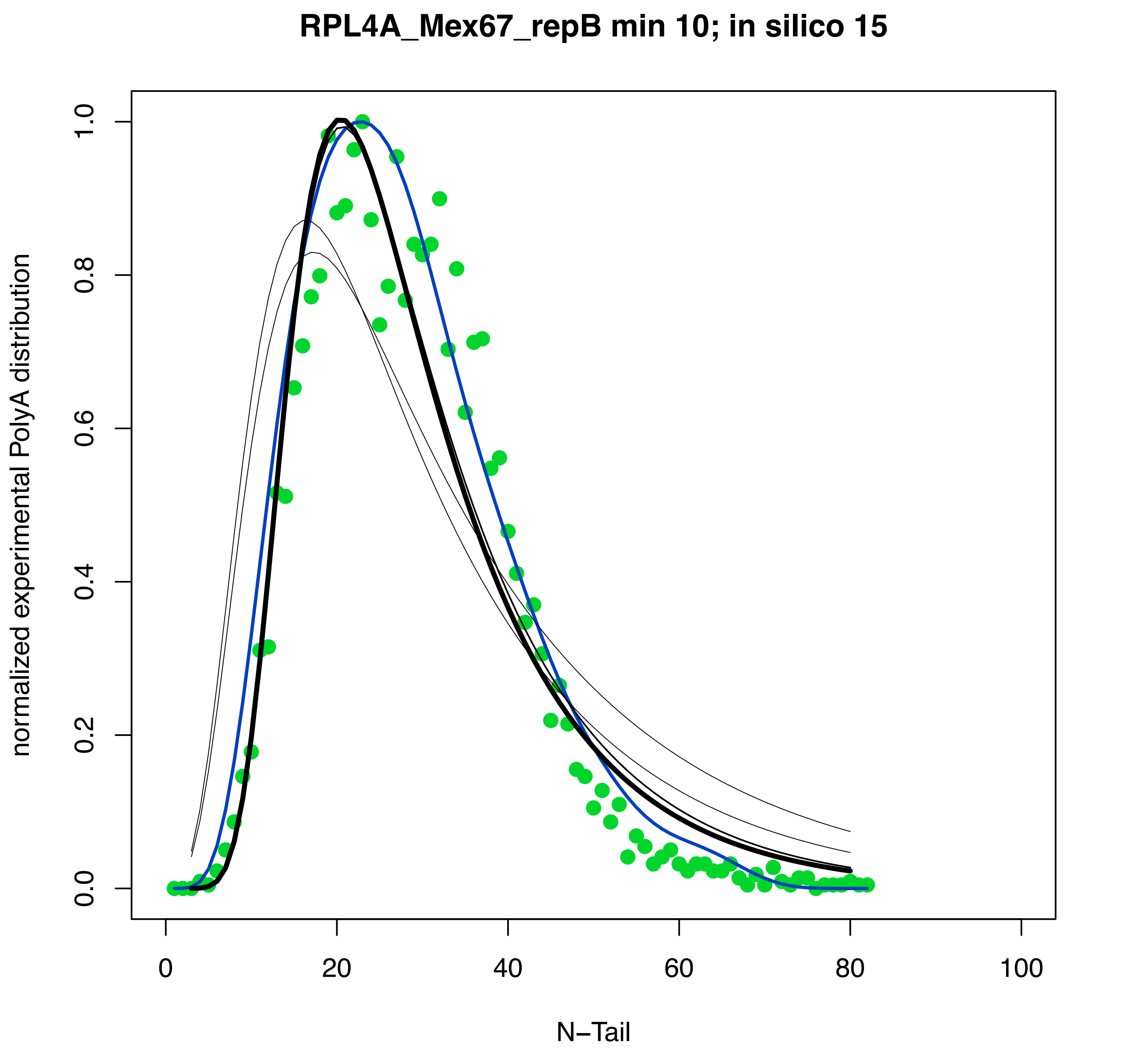
RPL4A_Mex67_repB min 10; in silico 15
normalized experimental PolyA distribution
N−Tail
0.0
0.2
0.4
0.6
0.8
1.0
0
20
40
60
80
100

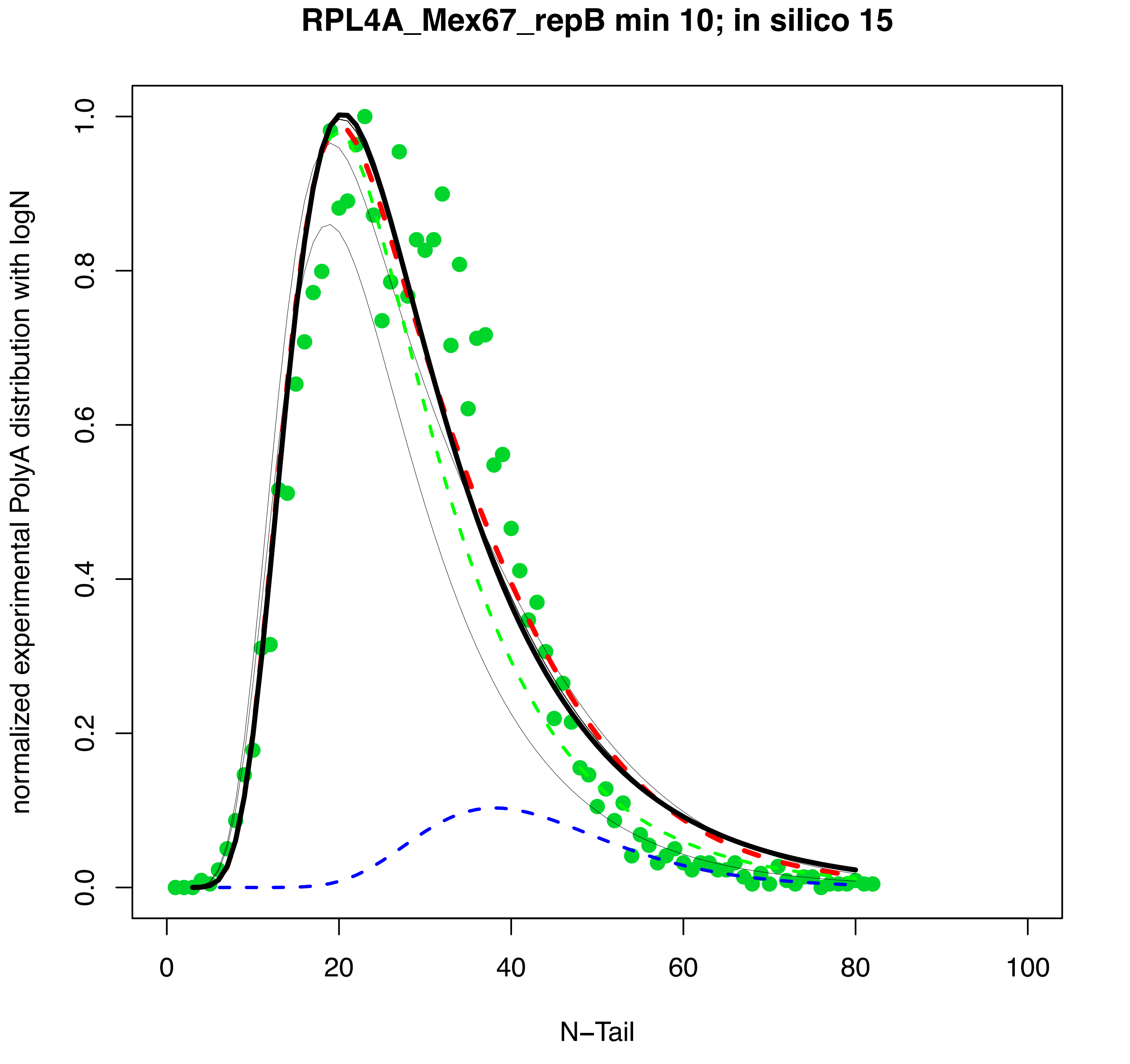
RPL4A_Mex67_repB min 10; in silico 15
normalized experimental PolyA distribution with logN
N−Tail
0.0
0.2
0.4
0.6
0.8
1.0
0
20
40
60
80
100

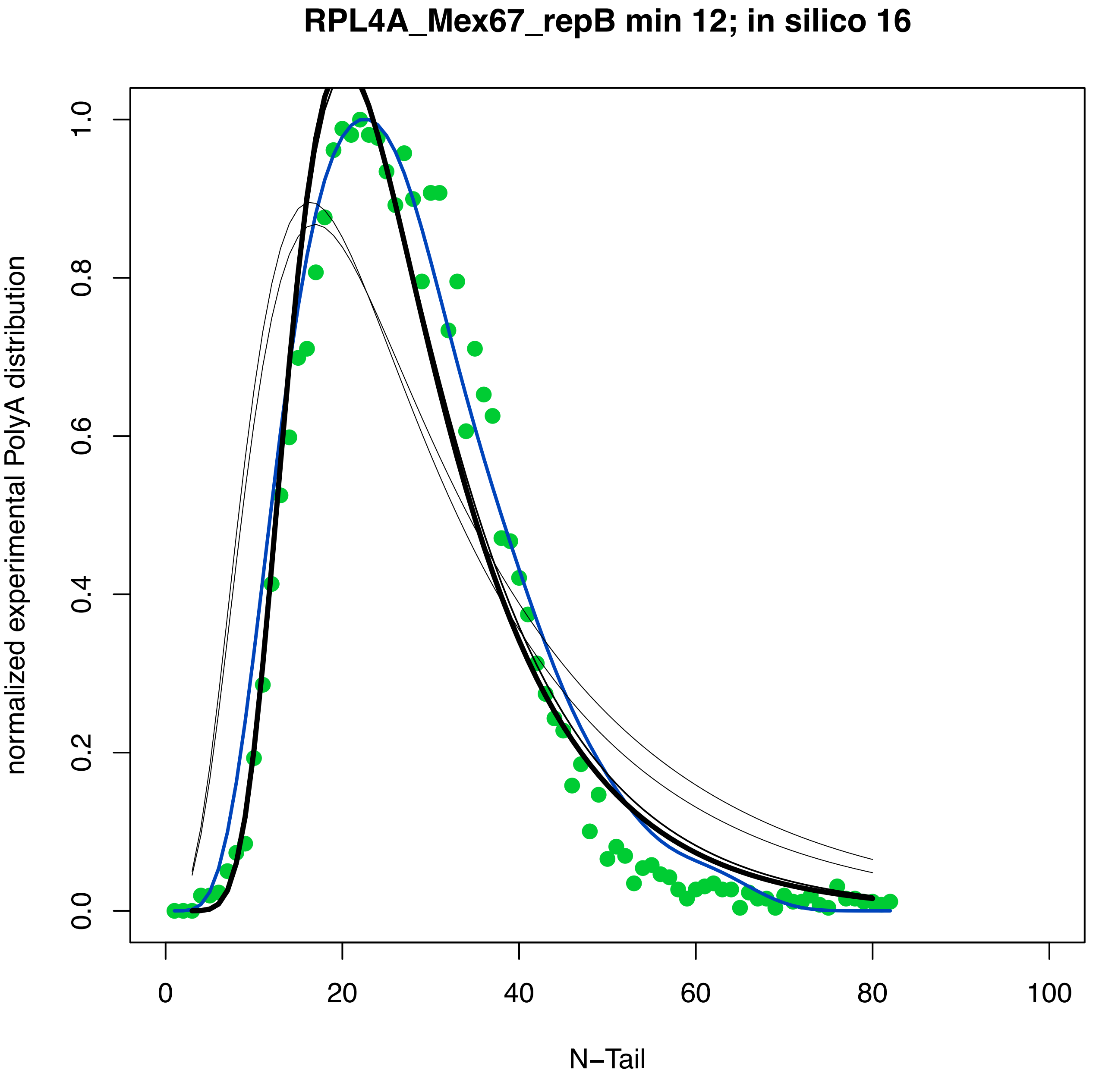
RPL4A_Mex67_repB min 12; in silico 16
normalized experimental PolyA distribution
0.0
0.2
0.4
0.6
0.8
1.0
0
20
40
60
80
100
N−Tail

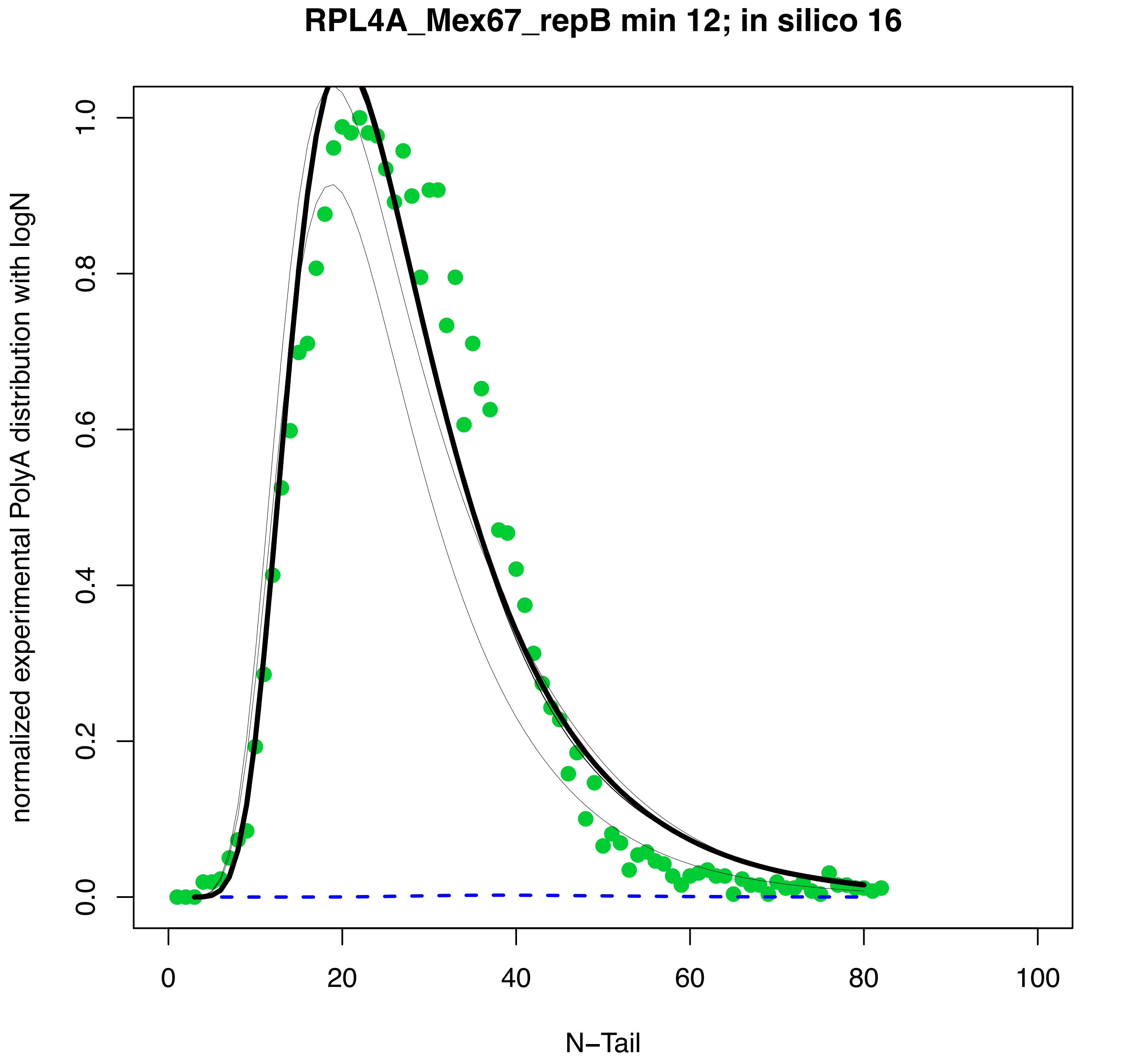
RPL4A_Mex67_repB min 12; in silico 16
normalized experimental PolyA distribution with logN
N−Tail
0.0
0.2
0.4
0.6
0.8
1.0
0
20
40
60
80
100

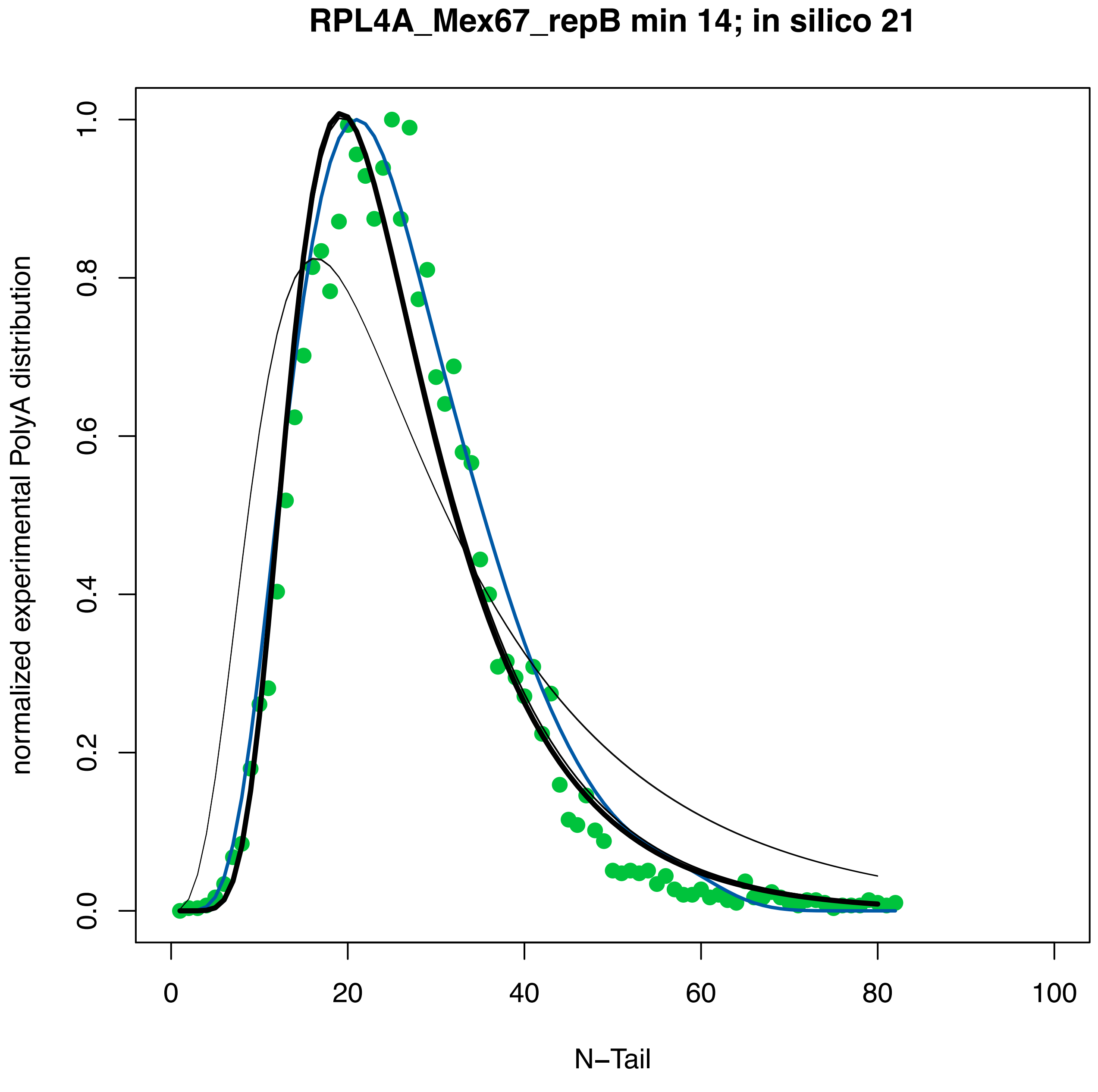
RPL4A_Mex67_repB min 14; in silico 21
normalized experimental PolyA distribution
N−Tail
0.0
0.2
0.4
0.6
0.8
1.0
0
20
40
60
80
100

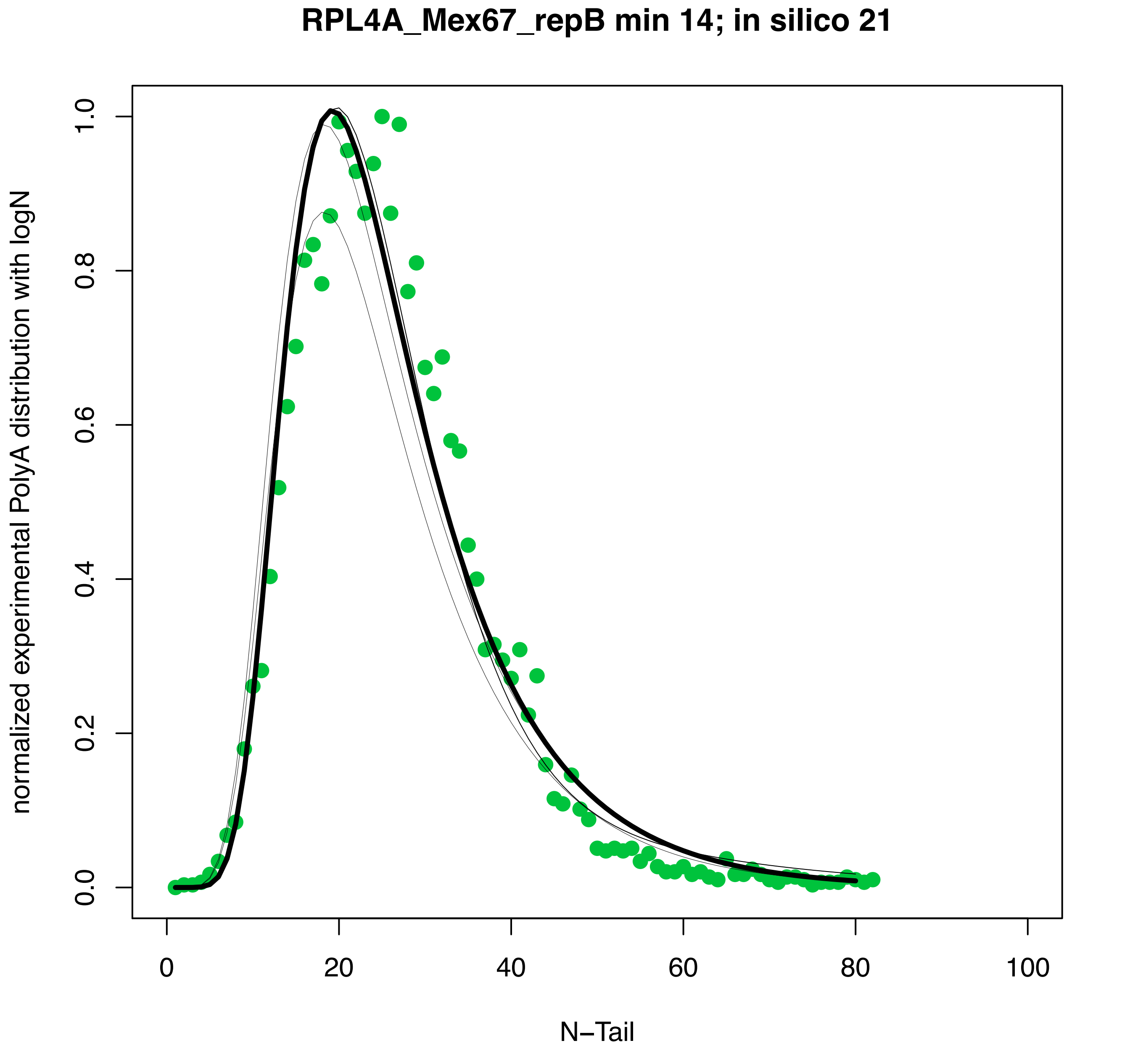

RPL4A_Mex67_repB min 14; in silico 21
normalized experimental PolyA distribution with logN
N−Tail
0.0
0.2
0.4
0.6
0.8
1.0
0
20
40
60
80
100

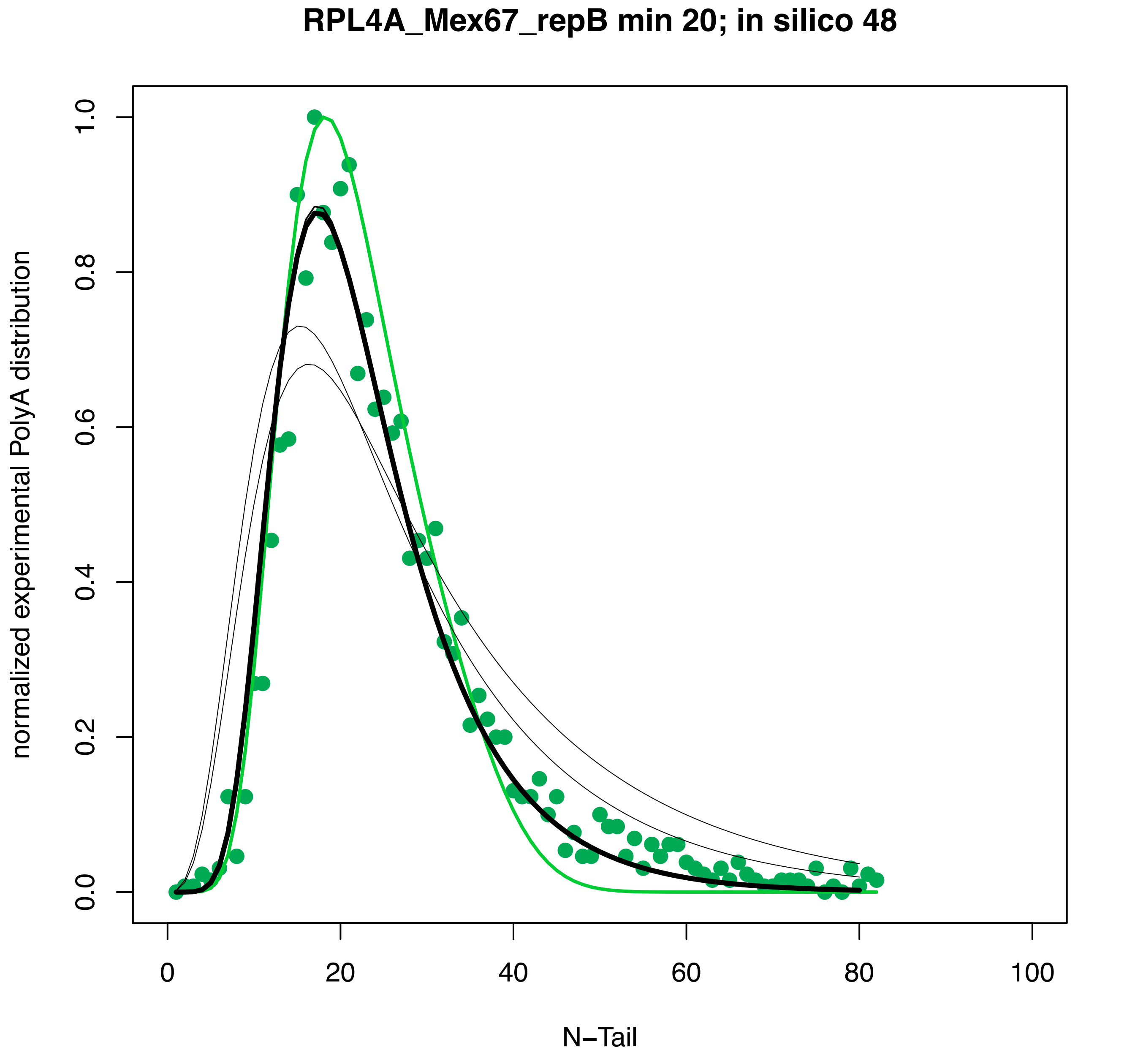

RPL4A_Mex67_repB min 20; in silico 48
normalized experimental PolyA distribution
N−Tail
0.0
0.2
0.4
0.6
0.8
1.0
0
20
40
60
80
100

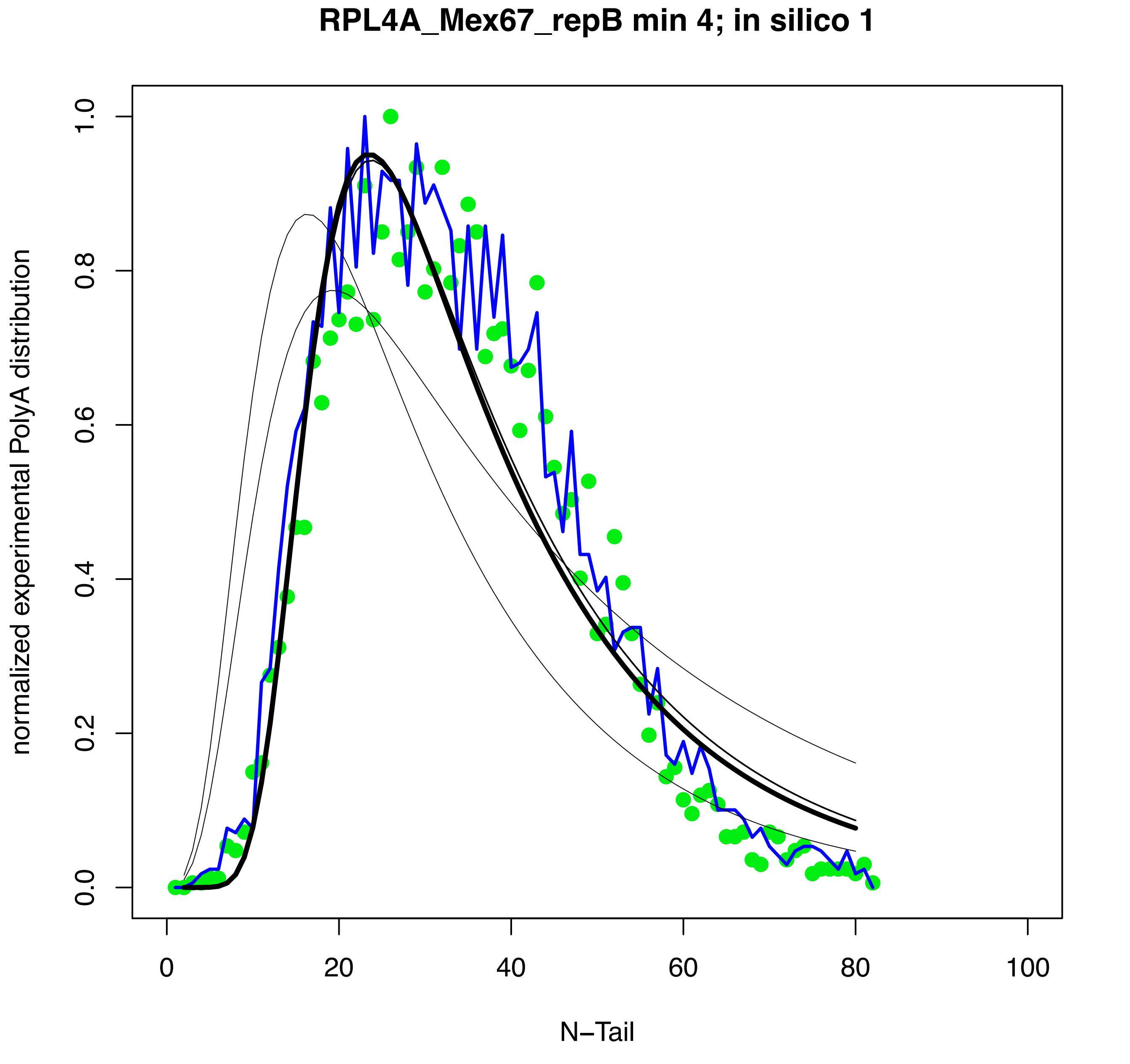
RPL4A_Mex67_repB min 4; in silico 1
normalized experimental PolyA distribution
N−Tail
0.0
0.2
0.4
0.6
0.8
1.0
0
20
40
60
80
100

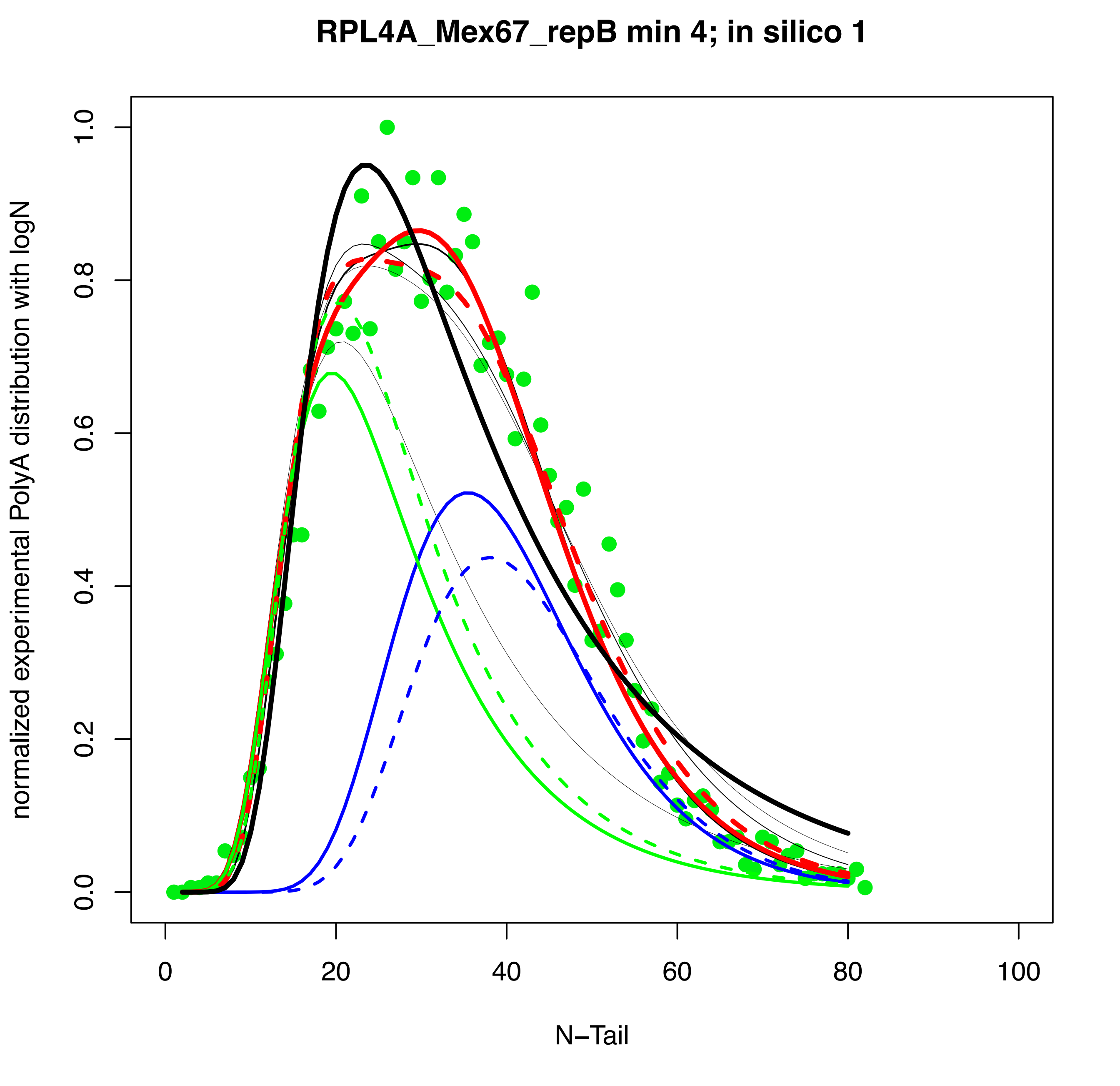

RPL4A_Mex67_repB min 4; in silico 1
normalized experimental PolyA distribution with logN
N−Tail
0.0
0.2
0.4
0.6
0.8
1.0
0
20
40
60
80
100

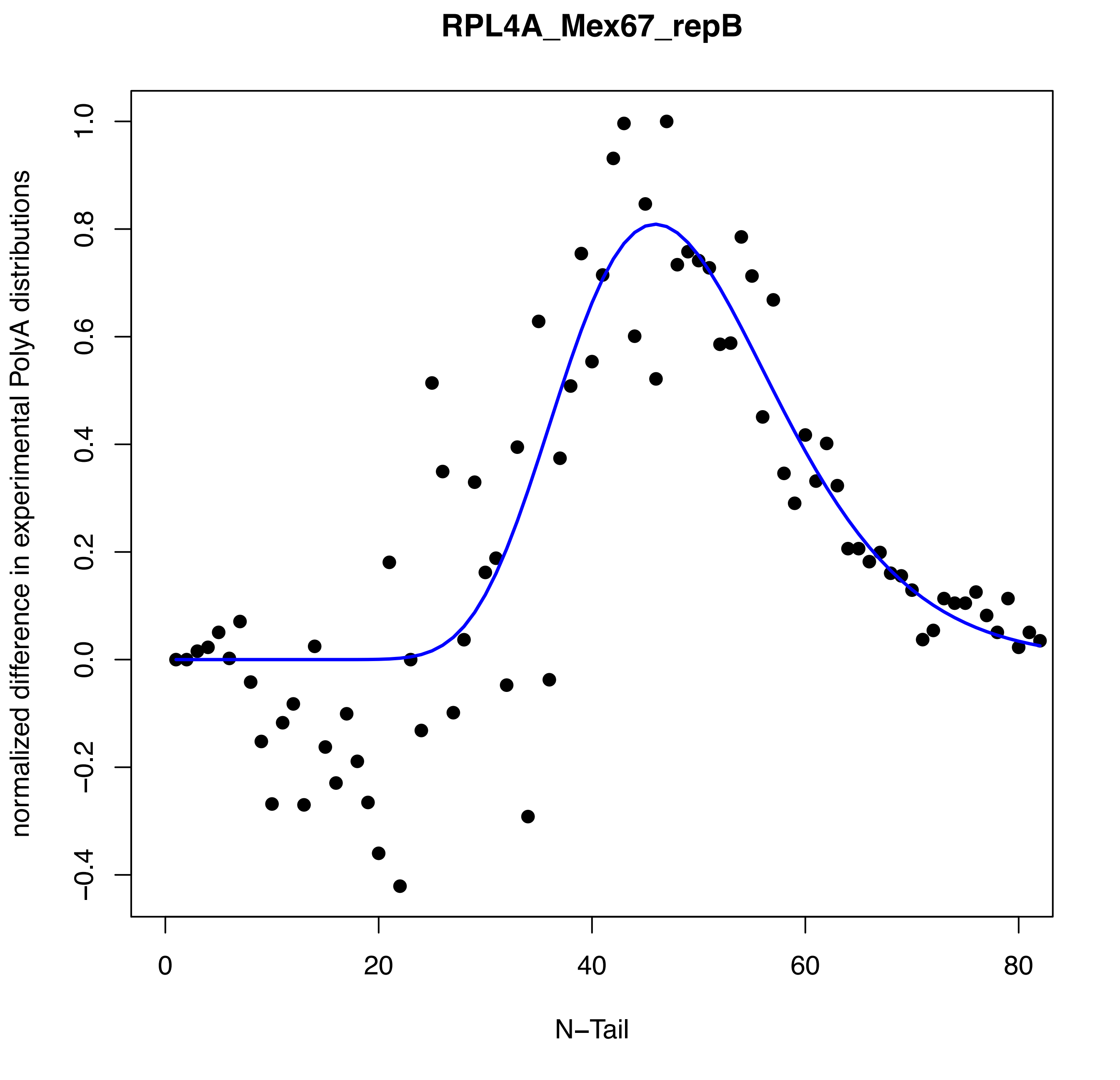

RPL4A_Mex67_repB
normalized difference in experimental PolyA distributions
N−Tail
1.0
0.8
0.6
0.4
0.2
0.0
−0.2
−0.4
0
20
40
60
80

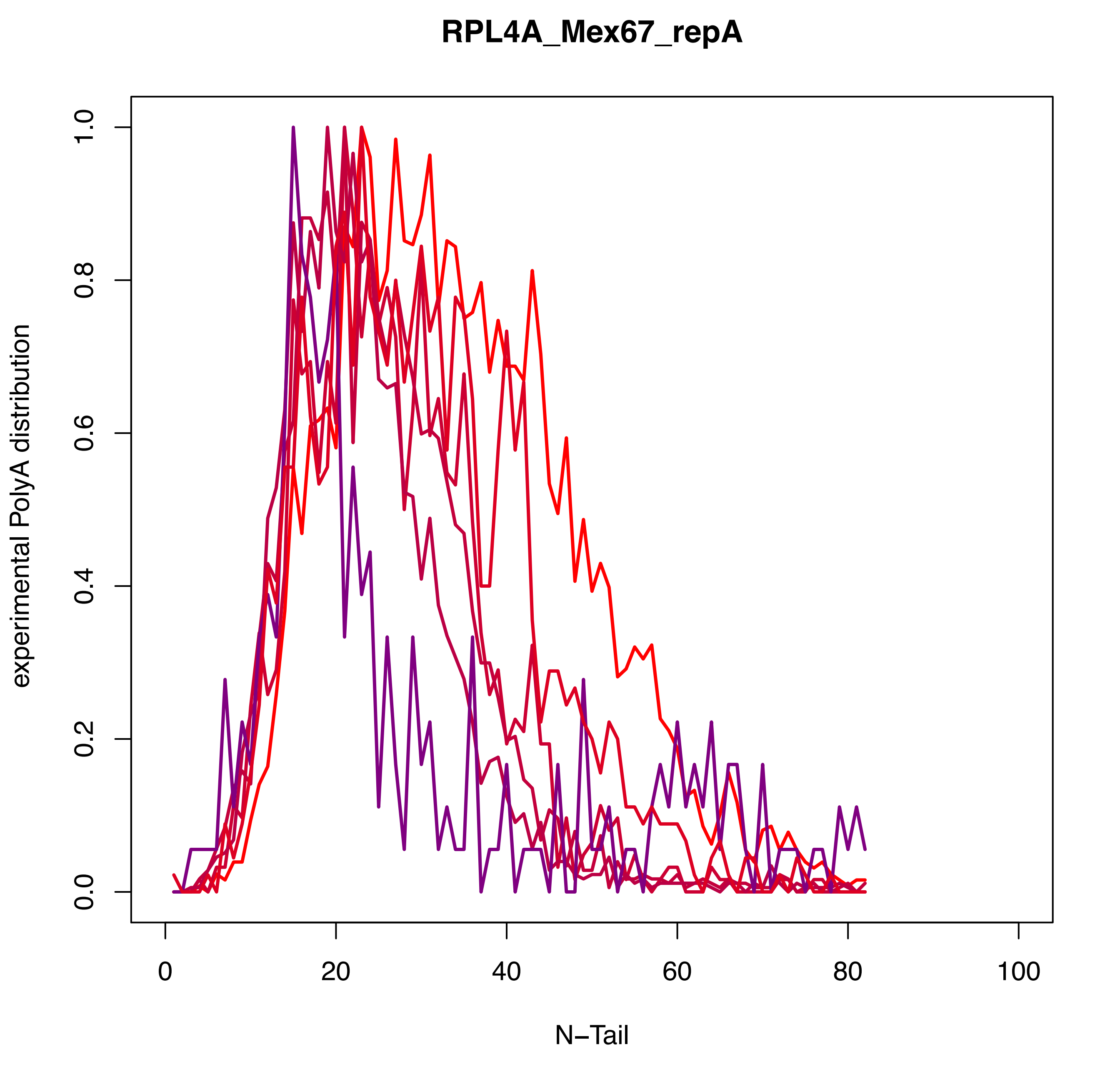
RPL4A_Mex67_repA
experimental PolyA distribution
N−Tail
0.0
0.2
0.4
0.6
0.8
1.0
0
20
40
60
80
100

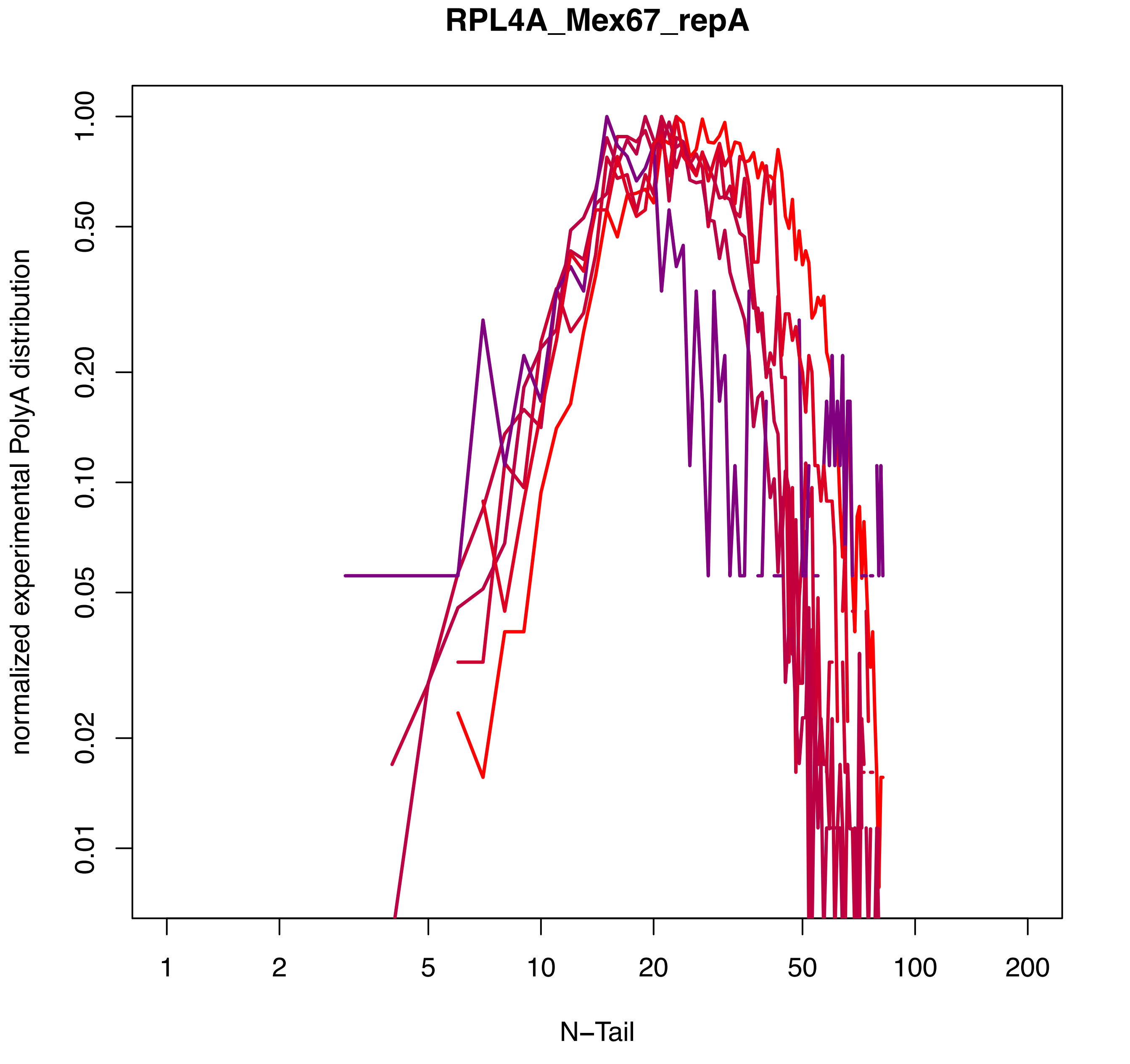

RPL4A_Mex67_repA
normalized experimental PolyA distribution
1.00
0.50
0.20
0.10
0.05
0.02
0.01
1
2
5
10
20
50
100
200
N−Tail

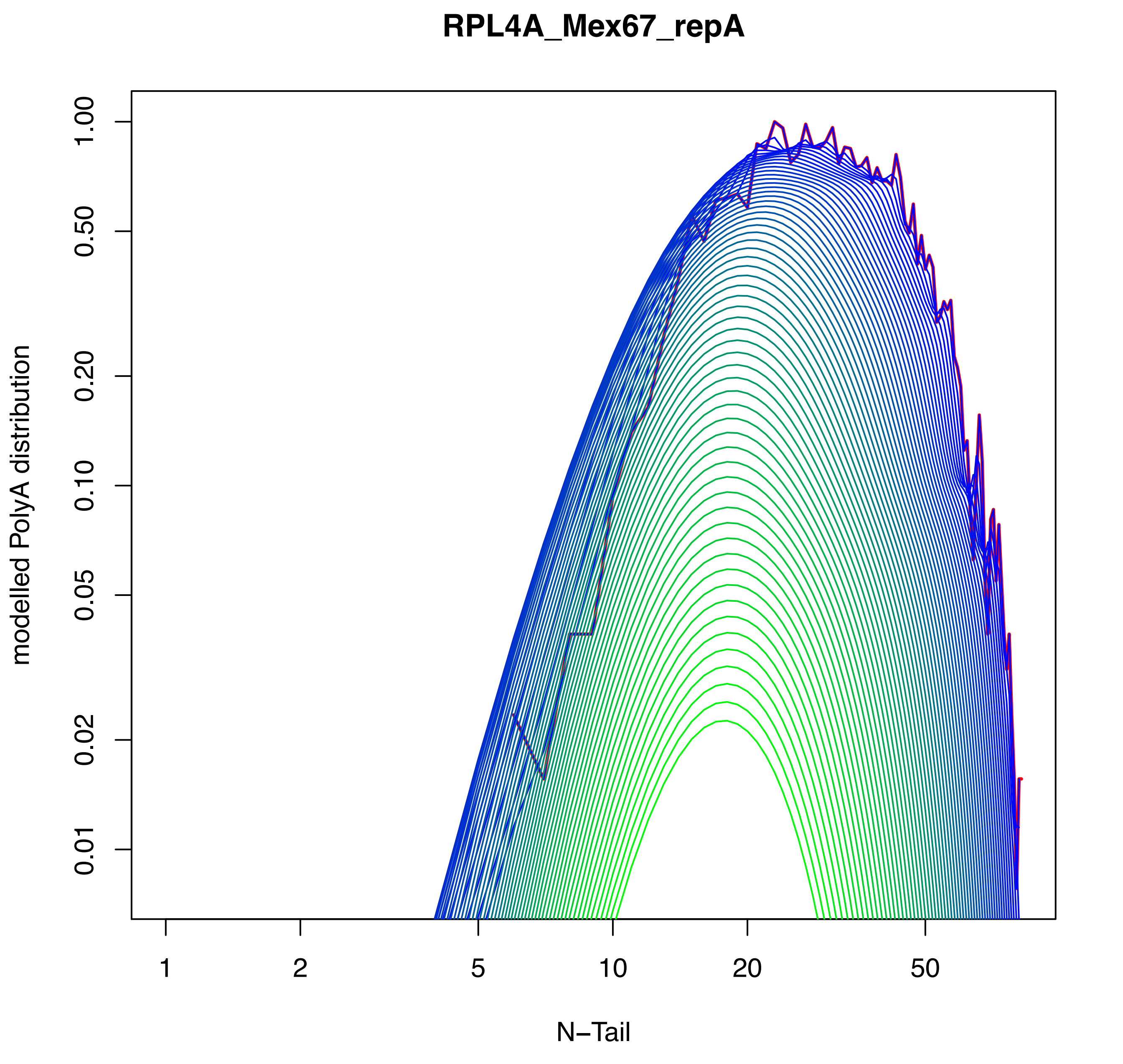

RPL4A_Mex67_repA
modelled PolyA distribution
1.00
0.50
0.20
0.10
0.05
0.02
0.01
1
2
5
10
20
50
N−Tail

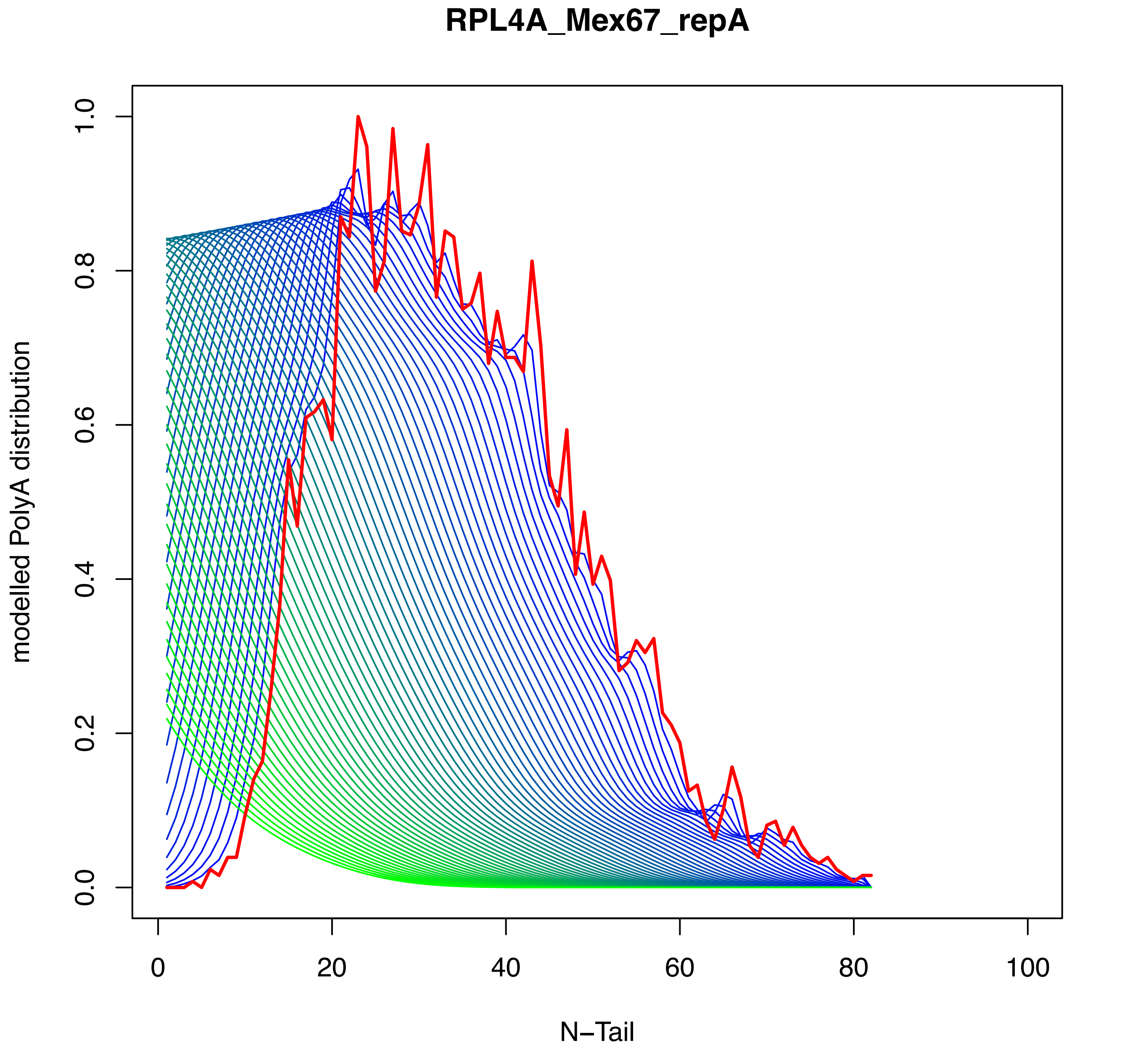
RPL4A_Mex67_repA
modelled PolyA distribution
N−Tail
0.0
0.2
0.4
0.6
0.8
1.0
0
20
40
60
80
100

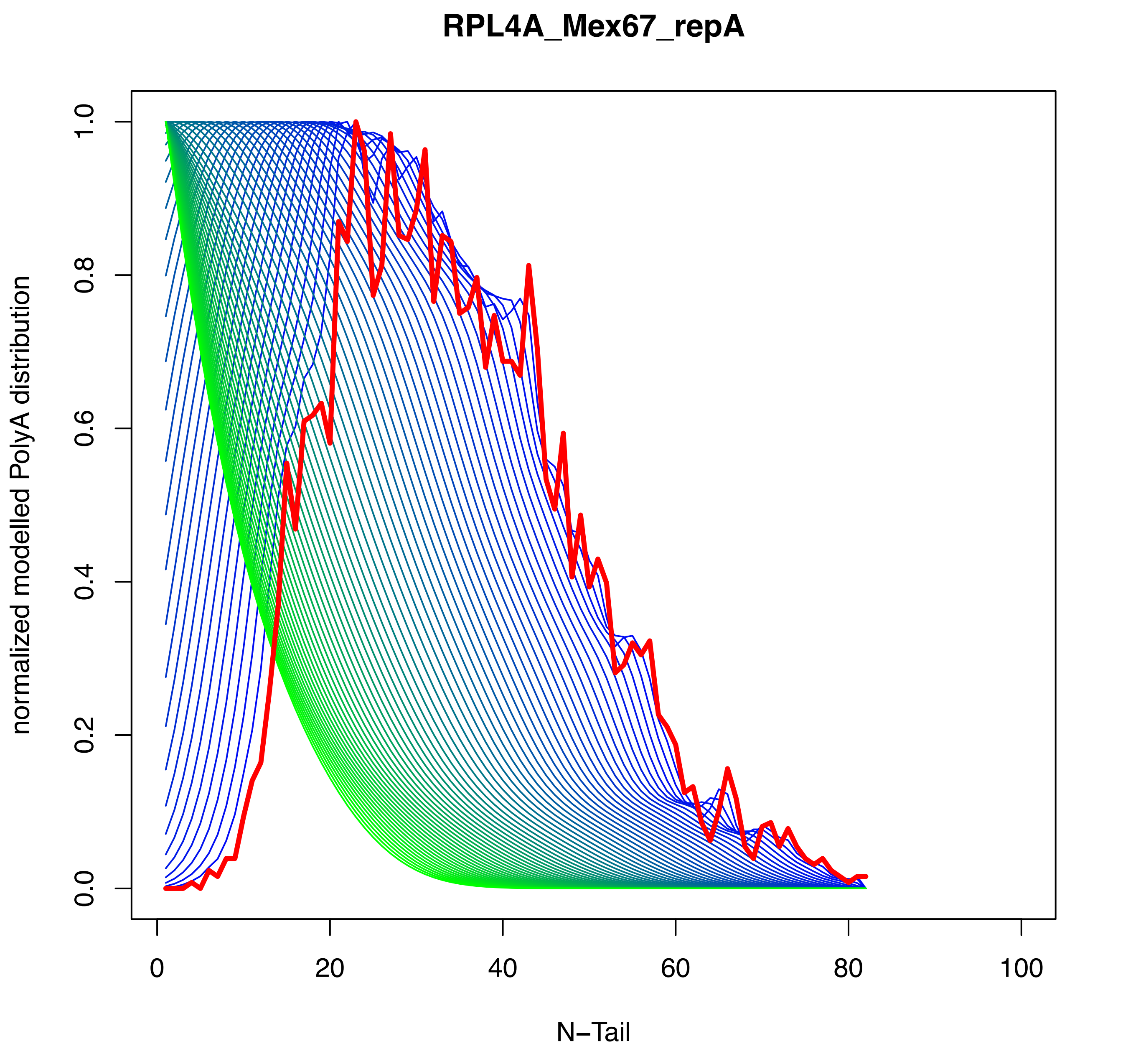

RPL4A_Mex67_repA
normalized modelled PolyA distribution
N−Tail
0.0
0.2
0.4
0.6
0.8
1.0
0
20
40
60
80
100

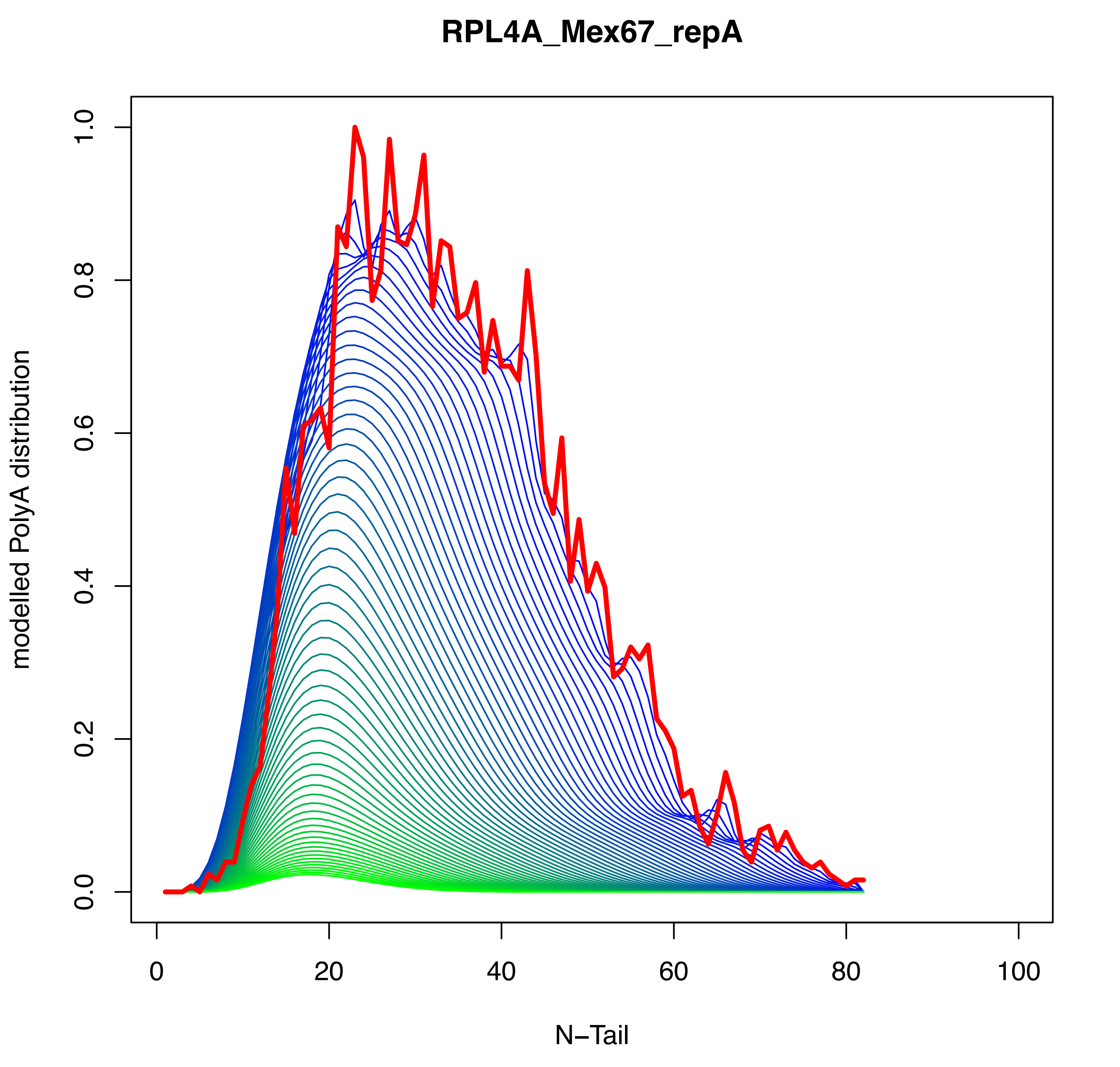

RPL4A_Mex67_repA
modelled PolyA distribution
N−Tail
0.0
0.2
0.4
0.6
0.8
1.0
0
20
40
60
80
100

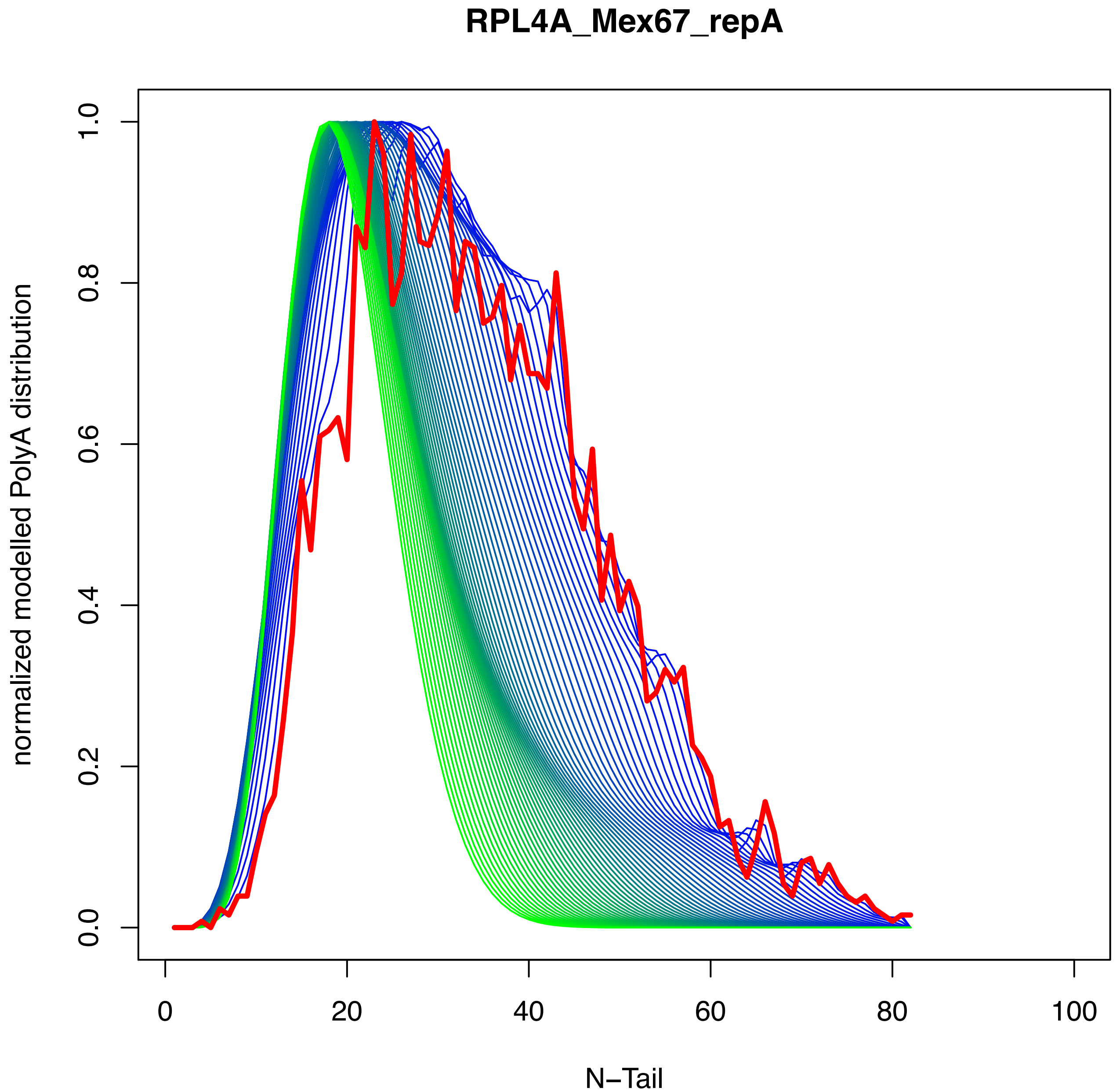

RPL4A_Mex67_repA
normalized modelled PolyA distribution
N−Tail
0.0
0.2
0.4
0.6
0.8
1.0
0
20
40
60
80
100

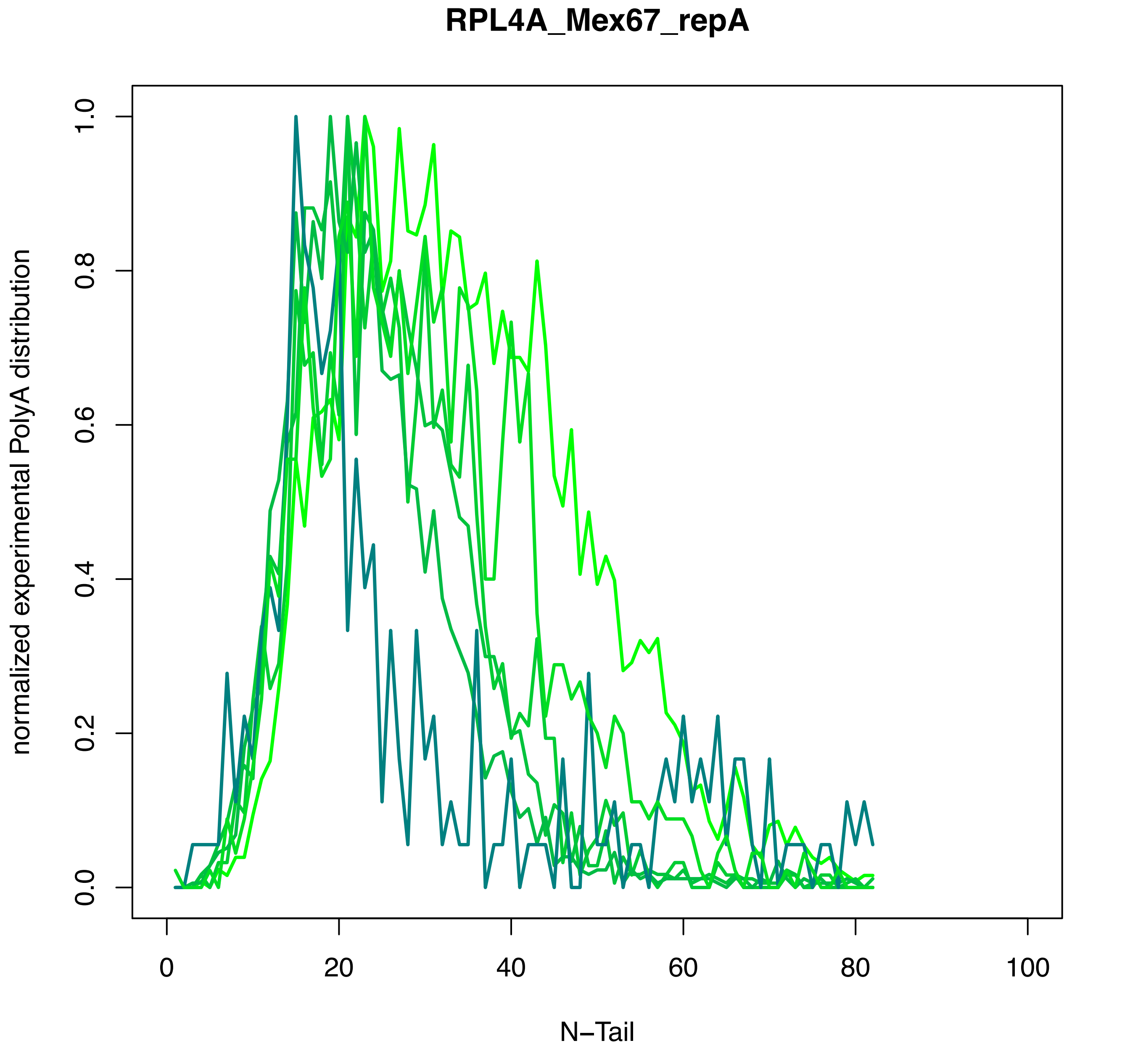

RPL4A_Mex67_repA
normalized experimental PolyA distribution
N−Tail
0.0
0.2
0.4
0.6
0.8
1.0
0
20
40
60
80
100

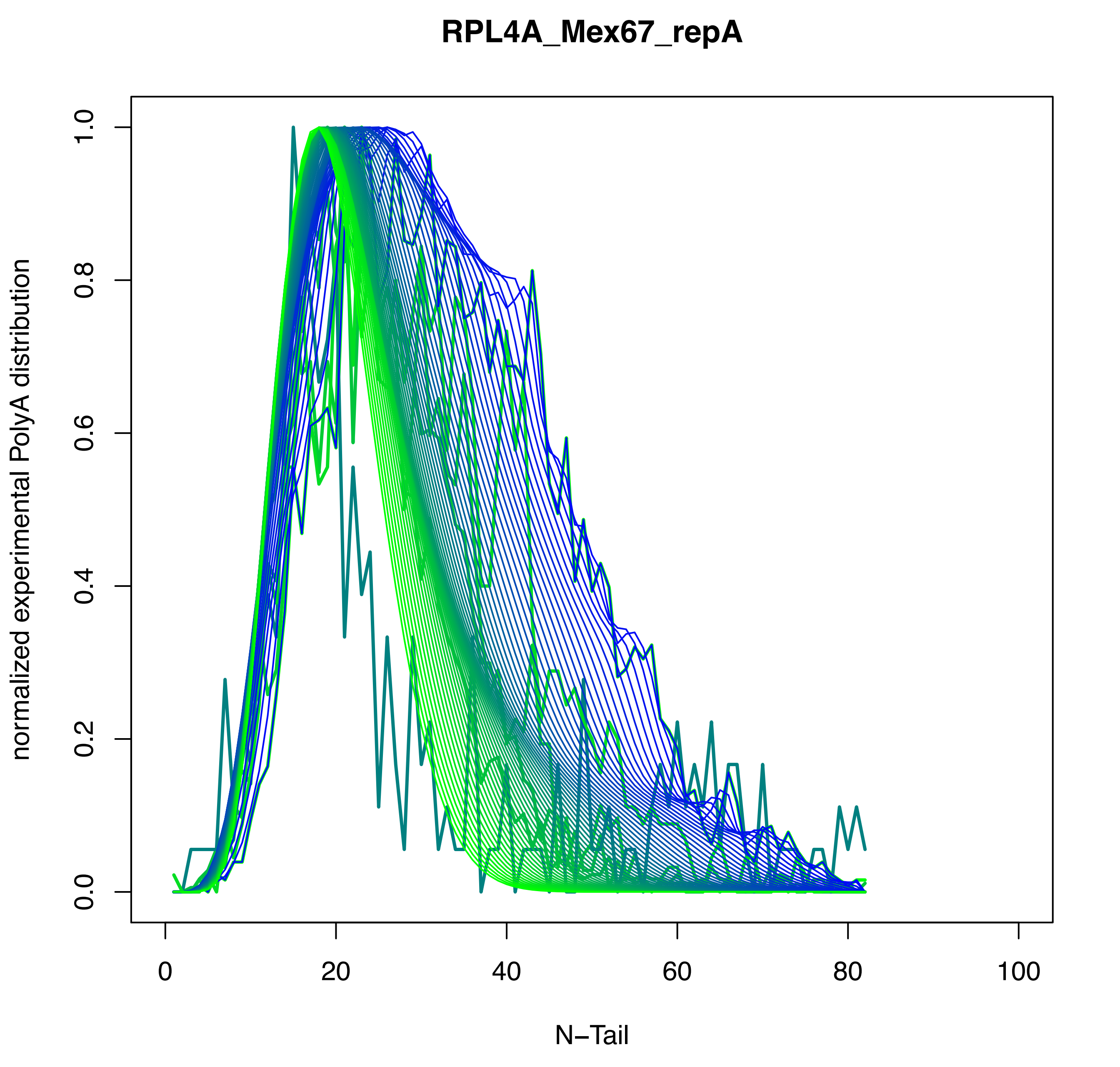

RPL4A_Mex67_repA
normalized experimental PolyA distribution
N−Tail
0.0
0.2
0.4
0.6
0.8
1.0
0
20
40
60
80
100

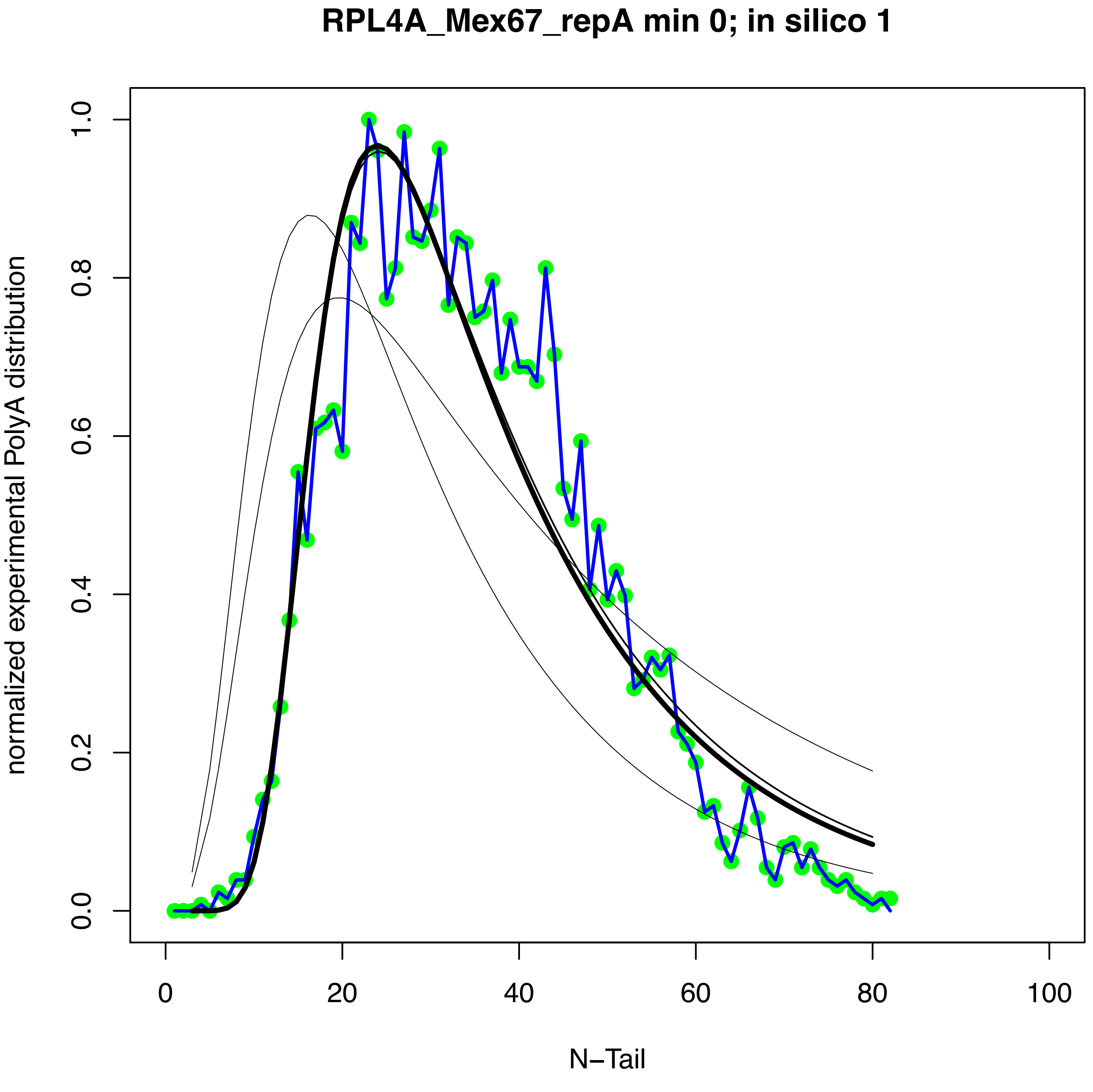
RPL4A_Mex67_repA min 0; in silico 1
normalized experimental PolyA distribution
N−Tail
0.0
0.2
0.4
0.6
0.8
1.0
0
20
40
60
80
100

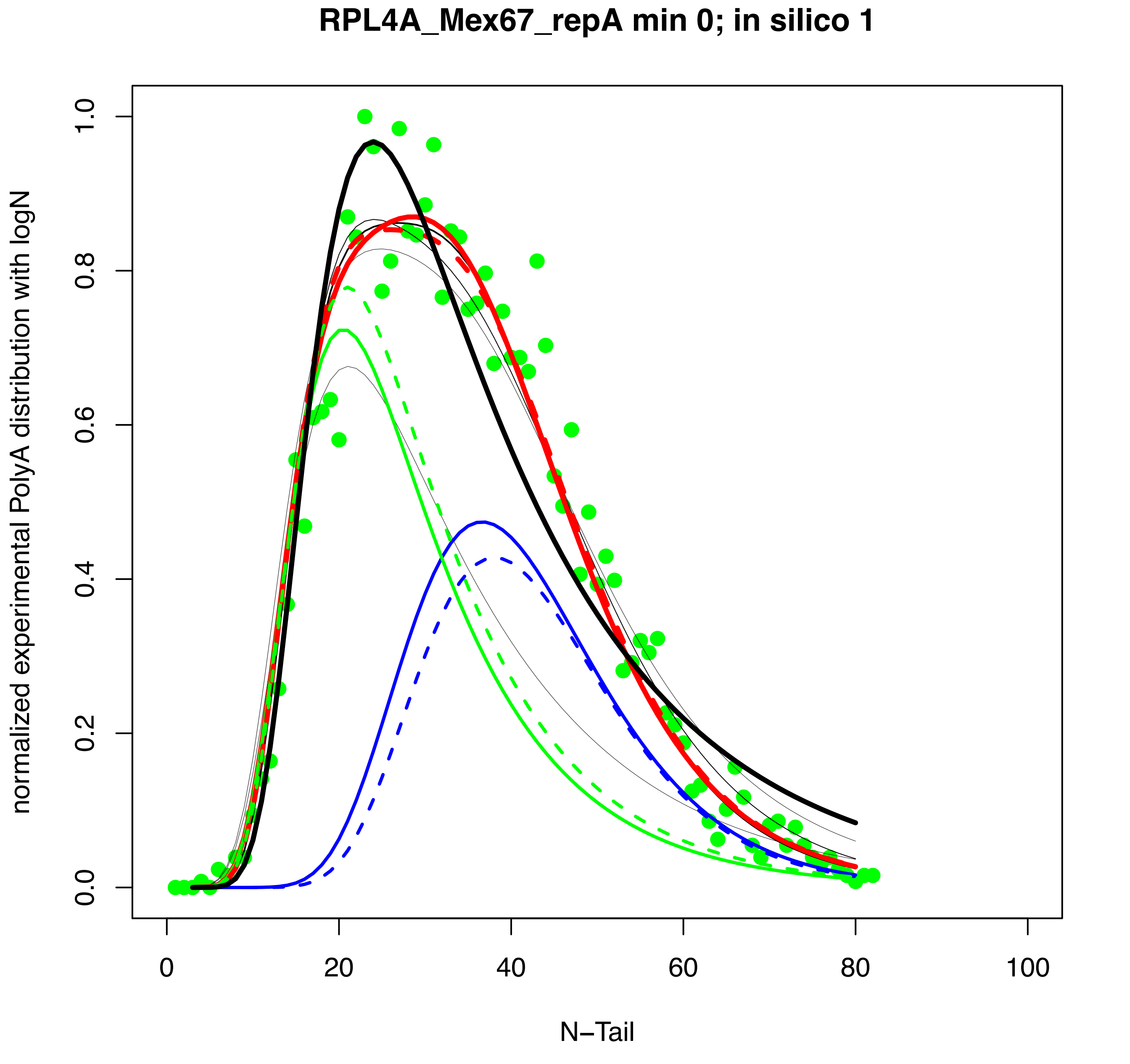

RPL4A_Mex67_repA min 0; in silico 1
normalized experimental PolyA distribution with logN
N−Tail
0.0
0.2
0.4
0.6
0.8
1.0
0
20
40
60
80
100

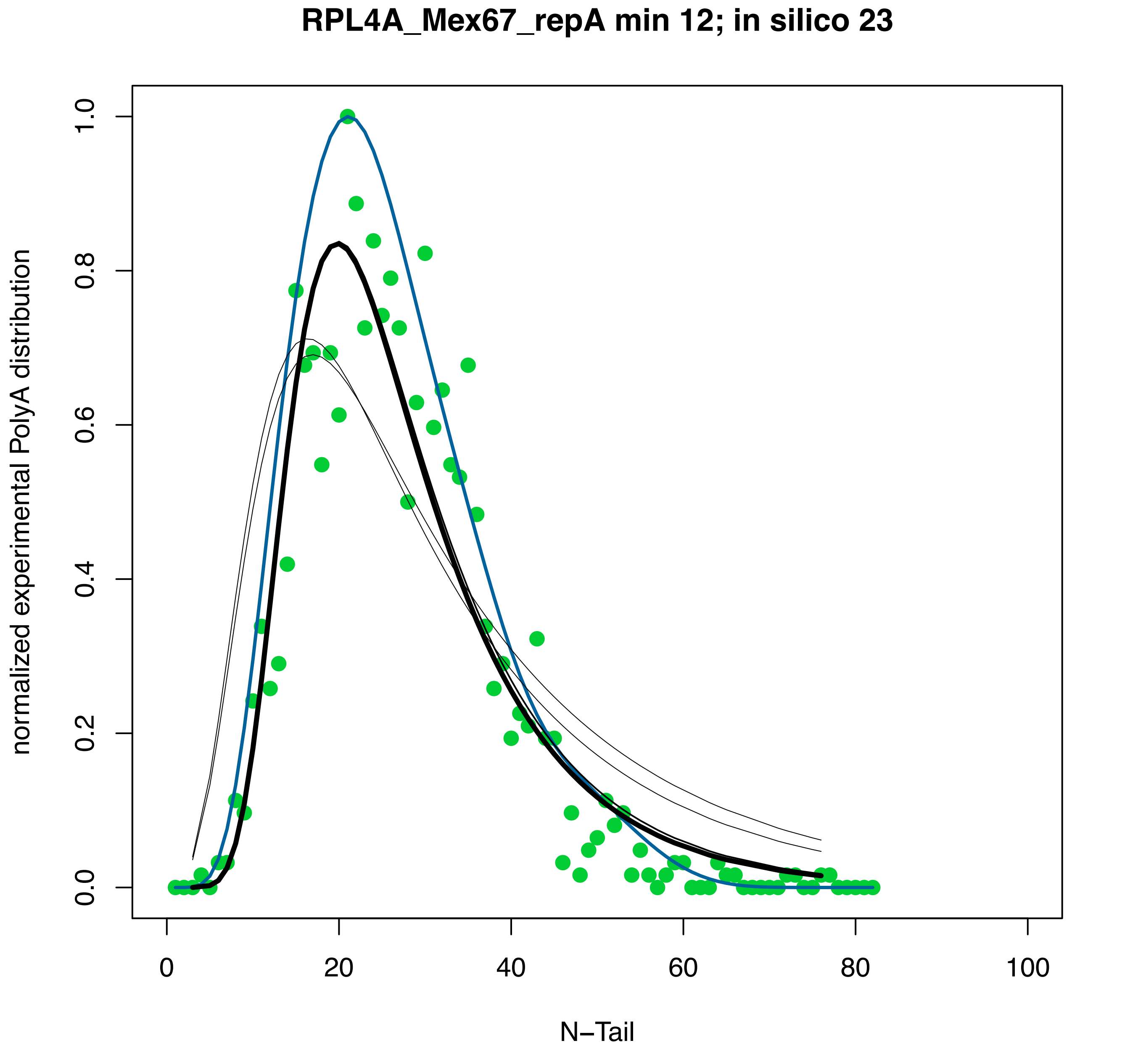

RPL4A_Mex67_repA min 12; in silico 23
normalized experimental PolyA distribution
N−Tail
0.0
0.2
0.4
0.6
0.8
1.0
0
20
40
60
80
100

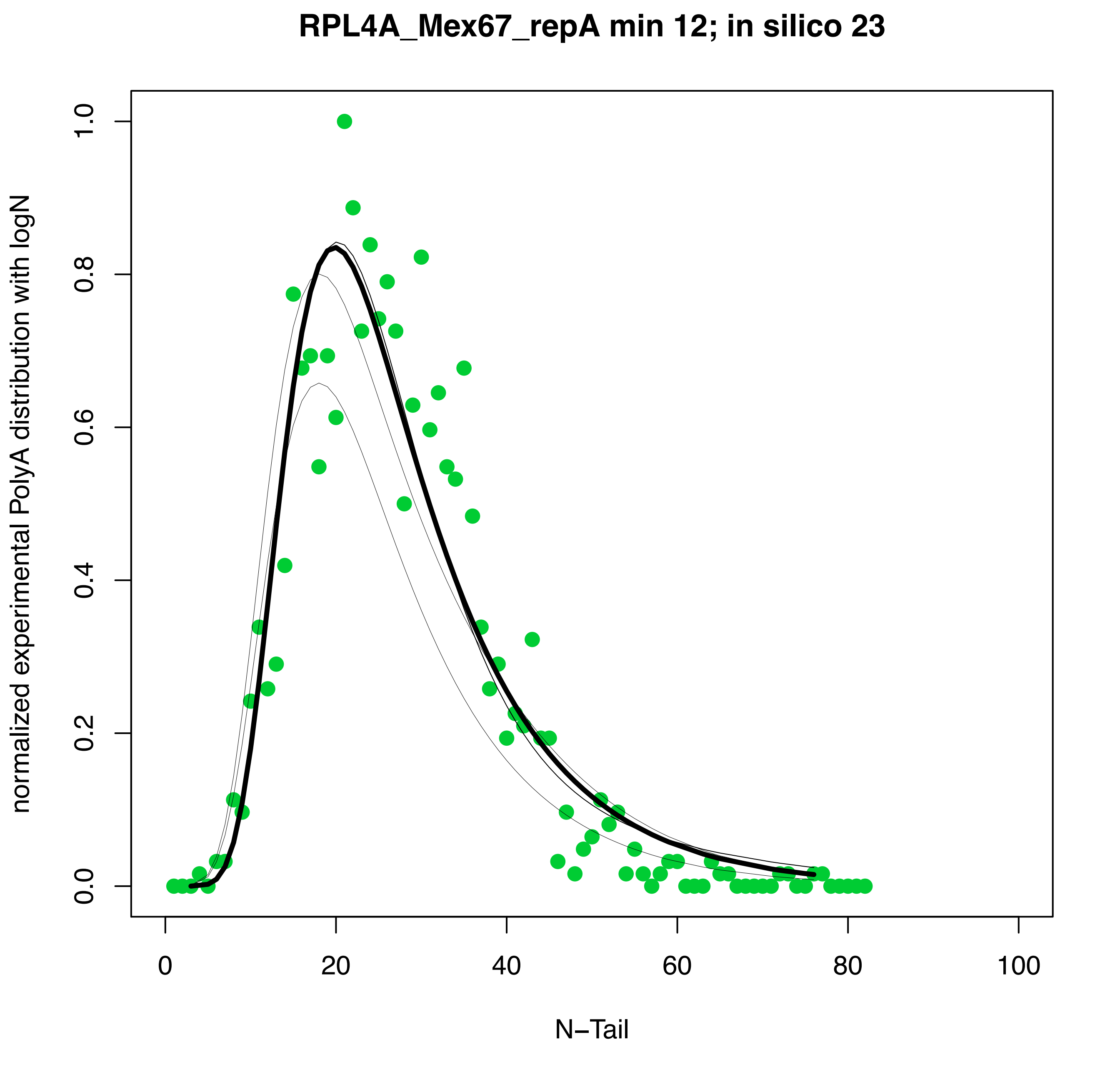
RPL4A_Mex67_repA min 12; in silico 23
normalized experimental PolyA distribution with logN
N−Tail
0.0
0.2
0.4
0.6
0.8
1.0
0
20
40
60
80
100

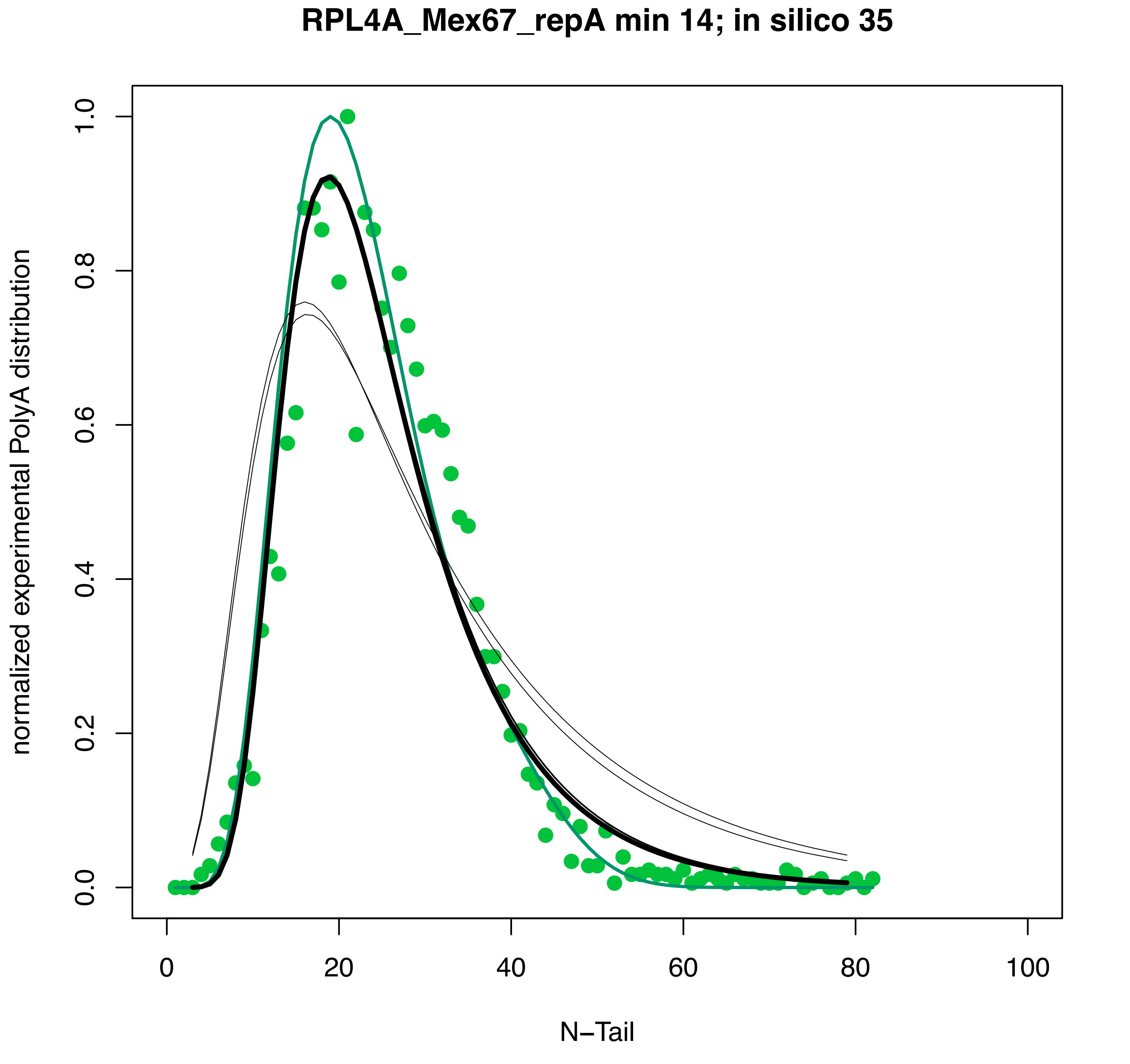
RPL4A_Mex67_repA min 14; in silico 35
normalized experimental PolyA distribution
N−Tail
0.0
0.2
0.4
0.6
0.8
1.0
0
20
40
60
80
100

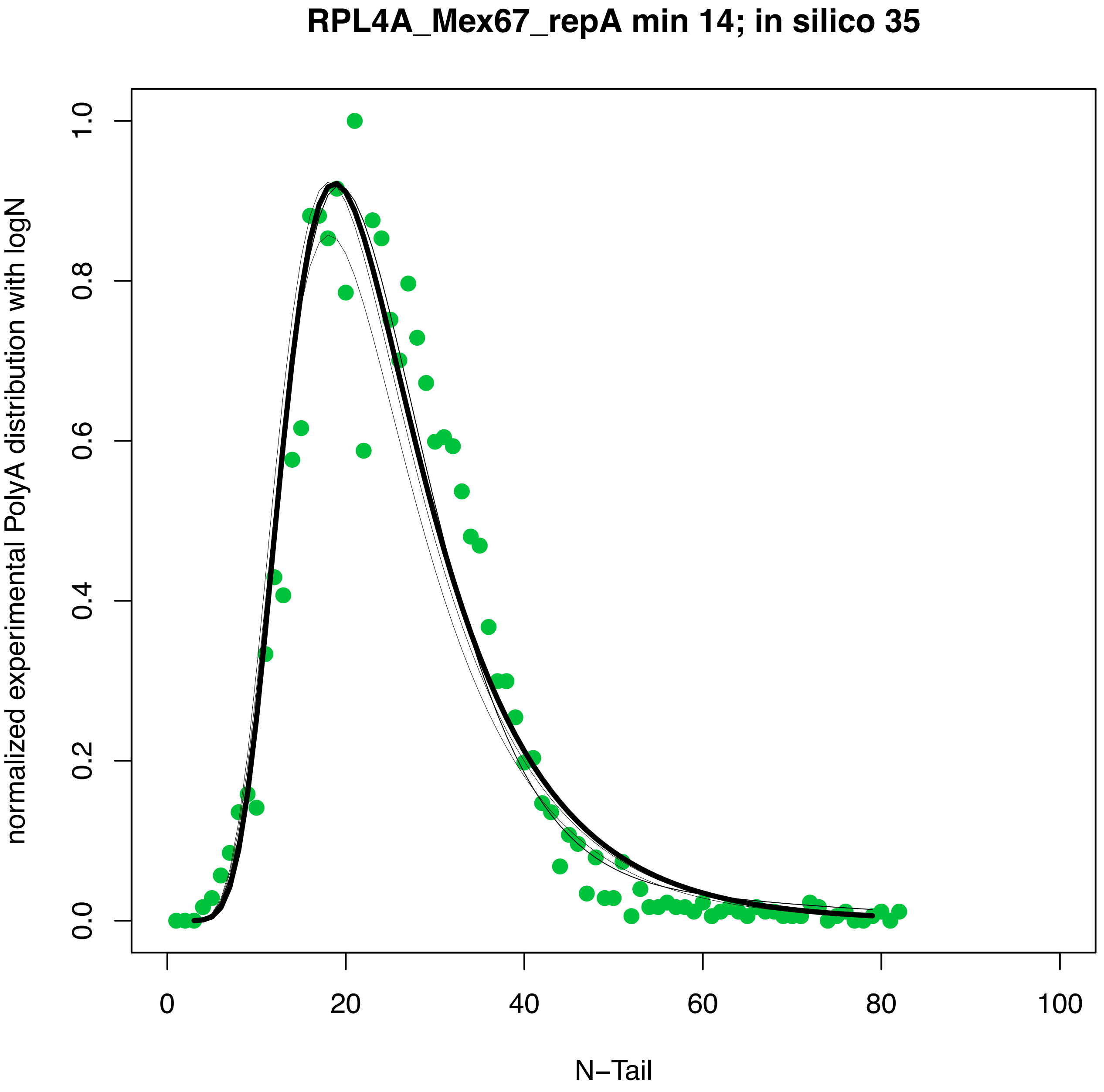

RPL4A_Mex67_repA min 14; in silico 35
normalized experimental PolyA distribution with logN
N−Tail
0.0
0.2
0.4
0.6
0.8
1.0
0
20
40
60
80
100

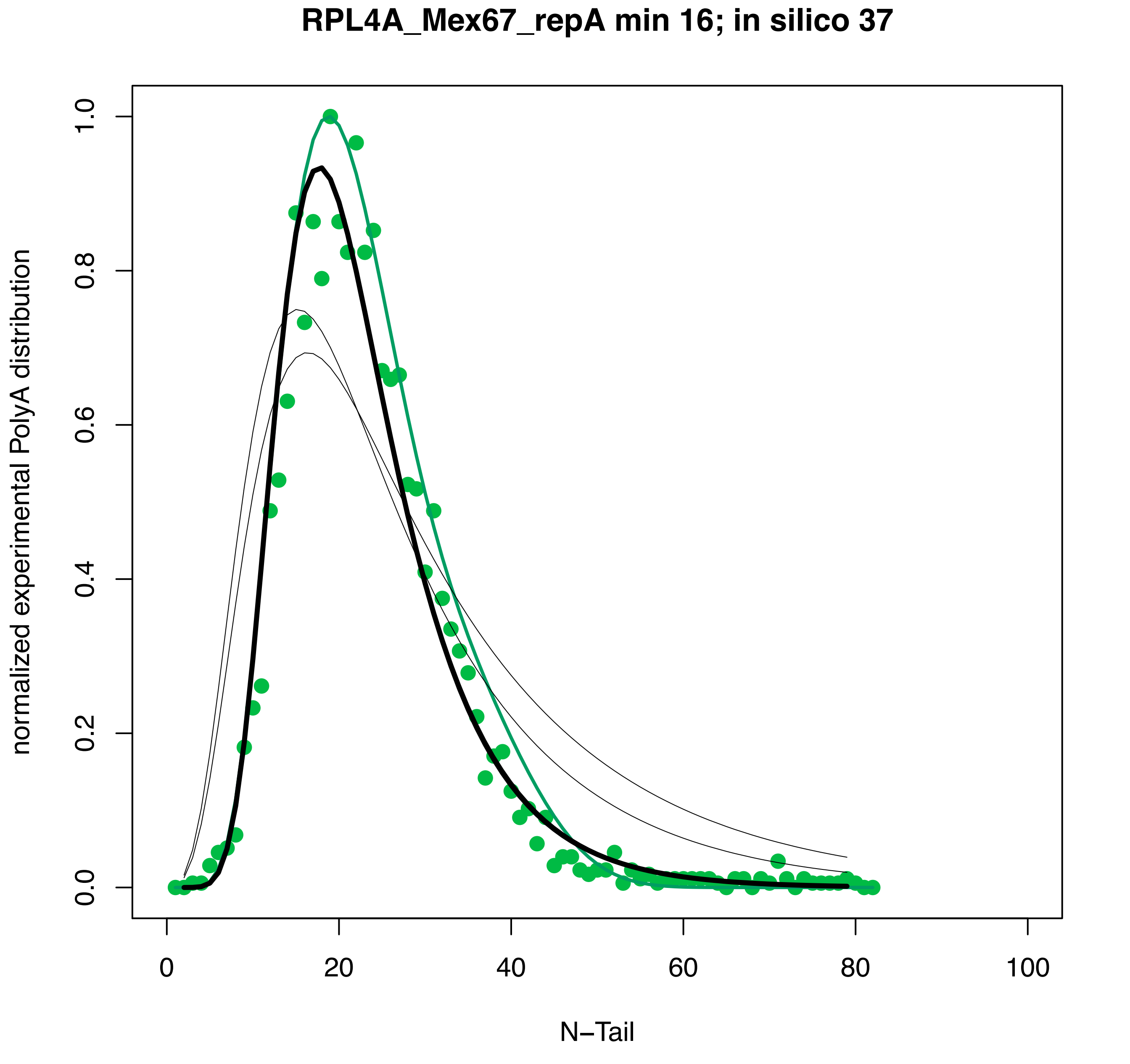

RPL4A_Mex67_repA min 16; in silico 37
normalized experimental PolyA distribution
N−Tail
0.0
0.2
0.4
0.6
0.8
1.0
0
20
40
60
80
100

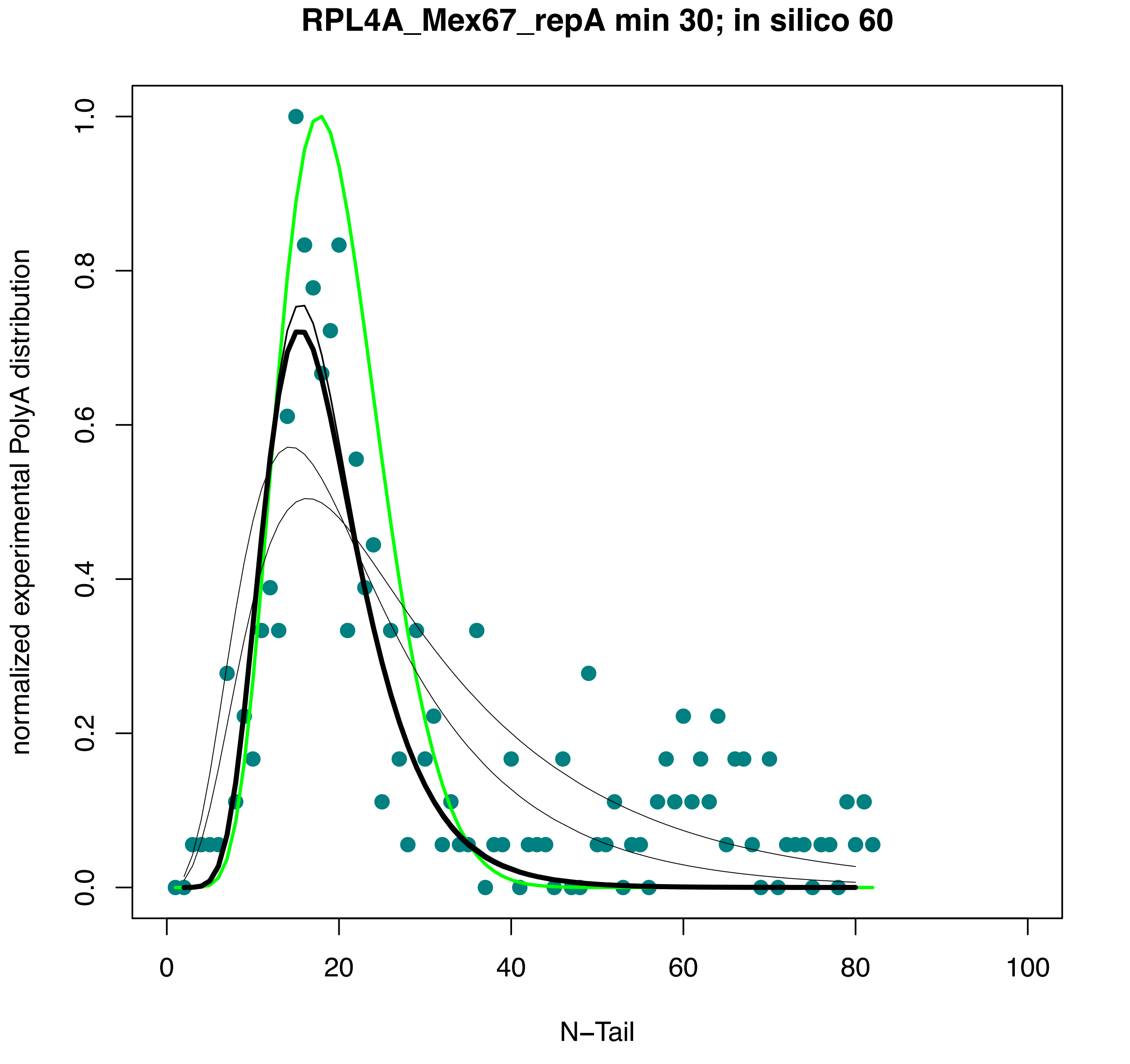
RPL4A_Mex67_repA min 30; in silico 60
normalized experimental PolyA distribution
N−Tail
0.0
0.2
0.4
0.6
0.8
1.0
0
20
40
60
80
100

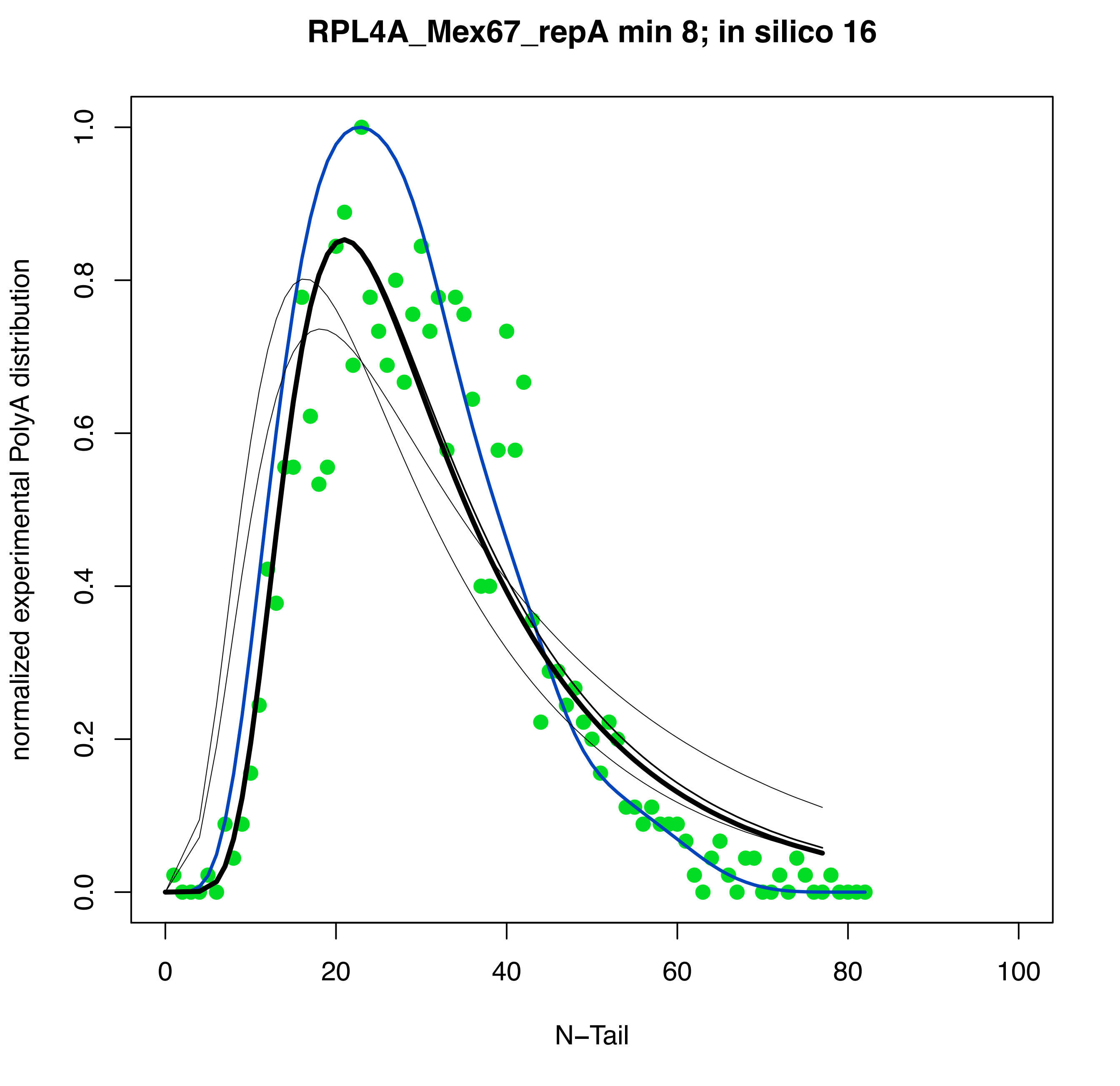

RPL4A_Mex67_repA min 8; in silico 16
normalized experimental PolyA distribution
N−Tail
0.0
0.2
0.4
0.6
0.8
1.0
0
20
40
60
80
100

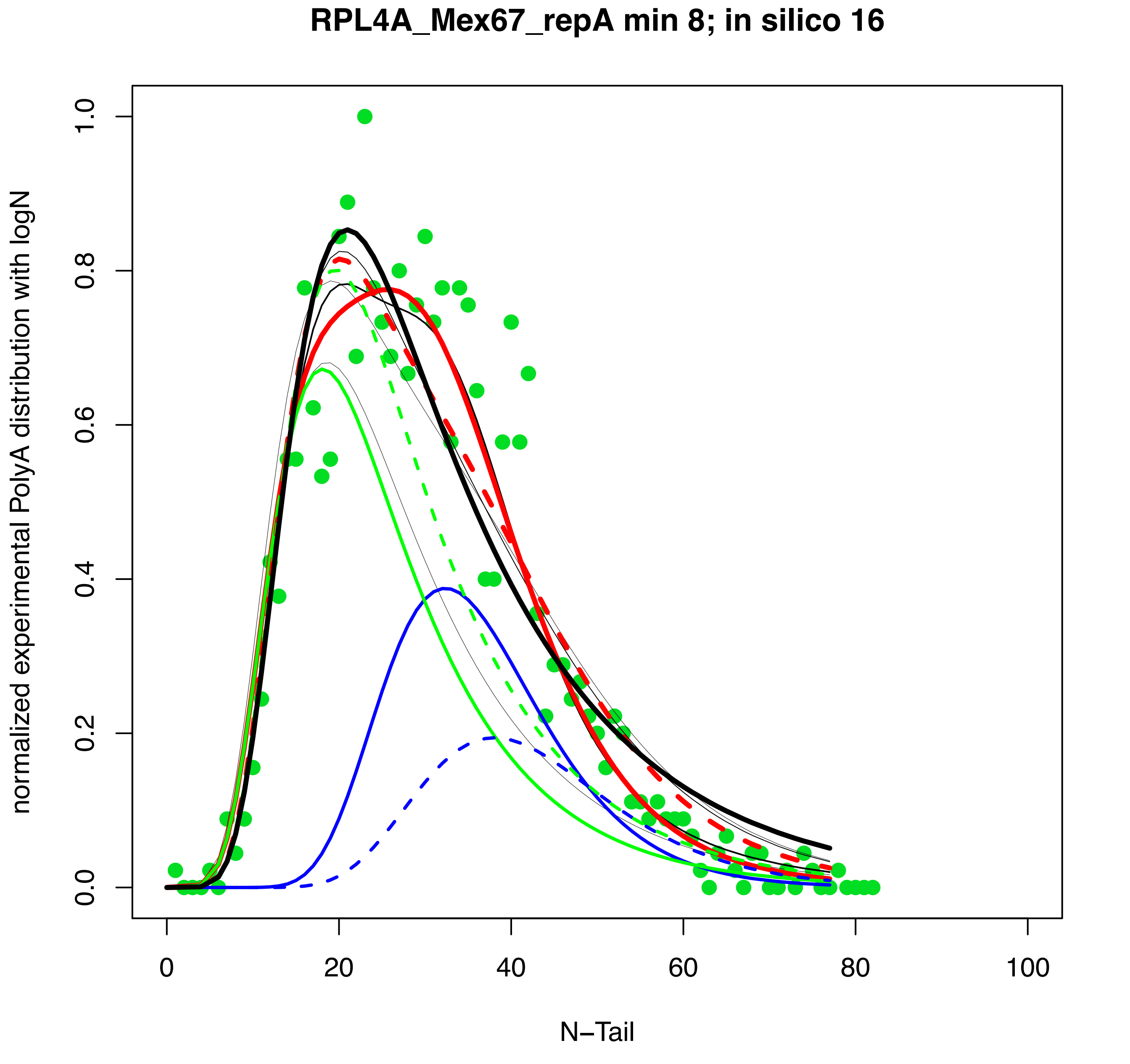
RPL4A_Mex67_repA min 8; in silico 16
normalized experimental PolyA distribution with logN
N−Tail
0.0
0.2
0.4
0.6
0.8
1.0
0
20
40
60
80
100

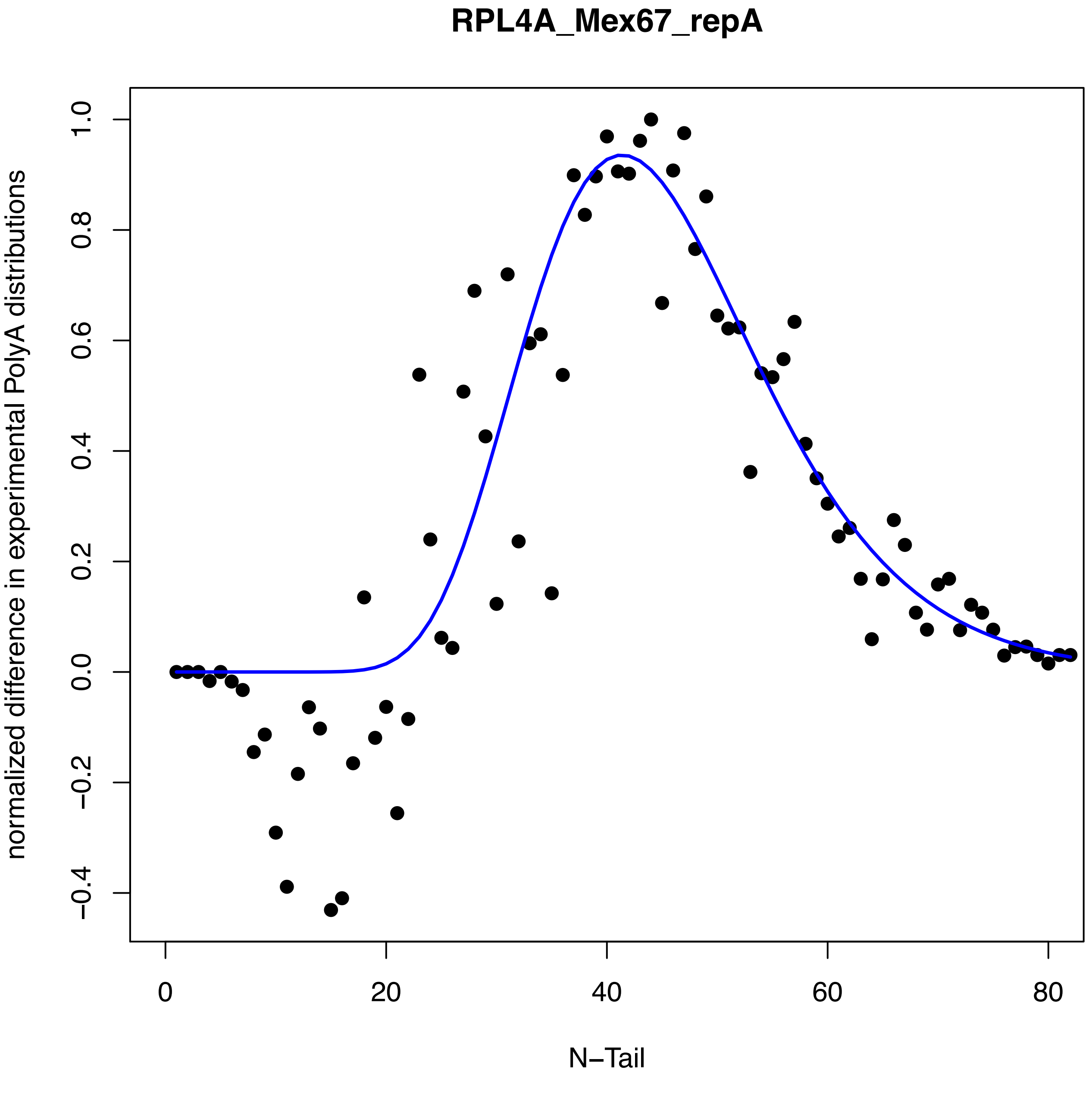
RPL4A_Mex67_repA
normalized difference in experimental PolyA distributions
N−Tail
1.0
0.8
0.6
0.4
0.2
0.0
−0.2
−0.4
0
20
40
60
80

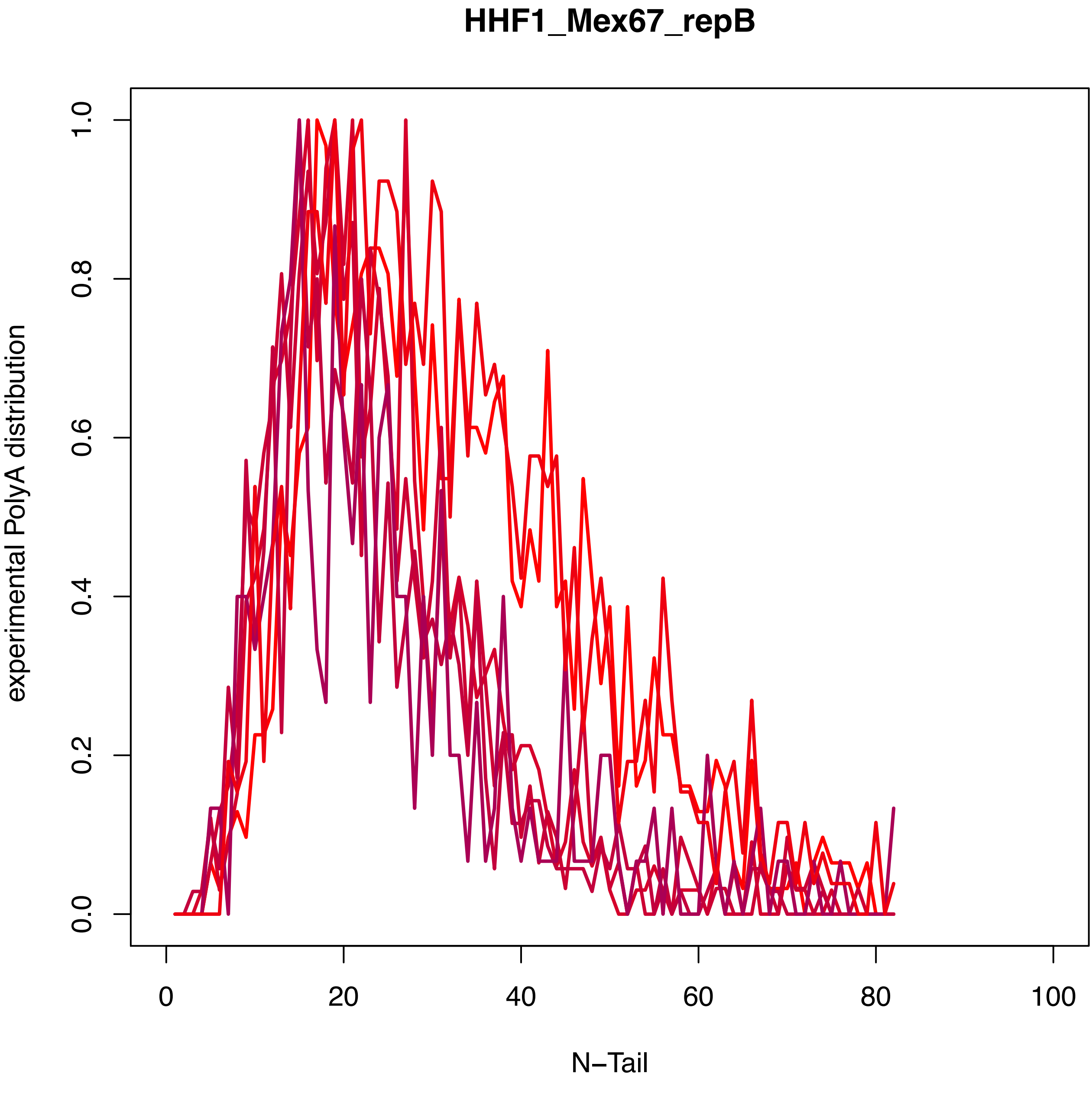

HHF1_Mex67_repB
experimental PolyA distribution
N−Tail
0.0
0.2
0.4
0.6
0.8
1.0
0
20
40
60
80
100

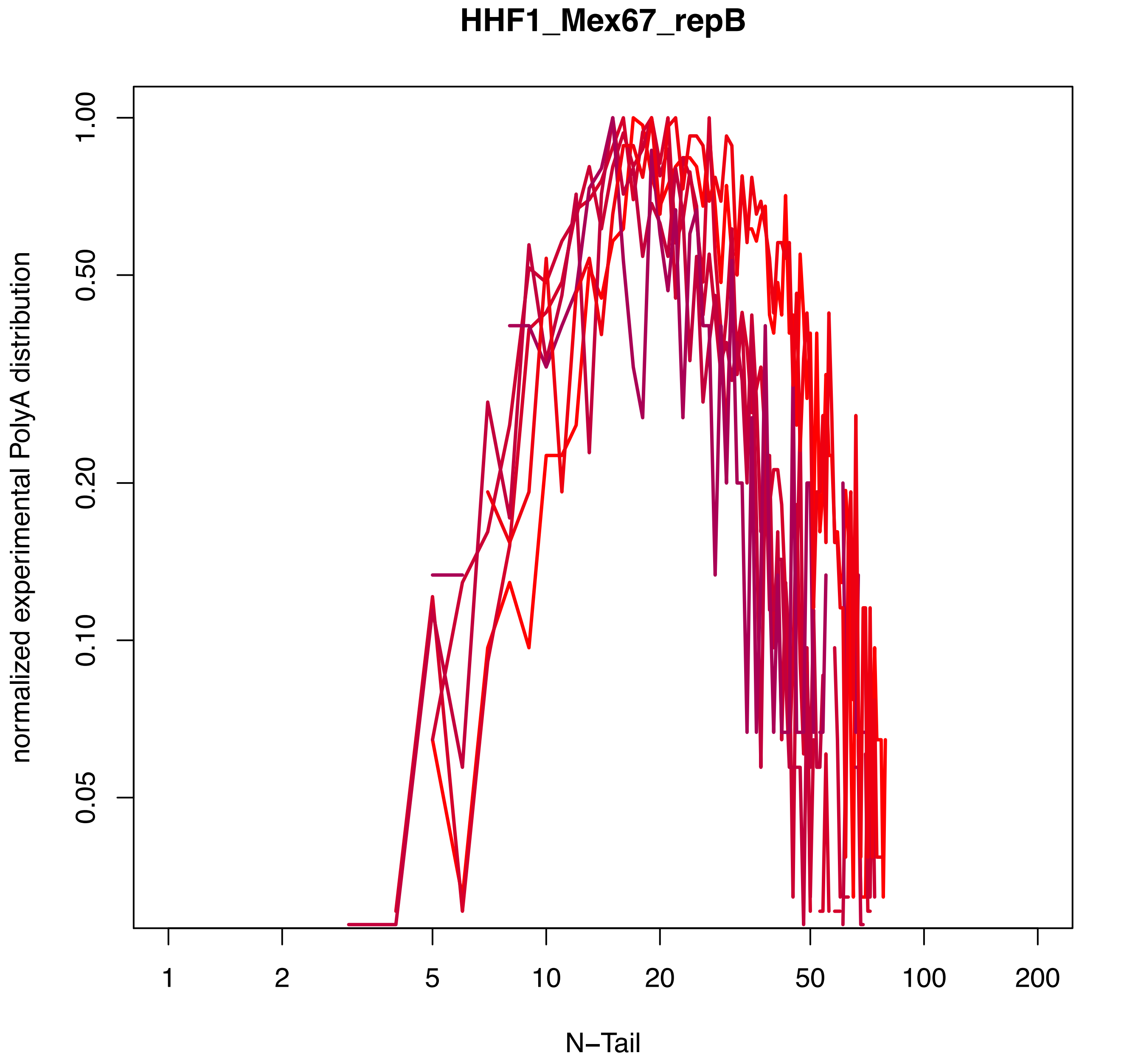

HHF1_Mex67_repB
normalized experimental PolyA distribution
1.00
0.50
0.20
0.10
0.05
1
2
5
10
20
50
100
200
N−Tail

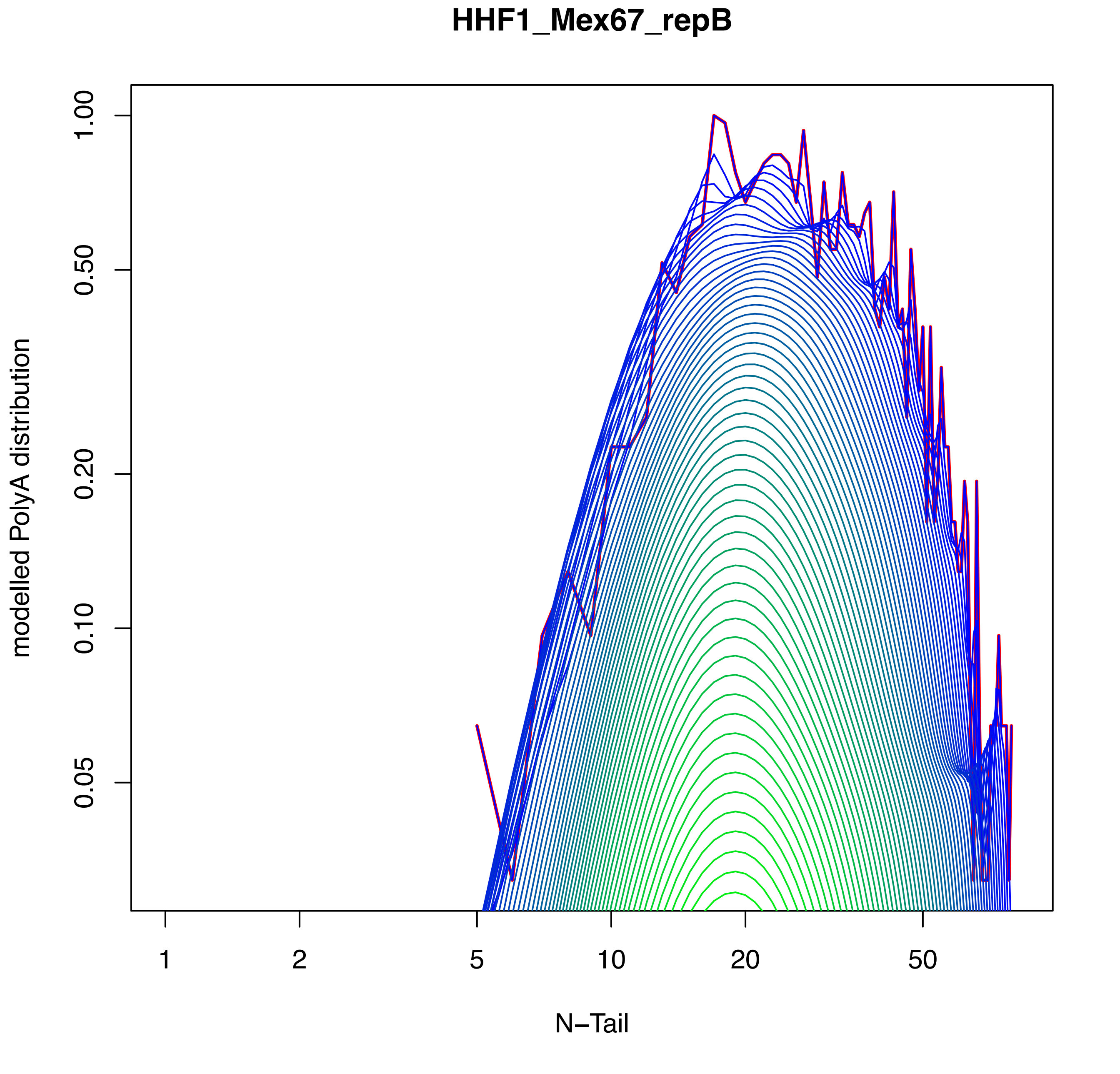

HHF1_Mex67_repB
modelled PolyA distribution
N−Tail
1.00
0.50
0.20
0.10
0.05
1
2
5
10
20
50

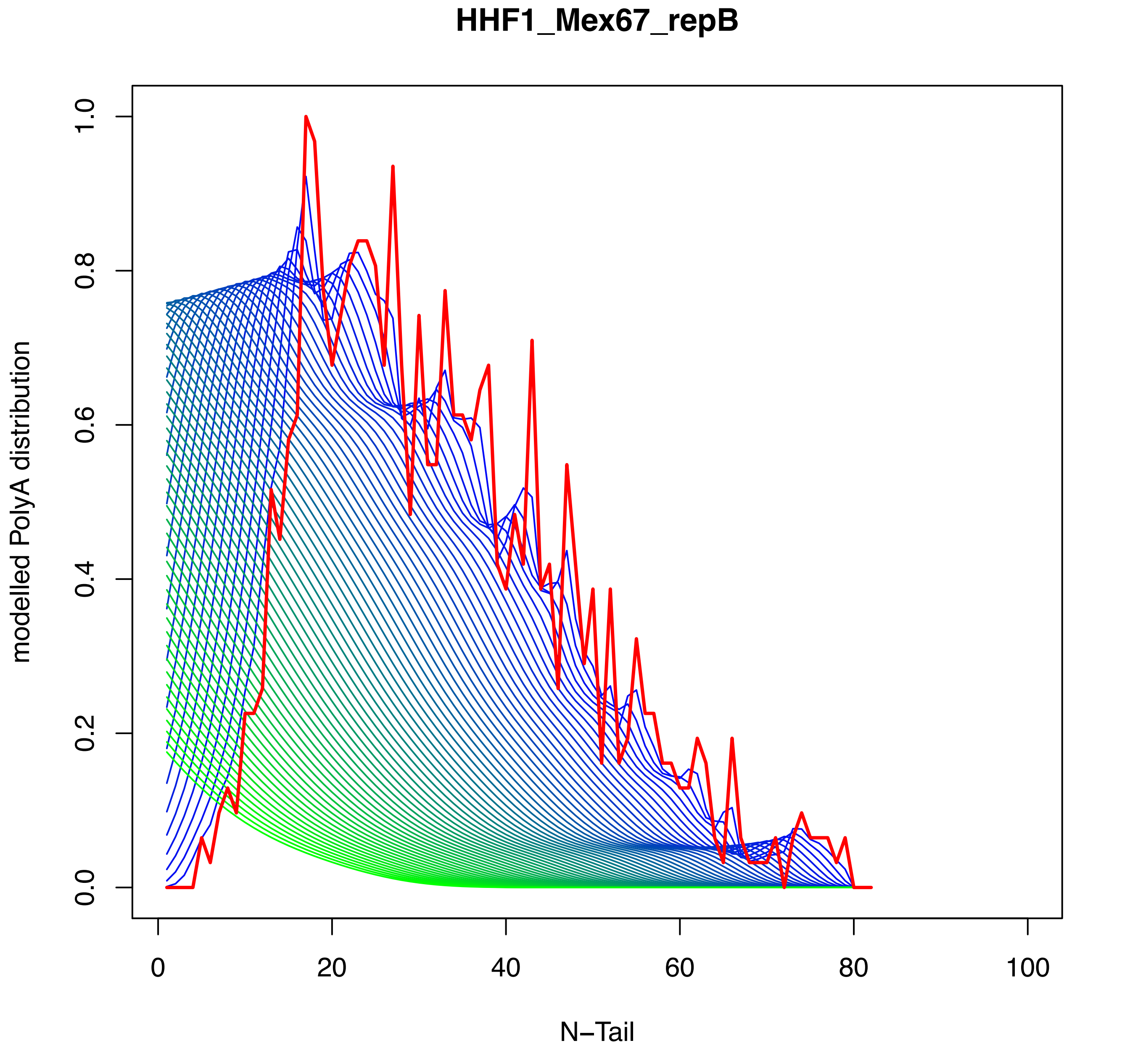

HHF1_Mex67_repB
modelled PolyA distribution
N−Tail
0.0
0.2
0.4
0.6
0.8
1.0
0
20
40
60
80
100

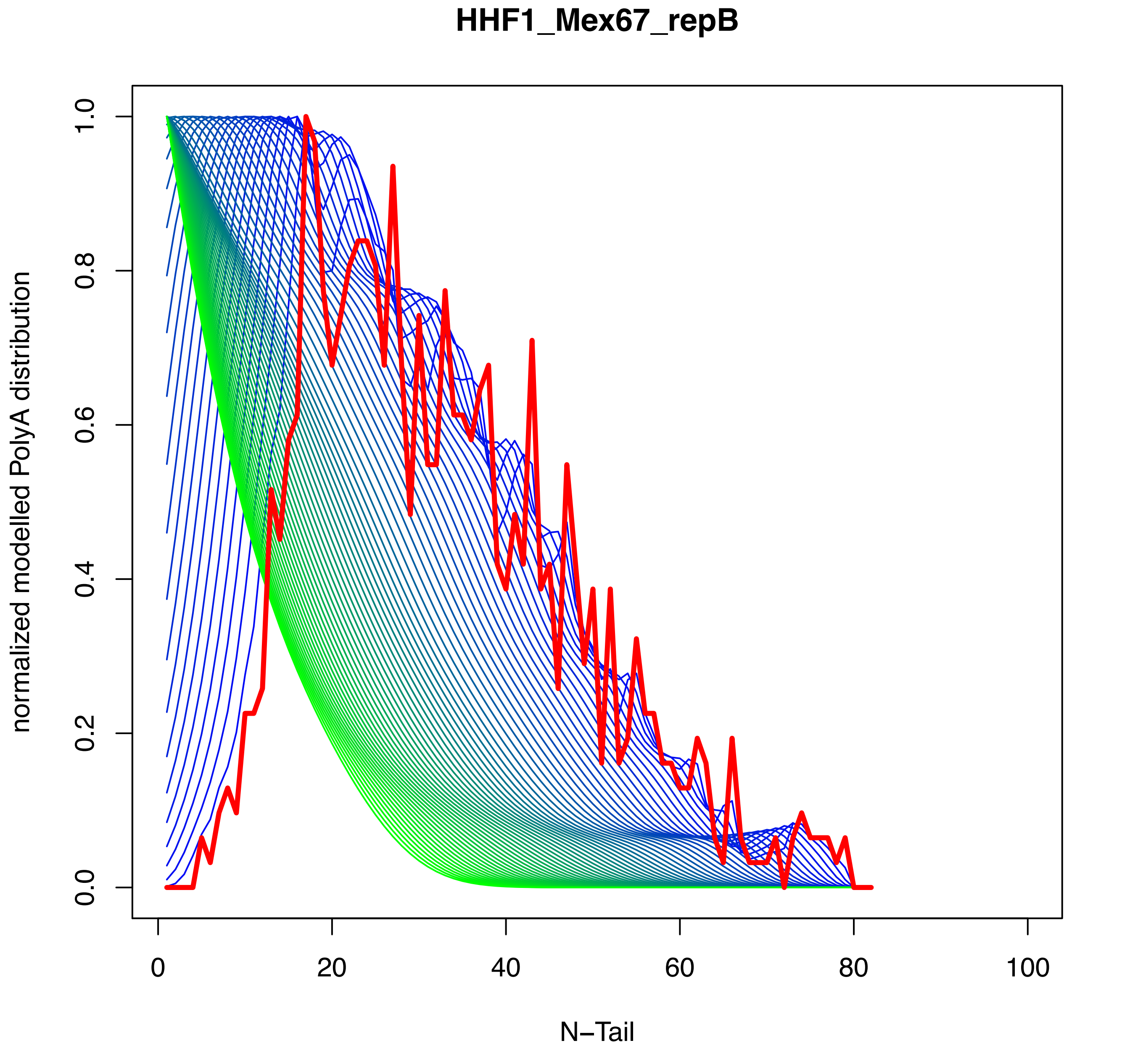
HHF1_Mex67_repB
normalized modelled PolyA distribution
0.0
0.2
0.4
0.6
0.8
1.0
0
20
40
60
80
100
N−Tail

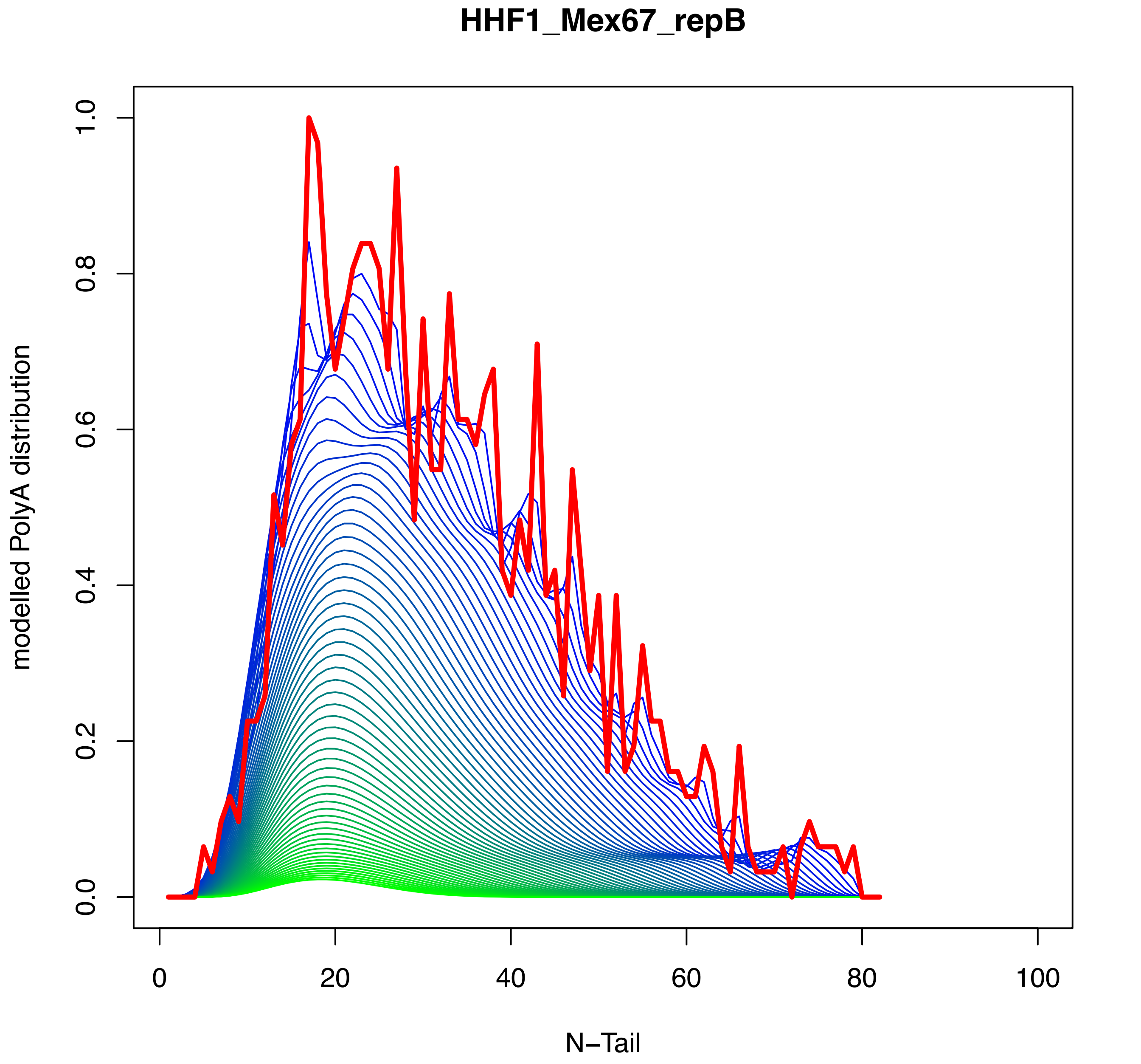

HHF1_Mex67_repB
modelled PolyA distribution
N−Tail
0.0
0.2
0.4
0.6
0.8
1.0
0
20
40
60
80
100

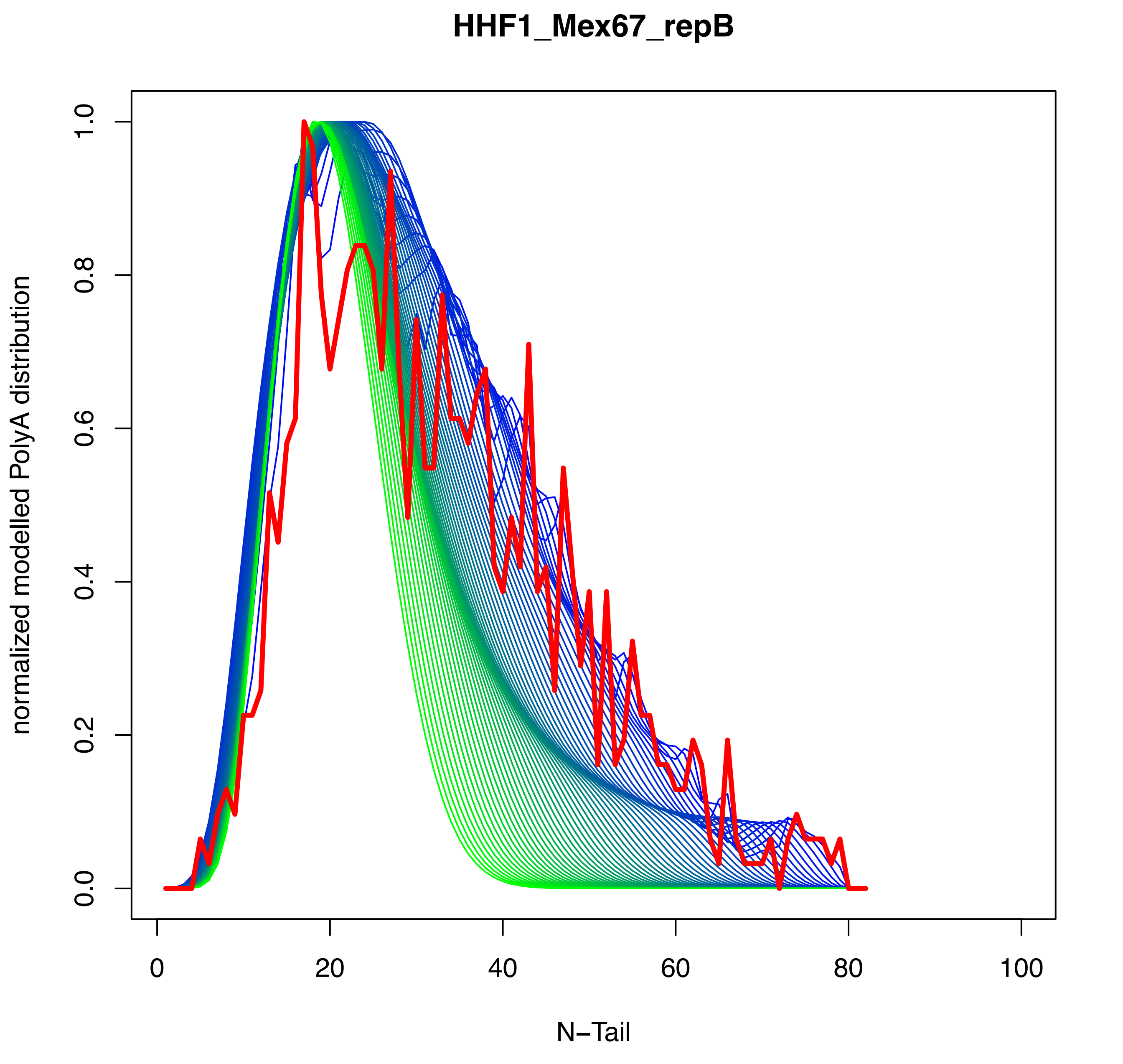

HHF1_Mex67_repB
normalized modelled PolyA distribution
N−Tail
0.0
0.2
0.4
0.6
0.8
1.0
0
20
40
60
80
100

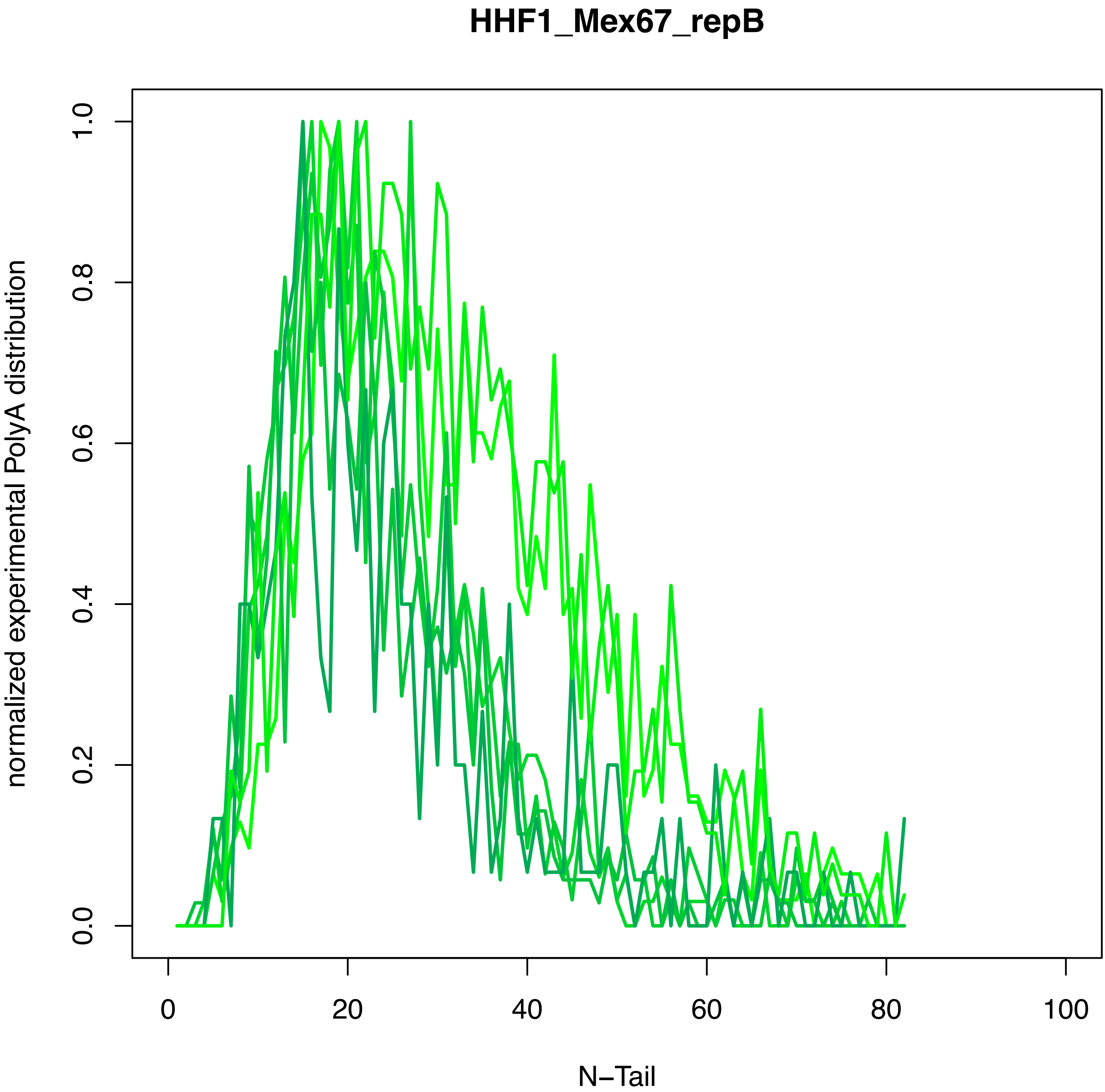

HHF1_Mex67_repB
normalized experimental PolyA distribution
N−Tail
0.0
0.2
0.4
0.6
0.8
1.0
0
20
40
60
80
100

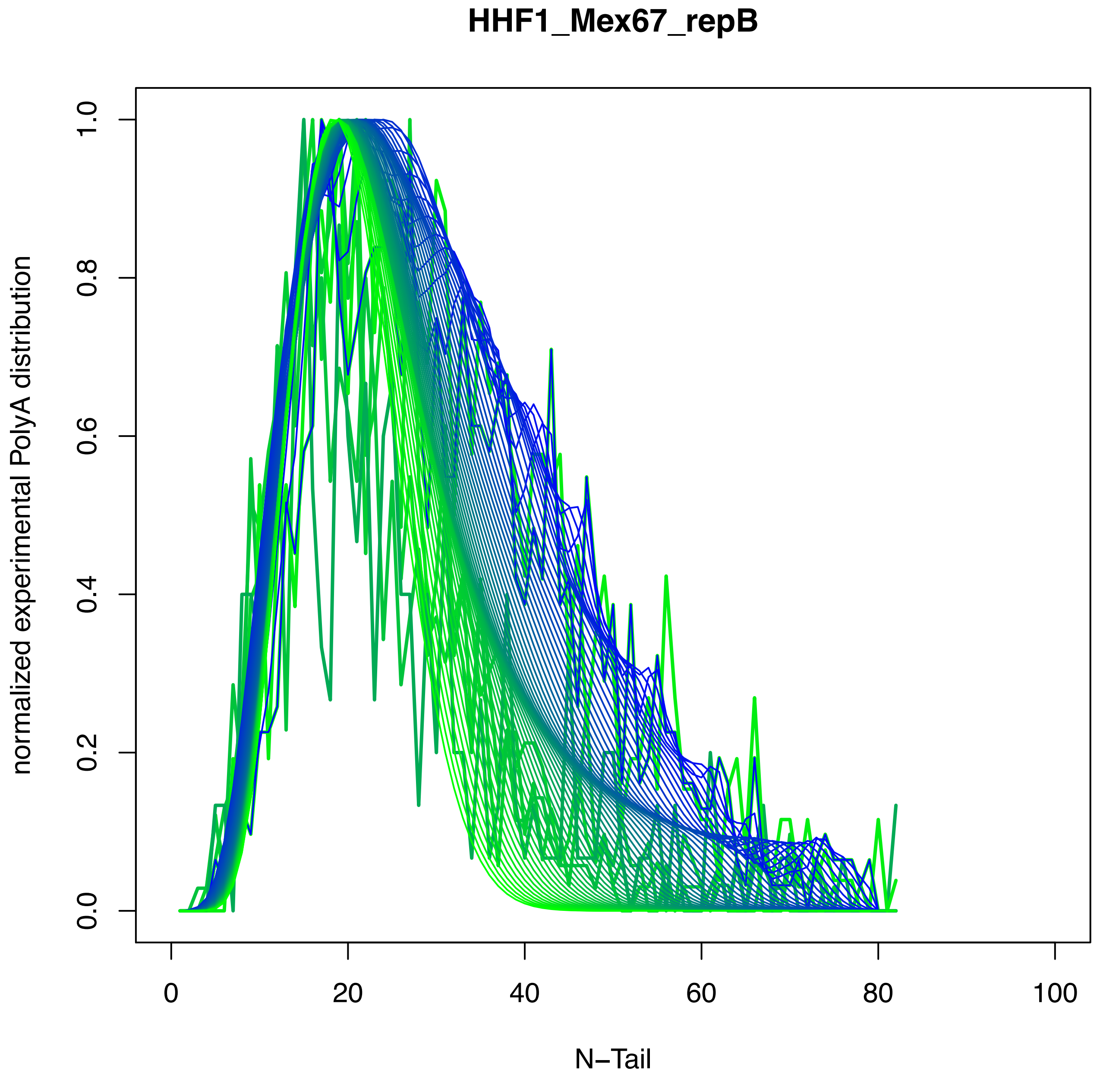

HHF1_Mex67_repB
normalized experimental PolyA distribution
0.0
0.2
0.4
0.6
0.8
1.0
0
20
40
60
80
100
N−Tail

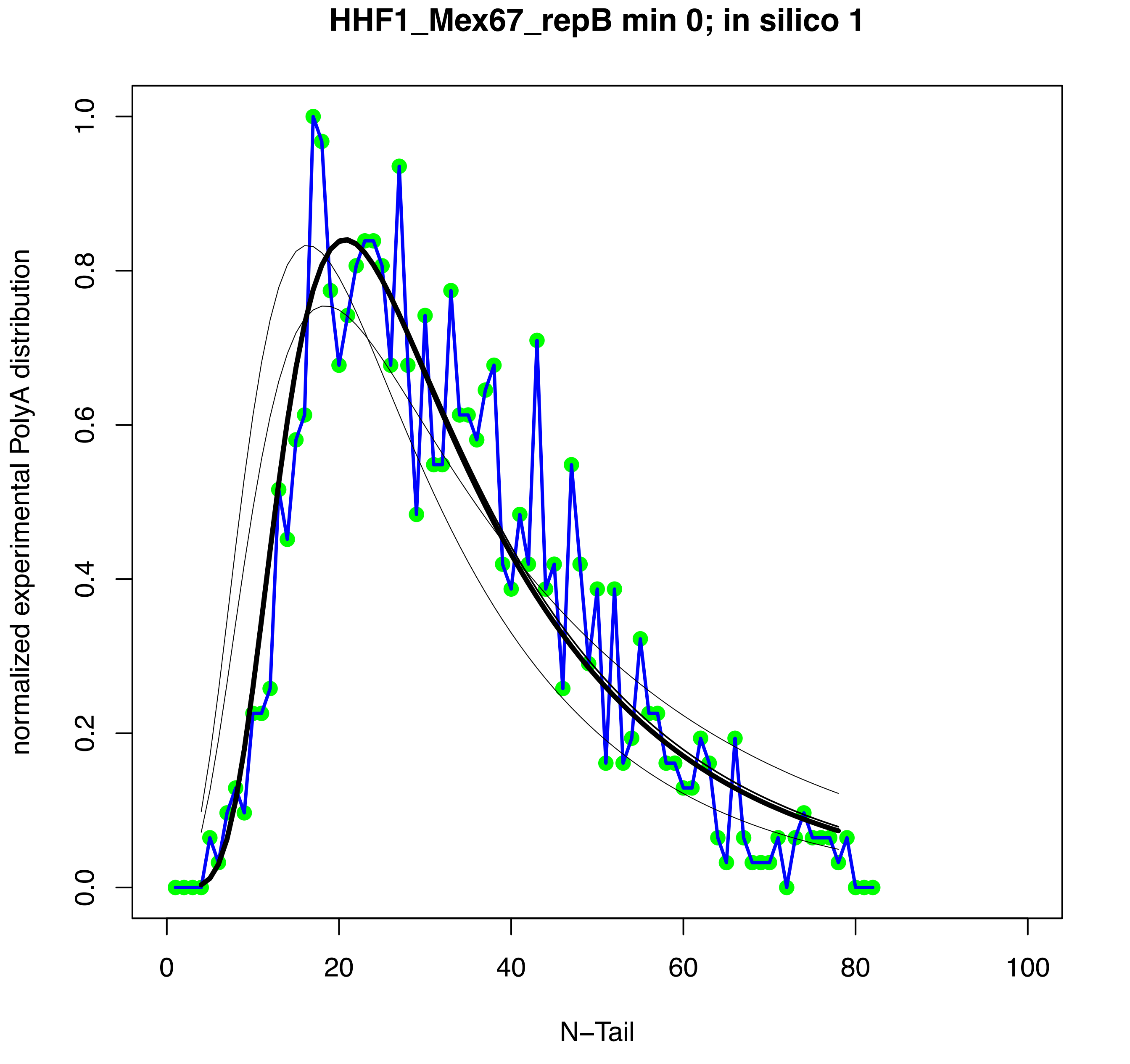
HHF1_Mex67_repB min 0; in silico 1
normalized experimental PolyA distribution
N−Tail
0.0
0.2
0.4
0.6
0.8
1.0
0
20
40
60
80
100

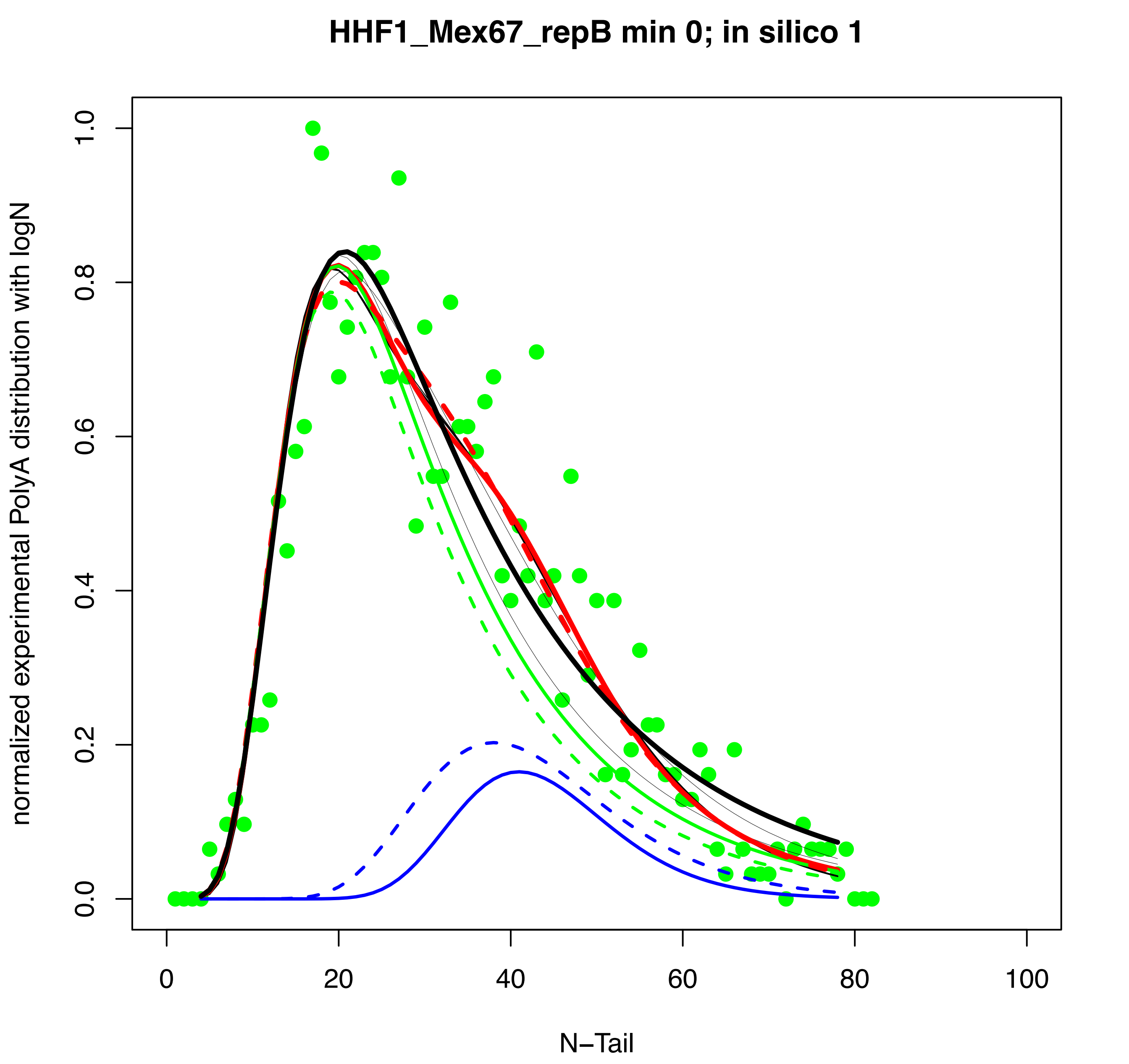
HHF1_Mex67_repB min 0; in silico 1
normalized experimental PolyA distribution with logN
N−Tail
0.0
0.2
0.4
0.6
0.8
1.0
0
20
40
60
80
100

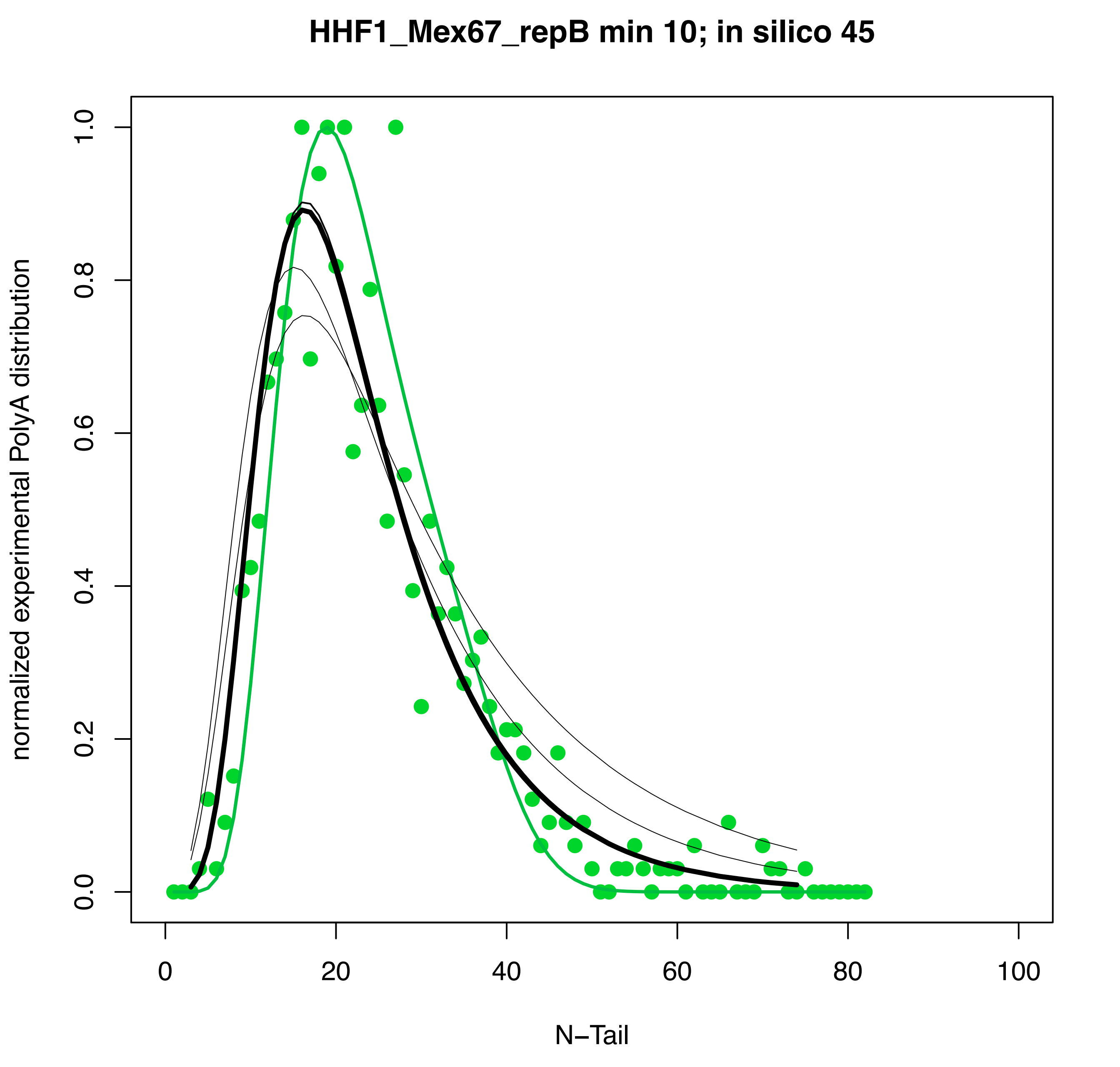
HHF1_Mex67_repB min 10; in silico 45
normalized experimental PolyA distribution
N−Tail
0.0
0.2
0.4
0.6
0.8
1.0
0
20
40
60
80
100

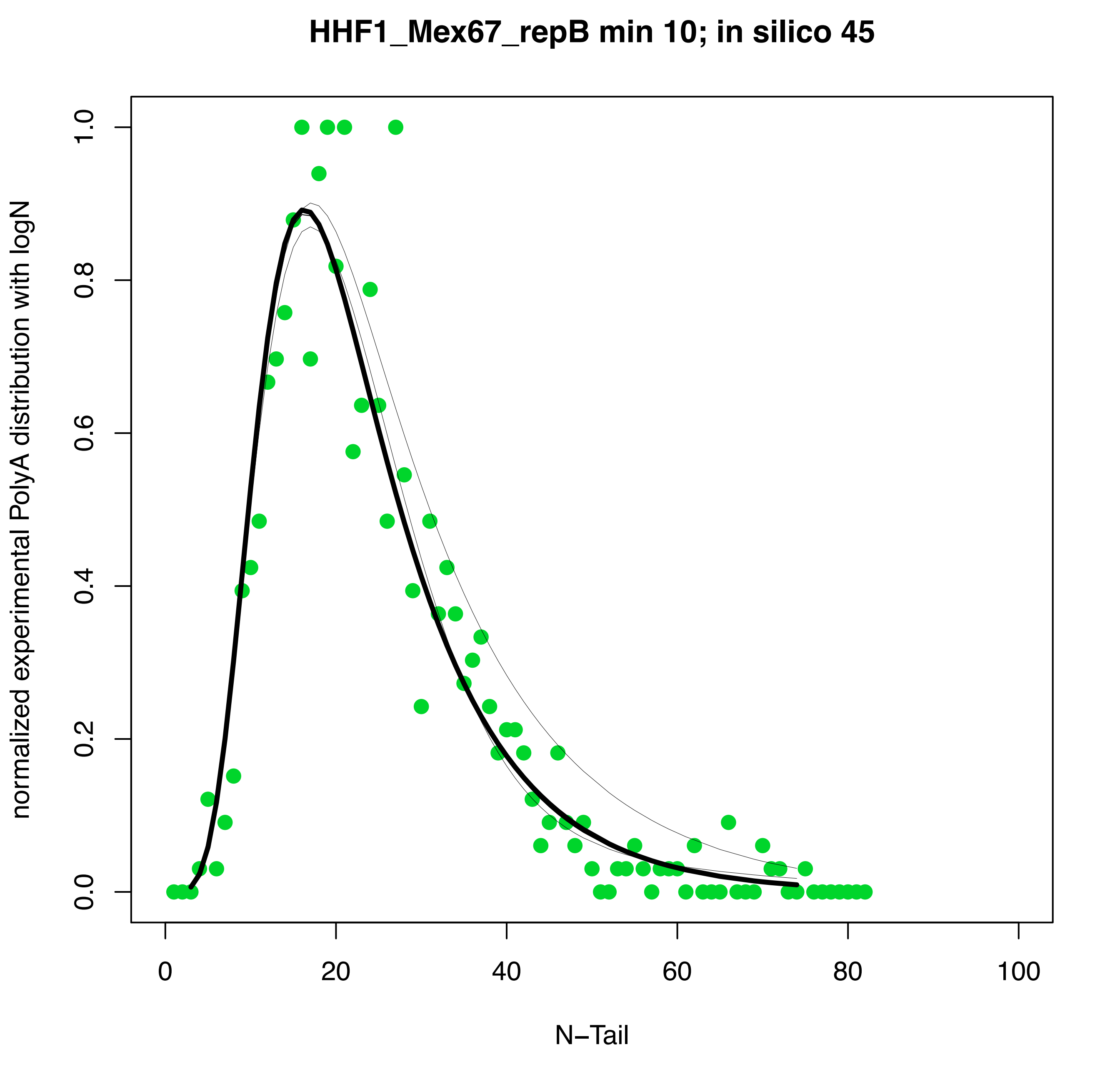
HHF1_Mex67_repB min 10; in silico 45
normalized experimental PolyA distribution with logN
N−Tail
0.0
0.2
0.4
0.6
0.8
1.0
0
20
40
60
80
100

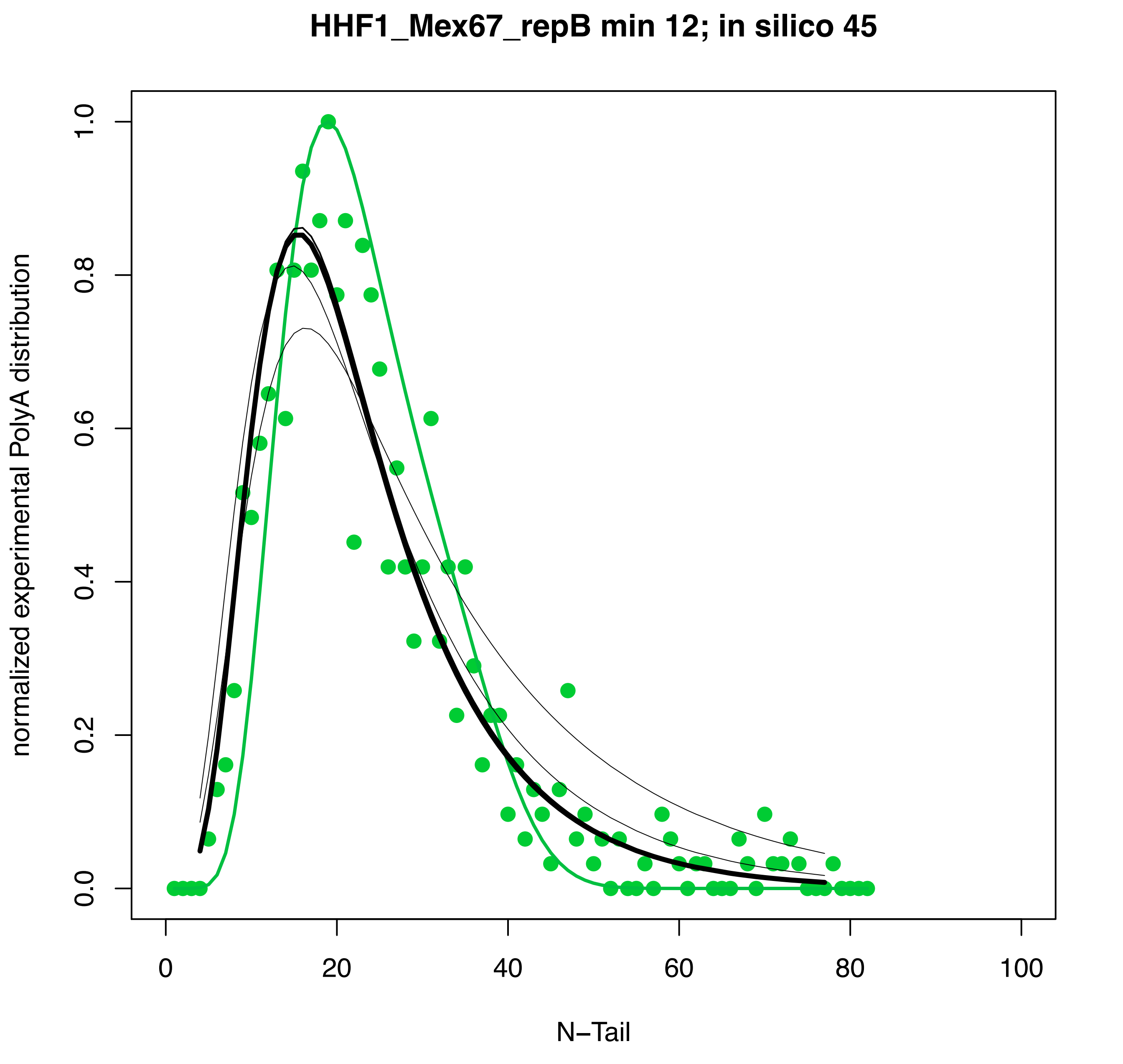

HHF1_Mex67_repB min 12; in silico 45
normalized experimental PolyA distribution
0.0
0.2
0.4
0.6
0.8
1.0
0
20
40
60
80
100
N−Tail

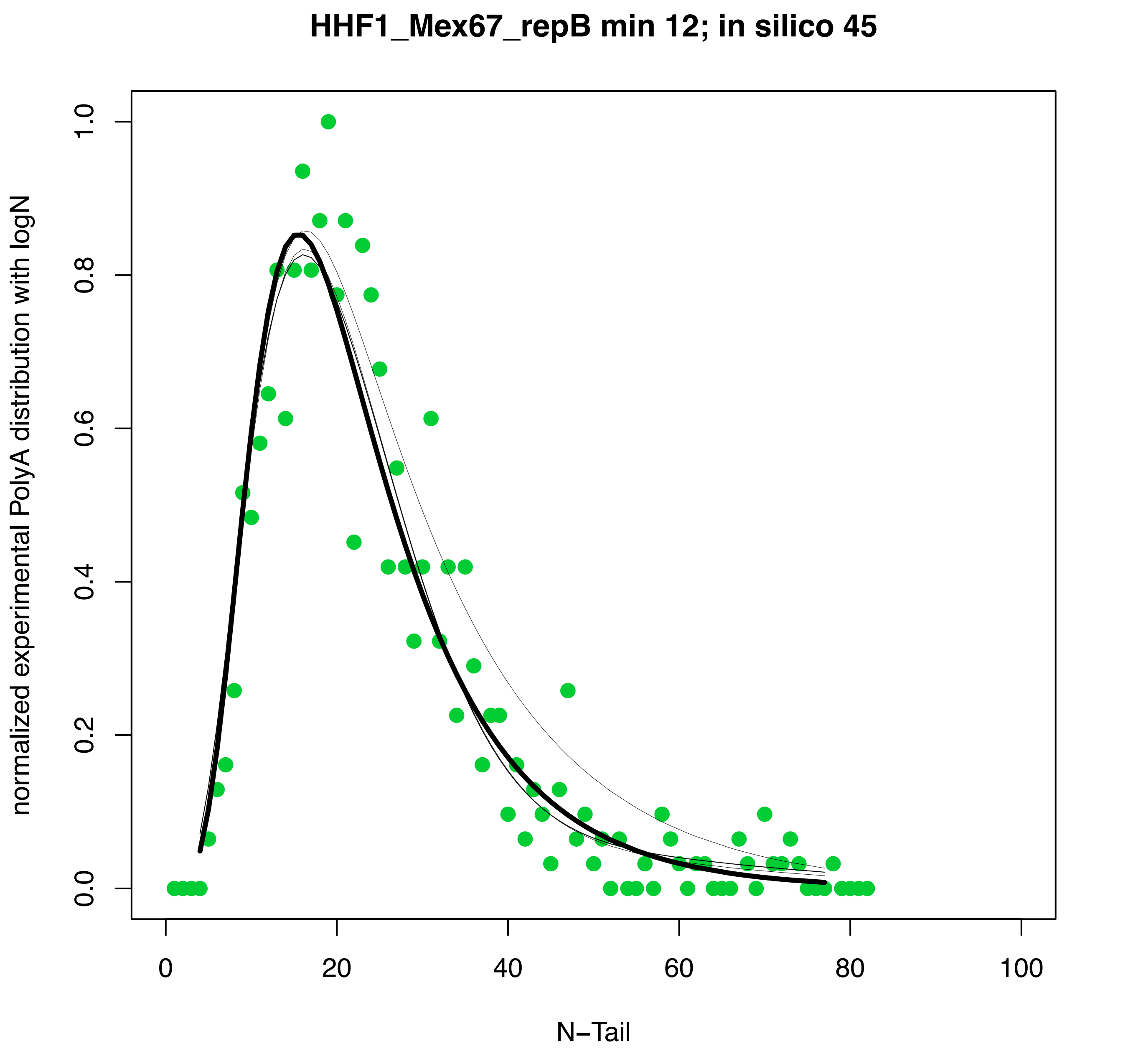

HHF1_Mex67_repB min 12; in silico 45
normalized experimental PolyA distribution with logN
N−Tail
0.0
0.2
0.4
0.6
0.8
1.0
0
20
40
60
80
100

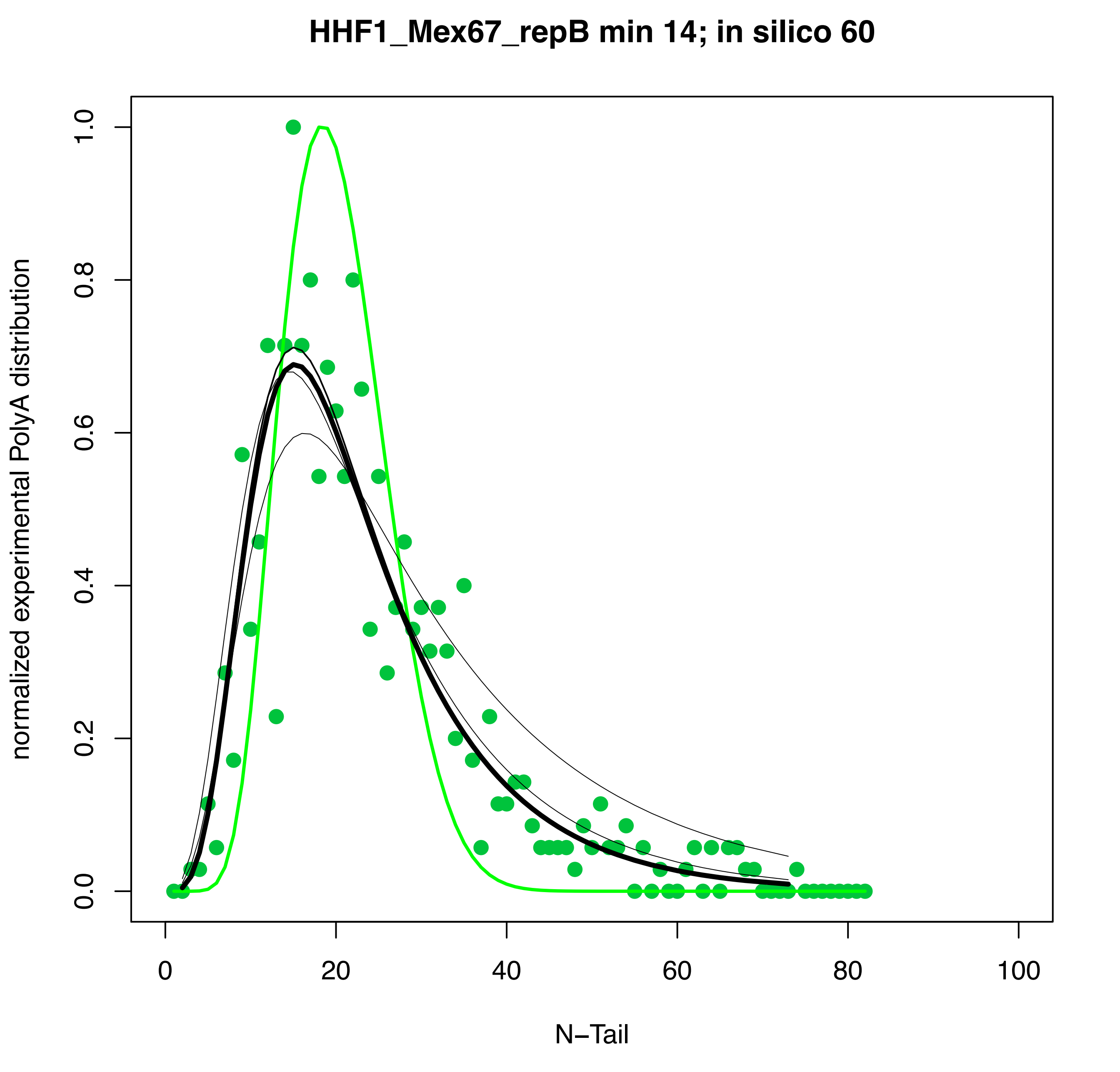

HHF1_Mex67_repB min 14; in silico 60
normalized experimental PolyA distribution
N−Tail
0.0
0.2
0.4
0.6
0.8
1.0
0
20
40
60
80
100

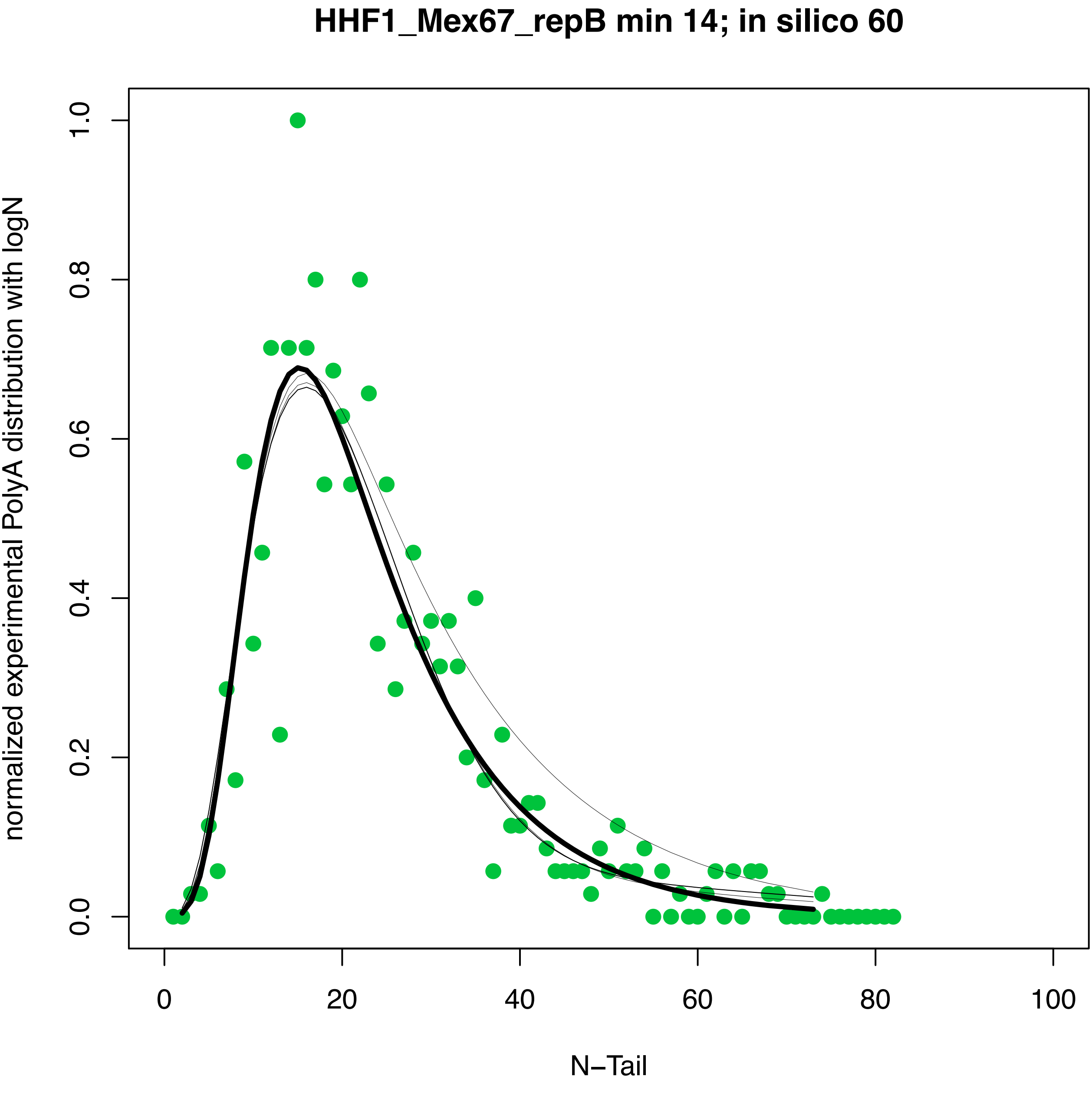

HHF1_Mex67_repB min 14; in silico 60
normalized experimental PolyA distribution with logN
N−Tail
0.0
0.2
0.4
0.6
0.8
1.0
0
20
40
60
80
100

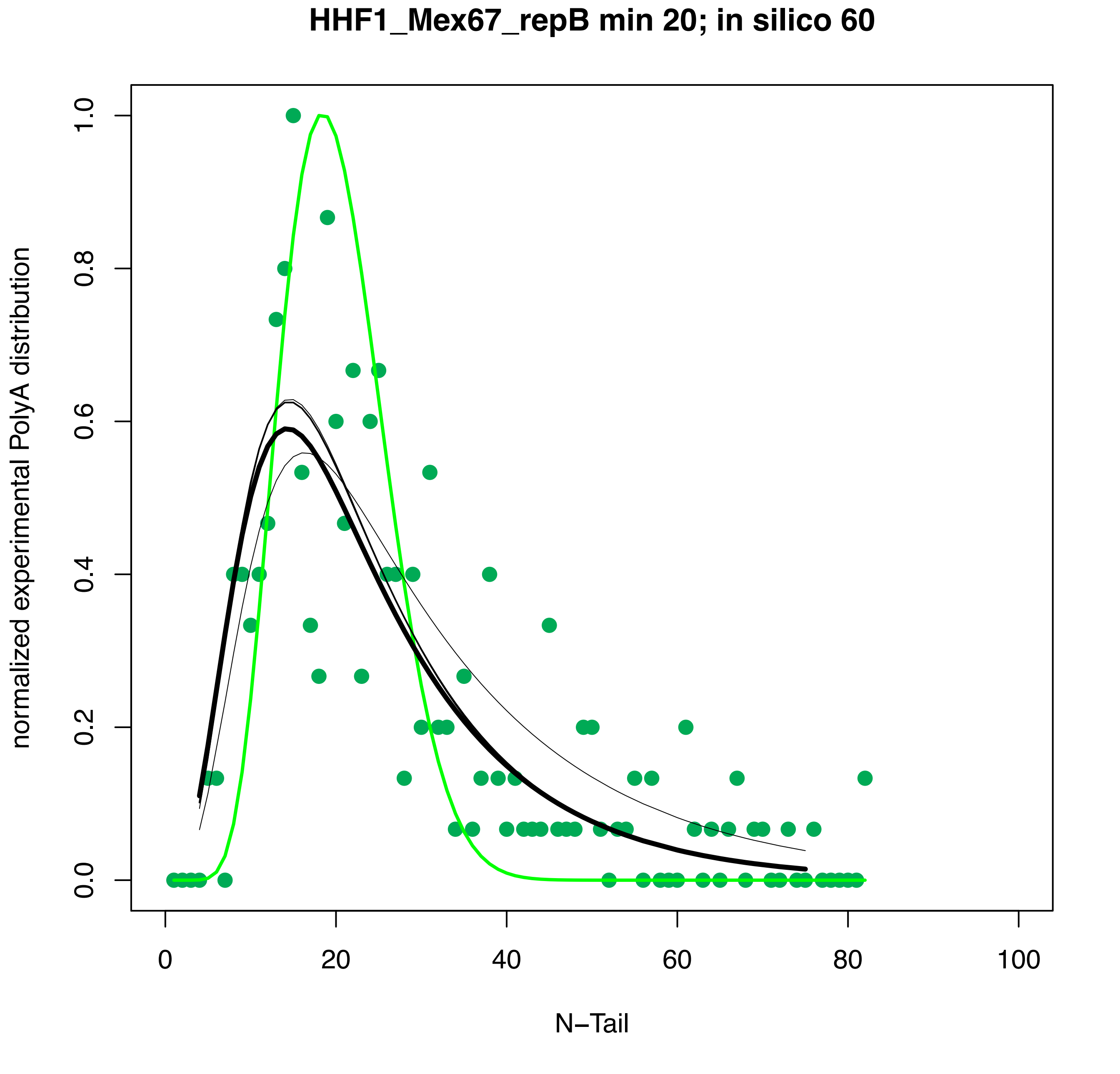

HHF1_Mex67_repB min 20; in silico 60
normalized experimental PolyA distribution
N−Tail
0.0
0.2
0.4
0.6
0.8
1.0
0
20
40
60
80
100

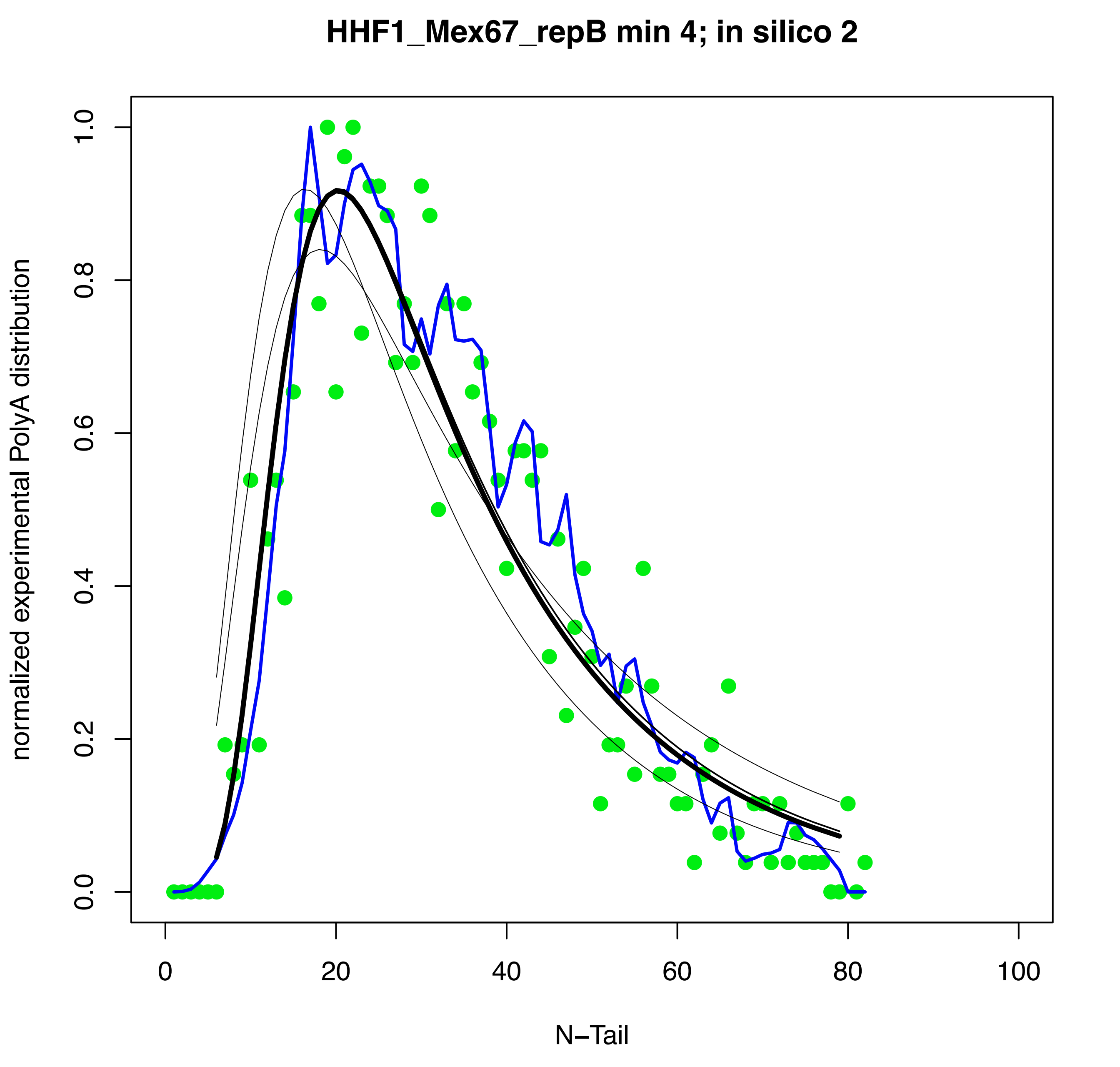
HHF1_Mex67_repB min 4; in silico 2
normalized experimental PolyA distribution
N−Tail
0.0
0.2
0.4
0.6
0.8
1.0
0
20
40
60
80
100

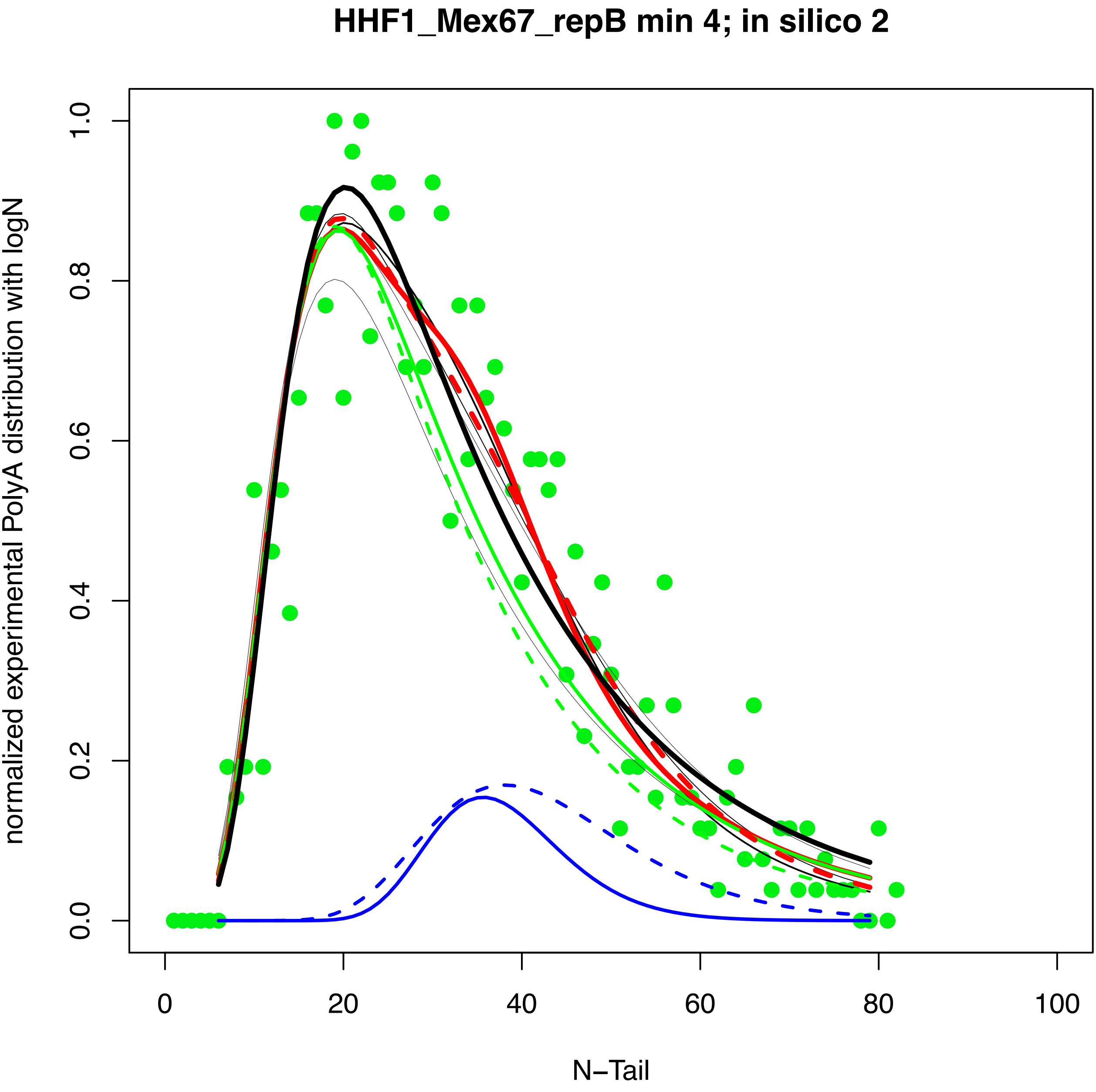

HHF1_Mex67_repB min 4; in silico 2
normalized experimental PolyA distribution with logN
N−Tail
0.0
0.2
0.4
0.6
0.8
1.0
0
20
40
60
80
100

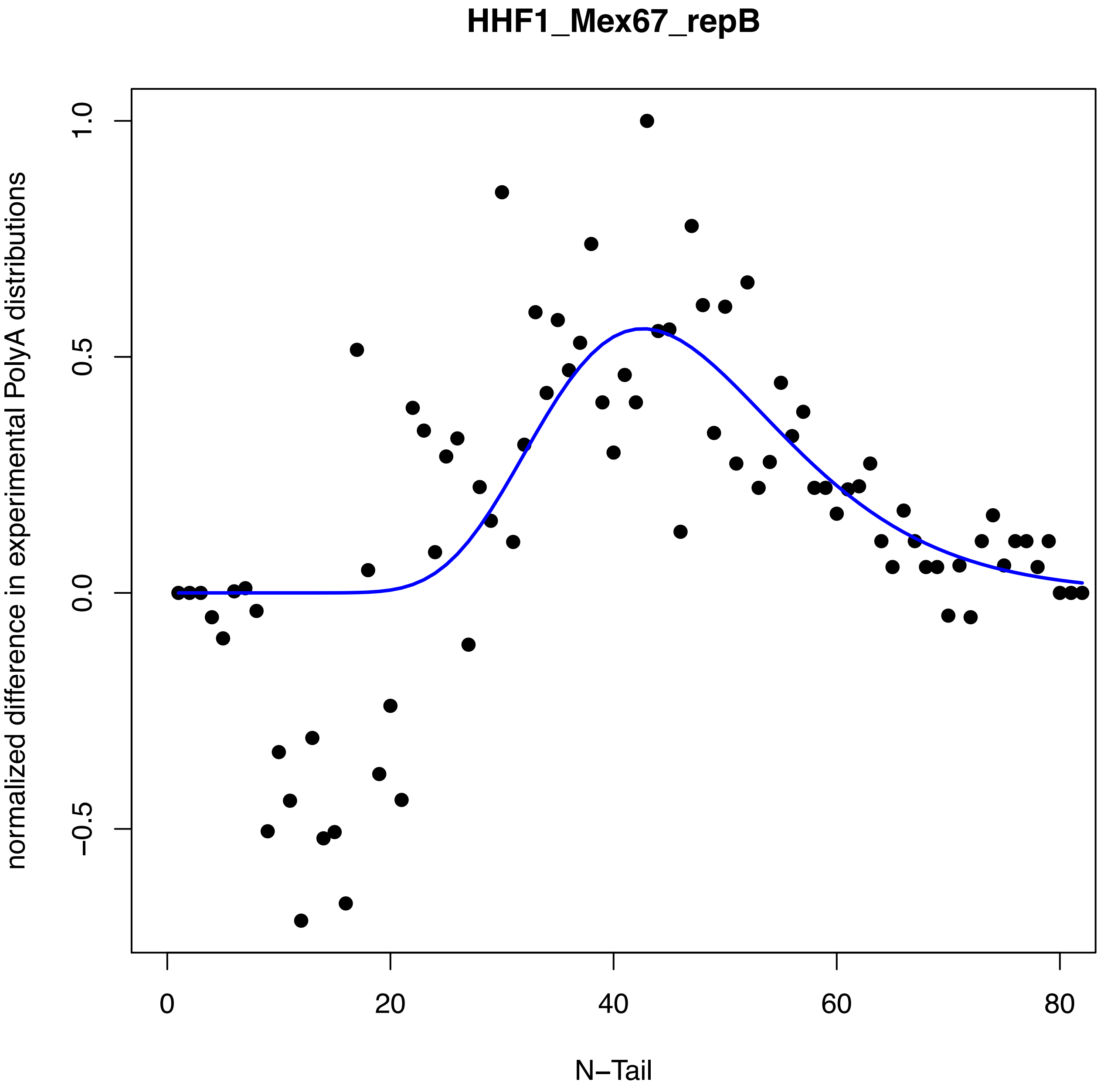
HHF1_Mex67_repB
normalized difference in experimental PolyA distributions
N−Tail
−0.5
0.0
0.5
1.0
0
20
40
60
80

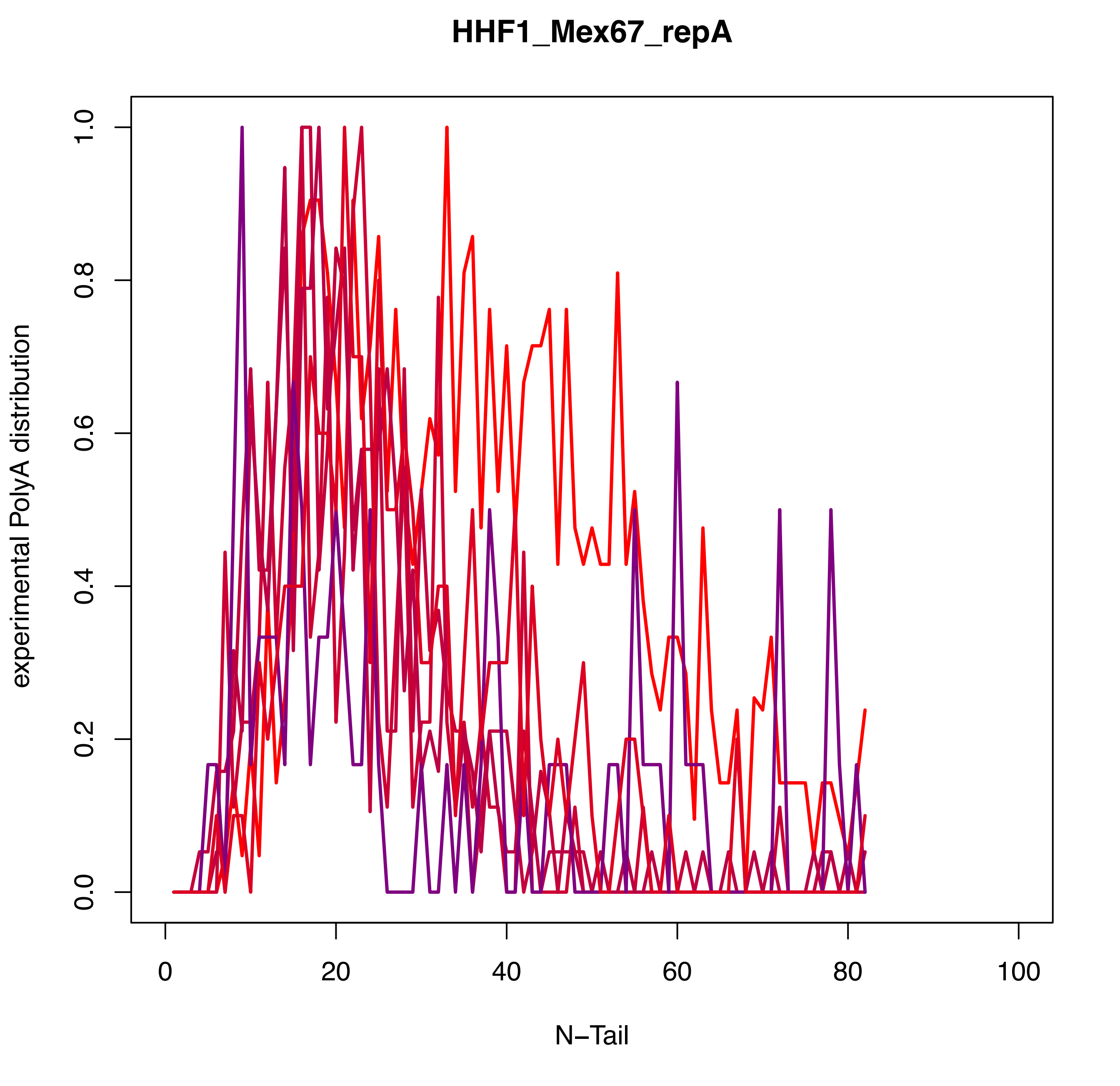
HHF1_Mex67_repA
experimental PolyA distribution
N−Tail
0.0
0.2
0.4
0.6
0.8
1.0
0
20
40
60
80
100

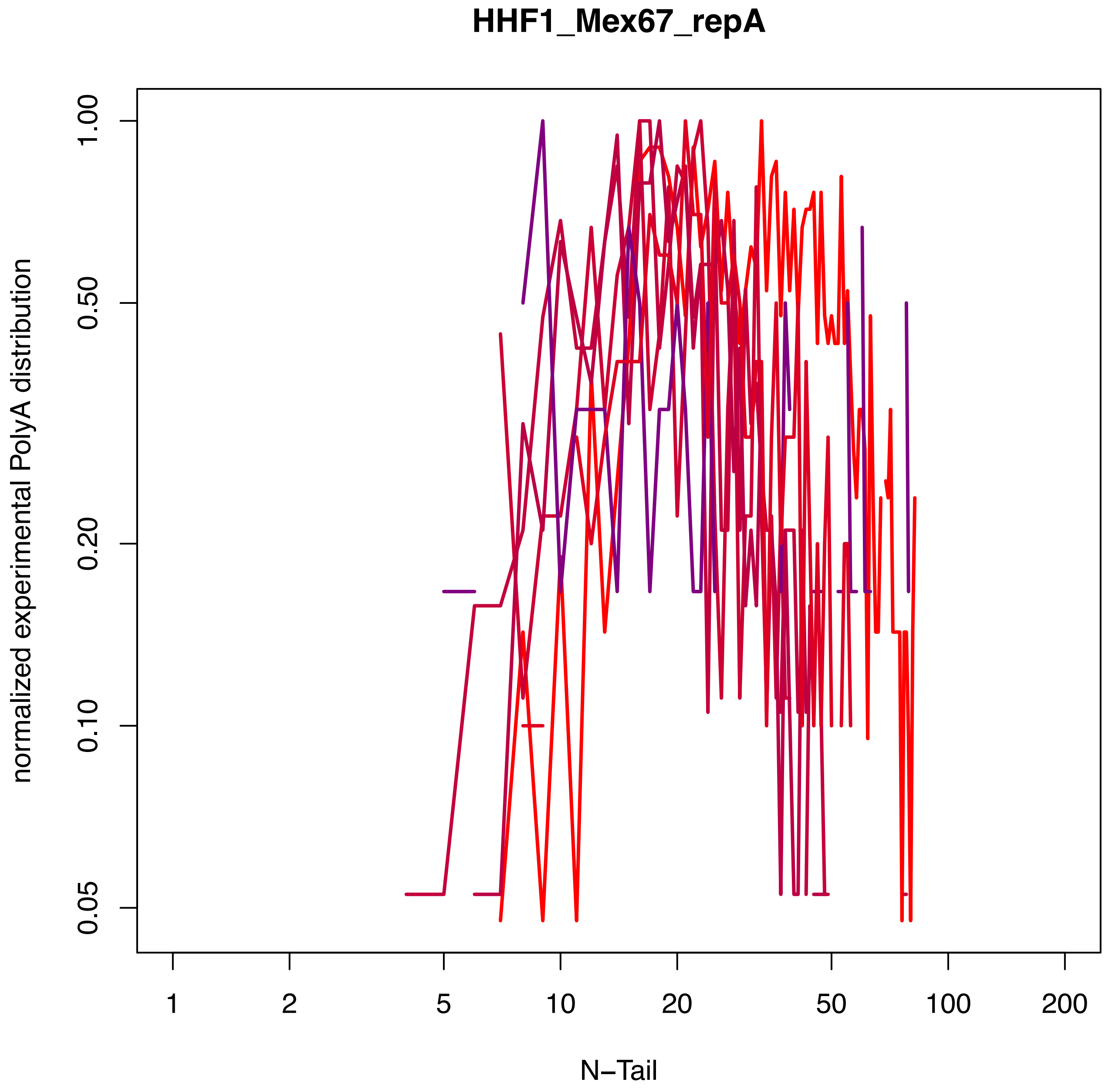

HHF1_Mex67_repA
normalized experimental PolyA distribution
N−Tail
1.00
0.50
0.20
0.10
0.05
1
2
5
10
20
50
100
200

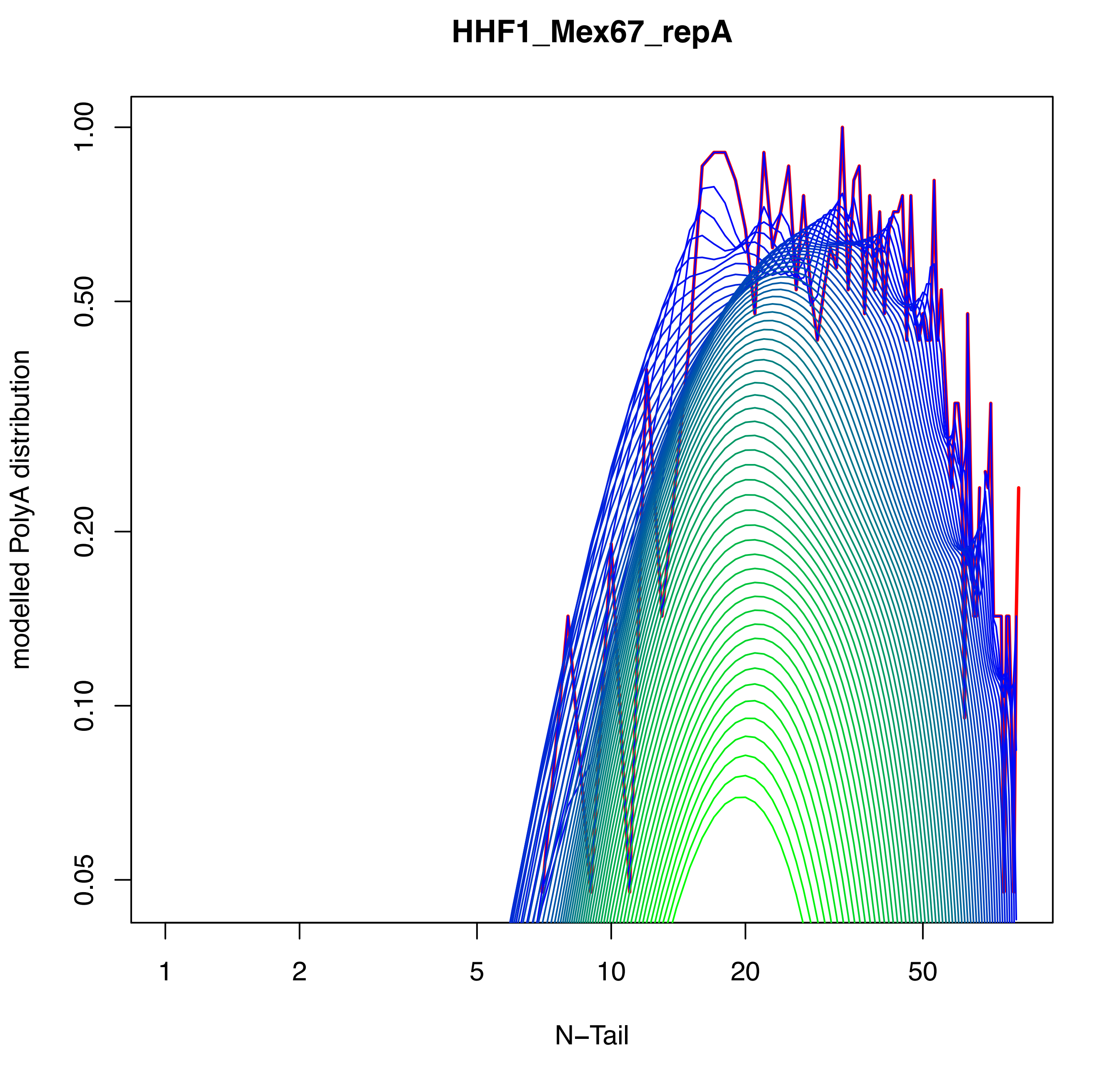

HHF1_Mex67_repA
modelled PolyA distribution
N−Tail
1.00
0.50
0.20
0.10
0.05
1
2
5
10
20
50

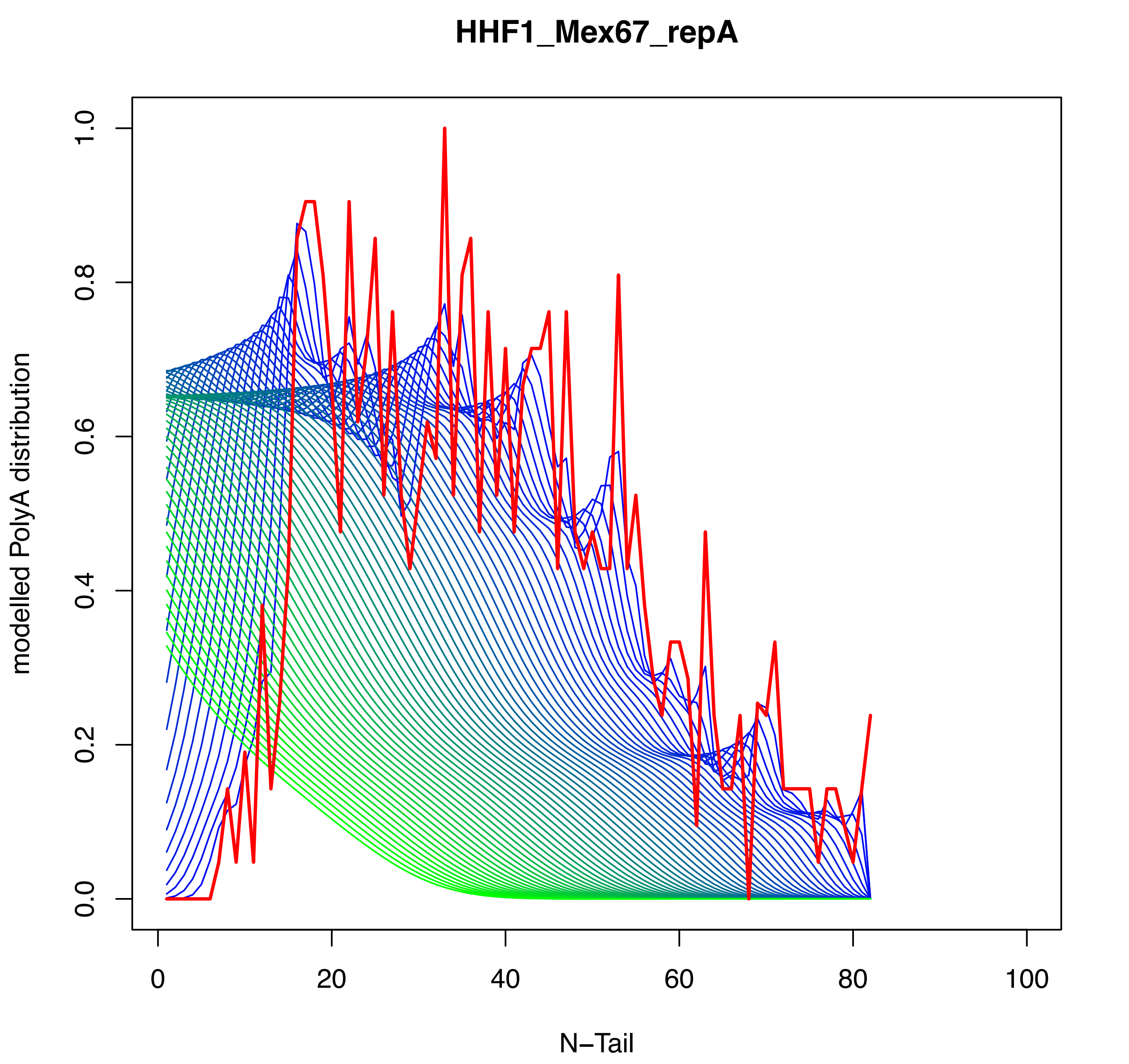
HHF1_Mex67_repA
modelled PolyA distribution
N−Tail
0.0
0.2
0.4
0.6
0.8
1.0
0
20
40
60
80
100

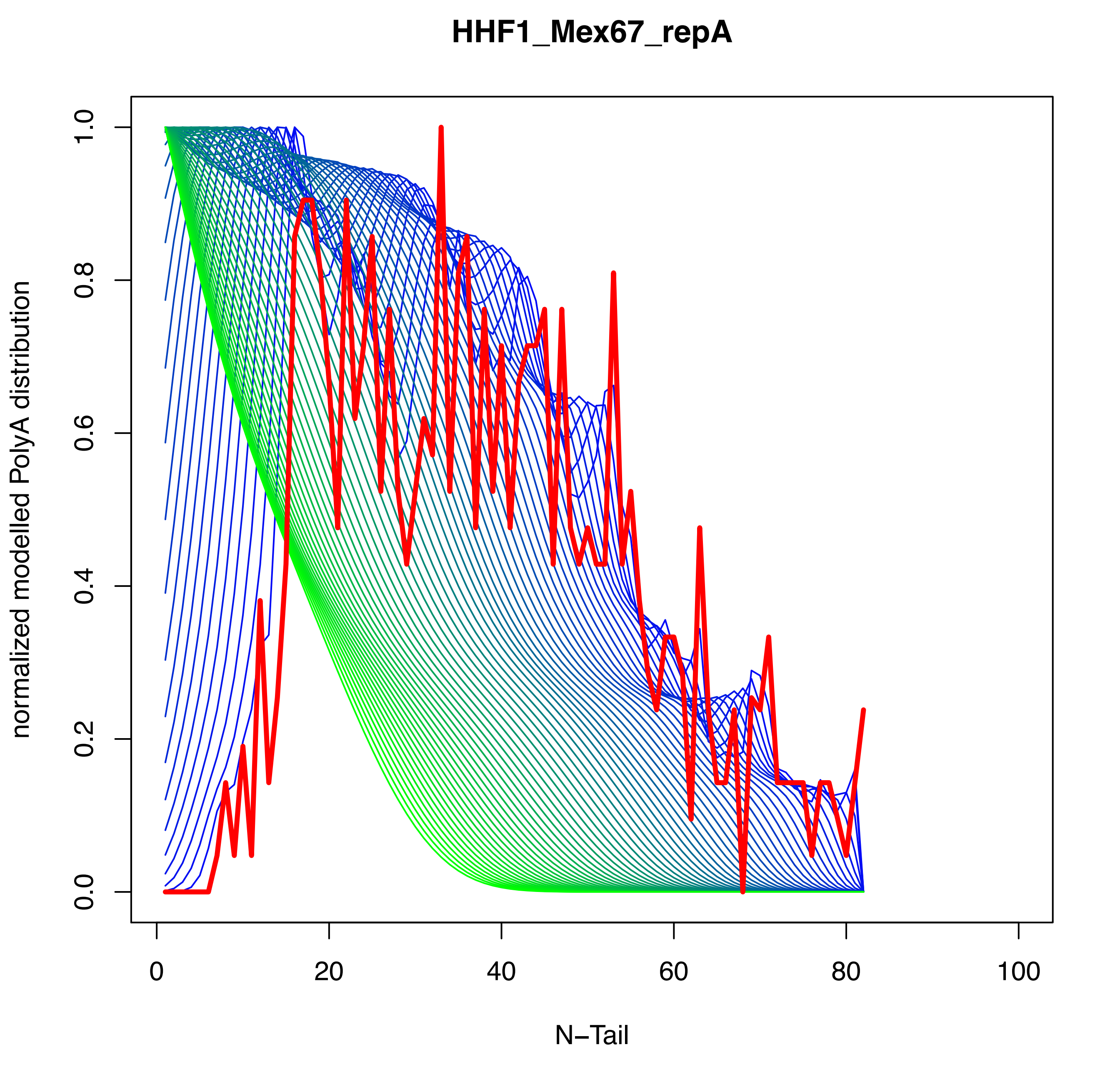

HHF1_Mex67_repA
normalized modelled PolyA distribution
N−Tail
0.0
0.2
0.4
0.6
0.8
1.0
0
20
40
60
80
100

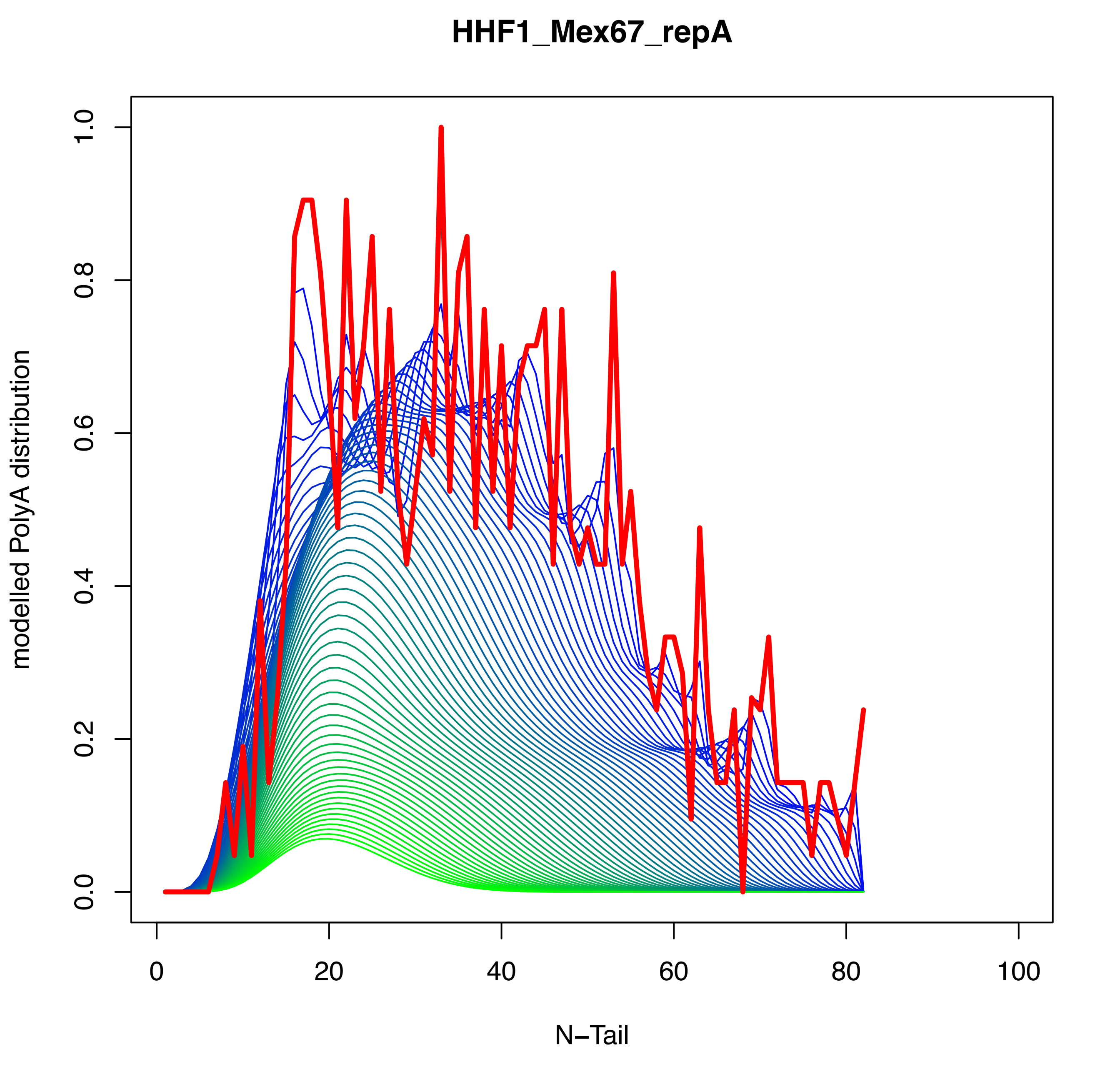

HHF1_Mex67_repA
modelled PolyA distribution
N−Tail
0.0
0.2
0.4
0.6
0.8
1.0
0
20
40
60
80
100

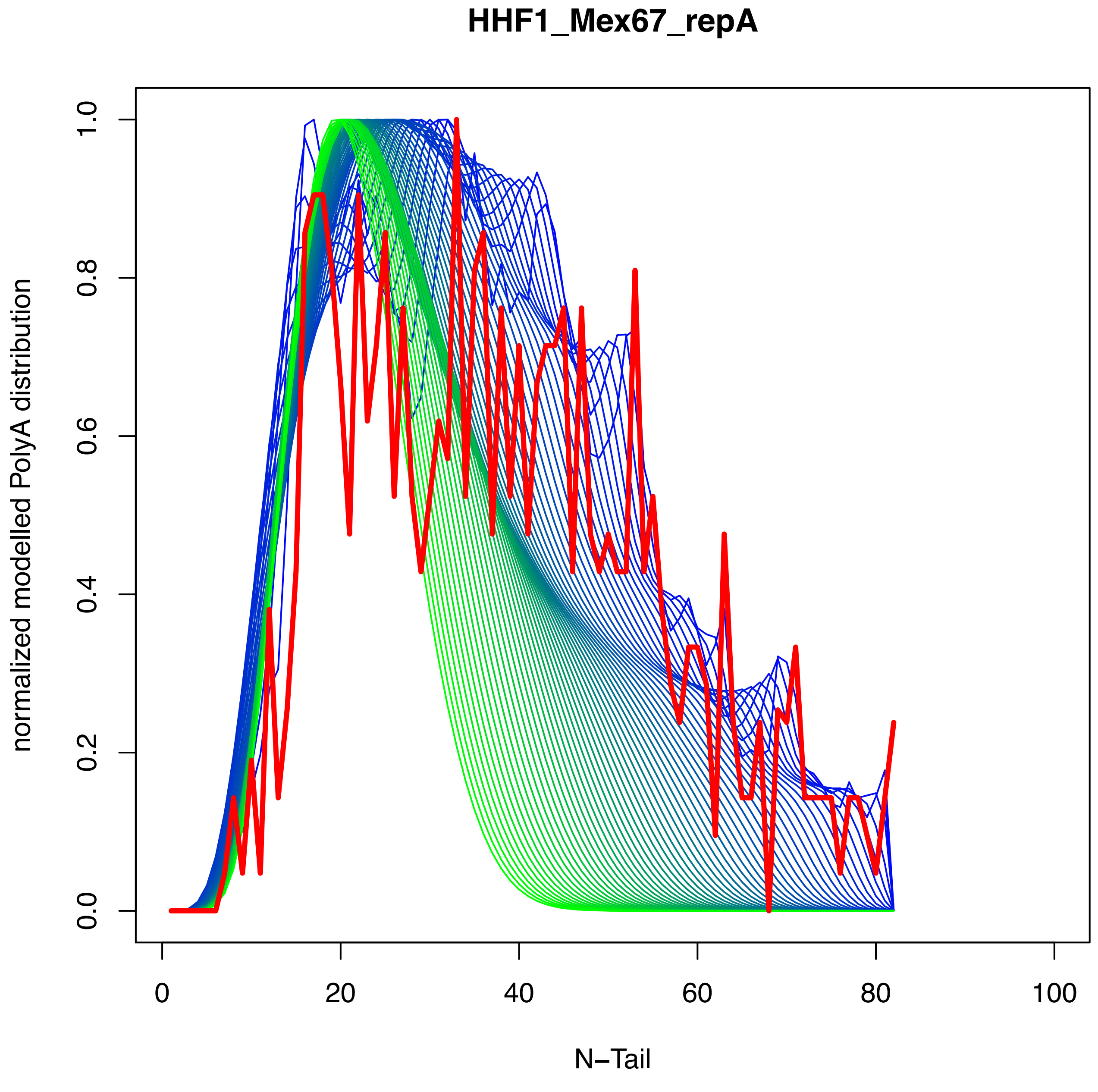
HHF1_Mex67_repA
normalized modelled PolyA distribution
N−Tail
0.0
0.2
0.4
0.6
0.8
1.0
0
20
40
60
80
100

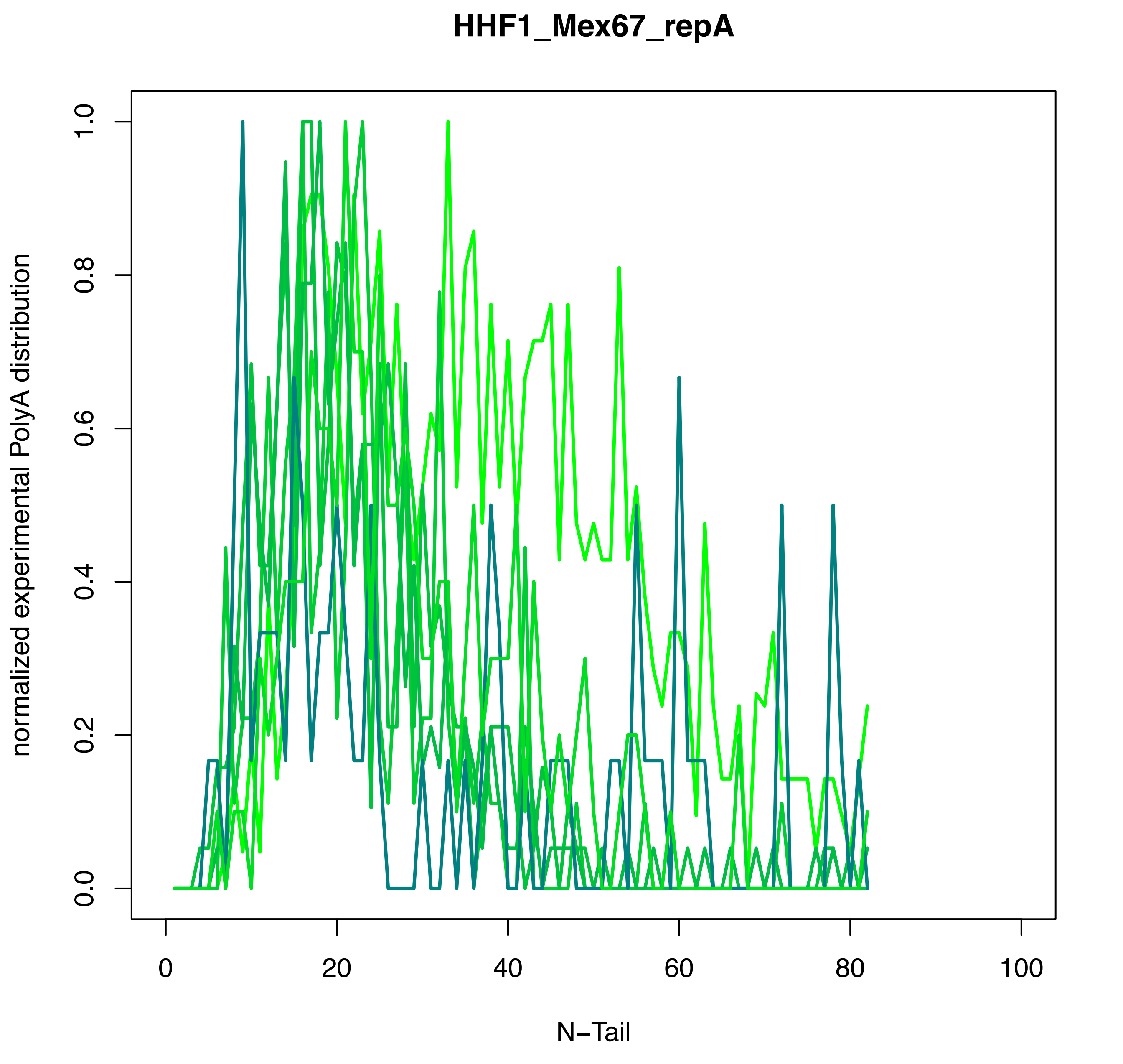
HHF1_Mex67_repA
normalized experimental PolyA distribution
0.0
0.2
0.4
0.6
0.8
1.0
0
20
40
60
80
100
N−Tail

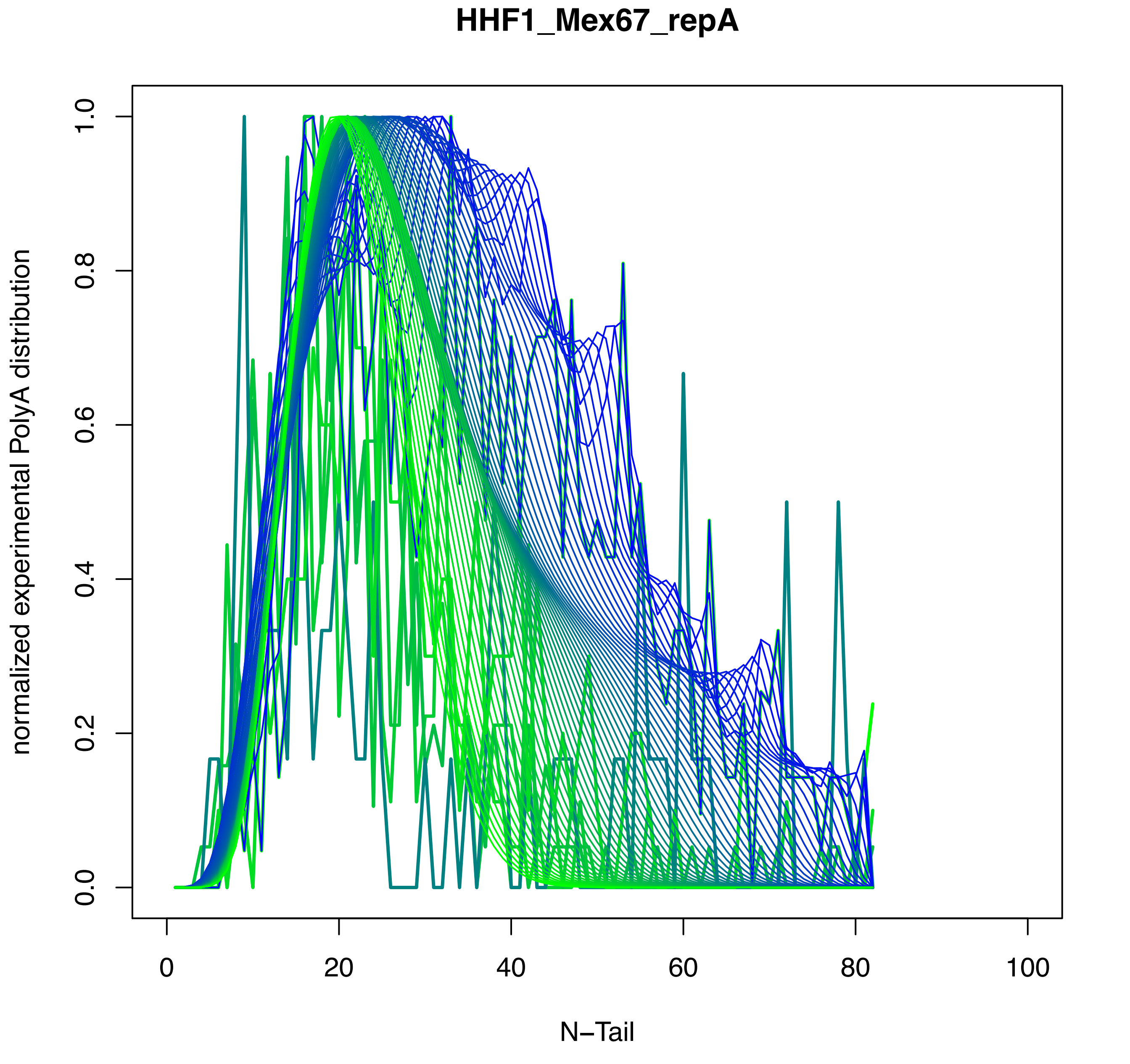
HHF1_Mex67_repA
normalized experimental PolyA distribution
N−Tail
0.0
0.2
0.4
0.6
0.8
1.0
0
20
40
60
80
100

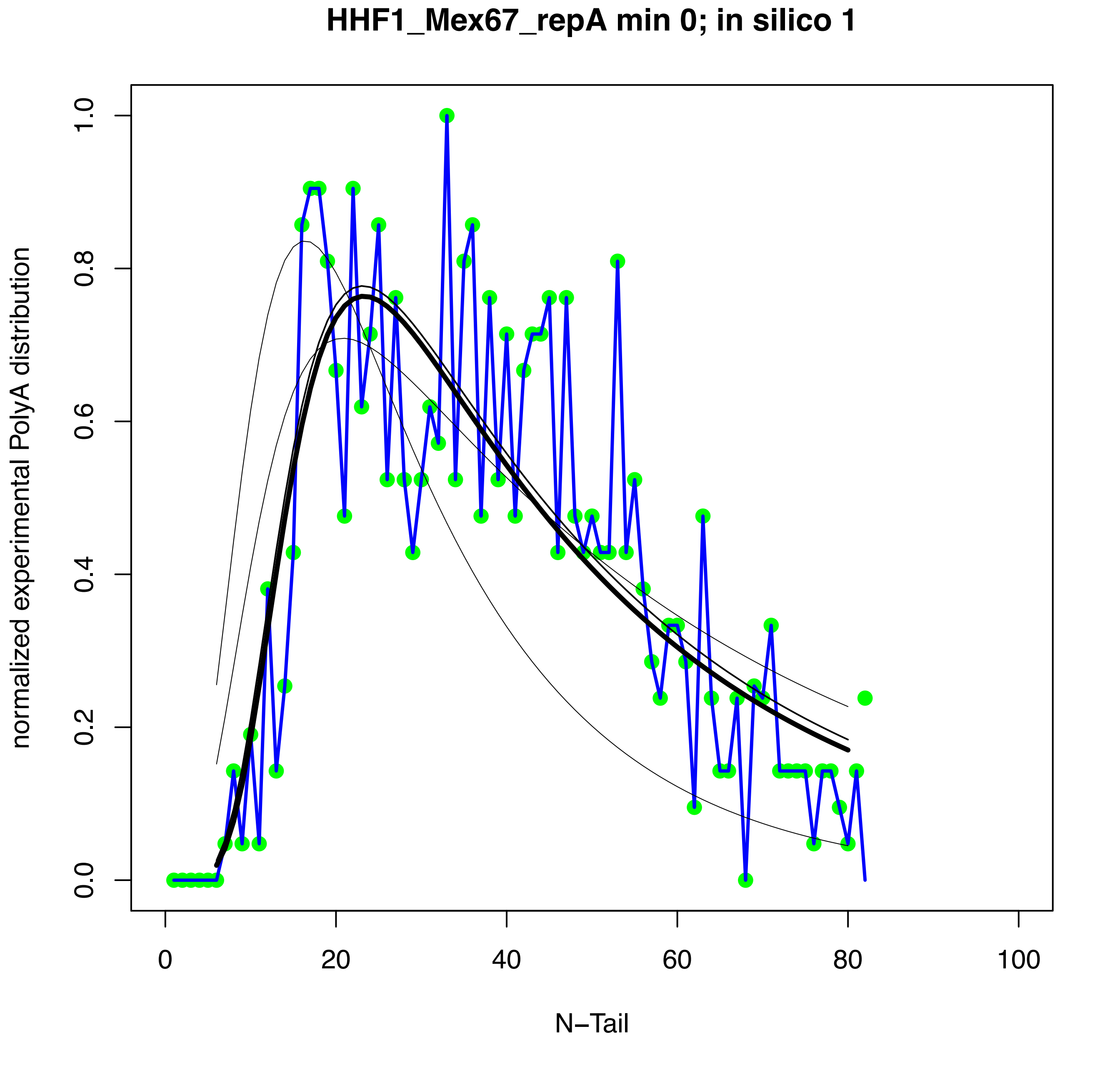

HHF1_Mex67_repA min 0; in silico 1
normalized experimental PolyA distribution
N−Tail
0.0
0.2
0.4
0.6
0.8
1.0
0
20
40
60
80
100

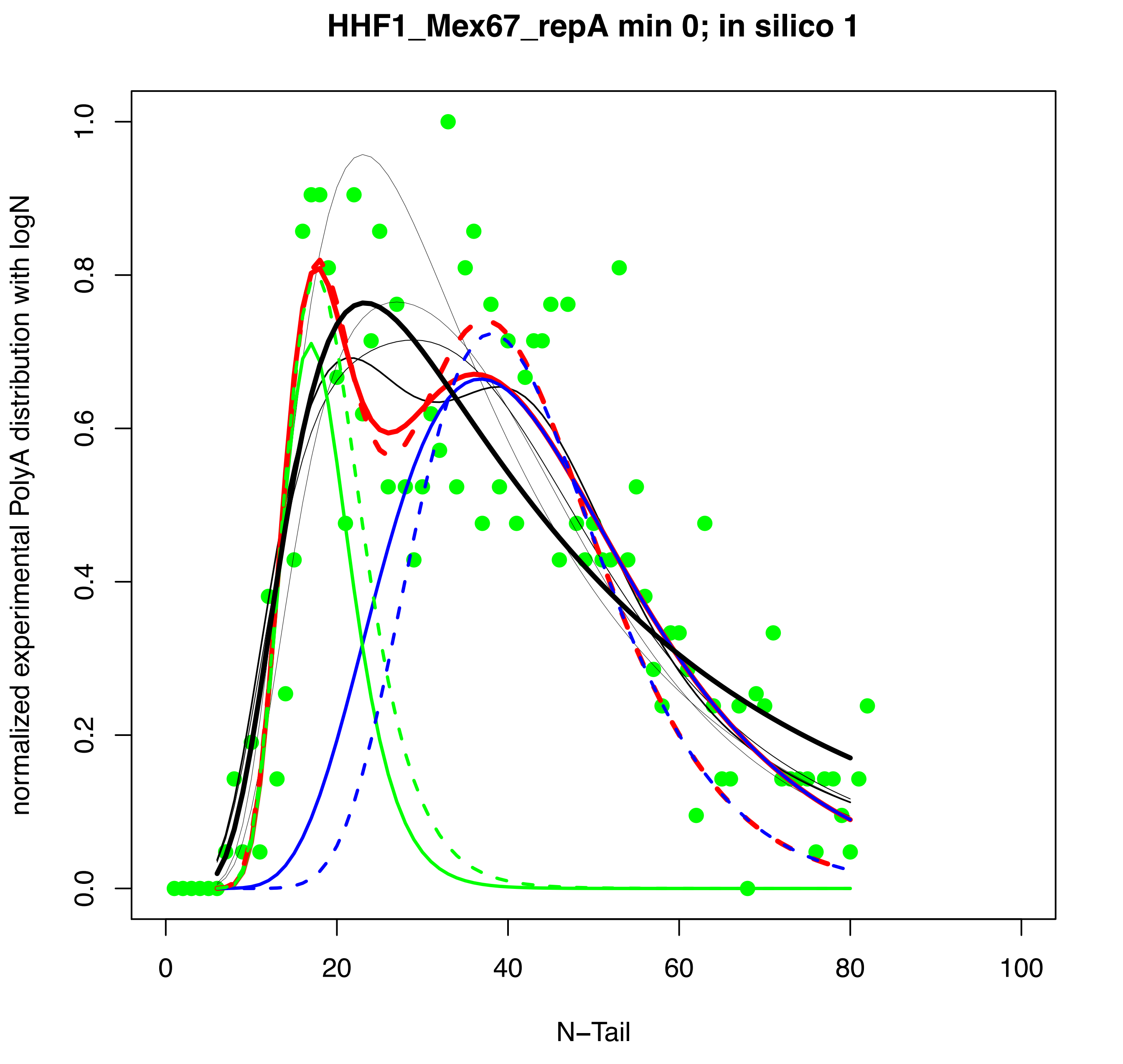

HHF1_Mex67_repA min 0; in silico 1
normalized experimental PolyA distribution with logN
N−Tail
0.0
0.2
0.4
0.6
0.8
1.0
0
20
40
60
80
100

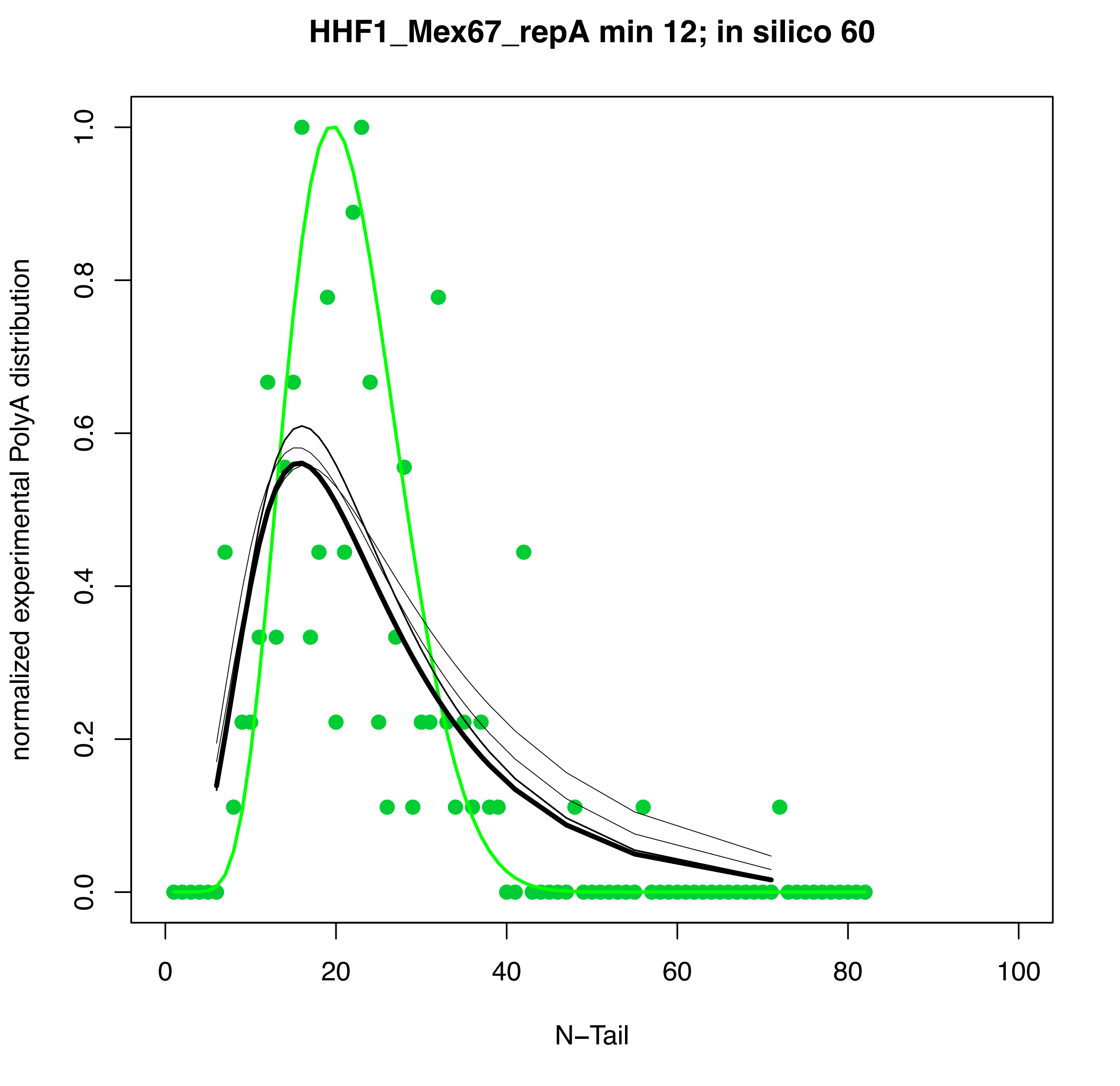
HHF1_Mex67_repA min 12; in silico 60
normalized experimental PolyA distribution
N−Tail
0.0
0.2
0.4
0.6
0.8
1.0
0
20
40
60
80
100

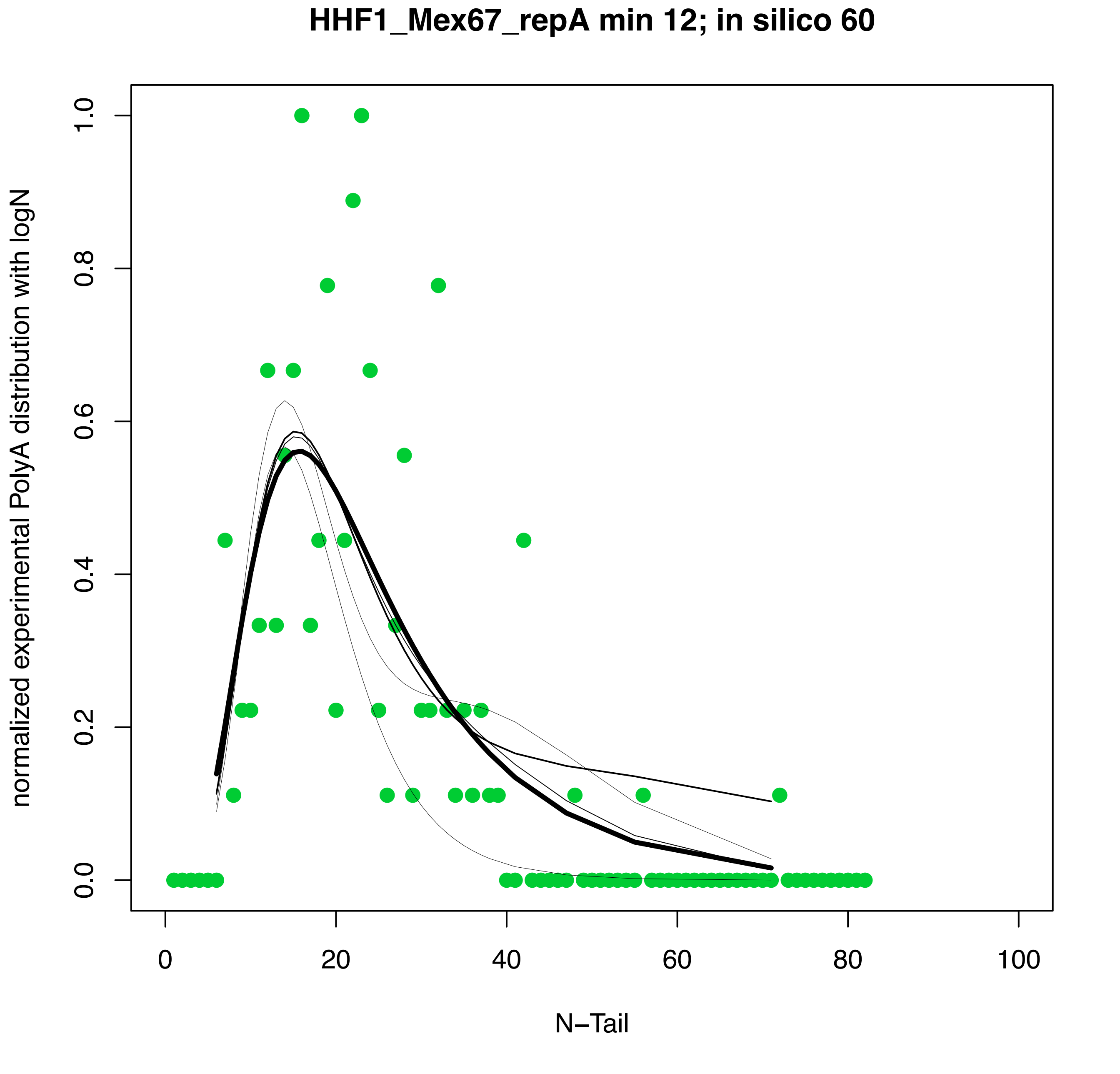

HHF1_Mex67_repA min 12; in silico 60
normalized experimental PolyA distribution with logN
N−Tail
0.0
0.2
0.4
0.6
0.8
1.0
0
20
40
60
80
100

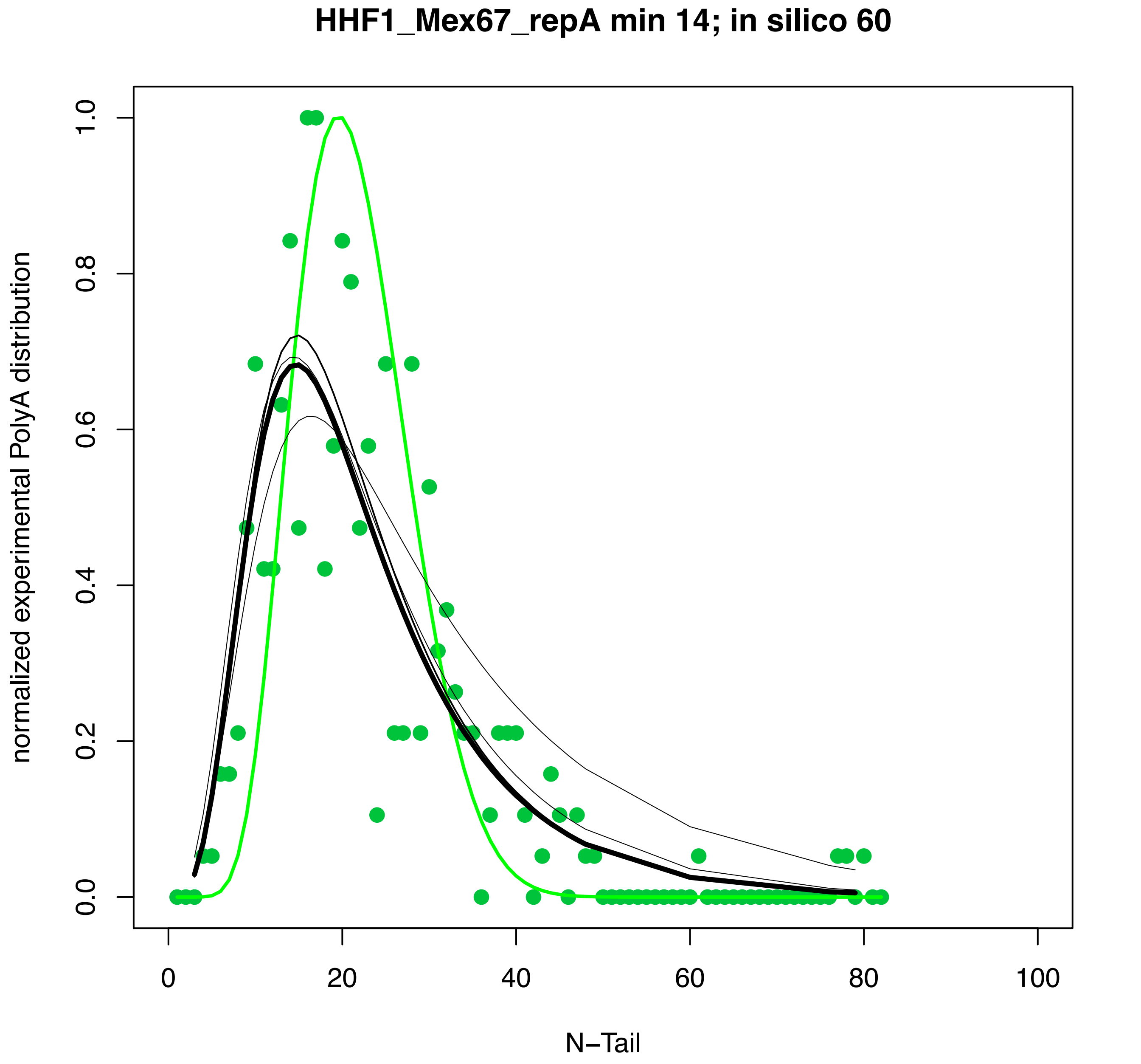

HHF1_Mex67_repA min 14; in silico 60
normalized experimental PolyA distribution
N−Tail
0.0
0.2
0.4
0.6
0.8
1.0
0
20
40
60
80
100

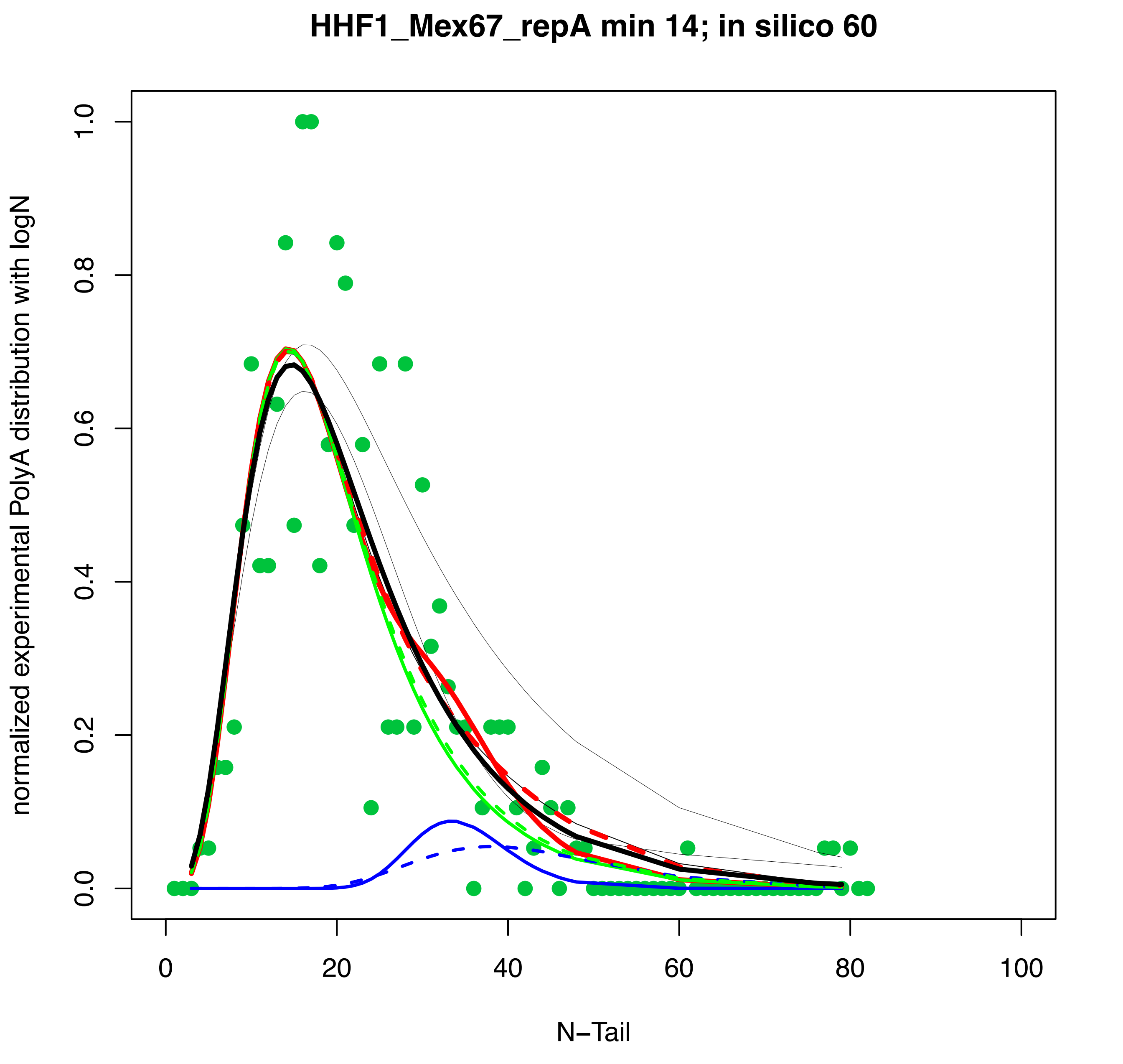

HHF1_Mex67_repA min 14; in silico 60
normalized experimental PolyA distribution with logN
N−Tail
0.0
0.2
0.4
0.6
0.8
1.0
0
20
40
60
80
100

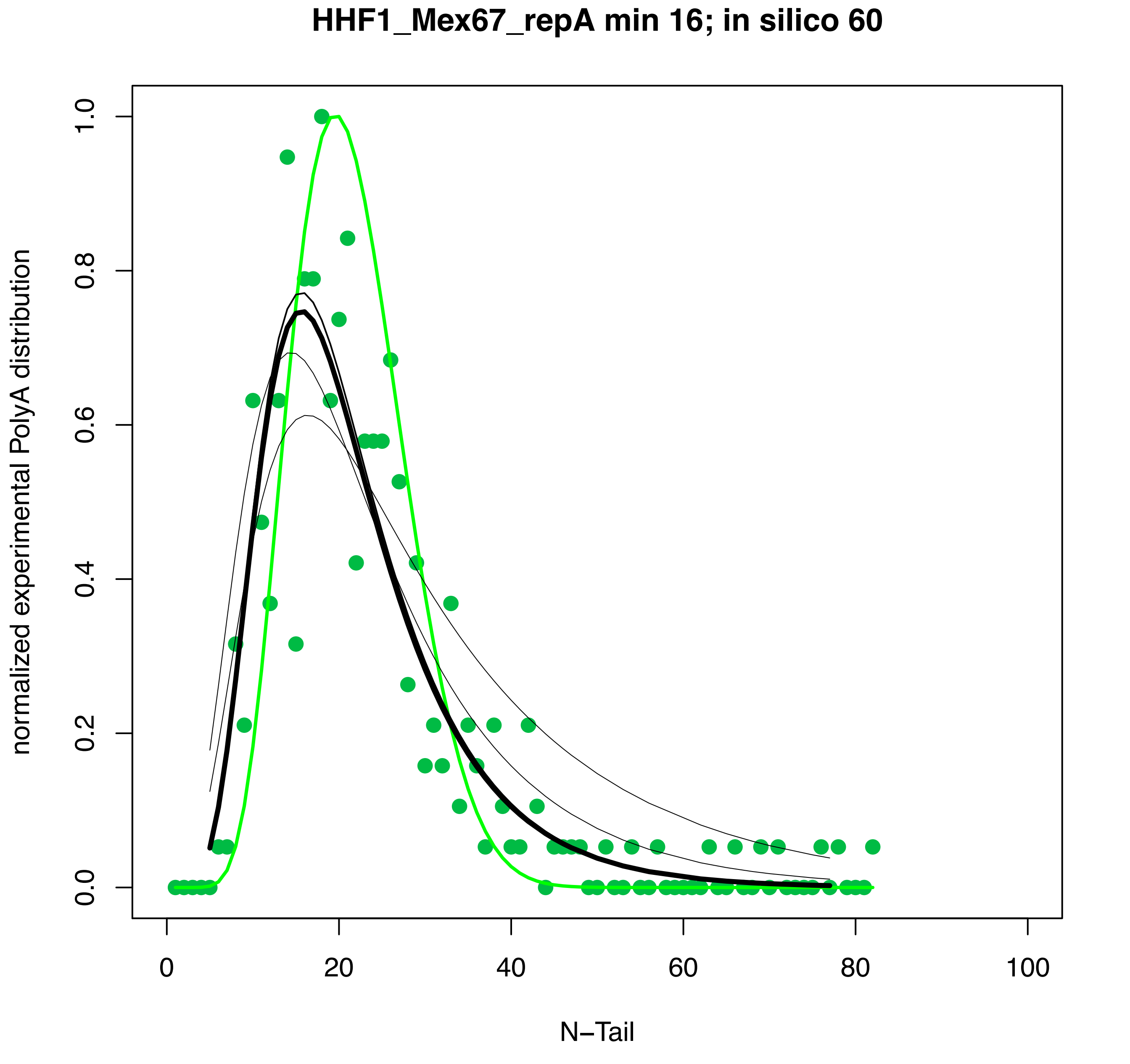

HHF1_Mex67_repA min 16; in silico 60
normalized experimental PolyA distribution
N−Tail
0.0
0.2
0.4
0.6
0.8
1.0
0
20
40
60
80
100

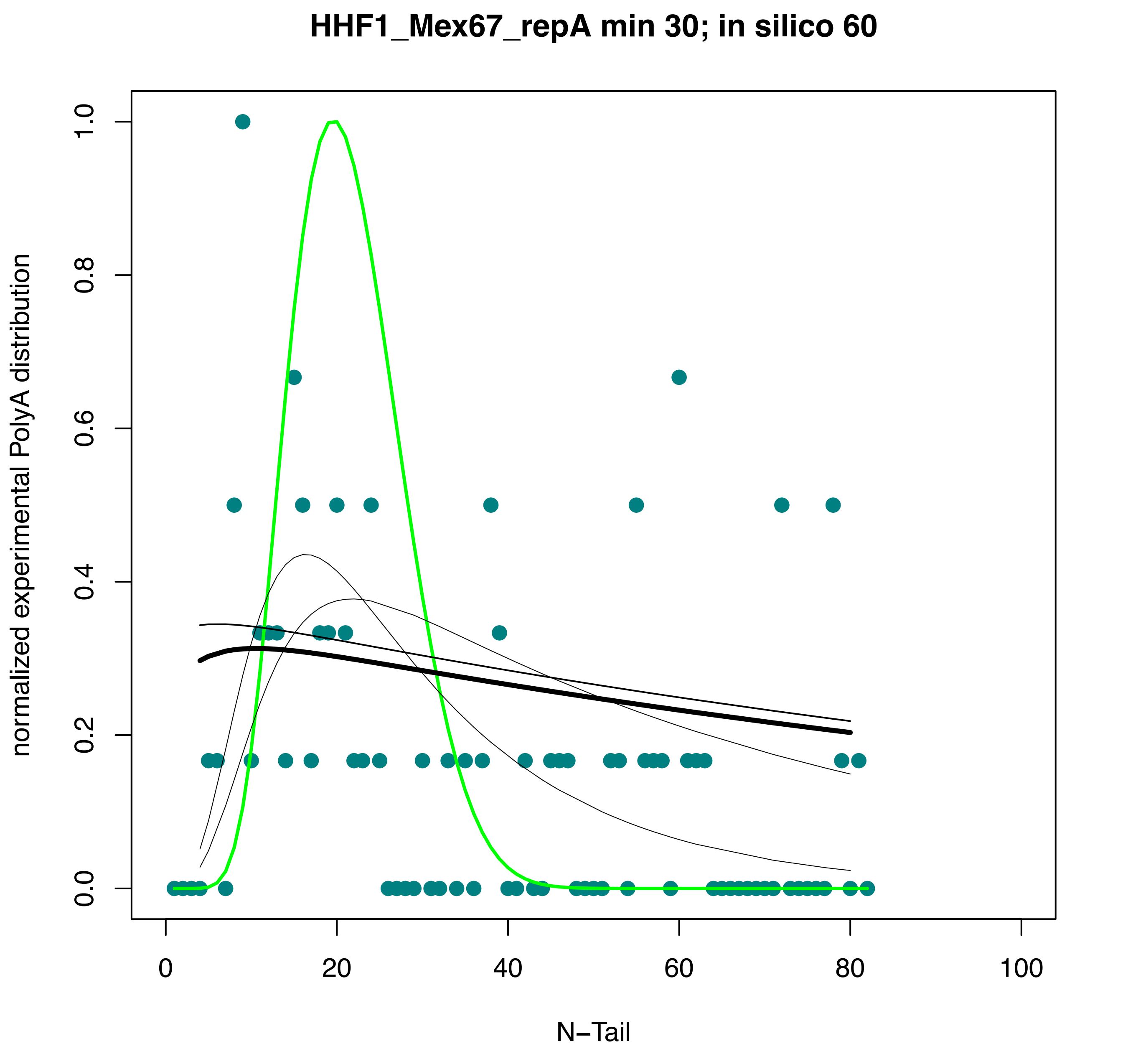
HHF1_Mex67_repA min 30; in silico 60
normalized experimental PolyA distribution
0.0
0.2
0.4
0.6
0.8
1.0
0
20
40
60
80
100
N−Tail

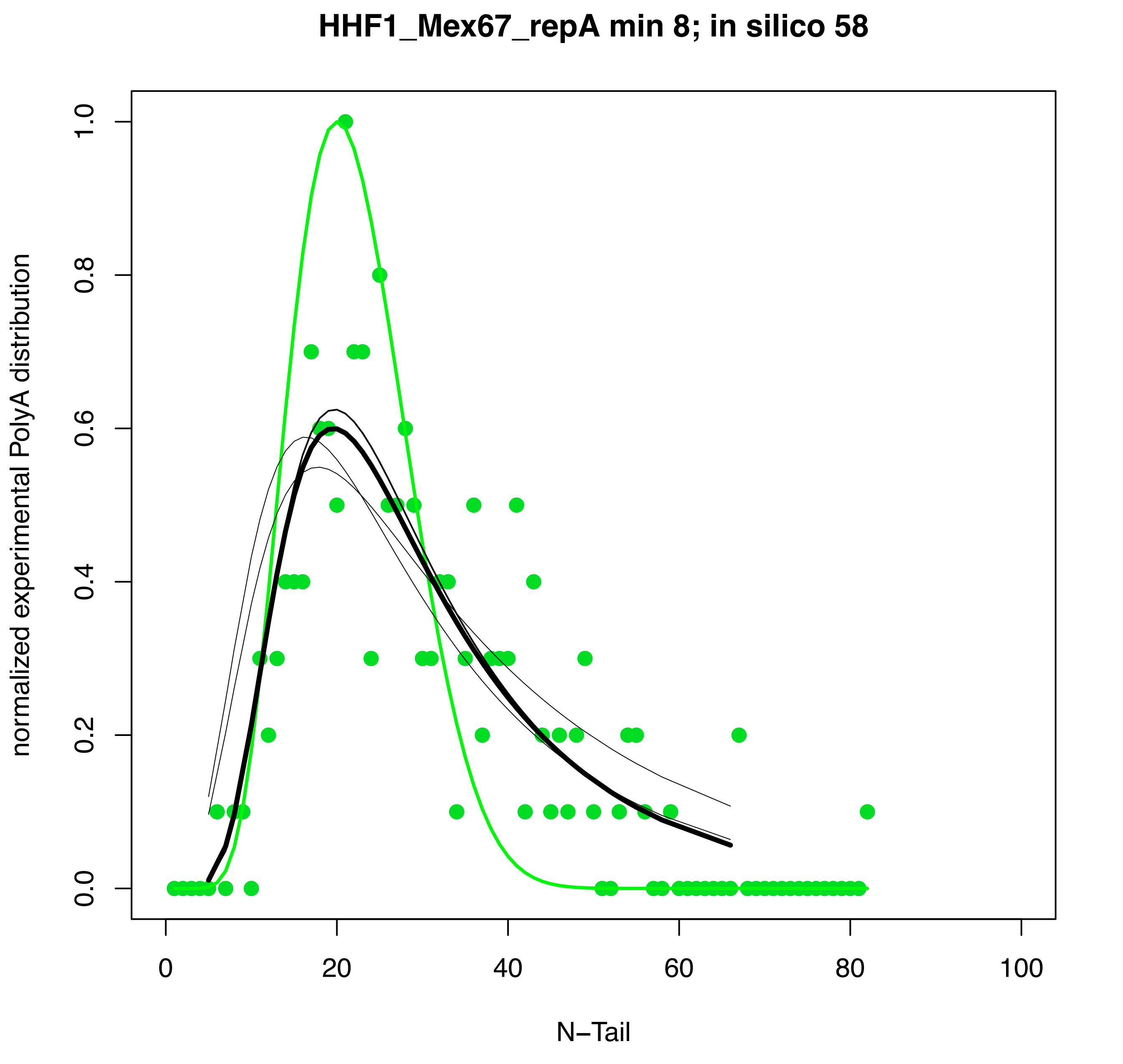
HHF1_Mex67_repA min 8; in silico 58
normalized experimental PolyA distribution
N−Tail
0.0
0.2
0.4
0.6
0.8
1.0
0
20
40
60
80
100

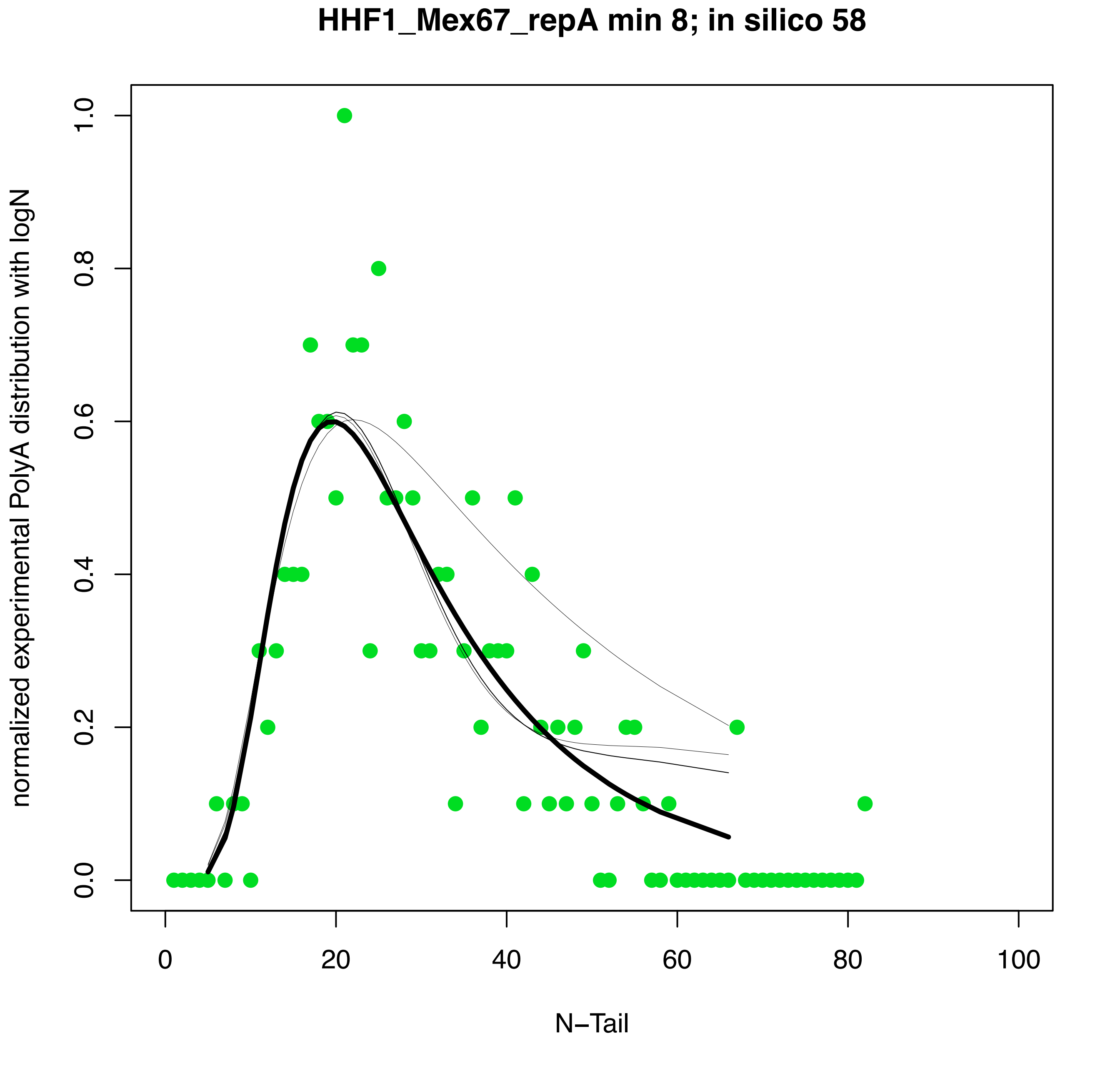

HHF1_Mex67_repA min 8; in silico 58
normalized experimental PolyA distribution with logN
N−Tail
0.0
0.2
0.4
0.6
0.8
1.0
0
20
40
60
80
100

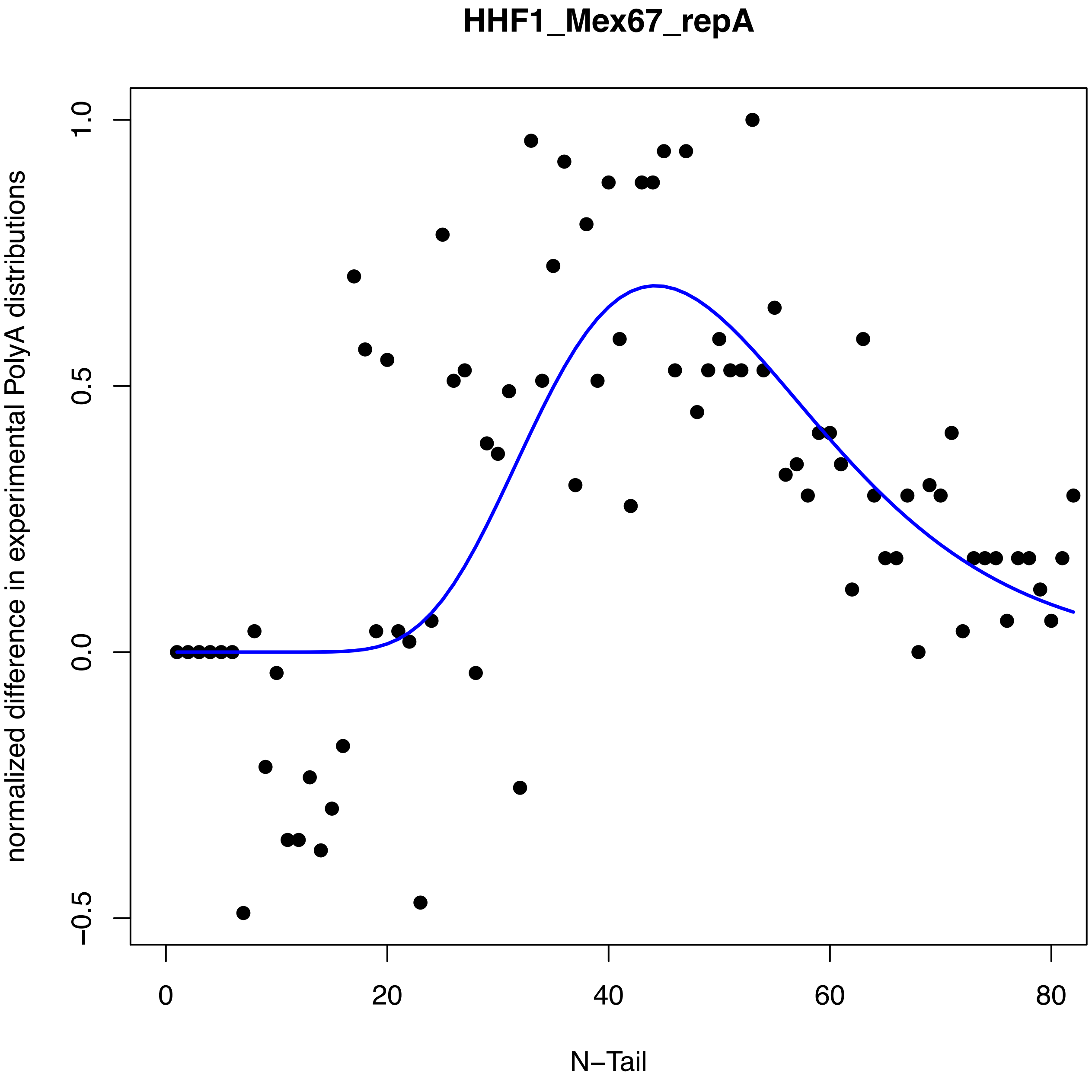
HHF1_Mex67_repA
normalized difference in experimental PolyA distributions
1.0
0.5
0.0
−0.5
0
20
40
60
80
N−Tail
