# Supplementary material for: Modeling of mRNA deadenylation rates reveal a complex relationship between mRNA deadenylation and decay
Source: EMBO J. 2024 Oct 11;43(24):6525–54. doi: 10.1038/s44318-024-00258-3 (PMC11649921; doi:10.1038/s44318-024-00258-3)
Supplement: Supplementary file 10 — Source data Fig. 4 [file 44318_2024_258_MOESM10_ESM.zip › Figure 4/FIGURE 4I/RNA_concetration_chase_.pdf]

## Mex67-AA chase replicate A

| # | Sample ID | Nucleic Acid Conc. | Unit | A260 | A280 | 260/280 | 260/230 |
|---|---|---|---|---|---|---|---|
| 1 | | 0,1 | ng/µl | 0,002 | -0,018 | -0,11 | -0,06 |
| 2 | dTsel A. Mex67-aa 0min | 84,3 | ng/µl | 2,107 | 0,943 | 2,23 | 2,46 |
| 3 | dTsel A. Mex67-aa 0min - bis | 82,8 | ng/µl | 2,069 | 0,926 | 2,23 | 2,47 |
| 4 | dTsel A. Mex67-aa 8min | 83 | ng/µl | 2,076 | 0,934 | 2,22 | 2,37 |
| 5 | dTsel A. Mex67-aa 12min | 83,6 | ng/µl | 2,089 | 0,93 | 2,25 | 2,43 |
| 6 | dTsel A. Mex67-aa 14min | 74,1 | ng/µl | 1,852 | 0,828 | 2,24 | 2,36 |
| 7 | dTsel A. Mex67-aa 16min | 61,7 | ng/µl | 1,542 | 0,684 | 2,25 | 2,27 |
| 8 | dTsel A. Mex67-aa 30min | 47,3 | ng/µl | 1,182 | 0,524 | 2,25 | 2,26 |
| 9 | dTsel A. Mex67-aa -rapa 38C | 45,5 | ng/µl | 1,136 | 0,491 | 2,31 | 2,39 |
| 10 | dTsel A. Mex67-aa +rapa 38C | 48,7 | ng/µl | 1,217 | 0,528 | 2,3 | 2,37 |
| 11 | input A. Mex67-aa 0min | 728 | ng/µl | 18,2 | 8,814 | 2,06 | 2,57 |
| 12 | input A. Mex67-aa 8min | 738,1 | ng/µl | 18,452 | 8,865 | 2,08 | 2,55 |
| 13 | input A. Mex67-aa 12min | 711,5 | ng/µl | 17,787 | 8,523 | 2,09 | 2,58 |
| 14 | input A. Mex67-aa 14min | 708,4 | ng/µl | 17,711 | 8,504 | 2,08 | 2,55 |
| 15 | input A. Mex67-aa 16min | 701,1 | ng/µl | 17,528 | 8,407 | 2,08 | 2,57 |
| 16 | input A. Mex67-aa 30min | 710,5 | ng/µl | 17,764 | 8,5 | 2,09 | 2,58 |
| 17 | input A. Mex67-aa -rapa 38C | 722,1 | ng/µl | 18,051 | 8,886 | 2,03 | 2,53 |
| 18 | input A. Mex67-aa +rapa 38C | 704,8 | ng/µl | 17,619 | 8,356 | 2,11 | 2,56 |
| 19 | blank | 0 | ng/µl | 0 | -0,009 | -0,02 | 0 |

## Mex67-AA chase replicate B

| # | Sample ID | Nucleic Acid Conc. | Unit | A260 | A280 | 260/280 | 260/230 |
|---|---|---|---|---|---|---|---|
| 1 | blank | 0,1 | ng/µl | 0,002 | -0,002 | -0,94 | -0,28 |
| 2 | dT sel Mex67 0min | 103,8 | ng/µl | 2,595 | 1,18 | 2,2 | 2,34 |
| 3 | dT sel Mex67 4min | 101,1 | ng/µl | 2,527 | 1,143 | 2,21 | 2,32 |
| 4 | dT sel Mex67 10min | 69,6 | ng/µl | 1,741 | 0,797 | 2,18 | 2,31 |
| 5 | dT sel Mex67 12min | 72,7 | ng/µl | 1,817 | 0,832 | 2,18 | 2,26 |
| 6 | dT sel Mex67 14min | 85,5 | ng/µl | 2,137 | 0,979 | 2,18 | 2,31 |
| 7 | dT sel Mex67 20min | 69,8 | ng/µl | 1,746 | 0,797 | 2,19 | 2,3 |
| 8 | dT sel Mex67 38C 0min | 62,4 | ng/µl | 1,559 | 0,723 | 2,16 | 2,29 |
| 9 | dT sel Mex67 38C 16min | 45,1 | ng/µl | 1,128 | 0,512 | 2,2 | 2,18 |
| 10 | total Mex67 0min | 773,3 | ng/µl | 19,332 | 9,136 | 2,12 | 2,56 |
| 11 | total Mex67 4min | 769,4 | ng/µl | 19,235 | 9,142 | 2,1 | 2,55 |
| 12 | total Mex67 10min | 709,6 | ng/µl | 17,74 | 8,403 | 2,11 | 2,55 |
| 13 | total Mex67 12min | 733 | ng/µl | 18,324 | 8,614 | 2,13 | 2,59 |
| 14 | total Mex67 14min | 727,2 | ng/µl | 18,181 | 8,592 | 2,12 | 2,56 |
| 15 | total Mex67 20min | 708,9 | ng/µl | 17,723 | 8,432 | 2,1 | 2,54 |
| 16 | total Mex67 38C 0min | 641,1 | ng/µl | 16,027 | 7,622 | 2,1 | 2,51 |
| 17 | total Mex67 38C 16min | 676,3 | ng/µl | 16,907 | 7,959 | 2,12 | 2,52 |
| 18 | blank | -0,2 | ng/µl | -0,006 | -0,002 | 2,3 | 0,33 |

## Mex67-AA chase replicate H

| # | Sample ID | Nucleic Acid Conc. | Unit | A260 | A280 | 260/280 | 260/230 |
|---|---|---|---|---|---|---|---|
| 1 | dTsel mex67 0 | 75,4 | ng/µl | 1,886 | 0,827 | 2,28 | 2,23 |

| | | | | | | | |
|---|---|---|---|---|---|---|---|
| 2 | dTsel mex67 4 | 62 | ng/µl | 1,551 | 0,687 | 2,26 | 2,07 |
| 3 | dTsel mex67 8 | 53 | ng/µl | 1,325 | 0,583 | 2,27 | 2,16 |
| 4 | dTsel mex67 16 | 41,7 | ng/µl | 1,043 | 0,465 | 2,25 | 1,78 |
| 5 | dTsel mex67 30 | 31,3 | ng/µl | 0,782 | 0,344 | 2,27 | 1,78 |
| 6 | dTsel mex67 60 | 27,1 | ng/µl | 0,678 | 0,306 | 2,21 | 1,72 |
| 7 | total mex67aa 0 | 719,1 | ng/µl | 17,978 | 8,826 | 2,04 | 2,59 |
| 8 | total mex67aa 8 | 716,7 | ng/µl | 17,916 | 8,796 | 2,04 | 2,57 |
| 9 | total mex67aa 16 | 748,9 | ng/µl | 18,721 | 9,207 | 2,03 | 2,55 |
| 10 | total mex67aa 30 | 753,4 | ng/µl | 18,835 | 9,194 | 2,05 | 2,59 |
| 11 | total mex67aa 60 | 744,3 | ng/µl | 18,608 | 9,086 | 2,05 | 2,53 |
