# Supplementary material for: Modeling of mRNA deadenylation rates reveal a complex relationship between mRNA deadenylation and decay
Source: EMBO J. 2024 Oct 11;43(24):6525–54. doi: 10.1038/s44318-024-00258-3 (PMC11649921; doi:10.1038/s44318-024-00258-3)
Supplement: Supplementary file 16 — Expanded View Figures [file 44318_2024_258_MOESM16_ESM.pdf]

# Expanded View Figures

**Figure EV1. Following nuclear decay of pre-mRNAs in export-block conditions the cytoplasmic deadenylation and decay of mRNAs is revealed.**

(**A**) Time-dependent change in mRNA abundance relative to control samples in Mex67-depleted cells shown in the form of a series of boxplots. The boxplot central line is the median. The box edges are the 25th and 75th percentiles. Whiskers extend to 1.5 times the IQR (Inter Quartile Range). All three replicates were combined into time point ranges. For each time-slot the number of transcripts assessed in each replicate is given (3875, 3989 and 3032). Sum of 10896 mRNAs is assessed in each time-slot. Note that those are largely overlapping). (**B**) Time-dependent abundance of selected mRNAs in DRS datasets from Mex67-depleted cells. Fitted lines demonstrate the decay factor calculated for each mRNA based on DRS data. (**C**) Scatterplot comparing half-life times in Mex67-depleted DRS datasets to those reported by Chan et al (2018). (**D**) Scatterplot comparing half-life times reported by Miller et al (2011) to those by Chan et al (2018). (**E**) Global distributions of pA-tail lengths of total mRNA in three replicates of Mex67-AA chase experiments used to model deadenylation. The number of transcripts (pA-tail estimates) in each density plot is given on the panel. (**F**) Scatterplot comparing single mRNA log2 abundance to half-life. Abundant mRNAs and RPG mRNAs are highlighted with gold and blue dots. (**G**) Scatterplot comparing mean pA-tail length to mRNA half-life. (**H**) Global distribution of pA-tail lengths of highly abundant mRNAs isolated from control samples and at various times of Mex67 depletion, presented as a density plot (left panel) and violin plot (right panel). Replicates were merged. The number of transcripts (pA-tail estimates) in each density plot is given on the panel. (**I**) Global distribution of pA-tail length of low-abundance mRNAs. Replicates were merged. (**J**) Global pA-tail distribution of RPG and non-RPG mRNAs. Replicates were merged. The number of transcripts (pA-tail estimates) in each density plot is given on the panel. (**K**) Global pA-tail distribution of individual *RPL36A*, *GAS1*, *RPL4A*, and *HHF1* mRNAs. Stars mark long pA-tailed mRNAs accumulating in later Mex67-depletion time points, indicative of hyperadenylation (Jensen et al, 2001). The number of transcripts (pA-tail estimates) in each density plot is given on the panel.

▶

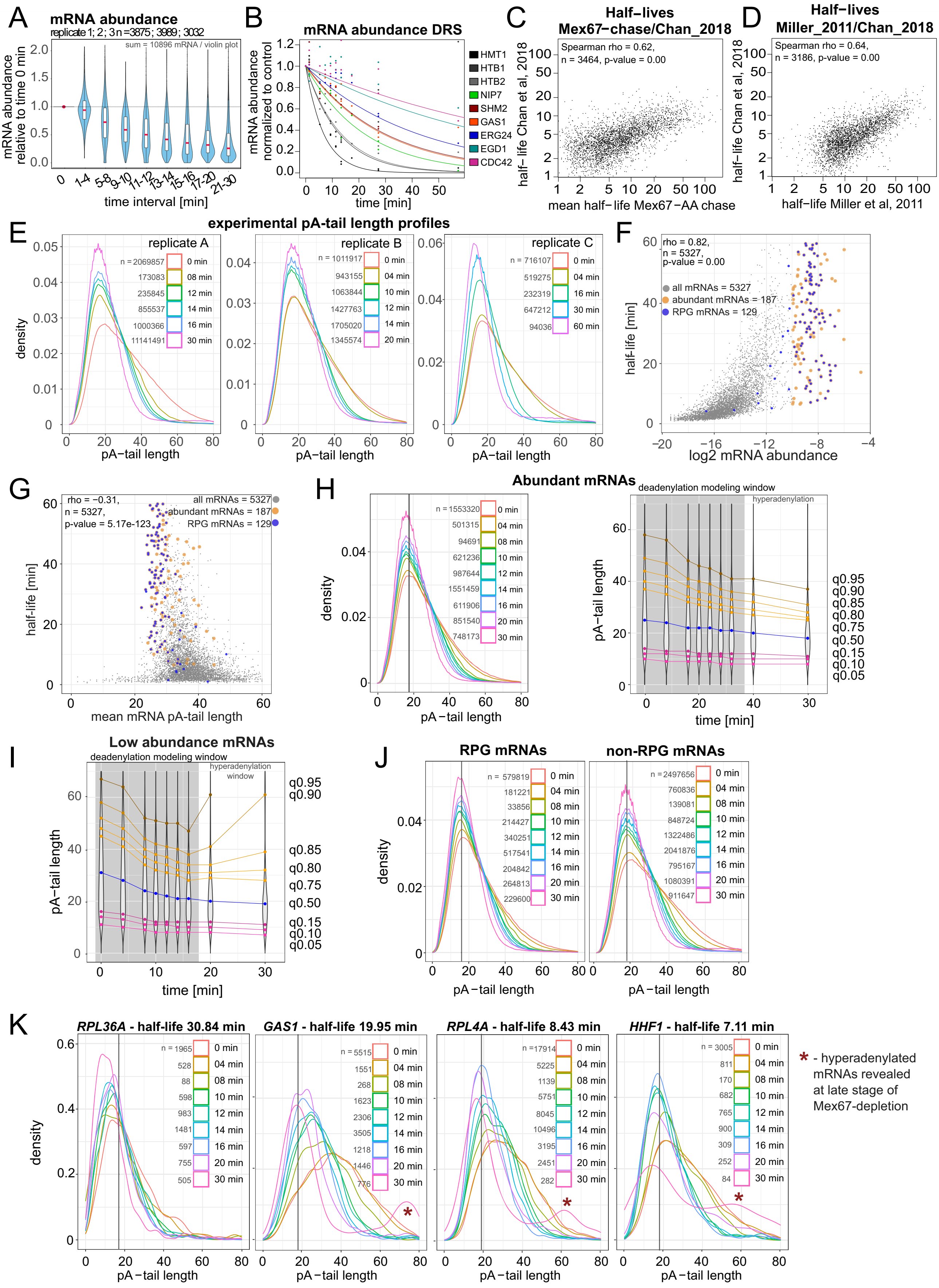
A
mRNA abundance
replicate 1; 2; 3 n = 3875; 3989; 3032
sum = 10896 mRNA / violin plot
mRNA abundance relative to time 0 min
time interval [min]
B
mRNA abundance DRS
mRNA abundance normalized to control
time [min]
HMT1
HTB1
HTB2
NIP7
SHM2
GAS1
ERG24
EGD1
CDC42
C
Half−lives Mex67−chase/Chan_2018
Spearman rho = 0.62, n = 3464, p-value = 0.00
half−life Chan et al, 2018
mean half−life Mex67−AA chase
D
Half−lives Miller_2011/Chan_2018
Spearman rho = 0.64, n = 3186, p-value = 0.00
half−life Chan et al, 2018
half−life Miller et al, 2011
E
experimental pA-tail length profiles
replicate A
replicate B
replicate C
density
pA−tail length
F
rho = 0.82, n = 5327, p-value = 0.00
all mRNAs = 5327
abundant mRNAs = 187
RPG mRNAs = 129
half-life [min]
log2 mRNA abundance
G
rho = −0.31, n = 5327, p-value = 5.17e-123
mean mRNA pA-tail length
H
Abundant mRNAs
deadenylation modeling window
hyperadenylation
pA−tail length
time [min]
I
Low abundance mRNAs
deadenylation modeling window
hyperadenylation window
J
RPG mRNAs
non-RPG mRNAs
K
RPL36A - half-life 30.84 min
GAS1 - half-life 19.95 min
RPL4A - half-life 8.43 min
HHF1 - half-life 7.11 min
* - hyperadenylated mRNAs revealed at late stage of Mex67-depletion

 © The Author(s)

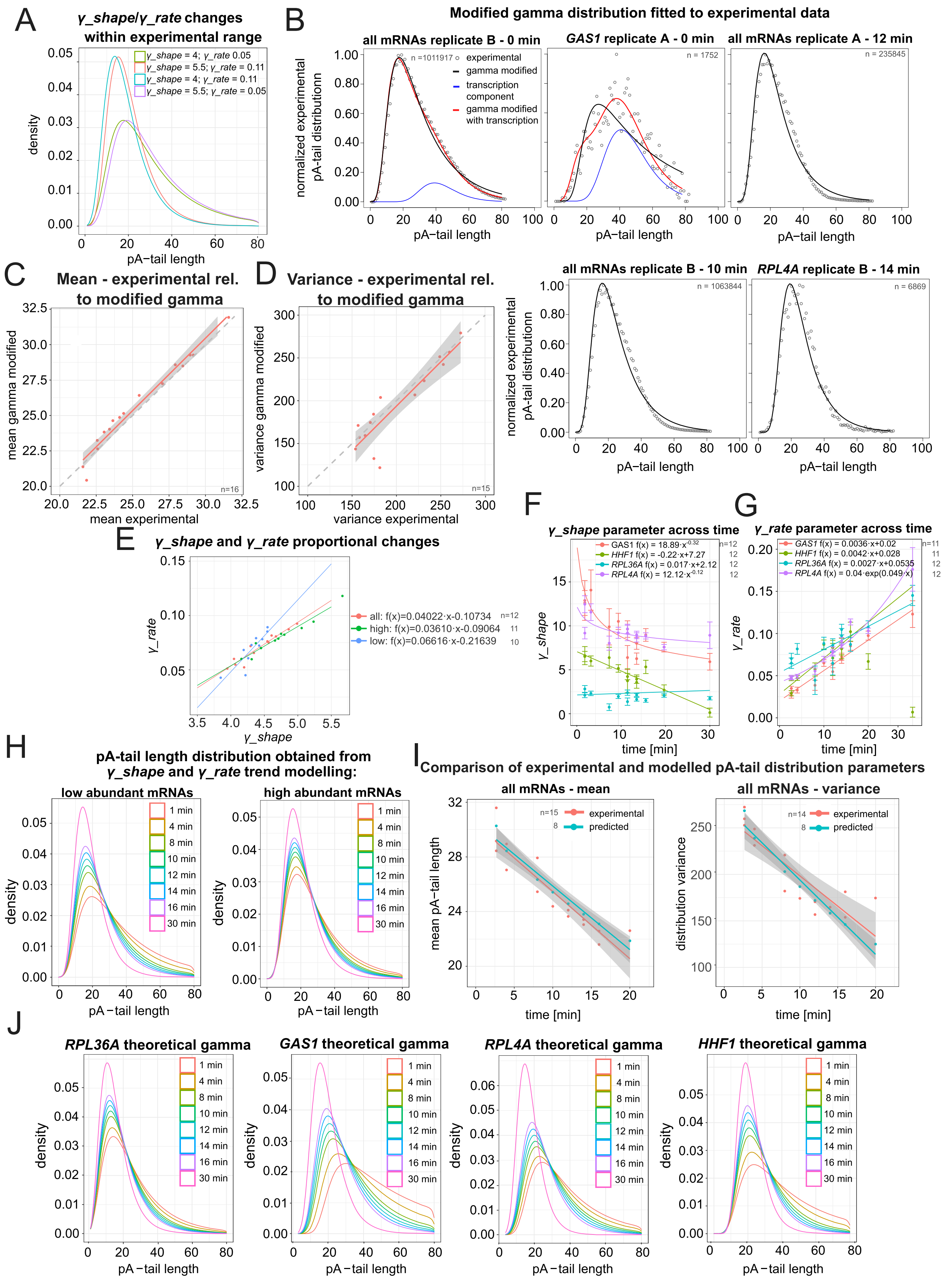
A
γ_shape/γ_rate changes within experimental range
B
Modified gamma distribution fitted to experimental data
all mRNAs replicate B - 0 min
GAS1 replicate A - 0 min
all mRNAs replicate A - 12 min
experimental
gamma modified
transcription component
gamma modified with transcription
normalized experimental pA-tail distribution
pA−tail length
C
Mean - experimental rel. to modified gamma
mean gamma modified
mean experimental
D
Variance - experimental rel. to modified gamma
variance gamma modified
variance experimental
all mRNAs replicate B - 10 min
RPL4A replicate B - 14 min
E
γ_shape and γ_rate proportional changes
all: f(x)=0.04022·x-0.10734
high: f(x)=0.03610·x-0.09064
low: f(x)=0.06616·x-0.21639
F
γ_shape parameter across time
G
γ_rate parameter across time
time [min]
H
pA-tail length distribution obtained from γ_shape and γ_rate trend modelling:
low abundant mRNAs
high abundant mRNAs
I
Comparison of experimental and modelled pA-tail distribution parameters
all mRNAs - mean
all mRNAs - variance
mean pA-tail length
distribution variance
experimental
predicted
J
RPL36A theoretical gamma
GAS1 theoretical gamma
RPL4A theoretical gamma
HHF1 theoretical gamma
density
pA−tail length

◀ **Figure EV2. A modified gamma distribution accurately describes experimental yeast pA-tail distributions.**

(**A**) Plot displaying modified gamma distributions for *γ_shape* and *γ_rate* parameters within experimental values. The green line represents a function that effectively describes the pA-tail distribution of the entire transcriptome in control cells (*γ_shape* = 4; *γ_rate* = 0.05). (**B**) Fitting of the modified gamma distribution to experimental datasets. Figure complements examples shown in Fig. 4C. (**C**) Comparison of the experimental whole-transcriptome mean pA-tail length to the mean pA-tail length of the fitted modified gamma model. A regression line with a 95% confidence interval was fitted to the data points for comparison with the diagonal. The modified gamma distribution slightly overestimates the mean due to discrepancies in estimating very long pA-tails, as seen in Fig. EV2B for pA-tails of 50 and greater. (**D**) Graph comparing the experimental whole-transcriptome pA-tail length variance length to the variance of the fitted modified gamma model. A regression line with a 95% confidence interval was fitted. (**E**) Graph comparing changes in the *γ_shape* and *γ_rate* parameters of the modified gamma distribution fitted to Mex67-depletion chase time points for all mRNAs and those of high and low abundance. The graph complements plots shown in Fig. 4D, E. (**F**, **G**) Graphs showing time-dependent changes in the value of *γ_shape* (**F**) and *γ_rate* (**G**) parameters of the modified gamma probability distribution fitted into experimental data for selected mRNAs: *GAS1*, *HHF1*, *RPL36A*, and *RPL4A*. The parameters are given as full-colored dots supplemented with vertical standard error bars. Each estimate was derived from distributions shown in Fig. EV1K (refer to the panel for the number of reads). Equations and continuous lines describe functions fitted to modified gamma parameters to predict the evolution of theoretical distributions (Fig. EV2J). (**H**) Density plots showing time-dependent evolution of modeled pA-tail length distributions for mRNAs of low and high abundance. The evolution of the median of those distributions is shown in Fig. 4G. (**I**) Graphs comparing the time-dependent evolution of the modeled modified gamma distribution mean and variance to the experimental one for the whole coding transcriptome. Lines were fitted to both datasets and are displayed with a 95% confidence interval. (**J**) Density plots showing predicted *GAS1*, *HHF1*, *RPL36A*, and *RPL4A* pA-tail length distributions obtained by modeling changes in modified gamma parameters (*γ_shape* and *γ_rate*), as shown in Fig. EV3F, G. These should be compared to experimental distributions shown in Fig. EV1K.

 © The Author(s)

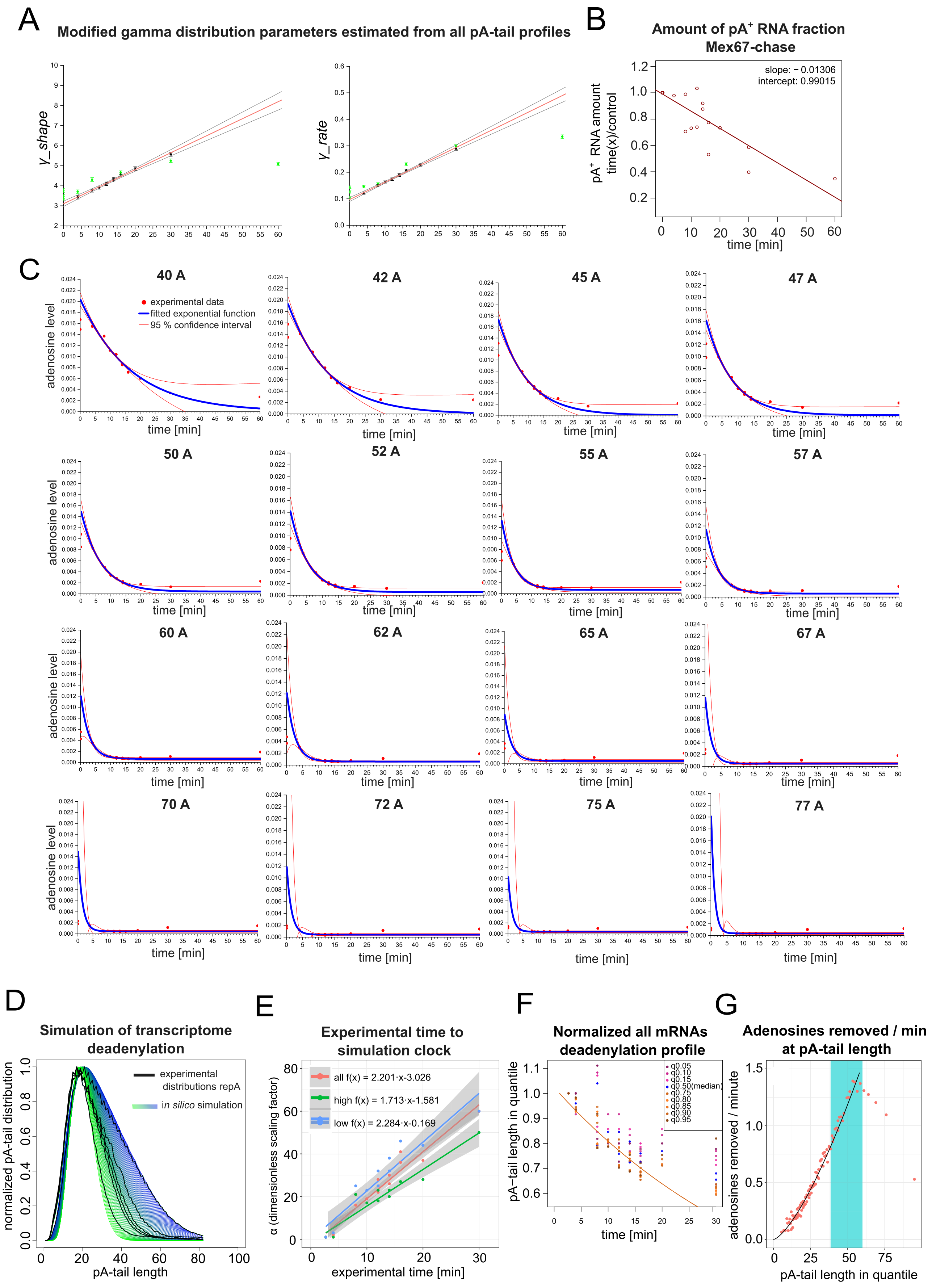
A
Modified gamma distribution parameters estimated from all pA-tail profiles
γ_shape
γ_rate
B
Amount of pA+ RNA fraction Mex67-chase
slope: − 0.01306
intercept: 0.99015
pA+ RNA amount time(x)/control
time [min]
C
40 A
42 A
45 A
47 A
50 A
52 A
55 A
57 A
60 A
62 A
65 A
67 A
70 A
72 A
75 A
77 A
experimental data
fitted exponential function
95 % confidence interval
adenosine level
time [min]
D
Simulation of transcriptome deadenylation
experimental distributions repA
in silico simulation
normalized pA-tail distribution
pA-tail length
E
Experimental time to simulation clock
all f(x) = 2.201·x-3.026
high f(x) = 1.713·x-1.581
low f(x) = 2.284·x-0.169
α (dimensionless scaling factor)
experimental time [min]
F
Normalized all mRNAs deadenylation profile
q0.05
q0.10
q0.15
q0.50(median)
q0.75
q0.80
q0.85
q0.90
q0.95
pA-tail length in quantile
time [min]
G
Adenosines removed / min at pA-tail length
adenosines removed / minute
pA-tail length in quantile

**Figure EV3. The deadenylation process can be reconstituted in silico using the modified gamma distribution.**

(**A**) Graphs illustrating the time-dependent evolution of the *γ_shape* and *γ_rate* parameters of the 3-dimensional modified gamma distributions presented in Fig. 4H. (**B**) Graph depicting time-dependent change in $pA^+$ RNA fraction recovery from total using beads coated with oligo-$dT_{(25)}$. A linear function was fitted to predict changes in whole-transcriptome $pA^+$ RNA levels and was used to normalize pA-tail levels for adenosine half-life calculations shown in Fig. EV3C. (**C**) Series of graphs showing absolute change in levels of adenosines at specific pA-tail positions for the whole transcriptome. Half-lives of adenosines at each position were calculated and plotted in Fig. 4I to derive a transcriptomic apparent deadenylation rate. (**D**) Graph comparing experimental pA-tail density plots for the coding transcriptome in Mex67-depletion chase replicate A (black lines) to distributions resulting from consecutive deadenylation simulation cycles (series of blue-green lines). The constant dictating each deadenylation simulation interval was defined by the dimensionless parameter $\alpha$ (see "Methods"). This was carried out to ensure marked differences between consecutive distributions. All distributions were normalized to the distribution peak (distribution maximum). The in silico distribution that best overlapped with the experimental distributions was selected, assigning a specific value of $\alpha$. (**E**) Graph comparing experimental deadenylation times [min] with the ordinal number of the dimensionless $\alpha$ parameter (dimensionless scaling factor) describing the number of in silico deadenylation steps. (**F**) Graph displaying the time-dependent decrease in pA-tail length values in each quantile normalized to control. The dots designating the upper quantiles (orange dots) cluster around a steeper slope than those designating lower quantiles (blue and maroon dots). (**G**) Graph showing the number of adenosines removed per minute as a function of pA-tail length. This graph complements the one shown in Fig. 5B.


© The Author(s)

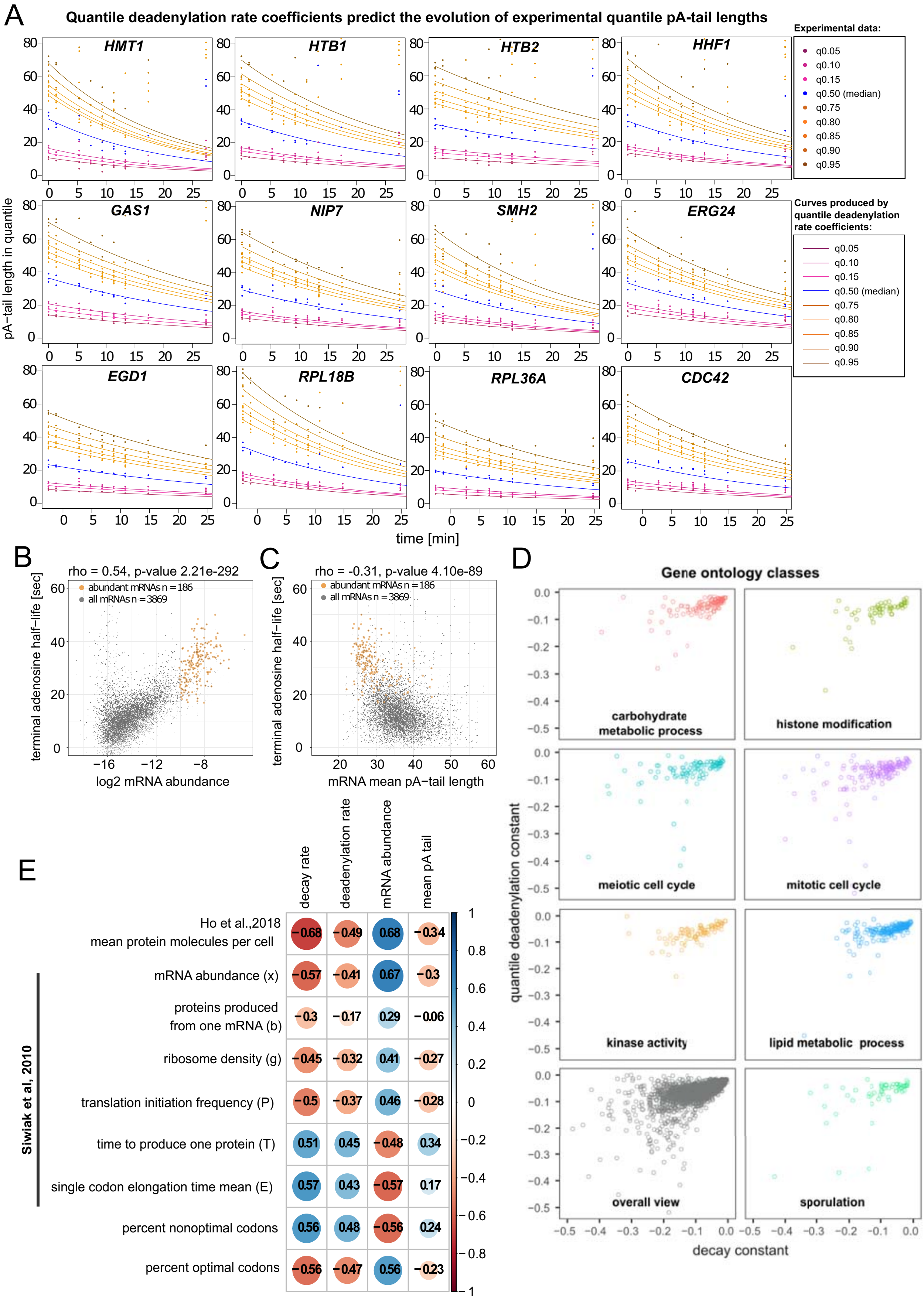
A
Quantile deadenylation rate coefficients predict the evolution of experimental quantile pA-tail lengths
HMT1
HTB1
HTB2
HHF1
GAS1
NIP7
SMH2
ERG24
EGD1
RPL18B
RPL36A
CDC42
pA−tail length in quantile
time [min]
Experimental data:
q0.05
q0.10
q0.15
q0.50 (median)
q0.75
q0.80
q0.85
q0.90
q0.95
Curves produced by quantile deadenylation rate coefficients:
q0.05
q0.10
q0.15
q0.50 (median)
q0.75
q0.80
q0.85
q0.90
q0.95
B
rho = 0.54, p-value 2.21e-292
abundant mRNAs n = 186
all mRNAs n = 3869
terminal adenosine half−life [sec]
log2 mRNA abundance
C
rho = -0.31, p-value 4.10e-89
abundant mRNAs n = 186
all mRNAs n = 3869
terminal adenosine half−life [sec]
mRNA mean pA−tail length
D
Gene ontology classes
carbohydrate metabolic process
histone modification
meiotic cell cycle
mitotic cell cycle
kinase activity
lipid metabolic process
overall view
sporulation
quantile deadenylation constant
decay constant
E
decay rate
deadenylation rate
mRNA abundance
mean pA tail
Ho et al.,2018 mean protein molecules per cell −0.68 −0.49 0.68 −0.34
Siwiak et al, 2010
mRNA abundance (x) −0.57 −0.41 0.67 −0.3
proteins produced from one mRNA (b) −0.3 −0.17 0.29 −0.06
ribosome density (g) −0.45 −0.32 0.41 −0.27
translation initiation frequency (P) −0.5 −0.37 0.46 −0.28
time to produce one protein (T) 0.51 0.45 −0.48 0.34
single codon elongation time mean (E) 0.57 0.43 −0.57 0.17
percent nonoptimal codons 0.56 0.48 −0.56 0.24
percent optimal codons −0.56 −0.47 0.56 −0.23

**Figure EV4. A simplified method based on changes to distribution quantile values can measure deadenylation rates.**

(**A**) Series of graphs depicting as color-coded dots the changes in pA-tail length in selected quantiles (upper—75–80–85–90–95$^{th}$, median—50$^{th}$, and lower—15–10–5$^{th}$) over time for selected single mRNA examples. The continuous lines represent the quantile deadenylation coefficients calculated from the upper quantiles. (**B**, **C**) Scatterplots comparing terminal adenosine half-life to log2 mRNA abundance (**B**) or mean mRNA pA-tail length (**C**). Spearman's rho correlations and p-values were calculated separately for each set using the rstatix package in R. (**D**) Comparison of decay to deadenylation rates for various gene ontology groups, which are also shown in Fig. 5H. (**E**) Correlation matrix comparing decay, deadenylation rates, mRNA abundance, and mean pA-tail length derived from the Mex67-depletion time course to estimates of protein abundance from Ho et al (2018), various translation rate parameters (Siwiak and Zielenkiewicz (2010)), and percent optimal or non-optimal codons.

 © The Author(s)

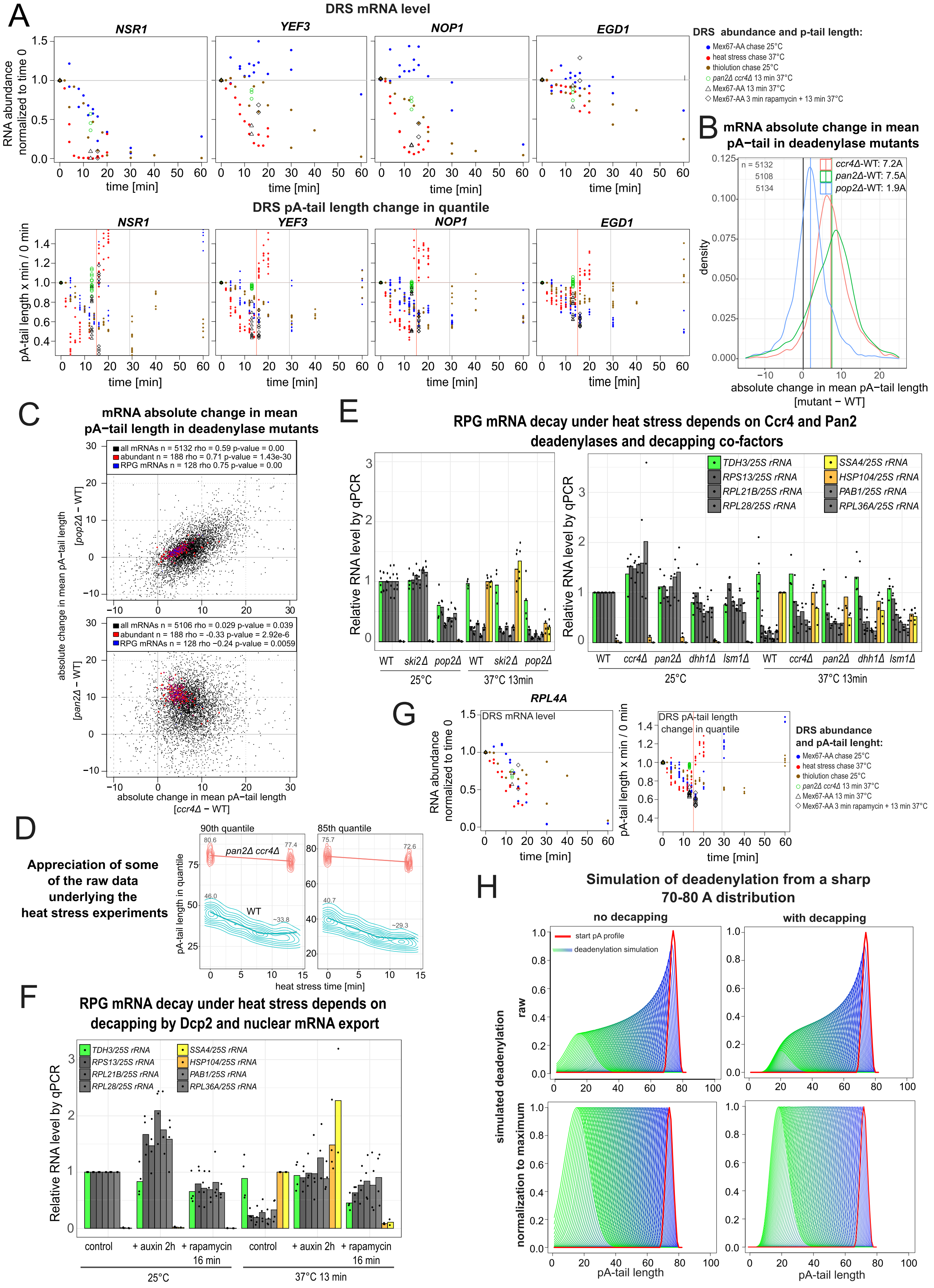
A
DRS mRNA level
NSR1
YEF3
NOP1
EGD1
RNA abundance normalized to time 0
time [min]
DRS pA-tail length change in quantile
pA-tail length x min / 0 min
DRS abundance and p-tail length:
Mex67-AA chase 25°C
heat stress chase 37°C
thiolution chase 25°C
pan2Δ ccr4Δ 13 min 37°C
Mex67-AA 13 min 37°C
Mex67-AA 3 min rapamycin + 13 min 37°C
B
mRNA absolute change in mean pA−tail in deadenylase mutants
n = 5132
5108
5134
ccr4Δ-WT: 7.2A
pan2Δ-WT: 7.5A
pop2Δ-WT: 1.9A
density
absolute change in mean pA−tail length [mutant − WT]
C
mRNA absolute change in mean pA−tail length in deadenylase mutants
all mRNAs n = 5132 rho = 0.59 p-value = 0.00
abundant n = 188 rho = 0.71 p-value = 1.43e-30
RPG mRNAs n = 128 rho 0.75 p-value = 0.00
all mRNAs n = 5106 rho = 0.029 p-value = 0.039
abundant n = 188 rho = -0.33 p-value = 2.92e-6
RPG mRNAs n = 128 rho −0.24 p-value = 0.0059
absolute change in mean pA−tail length
[pop2Δ − WT]
[pan2Δ − WT]
absolute change in mean pA−tail length [ccr4Δ − WT]
D
Appreciation of some of the raw data underlying the heat stress experiments
90th quantile
85th quantile
pan2Δ ccr4Δ
WT
pA-tail length in quantile
heat stress time [min]
E
RPG mRNA decay under heat stress depends on Ccr4 and Pan2 deadenylases and decapping co-factors
Relative RNA level by qPCR
TDH3/25S rRNA
RPS13/25S rRNA
RPL21B/25S rRNA
RPL28/25S rRNA
SSA4/25S rRNA
HSP104/25S rRNA
PAB1/25S rRNA
RPL36A/25S rRNA
WT ski2Δ pop2Δ WT ski2Δ pop2Δ
25°C 37°C 13min
WT ccr4Δ pan2Δ dhh1Δ lsm1Δ WT ccr4Δ pan2Δ dhh1Δ lsm1Δ
25°C 37°C 13min
G
RPL4A
DRS mRNA level
DRS pA-tail length change in quantile
RNA abundance normalized to time 0
pA-tail length x min / 0 min
time [min]
DRS abundance and pA-tail lenght:
Mex67-AA chase 25°C
heat stress chase 37°C
thiolution chase 25°C
pan2Δ ccr4Δ 13 min 37°C
Mex67-AA 13 min 37°C
Mex67-AA 3 min rapamycin + 13 min 37°C
F
RPG mRNA decay under heat stress depends on decapping by Dcp2 and nuclear mRNA export
Relative RNA level by qPCR
TDH3/25S rRNA
RPS13/25S rRNA
RPL21B/25S rRNA
RPL28/25S rRNA
SSA4/25S rRNA
HSP104/25S rRNA
PAB1/25S rRNA
RPL36A/25S rRNA
control + auxin 2h + rapamycin 16 min control + auxin 2h + rapamycin 16 min
25°C 37°C 13 min
H
Simulation of deadenylation from a sharp 70-80 A distribution
no decapping
with decapping
start pA profile
deadenylation simulation
simulated deadenylation
raw
normalization to maximum
pA-tail length

**Figure EV5. RPG and non-RPG mRNAs have altered deadenylation and decay rates during response to heat stress.**

(**A**) Series of graphs showing, in rows, the time-dependent and normalized to control changes in: (top) mRNA abundance by DRS, and (bottom) pA-tail length in upper quantiles (75–95$^{th}$) for single RPG mRNAs: *NSR1*, *YEF 3*, *NOP3*, and *EGD1* in Mex67 depletion, heat stress 37 °C, and thiolutin 25 °C chase sequencing data. (**B**) Mean pA-tail lengths of individual mRNAs in wild-type cells were subtracted from the mean lengths observed in deadenylase mutants (*ccr4Δ* or *pan2Δ*, or *pop2Δ*) and displayed in the form of a density plot. The mean change in each strain is marked as a vertical line and specified in the figure legend. (**C**) Scatterplots showing the absolute change in mean pA-tail length in *ccr4Δ* - WT on the x-axis compared to *pop2Δ* – WT (top graph) or *pan2Δ* – WT (bottom graph) on the y-axis. Transcripts of high abundance or RPG mRNAs are highlighted in blue and red, respectively. Spearman's rho coefficients and the number of mRNAs compared (n) are listed in each panel legend. (**D**) Plots displaying the raw pA-tail length density distribution of two example upper quantiles in the wild-type strain heat stress chase compared to the control and 13 min heat shock of a double *pan2Δ ccr4Δ* mutant strain. The plots present raw data also shown in Fig. 6E. The fitted lines are for orientation purposes only and connect the local maxima either with local or linear regression. (**E**) Abundance of selected RPG mRNAs normalized to *25S rRNA* in control or *ski2Δ*, *pop2Δ*, *ccr4Δ*, *pan2Δ*, *lsm1Δ*, and *dhh1Δ* cells at 25 °C compared to 13 min heat shock at 37 °C determined using reverse transcription coupled to qPCR. Single dots show biological replicate values used to calculate the mean. (**F**) Abundance of selected RPG mRNAs normalized to *25S rRNA* in a double Dcp2-AID and Mex67-AA strain under steady-state and after 13 min heat stress determined using reverse transcription coupled to qPCR. Prior to heat stress the strains were either treated with auxin for 2 h to deplete Dcp2 or with rapamycin for 3 min to deplete Mex67 prior (25 °C). Heat stress was conducted at 37 °C for 13 min. Single dots represent biological replicates. (**G**) Graphs showing for *RPL4A* (top) mRNA abundance by DRS, and (bottom) change in pA-tail length in upper quantiles (75–95th) normalized to control for Mex67 depletion, heat stress 37 °C, and thiolutin 25 °C chase sequencing data, along with point heat stress of *ccr4Δ pan2Δ* double mutant and cells Mex67-depleted 3 min prior to heat stress. (**H**) A theoretical pA-tail distribution of median 75As and low variance (red line) subjected to deadenylation simulation (series of blue and green lines). Artificial deadenylation was performed with and without inducing decapping, displayed with or without normalization to each distribution maximum value. Normalization to the maximum is shown to better display the systematic change in distribution variance and location of the maximum.

 © The Author(s)
